# Expansion of the Materials Cloud 2D Database

**DOI:** 10.1021/acsnano.2c11510

**Published:** 2023-06-13

**Authors:** Davide Campi, Nicolas Mounet, Marco Gibertini, Giovanni Pizzi, Nicola Marzari

**Affiliations:** † Theory and Simulation of Materials (THEOS), and National Centre for Computational Design and Discovery of Novel Materials (MARVEL), École Polytechnique Fédérale de Lausanne, CH-1015 Lausanne, Switzerland; ‡ Dipartimento di Scienza dei Materiali, University of Milano-Bicocca, Via R.Cozzi 55, 20125 Milano, Italy; § Dipartimento di Scienze Fisiche, Informatiche e Matematiche, University of Modena and Reggio Emilia, I-41125 Modena, Italy; ∥ Centro S3, Istituto di Nanoscienze-CNR, I-41125 Modena, Italy; ⊥ Laboratory for Materials Simulations (LMS), Paul Scherrer Institut, CH-5232 Villigen PSI, Switzerland

**Keywords:** two-dimensional materials, monolayers, high-throughput screening, first-principles calculations, electronic properties

## Abstract

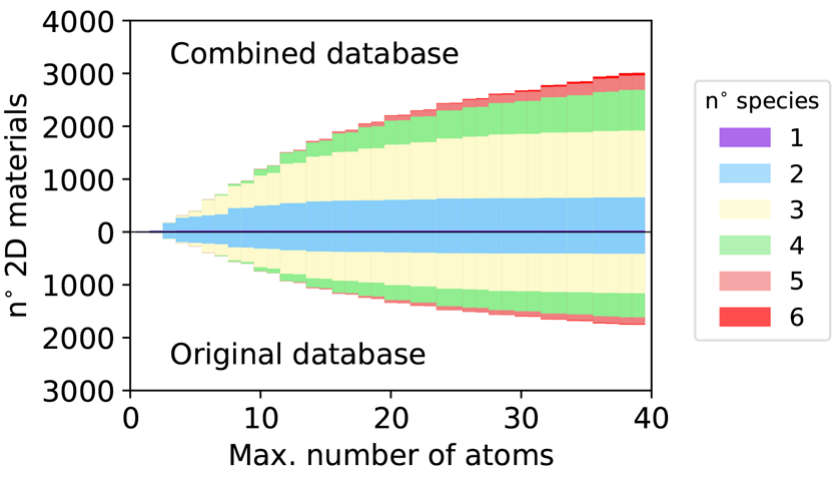

Two-dimensional (2D) materials are among the most promising candidates for beyond-silicon electronic, optoelectronic, and quantum computing applications. Recently, their recognized importance sparked a push to discover and characterize novel 2D materials. Within a few years, the number of experimentally exfoliated or synthesized 2D materials went from a few to more than a hundred, with the number of theoretically predicted compounds reaching a few thousand. In 2018 we first contributed to this effort with the identification of 1825 compounds that are either easily (1036) or potentially (789) exfoliable from experimentally known 3D compounds. Here, we report on a major expansion of this 2D portfolio thanks to the extension of the screening protocol to an additional experimental database (MPDS) as well as the updated versions of the two databases (ICSD and COD) used in our previous work. This expansion leads to the discovery of an additional 1252 monolayers, bringing the total to 3077 compounds and, notably, almost doubling the number of easily exfoliable materials to 2004. We optimize the structural properties of all these monolayers and explore their electronic structure with a particular emphasis on those rare large-bandgap 2D materials that could be precious in isolating 2D field-effect-transistor channels. Finally, for each material containing up to 6 atoms per unit cell, we identify the best candidates to form commensurate heterostructures, balancing requirements on supercell size and minimal strain.

## Introduction

Two-dimensional (2D) materials represent a vast and broadly unexplored region of materials space. Thanks to their extreme thinness they are regarded as ideal platforms for electronic and optoelectronic applications in the beyond-silicon era^1−6^ as well as powerful candidates for electro- and photocatalysis^7,8^ and for the realization of exotic states of matter.^9−11^ Moreover, their properties are much easier to tune with strain, electric fields, and doping with respect to their bulk counterparts, and they can be combined in virtually endless van der Waals (vdW) heterostructures^12^ to engineer novel functionalities. Although until a few years ago only a few dozen of 2D materials had been actively studied, in recent years major progress has taken place in the experimental synthesis or exfoliation of a variety of more than a hundred 2D materials.^13,14^ Even more impressively, the number of 2D materials theoretically predicted using high-throughput computational methods^15^ has grown from less than two hundred^16,17^ to a range of the thousands.^18−23^ In our first contribution to this effort^21^ we screened the Inorganic Crystal Structure Database^24,25^ (ICSD) and the Crystallography Open Database^26^ (COD) for layered materials, identifying a total of 1825 2D materials that, on the basis of their computed binding energies, could be easily (1036) or potentially (789) exfoliated from their layered parent structures with mechanical^27^ or liquid-phase^28,29^ methods. In this work we follow a refined protocol based on the one used in ref (21) (starting from geometric and bonding criteria to identify layered materials, followed by the calculation of binding energies using first-principles vdW density functional theory (DFT) simulations), adding as a source a third database, the Pauling File^30−32^ (MPDS), and we repeat the screening on ICSD^24^ and COD^26^ databases using their most up-to-date versions, as well as allowing the inclusion of larger structures and using less stringent thresholds on the initial geometrical selection. The latter condition results in a more inclusive selection at the price of a slightly larger rate of false positives that are later ruled out by DFT calculations. This extended screening allows us to identify 1252 additional 2D materials that could be exfoliated from experimentally known, stoichiometric compounds, bringing the total to 3077 and notably doubling to 2004 the number of compounds that should be most easily exfoliable. Furthermore, for each of these 3077 monolayers, we optimize their in-plane unit cells and internal geometries, treating them as isolated 2D systems, and we compute their electronic band structures. On the basis of the optimized geometries, we suggest, for each material up to 6 atoms per unit cell, ideal candidates to build simple, commensurate vertical heterostructures or lattice-matched lateral heterostructures with minimal strain. Finally, we study a handful of materials with exceptionally large bandgaps that could serve as insulating layers for 2D field-effect-transistor (FET) channels, with superior performance with respect to the widely used BN.^33^ The full reproducibility of the study is ensured by the AiiDA^34,35^ materials informatics infrastructure, which keeps track of the provenance of each calculation; therefore, the results are openly available together with their entire provenance through the Materials Cloud in the form of Discover, Explore, and Archive entries and the associated AiiDA database.^36,37^

## Results

### Identification of Layered Materials and Optimization of Bulk Compounds

Following the recipes and tools detailed in ref (21), we start the computational exfoliation protocol by extracting the bulk 3D crystal structures, in the form of CIF files,^38^ from three experimental repositories: ICSD,^24^ COD,^26^ and the Pauling File (MPDS).^30,31^ We exclude structures with partial occupations together with CIF files that do not provide the explicit positions of one or several atoms, cannot be parsed, or are obviously wrong. Theoretically predicted structures are also discarded when signaled. This results in a total of 147731 structural entries for ICSD, 279885 for COD, and 355016 for MPDS. In this work we focus on entries containing at most 6 different species and 100 atoms or less in the primitive unit cell, reducing the number of entries to 140586, 91161, and 262010 for ICSD, COD, and MPDS, respectively. The CIF files are extracted and then converted into AiiDA structures using pymatgen.^39^ All these 3D structures are separately analyzed to find possible candidates for exfoliation using the same geometrical screening procedure originally described in ref (21), building a connection between two atoms when their distance is smaller than the sum of their respective vdW radii at least by Δ, which is a parameter in the protocol. In the current screening we assume slightly larger uncertainties in the vdW radii,^40^ thus allowing Δ to range between 1.0 and 1.5 Å (1.1–1.5 Å was used in the original screening). The geometrical selection thus identifies a total of 8963 layered materials in ICSD, 6794 in COD, and 11530 in MPDS. These selected structures are then processed with the spglib software^41^ to find the primitive cells and filtered for uniqueness (separately for each source) using the pymatgen structure matcher.^42^ Finally a second cutoff on the number of atoms (≤40 atoms/unit cell independently of the number of atomic species) is applied, leaving respectively 6933, 6283, and 5907 structures for the three databases. The results of this process are summarized in Table 1.

**Table 1:** Summary of the Results of the Extraction Procedure and Geometrical Selection on the Three Databases Considered in the Present Work

	MPDS	ICSD	COD
total	355016	147731	279885
*N*_atoms_ ≤ 100, *N*_species_ ≤ 6	262010	140586	91161
layered	11530	8963	6794
layered, unique and *N*_atoms_ ≤ 40	6933	6283	5907

These structures coming from the three different databases are combined and filtered for uniqueness a second time (this time across the different databases), giving a total of 9306 layered candidates, 3689 of which were not included in our previous screening.^21^ In ref (21) the structures of the layered materials obtained from the geometrical selection were optimized using two different nonlocal vdW-compliant functionals: the vdW-DF2 functional^43^ with c09 exchange^44,45^ (DF2-c09) and the revised Vydrov–Van Voorhis^46−48^ (rVV10) functional. Subsequently the binding energy (the difference between the total energy of the optimized 3D bulk structure and the sum of the energies of each isolated substructure of any dimensionality^49^ per unit area) was computed with both functionals. The two resulting binding energies turned out to be rather similar and very rarely changed the classification of a material. For this reason, we abandon this redundancy here and employ only the vdW DF2-c09 functional for the structural optimization and subsequently the calculation of the binding energies for the 3689 new entries. As in our previous study, the optimization of the 3D parent and the calculation of the binding energies are always done by considering only a spin unpolarized ground state even for materials that might present magnetic order. This approximation, tested on 52 materials in ref (21), was found to introduce only small errors, with deviations on the order of a few meV/Å^2^ that do not affect the classification of the material.

### Binding Energies

**Figure 1:**
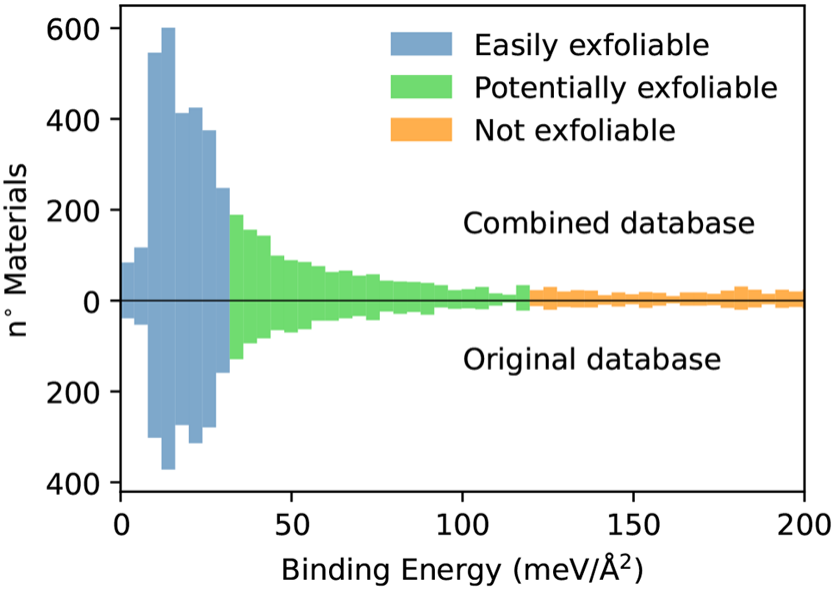
Distribution of the 5461 binding energies computed in the combined (MC2D) database compared with the distribution of the binding energies computed in our previous study.^21^ The color code reflects the classification of easily exfoliable (binding energies ≤30 meV/Å^2^), potentially exfoliable (binding energies ranging from 30 to 120 meV/Å^2^) and nonexfoliable (binding energies larger than 120 meV/Å^2^).

Before the binding energies were calculated, all of the structures, optimized with vdW DF2-c09, are further screened with the geometrical selection algorithm to assess whether they maintain their layered nature after relaxation. This further selection, together with some unavoidable convergence failures, results in a total of 2251 binding energies successfully computed, to be added to the 3210 computed in our previous work.^21^ We report in Figure 1 the distribution of the binding energies for the overall combined database compared with the distribution of the 3210 binding energies obtained in ref (21). The colors reflect the classification of the materials developed in ref (21) into three classes according to their binding energies: easily exfoliable (with binding energies below 30 meV/Å^2^, which is close to that of materials routinely exfoliated with standard techniques like graphene or MoS_2_), potentially exfoliable (binding energies between 30 and 120 meV/Å^2^), and nonexfoliable (binding energies greater than 120 meV/Å^2^).

Similar to ref (21), the majority of the materials exhibit binding energies below 30 meV/Å^2^. In the current work, the predominance of materials with a low binding energy is even more prominent, with a sharper main peak positioned at lower energies and a relatively faster decay of the tail in the potentially exfoliable region. A similar plateau, extending up to 400 meV/Å^2^, can instead be observed in the non-exfoliable region.

**Figure 2:**
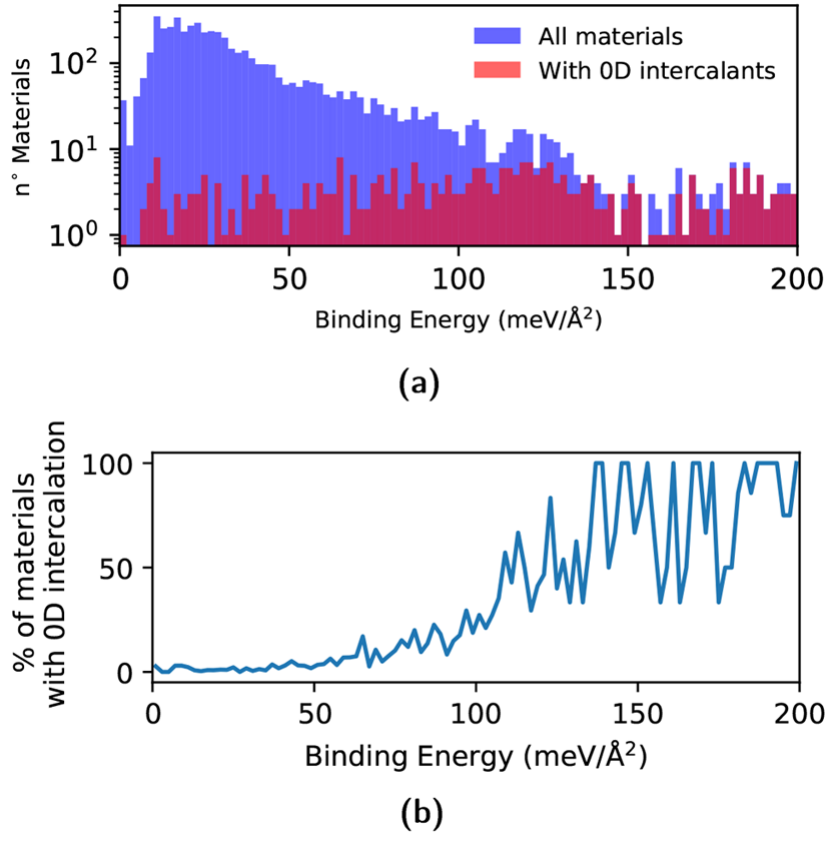
(a) Distribution of the binding energies computed in this work classified according to the dimensionality of the bulk parent. In blue we report the binding energies of materials coming from bulk parents composed only of 2D structures, while in red we report materials deriving from parents with a mixed dimensionality, i.e., including also 0D structures besides the 2D layers. (b) Percentage of materials containing 0D structures as a function of the binding energy.

The materials in the region roughly below 30 meV/Å^2^ are, in a great majority, derived from purely vdW-bonded systems, and the differences in binding energies can be rationalized with the typical differences in the strength of vdW forces. These materials are ideal candidates for “Scotch-tape” mechanical exfoliation and are expected to provide high yields also in liquid exfoliation. Materials classified as potentially exfoliable, with moderately higher binding energies, present a variety of behaviors that cannot be rationalized into a single class. These structures can come from vdW-bonded structures but with a high degree of interpenetration between different layers, resulting in higher total binding energies per unit of area, or from layered structures that are not purely vdW-bonded but might show a strong resonant bond or a weak residual interlayer covalent interaction or hydrogen bonding. Finally they might come from bulk parents with 0D intercalated units. Similarly to our previous results^21^ we can in fact note that, in percentage, the region of lower binding energies is largely dominated by materials whose parent 3D structure is composed exclusively of 2D substructures;^50^ instead, moving toward higher binding energies, materials with mixed dimensionality (typically 0D and 2D) become more common (see Figure 2), even if with less prominence than what was previously observed. This is particularly true entering the range of binding energies that we classify as nonexfoliable by simple mechanical methods. Here the great majority of structures come from parent layered materials that present intercalated 0D components, which typically give rise to a charge transfer between intercalated units and 2D layers and result in stronger ionic bonds. These materials would be hard or impossible to obtain via the mechanical exfoliation that this screening seeks to mimic, and we therefore decided to exclude them from the study. Nevertheless it is possible that these materials could be exfoliated via chemical processes in solution or the choice of a proper surfactant to reduce the interaction between the 2D layers and the ionic intercalated components.^28,29,51,52^

All of the 2D substructures obtained from the exfoliation process are filtered for duplicates: it is in fact common that the same monolayer can be obtained from different bulk parents. Each monolayer can therefore be associated with multiple binding energies, according to the parent from which they have been isolated. For practical purposes, in the analysis that follows, we associate each monolayer to the lowest possible binding energy. Upon removal of duplicates, we end up with 1252 monolayers in addition to the 1825 found in our previous work.^21^ Interestingly, a large number of these materials fall in the easily exfoliable category, 968 of 1252, representing more than 77% of the materials, a significantly larger percentage than the 57% found in our previous study. The net result is that we have now doubled the number of easily exfoliable materials, bringing the total to 2004 in the combined database. These represent an optimal target for future experimental or theoretical studies, driven by the attractive combination of having an established experimental bulk parent and being easily exfoliable. All the 3077 materials and their properties are available in the MC2D Discover section of the Materials Cloud together with the full AiiDA database in the Archive section^37^ and allowing also for a full exploration of the provenance graph in the Explore section.^53^ A comprehensive summary of the properties including the optimized structure, the electronic bands, the binding energies, and information on the parent 3D material for all the materials up to 12 atoms per unit cell is included in the Supporting Information.

**Figure 3:**
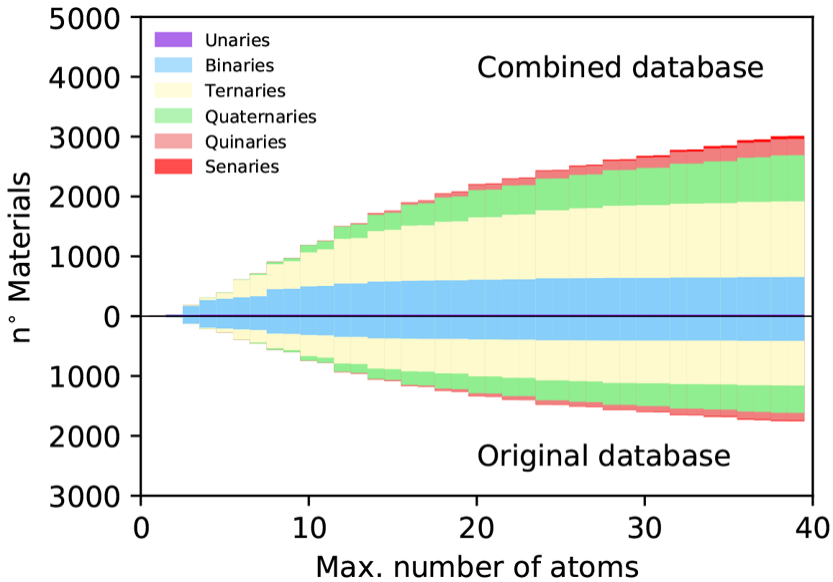
Number of structures as a function of the number of atoms in the primitive cell (top, exfoliable 2D structures in the overall database; bottom, exfoliable 2D structures obtained in ref (21)).

In Figure 3 we show how the 2D compounds are distributed in terms of the number of species and number of atoms in the unit cell in the combined database compared with old results. Overall, the distributions are fairly similar; among the new structures, there are, in proportion, fewer unary and binary structures with small unit cells (up to 6 atoms per unit cell). This is to be expected since the simpler unary and binary monolayers as well as their parent compounds have been heavily studied and, therefore, had a higher chance to be included in our previous screening. Nevertheless we found 10 additional unary compounds. The greater number of quinary and senary materials with larger unit cells reflects instead the choice to include in the current screening all the materials up to 40 atoms per unit cell regardless of the number of species, while previously a cutoff of 32 atoms per unit cell had been applied for materials with more than 4 atomic types.

**Figure 4:**
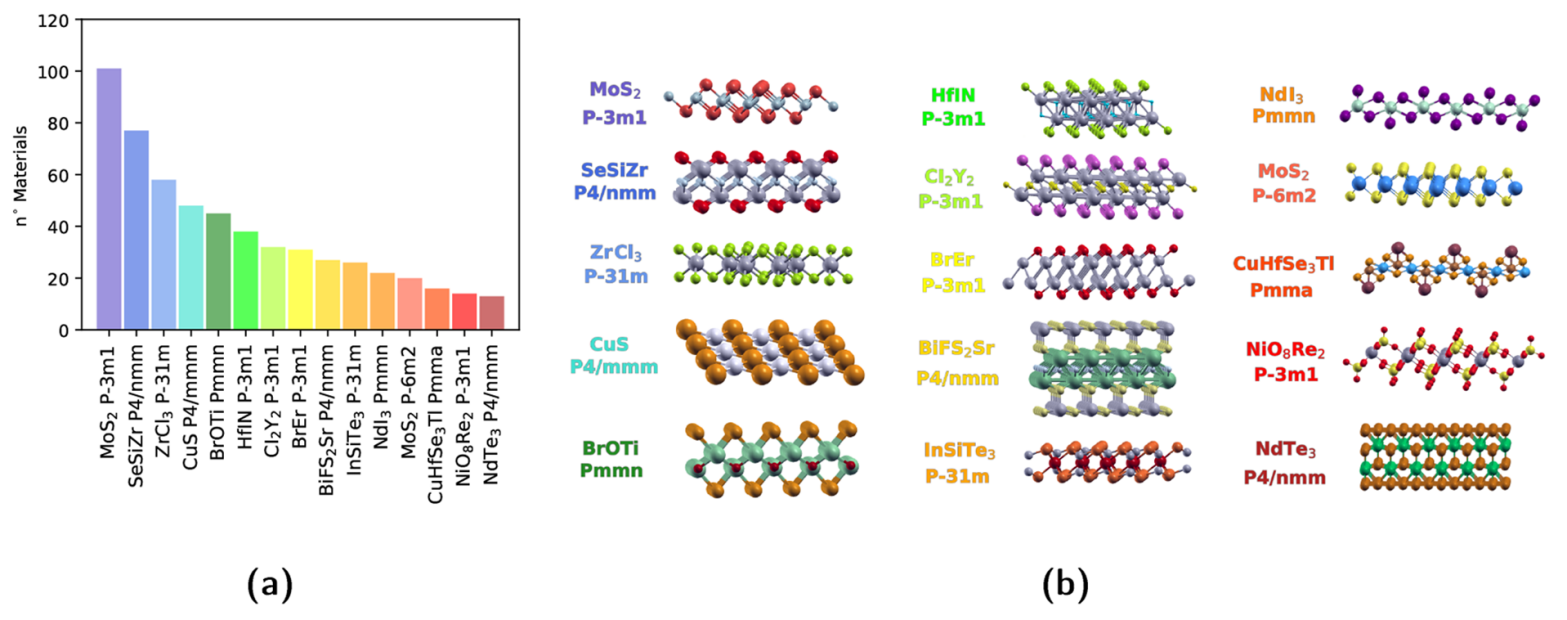
(a) Most common 2D structural prototypes: histogram with the number of structures belonging to the 15 most common 2D structural prototypes in the database regardless of their classification as easily or potentially exfoliable. (b) Graphical representation of each prototype, together with the structure-type formula and the space group of the 2D systems.

In order to provide a more precise overview, we classify the 2D materials into different prototypes, according to their space groups and their structural similarity, considering all of the elements indistinguishable. In the combined database, we find a total of 1124 prototypes, effectively doubling the number of prototypes found in our previous screening (566). The 15 most common prototypes, accounting for a total of 568 structures, are reported in Figure 4. The most common structural prototype is the one including transition-metal dichalcogenides and dihalides such as MoS_2_ and CdI_2_ in the 1T structure with the hexagonal space group *P*3̅*m*1; this counts 102 similar structures. In the second and third positions with 77 and 58 representatives, respectively, we find the rectangular SeSiZr prototype and the hexagonal trihalides, many of which, like CrI_3_, are currently heavily studied for their outstanding magnetic properties.^54^ Surprisingly, 2H-TMDs are only ranked 12th with 20 structures. Eighth in order of abundance (with 31 structures), we find a previously unreported prototype of a binary rare-earth/halide structure represented in Figure 4 by BrEr.

**Figure 5:**
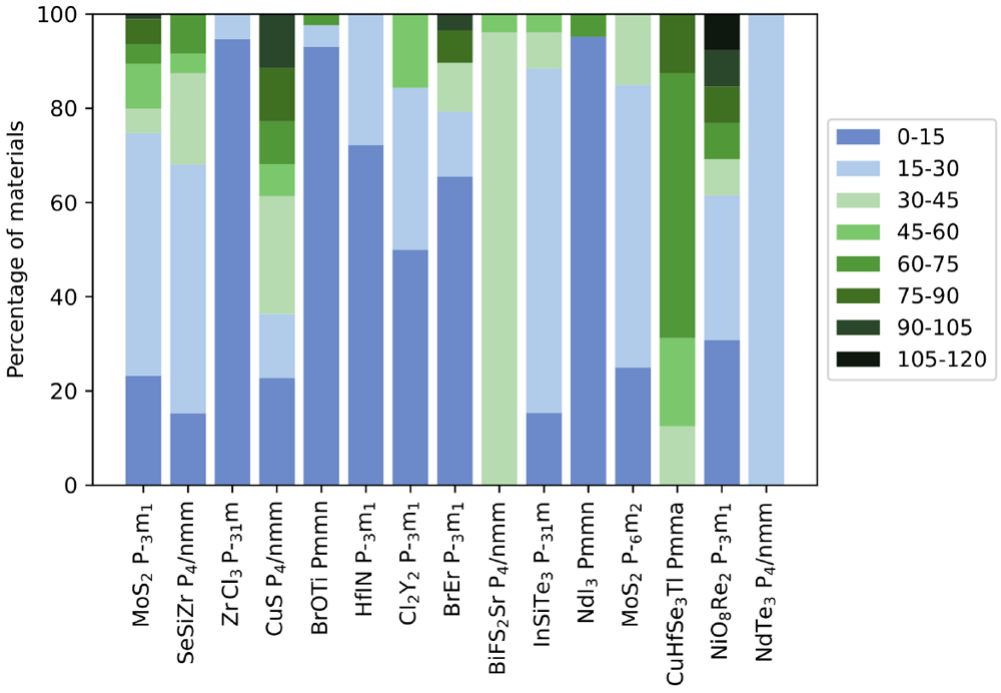
Distribution of the binding energies for the materials in the 15 most common prototype classes. The 0–120 meV/Å^2^ range has been equally divided into eight parts, each represented by a different color.

To provide further insight, we report in Figure 5 the distribution of the binding energies for the 15 prototype classes enumerated in Figure 4. It can be noted that while some classes, like trihalides and trichalcogenides represented respectively by ZrCl_3_ and NdI_3_, present a distribution of the binding energies covering only one or two ranges in the figure; in many cases the classes span the entire range of binding energies with larger binding energies often associated with heavier atoms in the outermost layers. The relative abundance of high binding energies in the class of square-lattice transition-metal monochalcogenides represented by CuS explains why this prototype did not appear in our previous analysis,^21^ which was focused exclusively on easily exfoliable materials. The same holds for the prototypes represented by BiFS_2_Sr, important for its potential applications in spin-FET transistors, as well as the one represented by CuHfSe_3_Tl that both contain exclusively materials in the potentially exfoliable category.

### Optimization of the 2D Monolayers

**Figure 6:**
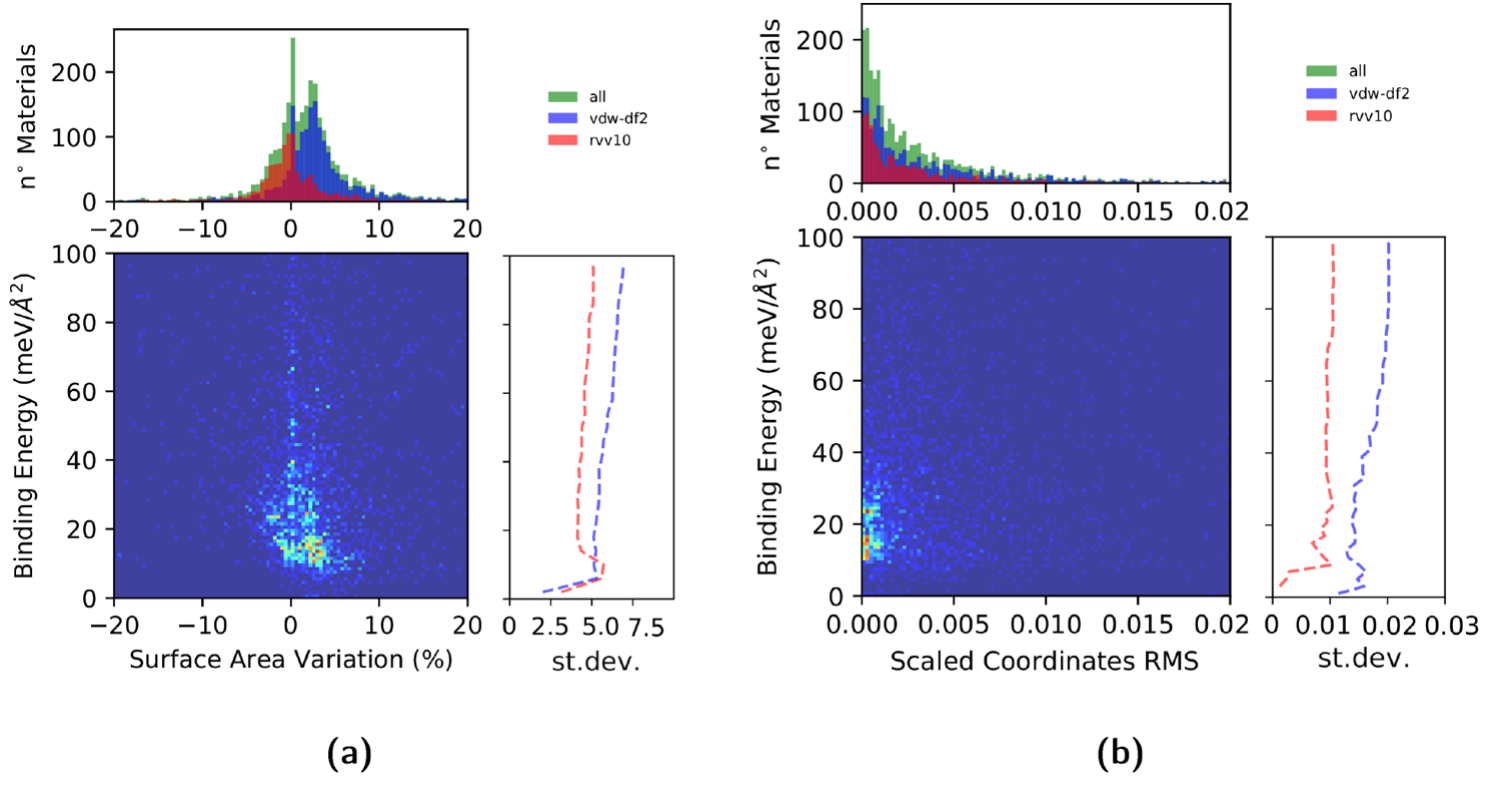
(a) Distribution of the surface area variation during the variable cell relaxation as isolated 2D structures with the PBE functional. In green are given all the structures, in blue are given the structures whose 3D parent has been relaxed with vdW DF2-c09, and in red are given the ones whose 3D parent has been relaxed with rVV10 in ref (21). Below we report a heatmap representing the distribution of the number of structures according to their binding energy and the variation of their surface area during the optimization as isolated 2D materials. In addition, we report the normalized standard deviation of the surface variation distribution as a function of the binding energy, where a slow but steady increase can be observed. (b) Distribution, heatmap, and evolution of the normalized standard deviation for the root-mean-square (RMS) of the scaled positions during the relaxation.

All the 3077 monolayers composing the combined database have been optimized as isolated 2D structures performing a variable cell relaxation under open-boundary conditions^55^ and using the PBE^56^ approximation for the exchange-correlation functional, without any nonlocal, vdW correction. The choice is dictated by the fact that in isolated, covalently bonded 2D monolayers, van der Waals interactions should be contributing only marginally to the equilibrium geometry of the structure. In Figure 6a we report the variation of the primitive cell area during the variable cell optimization computed as the difference between the fully optimized structure and the structure as extracted from the 3D parent. Overall, the surface variations are distributed according to a bimodal distribution, with a first peak corresponding to nearly zero variation and a second peak around an expansion of 3%, while a negligible fraction of materials experienced a variation larger than 10%. Distinguishing the data according to the functional used to optimize the 3D bulk parent (vdw DF2-c09 for all the structures unraveled in this work and vdw DF2-c09 or rVV10 for the ones obtained in ref (21)) suggests that the choice of the functional used for the bulk has a distinguishable effect on the surface area differences, with 2D structures extracted from parents optimized with vdW DF2-c09 showing on average a larger expansion during the 2D optimization. This is largely ascribed to the intrinsic differences between the PBE functional and vdW DF2-c09, with the former giving on average an equilibrium surface area 2.5% larger than that of the latter (see the Supporting Information). In Figure 6b we report instead the distribution of the normalized root-mean-squared variation of the scaled positions, to account for the internal reorganization of the atomic positions during the optimization of the monolayers. In this case, the distribution is characterized by a monotonic exponential decay with the vast majority of structures showing little or no internal rearrangement. The distribution also seems unbiased by the vdW approximation used for the bulk parent. Both of these results suggest that, as expected, the isolated optimized monolayers retain a marked structural similarity to the materials as extracted from the layered bulk parent, reiterating their aptitude to be exfoliated. Although explicit calculations of the phonon dispersion over the full Brillouin zone would be needed to make definitive statements, the marginal structural relaxations also suggest, based on previous results,^21^ that the vast majority of structures should display dynamic stability.

In the heatmaps of Figures 6a,b we can observe how the binding energy of a material correlates with the structural changes found during the optimization of the isolated monolayer. As one might expect, it can be observed in the standard deviation plots that, on average, materials with lower binding energies have a smaller probability to experience large structural variations during the cell relaxation; however, this probability does not grow linearly with the binding energy but keeps a rather constant value above 50 meV/Å^2^ after an abrupt decay. This behavior might signal a more prominent presence above 50 meV/Å^2^ of structures erroneously classified as 2D (for example, structures in which a 0D component has been erroneously separated including the 0D unit as a part of the 2D material instead of being properly isolated).

### Lattice Matching for Lateral and Vertical Heterostructures

Thanks to the progress in synthesis techniques, highly ordered lateral heterostructures have recently been realized, especially in transition-metal dichalcogenides and III–IV 2D semiconductors. These structures could enable the realization of a wide range of applications from field-effect transistors to electronic oscillators, nonvolatile memory elements, and plasmonics,^57−59^ but they require a nearly perfect lattice match between the two components in order to have atomically ordered defect-free interfaces. Moreover, although lattice matching is not a strict necessity in the realization of vertically stacked heterostructures, the stacking order and possible strain effects could significantly change the electronic properties of 2D materials,^60^ especially when a 2D material is used as a substrate for the vdW epitaxial growth of another.^61^ For example, heterostructures formed by lattice-mismatched materials could lead to an inhomogeneous electron distribution and surface distortion that could negatively impact materials properties such as the carrier mobility.^62^ Finally, theoretical calculations, usually performed under periodic-boundary conditions, struggle with the incommensurability of target layered systems. A compromise between a favorable unstrained stacking and system size of the unit cell that can accommodate both materials is often necessary in order to lower the computational cost; pairs of materials that could form lattice matched heterostructures with small supercells and low strain then become especially appealing. For these reasons, several works already tackled the problem of searching for optimally matched heterostructures^63,64^ or used such methods to study experimentally observed heterostructures.

**Figure 7:**
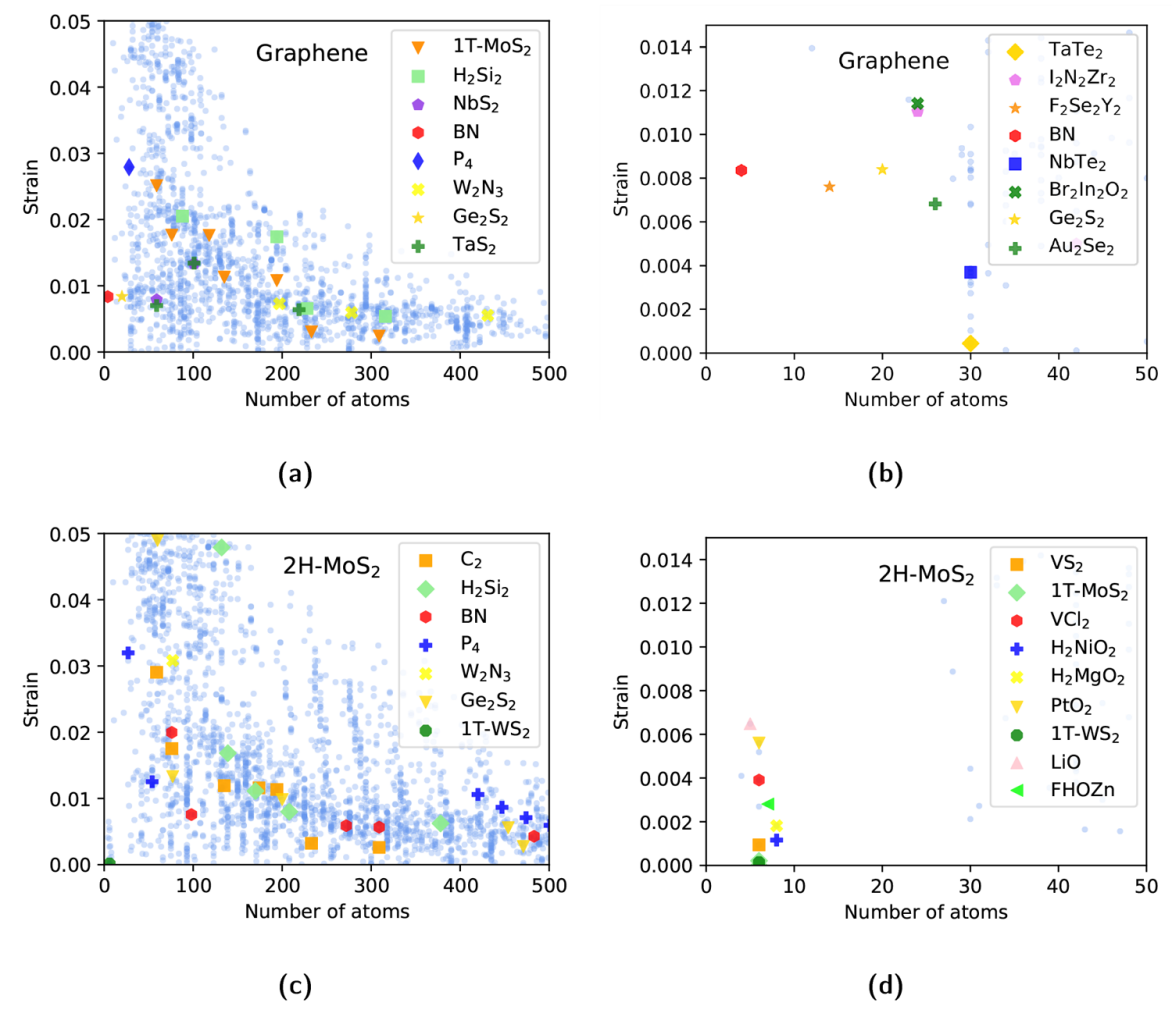
(a, b) Low-strain heterostructures of graphene with materials with ≤6 atoms per unit cell. On the *x* axis we report the number of atoms in the heterostructure of a certain material with graphene and on the *y* axis, the average strain (or total deformation) applied to such a material to match the proper graphene supercell. The light blue points represent the minimum strain option for each material. Some noticeable 2D materials are highlighted as an example. (c, d) Possible low-strain heterostructures of 2H-MoS_2_ under the same conditions.

In this work, following the approach proposed in ref (63) and on the basis of the aforementioned optimized lattice parameters, for each monolayer with up to 6 atoms per unit cell we search, within our database, for ideal materials that could give rise to lattice-matched vertical heterostructures. In Figure 7 we present two examples of this search for two well-known materials, graphene and 2H-MoS_2_, reporting the supercell size that each material (with up to 6 atoms in the unit cell) would need to form a commensurate heterostructure with a properly sized supercell of either graphene or 2H-MoS_2_, together with the average unit cell strain (see ref (63) and references therein) necessary to achieve a perfect lattice match. Unsurprisingly, for graphene, boron nitride represents an excellent option but also Ge_2_S_2_ or less known materials like F_2_Se_2_Y_2_ can form lattice-matched heterostructures with small unit cells and relatively small strain, while for example TaTe_2_ can form a 30-atom heterostructure with almost zero strain. 2H-MoS_2_ instead offers many more options for small supercells; in particular it is interesting to notice how, beside the well-known 1T phase of MoS_2_ and WS_2_, materials belonging to other structural prototypes like H_2_NiO_2_ or H_2_MgO_2_ can give rise to nearly perfectly matched (1 × 1) heterostructures.

**Figure 8:**
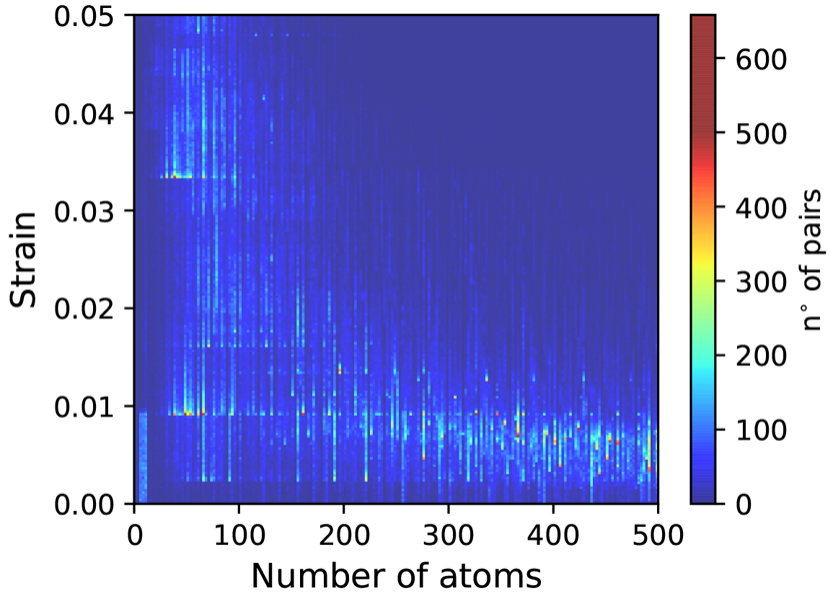
Distribution of the number of pairs generated from the combination of 2D materials with up to 6 atoms in the unit cell, as a function of both the strain necessary to match their lattice parameters and the number of atoms in the resulting supercell. The distribution follows a predictable inverse correlation between the strain and the number of atoms, but notably a cluster of interesting pairs can be found in the bottom-left corner, representing a group of 5989 pairs that can be realized with a small strain and a simple 1 × 1 reconstruction.

In Figure 8 we report instead the distribution of the possible combinations obtained matching all 606 structures with up to 6 atoms per unit cell among each other. As one might expect, the distribution follows an inverse correlation between the applied strain and the number of atoms in the resulting matched heterostructures. However, it is interesting to notice that in the bottom-left corner of the distribution, a dense cluster of uniformly distributed pairs is present, with up to a maximum of 12 atoms in the unit cell and a maximum strain lower than 1%. This cluster contains a group of 5989 combinations composed of pairs of materials that can be commensurate with a 1 × 1 construction and very low strain. The cluster is dominated by pairs showing hexagonal space groups with structures coming from the most common hexagonal structural prototypes forming commensurate 1 × 1 heterostructures with other structures belonging to the same prototype or, with the same probability, with structures belonging to a different hexagonal prototype. This is particularly interesting since the possibility to match materials coming from different structural prototypes gives access to a large variety of properties that can be combined to realize heterostructures with unique characteristics. Rectangular/square cells are present with 1570 pairs, but in this case the majority of pairs comes from the same prototype, namely SeSiZr (*P*4/*nmm*). Tables and lists for all the 606 materials studied can be found in the Supporting Information, providing key information for designing stacked heterostructures.

### Electronic Properties of the 2D Monolayers

**Figure 9:**
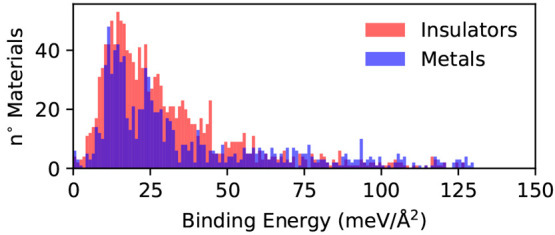
Distribution of the binding energies in the entire database of 3077 materials distinguished according to the electronic properties of the monolayer at the PBE level. Insulators are reported in red, while metals are shown in blue.

**Figure 10:**
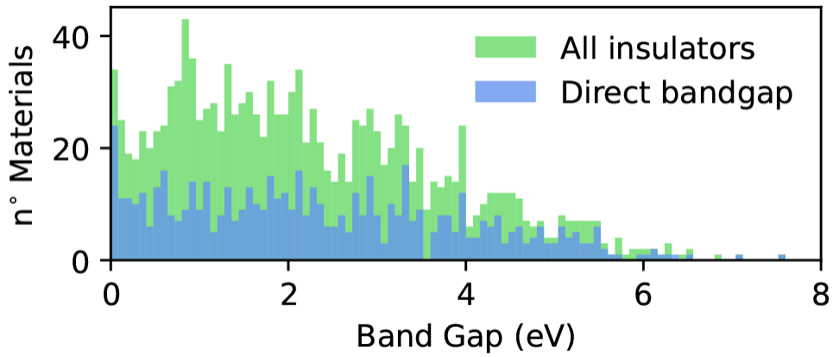
Distribution of the fundamental bandgap at the PBE level for the semiconducting and insulating materials present in the database obtained on the basis of the band structures computed along high-symmetry paths. In blue, we highlight the contribution of direct-bandgap materials.

The structural optimization proved to be successful for a total of 2742 materials, while 335 failed to converge even with all the failsafe recoveries of the AiiDA workflow (these will be studied in the future with variational minimization^65^ as it is being implemented in the Sirius code^66^). For these 2742 materials, we compute band structures at the PBE level along high-symmetry paths. Magnetism is not considered, and all the structures, even the ones with atoms that might support a magnetically ordered ground state, have been treated as nonmagnetic. We find that 65% of the materials are metallic, while the rest are insulating or semiconducting. In Figure 9 we report the distribution of the binding energies for the entire database, classified according to the electronic properties of the monolayer. We can note that at very low binding energies both metallic and semiconducting/insulating materials are distributed in a relatively similar way, but at binding energies between 25 and 50 meV/Å^2^ the presence of insulating compounds is marginally higher. On the contrary, for higher binding energies (above 50 meV) the presence of metallic compounds becomes more dominant, possibly signaling again a higher presence of materials with unsaturated bonds. In Figure 10 we report instead the distribution of the fundamental bandgaps at the PBE level for the insulating and semiconducting monolayers. The bandgaps follow a quite broad distribution with a peak around 1 eV, followed by a wide plateau in the 1–2.5 eV energy range and a subsequent slow decay. In the picture, we also highlight the fraction of direct-bandgap materials, particularly relevant for optical and optoelectronic applications. These materials are distributed fairly uniformly throughout the energy range, accounting for roughly one-third of the materials for moderate bandgaps but with ratios increasing up to one-half for extremely small or very large gaps. Interestingly, if one accounts for the well-known underestimation of the gap with DFT, materials with bandgaps around 1 eV would tend to be shifted toward higher values, offering hundreds of candidates in the optimal range for solar light harvesting.

**Figure 11:**
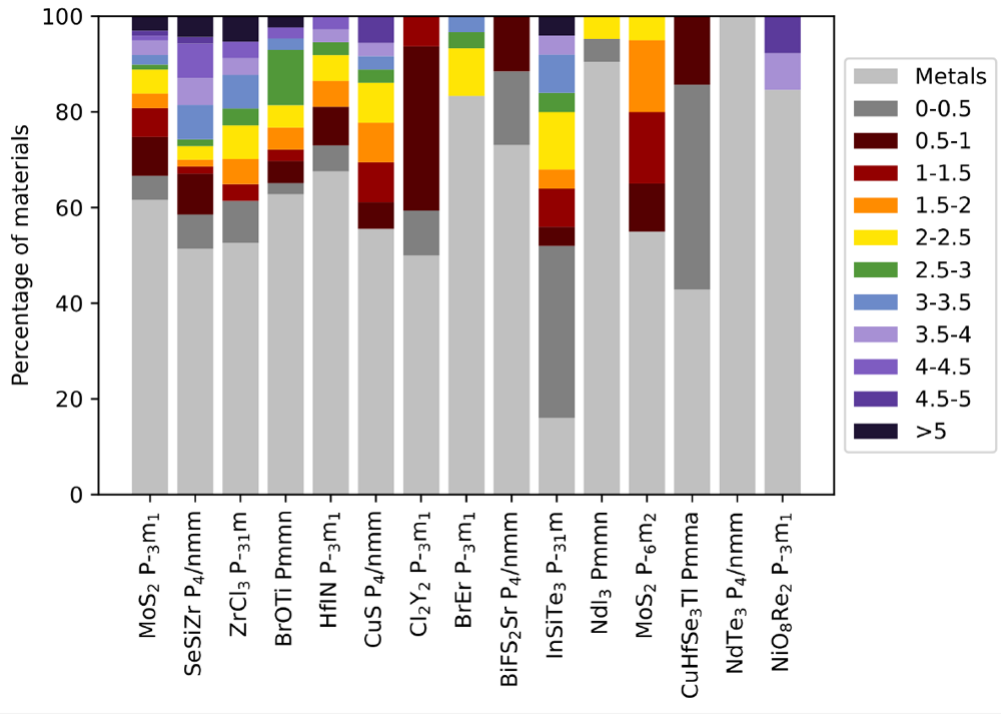
Distribution of the PBE band gaps for the materials in the 15 most common prototype classes. The 0–5 eV range has been equally divided into ten parts, each represented by different colors.

In Figure 11 we show the distribution of the PBE fundamental bandgaps within each of the prototypical families identified in Figure 4. It can be noted that while certain classes like the trichalcogenides and trichlorides represented respectively by NdTe_3_ and NdI_3_ are almost entirely dominated by metallic compounds or at most small-bandgap materials, (e.g., Cl_2_Y_2_, BiFS_2_Sr, and CuHfSe_3_Tl), others offer a broad range of properties ranging from metallic to wide bandgaps. This classification could be used as a guide in a combinatorial search through atomic substitutions for specific applications, or to highlight the potential of bandgap tuning through atomic alloying within each class.

**Figure 12:**
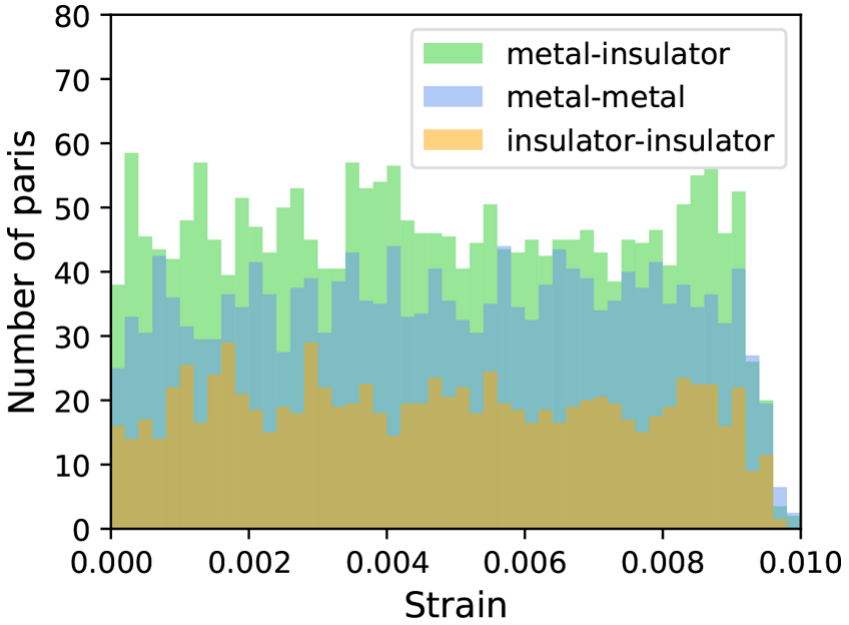
Superimposed histograms with the distribution of the number of pairs forming commensurate 1 × 1 heterostructures with up to 12 atoms per unit cell as a function of strain, classified according to the type of heterostructure (metal–insulator, metal–metal, insulator–insulator) they can form. The metal–insulator pairs are the most common, followed by metal–metal and finally insulator–insulator pairs.

Finally, in Figure 12 we show how the group of simple heterostructures identified earlier (1 × 1 reconstruction and small strain) can be classified according to the type of junction they can form, metal–metal, metal–insulator, or insulator–insulator. Interestingly, despite metallic materials being more abundant, the majority of these simple heterostructures form metal–insulator, instead of metal–metal, junctions. Still, more than 900 (out of 5989) are made of two layers both exhibiting semiconducting or insulating behavior that could be crucially relevant for photocatalytic and optoelectronic applications.^67,68^

### Large-Bandgap 2D Insulators

At the extreme edge of the distribution in Figure 10 we can observe a few materials with large bandgaps that could be very promising as insulating layers in nanoscale electronic devices. In a recent work^33^ it has been systematically shown how properties such as the bandgap, the dielectric constant, and the effective masses of the insulating capping layer could influence the performance of ultrascaled tunneling transistors realized with 2D channels, especially through their influence on the leakage current (thus the off-current of the device) and carrier mobility. In particular, the leakage current is exponentially suppressed by large tunneling masses as well as high energy barriers, directly linked to a large bandgap. The role of the dielectric constant is more complex, and a tradeoff needs to be found between two competing effects. On the one hand, a large dielectric constant ensures small leakage currents and a better gate control, but on the other hand, it can degrade the high mobility of the channel through interfacial optical phonon scattering. Finally, layered materials, able to form a clean vdW interface, are more promising than 3D or amorphous counterparts since local defects such as dangling bonds at the interface can act as an additional short-range scattering mechanism. This is particularly crucial for transistors relying on 2D semiconductors as channels, since their extreme thinness makes them highly sensitive to their surroundings. According to these considerations, van der Waals layered materials with a large bandgap (approximately around 12 eV), with large effective masses and a moderate static dielectric constant (around 10), would be the ideal candidates to realize the insulating layer in ultrascaled devices.

Unfortunately materials with such characteristics are extremely rare, and materials with lower bandgaps like the widely used hBN are insufficient to reach the desired scaling and performances. With this application in mind, here we study in further detail the materials that present the largest bandgaps at the DFT level in our screening, namely B_2_F_8_Na (7.5 eV), F_2_H_6_O_2_ (7.1 eV), and B_2_O_9_S_2_ (6.9 eV). All these materials are dynamically stable, as proven by their phonon dispersions (see the Supporting Information), and have dielectric constants of 4.7ϵ_0_, 5.8ϵ_0_, and 5.15ϵ_0_, respectively (comparable to or slightly higher than those of h-BN, whose values have been reported in a range between 3.76ϵ_0_^69^ and 5.06ϵ_0_,^70^ 4.7ϵ_0_ for us, when computed with the same scheme used for the other three materials). However, they all show significantly larger bandgaps with h-BN reaching only 4.7 eV when computed with the same level of theory. Moreover, they have only slightly larger effective masses, computed from the curvature of the band structure at the band edges: (*m*_*e*1_^*^ = 0.65, *m*_*e*2_^*^ = 0.64), (*m*_*e*1_^*^ = 0.57, *m*_*e*2_^*^ = 0.55), and (*m*_*e*1_^*^ = 0.64, *m*_*e*2_^*^ = 0.52), respectively, for the electrons. They instead display extremely large and anisotropic effective masses for the holes (*m*_*h*1_^*^ = 17.7, *m*_*h*2_^*^ = 34.1), (*m*_*h*1_^*^ = 5.38, *m*_*h*2_^*^ = 47.9), and (*m*_*h*1_^*^ = 2.86, *m*_*h*2_^*^ = 7.69) compared to 2D h-BN (m* = 0.54 for both holes and electrons in all directions, when computed with the same method). Although these effective masses differ in their definition from the tunneling masses used in ref (33), their relative value can nonetheless be used as a rough comparison. These figures indicate that the proposed materials should outperform h-BN as insulating layers as well as other proposed 2D insulators with smaller bandgaps such as GaS and TiO_2_,^33^ while still maintaining the possibility of forming ideal vdW interfaces, and they thus deserve more in-depth theoretical and experimental studies to assess their performance under operational conditions.

## Conclusions

In conclusion, in this work we screened for two-dimensional monolayers that can be exfoliated from experimentally known stoichiometric materials, including a new database of experimental structures (MPDS)^30,31^ as well as the most up-to-date versions of the two experimental databases already included in our previous study.^24,26^ This work has led to the discovery of 1252 additional monolayers, bringing the total to 3077 compounds and, notably, almost doubling the number of easily exfoliable materials (2004) compared with our previous survey. Moreover, we optimized the structural properties of each material treated as an isolated monolayer and studied its electronic properties. On the basis of the optimized geometries, for the subset of materials with up to 6 atoms in the unit cell, we suggest all possible combinations that can give rise to lattice-matched lateral or vertical heterostructures, identifying a group of 5989 pairs that can be realized with a simple 1 × 1 match and a very small strain. We computed and analyzed the electronic band structure at the PBE level for each compound and studied the distribution of the fundamental electronic properties throughout the database and within the most common prototype classes. Finally, we highlighted a handful of large-gap insulating materials that could possibly outperform boron nitride as insulating layers for ultrascaled transistors. All the information about the materials and properties of this study are presented in the Supporting Information and are available on the Materials Cloud in the Discover, Explore, and Archive^37,53^ sections for the benefit of the experimental and theoretical community.

This bulk of data can provide indications of the feasibility and possible quality of the exfoliation and provides a starting point for a treasure trove of materials with specific properties and functionalities to be studied in depth either in their isolated 2D form or possibly as part of an optimal heterostructure. Moreover, it could be used to train machine-learning models targeted at certain relations between properties and structures (like the exfoliation energies or electronic properties) or as a starting point for a further database expansion based upon generative models.

## Methods

We use the Quantum ESPRESSO^71,72^ package with the SSSP PBE efficiency pseudopotentials library (version 1.0),^73^ a library of carefully tested pseudopotentials from different sources.^74−79^ For each material, the wave function and charge-density cutoffs are chosen as the highest suggested by the SSSP library for all the elements in the compound. For bulk materials we use two properly vdW-corrected functionals, namely the vdW-DF2 functional^43^ with c09 exchange^44,45^ (DF2-c09) and the revised Vydrov–Van Voorhis^46−48^ (rVV10) functional. Only vdW-DF2-c09 is used for the materials added in this study. Sampling of the Brillouin zone is performed using a Γ-centered Monkhorst–Pack grid,^80^ with the smallest number of *k*-points in each direction of the reciprocal lattice guaranteeing a spacing of at least 0.2 Å^–1^. All relaxations and binding energies of 3D structures are computed using a Marzari–Vanderbilt–DeVita–Payne cold smearing^81^ of 0.02 Ry. The volume is optimized, letting all the cell vectors and angles free to move until the pressure is below 0.5 kbar and the internal forces are lower than 10^–3^ au. When optimizing and computing the electronic properties of isolated 2D units, a vacuum space of 20 Å is used along the orthogonal direction and a variable cell relaxation involving only the in-plane coordinates is performed under open-boundary conditions^55^ and using the PBE^56^ functional with the same pseudopotentials and equivalent *k*-point sampling. Band structures are computed along high-symmetry paths with a *k*-point density of 0.01 Å^–1^. All the structures, even the ones with atoms that might allow a magnetically ordered ground state, are treated as nonmagnetic in a spin-unpolarized approximation.

## Supporting Information Available

Optimized structure, the electronic bands, the binding energies, and information on the parent 3D material for all the materials up to 12 atoms per unit cell and tables of lattice-matching materials with the smallest strain and smallest number of atoms in the resulting heterostructure for all the materials up to 6 atoms per unit cell (PDF)
