# Supplementary material for: Expansion of the Materials Cloud 2D Database
Source: ACS Nano. 2023 Jun 13;17(12):11268–78. doi: 10.1021/acsnano.2c11510 (PMC10403156; doi:10.1021/acsnano.2c11510)
Supplement: Supplementary file 1 — nn2c11510_si_001.pdf [file nn2c11510_si_001.pdf]

Supplementary information for "Expansion of the Materials Cloud 2D database"

Davide Campi,*,†,‡ Nicolas Mounet,†,¶ Marco Gibertini,†,§,∥ Giovanni Pizzi,†,⊥ and Nicola Marzari*,†,⊥

†*Theory and Simulation of Materials (THEOS), and National Centre for Computational Design and Discovery of Novel Materials (MARVEL), École Polytechnique Fédérale de Lausanne, CH-1015 Lausanne, Switzerland*

‡*Dipartimento di Scienza dei Materiali, University of Milano-Bicocca, Via R.Cozzi 55, 20125 Milano, Italy*

¶*Current adrress: CERN - Esplanade des Particules 1, CH-1217 Meyrin, Switzerland*

§*Dipartimento di Scienze Fisiche, Informatiche e Matematiche, University of Modena and Reggio Emilia, I-41125 Modena, Italy*

∥*Centro S3, Istituto di Nanoscienze - CNR, I-41125 Modena, Italy*

⊥*Laboratory for Materials Simulations (LMS), Paul Scherrer Institut, CH-5232 Villigen PSI, Switzerland*

E-mail: davide.campi@unimib.it; nicola.marzari@epfl.ch

Structure of the document The document is divided into five sections, in the first one are reported the supplementary figures referenced in the main text, the other four sections include lists of all the materials, divided according to their binding energies (easily or potentially exfoliable) and the number of atoms in the unit cell (up to 6 atoms per unit cell for the first two sections and from 7 up yo 12 atoms per unit cell for the last two). For each one we report a summary detailing its provenance, structure, and all the computed properties.

Pages in each section are numbered independently from the others.

Supplementary figures

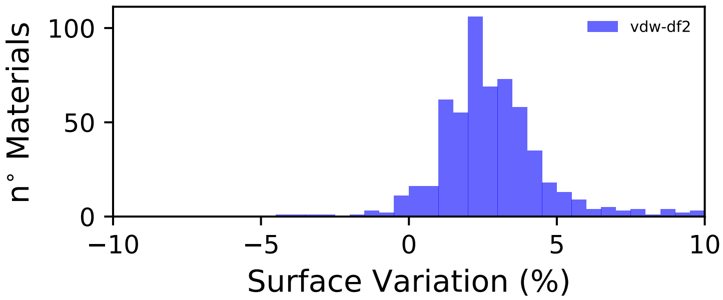

Figure 1: Distribution of the surface area difference for a sample of 300 materials optimized with a variable cell relaxation as isolated 2D structures using the PBE functional and the vdW-df2-c09 functional. The distribution is similar to the observed in Fig.6 of the main text, suggesting that a great part of the variation can be ascribed to the differences in the functional rather than the dimensionality change.

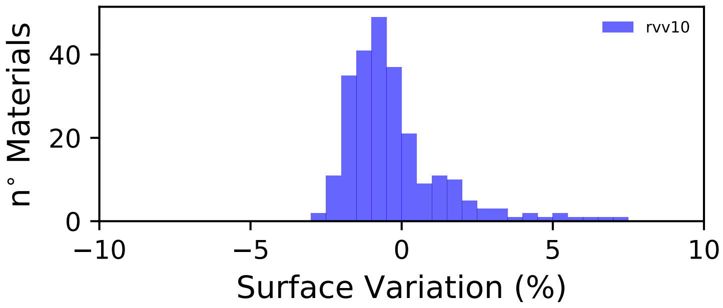

Figure 2: Distribution of the surface area difference for a sample of 300 materials optimized with a variable cell relaxation as isolated 2D structures using the PBE functional and the rvv10 functional. The distribution is similar to the observed in Fig.6 of the main text, suggesting that a great part of the variation can be ascribed to the differences in the functional rather than the dimensionality change.

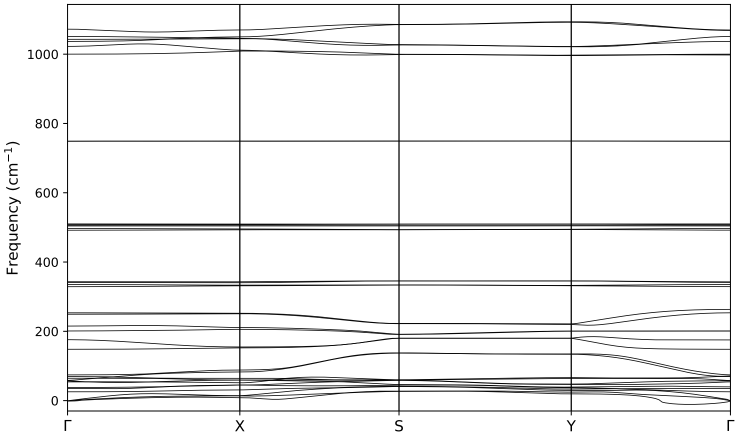

Figure 3: Phonon dispersion for $B_2Na_2F_8$.

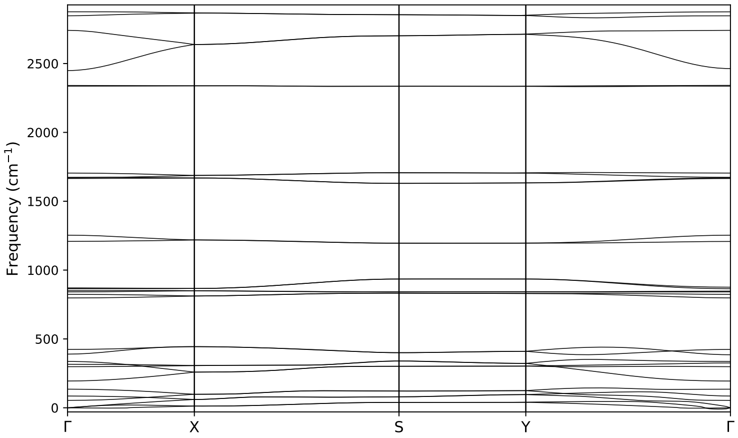

Figure 4: Phonon dispersion for $F_2H_6O_2$.

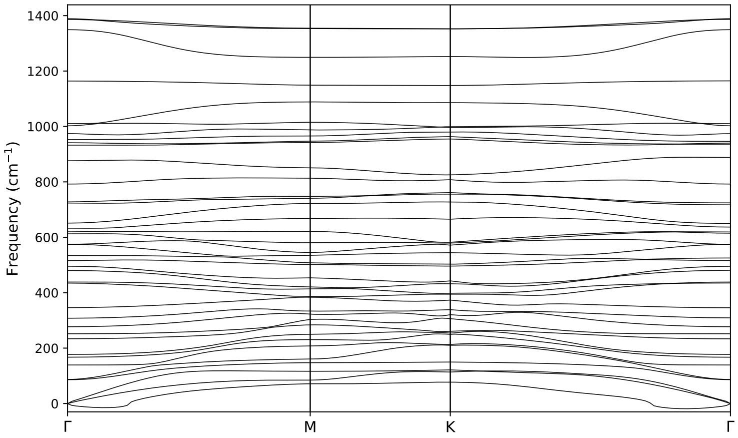

Figure 5: Phonon dispersion for $B_2O_9S_2$.

Definitions and details

- All the properties are computed at the DFT-PBE level. The only exceptions are binding energies, which were calculated using the DF2-C09 and the rVV10 van-der-Waals functionals. All 3D and 2D structures are treated as non-magnetic using spin-unpolarized DFT. The magnetic order has a negligible effect on the binding energies as discussed in Ref.[1] but caution is needed when looking at the electronic properties of materials with elements that might support a magnetic ground state. The magnetic properties of a subset of materials, easily exfoliable with at most 6 atoms per unit cell, have been computed in Ref.[1] and can be browsed at https://www.materialscloud.org/discover/mc2d/dashboard/ptable or downloaded https://doi.org/10.24435/materialscloud:2017.0008/v1. For consistency with the rest of the database, these materials are reported in this archive in their non-magnetic state.

- Binding energies are calculated as total energy differences between relaxed 3D parents and as-exfoliated, unrelaxed 2D children. More details can be found in the Methods section of the main paper.

- Each 2D structure has at least one 3D parent structure. In those cases when the monolayers can be exfoliated from more than one parent, for each functional we choose as parent the one with the lowest binding energy, and then we select the functional giving the most favourable 2D classification, with a preference for DF2-C09 when an ambiguity persists. The list of all possible 3D parents of a given monolayer is reported in the dataset.

- In metals, the zero for energy bands is the Fermi energy, while for semiconductors the zero is set midway between the highest occupied state in the valence bands and the lowest unoccupied state in the conduction bands. For simplicity, we call this Fermi energy in the captions. Calculations have been performed with a finite smearing that might slightly influence its position

- Paths and special k-points follow the conventions for 2D systems from Ref. [2] as implemented in AiiDA [3,4].

References:

[1] N. Mounet, M. Gibertini, P. Schwaller, D. Campi, A. Merkys, A. Marrazzo, T. Sohier, I. E. Castelli, A. Cepellotti, G. Pizzi, N. Marzari, Two-dimensional materials from high-throughput computational exfoliation of experimentally known compounds, Nature Nanotech., (2018). DOI:10.1038/s41565-017-0035-5.

[2] R. Ramírez and M. C. Bohm, Simple geometric generation of special points in Brillouin-zone integrations. Two-dimensional Bravais lattices. Int. J. Quantum Chem. 30, 391-411 (1986).

[3] G. Pizzi, A. Cepellotti, R. Sabatini, N. Marzari, and B. Kozinsky. AiiDA: automated interactive infrastructure and database for computational science. Computational Materials Science 111, 218 - 230 (2016).

[4] S.P. Huber, S. Zoupanos, M. Uhrin, L. Talirz, L. Kahle, R. Häuselmann, D. Gresch, T. Müller, A. V. Yakutovich, C. W. Andersen, F. F. Ramirez, C. S. Adorf, F. Gargiulo, S. Kumbhar, E. Passaro, C. Johnston, A. Merkys, A. Cepellotti, N. Mounet, N. Marzari, B. Kozinsky, and G. Pizzi. AiiDA 1.0, a scalable computational infrastructure for automated reproducible workflows and data provenance. Scientific Data 7, 300 (2020).

Easily exfoliable materials up to 6 atoms per unit cell

Contents

AgBr (P2$_1$/m) . . . 4
AgF_2 (P2$_1$/c) . . . 6
AgI (P4/nmm) . . . 8
$AgNO_2$ (P1) . . . 10
AgO_2Br (P1) . . . 12
AgO_2Cl (P1) . . . 14
AgO_4Cl (P-42m) . . . 16
AlOCl (Pmmn) . . . 18
As (Pmna) . . . 20
$AsSe_2$ (P3m1) . . . 22
AuBr (Cmme) . . . 24
AuI (Cmme) . . . 26
AuSe (P2/m) . . . 28
BN (P-6m2) . . . 30
$Ba_2I_2F_2$ (P4/nmm) . . . 32
BaF_2 (P3m1) . . . 34
BaHI (P4/nmm) . . . 36
Bi (P-3m1) . . . 38
Bi_2Se_2 (C2/m) . . . 40
Bi_2Se_3 (P-3m1) . . . 42
Bi_2Te_2S (P-3m1) . . . 44
Bi_2Te_2S (P3m1) . . . 46
Bi_2Te_2Se (P-3m1) . . . 48
Bi_2Te_3 (P-3m1) . . . 50
Bi_2TeSe_2 (P-3m1) . . . 51
BiOBr (P4/nmm) . . . 53
BiOCl (P4/nmm) . . . 55
BiOI (P4/nmm) . . . 57
BiTeBr (P3m1) . . . 59
BiTeCl (P3m1) . . . 61
BiTeI (P3m1) . . . 63
C (P6/mmm) . . . 65
C_2F_2 (P-3m1) . . . 67
CLu_2Br_2 (P-3m1) . . . 69
CLu_2Cl_2 (P-3m1) . . . 71
$Ca(OH)_2$ (P-3m1) . . . 73
$Ca_2Br_2F_2$ (P4/nmm) . . . 75
Ca_2O_2 (P1) . . . 77
CaHBr (P4/nmm) . . . 79
CaHI (P4/nmm) . . . 81
CaI_2 (P-3m1) . . . 83
$Cd(OH)_2$ (P-3m1) . . . 85
$CdBr_2$ (P-3m1) . . . 87
$CdCl_2$ (P-3m1) . . . 89
CdI_2 (P-3m1) . . . 91
CdIBr (P3m1) . . . 93
CdOCl (P3m1) . . . 95
$Ce_2O_2Br_2$ (P4/nmm) . . . 97
$Ce_2S_2I_2$ (P-3m1) . . . 99
CeI_2 (P4/mmm) . . . 101
CeSiI (P-3m1) . . . 103
$Co(OH)_2$ (C2/m) . . . 105
$CoBr_2$ (P-3m1) . . . 107
$CoCl_2$ (P-3m1) . . . 109
CoI_2 (C2/m) . . . 111
CoO_2 (P-3m1) . . . 113
Cr_2O_4 (Pmmn) . . . 115
$CrBr_2$ (P-1) . . . 117
CrI_2 (P2$_1$/m) . . . 119
CrO_2 (P-3m1) . . . 121
CrOBr (Pmmn) . . . 123
CrOCl (Pmmn) . . . 125
CrSBr (Pmmn) . . . 127
$CrSe_2$ (C2/m) . . . 129
Cu_2O_2 (P1) . . . 131
Cu_2O_4 (Pmmn) . . . 133
Cu_2Te (C2/m) . . . 135
$Cu_2Tl_2Se_2$ (P4/nmm) . . . 137
Cu_4Te_2 (C2/m) . . . 139
$CuAgTe_2$ (Pm) . . . 141
CuBr (P4/nmm) . . . 143
$CuBr_2$ (C2/m) . . . 145
$CuCl_2$ (C2/m) . . . 147
$CuGeO_3$ (Pm) . . . 149
CuI (P-3m1) . . . 151
CuO_2 (P-1) . . . 153
CuTe (Pmmn) . . . 155
$Dy_2O_2Br_2$ (P4/nmm) . . . 157
$Dy_2S_2I_2$ (P-3m1) . . . 159
DyI_2 (P-3m1) . . . 161
DySBr (Pmmn) . . . 163
DySI (Pmmn) . . . 165
Er_2Br_2 (P-3m1) . . . 167
$Er_2O_2Br_2$ (P4/nmm) . . . 169
$Er_2O_2Cl_2$ (P-3m1) . . . 171
$Er_2O_2I_2$ (P4/nmm) . . . 173
$Er_2S_2Br_2$ (Pmm2) . . . 175
$Er_2S_2I_2$ (Pmm2) . . . 177
$Er_2Se_2Br_2$ (Pmm2) . . . 179
$Er_2Se_2F_2$ (P-3m1) . . . 181
ErHCl (P-3m1) . . . 183
ErSCl (Pmmn) . . . 185
ErSeI (Pmmn) . . . 187
$Eu_2H_2I_2$ (P4/nmm) . . . 189
$Eu_2I_2F_2$ (P4/nmm) . . . 191
EuOBr (P4/nmm) . . . 193
Fe_2TeSe (P4mm) . . . 195
$FeBr_2$ (P-3m1) . . . 197
$FeCl_2$ (P-3m1) . . . 199
FeI_2 (P-3m1) . . . 201
FeO_2 (P-3m1) . . . 203
FeO_2 (Pmmn) . . . 205
FeOCl (Pmmn) . . . 207
FeS (P4/nmm) . . . 209
FeSe (P4/nmm) . . . 211
FeTe (P4/nmm) . . . 213
Ga_2S_3 (P-3m1) . . . 215
Ga_2Se_2 (P3m1) . . . 217
$Ga_2Te_2Br_2$ (Pm) . . . 219
GaGeTe (P-3m1) . . . 221
GaS (P-3m1) . . . 223
GaS (P-6m2) . . . 225
GaSe (P-6m2) . . . 227
GaTe (P-6m2) . . . 229
GaTeCl (Pmn2$_1$) . . . 231
Gd (Pmmm) . . . 233
Gd_2Br_2 (P-3m1) . . . 235
$Gd_2C_2Br_2$ (C2/m) . . . 237
Gd_2CCl_2 (P-3m1) . . . 239
Gd_2Cl_2 (P-3m1) . . . 241
$Gd_2Ga_2I_2$ (P-3m1) . . . 243
Gd_2GeBr_2 (P-3m1) . . . 245
Gd_2GeI_2 (P-3m1) . . . 247
Gd_2I_2 (P-3m1) . . . 249
$Gd_2O_2Br_2$ (P4/nmm) . . . 251
$Gd_2S_2I_2$ (Pmm2) . . . 253
$Gd_2S_2I_2$ (P-3m1) . . . 255
$Gd_2Se_2I_2$ (Pmm2) . . . 257
GdI_2 (P-6m2) . . . 259
GeI_2 (P-3m1) . . . 261
GeI_2 (P-6m2) . . . 263
GeS (Pmn2$_1$) . . . 265
GeS_2 (P-4m2) . . . 267
GeSe (Pmn2$_1$) . . . 269
Hf_2Br_2 (P-3m1) . . . 271
Hf_2Cl_2 (P-3m1) . . . 273
Hf_3Te_2 (P4/mmm) . . . 275
HfNBr (P-3m1) . . . 277
HfNBr (Pmmn) . . . 279
HfNCl (P-3m1) . . . 281
HfNI (P-3m1) . . . 283
HfS_2 (P-3m1) . . . 285
$HfSe_2$ (P-3m1) . . . 287
HfSiTe (P4/nmm) . . . 289
$HfTe_2$ (P-3m1) . . . 291
HgI_2 (P-4m2) . . . 293
Ho_2Br_2 (P-3m1) . . . 295
$Ho_2O_2Br_2$ (P4/nmm) . . . 297
$Ho_2O_2Cl_2$ (P-3m1) . . . 299
$Ho_2S_2Br_2$ (Pmm2) . . . 301
$Ho_2Se_2F_2$ (P-3m1) . . . 303
$Ho_2Se_2I_2$ (Pmm2) . . . 305
In_2Se_3 (C2) . . . 307
InOBr (Pmmn) . . . 309
InSe (C2/m) . . . 311
InSe (P-6m2) . . . 313
$K_2Cd_2As_2$ (P4/nmm) . . . 315
KAgSe (P4/nmm) . . . 317
$KTlCl_4$ (P-4) . . . 319
KTlO (P2$_1$/m) . . . 321
La_2AsI_2 (P-3m1) . . . 323
$La_2C_2Br_2$ (C2/m) . . . 325
$La_2C_2I_2$ (C2/m) . . . 327
La_2GeI_2 (P-3m1) . . . 329
La_2I_2 (P-3m1) . . . 331
La_2PBr_2 (P-3m1) . . . 333
La_2PI_2 (P-3m1) . . . 335
La_2S_2 (Pmm2) . . . 337
La_2SbI_2 (P-3m1) . . . 339
$La_2Si_2I_2$ (P-3m1) . . . 341
La_2TeI_2 (P-3m1) . . . 343
$LaBr_2$ (P-6m2) . . . 345
LaCl (P-3m1) . . . 347
LaGeI (P-3m1) . . . 349
$LaHBr_2$ (P-6m2) . . . 351
LaI_2 (P4/mmm) . . . 353
LaOBr (P4/nmm) . . . 355
LaOI (P4/nmm) . . . 357

$LiAlTe_2$ (P3m1) . . 359
$LiAuI_4$ (P-1) 361
$LiBH_4$ (C2) 363
LiOH (P4/nmm) . . 365
LuHCl (P-3m1) . . . 367
$Mg(OH)_2$ (P-3m1) . 369
Mg_2 (P4/nmm) . . . 371
Mg_2Cl_4 (P2/m) . . . 373
Mg_2Cl_4 (P-1) 375
Mg_3 (P4/mmm) . . 377
Mg_4 (P4/nmm) . . . 379
Mg_6 (P4/nmm) . . . 381
$MgBr_2$ (P-3m1) . . . 383
$MgCl_2$ (P-3m1) . . . 385
MgI_2 (P-3m1) 387
$MnBr_2$ (C2/m) . . . 389
$MnCl_2$ (C2/m) . . . 391
MnI_2 (C2/m) 393
MnO_2 (P-3m1) . . . 395
MoS_2 (P-3m1) . . . 397
MoS_2 (P-6m2) . . . 399
$MoSe_2$ (P-6m2) . . . 401
$MoTe_2$ ($P2_1/m$) . . . 403
$MoTe_2$ (P-6m2) . . . 405
N_4 ($Pmn2_1$) 407
Na_2PdH_2 (P4/mmm) 409
$NaAlH_4$ (P-4) 411
NaCN (Pmm2) . . . 413
NaOH (Pmmn) . . . 415
Nb_2CS_2 (P-3m1) . . 417
NbF_4 (P4/mmm) . . 419
NbS_2 (P-3m1) 421
NbS_2 (P-6m2) 423
$NbSe_2$ (P-6m2) . . . 425
$NbSe_2$ (P-3m1) . . . 427
$NbTe_2$ (P-3m1) . . . 429
$Nd_2O_2I_2$ (P4/nmm) 431
$Nd_2S_2I_2$ (P-3m1) . . 433
NdI_2 (P4/mmm) . . 435
NdOBr (C2/m) . . . 437
$NiBr_2$ (P-3m1) . . . 439
$NiCl_2$ (P-3m1) . . . 441
NiI_2 (P-3m1) 443
NiO_2 (P-3m1) 445
OH_3Cl (P3m1) . . . 447
OLuBr (P4/nmm) . 449
OLuI (P4/nmm) . . 451
$OsOCl_2$ (Pmmm) . . 453
P (Pmna) 455
P (P-3m1) 457
PbBrF (P4/nmm) . 459
PbClF (P4/nmm) . 461
PbF_4 (P4/mmm) . . 463
PbI_2 (P-3m1) 465
PbIF (P4/nmm) . . 467
PbO (P4/nmm) . . . 469
PbTe (P3m1) 471
$PdCl_2$ ($P2_1/c$) . . . 473
PdS_2 ($P2_1/c$) 475
Pr_2Br_2 (P-3m1) . . . 477
$Pr_2S_2I_2$ (P-3m1) . . 479
$Pr_2Si_2I_2$ (P-3m1) . . 481
PrI_2 (P-6m2) 483
PrI_2 (P4/mmm) . . 485
PrOBr (P4/nmm) . 487
PtO_2 (P-3m1) 489
PtS_2 (P-3m1) 491
$PtSe_2$ (P-3m1) . . . 493
RbCl (P4/nmm) . . 495
$ReSe_2$ (P-6m2) . . . 497
RhTeCl ($P2_1/m$) . . 499
$RuOCl_2$ (Pmmm) . . 501
$S_2Lu_2Br_2$ (Pmm2) . 503
$S_2Lu_2I_2$ (Pmmn) . . 505
Sb (P-3m1) 507
Sb_2Te_2S (P-3m1) . . 509
Sb_2Te_2Se (P-3m1) . 511
Sb_2Te_3 (P-3m1) . . . 513
Sb_2TeSe_2 (P-3m1) . 515
Sb_2TeSe_2 (P3m1) . . 517
SbTeI (C2/m) 519
Sc_2CCl_2 (P-3m1) . . 521
Sc_2NCl_2 (P-3m1) . . 523
$Sc_2O_2Cl_2$ (Pmmn) . 525
ScCl (P-3m1) 527
ScHCl (P-3m1) . . . 529
ScOBr (Pmmn) . . . 531
$Se_2Lu_2F_2$ (P-3m1) . 533
$Se_2Lu_2I_2$ (Pmmn) . 535
SiH (P-3m1) 537
$SiTe_2$ (P-3m1) . . . 539
Sm (P6/mmm) . . . 541
$Sm_2I_2F_2$ (P4/nmm) 543
$Sm_2O_2I_2$ (P4/nmm) 545
SnF_4 (P4/mmm) . . 547
SnO (C2/m) 549
SnO (P4/nmm) . . . 551
SnS_2 (P-3m1) 553
$SnSe_2$ (P-3m1) . . . 555
SnTe ($Pmn2_1$) . . . 557
SrBrF (P4/nmm) . . 559
SrHBr (P4/nmm) . . 561
SrHI (P4/nmm) . . 563
SrI_2 (Pmmn) 565
Ta_2CS_2 (P-3m1) . . 567
$TaNS_2$ (P3m1) . . . 569
TaS_2 (P-3m1) 571
TaS_2 (P-6m2) 573
$TaSe_2$ (P-3m1) . . . 575
$TaSe_2$ (P-6m2) . . . 577
$TaTe_2$ (P-3m1) . . . 579
$Tb_2C_2Br_2$ (C2/m) . 581
$Tb_2Ga_2I_2$ (P-3m1) . 583
$Tb_2O_2Br_2$ (P4/nmm) 585
$Tb_2S_2I_2$ (Pmm2) . . 587
$Tb_2Se_2I_2$ (Pmm2) . 589
TbCl (P-3m1) 591
Ti_2PTe_2 (P-3m1) . . 593
$TiBr_2$ (P-3m1) . . . 595
$TiCl_2$ (P-3m1) . . . 597
TiH_4 (P4/mmm) . . 599
TiI_2 (P-3m1) 601
TiNBr (Pmmn) . . . 603
TiNCl (Pmmn) . . . 605
TiNI (Pmmn) 607
TiOBr (Pmmn) . . . 609
TiOCl (Pmmn) . . . 611
TiS_2 (P-3m1) 613
$TiSe_2$ (P-3m1) . . . 615
$TiTe_2$ (P-3m1) . . . 617
Tl_2O (P-3m1) 619
Tl_2S (P-3m1) 621
$Tl_2S_2I_2$ (P4/nmm) . 623
TlF (P4/nmm) . . . 625
$Tm_2Br_2F_2$ (P4/nmm) 627
$Tm_2O_2Br_2$ (P4/nmm) 629
$Tm_2O_2Cl_2$ (P-3m1) 631
$Tm_2S_2I_2$ (Pmm2) . . 633
$Tm_2Se_2F_2$ (P-3m1) . 635
$Tm_2Se_2I_2$ (Pmm2) . 637
TmI_2 (P-3m1) . . . 639
TmOI (C2/m) . . . 641
VBr_2 (C2/m) 643
VCl_2 (C2/m) 645
VI_2 (C2/m) 647
VOBr (Pmmn) . . . 649
VOCl (Pmmn) . . . 651
VS_2 (P-3m1) 653
VSe_2 (P-3m1) 655
VTe_2 (P-3m1) 657
W_2N_3 (P-6m2) . . . 659
WS_2 (P-6m2) 661
WSe_2 (P-6m2) . . . 663
WTe_2 ($P2_1/m$) . . . 665
WTe_2 (P-6m2) . . . 667
Y_2Br_2 (P-3m1) . . . 669
$Y_2C_2Cl_2$ (C2/m) . . 671
Y_2CBr_2 (P-3m1) . . 673
Y_2CI_2 (P-3m1) . . . 675
Y_2GeI_2 (P-3m1) . . 677
Y_2I_2 (P-3m1) 679
$Y_2O_2Br_2$ (P4/nmm) 681
$Y_2O_2I_2$ (P4/nmm) . 683
Y_2PBr_2 (P-3m1) . . 685
$Y_2S_2Br_2$ (Pmm2) . . 687
YCBr (C2/m) 689
YCI (C2/m) 691
YCl (P-3m1) 693
YGaI (P-3m1) . . . 695
YOCl (P-3m1) . . . 697
$Yb_2Br_2F_2$ (P4/nmm) 699
$Yb_2H_2Br_2$ (P4/nmm) 701
$Yb_2I_2F_2$ (P4/nmm) 703
$Yb_2S_2Br_2$ (Pmm2) . 705
$Yb_2S_2I_2$ (Pmm2) . . 707
$Yb_2Se_2F_2$ (P-3m1) . 709
$Yb_2Se_2I_2$ (Pmm2) . 711
YbI_2 (P-3m1) 713
YbOBr (Cmme) . . 715
YbOCl (P-3m1) . . . 717
$ZnBr_2$ (P-3m1) . . . 719
$ZnCl_2$ (P-3m1) . . . 721
$ZnCl_2$ (P-4m2) . . . 723
ZnI_2 (P-3m1) 725
$Zr_2H_2Br_2$ (P-3m1) . 727
$Zr_2H_2Br_2$ (P-3m1) . 729
$Zr_2H_2Cl_2$ (P-3m1) . 731
$Zr_2N_2Cl_2$ (Pmmn) . 733
Zr_2PTe_2 (P-3m1) . . 735
ZrBr (P-3m1) 737
ZrCl (P-3m1) 739
$ZrCl_2$ (P-6m2) . . . 741
ZrI_2 ($P2_1/m$) 743
ZrNBr (P-3m1) . . . 745
ZrNBr (P3m1) . . . 747
ZrNBr (Pmmn) . . . 749
ZrNCl (P3m1) . . . 751
ZrNCl (P-3m1) . . . 753
ZrNI (P-3m1) 755
ZrNI (Pmmn) 757
$ZrNS_2$ (P3m1) . . . 759
ZrS_2 (P-3m1) 761
$ZrSe_2$ (P-3m1) . . . 763
ZrSiTe (P4/nmm) . 765
$ZrTe_2$ (P-3m1) . . . 767
$ZrTiSe_4$ (P2) 769
$ZrTiTe_4$ (P2/m) . . 771

AgBr ($P2_1/m$)

Structural and electronic properties

**Formula**	AgBr
**Spacegroup**	$P2_1/m$
**Prototype**	AgBr
**Parent 3D**	$Ag_2Pb_2O_2Br_2$
**Source DB**	ICSD
**DB ID**	33913
**DF2-C09 Binding energy [meV/ Å^2]**	7.84
**RVV10 Binding energy [meV/ Å^2]**	14.32
**Band gap (PBE) [eV]**	1.26

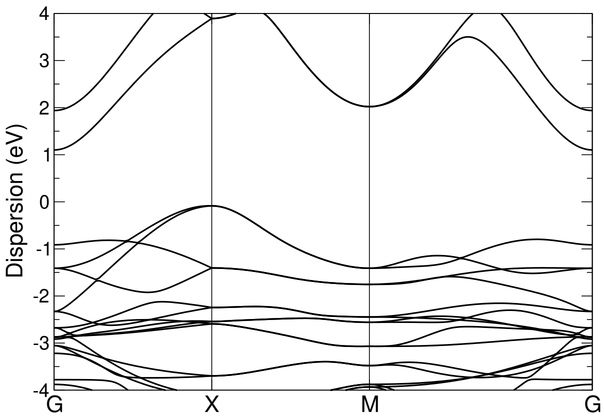

Band structure: Electronic band structure of AgBr ($P2_1/m$) in a representative window around the Fermi energy and along a high-symmetry path.

Optimized crystal structure

Structural parameters: relaxed cell (top) and atomic positions (bottom) of AgBr ($P2_1/m$) in Cartesian coordinates.

	x [Å]	y [Å]	z [Å]
$\mathbf{a_1}$	3.95797861	0.00000000	0.00000000
$\mathbf{a_2}$	−0.00000000	3.95797861	0.00000000
$\mathbf{a_3}$	0.00000000	0.00000000	23.27354698

		x [Å]	y [Å]	z [Å]
●	Ag	1.97898930	0.00000000	10.47019933
●	Ag	-0.00000000	1.97898930	12.80334765
●	Br	1.97898930	0.00000000	13.34766873
●	Br	-0.00000000	1.97898930	9.92587825

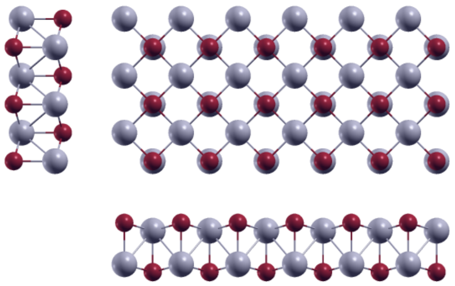

Orthographic projections: views of AgBr ($P2_1/m$) as seen from the x axis (left), the y axis (bottom) and the z axis (center).

Lattice matching - minimal size

Formula	N° atoms	strain	cell size 1	cell size 2
K	5	0.1764	1	1
InSe	6	0.1401	1	1
Bi_2	6	0.1452	1	1
AgTl	6	0.0173	1	1
Ag_2	6	0.1831	1	1
LiO	6	0.1111	1	1
P_2	6	0.1094	1	1
PbTe	6	0.1416	1	1
I_2Mg	7	0.132	1	1
CdI_2	7	0.1431	1	1
Nd	7	0.1907	1	3
Ba_2Pt	7	0.1828	1	1
Br_2Zn	7	0.1089	1	1
Br_2Ca	7	0.1442	1	1
CaI_2	7	0.1658	1	1
I_2Pr	7	0.0061	1	1
Br_2La	7	0.1322	1	1
Br_2Cu	7	0.1029	1	1
Ca_2Si	7	0.1885	1	1
I_2Yb	7	0.163	1	1
BiClTe	7	0.1435	1	1
Cl_2Ti	7	0.1094	1	1
BrCdI	7	0.1343	1	1
HgI_2	7	0.4009	1	1
Te_2Ti	7	0.1089	1	1
BaF_2	7	0.137	1	1
BiBrTe	7	0.1485	1	1
GeI_2	7	0.1306	1	1
AsKSn	7	0.1358	1	1
$PbTe_2$	7	0.1336	1	1
I_2Nd	7	0.0052	1	1
Cl_2Cu	7	0.0974	1	1
I_2Tm	7	0.1645	1	1
GeI_2	7	0.1418	1	1
I_2Pb	7	0.1851	1	1
STl_2	7	0.1377	1	1
BiITe	7	0.1549	1	1
GeS_2	7	0.2114	1	1
DyI_2	7	0.169	1	1
CeI_2	7	0.0069	1	1
Se_2Yb	7	0.1308	1	1
$BiTe_2$	7	0.131	1	1
GdI_2	7	0.1512	1	1
I_2La	7	0.0005	1	1
$CrSe_2$	7	0.1114	1	1
CdI_2	7	0.1428	1	1
I_2Pr	7	0.1435	1	1
$HfSe_2$	7	0.1089	1	1
Bi_2Te_2	8	0.7742	1	1
Bi_2In_2	8	1.1722	1	1

Table showing the first 50 lattice matching combinations with the smallest number of atoms in the supercell.

Lattice matching - minimal strain

Formula	N° atoms	strain	cell size 1	cell size 2
$H_2Li_2O_2$	934	0.0001	82	101
$Hf_2Se_2Si_2$	742	0.0001	64	81
$Ba_2F_2I_2$	550	0.0001	64	49
As_2Rh_2	8	0.0003	1	1
Br_2CsF	340	0.0003	49	36
$Br_2Lu_2S_2$	34	0.0004	4	3
$Cu_2K_2Te_2$	962	0.0004	113	85
As_2Fe_2	580	0.0004	64	81
$Br_2Lu_2S_2$	34	0.0004	4	3
$Br_2O_2Pr_2$	10	0.0005	1	1
Tl	89	0.0005	16	25
Mg_3	995	0.0005	113	181
I_2La	7	0.0005	1	1
$H_2Li_2O_2$	924	0.0006	81	100
O_4PTl	550	0.0006	64	49
O_2Zn	197	0.0006	20	39
HgI_2	919	0.0006	145	113
$Br_2N_2Zr_2$	582	0.0007	48	65
CBr_2Lu_2	517	0.0007	48	65
Mg_2	426	0.0007	58	97
P_4	284	0.0008	35	36
Mg_4	164	0.0008	16	25
$Cl_2H_2Lu_2$	582	0.0009	48	65
O_2Sn_2	920	0.001	102	128
Bi_2Se_4	942	0.001	138	65
F_4Pb	805	0.001	100	81
Bi_2Se_4	914	0.001	134	63
H_4Ti	601	0.001	49	81
$Ag_2K_2Se_2$	708	0.0011	81	64
Cl_2NSc_2	946	0.0011	79	126
Br_2OV	640	0.0011	71	89
O_2Sn_2	920	0.0012	102	128
Bi_2Se_4	246	0.0012	36	17
$H_2I_2Yb_2$	10	0.0012	1	1
Se_2Ta_4	438	0.0012	36	49
H_2Li_2Pd	717	0.0012	58	97
Cl_2Mg	387	0.0012	48	65
GeS_2	499	0.0012	64	81
Br_2Ni	387	0.0013	48	65
Bi_2Se_4	218	0.0013	32	15
NbS_2	694	0.0013	79	126
Cl_2Mn	694	0.0013	79	126
Cl_4KTl	154	0.0014	25	9
$Cu_2Rb_2Te_2$	412	0.0014	49	36
K	501	0.0014	103	89
PTe_2Ti_2	517	0.0014	48	65
Br_2Zr_2	796	0.0014	81	118
O_4PSn	962	0.0014	113	85
$I_2S_2Sm_2$	946	0.0015	103	89
Ho_2S_2	860	0.0015	106	109

Table showing the first 50 lattice matching combinations with the smallest strain needed to build the supercell.

AgF_2 ($P2_1/c$)

Structural and electronic properties

**Formula**	AgF_2
**Spacegroup**	$P2_1/c$
**Prototype**	PdS2
**Parent 3D**	Ag_4F_8
**Source DB**	COD
**DB ID**	1509321
**DF2-C09 Binding energy [meV/ Å^2]**	28.24
**RVV10 Binding energy [meV/ Å^2]**	42.45
**Band gap (PBE) [eV]**	N/A

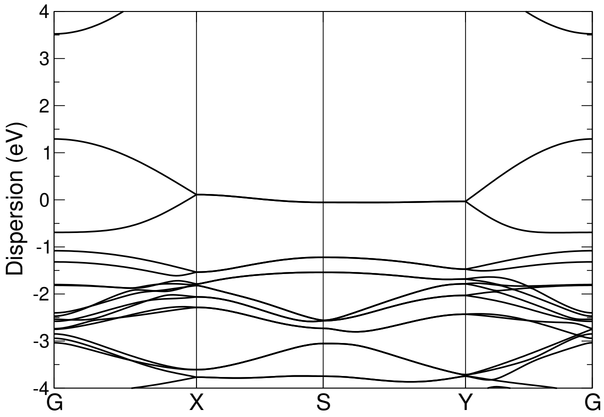

Band structure: Electronic band structure of AgF_2 ($P2_1/c$) in a representative window around the Fermi energy and along a high-symmetry path.

Optimized crystal structure

Structural parameters: relaxed cell (top) and atomic positions (bottom) of AgF_2 ($P2_1/c$) in Cartesian coordinates.

	x [Å]	y [Å]	z [Å]
$\mathbf{a_1}$	5.24153122	0.00000000	0.00000000
$\mathbf{a_2}$	0.00000000	5.88865471	0.00000000
$\mathbf{a_3}$	0.00000000	0.00000000	21.40657674

	x [Å]	y [Å]	z [Å]
F	1.09373287	1.66410444	10.10952238
F	1.52703274	4.60843179	10.10952238
Ag	0.00000000	0.00000000	10.70328837
Ag	2.62076561	2.94432735	10.70328837
F	3.71449848	1.28022292	11.29705436
F	4.14779835	4.22455027	11.29705436

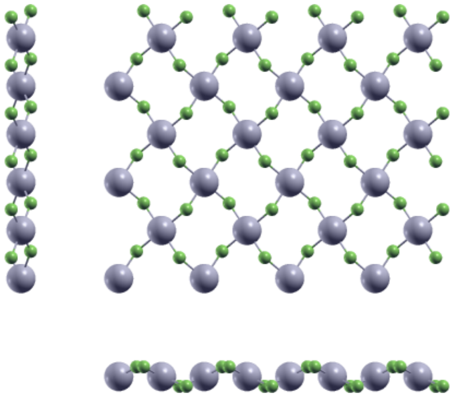

Orthographic projections: views of AgF_2 ($P2_1/c$) as seen from the x axis (left), the y axis (bottom) and the z axis (center).

Lattice matching - minimal size

Formula	N° atoms	strain	cell size 1	cell size 2
Tl	8	0.1382	1	2
Sn	8	0.1387	1	2
In	8	0.1397	1	2
AsSb	10	0.7377	1	2
Br_3Cs	10	0.2148	1	1
IO_3Tl	11	0.2094	1	1
$ClKO_3$	11	1.6625	1	1
KNO_3	11	0.1949	1	1
$I_2Lu_2Se_2$	12	0.1722	1	1
Cl_2Mn	12	0.1401	1	2
$MoSe_2$	12	0.138	1	2
S_2Ta	12	0.1409	1	2
$SiTe_2$	12	0.7156	1	2
Cl_4Cu_2	12	0.3622	1	1
HgI_2	12	1.2573	1	2
S_2Ti	12	0.1428	1	2
NbS_2	12	0.1407	1	2
Cl_2Co	12	0.1427	1	2
$Br_2Er_2Se_2$	12	0.4095	1	1
NbS_2	12	0.1392	1	2
ClNZr	12	0.1442	1	2
Cl_2Fe	12	0.1424	1	2
S_2Ta	12	0.1389	1	2
CKN	12	1.2455	1	2
Se_2V	12	0.1385	1	2
I_2V	12	0.7204	1	2
$Br_2Ga_2Te_2$	12	0.1702	1	1
Ca_4Cu_2	12	0.0601	1	1
Se_2Zr	12	0.7171	1	2
CoI_2	12	0.7393	1	2
Cl_2Zr	12	0.1425	1	2
Cu_2F_4	12	0.0316	1	1
Br_2Ca_3Si	12	0.0413	1	1
$Cl_2S_2Tl_2$	12	0.2365	1	1
Se_2W	12	0.138	1	2
Bi_2Mn_2	14	0.1649	1	2
Pb_2Se_2	14	0.3672	1	2
Ga_2Se_2	14	0.7261	1	2
Cl_2Zr_2	14	0.1448	1	2
Bi_2Se_2	14	0.0498	1	2
$LiNbS_2$	14	0.1409	1	2
Br_2CsF	14	1.345	1	2
$CuTe_2$	15	0.1689	1	3
K	15	0.0571	2	3
Te_2Zn	15	0.16	1	3
$TaTe_2$	15	0.1802	1	3
PTe_2Zr_2	16	0.7351	1	2
Cl_2NSc_2	16	0.1403	1	2
$AgBrO_2$	16	0.1577	2	1
CCl_2Sc_2	16	0.1446	1	2

Table showing the first 50 lattice matching combinations with the smallest number of atoms in the supercell.

Lattice matching - minimal strain

Formula	N° atoms	strain	cell size 1	cell size 2
$Br_2Eu_2F_2$	108	0.0003	6	12
$Er_2I_2O_2$	108	0.0003	6	12
AlH_4Na	516	0.0004	36	50
I_2Zn	522	0.0004	42	90
$Br_2Ho_2S_2$	918	0.0004	63	90
ClH_3O	970	0.0005	70	110
$Ho_2I_2S_2$	516	0.0005	36	50
$Bi_2Br_2O_2$	108	0.0006	6	12
Se_2Sn	792	0.0006	60	144
I_2La_2	452	0.0007	32	65
NSr_2	792	0.0007	60	144
$I_2O_2Tm_2$	108	0.0008	6	12
Fe_2Se_2	530	0.0008	35	80
Cu_2Sr_2	746	0.0008	55	104
$F_2Se_2Y_2$	906	0.001	60	91
CoI_2	792	0.001	60	144
C_4Ca_2	678	0.001	40	73
I_2La_2P	850	0.001	55	104
SSb_2Te_2	517	0.001	32	65
PbS_2Sn	686	0.001	53	92
Ba_2Cd	225	0.001	20	35
$Si_2Te_2Zr_2$	690	0.001	35	80
F_2Ni	450	0.001	35	80
Sn_2Te_2	548	0.0011	46	68
Co_2Se_2	626	0.0011	41	95
Te_2Zr	843	0.0011	66	149
$F_2I_2Pb_2$	330	0.0011	20	35
$HfTe_2$	843	0.0012	66	149
Br_2CsF	954	0.0012	81	117
$F_2Lu_2Se_2$	792	0.0012	42	90
$Hf_2Si_2Te_2$	816	0.0012	41	95
$Br_2Er_2S_2$	918	0.0012	63	90
$Au_2K_2S_2$	192	0.0013	20	12
$I_2S_2Tl_2$	690	0.0013	35	80
AsSb	648	0.0014	60	144
$Ba_2H_2I_2$	726	0.0014	49	72
$I_2O_2Y_2$	108	0.0014	6	12
BiBrTe	642	0.0014	55	104
$I_2N_2Zr_2$	630	0.0014	35	70
In_2Se_2	612	0.0014	42	90
Br_2Ca_3Si	654	0.0015	40	69
CrI_2	792	0.0015	60	144
I_2La_2Te	585	0.0015	40	69
Bi_2In_2	954	0.0015	81	117
Cu_2Se_2	530	0.0015	35	80
Pt_2Te_2	992	0.0015	66	149
PTe_2Zr_2	868	0.0015	48	116
AsSb	520	0.0016	48	116
$Fe_2Li_2P_2$	690	0.0016	35	80
$Br_2Nd_2O_2$	108	0.0016	6	12

Table showing the first 50 lattice matching combinations with the smallest strain needed to build the supercell.

AgI (P4/nmm)

Structural and electronic properties

**Formula**	AgI
**Spacegroup**	P4/nmm
**Prototype**	FeSe
**Parent 3D**	Ag_2I_2
**Source DB**	COD
**DB ID**	1509387
**DF2-C09 Binding energy [meV/ Å^2]**	13.5
**RVV10 Binding energy [meV/ Å^2]**	20.78
**Band gap (PBE) [eV]**	2.15

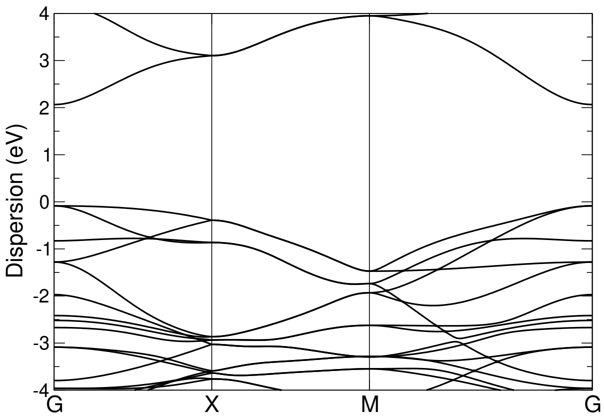

Band structure: Electronic band structure of AgI (P4/nmm) in a representative window around the Fermi energy and along a high-symmetry path.

Optimized crystal structure

Structural parameters: relaxed cell (top) and atomic positions (bottom) of AgI (P4/nmm) in Cartesian coordinates.

	x [Å]	y [Å]	z [Å]
$\mathbf{a_1}$	4.48720527	0.00000000	0.00000000
$\mathbf{a_2}$	−0.00000000	4.48720527	0.00000000
$\mathbf{a_3}$	0.00000000	0.00000000	23.77814454

		x [Å]	y [Å]	z [Å]
●	I	2.24360263	0.00000000	13.74921428
●	Ag	0.00000000	0.00000000	11.88907227
●	Ag	2.24360263	2.24360263	11.88907227
●	I	-0.00000000	2.24360263	10.02893026

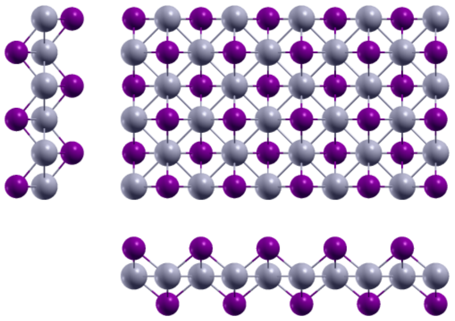

Orthographic projections: views of AgI (P4/nmm) as seen from the x axis (left), the y axis (bottom) and the z axis (center).

Lattice matching - minimal size

Formula	N° atoms	strain	cell size 1	cell size 2
K	6	0.971	1	2
AgTl	6	0.2137	1	1
PbTe	6	0.1088	1	1
Sm	6	0.1485	1	2
PSn_2	7	0.1107	1	1
$AsSn_2$	7	0.1099	1	1
I_2Pr	7	0.5876	1	1
S_2Zr	7	0.1109	1	1
HgI_2	7	0.001	1	1
$RhTe_2$	7	0.1093	1	1
CKN	7	0.3682	1	1
I_2Nd	7	0.5905	1	1
Cl_2Cu	7	0.1409	1	1
S_2Sn	7	0.1108	1	1
GeI_2	7	0.1088	1	1
$PtSe_2$	7	0.1097	1	1
CeI_2	7	0.5853	1	1
$NbTe_2$	7	0.1109	1	1
I_2La	7	0.2082	1	1
CdI_2	7	0.1091	1	1
Bi_2Te_2	8	0.139	1	1
Li_2Tl_2	8	0.1621	1	1
CdClHO	8	0.1101	1	1
$LiMnTe_2$	8	0.1089	1	1
Ir_2P_2	8	0.5899	1	1
Ag_2Br_2	8	0.2088	1	1
CdClHO	8	0.1094	1	1
$AsLi_3$	8	0.1084	1	1
S_2Sn_2	8	0.2301	1	1
Cu_2S_2	8	0.5665	1	1
Cl_2Y_2	8	0.1087	1	1
Ge_2Te_2	8	0.0207	1	1
As_2Ir_2	8	0.2114	1	1
O_2Sn_2	8	0.5682	1	1
P_2Rh_2	8	0.5646	1	1
F_2Tl_2	8	0.565	1	1
Au_2I_2	8	0.0188	1	1
Ge_2Se_2	8	0.218	1	1
Ag_2Te_2	8	0.5735	1	1
Ni_2Se_2	8	0.5846	1	1
As_2Rh_2	8	0.2085	1	1
Ga_2Se_2	8	0.1089	1	1
Sn_2Te_2	8	0.008	1	1
As_2O_3	9	0.7703	1	1
F_4Pb	9	0.0084	1	1
Bi_2STe_2	9	0.1086	1	1
As_2CeLi_2	9	0.1088	1	1
Bi_2STe_2	9	0.109	1	1
NaO_4	9	0.1728	1	1
$AgNO_3$	9	0.1737	1	1

Table showing the first 50 lattice matching combinations with the smallest number of atoms in the supercell.

Lattice matching - minimal strain

Formula	N° atoms	strain	cell size 1	cell size 2
$Br_2Dy_2O_2$	438	0.0	36	49
I_3Sn	520	0.0001	81	49
$Br_2O_2Y_2$	438	0.0001	36	49
Fe_2Te_2	584	0.0001	61	85
$K_2Mn_2Sb_2$	752	0.0001	65	82
$Br_2Ce_2O_2$	752	0.0001	65	82
$Ge_2Hf_2Te_2$	316	0.0001	25	36
Tl	445	0.0001	74	149
Cu_2S_2	340	0.0002	36	49
Mg_4	892	0.0002	74	149
As_2Fe_2	684	0.0002	65	106
Sb_2Se_2Te	517	0.0002	48	65
Ho_2S_2	680	0.0003	73	97
$Ca_2H_2I_2$	742	0.0003	64	81
$BiTe_2$	387	0.0003	48	65
$Br_2Eu_2F_2$	580	0.0003	49	64
$Er_2I_2O_2$	580	0.0003	49	64
$Br_2O_2Yb_2$	754	0.0003	61	85
$Eu_2I_2O_2$	752	0.0004	65	82
$Cl_2S_2Tl_2$	896	0.0004	101	82
Ca_2Cl_2	900	0.0004	89	136
F_2Tl_2	340	0.0004	36	49
$Bi_2Br_2O_2$	590	0.0004	50	65
Br_2V	197	0.0004	20	39
Mg_6	394	0.0004	25	49
$I_2O_2Tm_2$	580	0.0004	49	64
$As_2Co_2Li_2$	316	0.0004	25	36
$I_2Nd_2O_2$	924	0.0005	81	100
$ReSe_2$	197	0.0005	20	39
P_2Rh_2	340	0.0005	36	49
Se_2Yb	387	0.0005	48	65
O_2Sn_2	596	0.0005	57	92
$I_2O_2Y_2$	590	0.0005	50	65
$Cl_2O_2Sc_2$	710	0.0005	56	81
Ca_2Cl_2	584	0.0005	61	85
Sb_2Se_2Te	517	0.0006	48	65
$Br_2Ce_2O_2$	742	0.0006	64	81
$K_2Mn_2Sb_2$	742	0.0006	64	81
GeS_2	578	0.0007	65	106
$AgBrO_2$	688	0.0007	72	100
$Hf_2Se_2Si_2$	896	0.0007	65	106
Fe_2S_2	164	0.0007	16	25
O_2Sn_2	340	0.0007	36	49
$Bi_2Br_2O_2$	580	0.0007	49	64
As_2Ru_2	584	0.0007	61	85
$Br_2Nd_2O_2$	590	0.0007	50	65
Ni_2Se_2	792	0.0007	85	113
GeI_2	387	0.0007	48	65
Au_2Se_2	260	0.0007	35	30
Mg_3	539	0.0008	53	109

Table showing the first 50 lattice matching combinations with the smallest strain needed to build the supercell.

$AgNO_2$ (P1)

Structural and electronic properties

**Formula**	$AgNO_2$
**Spacegroup**	P1
**Prototype**	AgNO2
**Parent 3D**	$AgNO_2$
**Source DB**	COD
**DB ID**	2105346
**DF2-C09 Binding energy [meV/ Å^2]**	25.05
**RVV10 Binding energy [meV/ Å^2]**	38.47
**Band gap (PBE) [eV]**	1.65

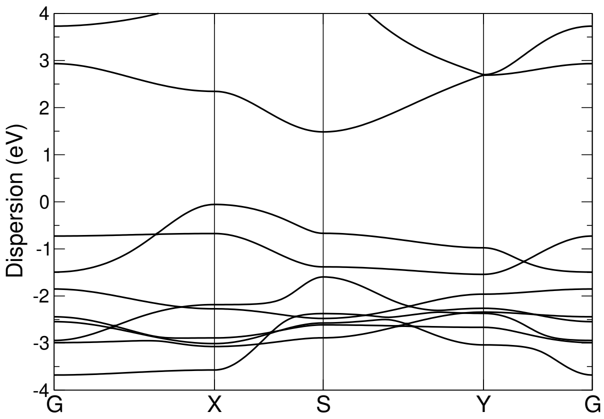

Band structure: Electronic band structure of $AgNO_2$ (P1) in a representative window around the Fermi energy and along a high-symmetry path.

Optimized crystal structure

Structural parameters: relaxed cell (top) and atomic positions (bottom) of $AgNO_2$ (P1) in Cartesian coordinates.

		x [Å]	y [Å]	z [Å]
$\mathbf{a_1}$		3.33863553	0.00000000	0.00000000
$\mathbf{a_2}$		0.00000000	4.92296294	0.00000000
$\mathbf{a_3}$		0.00000000	0.00000000	22.13508499
		x [Å]	y [Å]	z [Å]
•	O	1.66931776	0.35905440	12.13873434
•	Ag	1.66931776	2.49151995	11.06754250
•	N	1.66931776	4.63757419	11.06754250
•	O	1.66931776	0.35905440	9.99635065

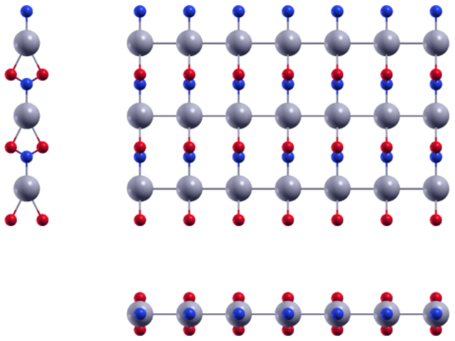

Orthographic projections: views of $AgNO_2$ (P1) as seen from the x axis (left), the y axis (bottom) and the z axis (center).

Lattice matching - minimal size

Formula	N° atoms	strain	cell size 1	cell size 2
K	6	0.7473	1	2
AgTl	6	0.1095	1	1
CNRb	7	0.1679	1	1
Cu_2O_2	8	0.5896	1	1
Au_2Br_2	8	0.4441	1	1
Ge_2S_2	8	0.1403	1	1
As_4	8	0.2148	1	1
P_4	8	0.0205	1	1
Bi_2Se_2	8	1.3943	1	1
Br_3Cs	8	1.3617	1	1
Au_2I_2	8	0.4508	1	1
Ge_2Se_2	8	0.1247	1	1
Ag_2Te_2	8	1.0431	1	1
$AgClO_2$	8	0.3413	1	1
$SbSe_2Tl$	8	1.359	1	1
IO_3Tl	9	1.3375	1	1
$CuGeO_3$	9	0.301	1	1
KNO_3	9	1.272	1	1
I_2Mg	10	0.833	1	2
$Cl_2O_2V_2$	10	0.3018	1	1
$C_2I_2La_2$	10	0.7452	1	1
FeO_2	10	0.0481	1	2
Br_2La	10	0.8344	1	2
NiO_2	10	0.1346	1	2
$Cl_2N_2Zr_2$	10	0.3751	1	1
$Br_2Cr_2S_2$	10	0.0253	1	1
$K_2O_2Tl_2$	10	2.6185	1	1
N_2W	10	0.1584	1	2
$I_2S_2Tb_2$	10	0.7379	1	1
Cu_2O_4	10	0.6587	1	1
$Br_2O_2V_2$	10	0.3111	1	1
$C_2Cl_2Y_2$	10	0.1375	1	1
$BiTe_2$	10	0.8273	1	2
$C_2Br_2La_2$	10	0.7106	1	1
CoO_2	10	0.1343	1	2
CNRb	11	0.0872	2	1
Cl_2Rb_2	12	0.8637	2	1
$HNiO_2$	12	0.1519	1	2
Ge_2Te_2	12	0.7163	1	2
Cu_2Te_2	12	0.1779	2	1
Cu_2I_2	12	0.8369	1	2
Ag_2Te_2	12	0.188	2	1
La_2S_2	12	0.1177	2	1
Gd_2I_2	12	0.8356	1	2
Fe_2SeTe	12	0.1797	2	1
Se_2Sn_2	12	0.1175	2	1
NS_2Ta	12	0.1564	1	2
F_4Pb	14	0.7854	1	2
$Ge_2Hf_2Te_2$	14	0.1785	2	1
Sb_2Se_2Te	14	0.8287	1	2

Table showing the first 50 lattice matching combinations with the smallest number of atoms in the supercell.

Lattice matching - minimal strain

Formula	N° atoms	strain	cell size 1	cell size 2
$C_2Br_2La_2$	468	0.0004	45	48
Cl_2Gd_2	876	0.0008	98	121
Cu_2O_4	810	0.0008	66	91
$Br_2Er_2O_2$	602	0.0008	56	63
$CuGeO_3$	725	0.0008	80	81
$I_2Se_2Tb_2$	582	0.0008	72	49
Te_2Zr	494	0.0009	65	78
Se_2Sn_2	540	0.0009	72	63
Cu_3Se_3	728	0.001	65	78
Ge_2S_2	696	0.001	86	88
$AuCrTe_4$	778	0.0011	100	63
Br_2Zr_2	464	0.0011	46	70
LiO_2	433	0.0011	49	79
$Cu_2Na_2Te_2$	912	0.0011	99	86
In_2Se_3	650	0.0011	65	78
$Ca_2Cl_2F_2$	938	0.0011	86	99
Ba_2N	494	0.0011	65	78
I_4Zr_2	492	0.0011	63	40
$HfTe_2$	494	0.0011	65	78
Hf_3Te_2	300	0.0012	30	36
$Br_2Ca_2H_2$	602	0.0012	56	63
$H_2I_2Sr_2$	588	0.0012	63	56
Se_2Ti	394	0.0012	46	70
$Er_2F_2Se_2$	728	0.0013	65	78
Cu_4Te_2	246	0.0013	24	25
Br_2Ca_3Si	758	0.0013	98	61
$Ca_2Mn_2Si_2$	602	0.0014	56	63
$Br_2F_2Yb_2$	848	0.0014	80	88
Br_2Cu_2	672	0.0014	80	88
$CuTe_2$	474	0.0014	57	82
$Ca_2Cl_2H_2$	336	0.0014	30	36
$F_2Se_2Y_2$	846	0.0014	96	77
$Br_2Ca_2F_2$	848	0.0015	80	88
$Br_2Ho_2O_2$	602	0.0015	56	63
$Br_2O_2Tb_2$	848	0.0016	80	88
AgTl	426	0.0016	71	71
Sn	141	0.0016	24	45
CdI_2	192	0.0016	27	28
$Ge_2I_2La_2$	274	0.0016	28	27
Cl_4Pd_2	966	0.0016	126	77
BiClTe	192	0.0016	27	28
Gd_2GeI_2	248	0.0016	27	28
I_2Pr	192	0.0016	27	28
I_2V	127	0.0016	16	21
Br_2La_2P	248	0.0017	27	28
F_2Zn	584	0.0017	80	88
Au_2Br_2	680	0.0017	89	81
$Er_2I_2Se_2$	954	0.0017	117	81
$F_2Ho_2Se_2$	468	0.0017	42	50
CdI_2	192	0.0017	27	28

Table showing the first 50 lattice matching combinations with the smallest strain needed to build the supercell.

AgO_2Br (P1)

Structural and electronic properties

**Formula**	AgO_2Br
**Spacegroup**	P1
**Prototype**	AgClO2
**Parent 3D**	$AgBrO_2$
**Source DB**	ICSD
**DB ID**	670051
**DF2-C09 Binding energy [meV/ Å^2]**	10.3
**RVV10 Binding energy [meV/ Å^2]**	N/A
**Band gap (PBE) [eV]**	0.4

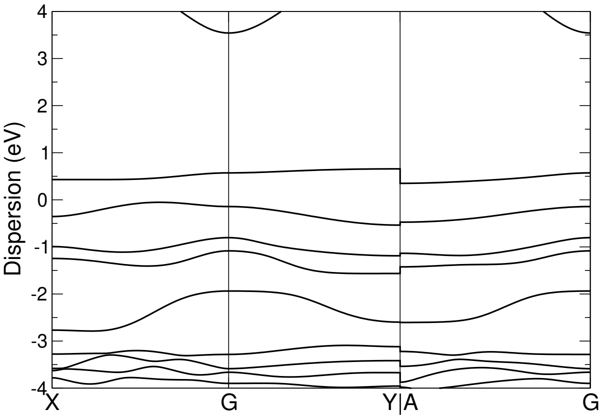

Band structure: Electronic band structure of AgO_2Br (P1) in a representative window around the Fermi energy and along a high-symmetry path.

Optimized crystal structure

Structural parameters: relaxed cell (top) and atomic positions (bottom) of AgO_2Br (P1) in Cartesian coordinates.

	x [Å]	y [Å]	z [Å]
$\mathbf{a_1}$	−4.03569934	0.07360202	0.00000000
$\mathbf{a_2}$	−1.64439482	−3.55877413	0.00000000
$\mathbf{a_3}$	0.00000000	0.00000000	17.88906127

	x [Å]	y [Å]	z [Å]
Ag	-3.11816475	-1.60533905	0.32622936
Br	-1.39070235	-0.22541627	1.73947505
O	-4.76767395	-2.44437180	-0.83771578
O	-3.01092928	-0.11069335	-1.22798864

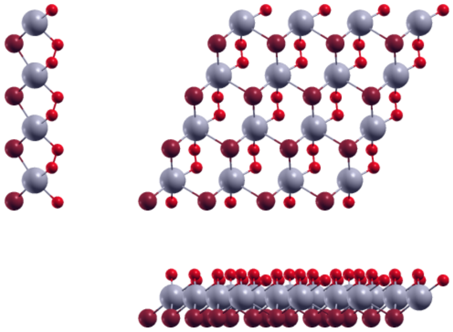

Orthographic projections: views of AgO_2Br (P1) as seen from the x axis (left), the y axis (bottom) and the z axis (center).

Lattice matching - minimal size

Formula	N° atoms	strain	cell size 1	cell size 2
K	5	0.2482	1	1
K	5	0.5309	1	1
Na	5	0.2781	1	1
InSe	6	0.0381	1	1
AsSb	6	0.0369	1	1
Bi_2	6	0.0425	1	1
GeTe	6	0.0339	1	1
Ag_2	6	0.5464	1	1
S_2	6	0.0332	1	1
PbTe	6	0.0394	1	1
Sb_2	6	0.0303	1	1
CaCl	6	0.0932	1	1
$IrTe_2$	7	0.0335	1	1
I_2Mg	7	0.0323	1	1
$CdCl_2$	7	0.0344	1	1
CdI_2	7	0.0406	1	1
$MoTe_2$	7	0.2815	1	1
Ba_2Pt	7	0.5457	1	1
$ReSe_2$	7	0.2605	1	1
Br_2Ca	7	0.0416	1	1
$InSe_2$	7	0.0342	1	1
$GeTe_2$	7	0.0349	1	1
$HfTe_2$	7	0.0305	1	1
Te_2V	7	0.2844	1	1
I_2Pr	7	0.1152	1	1
I_2Mn	7	0.0343	1	1
Br_2La	7	0.0324	1	1
NSr_2	7	0.0361	1	1
Ca_2Si	7	0.5588	1	1
PbS_2	7	0.0384	1	1
BiClTe	7	0.0409	1	1
$AuTe_2$	7	0.0295	1	1
BrCdI	7	0.0337	1	1
Cl_2Zn	7	0.0994	1	1
$PdTe_2$	7	0.0296	1	1
FeI_2	7	0.0354	1	1
I_2Ni	7	0.0348	1	1
S_2Ti	7	0.2525	1	1
CrI_2	7	0.0356	1	1
I_2Zn	7	0.0297	1	1
BaF_2	7	0.0356	1	1
Te_2Zn	7	0.2813	1	1
BiBrTe	7	0.0455	1	1
Bi_2Pd	7	0.1247	1	1
GeI_2	7	0.0315	1	1
Cl_2Ni	7	0.2618	1	1
Cl_2Co	7	0.252	1	1
$CrTe_2$	7	0.2703	1	1
PtS_2	7	0.2797	1	1
Br_2V	7	0.2594	1	1

Table showing the first 50 lattice matching combinations with the smallest number of atoms in the supercell.

Lattice matching - minimal strain

Formula	N° atoms	strain	cell size 1	cell size 2
Sb_2Se_2Te	674	0.0002	76	74
Sb_2Se_2Te	665	0.0003	75	73
$BiTe_2$	526	0.0004	76	74
$BiTe_2$	519	0.0005	75	73
Sb_2Se_2Te	665	0.0005	75	73
Se_2Yb	291	0.0006	42	41
GeI_2	291	0.0006	42	41
LiO	418	0.0006	58	93
$BiTe_2$	291	0.0006	42	41
Ag_2I_2	688	0.0007	100	72
Ba_2Ni_3	373	0.0007	42	41
Ga_2Te_2	332	0.0007	42	41
Sb_2Se_2Te	656	0.0007	74	72
$I_2Pr_2Si_2$	414	0.0007	42	41
$I_2O_2Tm_2$	984	0.0008	102	96
I_2Nd	717	0.0008	105	99
Ir_2P_2	724	0.0008	93	88
P_4	632	0.0008	81	77
Fe_2S_2	136	0.0008	16	18
$Br_2O_2Sm_2$	900	0.0008	93	88
$Ce_2I_2Si_2$	738	0.0008	75	73
I_2Nd	636	0.0008	93	88
$I_2Pr_2S_2$	746	0.0008	86	67
O_2Sn_2	712	0.0008	90	88
Sb_2Se_2Te	373	0.0008	42	41
Ag_2	478	0.0008	86	67
$I_2O_2Yb_2$	900	0.0009	93	88
$AsCuLi_2$	332	0.0009	42	41
Ca_2N	502	0.0009	64	82
Ag_2	456	0.0009	82	64
$Br_2Hf_2N_2$	824	0.0009	83	82
Ba_2Pt	520	0.0009	82	64
Br_2Ca_3Si	964	0.0009	109	88
NbS_2	731	0.0009	86	129
Tl	316	0.001	58	84
Mg_4	568	0.001	58	84
I_2Mg	519	0.001	75	73
HgI_2	616	0.001	100	72
$Br_2Eu_2F_2$	984	0.001	102	96
As_2	420	0.0011	64	82
$CrSe_2$	511	0.0011	58	93
$Er_2I_2O_2$	984	0.0011	102	96
Br_2Ca_3Si	772	0.0011	106	58
I_2Pr	636	0.0011	93	88
$Br_2Hf_2N_2$	748	0.0011	64	82
I_2Mg	228	0.0011	33	32
$Ce_2I_2Si_2$	324	0.0011	33	32
CCl_2Lu_2	666	0.0011	64	82
$I_2Se_2Tb_2$	698	0.0011	92	55
Sb_2Se_2Te	301	0.0012	34	33

Table showing the first 50 lattice matching combinations with the smallest strain needed to build the supercell.

AgO_2Cl (P1)

Structural and electronic properties

**Formula**	AgO_2Cl
**Spacegroup**	P1
**Prototype**	AgClO2
**Parent 3D**	$AgClO_2$
**Source DB**	ICSD
**DB ID**	670052
**DF2-C09 Binding energy [meV/ Å^2]**	8.44
**RVV10 Binding energy [meV/ Å^2]**	N/A
**Band gap (PBE) [eV]**	0.38

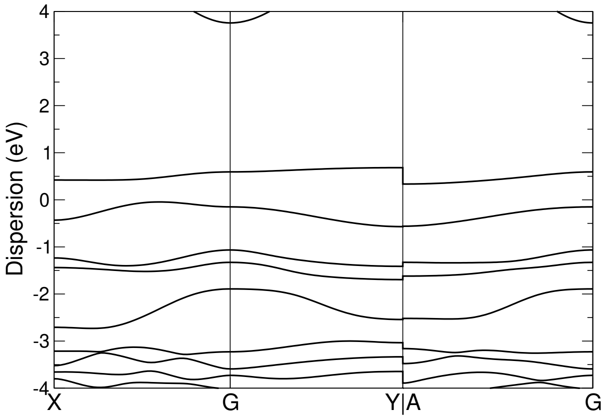

Band structure: Electronic band structure of AgO_2Cl (P1) in a representative window around the Fermi energy and along a high-symmetry path.

Optimized crystal structure

Structural parameters: relaxed cell (top) and atomic positions (bottom) of AgO_2Cl (P1) in Cartesian coordinates.

	x [Å]	y [Å]	z [Å]
$\mathbf{a_1}$	−3.92783597	0.17718089	0.00000000
$\mathbf{a_2}$	−1.72614525	−3.45236652	0.00000000
$\mathbf{a_3}$	0.00000000	0.00000000	17.62666264

		x [Å]	y [Å]	z [Å]
●	Ag	-3.32760019	-1.77220401	-0.37062026
●	Cl	-1.62191561	-0.44635808	-1.63436312
●	O	-3.20195700	-0.30435787	1.19573639
●	O	-4.99573155	-2.52936982	0.80924699

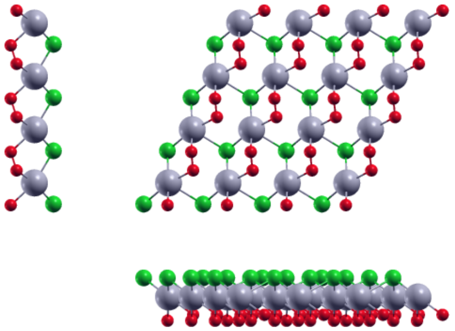

Orthographic projections: views of AgO_2Cl (P1) as seen from the x axis (left), the y axis (bottom) and the z axis (center).

Lattice matching - minimal size

Formula	N° atoms	strain	cell size 1	cell size 2
K	5	0.2598	1	1
Tl	5	0.119	1	1
InSe	6	0.0448	1	1
AsSb	6	0.031	1	1
Bi_2	6	0.0502	1	1
LiO	6	1.1984	1	1
PbTe	6	0.0464	1	1
Mg_2	6	0.1161	1	1
CaCl	6	0.152	1	1
CdI_2	7	0.048	1	1
Br_2Zn	7	0.036	1	1
Br_2Ca	7	0.0491	1	1
$AsSn_2$	7	0.0401	1	1
$SiTe_2$	7	0.0338	1	1
PbS_2	7	0.0321	1	1
BiClTe	7	0.0483	1	1
BrCdI	7	0.0388	1	1
Te_2Ti	7	0.0359	1	1
BaF_2	7	0.0415	1	1
BiBrTe	7	2.2704	1	1
$RhTe_2$	7	0.0384	1	1
NbS_2	7	1.2921	1	1
CKN	7	0.324	1	1
AsKSn	7	0.0403	1	1
$PbTe_2$	7	0.0381	1	1
$NiTe_2$	7	0.0341	1	1
I_2V	7	0.0331	1	1
GeI_2	7	0.0466	1	1
Se_2Zr	7	0.0336	1	1
STl_2	7	0.0423	1	1
$PtSe_2$	7	0.0397	1	1
CdO_2	7	0.2613	1	1
CoI_2	7	0.0308	1	1
$MnSe_2$	7	0.152	1	1
CuO_2	7	0.0882	1	1
I_2Ti	7	0.0307	1	1
F_2Na	7	0.0349	1	1
CdI_2	7	0.0476	1	1
I_2Pr	7	0.0484	1	1
$HfSe_2$	7	0.0358	1	1
Bi_2Te_2	8	0.6205	1	1
$LiMnTe_2$	8	0.0469	1	1
Br_2Pr_2	8	0.0354	1	1
Cu_2Te_2	8	0.1145	1	1
Cl_2Hf_2	8	0.8004	1	1
$AgNO_2$	8	0.5247	1	1
CdClHO	8	0.0389	1	1
$AsLi_3$	8	0.0451	1	1
Cl_2Y_2	8	0.0367	1	1
Ge_2Te_2	8	0.2585	1	1

Table showing the first 50 lattice matching combinations with the smallest number of atoms in the supercell.

Lattice matching - minimal strain

Formula	N° atoms	strain	cell size 1	cell size 2
$Ba_2Ge_2Mn_2$	852	0.0003	93	80
$F_2I_2Yb_2$	852	0.0004	93	80
$I_2N_2Zr_2$	984	0.0006	105	94
Cl_2NSc_2	697	0.0006	63	89
$Br_2Hf_2N_2$	850	0.0007	88	83
$Eu_2H_2I_2$	852	0.0007	93	80
Ge_2Te_2	656	0.0008	94	70
CNRb	221	0.0009	38	23
Cl_2Mn	519	0.0009	63	89
$Cu_2Na_2Te_2$	780	0.0009	93	68
$Ho_2I_2S_2$	596	0.001	77	48
$Br_2O_2V_2$	500	0.001	47	52
$CrSe_2$	638	0.001	74	114
$Er_2I_2S_2$	668	0.001	86	54
Br_2Ca_3Si	780	0.001	90	70
Au_2Br_2	352	0.0011	50	38
$Cl_2O_2Ti_2$	764	0.0011	74	78
$I_2O_2Sm_2$	852	0.0011	93	80
$F_2I_2Tm_2$	384	0.0011	42	36
$Gd_2I_2S_2$	780	0.0011	90	70
$I_2Sb_2Te_2$	424	0.0011	64	28
O_4PTl	938	0.0011	116	79
N_4	532	0.0011	52	81
Ag_2I_2	540	0.0011	80	55
NbS_2	519	0.0011	63	89
$Cu_2Na_2Se_2$	384	0.0011	42	36
$Bi_2I_2O_2$	384	0.0011	42	36
FeH_2O_2	765	0.0011	60	105
$Br_2Lu_2O_2$	928	0.0011	94	92
Sm	209	0.0011	33	77
$Ge_2Te_2Zr_2$	928	0.0011	94	92
$Cl_2H_2Zr_2$	730	0.0012	58	83
$Cl_2F_2Pb_2$	384	0.0012	42	36
$Cu_2Na_2Te_2$	538	0.0012	64	47
Mg_2	586	0.0012	84	125
NbS_2	481	0.0012	58	83
Bi_2Pd	902	0.0013	116	146
$Ba_2F_2I_2$	596	0.0013	74	50
$Br_2S_2Yb_2$	656	0.0013	83	54
$Ba_2Ge_2Mn_2$	384	0.0013	42	36
Cl_4Pd_2	576	0.0013	81	42
$Br_2S_2Yb_2$	584	0.0013	74	48
$F_2I_2Pb_2$	990	0.0014	114	89
$Gd_2I_2S_2$	766	0.0014	100	61
$Eu_2H_2I_2$	468	0.0014	51	44
Se_2Sn_2	500	0.0014	72	53
$CuGeO_3$	736	0.0014	89	76
Fe_2SeTe	744	0.0014	94	92
Cl_2Hf_2	652	0.0014	68	95
$I_2O_2Sm_2$	468	0.0014	51	44

Table showing the first 50 lattice matching combinations with the smallest strain needed to build the supercell.

AgO_4Cl (P-42m)

Structural and electronic properties

**Formula**	AgO_4Cl
**Spacegroup**	P-42m
**Prototype**	AgO4Cl
**Parent 3D**	AgO_4Cl
**Source DB**	COD
**DB ID**	9008281
**DF2-C09 Binding energy [meV/ Å^2]**	19.1
**RVV10 Binding energy [meV/ Å^2]**	28.96
**Band gap (PBE) [eV]**	2.9

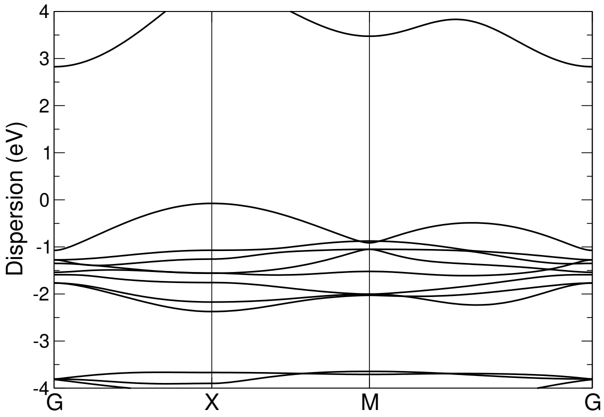

Band structure: Electronic band structure of AgO_4Cl (P-42m) in a representative window around the Fermi energy and along a high-symmetry path.

Optimized crystal structure

Structural parameters: relaxed cell (top) and atomic positions (bottom) of AgO_4Cl (P-42m) in Cartesian coordinates.

		x [Å]	y [Å]	z [Å]
$\mathbf{a_1}$		4.84412677	0.00000000	0.00000000
$\mathbf{a_2}$		0.00000000	4.84412677	0.00000000
$\mathbf{a_3}$		0.00000000	0.00000000	21.75213208
		x [Å]	y [Å]	z [Å]
●	Cl	2.42206338	2.42206338	10.87606604
●	O	3.26235187	1.58177489	11.74599586
●	O	1.58177489	3.26235187	11.74599586
●	O	3.26235187	3.26235187	10.00613623
●	Ag	0.00000000	4.84412677	10.87606604
●	O	1.58177489	1.58177489	10.00613623

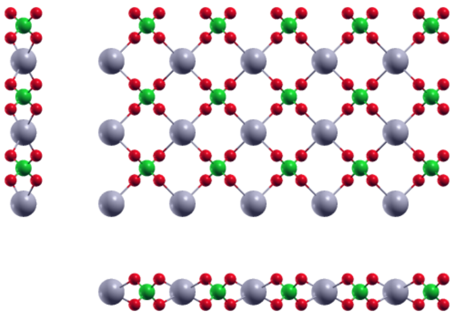

Orthographic projections: views of AgO_4Cl (P-42m) as seen from the x axis (left), the y axis (bottom) and the z axis (center).

Lattice matching - minimal size

Formula	N° atoms	strain	cell size 1	cell size 2
K	7	0.2142	1	1
$HfTe_2$	9	0.1107	1	1
$AuTe_2$	9	0.109	1	1
$PdTe_2$	9	0.1095	1	1
$CNRb$	9	0.033	1	1
CKN	9	0.2303	1	1
Ba_2N	9	0.1103	1	1
Te_2Zr	9	0.1106	1	1
$PtTe_2$	9	0.1091	1	1
Br_2Cd	9	0.1097	1	1
In	9	0.164	1	3
Ba_2Cd	9	0.5841	1	1
$NaPSn$	9	0.1109	1	1
Li_2Tl_2	10	0.1298	1	1
Au_2Br_2	10	0.2151	1	1
Ge_2Te_2	10	0.2074	1	1
Br_2Tb_2	10	0.1094	1	1
N_3Na	10	0.22	1	1
As_4	10	0.473	1	1
Au_2I_2	10	0.6078	1	1
O_2Sn_2	10	0.3274	1	1
La_2S_2	10	0.638	1	1
PbS_2Sn	10	0.6047	1	1
As_2Sn_2	10	0.1088	1	1
Pt_2Te_2	10	0.1109	1	1
Se_2Sn_2	10	0.2127	1	1
As_2O_3	11	0.1599	1	1
In_2Se_3	11	0.1107	1	1
Bi_2S_3	11	0.1094	1	1
NaO_4	11	0.137	1	1
$AgNO_3$	11	0.1377	1	1
$Ho_2I_2S_2$	12	0.2409	1	1
NiO_2	12	0.1487	1	2
$Cu_2Na_2Te_2$	12	0.2182	1	1
$Gd_2I_2S_2$	12	0.052	1	1
Pd_2S_4	12	1.1171	1	1
$Br_2Er_2Se_2$	12	0.0715	1	1
$GeNi_3Te_2$	12	0.111	1	1
$Er_2I_2Se_2$	12	0.0676	1	1
$I_2Se_2Tm_2$	12	0.0678	1	1
$Ag_2K_2Te_2$	12	0.0071	1	1
Cu_3Se_3	12	0.1104	1	1
AuI_4Li	12	0.3335	1	1
Br_2Ca_3Si	12	0.1075	1	1
$H_2I_2Sr_2$	12	0.6132	1	1
$I_2Se_2Yb_2$	12	0.0682	1	1
$As_2Mg_2Na_2$	12	0.2138	1	1
N_2Re	12	0.1512	1	2
$I_2La_2O_2$	12	0.569	1	1
CoO_2	12	0.1485	1	2

Table showing the first 50 lattice matching combinations with the smallest number of atoms in the supercell.

Lattice matching - minimal strain

Formula	N° atoms	strain	cell size 1	cell size 2
Ca_2Cl_2	118	0.0	9	16
$Ge_2Se_2Zr_2$	150	0.0	9	16
$Br_2Gd_2O_2$	246	0.0	16	25
$Ge_2Mn_2Sr_2$	612	0.0001	41	61
Sn	530	0.0001	61	164
Pb_2Se_2	878	0.0001	81	98
GeI_2La_2	613	0.0002	48	65
$As_2Mg_2Na_2$	882	0.0002	65	82
F_2Zn	171	0.0002	16	25
$I_2Nd_2O_2$	366	0.0003	25	36
$RhTe_2$	237	0.0003	20	39
$Ge_2Te_2Zr_2$	780	0.0003	49	81
Cl_2Rb_2	132	0.0003	16	9
$GeTe_2$	411	0.0003	36	65
$Br_2F_2Pb_2$	876	0.0004	61	85
Cl_2Zn	945	0.0004	85	145
I_2Tm	483	0.0004	48	65
$Br_2Lu_2O_2$	780	0.0005	49	81
K	472	0.0005	65	82
Ba_2Cd	849	0.0006	85	113
I_2Nd	942	0.0006	89	136
$CuGeO_3$	925	0.0006	70	101
$CdClHO$	276	0.0006	20	39
$LiOS_2Ti$	974	0.0006	54	130
$Br_2Ca_2F_2$	246	0.0006	16	25
As_2Co_2	118	0.0007	9	16
$Br_2F_2Sr_2$	876	0.0007	61	85
$Ge_2Hf_2Te_2$	930	0.0007	58	97
$I_2Lu_2O_2$	246	0.0007	16	25
K_2PtTe_2	300	0.0007	35	18
I_4Zr_2	798	0.0007	70	63
$Br_2F_2Tm_2$	246	0.0008	16	25
IO_3Tl	973	0.0008	108	65
Se_2Ta_4	972	0.0009	53	109
Ag_2Te_2	348	0.0009	28	45
$Ca_2Ge_2Mn_2$	246	0.0009	16	25
$Br_2H_2Sr_2$	876	0.0009	61	85
Se_2Ta_4	894	0.0009	49	100
$Br_2O_2V_2$	138	0.001	8	15
Cl_2Mn	633	0.001	48	115
$As_2Mg_2Na_2$	870	0.001	64	81
$I_2La_2O_2$	510	0.001	36	49
I_2Yb	483	0.001	48	65
Cl_2NSc_2	863	0.001	48	115
$Br_2F_2Yb_2$	246	0.001	16	25
$I_2S_2Tb_2$	930	0.001	76	79
$FeSe_2$	129	0.0011	9	25
Fe_2Te_2	814	0.0011	65	106
$Cl_2F_2Pb_2$	366	0.0011	25	36
$As_2Fe_2Li_2$	930	0.0012	58	97

Table showing the first 50 lattice matching combinations with the smallest strain needed to build the supercell.

AlOCl (Pmmn)

Structural and electronic properties

**Formula**	AlOCl
**Spacegroup**	Pmmn
**Prototype**	FeOCl
**Parent 3D**	$Al_2O_2Cl_2$
**Source DB**	COD
**DB ID**	9009194
**DF2-C09 Binding energy [meV/ Å^2]**	11.76
**RVV10 Binding energy [meV/ Å^2]**	19.01
**Band gap (PBE) [eV]**	5.77

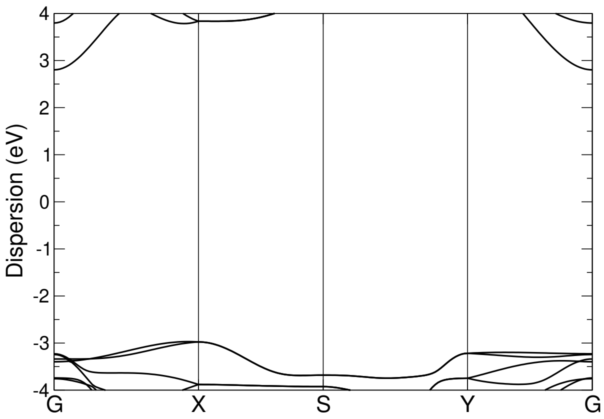

Band structure: Electronic band structure of AlOCl (Pmmn) in a representative window around the Fermi energy and along a high-symmetry path.

Optimized crystal structure

Structural parameters: relaxed cell (top) and atomic positions (bottom) of AlOCl (Pmmn) in Cartesian coordinates.

		x [Å]	y [Å]	z [Å]
$\mathbf{a_1}$		3.17122695	0.00000000	0.00000000
$\mathbf{a_2}$		0.00000000	3.66843396	0.00000000
$\mathbf{a_3}$		0.00000000	0.00000000	25.06678055
		x [Å]	y [Å]	z [Å]
●	Al	1.58561347	0.00000000	11.70192284
●	Cl	0.00000000	0.00000000	10.01373227
●	O	1.58561347	1.83421698	12.14566545
●	Al	0.00000000	1.83421698	13.36485905
●	Cl	1.58561347	1.83421698	15.05304913
●	O	0.00000000	0.00000000	12.92111669

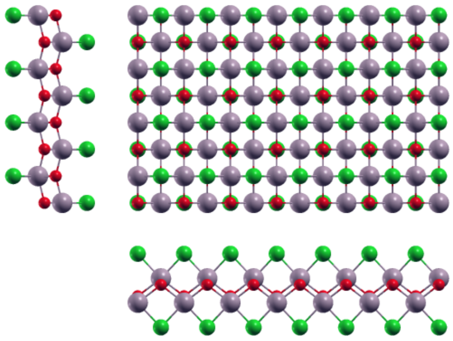

Orthographic projections: views of AlOCl (Pmmn) as seen from the x axis (left), the y axis (bottom) and the z axis (center).

Lattice matching - minimal size

Formula	N° atoms	strain	cell size 1	cell size 2
Tl	7	0.0428	1	1
HgO	8	0.2832	1	1
Bi_2	8	0.2414	1	1
CaCl	8	0.3084	1	1
I_2Mg	9	0.2167	1	1
$AgTe_2$	9	0.0412	1	1
Br_2Zn	9	0.5642	1	1
$HfTe_2$	9	0.6434	1	1
Br_2La	9	0.2172	1	1
NSr_2	9	0.5998	1	1
LiO_2	9	0.0931	1	1
Cl_2Zn	9	0.0618	1	1
FeI_2	9	0.6038	1	1
Te_2Ti	9	0.5651	1	1
CrI_2	9	0.6024	1	1
I_2Zn	9	0.204	1	1
Bi_2Pd	9	0.2985	1	1
Te_2Zr	9	0.6451	1	1
$NiTe_2$	9	0.5746	1	1
CoI_2	9	0.5962	1	1
GeS_2	9	0.2877	1	1
$MnSe_2$	9	0.3082	1	1
Br_2Mg	9	0.6034	1	1
I_2Ti	9	0.5972	1	1
F_2Ni	9	0.0579	1	1
F_2Na	9	0.57	1	1
Se_2Sn	9	0.5991	1	1
$HfSe_2$	9	0.5652	1	1
Cl_2OOs	10	0.0505	1	1
Br_2Pr_2	10	0.5677	1	1
$AgCuTe_2$	10	0.4706	1	1
Pb_2Se_2	10	0.5258	1	1
O_2Sn_2	10	0.3433	1	1
Ca_2O_2	10	0.3006	1	1
Cl_2OV	10	0.0121	1	1
Fe_2Se_2	10	0.0573	1	1
Cl_2ORu	10	0.0449	1	1
As_2Co_2	10	0.0528	1	1
Cu_2Te_2	10	0.34	1	1
$AgBrO_2$	10	0.1671	1	1
Ge_2S_2	10	1.2346	1	1
C_2Li_2	10	0.2786	1	1
Br_2OV	10	0.0337	1	1
Mg_4	10	0.0429	1	1
Cu_2I_2	10	0.2181	1	1
Fe_2S_2	10	0.3047	1	1
CaClHO	10	0.5728	1	1
Co_2S_2	10	0.3095	1	1
As_2Fe_2	10	0.2891	1	1
O_2Sn_2	10	0.9216	1	1

Table showing the first 50 lattice matching combinations with the smallest number of atoms in the supercell.

Lattice matching - minimal strain

Formula	N° atoms	strain	cell size 1	cell size 2
Bi_2O_2	792	0.0003	90	63
CNNa	654	0.0003	81	56
Br_2Mn	666	0.0004	73	76
CoO_2	177	0.0004	16	27
Cl_2Hf_2	684	0.0004	64	75
Bi_2Se_2Te	39	0.0004	4	3
$CuTe_2$	435	0.0004	48	49
$TaTe_2$	438	0.0004	49	48
$Cl_2Ho_2O_2$	624	0.0004	54	50
$HNiO_2$	628	0.0005	52	79
$I_2N_2Zr_2$	894	0.0005	76	73
$Br_2Hf_2N_2$	870	0.0005	81	64
H_2MgO_2	38	0.0005	3	4
HfS_2	435	0.0005	48	49
Sb_2SeTe_2	39	0.0005	4	3
KS_2Ti	742	0.0005	73	76
In_2Te_3	39	0.0005	4	3
N_3W_2	862	0.0005	62	98
As_4	546	0.0006	63	42
I_2V	474	0.0006	54	50
NiO_2	177	0.0006	16	27
$AsSn_2$	438	0.0007	49	48
$Cu_2Na_2Te_2$	678	0.0007	70	43
ClH_3O	242	0.0007	27	16
CaH_2O_2	533	0.0007	48	49
GeI_2Y_2	39	0.0007	4	3
$Cl_2O_2Y_2$	624	0.0007	54	50
$CoTe_2$	435	0.0007	48	49
$Br_2La_2O_2$	918	0.0008	90	63
$Hf_2I_2N_2$	582	0.0008	49	48
CdClHO	486	0.0008	49	48
$Eu_2F_2I_2$	918	0.0008	90	63
Ga_2S_2	484	0.0009	48	49
$Cu_2Se_2Tl_2$	918	0.0009	90	63
$Cl_2N_2Zr_2$	582	0.0009	48	49
As_2Li_2Nd	39	0.0009	4	3
Ga_2Se_2	748	0.0009	76	73
FeO_2	177	0.0009	16	27
Sn_2Te_2	412	0.0009	50	28
Br_2Zr_2	516	0.001	50	54
Ag_2I_2	764	0.001	92	53
Cl_2ORu	86	0.0011	9	8
Bi_2Se_4	324	0.0011	40	14
As_2O_3	893	0.0011	108	49
$F_2I_2Sm_2$	972	0.0012	95	67
O_2Pb_2	838	0.0012	95	67
Ga_2Se_2	524	0.0012	54	50
Cl_2Zn	666	0.0012	73	76
$PtSe_2$	438	0.0012	49	48
S_2V	30	0.0012	3	4

Table showing the first 50 lattice matching combinations with the smallest strain needed to build the supercell.

As (Pmna)

Structural and electronic properties

**Formula**	As
**Spacegroup**	Pmna
**Prototype**	P(black)
**Parent 3D**	As_4
**Source DB**	COD
**DB ID**	9008573
**DF2-C09 Binding energy [meV/ Å^2]**	35.57
**RVV10 Binding energy [meV/ Å^2]**	32.71
**Band gap (PBE) [eV]**	0.75

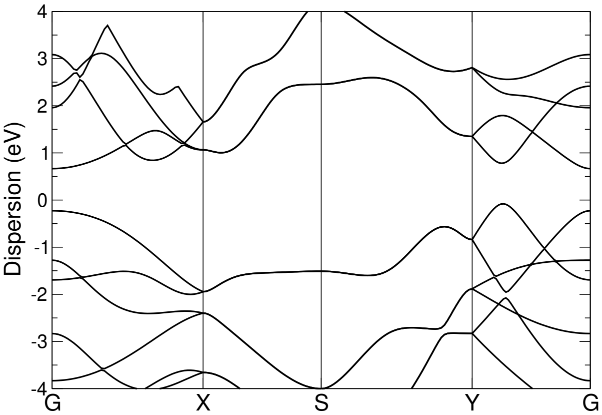

Band structure: Electronic band structure of As (Pmna) in a representative window around the Fermi energy and along a high-symmetry path.

Optimized crystal structure

Structural parameters: relaxed cell (top) and atomic positions (bottom) of As (Pmna) in Cartesian coordinates.

		x [Å]	y [Å]	z [Å]
$\mathbf{a_1}$		3.69380424	0.00000000	0.00000000
$\mathbf{a_2}$		0.00000000	4.71939778	0.00000000
$\mathbf{a_3}$		0.00000000	0.00000000	22.45344252
		x [Å]	y [Å]	z [Å]
●	As	1.84690212	2.68942336	10.02558183
●	As	1.84690212	2.02997442	12.42786069
●	As	0.00000000	4.38967152	10.02558174
●	As	3.69380424	0.32972625	12.42786078

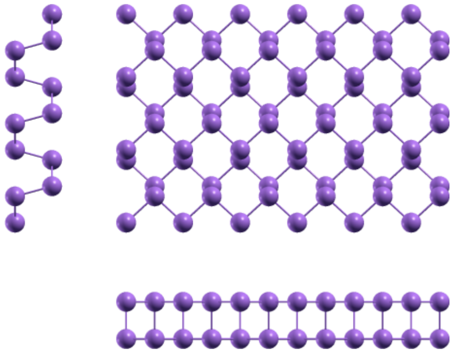

Orthographic projections: views of As (Pmna) as seen from the x axis (left), the y axis (bottom) and the z axis (center).

Lattice matching - minimal size

Formula	N° atoms	strain	cell size 1	cell size 2
Tl	5	0.1169	1	1
AgTl	6	0.3332	1	1
CaCl	6	0.2409	1	1
$MoSe_2$	7	0.117	1	1
Cl_2Zn	7	0.2601	1	1
Ba_2Hg	7	0.0591	1	1
NbS_2	7	0.1163	1	1
S_2Ta	7	0.1164	1	1
CKN	7	0.2883	1	1
Se_2V	7	0.1167	1	1
$MnSe_2$	7	0.2408	1	1
CNNa	7	0.0115	1	1
F_2Ni	7	0.2546	1	1
Ba_2Cd	7	0.0602	1	1
Se_2W	7	0.117	1	1
Cu_2I_2	8	0.3343	1	1
Cl_2OOs	8	0.2552	1	1
$AgNO_2$	8	0.1824	1	1
$AgCuTe_2$	8	0.2998	1	1
O_2Sn_2	8	0.0447	1	1
Au_2Br_2	8	0.0636	1	1
Ge_2Te_2	8	0.269	1	1
Fe_2Se_2	8	0.2538	1	1
As_2Ir_2	8	0.3106	1	1
Cu_2Te_2	8	0.2637	1	1
O_2Pb_2	8	0.3282	1	1
Ge_2S_2	8	0.0235	1	1
P_4	8	0.1687	1	1
Au_2I_2	8	0.0743	1	1
Co_2S_2	8	0.2418	1	1
O_2Sn_2	8	0.0791	1	1
Cu_2Se_2	8	0.2553	1	1
Bi_2O_2	8	0.3306	1	1
$AgClO_2$	8	0.1024	1	1
PbS_2Sn	8	0.358	1	1
Se_2Sn_2	8	0.279	1	1
Co_2Se_2	8	0.2491	1	1
As_2O_3	9	0.2829	1	1
F_4Sn	9	0.0588	1	1
Hf_3Te_2	9	0.2586	1	1
F_4Nb	9	0.0569	1	1
$LiOS_2Ti$	9	0.116	1	1
Cl_4Mn	9	0.3539	1	1
$Br_2In_2O_2$	10	0.3113	1	1
$Br_2Ho_2S_2$	10	0.5377	1	1
$Ge_2Hf_2Te_2$	10	0.2651	1	1
$Br_2F_2Sr_2$	10	0.0565	1	1
$Cl_2O_2V_2$	10	0.5566	1	1
$Ho_2I_2S_2$	10	0.5487	1	1
Cu_4Te_2	10	0.5534	1	1

Table showing the first 50 lattice matching combinations with the smallest number of atoms in the supercell.

Lattice matching - minimal strain

Formula	N° atoms	strain	cell size 1	cell size 2
$Br_2Dy_2S_2$	792	0.0001	90	72
Sn	120	0.0005	20	40
$Al_2Cl_2O_2$	546	0.0006	42	63
AsKSn	527	0.0007	71	81
Bi_2Pd	535	0.0008	61	97
$Dy_2I_2S_2$	624	0.0009	72	56
GdI_2	575	0.0009	80	85
I_2La_2P	745	0.0009	80	85
Bi_2Te_2	608	0.0009	81	71
$Cl_2Rh_2Te_2$	58	0.0009	7	5
Bi_2SeTe_2	642	0.0009	68	74
Au_2I_2	596	0.0009	78	71
Sb_2SeTe_2	539	0.0009	56	63
In_2Te_3	539	0.001	56	63
Bi_2	420	0.001	68	74
$I_2S_2Tm_2$	708	0.001	81	64
Bi_2Se_2Te	539	0.001	56	63
I_2La_2	608	0.001	71	81
$Br_2Er_2S_2$	896	0.0011	101	82
$I_2S_2Tb_2$	624	0.0011	72	56
$Ga_2I_2Y_2$	770	0.0011	71	81
CNb_2S_2	321	0.0012	24	45
$Er_2I_2S_2$	708	0.0012	81	64
ClH_3O	532	0.0012	63	56
Bi_2Mn_2	468	0.0012	48	69
Br_2La_2	264	0.0012	30	36
GeI_2Y_2	539	0.0012	56	63
Se_2V	463	0.0013	49	89
Ca_2Si	500	0.0013	74	68
$F_2Lu_2Se_2$	336	0.0013	30	36
$Br_2F_2Sr_2$	702	0.0013	69	71
BaF_2	527	0.0013	71	81
C_4Ca_2	908	0.0013	89	92
$I_2Se_2Yb_2$	888	0.0013	105	78
$Br_2O_2V_2$	790	0.0013	64	89
HgO	584	0.0014	80	132
Cr_2O_4	896	0.0014	65	106
Sb_2	192	0.0014	30	36
$Br_2S_2Yb_2$	980	0.0014	110	90
As_2Li_2Nd	539	0.0014	56	63
$Br_2Ho_2S_2$	812	0.0014	92	74
$C_2Br_2Y_2$	570	0.0014	48	63
F_4Nb	631	0.0014	69	71
$Br_2F_2Pb_2$	702	0.0015	69	71
$F_2I_2Pb_2$	634	0.0015	64	63
$I_2S_2Tl_2$	82	0.0015	7	9
P_2	186	0.0015	24	45
F_2Ni	55	0.0015	7	9
$Si_2Te_2Zr_2$	82	0.0015	7	9
Ba_2Cd	445	0.0015	64	63

Table showing the first 50 lattice matching combinations with the smallest strain needed to build the supercell.

$AsSe_2$ (P3m1)

Structural and electronic properties

**Formula**	$AsSe_2$
**Spacegroup**	P3m1
**Prototype**	MoS2
**Parent 3D**	$AsSe_2$
**Source DB**	MPDS
**DB ID**	S1832857
**DF2-C09 Binding energy [meV/ Å^2]**	25.13
**RVV10 Binding energy [meV/ Å^2]**	N/A
**Band gap (PBE) [eV]**	0.0

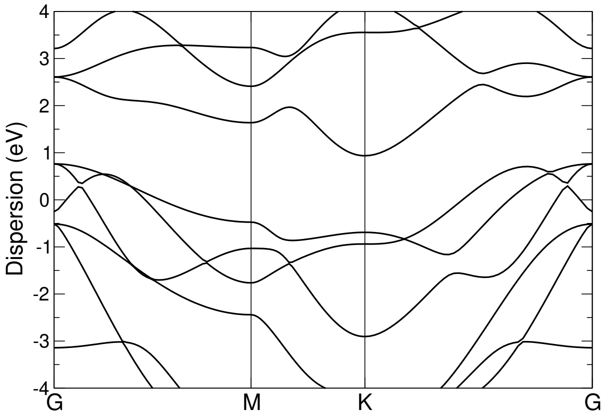

Band structure: Electronic band structure of $AsSe_2$ (P3m1) in a representative window around the Fermi energy and along a high-symmetry path.

Optimized crystal structure

Structural parameters: relaxed cell (top) and atomic positions (bottom) of $AsSe_2$ (P3m1) in Cartesian coordinates.

		x [Å]	y [Å]	z [Å]
$\mathbf{a_1}$		−1.73684213	−3.00829882	0.00000000
$\mathbf{a_2}$		3.47368426	0.00000000	0.00000000
$\mathbf{a_3}$		0.00000000	0.00000000	19.21153729
		x [Å]	y [Å]	z [Å]
●	As	0.00000000	-2.00553254	-0.00018816
●	Se	1.73684213	-1.00276627	1.79289947
●	Se	1.73684213	-1.00276627	-1.79271131

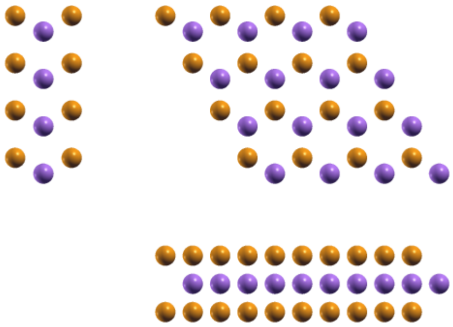

Orthographic projections: views of $AsSe_2$ (P3m1) as seen from the x axis (left), the y axis (bottom) and the z axis (center).

Lattice matching - minimal size

Formula	N° atoms	strain	cell size 1	cell size 2
Tl	4	0.1327	1	1
Sn	4	0.1139	1	1
In	4	0.1158	1	1
In	4	0.2751	1	1
HgO	5	0.1425	1	1
AsSb	5	0.4563	1	1
GeTe	5	0.4715	1	1
S_2	5	0.4755	1	1
Mg_2	5	0.1218	1	1
$IrTe_2$	6	0.4738	1	1
CrS_2	6	0.2542	1	1
$CdCl_2$	6	0.4688	1	1
$AgTe_2$	6	0.135	1	1
$ReSe_2$	6	0.0023	1	1
$InSe_2$	6	0.4697	1	1
$GeTe_2$	6	0.4663	1	1
$HfTe_2$	6	0.4955	1	1
I_2Mn	6	0.4691	1	1
NSr_2	6	0.4603	1	1
PbS_2	6	0.45	1	1
ReS_2	6	0.2624	1	1
FeI_2	6	0.4635	1	1
I_2Ni	6	0.4667	1	1
S_2Ti	6	0.0083	1	1
Mg_3	6	0.1284	1	1
CrI_2	6	0.4627	1	1
Bi_2Pd	6	0.1513	1	1
Ba_2Hg	6	0.3167	1	1
Cl_2Ni	6	0.0014	1	1
Cl_2Co	6	0.0086	1	1
$CrTe_2$	6	0.0047	1	1
Br_2V	6	0.0031	1	1
ClNZr	6	0.0052	1	1
Ba_2N	6	0.4996	1	1
Se_2Ti	6	0.0086	1	1
Br_2Ti	6	0.0041	1	1
Te_2Zr	6	0.4968	1	1
CdO_2	6	0.0087	1	1
BrNZr	6	0.0013	1	1
$NbSe_2$	6	0.001	1	1
CoI_2	6	0.4574	1	1
O_2Zn	6	0.2497	1	1
Br_2Cr	6	0.0044	1	1
Cl_2Zr	6	0.0089	1	1
$FeSe_2$	6	0.1115	1	1
Se_2Ta	6	0.0009	1	1
Br_2Mg	6	0.4632	1	1
I_2Ti	6	0.4582	1	1
$NbSe_2$	6	0.0003	1	1
Se_2Ta	6	0.0033	1	1

Table showing the first 50 lattice matching combinations with the smallest number of atoms in the supercell.

Lattice matching - minimal strain

Formula	N° atoms	strain	cell size 1	cell size 2
Ni_2SbTe_2	563	0.0	81	64
$HfTe_2$	339	0.0	64	49
Cl_2Tb_2	447	0.0	73	57
C_2F_2	91	0.0	9	16
I_2Ni	492	0.0001	91	73
In_2Se_3	437	0.0001	64	49
$Er_2F_2Se_2$	486	0.0002	64	49
S_2	371	0.0002	81	64
$AgCuTe_2$	553	0.0002	91	70
Ba_2Cu_2	331	0.0002	61	37
S_2Sn_2	378	0.0002	70	42
Ag_2	66	0.0002	16	9
$GeTe_2$	492	0.0002	91	73
I_2Yb	390	0.0003	81	49
Nd	172	0.0003	37	61
Cu_2Sr_2	139	0.0003	25	16
$NbSe_2$	6	0.0003	1	1
$Br_2Lu_2O_2$	483	0.0003	65	48
$F_2Ho_2Se_2$	429	0.0003	57	43
Br_2OV	38	0.0003	6	5
NSr_2	543	0.0003	100	81
Cl_2La_2	291	0.0003	49	36
O_2Zn	339	0.0003	49	64
N_2W	300	0.0004	43	57
I_2Yb	294	0.0004	61	37
$PbTe_2$	183	0.0005	36	25
$Ge_2Te_2Zr_2$	483	0.0005	65	48
I_2Y_2	447	0.0005	73	57
Ba_2Pt	75	0.0005	16	9
$I_2Pr_2S_2$	102	0.0005	16	9
Te_2Zr	339	0.0005	64	49
Ni_2Te_2	565	0.0005	91	73
CrI_2	543	0.0006	100	81
Fe_2SeTe	387	0.0006	65	48
Se_2Sn	543	0.0006	100	81
$Br_2Eu_2O_2$	840	0.0006	118	81
I_2La_2P	155	0.0006	25	16
Ga_2Se_2	499	0.0007	81	64
CeI_2	597	0.0007	118	81
Pt_2Te_2	388	0.0007	64	49
Hg_3N_2	17	0.0007	4	1
As_2Sn_2	291	0.0007	49	36
Br_2Gd_2Ge	233	0.0007	36	25
Cu_2I_2	208	0.0007	36	25
CI_2Y_2	705	0.0007	100	81
$CdCl_2$	492	0.0007	91	73
$Er_2I_2S_2$	519	0.0008	89	42
P_2Sn_2	499	0.0008	81	64
Cl_2Gd_2	447	0.0008	73	57
Br_2PY_2	386	0.0008	57	43

Table showing the first 50 lattice matching combinations with the smallest strain needed to build the supercell.

AuBr (Cmme)

Structural and electronic properties

**Formula**	AuBr
**Spacegroup**	Cmme
**Prototype**	AuI
**Parent 3D**	Au_4Br_4
**Source DB**	COD
**DB ID**	1510610
**DF2-C09 Binding energy [meV/ Å^2]**	12.72
**RVV10 Binding energy [meV/ Å^2]**	20.95
**Band gap (PBE) [eV]**	2.03

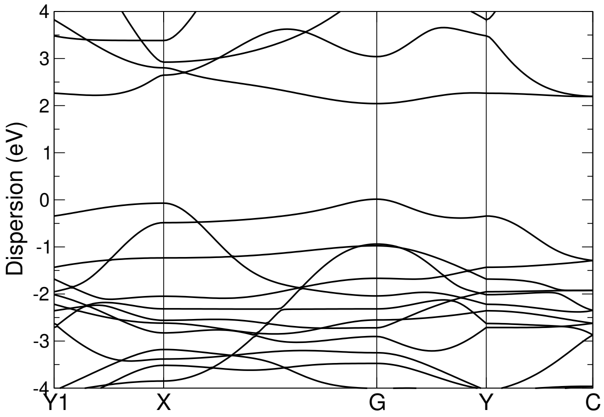

Band structure: Electronic band structure of AuBr (Cmme) in a representative window around the Fermi energy and along a high-symmetry path.

Optimized crystal structure

Structural parameters: relaxed cell (top) and atomic positions (bottom) of AuBr (Cmme) in Cartesian coordinates.

	x [Å]	y [Å]	z [Å]
$\mathbf{a_1}$	4.26295290	0.05761046	0.00000000
$\mathbf{a_2}$	0.05761046	4.26295290	0.00000000
$\mathbf{a_3}$	0.00000000	0.00000000	24.03584981

		x [Å]	y [Å]	z [Å]
●	Au	3.24042086	3.24042086	12.01792491
●	Au	1.08014249	1.08014249	12.01792491
●	Br	0.02880523	2.13147645	10.01723859
●	Br	2.13147645	0.02880523	14.01861123

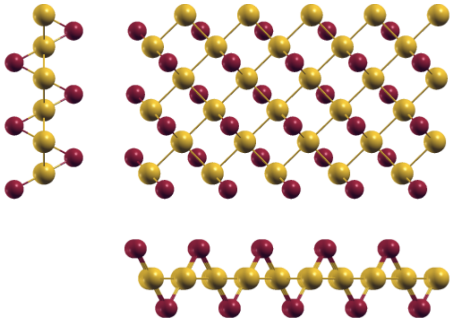

Orthographic projections: views of AuBr (Cmme) as seen from the x axis (left), the y axis (bottom) and the z axis (center).

Lattice matching - minimal size

Formula	N° atoms	strain	cell size 1	cell size 2
K	5	0.009	1	1
K	5	0.1325	1	1
Ag_2	6	0.1371	1	1
Sb_2	6	0.1161	1	1
CaCl	6	0.5336	1	1
Nd	7	0.1425	1	3
Ba_2Pt	7	0.1369	1	1
$ReSe_2$	7	0.1134	1	1
CaI_2	7	0.1256	1	1
$HfTe_2$	7	0.1115	1	1
Br_2Cu	7	0.0999	1	1
Ca_2Si	7	0.1409	1	1
I_2Yb	7	0.1238	1	1
$AuTe_2$	7	0.1135	1	1
Cl_2Zn	7	0.2098	1	1
$PdTe_2$	7	0.1128	1	1
I_2Zn	7	0.1149	1	1
Ba_2Hg	7	0.0106	1	1
Cl_2Ni	7	0.1132	1	1
CNRb	7	0.3326	1	1
Ba_2N	7	0.1118	1	1
Te_2Zr	7	0.1116	1	1
I_2Tm	7	0.1248	1	1
I_2Pb	7	0.1385	1	1
BrNZr	7	0.1126	1	1
$NbSe_2$	7	0.1131	1	1
$MnSe_2$	7	0.5333	1	1
Br_2Cr	7	0.1088	1	1
DyI_2	7	0.1276	1	1
Se_2Ta	7	0.1131	1	1
$NbSe_2$	7	0.1128	1	1
CNNa	7	0.3175	1	1
F_2Ni	7	0.2056	1	1
$PtTe_2$	7	0.1133	1	1
Br_2Cd	7	0.1125	1	1
F_2Zn	7	0.6667	1	1
Ba_2Cd	7	0.0087	1	1
NaPSn	7	0.1113	1	1
Bi_2Te_2	8	0.1532	1	1
Fe_2Te_2	8	0.2224	1	1
Li_2Tl_2	8	0.2011	1	1
Ca_2Cl_2	8	0.2229	1	1
Cl_2OOs	8	0.2075	1	1
NS_2Zr	8	0.1073	1	1
$AgNO_2$	8	0.3435	1	1
Br_2Er_2	8	0.1136	1	1
$AgCuTe_2$	8	0.0716	1	1
O_2Sn_2	8	0.2161	1	1
S_2Sn_2	8	0.0172	1	1
Cu_2S_2	8	0.2291	1	1

Table showing the first 50 lattice matching combinations with the smallest number of atoms in the supercell.

Lattice matching - minimal strain

Formula	N° atoms	strain	cell size 1	cell size 2
Se_2Ti	506	0.0003	56	94
PbTe	404	0.0003	64	74
As_2CeLi_2	626	0.0003	64	74
CNb_2S_2	825	0.0004	60	117
Br_2Zr_2	600	0.0004	56	94
Cu_2O_4	948	0.0004	72	110
C_2Li_2	708	0.0004	81	96
Bi_2Se_2	396	0.0004	49	50
$Ho_2I_2Se_2$	394	0.0004	46	35
Co_2S_2	576	0.0004	60	84
CaCl	408	0.0004	60	84
GeI_2	478	0.0004	64	74
$MnSe_2$	492	0.0005	60	84
In	296	0.0005	49	100
Cu_2O_4	672	0.0006	51	78
$Gd_2I_2S_2$	396	0.0006	45	36
$Cl_2Hg_2N_2$	472	0.0006	61	38
Cu_2O_4	646	0.0006	49	75
$Cl_2Hg_2N_2$	448	0.0006	58	36
P_2	474	0.0007	60	117
$Ca_2Cl_2F_2$	732	0.0007	63	80
Bi_2STe_2	626	0.0007	64	74
$LiMnTe_2$	552	0.0007	64	74
Bi_2STe_2	644	0.0008	66	76
$Hf_2Si_2Te_2$	438	0.0008	36	49
I_4Zr_2	810	0.0008	99	69
$LiMnTe_2$	508	0.0008	59	68
Bi_2STe_2	576	0.0008	59	68
C_2	240	0.0008	22	76
$Cl_2S_2Tl_2$	412	0.0008	49	36
Cl_2Ti	591	0.0008	60	117
GeI_2	440	0.0009	59	68
CdI_2	492	0.0009	66	76
$Cl_2Hg_2N_2$	410	0.0009	53	33
Cu_2O_4	620	0.0009	47	72
CS_2Ta_2	825	0.0009	60	117
$NbSe_2$	690	0.001	75	130
Br_2La_2P	644	0.001	66	76
As_2CeLi_2	576	0.001	59	68
PbTe	372	0.001	59	68
Co_2Se_2	340	0.001	36	49
$Er_2I_2Se_2$	866	0.001	101	77
$Hf_2I_2N_2$	396	0.001	30	46
CdClHO	304	0.001	30	46
Bi_2STe_2	558	0.001	57	66
CdI_2	440	0.001	59	68
$Er_2I_2Se_2$	832	0.001	97	74
BrNZr	690	0.001	75	130
$Ho_2I_2Se_2$	360	0.001	42	32
$Br_2Ho_2O_2$	914	0.001	80	99

Table showing the first 50 lattice matching combinations with the smallest strain needed to build the supercell.

AuI (Cmme)

Structural and electronic properties

**Formula**	AuI
**Spacegroup**	Cmme
**Prototype**	AuI
**Parent 3D**	Au_4I_4
**Source DB**	COD
**DB ID**	9008986
**DF2-C09 Binding energy [meV/ Å^2]**	15.1
**RVV10 Binding energy [meV/ Å^2]**	22.4
**Band gap (PBE) [eV]**	1.99

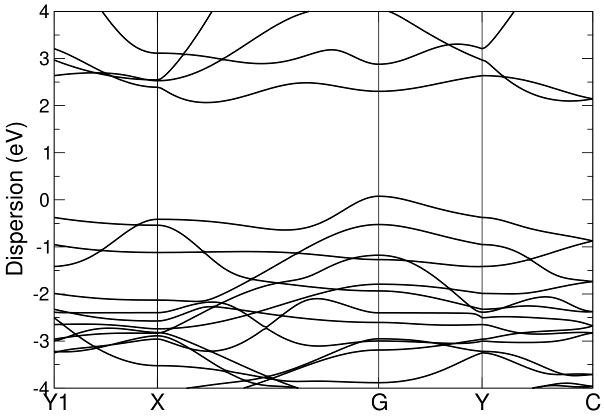

Band structure: Electronic band structure of AuI (Cmme) in a representative window around the Fermi energy and along a high-symmetry path.

Optimized crystal structure

Structural parameters: relaxed cell (top) and atomic positions (bottom) of AuI (Cmme) in Cartesian coordinates.

	x [Å]	y [Å]	z [Å]
$\mathbf{a_1}$	4.38422740	−0.15246095	0.00000000
$\mathbf{a_2}$	−0.15246095	4.38422737	0.00000000
$\mathbf{a_3}$	0.00000000	0.00000000	24.36756060

		x [Å]	y [Å]	z [Å]
●	Au	3.25044651	0.98204510	12.18393985
●	Au	0.98131994	3.24972132	12.18393985
●	I	-0.07623047	2.19211368	10.01195940
●	I	2.19211370	-0.07623047	14.35500646

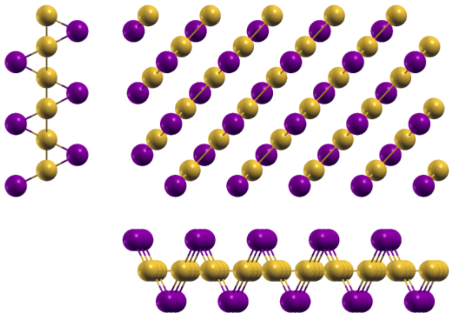

Orthographic projections: views of AuI (Cmme) as seen from the x axis (left), the y axis (bottom) and the z axis (center).

Lattice matching - minimal size

Formula	N° atoms	strain	cell size 1	cell size 2
As_2	6	0.3238	1	1
Na	6	0.6463	1	2
Sb_2	6	0.1199	1	1
Sm	6	0.1471	1	2
I_2Mg	7	0.122	1	1
PSn_2	7	0.1038	1	1
Br_2Zn	7	0.101	1	1
HfS_2	7	0.3285	1	1
$AsSn_2$	7	0.1027	1	1
$HfTe_2$	7	0.1159	1	1
I_2Pr	7	0.6614	1	1
$CuTe_2$	7	0.3279	1	1
S_2Zr	7	0.104	1	1
Br_2La	7	0.1221	1	1
Br_2Co	7	0.3231	1	1
Ca_2N	7	0.3247	1	1
$AuTe_2$	7	0.1176	1	1
BrCdI	7	0.1232	1	1
Cl_2Zn	7	0.5187	1	1
$PdTe_2$	7	0.117	1	1
HgI_2	7	0.0204	1	1
Te_2Ti	7	0.1009	1	1
I_2Zn	7	0.1189	1	1
$RhTe_2$	7	0.102	1	1
GeI_2	7	0.1213	1	1
Ba_2Hg	7	0.0253	1	1
CNRb	7	0.0789	1	1
$CoTe_2$	7	0.329	1	1
Ba_2N	7	0.1162	1	1
Te_2Zr	7	0.116	1	1
$PbTe_2$	7	0.1228	1	1
$NiTe_2$	7	0.1001	1	1
S_2Sn	7	0.1039	1	1
$SnTe_2$	7	0.1205	1	1
$PtSe_2$	7	0.1025	1	1
Br_2Fe	7	0.3232	1	1
Br_2Ni	7	0.3319	1	1
CeI_2	7	0.6588	1	1
Se_2Yb	7	0.1214	1	1
Cl_2Mg	7	0.332	1	1
$BiTe_2$	7	0.1214	1	1
F_2Ni	7	0.2037	1	1
$PtTe_2$	7	0.1175	1	1
Br_2Cd	7	0.1168	1	1
I_2La	7	0.6807	1	1
F_2Zn	7	0.6483	1	1
Ba_2Cd	7	0.0235	1	1
NaPSn	7	0.1157	1	1
$HfSe_2$	7	0.1009	1	1
CdClHO	8	0.103	1	1

Table showing the first 50 lattice matching combinations with the smallest number of atoms in the supercell.

Lattice matching - minimal strain

Formula	N° atoms	strain	cell size 1	cell size 2
CdClO	552	0.0004	60	104
OTl_2	552	0.0004	60	104
$Cl_2Hf_2N_2$	864	0.0004	60	104
NS_2Ta	664	0.0006	48	118
I_2Pb	552	0.0006	78	80
I_2Ni	548	0.0006	65	96
$CrSe_2$	398	0.0006	38	82
ReS_2	568	0.0006	52	120
$Ge_2Se_2Zr_2$	306	0.0006	24	35
Te_2V	552	0.0006	60	104
Ho_2S_2	336	0.0006	37	47
Ca_2Cl_2	236	0.0006	24	35
FHOZn	480	0.0006	38	82
CoH_2O_2	808	0.0007	52	120
K_2PdSe_2	562	0.0007	88	42
N_4	708	0.0007	56	121
$Cl_2Fe_2O_2$	608	0.0008	44	72
Ga_2Te_2	744	0.0008	81	105
Ba_2Ni_3	849	0.0008	81	105
$I_2Pr_2Si_2$	954	0.0009	81	105
GeI_2	639	0.0009	81	105
$AsCuLi_2$	744	0.0009	81	105
FeI_2	548	0.0009	65	96
CdH_2O_2	760	0.0009	60	104
H_2Li_2Pt	872	0.0009	63	124
$Br_2Eu_2O_2$	844	0.0009	73	92
As_4	596	0.0009	71	78
O_2Sn_2	528	0.001	52	80
I_2Pr	568	0.001	73	92
CeI_2	568	0.001	73	92
Se_2Yb	639	0.001	81	105
Ag_2	528	0.001	87	90
Sb_2Se_2Te	470	0.001	45	58
Br_2Cd	708	0.001	87	120
Ba_2Pt	618	0.001	87	90
Sb_2Se_2Te	470	0.001	45	58
Cl_2ORu	472	0.001	48	70
Sb_2Te_2	632	0.001	78	80
Br_2Mg	548	0.001	65	96
Ni_2Se_2	652	0.0011	72	91
O_2Sn_2	528	0.0011	52	80
CS_3Tl_2	786	0.0011	126	47
$Ce_2I_2Si_2$	528	0.0011	45	58
Cl_4Mg_2	594	0.0011	66	55
Br_2HLa	744	0.0011	81	105
CeI_2	561	0.0011	72	91
Br_2Ca_3Si	936	0.0011	90	96
Ni_2Te_2	644	0.0011	65	96
$BiTe_2$	639	0.0011	81	105
MnO_2	381	0.0011	30	87

Table showing the first 50 lattice matching combinations with the smallest strain needed to build the supercell.

AuSe (P2/m)

Structural and electronic properties

**Formula**	AuSe
**Spacegroup**	P2/m
**Prototype**	AuSe
**Parent 3D**	Au_2Se_2
**Source DB**	COD
**DB ID**	1510294
**DF2-C09 Binding energy [meV/ Å^2]**	27.72
**RVV10 Binding energy [meV/ Å^2]**	33.28
**Band gap (PBE) [eV]**	1.17

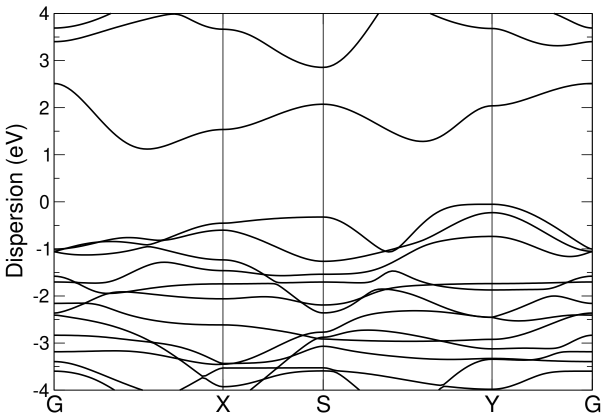

Band structure: Electronic band structure of AuSe (P2/m) in a representative window around the Fermi energy and along a high-symmetry path.

Optimized crystal structure

Structural parameters: relaxed cell (top) and atomic positions (bottom) of AuSe (P2/m) in Cartesian coordinates.

		x [Å]	y [Å]	z [Å]
$\mathbf{a_1}$		3.74010877	0.00000000	0.00000000
$\mathbf{a_2}$		0.00000000	6.29602181	0.00000000
$\mathbf{a_3}$		0.00000000	0.00000000	22.56787904
		x [Å]	y [Å]	z [Å]
●	Se	3.74010877	2.73958072	12.55823247
●	Au	1.87005439	3.82978083	11.28393842
●	Au	0.00000000	0.68177059	11.28394108
●	Se	0.00000000	4.91998273	10.00964611

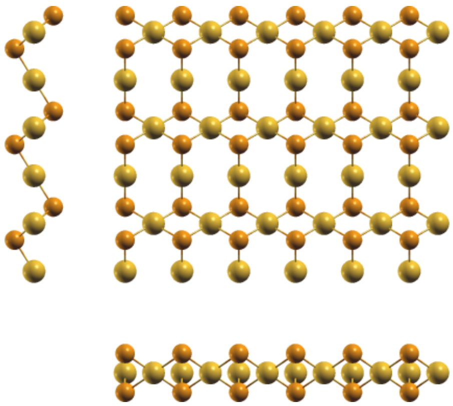

Orthographic projections: views of AuSe (P2/m) as seen from the x axis (left), the y axis (bottom) and the z axis (center).

Lattice matching - minimal size

Formula	N° atoms	strain	cell size 1	cell size 2
Tl	6	0.0475	1	2
$AuTe_2$	7	0.4921	1	1
HgO	8	0.1258	1	2
Cl_2La_2	8	0.4979	1	1
Bi_2Se_2	8	0.1845	1	1
As_2	8	0.0118	1	2
$AgClO_2$	8	0.241	1	1
In_2Se_2	8	0.501	1	1
$SbSe_2Tl$	8	0.0233	1	1
As_2Sn_2	8	0.4952	1	1
Sb_2	8	0.4723	1	2
Cl_2Zn	10	0.0126	1	2
Cl_2Mn	10	0.1199	1	2
$MoTe_2$	10	0.0165	1	2
PSn_2	10	0.0068	1	2
S_2Ta	10	0.1208	1	2
Br_2Zn	10	0.0119	1	2
HfS_2	10	0.0097	1	2
$AsSn_2$	10	0.0073	1	2
$SiTe_2$	10	0.0157	1	2
Te_2V	10	0.0148	1	2
$CuTe_2$	10	0.01	1	2
S_2Zr	10	0.0069	1	2
Br_2Cu	10	0.1365	1	2
Br_2Co	10	0.0121	1	2
$Cl_2Rh_2Te_2$	10	0.0173	1	1
Ca_2N	10	0.0113	1	2
$K_2O_2Tl_2$	10	0.006	1	1
S_2Ti	10	0.1233	1	2
$I_2Se_2Tb_2$	10	0.2279	1	1
Te_2Ti	10	0.0122	1	2
$Gd_2I_2Se_2$	10	0.5399	1	1
NbS_2	10	0.1206	1	2
I_2Zn	10	0.4641	1	2
Te_2Zn	10	0.0166	1	2
I_4Zr_2	10	0.0339	1	1
$RhTe_2$	10	0.0088	1	2
Bi_2Pd	10	0.132	1	2
$C_2Br_2Gd_2$	10	0.4623	1	1
Br_2Mn	10	0.0136	1	2
Cl_2Co	10	0.1232	1	2
$Br_2Er_2Se_2$	10	0.2259	1	1
NbS_2	10	0.1187	1	2
$CoTe_2$	10	0.0095	1	2
Cl_2Fe	10	0.1228	1	2
CdClO	10	0.0152	1	2
S_2Ta	10	0.1183	1	2
Se_2V	10	0.1178	1	2
Cl_4Mg_2	10	0.4787	1	1
$Br_2Dy_2S_2$	10	0.4675	1	1

Table showing the first 50 lattice matching combinations with the smallest number of atoms in the supercell.

Lattice matching - minimal strain

Formula	N° atoms	strain	cell size 1	cell size 2
I_2Mg	349	0.0002	40	63
Br_2La	349	0.0002	40	63
Bi_2Se_3	475	0.0003	40	63
$Ce_2I_2S_2$	516	0.0003	45	56
$Ce_2I_2Si_2$	538	0.0004	40	63
Cl_2OV	336	0.0004	28	56
$Au_2K_2S_2$	236	0.0005	35	16
Gd_2I_2	412	0.0005	40	63
$Br_2Er_2Se_2$	872	0.0005	86	88
Ge_2Te_2	996	0.0006	110	139
GdI_2	348	0.0007	42	60
Ba_2Cd	144	0.0007	18	24
Sb_2Se_2Te	475	0.0007	40	63
Ag_2I_2	260	0.0007	30	35
N_3W_2	562	0.0007	28	90
Cu_2I_2	412	0.0008	40	63
Br_2Gd_2Ge	475	0.0008	40	63
$F_2I_2Pb_2$	216	0.0008	18	24
$Ho_2I_2Se_2$	634	0.001	64	63
$I_2Se_2Tm_2$	700	0.001	70	70
$I_2Se_2Yb_2$	720	0.001	72	72
In_2Se_2	780	0.0011	96	99
Br_2Ca_3Si	514	0.0012	43	57
Sb_2Se_2Te	475	0.0012	40	63
I_2Zn	490	0.0013	55	90
CuO_2	457	0.0014	40	99
Br_2Gd_2	692	0.0014	65	108
As_2Sn_2	692	0.0014	65	108
LiO_2	307	0.0014	28	65
$Br_2N_2Ti_2$	660	0.0014	45	80
Br_2Er_2	692	0.0015	65	108
$BiTe_2$	349	0.0015	40	63
$I_2Pr_2S_2$	696	0.0015	60	76
LiO	478	0.0015	52	135
Ag_2	392	0.0015	60	76
$Cl_2N_2Ti_2$	524	0.0016	35	64
CNRb	248	0.0016	35	36
I_2La_2P	468	0.0016	42	60
Sb_2Te_2	404	0.0016	45	56
Ba_2Pt	468	0.0016	60	76
HgI_2	225	0.0017	30	35
Bi_2Se_2	676	0.0017	73	96
$AuTe_2$	584	0.0017	65	108
Se_2Yb	349	0.0018	40	63
In_2Se_2	580	0.0018	55	90
Ba_2Hg	915	0.0018	114	153
C_4Ca_2	888	0.0018	72	100
Cl_2La_2	692	0.0018	65	108
FeO_2	300	0.0019	21	72
$Cu_2Na_2Te_2$	746	0.0019	65	81

Table showing the first 50 lattice matching combinations with the smallest strain needed to build the supercell.

BN (P-6m2)

Structural and electronic properties

**Formula**	BN
**Spacegroup**	P-6m2
**Prototype**	BN
**Parent 3D**	B_2N_2
**Source DB**	ICSD
**DB ID**	186248
**DF2-C09 Binding energy [meV/ Å^2]**	19.38
**RVV10 Binding energy [meV/ Å^2]**	24.43
**Band gap (PBE) [eV]**	4.68

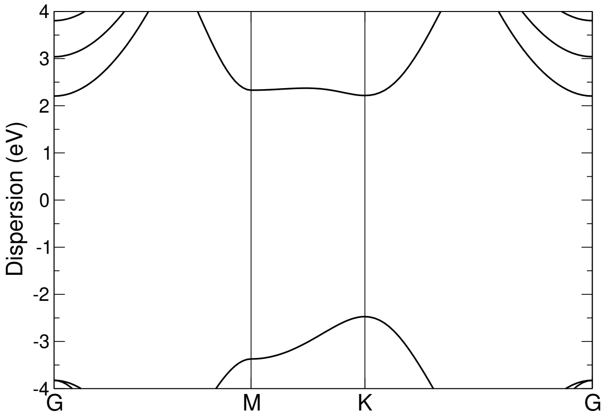

Band structure: Electronic band structure of BN (P-6m2) in a representative window around the Fermi energy and along a high-symmetry path.

Optimized crystal structure

Structural parameters: relaxed cell (top) and atomic positions (bottom) of BN (P-6m2) in Cartesian coordinates.

	x [Å]	y [Å]	z [Å]
$\mathbf{a_1}$	2.50930018	0.00000000	0.00000000
$\mathbf{a_2}$	−1.25465009	2.17311770	0.00000000
$\mathbf{a_3}$	0.00000000	0.00000000	20.00000000

		x [Å]	y [Å]	z [Å]
•	B	0.00000000	1.44874513	10.00000000
•	N	1.25465009	0.72437257	10.00000000

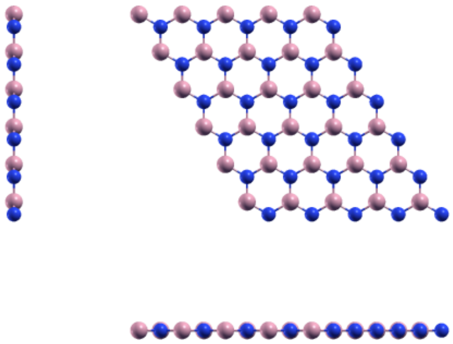

Orthographic projections: views of BN (P-6m2) as seen from the x axis (left), the y axis (bottom) and the z axis (center).

Lattice matching - minimal size

Formula	N° atoms	strain	cell size 1	cell size 2
C_2	4	0.0081	1	1
Mg_2	4	0.3277	1	1
K	5	0.5436	2	1
FeO_2	5	0.4732	1	1
NiO_2	5	0.4765	1	1
MnO_2	5	0.45	1	1
Na	5	0.2222	2	1
N_2Re	5	2.8541	1	1
CoO_2	5	0.4751	1	1
InSe	6	6.0468	1	2
Bi_2	6	6.2011	1	2
C_2Li_2	6	2.4727	1	1
PbTe	6	6.0934	1	2
$MoTe_2$	7	0.2251	2	1
Te_2Zn	7	0.2249	2	1
PtS_2	7	0.2235	2	1
Se_2Ti	7	0.2201	2	1
Te_2W	7	0.2253	2	1
H_2MnO_2	7	2.9974	1	1
H_2Li_2Pd	7	0.3317	1	1
N_3W_2	7	2.9657	1	1
H_4Ti	7	0.3329	1	1
CdI_2	8	6.1393	1	2
Cu_4Te_2	8	1.18	1	1
Br_2Ca	8	6.1721	1	2
NS_2Zr	8	0.2227	2	1
$AgCuTe_2$	8	0.2624	2	1
BiClTe	8	6.1489	1	2
S_2Sn_2	8	0.4673	2	1
Br_2Zr_2	8	0.2196	2	1
N_4	8	0.2782	2	1
GeI_2	8	6.0997	1	2
Ge_2Se_2	8	0.4353	2	1
La_2S_2	8	0.5193	2	1
CdI_2	8	6.1277	1	2
$HfLiS_2$	8	0.2245	2	1
I_2Pr	8	6.1512	1	2
K	9	0.1163	4	1
Sn	9	0.1398	4	1
In	9	0.1393	4	1
CdH_2O_2	9	0.2259	2	1
F_2Zn	9	0.1256	3	1
Ga_2S_3	9	0.2226	2	1
$LiMnTe_2$	10	6.1079	1	2
Bi_2Mn_2	10	6.1222	1	2
HgO	10	0.1355	3	2
$Cu_2Na_2Te_2$	10	0.554	2	1
$AsLi_3$	10	6.0548	1	2
Cu_2S_2	10	0.1236	3	1
$C_2Br_2Gd_2$	10	0.2839	2	1

Table showing the first 50 lattice matching combinations with the smallest number of atoms in the supercell.

Lattice matching - minimal strain

Formula	N° atoms	strain	cell size 1	cell size 2
$HfSe_2$	30	0.0	9	4
Te_2Ti	30	0.0	9	4
$PdTe_2$	203	0.0	64	25
H_2MnO_2	278	0.0001	49	36
BiITe	146	0.0001	49	16
BrCdI	77	0.0001	25	9
Bi_2Te_3	178	0.0001	49	16
SSb_2Te_2	95	0.0002	25	9
$CrSe_2$	233	0.0002	61	37
$Cl_2H_2Zr_2$	86	0.0002	16	9
CdH_2O_2	353	0.0002	79	39
DyI_2	237	0.0002	81	25
NiO_2	354	0.0002	81	64
$I_2S_2Tb_2$	312	0.0002	81	25
Br_2Zn	30	0.0003	9	4
CoO_2	354	0.0003	81	64
CCl_2Gd_2	38	0.0004	9	4
NbS_2	59	0.0004	16	9
$MnNaTe_2$	86	0.0005	25	9
H_2Li_2Pt	172	0.0006	36	20
Te_2W	275	0.0006	79	39
Te_2V	347	0.0006	100	49
Br_2Tb_2	228	0.0006	64	25
CdClO	275	0.0006	79	39
$CrTe_2$	173	0.0007	49	25
$LiOS_2Ti$	77	0.0007	16	9
Cl_2Sc_2	198	0.0007	49	25
$MoTe_2$	275	0.0008	79	39
LiO	260	0.0008	81	49
OTl_2	275	0.0008	79	39
$Cl_2Hf_2N_2$	392	0.0008	79	39
Br_2Pr_2	34	0.0009	9	4
Br_2Cd	203	0.0009	64	25
Bi_2S_3	253	0.0009	64	25
$Dy_2I_2S_2$	312	0.0009	81	25
Br_2Mn	347	0.0009	100	49
NS_2Ta	172	0.0009	36	25
Br_2Cr	173	0.001	49	25
Te_2Zn	275	0.001	79	39
CBr_2Y_2	38	0.001	9	4
$Br_2H_2Zr_2$	248	0.0011	49	25
Br_2PY_2	253	0.0011	64	25
FeO_2	354	0.0011	81	64
$PbTe_2$	77	0.0011	25	9
Cl_2V	233	0.0011	61	37
GeI_3Rb	37	0.0011	16	1
Br_2Cu	281	0.0011	88	35
Br_2Ho_2	228	0.0011	64	25
Te_2V	275	0.0011	79	39
N_2Re	275	0.0012	64	49

Table showing the first 50 lattice matching combinations with the smallest strain needed to build the supercell.

$Ba_2I_2F_2$ (P4/nmm)

Structural and electronic properties

**Formula**	$Ba_2I_2F_2$
**Spacegroup**	P4/nmm
**Prototype**	PbClF
**Parent 3D**	$Ba_2F_2I_2$
**Source DB**	MPDS
**DB ID**	S1935141
**DF2-C09 Binding energy [meV/ Å^2]**	22.38
**RVV10 Binding energy [meV/ Å^2]**	N/A
**Band gap (PBE) [eV]**	4.29

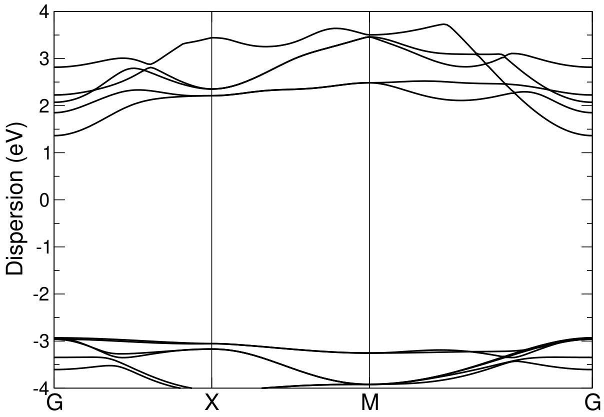

Band structure: Electronic band structure of $Ba_2I_2F_2$ (P4/nmm) in a representative window around the Fermi energy and along a high-symmetry path.

Optimized crystal structure

Structural parameters: relaxed cell (top) and atomic positions (bottom) of $Ba_2I_2F_2$ (P4/nmm) in Cartesian coordinates.

	x [Å]	y [Å]	z [Å]
$\mathbf{a_1}$	4.52199767	0.00000000	0.00000000
$\mathbf{a_2}$	0.00000000	4.52199767	0.00000000
$\mathbf{a_3}$	0.00000000	0.00000000	23.04910023

		x [Å]	y [Å]	z [Å]
●	Ba	1.13049942	-1.13049942	1.45161642
●	I	-1.13049942	-3.39149825	3.10075024
●	Ba	-1.13049942	-3.39149825	-1.45161642
●	I	1.13049942	-1.13049942	-3.10075024
●	F	-1.13049942	-1.13049942	0.00000000
●	F	1.13049942	-3.39149825	0.00000000

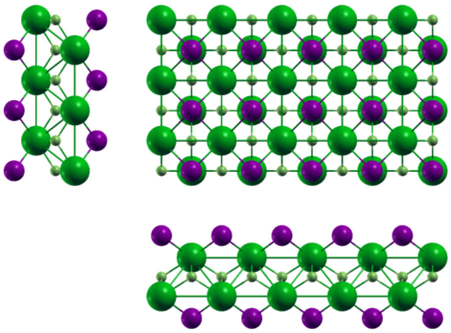

Orthographic projections: views of $Ba_2I_2F_2$ (P4/nmm) as seen from the x axis (left), the y axis (bottom) and the z axis (center).

Lattice matching - minimal size

Formula	N° atoms	strain	cell size 1	cell size 2
Bi_2	8	0.1089	1	1
Sm	8	0.1472	1	2
Br_2Zn	9	0.1092	1	1
Br_2Ca	9	0.1086	1	1
$AsSn_2$	9	0.1107	1	1
BiClTe	9	0.1084	1	1
HgI_2	9	0.0046	1	1
Te_2Ti	9	0.1091	1	1
$RhTe_2$	9	0.11	1	1
CNRb	9	0.0491	1	1
CKN	9	0.3608	1	1
Cl_2Cu	9	0.1397	1	1
$PtSe_2$	9	0.1105	1	1
CNNa	9	0.1985	1	1
I_2La	9	0.5938	1	1
F_2Zn	9	0.5652	1	1
I_2Pr	9	0.1084	1	1
$HfSe_2$	9	0.1091	1	1
Bi_2Te_2	10	0.136	1	1
Li_2Tl_2	10	0.1582	1	1
CdClHO	10	0.1109	1	1
Ag_2Br_2	10	0.5954	1	1
CdClHO	10	0.1102	1	1
S_2Sn_2	10	0.2256	1	1
Cl_2Y_2	10	0.1094	1	1
Ge_2Te_2	10	0.0234	1	1
Br_2Cu_2	10	0.5613	1	1
Ag_2	10	0.9798	1	2
As_2Ir_2	10	0.2074	1	1
O_2Pb_2	10	0.2185	1	1
Au_2I_2	10	0.0207	1	1
Ge_2Se_2	10	0.2138	1	1
Bi_2O_2	10	0.22	1	1
$AgClO_2$	10	0.1163	1	1
As_2Rh_2	10	0.5946	1	1
Ag_2I_2	10	0.0036	1	1
Ga_2Se_2	10	0.1096	1	1
Sn_2Te_2	10	0.0045	1	1
NaO_4	11	0.1686	1	1
$AgNO_3$	11	0.1695	1	1
ClH_3O	11	0.1335	1	1
Bi_2SeTe_2	11	0.109	1	1
$Ba_2H_2I_2$	12	0.0078	1	1
$Er_2I_2O_2$	12	0.5849	1	1
Ba_2Pt	12	0.9785	1	2
$Eu_2F_2I_2$	12	0.2194	1	1
$Cl_2F_2Pb_2$	12	0.2137	1	1
$F_2I_2Sm_2$	12	0.2183	1	1
$Ba_2Ge_2Mn_2$	12	0.2122	1	1
$Br_2Ce_2O_2$	12	0.2094	1	1

Table showing the first 50 lattice matching combinations with the smallest number of atoms in the supercell.

Lattice matching - minimal strain

Formula	N° atoms	strain	cell size 1	cell size 2
Bi_2O_2	896	0.0	82	101
$Ca_2Mn_2Si_2$	876	0.0	61	85
$Br_2Gd_2O_2$	510	0.0	36	49
$F_2I_2Tm_2$	882	0.0001	65	82
$Cu_2Na_2Se_2$	882	0.0001	65	82
$Bi_2I_2O_2$	882	0.0001	65	82
$Ca_2Cl_2H_2$	612	0.0001	41	61
As_2Rh_2	550	0.0001	49	64
$Cl_2F_2Pb_2$	882	0.0001	65	82
Ag_2Br_2	550	0.0001	49	64
Br_2Hf_2	276	0.0002	20	39
$Br_2Ca_2H_2$	876	0.0002	61	85
Hg_3N_2	675	0.0002	80	39
$Br_2Ho_2O_2$	876	0.0002	61	85
$Hf_2Se_2Si_2$	780	0.0002	49	81
As_2Fe_2	618	0.0002	49	81
$MnNaTe_2$	548	0.0003	48	65
F_2Zn	363	0.0003	36	49
Tl	394	0.0003	49	100
$Ge_2Te_2Zr_2$	366	0.0004	25	36
I_2La	486	0.0004	49	64
I_3Sn	896	0.0004	106	65
Cl_2Rb_2	796	0.0004	100	49
$PbTe_2$	483	0.0004	48	65
$Br_2O_2Pr_2$	690	0.0005	50	65
$AgTe_2$	891	0.0005	74	149
$Br_2Lu_2S_2$	582	0.0005	49	48
$Ba_2Ge_2Mn_2$	870	0.0005	64	81
BrNZr	237	0.0005	20	39
Bi_2O_2	886	0.0006	81	100
$Br_2Lu_2S_2$	582	0.0006	49	48
$Ge_2Mn_2Sr_2$	690	0.0006	50	65
O_4PTl	12	0.0006	1	1
$NbSe_2$	237	0.0006	20	39
SSb_2Te_2	613	0.0006	48	65
$Br_2Lu_2O_2$	366	0.0006	25	36
$Br_2O_2Pr_2$	678	0.0006	49	64
$I_2Lu_2O_2$	510	0.0006	36	49
BrCdI	483	0.0006	48	65
Mg_4	694	0.0006	49	100
$Br_2F_2Tm_2$	510	0.0007	36	49
$Br_2Ca_2F_2$	510	0.0007	36	49
$F_2I_2Tm_2$	870	0.0007	64	81
O_2Pb_2	886	0.0007	81	100
$Br_2O_2Ti_2$	402	0.0008	27	40
$F_2I_2Yb_2$	870	0.0008	64	81
$Cu_2Na_2Se_2$	870	0.0008	64	81
$AsSe_2$	237	0.0008	20	39
$Bi_2I_2O_2$	870	0.0008	64	81
$Ca_2Ge_2Mn_2$	510	0.0008	36	49

Table showing the first 50 lattice matching combinations with the smallest strain needed to build the supercell.

BaF_2 (P3m1)

Structural and electronic properties

**Formula**	BaF_2
**Spacegroup**	P3m1
**Prototype**	CdI2
**Parent 3D**	$C_4Ba_2Ce_2F_2O_{12}$
**Source DB**	MPDS
**DB ID**	S1813537
**DF2-C09 Binding energy [meV/ Å^2]**	28.8
**RVV10 Binding energy [meV/ Å^2]**	N/A
**Band gap (PBE) [eV]**	6.32

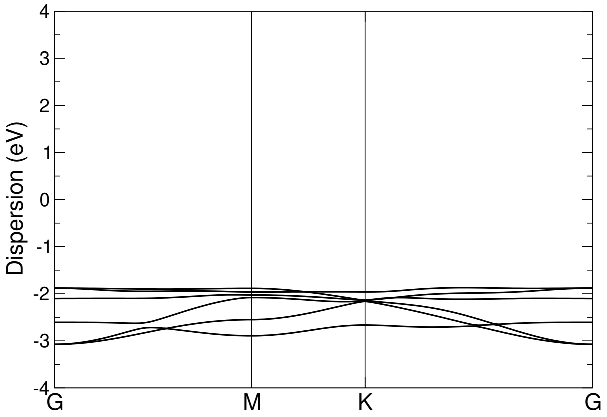

Band structure: Electronic band structure of BaF_2 (P3m1) in a representative window around the Fermi energy and along a high-symmetry path.

Optimized crystal structure

Structural parameters: relaxed cell (top) and atomic positions (bottom) of BaF_2 (P3m1) in Cartesian coordinates.

	x [Å]	y [Å]	z [Å]
$\mathbf{a_1}$	−2.10535136	−3.64657552	0.00000000
$\mathbf{a_2}$	4.21070272	0.00000000	0.00000000
$\mathbf{a_3}$	0.00000000	0.00000000	12.91899271

		x [Å]	y [Å]	z [Å]
●	Ba	-0.00000000	-2.43105035	0.00000745
•	F	2.10535136	-3.64657552	0.96987827
•	F	2.10535136	-1.21552517	-0.96988571

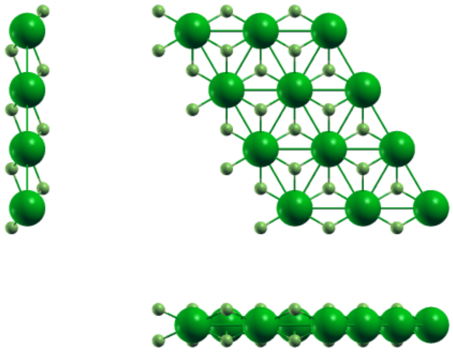

Orthographic projections: views of BaF_2 (P3m1) as seen from the x axis (left), the y axis (bottom) and the z axis (center).

Lattice matching - minimal size

Formula	N° atoms	strain	cell size 1	cell size 2
InSe	5	0.0039	1	1
AgTl	5	0.1563	1	1
Ag_2	5	0.4489	1	1
PbTe	5	0.0057	1	1
CaCl	5	0.115	1	1
I_2Mg	6	0.0064	1	1
CdI_2	6	0.0074	1	1
PSn_2	6	0.2481	1	1
Ba_2Pt	6	0.4482	1	1
Br_2Zn	6	0.2626	1	1
Br_2Ca	6	0.0087	1	1
$SiTe_2$	6	0.2685	1	1
I_2Pr	6	0.1396	1	1
S_2Zr	6	0.2471	1	1
Br_2La	6	0.006	1	1
Ca_2Si	6	0.4593	1	1
PbS_2	6	0.2736	1	1
BiClTe	6	0.0078	1	1
BrCdI	6	0.0033	1	1
Cl_2Zn	6	0.1224	1	1
Te_2Ti	6	0.263	1	1
$RhTe_2$	6	0.2569	1	1
GeI_2	6	0.0082	1	1
AsKSn	6	0.0014	1	1
$PbTe_2$	6	0.0043	1	1
I_2Nd	6	0.1404	1	1
$NiTe_2$	6	0.2676	1	1
Cl_2Cu	6	0.0664	1	1
S_2Sn	6	0.2474	1	1
I_2V	6	0.2704	1	1
GeI_2	6	0.0059	1	1
Se_2Zr	6	0.269	1	1
I_2Pb	6	0.4527	1	1
STl_2	6	0.001	1	1
$PtSe_2$	6	0.254	1	1
$MnSe_2$	6	0.115	1	1
CeI_2	6	0.139	1	1
$NbTe_2$	6	0.2468	1	1
In	6	0.6207	1	3
Se_2Yb	6	0.0079	1	1
$BiTe_2$	6	0.0077	1	1
F_2Ni	6	0.1201	1	1
I_2La	6	0.1446	1	1
F_2Na	6	0.2654	1	1
CdI_2	6	0.007	1	1
F_2Zn	6	0.1364	1	1
I_2Pr	6	0.0079	1	1
$HfSe_2$	6	0.263	1	1
Fe_2Te_2	7	0.1298	1	1
Ca_2Cl_2	7	0.1301	1	1

Table showing the first 50 lattice matching combinations with the smallest number of atoms in the supercell.

Lattice matching - minimal strain

Formula	N° atoms	strain	cell size 1	cell size 2
$NiTe_2$	543	0.0	81	100
Br_2Pr_2	583	0.0001	73	91
As_2	206	0.0001	36	49
$CrTe_2$	183	0.0001	25	36
ClH_3O	504	0.0001	73	57
Li_2Tl_2	208	0.0002	36	25
Cl_2Sc_2	219	0.0002	25	36
CCl_2Lu_2	353	0.0002	36	49
CNb_2S_2	416	0.0002	37	61
$Ga_2I_2Y_2$	9	0.0002	1	1
CdClHO	463	0.0003	57	73
$Br_2N_2Zr_2$	471	0.0003	43	57
In	43	0.0003	9	16
NS_2Ta	271	0.0004	25	49
$F_2Se_2Y_2$	429	0.0004	57	43
Ga_2Se_2	516	0.0004	64	81
CBr_2Lu_2	414	0.0004	43	57
Br_2Cr	183	0.0004	25	36
$BrKO_3$	120	0.0004	25	9
Br_2Fe	255	0.0004	36	49
Ca_2N	255	0.0005	36	49
Cl_2NSc_2	173	0.0005	16	25
Br_2Co	255	0.0005	36	49
$Br_2H_2Zr_2$	291	0.0005	25	36
Ge_2S_2	286	0.0006	42	40
$SiTe_2$	543	0.0006	81	100
CaClHO	643	0.0006	81	100
$I_2N_2Zr_2$	678	0.0006	64	81
NbS_2	123	0.0007	16	25
$Cl_2O_2Yb_2$	765	0.0007	73	91
Br_2Ti	183	0.0007	25	36
$Br_2Hf_2N_2$	675	0.0007	73	76
P_2	233	0.0007	37	61
HgO	479	0.0007	81	118
$Ce_2I_2S_2$	786	0.0007	100	81
F_2Na	492	0.0007	73	91
Ca_2Si	543	0.0007	100	81
$Cl_2O_2Tm_2$	843	0.0007	81	100
$Cu_2K_2Te_2$	483	0.0008	65	48
$Cl_2Er_2O_2$	843	0.0008	81	100
Cl_2Ti	294	0.0009	37	61
PSn_2	339	0.0009	49	64
I_4Sr_2	435	0.0009	81	32
$Cl_2Hg_2N_2$	561	0.0009	91	48
FKO_2Se	495	0.0009	85	48
$HfSe_2$	492	0.0009	73	91
Te_2Ti	492	0.0009	73	91
CS_2Ta_2	416	0.0009	37	61
STl_2	6	0.001	1	1
Cl_2Mn	123	0.001	16	25

Table showing the first 50 lattice matching combinations with the smallest strain needed to build the supercell.

BaHI (P4/nmm)

Structural and electronic properties

**Formula**	BaHI
**Spacegroup**	P4/nmm
**Prototype**	PbClF
**Parent 3D**	$Ba_2H_2I_2$
**Source DB**	COD
**DB ID**	9009160
**DF2-C09 Binding energy [meV/ Å^2]**	24.3
**RVV10 Binding energy [meV/ Å^2]**	28.7
**Band gap (PBE) [eV]**	3.36

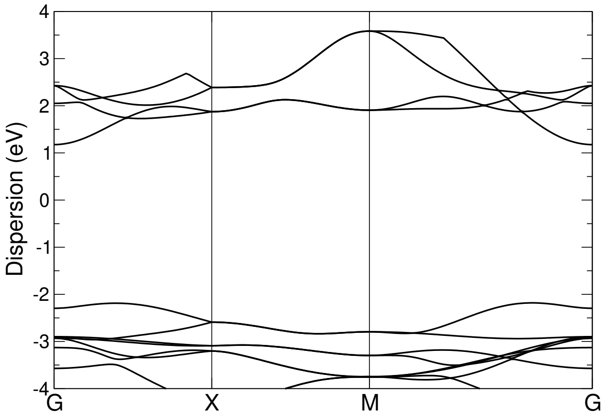

Band structure: Electronic band structure of BaHI (P4/nmm) in a representative window around the Fermi energy and along a high-symmetry path.

Optimized crystal structure

Structural parameters: relaxed cell (top) and atomic positions (bottom) of BaHI (P4/nmm) in Cartesian coordinates.

	x [Å]	y [Å]	z [Å]
$\mathbf{a_1}$	4.59618322	0.00000000	0.00000000
$\mathbf{a_2}$	0.00000000	4.59618322	0.00000000
$\mathbf{a_3}$	0.00000000	0.00000000	25.36392350

		x [Å]	y [Å]	z [Å]
	Ba	0.00000000	2.29809161	14.07856504
	I	2.29809161	0.00000000	15.57236807
	Ba	2.29809161	0.00000000	11.28535846
	H	0.00000000	0.00000000	12.68196175
	H	2.29809161	2.29809161	12.68196175
	I	0.00000000	2.29809161	9.79155543

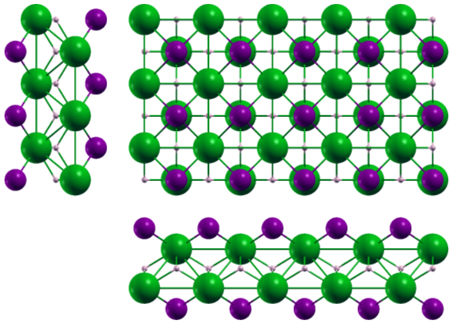

Orthographic projections: views of BaHI (P4/nmm) as seen from the x axis (left), the y axis (bottom) and the z axis (center).

Lattice matching - minimal size

Formula	N° atoms	strain	cell size 1	cell size 2
AsSb	8	0.1087	1	1
Br_2Zn	9	0.1109	1	1
$SiTe_2$	9	0.1099	1	1
Br_2Cu	9	0.1004	1	1
Te_2Ti	9	0.1108	1	1
CNRb	9	0.0431	1	1
CKN	9	0.0504	1	1
$NiTe_2$	9	0.1101	1	1
I_2V	9	0.1096	1	1
Se_2Zr	9	0.1098	1	1
GdI_2	9	0.1086	1	1
$HfSe_2$	9	0.1108	1	1
Bi_2Te_2	10	0.1302	1	1
Li_2Tl_2	10	0.1505	1	1
Bi_2In_2	10	0.0039	1	1
Cu_2I_2	10	0.2136	1	1
S_2Sn_2	10	0.2165	1	1
Cl_2Y_2	10	0.1111	1	1
N_3Na	10	0.0241	1	1
O_2Pb_2	10	0.2099	1	1
Ga_2Se_2	10	0.1093	1	1
CaClHO	10	0.1102	1	1
Au_2I_2	10	0.0257	1	1
Ag_2Te_2	10	0.5411	1	1
Bi_2O_2	10	0.2113	1	1
PbS_2Sn	10	0.2263	1	1
Br_2CsF	10	0.0019	1	1
Ga_2Se_2	10	0.1113	1	1
Sn_2Te_2	10	0.0041	1	1
O_2Sn_2	10	0.5124	1	1
As_2O_3	11	0.189	1	1
PTe_2Zr_2	11	0.1088	1	1
F_4Nb	11	0.2178	1	1
NaO_4	11	0.1602	1	1
$AgNO_3$	11	0.161	1	1
$Br_2Ho_2S_2$	12	0.065	1	1
$Er_2I_2O_2$	12	0.5624	1	1
$Br_2F_2Sr_2$	12	0.2158	1	1
$Ho_2I_2S_2$	12	0.3738	1	1
$Eu_2F_2I_2$	12	0.2108	1	1
$Cl_2F_2Pb_2$	12	0.5969	1	1
$F_2I_2Sm_2$	12	0.2097	1	1
$Ba_2Ge_2Mn_2$	12	0.5926	1	1
$Br_2Ce_2O_2$	12	0.5851	1	1
$Cl_2O_2Y_2$	12	0.1094	1	1
$Br_2S_2Y_2$	12	0.0699	1	1
$Eu_2I_2O_2$	12	0.5866	1	1
$Gd_2I_2S_2$	12	0.3802	1	1
$Br_2F_2Pb_2$	12	0.2155	1	1
$Cu_2Na_2Se_2$	12	0.5967	1	1

Table showing the first 50 lattice matching combinations with the smallest number of atoms in the supercell.

Lattice matching - minimal strain

Formula	N° atoms	strain	cell size 1	cell size 2
Cu_2I_2	718	0.0	65	82
$MnSe_2$	708	0.0	65	106
$I_2O_2Y_2$	510	0.0	36	49
Fe_2Se_2	196	0.0001	16	25
$Ca_2Mn_2Si_2$	366	0.0001	25	36
CaCl	602	0.0001	65	106
$Br_2Ca_2H_2$	366	0.0001	25	36
$Cu_2Se_2Tl_2$	870	0.0001	64	81
$I_2La_2Si_2$	678	0.0002	48	65
Se_2Ti	237	0.0002	20	39
As_2Li_2Pr	613	0.0002	48	65
InSe	418	0.0002	48	65
$Br_2Nd_2O_2$	510	0.0002	36	49
S_2V	714	0.0003	54	130
Cl_2ORu	348	0.0003	28	45
$Br_2Ho_2O_2$	366	0.0003	25	36
Mg_6	972	0.0004	53	109
$Br_2H_2Sr_2$	882	0.0004	65	82
$I_2Nd_2O_2$	690	0.0004	50	65
$AsLi_3$	548	0.0004	48	65
$Cl_2F_2Pb_2$	690	0.0005	50	65
$F_2I_2Tm_2$	678	0.0005	49	64
$Si_2Te_2Zr_2$	246	0.0005	16	25
F_2Ni	171	0.0005	16	25
Br_2Zr_2	276	0.0005	20	39
Ni_2Se_2	706	0.0005	61	85
$Bi_2I_2O_2$	690	0.0005	50	65
$Cu_2Na_2Se_2$	690	0.0005	50	65
MoS_2	714	0.0006	54	130
$Cu_2Na_2Se_2$	678	0.0006	49	64
$Bi_2I_2O_2$	678	0.0006	49	64
As_2Li_2Nd	613	0.0006	48	65
$Se_2Si_2Zr_2$	930	0.0006	58	97
S_2W	714	0.0006	54	130
H_2Li_2Pd	69	0.0006	4	9
$Cl_2F_2Pb_2$	678	0.0006	49	64
$Bi_2Cl_2O_2$	876	0.0007	61	85
CeI_2	621	0.0007	61	85
Cu_2I_2	708	0.0008	64	81
MoS_2	714	0.0008	54	130
Co_2S_2	814	0.0008	65	106
Cl_2Zn	942	0.0008	89	136
$Ba_2Ge_2Mn_2$	678	0.0008	49	64
$I_2S_2Tl_2$	246	0.0008	16	25
Cl_4KTl	894	0.0008	100	49
GeI_2Y_2	613	0.0008	48	65
$Br_2Eu_2O_2$	876	0.0008	61	85
$Bi_2Br_2O_2$	510	0.0009	36	49
$Br_2Er_2O_2$	366	0.001	25	36
HgO	742	0.001	74	149

Table showing the first 50 lattice matching combinations with the smallest strain needed to build the supercell.

Bi (P-3m1)

Structural and electronic properties

**Formula**	Bi
**Spacegroup**	P-3m1
**Prototype**	As
**Parent 3D**	$Bi_4Te_2I_2$
**Source DB**	ICSD
**DB ID**	153858
**DF2-C09 Binding energy [meV/ Å^2]**	17.92
**RVV10 Binding energy [meV/ Å^2]**	24.92
**Band gap (PBE) [eV]**	0.57

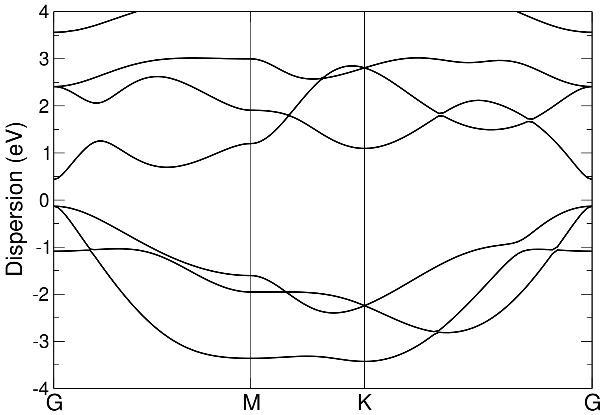

Band structure: Electronic band structure of Bi (P-3m1) in a representative window around the Fermi energy and along a high-symmetry path.

Optimized crystal structure

Structural parameters: relaxed cell (top) and atomic positions (bottom) of Bi (P-3m1) in Cartesian coordinates.

		x [Å]	y [Å]	z [Å]
$\mathbf{a_1}$		4.29744687	0.00000000	0.00000000
$\mathbf{a_2}$		−2.14872343	3.72169816	0.00000000
$\mathbf{a_3}$		0.00000000	0.00000000	21.66627696
		x [Å]	y [Å]	z [Å]
●	Bi	2.14872343	1.24056605	9.95994369
●	Bi	0.00000000	2.48113211	11.70633327

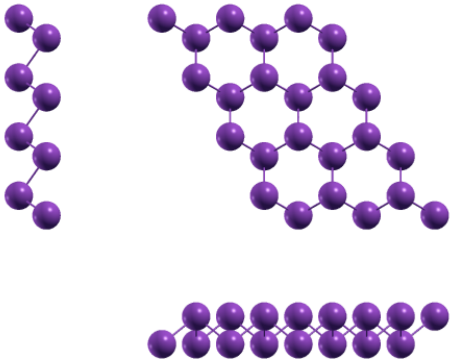

Orthographic projections: views of Bi (P-3m1) as seen from the x axis (left), the y axis (bottom) and the z axis (center).

Lattice matching - minimal size

Formula	N° atoms	strain	cell size 1	cell size 2
InSe	4	0.0057	1	1
AsSb	4	0.2634	1	1
GeTe	4	0.2721	1	1
AgTl	4	0.147	1	1
S_2	4	0.2744	1	1
PbTe	4	0.004	1	1
CaCl	4	0.1113	1	1
$IrTe_2$	5	0.2735	1	1
$CdCl_2$	5	0.2706	1	1
CdI_2	5	0.0023	1	1
PSn_2	5	1.5413	1	1
Br_2Zn	5	0.2494	1	1
Br_2Ca	5	0.0011	1	1
$InSe_2$	5	0.2711	1	1
$GeTe_2$	5	0.2691	1	1
$SiTe_2$	5	0.255	1	1
I_2Pr	5	0.132	1	1
I_2Mn	5	0.2707	1	1
S_2Zr	5	1.5358	1	1
NSr_2	5	0.2657	1	1
PbS_2	5	0.2598	1	1
BiClTe	5	0.0019	1	1
Cl_2Zn	5	0.1172	1	1
FeI_2	5	0.2675	1	1
I_2Ni	5	0.2694	1	1
Te_2Ti	5	0.2498	1	1
CrI_2	5	0.2669	1	1
BiBrTe	5	0.0036	1	1
$RhTe_2$	5	1.5892	1	1
S_2Ta	5	4.8574	1	1
I_2Nd	5	0.1327	1	1
$NiTe_2$	5	0.2541	1	1
Cl_2Cu	5	0.0694	1	1
S_2Sn	5	1.5377	1	1
I_2V	5	0.2568	1	1
GeI_2	5	0.0038	1	1
Se_2Zr	5	0.2555	1	1
STl_2	5	0.0085	1	1
CoI_2	5	0.264	1	1
$MnSe_2$	5	0.1113	1	1
CeI_2	5	0.1314	1	1
Br_2Mg	5	0.2673	1	1
I_2Ti	5	0.2645	1	1
$NbTe_2$	5	1.5343	1	1
GdI_2	5	0.0065	1	1
F_2Ni	5	0.1154	1	1
I_2La	5	0.1364	1	1
CdI_2	5	0.0027	1	1
Se_2Sn	5	0.2654	1	1
F_2Zn	5	0.1292	1	1

Table showing the first 50 lattice matching combinations with the smallest number of atoms in the supercell.

Lattice matching - minimal strain

Formula	N° atoms	strain	cell size 1	cell size 2
N_2W	315	0.0	39	79
$CrSe_2$	66	0.0	9	16
NbS_2	341	0.0	49	81
C	33	0.0	4	25
CCl_2Gd_2	418	0.0	49	64
FeI_2	462	0.0	81	100
Se_2Zr	333	0.0	57	73
Bi_2Te_2	474	0.0001	91	73
Br_2Y_2	452	0.0001	64	81
I_2Ti	419	0.0001	73	91
$Cl_2Er_2O_2$	552	0.0001	57	73
Br_2Zn	290	0.0001	49	64
Te_2V	158	0.0002	25	36
$Br_2H_2Zr_2$	182	0.0002	16	25
Br_2Mg	462	0.0002	81	100
H_2Li_2Pt	498	0.0002	49	80
CoI_2	419	0.0002	73	91
NaO_4	197	0.0002	36	25
Ga_2Se_2	452	0.0002	64	81
$Cl_2H_2Zr_2$	584	0.0003	49	81
PbS_2	371	0.0003	64	81
$Cl_2H_2Zr_2$	440	0.0003	37	61
$SiTe_2$	333	0.0004	57	73
ClNZr	107	0.0004	16	25
CrI_2	462	0.0004	81	100
$Ga_2Gd_2I_2$	8	0.0004	1	1
$RhTe_2$	257	0.0004	43	57
Te_2Ti	290	0.0004	49	64
NS_2Ta	498	0.0005	49	100
$Cl_2Hf_2N_2$	266	0.0005	25	36
$HfSe_2$	290	0.0005	49	64
OTl_2	158	0.0005	25	36
CCl_2Sc_2	157	0.0005	16	25
CBr_2Y_2	418	0.0005	49	64
$LiOS_2Ti$	379	0.0005	37	61
CI_2Y_2	601	0.0006	73	91
$AgNO_3$	197	0.0006	36	25
Bi_2SeTe_2	7	0.0006	1	1
In_2Se_2	274	0.0006	57	40
NbS_2	257	0.0006	37	61
CdClO	158	0.0006	25	36
AsSb	328	0.0007	73	91
Bi_2Pd	516	0.0007	81	118
$I_2N_2Zr_2$	428	0.0007	43	57
$Ga_2I_2Tb_2$	8	0.0007	1	1
N_2Re	35	0.0007	4	9
Se_2Sn	419	0.0007	73	91
$Cl_2O_2Y_2$	614	0.0008	64	81
PSn_2	219	0.0008	36	49
Cl_4Cu_2	584	0.0008	130	54

Table showing the first 50 lattice matching combinations with the smallest strain needed to build the supercell.

Bi_2Se_2 (C2/m)

Structural and electronic properties

**Formula**	Bi_2Se_2
**Spacegroup**	C2/m
**Prototype**	FeSe
**Parent 3D**	Bi_2Se_2
**Source DB**	COD
**DB ID**	4342500
**DF2-C09 Binding energy [meV/ Å^2]**	0.18
**RVV10 Binding energy [meV/ Å^2]**	N/A
**Band gap (PBE) [eV]**	0.0

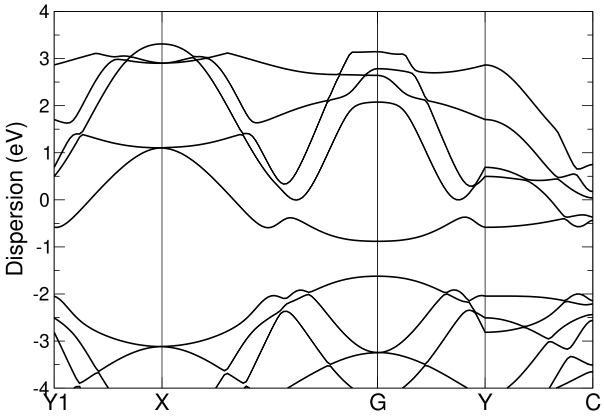

Band structure: Electronic band structure of Bi_2Se_2 (C2/m) in a representative window around the Fermi energy and along a high-symmetry path.

Optimized crystal structure

Structural parameters: relaxed cell (top) and atomic positions (bottom) of Bi_2Se_2 (C2/m) in Cartesian coordinates.

		x [Å]	y [Å]	z [Å]
$\mathbf{a_1}$		2.98529667	−2.98175713	0.00000000
$\mathbf{a_2}$		2.98529667	2.98175713	0.00000000
$\mathbf{a_3}$		0.00000000	0.00000000	18.79750304
		x [Å]	y [Å]	z [Å]
●	Bi	-4.45870062	2.98175713	-1.66730948
●	Bi	-1.51189271	2.98175713	1.66730948
●	Se	-1.52837594	2.98175713	-1.28768947
●	Se	-4.44221740	2.98175713	1.28768947

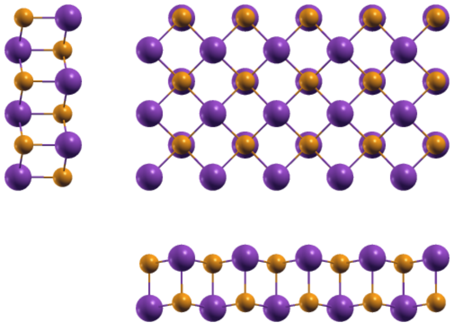

Orthographic projections: views of Bi_2Se_2 (C2/m) as seen from the x axis (left), the y axis (bottom) and the z axis (center).

Lattice matching - minimal size

Formula	N° atoms	strain	cell size 1	cell size 2
Tl	5	0.2695	1	1
Gd	5	0.3013	1	1
Tl	6	0.1565	1	2
Sn	6	0.0047	1	2
Na	6	0.1932	1	2
In	6	0.0004	1	2
In	6	0.1353	1	2
Cl_2Mn	7	0.2775	1	1
S_2Ta	7	0.2804	1	1
NbS_2	7	0.2798	1	1
NbS_2	7	0.2737	1	1
S_2Ta	7	0.2725	1	1
Se_2V	7	0.2707	1	1
Bi_2Mn_2	8	0.1603	1	1
$AgNO_2$	8	1.1668	1	1
Cu_2O_2	8	0.2325	1	1
Au_2Se_2	8	0.3844	1	1
LiO	8	0.1442	1	2
P_2	8	0.1513	1	2
O_2Sn_2	8	0.1481	1	1
Ge_2Se_2	8	0.0224	1	1
$AgClO_2$	8	0.3649	1	1
$LiNbS_2$	8	0.2805	1	1
O_2Sn_2	8	0.1482	1	1
H_2Li_2Pt	9	0.146	1	1
Cl_2NSc_2	9	0.2781	1	1
$LiOS_2Ti$	9	0.2753	1	1
$CuGeO_3$	9	0.2437	1	1
CrS_2	10	0.2359	1	2
S_2V	10	0.1379	1	2
MoS_2	10	0.1385	1	2
Cl_2Mn	10	0.1626	1	2
$MoSe_2$	10	0.1558	1	2
$Cl_2O_2V_2$	10	0.1611	1	1
$ReSe_2$	10	0.1775	1	2
$C_2Br_2Y_2$	10	0.1185	1	1
S_2Ta	10	0.1648	1	2
Br_2Zn	10	0.4104	1	2
FeO_2	10	1.0922	1	2
$CuTe_2$	10	0.3717	1	2
Cr_2O_4	10	0.1476	1	1
CrO_2	10	1.0332	1	2
Cl_2Ti	10	0.1514	1	2
LiO_2	10	0.3765	1	2
S_2Ti	10	0.1704	1	2
Te_2Ti	10	0.4111	1	2
NbS_2	10	0.1643	1	2
S_2W	10	0.1385	1	2
Bi_2Pd	10	0.3807	1	2
Cl_2Ni	10	0.1786	1	2

Table showing the first 50 lattice matching combinations with the smallest number of atoms in the supercell.

Lattice matching - minimal strain

Formula	N° atoms	strain	cell size 1	cell size 2
I_2Tm	276	0.0001	39	40
As_2Ir_2	68	0.0003	8	9
K	441	0.0003	89	85
Bi_2Pd	896	0.0003	101	164
Br_2CsF	852	0.0003	116	97
F_4Sn	446	0.0003	49	50
$Br_2O_2Tm_2$	602	0.0003	53	65
GeI_2La_2	356	0.0003	39	40
F_4Nb	785	0.0003	85	89
HgI_2	60	0.0004	9	8
Cu_2F_4	998	0.0004	128	81
$Ag_2K_2Te_2$	278	0.0004	32	25
$As_2Mg_2Na_2$	866	0.0004	89	85
Au_2Br_2	396	0.0004	50	49
$Cl_2S_2Tl_2$	208	0.0004	25	18
In	6	0.0004	1	2
As_2Ru_2	472	0.0005	53	65
Cu_2Te_2	228	0.0005	25	32
Cu_2Se_2	628	0.0005	68	89
$Br_2Ho_2O_2$	912	0.0005	81	98
$Fe_2Li_2P_2$	874	0.0005	73	97
Bi_2Pd	443	0.0006	50	81
Br_2Cu_2	852	0.0006	97	116
Cu_2F_4	506	0.0006	65	41
Ca_2Cl_2	472	0.0006	53	65
Fe_2S_2	172	0.0006	18	25
$Ca_2Mn_2Si_2$	912	0.0007	81	98
$I_2S_2Tl_2$	806	0.0007	68	89
Bi_2In_2	716	0.0007	98	81
Sb_2Te_3	620	0.0007	65	72
Bi_2SeTe_2	620	0.0007	65	72
Ba_2Hg	346	0.0007	49	50
P_2Rh_2	716	0.0008	81	98
$Er_2I_2S_2$	988	0.0008	112	90
O_2Sn_2	852	0.0008	97	116
$As_2Co_2Li_2$	292	0.0009	25	32
$Br_2Ho_2S_2$	912	0.0009	102	84
$Br_2Ca_2H_2$	912	0.0009	81	98
Ca_2O_2	524	0.0009	50	81
$Ge_2Mn_2Sr_2$	86	0.0009	8	9
F_2Tl_2	716	0.0009	81	98
Br_2V	101	0.0009	11	19
$F_2Lu_2Se_2$	934	0.0009	82	101
$Hf_2Si_2Te_2$	874	0.0009	73	97
$Cl_2Er_2S_2$	290	0.001	32	27
Br_2Ca_3Si	496	0.001	49	50
$F_2Lu_2Se_2$	796	0.001	70	86
F_2Ni	539	0.001	68	89
$Si_2Te_2Zr_2$	806	0.001	68	89
Fe_2Te_2	472	0.001	53	65

Table showing the first 50 lattice matching combinations with the smallest strain needed to build the supercell.

Bi_2Se_3 (P-3m1)

Structural and electronic properties

**Formula**	Bi_2Se_3
**Spacegroup**	P-3m1
**Prototype**	Bi2Te3
**Parent 3D**	Bi_2Se_3
**Source DB**	COD
**DB ID**	9011965
**DF2-C09 Binding energy [meV/ Å^2]**	19.6
**RVV10 Binding energy [meV/ Å^2]**	26.58
**Band gap (PBE) [eV]**	0.95

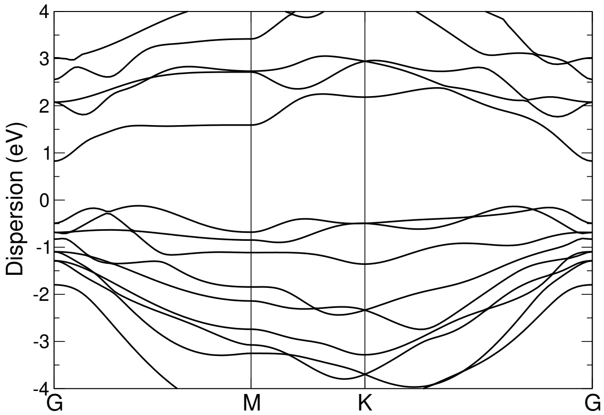

Band structure: Electronic band structure of Bi_2Se_3 (P-3m1) in a representative window around the Fermi energy and along a high-symmetry path.

Optimized crystal structure

Structural parameters: relaxed cell (top) and atomic positions (bottom) of Bi_2Se_3 (P-3m1) in Cartesian coordinates.

		x [Å]	y [Å]	z [Å]
$\mathbf{a_1}$		4.15711472	0.00000000	0.00000000
$\mathbf{a_2}$		−2.07855736	3.60016695	0.00000000
$\mathbf{a_3}$		0.00000000	0.00000000	27.02816386
		x [Å]	y [Å]	z [Å]
●	Bi	-0.00000000	2.40011130	11.54799129
●	Se	-0.00000000	2.40011130	17.06203452
●	Bi	2.07855736	3.60016695	15.48017257
●	Se	2.07855736	1.20005565	13.51408193
●	Se	2.07855736	3.60016695	9.96612934

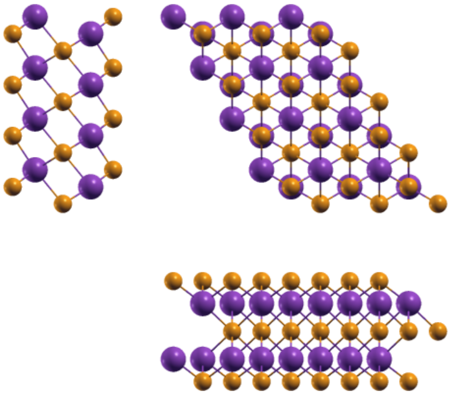

Orthographic projections: views of Bi_2Se_3 (P-3m1) as seen from the x axis (left), the y axis (bottom) and the z axis (center).

Lattice matching - minimal size

Formula	N° atoms	strain	cell size 1	cell size 2
K	6	0.4503	1	1
Na	6	1.5328	1	1
AgTl	7	0.1626	1	1
Ag_2	7	0.4638	1	1
As_2	7	1.5904	1	1
Sb_2	7	0.006	1	1
CaCl	7	0.1178	1	1
Cl_2Zn	8	1.5827	1	1
I_2Mg	8	0.0004	1	1
$MoTe_2$	8	1.549	1	1
PSn_2	8	0.2562	1	1
Ba_2Pt	8	0.4632	1	1
$ReSe_2$	8	1.4467	1	1
Br_2Zn	8	0.2713	1	1
HfS_2	8	0.2479	1	1
$AsSn_2$	8	0.2614	1	1
Te_2V	8	1.5629	1	1
I_2Pr	8	0.1449	1	1
$CuTe_2$	8	0.2474	1	1
S_2Zr	8	0.2551	1	1
Br_2La	8	0.0001	1	1
Ca_2Si	8	0.4746	1	1
Br_2Co	8	1.5873	1	1
Ca_2N	8	1.5943	1	1
BrCdI	8	0.0027	1	1
Cl_2Zn	8	0.1262	1	1
Te_2Ti	8	0.2717	1	1
I_2Zn	8	0.0092	1	1
BaF_2	8	0.0061	1	1
$RhTe_2$	8	0.2654	1	1
GeI_2	8	0.0023	1	1
PtS_2	8	1.5401	1	1
$CoTe_2$	8	0.2483	1	1
CdClO	8	1.5593	1	1
Se_2Ti	8	1.5213	1	1
AsKSn	8	0.0046	1	1
Te_2W	8	1.5501	1	1
$PbTe_2$	8	0.0018	1	1
I_2Nd	8	0.1457	1	1
S_2Sn	8	0.2555	1	1
$SnTe_2$	8	0.0045	1	1
Sn	8	0.6261	1	3
I_2Pb	8	0.4678	1	1
STl_2	8	0.0071	1	1
$PtSe_2$	8	0.2623	1	1
OTl_2	8	1.5607	1	1
Br_2Fe	8	1.5878	1	1
GeS_2	8	0.1126	1	1
$TaTe_2$	8	0.2606	1	1
$MnSe_2$	8	0.1178	1	1

Table showing the first 50 lattice matching combinations with the smallest number of atoms in the supercell.

Lattice matching - minimal strain

Formula	N° atoms	strain	cell size 1	cell size 2
S_2Sn	504	0.0	57	73
CdClHO	729	0.0	73	91
Br_2La	8	0.0001	1	1
Te_2Zn	327	0.0001	36	49
$HfLiS_2$	376	0.0002	36	49
$Br_2N_2Ti_2$	857	0.0002	73	82
$F_2Se_2Y_2$	461	0.0002	49	36
Cl_2Ni	233	0.0002	25	36
Se_2V	155	0.0002	16	25
Gd_2I_2	9	0.0002	1	1
LiO	407	0.0002	49	81
S_2Zr	504	0.0002	57	73
$NbSe_2$	233	0.0002	25	36
Au_2Se_2	475	0.0003	63	40
As_2	329	0.0003	43	57
Ca_2N	386	0.0003	43	57
$MoTe_2$	327	0.0003	36	49
Se_2Ta	233	0.0003	25	36
Cl_2OV	111	0.0003	11	14
I_2Pb	674	0.0003	91	73
CdClHO	644	0.0004	64	81
$Hf_2I_2N_2$	806	0.0004	64	81
CCl_2Lu_2	500	0.0004	43	57
I_2Mg	8	0.0004	1	1
Te_2W	327	0.0004	36	49
$HNiO_2$	321	0.0005	25	49
$NbTe_2$	504	0.0005	57	73
N_3W_2	745	0.0005	49	100
Ga_2S_2	501	0.0005	49	64
$I_2Pr_2S_2$	893	0.0005	91	73
PSn_2	504	0.0005	57	73
Cu_2I_2	9	0.0005	1	1
$Ce_2I_2S_2$	789	0.0005	81	64
Br_2Gd_2Ge	10	0.0005	1	1
Ca_2Si	597	0.0005	81	64
Ga_2S_2	501	0.0006	49	64
Ga_2Se_2	805	0.0006	81	100
Br_2Fe	386	0.0006	43	57
$Ce_2I_2Si_2$	11	0.0006	1	1
Tl	105	0.0007	16	25
C_2Li_2	356	0.0007	40	39
Br_2Co	386	0.0007	43	57
$CoTe_2$	437	0.0007	49	64
$Br_2Hf_2N_2$	534	0.0007	48	49
$RhTe_2$	638	0.0007	73	91
Ba_2Pt	743	0.0008	100	81
PTe_2Ti_2	565	0.0008	49	64
LiO	307	0.0009	37	61
CdH_2O_2	425	0.0009	36	49
$Br_2Hf_2N_2$	557	0.0009	43	57

Table showing the first 50 lattice matching combinations with the smallest strain needed to build the supercell.

Bi_2Te_2S (P-3m1)

Structural and electronic properties

**Formula**	Bi_2Te_2S
**Spacegroup**	P-3m1
**Prototype**	Bi2Te2S
**Parent 3D**	Bi_2Te_2S
**Source DB**	COD
**DB ID**	9008043
**DF2-C09 Binding energy [meV/ Å^2]**	20.94
**RVV10 Binding energy [meV/ Å^2]**	26.56
**Band gap (PBE) [eV]**	1.0

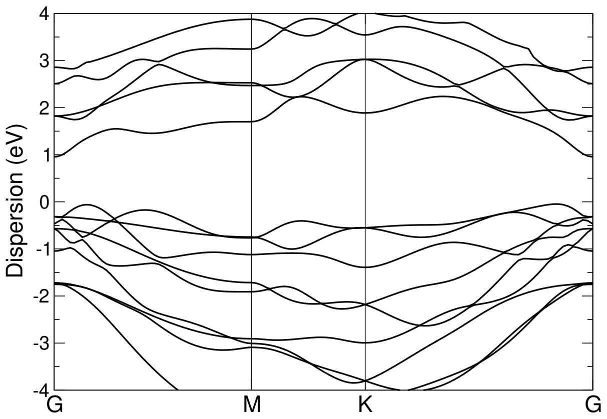

Band structure: Electronic band structure of Bi_2Te_2S (P-3m1) in a representative window around the Fermi energy and along a high-symmetry path.

Optimized crystal structure

Structural parameters: relaxed cell (top) and atomic positions (bottom) of Bi_2Te_2S (P-3m1) in Cartesian coordinates.

	x [Å]	y [Å]	z [Å]
$\mathbf{a_1}$	4.25355858	0.00000000	0.00000000
$\mathbf{a_2}$	−2.12677929	3.68368979	0.00000000
$\mathbf{a_3}$	0.00000000	0.00000000	27.13717753

		x [Å]	y [Å]	z [Å]
	Bi	2.12677929	1.22789660	15.34637259
	Te	2.12677929	1.22789660	9.98709663
	Bi	0.00000000	0.00000000	11.79080494
	Te	0.00000000	0.00000000	17.15008091
	S	-0.00000000	2.45579319	13.56858877

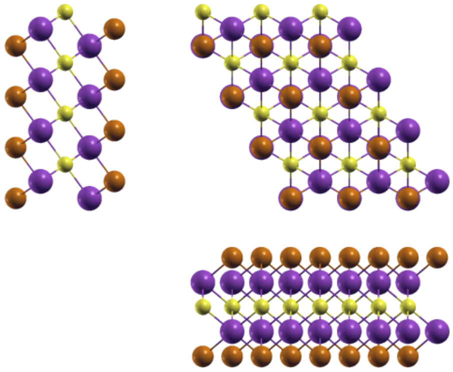

Orthographic projections: views of Bi_2Te_2S (P-3m1) as seen from the x axis (left), the y axis (bottom) and the z axis (center).

Lattice matching - minimal size

Formula	N° atoms	strain	cell size 1	cell size 2
InSe	7	0.0009	1	1
AsSb	7	0.2704	1	1
Bi_2	7	0.0049	1	1
AgTl	7	0.1516	1	1
PbTe	7	0.0008	1	1
CaCl	7	0.1131	1	1
CdI_2	8	0.0026	1	1
Br_2Zn	8	0.2559	1	1
Br_2Ca	8	0.0038	1	1
HfS_2	8	1.5318	1	1
$AsSn_2$	8	0.2468	1	1
$SiTe_2$	8	0.2616	1	1
I_2Pr	8	0.1357	1	1
Br_2Cu	8	0.7148	1	1
NSr_2	8	0.2727	1	1
Ca_2Si	8	0.4476	1	1
PbS_2	8	0.2666	1	1
BiClTe	8	0.0029	1	1
BrCdI	8	0.0079	1	1
Cl_2Zn	8	0.1197	1	1
FeI_2	8	0.2746	1	1
HgI_2	8	0.2073	1	1
Te_2Ti	8	0.2563	1	1
CrI_2	8	0.274	1	1
BaF_2	8	0.0047	1	1
BiBrTe	8	0.0086	1	1
$RhTe_2$	8	0.2505	1	1
$CoTe_2$	8	1.5341	1	1
Se_2Ti	8	1.4465	1	1
AsKSn	8	0.0061	1	1
$PbTe_2$	8	0.0089	1	1
I_2Nd	8	0.1365	1	1
$NiTe_2$	8	0.2608	1	1
Cl_2Cu	8	0.0678	1	1
I_2V	8	0.2635	1	1
GeI_2	8	0.0011	1	1
Se_2Zr	8	0.2622	1	1
STl_2	8	0.0038	1	1
$PtSe_2$	8	0.2476	1	1
CoI_2	8	0.271	1	1
GeS_2	8	0.4307	1	1
$MnSe_2$	8	0.113	1	1
Br_2Ni	8	1.546	1	1
CeI_2	8	0.1351	1	1
Br_2Mg	8	0.2744	1	1
I_2Ti	8	0.2715	1	1
Cl_2Mg	8	1.5464	1	1
F_2Ni	8	0.1177	1	1
I_2La	8	0.1404	1	1
F_2Na	8	0.2586	1	1

Table showing the first 50 lattice matching combinations with the smallest number of atoms in the supercell.

Lattice matching - minimal strain

Formula	N° atoms	strain	cell size 1	cell size 2
Cl_2Mg	327	0.0001	36	49
Br_2Ni	327	0.0001	36	49
CdClHO	501	0.0002	49	64
$Cl_2Ho_2O_2$	911	0.0002	73	91
H_4Ti	865	0.0002	65	108
CCl_2Gd_2	650	0.0002	57	73
$Cl_2H_2Lu_2$	474	0.0002	36	49
Cl_2Fe	155	0.0002	16	25
PTe_2Ti_2	425	0.0003	36	49
MoS_2	93	0.0003	9	16
CBr_2Y_2	650	0.0003	57	73
$Cl_2O_2Tm_2$	806	0.0003	64	81
Br_2Zn	504	0.0004	57	73
H_2Li_2Pd	865	0.0004	65	108
$MoSe_2$	368	0.0004	37	61
S_2W	93	0.0004	9	16
CdClHO	443	0.0005	43	57
$Cl_2N_2Zr_2$	513	0.0005	45	48
$Hf_2I_2N_2$	557	0.0005	43	57
CaClHO	644	0.0005	64	81
F_2Na	563	0.0005	64	81
$Br_2S_2Yb_2$	857	0.0005	91	67
Na	161	0.0005	25	36
$Cl_2O_2Y_2$	911	0.0005	73	91
$I_2Lu_2S_2$	765	0.0005	81	60
MoS_2	93	0.0005	9	16
$Cl_2O_2Yb_2$	806	0.0005	64	81
O_2Zn	272	0.0005	25	49
I_2V	638	0.0006	73	91
Ga_2S_2	376	0.0006	36	49
Se_2W	368	0.0006	37	61
Cl_2Zr	155	0.0006	16	25
$AsLi_3$	9	0.0006	1	1
Te_2Ti	504	0.0007	57	73
$HfSe_2$	504	0.0007	57	73
PbS_2	705	0.0007	81	100
Au_2Br_2	626	0.0007	74	64
P_4	638	0.0008	70	72
Cu_4Te_2	649	0.0008	59	59
CdO_2	155	0.0008	16	25
PbTe	7	0.0008	1	1
$HNiO_2$	645	0.0008	49	100
Ga_2S_3	305	0.0009	25	36
As_2CeLi_2	10	0.0009	1	1
H_2NiO_2	125	0.0009	9	16
Cl_2Y_2	577	0.0009	57	73
Br_2CsF	517	0.0009	65	48
Br_2Y_2	805	0.0009	81	100
NS_2Zr	269	0.0009	25	36
$F_2Se_2Y_2$	614	0.0009	64	49

Table showing the first 50 lattice matching combinations with the smallest strain needed to build the supercell.

Bi_2Te_2S (P3m1)

Structural and electronic properties

**Formula**	Bi_2Te_2S
**Spacegroup**	P3m1
**Prototype**	Cu5FeS4(hR5)
**Parent 3D**	$Bi_6Te_4S_2$
**Source DB**	ICSD
**DB ID**	107587
**DF2-C09 Binding energy [meV/ Å^2]**	26.1
**RVV10 Binding energy [meV/ Å^2]**	31.67
**Band gap (PBE) [eV]**	1.19

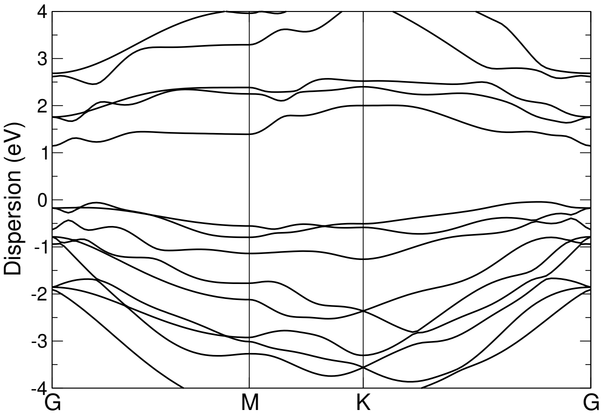

Band structure: Electronic band structure of Bi_2Te_2S (P3m1) in a representative window around the Fermi energy and along a high-symmetry path.

Optimized crystal structure

Structural parameters: relaxed cell (top) and atomic positions (bottom) of Bi_2Te_2S (P3m1) in Cartesian coordinates.

	x [Å]	y [Å]	z [Å]
$\mathbf{a_1}$	4.26886545	0.00000000	0.00000000
$\mathbf{a_2}$	−2.13443273	3.69694593	0.00000000
$\mathbf{a_3}$	0.00000000	0.00000000	27.14234394

		x [Å]	y [Å]	z [Å]
●	Bi	0.00000000	0.00000000	11.15134141
●	Bi	-0.00000000	2.46463062	15.44149163
●	Te	0.00000000	0.00000000	17.25720546
●	Te	2.13443273	1.23231531	13.32465376
●	S	-0.00000000	2.46463062	9.86775259

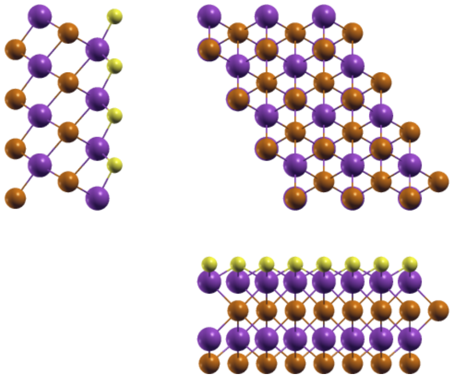

Orthographic projections: views of Bi_2Te_2S (P3m1) as seen from the x axis (left), the y axis (bottom) and the z axis (center).

Lattice matching - minimal size

Formula	N° atoms	strain	cell size 1	cell size 2
Tl	6	4.8777	1	1
Na	6	1.4459	1	1
InSe	7	0.0026	1	1
AsSb	7	0.2679	1	1
Bi_2	7	0.0032	1	1
PbTe	7	0.0009	1	1
CaCl	7	0.1124	1	1
$CdCl_2$	8	0.2752	1	1
CdI_2	8	0.0008	1	1
PSn_2	8	1.564	1	1
$MoSe_2$	8	4.8618	1	1
Br_2Zn	8	0.2536	1	1
Br_2Ca	8	0.0021	1	1
$InSe_2$	8	0.2758	1	1
$AsSn_2$	8	1.5916	1	1
$GeTe_2$	8	0.2737	1	1
$SiTe_2$	8	0.2593	1	1
I_2Pr	8	0.1344	1	1
I_2Mn	8	0.2754	1	1
S_2Zr	8	1.5585	1	1
Br_2Cu	8	0.7088	1	1
NSr_2	8	0.2703	1	1
PbS_2	8	0.2642	1	1
BiClTe	8	0.0012	1	1
Cl_2Zn	8	0.1188	1	1
FeI_2	8	0.2721	1	1
I_2Ni	8	0.274	1	1
Te_2Ti	8	0.254	1	1
CrI_2	8	0.2715	1	1
BaF_2	8	0.0064	1	1
BiBrTe	8	0.0068	1	1
$RhTe_2$	8	0.2482	1	1
$CoTe_2$	8	1.5221	1	1
AsKSn	8	0.0078	1	1
I_2Nd	8	0.1351	1	1
$NiTe_2$	8	0.2584	1	1
Cl_2Cu	8	0.0684	1	1
S_2Sn	8	1.5604	1	1
I_2V	8	0.2611	1	1
GeI_2	8	0.0006	1	1
Se_2Zr	8	0.2598	1	1
STl_2	8	0.0055	1	1
$PtSe_2$	8	1.5964	1	1
CoI_2	8	0.2685	1	1
GeS_2	8	0.4269	1	1
$MnSe_2$	8	0.1124	1	1
Br_2Ni	8	1.5339	1	1
CeI_2	8	0.1338	1	1
Br_2Mg	8	0.2719	1	1
I_2Ti	8	0.269	1	1

Table showing the first 50 lattice matching combinations with the smallest number of atoms in the supercell.

Lattice matching - minimal strain

Formula	N° atoms	strain	cell size 1	cell size 2
$SiTe_2$	563	0.0	64	81
ClH_3O	820	0.0	91	73
PbS_2	638	0.0001	73	91
$AsSn_2$	386	0.0001	43	57
$Br_2N_2Zr_2$	474	0.0001	36	49
Tl	326	0.0001	49	81
Br_2Pr_2	577	0.0002	57	73
CBr_2Lu_2	425	0.0002	36	49
PtS_2	233	0.0002	25	36
AsSb	605	0.0002	81	100
$Cl_2Er_2O_2$	806	0.0002	64	81
Br_2Y_2	729	0.0003	73	91
$LiMnTe_2$	9	0.0003	1	1
$I_2N_2Zr_2$	629	0.0003	49	64
Se_2V	368	0.0003	37	61
Bi_2Te_2	661	0.0003	81	64
S_2Ti	155	0.0004	16	25
Se_2Zr	563	0.0004	64	81
CdI_2	8	0.0004	1	1
$ClKO_3$	445	0.0004	64	25
Se_2W	488	0.0005	49	81
PTe_2Zr_2	905	0.0005	81	100
Tl	246	0.0005	37	61
Br_2La_2P	10	0.0005	1	1
$PtSe_2$	386	0.0005	43	57
$Cl_2O_2Yb_2$	723	0.0006	57	73
$Br_2Er_2S_2$	949	0.0006	101	74
$HfLiS_2$	269	0.0006	25	36
IKO_3	170	0.0006	25	9
$NiTe_2$	563	0.0006	64	81
F_2Na	504	0.0006	57	73
$MoSe_2$	488	0.0006	49	81
GeI_2	8	0.0006	1	1
CoI_2	705	0.0006	81	100
Ga_2Se_2	729	0.0007	73	91
$TaTe_2$	386	0.0007	43	57
$CrSe_2$	93	0.0007	9	16
Cl_2Co	155	0.0007	16	25
Au_2Br_2	644	0.0008	76	66
Pb_2Se_2	710	0.0008	86	70
$RhTe_2$	437	0.0008	49	64
NS_2Zr	269	0.0008	25	36
FHOZn	109	0.0008	9	16
Au_2Br_2	576	0.0008	68	59
H_2NiO_2	125	0.0008	9	16
As_2CeLi_2	10	0.0008	1	1
Br_2CsF	517	0.0008	65	48
Ga_2S_3	305	0.0008	25	36
CdI_2	8	0.0008	1	1
PbTe	7	0.0009	1	1

Table showing the first 50 lattice matching combinations with the smallest strain needed to build the supercell.

Bi_2Te_2Se (P-3m1)

Structural and electronic properties

**Formula**	Bi_2Te_2Se
**Spacegroup**	P-3m1
**Prototype**	Bi2Te2S
**Parent 3D**	Bi_2Te_2Se
**Source DB**	ICSD
**DB ID**	43512
**DF2-C09 Binding energy [meV/ Å^2]**	21.3
**RVV10 Binding energy [meV/ Å^2]**	27.54
**Band gap (PBE) [eV]**	0.93

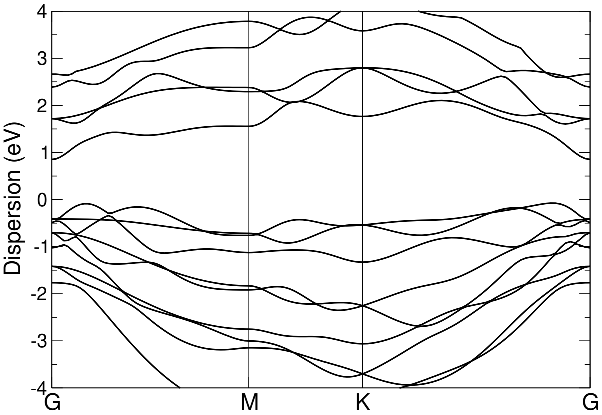

Band structure: Electronic band structure of Bi_2Te_2Se (P-3m1) in a representative window around the Fermi energy and along a high-symmetry path.

Optimized crystal structure

Structural parameters: relaxed cell (top) and atomic positions (bottom) of Bi_2Te_2Se (P-3m1) in Cartesian coordinates.

	x [Å]	y [Å]	z [Å]
$\mathbf{a_1}$	4.30272908	0.00000000	0.00000000
$\mathbf{a_2}$	−2.15136454	3.72627269	0.00000000
$\mathbf{a_3}$	0.00000000	0.00000000	27.33571239

		x [Å]	y [Å]	z [Å]
●	Bi	0.00000000	0.00000000	11.77527161
●	Te	0.00000000	0.00000000	17.34379842
●	Se	0.00000000	2.48418179	13.66785619
●	Bi	2.15136454	1.24209090	15.56044078
●	Te	2.15136454	1.24209090	9.99191397

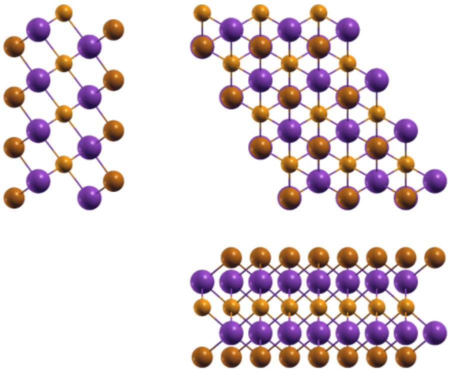

Orthographic projections: views of Bi_2Te_2Se (P-3m1) as seen from the x axis (left), the y axis (bottom) and the z axis (center).

Lattice matching - minimal size

Formula	N° atoms	strain	cell size 1	cell size 2
InSe	7	0.0063	1	1
AsSb	7	0.2626	1	1
Bi_2	7	0.0006	1	1
GeTe	7	0.2713	1	1
AgTl	7	0.1464	1	1
S_2	7	0.2736	1	1
PbTe	7	0.0046	1	1
CaCl	7	0.1111	1	1
$IrTe_2$	8	0.2726	1	1
$CdCl_2$	8	0.2697	1	1
CdI_2	8	0.0029	1	1
PSn_2	8	1.5371	1	1
Br_2Zn	8	0.2487	1	1
Br_2Ca	8	0.0016	1	1
$InSe_2$	8	0.2703	1	1
$AsSn_2$	8	1.5643	1	1
$GeTe_2$	8	0.2683	1	1
$SiTe_2$	8	0.2542	1	1
Te_2V	8	1.449	1	1
I_2Pr	8	0.1316	1	1
I_2Mn	8	0.2699	1	1
S_2Zr	8	1.5317	1	1
NSr_2	8	0.2649	1	1
PbS_2	8	0.259	1	1
BiClTe	8	0.0025	1	1
Cl_2Zn	8	0.117	1	1
FeI_2	8	0.2667	1	1
I_2Ni	8	0.2685	1	1
Te_2Ti	8	0.2491	1	1
CrI_2	8	0.2661	1	1
BiBrTe	8	0.003	1	1
$RhTe_2$	8	1.5849	1	1
CdClO	8	1.4457	1	1
S_2Ta	8	4.8451	1	1
I_2Nd	8	0.1323	1	1
$NiTe_2$	8	0.2534	1	1
Cl_2Cu	8	0.0696	1	1
S_2Sn	8	1.5336	1	1
I_2V	8	0.256	1	1
GeI_2	8	0.0043	1	1
Se_2Zr	8	0.2547	1	1
STl_2	8	0.0091	1	1
OTl_2	8	1.4469	1	1
CoI_2	8	0.2632	1	1
$MnSe_2$	8	0.1111	1	1
CeI_2	8	0.131	1	1
Br_2Mg	8	0.2665	1	1
I_2Ti	8	0.2637	1	1
$NbTe_2$	8	1.5302	1	1
GdI_2	8	0.0059	1	1

Table showing the first 50 lattice matching combinations with the smallest number of atoms in the supercell.

Lattice matching - minimal strain

Formula	N° atoms	strain	cell size 1	cell size 2
$AgNO_3$	305	0.0	36	25
CI_2Y_2	820	0.0	73	91
$LiOS_2Ti$	490	0.0	37	61
CCl_2Sc_2	205	0.0	16	25
$I_2N_2Zr_2$	557	0.0001	43	57
$HfSe_2$	437	0.0001	49	64
Te_2Ti	437	0.0001	49	64
Se_2Sn	638	0.0001	73	91
PbS_2	563	0.0002	64	81
$Cl_2H_2Zr_2$	731	0.0003	49	81
Cl_2Zr_2	180	0.0003	16	25
I_2V	504	0.0004	57	73
NSr_2	638	0.0004	73	91
$Br_2H_2Zr_2$	230	0.0004	16	25
Br_2Zn	437	0.0004	49	64
I_2Ti	638	0.0005	73	91
$GeTe_2$	705	0.0005	81	100
Br_2Y_2	644	0.0005	64	81
Bi_2Te_2	747	0.0005	91	73
Se_2Zr	504	0.0005	57	73
$C_2I_2La_2$	638	0.0005	58	58
CCl_2Gd_2	565	0.0006	49	64
C	45	0.0006	4	25
NbS_2	488	0.0006	49	81
N_2W	432	0.0006	39	79
Bi_2	7	0.0006	1	1
$CrSe_2$	93	0.0006	9	16
FeI_2	705	0.0006	81	100
FeH_2O_2	590	0.0006	39	79
I_2Ni	705	0.0007	81	100
LiO	77	0.0007	9	16
$Cl_2Er_2O_2$	723	0.0007	57	73
H_2Li_2Pt	645	0.0007	49	80
Bi_2Se_2	620	0.0007	72	65
$Cl_2Ho_2O_2$	723	0.0007	57	73
Te_2V	233	0.0007	25	36
Br_2Mg	705	0.0007	81	100
Br_2Pr_2	501	0.0008	49	64
Te_4TiZr	705	0.0008	81	50
Br_2Mn	233	0.0008	25	36
Ca_2O_2	877	0.0008	81	118
CoI_2	638	0.0008	73	91
NaO_4	305	0.0008	36	25
$Br_2S_2Y_2$	765	0.0008	81	60
Ga_2Se_2	644	0.0008	64	81
$Cl_2H_2Zr_2$	551	0.0009	37	61
As_4	642	0.0009	74	68
$SiTe_2$	504	0.0009	57	73
ClNZr	155	0.001	16	25
GeI_3Rb	290	0.001	49	9

Table showing the first 50 lattice matching combinations with the smallest strain needed to build the supercell.

Bi_2Te_3 (P-3m1)

Structural and electronic properties

**Formula**	Bi_2Te_3
**Spacegroup**	P-3m1
**Prototype**	Bi2Te3
**Parent 3D**	$SnBi_4Te_7$
**Source DB**	ICSD
**DB ID**	236253
**DF2-C09 Binding energy [meV/ Å^2]**	23.05
**RVV10 Binding energy [meV/ Å^2]**	28.73
**Band gap (PBE) [eV]**	1.02

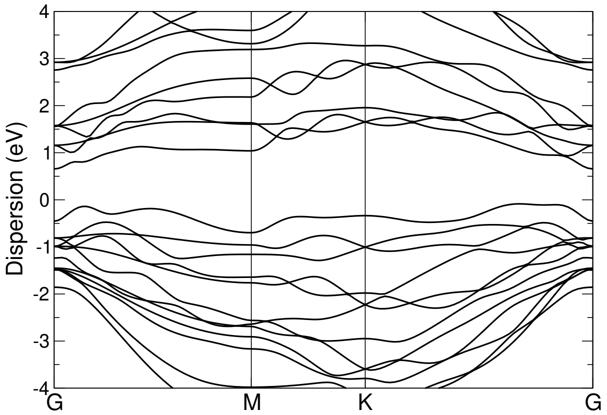

Band structure: Electronic band structure of Bi_2Te_3 (P-3m1) in a representative window around the Fermi energy and along a high-symmetry path.

Optimized crystal structure

Structural parameters: relaxed cell (top) and atomic positions (bottom) of Bi_2Te_3 (P-3m1) in Cartesian coordinates.

	x [Å]	y [Å]	z [Å]
$\mathbf{a_1}$	4.44039625	−0.00000000	0.00000000
$\mathbf{a_2}$	−2.22019813	3.84549596	0.00000000
$\mathbf{a_3}$	0.00000000	0.00000000	31.27050965

		x [Å]	y [Å]	z [Å]
●	Bi	2.22019813	1.28183199	19.58214488
●	Te	0.00000000	0.00000000	21.28374927
●	Te	-0.00000000	2.56366397	17.51147466
●	Sn	-0.00000000	-0.00000000	15.63525483
●	Bi	-0.00000000	2.56366397	11.68836477
●	Te	0.00000000	0.00000000	9.98676038
●	Te	2.22019813	1.28183199	13.75903499

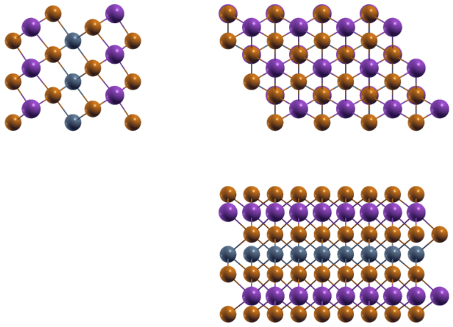

Orthographic projections: views of Bi_2Te_3 (P-3m1) as seen from the x axis (left), the y axis (bottom) and the z axis (center).

Bi_2TeSe_2 (P-3m1)

Structural and electronic properties

**Formula**	Bi_2TeSe_2
**Spacegroup**	P-3m1
**Prototype**	Bi2Te2S
**Parent 3D**	Bi_2TeSe_2
**Source DB**	COD
**DB ID**	9004849
**DF2-C09 Binding energy [meV/ Å^2]**	20.92
**RVV10 Binding energy [meV/ Å^2]**	28.22
**Band gap (PBE) [eV]**	1.15

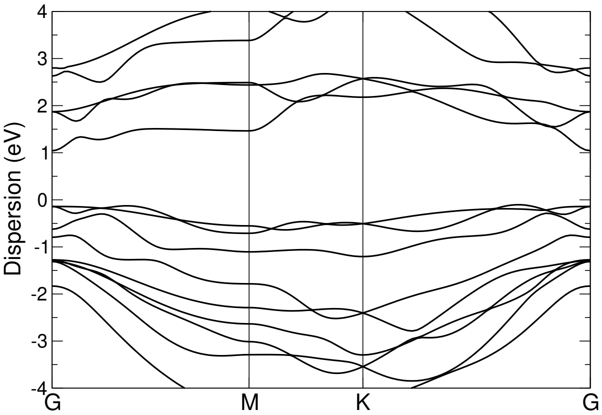

Band structure: Electronic band structure of Bi_2TeSe_2 (P-3m1) in a representative window around the Fermi energy and along a high-symmetry path.

Optimized crystal structure

Structural parameters: relaxed cell (top) and atomic positions (bottom) of Bi_2TeSe_2 (P-3m1) in Cartesian coordinates.

	x [Å]	y [Å]	z [Å]
$\mathbf{a_1}$	4.23288085	0.00000000	0.00000000
$\mathbf{a_2}$	−2.11644042	3.66578234	0.00000000
$\mathbf{a_3}$	0.00000000	0.00000000	27.36786039

		x [Å]	y [Å]	z [Å]
●	Bi	2.11644042	1.22192745	15.84463395
●	Se	2.11644042	1.22192745	9.98688056
●	Bi	0.00000000	0.00000000	11.52322644
●	Se	0.00000000	0.00000000	17.38097983
●	Te	-0.00000000	2.44385490	13.68393020

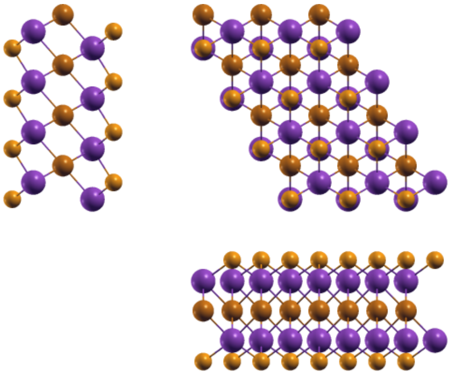

Orthographic projections: views of Bi_2TeSe_2 (P-3m1) as seen from the x axis (left), the y axis (bottom) and the z axis (center).

Lattice matching - minimal size

Formula	N° atoms	strain	cell size 1	cell size 2
InSe	7	0.0014	1	1
AsSb	7	0.2737	1	1
Bi_2	7	0.0072	1	1
AgTl	7	0.1538	1	1
As_2	7	1.5287	1	1
P_2	7	4.8509	1	1
PbTe	7	0.0031	1	1
CaCl	7	0.114	1	1
Cl_2Zn	8	1.5213	1	1
I_2Mg	8	0.0088	1	1
CdI_2	8	0.0049	1	1
PSn_2	8	1.5933	1	1
Br_2Zn	8	0.2591	1	1
Br_2Ca	8	0.0061	1	1
HfS_2	8	1.5483	1	1
$AsSn_2$	8	0.2498	1	1
$SiTe_2$	8	0.2649	1	1
I_2Pr	8	0.1376	1	1
S_2Zr	8	1.5876	1	1
Br_2La	8	0.0084	1	1
Br_2Cu	8	0.7231	1	1
Ca_2Si	8	0.4532	1	1
PbS_2	8	0.2699	1	1
Br_2Co	8	1.5257	1	1
BiClTe	8	0.0053	1	1
Ca_2N	8	1.5325	1	1
Cl_2Ti	8	4.8542	1	1
BrCdI	8	0.0057	1	1
Cl_2Zn	8	0.121	1	1
Te_2Ti	8	0.2595	1	1
BaF_2	8	0.0025	1	1
$RhTe_2$	8	0.2535	1	1
$CoTe_2$	8	1.5506	1	1
AsKSn	8	0.0039	1	1
$PbTe_2$	8	0.0067	1	1
I_2Nd	8	0.1383	1	1
$NiTe_2$	8	0.264	1	1
Cl_2Cu	8	0.0671	1	1
S_2Sn	8	1.5896	1	1
I_2V	8	0.2668	1	1
GeI_2	8	0.0034	1	1
Se_2Zr	8	0.2655	1	1
STl_2	8	0.0015	1	1
$PtSe_2$	8	0.2506	1	1
CoI_2	8	0.2744	1	1
Br_2Fe	8	1.5262	1	1
$TaTe_2$	8	0.249	1	1
$MnSe_2$	8	0.1139	1	1
Br_2Ni	8	1.5626	1	1
CeI_2	8	0.1369	1	1

Table showing the first 50 lattice matching combinations with the smallest number of atoms in the supercell.

Lattice matching - minimal strain

Formula	N° atoms	strain	cell size 1	cell size 2
HN_3OZn	731	0.0	49	81
PSn_2	386	0.0001	43	57
Br_2Zn	563	0.0001	64	81
Te_2Ti	563	0.0002	64	81
CoO_2	47	0.0002	4	9
Cl_2Hf_2	180	0.0002	16	25
$CuTe_2$	327	0.0002	36	49
$TaTe_2$	437	0.0002	49	64
$HfSe_2$	563	0.0002	64	81
Mg_2	541	0.0002	65	108
H_2MgO_2	125	0.0002	9	16
HfS_2	327	0.0002	36	49
Br_3Cs	420	0.0002	64	25
$NiTe_2$	638	0.0002	73	91
Ho_2S_2	355	0.0002	39	40
Cu_2O_2	355	0.0002	39	40
CCl_2Gd_2	725	0.0003	64	81
$HNiO_2$	511	0.0003	39	79
$I_2N_2Zr_2$	723	0.0003	57	73
Sb_2SeTe_2	10	0.0004	1	1
S_2Sn	386	0.0004	43	57
NiO_2	47	0.0004	4	9
$SiTe_2$	638	0.0004	73	91
$AsSn_2$	437	0.0004	49	64
In_2Te_3	10	0.0004	1	1
$Al_2Cl_2O_2$	39	0.0004	3	4
ClH_3O	725	0.0004	81	64
GeI_2Y_2	10	0.0005	1	1
As_2O_3	490	0.0005	61	37
$CoTe_2$	327	0.0005	36	49
Sn_2Te_2	599	0.0005	75	56
I_2V	705	0.0005	81	100
FeH_2O_2	370	0.0006	25	49
HN_3OZn	551	0.0006	37	61
CaH_2O_2	425	0.0006	36	49
N_2W	272	0.0006	25	49
S_2Zr	386	0.0006	43	57
$Cl_2Er_2O_2$	911	0.0006	73	91
Ga_2S_2	376	0.0007	36	49
As_2Li_2Nd	10	0.0007	1	1
Ga_2Se_2	577	0.0007	57	73
$Hf_2I_2N_2$	629	0.0007	49	64
CdClHO	501	0.0007	49	64
Br_2Zr_2	269	0.0008	25	36
$Cl_2Hg_2N_2$	385	0.0008	47	25
CaClHO	729	0.0008	73	91
Se_2Zr	638	0.0008	73	91
CBr_2Y_2	725	0.0008	64	81
$Br_2O_2Ti_2$	470	0.0008	40	45
$NbTe_2$	386	0.0008	43	57

Table showing the first 50 lattice matching combinations with the smallest strain needed to build the supercell.

BiOBr (P4/nmm)

Structural and electronic properties

**Formula**	BiOBr
**Spacegroup**	P4/nmm
**Prototype**	PbClF
**Parent 3D**	$Bi_2O_2Br_2$
**Source DB**	COD
**DB ID**	9009161
**DF2-C09 Binding energy [meV/ Å^2]**	18.12
**RVV10 Binding energy [meV/ Å^2]**	26.08
**Band gap (PBE) [eV]**	2.44

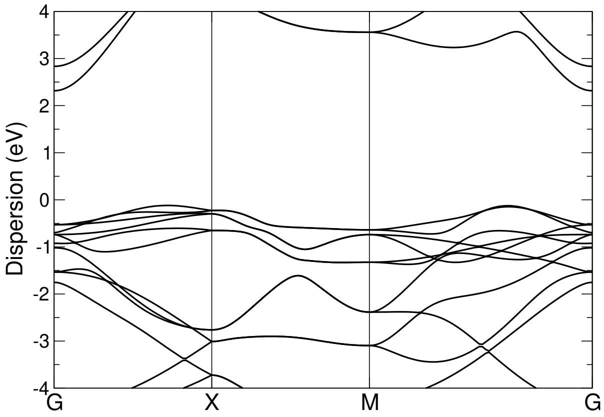

Band structure: Electronic band structure of BiOBr (P4/nmm) in a representative window around the Fermi energy and along a high-symmetry path.

Optimized crystal structure

Structural parameters: relaxed cell (top) and atomic positions (bottom) of BiOBr (P4/nmm) in Cartesian coordinates.

	x [Å]	y [Å]	z [Å]
$\mathbf{a_1}$	3.93231841	0.00000000	0.00000000
$\mathbf{a_2}$	0.00000000	3.93231841	0.00000000
$\mathbf{a_3}$	0.00000000	0.00000000	25.67241585

		x [Å]	y [Å]	z [Å]
●	Bi	0.00000000	1.96615920	14.12107722
●	Br	1.96615920	0.00000000	15.73157682
●	Bi	1.96615920	0.00000000	11.55133863
●	Br	0.00000000	1.96615920	9.94083904
•	O	0.00000000	0.00000000	12.83620793
•	O	1.96615920	1.96615920	12.83620793

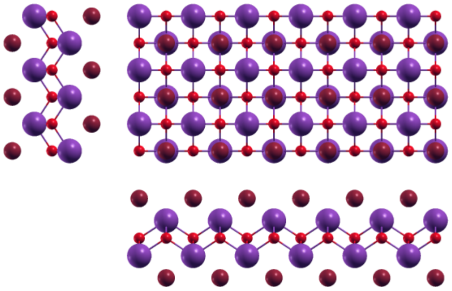

Orthographic projections: views of BiOBr (P4/nmm) as seen from the x axis (left), the y axis (bottom) and the z axis (center).

Lattice matching - minimal size

Formula	N° atoms	strain	cell size 1	cell size 2
K	7	0.1801	1	1
InSe	8	0.1428	1	1
Bi_2	8	0.148	1	1
AgTl	8	0.0197	1	1
Ag_2	8	0.187	1	1
LiO	8	0.1104	1	1
P_2	8	0.1088	1	1
PbTe	8	0.1443	1	1
Sb_2	8	0.1301	1	1
I_2Mg	9	0.1343	1	1
CdI_2	9	0.1459	1	1
Nd	9	0.7441	1	3
Ba_2Pt	9	0.1866	1	1
Br_2Ca	9	0.147	1	1
CaI_2	9	0.1692	1	1
I_2Pr	9	0.0031	1	1
Br_2La	9	0.1345	1	1
Br_2Cu	9	0.1041	1	1
Ca_2Si	9	0.1925	1	1
I_2Yb	9	0.1664	1	1
BiClTe	9	0.1462	1	1
Cl_2Ti	9	0.1088	1	1
BrCdI	9	0.1368	1	1
HgI_2	9	0.4078	1	1
BaF_2	9	0.1395	1	1
BiBrTe	9	0.1514	1	1
$RhTe_2$	9	0.1086	1	1
GeI_2	9	0.1329	1	1
AsKSn	9	0.1383	1	1
$PbTe_2$	9	0.136	1	1
I_2Nd	9	0.0022	1	1
Cl_2Cu	9	0.0979	1	1
I_2Tm	9	0.1679	1	1
$SnTe_2$	9	0.1312	1	1
Cl_2V	9	0.111	1	1
GeI_2	9	0.1445	1	1
I_2Pb	9	0.189	1	1
STl_2	9	0.1403	1	1
BiITe	9	0.158	1	1
GeS_2	9	0.2148	1	1
DyI_2	9	0.1725	1	1
CeI_2	9	0.0039	1	1
Se_2Yb	9	0.133	1	1
$BiTe_2$	9	0.1332	1	1
GdI_2	9	0.1543	1	1
$CrSe_2$	9	0.1113	1	1
I_2La	9	0.0026	1	1
$CrSe_2$	9	0.1107	1	1
CdI_2	9	0.1455	1	1
F_2Zn	9	0.007	1	1

Table showing the first 50 lattice matching combinations with the smallest number of atoms in the supercell.

Lattice matching - minimal strain

Formula	N° atoms	strain	cell size 1	cell size 2
$CoTe_2$	483	0.0002	48	65
FeH_2O_2	315	0.0003	20	39
H_2Na_2Pd	800	0.0003	65	82
Ho_2S_2	494	0.0003	49	50
HfS_2	483	0.0003	48	65
Ga_2S_2	548	0.0003	48	65
AlH_4Na	366	0.0003	36	25
$AgTe_2$	942	0.0003	89	136
Ag_2I_2	590	0.0004	65	50
$Er_2I_2O_2$	12	0.0004	1	1
Mg_2	456	0.0004	49	81
$Br_2Eu_2F_2$	12	0.0004	1	1
Ag_2F_4	108	0.0006	12	6
$CuTe_2$	483	0.0006	48	65
HgI_2	540	0.0007	65	50
Cu_2O_2	494	0.0007	49	50
Sn	70	0.0007	9	16
Ag_2I_2	580	0.0007	64	49
Se_2Ta	840	0.0007	81	118
Br_2Ti	840	0.0007	81	118
Bi_2In_2	754	0.0007	85	61
$Ba_2H_2I_2$	510	0.0009	49	36
$I_2O_2Y_2$	12	0.0009	1	1
Br_2Cr	840	0.0009	81	118
$Au_2K_2S_2$	78	0.001	10	3
H_2Na_2Pd	789	0.001	64	81
CaH_2O_2	613	0.001	48	65
N_2W	237	0.001	20	39
F_4Pb	817	0.001	82	65
GeS_2	636	0.0011	65	82
$Cu_2Rb_2Te_2$	876	0.0011	85	61
Cl_2Sc_2	958	0.0011	81	118
$Br_2Nd_2O_2$	12	0.0011	1	1
$I_2O_2Tm_2$	12	0.0011	1	1
NbS_2	852	0.0011	79	126
H_4Ti	920	0.0011	65	106
$CrTe_2$	840	0.0012	81	118
Br_2Cu	969	0.0012	103	117
$Cl_2N_2Zr_2$	678	0.0013	48	65
Br_2Cu	837	0.0013	89	101
Ga_2S_2	548	0.0013	48	65
$AgBrO_2$	984	0.0014	96	102
Br_2Cu	762	0.0014	81	92
Ca_2O_2	706	0.0015	61	85
Br_2Cu	705	0.0015	75	85
Te_2W	531	0.0015	52	73
$MoTe_2$	531	0.0015	52	73
H_2Li_2Pd	699	0.0015	49	81
As_2O_3	973	0.0015	108	65
CdH_2O_2	677	0.0016	52	73

Table showing the first 50 lattice matching combinations with the smallest strain needed to build the supercell.

BiOCl (P4/nmm)

Structural and electronic properties

**Formula**	BiOCl
**Spacegroup**	P4/nmm
**Prototype**	PbClF
**Parent 3D**	$Bi_2O_2Cl_2$
**Source DB**	COD
**DB ID**	4509949
**DF2-C09 Binding energy [meV/ Å^2]**	23.24
**RVV10 Binding energy [meV/ Å^2]**	31.21
**Band gap (PBE) [eV]**	2.75

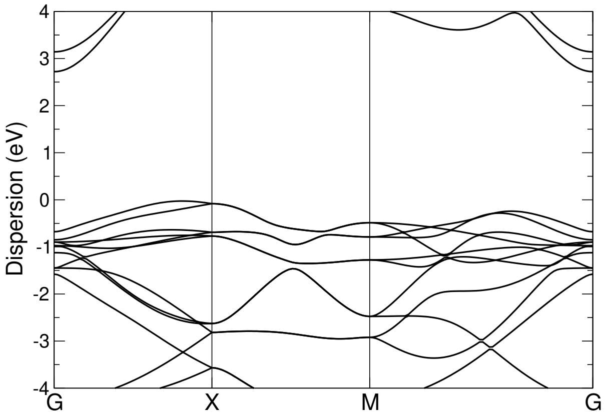

Band structure: Electronic band structure of BiOCl (P4/nmm) in a representative window around the Fermi energy and along a high-symmetry path.

Optimized crystal structure

Structural parameters: relaxed cell (top) and atomic positions (bottom) of BiOCl (P4/nmm) in Cartesian coordinates.

		x [Å]	y [Å]	z [Å]
$\mathbf{a_1}$		3.88753240	0.00000000	0.00000000
$\mathbf{a_2}$		0.00000000	3.88753240	0.00000000
$\mathbf{a_3}$		0.00000000	0.00000000	25.27720527
		x [Å]	y [Å]	z [Å]
	Bi	0.00000000	1.94376620	13.93245856
	Bi	1.94376620	0.00000000	11.34474670
	Cl	1.94376620	0.00000000	15.34289105
	Cl	0.00000000	1.94376620	9.93431421
	O	0.00000000	0.00000000	12.63860263
	O	1.94376620	1.94376620	12.63860263

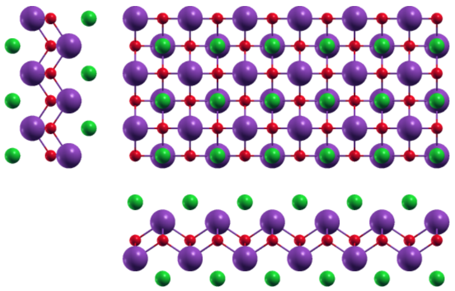

Orthographic projections: views of BiOCl (P4/nmm) as seen from the x axis (left), the y axis (bottom) and the z axis (center).

Lattice matching - minimal size

Formula	N° atoms	strain	cell size 1	cell size 2
K	7	0.3819	1	1
K	7	0.1869	1	1
InSe	8	0.1477	1	1
Bi_2	8	0.1532	1	1
AgTl	8	0.0245	1	1
Ag_2	8	0.194	1	1
LiO	8	0.1092	1	1
PbTe	8	0.1493	1	1
Sb_2	8	0.1341	1	1
I_2Mg	9	0.1386	1	1
S_2V	9	0.1108	1	1
MoS_2	9	0.1107	1	1
CdI_2	9	0.151	1	1
Ba_2Pt	9	0.1937	1	1
Br_2Ca	9	0.1522	1	1
CaI_2	9	0.1755	1	1
I_2Pr	9	0.0023	1	1
Br_2La	9	0.1389	1	1
Br_2Cu	9	0.1065	1	1
I_2Yb	9	0.1726	1	1
BiClTe	9	0.1513	1	1
BrCdI	9	0.1413	1	1
HgI_2	9	1.1292	1	1
I_2Zn	9	0.1316	1	1
BaF_2	9	0.1442	1	1
BiBrTe	9	0.1568	1	1
S_2W	9	0.1107	1	1
GeI_2	9	0.1371	1	1
AsKSn	9	0.1429	1	1
$PbTe_2$	9	0.1404	1	1
I_2Nd	9	0.0032	1	1
Cl_2Cu	9	0.099	1	1
I_2Tm	9	0.1741	1	1
$SnTe_2$	9	0.1353	1	1
Cl_2V	9	0.1098	1	1
GeI_2	9	0.1495	1	1
I_2Pb	9	0.1961	1	1
STl_2	9	0.145	1	1
BiITe	9	0.1638	1	1
GeS_2	9	0.221	1	1
DyI_2	9	0.1789	1	1
CeI_2	9	0.0015	1	1
Se_2Yb	9	0.1373	1	1
MoS_2	9	0.1106	1	1
$BiTe_2$	9	0.1375	1	1
GdI_2	9	0.1598	1	1
$CrSe_2$	9	0.11	1	1
I_2La	9	0.0081	1	1
$CrSe_2$	9	0.1095	1	1
CdI_2	9	0.1506	1	1

Table showing the first 50 lattice matching combinations with the smallest number of atoms in the supercell.

Lattice matching - minimal strain

Formula	N° atoms	strain	cell size 1	cell size 2
H_2Na_2Pd	997	0.0	82	101
K	681	0.0002	100	81
Br_2Mn	483	0.0003	48	65
$FeSe_2$	102	0.0004	9	16
$Br_2Cr_2S_2$	504	0.0005	42	42
Pb_2Se_2	292	0.0005	32	25
HgI_2	933	0.0005	113	85
$Ca_2Ge_2Mn_2$	12	0.0006	1	1
H_2Na_2Pd	986	0.0006	81	100
$As_2Cd_2K_2$	510	0.0006	49	36
KS_2Ti	548	0.0006	48	65
Cl_2Ni	840	0.0007	81	118
$Br_2F_2Tm_2$	12	0.0007	1	1
$Ba_2H_2I_2$	876	0.0007	85	61
$Br_2O_2V_2$	66	0.0008	5	6
In	655	0.0008	85	145
$I_2Lu_2O_2$	12	0.0008	1	1
GeS_2	795	0.0008	82	101
HgO	222	0.0009	25	36
Br_2Ca_3Si	198	0.0009	21	12
$ReSe_2$	840	0.0009	81	118
$NbSe_2$	840	0.0009	81	118
$C_2I_2Y_2$	864	0.001	70	74
Se_2Ta	840	0.001	81	118
$C_2I_2Y_2$	666	0.0012	54	57
Ni_2Se_2	10	0.0012	1	1
Te_2V	483	0.0013	48	65
$H_2Na_2O_2$	690	0.0013	50	65
CuO_2	678	0.0014	63	100
GeS_2	786	0.0014	81	100
Se_2Sn_2	924	0.0014	100	81
$Ba_2F_2I_2$	510	0.0014	49	36
$Br_2Gd_2O_2$	12	0.0014	1	1
Cl_2Zn	483	0.0014	48	65
$AgClO_4$	246	0.0015	25	16
CeI_2	9	0.0015	1	1
Pd_2S_4	630	0.0015	70	35
$MoSe_2$	852	0.0015	79	126
O_4PSn	510	0.0016	49	36
CaI_2	885	0.0016	103	89
Br_2V	840	0.0016	81	118
$Br_2Eu_2O_2$	12	0.0016	1	1
H_2NiO_2	929	0.0016	64	109
CaI_2	765	0.0016	89	77
Ca_2O_2	412	0.0016	36	49
$Cl_2Hf_2N_2$	678	0.0016	48	65
OTl_2	483	0.0016	48	65
Au_2I_2	834	0.0016	91	72
$CrSe_2$	711	0.0016	64	109
Ga_2Se_2	680	0.0016	62	77

Table showing the first 50 lattice matching combinations with the smallest strain needed to build the supercell.

BiOI (P4/nmm)

Structural and electronic properties

**Formula**	BiOI
**Spacegroup**	P4/nmm
**Prototype**	PbClF
**Parent 3D**	$Bi_2O_2I_2$
**Source DB**	COD
**DB ID**	9009164
**DF2-C09 Binding energy [meV/ Å^2]**	14.48
**RVV10 Binding energy [meV/ Å^2]**	21.05
**Band gap (PBE) [eV]**	1.53

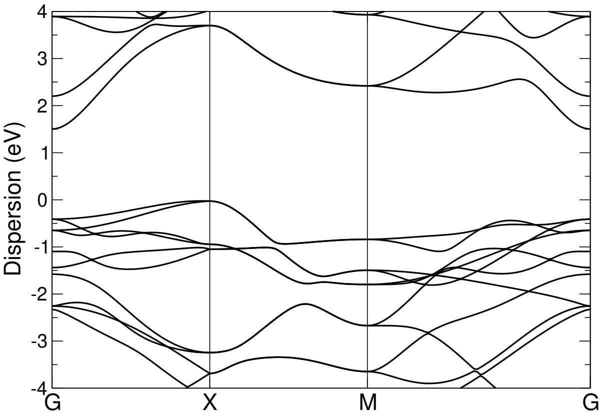

Band structure: Electronic band structure of BiOI (P4/nmm) in a representative window around the Fermi energy and along a high-symmetry path.

Optimized crystal structure

Structural parameters: relaxed cell (top) and atomic positions (bottom) of BiOI (P4/nmm) in Cartesian coordinates.

		x [Å]	y [Å]	z [Å]
$\mathbf{a_1}$		4.02670084	0.00000000	0.00000000
$\mathbf{a_2}$		0.00000000	4.02670084	0.00000000
$\mathbf{a_3}$		0.00000000	0.00000000	26.29854825
		x [Å]	y [Å]	z [Å]
●	Bi	0.00000000	2.01335042	14.39409000
●	I	2.01335042	0.00000000	16.28756732
●	Bi	2.01335042	0.00000000	11.90445824
●	I	0.00000000	2.01335042	10.01098092
•	O	0.00000000	0.00000000	13.14927412
•	O	2.01335042	2.01335042	13.14927412

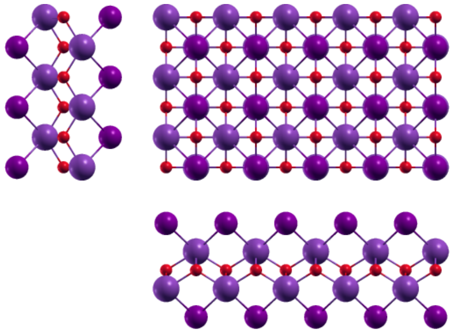

Orthographic projections: views of BiOI (P4/nmm) as seen from the x axis (left), the y axis (bottom) and the z axis (center).

Lattice matching - minimal size

Formula	N° atoms	strain	cell size 1	cell size 2
K	7	0.167	1	1
Tl	7	0.11	1	1
InSe	8	0.1336	1	1
Bi_2	8	0.1382	1	1
Ag_2	8	0.1732	1	1
P_2	8	0.1112	1	1
PbTe	8	0.1349	1	1
CaCl	8	0.2158	1	1
Cl_2Mn	9	0.1088	1	1
CdI_2	9	0.1363	1	1
Nd	9	0.1804	1	3
$MoSe_2$	9	0.1102	1	1
Ba_2Pt	9	0.1729	1	1
Br_2Ca	9	0.1373	1	1
CaI_2	9	0.1571	1	1
Br_2Cu	9	0.1002	1	1
Ca_2Si	9	0.1783	1	1
I_2Yb	9	0.1545	1	1
BiClTe	9	0.1366	1	1
Cl_2Ti	9	0.1112	1	1
HgI_2	9	0.383	1	1
BaF_2	9	0.1307	1	1
BiBrTe	9	0.1412	1	1
NbS_2	9	0.1094	1	1
S_2Ta	9	0.1096	1	1
CKN	9	0.4867	1	1
Se_2V	9	0.1098	1	1
AsKSn	9	0.1297	1	1
Cl_2Cu	9	0.0966	1	1
I_2Tm	9	0.1559	1	1
GeI_2	9	0.1351	1	1
I_2Pb	9	0.1751	1	1
STl_2	9	0.1314	1	1
BiITe	9	0.147	1	1
GeS_2	9	0.5891	1	1
$MnSe_2$	9	0.2157	1	1
DyI_2	9	0.1601	1	1
GdI_2	9	0.1437	1	1
I_2La	9	0.0085	1	1
CdI_2	9	0.136	1	1
Sm	9	0.1615	1	3
I_2Pr	9	0.1367	1	1
Se_2W	9	0.1102	1	1
Bi_2Te_2	10	0.1945	1	1
Bi_2In_2	10	1.1275	1	1
Cu_2I_2	10	0.0077	1	1
Cu_2Sr_2	10	0.1421	1	1
Cl_2OOs	10	0.2221	1	1
$LiMnTe_2$	10	0.1354	1	1
Cu_2Te_2	10	0.0575	1	1

Table showing the first 50 lattice matching combinations with the smallest number of atoms in the supercell.

Lattice matching - minimal strain

Formula	N° atoms	strain	cell size 1	cell size 2
$Cu_2Na_2Se_2$	12	0.0	1	1
$Cl_2F_2Pb_2$	12	0.0001	1	1
$Ba_2F_2I_2$	882	0.0001	82	65
Fe_2S_2	718	0.0001	65	82
$F_2I_2Tm_2$	12	0.0001	1	1
HgI_2	843	0.0003	100	81
I_3Sn	866	0.0004	109	53
CoH_2O_2	315	0.0005	20	39
$Ba_2H_2I_2$	690	0.0005	65	50
$Ba_2H_2I_2$	678	0.0006	64	49
$Hf_2Se_2Si_2$	678	0.0006	49	64
O_4PTl	882	0.0006	82	65
Ca_2Cl_2	896	0.0006	82	101
ReS_2	237	0.0006	20	39
CdClHO	548	0.0006	48	65
Fe_2S_2	708	0.0007	64	81
Cl_4Mn	474	0.0008	49	36
$PtSe_2$	483	0.0008	48	65
$Ba_2F_2I_2$	870	0.0008	81	64
Cl_2Rb_2	484	0.0009	64	25
Te_2V	840	0.0009	81	118
$I_2Nd_2O_2$	12	0.0009	1	1
Br_2Mn	840	0.0011	81	118
As_2Fe_2	550	0.0011	49	64
$AgClO_2$	384	0.0011	36	42
OTl_2	840	0.0011	81	118
Ca_2Cl_2	886	0.0012	81	100
O_4PTl	870	0.0012	81	64
$As_2Cd_2K_2$	870	0.0012	81	64
Mg_4	814	0.0012	65	106
$AgClO_4$	366	0.0012	36	25
Mg_3	537	0.0012	49	81
O_2Sn_2	740	0.0012	66	86
FKO_2Se	270	0.0012	30	18
As_2Co_2	896	0.0013	82	101
CdClO	840	0.0013	81	118
$H_2Na_2O_2$	876	0.0013	61	85
Br_2CsF	580	0.0013	64	49
$Ba_2Ge_2Mn_2$	12	0.0014	1	1
Ag_2I_2	924	0.0014	100	81
$RhTe_2$	483	0.0014	48	65
$MoSe_2$	711	0.0014	64	109
KS_2Ti	958	0.0014	81	118
Se_2W	711	0.0014	64	109
I_3Sn	796	0.0014	100	49
$AsSn_2$	483	0.0014	48	65
CNNa	489	0.0015	55	53
Tl	496	0.0015	65	106
Ni_2Te_2	680	0.0016	62	77
$Cl_2H_2Lu_2$	750	0.0016	52	73

Table showing the first 50 lattice matching combinations with the smallest strain needed to build the supercell.

BiTeBr (P3m1)

Structural and electronic properties

**Formula**	BiTeBr
**Spacegroup**	P3m1
**Prototype**	BiTeI
**Parent 3D**	BiBrTe
**Source DB**	ICSD
**DB ID**	672482
**DF2-C09 Binding energy [meV/ Å^2]**	16.02
**RVV10 Binding energy [meV/ Å^2]**	N/A
**Band gap (PBE) [eV]**	1.64

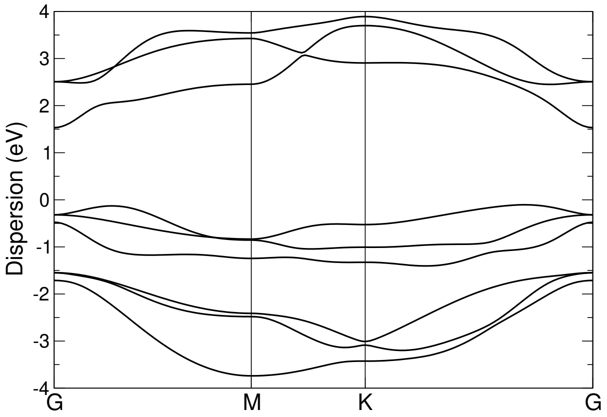

Band structure: Electronic band structure of BiTeBr (P3m1) in a representative window around the Fermi energy and along a high-symmetry path.

Optimized crystal structure

Structural parameters: relaxed cell (top) and atomic positions (bottom) of BiTeBr (P3m1) in Cartesian coordinates.

	x [Å]	y [Å]	z [Å]
$\mathbf{a_1}$	−2.16513804	−3.75012910	0.00000000
$\mathbf{a_2}$	4.33027609	0.00000000	0.00000000
$\mathbf{a_3}$	0.00000000	0.00000000	19.32564878

	x [Å]	y [Å]	z [Å]
Bi	0.00000000	0.00000000	-0.02723203
Te	2.16513804	-1.25004303	-1.77650254
Br	0.00000000	-2.50008606	1.80373457

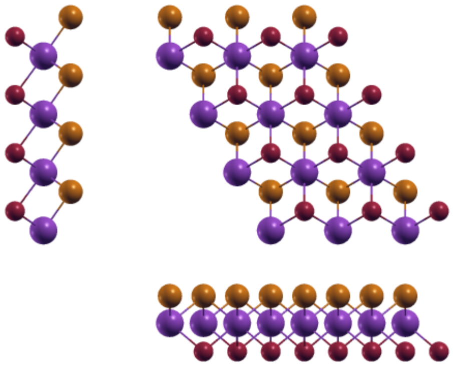

Orthographic projections: views of BiTeBr (P3m1) as seen from the x axis (left), the y axis (bottom) and the z axis (center).

Lattice matching - minimal size

Formula	N° atoms	strain	cell size 1	cell size 2
InSe	5	0.0092	1	1
AsSb	5	0.2584	1	1
Bi_2	5	0.0036	1	1
GeTe	5	0.2669	1	1
S_2	5	0.2692	1	1
PbTe	5	0.0075	1	1
$IrTe_2$	6	0.2682	1	1
$CdCl_2$	6	0.2654	1	1
CdI_2	6	0.0058	1	1
Br_2Ca	6	0.0046	1	1
$InSe_2$	6	0.2659	1	1
$GeTe_2$	6	0.264	1	1
$SiTe_2$	6	0.2501	1	1
I_2Pr	6	0.1294	1	1
I_2Mn	6	0.2655	1	1
Br_2Cu	6	0.6852	1	1
NSr_2	6	0.2606	1	1
PbS_2	6	0.2548	1	1
BiClTe	6	0.0055	1	1
Cl_2Zn	6	0.1156	1	1
FeI_2	6	0.2624	1	1
I_2Ni	6	0.2642	1	1
CrI_2	6	0.2618	1	1
I_2Nd	6	0.1301	1	1
$NiTe_2$	6	0.2494	1	1
Cl_2Cu	6	0.0707	1	1
GeI_2	6	0.0073	1	1
Se_2Zr	6	0.2507	1	1
BiITe	6	0.0066	1	1
CoI_2	6	0.259	1	1
CeI_2	6	0.1289	1	1
Br_2Mg	6	0.2622	1	1
I_2Ti	6	0.2594	1	1
GdI_2	6	0.0029	1	1
F_2Ni	6	0.1139	1	1
I_2La	6	0.1336	1	1
F_2Na	6	0.2473	1	1
CdI_2	6	0.0062	1	1
Se_2Sn	6	0.2603	1	1
F_2Zn	6	0.1267	1	1
Gd	6	0.1769	1	3
I_2Pr	6	0.0054	1	1
H_2Si_2	7	0.2664	1	1
Bi_2Te_2	7	0.4581	1	1
Fe_2Te_2	7	0.1213	1	1
Ca_2Cl_2	7	0.1216	1	1
Cu_2I_2	7	0.1473	1	1
Cl_2Gd_2	7	0.2724	1	1
Cu_2Sr_2	7	0.001	1	1
Cl_2OOs	7	0.1133	1	1

Table showing the first 50 lattice matching combinations with the smallest number of atoms in the supercell.

Lattice matching - minimal strain

Formula	N° atoms	strain	cell size 1	cell size 2
As_2	147	0.0	25	36
Br_2Zn	300	0.0	43	57
$PtSe_2$	255	0.0	36	49
I_2Ni	492	0.0001	73	91
Li_2Tl_2	291	0.0001	49	36
$NiTe_2$	339	0.0001	49	64
CCl_2Gd_2	414	0.0001	43	57
Nd	139	0.0001	25	64
I_2Ti	435	0.0001	64	81
NbS_2	294	0.0001	37	61
CCl_2Lu_2	255	0.0001	25	36
O_2Zn	354	0.0001	39	79
Cl_2Ni	123	0.0002	16	25
Cl_4Mn	435	0.0002	65	48
CoI_2	435	0.0002	64	81
$GeTe_2$	492	0.0003	73	91
$NbSe_2$	123	0.0003	16	25
In_2S_3	743	0.0003	81	100
Te_2Ti	300	0.0003	43	57
Br_2Fe	183	0.0003	25	36
Se_2Ta	123	0.0003	16	25
$HfSe_2$	300	0.0004	43	57
Mg_3	519	0.0004	65	108
CoH_2O_2	320	0.0004	25	49
Cl_2NSc_2	552	0.0004	49	81
H_2Li_2Pt	926	0.0004	82	136
Br_2Co	183	0.0004	25	36
$IrTe_2$	543	0.0004	81	100
PbS_2	390	0.0005	57	73
GeTe	443	0.0005	81	100
CaClHO	403	0.0005	49	64
Ni_2Te_2	583	0.0005	73	91
CI_2Y_2	597	0.0005	64	81
Ca_2N	183	0.0005	25	36
S_2Ta	294	0.0006	37	61
$AsSn_2$	255	0.0006	36	49
Ga_2Se_2	643	0.0006	81	100
FeH_2O_2	647	0.0006	49	100
$LiNbS_2$	355	0.0006	37	61
CBr_2Y_2	414	0.0006	43	57
$Cl_2O_2Tm_2$	531	0.0007	49	64
AsSb	354	0.0007	64	81
$SiTe_2$	339	0.0007	49	64
Br_2Y_2	463	0.0007	57	73
Se_2Sn	435	0.0007	64	81
$CdCl_2$	492	0.0007	73	91
$Gd_2I_2Se_2$	42	0.0008	6	4
S_2Sn_2	390	0.0008	58	54
H_2Si_2	643	0.0009	81	100
I_2Mn	492	0.0009	73	91

Table showing the first 50 lattice matching combinations with the smallest strain needed to build the supercell.

BiTeCl (P3m1)

Structural and electronic properties

**Formula**	BiTeCl
**Spacegroup**	P3m1
**Prototype**	BiTeI
**Parent 3D**	$Bi_2Te_2Cl_2$
**Source DB**	ICSD
**DB ID**	79362
**DF2-C09 Binding energy [meV/ Å^2]**	16.37
**RVV10 Binding energy [meV/ Å^2]**	23.69
**Band gap (PBE) [eV]**	1.84

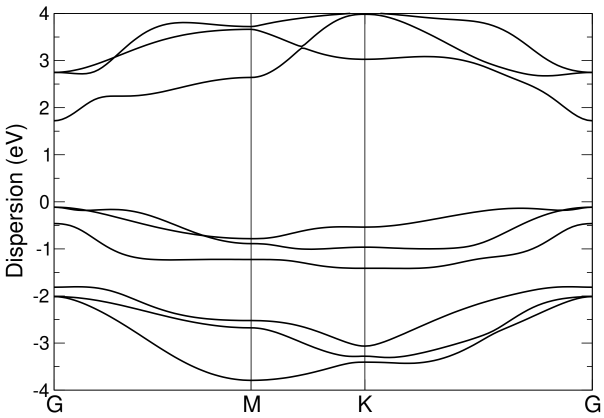

Band structure: Electronic band structure of BiTeCl (P3m1) in a representative window around the Fermi energy and along a high-symmetry path.

Optimized crystal structure

Structural parameters: relaxed cell (top) and atomic positions (bottom) of BiTeCl (P3m1) in Cartesian coordinates.

		x [Å]	y [Å]	z [Å]
$\mathbf{a_1}$		4.27980647	0.00000000	0.00000000
$\mathbf{a_2}$		−2.13990323	3.70642112	0.00000000
$\mathbf{a_3}$		0.00000000	0.00000000	23.47438975
		x [Å]	y [Å]	z [Å]
●	Bi	2.13990323	1.23547371	11.67902269
●	Te	0.00000000	2.47094742	13.46069513
●	Cl	0.00000000	0.00000000	10.04678868

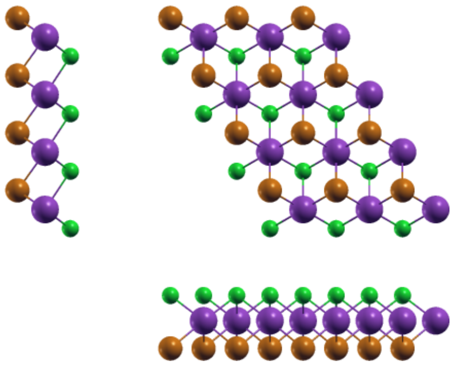

Orthographic projections: views of BiTeCl (P3m1) as seen from the x axis (left), the y axis (bottom) and the z axis (center).

Lattice matching - minimal size

Formula	N° atoms	strain	cell size 1	cell size 2
Tl	4	4.852	1	1
InSe	5	0.0038	1	1
AsSb	5	0.2662	1	1
Bi_2	5	0.0019	1	1
GeTe	5	0.275	1	1
AgTl	5	0.1488	1	1
PbTe	5	0.0021	1	1
CaCl	5	0.112	1	1
$CdCl_2$	6	0.2734	1	1
CdI_2	6	0.0004	1	1
PSn_2	6	1.5553	1	1
Br_2Zn	6	1.634	1	1
Br_2Ca	6	0.0009	1	1
$InSe_2$	6	0.274	1	1
$AsSn_2$	6	1.5827	1	1
$GeTe_2$	6	0.272	1	1
$SiTe_2$	6	0.2576	1	1
I_2Pr	6	0.1335	1	1
I_2Mn	6	0.2736	1	1
S_2Zr	6	1.5498	1	1
NSr_2	6	0.2685	1	1
PbS_2	6	0.2625	1	1
Cl_2Zn	6	0.1182	1	1
FeI_2	6	0.2704	1	1
I_2Ni	6	0.2722	1	1
Te_2Ti	6	1.6363	1	1
CrI_2	6	0.2697	1	1
BaF_2	6	0.0076	1	1
BiBrTe	6	0.0056	1	1
$RhTe_2$	6	0.2467	1	1
Se_2V	6	4.8702	1	1
AsKSn	6	0.0089	1	1
I_2Nd	6	0.1342	1	1
$NiTe_2$	6	0.2568	1	1
Cl_2Cu	6	0.0688	1	1
S_2Sn	6	1.5516	1	1
I_2V	6	0.2595	1	1
GeI_2	6	0.0018	1	1
Se_2Zr	6	0.2582	1	1
STl_2	6	0.0066	1	1
$PtSe_2$	6	1.5875	1	1
CoI_2	6	0.2668	1	1
GeS_2	6	0.4242	1	1
$MnSe_2$	6	0.1119	1	1
Br_2Ni	6	1.5253	1	1
CeI_2	6	0.1329	1	1
Br_2Mg	6	0.2702	1	1
I_2Ti	6	0.2673	1	1
$NbTe_2$	6	1.5483	1	1
Cl_2Mg	6	1.5256	1	1

Table showing the first 50 lattice matching combinations with the smallest number of atoms in the supercell.

Lattice matching - minimal strain

Formula	N° atoms	strain	cell size 1	cell size 2
Te_2W	183	0.0	25	36
I_2Pr	6	0.0001	1	1
I_2V	435	0.0001	64	81
Ga_2Se_2	403	0.0001	49	64
$MoTe_2$	183	0.0001	25	36
Bi_2In_2	387	0.0002	65	48
$NbTe_2$	255	0.0002	36	49
CI_2Y_2	743	0.0002	81	100
I_2Ti	543	0.0002	81	100
$Cl_2O_2Tm_2$	609	0.0002	57	73
Se_2V	390	0.0002	49	81
Gd_2GeI_2	8	0.0002	1	1
NS_2Ta	433	0.0003	39	79
$Br_2Hf_2N_2$	474	0.0003	50	54
Te_2Zn	183	0.0003	25	36
CdI_2	6	0.0004	1	1
CaClHO	463	0.0004	57	73
Se_2Sn	543	0.0004	81	100
$Cu_2Rb_2Te_2$	483	0.0004	65	48
S_2Zr	255	0.0004	36	49
FHOZn	91	0.0004	9	16
CdH_2O_2	255	0.0004	25	36
$Cl_2Ho_2O_2$	678	0.0005	64	81
S_2Ta	294	0.0005	37	61
$CrSe_2$	75	0.0005	9	16
CoI_2	543	0.0006	81	100
Pd_2S_4	423	0.0006	69	36
F_2Na	390	0.0006	57	73
PTe_2Zr_2	674	0.0006	73	91
$HfLiS_2$	219	0.0006	25	36
NSr_2	543	0.0006	81	100
$Cl_2O_2Yb_2$	609	0.0006	57	73
S_2Sn	255	0.0007	36	49
$PtSe_2$	300	0.0007	43	57
Br_2La_2P	8	0.0007	1	1
Cl_2V	75	0.0007	9	16
CdClHO	357	0.0007	43	57
CdI_2	6	0.0008	1	1
Se_2Zr	435	0.0008	64	81
$F_2Se_2Y_2$	561	0.0008	73	57
Cl_2Y_2	403	0.0008	49	64
$FeSe_2$	603	0.0009	70	131
Br_2Ca	6	0.0009	1	1
Se_2V	294	0.0009	37	61
$I_2N_2Zr_2$	531	0.0009	49	64
$NiTe_2$	390	0.001	57	73
CrS_2	222	0.001	25	49
$Cl_2Er_2O_2$	678	0.001	64	81
AsSb	443	0.001	81	100
$Br_2Ho_2S_2$	747	0.001	101	74

Table showing the first 50 lattice matching combinations with the smallest strain needed to build the supercell.

BiTeI (P3m1)

Structural and electronic properties

**Formula**	BiTeI
**Spacegroup**	P3m1
**Prototype**	BiTeI
**Parent 3D**	BiTeI
**Source DB**	COD
**DB ID**	1523008
**DF2-C09 Binding energy [meV/ Å^2]**	15.31
**RVV10 Binding energy [meV/ Å^2]**	21.92
**Band gap (PBE) [eV]**	1.58

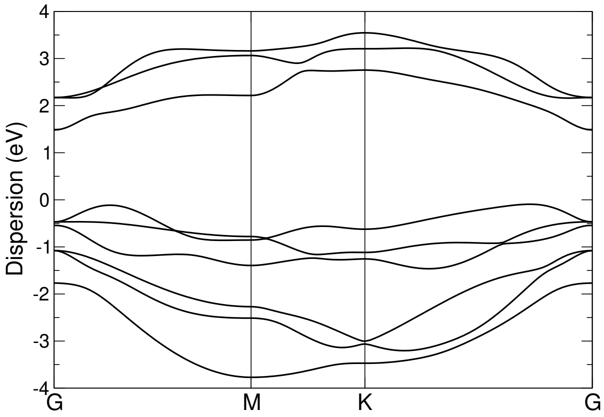

Band structure: Electronic band structure of BiTeI (P3m1) in a representative window around the Fermi energy and along a high-symmetry path.

Optimized crystal structure

Structural parameters: relaxed cell (top) and atomic positions (bottom) of BiTeI (P3m1) in Cartesian coordinates.

	x [Å]	y [Å]	z [Å]
$\mathbf{a_1}$	4.39068384	0.00000000	0.00000000
$\mathbf{a_2}$	−2.19534192	3.80244374	0.00000000
$\mathbf{a_3}$	0.00000000	0.00000000	23.89731099

		x [Å]	y [Å]	z [Å]
●	Te	-0.00000000	2.53496250	13.86204824
●	Bi	0.00000000	0.00000000	12.13969434
●	I	2.19534192	1.26748125	10.05451543

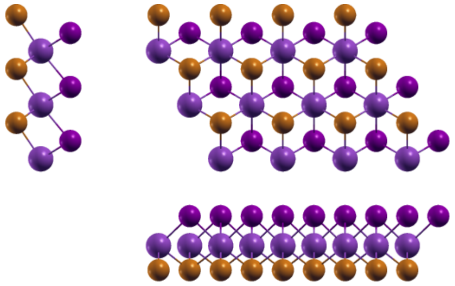

Orthographic projections: views of BiTeI (P3m1) as seen from the x axis (left), the y axis (bottom) and the z axis (center).

Lattice matching - minimal size

Formula	N° atoms	strain	cell size 1	cell size 2
AsSb	5	0.2495	1	1
GeTe	5	0.2577	1	1
S_2	5	0.2599	1	1
CaCl	5	0.4242	1	1
$IrTe_2$	6	0.259	1	1
$CdCl_2$	6	0.2562	1	1
Br_2Zn	6	1.5451	1	1
$InSe_2$	6	0.2568	1	1
$GeTe_2$	6	0.2549	1	1
$HfTe_2$	6	0.2708	1	1
I_2Pr	6	0.125	1	1
I_2Mn	6	0.2564	1	1
NSr_2	6	1.6322	1	1
I_2Yb	6	0.0079	1	1
Cl_2Zn	6	0.1129	1	1
FeI_2	6	0.2534	1	1
I_2Ni	6	0.2551	1	1
S_2Ti	6	4.8673	1	1
Te_2Ti	6	1.5472	1	1
BiBrTe	6	0.0064	1	1
Ba_2Hg	6	0.151	1	1
Cl_2Co	6	4.8599	1	1
Cl_2Fe	6	4.8445	1	1
Ba_2N	6	0.2731	1	1
Te_2Zr	6	0.2715	1	1
I_2Nd	6	0.1257	1	1
I_2Tm	6	0.0093	1	1
I_2V	6	1.5847	1	1
CoI_2	6	0.2501	1	1
$MnSe_2$	6	0.4241	1	1
CeI_2	6	0.1246	1	1
Cl_2Zr	6	4.8528	1	1
Br_2Mg	6	1.6409	1	1
I_2Ti	6	0.2506	1	1
GdI_2	6	0.0036	1	1
F_2Ni	6	0.1114	1	1
I_2La	6	0.1288	1	1
Se_2Sn	6	1.6304	1	1
F_2Zn	6	0.1226	1	1
Ba_2Cd	6	0.1535	1	1
NaPSn	6	0.2695	1	1
$HfSe_2$	6	1.5474	1	1
H_2Si_2	7	0.2572	1	1
Fe_2Te_2	7	0.1178	1	1
Li_2Tl_2	7	2.9096	1	1
Bi_2In_2	7	0.2092	1	1
Ca_2Cl_2	7	0.118	1	1
Cu_2I_2	7	0.1415	1	1
Cl_2Gd_2	7	0.263	1	1
Cu_2Sr_2	7	0.0055	1	1

Table showing the first 50 lattice matching combinations with the smallest number of atoms in the supercell.

Lattice matching - minimal strain

Formula	N° atoms	strain	cell size 1	cell size 2
$Cl_2O_2Y_2$	471	0.0	43	57
Te_2Ti	255	0.0	36	49
Ga_2Se_2	516	0.0001	64	81
BN	146	0.0001	16	49
$HfSe_2$	255	0.0001	36	49
Bi_2Te_3	8	0.0001	1	1
$BrKO_3$	317	0.0001	64	25
HN_3OZn	123	0.0001	9	16
H_2MnO_2	57	0.0001	4	9
$Br_2N_2Zr_2$	291	0.0001	25	36
CBr_2Lu_2	255	0.0002	25	36
Cl_2Tb_2	583	0.0002	73	91
AsSb	275	0.0002	49	64
I_2Ni	390	0.0002	57	73
$IrTe_2$	435	0.0002	64	81
Br_2Zn	255	0.0003	36	49
I_2Y_2	583	0.0003	73	91
Li_2Tl_2	343	0.0004	57	43
Ni_2Te_2	463	0.0004	57	73
S_2Ti	390	0.0004	49	81
CCl_2Gd_2	353	0.0004	36	49
S_2	354	0.0004	64	81
$GeTe_2$	390	0.0004	57	73
PTe_2Zr_2	467	0.0005	49	64
Ga_2Se_2	357	0.0005	43	57
$CdCl_2$	390	0.0006	57	73
Ni_2SbTe_2	597	0.0006	64	81
$CrTe_2$	123	0.0006	16	25
Se_2Ta_4	951	0.0006	81	118
CoI_2	339	0.0007	49	64
$Cl_2Hg_2N_2$	732	0.0007	114	65
Cl_2Sc_2	148	0.0007	16	25
$Cl_2Ho_2O_2$	471	0.0007	43	57
I_2Mn	390	0.0007	57	73
Cl_2Co	390	0.0007	49	81
CrO_2	267	0.0007	25	64
$GeNi_3Te_2$	843	0.0007	81	100
$Cl_2Er_2H_2$	609	0.0008	57	73
$Br_2Cr_2O_2$	33	0.0008	3	4
CdO_2	390	0.0009	49	81
Br_2Cr	123	0.0009	16	25
Br_2Y_2	357	0.0009	43	57
$LiMnSe_2$	583	0.0009	73	91
Cl_2Gd_2	583	0.0009	73	91
Br_2Pr_2	304	0.0009	36	49
In_2S_3	597	0.0009	64	81
CBr_2Y_2	353	0.0009	36	49
$InSe_2$	390	0.001	57	73
KNO_3	47	0.001	9	4
$Br_2H_2Zr_2$	198	0.001	16	25

Table showing the first 50 lattice matching combinations with the smallest strain needed to build the supercell.

C (P6/mmm)

Structural and electronic properties

**Formula**	C
**Spacegroup**	P6/mmm
**Prototype**	Graphite(2H)
**Parent 3D**	C_2
**Source DB**	COD
**DB ID**	9000046
**DF2-C09 Binding energy [meV/ Å^2]**	20.29
**RVV10 Binding energy [meV/ Å^2]**	25.45
**Band gap (PBE) [eV]**	0.0

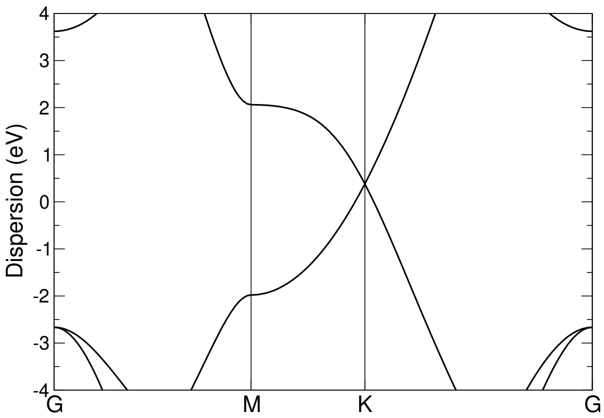

Band structure: Electronic band structure of C (P6/mmm) in a representative window around the Fermi energy and along a high-symmetry path.

Optimized crystal structure

Structural parameters: relaxed cell (top) and atomic positions (bottom) of C (P6/mmm) in Cartesian coordinates.

	x [Å]	y [Å]	z [Å]
$\mathbf{a_1}$	2.46596975	−0.00000000	0.00000000
$\mathbf{a_2}$	−1.23298487	2.13559245	0.00000000
$\mathbf{a_3}$	0.00000000	0.00000000	20.00000021

		x [Å]	y [Å]	z [Å]
•	C	-0.00000000	1.42372830	10.00000021
•	C	-0.00000000	0.00000000	10.00000000

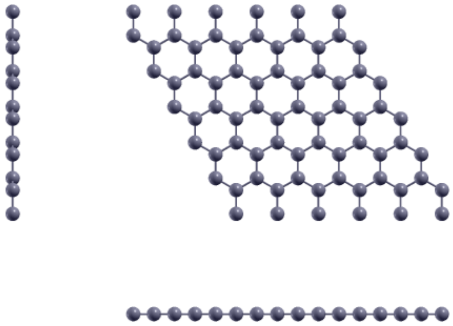

Orthographic projections: views of C (P6/mmm) as seen from the x axis (left), the y axis (bottom) and the z axis (center).

Lattice matching - minimal size

Formula	N° atoms	strain	cell size 1	cell size 2
Sn	3	0.3116	1	1
In	3	0.3206	1	1
BN	4	0.0084	1	1
K	5	0.5682	2	1
FeO_2	5	0.4946	1	1
Br_2Cu	5	1.0098	1	1
NiO_2	5	0.4981	1	1
CrO_2	5	0.4648	1	1
MnO_2	5	0.4705	1	1
N_2Re	5	2.9608	1	1
CoO_2	5	0.4966	1	1
InSe	6	6.2671	1	2
AgTl	6	0.4545	2	1
N_4	6	1.5954	1	1
Ba_2Hg	7	0.5241	2	1
$CrTe_2$	7	0.2259	2	1
Br_2Ti	7	0.2251	2	1
$AsSe_2$	7	0.2198	2	1
Cl_2Cu	7	0.1774	2	1
BrNZr	7	0.2215	2	1
$NbSe_2$	7	0.2185	2	1
Br_2Cr	7	0.2255	2	1
Se_2Ta	7	0.2186	2	1
H_2MgO_2	7	13.6508	1	1
$NbSe_2$	7	0.2202	2	1
Se_2Ta	7	0.2241	2	1
$AgCuTe_2$	8	0.2761	2	1
S_2Sn_2	8	0.4891	2	1
BrCdI	8	6.075	1	2
Br_2Hf_2	8	0.221	2	1
BaF_2	8	6.1633	1	2
AsKSn	8	6.125	1	2
$PbTe_2$	8	6.0491	1	2
STl_2	8	6.1889	1	2
Ge_2Se_2	8	0.4559	2	1
PbS_2Sn	8	0.5545	2	1
Cl_2Sc_2	8	0.2258	2	1
Se_2Sn_2	8	0.5589	2	1
F_4Sn	9	0.5218	2	1
Sn	9	0.139	4	1
Fe_2Te_2	10	0.1252	3	1
Ca_2Cl_2	10	0.1255	3	1
$Br_2H_2Zr_2$	10	0.2254	2	1
HgO	10	0.1457	3	2
$Cu_2Na_2Te_2$	10	0.579	2	1
$AsLi_3$	10	6.2753	1	2
Pb_2Se_2	10	8.8761	1	2
$Cl_2N_2Zr_2$	10	0.3483	2	1
Br_2Ca_3Si	10	0.5248	2	1
AgTl	10	0.1091	4	1

Table showing the first 50 lattice matching combinations with the smallest number of atoms in the supercell.

Lattice matching - minimal strain

Formula	N° atoms	strain	cell size 1	cell size 2
$Ga_2Ge_2Te_2$	104	0.0	25	9
$Hf_2I_2N_2$	42	0.0001	9	4
CdClHO	34	0.0001	9	4
Li_2P_2Pr	95	0.0001	25	9
$CrTe_2$	275	0.0002	79	39
Sb_2Te_3	178	0.0003	49	16
KNO_3	173	0.0003	64	9
Br_2V	173	0.0003	49	25
FeO_2	275	0.0003	64	49
CS_2Ta_2	77	0.0003	16	9
Cl_2Sc_2	314	0.0003	79	39
Cu_4Te_2	194	0.0003	49	16
Mg_6	198	0.0004	39	20
Cl_2Ti	59	0.0004	16	9
$TaTe_2$	30	0.0004	9	4
$Ge_2I_2La_2$	312	0.0005	81	25
CoO_2	275	0.0005	64	49
Br_2Cr	275	0.0005	79	39
$F_2Se_2Tm_2$	278	0.0005	64	25
P_2	50	0.0005	16	9
$ReSe_2$	173	0.0005	49	25
$Br_2H_2Zr_2$	392	0.0006	79	39
HN_3OZn	86	0.0006	16	9
$LiMnSe_2$	228	0.0006	64	25
Sb_2	68	0.0007	25	9
$Br_2S_2Y_2$	398	0.0007	115	28
Br_2Ti	275	0.0008	79	39
H_2MgO_2	407	0.0008	81	49
Au_2Br_2	240	0.0008	76	22
$H_2Li_2O_2$	584	0.0008	130	54
CrO_2	401	0.0008	91	73
$SnTe_2$	77	0.0009	25	9
In	159	0.0009	61	37
Br_2La_2	86	0.0009	25	9
NiO_2	275	0.001	64	49
$Se_2Si_2Zr_2$	518	0.001	115	48
$AsSn_2$	30	0.001	9	4
$I_2S_2Tb_2$	408	0.001	120	28
CNb_2S_2	77	0.001	16	9
Cl_2Zr_2	198	0.001	49	25
$H_2Na_2O_2$	416	0.0011	88	40
BH_4Li	104	0.0011	25	9
I_2Y_2	228	0.0012	64	25
$Cl_2H_2Sc_2$	392	0.0013	79	39
$Br_2Ho_2O_2$	510	0.0014	123	44
Fe_2O_4	518	0.0014	103	52
Bi_2SeTe_2	178	0.0014	49	16
Br_2Zr_2	396	0.0014	100	49
MnO_2	401	0.0014	91	73
H_2MgO_2	307	0.0014	61	37

Table showing the first 50 lattice matching combinations with the smallest strain needed to build the supercell.

C_2F_2 (P-3m1)

Structural and electronic properties

**Formula**	C_2F_2
**Spacegroup**	P-3m1
**Prototype**	PtTe
**Parent 3D**	C_2F_2
**Source DB**	MPDS
**DB ID**	S1504243
**DF2-C09 Binding energy [meV/ Å^2]**	11.02
**RVV10 Binding energy [meV/ Å^2]**	N/A
**Band gap (PBE) [eV]**	3.14

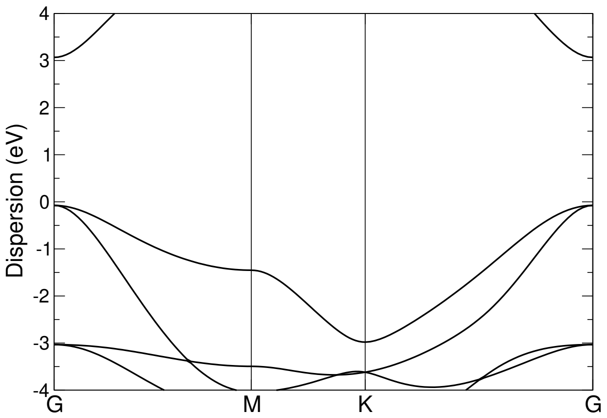

Band structure: Electronic band structure of C_2F_2 (P-3m1) in a representative window around the Fermi energy and along a high-symmetry path.

Optimized crystal structure

Structural parameters: relaxed cell (top) and atomic positions (bottom) of C_2F_2 (P-3m1) in Cartesian coordinates.

		x [Å]	y [Å]	z [Å]
$\mathbf{a_1}$		1.30275764	−2.25644242	0.00000000
$\mathbf{a_2}$		1.30275764	2.25644242	0.00000000
$\mathbf{a_3}$		0.00000000	0.00000000	18.50017142
		x [Å]	y [Å]	z [Å]
•	C	-0.65137882	-0.37607374	-0.24420247
•	C	0.65137882	0.37607374	0.24420247
•	F	-0.65137882	-0.37607374	-1.62515422
•	F	0.65137882	0.37607374	1.62515422

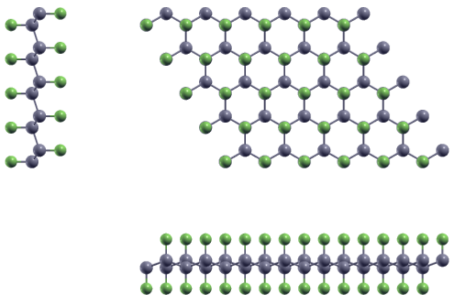

Orthographic projections: views of C_2F_2 (P-3m1) as seen from the x axis (left), the y axis (bottom) and the z axis (center).

Lattice matching - minimal size

Formula	N° atoms	strain	cell size 1	cell size 2
Sm	5	0.0015	1	1
CNRb	7	2.9543	1	1
O_2Zn	7	3.0008	1	1
N_2Re	7	0.4473	1	1
$HNiO_2$	8	0.4931	1	1
H_2MnO_2	9	0.4746	1	1
N_3W_2	9	0.4685	1	1
FeH_2O_2	9	2.9707	1	1
CaI_2	10	6.2742	1	2
I_2Yb	10	6.2055	1	2
I_2Tm	10	6.2422	1	2
PSn_2	11	0.221	2	1
$AsSn_2$	11	0.2259	2	1
S_2Zr	11	0.22	2	1
S_2Sn	11	0.2204	2	1
$TaTe_2$	11	0.2252	2	1
$NbTe_2$	11	0.2198	2	1
CdClHO	12	0.2244	2	1
Cu_2Te_2	12	0.588	2	1
C_2Li_2	12	0.3224	2	1
Ho_2S_2	12	1.9225	2	1
Ba_2Cu_2	12	6.2008	1	2
AsI_2La_2	14	6.0766	1	2
$Br_2Cr_2S_2$	14	0.1067	2	1
GeI_2La_2	14	6.2352	1	2
Cd_2I_3	14	6.0774	1	2
AgTl	14	0.1285	3	1
$C_2Br_2Gd_2$	14	0.2547	2	1
$Hf_2I_2N_2$	14	0.2244	2	1
$Cl_2O_2Ti_2$	14	0.5908	2	1
$C_2Br_2Tb_2$	14	0.2547	2	1
I_2Pr	15	0.1177	3	1
Cl_2Zn	15	0.4276	3	1
Mg_3	15	0.1566	3	1
Ba_2Hg	15	0.1393	3	1
I_2Nd	15	0.1182	3	1
CeI_2	15	0.1173	3	1
F_2Zn	15	0.1158	3	1
Ba_2Cd	15	0.1415	3	1
Fe_2Te_2	16	0.1122	3	1
Ca_2Cl_2	16	0.1124	3	1
Cu_2I_2	16	0.1312	3	1
Cl_2OOs	16	0.4231	3	1
Ir_2P_2	16	0.1181	3	1
S_2Sn_2	16	0.1396	3	1
Au_2Br_2	16	0.1388	3	1
Cu_2Te_2	16	0.4335	3	1
O_2Pb_2	16	0.1288	3	1
O_2Sn_2	16	0.594	2	2
Ge_2Se_2	16	0.1351	3	1

Table showing the first 50 lattice matching combinations with the smallest number of atoms in the supercell.

Lattice matching - minimal strain

Formula	N° atoms	strain	cell size 1	cell size 2
$Cl_2H_2Lu_2$	346	0.0	49	25
$AsSe_2$	91	0.0	16	9
Ni_2SbTe_2	56	0.0001	9	4
$LiOS_2Ti$	569	0.0001	81	49
CdClHO	596	0.0001	100	49
$Cl_2H_2Zr_2$	466	0.0001	61	37
$AsSn_2$	433	0.0002	79	39
$NbSe_2$	91	0.0002	16	9
Gd	377	0.0002	76	73
S_2	44	0.0002	9	4
Br_2OV	188	0.0003	32	15
O_2Zn	304	0.0003	49	36
Se_2Ta_4	276	0.0003	39	20
Cl_2Mg	271	0.0003	49	25
Cu_2Sr_2	136	0.0003	25	9
CNRb	558	0.0003	117	30
Br_2Ni	271	0.0004	49	25
Cu_2Te_2	464	0.0004	80	36
NbS_2	355	0.0004	61	37
$PbTe_2$	331	0.0004	64	25
$PtSe_2$	433	0.0005	79	39
PTe_2Ti_2	321	0.0005	49	25
H_2MnO_2	644	0.0005	81	64
I_2La_2P	145	0.0006	25	9
Br_2Ca_3Si	292	0.0006	49	16
N_3W_2	729	0.0006	91	73
Hg_3N_2	301	0.0007	64	9
I_2La_2Te	276	0.0007	49	16
Ga_2Se_2	52	0.0007	9	4
P_2Sn_2	52	0.0007	9	4
$TaTe_2$	433	0.0007	79	39
$LiOS_2Ti$	429	0.0008	61	37
Br_2Gd_2Ge	381	0.0008	64	25
Cu_2I_2	356	0.0008	64	25
Ga_2S_2	296	0.0008	49	25
$F_2Se_2Yb_2$	60	0.0008	9	4
Br_2Hf_2	100	0.0009	16	9
NS_2Ta	400	0.0009	57	43
$HNiO_2$	452	0.0009	64	49
$RhTe_2$	547	0.0009	100	49
$IrTe_2$	48	0.0009	9	4
Se_2Ta	91	0.001	16	9
$MnNaTe_2$	356	0.001	64	25
$NbSe_2$	91	0.0011	16	9
S_2Zn_2	52	0.0011	9	4
Gd_2I_2	356	0.0011	64	25
$Cl_2O_2Ti_2$	536	0.0013	80	36
S_2Ta	355	0.0013	61	37
$Gd_2I_2Se_2$	272	0.0013	50	12
BrNZr	91	0.0013	16	9

Table showing the first 50 lattice matching combinations with the smallest strain needed to build the supercell.

CLu_2Br_2 (P-3m1)

Structural and electronic properties

**Formula**	CLu_2Br_2
**Spacegroup**	P-3m1
**Prototype**	Bi2Te2S
**Parent 3D**	CBr_2Lu_2
**Source DB**	MPDS
**DB ID**	S1708510
**DF2-C09 Binding energy [meV/ Å^2]**	13.5
**RVV10 Binding energy [meV/ Å^2]**	N/A
**Band gap (PBE) [eV]**	0.87

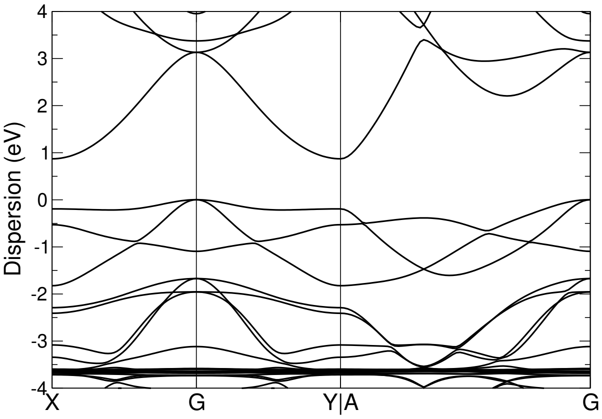

Band structure: Electronic band structure of CLu_2Br_2 (P-3m1) in a representative window around the Fermi energy and along a high-symmetry path.

Optimized crystal structure

Structural parameters: relaxed cell (top) and atomic positions (bottom) of CLu_2Br_2 (P-3m1) in Cartesian coordinates.

		x [Å]	y [Å]	z [Å]
$\mathbf{a_1}$		1.83015549	−3.16992230	0.00000000
$\mathbf{a_2}$		1.83017618	3.16993424	0.00000000
$\mathbf{a_3}$		0.00000000	0.00000000	24.71568477
		x [Å]	y [Å]	z [Å]
●	Lu	-0.91508121	-0.52832238	1.28962779
●	Br	0.91508219	0.52832295	3.20935491
●	Lu	0.91508121	0.52832238	-1.28962779
●	Br	-0.91508219	-0.52832295	-3.20935491
●	C	0.91507775	-1.58496115	0.00000000

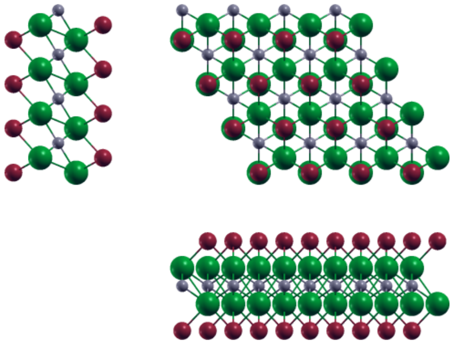

Orthographic projections: views of CLu_2Br_2 (P-3m1) as seen from the x axis (left), the y axis (bottom) and the z axis (center).

Lattice matching - minimal size

Formula	N° atoms	strain	cell size 1	cell size 2
Tl	6	0.1174	1	1
Tl	6	0.2732	1	1
HgO	7	0.1241	1	1
As_2	7	0.0066	1	1
LiO	7	0.2549	1	1
P_2	7	0.2655	1	1
Mg_2	7	0.4437	1	1
Sb_2	7	0.4719	1	1
Cl_2Zn	8	0.0076	1	1
I_2Mg	8	0.4865	1	1
$AgTe_2$	8	0.1189	1	1
PSn_2	8	0.0022	1	1
$MoSe_2$	8	0.2721	1	1
HfS_2	8	0.0039	1	1
$AsSn_2$	8	0.006	1	1
$CuTe_2$	8	0.0043	1	1
S_2Zr	8	0.0014	1	1
Br_2La	8	0.4874	1	1
Br_2Co	8	0.007	1	1
Ca_2N	8	0.0061	1	1
Cl_2Ti	8	0.2657	1	1
$AuTe_2$	8	0.4523	1	1
BrCdI	8	0.4948	1	1
Mg_3	8	0.1147	1	1
I_2Zn	8	0.4636	1	1
$RhTe_2$	8	0.0089	1	1
S_2W	8	0.2462	1	1
Bi_2Pd	8	0.1304	1	1
GeI_2	8	0.4817	1	1
Br_2Mn	8	0.0088	1	1
$CoTe_2$	8	0.0036	1	1
Se_2V	8	0.2744	1	1
AsKSn	8	0.4998	1	1
$PbTe_2$	8	2.8538	1	1
S_2Sn	8	0.0017	1	1
$SnTe_2$	8	0.4759	1	1
$PtSe_2$	8	0.0067	1	1
Br_2Fe	8	0.007	1	1
GeS_2	8	0.1539	1	1
$TaTe_2$	8	0.0054	1	1
Br_2Ni	8	0.002	1	1
$NbTe_2$	8	0.0012	1	1
Se_2Yb	8	0.4823	1	1
MoS_2	8	0.2464	1	1
Cl_2Mg	8	0.0019	1	1
$BiTe_2$	8	0.483	1	1
$CrSe_2$	8	0.2499	1	1
$PtTe_2$	8	0.4513	1	1
Se_2W	8	0.2723	1	1
CdClHO	9	0.0049	1	1

Table showing the first 50 lattice matching combinations with the smallest number of atoms in the supercell.

Lattice matching - minimal strain

Formula	N° atoms	strain	cell size 1	cell size 2
$SnTe_2$	597	0.0	81	64
K	141	0.0001	25	16
$Br_2N_2Zr_2$	11	0.0001	1	1
H_2MnO_2	205	0.0001	16	25
$BiTe_2$	536	0.0001	73	57
Sm	174	0.0001	25	49
In_2Se_2	824	0.0001	100	81
Bi_2STe_2	425	0.0002	49	36
BiITe	255	0.0002	36	25
$F_2Lu_2Se_2$	893	0.0002	91	73
BH_4Li	789	0.0002	81	64
BrCdI	467	0.0002	64	49
CdI_2	353	0.0002	49	36
Bi_2Te_3	305	0.0002	36	25
Sb_2Se_2Te	650	0.0003	73	57
C_4Ca_2	957	0.0003	105	72
SSb_2Te_2	565	0.0003	64	49
$I_2S_2Sm_2$	221	0.0003	25	16
$Br_2O_2Pr_2$	613	0.0003	65	48
CNb_2S_2	820	0.0003	73	91
Se_2Yb	536	0.0003	73	57
Br_2La_2P	425	0.0004	49	36
S_2V	386	0.0004	43	57
LiO	431	0.0004	57	73
BaF_2	414	0.0004	57	43
I_2La_2Sb	205	0.0004	25	16
H_2MgO_2	500	0.0005	43	57
$LiMnTe_2$	389	0.0005	49	36
$Br_2Er_2S_2$	824	0.0005	100	54
$CrSe_2$	437	0.0005	49	64
CrO_2	93	0.0005	9	16
STl_2	414	0.0006	57	43
$Br_2S_2Yb_2$	455	0.0006	55	30
GeI_2	536	0.0006	73	57
$Br_2Lu_2S_2$	541	0.0006	65	36
$Ga_2I_2Y_2$	543	0.0006	57	43
$MnNaTe_2$	516	0.0006	64	49
$Br_2Lu_2S_2$	541	0.0006	65	36
FHOZn	501	0.0006	49	64
$Br_2Cr_2O_2$	570	0.0006	54	50
CdI_2	353	0.0007	49	36
Sb_2Se_2Te	650	0.0007	73	57
Ag_2Br_2	517	0.0007	65	48
La_2S_2	503	0.0008	67	42
Ba_2Ni_3	650	0.0008	73	57
Ga_2Te_2	593	0.0008	73	57
Gd_2GeI_2	425	0.0008	49	36
GeI_2	353	0.0008	49	36
$Ge_2Mn_2Sr_2$	613	0.0008	65	48
P_2	547	0.0008	73	91

Table showing the first 50 lattice matching combinations with the smallest strain needed to build the supercell.

CLu_2Cl_2 (P-3m1)

Structural and electronic properties

**Formula**	CLu_2Cl_2
**Spacegroup**	P-3m1
**Prototype**	Bi2Te2S
**Parent 3D**	CLu_2Cl_2
**Source DB**	ICSD
**DB ID**	62227
**DF2-C09 Binding energy [meV/ Å^2]**	12.38
**RVV10 Binding energy [meV/ Å^2]**	19.42
**Band gap (PBE) [eV]**	0.95

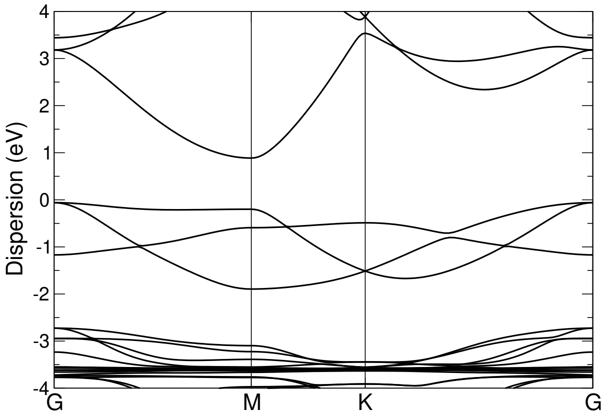

Band structure: Electronic band structure of CLu_2Cl_2 (P-3m1) in a representative window around the Fermi energy and along a high-symmetry path.

Optimized crystal structure

Structural parameters: relaxed cell (top) and atomic positions (bottom) of CLu_2Cl_2 (P-3m1) in Cartesian coordinates.

	x [Å]	y [Å]	z [Å]
$\mathbf{a_1}$	3.60766193	0.00000000	0.00000000
$\mathbf{a_2}$	−1.80383097	3.12432688	0.00000000
$\mathbf{a_3}$	0.00000000	0.00000000	26.10405056

		x [Å]	y [Å]	z [Å]
●	Lu	1.80383097	1.04144229	11.75151999
●	Cl	1.80383097	1.04144229	16.09122033
●	C	0.00000000	0.00000000	13.05202528
●	Lu	0.00000000	2.08288459	14.35253057
●	Cl	0.00000000	2.08288459	10.01283023

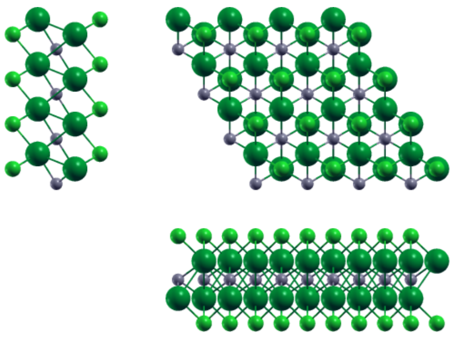

Orthographic projections: views of CLu_2Cl_2 (P-3m1) as seen from the x axis (left), the y axis (bottom) and the z axis (center).

Lattice matching - minimal size

Formula	N° atoms	strain	cell size 1	cell size 2
K	6	0.3109	1	1
Tl	6	0.1209	1	1
Sn	6	0.4227	1	1
Na	6	0.0077	1	1
In	6	0.4326	1	1
In	6	0.25	1	1
HgO	7	0.1285	1	1
As_2	7	0.0001	1	1
LiO	7	0.2644	1	1
P_2	7	0.2755	1	1
Mg_2	7	0.1132	1	1
Sb_2	7	2.8413	1	1
Cl_2Zn	8	0.0009	1	1
I_2Mg	8	2.9142	1	1
S_2V	8	0.2542	1	1
MoS_2	8	0.2552	1	1
$MoTe_2$	8	0.0055	1	1
$AgTe_2$	8	0.1227	1	1
PSn_2	8	0.0092	1	1
HfS_2	8	0.0029	1	1
$HfTe_2$	8	0.45	1	1
Te_2V	8	0.0036	1	1
$CuTe_2$	8	0.0025	1	1
S_2Zr	8	0.0084	1	1
Br_2La	8	2.9186	1	1
Br_2Co	8	0.0003	1	1
ReS_2	8	1.5581	1	1
Ca_2N	8	0.0007	1	1
Cl_2Ti	8	0.2758	1	1
$AuTe_2$	8	0.4693	1	1
$PdTe_2$	8	0.4632	1	1
Mg_3	8	0.1178	1	1
I_2Zn	8	0.4811	1	1
Te_2Zn	8	0.0057	1	1
S_2W	8	0.2553	1	1
Bi_2Pd	8	0.1356	1	1
GeI_2	8	0.4997	1	1
Br_2Mn	8	0.0021	1	1
PtS_2	8	0.0067	1	1
$CoTe_2$	8	0.0032	1	1
CdClO	8	0.0041	1	1
Ba_2N	8	0.4538	1	1
Se_2Ti	8	0.0092	1	1
AsKSn	8	2.9802	1	1
Te_2Zr	8	0.4512	1	1
Te_2W	8	0.0053	1	1
Cl_2Cu	8	0.5802	1	1
S_2Sn	8	0.0087	1	1
$SnTe_2$	8	0.4937	1	1
Cl_2V	8	0.2609	1	1

Table showing the first 50 lattice matching combinations with the smallest number of atoms in the supercell.

Lattice matching - minimal strain

Formula	N° atoms	strain	cell size 1	cell size 2
I_2Mg	414	0.0	57	43
$Ga_2I_2Y_2$	461	0.0	49	36
NaO_4	745	0.0	100	49
Li_2Tl_2	345	0.0	49	25
MoS_2	504	0.0	57	73
FHOZn	644	0.0	64	81
$CrSe_2$	563	0.0001	64	81
LiO	547	0.0001	73	91
S_2W	504	0.0001	57	73
BiBrTe	255	0.0001	36	25
Br_2Cd	743	0.0001	100	81
As_2	7	0.0001	1	1
$Dy_2I_2S_2$	221	0.0001	25	16
NS_2Ta	269	0.0002	25	36
MoS_2	504	0.0002	57	73
BaF_2	353	0.0002	49	36
I_2Pr	469	0.0002	65	48
Nd	61	0.0002	9	16
$I_2Pr_2S_2$	699	0.0002	81	49
Br_2Fe	8	0.0002	1	1
$Ce_2I_2Si_2$	543	0.0002	57	43
Cl_4Mn	295	0.0002	39	20
Ag_2	379	0.0003	61	37
Br_2Co	8	0.0003	1	1
$CeLi_2P_2$	565	0.0003	64	49
Br_2PY_2	905	0.0003	100	81
Br_2La	414	0.0003	57	43
$I_2O_2Yb_2$	613	0.0003	65	48
Cl_2La_2	661	0.0004	81	64
BH_4Li	614	0.0004	64	49
Bi_2Se_3	500	0.0004	57	43
Br_2Ho_2	747	0.0004	91	73
$I_2Pr_2S_2$	527	0.0004	61	37
CoH_2O_2	425	0.0005	36	49
DyI_2	173	0.0005	25	16
In	309	0.0005	49	64
Ba_2Pt	416	0.0006	61	37
$PtTe_2$	674	0.0006	91	73
Sb_2Se_2Te	500	0.0006	57	43
Gd_2I_2	457	0.0006	57	43
$SnTe_2$	467	0.0006	64	49
Bi_2S_3	820	0.0006	91	73
I_2Pb	552	0.0007	81	49
Ca_2N	8	0.0007	1	1
$F_2Lu_2Se_2$	707	0.0007	73	57
Ir_2P_2	517	0.0007	65	48
Br_2HLa	516	0.0007	64	49
$Br_2Eu_2O_2$	613	0.0008	65	48
$PdTe_2$	743	0.0008	100	81
I_2Zn	536	0.0008	73	57

Table showing the first 50 lattice matching combinations with the smallest strain needed to build the supercell.

$Ca(OH)_2$ (P-3m1)

Structural and electronic properties

**Formula**	$Ca(OH)_2$
**Spacegroup**	P-3m1
**Prototype**	Mg(OH)2
**Parent 3D**	$Ca(OH)_2$
**Source DB**	COD
**DB ID**	9000113
**DF2-C09 Binding energy [meV/ Å^2]**	21.74
**RVV10 Binding energy [meV/ Å^2]**	27.43
**Band gap (PBE) [eV]**	3.71

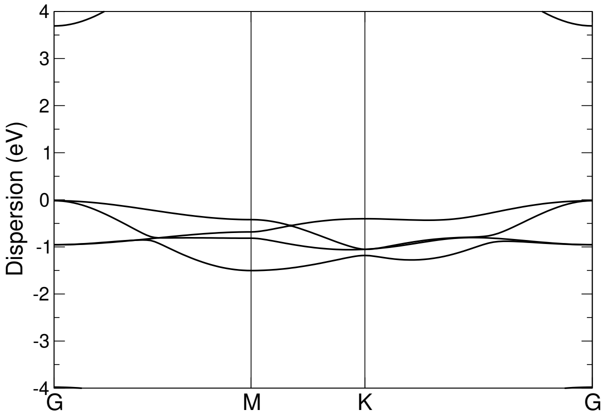

Band structure: Electronic band structure of $Ca(OH)_2$ (P-3m1) in a representative window around the Fermi energy and along a high-symmetry path.

Optimized crystal structure

Structural parameters: relaxed cell (top) and atomic positions (bottom) of $Ca(OH)_2$ (P-3m1) in Cartesian coordinates.

	x [Å]	y [Å]	z [Å]
$\mathbf{a_1}$	3.62341352	−0.00000000	0.00000000
$\mathbf{a_2}$	−1.81170676	3.13796816	0.00000000
$\mathbf{a_3}$	0.00000000	0.00000000	24.24486899

		x [Å]	y [Å]	z [Å]
•	H	1.81170676	1.04598939	10.01935193
•	O	1.81170676	1.04598939	10.98652768
•	Ca	0.00000000	0.00000000	12.12243449
•	H	-0.00000000	2.09197877	14.22551706
•	O	-0.00000000	2.09197877	13.25834131

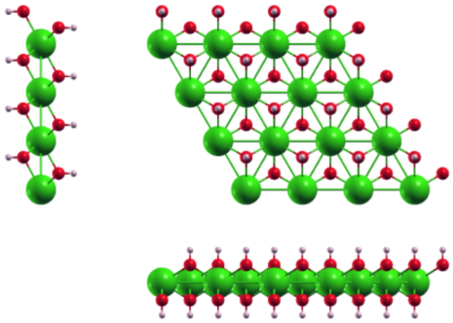

Orthographic projections: views of $Ca(OH)_2$ (P-3m1) as seen from the x axis (left), the y axis (bottom) and the z axis (center).

Lattice matching - minimal size

Formula	N° atoms	strain	cell size 1	cell size 2
Tl	6	0.1198	1	1
In	6	0.428	1	1
In	6	0.2473	1	1
InSe	7	3.0233	1	1
HgO	7	0.1271	1	1
As_2	7	0.0019	1	1
LiO	7	0.2615	1	1
P_2	7	0.2725	1	1
Mg_2	7	0.1124	1	1
Sb_2	7	0.4842	1	1
Cl_2Zn	8	0.0029	1	1
I_2Mg	8	0.4992	1	1
S_2V	8	1.631	1	1
MoS_2	8	1.6365	1	1
$MoTe_2$	8	0.0075	1	1
$AgTe_2$	8	0.1215	1	1
PSn_2	8	0.0071	1	1
HfS_2	8	0.0008	1	1
Te_2V	8	0.0056	1	1
$CuTe_2$	8	0.0004	1	1
S_2Zr	8	0.0063	1	1
Br_2La	8	0.5001	1	1
Br_2Cu	8	1.0369	1	1
Ca_2Si	8	13.6791	1	1
Br_2Co	8	0.0023	1	1
ReS_2	8	1.5432	1	1
Ca_2N	8	0.0014	1	1
Cl_2Ti	8	0.2727	1	1
$AuTe_2$	8	0.4641	1	1
$PdTe_2$	8	0.458	1	1
Mg_3	8	0.1168	1	1
I_2Zn	8	0.4758	1	1
Te_2Zn	8	0.0077	1	1
S_2W	8	1.637	1	1
Bi_2Pd	8	0.134	1	1
GeI_2	8	0.4943	1	1
Br_2Mn	8	0.0041	1	1
PtS_2	8	0.0087	1	1
$CoTe_2$	8	0.0011	1	1
CdClO	8	0.0061	1	1
Ba_2N	8	0.4487	1	1
Te_2W	8	0.0073	1	1
S_2Sn	8	0.0066	1	1
Cl_2V	8	0.258	1	1
STl_2	8	2.9846	1	1
OTl_2	8	0.0059	1	1
Br_2Fe	8	0.0023	1	1
Br_2Ni	8	0.0028	1	1
$NbTe_2$	8	0.0061	1	1
Se_2Yb	8	0.4949	1	1

Table showing the first 50 lattice matching combinations with the smallest number of atoms in the supercell.

Lattice matching - minimal strain

Formula	N° atoms	strain	cell size 1	cell size 2
I_2La_2P	305	0.0	36	25
N_2W	233	0.0	25	36
Sb_2Te_2	601	0.0	81	49
Br_2Gd_2	747	0.0	91	73
$BiTe_2$	467	0.0001	64	49
$PbTe_2$	414	0.0001	57	43
I_2Zn	597	0.0001	81	64
As_2Sn_2	747	0.0001	91	73
Br_2La_2	593	0.0002	73	57
CoO_2	368	0.0002	37	61
Br_2Ho_2	824	0.0002	100	81
In_2Te_3	425	0.0002	49	36
Se_2Yb	467	0.0002	64	49
$I_2O_2Tm_2$	613	0.0002	65	48
$Cl_2N_2Zr_2$	11	0.0002	1	1
NaO_4	590	0.0003	79	39
Sb_2SeTe_2	425	0.0003	49	36
$I_2N_2Zr_2$	564	0.0003	60	44
FeO_2	488	0.0003	49	81
$CrSe_2$	563	0.0003	64	81
NiO_2	368	0.0004	37	61
Sb_2	479	0.0004	73	57
Sb_2Se_2Te	565	0.0004	64	49
Bi_2S_3	905	0.0004	100	81
GeI_2	467	0.0004	64	49
O_2Pt	386	0.0004	43	57
$CuTe_2$	8	0.0004	1	1
ReS_2	327	0.0005	36	49
As_2O_3	65	0.0005	9	4
Br_2Er_2	747	0.0005	91	73
$AgNO_3$	590	0.0006	79	39
$Br_2Eu_2F_2$	613	0.0006	65	48
La_2S_2	365	0.0006	49	30
Sb_2Te_2	453	0.0006	61	37
Bi_2Se_2Te	425	0.0006	49	36
Ba_2Ni_3	565	0.0006	64	49
$Er_2I_2O_2$	613	0.0006	65	48
Ga_2Te_2	516	0.0006	64	49
Br_2Tb_2	824	0.0007	100	81
$CrSe_2$	504	0.0007	57	73
$MnNaTe_2$	457	0.0007	57	43
$Al_2Cl_2O_2$	533	0.0007	49	48
AlH_4Na	315	0.0007	39	20
$Cl_2O_2Ti_2$	825	0.0007	81	70
$I_2Pr_2Si_2$	614	0.0007	64	49
I_2Pb	416	0.0008	61	37
$Br_2Hf_2N_2$	11	0.0008	1	1
FHOZn	577	0.0008	57	73
$PtTe_2$	743	0.0008	100	81
H_2MgO_2	565	0.0008	49	64

Table showing the first 50 lattice matching combinations with the smallest strain needed to build the supercell.

$Ca_2Br_2F_2$ (P4/nmm)

Structural and electronic properties

**Formula**	$Ca_2Br_2F_2$
**Spacegroup**	P4/nmm
**Prototype**	PbClF
**Parent 3D**	$Br_2Ca_2F_2$
**Source DB**	MPDS
**DB ID**	S1900226
**DF2-C09 Binding energy [meV/ Å^2]**	14.72
**RVV10 Binding energy [meV/ Å^2]**	N/A
**Band gap (PBE) [eV]**	5.29

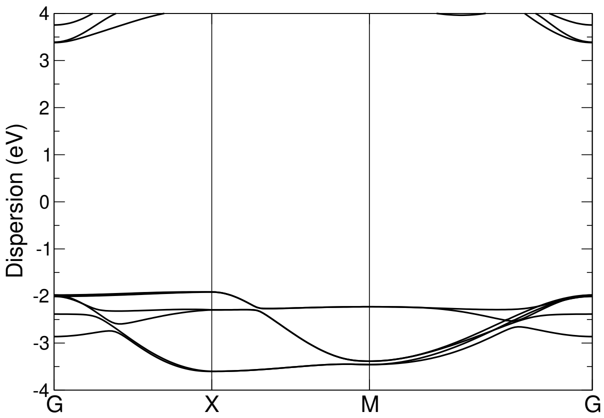

Band structure: Electronic band structure of $Ca_2Br_2F_2$ (P4/nmm) in a representative window around the Fermi energy and along a high-symmetry path.

Optimized crystal structure

Structural parameters: relaxed cell (top) and atomic positions (bottom) of $Ca_2Br_2F_2$ (P4/nmm) in Cartesian coordinates.

	x [Å]	y [Å]	z [Å]
$\mathbf{a_1}$	3.87037288	−0.00000017	0.00000000
$\mathbf{a_2}$	−0.00000017	3.87037288	0.00000000
$\mathbf{a_3}$	0.00000000	0.00000000	22.70956275

	x [Å]	y [Å]	z [Å]
Ca	0.96759348	-0.96759348	1.34028309
Br	-0.96759334	-2.90277937	2.77251628
Ca	-0.96759332	-2.90277939	-1.34028309
Br	0.96759351	-0.96759351	-2.77251628
F	-0.96759273	-0.96759273	0.00000000
F	0.96759290	-2.90278015	0.00000000

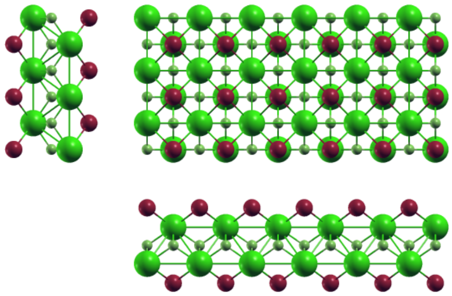

Orthographic projections: views of $Ca_2Br_2F_2$ (P4/nmm) as seen from the x axis (left), the y axis (bottom) and the z axis (center).

Lattice matching - minimal size

Formula	N° atoms	strain	cell size 1	cell size 2
K	7	0.3864	1	1
K	7	0.1896	1	1
In	7	0.1111	1	1
InSe	8	0.1496	1	1
Bi_2	8	0.1553	1	1
Ag_2	8	0.1968	1	1
LiO	8	0.1088	1	1
PbTe	8	0.1513	1	1
Sb_2	8	0.1357	1	1
I_2Mg	9	0.1404	1	1
S_2V	9	0.1104	1	1
MoS_2	9	0.1102	1	1
CdI_2	9	0.153	1	1
Nd	9	0.7725	1	3
PSn_2	9	0.1088	1	1
Ba_2Pt	9	0.1965	1	1
Br_2Ca	9	0.1542	1	1
CaI_2	9	0.178	1	1
I_2Pr	9	0.0044	1	1
S_2Zr	9	0.1086	1	1
Br_2La	9	0.1406	1	1
Br_2Cu	9	0.1075	1	1
I_2Yb	9	0.175	1	1
BiClTe	9	0.1534	1	1
$AuTe_2$	9	0.1298	1	1
BrCdI	9	0.1431	1	1
I_2Zn	9	0.1332	1	1
BaF_2	9	0.146	1	1
BiBrTe	9	0.159	1	1
S_2W	9	0.1102	1	1
Bi_2Pd	9	0.5637	1	1
GeI_2	9	0.1388	1	1
AsKSn	9	0.1447	1	1
$PbTe_2$	9	0.1422	1	1
I_2Nd	9	0.0053	1	1
Cl_2Cu	9	0.0995	1	1
I_2Tm	9	0.1766	1	1
S_2Sn	9	0.1087	1	1
$SnTe_2$	9	0.1369	1	1
Cl_2V	9	0.1093	1	1
GeI_2	9	0.1516	1	1
STl_2	9	0.1469	1	1
BiITe	9	0.1661	1	1
DyI_2	9	0.1815	1	1
CeI_2	9	0.0036	1	1
$NbTe_2$	9	0.1086	1	1
Se_2Yb	9	0.139	1	1
MoS_2	9	0.1102	1	1
$BiTe_2$	9	0.1392	1	1
GdI_2	9	0.162	1	1

Table showing the first 50 lattice matching combinations with the smallest number of atoms in the supercell.

Lattice matching - minimal strain

Formula	N° atoms	strain	cell size 1	cell size 2
$Cl_2S_2Tl_2$	780	0.0	81	49
$Cu_2K_2Te_2$	876	0.0002	85	61
Cl_4Cu_2	534	0.0002	64	25
Cl_2Rb_2	186	0.0003	25	9
$Cu_2Na_2Te_2$	882	0.0003	82	65
Se_2Ta_4	678	0.0003	49	64
CdClO	483	0.0003	48	65
F_2Zn	9	0.0004	1	1
$Br_2F_2Yb_2$	12	0.0004	1	1
$Cu_2Na_2Te_2$	870	0.0005	81	64
Ca_2O_2	412	0.0005	36	49
OTl_2	483	0.0005	48	65
$Cl_2Hf_2N_2$	678	0.0005	48	65
CdH_2O_2	613	0.0006	48	65
Pd_2S_4	708	0.0006	79	39
$AgClO_4$	246	0.0006	25	16
LiO_2	510	0.0006	49	72
F_4Pb	640	0.0006	65	50
Cl_2Zr_2	958	0.0006	81	118
$Br_2Gd_2O_2$	12	0.0006	1	1
$Ba_2F_2I_2$	510	0.0007	49	36
O_4PTl	510	0.0007	49	36
Te_2V	483	0.0008	48	65
Bi_2Pd	363	0.0008	36	49
Br_2V	840	0.0009	81	118
Br_2Cu_2	10	0.001	1	1
$HNiO_2$	276	0.001	20	39
Te_2W	483	0.001	48	65
Bi_2In_2	316	0.001	36	25
$Cl_2O_2V_2$	54	0.001	4	5
Mg_3	942	0.0011	89	136
$MoTe_2$	483	0.0011	48	65
$I_2Lu_2O_2$	12	0.0013	1	1
Te_2Zn	483	0.0013	48	65
$Cu_2Rb_2Te_2$	366	0.0013	36	25
$Br_2F_2Tm_2$	12	0.0014	1	1
$Br_2O_2Tb_2$	12	0.0014	1	1
MoS_2	711	0.0014	64	109
S_2W	711	0.0014	64	109
MoS_2	711	0.0014	64	109
I_2Tm	885	0.0015	103	89
FKO_2Se	700	0.0015	80	44
$AgNO_2$	848	0.0015	88	80
Cl_2Ti	852	0.0015	79	126
$Br_2O_2V_2$	66	0.0015	5	6
$Ca_2Ge_2Mn_2$	12	0.0015	1	1
Sn	655	0.0015	85	145
$CrTe_2$	531	0.0015	52	73
Cl_2Sc_2	604	0.0015	52	73
CuO_2	750	0.0016	70	110

Table showing the first 50 lattice matching combinations with the smallest strain needed to build the supercell.

Ca_2O_2 (P1)

Structural and electronic properties

**Formula**	Ca_2O_2
**Spacegroup**	P1
**Prototype**	FeSe
**Parent 3D**	Ca_2O_2
**Source DB**	ICSD
**DB ID**	160696
**DF2-C09 Binding energy [meV/ Å^2]**	4.13
**RVV10 Binding energy [meV/ Å^2]**	N/A
**Band gap (PBE) [eV]**	2.62

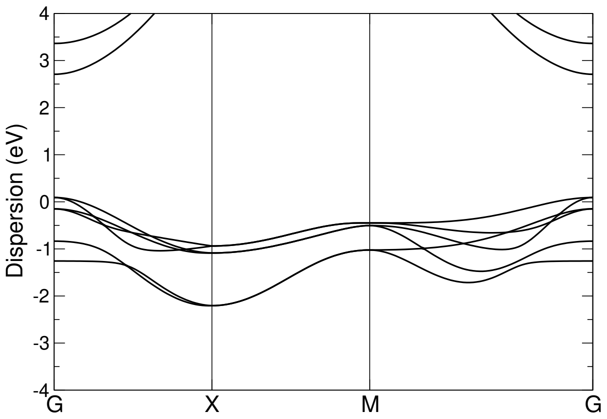

Band structure: Electronic band structure of Ca_2O_2 (P1) in a representative window around the Fermi energy and along a high-symmetry path.

Optimized crystal structure

Structural parameters: relaxed cell (top) and atomic positions (bottom) of Ca_2O_2 (P1) in Cartesian coordinates.

	x [Å]	y [Å]	z [Å]
$\mathbf{a_1}$	−3.32091153	0.00000000	0.00000000
$\mathbf{a_2}$	0.00000000	−3.32091153	0.00000000
$\mathbf{a_3}$	0.00000000	0.00000000	16.75685861

		x [Å]	y [Å]	z [Å]
●	Ca	-0.83022788	-0.83022788	1.19809328
●	Ca	-2.49068365	-2.49068365	-1.19809328
•	O	-0.83022788	-0.83022788	-1.16062944
•	O	-2.49068365	-2.49068365	1.16062944

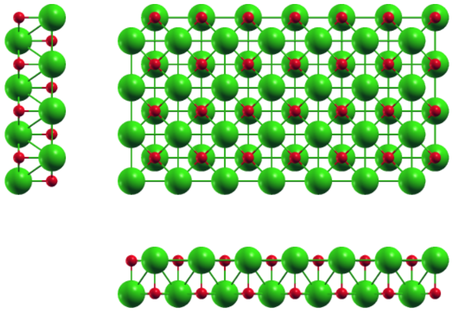

Orthographic projections: views of Ca_2O_2 (P1) as seen from the x axis (left), the y axis (bottom) and the z axis (center).

Lattice matching - minimal size

Formula	N° atoms	strain	cell size 1	cell size 2
Sn	5	0.213	1	1
Na	5	0.1386	1	1
In	5	0.218	1	1
In	5	0.109	1	1
AsSb	6	0.177	1	1
GeTe	6	0.1845	1	1
As_2	6	0.1455	1	1
S_2	6	0.1864	1	1
$IrTe_2$	7	0.1856	1	1
Cl_2Zn	7	0.1446	1	1
$CdCl_2$	7	0.1831	1	1
$MoTe_2$	7	0.1405	1	1
PSn_2	7	0.154	1	1
Br_2Zn	7	0.1652	1	1
HfS_2	7	0.1481	1	1
$InSe_2$	7	0.1836	1	1
$AsSn_2$	7	0.1579	1	1
$GeTe_2$	7	0.1819	1	1
$SiTe_2$	7	0.1699	1	1
$HfTe_2$	7	0.1965	1	1
Te_2V	7	0.1422	1	1
I_2Mn	7	0.1833	1	1
$CuTe_2$	7	0.1477	1	1
S_2Zr	7	0.1533	1	1
NSr_2	7	0.179	1	1
PbS_2	7	0.1739	1	1
Br_2Co	7	0.1451	1	1
Ca_2N	7	0.146	1	1
$AuTe_2$	7	0.7785	1	1
LiO_2	7	0.1091	1	1
Cl_2Zn	7	0.3884	1	1
$PdTe_2$	7	0.7691	1	1
FeI_2	7	0.1805	1	1
I_2Ni	7	0.1821	1	1
Te_2Ti	7	0.1656	1	1
CrI_2	7	0.18	1	1
Te_2Zn	7	0.1404	1	1
$RhTe_2$	7	0.1608	1	1
Bi_2Pd	7	0.0013	1	1
Br_2Mn	7	0.1435	1	1
Cl_2Ni	7	0.1298	1	1
$CrTe_2$	7	0.1343	1	1
PtS_2	7	0.1395	1	1
$CoTe_2$	7	0.1484	1	1
CdClO	7	0.1418	1	1
Ba_2N	7	0.7546	1	1
Se_2Ti	7	0.1373	1	1
Br_2Ti	7	0.1339	1	1
Te_2Zr	7	0.1972	1	1
Te_2W	7	0.1407	1	1

Table showing the first 50 lattice matching combinations with the smallest number of atoms in the supercell.

Lattice matching - minimal strain

Formula	N° atoms	strain	cell size 1	cell size 2
$Br_2Ca_2H_2$	962	0.0	113	85
$As_2Co_2Li_2$	708	0.0	81	64
$FeSe_2$	395	0.0001	50	65
Cu_2Se_2	732	0.0001	101	82
F_2Zn	304	0.0001	49	36
$Br_2Gd_2O_2$	412	0.0002	49	36
$Ca_2H_2I_2$	294	0.0002	36	25
$Ca_2Mn_2Si_2$	962	0.0002	113	85
$H_2I_2Sr_2$	736	0.0003	97	58
Sn	337	0.0003	64	81
$I_2S_2Tl_2$	896	0.0003	101	82
Cu_2Te_2	588	0.0003	82	65
$I_2O_2Tm_2$	706	0.0003	85	61
$Br_2O_2Yb_2$	550	0.0004	64	49
$Br_2Ho_2O_2$	962	0.0004	113	85
Cu_2Te_2	580	0.0004	81	64
$Br_2Ca_2F_2$	412	0.0005	49	36
$Cl_2S_2Tl_2$	60	0.0005	9	4
CrS_2	387	0.0005	48	65
$Ge_2Hf_2Te_2$	708	0.0006	81	64
Cu_2F_4	406	0.0006	64	25
Cu_2Se_2	724	0.0006	100	81
F_2Ni	650	0.0006	101	82
$Si_2Te_2Zr_2$	896	0.0006	101	82
I_2Nd	523	0.0007	85	61
Bi_2SeTe_2	877	0.0008	118	81
$Br_2O_2Sm_2$	706	0.0008	85	61
$I_2Lu_2O_2$	412	0.0008	49	36
$Br_2Er_2O_2$	962	0.0008	113	85
$Ag_2K_2Te_2$	754	0.0008	109	53
As_2Ir_2	244	0.0008	36	25
Fe_2Te_2	452	0.0008	64	49
$I_2Nd_2S_2$	822	0.0008	108	65
$I_2S_2Tl_2$	886	0.0009	100	81
$Br_2F_2Tm_2$	412	0.0009	49	36
Bi_2Se_2	524	0.0009	81	50
$Br_2F_2Yb_2$	412	0.0009	49	36
Sb_2Te_3	877	0.0009	118	81
Ir_2P_2	584	0.0009	85	61
$I_2S_2Tm_2$	280	0.001	40	20
$Br_2Eu_2F_2$	706	0.001	85	61
$Ca_2Ge_2Mn_2$	412	0.001	49	36
$Br_2Ce_2O_2$	294	0.001	36	25
$K_2Mn_2Sb_2$	294	0.001	36	25
NiO_2	694	0.0011	79	126
$Er_2I_2O_2$	706	0.0011	85	61
$I_2La_2O_2$	196	0.0011	25	16
$Ca_2Cl_2F_2$	560	0.0011	65	50
$FeSe_2$	388	0.0012	49	64
In	424	0.0012	81	100

Table showing the first 50 lattice matching combinations with the smallest strain needed to build the supercell.

CaHBr (P4/nmm)

Structural and electronic properties

**Formula**	CaHBr
**Spacegroup**	P4/nmm
**Prototype**	PbClF
**Parent 3D**	$Ca_2H_2Br_2$
**Source DB**	COD
**DB ID**	9009166
**DF2-C09 Binding energy [meV/ Å^2]**	18.74
**RVV10 Binding energy [meV/ Å^2]**	24.48
**Band gap (PBE) [eV]**	4.22

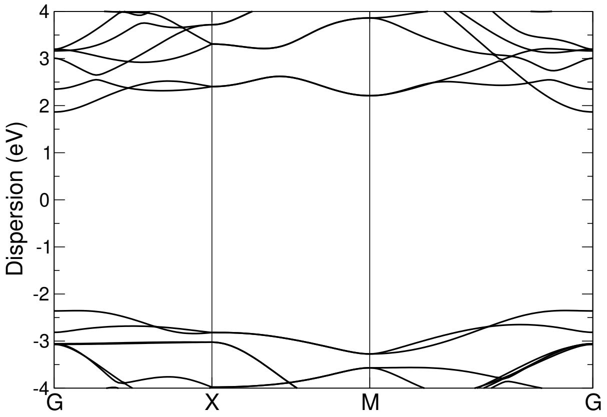

Band structure: Electronic band structure of CaHBr (P4/nmm) in a representative window around the Fermi energy and along a high-symmetry path.

Optimized crystal structure

Structural parameters: relaxed cell (top) and atomic positions (bottom) of CaHBr (P4/nmm) in Cartesian coordinates.

	x [Å]	y [Å]	z [Å]
$\mathbf{a_1}$	3.82915833	0.00000000	0.00000000
$\mathbf{a_2}$	0.00000000	3.82915833	0.00000000
$\mathbf{a_3}$	0.00000000	0.00000000	25.26239000

		x [Å]	y [Å]	z [Å]
●	Ca	1.91457916	0.00000000	11.33429547
●	Br	0.00000000	1.91457916	9.95488964
●	Ca	0.00000000	1.91457916	13.92809453
•	H	0.00000000	0.00000000	12.63119500
•	H	1.91457916	1.91457916	12.63119500
●	Br	1.91457916	0.00000000	15.30750036

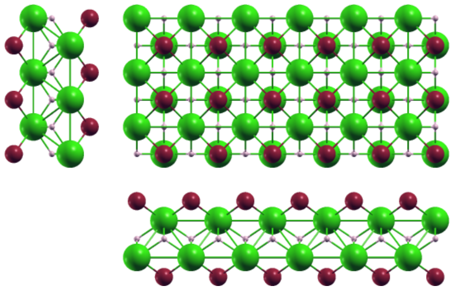

Orthographic projections: views of CaHBr (P4/nmm) as seen from the x axis (left), the y axis (bottom) and the z axis (center).

Lattice matching - minimal size

Formula	N° atoms	strain	cell size 1	cell size 2
K	7	0.3975	1	1
K	7	0.1962	1	1
In	7	0.11	1	1
InSe	8	0.1546	1	1
Bi_2	8	0.1606	1	1
Ag_2	8	0.7689	1	1
PbTe	8	0.1564	1	1
Sb_2	8	0.1398	1	1
I_2Mg	9	0.1448	1	1
S_2V	9	0.1093	1	1
MoS_2	9	0.1091	1	1
CdI_2	9	0.1581	1	1
Ba_2Pt	9	0.7679	1	1
Br_2Ca	9	0.1594	1	1
HfS_2	9	0.1086	1	1
CaI_2	9	0.1843	1	1
I_2Pr	9	0.0096	1	1
Br_2La	9	0.1451	1	1
Br_2Cu	9	0.1102	1	1
Ca_2Si	9	0.7854	1	1
I_2Yb	9	0.1811	1	1
BiClTe	9	0.1585	1	1
$AuTe_2$	9	0.1335	1	1
BrCdI	9	0.1476	1	1
$PdTe_2$	9	0.1317	1	1
HgI_2	9	1.1684	1	1
I_2Zn	9	0.1371	1	1
BaF_2	9	0.1508	1	1
BiBrTe	9	0.1644	1	1
S_2W	9	0.1091	1	1
GeI_2	9	0.1431	1	1
$CoTe_2$	9	0.1086	1	1
AsKSn	9	0.1494	1	1
$PbTe_2$	9	0.1467	1	1
Cl_2Cu	9	0.1009	1	1
I_2Tm	9	0.1828	1	1
$SnTe_2$	9	0.1412	1	1
GeI_2	9	0.1566	1	1
I_2Pb	9	0.775	1	1
STl_2	9	0.1517	1	1
BiITe	9	0.1718	1	1
DyI_2	9	0.1879	1	1
Br_2Ni	9	0.1091	1	1
CeI_2	9	0.0088	1	1
Se_2Yb	9	0.1433	1	1
MoS_2	9	0.1091	1	1
Cl_2Mg	9	0.1091	1	1
$BiTe_2$	9	0.1436	1	1
GdI_2	9	0.1676	1	1
$PtTe_2$	9	0.1332	1	1

Table showing the first 50 lattice matching combinations with the smallest number of atoms in the supercell.

Lattice matching - minimal strain

Formula	N° atoms	strain	cell size 1	cell size 2
Ca_2O_2	962	0.0	85	113
$Ba_2H_2I_2$	366	0.0001	36	25
Cl_4Cu_2	738	0.0001	89	34
Se_2Ti	483	0.0002	48	65
$Ba_2F_2I_2$	876	0.0002	85	61
$Ca_2Mn_2Si_2$	12	0.0002	1	1
HgO	536	0.0003	61	85
Br_2Zr_2	548	0.0004	48	65
$Br_2Ho_2O_2$	12	0.0004	1	1
$Ag_2K_2Se_2$	510	0.0005	49	36
In	375	0.0005	49	81
O_4PTl	876	0.0005	85	61
$CuGeO_3$	735	0.0006	70	63
C_2Li_2	246	0.0006	25	24
Cl_2Co	840	0.0006	81	118
Cl_2Zr	840	0.0007	81	118
C_4Ca_2	906	0.0008	81	70
S_2Ti	840	0.0008	81	118
H_2Li_2Pd	221	0.0008	16	25
$As_2Mg_2Na_2$	870	0.0008	81	64
$Br_2Er_2O_2$	12	0.0008	1	1
Bi_2Se_2	912	0.0009	98	81
Cl_2Fe	840	0.0009	81	118
$H_2I_2Sr_2$	882	0.001	82	65
HgI_2	402	0.0011	49	36
K	550	0.0011	81	64
Ge_2Te_2	712	0.0011	78	61
$AgTe_2$	258	0.0011	25	36
Au_2Br_2	914	0.0011	99	80
Mg_2	146	0.0012	16	25
Sn	445	0.0012	58	97
$AgNO_2$	602	0.0012	63	56
Pb_2Se_2	874	0.0012	97	73
H_4Ti	221	0.0013	16	25
Bi_2Pd	849	0.0013	85	113
$H_2Na_2O_2$	882	0.0013	65	82
C_4Ca_2	774	0.0013	69	60
$C_2I_2La_2$	138	0.0014	12	11
O_2Pt	711	0.0015	64	109
$AsSe_2$	531	0.0015	52	73
$Ag_2K_2Te_2$	246	0.0015	25	16
$NbSe_2$	531	0.0016	52	73
Mo_2Te_4	450	0.0016	45	30
P_2Rh_2	10	0.0016	1	1
$Gd_2I_2S_2$	690	0.0016	70	45
C_2	510	0.0016	44	123
Cd_2I_3	919	0.0016	89	77
CNRb	609	0.0016	77	49
Ba_2N	975	0.0016	106	113
$H_2Na_2O_2$	870	0.0017	64	81

Table showing the first 50 lattice matching combinations with the smallest strain needed to build the supercell.

CaHI (P4/nmm)

Structural and electronic properties

**Formula**	CaHI
**Spacegroup**	P4/nmm
**Prototype**	PbClF
**Parent 3D**	$Ca_2H_2I_2$
**Source DB**	COD
**DB ID**	9009167
**DF2-C09 Binding energy [meV/ Å^2]**	15.92
**RVV10 Binding energy [meV/ Å^2]**	20.92
**Band gap (PBE) [eV]**	3.74

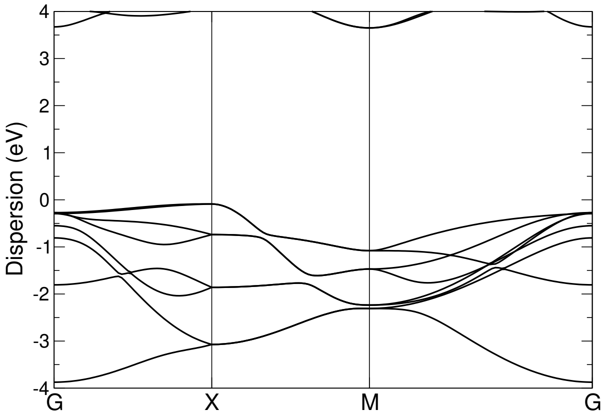

Band structure: Electronic band structure of CaHI (P4/nmm) in a representative window around the Fermi energy and along a high-symmetry path.

Optimized crystal structure

Structural parameters: relaxed cell (top) and atomic positions (bottom) of CaHI (P4/nmm) in Cartesian coordinates.

	x [Å]	y [Å]	z [Å]
$\mathbf{a_1}$	3.98649488	0.00000000	0.00000000
$\mathbf{a_2}$	0.00000000	3.98649488	0.00000000
$\mathbf{a_3}$	0.00000000	0.00000000	25.42636886

		x [Å]	y [Å]	z [Å]
●	Ca	0.00000000	1.99324744	13.94351752
●	I	1.99324744	0.00000000	15.60133151
●	Ca	1.99324744	0.00000000	11.48285133
•	H	0.00000000	0.00000000	12.71318443
•	H	1.99324744	1.99324744	12.71318443
●	I	0.00000000	1.99324744	9.82503735

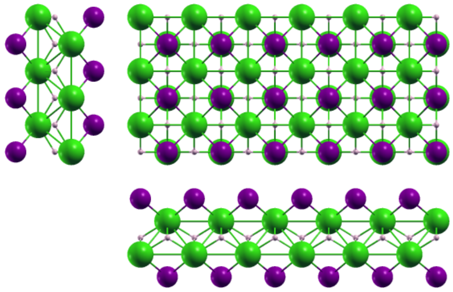

Orthographic projections: views of CaHI (P4/nmm) as seen from the x axis (left), the y axis (bottom) and the z axis (center).

Lattice matching - minimal size

Formula	N° atoms	strain	cell size 1	cell size 2
K	7	0.1724	1	1
Tl	7	0.109	1	1
InSe	8	0.1373	1	1
Bi_2	8	0.1422	1	1
AgTl	8	0.015	1	1
Ag_2	8	0.1789	1	1
P_2	8	0.1101	1	1
PbTe	8	0.1388	1	1
CaCl	8	0.2213	1	1
I_2Mg	9	0.1295	1	1
CdI_2	9	0.1402	1	1
Nd	9	0.1864	1	3
$MoSe_2$	9	0.1092	1	1
Ba_2Pt	9	0.1786	1	1
Br_2Ca	9	0.1412	1	1
CaI_2	9	0.1621	1	1
$SiTe_2$	9	0.109	1	1
Br_2La	9	0.1298	1	1
Br_2Cu	9	0.1017	1	1
Ca_2Si	9	0.1842	1	1
I_2Yb	9	0.1594	1	1
BiClTe	9	0.1405	1	1
Cl_2Ti	9	0.1101	1	1
BrCdI	9	0.1318	1	1
HgI_2	9	0.3933	1	1
BaF_2	9	0.1343	1	1
BiBrTe	9	0.1454	1	1
Se_2V	9	0.1089	1	1
AsKSn	9	0.1332	1	1
$PbTe_2$	9	0.1311	1	1
I_2Nd	9	0.0085	1	1
$NiTe_2$	9	0.1089	1	1
Cl_2Cu	9	0.097	1	1
I_2Tm	9	0.1608	1	1
GeI_2	9	0.139	1	1
I_2Pb	9	0.1808	1	1
STl_2	9	0.135	1	1
BiITe	9	0.1515	1	1
GeS_2	9	0.2077	1	1
$MnSe_2$	9	0.2212	1	1
DyI_2	9	0.1652	1	1
GdI_2	9	0.148	1	1
I_2La	9	0.0039	1	1
CdI_2	9	0.1398	1	1
I_2Pr	9	0.1406	1	1
Se_2W	9	0.1092	1	1
Cu_2Sr_2	10	0.1463	1	1
$LiMnTe_2$	10	0.1392	1	1
Cu_2Te_2	10	0.0553	1	1
Ir_2P_2	10	0.0087	1	1

Table showing the first 50 lattice matching combinations with the smallest number of atoms in the supercell.

Lattice matching - minimal strain

Formula	N° atoms	strain	cell size 1	cell size 2
$Se_2Si_2Zr_2$	882	0.0	65	82
HgI_2	687	0.0	82	65
Ca_2O_2	294	0.0002	25	36
Ag_2I_2	742	0.0003	81	64
$Cl_2O_2Sc_2$	90	0.0003	7	8
Mg_3	708	0.0003	65	106
Cl_2Rb_2	670	0.0004	89	34
$As_2Cd_2K_2$	690	0.0004	65	50
Fe_2S_2	886	0.0005	81	100
PSn_2	483	0.0005	48	65
Mg_2	800	0.0006	85	145
O_4PSn	690	0.0006	65	50
Na	604	0.0006	81	118
Ho_2S_2	866	0.0006	85	89
CNNa	972	0.0006	110	104
$Cl_2S_2Tl_2$	246	0.0007	25	16
CrS_2	237	0.0007	20	39
NS_2Zr	958	0.0007	81	118
O_4PSn	678	0.0007	64	49
Cu_2F_4	150	0.0007	16	9
$Se_2Si_2Zr_2$	870	0.0008	64	81
HgI_2	678	0.0008	81	64
C_2Li_2	244	0.0008	24	25
$AgTe_2$	171	0.0009	16	25
$Br_2Ce_2O_2$	12	0.0009	1	1
$K_2Mn_2Sb_2$	12	0.0009	1	1
H_2Na_2Pd	625	0.0009	50	65
$MnSe_2$	795	0.001	82	101
AlH_4Na	876	0.001	85	61
Se_2Ta_4	876	0.001	61	85
S_2Sn	483	0.001	48	65
As_2Ir_2	10	0.001	1	1
Bi_2Se_2	86	0.001	9	8
CaCl	694	0.0011	82	101
PtS_2	840	0.0011	81	118
$Cu_2K_2Te_2$	678	0.0012	64	49
S_2Zr	483	0.0013	48	65
Pb_2Se_2	602	0.0013	65	53
$Ag_2K_2Te_2$	366	0.0013	36	25
In	70	0.0013	9	16
$Eu_2I_2O_2$	12	0.0014	1	1
CNb_2S_2	929	0.0014	64	109
P_2	602	0.0014	64	109
$NbTe_2$	483	0.0015	48	65
Cl_2Ti	711	0.0015	64	109
Bi_2Pd	258	0.0015	25	36
CS_2Ta_2	929	0.0015	64	109
$MnSe_2$	786	0.0015	81	100
$As_2Cd_2K_2$	678	0.0015	64	49
$Br_2Hf_2N_2$	750	0.0015	52	73

Table showing the first 50 lattice matching combinations with the smallest strain needed to build the supercell.

CaI_2 (P-3m1)

Structural and electronic properties

**Formula**	CaI_2
**Spacegroup**	P-3m1
**Prototype**	CdI2
**Parent 3D**	CaI_2
**Source DB**	COD
**DB ID**	9009097
**DF2-C09 Binding energy [meV/ Å^2]**	9.94
**RVV10 Binding energy [meV/ Å^2]**	15.29
**Band gap (PBE) [eV]**	3.84

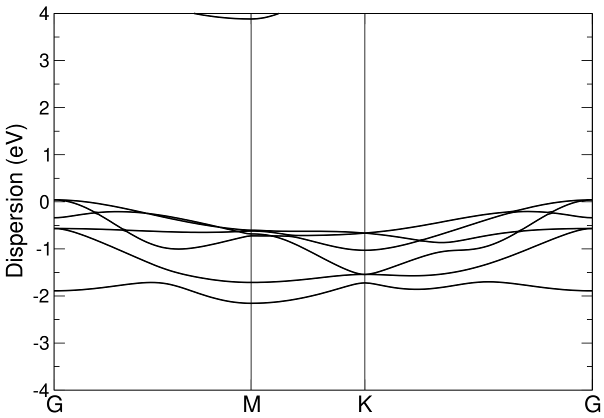

Band structure: Electronic band structure of CaI_2 (P-3m1) in a representative window around the Fermi energy and along a high-symmetry path.

Optimized crystal structure

Structural parameters: relaxed cell (top) and atomic positions (bottom) of CaI_2 (P-3m1) in Cartesian coordinates.

	x [Å]	y [Å]	z [Å]
$\mathbf{a_1}$	4.48777269	−0.00000000	0.00000000
$\mathbf{a_2}$	−2.24388634	3.88652516	0.00000000
$\mathbf{a_3}$	0.00000000	0.00000000	23.56424925

		x [Å]	y [Å]	z [Å]
●	I	2.24388634	1.29550838	13.57789714
●	Ca	0.00000000	-0.00000000	11.78212462
●	I	-0.00000000	2.59101677	9.98635210

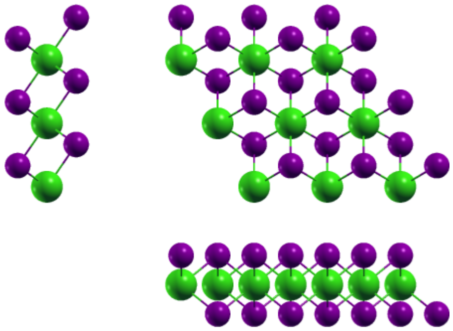

Orthographic projections: views of CaI_2 (P-3m1) as seen from the x axis (left), the y axis (bottom) and the z axis (center).

Lattice matching - minimal size

Formula	N° atoms	strain	cell size 1	cell size 2
K	4	0.0094	1	1
GeTe	5	1.5883	1	1
AgTl	5	0.1303	1	1
In	5	0.2197	1	2
$IrTe_2$	6	1.595	1	1
$CdCl_2$	6	1.5805	1	1
$InSe_2$	6	1.5832	1	1
$HfTe_2$	6	0.2562	1	1
I_2Pr	6	0.1191	1	1
I_2Mn	6	1.5813	1	1
NSr_2	6	1.556	1	1
I_2Yb	6	0.0025	1	1
$AuTe_2$	6	0.2671	1	1
Cl_2Zn	6	0.4336	1	1
$PdTe_2$	6	0.2636	1	1
FeI_2	6	1.5652	1	1
I_2Zn	6	0.2738	1	1
Ba_2Hg	6	0.1415	1	1
Ba_2N	6	0.2583	1	1
Te_2Zr	6	0.2569	1	1
I_2Nd	6	0.1196	1	1
I_2Tm	6	0.0012	1	1
BrNZr	6	4.855	1	1
CoI_2	6	1.5474	1	1
DyI_2	6	0.0028	1	1
CeI_2	6	0.1187	1	1
Br_2Mg	6	1.5643	1	1
F_2Ni	6	0.4245	1	1
$PtTe_2$	6	0.2665	1	1
Br_2Cd	6	0.2623	1	1
I_2La	6	0.1222	1	1
Se_2Sn	6	1.5542	1	1
F_2Zn	6	0.1171	1	1
Ba_2Cd	6	0.1438	1	1
NaPSn	6	0.255	1	1
H_2Si_2	7	1.5855	1	1
Fe_2Te_2	7	0.1132	1	1
Li_2Tl_2	7	0.4766	1	1
Ca_2Cl_2	7	0.1134	1	1
Cu_2I_2	7	0.1331	1	1
Cl_2Gd_2	7	0.2489	1	1
Cl_2OOs	7	0.1082	1	1
In_2Se_2	7	0.1792	1	1
Ir_2P_2	7	0.1195	1	1
Ag_2Br_2	7	0.1225	1	1
Br_2Er_2	7	0.2679	1	1
Cu_2S_2	7	0.1156	1	1
Au_2Br_2	7	0.1412	1	1
Ge_2Te_2	7	0.157	1	1
Br_2Tb_2	7	0.2645	1	1

Table showing the first 50 lattice matching combinations with the smallest number of atoms in the supercell.

Lattice matching - minimal strain

Formula	N° atoms	strain	cell size 1	cell size 2
CoI_2	255	0.0001	36	49
Br_2Tb_2	583	0.0001	73	91
Cl_2NSc_2	107	0.0001	9	16
$CrSe_2$	222	0.0001	25	49
Pt_2Te_2	463	0.0002	57	73
Br_2Mn	123	0.0002	16	25
Br_2Er_2	643	0.0002	81	100
Li_2Tl_2	499	0.0002	81	64
FHOZn	271	0.0002	25	49
$I_2N_2Zr_2$	291	0.0002	25	36
Cl_2Gd_2	403	0.0002	49	64
$Er_2F_2Se_2$	609	0.0003	57	73
NaPSn	390	0.0003	57	73
$AuTe_2$	543	0.0003	81	100
$AgNO_3$	386	0.0003	57	43
Bi_2S_3	674	0.0003	73	91
In_2S_3	414	0.0003	43	57
$IrTe_2$	300	0.0004	43	57
AsSb	206	0.0004	36	49
Se_2Ta	294	0.0004	37	61
I_2Ti	255	0.0004	36	49
O_2Pt	354	0.0005	39	79
Cl_2Tb_2	403	0.0005	49	64
$PdTe_2$	492	0.0005	73	91
$FeSe_2$	357	0.0005	39	80
Ga_2Se_2	357	0.0005	43	57
GeTe	243	0.0005	43	57
C_4Ca_2	312	0.0006	34	35
$HfTe_2$	390	0.0006	57	73
KS_2Ti	148	0.0006	16	25
Cl_2Mn	75	0.0006	9	16
In_2Se_3	536	0.0006	57	73
Br_2Ho_2	583	0.0006	73	91
$Cl_2H_2Sc_2$	477	0.0006	37	61
Ba_2N	435	0.0007	64	81
IO_3Tl	47	0.0007	9	4
$Br_2O_2V_2$	147	0.0007	13	18
$PtTe_2$	543	0.0007	81	100
Br_2Gd_2	643	0.0008	81	100
Ga_2Se_2	219	0.0008	25	36
$Cl_2O_2V_2$	129	0.0008	11	16
Pb_2Se_2	476	0.0008	72	65
CI_2Y_2	353	0.0008	36	49
As_2Sn_2	643	0.0009	81	100
HgO	411	0.0009	65	108
$C_2Br_2Gd_2$	168	0.0009	16	20
$F_2Ho_2Se_2$	678	0.0009	64	81
H_2Si_2	357	0.0009	43	57
$GeNi_3Te_2$	609	0.0009	57	73
Mg_2	472	0.0009	70	131

Table showing the first 50 lattice matching combinations with the smallest strain needed to build the supercell.

$Cd(OH)_2$ (P-3m1)

Structural and electronic properties

**Formula**	$Cd(OH)_2$
**Spacegroup**	P-3m1
**Prototype**	Mg(OH)2
**Parent 3D**	$Cd(OH)_2$
**Source DB**	ICSD
**DB ID**	165224
**DF2-C09 Binding energy [meV/ Å^2]**	29.17
**RVV10 Binding energy [meV/ Å^2]**	36.98
**Band gap (PBE) [eV]**	2.26

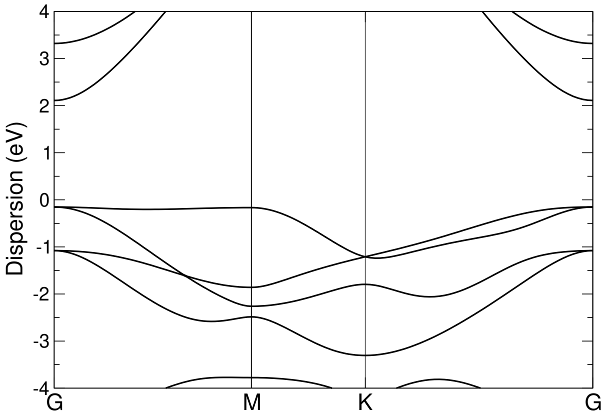

Band structure: Electronic band structure of $Cd(OH)_2$ (P-3m1) in a representative window around the Fermi energy and along a high-symmetry path.

Optimized crystal structure

Structural parameters: relaxed cell (top) and atomic positions (bottom) of $Cd(OH)_2$ (P-3m1) in Cartesian coordinates.

	x [Å]	y [Å]	z [Å]
$\mathbf{a_1}$	3.56987991	0.00000000	0.00000000
$\mathbf{a_2}$	−1.78493996	3.09160669	0.00000000
$\mathbf{a_3}$	0.00000000	0.00000000	24.16692344

	x [Å]	y [Å]	z [Å]
H	1.78493996	1.03053556	10.01409143
O	1.78493996	1.03053556	10.98266311
Cd	0.00000000	0.00000000	12.08346172
H	-0.00000000	2.06107113	14.15283201
O	-0.00000000	2.06107113	13.18426033

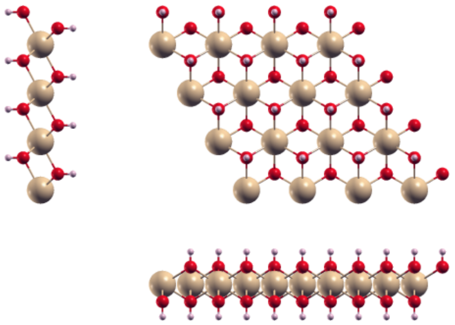

Orthographic projections: views of $Cd(OH)_2$ (P-3m1) as seen from the x axis (left), the y axis (bottom) and the z axis (center).

Lattice matching - minimal size

Formula	N° atoms	strain	cell size 1	cell size 2
K	6	0.321	1	1
Tl	6	0.1239	1	1
Sn	6	0.4339	1	1
Na	6	0.0028	1	1
In	6	0.1108	1	1
In	6	0.2567	1	1
HgO	7	0.1321	1	1
As_2	7	0.0052	1	1
LiO	7	0.2716	1	1
Mg_2	7	0.1153	1	1
Sb_2	7	2.9051	1	1
CrS_2	8	1.5507	1	1
Cl_2Zn	8	0.0041	1	1
I_2Mg	8	2.9796	1	1
S_2V	8	0.2611	1	1
MoS_2	8	0.2621	1	1
$MoTe_2$	8	0.0006	1	1
$AgTe_2$	8	0.1258	1	1
HfS_2	8	0.008	1	1
$HfTe_2$	8	0.4623	1	1
Te_2V	8	0.0013	1	1
$CuTe_2$	8	0.0076	1	1
Br_2La	8	2.9841	1	1
Br_2Cu	8	1.0726	1	1
Br_2Co	8	0.0047	1	1
ReS_2	8	1.5944	1	1
Ca_2N	8	0.0057	1	1
$AuTe_2$	8	0.482	1	1
$PdTe_2$	8	0.4758	1	1
Mg_3	8	0.1204	1	1
I_2Zn	8	0.4941	1	1
Te_2Zn	8	0.0008	1	1
S_2W	8	0.2622	1	1
Bi_2Pd	8	0.1396	1	1
Br_2Mn	8	0.0029	1	1
$CrTe_2$	8	0.0081	1	1
PtS_2	8	0.0018	1	1
$CoTe_2$	8	0.0083	1	1
CdClO	8	0.0008	1	1
Ba_2N	8	0.4661	1	1
Se_2Ti	8	0.0044	1	1
Br_2Ti	8	0.0086	1	1
Te_2Zr	8	0.4635	1	1
Te_2W	8	0.0004	1	1
Cl_2Cu	8	0.5953	1	1
Cl_2V	8	0.2679	1	1
OTl_2	8	0.001	1	1
Br_2Fe	8	0.0048	1	1
Br_2Cr	8	0.0084	1	1
MoS_2	8	0.2624	1	1

Table showing the first 50 lattice matching combinations with the smallest number of atoms in the supercell.

Lattice matching - minimal strain

Formula	N° atoms	strain	cell size 1	cell size 2
Ba_2Cu_2	189	0.0	25	16
$Ga_2Ge_2Te_2$	543	0.0	57	43
$PdTe_2$	597	0.0001	81	64
Br_2Er_2	593	0.0001	73	57
Li_2P_2Pr	500	0.0001	57	43
I_2Yb	173	0.0002	25	16
$Br_2Hf_2N_2$	38	0.0002	4	3
BN	353	0.0002	39	79
H_2NiO_2	820	0.0002	73	91
$Er_2F_2Se_2$	986	0.0002	100	81
$Br_2F_2Yb_2$	613	0.0002	65	48
Cl_2V	705	0.0002	81	100
Pt_2Te_2	824	0.0003	100	81
ReS_2	386	0.0003	43	57
Ba_2N	674	0.0003	91	73
Br_2Gd_2Ge	425	0.0003	49	36
Cu_2I_2	389	0.0003	49	36
I_2Pr	255	0.0004	36	25
H_2MgO_2	725	0.0004	64	81
Br_2Ca	255	0.0004	36	25
$HNiO_2$	269	0.0004	25	36
Te_2W	8	0.0004	1	1
I_2La_2Sb	490	0.0004	61	37
BiClTe	255	0.0004	36	25
NaPSn	743	0.0004	100	81
$HfTe_2$	743	0.0004	100	81
$AuTe_2$	536	0.0004	73	57
Br_2Cu_2	517	0.0005	65	48
In_2Se_3	905	0.0005	100	81
Br_2Tb_2	661	0.0005	81	64
I_2Zn	467	0.0005	64	49
Bi_2In_2	275	0.0005	39	20
CrS_2	327	0.0005	36	49
Cu_3Se_3	893	0.0006	91	73
$Br_2Ca_2F_2$	613	0.0006	65	48
$MoTe_2$	8	0.0006	1	1
Cl_2OV	673	0.0006	77	72
Gd_2I_2	389	0.0006	49	36
ClH_3O	125	0.0006	16	9
Br_2Gd_2	593	0.0006	73	57
O_2Pt	504	0.0007	57	73
Gd_2GeI_2	305	0.0007	36	25
Sb_2	371	0.0007	57	43
Bi_2S_3	725	0.0007	81	64
As_2Sn_2	593	0.0007	73	57
CoH_2O_2	500	0.0007	43	57
Te_2Zn	8	0.0008	1	1
N_2Re	155	0.0008	16	25
$Ga_2I_2Tb_2$	330	0.0008	36	25
K	342	0.0008	61	37

Table showing the first 50 lattice matching combinations with the smallest strain needed to build the supercell.

$CdBr_2$ (P-3m1)

Structural and electronic properties

**Formula**	$CdBr_2$
**Spacegroup**	P-3m1
**Prototype**	CdI2
**Parent 3D**	$CdBr_2$
**Source DB**	COD
**DB ID**	9009124
**DF2-C09 Binding energy [meV/ Å^2]**	10.75
**RVV10 Binding energy [meV/ Å^2]**	17.49
**Band gap (PBE) [eV]**	3.24

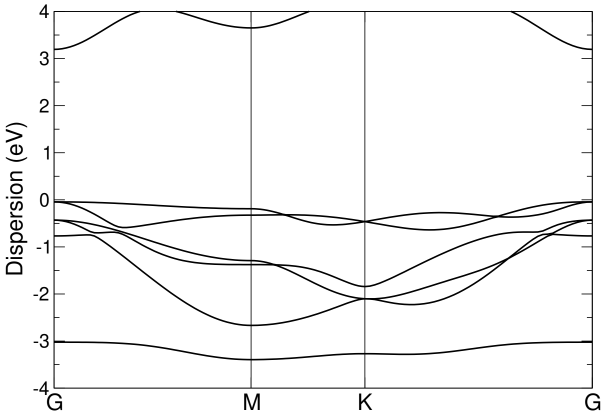

Band structure: Electronic band structure of $CdBr_2$ (P-3m1) in a representative window around the Fermi energy and along a high-symmetry path.

Optimized crystal structure

Structural parameters: relaxed cell (top) and atomic positions (bottom) of $CdBr_2$ (P-3m1) in Cartesian coordinates.

		x [Å]	y [Å]	z [Å]
$\mathbf{a_1}$		4.00742654	0.00000000	0.00000000
$\mathbf{a_2}$		−2.00371327	3.47053319	0.00000000
$\mathbf{a_3}$		0.00000000	0.00000000	23.17657330
		x [Å]	y [Å]	z [Å]
●	Br	0.00000000	2.31368879	13.19333236
●	Cd	2.00371327	1.15684440	11.58828665
●	Br	2.00371327	3.47053319	9.98324094

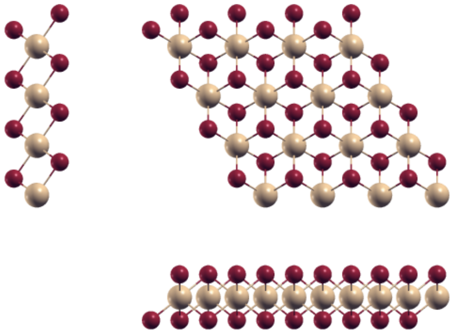

Orthographic projections: views of $CdBr_2$ (P-3m1) as seen from the x axis (left), the y axis (bottom) and the z axis (center).

Lattice matching - minimal size

Formula	N° atoms	strain	cell size 1	cell size 2
K	4	0.4943	1	1
Na	4	0.2568	1	1
Gd	4	0.2388	1	1
As_2	5	0.268	1	1
CaCl	5	0.1281	1	1
Cl_2Zn	6	0.2665	1	1
$MoTe_2$	6	0.2599	1	1
HfS_2	6	0.272	1	1
CaI_2	6	0.4705	1	1
$HfTe_2$	6	0.0043	1	1
Te_2V	6	0.2626	1	1
$CuTe_2$	6	0.2714	1	1
Ca_2Si	6	2.9913	1	1
I_2Yb	6	0.4641	1	1
Br_2Co	6	0.2673	1	1
Ca_2N	6	0.2687	1	1
$AuTe_2$	6	0.0034	1	1
Cl_2Zn	6	0.139	1	1
$PdTe_2$	6	0.0009	1	1
S_2Ti	6	1.5246	1	1
I_2Zn	6	0.0081	1	1
Te_2Zn	6	0.2597	1	1
Bi_2Pd	6	0.4326	1	1
Br_2Mn	6	0.2648	1	1
Cl_2Co	6	1.5221	1	1
$CrTe_2$	6	0.2495	1	1
PtS_2	6	0.2582	1	1
$CoTe_2$	6	0.2724	1	1
Br_2V	6	1.5623	1	1
ClNZr	6	1.5471	1	1
CdClO	6	0.2619	1	1
Ba_2N	6	0.0028	1	1
Se_2Ti	6	0.2546	1	1
Br_2Ti	6	0.2488	1	1
Te_2Zr	6	0.0039	1	1
Te_2W	6	0.2601	1	1
I_2Tm	6	0.4675	1	1
OTl_2	6	0.2622	1	1
BrNZr	6	1.5952	1	1
Br_2Fe	6	0.2674	1	1
GeS_2	6	0.1206	1	1
$MnSe_2$	6	0.128	1	1
Br_2Cr	6	0.2491	1	1
DyI_2	6	0.4777	1	1
Br_2Ni	6	0.2749	1	1
$NbSe_2$	6	1.5874	1	1
Cl_2Mg	6	0.275	1	1
F_2Ni	6	0.1358	1	1
Se_2Ta	6	0.2478	1	1
$PtTe_2$	6	0.003	1	1

Table showing the first 50 lattice matching combinations with the smallest number of atoms in the supercell.

Lattice matching - minimal strain

Formula	N° atoms	strain	cell size 1	cell size 2
$Dy_2I_2S_2$	627	0.0	81	64
GeI_2La_2	638	0.0	91	73
ClNZr	255	0.0	36	49
$HfLiS_2$	516	0.0	64	81
NS_2Ta	91	0.0	9	16
Br_2Hf_2	357	0.0	43	57
I_2La_2Sb	437	0.0	64	49
$CrSe_2$	123	0.0001	16	25
Br_2Cr	339	0.0001	49	64
Br_2Fe	543	0.0001	81	100
CCl_2Lu_2	743	0.0001	81	100
Cl_2Sc_2	403	0.0001	49	64
FeO_2	354	0.0001	39	79
Br_2Co	543	0.0002	81	100
NaO_4	488	0.0002	81	49
FHOZn	148	0.0002	16	25
$Br_2H_2Zr_2$	531	0.0002	49	64
Br_2PY_2	8	0.0002	1	1
$CrTe_2$	339	0.0002	49	64
I_2Tm	492	0.0002	91	73
$Gd_2I_2S_2$	561	0.0003	73	57
Te_2Zn	435	0.0003	64	81
As_2	443	0.0003	81	100
Br_2Mn	492	0.0003	73	91
N_2Re	222	0.0003	25	49
Br_2Ti	339	0.0004	49	64
BrNZr	300	0.0004	43	57
$Ce_2I_2S_2$	363	0.0004	49	36
Ca_2Si	255	0.0004	49	36
K	241	0.0004	64	49
S_2Ta	183	0.0004	25	36
$MoTe_2$	435	0.0004	64	81
NbS_2	183	0.0004	25	36
C	76	0.0004	9	49
Br_2Ca_3Si	561	0.0005	73	57
$Br_2H_2Zr_2$	402	0.0006	36	49
Te_2W	435	0.0006	64	81
$I_2Nd_2S_2$	429	0.0006	57	43
Se_2Ti	390	0.0006	57	73
DyI_2	435	0.0007	81	64
$NbSe_2$	300	0.0007	43	57
KS_2Ti	583	0.0007	73	91
$Cl_2H_2Zr_2$	291	0.0007	25	36
$BrKO_3$	227	0.0007	49	16
$I_2S_2Sm_2$	486	0.0008	64	49
Cl_2Zn	543	0.0008	81	100
Ca_2N	543	0.0008	81	100
PtS_2	435	0.0008	64	81
NaO_4	368	0.0008	61	37
BN	203	0.0009	25	64

Table showing the first 50 lattice matching combinations with the smallest strain needed to build the supercell.

$CdCl_2$ (P-3m1)

Structural and electronic properties

**Formula**	$CdCl_2$
**Spacegroup**	P-3m1
**Prototype**	CdI2
**Parent 3D**	$CdCl_2$
**Source DB**	COD
**DB ID**	9009125
**DF2-C09 Binding energy [meV/ Å^2]**	10.98
**RVV10 Binding energy [meV/ Å^2]**	17.83
**Band gap (PBE) [eV]**	3.89

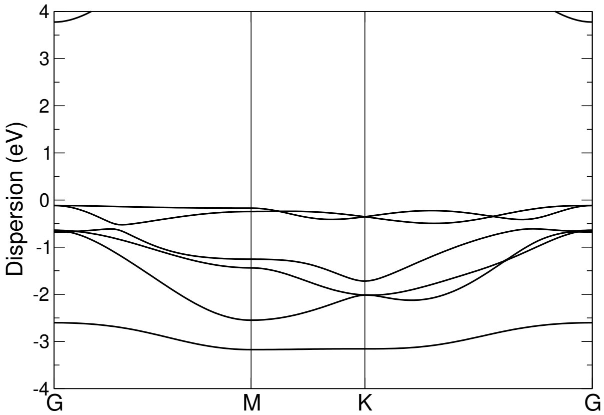

Band structure: Electronic band structure of $CdCl_2$ (P-3m1) in a representative window around the Fermi energy and along a high-symmetry path.

Optimized crystal structure

Structural parameters: relaxed cell (top) and atomic positions (bottom) of $CdCl_2$ (P-3m1) in Cartesian coordinates.

	x [Å]	y [Å]	z [Å]
$\mathbf{a_1}$	3.88453618	−0.00000000	0.00000000
$\mathbf{a_2}$	−1.94226809	3.36410702	0.00000000
$\mathbf{a_3}$	0.00000000	0.00000000	22.88766288

		x [Å]	y [Å]	z [Å]
●	Cl	1.94226809	1.12136901	9.99068083
●	Cd	-0.00000000	2.24273801	11.44383144
●	Cl	1.94226809	3.36410702	12.89698205

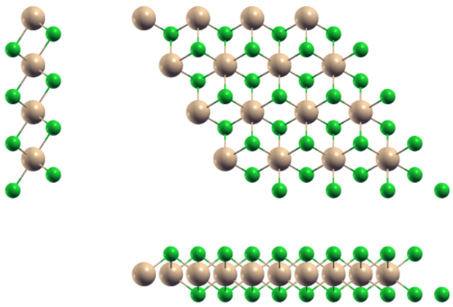

Orthographic projections: views of $CdCl_2$ (P-3m1) as seen from the x axis (left), the y axis (bottom) and the z axis (center).

Lattice matching - minimal size

Formula	N° atoms	strain	cell size 1	cell size 2
Tl	4	1.5382	1	1
HgO	5	0.1109	1	1
AsSb	5	0.0049	1	1
Bi_2	5	0.4561	1	1
GeTe	5	0.0011	1	1
S_2	5	0.0026	1	1
CaCl	5	0.1392	1	1
$IrTe_2$	6	0.002	1	1
CdI_2	6	0.4504	1	1
$AgTe_2$	6	0.4237	1	1
$MoSe_2$	6	1.5329	1	1
$ReSe_2$	6	0.26	1	1
S_2Ta	6	1.5951	1	1
Br_2Ca	6	0.4534	1	1
CaI_2	6	2.9355	1	1
$InSe_2$	6	0.0004	1	1
$GeTe_2$	6	0.001	1	1
I_2Mn	6	0.0001	1	1
NSr_2	6	0.0033	1	1
Ca_2Si	6	3.1938	1	1
I_2Yb	6	2.9026	1	1
PbS_2	6	0.0075	1	1
BiClTe	6	0.4513	1	1
LiO_2	6	0.067	1	1
Cl_2Zn	6	0.1524	1	1
FeI_2	6	0.0021	1	1
I_2Ni	6	0.0008	1	1
S_2Ti	6	1.6322	1	1
NbS_2	6	1.5917	1	1
CrI_2	6	0.0025	1	1
BiBrTe	6	0.465	1	1
Bi_2Pd	6	0.1144	1	1
N_2W	6	4.8691	1	1
Cl_2Ni	6	0.2612	1	1
Cl_2Co	6	1.6296	1	1
$CrTe_2$	6	0.2699	1	1
Br_2V	6	0.2588	1	1
ClNZr	6	0.2559	1	1
Cl_2Fe	6	0.2503	1	1
S_2Ta	6	1.554	1	1
Se_2V	6	1.5443	1	1
Se_2Ti	6	0.2755	1	1
Br_2Ti	6	0.2691	1	1
$AsSe_2$	6	0.2633	1	1
I_2Tm	6	2.9202	1	1
BiITe	6	0.4817	1	1
BrNZr	6	0.2651	1	1
$NbSe_2$	6	0.2618	1	1
CoI_2	6	0.0045	1	1
GeS_2	6	0.1299	1	1

Table showing the first 50 lattice matching combinations with the smallest number of atoms in the supercell.

Lattice matching - minimal strain

Formula	N° atoms	strain	cell size 1	cell size 2
N_3W_2	107	0.0001	9	16
NbS_2	300	0.0001	43	57
I_2Mn	6	0.0001	1	1
Br_2Hf_2	583	0.0002	73	91
Ba_2Cd	339	0.0002	65	48
GeI_2La_2	386	0.0002	57	43
$F_2I_2Pb_2$	483	0.0002	65	48
Ni_2Te_2	7	0.0002	1	1
$Cl_2Er_2H_2$	9	0.0002	1	1
Cu_4Te_2	786	0.0002	100	81
Se_2Ta	543	0.0003	81	100
Cu_2Sr_2	565	0.0003	91	73
N_2W	390	0.0003	49	81
FeH_2O_2	416	0.0003	37	61
Sb_2Te_2	208	0.0003	36	25
$Br_2Cr_2O_2$	675	0.0003	73	76
Sb_2Te_3	705	0.0003	100	81
Br_2V	435	0.0003	64	81
ClNZr	390	0.0004	57	73
Se_2V	255	0.0004	36	49
S_2Ta	300	0.0004	43	57
$InSe_2$	6	0.0004	1	1
$F_2Se_2Y_2$	171	0.0004	25	16
$LiNbS_2$	357	0.0004	43	57
I_2Tm	300	0.0004	57	43
$ReSe_2$	435	0.0005	64	81
$NbSe_2$	492	0.0005	73	91
$Cl_2H_2Sc_2$	843	0.0005	81	100
$Ge_2I_2La_2$	486	0.0005	64	49
BiITe	390	0.0006	73	57
BrNZr	492	0.0006	73	91
$Gd_2I_2S_2$	363	0.0006	49	36
Cl_2Cu	375	0.0006	61	64
CNNa	411	0.0006	77	60
Bi_2Te_3	504	0.0006	73	57
H_2Si_2	7	0.0007	1	1
BiBrTe	492	0.0007	91	73
$AsSe_2$	492	0.0007	73	91
Cl_2Fe	339	0.0008	49	64
LiO	147	0.0008	25	36
ReS_2	123	0.0008	16	25
As_2O_3	272	0.0008	49	25
I_2Ni	6	0.0008	1	1
Br_2Ca_3Si	363	0.0008	49	36
I_2Yb	300	0.0009	57	43
$Br_2H_2Zr_2$	609	0.0009	57	73
N_2W	294	0.0009	37	61
$Ho_2I_2Se_2$	345	0.0009	55	30
S_2Ta	255	0.001	36	49
$GeTe_2$	6	0.001	1	1

Table showing the first 50 lattice matching combinations with the smallest strain needed to build the supercell.

CdI_2 (P-3m1)

Structural and electronic properties

**Formula**	CdI_2
**Spacegroup**	P-3m1
**Prototype**	CdI2
**Parent 3D**	Cd_3I_6
**Source DB**	ICSD
**DB ID**	20745
**DF2-C09 Binding energy [meV/ Å^2]**	10.69
**RVV10 Binding energy [meV/ Å^2]**	16.92
**Band gap (PBE) [eV]**	2.41

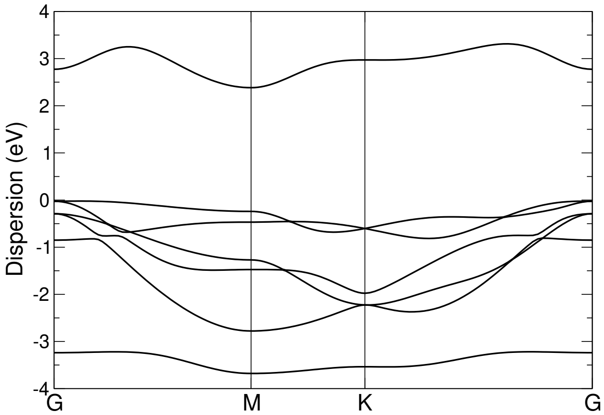

Band structure: Electronic band structure of CdI_2 (P-3m1) in a representative window around the Fermi energy and along a high-symmetry path.

Optimized crystal structure

Structural parameters: relaxed cell (top) and atomic positions (bottom) of CdI_2 (P-3m1) in Cartesian coordinates.

	x [Å]	y [Å]	z [Å]
$\mathbf{a_1}$	4.27654377	0.00000000	0.00000000
$\mathbf{a_2}$	−2.13827189	3.70359555	0.00000000
$\mathbf{a_3}$	0.00000000	0.00000000	23.48285095

	x [Å]	y [Å]	z [Å]
I	0.00000000	2.46906370	13.49554256
Cd	0.00000000	0.00000000	11.74142548
I	2.13827189	1.23453185	9.98730839

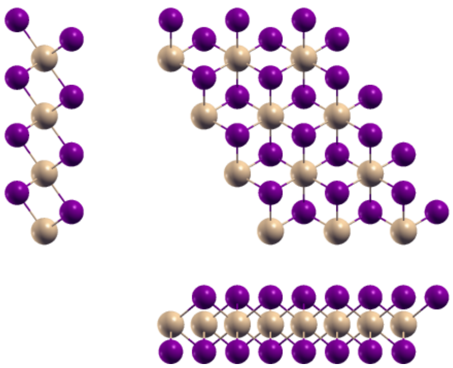

Orthographic projections: views of CdI_2 (P-3m1) as seen from the x axis (left), the y axis (bottom) and the z axis (center).

Lattice matching - minimal size

Formula	N° atoms	strain	cell size 1	cell size 2
Tl	4	4.8597	1	1
InSe	5	0.0035	1	1
AsSb	5	0.2667	1	1
Bi_2	5	0.0023	1	1
GeTe	5	0.2755	1	1
AgTl	5	0.1491	1	1
PbTe	5	0.0017	1	1
CaCl	5	0.1121	1	1
$CdCl_2$	6	0.274	1	1
PSn_2	6	1.5579	1	1
Br_2Zn	6	1.6367	1	1
Br_2Ca	6	0.0012	1	1
$InSe_2$	6	0.2745	1	1
$AsSn_2$	6	1.5854	1	1
$GeTe_2$	6	0.2725	1	1
$SiTe_2$	6	0.2581	1	1
I_2Pr	6	0.1337	1	1
I_2Mn	6	0.2741	1	1
S_2Zr	6	1.5523	1	1
NSr_2	6	0.269	1	1
PbS_2	6	0.263	1	1
BiClTe	6	0.0004	1	1
Cl_2Zn	6	0.1184	1	1
FeI_2	6	0.2709	1	1
I_2Ni	6	0.2728	1	1
Te_2Ti	6	1.639	1	1
CrI_2	6	0.2702	1	1
BaF_2	6	0.0072	1	1
BiBrTe	6	0.006	1	1
$RhTe_2$	6	0.2471	1	1
PtS_2	6	1.4472	1	1
Se_2V	6	4.8778	1	1
AsKSn	6	0.0086	1	1
I_2Nd	6	0.1345	1	1
$NiTe_2$	6	0.2573	1	1
Cl_2Cu	6	0.0686	1	1
S_2Sn	6	1.5542	1	1
I_2V	6	0.26	1	1
GeI_2	6	0.0015	1	1
Se_2Zr	6	0.2587	1	1
STl_2	6	0.0063	1	1
$PtSe_2$	6	1.5901	1	1
CoI_2	6	0.2673	1	1
GeS_2	6	0.425	1	1
$MnSe_2$	6	0.1121	1	1
Br_2Ni	6	1.5278	1	1
CeI_2	6	0.1332	1	1
Br_2Mg	6	0.2707	1	1
I_2Ti	6	0.2678	1	1
$NbTe_2$	6	1.5508	1	1

Table showing the first 50 lattice matching combinations with the smallest number of atoms in the supercell.

Lattice matching - minimal strain

Formula	N° atoms	strain	cell size 1	cell size 2
Te_2Zn	183	0.0	25	36
FHOZn	91	0.0	9	16
NS_2Ta	433	0.0001	39	79
Se_2V	390	0.0001	49	81
Gd_2GeI_2	8	0.0001	1	1
I_2Ti	543	0.0001	81	100
$CrSe_2$	75	0.0002	9	16
$Cu_2Rb_2Te_2$	483	0.0002	65	48
CoI_2	543	0.0002	81	100
$MoTe_2$	183	0.0002	25	36
F_2Na	390	0.0002	57	73
$HfLiS_2$	219	0.0002	25	36
$Cl_2O_2Yb_2$	609	0.0003	57	73
$PtSe_2$	300	0.0003	43	57
Br_2La_2P	8	0.0003	1	1
BiClTe	6	0.0004	1	1
Te_2W	183	0.0004	25	36
Bi_2In_2	387	0.0004	65	48
CdI_2	6	0.0004	1	1
I_2Pr	6	0.0004	1	1
Se_2Zr	435	0.0005	64	81
I_2V	435	0.0005	64	81
Ga_2Se_2	403	0.0005	49	64
Se_2V	294	0.0005	37	61
$I_2N_2Zr_2$	531	0.0005	49	64
$NbTe_2$	255	0.0005	36	49
CI_2Y_2	743	0.0006	81	100
$Cl_2O_2Tm_2$	609	0.0006	57	73
$Cl_2Er_2O_2$	678	0.0006	64	81
$Br_2Hf_2N_2$	474	0.0006	50	54
AsSb	443	0.0006	81	100
CBr_2Lu_2	353	0.0007	36	49
CaClHO	463	0.0007	57	73
Pd_2S_4	423	0.0007	69	36
Tl	228	0.0007	49	81
$Br_2N_2Zr_2$	402	0.0007	36	49
Se_2Sn	543	0.0007	81	100
S_2Zr	255	0.0008	36	49
CdH_2O_2	255	0.0008	25	36
ClH_3O	638	0.0008	91	73
$Cl_2Ho_2O_2$	678	0.0008	64	81
S_2Ta	294	0.0008	37	61
Bi_2STe_2	8	0.0008	1	1
$Br_2Ho_2S_2$	747	0.0008	101	74
$SiTe_2$	435	0.0009	64	81
PbS_2	492	0.0009	73	91
PTe_2Zr_2	674	0.0009	73	91
$AsSn_2$	300	0.0009	43	57
NSr_2	543	0.001	81	100
Br_2Pr_2	463	0.001	57	73

Table showing the first 50 lattice matching combinations with the smallest strain needed to build the supercell.

CdIBr (P3m1)

Structural and electronic properties

**Formula**	CdIBr
**Spacegroup**	P3m1
**Prototype**	BiTeI
**Parent 3D**	$Br_2Cd_2I_2$
**Source DB**	MPDS
**DB ID**	S1815969
**DF2-C09 Binding energy [meV/ Å^2]**	10.69
**RVV10 Binding energy [meV/ Å^2]**	N/A
**Band gap (PBE) [eV]**	2.54

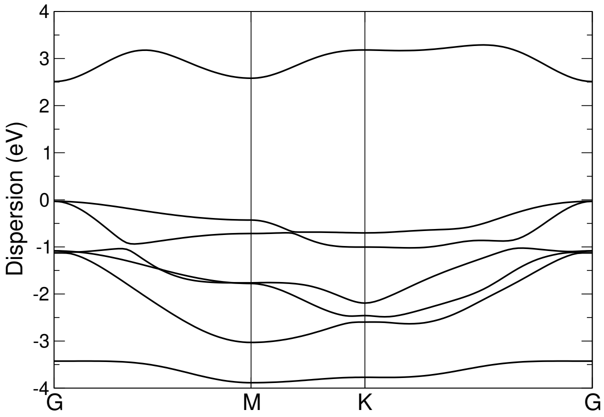

Band structure: Electronic band structure of CdIBr (P3m1) in a representative window around the Fermi energy and along a high-symmetry path.

Optimized crystal structure

Structural parameters: relaxed cell (top) and atomic positions (bottom) of CdIBr (P3m1) in Cartesian coordinates.

	x [Å]	y [Å]	z [Å]
$\mathbf{a_1}$	−2.09060649	−3.62103665	0.00000000
$\mathbf{a_2}$	4.18121297	0.00000000	0.00000000
$\mathbf{a_3}$	0.00000000	0.00000000	18.64131232

	x [Å]	y [Å]	z [Å]
Cd	0.00000000	-2.41402443	0.09007680
I	0.00000000	-0.00000000	-1.69939575
Br	2.09060649	-1.20701222	1.60931895

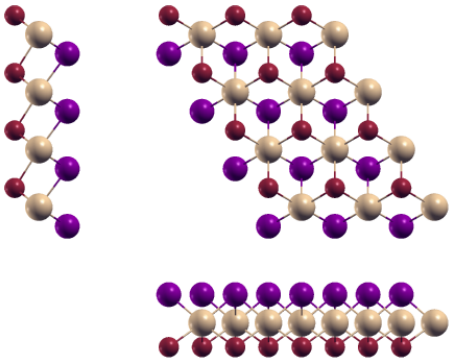

Orthographic projections: views of CdIBr (P3m1) as seen from the x axis (left), the y axis (bottom) and the z axis (center).

Lattice matching - minimal size

Formula	N° atoms	strain	cell size 1	cell size 2
InSe	5	0.0073	1	1
AgTl	5	0.1597	1	1
Ag_2	5	0.457	1	1
PbTe	5	0.0091	1	1
Sb_2	5	0.0086	1	1
CaCl	5	0.1165	1	1
I_2Mg	6	0.0031	1	1
Ba_2Pt	6	0.4564	1	1
Br_2Zn	6	0.2673	1	1
$AsSn_2$	6	0.2576	1	1
$SiTe_2$	6	0.2733	1	1
I_2Pr	6	0.1425	1	1
Br_2La	6	0.0028	1	1
Ca_2Si	6	0.4677	1	1
Cl_2Zn	6	0.1244	1	1
Te_2Ti	6	0.2677	1	1
BaF_2	6	0.0033	1	1
$RhTe_2$	6	0.2615	1	1
GeI_2	6	0.0049	1	1
AsKSn	6	0.0019	1	1
$PbTe_2$	6	0.001	1	1
I_2Nd	6	0.1433	1	1
$NiTe_2$	6	0.2724	1	1
Cl_2Cu	6	0.0656	1	1
$SnTe_2$	6	0.0071	1	1
I_2V	6	0.2753	1	1
GeI_2	6	0.0093	1	1
Se_2Zr	6	0.2739	1	1
I_2Pb	6	0.4609	1	1
STl_2	6	0.0043	1	1
$PtSe_2$	6	0.2585	1	1
GeS_2	6	0.1116	1	1
$TaTe_2$	6	0.2568	1	1
$MnSe_2$	6	0.1165	1	1
Br_2Ni	6	0.2469	1	1
CeI_2	6	0.1418	1	1
In	6	0.6316	1	3
Se_2Yb	6	0.0047	1	1
Cl_2Mg	6	0.247	1	1
$BiTe_2$	6	0.0044	1	1
F_2Ni	6	0.122	1	1
I_2La	6	0.1476	1	1
F_2Na	6	0.2701	1	1
F_2Zn	6	0.1391	1	1
$HfSe_2$	6	0.2677	1	1
Bi_2Te_2	7	0.5008	1	1
Fe_2Te_2	7	0.1322	1	1
Ca_2Cl_2	7	0.1325	1	1
CdClHO	7	0.256	1	1
Cl_2OOs	7	0.1212	1	1

Table showing the first 50 lattice matching combinations with the smallest number of atoms in the supercell.

Lattice matching - minimal strain

Formula	N° atoms	strain	cell size 1	cell size 2
SSb_2Te_2	8	0.0001	1	1
$Cl_2H_2Zr_2$	198	0.0001	16	25
$CoTe_2$	300	0.0001	43	57
Te_2Ti	543	0.0001	81	100
I_2Pb	543	0.0001	100	81
BN	77	0.0001	9	25
$HfSe_2$	543	0.0001	81	100
Sm	139	0.0001	25	64
BrNZr	183	0.0001	25	36
Ga_2Se_2	583	0.0001	73	91
H_2MnO_2	647	0.0002	49	100
$Br_2N_2Zr_2$	531	0.0002	49	64
Ga_2S_2	357	0.0002	43	57
Br_2Zn	543	0.0002	81	100
CBr_2Lu_2	467	0.0002	49	64
HfS_2	300	0.0003	43	57
Ca_2Si	492	0.0003	91	73
$Ce_2I_2S_2$	711	0.0003	91	73
CCl_2Gd_2	743	0.0003	81	100
NbS_2	123	0.0003	16	25
$MnNaTe_2$	7	0.0004	1	1
CdClHO	463	0.0004	57	73
$Hf_2I_2N_2$	609	0.0004	57	73
AgTl	320	0.0004	66	61
Te_2V	255	0.0005	36	49
Br_2Hf_2	219	0.0005	25	36
$PtSe_2$	435	0.0006	64	81
$CuTe_2$	300	0.0006	43	57
$Ba_2F_2I_2$	483	0.0006	65	48
Hg_3N_2	120	0.0007	25	9
$Cl_2Hf_2N_2$	402	0.0008	36	49
OTl_2	255	0.0008	36	49
$LiOS_2Ti$	173	0.0008	16	25
CdClHO	516	0.0008	64	81
Cl_2Y_2	583	0.0008	73	91
$I_2N_2Zr_2$	765	0.0009	73	91
CBr_2Y_2	743	0.0009	81	100
Cl_4Mn	759	0.0009	118	81
$Cl_2O_2Ti_2$	696	0.0009	70	81
$I_2Pr_2S_2$	786	0.001	100	81
$Br_2Cr_2S_2$	360	0.001	40	40
$PbTe_2$	6	0.001	1	1
Br_2Pr_2	643	0.001	81	100
$TaTe_2$	390	0.001	57	73
Sb_2Te_2	565	0.001	91	73
CdClO	255	0.001	36	49
Br_2Mn	255	0.001	36	49
Cu_2Te_2	534	0.001	70	81
CaH_2O_2	414	0.0011	43	57
O_4PTl	483	0.0011	65	48

Table showing the first 50 lattice matching combinations with the smallest strain needed to build the supercell.

CdOCl (P3m1)

Structural and electronic properties

**Formula**	CdOCl
**Spacegroup**	P3m1
**Prototype**	BiTeI
**Parent 3D**	$Cd_2O_2Cl_2$
**Source DB**	COD
**DB ID**	9016472
**DF2-C09 Binding energy [meV/ Å^2]**	25.6
**RVV10 Binding energy [meV/ Å^2]**	34.29
**Band gap (PBE) [eV]**	0.25

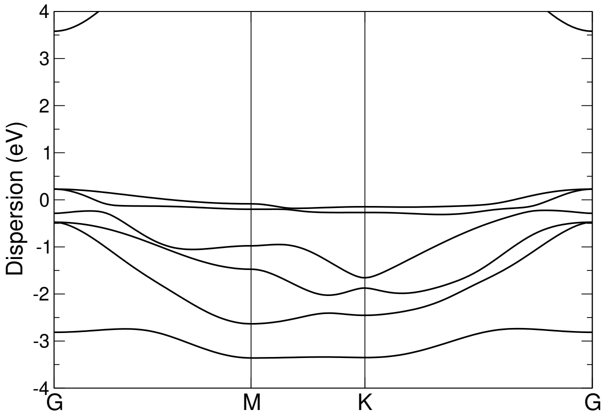

Band structure: Electronic band structure of CdOCl (P3m1) in a representative window around the Fermi energy and along a high-symmetry path.

Optimized crystal structure

Structural parameters: relaxed cell (top) and atomic positions (bottom) of CdOCl (P3m1) in Cartesian coordinates.

		x [Å]	y [Å]	z [Å]
$\mathbf{a_1}$		3.57626472	0.00000000	0.00000000
$\mathbf{a_2}$		−1.78813236	3.09713610	0.00000000
$\mathbf{a_3}$		0.00000000	0.00000000	22.47582186
		x [Å]	y [Å]	z [Å]
●	Cd	1.78813236	1.03237870	10.71806643
●	Cl	0.00000000	2.06475740	12.50157924
●	O	0.00000000	0.00000000	9.96531483

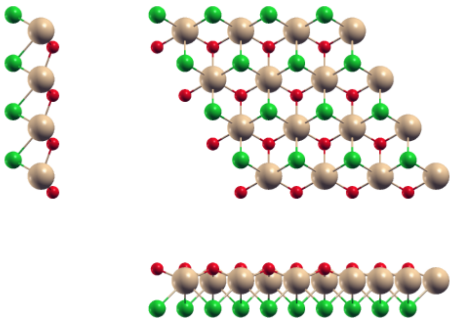

Orthographic projections: views of CdOCl (P3m1) as seen from the x axis (left), the y axis (bottom) and the z axis (center).

Lattice matching - minimal size

Formula	N° atoms	strain	cell size 1	cell size 2
K	4	0.3192	1	1
Tl	4	0.1234	1	1
Sn	4	0.432	1	1
Na	4	0.0037	1	1
In	4	0.4421	1	1
In	4	0.2555	1	1
HgO	5	0.1315	1	1
As_2	5	0.0043	1	1
LiO	5	0.2704	1	1
Mg_2	5	0.1149	1	1
Sb_2	5	0.5006	1	1
CrS_2	6	1.5446	1	1
Cl_2Zn	6	0.0032	1	1
I_2Mg	6	2.9684	1	1
S_2V	6	0.2599	1	1
MoS_2	6	0.2609	1	1
$MoTe_2$	6	0.0014	1	1
$AgTe_2$	6	0.1252	1	1
HfS_2	6	0.0071	1	1
$HfTe_2$	6	0.4602	1	1
Te_2V	6	0.0005	1	1
$CuTe_2$	6	0.0067	1	1
Br_2La	6	2.9729	1	1
Br_2Co	6	0.0039	1	1
ReS_2	6	1.5882	1	1
Ca_2N	6	0.0048	1	1
$AuTe_2$	6	0.4799	1	1
$PdTe_2$	6	0.4736	1	1
Mg_3	6	0.12	1	1
I_2Zn	6	2.8522	1	1
Te_2Zn	6	0.0016	1	1
S_2W	6	0.261	1	1
Bi_2Pd	6	0.1389	1	1
Br_2Mn	6	0.002	1	1
$CrTe_2$	6	0.0089	1	1
PtS_2	6	0.0026	1	1
$CoTe_2$	6	0.0074	1	1
Ba_2N	6	0.464	1	1
Se_2Ti	6	0.0052	1	1
AsKSn	6	3.0356	1	1
Te_2Zr	6	0.4614	1	1
Te_2W	6	0.0013	1	1
Cl_2Cu	6	0.5927	1	1
Cl_2V	6	0.2667	1	1
OTl_2	6	0.0002	1	1
Br_2Fe	6	0.0039	1	1
Br_2Cr	6	0.0092	1	1
Br_2Ni	6	0.0091	1	1
MoS_2	6	0.2612	1	1
Cl_2Mg	6	0.0092	1	1

Table showing the first 50 lattice matching combinations with the smallest number of atoms in the supercell.

Lattice matching - minimal strain

Formula	N° atoms	strain	cell size 1	cell size 2
$SnTe_2$	300	0.0	57	43
$PbTe_2$	255	0.0	49	36
In	244	0.0	57	73
$Ga_2I_2Tb_2$	258	0.0001	36	25
N_2Re	123	0.0001	16	25
Te_2Zr	543	0.0001	100	81
As_2Sn_2	447	0.0001	73	57
Bi_2S_3	563	0.0001	81	64
Br_2Ho_2	499	0.0001	81	64
$F_2Lu_2Se_2$	486	0.0002	64	49
OTl_2	6	0.0002	1	1
F_2Zn	339	0.0002	65	48
Br_2Gd_2	447	0.0002	73	57
ClH_3O	93	0.0002	16	9
$Cl_2Hf_2N_2$	9	0.0002	1	1
$Ga_2Gd_2I_2$	258	0.0002	36	25
BH_4Li	429	0.0003	57	43
CrS_2	255	0.0003	36	49
$Br_2Ca_2F_2$	483	0.0003	65	48
Br_2Tb_2	499	0.0004	81	64
Au_2I_2	552	0.0004	104	60
In_2Se_3	705	0.0004	100	81
$Br_2Gd_2O_2$	483	0.0004	65	48
$HfTe_2$	543	0.0004	100	81
GeI_2La_2	155	0.0004	25	16
Br_2Ca	183	0.0004	36	25
S_2V	435	0.0004	64	81
H_2MgO_2	597	0.0004	64	81
$F_2Ho_2Se_2$	711	0.0005	91	73
$CrSe_2$	492	0.0005	73	91
Te_2V	6	0.0005	1	1
ReS_2	300	0.0006	43	57
FHOZn	583	0.0006	73	91
Cl_2V	543	0.0006	81	100
$MnNaTe_2$	291	0.0006	49	36
$Er_2F_2Se_2$	786	0.0006	100	81
BN	275	0.0006	39	79
$CrSe_2$	543	0.0006	81	100
Bi_2	158	0.0007	36	25
$Ba_2Ge_2Mn_2$	840	0.0007	118	81
I_2Tm	123	0.0007	25	16
I_2Yb	123	0.0007	25	16
Br_2Er_2	447	0.0007	73	57
$Br_2F_2Yb_2$	483	0.0007	65	48
$C_2Br_2Tb_2$	156	0.0007	20	16
$F_2I_2Yb_2$	840	0.0008	118	81
CdH_2O_2	8	0.0008	1	1
Ba_2Cu_2	139	0.0009	25	16
Cu_3Se_3	786	0.0009	100	81
$Ga_2Ge_2Te_2$	429	0.0009	57	43

Table showing the first 50 lattice matching combinations with the smallest strain needed to build the supercell.

$Ce_2O_2Br_2$ (P4/nmm)

Structural and electronic properties

**Formula**	$Ce_2O_2Br_2$
**Spacegroup**	P4/nmm
**Prototype**	PbClF
**Parent 3D**	$Br_2Ce_2O_2$
**Source DB**	MPDS
**DB ID**	S1903359
**DF2-C09 Binding energy [meV/ Å^2]**	25.83
**RVV10 Binding energy [meV/ Å^2]**	N/A
**Band gap (PBE) [eV]**	0.0

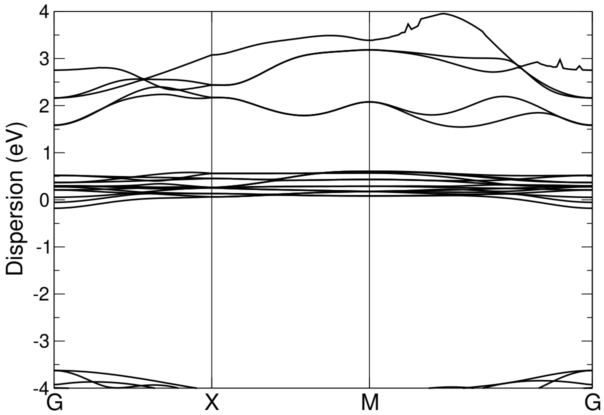

Band structure: Electronic band structure of $Ce_2O_2Br_2$ (P4/nmm) in a representative window around the Fermi energy and along a high-symmetry path.

Optimized crystal structure

Structural parameters: relaxed cell (top) and atomic positions (bottom) of $Ce_2O_2Br_2$ (P4/nmm) in Cartesian coordinates.

	x [Å]	y [Å]	z [Å]
$\mathbf{a_1}$	3.99393267	0.00000000	0.00000000
$\mathbf{a_2}$	0.00000000	3.99393267	0.00000000
$\mathbf{a_3}$	0.00000000	0.00000000	22.68993675

		x [Å]	y [Å]	z [Å]
●	Ce	0.99848317	-0.99848317	1.23222961
●	Br	-0.99848317	-2.99544950	2.87767479
●	Ce	-0.99848317	-2.99544950	-1.23222961
●	Br	0.99848317	-0.99848317	-2.87767479
●	O	-0.99848317	-0.99848317	0.00000000
●	O	0.99848317	-2.99544950	0.00000000

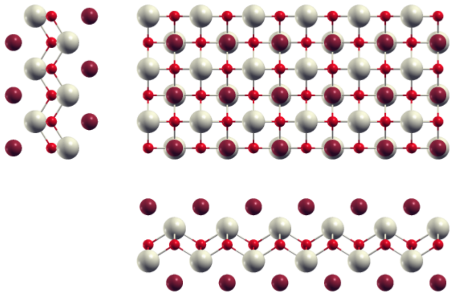

Orthographic projections: views of $Ce_2O_2Br_2$ (P4/nmm) as seen from the x axis (left), the y axis (bottom) and the z axis (center).

Lattice matching - minimal size

Formula	N° atoms	strain	cell size 1	cell size 2
K	7	0.1714	1	1
Tl	7	0.1092	1	1
InSe	8	0.1366	1	1
Bi_2	8	0.1414	1	1
AgTl	8	0.0145	1	1
Ag_2	8	0.1778	1	1
P_2	8	0.1103	1	1
PbTe	8	0.138	1	1
CaCl	8	0.2202	1	1
CdI_2	9	0.1395	1	1
$MoSe_2$	9	0.1094	1	1
Ba_2Pt	9	0.1775	1	1
Br_2Ca	9	0.1405	1	1
CaI_2	9	0.1612	1	1
$SiTe_2$	9	0.1088	1	1
Br_2Cu	9	0.1014	1	1
Ca_2Si	9	0.1831	1	1
I_2Yb	9	0.1585	1	1
BiClTe	9	0.1398	1	1
Cl_2Ti	9	0.1103	1	1
BrCdI	9	0.1311	1	1
HgI_2	9	0.3914	1	1
BaF_2	9	0.1336	1	1
BiBrTe	9	0.1446	1	1
S_2Ta	9	0.1088	1	1
Se_2V	9	0.1091	1	1
AsKSn	9	0.1325	1	1
$PbTe_2$	9	0.1304	1	1
$NiTe_2$	9	0.1087	1	1
Cl_2Cu	9	0.0969	1	1
I_2Tm	9	0.1599	1	1
GeI_2	9	0.1382	1	1
Se_2Zr	9	0.1089	1	1
I_2Pb	9	0.1797	1	1
STl_2	9	0.1343	1	1
BiITe	9	0.1507	1	1
GeS_2	9	0.2068	1	1
$MnSe_2$	9	0.2201	1	1
DyI_2	9	0.1642	1	1
GdI_2	9	0.1472	1	1
I_2La	9	0.0047	1	1
CdI_2	9	0.1391	1	1
Sm	9	0.1656	1	3
I_2Pr	9	0.1398	1	1
Se_2W	9	0.1093	1	1
Cu_2Sr_2	10	0.1455	1	1
$LiMnTe_2$	10	0.1385	1	1
Cu_2Te_2	10	0.0557	1	1
Ag_2Br_2	10	0.0042	1	1
$AsLi_3$	10	0.1368	1	1

Table showing the first 50 lattice matching combinations with the smallest number of atoms in the supercell.

Lattice matching - minimal strain

Formula	N° atoms	strain	cell size 1	cell size 2
$K_2Mn_2Sb_2$	12	0.0	1	1
H_2Na_2Pd	625	0.0	50	65
$MnSe_2$	795	0.0001	82	101
AlH_4Na	876	0.0001	85	61
$Se_2Si_2Zr_2$	870	0.0001	64	81
Se_2Ta_4	876	0.0001	61	85
Ag_2I_2	752	0.0001	82	65
CaCl	694	0.0002	82	101
C_2Li_2	244	0.0003	24	25
$Cu_2K_2Te_2$	678	0.0003	64	49
Mg_2	800	0.0003	85	145
$Eu_2I_2O_2$	12	0.0005	1	1
O_4PSn	690	0.0005	65	50
Cl_2Rb_2	670	0.0005	89	34
Mg_3	708	0.0005	65	106
Ag_2I_2	742	0.0006	81	64
PtS_2	840	0.0006	81	118
$MnSe_2$	786	0.0006	81	100
CaCl	686	0.0007	81	100
GeS_2	495	0.0008	50	65
Co_2S_2	896	0.0008	82	101
NS_2Zr	958	0.0008	81	118
$Ca_2H_2I_2$	12	0.0009	1	1
HgI_2	687	0.0009	82	65
CNNa	972	0.001	110	104
$HfLiS_2$	958	0.001	81	118
Ca_2O_2	294	0.001	25	36
Pb_2Se_2	912	0.0011	98	81
Mg_6	246	0.0011	16	25
H_2Na_2Pd	614	0.0011	49	64
Tl	859	0.0011	113	181
$Cl_2O_2Sc_2$	90	0.0011	7	8
Na	604	0.0012	81	118
CdClHO	548	0.0013	48	65
$Hf_2I_2N_2$	678	0.0013	48	65
$As_2Cd_2K_2$	690	0.0013	65	50
Fe_2S_2	886	0.0013	81	100
Cl_2Fe	852	0.0014	79	126
PSn_2	483	0.0014	48	65
Co_2S_2	886	0.0014	81	100
$MoTe_2$	840	0.0014	81	118
$H_2Li_2O_2$	882	0.0014	65	82
CS_2Ta_2	929	0.0015	64	109
$I_2O_2Sm_2$	12	0.0015	1	1
Cl_2Ti	711	0.0015	64	109
Ho_2S_2	866	0.0015	85	89
$Cl_2N_2Zr_2$	750	0.0015	52	73
CaH_2O_2	677	0.0015	52	73
O_4PSn	678	0.0015	64	49
P_2	602	0.0015	64	109

Table showing the first 50 lattice matching combinations with the smallest strain needed to build the supercell.

$Ce_2S_2I_2$ (P-3m1)

Structural and electronic properties

**Formula**	$Ce_2S_2I_2$
**Spacegroup**	P-3m1
**Prototype**	SmSI
**Parent 3D**	$Ce_2I_2S_2$
**Source DB**	MPDS
**DB ID**	S1703861
**DF2-C09 Binding energy [meV/ Å^2]**	10.42
**RVV10 Binding energy [meV/ Å^2]**	N/A
**Band gap (PBE) [eV]**	0.0

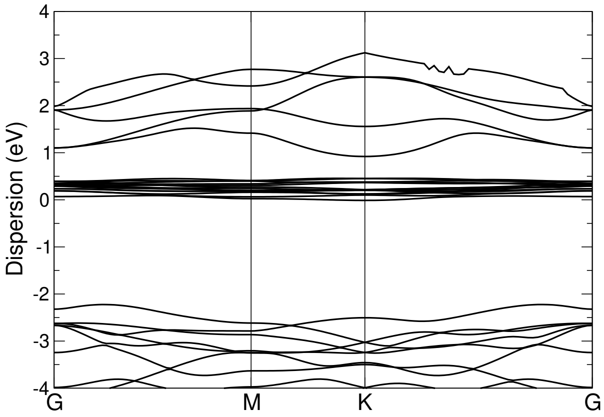

Band structure: Electronic band structure of $Ce_2S_2I_2$ (P-3m1) in a representative window around the Fermi energy and along a high-symmetry path.

Optimized crystal structure

Structural parameters: relaxed cell (top) and atomic positions (bottom) of $Ce_2S_2I_2$ (P-3m1) in Cartesian coordinates.

	x [Å]	y [Å]	z [Å]
$\mathbf{a_1}$	2.33569047	−4.04553457	0.00000000
$\mathbf{a_2}$	2.33569047	4.04553457	0.00000000
$\mathbf{a_3}$	0.00000000	0.00000000	27.03867591

	x [Å]	y [Å]	z [Å]
Ce	-1.16784524	0.67425576	-1.77857360
Ce	1.16784524	-0.67425576	1.77857360
S	-1.16784524	0.67425576	1.05376101
S	1.16784524	-0.67425576	-1.05376101
I	1.16784524	2.02276729	-3.78851795
I	1.16784524	2.02276729	3.78851795

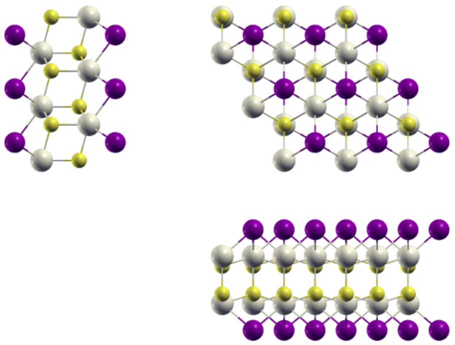

Orthographic projections: views of $Ce_2S_2I_2$ (P-3m1) as seen from the x axis (left), the y axis (bottom) and the z axis (center).

Lattice matching - minimal size

Formula	N° atoms	strain	cell size 1	cell size 2
K	7	0.1382	1	1
InSe	8	0.2743	1	1
AgTl	8	0.1188	1	1
Tl	8	0.2256	1	2
Ag_2	8	0.0042	1	1
I_2Mg	9	0.2595	1	1
Ba_2Pt	9	0.0045	1	1
I_2Pr	9	0.111	1	1
$CuTe_2$	9	4.8574	1	1
Br_2La	9	0.26	1	1
Ca_2Si	9	0.0	1	1
BrCdI	9	0.2639	1	1
HgI_2	9	0.1538	1	1
I_2Zn	9	0.2475	1	1
BaF_2	9	0.2686	1	1
GeI_2	9	0.2569	1	1
Ba_2Hg	9	0.127	1	1
AsKSn	9	0.2666	1	1
$PbTe_2$	9	0.2625	1	1
I_2Nd	9	0.1113	1	1
Cl_2Cu	9	0.3049	1	1
$SnTe_2$	9	0.2539	1	1
I_2Pb	9	0.0027	1	1
STl_2	9	0.27	1	1
CeI_2	9	0.4437	1	1
Se_2Yb	9	0.2573	1	1
$BiTe_2$	9	0.2576	1	1
I_2La	9	0.1131	1	1
Ba_2Cd	9	0.1288	1	1
Cu_2I_2	10	0.1207	1	1
Cu_2Te_2	10	0.1109	1	1
Ir_2P_2	10	0.1113	1	1
Ag_2Br_2	10	0.1133	1	1
$AsLi_3$	10	0.2747	1	1
O_2Sn_2	10	0.1107	1	1
Cu_2S_2	10	0.429	1	1
Au_2Br_2	10	0.1258	1	1
Br_2Cu_2	10	0.4332	1	1
As_2Ir_2	10	0.1142	1	1
O_2Pb_2	10	0.1189	1	1
$AgBrO_2$	10	0.2844	1	1
Br_2Gd_2	10	1.5822	1	1
$MnNaTe_2$	10	0.2633	1	1
O_2Sn_2	10	0.4303	1	1
$AsCuLi_2$	10	0.2563	1	1
Cu_2I_2	10	0.2608	1	1
P_2Rh_2	10	0.4275	1	1
F_2Tl_2	10	0.4278	1	1
I_2La_2	10	0.2658	1	1
Br_2Ho_2	10	1.5643	1	1

Table showing the first 50 lattice matching combinations with the smallest number of atoms in the supercell.

Lattice matching - minimal strain

Formula	N° atoms	strain	cell size 1	cell size 2
Ca_2Si	9	0.0	1	1
Ga_2S_2	618	0.0	49	81
S_2Ta	297	0.0	25	49
Br_2Ti	102	0.0	9	16
GeTe	222	0.0001	25	36
$Ce_2I_2Si_2$	870	0.0001	64	81
$I_2N_2Zr_2$	246	0.0001	16	25
$F_2Lu_2Se_2$	678	0.0001	49	64
I_2Mg	627	0.0001	64	81
Br_2HLa	634	0.0001	57	73
$CoTe_2$	537	0.0001	49	81
Pb_2Se_2	396	0.0002	40	39
CNb_2S_2	629	0.0002	39	79
Br_2PY_2	461	0.0002	36	49
Cl_2La_2	486	0.0002	43	57
$Br_2H_2Zr_2$	150	0.0002	9	16
$CeLi_2P_2$	707	0.0002	57	73
In_2S_3	330	0.0003	25	36
P_2	392	0.0003	39	79
BrCdI	711	0.0003	73	91
Au_2Se_2	516	0.0003	56	45
H_2Si_2	294	0.0003	25	36
Br_2Cr	102	0.0003	9	16
SSb_2Te_2	893	0.0004	73	91
Br_2Cd	363	0.0004	36	49
Ga_2S_2	466	0.0004	37	61
Sb_2Se_2Te	789	0.0004	64	81
Cl_2OV	696	0.0004	56	90
Cl_2Ti	471	0.0005	39	79
HfS_2	537	0.0005	49	81
Br_2La	627	0.0005	64	81
$Cl_2H_2Sc_2$	150	0.0005	9	16
Ge_2Te_2	994	0.0005	99	100
Cl_2Sc_2	118	0.0005	9	16
CS_2Ta_2	629	0.0005	39	79
Bi_2Se_3	789	0.0005	64	81
$AsCuLi_2$	634	0.0006	57	73
$CrTe_2$	102	0.0006	9	16
$InSe_2$	258	0.0006	25	36
Ga_2S_2	466	0.0006	37	61
CaCl	722	0.0007	81	118
$MnSe_2$	840	0.0007	81	118
$MnNaTe_2$	802	0.0007	73	91
AsKSn	786	0.0007	81	100
BaF_2	786	0.0007	81	100
O_2Sn_2	122	0.0007	11	14
Se_2Ta	102	0.0007	9	16
PTe_2Ti_2	527	0.0007	37	61
Co_2S_2	958	0.0008	81	118
$I_2Pr_2Si_2$	780	0.0008	57	73

Table showing the first 50 lattice matching combinations with the smallest strain needed to build the supercell.

CeI_2 (P4/mmm)

Structural and electronic properties

**Formula**	CeI_2
**Spacegroup**	P4/mmm
**Prototype**	Zr2Cu
**Parent 3D**	CeI_2
**Source DB**	MPDS
**DB ID**	S1721470
**DF2-C09 Binding energy [meV/ Å^2]**	16.77
**RVV10 Binding energy [meV/ Å^2]**	N/A
**Band gap (PBE) [eV]**	0.0

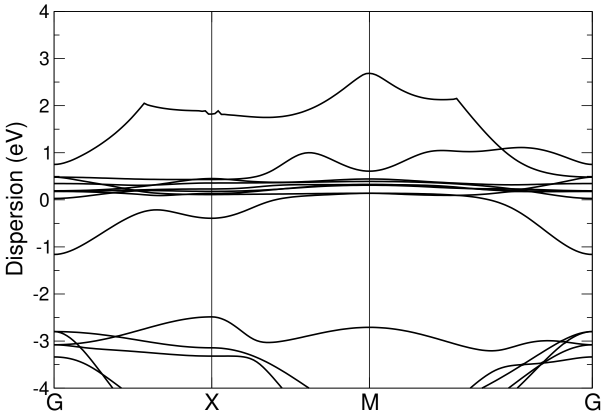

Band structure: Electronic band structure of CeI_2 (P4/mmm) in a representative window around the Fermi energy and along a high-symmetry path.

Optimized crystal structure

Structural parameters: relaxed cell (top) and atomic positions (bottom) of CeI_2 (P4/mmm) in Cartesian coordinates.

	x [Å]	y [Å]	z [Å]
$\mathbf{a_1}$	3.89976437	0.00000000	0.00000000
$\mathbf{a_2}$	0.00000000	3.89976437	0.00000000
$\mathbf{a_3}$	0.00000000	0.00000000	19.41913996

		x [Å]	y [Å]	z [Å]
●	I	0.00000000	0.00000000	-1.87856208
●	Ce	1.94988218	-1.94988218	0.00000000
●	I	0.00000000	0.00000000	1.87856208

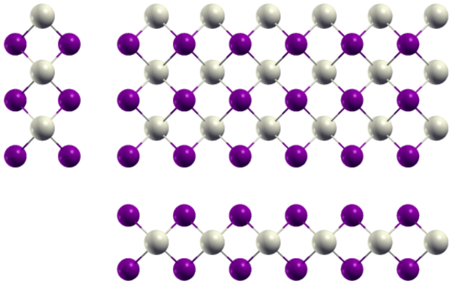

Orthographic projections: views of CeI_2 (P4/mmm) as seen from the x axis (left), the y axis (bottom) and the z axis (center).

Lattice matching - minimal size

Formula	N° atoms	strain	cell size 1	cell size 2
K	4	0.3787	1	1
K	4	0.185	1	1
InSe	5	0.1463	1	1
Bi_2	5	0.1518	1	1
AgTl	5	0.0231	1	1
Ag_2	5	0.192	1	1
LiO	5	0.1096	1	1
PbTe	5	0.1479	1	1
Sb_2	5	0.1329	1	1
I_2Mg	6	0.1374	1	1
S_2V	6	0.1112	1	1
MoS_2	6	0.111	1	1
CdI_2	6	0.1496	1	1
Ba_2Pt	6	0.1917	1	1
Br_2Ca	6	0.1507	1	1
CaI_2	6	0.1738	1	1
$AsSn_2$	6	0.1089	1	1
I_2Pr	6	0.0008	1	1
Br_2La	6	0.1377	1	1
Br_2Cu	6	0.1058	1	1
Ca_2Si	6	0.7523	1	1
I_2Yb	6	0.1708	1	1
BiClTe	6	0.1499	1	1
BrCdI	6	0.14	1	1
HgI_2	6	1.1212	1	1
I_2Zn	6	0.1305	1	1
BaF_2	6	0.1428	1	1
BiBrTe	6	0.1553	1	1
S_2W	6	0.111	1	1
GeI_2	6	0.1359	1	1
AsKSn	6	0.1416	1	1
$PbTe_2$	6	0.1392	1	1
I_2Nd	6	0.0017	1	1
Cl_2Cu	6	0.0987	1	1
I_2Tm	6	0.1724	1	1
$SnTe_2$	6	0.1341	1	1
Cl_2V	6	0.1101	1	1
GeI_2	6	0.1481	1	1
I_2Pb	6	0.1941	1	1
STl_2	6	0.1437	1	1
$PtSe_2$	6	0.1091	1	1
BiITe	6	0.1622	1	1
GeS_2	6	0.2192	1	1
DyI_2	6	0.1771	1	1
Se_2Yb	6	0.1361	1	1
MoS_2	6	0.111	1	1
$BiTe_2$	6	0.1363	1	1
GdI_2	6	0.1583	1	1
$CrSe_2$	6	0.1104	1	1
I_2La	6	0.0066	1	1

Table showing the first 50 lattice matching combinations with the smallest number of atoms in the supercell.

Lattice matching - minimal strain

Formula	N° atoms	strain	cell size 1	cell size 2
$Br_2Eu_2O_2$	9	0.0001	1	1
GeS_2	543	0.0001	81	100
Cl_2Zn	339	0.0002	48	65
As_2Fe_2	650	0.0002	82	101
Ni_2Se_2	7	0.0002	1	1
Se_2Sn_2	624	0.0002	100	81
Se_2Ta_4	933	0.0003	85	113
Pd_2S_4	420	0.0003	70	35
Mg_2	407	0.0003	65	106
O_4PSn	363	0.0003	49	36
$Cl_2S_2Tl_2$	708	0.0004	106	65
AuI_4Li	477	0.0005	81	39
Br_2Co	339	0.0006	48	65
Mg_3	123	0.0006	16	25
$Ag_2K_2Se_2$	486	0.0006	64	49
HgO	147	0.0006	25	36
Br_2Fe	339	0.0007	48	65
$AsSe_2$	597	0.0007	81	118
$Hf_2Se_2Si_2$	852	0.0007	82	101
In	400	0.0007	85	145
NS_2Ta	216	0.0007	20	39
As_2Fe_2	643	0.0007	81	100
$Ba_2H_2I_2$	621	0.0007	85	61
I_2Pr	6	0.0008	1	1
$NbSe_2$	597	0.0008	81	118
CCl_2Lu_2	469	0.0009	48	65
KS_2Ti	404	0.0009	48	65
Li_2Tl_2	678	0.0009	118	81
$As_2Cd_2K_2$	363	0.0009	49	36
H_2Na_2Pd	743	0.0009	81	100
Br_2Ca_3Si	135	0.0009	21	12
Se_2Ta	597	0.0009	81	118
Mg_4	811	0.0009	89	136
Au_2I_2	568	0.001	92	73
Ag_2I_2	679	0.001	113	85
Pb_2Se_2	196	0.001	32	25
Cl_2Cu	588	0.001	88	108
As_2	274	0.001	48	65
$NbSe_2$	597	0.001	81	118
Cl_4Mn	233	0.001	36	25
Se_2W	615	0.001	79	126
$MoSe_2$	615	0.0011	79	126
Au_2I_2	561	0.0011	91	72
Tl	363	0.0011	79	126
K	385	0.0011	101	82
F_4Pb	1000	0.0011	145	113
$I_2O_2Yb_2$	9	0.0011	1	1
Bi_2Pd	438	0.0011	61	85
Br_2CsF	499	0.0012	85	61
$Hf_2Se_2Si_2$	843	0.0012	81	100

Table showing the first 50 lattice matching combinations with the smallest strain needed to build the supercell.

CeSiI (P-3m1)

Structural and electronic properties

**Formula**	CeSiI
**Spacegroup**	P-3m1
**Prototype**	Ce2Si2I2
**Parent 3D**	$Ce_2Si_2I_2$
**Source DB**	ICSD
**DB ID**	407246
**DF2-C09 Binding energy [meV/ Å^2]**	14.09
**RVV10 Binding energy [meV/ Å^2]**	20.55
**Band gap (PBE) [eV]**	N/A

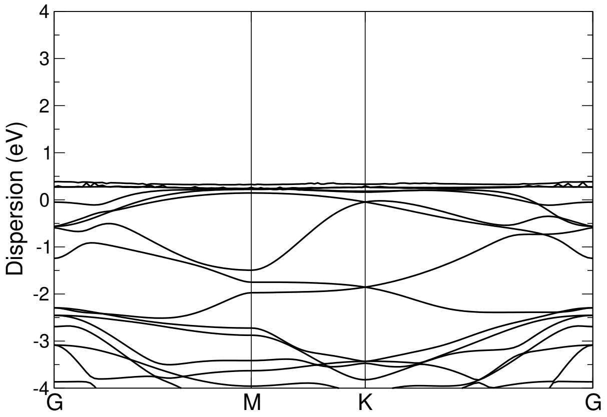

Band structure: Electronic band structure of CeSiI (P-3m1) in a representative window around the Fermi energy and along a high-symmetry path.

Optimized crystal structure

Structural parameters: relaxed cell (top) and atomic positions (bottom) of CeSiI (P-3m1) in Cartesian coordinates.

		x [Å]	y [Å]	z [Å]
$\mathbf{a_1}$		4.15160363	0.00000000	0.00000000
$\mathbf{a_2}$		−2.07580182	3.59539421	0.00000000
$\mathbf{a_3}$		0.00000000	0.00000000	28.28294219
		x [Å]	y [Å]	z [Å]
●	Ce	0.00000000	0.00000000	16.07257531
●	Si	2.07580182	1.19846474	14.18590435
●	I	0.00000000	2.39692948	18.24431625
●	Ce	0.00000000	0.00000000	12.21036688
●	Si	0.00000000	2.39692948	14.09703783
●	I	2.07580182	1.19846474	10.03862594

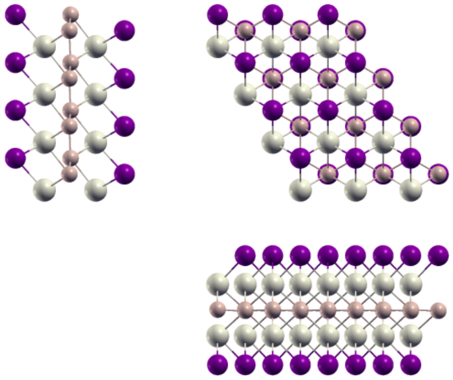

Orthographic projections: views of CeSiI (P-3m1) as seen from the x axis (left), the y axis (bottom) and the z axis (center).

Lattice matching - minimal size

Formula	N° atoms	strain	cell size 1	cell size 2
K	7	0.4518	1	1
Na	7	1.5372	1	1
AgTl	8	0.1633	1	1
Ag_2	8	0.4654	1	1
As_2	8	1.5951	1	1
Sb_2	8	0.0054	1	1
CaCl	8	0.1182	1	1
Cl_2Zn	9	1.5873	1	1
I_2Mg	9	0.0002	1	1
$MoTe_2$	9	1.5535	1	1
PSn_2	9	0.257	1	1
Ba_2Pt	9	0.4647	1	1
Br_2Zn	9	0.2722	1	1
HfS_2	9	0.2487	1	1
$AsSn_2$	9	0.2623	1	1
I_2Pr	9	0.1454	1	1
$CuTe_2$	9	0.2483	1	1
S_2Zr	9	0.256	1	1
Br_2La	9	0.0006	1	1
Ca_2Si	9	0.4762	1	1
Br_2Co	9	1.5919	1	1
Ca_2N	9	1.5989	1	1
BrCdI	9	0.0034	1	1
Cl_2Zn	9	0.1266	1	1
Te_2Ti	9	0.2726	1	1
I_2Zn	9	0.0086	1	1
BaF_2	9	0.0068	1	1
$RhTe_2$	9	0.2663	1	1
GeI_2	9	0.0016	1	1
PtS_2	9	1.5446	1	1
$CoTe_2$	9	0.2492	1	1
CdClO	9	1.5639	1	1
Se_2Ti	9	1.5258	1	1
AsKSn	9	0.0053	1	1
Te_2W	9	1.5546	1	1
$PbTe_2$	9	0.0024	1	1
I_2Nd	9	0.1463	1	1
S_2Sn	9	0.2563	1	1
$SnTe_2$	9	0.0039	1	1
Sn	9	0.6281	1	3
I_2Pb	9	0.4694	1	1
STl_2	9	0.0077	1	1
$PtSe_2$	9	0.2632	1	1
OTl_2	9	1.5652	1	1
Br_2Fe	9	1.5924	1	1
GeS_2	9	0.1128	1	1
$TaTe_2$	9	0.2615	1	1
$MnSe_2$	9	0.1181	1	1
Br_2Ni	9	1.6303	1	1
CeI_2	9	0.1448	1	1

Table showing the first 50 lattice matching combinations with the smallest number of atoms in the supercell.

Lattice matching - minimal strain

Formula	N° atoms	strain	cell size 1	cell size 2
Br_2Fe	429	0.0	43	57
Tl	121	0.0	16	25
Br_2Co	429	0.0001	43	57
$CoTe_2$	486	0.0001	49	64
Ga_2S_2	550	0.0001	49	64
Ca_2Si	678	0.0001	81	64
$Ce_2I_2S_2$	870	0.0001	81	64
C_2Li_2	396	0.0001	40	39
$I_2Pr_2S_2$	984	0.0001	91	73
$NbTe_2$	561	0.0002	57	73
I_2Mg	9	0.0002	1	1
CCl_2Lu_2	543	0.0002	43	57
LiO	344	0.0002	37	61
PtS_2	363	0.0003	36	49
Sb_2Se_2Te	11	0.0003	1	1
As_2	372	0.0004	43	57
S_2Zr	561	0.0004	57	73
HfS_2	486	0.0004	49	64
Au_2Se_2	538	0.0004	63	40
$ReSe_2$	258	0.0005	25	36
Cl_2Ni	258	0.0005	25	36
$HfLiS_2$	412	0.0005	36	49
$Br_2N_2Ti_2$	930	0.0005	73	82
Br_2La	9	0.0006	1	1
CdClHO	802	0.0006	73	91
Ag_2	692	0.0006	91	73
Se_2W	171	0.0006	16	25
Bi_2Se_3	11	0.0006	1	1
N_3W_2	629	0.0006	39	79
S_2Sn	561	0.0006	57	73
Cl_2Zn	429	0.0007	43	57
Te_2Zn	363	0.0007	36	49
$CuTe_2$	486	0.0008	49	64
$MoSe_2$	171	0.0008	16	25
Sb_2Se_2Te	11	0.0008	1	1
$AgBrO_2$	738	0.0008	73	75
$F_2Se_2Y_2$	510	0.0008	49	36
$PtSe_2$	711	0.0008	73	91
Se_2V	171	0.0008	16	25
Gd_2I_2	10	0.0009	1	1
Ba_2Pt	765	0.0009	91	73
$CrSe_2$	537	0.0009	49	81
$NbSe_2$	258	0.0009	25	36
Ca_2N	429	0.0009	43	57
$MoTe_2$	363	0.0009	36	49
$RhTe_2$	786	0.0009	81	100
Cl_2OV	122	0.0009	11	14
Ag_2I_2	582	0.0009	65	48
NS_2Zr	412	0.0009	36	49
Se_2Ta	258	0.001	25	36

Table showing the first 50 lattice matching combinations with the smallest strain needed to build the supercell.

$Co(OH)_2$ (C2/m)

Structural and electronic properties

**Formula**	$Co(OH)_2$
**Spacegroup**	C2/m
**Prototype**	Mg(OH)2
**Parent 3D**	$Co(OH)_2$
**Source DB**	ICSD
**DB ID**	88940
**DF2-C09 Binding energy [meV/ Å^2]**	28.27
**RVV10 Binding energy [meV/ Å^2]**	37.87
**Band gap (PBE) [eV]**	N/A

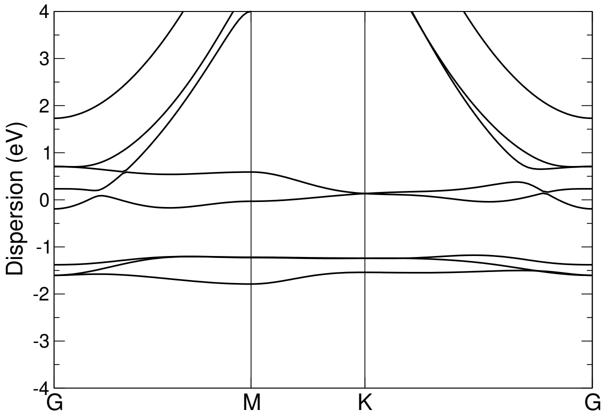

Band structure: Electronic band structure of $Co(OH)_2$ (C2/m) in a representative window around the Fermi energy and along a high-symmetry path.

Optimized crystal structure

Structural parameters: relaxed cell (top) and atomic positions (bottom) of $Co(OH)_2$ (C2/m) in Cartesian coordinates.

		x [Å]	y [Å]	z [Å]
$\mathbf{a_1}$		3.09571809	0.00000000	0.00000000
$\mathbf{a_2}$		−1.54785904	2.68097051	0.00000000
$\mathbf{a_3}$		0.00000000	0.00000000	23.81423132
		x [Å]	y [Å]	z [Å]
•	H	0.00000000	1.78731367	9.98825264
•	O	0.00000000	1.78731367	10.96571273
•	Co	0.00000000	0.00000000	11.90711566
•	H	1.54785904	0.89365684	13.82597868
•	O	1.54785904	0.89365684	12.84851859

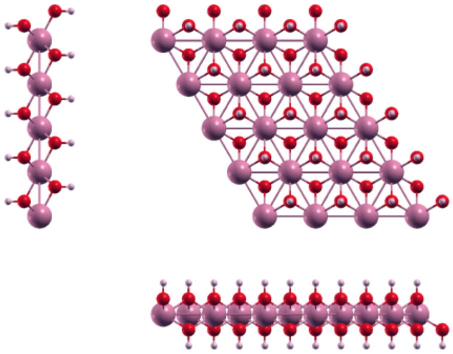

Orthographic projections: views of $Co(OH)_2$ (C2/m) as seen from the x axis (left), the y axis (bottom) and the z axis (center).

Lattice matching - minimal size

Formula	N° atoms	strain	cell size 1	cell size 2
Nd	6	0.2488	1	1
Sn	6	0.1517	1	1
Na	6	0.4991	1	1
As_2	7	2.9915	1	1
CrS_2	8	0.0049	1	1
Cl_2Zn	8	2.9776	1	1
$MoTe_2$	8	2.9164	1	1
$ReSe_2$	8	0.4669	1	1
HfS_2	8	3.0282	1	1
FeO_2	8	0.2751	1	1
Br_2Co	8	2.9858	1	1
ReS_2	8	0.001	1	1
CrO_2	8	0.2585	1	1
Ca_2N	8	2.9985	1	1
Cl_2Zn	8	0.3127	1	1
MnO_2	8	0.2616	1	1
S_2Ti	8	0.4518	1	1
Br_2Mn	8	2.9617	1	1
Cl_2Ni	8	0.4692	1	1
Cl_2Co	8	0.451	1	1
$CrTe_2$	8	0.4849	1	1
PtS_2	8	2.9004	1	1
$CoTe_2$	8	3.0326	1	1
Br_2V	8	0.4649	1	1
ClNZr	8	0.4596	1	1
Cl_2Fe	8	0.4492	1	1
CdClO	8	2.9352	1	1
Ba_2N	8	13.6249	1	1
Se_2Ti	8	0.4948	1	1
Br_2Ti	8	0.4834	1	1
Te_2W	8	2.9185	1	1
$AsSe_2$	8	0.4729	1	1
CdO_2	8	0.4506	1	1
BrNZr	8	0.4763	1	1
$NbSe_2$	8	0.4702	1	1
Br_2Fe	8	2.9867	1	1
O_2Zn	8	0.0082	1	1
Br_2Cr	8	0.4841	1	1
Cl_2Zr	8	0.4502	1	1
$FeSe_2$	8	0.1455	1	1
Se_2Ta	8	0.4705	1	1
$NbSe_2$	8	0.4736	1	1
Se_2Ta	8	0.4814	1	1
O_2Pt	8	0.0084	1	1
Fe_2Te_2	9	0.3362	1	1
NS_2Zr	9	0.5002	1	1
Br_2Hf_2	9	0.4752	1	1
Cu_2Te_2	9	0.3179	1	1
Cl_2Zr_2	9	0.4629	1	1
Br_2Zr_2	9	0.4938	1	1

Table showing the first 50 lattice matching combinations with the smallest number of atoms in the supercell.

Lattice matching - minimal strain

Formula	N° atoms	strain	cell size 1	cell size 2
$ReSe_2$	674	0.0	91	73
Te_2Zn	414	0.0	57	43
Te_2Zr	416	0.0001	61	37
Br_2Ti	536	0.0001	73	57
$Br_2H_2Zr_2$	986	0.0001	100	81
Cu_3Se_3	699	0.0001	81	49
BrNZr	597	0.0001	81	64
I_2Pb	57	0.0001	9	4
Ca_2N	353	0.0001	49	36
$MoTe_2$	414	0.0002	57	43
Br_2HLa	116	0.0002	16	9
C	206	0.0002	25	81
$CeLi_2P_2$	125	0.0002	16	9
Se_2Ti	467	0.0002	64	49
$Br_2H_2Zr_2$	707	0.0002	73	57
FeI_2	173	0.0003	25	16
CCl_2Sc_2	905	0.0003	100	81
$HfLiS_2$	457	0.0003	57	43
Br_2Hf_2	661	0.0003	81	64
Te_2W	414	0.0003	57	43
Nd	309	0.0003	49	64
$AgNO_3$	170	0.0003	25	9
Br_2Cr	536	0.0003	73	57
Ba_2N	552	0.0004	81	49
$F_2I_2Tm_2$	315	0.0004	39	20
$PtSe_2$	255	0.0004	36	25
As_2	317	0.0004	49	36
Cd_2I_3	745	0.0004	100	49
BiBrTe	320	0.0004	49	25
Br_2Mg	173	0.0004	25	16
AsI_2La_2	745	0.0004	100	49
$Cu_2Na_2Se_2$	315	0.0005	39	20
$Bi_2I_2O_2$	315	0.0005	39	20
NaO_4	170	0.0005	25	9
$Cl_2H_2Sc_2$	707	0.0005	73	57
Hg_3N_2	485	0.0005	81	16
In_2Se_3	490	0.0005	61	37
CCl_2Lu_2	425	0.0005	49	36
$Cl_2F_2Pb_2$	315	0.0005	39	20
$HfTe_2$	416	0.0005	61	37
Cl_2Sc_2	593	0.0006	73	57
CrO_2	563	0.0006	64	81
Cu_2Sr_2	345	0.0006	49	25
Br_2Zr_2	516	0.0006	64	49
$AsCuLi_2$	116	0.0006	16	9
ClNZr	743	0.0006	100	81
$CrTe_2$	536	0.0006	73	57
CrI_2	173	0.0007	25	16
Au_2I_2	808	0.0007	120	52
Cl_2Zr_2	824	0.0007	100	81

Table showing the first 50 lattice matching combinations with the smallest strain needed to build the supercell.

$CoBr_2$ (P-3m1)

Structural and electronic properties

**Formula**	$CoBr_2$
**Spacegroup**	P-3m1
**Prototype**	CdI2
**Parent 3D**	$CoBr_2$
**Source DB**	COD
**DB ID**	9016149
**DF2-C09 Binding energy [meV/ Å^2]**	16.84
**RVV10 Binding energy [meV/ Å^2]**	23.27
**Band gap (PBE) [eV]**	0.2

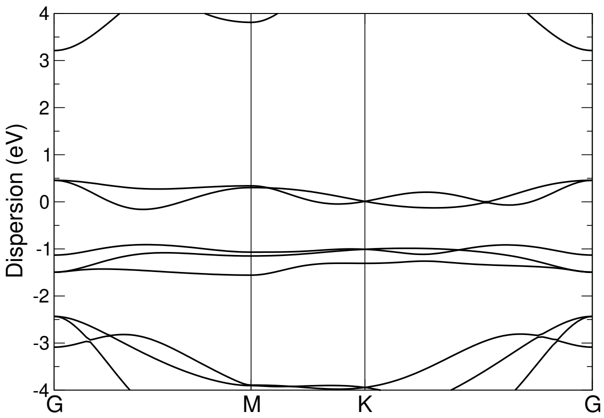

Band structure: Electronic band structure of $CoBr_2$ (P-3m1) in a representative window around the Fermi energy and along a high-symmetry path.

Optimized crystal structure

Structural parameters: relaxed cell (top) and atomic positions (bottom) of $CoBr_2$ (P-3m1) in Cartesian coordinates.

	x [Å]	y [Å]	z [Å]
$\mathbf{a_1}$	3.60539056	0.00000000	0.00000000
$\mathbf{a_2}$	−1.80269528	3.12235982	0.00000000
$\mathbf{a_3}$	0.00000000	0.00000000	22.74037882

		x [Å]	y [Å]	z [Å]
●	Br	0.00000000	2.08157321	12.71967656
●	Co	0.00000000	0.00000000	11.37018941
●	Br	1.80269528	1.04078661	10.02070227

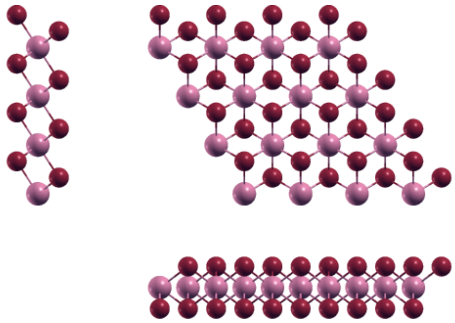

Orthographic projections: views of $CoBr_2$ (P-3m1) as seen from the x axis (left), the y axis (bottom) and the z axis (center).

Lattice matching - minimal size

Formula	N° atoms	strain	cell size 1	cell size 2
K	4	0.3115	1	1
Tl	4	0.1211	1	1
Sn	4	0.4234	1	1
Na	4	0.0074	1	1
In	4	0.4333	1	1
In	4	0.2504	1	1
HgO	5	0.1287	1	1
As_2	5	0.0004	1	1
LiO	5	0.2649	1	1
P_2	5	0.276	1	1
Mg_2	5	0.1133	1	1
Sb_2	5	2.8451	1	1
Cl_2Zn	6	0.0006	1	1
I_2Mg	6	2.9181	1	1
S_2V	6	0.2546	1	1
MoS_2	6	0.2556	1	1
$MoTe_2$	6	0.0052	1	1
$AgTe_2$	6	0.1229	1	1
PSn_2	6	0.0095	1	1
HfS_2	6	0.0032	1	1
$HfTe_2$	6	0.4507	1	1
Te_2V	6	0.0033	1	1
$CuTe_2$	6	0.0028	1	1
S_2Zr	6	0.0087	1	1
Br_2La	6	2.9225	1	1
ReS_2	6	1.5602	1	1
Ca_2N	6	0.001	1	1
$AuTe_2$	6	0.4701	1	1
$PdTe_2$	6	0.4639	1	1
Mg_3	6	0.118	1	1
I_2Zn	6	0.4818	1	1
Te_2Zn	6	0.0054	1	1
S_2W	6	0.2557	1	1
Bi_2Pd	6	0.1358	1	1
GeI_2	6	0.5005	1	1
Br_2Mn	6	0.0018	1	1
PtS_2	6	0.0064	1	1
$CoTe_2$	6	0.0035	1	1
CdClO	6	0.0038	1	1
Ba_2N	6	0.4545	1	1
Se_2Ti	6	0.009	1	1
AsKSn	6	2.9842	1	1
Te_2Zr	6	0.4519	1	1
Te_2W	6	0.005	1	1
Cl_2Cu	6	0.5811	1	1
S_2Sn	6	0.009	1	1
$SnTe_2$	6	0.4945	1	1
Cl_2V	6	0.2613	1	1
I_2Pb	6	13.6598	1	1
STl_2	6	3.0161	1	1

Table showing the first 50 lattice matching combinations with the smallest number of atoms in the supercell.

Lattice matching - minimal strain

Formula	N° atoms	strain	cell size 1	cell size 2
Ag_2	257	0.0	61	37
Br_2PY_2	705	0.0	100	81
$Ce_2I_2Si_2$	429	0.0001	57	43
Cl_2La_2	499	0.0001	81	64
Br_2Fe	6	0.0001	1	1
BH_4Li	486	0.0001	64	49
$I_2Pr_2S_2$	537	0.0001	81	49
Br_2Ho_2	565	0.0001	91	73
MoS_2	390	0.0001	57	73
NS_2Ta	219	0.0001	25	36
$Dy_2I_2S_2$	171	0.0002	25	16
Br_2Cd	543	0.0002	100	81
S_2W	390	0.0002	57	73
$CrSe_2$	435	0.0002	64	81
Ba_2Pt	294	0.0003	61	37
I_2Pr	339	0.0003	65	48
Sb_2Se_2Te	386	0.0003	57	43
$Ga_2I_2Y_2$	363	0.0003	49	36
I_2Mg	300	0.0003	57	43
CCl_2Lu_2	8	0.0003	1	1
NaO_4	545	0.0003	100	49
Li_2Tl_2	247	0.0003	49	25
MoS_2	390	0.0003	57	73
FHOZn	516	0.0003	64	81
$SnTe_2$	339	0.0003	64	49
Bi_2S_3	638	0.0004	91	73
LiO	401	0.0004	73	91
$Br_2Er_2Se_2$	801	0.0004	135	66
BiBrTe	183	0.0004	36	25
As_2	5	0.0004	1	1
Cl_4Mn	217	0.0005	39	20
BaF_2	255	0.0005	49	36
Nd	43	0.0005	9	16
$Br_2Eu_2O_2$	483	0.0005	65	48
I_2Zn	390	0.0005	73	57
CeI_2	339	0.0006	65	48
$CeLi_2P_2$	437	0.0006	64	49
S_2V	390	0.0006	57	73
Br_2Tb_2	565	0.0006	91	73
$I_2O_2Yb_2$	483	0.0006	65	48
Cl_2Zn	6	0.0006	1	1
Br_2La	300	0.0006	57	43
O_2Pt	339	0.0007	49	64
Bi_2Se_3	386	0.0007	57	43
$I_2O_2Pr_2$	840	0.0007	118	81
$I_2Pr_2S_2$	405	0.0007	61	37
Sb_2Se_2Te	386	0.0007	57	43
Bi_2Te_2	84	0.0008	16	9
Ni_2Se_2	387	0.0008	65	48
CoH_2O_2	353	0.0008	36	49

Table showing the first 50 lattice matching combinations with the smallest strain needed to build the supercell.

$CoCl_2$ (P-3m1)

Structural and electronic properties

**Formula**	$CoCl_2$
**Spacegroup**	P-3m1
**Prototype**	CdI2
**Parent 3D**	$CoCl_2$
**Source DB**	COD
**DB ID**	9014719
**DF2-C09 Binding energy [meV/ Å^2]**	10.68
**RVV10 Binding energy [meV/ Å^2]**	15.92
**Band gap (PBE) [eV]**	0.17

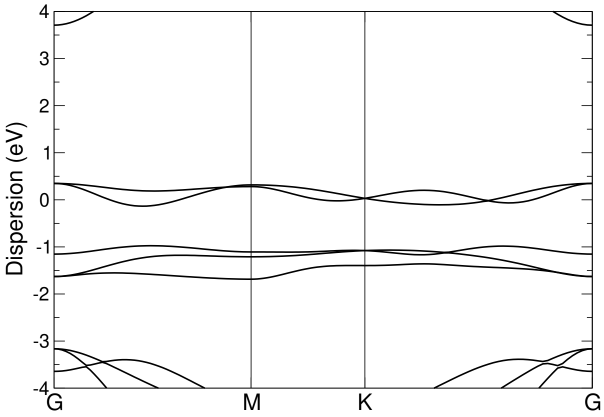

Band structure: Electronic band structure of $CoCl_2$ (P-3m1) in a representative window around the Fermi energy and along a high-symmetry path.

Optimized crystal structure

Structural parameters: relaxed cell (top) and atomic positions (bottom) of $CoCl_2$ (P-3m1) in Cartesian coordinates.

		x [Å]	y [Å]	z [Å]
$\mathbf{a_1}$		3.40975629	0.00000000	0.00000000
$\mathbf{a_2}$		−1.70487815	2.95293557	0.00000000
$\mathbf{a_3}$		0.00000000	0.00000000	22.58069729
		x [Å]	y [Å]	z [Å]
●	Cl	1.70487815	0.98431186	12.55009550
●	Co	-0.00000000	1.96862371	11.29034864
●	Cl	0.00000000	0.00000000	10.03060179

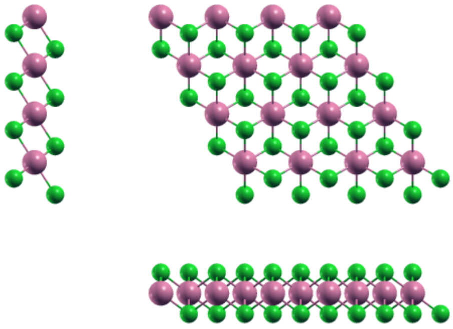

Orthographic projections: views of $CoCl_2$ (P-3m1) as seen from the x axis (left), the y axis (bottom) and the z axis (center).

Lattice matching - minimal size

Formula	N° atoms	strain	cell size 1	cell size 2
Tl	4	0.1396	1	1
Sn	4	0.1179	1	1
In	4	0.1202	1	1
HgO	5	0.1506	1	1
AsSb	5	0.4784	1	1
GeTe	5	0.4943	1	1
S_2	5	0.4984	1	1
Mg_2	5	0.1273	1	1
CaCl	5	0.2093	1	1
$IrTe_2$	6	0.4967	1	1
CrS_2	6	0.2664	1	1
$CdCl_2$	6	2.8502	1	1
Cl_2Mn	6	0.0066	1	1
$AgTe_2$	6	0.1422	1	1
$ReSe_2$	6	0.0065	1	1
S_2Ta	6	0.0046	1	1
Br_2Zn	6	0.4528	1	1
$InSe_2$	6	2.855	1	1
$SiTe_2$	6	0.463	1	1
$HfTe_2$	6	2.9845	1	1
I_2Mn	6	2.8517	1	1
NSr_2	6	0.4826	1	1
PbS_2	6	0.4718	1	1
ReS_2	6	0.2751	1	1
FeI_2	6	0.486	1	1
I_2Ni	6	2.8398	1	1
S_2Ti	6	0.0003	1	1
Mg_3	6	0.1349	1	1
Te_2Ti	6	0.4535	1	1
NbS_2	6	0.005	1	1
CrI_2	6	0.4848	1	1
Ba_2Hg	6	0.335	1	1
N_2W	6	0.2568	1	1
Cl_2Ni	6	0.0074	1	1
NbS_2	6	0.0092	1	1
Br_2V	6	0.0057	1	1
ClNZr	6	0.0035	1	1
Cl_2Fe	6	0.0007	1	1
Ba_2N	6	3.0049	1	1
Te_2Zr	6	2.991	1	1
$AsSe_2$	6	0.0089	1	1
$NiTe_2$	6	0.4615	1	1
Cl_2Cu	6	0.1079	1	1
I_2V	6	0.4663	1	1
Se_2Zr	6	0.464	1	1
BiITe	6	13.6465	1	1
CdO_2	6	0.0001	1	1
$NbSe_2$	6	0.0079	1	1
CoI_2	6	0.4796	1	1
O_2Zn	6	0.2616	1	1

Table showing the first 50 lattice matching combinations with the smallest number of atoms in the supercell.

Lattice matching - minimal strain

Formula	N° atoms	strain	cell size 1	cell size 2
GeI_2	123	0.0001	25	16
As_2CeLi_2	155	0.0001	25	16
$F_2Se_2Y_2$	471	0.0001	79	39
$NiTe_2$	543	0.0001	100	81
Cl_2Gd_2	343	0.0001	57	43
$Cl_2Ho_2O_2$	711	0.0001	91	73
PbTe	107	0.0001	25	16
CdO_2	6	0.0001	1	1
$AlLiTe_2$	331	0.0002	61	37
NSr_2	390	0.0002	73	57
In_2S_3	437	0.0002	64	49
I_2V	492	0.0002	91	73
PTe_2Zr_2	563	0.0002	81	64
$ClKO_3$	17	0.0003	4	1
Cl_2Zr	6	0.0003	1	1
S_2Ti	6	0.0003	1	1
Cu_3Se_3	363	0.0004	49	36
$F_2Lu_2Se_2$	258	0.0004	36	25
$LiMnTe_2$	139	0.0004	25	16
GeTe	290	0.0004	64	49
Te_2Zr	255	0.0004	49	36
Se_2Sn	390	0.0005	73	57
$IrTe_2$	339	0.0005	64	49
CaClHO	624	0.0005	100	81
$I_2N_2Zr_2$	531	0.0005	77	50
FeH_2O_2	597	0.0006	64	81
Cl_2Tb_2	343	0.0006	57	43
Ba_2N	255	0.0006	49	36
$Cl_2O_2Tm_2$	786	0.0006	100	81
$Br_2Ca_2H_2$	840	0.0006	118	81
CI_2Y_2	504	0.0006	73	57
$Ca_2Mn_2Si_2$	840	0.0006	118	81
Bi_2Te_3	488	0.0007	81	49
Ga_2Se_2	388	0.0007	64	49
I_2La_2Te	93	0.0007	16	9
Br_2Ca_3Si	102	0.0007	16	9
CrI_2	390	0.0007	73	57
ClH_3O	272	0.0007	49	25
$SiTe_2$	543	0.0007	100	81
BiITe	390	0.0007	81	49
Cl_2Fe	6	0.0007	1	1
$Br_2Ho_2O_2$	840	0.0007	118	81
Bi_2STe_2	155	0.0007	25	16
H_2Si_2	388	0.0008	64	49
Br_2Ca_3Si	102	0.0008	16	9
$Cl_2O_2Y_2$	711	0.0008	91	73
H_2MnO_2	353	0.0008	36	49
CrS_2	543	0.0008	81	100
In_2Se_3	327	0.0009	49	36
$Gd_2I_2S_2$	102	0.0009	16	9

Table showing the first 50 lattice matching combinations with the smallest strain needed to build the supercell.

CoI_2 (C2/m)

Structural and electronic properties

**Formula**	CoI_2
**Spacegroup**	C2/m
**Prototype**	CdI2
**Parent 3D**	CoI_2
**Source DB**	COD
**DB ID**	9009100
**DF2-C09 Binding energy [meV/ Å^2]**	18.26
**RVV10 Binding energy [meV/ Å^2]**	24.28
**Band gap (PBE) [eV]**	N/A

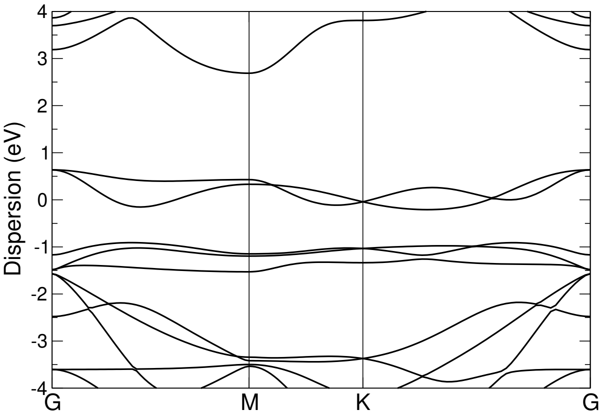

Band structure: Electronic band structure of CoI_2 (C2/m) in a representative window around the Fermi energy and along a high-symmetry path.

Optimized crystal structure

Structural parameters: relaxed cell (top) and atomic positions (bottom) of CoI_2 (C2/m) in Cartesian coordinates.

	x [Å]	y [Å]	z [Å]
$\mathbf{a_1}$	3.84721992	0.00000000	0.00000000
$\mathbf{a_2}$	−1.92360996	3.33179019	0.00000000
$\mathbf{a_3}$	0.00000000	0.00000000	22.93592357

		x [Å]	y [Å]	z [Å]
●	I	0.00000000	2.22119346	12.91426460
●	Co	0.00000000	0.00000000	11.46796178
●	I	1.92360996	1.11059673	10.02165897

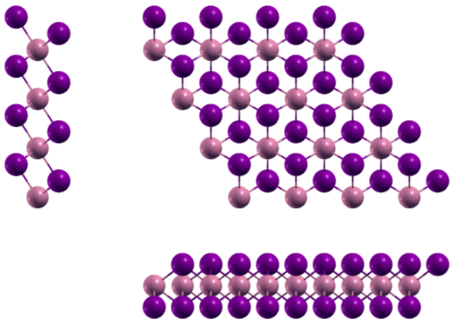

Orthographic projections: views of CoI_2 (C2/m) as seen from the x axis (left), the y axis (bottom) and the z axis (center).

Lattice matching - minimal size

Formula	N° atoms	strain	cell size 1	cell size 2
Tl	4	0.4272	1	1
InSe	5	0.453	1	1
Nd	5	0.2192	1	2
HgO	5	0.1125	1	1
AsSb	5	0.0004	1	1
Bi_2	5	0.4675	1	1
GeTe	5	0.0057	1	1
S_2	5	0.0073	1	1
P_2	5	1.5332	1	1
PbTe	5	0.4574	1	1
CaCl	5	0.1431	1	1
$IrTe_2$	6	0.0066	1	1
$CdCl_2$	6	0.0046	1	1
Cl_2Mn	6	0.2484	1	1
CdI_2	6	0.4617	1	1
$AgTe_2$	6	0.434	1	1
$MoSe_2$	6	1.5657	1	1
$ReSe_2$	6	0.2664	1	1
S_2Ta	6	1.6291	1	1
Br_2Ca	6	0.4647	1	1
CaI_2	6	2.9958	1	1
$InSe_2$	6	0.005	1	1
$GeTe_2$	6	0.0036	1	1
$SiTe_2$	6	0.0064	1	1
I_2Mn	6	0.0047	1	1
NSr_2	6	0.0012	1	1
I_2Yb	6	2.9622	1	1
PbS_2	6	0.003	1	1
BiClTe	6	0.4626	1	1
Cl_2Ti	6	1.5343	1	1
FeI_2	6	0.0025	1	1
I_2Ni	6	0.0038	1	1
S_2Ti	6	0.2579	1	1
NbS_2	6	0.2505	1	1
CrI_2	6	0.0021	1	1
BiBrTe	6	0.4766	1	1
Bi_2Pd	6	0.1165	1	1
Cl_2Ni	6	0.2677	1	1
Cl_2Co	6	0.2574	1	1
Br_2V	6	0.2652	1	1
ClNZr	6	0.2623	1	1
Cl_2Fe	6	0.2564	1	1
S_2Ta	6	1.5872	1	1
Br_2Ti	6	0.2758	1	1
$AsSe_2$	6	0.2698	1	1
$NiTe_2$	6	0.007	1	1
I_2Tm	6	2.9802	1	1
I_2V	6	0.0051	1	1
GeI_2	6	0.458	1	1
Se_2Zr	6	0.006	1	1

Table showing the first 50 lattice matching combinations with the smallest number of atoms in the supercell.

Lattice matching - minimal strain

Formula	N° atoms	strain	cell size 1	cell size 2
$CrSe_2$	183	0.0	25	36
CrO_2	222	0.0001	25	49
Cl_2Ni	543	0.0001	81	100
CaI_2	255	0.0001	49	36
Br_2La_2P	705	0.0001	100	81
NbS_2	300	0.0001	43	57
Cl_2Zr_2	583	0.0001	73	91
FHOZn	219	0.0002	25	36
Cl_2NSc_2	467	0.0002	49	64
CdI_2	543	0.0002	100	81
$Ga_2Gd_2I_2$	711	0.0002	91	73
Bi_2	419	0.0002	91	73
CdI_2	543	0.0002	100	81
BiBrTe	435	0.0002	81	64
$Br_2Cr_2O_2$	438	0.0003	48	49
GdI_2	390	0.0003	73	57
Gd_2GeI_2	705	0.0003	100	81
I_2Ti	6	0.0003	1	1
$Cl_2H_2Zr_2$	471	0.0004	43	57
AsSb	5	0.0004	1	1
$Cl_2Hg_2N_2$	732	0.0005	130	57
$NbSe_2$	543	0.0005	81	100
$Ga_2I_2Tb_2$	711	0.0005	91	73
$I_2S_2Tb_2$	564	0.0005	88	50
$Ge_2I_2La_2$	429	0.0005	57	43
CCl_2Sc_2	674	0.0005	73	91
BiClTe	543	0.0006	100	81
Se_2Ta	543	0.0006	81	100
I_2La_2P	504	0.0006	73	57
$I_2La_2O_2$	483	0.0006	65	48
Br_2V	492	0.0006	73	91
Bi_2STe_2	705	0.0006	100	81
Cl_2Mn	339	0.0006	49	64
I_2Pr	543	0.0006	100	81
BiITe	339	0.0007	64	49
Nd	196	0.0007	39	79
Cl_2Fe	390	0.0007	57	73
S_2Ta	300	0.0007	43	57
Bi_2Te_3	437	0.0007	64	49
K	435	0.0007	118	81
HN_3OZn	402	0.0008	36	49
CI_2Y_2	8	0.0008	1	1
Cl_2Cu	438	0.0008	72	74
$C_2Cl_2Y_2$	522	0.0008	58	58
IO_3Tl	227	0.0008	49	16
Bi_2SeTe_2	638	0.0008	91	73
$ReSe_2$	543	0.0008	81	100
Br_2Ca	492	0.0009	91	73
$Cl_2Cr_2O_2$	306	0.0009	32	35
$I_2Nd_2S_2$	258	0.0009	36	25

Table showing the first 50 lattice matching combinations with the smallest strain needed to build the supercell.

CoO_2 (P-3m1)

Structural and electronic properties

**Formula**	CoO_2
**Spacegroup**	P-3m1
**Prototype**	CdI2
**Parent 3D**	CoO_2
**Source DB**	COD
**DB ID**	1522027
**DF2-C09 Binding energy [meV/ Å^2]**	22.61
**RVV10 Binding energy [meV/ Å^2]**	34.25
**Band gap (PBE) [eV]**	N/A

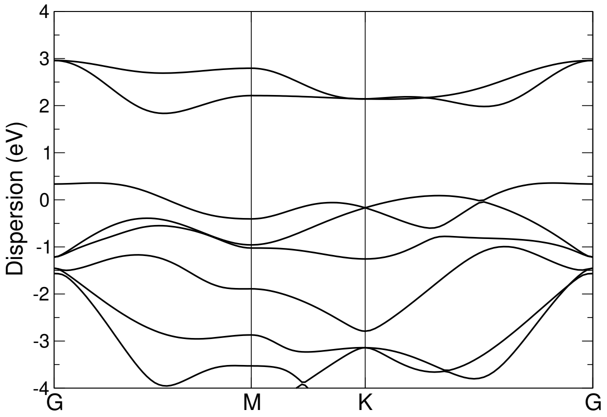

Band structure: Electronic band structure of CoO_2 (P-3m1) in a representative window around the Fermi energy and along a high-symmetry path.

Optimized crystal structure

Structural parameters: relaxed cell (top) and atomic positions (bottom) of CoO_2 (P-3m1) in Cartesian coordinates.

		x [Å]	y [Å]	z [Å]
$\mathbf{a_1}$		2.82097859	0.00000000	0.00000000
$\mathbf{a_2}$		−1.41048929	2.44303912	0.00000000
$\mathbf{a_3}$		0.00000000	0.00000000	21.86560631
		x [Å]	y [Å]	z [Å]
•	O	0.00000000	1.62869275	10.01277818
•	Co	0.00000000	0.00000000	10.93280316
•	O	1.41048929	0.81434637	11.85282813

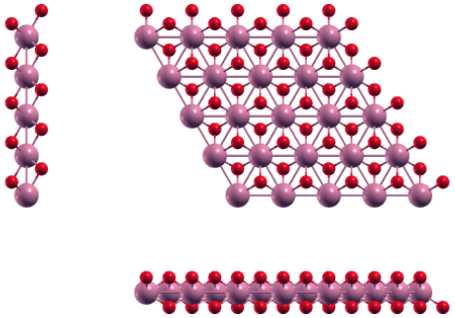

Orthographic projections: views of CoO_2 (P-3m1) as seen from the x axis (left), the y axis (bottom) and the z axis (center).

Lattice matching - minimal size

Formula	N° atoms	strain	cell size 1	cell size 2
In	4	0.471	1	1
LiO	5	0.4984	1	1
BN	5	0.2598	1	1
P_2	5	2.9836	1	1
C_2	5	0.2487	1	1
S_2V	6	0.4791	1	1
MoS_2	6	0.481	1	1
$MoSe_2$	6	3.0442	1	1
HfS_2	6	13.6246	1	1
FeO_2	6	0.0007	1	1
NiO_2	6	0.0005	1	1
ReS_2	6	0.4492	1	1
Cl_2Ti	6	2.9857	1	1
S_2W	6	0.4812	1	1
$CoTe_2$	6	13.6436	1	1
Cl_2V	6	2.8515	1	1
MoS_2	6	0.4816	1	1
$CrSe_2$	6	2.8363	1	1
$CrSe_2$	6	0.4951	1	1
O_2Pt	6	0.4671	1	1
N_2Re	6	0.0067	1	1
Ga_2S_2	7	13.6517	1	1
O_2Sn_2	7	1.3852	1	1
$SbSe_2Tl$	7	3.0134	1	1
CS_2Ta_2	8	2.9867	1	1
H_2NiO_2	8	0.4846	1	1
H_2MgO_2	8	0.4769	1	1
CNb_2S_2	8	2.9769	1	1
$HfTe_2$	9	0.2193	2	1
Br_2Cu	9	5.3042	1	2
$H_2Na_2O_2$	9	0.3207	1	1
Se_2Ta_4	9	0.3131	1	1
CNRb	9	0.2985	2	1
Ba_2N	9	0.2213	2	1
Te_2Zr	9	0.2199	2	1
CNNa	9	0.5627	2	1
Br_2Cd	9	0.225	2	1
K	10	0.1231	3	1
$AgNO_2$	10	0.2174	2	1
As_4	10	0.6072	2	1
Ho_2S_2	10	0.3027	2	1
Bi_2Te_2	11	6.1162	1	2
Bi_2Mn_2	11	1.8181	1	2
Br_2PY_2	11	0.2247	2	1
In_2Se_3	11	0.2193	2	1
ReS_2	12	0.1968	2	2
$C_2Br_2Gd_2$	12	0.9118	2	1
$Ag_2K_2Te_2$	12	0.52	2	1
Cu_3Se_3	12	0.2209	2	1
Cl_2V	12	0.8787	2	2

Table showing the first 50 lattice matching combinations with the smallest number of atoms in the supercell.

Lattice matching - minimal strain

Formula	N° atoms	strain	cell size 1	cell size 2
Cl_2Hf_2	208	0.0	36	25
Br_2Zn	75	0.0	16	9
$CuTe_2$	390	0.0	81	49
$PdTe_2$	354	0.0	79	39
O_2Pt	492	0.0001	91	73
$Cl_2N_2Zr_2$	405	0.0001	61	37
CCl_2Gd_2	93	0.0001	16	9
DyI_2	267	0.0001	64	25
$CrSe_2$	339	0.0001	64	49
Bi_2Se_2Te	47	0.0002	9	4
CaH_2O_2	368	0.0002	61	37
HN_3OZn	363	0.0002	49	36
Sb_2SeTe_2	47	0.0002	9	4
Mg_2	291	0.0002	65	48
$C_2Br_2Gd_2$	759	0.0002	127	63
Bi_2O_2	606	0.0002	130	54
In_2Te_3	47	0.0003	9	4
Te_2Ti	75	0.0003	16	9
$PtTe_2$	447	0.0003	100	49
BN	354	0.0003	64	81
$HfSe_2$	75	0.0003	16	9
H_2MgO_2	563	0.0003	81	64
$Fe_2Li_2P_2$	237	0.0003	39	20
HfS_2	390	0.0003	81	49
$Br_2La_2O_2$	714	0.0004	130	54
$Eu_2F_2I_2$	714	0.0004	130	54
$Al_2Cl_2O_2$	177	0.0004	27	16
$C_2Br_2Tb_2$	759	0.0004	127	63
C_2	275	0.0005	49	64
Ge_2S_2	528	0.0005	112	48
H_2NiO_2	504	0.0005	73	57
NiO_2	6	0.0005	1	1
$I_2S_2Tb_2$	342	0.0006	64	25
$Dy_2I_2S_2$	342	0.0006	64	25
$Br_2Hf_2N_2$	405	0.0006	61	37
$CuTe_2$	294	0.0006	61	37
GeI_2Y_2	47	0.0006	9	4
Br_2Tb_2	393	0.0006	79	39
MoS_2	390	0.0006	73	57
Br_2Ho_2	496	0.0006	100	49
$CoTe_2$	390	0.0007	81	49
CBr_2Y_2	93	0.0007	16	9
KNO_3	192	0.0007	49	9
$AuTe_2$	447	0.0007	100	49
FeO_2	6	0.0007	1	1
CS_2Ta_2	327	0.0007	49	36
S_2W	390	0.0008	73	57
Ga_2S_2	439	0.0008	81	49
Cl_2Ti	255	0.0008	49	36
$I_2Sb_2Te_2$	297	0.0008	69	15

Table showing the first 50 lattice matching combinations with the smallest strain needed to build the supercell.

Cr_2O_4 (Pmmn)

Structural and electronic properties

**Formula**	Cr_2O_4
**Spacegroup**	Pmmn
**Prototype**	FeO2
**Parent 3D**	Cr_2O_4
**Source DB**	MPDS
**DB ID**	S1718625
**DF2-C09 Binding energy [meV/ Å^2]**	10.77
**RVV10 Binding energy [meV/ Å^2]**	N/A
**Band gap (PBE) [eV]**	0.0

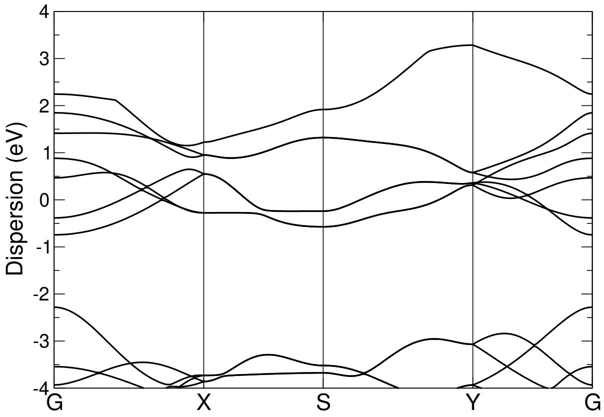

Band structure: Electronic band structure of Cr_2O_4 (Pmmn) in a representative window around the Fermi energy and along a high-symmetry path.

Optimized crystal structure

Structural parameters: relaxed cell (top) and atomic positions (bottom) of Cr_2O_4 (Pmmn) in Cartesian coordinates.

	x [Å]	y [Å]	z [Å]
$\mathbf{a_1}$	2.92404027	0.00000000	0.00000000
$\mathbf{a_2}$	0.00000000	3.66177558	0.00000000
$\mathbf{a_3}$	0.00000000	0.00000000	19.98117840

		x [Å]	y [Å]	z [Å]
●	Cr	0.73101007	-2.74634765	-1.06267859
●	Cr	-0.73101007	-4.57720351	1.06267859
•	O	-0.73101007	-2.74636595	-2.00983777
•	O	0.73101007	-4.57718520	2.00983777
•	O	-0.73101007	-2.74632290	0.48249107
•	O	0.73101007	-4.57722825	-0.48249107

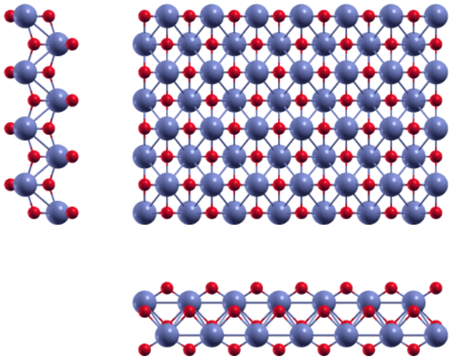

Orthographic projections: views of Cr_2O_4 (Pmmn) as seen from the x axis (left), the y axis (bottom) and the z axis (center).

Lattice matching - minimal size

Formula	N° atoms	strain	cell size 1	cell size 2
Tl	7	0.3166	1	1
InSe	8	0.277	1	1
HgO	8	0.0528	1	1
Bi_2	8	0.2875	1	1
PbTe	8	0.2801	1	1
I_2Mg	9	0.2591	1	1
CdI_2	9	0.8163	1	1
$AgTe_2$	9	0.3219	1	1
Br_2Ca	9	0.2855	1	1
$SiTe_2$	9	1.9156	1	1
Br_2La	9	0.2596	1	1
Ca_2Si	9	0.3682	1	1
BiClTe	9	0.8177	1	1
BrCdI	9	0.2644	1	1
Cl_2Zn	9	0.3651	1	1
BaF_2	9	0.2702	1	1
Bi_2Pd	9	0.056	1	1
GeI_2	9	0.2559	1	1
AsKSn	9	0.2677	1	1
$PbTe_2$	9	0.2627	1	1
$NiTe_2$	9	1.9104	1	1
$SnTe_2$	9	0.2522	1	1
I_2V	9	1.9274	1	1
GeI_2	9	0.2806	1	1
Se_2Zr	9	1.9192	1	1
I_2Pb	9	0.3621	1	1
STl_2	9	0.2718	1	1
$FeSe_2$	9	0.7612	1	1
CuO_2	9	0.0848	1	1
Se_2Yb	9	0.2563	1	1
$BiTe_2$	9	0.2568	1	1
F_2Ni	9	0.9062	1	1
CdI_2	9	0.2825	1	1
I_2Pr	9	0.8181	1	1
Bi_2Te_2	10	0.3978	1	1
$LiMnTe_2$	10	0.2811	1	1
Cu_2Te_2	10	0.2895	1	1
$AgCuTe_2$	10	0.5484	1	1
$AsLi_3$	10	0.2775	1	1
Ca_2O_2	10	0.0567	1	1
Cl_2OV	10	0.0421	1	1
Fe_2Se_2	10	0.9038	1	1
Cl_2ORu	10	0.0822	1	1
Cu_2Te_2	10	0.936	1	1
$AgBrO_2$	10	0.2075	1	1
Ga_2Se_2	10	1.9414	1	1
$MnNaTe_2$	10	0.2637	1	1
Br_2OV	10	0.3107	1	1
Bi_2Se_2	10	0.5115	1	1
Mg_4	10	0.316	1	1

Table showing the first 50 lattice matching combinations with the smallest number of atoms in the supercell.

Lattice matching - minimal strain

Formula	N° atoms	strain	cell size 1	cell size 2
Ba_2Hg	627	0.0005	80	49
CKN	525	0.0006	70	35
Br_2Ca_3Si	774	0.0006	80	49
$I_2N_2Zr_2$	600	0.0006	53	47
Cl_4KTl	606	0.0007	81	20
$RhTe_2$	459	0.0007	53	47
F_4Sn	725	0.0007	80	49
$Hf_2Si_2Te_2$	54	0.0008	5	4
$Fe_2Li_2P_2$	54	0.0008	5	4
HgO	642	0.0009	80	81
Bi_2Te_2	944	0.0011	116	62
$Cu_2Se_2Tl_2$	660	0.0011	67	43
N_3Na	456	0.0011	56	30
$BrKO_3$	435	0.0011	60	15
$NiTe_2$	807	0.0012	94	81
HgO	634	0.0012	79	80
$F_2I_2Pb_2$	972	0.0012	101	61
CaClHO	888	0.0012	94	81
Ba_2Cd	789	0.0012	101	61
$MoTe_2$	669	0.0013	75	73
$HfLiS_2$	742	0.0013	75	73
Co_2Se_2	46	0.0013	5	4
Se_2Ta	663	0.0013	73	75
Te_2W	669	0.0013	75	73
$NbSe_2$	663	0.0013	73	75
As_4	896	0.0014	106	65
$I_2N_2Zr_2$	780	0.0014	77	53
Cu_2I_2	574	0.0014	67	43
As_2Li_2Nd	151	0.0014	16	11
STl_2	945	0.0014	117	81
$SiTe_2$	807	0.0014	94	81
CdClHO	506	0.0014	53	47
Ca_2N	663	0.0014	75	71
Sb_2SeTe_2	917	0.0014	97	67
$AsSe_2$	663	0.0014	73	75
GeI_2Y_2	151	0.0014	16	11
In_2Te_3	917	0.0014	97	67
$I_2La_2Si_2$	162	0.0014	16	11
Bi_2Se_2Te	917	0.0015	97	67
$CrTe_2$	270	0.0015	30	30
As_2Li_2Pr	151	0.0015	16	11
InSe	118	0.0015	16	11
$Br_2F_2Pb_2$	618	0.0015	63	40
$Br_2Hf_2N_2$	876	0.0015	75	71
CdH_2O_2	815	0.0015	75	73
Au_2Br_2	936	0.0015	112	66
Cl_2Ni	663	0.0015	73	75
Cl_4Mn	607	0.0015	72	35
STl_2	816	0.0015	101	70
Cl_2Sc_2	300	0.0015	30	30

Table showing the first 50 lattice matching combinations with the smallest strain needed to build the supercell.

$CrBr_2$ (P-1)

Structural and electronic properties

**Formula**	$CrBr_2$
**Spacegroup**	P-1
**Prototype**	NbTe2
**Parent 3D**	$CrBr_2$
**Source DB**	ICSD
**DB ID**	23903
**DF2-C09 Binding energy [meV/ Å^2]**	15.55
**RVV10 Binding energy [meV/ Å^2]**	21.96
**Band gap (PBE) [eV]**	0.83

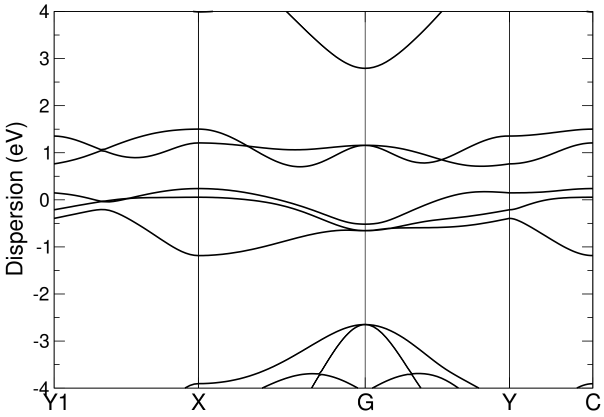

Band structure: Electronic band structure of $CrBr_2$ (P-1) in a representative window around the Fermi energy and along a high-symmetry path.

Optimized crystal structure

Structural parameters: relaxed cell (top) and atomic positions (bottom) of $CrBr_2$ (P-1) in Cartesian coordinates.

		x [Å]	y [Å]	z [Å]
$\mathbf{a_1}$		3.50602543	−0.00022118	0.00000000
$\mathbf{a_2}$		1.75347714	3.03603973	0.00000000
$\mathbf{a_3}$		0.00000000	0.00000000	23.03650683
		x [Å]	y [Å]	z [Å]
●	Br	1.76611950	1.01941577	13.02664741
●	Cr	0.01309878	0.00756068	11.51825436
●	Br	3.51956305	2.03151404	10.00985847

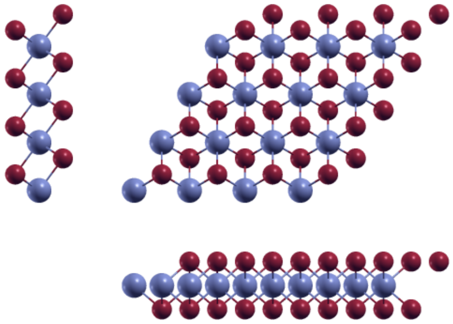

Orthographic projections: views of $CrBr_2$ (P-1) as seen from the x axis (left), the y axis (bottom) and the z axis (center).

Lattice matching - minimal size

Formula	N° atoms	strain	cell size 1	cell size 2
Tl	4	0.1295	1	1
Sn	4	0.1123	1	1
Na	4	0.0057	1	1
In	4	0.1139	1	1
In	4	0.2687	1	1
Gd	4	0.1125	1	1
HgO	5	0.1388	1	1
GeTe	5	0.4606	1	1
S_2	5	0.4645	1	1
Mg_2	5	0.1194	1	1
$IrTe_2$	6	0.4629	1	1
CrS_2	6	0.2483	1	1
S_2V	6	0.2733	1	1
MoS_2	6	0.2744	1	1
$CdCl_2$	6	0.4579	1	1
$MoTe_2$	6	0.0081	1	1
$AgTe_2$	6	0.1317	1	1
$ReSe_2$	6	0.0066	1	1
$InSe_2$	6	0.4589	1	1
$GeTe_2$	6	0.4555	1	1
$HfTe_2$	6	0.484	1	1
I_2Mn	6	0.4582	1	1
NSr_2	6	0.4497	1	1
ReS_2	6	0.2564	1	1
$PdTe_2$	6	0.498	1	1
FeI_2	6	0.4528	1	1
I_2Ni	6	0.4559	1	1
Mg_3	6	0.1255	1	1
CrI_2	6	0.4519	1	1
Te_2Zn	6	0.0079	1	1
S_2W	6	0.2745	1	1
Bi_2Pd	6	0.1472	1	1
Cl_2Ni	6	0.0057	1	1
$CrTe_2$	6	0.0003	1	1
PtS_2	6	0.0068	1	1
Br_2V	6	0.0074	1	1
CdClO	6	0.0096	1	1
Se_2Ti	6	0.0041	1	1
Br_2Ti	6	0.0003	1	1
Te_2Zr	6	0.4853	1	1
Te_2W	6	0.0082	1	1
$AsSe_2$	6	0.0043	1	1
BrNZr	6	0.003	1	1
$NbSe_2$	6	0.0053	1	1
Se_2Ta	6	0.0052	1	1
Br_2Mg	6	0.4525	1	1
I_2Ti	6	0.4476	1	1
$NbSe_2$	6	0.0041	1	1
MoS_2	6	0.2747	1	1
Se_2Ta	6	0.0011	1	1

Table showing the first 50 lattice matching combinations with the smallest number of atoms in the supercell.

Lattice matching - minimal strain

Formula	N° atoms	strain	cell size 1	cell size 2
P_2Sn_2	565	0.0	91	73
NS_2Ta	304	0.0	36	49
$AuTe_2$	300	0.0001	57	43
In_2S_3	705	0.0001	100	81
$Dy_2I_2S_2$	537	0.0001	81	49
Br_2Cd	339	0.0001	64	49
$Er_2F_2Se_2$	561	0.0001	73	57
$Br_2H_2Zr_2$	9	0.0001	1	1
Br_2PY_2	437	0.0001	64	49
$F_2Se_2Yb_2$	711	0.0001	91	73
$Ga_2I_2Y_2$	258	0.0002	36	25
$F_2Lu_2Se_2$	363	0.0002	49	36
Cl_2Sc_2	7	0.0002	1	1
$AlLiTe_2$	139	0.0002	25	16
GeTe	462	0.0003	100	81
Br_2Ti	6	0.0003	1	1
$CrTe_2$	6	0.0003	1	1
$Br_2O_2Yb_2$	483	0.0003	65	48
$HfTe_2$	390	0.0003	73	57
$Ce_2I_2S_2$	102	0.0003	16	9
Ca_2Si	75	0.0003	16	9
S_2Zn_2	565	0.0003	91	73
CoH_2O_2	536	0.0003	57	73
In_2Se_3	504	0.0003	73	57
$F_2Se_2Tm_2$	627	0.0004	81	64
Pt_2Te_2	447	0.0004	73	57
BaF_2	183	0.0004	36	25
$PtTe_2$	300	0.0004	57	43
I_4Zr_2	627	0.0005	115	47
$LiMnSe_2$	499	0.0005	81	64
C_2	275	0.0005	39	79
Br_2Er_2	343	0.0005	57	43
O_2Zn	300	0.0005	43	57
NaPSn	390	0.0006	73	57
H_2Si_2	624	0.0006	100	81
Br_2Cu	384	0.0006	72	56
$IrTe_2$	543	0.0006	100	81
CrS_2	339	0.0007	49	64
ReS_2	390	0.0007	57	73
$I_2O_2Y_2$	840	0.0007	118	81
$Dy_2I_2S_2$	405	0.0007	61	37
Fe_2Te_2	387	0.0007	65	48
DyI_2	390	0.0007	81	49
In	343	0.0007	81	100
O_2Pt	543	0.0008	81	100
$Br_2Nd_2O_2$	840	0.0008	118	81
$Cl_2O_2V_2$	441	0.0008	53	47
O_4PSn	237	0.0008	39	20
$BrKO_3$	17	0.0008	4	1
Ni_2SbTe_2	638	0.0008	91	73

Table showing the first 50 lattice matching combinations with the smallest strain needed to build the supercell.

CrI_2 ($P2_1/m$)

Structural and electronic properties

**Formula**	CrI_2
**Spacegroup**	$P2_1/m$
**Prototype**	CrI2
**Parent 3D**	CrI_2
**Source DB**	ICSD
**DB ID**	4073
**DF2-C09 Binding energy [meV/ Å^2]**	16.79
**RVV10 Binding energy [meV/ Å^2]**	22.45
**Band gap (PBE) [eV]**	0.72

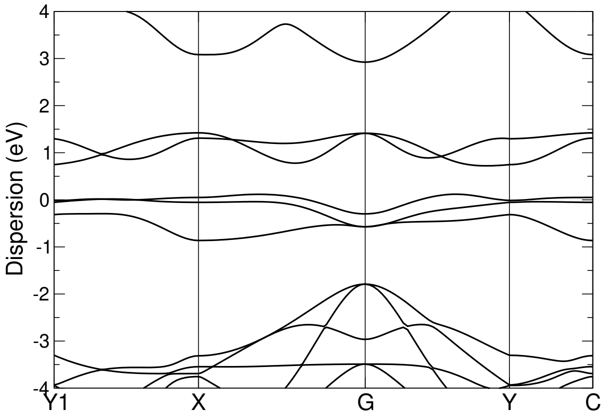

Band structure: Electronic band structure of CrI_2 ($P2_1/m$) in a representative window around the Fermi energy and along a high-symmetry path.

Optimized crystal structure

Structural parameters: relaxed cell (top) and atomic positions (bottom) of CrI_2 ($P2_1/m$) in Cartesian coordinates.

		x [Å]	y [Å]	z [Å]
$\mathbf{a_1}$		3.86401122	−0.00003630	0.00000000
$\mathbf{a_2}$		1.93130208	3.34673788	0.00000000
$\mathbf{a_3}$		0.00000000	0.00000000	23.10496863
		x [Å]	y [Å]	z [Å]
●	I	5.79384173	3.34585176	13.09728862
●	Cr	3.86208656	2.23029378	11.55248180
●	I	1.93033266	1.11473653	10.00768253

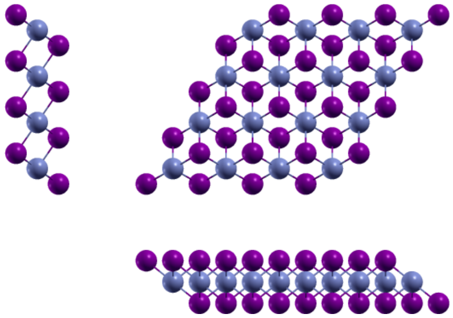

Orthographic projections: views of CrI_2 ($P2_1/m$) as seen from the x axis (left), the y axis (bottom) and the z axis (center).

Lattice matching - minimal size

Formula	N° atoms	strain	cell size 1	cell size 2
Tl	4	0.4224	1	1
InSe	5	0.4478	1	1
HgO	5	0.1118	1	1
AsSb	5	0.0025	1	1
Bi_2	5	0.4621	1	1
GeTe	5	0.0036	1	1
S_2	5	0.0052	1	1
PbTe	5	0.4521	1	1
CaCl	5	0.1412	1	1
$IrTe_2$	6	0.0045	1	1
$CdCl_2$	6	0.0025	1	1
CdI_2	6	0.4563	1	1
$AgTe_2$	6	0.4291	1	1
$ReSe_2$	6	0.2633	1	1
S_2Ta	6	0.2483	1	1
Br_2Ca	6	0.4594	1	1
$InSe_2$	6	0.0029	1	1
$GeTe_2$	6	0.0015	1	1
$SiTe_2$	6	0.0084	1	1
I_2Mn	6	0.0026	1	1
NSr_2	6	0.0009	1	1
PbS_2	6	0.005	1	1
BiClTe	6	0.4572	1	1
LiO_2	6	0.066	1	1
FeI_2	6	0.0004	1	1
I_2Ni	6	0.0017	1	1
S_2Ti	6	0.2549	1	1
NbS_2	6	0.2477	1	1
BiBrTe	6	0.4711	1	1
Bi_2Pd	6	0.1155	1	1
Cl_2Ni	6	0.2646	1	1
Cl_2Co	6	0.2545	1	1
$CrTe_2$	6	0.2735	1	1
Br_2V	6	0.2622	1	1
ClNZr	6	0.2592	1	1
Cl_2Fe	6	0.2535	1	1
Br_2Ti	6	0.2726	1	1
$AsSe_2$	6	0.2668	1	1
$NiTe_2$	6	0.009	1	1
I_2V	6	0.0072	1	1
GeI_2	6	0.4527	1	1
Se_2Zr	6	0.008	1	1
CdO_2	6	0.2543	1	1
BrNZr	6	0.2686	1	1
$NbSe_2$	6	0.2652	1	1
CoI_2	6	0.0021	1	1
GeS_2	6	0.1316	1	1
$MnSe_2$	6	0.1412	1	1
Br_2Cr	6	0.2732	1	1
Cl_2Zr	6	0.254	1	1

Table showing the first 50 lattice matching combinations with the smallest number of atoms in the supercell.

Lattice matching - minimal strain

Formula	N° atoms	strain	cell size 1	cell size 2
$Dy_2I_2S_2$	363	0.0001	49	36
$Ga_2Gd_2I_2$	786	0.0001	100	81
ClNZr	435	0.0001	64	81
NS_2Ta	471	0.0001	49	81
$I_2Pr_2S_2$	258	0.0001	36	25
I_2La_2P	563	0.0001	81	64
Sb_2Te_3	638	0.0002	91	73
Br_2Ca_3Si	483	0.0002	65	48
Hg_3N_2	368	0.0002	81	25
NaO_4	93	0.0002	16	9
AsI_2La_2	437	0.0002	64	49
MnO_2	222	0.0002	25	49
Ba_2Hg	339	0.0002	65	48
Br_2Mg	6	0.0003	1	1
Cd_2I_3	437	0.0003	64	49
Cl_2Ni	492	0.0003	73	91
$NbSe_2$	543	0.0003	81	100
$Ga_2I_2Tb_2$	786	0.0003	100	81
S_2Ti	390	0.0003	57	73
Nd	247	0.0003	49	100
Br_2Hf_2	643	0.0003	81	100
FeI_2	6	0.0004	1	1
$CrSe_2$	183	0.0004	25	36
Bi_2	462	0.0004	100	81
Cu_4Te_2	711	0.0004	91	73
C	129	0.0004	16	81
N_4	212	0.0005	24	35
$MoSe_2$	255	0.0005	36	49
O_2Sn_2	357	0.0005	55	48
Cl_2Hf_2	403	0.0006	49	64
$AsSe_2$	543	0.0006	81	100
$LiNbS_2$	403	0.0006	49	64
$Br_2H_2Zr_2$	678	0.0006	64	81
$ReSe_2$	492	0.0006	73	91
S_2Ta	339	0.0007	49	64
Ca_4Cu_2	624	0.0007	102	53
Br_2Ca	543	0.0007	100	81
CoH_2O_2	173	0.0007	16	25
NS_2Ta	355	0.0007	37	61
Cl_2Co	390	0.0007	57	73
DyI_2	255	0.0007	49	36
Se_2W	255	0.0007	36	49
$NbSe_2$	492	0.0007	73	91
In	361	0.0007	81	118
Cl_2Mn	300	0.0008	43	57
$AlLiTe_2$	447	0.0008	73	57
BrNZr	543	0.0008	81	100
$LiOS_2Ti$	414	0.0008	43	57
Se_2Ta	492	0.0008	73	91
I_2Pb	183	0.0008	36	25

Table showing the first 50 lattice matching combinations with the smallest strain needed to build the supercell.

CrO_2 (P-3m1)

Structural and electronic properties

**Formula**	CrO_2
**Spacegroup**	P-3m1
**Prototype**	CdI2
**Parent 3D**	Cr_2O_4
**Source DB**	MPDS
**DB ID**	S1718625
**DF2-C09 Binding energy [meV/ Å^2]**	10.77
**RVV10 Binding energy [meV/ Å^2]**	N/A
**Band gap (PBE) [eV]**	0.0

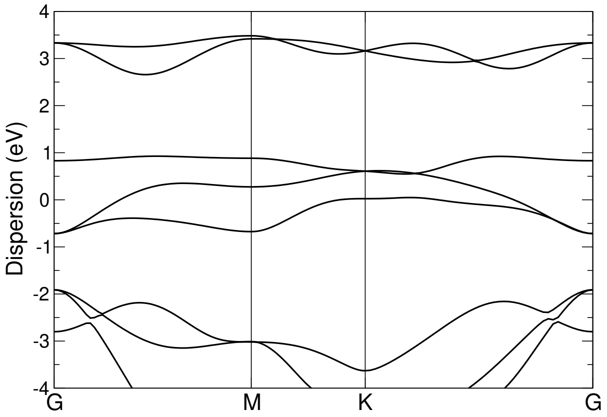

Band structure: Electronic band structure of CrO_2 (P-3m1) in a representative window around the Fermi energy and along a high-symmetry path.

Optimized crystal structure

Structural parameters: relaxed cell (top) and atomic positions (bottom) of CrO_2 (P-3m1) in Cartesian coordinates.

	x [Å]	y [Å]	z [Å]
$\mathbf{a_1}$	1.37418662	−2.38016105	0.00000000
$\mathbf{a_2}$	1.37418662	2.38016105	0.00000000
$\mathbf{a_3}$	0.00000000	0.00000000	16.26578325

		x [Å]	y [Å]	z [Å]
•	O	0.68709331	0.39669351	1.06255303
•	O	-0.68709331	-0.39669351	-1.06255303
•	Cr	0.68709331	-1.19008053	0.00000000

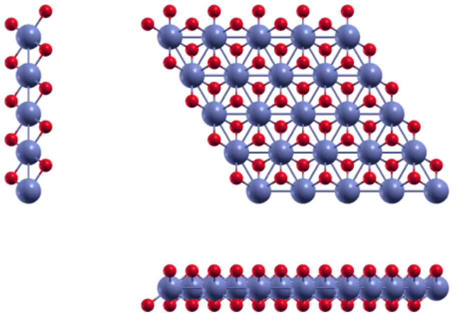

Orthographic projections: views of CrO_2 (P-3m1) as seen from the x axis (left), the y axis (bottom) and the z axis (center).

Lattice matching - minimal size

Formula	N° atoms	strain	cell size 1	cell size 2
Nd	4	0.0071	1	1
Gd	4	0.4876	1	1
C_2	5	0.2655	1	1
CrS_2	6	0.4649	1	1
ReS_2	6	0.4801	1	1
MnO_2	6	0.0023	1	1
N_2W	6	0.448	1	1
O_2Zn	6	0.4565	1	1
O_2Pt	6	0.4991	1	1
CoH_2O_2	8	0.4775	1	1
GeTe	8	0.2233	2	1
S_2	8	0.2253	2	1
FeH_2O_2	8	0.451	1	1
$IrTe_2$	9	0.2244	2	1
$CdCl_2$	9	0.2219	2	1
$InSe_2$	9	0.2224	2	1
$GeTe_2$	9	0.2206	2	1
I_2Mn	9	0.222	2	1
Ca_2Si	9	6.1052	1	2
FeI_2	9	0.2192	2	1
I_2Ni	9	0.2208	2	1
CrI_2	9	0.2187	2	1
Br_2Mg	9	0.219	2	1
H_2Si_2	10	0.2228	2	1
K	10	0.1313	3	1
Cu_2O_2	10	0.8622	2	1
$AgCuTe_2$	10	0.5561	2	1
Bi_2Se_2	10	2.0388	2	1
Ga_2Se_2	10	0.2247	2	1
Ni_2Te_2	10	0.2216	2	1
Ho_2S_2	10	0.3248	2	1
Sb_2Te_2	11	6.071	1	2
$CuGeO_3$	11	0.8782	2	1
In_2S_3	11	0.2235	2	1
Ni_2SbTe_2	11	0.2255	2	1
I_2Pr	12	0.425	3	1
HgI_2	12	0.1453	3	1
Bi_2Pd	12	0.1577	3	1
CKN	12	0.1743	3	1
I_2Nd	12	0.4272	3	1
Cu_2O_4	12	0.5235	2	1
O_2Zn	12	0.2008	2	2
CeI_2	12	0.4233	3	1
$Cl_2Er_2H_2$	12	0.2222	2	1
Ba_2Cd	12	0.1231	3	1
Cu_2I_2	13	0.1162	3	1
Ir_2P_2	13	0.4267	3	1
Bi_2Mn_2	13	0.1173	3	1
S_2Sn_2	13	0.1219	3	1
Au_2Br_2	13	0.1199	3	1

Table showing the first 50 lattice matching combinations with the smallest number of atoms in the supercell.

Lattice matching - minimal strain

Formula	N° atoms	strain	cell size 1	cell size 2
$CrSe_2$	255	0.0	49	36
Br_2Zr_2	439	0.0	81	49
ClNZr	123	0.0001	25	16
CoI_2	222	0.0001	49	25
FHOZn	291	0.0001	49	36
I_2La_2Sb	120	0.0001	25	9
Ge_2S_2	421	0.0001	91	37
$C_2Cl_2Y_2$	333	0.0002	55	28
Cl_2Gd_2	496	0.0002	100	49
$Br_2Cr_2O_2$	294	0.0003	48	25
I_2Ti	222	0.0003	49	25
P_2Sn_2	393	0.0003	79	39
BH_4Li	51	0.0003	9	4
Se_2Ti	390	0.0004	81	49
$CeLi_2P_2$	47	0.0004	9	4
C	139	0.0004	25	64
$I_2N_2Ti_2$	522	0.0004	90	42
$F_2Se_2Yb_2$	471	0.0004	79	39
Ni_2SbTe_2	432	0.0005	79	39
K	84	0.0005	25	9
AsSb	197	0.0005	49	25
$Br_2H_2Zr_2$	171	0.0005	25	16
CBr_2Lu_2	93	0.0005	16	9
Cl_2Tb_2	496	0.0005	100	49
CoH_2O_2	563	0.0006	81	64
$SnTe_2$	39	0.0006	9	4
In	214	0.0006	57	43
$Br_2N_2Zr_2$	102	0.0006	16	9
Br_2Zr_2	331	0.0006	61	37
S_2	315	0.0006	79	39
$H_2Li_2O_2$	237	0.0007	39	20
S_2Zn_2	393	0.0007	79	39
$NbTe_2$	75	0.0007	16	9
CI_2Y_2	272	0.0007	49	25
BiITe	267	0.0007	64	25
Br_2HLa	43	0.0007	9	4
Bi_2Te_3	317	0.0008	64	25
CrS_2	492	0.0008	91	73
HN_3OZn	258	0.0008	36	25
In	243	0.0008	65	48
$I_2S_2Sm_2$	129	0.0008	25	9
C_2	401	0.0008	73	91
CCl_2Sc_2	155	0.0009	25	16
Se_2Sn	222	0.0009	49	25
S_2Zr	75	0.0009	16	9
Cl_2Cu	417	0.001	91	48
Se_2Ti	294	0.001	61	37
Cl_2Cu	270	0.001	59	31
Bi_2Te_2	211	0.0011	49	16
I_2Y_2	496	0.0011	100	49

Table showing the first 50 lattice matching combinations with the smallest strain needed to build the supercell.

CrOBr (Pmmn)

Structural and electronic properties

**Formula**	CrOBr
**Spacegroup**	Pmmn
**Prototype**	FeOCl
**Parent 3D**	$Cr_2O_2Br_2$
**Source DB**	ICSD
**DB ID**	27092
**DF2-C09 Binding energy [meV/ Å^2]**	14.79
**RVV10 Binding energy [meV/ Å^2]**	23.05
**Band gap (PBE) [eV]**	0.49

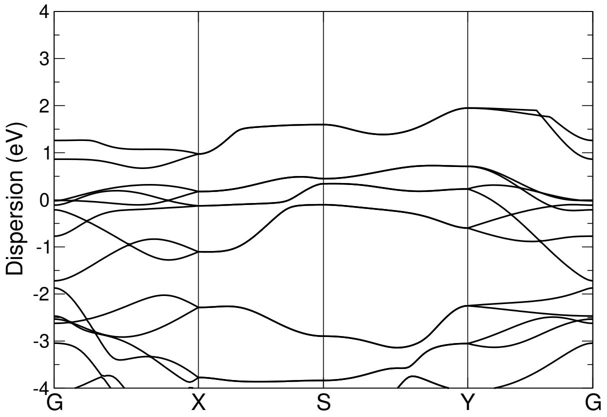

Band structure: Electronic band structure of CrOBr (Pmmn) in a representative window around the Fermi energy and along a high-symmetry path.

Optimized crystal structure

Structural parameters: relaxed cell (top) and atomic positions (bottom) of CrOBr (Pmmn) in Cartesian coordinates.

		x [Å]	y [Å]	z [Å]
$\mathbf{a_1}$		3.30018671	0.00000000	0.00000000
$\mathbf{a_2}$		0.00000000	3.80651337	0.00000000
$\mathbf{a_3}$		0.00000000	0.00000000	24.95301425
		x [Å]	y [Å]	z [Å]
●	Cr	1.65009335	1.90325669	11.81680396
●	Br	0.00000000	1.90325669	9.97260052
•	O	1.65009335	0.00000000	11.90708766
●	Cr	0.00000000	0.00000000	13.13619615
●	Br	1.65009335	0.00000000	14.98041008
•	O	0.00000000	1.90325669	13.04593936

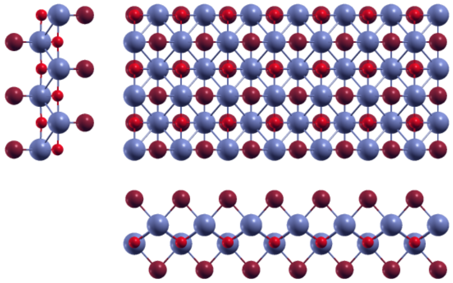

Orthographic projections: views of CrOBr (Pmmn) as seen from the x axis (left), the y axis (bottom) and the z axis (center).

Lattice matching - minimal size

Formula	N° atoms	strain	cell size 1	cell size 2
K	8	3.4781	1	2
InSe	8	0.2053	1	1
S_2	8	0.5643	1	1
PbTe	8	0.2077	1	1
CaI_2	9	0.2449	1	1
I_2Pr	9	0.347	1	1
Cl_2Zn	9	0.3023	1	1
$PdTe_2$	9	0.6035	1	1
BiBrTe	9	0.2187	1	1
Bi_2Pd	9	0.0405	1	1
Ba_2Hg	9	1.0707	1	1
I_2Nd	9	0.3489	1	1
I_2Tm	9	0.2429	1	1
GeI_2	9	0.2081	1	1
STl_2	9	0.2013	1	1
CeI_2	9	0.3455	1	1
F_2Ni	9	0.2954	1	1
Br_2Cd	9	0.6006	1	1
CdI_2	9	0.2095	1	1
Fe_2Te_2	10	0.0546	1	1
Li_2Tl_2	10	0.3514	1	1
Ca_2Cl_2	10	0.055	1	1
Cl_2Gd_2	10	0.5708	1	1
$LiMnTe_2$	10	0.2085	1	1
Cu_2Te_2	10	0.0192	1	1
Ir_2P_2	10	0.3485	1	1
$AgCuTe_2$	10	0.4261	1	1
$AsLi_3$	10	0.2057	1	1
O_2Sn_2	10	0.3096	1	1
Cu_2S_2	10	0.06	1	1
Au_2Br_2	10	0.5369	1	1
Ca_2O_2	10	0.0399	1	1
Cl_2OV	10	0.0178	1	1
Br_2Tb_2	10	0.6053	1	1
Fe_2Se_2	10	0.2945	1	1
Cu_2Te_2	10	0.3068	1	1
$AgBrO_2$	10	0.1457	1	1
S_2Zn_2	10	0.5681	1	1
Ge_2S_2	10	1.1347	1	1
C_2Li_2	10	0.2489	1	1
P_2Sn_2	10	0.5671	1	1
O_2Sn_2	10	0.0605	1	1
$LiMnSe_2$	10	0.5763	1	1
N_4	10	0.146	1	1
$AsCuLi_2$	10	0.6472	1	1
P_2Rh_2	10	0.0593	1	1
F_2Tl_2	10	0.0594	1	1
Br_2Ho_2	10	0.6068	1	1
Au_2I_2	10	0.6056	1	1
Cu_2Se_2	10	0.2964	1	1

Table showing the first 50 lattice matching combinations with the smallest number of atoms in the supercell.

Lattice matching - minimal strain

Formula	N° atoms	strain	cell size 1	cell size 2
$I_2Lu_2Se_2$	360	0.0002	40	20
$GeNi_3Te_2$	624	0.0003	54	50
$Cl_2Er_2H_2$	894	0.0003	76	73
CrO_2	294	0.0003	25	48
I_2Mn	675	0.0003	76	73
CoI_2	438	0.0003	49	48
Br_2Pr_2	484	0.0003	48	49
$InSe_2$	675	0.0003	76	73
$CdCl_2$	675	0.0003	76	73
I_2Ti	438	0.0004	49	48
Ni_2Te_2	748	0.0005	76	73
$RhTe_2$	666	0.0005	73	76
H_2Si_2	748	0.0006	76	73
AsSb	390	0.0006	49	48
NaPSn	474	0.0006	54	50
CBr_2Lu_2	570	0.0006	50	54
LiO_2	777	0.0007	80	99
$Br_2N_2Zr_2$	624	0.0007	50	54
$NbTe_2$	462	0.0007	50	54
H_2MnO_2	231	0.0007	16	27
$I_2N_2Zr_2$	894	0.0007	73	76
CI_2Y_2	534	0.0007	49	48
O_2Sn_2	904	0.0007	90	91
$HfSe_2$	435	0.0007	48	49
Se_2Sn_2	868	0.0008	100	67
Te_2Ti	435	0.0008	48	49
Pt_2Te_2	524	0.0008	54	50
La_2S_2	592	0.0008	68	46
BiITe	33	0.0008	4	3
Bi_2Te_3	39	0.0009	4	3
$C_2Br_2Y_2$	768	0.0009	66	62
Se_2Sn	438	0.0009	49	48
S_2Zr	462	0.0009	50	54
HN_3OZn	42	0.0009	3	4
CoH_2O_2	707	0.0009	52	79
GeTe	602	0.0009	76	73
$Cl_2O_2Yb_2$	582	0.0009	48	49
$Ba_2F_2I_2$	678	0.0009	70	43
F_2Na	435	0.001	48	49
Cl_4Pd_2	396	0.001	45	21
O_2Sn_2	904	0.001	90	91
I_2Ni	675	0.001	76	73
Br_2Zn	435	0.001	48	49
Au_2Br_2	746	0.0011	85	59
Gd	427	0.0011	53	109
$I_2S_2Tb_2$	588	0.0011	63	35
$Gd_2I_2S_2$	588	0.0011	63	35
O_2Sn_2	794	0.0011	79	80
In_2S_3	821	0.0011	76	73
NSr_2	438	0.0011	49	48

Table showing the first 50 lattice matching combinations with the smallest strain needed to build the supercell.

CrOCl (Pmmn)

Structural and electronic properties

**Formula**	CrOCl
**Spacegroup**	Pmmn
**Prototype**	FeOCl
**Parent 3D**	$Cr_2O_2Cl_2$
**Source DB**	ICSD
**DB ID**	4086
**DF2-C09 Binding energy [meV/ Å^2]**	13.8
**RVV10 Binding energy [meV/ Å^2]**	22.25
**Band gap (PBE) [eV]**	0.64

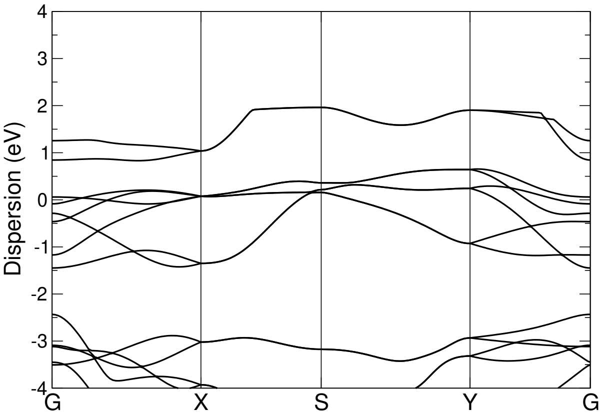

Band structure: Electronic band structure of CrOCl (Pmmn) in a representative window around the Fermi energy and along a high-symmetry path.

Optimized crystal structure

Structural parameters: relaxed cell (top) and atomic positions (bottom) of CrOCl (Pmmn) in Cartesian coordinates.

	x [Å]	y [Å]	z [Å]
$\mathbf{a_1}$	3.08329546	0.00000000	0.00000000
$\mathbf{a_2}$	0.00000000	3.81515344	0.00000000
$\mathbf{a_3}$	0.00000000	0.00000000	24.82003535

		x [Å]	y [Å]	z [Å]
●	Cr	0.00000000	0.00000000	13.13358777
●	Cr	1.54164773	1.90757672	11.68644627
●	Cl	1.54164773	0.00000000	14.83958546
●	Cl	0.00000000	1.90757672	9.98045108
●	O	1.54164773	0.00000000	11.77692703
●	O	0.00000000	1.90757672	13.04310951

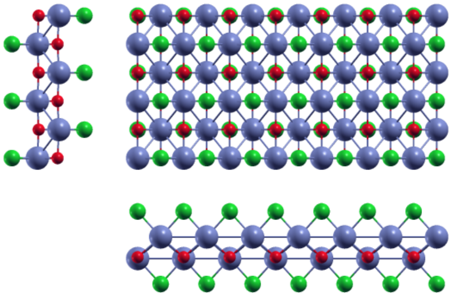

Orthographic projections: views of CrOCl (Pmmn) as seen from the x axis (left), the y axis (bottom) and the z axis (center).

Lattice matching - minimal size

Formula	N° atoms	strain	cell size 1	cell size 2
Bi_2	8	0.2468	1	1
CdI_2	9	0.2431	1	1
Br_2Ca	9	0.2451	1	1
CaI_2	9	0.2821	1	1
I_2Pr	9	0.3702	1	1
I_2Yb	9	0.2775	1	1
BiClTe	9	0.2437	1	1
BiBrTe	9	0.2527	1	1
Bi_2Pd	9	0.3122	1	1
Ba_2N	9	1.9271	1	1
Te_2Zr	9	1.9179	1	1
I_2Nd	9	0.3722	1	1
I_2Tm	9	0.2799	1	1
BiITe	9	0.2638	1	1
GeS_2	9	0.2767	1	1
CeI_2	9	0.3686	1	1
$FeSe_2$	9	0.6735	1	1
GdI_2	9	0.2575	1	1
CdI_2	9	0.2424	1	1
F_2Zn	9	0.922	1	1
I_2Pr	9	0.2438	1	1
Fe_2Te_2	10	0.0868	1	1
Ca_2Cl_2	10	0.0872	1	1
Cu_2Sr_2	10	0.2544	1	1
Ir_2P_2	10	0.3718	1	1
$AgCuTe_2$	10	0.4838	1	1
Pb_2Se_2	10	0.5077	1	1
Ca_2O_2	10	0.3144	1	1
$AlLiTe_2$	10	0.2618	1	1
Br_2Cu_2	10	0.916	1	1
Cl_2ORu	10	0.2946	1	1
As_2Co_2	10	0.3053	1	1
Ge_2S_2	10	1.2421	1	1
C_2Li_2	10	0.2739	1	1
Br_2OV	10	0.2742	1	1
Fe_2S_2	10	0.2932	1	1
Au_2I_2	10	0.6935	1	1
As_2Fe_2	10	0.2781	1	1
As_2Ru_2	10	0.0874	1	1
Ni_2Se_2	10	0.3681	1	1
Ba_2Cu_2	10	0.2772	1	1
O_2Sn_2	10	0.2269	1	1
Co_2Se_2	10	0.3078	1	1
AsI_2La_2	11	0.2689	1	1
Bi_2Te_3	11	0.2637	1	1
GeI_2La_2	11	0.2794	1	1
H_2Na_2Pd	11	0.2755	1	1
I_2La_2P	11	0.2559	1	1
Cd_2I_3	11	0.269	1	1
Sb_2Te_3	11	0.2496	1	1

Table showing the first 50 lattice matching combinations with the smallest number of atoms in the supercell.

Lattice matching - minimal strain

Formula	N° atoms	strain	cell size 1	cell size 2
Pt_2Te_2	766	0.0	81	70
Se_2Sn	306	0.0001	35	32
CI_2Y_2	370	0.0001	35	32
NaPSn	696	0.0002	81	70
NSr_2	306	0.0003	35	32
$Er_2F_2Se_2$	906	0.0005	81	70
I_2Ti	306	0.0005	35	32
$HfTe_2$	696	0.0007	81	70
In_2Se_3	836	0.0007	81	70
$GeNi_3Te_2$	906	0.0008	81	70
Cl_2Zr	837	0.0009	88	103
CoI_2	306	0.0009	35	32
Cl_2Fe	837	0.0009	88	103
CdO_2	837	0.0009	88	103
Cl_2Co	837	0.001	88	103
CrO_2	456	0.0011	40	72
$IrTe_2$	807	0.0011	93	83
Ga_2Se_2	890	0.0011	93	83
CaClHO	940	0.0012	96	91
Mo_2Te_4	414	0.0012	45	24
S_2Ti	837	0.0012	88	103
Te_2Zr	696	0.0012	81	70
N_4	34	0.0012	3	4
CrI_2	306	0.0012	35	32
ClNZr	663	0.0012	70	81
$I_2Lu_2Se_2$	282	0.0012	32	15
Br_2Zr_2	332	0.0012	32	35
$NiTe_2$	849	0.0013	96	91
H_2MnO_2	501	0.0013	36	57
AsSb	274	0.0013	35	32
Br_2Cu	945	0.0013	110	95
In_2S_3	973	0.0013	93	83
Ni_2SbTe_2	973	0.0014	93	83
NaO_4	300	0.0014	35	18
Li_2Tl_2	578	0.0014	71	38
AsI_2La_2	95	0.0014	10	7
GeTe	724	0.0015	93	83
Br_2Mg	306	0.0015	35	32
Cd_2I_3	95	0.0015	10	7
$As_2Mg_2Na_2$	588	0.0015	60	38
BiITe	81	0.0015	10	7
F_2Na	849	0.0016	96	91
FeI_2	306	0.0016	35	32
Bi_2Te_3	95	0.0016	10	7
C_4Ca_2	744	0.0016	73	51
P_2Sn_2	936	0.0016	98	87
$BrKO_3$	943	0.0016	128	35
$SiTe_2$	849	0.0016	96	91
Se_2Ti	297	0.0016	32	35
$Br_2Hf_2N_2$	954	0.0016	78	81

Table showing the first 50 lattice matching combinations with the smallest strain needed to build the supercell.

CrSBr (Pmmn)

Structural and electronic properties

**Formula**	CrSBr
**Spacegroup**	Pmmn
**Prototype**	FeOCl
**Parent 3D**	$Cr_2S_2Br_2$
**Source DB**	ICSD
**DB ID**	69659
**DF2-C09 Binding energy [meV/ Å^2]**	19.53
**RVV10 Binding energy [meV/ Å^2]**	27.17
**Band gap (PBE) [eV]**	0.44

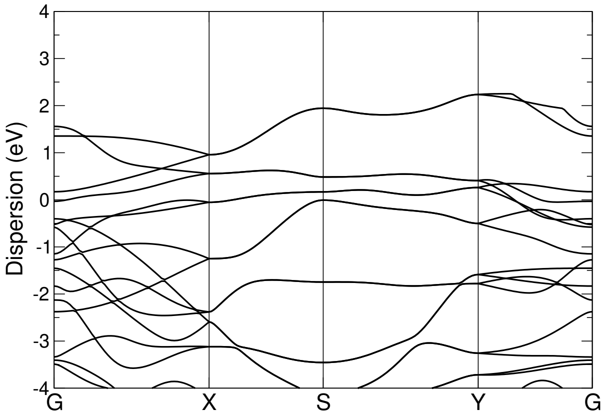

Band structure: Electronic band structure of CrSBr (Pmmn) in a representative window around the Fermi energy and along a high-symmetry path.

Optimized crystal structure

Structural parameters: relaxed cell (top) and atomic positions (bottom) of CrSBr (Pmmn) in Cartesian coordinates.

		x [Å]	y [Å]	z [Å]
$\mathbf{a_1}$		3.33683899	0.00000000	0.00000000
$\mathbf{a_2}$		0.00000000	4.53335096	0.00000000
$\mathbf{a_3}$		0.00000000	0.00000000	25.16942323
		x [Å]	y [Å]	z [Å]
●	Cr	1.66841950	0.00000000	11.97510005
●	S	1.66841950	2.26667548	11.60933270
●	Br	0.00000000	0.00000000	9.99829204
●	Cr	0.00000000	2.26667548	13.19432318
●	S	0.00000000	0.00000000	13.56009053
●	Br	1.66841950	2.26667548	15.17113120

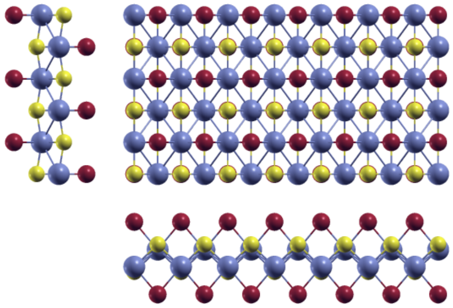

Orthographic projections: views of CrSBr (Pmmn) as seen from the x axis (left), the y axis (bottom) and the z axis (center).

Lattice matching - minimal size

Formula	N° atoms	strain	cell size 1	cell size 2
K	8	2.6522	1	2
Sm	8	0.136	1	2
CrS_2	9	0.1193	1	1
I_2Pr	9	0.0752	1	1
Br_2Cu	9	0.7362	1	1
I_2Nd	9	0.0757	1	1
CeI_2	9	0.0748	1	1
CuO_2	9	0.1772	1	1
InSe	10	1.0292	1	2
Cu_2Te_2	10	0.345	1	1
Ir_2P_2	10	0.0756	1	1
$AgNO_2$	10	0.0299	1	1
O_2Sn_2	10	0.0966	1	1
Bi_2	10	1.0586	1	2
Ca_2O_2	10	0.747	1	1
As_4	10	0.2493	1	1
C_2Li_2	10	0.1939	1	1
P_4	10	0.0068	1	1
Fe_2S_2	10	0.8922	1	1
Au_2I_2	10	0.9119	1	1
$AgClO_2$	10	0.3873	1	1
La_2S_2	10	0.3256	1	1
PbTe	10	1.0381	1	2
Ni_2Se_2	10	0.0747	1	1
Sn_2Te_2	10	0.9411	1	1
O_2Sn_2	10	0.0766	1	1
NaO_4	11	0.314	1	1
$AgNO_3$	11	0.3156	1	1
Cl_4Mn	11	0.4243	1	1
CdI_2	12	1.0469	1	2
Br_2Ca	12	1.0531	1	2
$Cl_2O_2Sc_2$	12	0.356	1	1
AlH_4Na	12	0.4305	1	1
$I_2N_2Zr_2$	12	0.2392	1	1
S_2Zr	12	0.3443	1	2
Ca_2Si	12	2.7706	1	2
BiClTe	12	1.0487	1	2
$Br_2O_2Sc_2$	12	0.3619	1	1
$C_2Br_2Gd_2$	12	0.6397	1	1
$I_2O_2Yb_2$	12	0.0754	1	1
$Bi_2Cl_2O_2$	12	0.0741	1	1
$Br_2Eu_2O_2$	12	0.0748	1	1
$Cl_2N_2Ti_2$	12	0.3357	1	1
GeI_2	12	1.0393	1	2
Ca_4Cu_2	12	5.3438	1	1
O_4PSn	12	0.9415	1	1
$Br_2Lu_2S_2$	12	1.2862	1	1
I_2Pb	12	2.7375	1	2
$Br_2O_2Sm_2$	12	0.0756	1	1
O_2Zn	12	0.5835	1	2

Table showing the first 50 lattice matching combinations with the smallest number of atoms in the supercell.

Lattice matching - minimal strain

Formula	N° atoms	strain	cell size 1	cell size 2
AuI_4Li	816	0.0004	92	44
$I_2Lu_2S_2$	432	0.0005	42	30
$Cl_2Ho_2O_2$	846	0.0005	64	77
$Bi_2Cl_2O_2$	504	0.0005	42	42
C_4Ca_2	636	0.0006	56	50
Ag_2Te_2	548	0.0007	54	56
CdClHO	966	0.0008	87	111
AuI_4Li	408	0.0008	46	22
$Ca_2Ge_2Mn_2$	504	0.0009	42	42
$I_2S_2Tb_2$	942	0.0009	94	63
SSb_2Te_2	440	0.001	40	40
$MnNaTe_2$	400	0.001	40	40
BrCdI	360	0.001	40	40
$Er_2I_2S_2$	354	0.001	35	24
$Br_2F_2Tm_2$	504	0.001	42	42
$I_2Lu_2O_2$	504	0.0011	42	42
Ni_2Se_2	420	0.0011	42	42
$AsSn_2$	855	0.0012	87	111
LiO_2	282	0.0012	27	40
$PbTe_2$	360	0.0012	40	40
PbS_2	699	0.0012	73	87
CeI_2	378	0.0013	42	42
CrS_2	474	0.0014	41	76
Br_2Y_2	692	0.0014	64	77
Br_2Ca_3Si	960	0.0014	87	73
Ca_2Si	990	0.0014	118	94
O_2Pt	543	0.0014	48	85
$Br_2Eu_2O_2$	504	0.0014	42	42
I_2Zn	705	0.0014	77	81
I_2La_2Te	887	0.0014	87	73
$I_2S_2Sm_2$	960	0.0015	87	73
$Ge_2Te_2Zr_2$	558	0.0015	45	48
$Ba_2Ge_2Mn_2$	558	0.0015	48	45
$Ga_2I_2Tb_2$	948	0.0015	81	77
$Br_2Lu_2O_2$	558	0.0015	45	48
$Cl_2H_2Sc_2$	582	0.0015	40	57
I_3Sn	688	0.0016	88	40
Br_2Ti	411	0.0016	40	57
Se_2Ta	411	0.0016	40	57
$F_2I_2Yb_2$	558	0.0016	48	45
F_2Tl_2	828	0.0016	82	84
P_2Rh_2	828	0.0016	82	84
PbS_2	615	0.0016	64	77
$Ga_2Gd_2I_2$	948	0.0016	81	77
$Br_2Dy_2O_2$	996	0.0016	82	84
$I_2O_2Y_2$	996	0.0016	84	82
$Bi_2Br_2O_2$	996	0.0017	84	82
Br_2Cr	411	0.0017	40	57
$Br_2O_2Y_2$	996	0.0017	82	84
$Cl_2Er_2S_2$	432	0.0017	42	30

Table showing the first 50 lattice matching combinations with the smallest strain needed to build the supercell.

$CrSe_2$ (C2/m)

Structural and electronic properties

**Formula**	$CrSe_2$
**Spacegroup**	C2/m
**Prototype**	CdI2
**Parent 3D**	$CrSe_2$
**Source DB**	ICSD
**DB ID**	626718
**DF2-C09 Binding energy [meV/ Å^2]**	23.28
**RVV10 Binding energy [meV/ Å^2]**	29.74
**Band gap (PBE) [eV]**	N/A

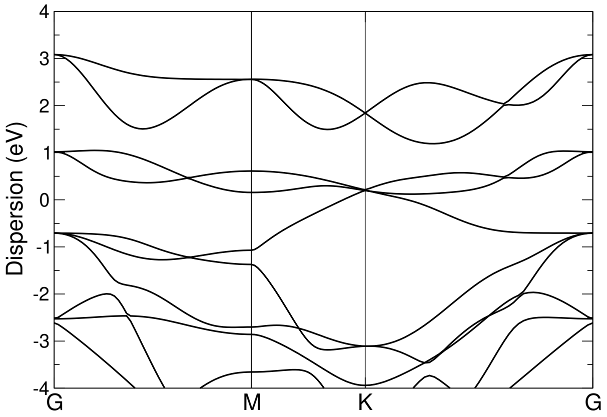

Band structure: Electronic band structure of $CrSe_2$ (C2/m) in a representative window around the Fermi energy and along a high-symmetry path.

Optimized crystal structure

Structural parameters: relaxed cell (top) and atomic positions (bottom) of $CrSe_2$ (C2/m) in Cartesian coordinates.

	x [Å]	y [Å]	z [Å]
$\mathbf{a_1}$	3.22307063	0.00000000	0.00000000
$\mathbf{a_2}$	−1.61153531	2.79126104	0.00000000
$\mathbf{a_3}$	0.00000000	0.00000000	22.80713185

		x [Å]	y [Å]	z [Å]
●	Se	1.61153531	0.93042035	9.82706634
●	Cr	-0.00000000	1.86084069	11.40356587
●	Se	1.61153531	2.79126104	12.98006557

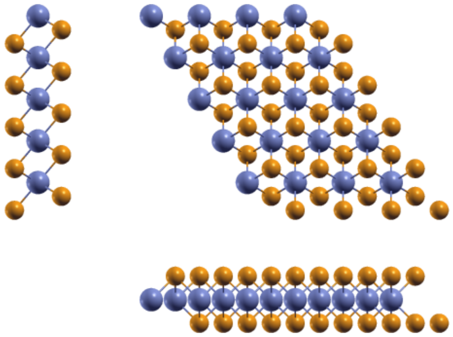

Orthographic projections: views of $CrSe_2$ (C2/m) as seen from the x axis (left), the y axis (bottom) and the z axis (center).

Lattice matching - minimal size

Formula	N° atoms	strain	cell size 1	cell size 2
Sn	4	0.1349	1	1
Na	4	0.4504	1	1
In	4	0.1385	1	1
In	4	0.0091	1	1
As_2	5	0.4702	1	1
LiO	5	0.0013	1	1
P_2	5	0.009	1	1
Mg_2	5	0.1488	1	1
Cl_2Zn	6	0.4676	1	1
I_2Mg	6	13.6681	1	1
S_2V	6	0.006	1	1
MoS_2	6	0.0053	1	1
$MoTe_2$	6	0.456	1	1
PSn_2	6	0.4933	1	1
Br_2Zn	6	2.9987	1	1
HfS_2	6	0.4772	1	1
FeO_2	6	0.2484	1	1
$AsSn_2$	6	2.908	1	1
Te_2V	6	0.4608	1	1
I_2Pr	6	0.3229	1	1
$CuTe_2$	6	0.4763	1	1
S_2Zr	6	2.8496	1	1
Br_2La	6	13.6879	1	1
Br_2Cu	6	1.3494	1	1
NiO_2	6	0.2501	1	1
Br_2Co	6	0.4692	1	1
Ca_2N	6	0.4716	1	1
Cl_2Ti	6	0.0092	1	1
I_2Ni	6	3.1972	1	1
Te_2Ti	6	3.0027	1	1
Te_2Zn	6	0.4556	1	1
S_2W	6	0.0052	1	1
Br_2Mn	6	0.4646	1	1
PtS_2	6	0.453	1	1
$CoTe_2$	6	0.4781	1	1
CdClO	6	0.4596	1	1
Te_2W	6	0.4564	1	1
I_2Nd	6	0.3249	1	1
$NiTe_2$	6	3.0462	1	1
S_2Sn	6	2.8529	1	1
Cl_2V	6	0.0013	1	1
$PtSe_2$	6	2.9164	1	1
OTl_2	6	0.46	1	1
Br_2Fe	6	0.4693	1	1
Br_2Ni	6	0.4824	1	1
CeI_2	6	0.3214	1	1
$FeSe_2$	6	0.1301	1	1
$NbTe_2$	6	2.8469	1	1
MoS_2	6	0.0051	1	1
Cl_2Mg	6	0.4825	1	1

Table showing the first 50 lattice matching combinations with the smallest number of atoms in the supercell.

Lattice matching - minimal strain

Formula	N° atoms	strain	cell size 1	cell size 2
Bi_2	66	0.0	16	9
CCl_2Gd_2	327	0.0	49	36
$Cl_2H_2Lu_2$	561	0.0	73	57
FeI_2	183	0.0	36	25
Sb_2Se_2Te	488	0.0001	81	49
Se_2Yb	294	0.0001	61	37
$CuTe_2$	435	0.0001	81	64
CoO_2	339	0.0001	49	64
GeI_2	294	0.0001	61	37
Br_2Zn	255	0.0001	49	36
$PtSe_2$	300	0.0002	57	43
Te_2V	543	0.0002	100	81
BN	233	0.0002	37	61
Br_2Mg	183	0.0002	36	25
MnO_2	255	0.0002	36	49
NaO_4	317	0.0002	64	25
DyI_2	222	0.0003	49	25
$BiTe_2$	390	0.0003	81	49
Cl_2Mg	390	0.0003	73	57
Cl_2Zn	492	0.0003	91	73
CaH_2O_2	563	0.0003	81	64
Br_2Ni	390	0.0003	73	57
Ba_2Ni_3	368	0.0003	61	37
Ga_2Te_2	331	0.0003	61	37
$BiTe_2$	294	0.0004	61	37
$I_2Nd_2S_2$	594	0.0004	100	49
In_2Se_2	220	0.0004	48	19
Br_2Ca_3Si	237	0.0004	39	20
CrI_2	183	0.0004	36	25
$Dy_2I_2S_2$	297	0.0004	49	25
$Ga_2Gd_2I_2$	102	0.0004	16	9
$I_2Pr_2Si_2$	405	0.0004	61	37
Te_2Ti	255	0.0004	49	36
$Cl_2Hf_2N_2$	786	0.0005	100	81
$PtTe_2$	123	0.0005	25	16
OTl_2	543	0.0005	100	81
$HfSe_2$	255	0.0005	49	36
PTe_2Ti_2	504	0.0005	73	57
HfS_2	435	0.0005	81	64
$AsSn_2$	300	0.0005	57	43
Br_2Ho_2	139	0.0005	25	16
KS_2Ti	565	0.0005	91	73
Sb_2Se_2Te	488	0.0005	81	49
CBr_2Y_2	327	0.0005	49	36
$Cl_2N_2Zr_2$	627	0.0006	81	64
$AgNO_3$	317	0.0006	64	25
Bi_2SeTe_2	93	0.0006	16	9
$AsCuLi_2$	331	0.0006	61	37
FeO_2	339	0.0006	49	64
Ba_2Hg	177	0.0006	39	20

Table showing the first 50 lattice matching combinations with the smallest strain needed to build the supercell.

Cu_2O_2 (P1)

Structural and electronic properties

**Formula**	Cu_2O_2
**Spacegroup**	P1
**Prototype**	FeSe
**Parent 3D**	$C_2Cu_2O_6$
**Source DB**	ICSD
**DB ID**	151296
**DF2-C09 Binding energy [meV/ Å^2]**	10.57
**RVV10 Binding energy [meV/ Å^2]**	N/A
**Band gap (PBE) [eV]**	0.0

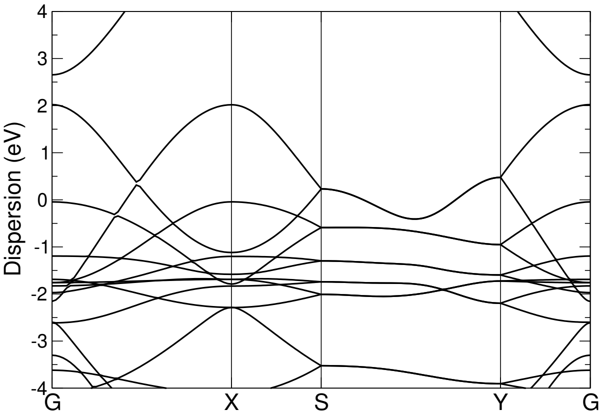

Band structure: Electronic band structure of Cu_2O_2 (P1) in a representative window around the Fermi energy and along a high-symmetry path.

Optimized crystal structure

Structural parameters: relaxed cell (top) and atomic positions (bottom) of Cu_2O_2 (P1) in Cartesian coordinates.

	x [Å]	y [Å]	z [Å]
$\mathbf{a_1}$	−2.75061692	−0.00000008	0.00000000
$\mathbf{a_2}$	−0.00000011	−5.49522980	0.00000000
$\mathbf{a_3}$	0.00000000	0.00000000	12.91295768

		x [Å]	y [Å]	z [Å]
●	Cu	-2.64216047	-0.07546726	0.00047377
•	O	-1.26690532	-4.19689680	0.00097886
●	Cu	-2.64216128	-2.82308369	-0.00043147
•	O	-1.26680006	-1.44928213	-0.00102117

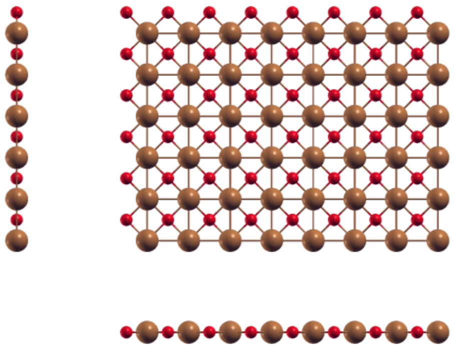

Orthographic projections: views of Cu_2O_2 (P1) as seen from the x axis (left), the y axis (bottom) and the z axis (center).

Lattice matching - minimal size

Formula	N° atoms	strain	cell size 1	cell size 2
In	6	0.0534	1	2
I_2Pr	7	0.1884	1	1
I_2Nd	7	0.1895	1	1
CeI_2	7	0.1876	1	1
F_2Na	7	2.2675	1	1
F_2Zn	7	0.184	1	1
In_2Se_2	8	0.2393	1	1
Br_2Pr_2	8	2.2595	1	1
Ir_2P_2	8	0.1893	1	1
$AgNO_2$	8	0.7207	1	1
Pb_2Se_2	8	0.4018	1	1
Cu_2S_2	8	0.1806	1	1
Br_2Cu_2	8	0.1826	1	1
Bi_2Se_2	8	0.3586	1	1
O_2Sn_2	8	0.1812	1	1
P_2Rh_2	8	0.18	1	1
F_2Tl_2	8	0.1801	1	1
Ni_2Se_2	8	0.1873	1	1
AsI_2La_2	9	0.3099	1	1
IKO_3	9	3.3847	1	1
$CuGeO_3$	9	0.0282	1	1
CrS_2	10	0.157	1	2
S_2V	10	0.0564	1	2
MoS_2	10	0.0571	1	2
$Er_2I_2O_2$	10	0.1916	1	1
FeO_2	10	0.6499	1	2
$Br_2O_2Y_2$	10	0.1805	1	1
ReS_2	10	0.1634	1	2
CrO_2	10	0.6128	1	2
$Ca_2Ge_2Mn_2$	10	0.1853	1	1
MnO_2	10	0.6199	1	2
Mg_3	10	1.0944	1	2
$Br_2F_2Tm_2$	10	0.1851	1	1
Te_2Zn	10	1.1236	1	2
$I_2Lu_2O_2$	10	0.1851	1	1
S_2W	10	0.0571	1	2
$Br_2O_2Tb_2$	10	0.1822	1	1
$C_2Br_2Gd_2$	10	0.8028	1	1
$Br_2Dy_2O_2$	10	0.1805	1	1
CNRb	10	2.0598	1	2
$I_2O_2Yb_2$	10	0.1889	1	1
C	10	0.1441	1	6
$Bi_2Cl_2O_2$	10	0.1859	1	1
$Br_2F_2Yb_2$	10	0.1832	1	1
$Br_2Eu_2O_2$	10	0.1877	1	1
$Br_2Ho_2O_2$	10	0.1787	1	1
$I_2O_2Tm_2$	10	0.1907	1	1
$I_2O_2Y_2$	10	0.1931	1	1
$Br_2Ca_2F_2$	10	0.1836	1	1
$Br_2O_2Sm_2$	10	0.1895	1	1

Table showing the first 50 lattice matching combinations with the smallest number of atoms in the supercell.

Lattice matching - minimal strain

Formula	N° atoms	strain	cell size 1	cell size 2
$Cl_2Ho_2O_2$	606	0.0001	54	65
Sb_2SeTe_2	355	0.0002	40	39
In_2Te_3	355	0.0002	40	39
Bi_2Se_2Te	355	0.0002	40	39
F_4Nb	76	0.0003	9	8
Cu_2S_2	396	0.0003	49	50
$Br_2Eu_2F_2$	494	0.0003	50	49
Mg_2	362	0.0003	50	81
$Er_2I_2O_2$	494	0.0003	50	49
I_2V	411	0.0003	54	65
$Fe_2Li_2P_2$	86	0.0003	8	9
$Br_2O_2Y_2$	496	0.0004	49	50
$Br_2Dy_2O_2$	496	0.0004	49	50
Ho_2S_2	16	0.0004	2	2
Cu_4Te_2	752	0.0004	77	74
O_2Sn_2	396	0.0005	49	50
$I_2O_2Tm_2$	494	0.0006	50	49
$MoTe_2$	479	0.0006	59	81
Te_2W	479	0.0006	59	81
Te_2Zn	479	0.0006	59	81
$I_2Lu_2Se_2$	228	0.0006	30	18
Cl_2Cu	428	0.0007	56	68
$Bi_2Br_2O_2$	494	0.0007	50	49
GeI_2Y_2	355	0.0007	40	39
Cl_4Cu_2	516	0.0007	81	32
$Cl_2O_2Y_2$	606	0.0007	54	65
F_2Tl_2	396	0.0007	49	50
Cl_4Pd_2	944	0.0008	128	72
Br_2Er_2	480	0.0008	58	62
$HfLiS_2$	560	0.0008	59	81
CdH_2O_2	641	0.0008	59	81
P_2Rh_2	396	0.0009	49	50
Cl_4Mg_2	756	0.0009	126	42
As_2Li_2Nd	355	0.0009	40	39
F_4Pb	253	0.0009	32	25
Br_2Gd_2	480	0.0009	58	62
Ga_2Se_2	476	0.0013	54	65
Se_2Zr	411	0.0013	54	65
$Br_2O_2Tb_2$	496	0.0013	49	50
STl_2	277	0.0013	40	39
$Cl_2Er_2O_2$	978	0.0013	87	105
$Cl_2Hg_2N_2$	248	0.0014	35	18
PtS_2	479	0.0014	59	81
Fe_2Se_2	68	0.0014	8	9
$Cl_2Er_2O_2$	606	0.0014	54	65
$Ba_2H_2I_2$	208	0.0015	25	18
$F_2Se_2Yb_2$	866	0.0015	80	91
$I_2La_2Si_2$	394	0.0015	40	39
$Cu_2K_2Te_2$	208	0.0015	25	18
$I_2O_2Y_2$	494	0.0015	50	49

Table showing the first 50 lattice matching combinations with the smallest strain needed to build the supercell.

Cu_2O_4 (Pmmn)

Structural and electronic properties

**Formula**	Cu_2O_4
**Spacegroup**	Pmmn
**Prototype**	FeO2
**Parent 3D**	CuO_2
**Source DB**	ICSD
**DB ID**	157973
**DF2-C09 Binding energy [meV/ Å^2]**	20.96
**RVV10 Binding energy [meV/ Å^2]**	N/A
**Band gap (PBE) [eV]**	0.0

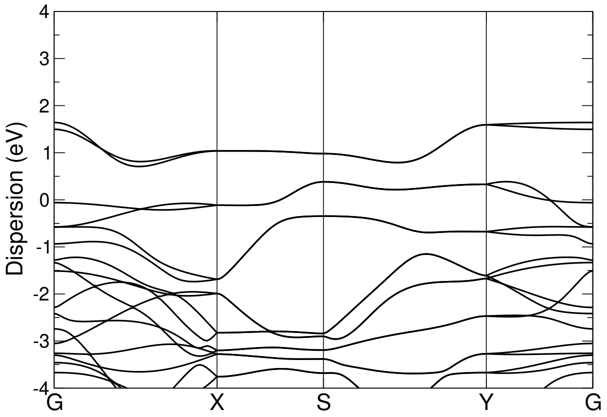

Band structure: Electronic band structure of Cu_2O_4 (Pmmn) in a representative window around the Fermi energy and along a high-symmetry path.

Optimized crystal structure

Structural parameters: relaxed cell (top) and atomic positions (bottom) of Cu_2O_4 (Pmmn) in Cartesian coordinates.

	x [Å]	y [Å]	z [Å]
$\mathbf{a_1}$	2.78968908	0.00000000	0.00000000
$\mathbf{a_2}$	0.00000000	4.26649975	0.00000000
$\mathbf{a_3}$	0.00000000	0.00000000	20.49742408

		x [Å]	y [Å]	z [Å]
●	Cu	-0.69742227	-5.33312469	-0.87176533
●	Cu	0.69742227	-3.19987481	0.87176533
•	O	0.69742227	-5.33312469	0.40506901
•	O	-0.69742227	-3.19987481	-0.40506901
•	O	0.69742227	-5.33312469	-2.05094946
•	O	-0.69742227	-3.19987481	2.05094946

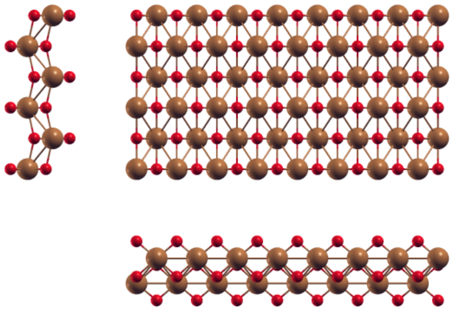

Orthographic projections: views of Cu_2O_4 (Pmmn) as seen from the x axis (left), the y axis (bottom) and the z axis (center).

Lattice matching - minimal size

Formula	N° atoms	strain	cell size 1	cell size 2
GeTe	8	0.9247	1	1
AgTl	8	0.3751	1	1
$IrTe_2$	9	0.9288	1	1
$CdCl_2$	9	0.9199	1	1
$InSe_2$	9	0.9216	1	1
I_2Mn	9	0.9204	1	1
Te_2Zn	9	0.1716	1	1
$CrTe_2$	9	0.7252	1	1
Br_2Ti	9	0.7231	1	1
$AsSe_2$	9	0.7085	1	1
BrNZr	9	0.7132	1	1
$NbSe_2$	9	0.7049	1	1
Br_2Cr	9	0.7243	1	1
Se_2Ta	9	0.7052	1	1
$NbSe_2$	9	0.7095	1	1
Se_2Ta	9	0.7203	1	1
H_2Si_2	10	0.923	1	1
K	10	7.7912	1	4
$AgNO_2$	10	1.3813	1	1
$AgCuTe_2$	10	0.0561	1	1
Au_2Br_2	10	0.4657	1	1
Cl_2OV	10	0.1306	1	1
S_2Zn_2	10	0.9378	1	1
Br_2Zr_2	10	0.7376	1	1
P_2Sn_2	10	0.9362	1	1
N_4	10	0.3922	1	1
Ga_2Se_2	10	0.9297	1	1
BN	10	0.1437	1	2
Ge_2Se_2	10	0.3775	1	1
C_2	10	0.0364	1	2
Ho_2S_2	10	0.3562	1	1
Cl_2Sc_2	10	0.7249	1	1
In_2S_3	11	0.9256	1	1
Ni_2SbTe_2	11	0.9326	1	1
Ga_2S_3	11	0.17	1	1
Cu_4Te_2	12	1.1664	1	1
FeO_2	12	0.6984	1	2
Cu_4Te_2	12	1.1708	1	1
Cr_2O_4	12	0.4057	1	1
$Cl_2N_2Zr_2$	12	0.2887	1	1
CrO_2	12	0.6587	1	2
MnO_2	12	0.6663	1	2
$Al_2Cl_2O_2$	12	0.1426	1	1
$Ga_2I_2Y_2$	12	1.1048	1	1
$Cl_2Fe_2O_2$	12	0.1495	1	1
$Cl_2Cr_2O_2$	12	0.1655	1	1
$Br_2Cr_2O_2$	12	0.4749	1	1
$F_2Se_2Y_2$	12	0.413	1	1
$Cl_2H_2Sc_2$	12	0.7212	1	1
$I_2N_2Ti_2$	12	0.6649	1	1

Table showing the first 50 lattice matching combinations with the smallest number of atoms in the supercell.

Lattice matching - minimal strain

Formula	N° atoms	strain	cell size 1	cell size 2
P_2	342	0.0002	40	51
Cl_2Ti	393	0.0004	40	51
CNb_2S_2	495	0.0004	40	51
Au_2Br_2	948	0.0004	110	72
CS_2Ta_2	495	0.0004	40	51
Au_2Br_2	672	0.0006	78	51
PbTe	774	0.0006	103	78
Au_2Br_2	646	0.0006	75	49
GeI_2	852	0.0007	103	78
$AgNO_2$	810	0.0008	91	66
$LiMnTe_2$	930	0.0009	103	78
Au_2Br_2	620	0.0009	72	47
CdI_2	702	0.0009	85	64
Br_2Tb_2	752	0.0009	80	68
$Cl_2H_2Sc_2$	840	0.0009	66	74
Br_2La_2P	830	0.0009	85	64
Bi_2S_3	820	0.0009	80	68
Br_2Ti	618	0.001	66	74
Se_2Ta	618	0.001	66	74
Bi_2STe_2	830	0.001	85	64
Br_2Ho_2	752	0.001	80	68
CdI_2	702	0.001	85	64
Gd_2GeI_2	830	0.001	85	64
Br_2Cr	618	0.0011	66	74
$PdTe_2$	684	0.0011	80	68
$LiMnTe_2$	766	0.0011	85	64
Cu_2Te_2	264	0.0012	28	24
S_2Ti	648	0.0012	68	80
BiClTe	702	0.0012	85	64
Cl_2Sc_2	692	0.0012	66	74
Au_2Br_2	594	0.0013	69	45
I_2Pr	702	0.0013	85	64
$CrTe_2$	618	0.0013	66	74
CKN	138	0.0013	18	10
$BrKO_3$	717	0.0013	97	27
HN_3OZn	546	0.0013	40	51
GeI_2	702	0.0014	85	64
GeI_2	678	0.0014	82	62
$AlLiTe_2$	780	0.0014	88	63
As_2CeLi_2	802	0.0014	82	62
PbTe	616	0.0014	82	62
Cl_2Co	648	0.0014	68	80
Au_2Br_2	276	0.0014	32	21
$AsLi_3$	930	0.0015	103	78
$As_2Co_2Li_2$	312	0.0015	28	24
PTe_2Zr_2	822	0.0015	77	72
Mg_6	312	0.0015	24	28
CNNa	738	0.0015	91	64
CdO_2	648	0.0015	68	80
$AsSn_2$	108	0.0016	12	12

Table showing the first 50 lattice matching combinations with the smallest strain needed to build the supercell.

Cu_2Te (C2/m)

Structural and electronic properties

**Formula**	Cu_2Te
**Spacegroup**	C2/m
**Prototype**	Cu2Te
**Parent 3D**	Cu_4Te_2
**Source DB**	ICSD
**DB ID**	77055
**DF2-C09 Binding energy [meV/ Å^2]**	14.58
**RVV10 Binding energy [meV/ Å^2]**	20.47
**Band gap (PBE) [eV]**	0.21

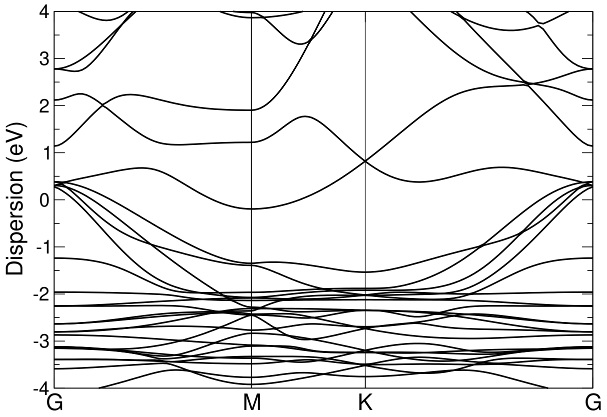

Band structure: Electronic band structure of Cu_2Te (C2/m) in a representative window around the Fermi energy and along a high-symmetry path.

Optimized crystal structure

Structural parameters: relaxed cell (top) and atomic positions (bottom) of Cu_2Te (C2/m) in Cartesian coordinates.

	x [Å]	y [Å]	z [Å]
$\mathbf{a_1}$	4.31842852	0.00000000	0.00000000
$\mathbf{a_2}$	−2.15921426	3.73986880	0.00000000
$\mathbf{a_3}$	0.00000000	0.00000000	24.75406767

		x [Å]	y [Å]	z [Å]
●	Cu	2.15921426	1.24662293	13.67503081
●	Cu	0.00000000	2.49324587	13.67503081
●	Te	0.00000000	-0.00000000	14.66950726
●	Cu	0.00000000	2.49324587	11.07903686
●	Cu	2.15921426	1.24662293	11.07903686
●	Te	0.00000000	-0.00000000	10.08456041

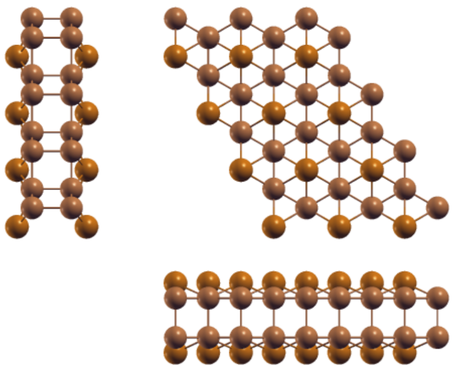

Orthographic projections: views of Cu_2Te (C2/m) as seen from the x axis (left), the y axis (bottom) and the z axis (center).

Lattice matching - minimal size

Formula	N° atoms	strain	cell size 1	cell size 2
InSe	8	0.0079	1	1
AsSb	8	0.2602	1	1
Bi_2	8	0.0023	1	1
GeTe	8	0.2688	1	1
S_2	8	0.2711	1	1
PbTe	8	0.0062	1	1
CaCl	8	0.4421	1	1
$IrTe_2$	9	0.2701	1	1
$CdCl_2$	9	0.2672	1	1
CdI_2	9	0.0046	1	1
PSn_2	9	1.5249	1	1
Br_2Zn	9	0.2464	1	1
Br_2Ca	9	0.0033	1	1
$InSe_2$	9	0.2678	1	1
$AsSn_2$	9	1.5519	1	1
$GeTe_2$	9	0.2658	1	1
$SiTe_2$	9	1.6331	1	1
I_2Pr	9	0.1303	1	1
I_2Mn	9	0.2674	1	1
Br_2Cu	9	0.6896	1	1
NSr_2	9	0.2624	1	1
PbS_2	9	0.2566	1	1
BiClTe	9	0.0042	1	1
Cl_2Zn	9	0.1162	1	1
FeI_2	9	0.2642	1	1
I_2Ni	9	0.2661	1	1
Te_2Ti	9	0.2468	1	1
CrI_2	9	0.2636	1	1
BiBrTe	9	0.0013	1	1
Br_2Mn	9	1.4477	1	1
I_2Nd	9	0.131	1	1
$NiTe_2$	9	1.6286	1	1
Cl_2Cu	9	0.0703	1	1
S_2Sn	9	1.5214	1	1
I_2V	9	0.2537	1	1
GeI_2	9	0.006	1	1
Se_2Zr	9	1.6362	1	1
$PtSe_2$	9	1.5566	1	1
BiITe	9	0.008	1	1
CoI_2	9	0.2608	1	1
$MnSe_2$	9	0.4419	1	1
CeI_2	9	0.1298	1	1
Br_2Mg	9	0.2641	1	1
I_2Ti	9	0.2613	1	1
GdI_2	9	0.0042	1	1
F_2Ni	9	0.1144	1	1
I_2La	9	0.1346	1	1
F_2Na	9	0.249	1	1
CdI_2	9	0.005	1	1
Se_2Sn	9	0.2621	1	1

Table showing the first 50 lattice matching combinations with the smallest number of atoms in the supercell.

Lattice matching - minimal strain

Formula	N° atoms	strain	cell size 1	cell size 2
FeI_2	711	0.0001	73	91
Bi_2Te_2	924	0.0001	100	81
PTe_2Zr_2	789	0.0001	64	81
Cl_2Y_2	486	0.0001	43	57
$TaTe_2$	363	0.0001	36	49
$InSe_2$	786	0.0001	81	100
I_2Mn	786	0.0001	81	100
F_2Na	486	0.0002	49	64
Br_2Mg	711	0.0002	73	91
Cl_2Mn	405	0.0002	37	61
$ReSe_2$	171	0.0002	16	25
$Cl_2O_2Yb_2$	678	0.0002	49	64
Ga_2Se_2	634	0.0002	57	73
$CdCl_2$	786	0.0002	81	100
Cl_2NSc_2	527	0.0003	37	61
Cl_2Zn	258	0.0003	25	36
$Cl_2O_2Y_2$	780	0.0003	57	73
C_2	194	0.0003	16	49
$Hf_2I_2N_2$	510	0.0004	36	49
H_2Si_2	886	0.0004	81	100
CrI_2	711	0.0004	73	91
CdClHO	412	0.0004	36	49
Ni_2Te_2	886	0.0005	81	100
N_2W	594	0.0005	49	100
KS_2Ti	294	0.0005	25	36
Sb_2Te_3	11	0.0006	1	1
Br_2V	171	0.0006	16	25
Br_2Y_2	634	0.0006	57	73
Ga_2Se_2	486	0.0006	43	57
AsSb	546	0.0006	64	81
$F_2Se_2Y_2$	870	0.0006	81	64
$Cl_2O_2Tm_2$	678	0.0006	49	64
CBr_2Y_2	543	0.0006	43	57
FeH_2O_2	794	0.0007	49	100
$AsSn_2$	363	0.0007	36	49
GeI_3Rb	339	0.0007	49	9
CaClHO	550	0.0008	49	64
$Br_2Ga_2Te_2$	900	0.0008	91	59
GeTe	686	0.0008	81	100
PbS_2	561	0.0008	57	73
Br_2Co	258	0.0009	25	36
Br_2Mn	258	0.0009	25	36
Br_2Fe	258	0.0009	25	36
Mg_3	714	0.001	65	108
Br_2Pr_2	550	0.001	49	64
$Cl_2Ho_2O_2$	780	0.001	57	73
$Br_2Ga_2Te_2$	852	0.001	86	56
In_2S_3	986	0.001	81	100
LiO	86	0.001	9	16
$GeTe_2$	711	0.001	73	91

Table showing the first 50 lattice matching combinations with the smallest strain needed to build the supercell.

$Cu_2Tl_2Se_2$ (P4/nmm)

Structural and electronic properties

**Formula**	$Cu_2Tl_2Se_2$
**Spacegroup**	P4/nmm
**Prototype**	PbClF
**Parent 3D**	$Cu_2Se_2Tl_2$
**Source DB**	MPDS
**DB ID**	S542660
**DF2-C09 Binding energy [meV/ Å^2]**	23.97
**RVV10 Binding energy [meV/ Å^2]**	N/A
**Band gap (PBE) [eV]**	0.73

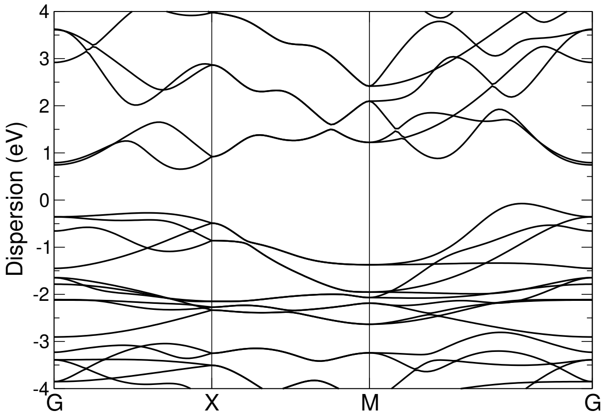

Band structure: Electronic band structure of $Cu_2Tl_2Se_2$ (P4/nmm) in a representative window around the Fermi energy and along a high-symmetry path.

Optimized crystal structure

Structural parameters: relaxed cell (top) and atomic positions (bottom) of $Cu_2Tl_2Se_2$ (P4/nmm) in Cartesian coordinates.

		x [Å]	y [Å]	z [Å]
$\mathbf{a_1}$		4.08425129	0.00000000	0.00000000
$\mathbf{a_2}$		0.00000000	4.08425129	0.00000000
$\mathbf{a_3}$		0.00000000	0.00000000	23.29129249
		x [Å]	y [Å]	z [Å]
●	Tl	1.02106282	-1.02106282	2.84415863
●	Se	-1.02106282	-3.06318847	1.51217595
●	Tl	-1.02106282	-3.06318847	-2.84415863
●	Cu	1.02106282	-3.06318847	0.00000000
●	Cu	-1.02106282	-1.02106282	0.00000000
●	Se	1.02106282	-1.02106282	-1.51217595

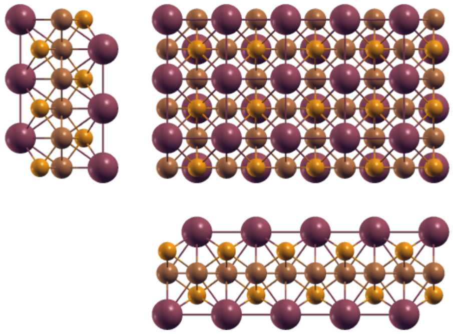

Orthographic projections: views of $Cu_2Tl_2Se_2$ (P4/nmm) as seen from the x axis (left), the y axis (bottom) and the z axis (center).

Lattice matching - minimal size

Formula	N° atoms	strain	cell size 1	cell size 2
K	7	0.1597	1	1
Bi_2	8	0.1329	1	1
AgTl	8	0.0127	1	1
Ag_2	8	0.1656	1	1
PbTe	8	0.13	1	1
CaCl	8	0.2084	1	1
$CdCl_2$	9	0.109	1	1
Cl_2Mn	9	0.1102	1	1
CdI_2	9	0.1312	1	1
Nd	9	0.1724	1	3
Ba_2Pt	9	0.1653	1	1
S_2Ta	9	0.1098	1	1
Br_2Ca	9	0.1321	1	1
CaI_2	9	0.1504	1	1
$InSe_2$	9	0.109	1	1
I_2Mn	9	0.109	1	1
Br_2Cu	9	0.0986	1	1
Ca_2Si	9	0.1704	1	1
I_2Yb	9	0.148	1	1
BiClTe	9	0.1315	1	1
FeI_2	9	0.1084	1	1
I_2Ni	9	0.1087	1	1
S_2Ti	9	0.1088	1	1
NbS_2	9	0.1099	1	1
BiBrTe	9	0.1357	1	1
Cl_2Co	9	0.1088	1	1
NbS_2	9	0.1109	1	1
Cl_2Fe	9	0.109	1	1
S_2Ta	9	0.1111	1	1
Se_2V	9	0.1114	1	1
I_2Tm	9	0.1493	1	1
GeI_2	9	0.1301	1	1
I_2Pb	9	0.1674	1	1
BiITe	9	0.1411	1	1
GeS_2	9	0.5693	1	1
$MnSe_2$	9	0.2083	1	1
DyI_2	9	0.1532	1	1
Cl_2Zr	9	0.1089	1	1
Br_2Mg	9	0.1084	1	1
GdI_2	9	0.138	1	1
CNNa	9	0.0684	1	1
F_2Ni	9	0.2201	1	1
CdI_2	9	0.1309	1	1
I_2Pr	9	0.1315	1	1
Bi_2Te_2	10	0.1858	1	1
Bi_2In_2	10	0.4041	1	1
Cu_2I_2	10	0.0009	1	1
Cu_2Sr_2	10	0.1365	1	1
Cl_2OOs	10	0.2144	1	1
$LiMnTe_2$	10	0.1303	1	1

Table showing the first 50 lattice matching combinations with the smallest number of atoms in the supercell.

Lattice matching - minimal strain

Formula	N° atoms	strain	cell size 1	cell size 2
F_2Ni	795	0.0001	82	101
Fe_2Se_2	886	0.0001	81	100
$Ba_2H_2I_2$	870	0.0001	81	64
Br_2Pr_2	548	0.0002	48	65
As_2Co_2	718	0.0002	65	82
H_2Na_2Pd	461	0.0003	36	49
Fe_2S_2	560	0.0003	50	65
$Ge_2Se_2Zr_2$	870	0.0003	64	81
Ca_2Cl_2	708	0.0003	64	81
Cl_2ORu	698	0.0003	63	80
Mg_4	736	0.0004	58	97
Mg_3	945	0.0005	85	145
$Ag_2K_2Te_2$	510	0.0006	49	36
F_2Ni	786	0.0006	81	100
Cu_2Se_2	896	0.0006	82	101
$C_2Br_2Gd_2$	858	0.0006	65	78
$Cl_2O_2Yb_2$	678	0.0007	48	65
Br_2Ca_3Si	978	0.0007	100	63
$FeSe_2$	297	0.0007	25	49
CaCl	968	0.0007	113	145
F_2Na	483	0.0007	48	65
Ga_2S_2	958	0.0007	81	118
Tl	445	0.0008	58	97
NiO_2	714	0.0008	54	130
H_2Li_2Pd	134	0.0008	9	16
$CoTe_2$	840	0.0008	81	118
$Al_2Cl_2O_2$	918	0.0009	63	90
Cu_2I_2	10	0.0009	1	1
Ga_2S_2	958	0.0009	81	118
$H_2Li_2O_2$	678	0.0009	49	64
Sn_2Te_2	838	0.0009	91	73
$HfSe_2$	483	0.001	48	65
$C_2Br_2Tb_2$	858	0.001	65	78
Te_2Ti	483	0.001	48	65
Br_2Ca_3Si	948	0.001	97	61
$AgTe_2$	537	0.001	49	81
As_2Co_2	708	0.001	64	81
$NbSe_2$	852	0.001	79	126
$AsSe_2$	852	0.0011	79	126
HfS_2	840	0.0011	81	118
Cr_2O_4	660	0.0011	43	67
GeS_2	363	0.0011	36	49
O_2Pt	237	0.0011	20	39
$Br_2O_2Ti_2$	66	0.0011	5	6
Bi_2Pd	942	0.0011	89	136
Bi_2O_2	10	0.0011	1	1
Mg_2	86	0.0011	9	16
Sn_2Te_2	828	0.0011	90	72
Cu_2Se_2	886	0.0012	81	100
Br_2Zn	483	0.0013	48	65

Table showing the first 50 lattice matching combinations with the smallest strain needed to build the supercell.

Cu_4Te_2 (C2/m)

Structural and electronic properties

**Formula**	Cu_4Te_2
**Spacegroup**	C2/m
**Prototype**	Cu2Te
**Parent 3D**	Cu_4Te_2
**Source DB**	ICSD
**DB ID**	671961
**DF2-C09 Binding energy [meV/ Å^2]**	26.35
**RVV10 Binding energy [meV/ Å^2]**	N/A
**Band gap (PBE) [eV]**	0.0

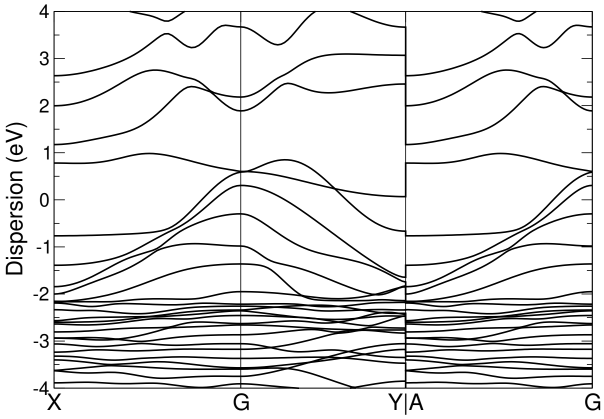

Band structure: Electronic band structure of Cu_4Te_2 (C2/m) in a representative window around the Fermi energy and along a high-symmetry path.

Optimized crystal structure

Structural parameters: relaxed cell (top) and atomic positions (bottom) of Cu_4Te_2 (C2/m) in Cartesian coordinates.

	x [Å]	y [Å]	z [Å]
$\mathbf{a_1}$	0.00000012	3.93273916	0.00000000
$\mathbf{a_2}$	3.99178906	1.96636970	0.00000000
$\mathbf{a_3}$	0.00000000	0.00000000	20.86581546

		x [Å]	y [Å]	z [Å]
●	Cu	0.39177959	1.96637045	-9.19771562
●	Cu	-0.39177948	1.96636871	-11.66809985
●	Cu	2.80085964	1.96637050	-10.00235105
●	Cu	1.19092943	-0.00000080	-10.86346442
●	Te	1.95227984	0.00000092	-8.24380680
●	Te	2.03950922	1.96636878	-12.62200866

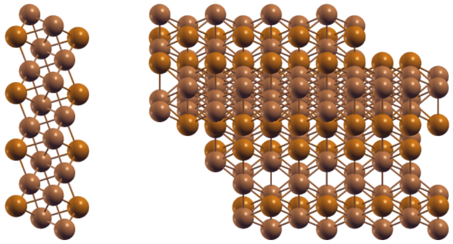

Orthographic projections: views of Cu_4Te_2 (C2/m) as seen from the x axis (left), the y axis (bottom) and the z axis (center).

Lattice matching - minimal size

Formula	N° atoms	strain	cell size 1	cell size 2
K	7	0.1634	1	1
AgTl	8	0.1376	1	1
BN	8	0.1461	1	1
Br_2Cu	9	0.0375	1	1
Cl_2Zn	9	0.1059	1	1
BaF_2	9	0.0368	1	1
BiBrTe	9	0.0404	1	1
Ba_2Hg	9	0.1469	1	1
Tl	9	0.8355	1	3
CNNa	9	0.7047	1	1
F_2Ni	9	0.1043	1	1
N_2Re	9	0.3522	1	1
CdI_2	9	0.0381	1	1
Ba_2Cd	9	0.1497	1	1
Bi_2In_2	10	0.7455	1	1
Cu_2I_2	10	0.1368	1	1
Cl_2OOs	10	0.1054	1	1
$HNiO_2$	10	0.3868	1	1
S_2Sn_2	10	0.1523	1	1
Au_2Br_2	10	0.1653	1	1
Ge_2Te_2	10	0.1702	1	1
Fe_2Se_2	10	0.1041	1	1
Cl_2ORu	10	0.1038	1	1
As_2Co_2	10	0.1024	1	1
Cu_2Te_2	10	0.107	1	1
Ge_2S_2	10	0.1511	1	1
As_4	10	0.7122	1	1
C_2Li_2	10	0.2928	1	1
Au_2I_2	10	0.1963	1	1
Co_2S_2	10	0.1012	1	1
Ge_2Se_2	10	0.1489	1	1
Cu_2Se_2	10	0.1045	1	1
Bi_2O_2	10	0.135	1	1
La_2S_2	10	0.1658	1	1
PbS_2Sn	10	0.1489	1	1
Fe_2SeTe	10	0.1085	1	1
Se_2Sn_2	10	0.1676	1	1
Co_2Se_2	10	0.1029	1	1
Ca_2Cl_2	10	0.1022	1	1
F_4Sn	11	0.1461	1	1
Hf_3Te_2	11	0.1055	1	1
F_4Nb	11	0.1404	1	1
H_2MnO_2	11	0.3728	1	1
H_2Li_2Pd	11	0.1229	1	1
N_3W_2	11	0.3682	1	1
IKO_3	11	1.1362	1	1
$BrKO_3$	11	1.1064	1	1
H_4Ti	11	0.1229	1	1
$Br_2In_2O_2$	12	0.1309	1	1
$Br_2Ho_2S_2$	12	0.2629	1	1

Table showing the first 50 lattice matching combinations with the smallest number of atoms in the supercell.

Lattice matching - minimal strain

Formula	N° atoms	strain	cell size 1	cell size 2
CaClHO	498	0.0003	45	57
Cu_2O_2	752	0.0004	74	77
Bi_2Te_2	522	0.0007	57	45
PbTe	472	0.0007	59	59
I_2V	507	0.0007	52	65
As_2CeLi_2	649	0.0008	59	59
Bi_2STe_2	649	0.0008	59	59
$Cl_2O_2Yb_2$	612	0.0008	45	57
$MnSe_2$	954	0.0008	99	120
CaCl	834	0.0008	99	120
$NiTe_2$	441	0.0008	45	57
GeI_2	531	0.0009	59	59
ClH_3O	650	0.0009	65	52
Br_2Mn	429	0.0009	42	59
$Cl_2N_2Zr_2$	918	0.0009	74	79
$Se_2Si_2Zr_2$	972	0.0009	72	90
KS_2Ti	488	0.0009	42	59
$Cl_2O_2Y_2$	702	0.001	52	65
Li_2Tl_2	522	0.001	59	42
Br_2Zr_2	626	0.0011	53	77
Ba_2N	66	0.0011	7	8
MoS_2	318	0.0011	28	50
I_2La_2Te	83	0.0011	8	7
S_2W	318	0.0011	28	50
Cu_3Se_3	90	0.0011	7	8
S_2Ti	192	0.0011	18	28
$LiMnTe_2$	590	0.0011	59	59
MoS_2	318	0.0011	28	50
Se_2Ti	549	0.0012	53	77
O_2Sn_2	708	0.0012	64	81
Br_2Ca_3Si	90	0.0012	8	7
Cl_2Co	192	0.0012	18	28
S_2V	318	0.0013	28	50
$AsLi_3$	590	0.0013	59	59
CdO_2	192	0.0013	18	28
$AgNO_2$	246	0.0013	25	24
GeS_2	903	0.0013	92	117
O_2Sn_2	816	0.0013	74	93
Co_2Se_2	718	0.0013	67	79
CBr_2Lu_2	575	0.0014	45	61
Ga_2Se_2	572	0.0014	52	65
Cl_2Zr	192	0.0014	18	28
Bi_2STe_2	649	0.0014	59	59
Cl_2Zn	429	0.0014	42	59
$SiTe_2$	441	0.0014	45	57
Se_2Zr	507	0.0014	52	65
Te_2Zr	66	0.0014	7	8
$Hf_2Si_2Te_2$	876	0.0015	67	79
Au_2I_2	556	0.0015	60	49
Ho_2S_2	638	0.0015	63	65

Table showing the first 50 lattice matching combinations with the smallest strain needed to build the supercell.

$CuAgTe_2$ (Pm)

Structural and electronic properties

**Formula**	$CuAgTe_2$
**Spacegroup**	Pm
**Prototype**	CuAgTe2
**Parent 3D**	$CuAgTe_2$
**Source DB**	COD
**DB ID**	1509294
**DF2-C09 Binding energy [meV/ Å^2]**	24.97
**RVV10 Binding energy [meV/ Å^2]**	29.11
**Band gap (PBE) [eV]**	N/A

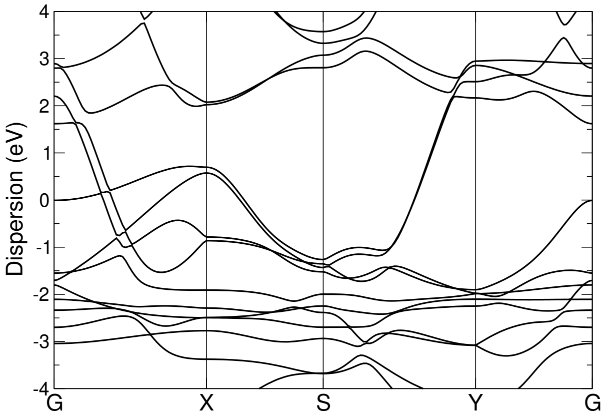

Band structure: Electronic band structure of $CuAgTe_2$ (Pm) in a representative window around the Fermi energy and along a high-symmetry path.

Optimized crystal structure

Structural parameters: relaxed cell (top) and atomic positions (bottom) of $CuAgTe_2$ (Pm) in Cartesian coordinates.

		x [Å]	y [Å]	z [Å]
$\mathbf{a_1}$		3.22380986	0.00000000	0.00000000
$\mathbf{a_2}$		0.00000000	4.21058054	0.00000000
$\mathbf{a_3}$		0.00000000	0.00000000	24.21166365
		x [Å]	y [Å]	z [Å]
●	Cu	1.61190493	0.00000000	12.58194615
●	Ag	0.00000000	2.10529027	11.75724533
●	Te	1.61190493	2.10529027	14.21329998
●	Te	0.00000000	0.00000000	9.99588358

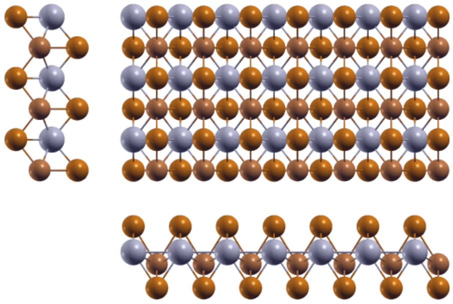

Orthographic projections: views of $CuAgTe_2$ (Pm) as seen from the x axis (left), the y axis (bottom) and the z axis (center).

Lattice matching - minimal size

Formula	N° atoms	strain	cell size 1	cell size 2
Nd	6	0.4892	1	2
HgO	6	0.2596	1	1
Sm	6	0.3736	1	2
Cl_2Zn	7	0.0658	1	1
Bi_2Pd	7	0.2731	1	1
Ba_2Hg	7	0.3628	1	1
CNNa	7	0.5535	1	1
F_2Ni	7	0.0641	1	1
Ba_2Cd	7	0.3683	1	1
Bi_2Te_2	8	0.289	1	1
Cu_2I_2	8	0.3419	1	1
Cl_2OOs	8	0.07	1	1
Cu_2Te_2	8	0.0156	1	1
O_2Sn_2	8	0.2637	1	1
Au_2Br_2	8	0.1252	1	1
Ca_2O_2	8	0.275	1	1
AgTl	8	1.0019	1	2
Fe_2Se_2	8	0.0638	1	1
Cl_2ORu	8	0.3492	1	1
As_2Co_2	8	0.348	1	1
Cu_2Te_2	8	0.067	1	1
Ge_2S_2	8	0.2782	1	1
As_4	8	0.5647	1	1
C_2Li_2	8	0.2239	1	1
BN	8	0.1455	1	2
Au_2I_2	8	0.9535	1	1
Cu_2Se_2	8	0.0643	1	1
Bi_2O_2	8	0.3379	1	1
C_2	8	0.1406	1	2
Fe_2SeTe	8	0.3783	1	1
O_2Sn_2	8	0.1863	1	1
Co_2Se_2	8	0.0626	1	1
Ca_2Cl_2	8	0.3467	1	1
F_4Sn	9	0.3612	1	1
Hf_3Te_2	9	0.0653	1	1
ClH_3O	9	0.2831	1	1
$Br_2In_2O_2$	10	0.0464	1	1
$Ge_2Hf_2Te_2$	10	0.3739	1	1
$Br_2F_2Sr_2$	10	0.3458	1	1
$Eu_2F_2I_2$	10	0.3369	1	1
FeO_2	10	0.5395	1	2
$Br_2N_2Zr_2$	10	0.0433	1	1
$I_2N_2Zr_2$	10	0.2808	1	1
Cr_2O_4	10	0.3004	1	1
$Cl_2N_2Zr_2$	10	0.0349	1	1
CrO_2	10	0.508	1	2
Br_2Ca_3Si	10	0.3633	1	1
$As_2Fe_2Li_2$	10	0.3777	1	1
$Br_2O_2Sc_2$	10	0.2467	1	1
MnO_2	10	0.514	1	2

Table showing the first 50 lattice matching combinations with the smallest number of atoms in the supercell.

Lattice matching - minimal strain

Formula	N° atoms	strain	cell size 1	cell size 2
Cu_2Se_2	448	0.0001	56	56
$I_2S_2Tl_2$	560	0.0001	56	56
$AsSe_2$	553	0.0002	70	91
$NbSe_2$	553	0.0005	70	91
F_2Ni	392	0.0005	56	56
$Si_2Te_2Zr_2$	560	0.0005	56	56
$I_2Se_2Tb_2$	118	0.0006	16	9
Fe_2O_4	580	0.0006	49	64
Se_2Ta	553	0.0008	70	91
$NbSe_2$	553	0.0008	70	91
Co_2Se_2	888	0.0009	110	112
Cl_2ORu	568	0.001	70	72
Te_4TiZr	342	0.001	48	25
$Ga_2I_2Y_2$	894	0.001	96	85
Fe_2Se_2	448	0.001	56	56
BaF_2	639	0.001	96	85
CNNa	590	0.0011	92	74
Cl_2Zn	778	0.0011	112	110
Br_2Hf_2	644	0.0011	70	91
$Br_2Ga_2Te_2$	654	0.0011	90	49
O_2Sn_2	368	0.0011	44	48
CrS_2	442	0.0012	49	82
$As_2Fe_2Li_2$	548	0.0012	56	54
$Br_2H_2Yb_2$	548	0.0012	56	54
Cl_2Fe	541	0.0012	67	91
H_2MnO_2	395	0.0013	30	55
Cl_2Ni	553	0.0013	70	91
$Br_2S_2Yb_2$	196	0.0013	25	16
Br_2Er_2	196	0.0013	25	24
LiO	28	0.0013	4	6
I_2Ni	171	0.0013	24	25
$Ca_2Cl_2F_2$	602	0.0013	62	59
C_4Ca_2	670	0.0013	76	61
$F_2Se_2Y_2$	48	0.0013	6	4
$GeTe_2$	171	0.0013	24	25
Sb_2Te_3	263	0.0013	32	27
Ga_2S_2	236	0.0013	27	32
Fe_2SeTe	440	0.0014	56	54
Ni_2Te_2	196	0.0014	24	25
O_2Sn_2	368	0.0014	44	48
$AuTe_2$	172	0.0014	25	24
Br_2Gd_2	196	0.0014	25	24
$CoTe_2$	204	0.0014	27	32
N_3W_2	716	0.0014	54	100
Ga_2S_2	236	0.0014	27	32
CNRb	646	0.0014	112	66
AsKSn	639	0.0014	96	85
As_2Sn_2	196	0.0014	25	24
$CdCl_2$	171	0.0014	24	25
$MnSe_2$	422	0.0015	59	62

Table showing the first 50 lattice matching combinations with the smallest strain needed to build the supercell.

CuBr (P4/nmm)

Structural and electronic properties

**Formula**	CuBr
**Spacegroup**	P4/nmm
**Prototype**	FeSe
**Parent 3D**	Cu_2Br_2
**Source DB**	COD
**DB ID**	9013931
**DF2-C09 Binding energy [meV/ Å^2]**	15.55
**RVV10 Binding energy [meV/ Å^2]**	22.91
**Band gap (PBE) [eV]**	1.52

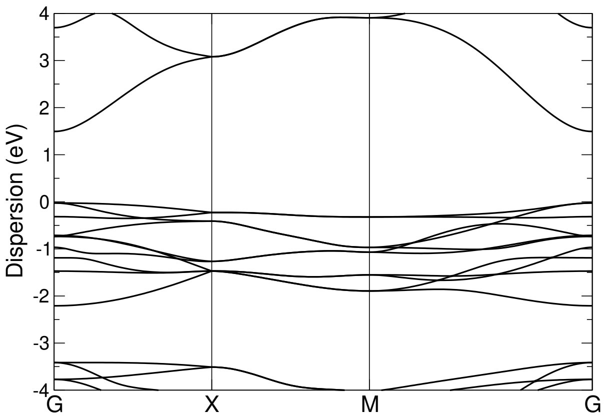

Band structure: Electronic band structure of CuBr (P4/nmm) in a representative window around the Fermi energy and along a high-symmetry path.

Optimized crystal structure

Structural parameters: relaxed cell (top) and atomic positions (bottom) of CuBr (P4/nmm) in Cartesian coordinates.

	x [Å]	y [Å]	z [Å]
$\mathbf{a_1}$	3.86242376	0.00000000	0.00000000
$\mathbf{a_2}$	0.00000000	3.86242376	0.00000000
$\mathbf{a_3}$	0.00000000	0.00000000	23.24795562

		x [Å]	y [Å]	z [Å]
●	Br	1.93121188	0.00000000	10.00356946
●	Cu	0.00000000	0.00000000	11.62397781
●	Cu	1.93121188	1.93121188	11.62397781
●	Br	0.00000000	1.93121188	13.24438617

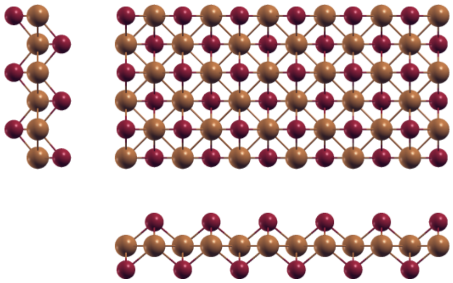

Orthographic projections: views of CuBr (P4/nmm) as seen from the x axis (left), the y axis (bottom) and the z axis (center).

Lattice matching - minimal size

Formula	N° atoms	strain	cell size 1	cell size 2
K	5	0.3885	1	1
K	5	0.1908	1	1
In	5	0.1109	1	1
InSe	6	0.1506	1	1
Bi_2	6	0.1563	1	1
Ag_2	6	0.7533	1	1
PbTe	6	0.1523	1	1
Sb_2	6	0.1365	1	1
I_2Mg	7	0.1412	1	1
S_2V	7	0.1102	1	1
MoS_2	7	0.11	1	1
CdI_2	7	0.154	1	1
Nd	7	0.7763	1	3
PSn_2	7	0.1091	1	1
Ba_2Pt	7	0.7524	1	1
Br_2Ca	7	0.1552	1	1
CaI_2	7	0.1792	1	1
I_2Pr	7	0.0054	1	1
S_2Zr	7	0.1089	1	1
Br_2La	7	0.1415	1	1
Br_2Cu	7	0.108	1	1
Ca_2Si	7	0.7696	1	1
I_2Yb	7	0.1762	1	1
BiClTe	7	0.1543	1	1
$AuTe_2$	7	0.1305	1	1
BrCdI	7	0.1439	1	1
I_2Zn	7	0.1339	1	1
BaF_2	7	0.1469	1	1
BiBrTe	7	0.16	1	1
S_2W	7	0.11	1	1
Bi_2Pd	7	0.5665	1	1
GeI_2	7	0.1396	1	1
AsKSn	7	0.1456	1	1
$PbTe_2$	7	0.1431	1	1
I_2Nd	7	0.0063	1	1
Cl_2Cu	7	0.0998	1	1
I_2Tm	7	0.1778	1	1
S_2Sn	7	0.109	1	1
$SnTe_2$	7	0.1377	1	1
Cl_2V	7	0.1091	1	1
GeI_2	7	0.1525	1	1
STl_2	7	0.1478	1	1
BiITe	7	0.1671	1	1
DyI_2	7	0.1827	1	1
CeI_2	7	0.0046	1	1
$NbTe_2$	7	0.1088	1	1
Se_2Yb	7	0.1398	1	1
MoS_2	7	0.1099	1	1
$BiTe_2$	7	0.14	1	1
GdI_2	7	0.1631	1	1

Table showing the first 50 lattice matching combinations with the smallest number of atoms in the supercell.

Lattice matching - minimal strain

Formula	N° atoms	strain	cell size 1	cell size 2
Bi_2In_2	244	0.0	36	25
$H_2I_2Sr_2$	886	0.0001	100	81
Mg_3	764	0.0001	89	136
Bi_2Pd	291	0.0001	36	49
Te_2W	387	0.0002	48	65
$HNiO_2$	236	0.0002	20	39
$MoTe_2$	387	0.0002	48	65
K	869	0.0003	181	145
F_4Pb	510	0.0003	65	50
$Cu_2Rb_2Te_2$	294	0.0004	36	25
Te_2Zn	387	0.0004	48	65
$Br_2O_2Tb_2$	10	0.0004	1	1
CdH_2O_2	517	0.0005	48	65
Se_2Ta_4	590	0.0005	50	65
$Cu_2Na_2Te_2$	708	0.0005	81	64
$Br_2F_2Yb_2$	10	0.0006	1	1
Sn	485	0.0006	85	145
Bi_2Se_2	852	0.0006	116	97
Mg_2	814	0.0006	113	181
Se_2Ta_4	580	0.0006	49	64
$HfLiS_2$	452	0.0006	48	65
CCl_2Sc_2	914	0.0008	81	118
F_4Pb	501	0.0008	64	49
ClNZr	678	0.0008	81	118
$Cu_2K_2Te_2$	706	0.0008	85	61
LiO_2	412	0.0008	49	72
In	329	0.0008	58	97
$Cl_2S_2Tl_2$	618	0.001	81	49
$Br_2Ca_2F_2$	10	0.001	1	1
$Ag_2K_2Se_2$	962	0.001	113	85
O_4PSn	706	0.001	85	61
Cl_2Zr_2	796	0.001	81	118
CS_2Ta_2	946	0.001	79	126
Cl_2Ti	694	0.001	79	126
P_2	568	0.0011	79	126
Cl_4Cu_2	406	0.0011	64	25
FKO_2Se	540	0.0012	80	44
Cl_2Rb_2	136	0.0013	25	9
O_2Sn_2	8	0.0013	1	1
CdClO	387	0.0013	48	65
CNb_2S_2	946	0.0013	79	126
$CuGeO_3$	963	0.0013	112	103
Ho_2S_2	396	0.0013	50	49
F_2Zn	7	0.0013	1	1
PtS_2	387	0.0014	48	65
$AgNO_2$	672	0.0014	88	80
S_2V	583	0.0014	64	109
I_2Yb	679	0.0014	103	89
Ba_2Cu_2	768	0.0015	103	89
Ca_2O_2	340	0.0015	36	49

Table showing the first 50 lattice matching combinations with the smallest strain needed to build the supercell.

$CuBr_2$ (C2/m)

Structural and electronic properties

**Formula**	$CuBr_2$
**Spacegroup**	C2/m
**Prototype**	NbTe2
**Parent 3D**	Br_2Cu
**Source DB**	MPDS
**DB ID**	S1252390
**DF2-C09 Binding energy [meV/ Å^2]**	15.6
**RVV10 Binding energy [meV/ Å^2]**	N/A
**Band gap (PBE) [eV]**	0.0

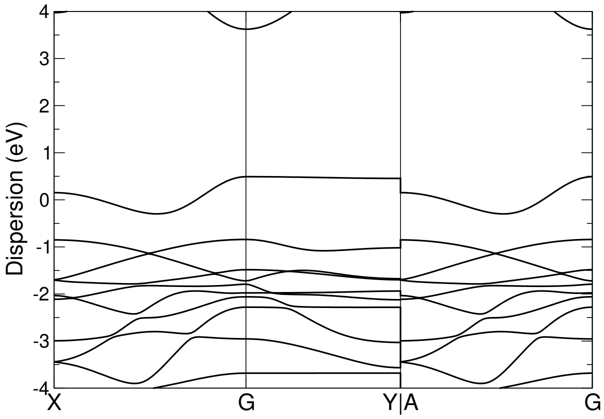

Band structure: Electronic band structure of $CuBr_2$ (C2/m) in a representative window around the Fermi energy and along a high-symmetry path.

Optimized crystal structure

Structural parameters: relaxed cell (top) and atomic positions (bottom) of $CuBr_2$ (C2/m) in Cartesian coordinates.

	x [Å]	y [Å]	z [Å]
$\mathbf{a_1}$	−0.00001241	3.46634109	0.00000000
$\mathbf{a_2}$	−3.93779806	1.73318220	0.00000000
$\mathbf{a_3}$	0.00000000	0.00000000	17.85889678

		x [Å]	y [Å]	z [Å]
●	Br	-0.77051585	1.73316983	-1.50026988
●	Cu	0.00000000	0.00000000	0.00000000
●	Br	-3.16728221	0.00001236	1.50026988

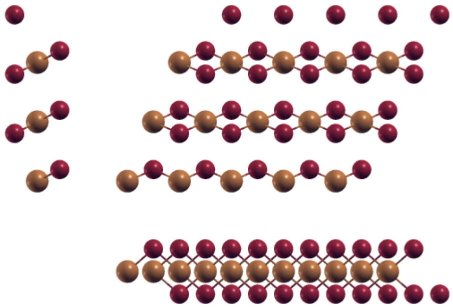

Orthographic projections: views of $CuBr_2$ (C2/m) as seen from the x axis (left), the y axis (bottom) and the z axis (center).

Lattice matching - minimal size

Formula	N° atoms	strain	cell size 1	cell size 2
Na	4	0.6449	1	1
InSe	5	0.9807	1	1
AgTl	5	0.1727	1	1
C_2	5	0.1433	1	1
PbTe	5	0.9892	1	1
CaCl	5	0.1129	1	1
CrS_2	6	1.6798	1	1
$MoTe_2$	6	0.6523	1	1
Br_2Zn	6	0.7405	1	1
HfS_2	6	0.6806	1	1
$AsSn_2$	6	0.7155	1	1
I_2Pr	6	0.1444	1	1
I_2Yb	6	1.0996	1	1
Cl_2Zn	6	0.1228	1	1
BiBrTe	6	1.0263	1	1
$RhTe_2$	6	0.7256	1	1
Ba_2Hg	6	0.6394	1	1
PtS_2	6	0.6483	1	1
CNRb	6	0.356	1	1
$CoTe_2$	6	0.6817	1	1
Se_2Ti	6	0.6396	1	1
AsKSn	6	0.9559	1	1
Te_2W	6	0.6528	1	1
I_2Nd	6	0.1454	1	1
Cl_2V	6	0.5081	1	1
GeI_2	6	0.9903	1	1
STl_2	6	0.9671	1	1
$PtSe_2$	6	0.7178	1	1
$MnSe_2$	6	0.1129	1	1
Br_2Ni	6	0.6874	1	1
CeI_2	6	0.1436	1	1
Cl_2Mg	6	0.6876	1	1
CNNa	6	1.0763	1	1
$CrSe_2$	6	0.5049	1	1
F_2Ni	6	0.1199	1	1
I_2La	6	0.1504	1	1
$CrSe_2$	6	0.5114	1	1
F_2Zn	6	0.1404	1	1
Sm	6	0.1177	1	3
Fe_2Te_2	7	0.1322	1	1
Bi_2In_2	7	0.2578	1	1
Ca_2Cl_2	7	0.1326	1	1
Cu_2I_2	7	0.1691	1	1
CdClHO	7	0.7114	1	1
Cu_2Sr_2	7	1.0313	1	1
Cl_2OOs	7	0.1224	1	1
$LiMnTe_2$	7	0.9918	1	1
Ir_2P_2	7	0.1451	1	1
Bi_2Mn_2	7	0.096	1	1
Ag_2Br_2	7	0.1509	1	1

Table showing the first 50 lattice matching combinations with the smallest number of atoms in the supercell.

Lattice matching - minimal strain

Formula	N° atoms	strain	cell size 1	cell size 2
$Cl_2H_2Sc_2$	600	0.0002	56	72
Br_2Ti	384	0.0004	56	72
N_2Re	351	0.0004	40	77
AsKSn	483	0.0004	85	76
Se_2Ta	384	0.0004	56	72
Cl_2Y_2	568	0.0005	76	85
$Br_2H_2Zr_2$	600	0.0005	56	72
AgTl	56	0.0006	12	10
Br_2Cr	384	0.0006	56	72
$Dy_2I_2S_2$	552	0.0007	72	56
I_2La_2	559	0.0007	85	76
$Br_2O_2V_2$	639	0.0008	67	73
Sn	233	0.0008	51	80
F_4Pb	861	0.0008	132	93
Cl_2Sc_2	456	0.0009	56	72
Cl_4KTl	465	0.0009	95	30
Cl_4Mg_2	624	0.0009	130	39
Cl_2ORu	593	0.0009	83	86
$CrTe_2$	384	0.0009	56	72
$ReSe_2$	390	0.001	56	74
Br_2V	390	0.001	56	74
$Br_2N_2Ti_2$	438	0.001	48	49
O_4PSn	750	0.001	108	71
Br_2Zn	483	0.001	76	85
$I_2O_2Y_2$	969	0.001	117	103
N_4	476	0.0011	52	80
Ga_2Se_2	568	0.0011	76	85
$I_2O_2Y_2$	894	0.0011	108	95
$Br_2Nd_2O_2$	969	0.0011	117	103
BN	281	0.0011	35	88
Na	366	0.0011	86	108
$Br_2Nd_2O_2$	894	0.0011	108	95
K	321	0.0012	86	63
$As_2Mg_2Na_2$	636	0.0012	86	63
$Cu_2K_2Te_2$	720	0.0012	104	68
$Bi_2Br_2O_2$	969	0.0012	117	103
Sn_2Te_2	608	0.0012	108	71
$MnNaTe_2$	615	0.0012	93	84
Se_2Ti	582	0.0012	86	108
$Ga_2I_2Y_2$	711	0.0012	85	76
$Bi_2Br_2O_2$	837	0.0013	101	89
$Br_2Nd_2O_2$	819	0.0013	99	87
SSb_2Te_2	699	0.0013	93	84
$I_2O_2Y_2$	819	0.0013	99	87
$I_2S_2Sm_2$	558	0.0013	74	56
BrCdI	531	0.0013	93	84
$Cl_2O_2Yb_2$	810	0.0013	84	93
CNNa	495	0.0013	91	74
$Cl_2Cr_2O_2$	945	0.0013	95	110
Te_2Ti	483	0.0013	76	85

Table showing the first 50 lattice matching combinations with the smallest strain needed to build the supercell.

$CuCl_2$ (C2/m)

Structural and electronic properties

**Formula**	$CuCl_2$
**Spacegroup**	C2/m
**Prototype**	NbTe2
**Parent 3D**	$CuCl_2$
**Source DB**	COD
**DB ID**	9001506
**DF2-C09 Binding energy [meV/ Å^2]**	13.37
**RVV10 Binding energy [meV/ Å^2]**	19.67
**Band gap (PBE) [eV]**	0.16

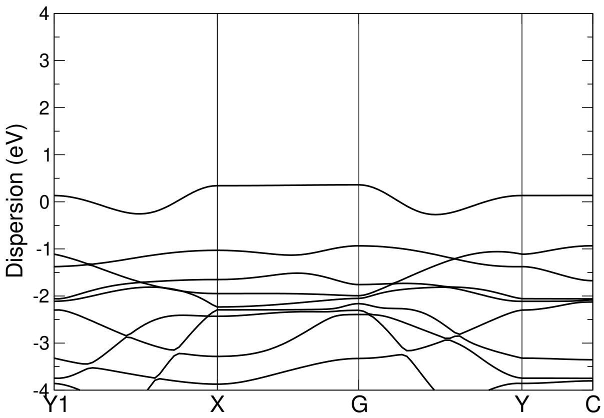

Band structure: Electronic band structure of $CuCl_2$ (C2/m) in a representative window around the Fermi energy and along a high-symmetry path.

Optimized crystal structure

Structural parameters: relaxed cell (top) and atomic positions (bottom) of $CuCl_2$ (C2/m) in Cartesian coordinates.

	x [Å]	y [Å]	z [Å]
$\mathbf{a_1}$	4.12449133	0.15049408	0.00000000
$\mathbf{a_2}$	2.70683904	3.11562190	0.00000000
$\mathbf{a_3}$	0.00000000	0.00000000	22.75964316

		x [Å]	y [Å]	z [Å]
●	Cl	5.96493224	2.85188372	12.76034848
●	Cu	3.22548543	1.54213137	11.37990100
●	Cl	0.48583342	0.23228121	9.99921525

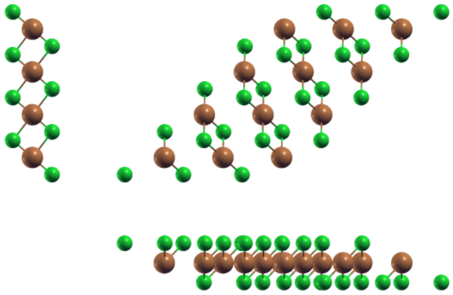

Orthographic projections: views of $CuCl_2$ (C2/m) as seen from the x axis (left), the y axis (bottom) and the z axis (center).

Lattice matching - minimal size

Formula	N° atoms	strain	cell size 1	cell size 2
Tl	4	0.0979	1	1
K	4	0.4991	1	1
Tl	4	0.0706	1	1
InSe	5	0.1118	1	1
Nd	5	0.1409	1	2
HgO	5	0.1009	1	1
AsSb	5	2.9203	1	1
Bi_2	5	0.1184	1	1
AgTl	5	0.2024	1	1
Ag_2	5	0.8319	1	1
As_2	5	0.4548	1	1
PbTe	5	0.1138	1	1
Gd	5	0.1335	1	2
Cl_2Zn	6	0.4523	1	1
Cl_2Mn	6	0.0685	1	1
CdI_2	6	0.1157	1	1
$MoTe_2$	6	0.4413	1	1
$AgTe_2$	6	0.0985	1	1
$MoSe_2$	6	0.0708	1	1
Ba_2Pt	6	0.8309	1	1
S_2Ta	6	0.0679	1	1
Br_2Ca	6	0.1172	1	1
Te_2V	6	0.4458	1	1
I_2Pr	6	0.1692	1	1
Ca_2Si	6	0.8497	1	1
Br_2Co	6	0.4537	1	1
BiClTe	6	0.1162	1	1
Ca_2N	6	0.456	1	1
BrCdI	6	0.1041	1	1
LiO_2	6	0.2224	1	1
Cl_2Zn	6	0.1424	1	1
MnO_2	6	0.153	1	1
HgI_2	6	0.4547	1	1
S_2Ti	6	0.0664	1	1
Mg_3	6	0.097	1	1
NbS_2	6	0.068	1	1
BaF_2	6	0.1076	1	1
Te_2Zn	6	0.4409	1	1
BiBrTe	6	0.1227	1	1
Bi_2Pd	6	0.1042	1	1
Br_2Mn	6	0.4494	1	1
Cl_2Co	6	0.0665	1	1
NbS_2	6	0.0695	1	1
ClNZr	6	0.0656	1	1
Cl_2Fe	6	0.0667	1	1
CdClO	6	0.4446	1	1
S_2Ta	6	0.0698	1	1
Se_2V	6	0.0703	1	1
AsKSn	6	0.106	1	1
Te_2W	6	0.4416	1	1

Table showing the first 50 lattice matching combinations with the smallest number of atoms in the supercell.

Lattice matching - minimal strain

Formula	N° atoms	strain	cell size 1	cell size 2
NbS_2	720	0.0001	105	135
$I_2O_2Yb_2$	852	0.0003	108	88
Bi_2Pd	525	0.0003	82	93
I_2Pr	588	0.0003	108	88
Fe_2Se_2	334	0.0004	50	46
HgI_2	243	0.0005	50	31
$C_2Br_2Tb_2$	654	0.0005	78	70
$Si_2Te_2Zr_2$	426	0.0005	50	46
F_2Ni	288	0.0006	50	46
Ni_2Te_2	436	0.0006	64	61
AsSb	366	0.0006	74	72
Ir_2P_2	676	0.0006	108	88
$CdCl_2$	375	0.0006	64	61
NS_2Ta	777	0.0006	83	132
Cu_2O_2	428	0.0007	68	56
I_2Mn	375	0.0007	64	61
$Br_2O_2Sm_2$	852	0.0007	108	88
$Cl_2Er_2H_2$	558	0.0007	64	61
I_2Nd	588	0.0008	108	88
$C_2Br_2Gd_2$	654	0.0008	78	70
CoI_2	438	0.0008	74	72
I_2Ni	375	0.0008	64	61
$I_2S_2Tl_2$	426	0.0008	50	46
PTe_2Zr_2	582	0.0008	74	72
$InSe_2$	375	0.0008	64	61
Ag_2I_2	274	0.0009	50	31
Ho_2S_2	214	0.0009	34	28
FHOZn	480	0.0009	56	78
MoS_2	384	0.0009	53	75
S_2W	384	0.0009	53	75
$Br_2Eu_2O_2$	852	0.0009	108	88
MoS_2	384	0.0009	53	75
AlH_4Na	546	0.0009	86	48
$GeTe_2$	375	0.0009	64	61
CrO_2	417	0.001	48	91
$Cl_2O_2Sc_2$	318	0.001	38	34
CeI_2	588	0.001	108	88
Cu_2Se_2	334	0.001	50	46
CrO_2	270	0.001	31	59
I_2Ti	438	0.0011	74	72
H_2Si_2	436	0.0011	64	61
$LiOS_2Ti$	990	0.0011	105	135
S_2V	384	0.0011	53	75
Ni_2Se_2	676	0.0012	108	88
N_3Na	386	0.0012	70	44
$I_2O_2Tm_2$	456	0.0012	58	47
Bi_2Pd	519	0.0012	81	92
I_2Nd	315	0.0012	58	47
Ca_2O_2	618	0.0013	82	93
$Br_2O_2Sm_2$	456	0.0013	58	47

Table showing the first 50 lattice matching combinations with the smallest strain needed to build the supercell.

$CuGeO_3$ (Pm)

Structural and electronic properties

**Formula**	$CuGeO_3$
**Spacegroup**	Pm
**Prototype**	CuGeO3
**Parent 3D**	$Cu_2Ge_2O_6$
**Source DB**	MPDS
**DB ID**	S1821730
**DF2-C09 Binding energy [meV/ Å^2]**	14.72
**RVV10 Binding energy [meV/ Å^2]**	N/A
**Band gap (PBE) [eV]**	0.0

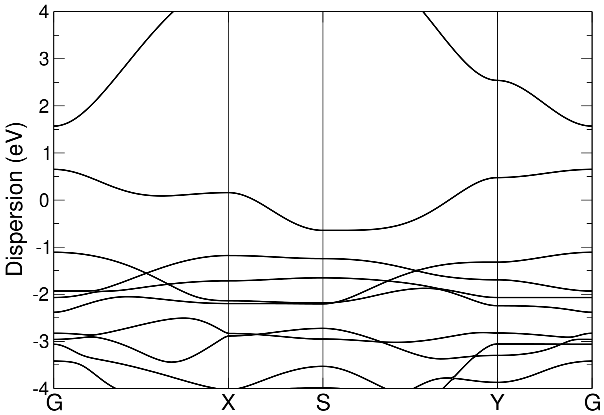

Band structure: Electronic band structure of $CuGeO_3$ (Pm) in a representative window around the Fermi energy and along a high-symmetry path.

Optimized crystal structure

Structural parameters: relaxed cell (top) and atomic positions (bottom) of $CuGeO_3$ (Pm) in Cartesian coordinates.

	x [Å]	y [Å]	z [Å]
$\mathbf{a_1}$	−2.97423286	0.00000000	0.00000000
$\mathbf{a_2}$	0.00000000	−5.47620939	0.00000000
$\mathbf{a_3}$	0.00000000	0.00000000	15.97498115

	x [Å]	y [Å]	z [Å]
Cu	-1.48711643	-1.60014792	0.58332535
O	0.00000000	-2.85432831	0.60140471
O	0.00000000	-0.34596387	0.60141001
Ge	0.00000000	-4.33825293	-0.39461303
O	-1.48711643	-4.33825830	-1.39152705

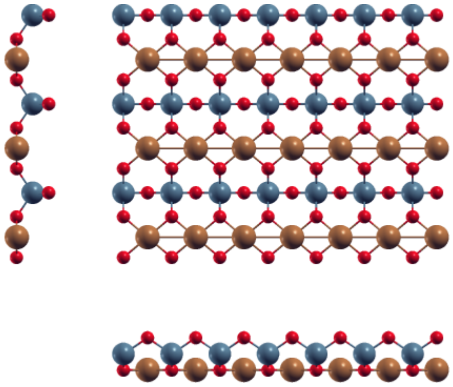

Orthographic projections: views of $CuGeO_3$ (Pm) as seen from the x axis (left), the y axis (bottom) and the z axis (center).

Lattice matching - minimal size

Formula	N° atoms	strain	cell size 1	cell size 2
Tl	7	0.1826	1	2
Sn	7	0.0274	1	2
Na	7	0.5535	1	2
In	7	0.0309	1	2
In	7	0.0215	1	2
DyI_2	8	3.9895	1	1
$AgNO_2$	9	0.3083	1	1
Cu_2O_2	9	0.0241	1	1
Pb_2Se_2	9	0.3471	1	1
C_2Li_2	9	0.2168	1	1
P_4	9	0.5396	1	1
Bi_2Se_2	9	0.3093	1	1
P_2	9	0.1762	1	2
AsI_2La_2	10	0.2526	1	1
S_2V	11	0.0242	1	2
MoS_2	11	0.0248	1	2
Cl_2Mn	11	0.1898	1	2
$MoSe_2$	11	0.1817	1	2
$Cl_2O_2V_2$	11	0.3774	1	1
$Ho_2I_2S_2$	11	0.3156	1	1
S_2Ta	11	0.1925	1	2
FeO_2	11	0.5529	1	2
$CuTe_2$	11	0.5844	1	2
Br_2Cu	11	0.4733	1	2
ReS_2	11	0.0159	1	2
CrO_2	11	0.5208	1	2
Cl_2Ti	11	0.1764	1	2
$K_2O_2Tl_2$	11	1.0075	1	1
MnO_2	11	0.527	1	2
NbS_2	11	0.1919	1	2
Te_2Zn	11	0.965	1	2
S_2W	11	0.0249	1	2
N_2W	11	0.0155	1	2
NbS_2	11	0.1864	1	2
CNRb	11	1.741	1	2
S_2Ta	11	0.1853	1	2
Se_2V	11	0.1836	1	2
$I_2S_2Tb_2$	11	0.3229	1	1
$I_2Se_2Tm_2$	11	0.3277	1	1
Cl_2V	11	0.0286	1	2
$I_2S_2Tb_2$	11	3.997	1	1
$Dy_2I_2S_2$	11	0.3195	1	1
MoS_2	11	0.025	1	2
$CrSe_2$	11	0.0274	1	2
$I_2Se_2Yb_2$	11	0.3254	1	1
Mo_2Te_4	11	1.546	1	1
Te_4W_2	11	1.0045	1	1
O_2Pt	11	0.0203	1	2
Se_2W	11	0.1819	1	2
$HNiO_2$	13	0.0194	1	2

Table showing the first 50 lattice matching combinations with the smallest number of atoms in the supercell.

Lattice matching - minimal strain

Formula	N° atoms	strain	cell size 1	cell size 2
$H_2Na_2O_2$	536	0.0003	40	56
$I_2Sb_2Te_2$	518	0.0005	64	33
Nd	227	0.0005	30	77
$NiTe_2$	375	0.0005	42	55
$AgClO_4$	925	0.0006	101	70
$Br_2Ca_2H_2$	735	0.0006	63	70
N_4	605	0.0006	49	90
$Ca_2Mn_2Si_2$	735	0.0006	63	70
$Br_2Ho_2O_2$	735	0.0007	63	70
$H_2I_2Sr_2$	968	0.0008	94	83
$SiTe_2$	375	0.0008	42	55
Bi_2Se_4	634	0.0008	80	39
$GeNi_3Te_2$	244	0.0008	20	24
Te_2Zn	510	0.0008	54	80
CaClHO	430	0.0008	42	55
$AgNO_2$	725	0.0008	81	80
I_4Sr_2	180	0.0009	24	10
$Cl_2O_2Tm_2$	540	0.0009	42	55
$Cl_2S_2Tl_2$	869	0.0009	97	64
Mo_2Te_4	461	0.0009	49	36
NaPSn	172	0.0009	20	24
$Br_2Er_2O_2$	735	0.001	63	70
$Cl_2Er_2O_2$	540	0.001	42	55
F_2Zn	851	0.001	103	112
CdO_2	553	0.001	56	91
Pt_2Te_2	196	0.001	20	24
$Br_2O_2Yb_2$	979	0.0011	83	94
$Br_2Hf_2N_2$	210	0.0011	18	20
Br_2Ca_3Si	504	0.0011	48	44
Se_2Zr	375	0.0011	42	55
Br_2Cu_2	963	0.0013	103	112
$C_2I_2La_2$	776	0.0014	70	71
Bi_2Se_2	104	0.0014	12	11
$AgClO_2$	736	0.0014	76	89
$Er_2F_2Se_2$	244	0.0014	20	24
$CrTe_2$	622	0.0014	65	99
Fe_2Te_2	791	0.0014	83	94
As_2O_3	985	0.0014	120	77
Cl_2Sc_2	721	0.0015	65	99
$AuCrTe_4$	393	0.0015	45	28
La_2S_2	340	0.0015	40	35
Cl_2Hf_2	705	0.0015	61	100
$HfTe_2$	172	0.0016	20	24
I_2V	615	0.0016	69	90
Br_2Cr	622	0.0016	65	99
O_2Sn_2	643	0.0016	63	82
In_2Se_3	220	0.0016	20	24
Au_2Br_2	602	0.0016	70	63
F_2Na	375	0.0016	42	55
$Ag_2K_2Te_2$	520	0.0017	56	40

Table showing the first 50 lattice matching combinations with the smallest strain needed to build the supercell.

CuI (P-3m1)

Structural and electronic properties

**Formula**	CuI
**Spacegroup**	P-3m1
**Prototype**	PtTe
**Parent 3D**	Cu_6I_6
**Source DB**	ICSD
**DB ID**	30363
**DF2-C09 Binding energy [meV/ Å^2]**	14.68
**RVV10 Binding energy [meV/ Å^2]**	20.82
**Band gap (PBE) [eV]**	2.04

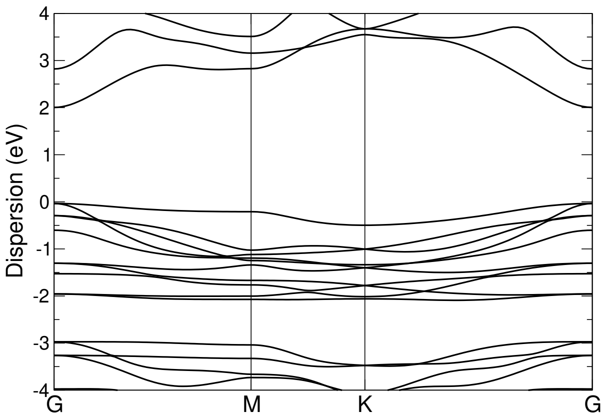

Band structure: Electronic band structure of CuI (P-3m1) in a representative window around the Fermi energy and along a high-symmetry path.

Optimized crystal structure

Structural parameters: relaxed cell (top) and atomic positions (bottom) of CuI (P-3m1) in Cartesian coordinates.

	x [Å]	y [Å]	z [Å]
$\mathbf{a_1}$	4.16187537	−0.00000000	0.00000000
$\mathbf{a_2}$	−2.08093768	3.60428979	0.00000000
$\mathbf{a_3}$	0.00000000	0.00000000	23.91576132

		x [Å]	y [Å]	z [Å]
●	Cu	0.00000000	0.00000000	11.26287865
●	Cu	2.08093768	1.20142993	12.65286238
●	I	0.00000000	0.00000000	13.87026314
●	I	2.08093768	1.20142993	10.04549818

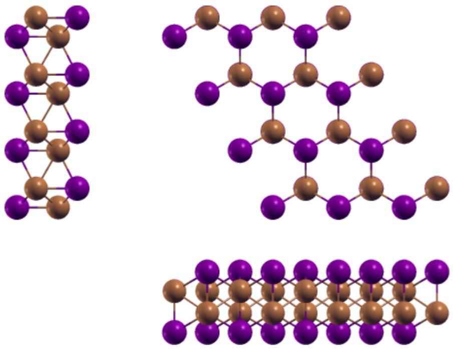

Orthographic projections: views of CuI (P-3m1) as seen from the x axis (left), the y axis (bottom) and the z axis (center).

Lattice matching - minimal size

Formula	N° atoms	strain	cell size 1	cell size 2
K	5	0.4489	1	1
Na	5	1.5289	1	1
InSe	6	0.0095	1	1
AgTl	6	0.162	1	1
Ag_2	6	0.4625	1	1
As_2	6	1.5865	1	1
Sb_2	6	0.0065	1	1
CaCl	6	0.1176	1	1
I_2Mg	7	0.0009	1	1
$MoTe_2$	7	1.5451	1	1
PSn_2	7	0.2554	1	1
Ba_2Pt	7	0.4618	1	1
Br_2Zn	7	0.2705	1	1
HfS_2	7	0.2472	1	1
$AsSn_2$	7	0.2606	1	1
Te_2V	7	1.5589	1	1
I_2Pr	7	0.1444	1	1
$CuTe_2$	7	0.2467	1	1
S_2Zr	7	0.2544	1	1
Br_2La	7	0.0006	1	1
Ca_2Si	7	0.4732	1	1
Br_2Co	7	1.5833	1	1
Ca_2N	7	1.5903	1	1
BrCdI	7	0.0022	1	1
Cl_2Zn	7	0.1258	1	1
Te_2Ti	7	0.2709	1	1
BaF_2	7	0.0056	1	1
$RhTe_2$	7	0.2646	1	1
GeI_2	7	0.0028	1	1
Cl_2Ni	7	1.4492	1	1
PtS_2	7	1.5363	1	1
$CoTe_2$	7	0.2476	1	1
CdClO	7	1.5554	1	1
AsKSn	7	0.0041	1	1
Te_2W	7	1.5462	1	1
$PbTe_2$	7	0.0012	1	1
I_2Nd	7	0.1453	1	1
$NiTe_2$	7	0.2757	1	1
S_2Sn	7	0.2548	1	1
$SnTe_2$	7	0.005	1	1
Sn	7	0.6243	1	3
I_2Pb	7	0.4664	1	1
STl_2	7	0.0065	1	1
$PtSe_2$	7	0.2615	1	1
OTl_2	7	1.5567	1	1
Br_2Fe	7	1.5838	1	1
GeS_2	7	0.1124	1	1
$TaTe_2$	7	0.2598	1	1
$MnSe_2$	7	0.1175	1	1
Br_2Ni	7	0.2498	1	1

Table showing the first 50 lattice matching combinations with the smallest number of atoms in the supercell.

Lattice matching - minimal strain

Formula	N° atoms	strain	cell size 1	cell size 2
Br_2Gd_2Ge	9	0.0	1	1
PSn_2	447	0.0	57	73
Ga_2Se_2	724	0.0	81	100
Ga_2S_2	452	0.0	49	64
N_3W_2	696	0.0	49	100
$HNiO_2$	296	0.0001	25	49
Te_2W	291	0.0001	36	49
$Br_2Hf_2N_2$	486	0.0002	48	49
$Hf_2I_2N_2$	742	0.0002	64	81
CdClHO	580	0.0002	64	81
I_2Pb	583	0.0002	91	73
$RhTe_2$	565	0.0002	73	91
Se_2Ta	208	0.0002	25	36
$MoTe_2$	291	0.0003	36	49
Ba_2Pt	643	0.0003	100	81
Ca_2N	343	0.0003	43	57
PTe_2Ti_2	516	0.0003	49	64
$NbSe_2$	208	0.0003	25	36
Gd_2I_2	8	0.0003	1	1
CdH_2O_2	389	0.0003	36	49
Cl_2OV	100	0.0003	11	14
$Br_2Hf_2N_2$	514	0.0003	43	57
Se_2V	139	0.0003	16	25
$F_2Se_2Y_2$	412	0.0004	49	36
$TaTe_2$	499	0.0004	64	81
Br_2Ni	388	0.0004	49	64
Te_2Zn	291	0.0004	36	49
Cl_2Mg	388	0.0005	49	64
Ag_2	562	0.0005	100	81
S_2Sn	447	0.0005	57	73
Bi_2Se_3	9	0.0005	1	1
CdClHO	656	0.0006	73	91
H_2MnO_2	551	0.0006	39	79
Br_2La	7	0.0006	1	1
$Br_2N_2Ti_2$	784	0.0007	73	82
$HfLiS_2$	340	0.0007	36	49
Bi_2Te_2	400	0.0007	57	43
Cl_2Ni	208	0.0007	25	36
Cl_2Y_2	724	0.0007	81	100
$AsSe_2$	208	0.0007	25	36
LiO	358	0.0008	49	81
$Cl_2H_2Lu_2$	580	0.0008	49	64
S_2Zr	447	0.0008	57	73
Au_2Se_2	412	0.0008	63	40
C_2F_2	356	0.0008	25	64
As_2	286	0.0008	43	57
$I_2N_2Ti_2$	936	0.0009	90	96
$Cl_2N_2Zr_2$	514	0.0009	43	57
CCl_2Lu_2	457	0.0009	43	57
I_2Mg	7	0.0009	1	1

Table showing the first 50 lattice matching combinations with the smallest strain needed to build the supercell.

CuO_2 (P-1)

Structural and electronic properties

**Formula**	CuO_2
**Spacegroup**	P-1
**Prototype**	FeO2
**Parent 3D**	CuO_2
**Source DB**	ICSD
**DB ID**	157973
**DF2-C09 Binding energy [meV/ Å^2]**	20.96
**RVV10 Binding energy [meV/ Å^2]**	N/A
**Band gap (PBE) [eV]**	0.0

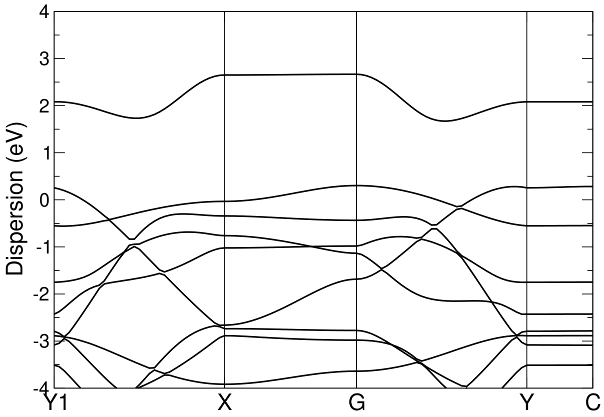

Band structure: Electronic band structure of CuO_2 (P-1) in a representative window around the Fermi energy and along a high-symmetry path.

Optimized crystal structure

Structural parameters: relaxed cell (top) and atomic positions (bottom) of CuO_2 (P-1) in Cartesian coordinates.

		x [Å]	y [Å]	z [Å]
$\mathbf{a_1}$		1.35702124	3.50966112	0.00000000
$\mathbf{a_2}$		−1.35702124	3.50966112	0.00000000
$\mathbf{a_3}$		0.00000000	0.00000000	16.53455927
		x [Å]	y [Å]	z [Å]
•	O	0.00000000	0.00000000	-9.45299191
•	Cu	0.00000000	3.50966112	-8.26727963
•	O	0.00000000	0.00000000	-7.08156735

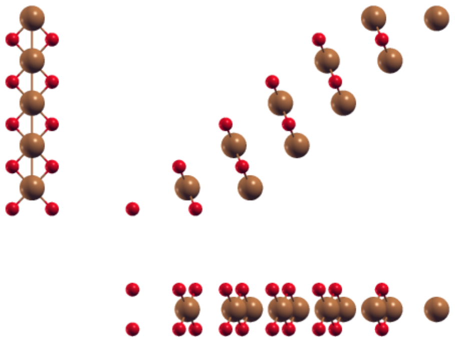

Orthographic projections: views of CuO_2 (P-1) as seen from the x axis (left), the y axis (bottom) and the z axis (center).

Lattice matching - minimal size

Formula	N° atoms	strain	cell size 1	cell size 2
HgO	5	0.4956	1	1
$CuTe_2$	6	3.8292	1	1
Te_2Zn	6	3.6924	1	1
GeI_2	6	0.5339	1	1
$SnTe_2$	6	0.217	1	1
Se_2Yb	6	0.5345	1	1
$BiTe_2$	6	0.5353	1	1
Br_2Er_2	7	0.2028	1	1
Cl_2OV	7	0.1587	1	1
Br_2Hf_2	7	3.5136	1	1
$AgBrO_2$	7	0.2069	1	1
Br_2Gd_2	7	0.2036	1	1
$AsCuLi_2$	7	0.5325	1	1
Fe_2S_2	7	0.2079	1	1
Br_2Ho_2	7	0.1998	1	1
O_2Sn_2	7	0.1573	1	1
$AgClO_2$	7	0.1872	1	1
Ga_2Te_2	7	0.5332	1	1
Br_2HLa	7	0.5312	1	1
$CeLi_2P_2$	8	0.5301	1	1
Ba_2Ni_3	8	0.5333	1	1
Sb_2Se_2Te	8	0.5363	1	1
Br_2PY_2	8	0.1965	1	1
Li_2P_2Pr	8	0.5246	1	1
Sb_2Se_2Te	8	0.5376	1	1
CBr_2Y_2	8	2.1002	1	1
I_2La_2Sb	8	6.2007	1	1
Ga_2S_3	8	3.6642	1	1
$Gd_2I_2S_2$	9	6.0848	1	1
$I_2Pr_2Si_2$	9	0.533	1	1
Cr_2O_4	9	0.1175	1	1
PbS_2	9	0.3174	2	1
$Se_2Si_2Zr_2$	9	0.2012	1	1
$Br_2Cr_2S_2$	9	0.5656	1	1
$AuTe_2$	9	1.3695	1	2
$PdTe_2$	9	0.0911	2	1
BH_4Li	9	0.5282	1	1
$Ga_2Ge_2Te_2$	9	0.5249	1	1
$CrTe_2$	9	0.8194	1	2
Se_2Ti	9	0.8352	1	2
Br_2Ti	9	0.817	1	2
$F_2Lu_2Se_2$	9	0.2116	1	1
$F_2Ho_2Se_2$	9	0.1948	1	1
BrNZr	9	0.8057	1	2
GeS_2	9	0.1047	2	1
Br_2Cr	9	0.8181	1	2
DyI_2	9	3.1541	1	2
I_2Ti	9	0.3231	2	1
Se_2Ta	9	0.8138	1	2
$PtTe_2$	9	1.3669	1	2

Table showing the first 50 lattice matching combinations with the smallest number of atoms in the supercell.

Lattice matching - minimal strain

Formula	N° atoms	strain	cell size 1	cell size 2
Cu_2Te_2	284	0.0004	48	35
$SbSe_2Tl$	330	0.0005	70	30
Tl	249	0.0006	63	60
Mg_4	429	0.0006	63	60
$K_2O_2Tl_2$	606	0.0008	112	45
$TaTe_2$	432	0.0009	80	64
Cl_2Hf_2	171	0.0011	25	24
Br_2Hf_2	716	0.0011	108	98
Nd	18	0.0011	4	6
DyI_2	462	0.0011	100	54
As_2	150	0.0011	32	27
I_2Pb	435	0.0011	96	49
Ca_2N	177	0.0011	32	27
CCl_2Lu_2	231	0.0011	32	27
Br_3Cs	198	0.0011	50	12
Te_4W_2	906	0.0012	162	70
In_2Se_2	34	0.0012	6	4
$Dy_2I_2S_2$	624	0.0012	100	54
Ga_2S_3	869	0.0012	118	103
Se_2Ti	513	0.0012	91	80
$I_2Pr_2S_2$	582	0.0012	96	49
Br_2Fe	177	0.0012	32	27
$I_2S_2Tb_2$	624	0.0012	100	54
$Hf_2Se_2Si_2$	99	0.0012	13	10
$Br_2O_2Ti_2$	249	0.0012	35	24
Br_2Co	177	0.0013	32	27
$Cl_2Ga_2Te_2$	273	0.0013	51	20
I_4Sr_2	219	0.0013	49	12
$Cl_2Er_2O_2$	795	0.0013	105	80
Br_2Zr_2	593	0.0013	91	80
O_2Zn	177	0.0013	27	32
$Cl_2O_2Ti_2$	354	0.0013	48	35
Cl_2La_2	34	0.0013	6	4
Cl_2Ni	483	0.0013	84	77
As_2Fe_2	79	0.0013	13	10
Au_2Se_2	457	0.0013	99	40
$NbSe_2$	483	0.0014	84	77
$Bi_2Cl_2O_2$	678	0.0014	100	63
$Cu_2Se_2Tl_2$	135	0.0014	21	12
Se_2Ta	483	0.0014	84	77
$Br_2Hf_2N_2$	258	0.0014	32	27
Br_2Hg_3	265	0.0014	75	8
H_2Na_2Pd	124	0.0014	18	14
$FeSe_2$	477	0.0015	75	84
$Cl_2Ho_2O_2$	846	0.0015	112	85
$Ca_2Ge_2Mn_2$	678	0.0015	100	63
S_2Sn_2	171	0.0015	33	18
$LiNbS_2$	171	0.0015	25	24
F_4Pb	355	0.0015	65	32
$Dy_2I_2S_2$	462	0.0015	74	40

Table showing the first 50 lattice matching combinations with the smallest strain needed to build the supercell.

CuTe (Pmmn)

Structural and electronic properties

**Formula**	CuTe
**Spacegroup**	Pmmn
**Prototype**	CuTe
**Parent 3D**	Cu_2Te_2
**Source DB**	COD
**DB ID**	1526237
**DF2-C09 Binding energy [meV/ Å^2]**	22.2
**RVV10 Binding energy [meV/ Å^2]**	27.0
**Band gap (PBE) [eV]**	N/A

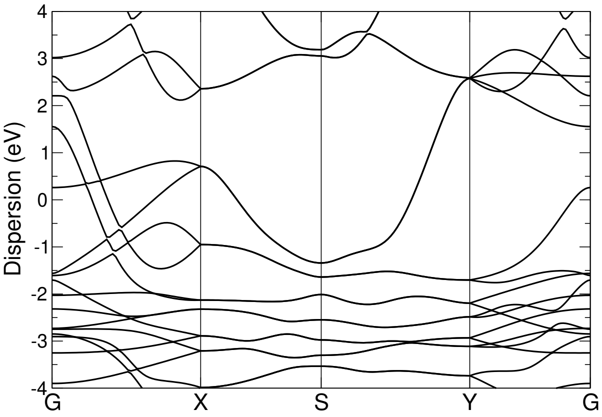

Band structure: Electronic band structure of CuTe (Pmmn) in a representative window around the Fermi energy and along a high-symmetry path.

Optimized crystal structure

Structural parameters: relaxed cell (top) and atomic positions (bottom) of CuTe (Pmmn) in Cartesian coordinates.

		x [Å]	y [Å]	z [Å]
$\mathbf{a_1}$		3.25949565	0.00000000	0.00000000
$\mathbf{a_2}$		0.00000000	4.01520561	0.00000000
$\mathbf{a_3}$		0.00000000	0.00000000	23.95023414
		x [Å]	y [Å]	z [Å]
●	Cu	0.00000000	2.00760281	11.71372175
●	Cu	1.62974783	0.00000000	12.23650835
●	Te	0.00000000	0.00000000	10.02594285
●	Te	1.62974783	2.00760281	13.92429571

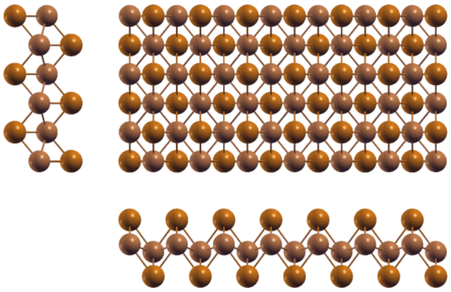

Orthographic projections: views of CuTe (Pmmn) as seen from the x axis (left), the y axis (bottom) and the z axis (center).

Lattice matching - minimal size

Formula	N° atoms	strain	cell size 1	cell size 2
Tl	5	0.2412	1	1
K	5	0.2534	1	1
Ag_2	6	0.2627	1	1
CaCl	6	0.0491	1	1
$AgTe_2$	7	0.245	1	1
Ba_2Pt	7	0.2622	1	1
Ca_2Si	7	0.2701	1	1
Cl_2Zn	7	0.2793	1	1
BaF_2	7	1.9383	1	1
I_2Pb	7	0.2654	1	1
GeS_2	7	0.3146	1	1
$MnSe_2$	7	0.0491	1	1
DyI_2	7	0.243	1	1
F_2Ni	7	0.2729	1	1
F_2Zn	7	0.3136	1	1
Cl_2OOs	8	0.2637	1	1
$AgCuTe_2$	8	0.017	1	1
O_2Sn_2	8	0.0441	1	1
Cu_2S_2	8	0.3079	1	1
Cl_2OV	8	0.0327	1	1
Br_2Cu_2	8	0.3112	1	1
Fe_2Se_2	8	0.2721	1	1
As_2Co_2	8	0.0507	1	1
Cu_2Te_2	8	0.2835	1	1
$AgBrO_2$	8	0.51	1	1
Ge_2S_2	8	0.3011	1	1
C_2Li_2	8	0.2341	1	1
P_4	8	0.5144	1	1
Br_2OV	8	0.3287	1	1
O_2Sn_2	8	0.3089	1	1
Mg_4	8	0.2407	1	1
P_2Rh_2	8	0.3067	1	1
Fe_2S_2	8	0.0486	1	1
F_2Tl_2	8	0.3069	1	1
Sb_2Te_2	8	0.2678	1	1
Co_2S_2	8	0.0493	1	1
As_2Fe_2	8	0.3161	1	1
Cu_2Se_2	8	0.2738	1	1
Ag_2Te_2	8	0.2939	1	1
$AgClO_2$	8	0.1351	1	1
Fe_2SeTe	8	0.2887	1	1
O_2Sn_2	8	0.1958	1	1
Co_2Se_2	8	0.0512	1	1
Ca_2Cl_2	8	0.0504	1	1
Hf_3Te_2	9	0.2776	1	1
F_4Nb	9	0.3717	1	1
NaO_4	9	0.3625	1	1
$AgNO_3$	9	0.3644	1	1
I_2La_2Sb	9	0.254	1	1
I_2La_2Te	9	0.2495	1	1

Table showing the first 50 lattice matching combinations with the smallest number of atoms in the supercell.

Lattice matching - minimal strain

Formula	N° atoms	strain	cell size 1	cell size 2
C_2F_2	464	0.0004	36	80
CuO_2	284	0.0004	35	48
$CeLi_2P_2$	827	0.0005	98	87
Br_2HLa	740	0.0006	98	87
BH_4Li	914	0.0009	98	87
$Cl_2O_2Ti_2$	10	0.0009	1	1
$AsCuLi_2$	740	0.001	98	87
CoH_2O_2	429	0.001	36	57
In_2Se_2	268	0.001	35	32
BrCdI	534	0.001	81	70
SSb_2Te_2	674	0.001	81	70
Ca_2N	523	0.001	70	81
I_2Pb	61	0.001	10	7
$MnNaTe_2$	604	0.0011	81	70
$Br_2Hf_2N_2$	766	0.0011	70	81
$As_2Cd_2K_2$	468	0.0011	60	38
Ag_2I_2	680	0.0012	103	67
Nd	212	0.0012	35	72
$PtSe_2$	233	0.0012	32	35
Ga_2Te_2	740	0.0012	98	87
As_2	442	0.0012	70	81
Ba_2Ni_3	827	0.0012	98	87
$C_2Br_2Y_2$	544	0.0012	55	54
As_2O_3	230	0.0013	35	18
ReS_2	349	0.0013	40	63
AlH_4Na	664	0.0013	88	52
CdClHO	268	0.0013	32	35
CCl_2Lu_2	685	0.0013	70	81
CKN	619	0.0014	106	65
O_4PSn	468	0.0014	60	38
$FeSe_2$	577	0.0014	67	103
GeI_2	653	0.0014	98	87
$I_2Pr_2S_2$	82	0.0014	10	7
HgI_2	613	0.0014	103	67
$Cl_2N_2Zr_2$	766	0.0014	70	81
Br_2Fe	523	0.0014	70	81
$PbTe_2$	534	0.0014	81	70
$MnSe_2$	689	0.0015	98	99
CaCl	590	0.0015	98	99
$AgClO_2$	608	0.0015	78	74
As_2Co_2	788	0.0015	99	98
Br_2Co	523	0.0015	70	81
$Cu_2Rb_2Te_2$	966	0.0015	126	77
Ca_2Cl_2	788	0.0015	99	98
$Ge_2Se_2Zr_2$	984	0.0015	99	98
Cl_2La_2	268	0.0015	35	32
I_2Zn	236	0.0015	35	32
Cl_2OV	76	0.0016	9	10
K_2PtSe_2	314	0.0016	56	18
$Cu_2K_2Te_2$	434	0.0016	56	35

Table showing the first 50 lattice matching combinations with the smallest strain needed to build the supercell.

$Dy_2O_2Br_2$ (P4/nmm)

Structural and electronic properties

**Formula**	$Dy_2O_2Br_2$
**Spacegroup**	P4/nmm
**Prototype**	PbClF
**Parent 3D**	$Br_2Dy_2O_2$
**Source DB**	MPDS
**DB ID**	S1903365
**DF2-C09 Binding energy [meV/ Å^2]**	15.77
**RVV10 Binding energy [meV/ Å^2]**	N/A
**Band gap (PBE) [eV]**	0.0

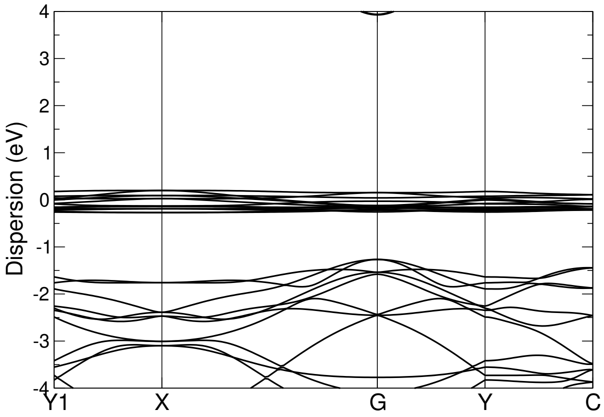

Band structure: Electronic band structure of $Dy_2O_2Br_2$ (P4/nmm) in a representative window around the Fermi energy and along a high-symmetry path.

Optimized crystal structure

Structural parameters: relaxed cell (top) and atomic positions (bottom) of $Dy_2O_2Br_2$ (P4/nmm) in Cartesian coordinates.

	x [Å]	y [Å]	z [Å]
$\mathbf{a_1}$	3.84648762	0.00022983	0.00000000
$\mathbf{a_2}$	0.00022983	3.84648762	0.00000000
$\mathbf{a_3}$	0.00000000	0.00000000	22.90384348

		x [Å]	y [Å]	z [Å]
●	Dy	0.96156980	-0.96156980	1.15289249
●	Br	-0.96156152	-2.88515594	2.81102854
●	Dy	-0.96179963	-2.88491783	-1.15289249
●	Br	0.96133169	-0.96133169	-2.81102854
•	O	-0.96168044	-0.96168044	0.00000000
•	O	0.96145060	-2.88480719	0.00000000

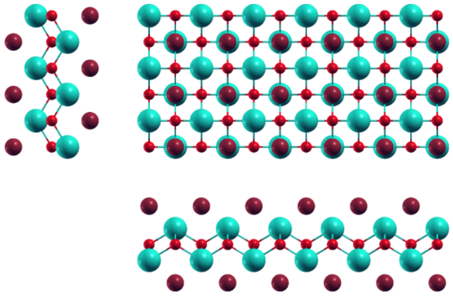

Orthographic projections: views of $Dy_2O_2Br_2$ (P4/nmm) as seen from the x axis (left), the y axis (bottom) and the z axis (center).

Lattice matching - minimal size

Formula	N° atoms	strain	cell size 1	cell size 2
K	7	0.3927	1	1
K	7	0.1933	1	1
In	7	0.1104	1	1
InSe	8	0.1524	1	1
Bi_2	8	0.1583	1	1
PbTe	8	0.1542	1	1
Sb_2	8	0.138	1	1
I_2Mg	9	0.1428	1	1
S_2V	9	0.1097	1	1
MoS_2	9	0.1096	1	1
CdI_2	9	0.1559	1	1
Nd	9	0.7838	1	3
Br_2Ca	9	0.1572	1	1
CaI_2	9	0.1816	1	1
I_2Pr	9	0.0074	1	1
Br_2La	9	0.1431	1	1
Br_2Cu	9	0.1091	1	1
Ca_2Si	9	0.777	1	1
I_2Yb	9	0.1785	1	1
BiClTe	9	0.1563	1	1
$AuTe_2$	9	0.1319	1	1
BrCdI	9	0.1456	1	1
$PdTe_2$	9	0.1301	1	1
HgI_2	9	1.1565	1	1
I_2Zn	9	0.1354	1	1
BaF_2	9	0.1487	1	1
BiBrTe	9	0.1621	1	1
S_2W	9	0.1096	1	1
GeI_2	9	0.1412	1	1
AsKSn	9	0.1474	1	1
$PbTe_2$	9	0.1448	1	1
I_2Nd	9	0.0084	1	1
Cl_2Cu	9	0.1003	1	1
I_2Tm	9	0.1801	1	1
$SnTe_2$	9	0.1393	1	1
Cl_2V	9	0.1088	1	1
GeI_2	9	0.1544	1	1
I_2Pb	9	0.7667	1	1
STl_2	9	0.1496	1	1
BiITe	9	0.1693	1	1
DyI_2	9	0.1851	1	1
Br_2Ni	9	0.1085	1	1
CeI_2	9	0.0066	1	1
Se_2Yb	9	0.1414	1	1
MoS_2	9	0.1095	1	1
Cl_2Mg	9	0.1086	1	1
$BiTe_2$	9	0.1417	1	1
GdI_2	9	0.1652	1	1
$CrSe_2$	9	0.109	1	1
$PtTe_2$	9	0.1316	1	1

Table showing the first 50 lattice matching combinations with the smallest number of atoms in the supercell.

Lattice matching - minimal strain

Formula	N° atoms	strain	cell size 1	cell size 2
I_3Sn	70	0.0	9	4
$Br_2O_2Y_2$	12	0.0	1	1
Ag_2I_2	438	0.0	49	36
$As_2Cd_2K_2$	876	0.0001	85	61
Br_2CsF	316	0.0001	36	25
Cu_2S_2	10	0.0001	1	1
$Cl_2S_2Tl_2$	930	0.0002	97	58
NS_2Zr	548	0.0002	48	65
Ga_2S_3	613	0.0002	48	65
Pb_2Se_2	806	0.0003	89	68
K	557	0.0003	82	65
Cu_2O_2	496	0.0004	50	49
La_2S_2	818	0.0004	89	71
F_2Tl_2	10	0.0004	1	1
Mg_6	366	0.0004	25	36
Na	353	0.0005	48	65
P_2Rh_2	10	0.0005	1	1
PtS_2	483	0.0006	48	65
$As_2Mg_2Na_2$	882	0.0006	82	65
Ho_2S_2	496	0.0006	50	49
O_2Sn_2	10	0.0007	1	1
H_4Ti	221	0.0008	16	25
P_4	356	0.0009	36	35
K	550	0.001	81	64
O_4PSn	876	0.0011	85	61
HgI_2	402	0.0011	49	36
In	445	0.0011	58	97
F_4Pb	629	0.0012	64	49
Se_2Sn_2	742	0.0012	81	64
Se_2Sn_2	732	0.0012	80	63
Bi_2Se_2	912	0.0013	98	81
$HfLiS_2$	548	0.0013	48	65
$As_2Mg_2Na_2$	870	0.0014	81	64
$C_2I_2La_2$	138	0.0014	12	11
H_2Li_2Pd	221	0.0014	16	25
Sn	655	0.0014	85	145
C_4Ca_2	774	0.0014	69	60
Se_2Ta_4	690	0.0015	50	65
AuI_4Li	792	0.0015	90	42
$Br_2O_2Tb_2$	12	0.0016	1	1
$Cu_2Rb_2Te_2$	366	0.0016	36	25
ClNZr	840	0.0016	81	118
$AsSn_2$	603	0.0016	62	77
C_2Li_2	246	0.0016	25	24
In	375	0.0016	49	81
$Br_2Cr_2S_2$	996	0.0016	84	82
Te_2Zn	483	0.0016	48	65
BrNZr	531	0.0016	52	73
$PtSe_2$	603	0.0017	62	77
Au_2Br_2	924	0.0017	100	81

Table showing the first 50 lattice matching combinations with the smallest strain needed to build the supercell.

$Dy_2S_2I_2$ (P-3m1)

Structural and electronic properties

**Formula**	$Dy_2S_2I_2$
**Spacegroup**	P-3m1
**Prototype**	SmSI
**Parent 3D**	$Dy_2I_2S_2$
**Source DB**	MPDS
**DB ID**	S1703875
**DF2-C09 Binding energy [meV/ Å^2]**	12.47
**RVV10 Binding energy [meV/ Å^2]**	N/A
**Band gap (PBE) [eV]**	0.0

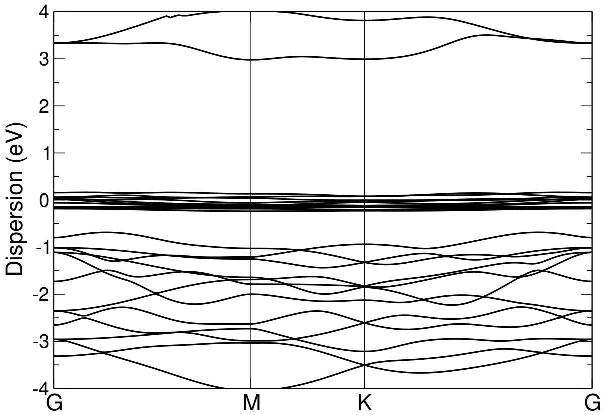

Band structure: Electronic band structure of $Dy_2S_2I_2$ (P-3m1) in a representative window around the Fermi energy and along a high-symmetry path.

Optimized crystal structure

Structural parameters: relaxed cell (top) and atomic positions (bottom) of $Dy_2S_2I_2$ (P-3m1) in Cartesian coordinates.

	x [Å]	y [Å]	z [Å]
$\mathbf{a_1}$	2.25412473	−3.90425855	0.00000000
$\mathbf{a_2}$	2.25412473	3.90425855	0.00000000
$\mathbf{a_3}$	0.00000000	0.00000000	26.85301539

		x [Å]	y [Å]	z [Å]
●	Dy	-1.12706236	0.65070976	-1.76139206
●	Dy	1.12706236	-0.65070976	1.76139206
●	S	-1.12706236	0.65070976	1.02666115
●	S	1.12706236	-0.65070976	-1.02666115
●	I	1.12706236	1.95212928	-3.73126782
●	I	1.12706236	1.95212928	3.73126782

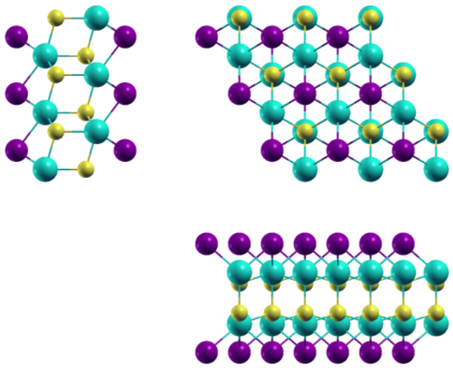

Orthographic projections: views of $Dy_2S_2I_2$ (P-3m1) as seen from the x axis (left), the y axis (bottom) and the z axis (center).

Lattice matching - minimal size

Formula	N° atoms	strain	cell size 1	cell size 2
K	7	0.1534	1	1
K	7	0.0071	1	1
S_2	8	1.584	1	1
Sb_2	8	0.2755	1	1
CaI_2	9	0.0021	1	1
$GeTe_2$	9	1.5576	1	1
$HfTe_2$	9	0.2533	1	1
I_2Pr	9	0.118	1	1
I_2Yb	9	0.0046	1	1
$AuTe_2$	9	0.264	1	1
Cl_2Zn	9	0.4287	1	1
$PdTe_2$	9	0.2606	1	1
I_2Zn	9	0.2706	1	1
Ba_2Hg	9	0.1397	1	1
Ba_2N	9	0.2554	1	1
Te_2Zr	9	0.254	1	1
I_2Nd	9	0.1184	1	1
Cl_2Cu	9	0.1968	1	1
I_2Tm	9	0.0033	1	1
DyI_2	9	0.0007	1	1
CeI_2	9	0.1176	1	1
I_2Ti	9	1.5345	1	1
$PtTe_2$	9	0.2635	1	1
Br_2Cd	9	0.2593	1	1
I_2La	9	0.121	1	1
F_2Zn	9	0.1161	1	1
Ba_2Cd	9	0.1419	1	1
Fe_2Te_2	10	0.1124	1	1
Li_2Tl_2	10	0.4711	1	1
Ca_2Cl_2	10	0.1126	1	1
Cu_2I_2	10	0.1315	1	1
Cl_2Gd_2	10	1.6005	1	1
Cu_2Te_2	10	0.1135	1	1
Ir_2P_2	10	0.1183	1	1
Ag_2Br_2	10	0.1213	1	1
Br_2Er_2	10	0.2648	1	1
Cu_2S_2	10	0.1146	1	1
Au_2Br_2	10	0.1393	1	1
Ge_2Te_2	10	0.1548	1	1
Br_2Tb_2	10	0.2614	1	1
Br_2Cu_2	10	0.1154	1	1
As_2Ir_2	10	0.1227	1	1
Cu_2Te_2	10	0.4346	1	1
O_2Pb_2	10	0.1291	1	1
Cl_2La_2	10	0.2673	1	1
Br_2Gd_2	10	0.2656	1	1
O_2Sn_2	10	0.1149	1	1
$LiMnSe_2$	10	0.2485	1	1
P_2Rh_2	10	0.1144	1	1
F_2Tl_2	10	0.1144	1	1

Table showing the first 50 lattice matching combinations with the smallest number of atoms in the supercell.

Lattice matching - minimal strain

Formula	N° atoms	strain	cell size 1	cell size 2
Br_2Cd	627	0.0	64	81
NS_2Ta	60	0.0	4	9
$F_2Se_2Yb_2$	600	0.0	43	57
Ba_2N	561	0.0001	57	73
CrI_2	363	0.0001	36	49
Br_2Cr	537	0.0001	49	81
Br_2Fe	171	0.0001	16	25
CBr_2Y_2	330	0.0001	25	36
CCl_2Lu_2	221	0.0001	16	25
Cl_2Sc_2	618	0.0001	49	81
Br_2Co	171	0.0002	16	25
$Br_2H_2Zr_2$	780	0.0002	49	81
Br_2PY_2	789	0.0002	64	81
P_2Sn_2	486	0.0002	43	57
NaO_4	629	0.0002	64	49
S_2Zn_2	486	0.0002	43	57
FeO_2	342	0.0002	25	64
$AuTe_2$	711	0.0002	73	91
Cl_2La_2	886	0.0002	81	100
$CrTe_2$	537	0.0002	49	81
Br_2Mg	363	0.0003	36	49
As_2	146	0.0003	16	25
Cu_3Se_3	780	0.0003	57	73
Nd	79	0.0003	9	25
Br_2Ti	537	0.0004	49	81
Br_2Er_2	802	0.0004	73	91
FeI_2	363	0.0004	36	49
S_2Sn_2	994	0.0004	99	100
$CrTe_2$	405	0.0004	37	61
$F_2Se_2Tm_2$	678	0.0004	49	64
$CrSe_2$	297	0.0004	25	49
CCl_2Gd_2	330	0.0004	25	36
Br_2Hg_3	566	0.0005	81	16
Cl_2Sc_2	466	0.0005	37	61
$LiMnSe_2$	550	0.0005	49	64
$Er_2I_2Se_2$	846	0.0006	81	60
Cl_2Hf_2	118	0.0006	9	16
CoO_2	342	0.0006	25	64
Br_2Zn	258	0.0006	25	36
$LiNbS_2$	118	0.0006	9	16
Ge_2Se_2	782	0.0006	77	80
$PtTe_2$	711	0.0006	73	91
S_2Ta	102	0.0006	9	16
Cl_2Y_2	294	0.0007	25	36
DyI_2	9	0.0007	1	1
In	394	0.0007	49	100
Br_2Cr	405	0.0007	37	61
Br_2Cu	552	0.0007	56	72
Cl_2Zn	171	0.0008	16	25
Ca_2N	171	0.0008	16	25

Table showing the first 50 lattice matching combinations with the smallest strain needed to build the supercell.

DyI_2 (P-3m1)

Structural and electronic properties

**Formula**	DyI_2
**Spacegroup**	P-3m1
**Prototype**	CdI2
**Parent 3D**	DyI_2
**Source DB**	MPDS
**DB ID**	S1707656
**DF2-C09 Binding energy [meV/ Å^2]**	10.21
**RVV10 Binding energy [meV/ Å^2]**	N/A
**Band gap (PBE) [eV]**	0.0

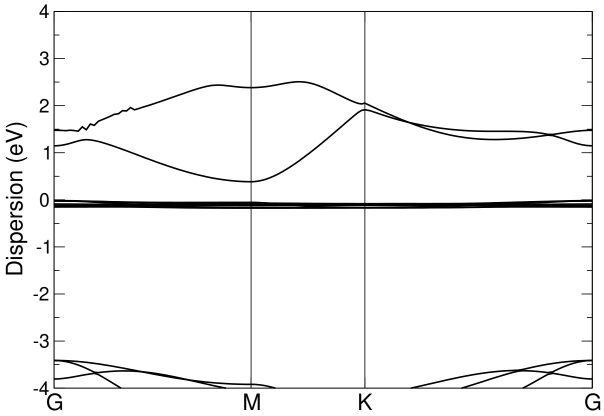

Band structure: Electronic band structure of DyI_2 (P-3m1) in a representative window around the Fermi energy and along a high-symmetry path.

Optimized crystal structure

Structural parameters: relaxed cell (top) and atomic positions (bottom) of DyI_2 (P-3m1) in Cartesian coordinates.

	x [Å]	y [Å]	z [Å]
$\mathbf{a_1}$	2.25738191	−3.90990016	0.00000000
$\mathbf{a_2}$	2.25738191	3.90990016	0.00000000
$\mathbf{a_3}$	0.00000000	0.00000000	19.28290362

	x [Å]	y [Å]	z [Å]
I	0.00000000	-1.30330005	1.80990000
Dy	2.25738191	0.00000000	0.00000000
I	0.00000000	1.30330005	-1.80990000

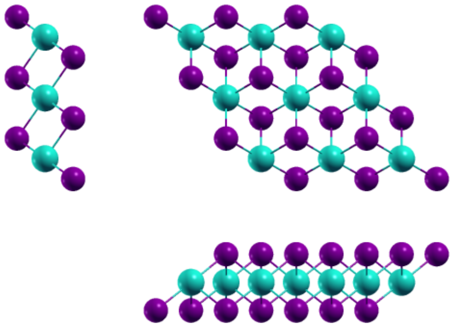

Orthographic projections: views of DyI_2 (P-3m1) as seen from the x axis (left), the y axis (bottom) and the z axis (center).

Lattice matching - minimal size

Formula	N° atoms	strain	cell size 1	cell size 2
K	4	0.1527	1	1
K	4	0.0064	1	1
Sb_2	5	0.2745	1	1
CaI_2	6	0.0028	1	1
$GeTe_2$	6	1.5527	1	1
I_2Pr	6	0.1176	1	1
I_2Yb	6	0.0053	1	1
$AuTe_2$	6	0.2631	1	1
Cl_2Zn	6	0.4272	1	1
$PdTe_2$	6	0.2597	1	1
I_2Zn	6	0.2697	1	1
Ba_2Hg	6	0.1391	1	1
Ba_2N	6	0.2545	1	1
I_2Nd	6	0.1181	1	1
Cl_2Cu	6	0.1961	1	1
I_2Tm	6	0.004	1	1
CeI_2	6	0.1172	1	1
I_2Ti	6	1.5296	1	1
$PtTe_2$	6	0.2625	1	1
Br_2Cd	6	0.2584	1	1
I_2La	6	0.1206	1	1
F_2Zn	6	0.1157	1	1
Ba_2Cd	6	0.1413	1	1
Fe_2Te_2	7	0.1122	1	1
Li_2Tl_2	7	0.4694	1	1
Ca_2Cl_2	7	0.1123	1	1
Cu_2I_2	7	0.131	1	1
Cl_2Gd_2	7	1.5954	1	1
Cu_2Te_2	7	0.1133	1	1
Ir_2P_2	7	0.118	1	1
Ag_2Br_2	7	0.1209	1	1
Br_2Er_2	7	0.2639	1	1
O_2Sn_2	7	0.1164	1	1
Cu_2S_2	7	0.1144	1	1
Au_2Br_2	7	0.1387	1	1
Ge_2Te_2	7	0.1542	1	1
Br_2Tb_2	7	0.2605	1	1
Br_2Cu_2	7	0.1151	1	1
As_2Ir_2	7	0.1223	1	1
Cu_2Te_2	7	0.4331	1	1
O_2Pb_2	7	0.1287	1	1
Cl_2La_2	7	0.2663	1	1
Br_2Gd_2	7	0.2646	1	1
O_2Sn_2	7	0.1146	1	1
$LiMnSe_2$	7	0.2476	1	1
P_2Rh_2	7	0.1141	1	1
F_2Tl_2	7	0.1141	1	1
Br_2Ho_2	7	0.2612	1	1
O_2Sn_2	7	0.1348	1	1
Ag_2Te_2	7	0.1133	1	1

Table showing the first 50 lattice matching combinations with the smallest number of atoms in the supercell.

Lattice matching - minimal strain

Formula	N° atoms	strain	cell size 1	cell size 2
In	247	0.0	49	100
Br_2Zn	183	0.0001	25	36
Ca_2N	123	0.0001	16	25
CoO_2	267	0.0001	25	64
Cl_2Hf_2	91	0.0001	9	16
Te_2Ti	183	0.0002	25	36
BN	237	0.0002	25	81
$HfSe_2$	183	0.0002	25	36
Br_2Gd_2	583	0.0002	73	91
Br_3Cs	43	0.0002	9	4
CCl_2Gd_2	255	0.0002	25	36
$PdTe_2$	435	0.0003	64	81
$CrSe_2$	222	0.0003	25	49
FeI_2	255	0.0003	36	49
In_2Se_2	643	0.0003	81	100
As_2Sn_2	583	0.0003	73	91
$AgNO_3$	437	0.0003	64	49
Br_2Er_2	583	0.0003	73	91
Nd	52	0.0003	9	25
As_2	98	0.0004	16	25
Cl_2Gd_2	357	0.0004	43	57
NiO_2	267	0.0004	25	64
Br_2Mg	255	0.0004	36	49
$I_2S_2Tb_2$	9	0.0004	1	1
$CrTe_2$	390	0.0005	49	81
S_2Zn_2	357	0.0005	43	57
NaO_4	437	0.0005	64	49
Cl_2Sc_2	471	0.0005	49	81
CCl_2Lu_2	173	0.0005	16	25
H_2MgO_2	512	0.0006	39	79
Br_2Cd	435	0.0007	64	81
CrI_2	255	0.0007	36	49
$Dy_2I_2S_2$	9	0.0007	1	1
S_2Sn_2	697	0.0007	99	100
NS_2Ta	48	0.0007	4	9
$F_2Se_2Yb_2$	471	0.0007	43	57
$Er_2I_2Se_2$	603	0.0007	81	60
$Br_2Hf_2N_2$	198	0.0007	16	25
Ba_2N	390	0.0007	57	73
Br_2Cr	390	0.0007	49	81
Br_2Fe	123	0.0008	16	25
$GeTe_2$	255	0.0008	36	49
CBr_2Y_2	255	0.0008	25	36
H_4Ti	865	0.0008	70	131
Te_4TiZr	510	0.0008	72	49
Br_2Co	123	0.0008	16	25
Br_2Tb_2	516	0.0008	64	81
$Br_2H_2Zr_2$	633	0.0008	49	81
Br_2PY_2	597	0.0009	64	81
P_2Sn_2	357	0.0009	43	57

Table showing the first 50 lattice matching combinations with the smallest strain needed to build the supercell.

DySBr (Pmmn)

Structural and electronic properties

**Formula**	DySBr
**Spacegroup**	Pmmn
**Prototype**	FeOCl
**Parent 3D**	$Dy_2S_2Br_2$
**Source DB**	ICSD
**DB ID**	79106
**DF2-C09 Binding energy [meV/ Å^2]**	10.42
**RVV10 Binding energy [meV/ Å^2]**	N/A
**Band gap (PBE) [eV]**	N/A

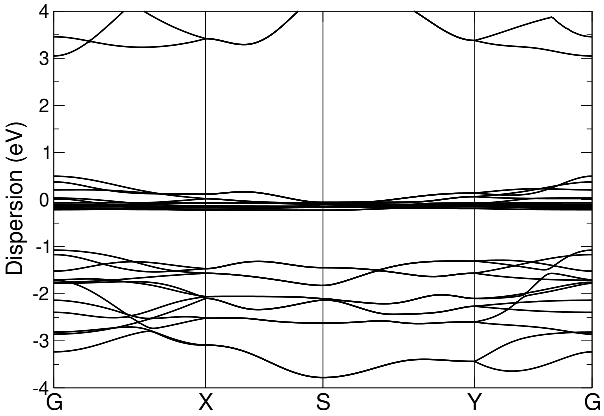

Band structure: Electronic band structure of DySBr (Pmmn) in a representative window around the Fermi energy and along a high-symmetry path.

Optimized crystal structure

Structural parameters: relaxed cell (top) and atomic positions (bottom) of DySBr (Pmmn) in Cartesian coordinates.

	x [Å]	y [Å]	z [Å]
$\mathbf{a_1}$	4.10354135	0.00000000	0.00000000
$\mathbf{a_2}$	0.00000000	5.30865565	0.00000000
$\mathbf{a_3}$	0.00000000	0.00000000	26.12509565

	x [Å]	y [Å]	z [Å]
Dy	2.05177068	2.65432782	11.88264205
S	2.05177068	0.00000000	12.41807628
Br	0.00000000	2.65432782	9.93469243
Dy	0.00000000	0.00000000	14.24244111
S	0.00000000	2.65432782	13.70702856
Br	2.05177068	0.00000000	16.19041957

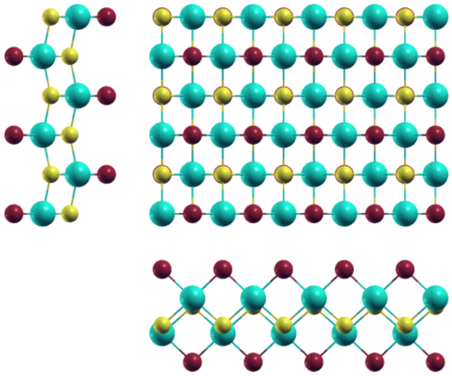

Orthographic projections: views of DySBr (Pmmn) as seen from the x axis (left), the y axis (bottom) and the z axis (center).

Lattice matching - minimal size

Formula	N° atoms	strain	cell size 1	cell size 2
Tl	8	0.3799	1	2
In	8	0.1453	1	2
Gd	8	0.1317	1	2
HgI_2	9	0.3199	1	1
Ba_2Hg	9	0.2686	1	1
CNRb	9	0.3108	1	1
CKN	9	0.0123	1	1
CNNa	9	0.3023	1	1
Ba_2Cd	9	0.2724	1	1
Bi_2In_2	10	0.0607	1	1
Cu_2I_2	10	0.2541	1	1
O_2Pb_2	10	0.2497	1	1
As_4	10	0.3081	1	1
S_2	10	2.0543	1	2
Au_2Se_2	10	0.5667	1	1
LiO	10	0.3545	1	2
O_2Sn_2	10	0.1673	1	1
Bi_2O_2	10	0.7501	1	1
Ag_2I_2	10	0.3217	1	1
Br_2CsF	10	0.06	1	1
Gd	10	0.0429	1	4
Sn_2Te_2	10	0.3367	1	1
F_4Sn	11	0.2674	1	1
FKO_2Se	11	0.563	1	1
F_4Nb	11	0.2592	1	1
Cl_4Mn	11	0.0628	1	1
$Ba_2H_2I_2$	12	0.0595	1	1
$Br_2Ho_2S_2$	12	0.0022	1	1
S_2V	12	0.1474	1	2
MoS_2	12	0.1479	1	2
$I_2Lu_2Se_2$	12	0.053	1	1
$MoSe_2$	12	0.3784	1	2
$Br_2F_2Sr_2$	12	0.2568	1	1
$Ho_2I_2S_2$	12	0.0074	1	1
Cu_4Te_2	12	0.1163	1	1
$Eu_2F_2I_2$	12	0.7482	1	1
AlH_4Na	12	0.064	1	1
$GeTe_2$	12	0.6567	1	2
$F_2I_2Sm_2$	12	0.2494	1	1
$Cl_2H_2Zr_2$	12	0.2654	1	1
ReS_2	12	0.1402	1	2
Br_2Ca_3Si	12	0.2689	1	1
$Br_2S_2Y_2$	12	0.0053	1	1
S_2W	12	0.1479	1	2
$Gd_2I_2S_2$	12	0.0127	1	1
$C_2Br_2Gd_2$	12	0.1079	1	1
N_2W	12	0.1341	1	2
$Br_2F_2Pb_2$	12	0.2564	1	1
$Br_2Er_2Se_2$	12	0.0178	1	1
Se_2V	12	0.3816	1	2

Table showing the first 50 lattice matching combinations with the smallest number of atoms in the supercell.

Lattice matching - minimal strain

Formula	N° atoms	strain	cell size 1	cell size 2
As_4	792	0.0001	72	90
F_2Ni	855	0.0007	79	127
Cl_2Y_2	788	0.0007	60	107
Cu_2Se_2	982	0.0008	79	127
$Br_2In_2O_2$	822	0.0009	56	81
Br_2Zn	681	0.0009	60	107
$AgTe_2$	261	0.0009	21	45
Cl_2ORu	770	0.0009	61	101
Fe_2Se_2	982	0.0009	79	127
O_4PSn	738	0.0009	60	63
$I_2Nd_2S_2$	354	0.001	27	32
$CrTe_2$	291	0.001	24	49
$BrKO_3$	821	0.001	96	49
Cl_2Sc_2	340	0.001	24	49
$Cu_2Rb_2Te_2$	750	0.0011	62	63
CdO_2	624	0.0011	50	108
Br_2Cr	291	0.0011	24	49
Te_2Ti	681	0.0011	60	107
FKO_2Se	983	0.0011	98	79
$HfSe_2$	681	0.0012	60	107
$Br_2H_2Zr_2$	438	0.0012	24	49
Bi_2In_2	624	0.0012	62	63
$F_2Lu_2Se_2$	60	0.0012	4	6
$Ag_2K_2Te_2$	738	0.0013	63	60
Br_2La_2	48	0.0013	4	6
Br_2Ti	291	0.0013	24	49
Na	384	0.0013	48	96
Cl_2Zn	729	0.0013	68	107
Ga_2Se_2	788	0.0013	60	107
In	313	0.0013	37	91
$Cu_2K_2Te_2$	738	0.0014	60	63
O_4PTl	804	0.0014	65	69
Sb_2	36	0.0014	4	6
HgO	416	0.0014	41	85
PSn_2	624	0.0014	54	100
NS_2Zr	672	0.0014	48	96
$Cl_2Hg_2N_2$	210	0.0015	20	15
Hf_3Te_2	878	0.0015	63	100
Cl_2Y_2	750	0.0015	57	102
$As_2Cd_2K_2$	738	0.0015	60	63
Ga_2Se_2	750	0.0015	57	102
$Ca_2Cl_2H_2$	978	0.0015	63	100
IKO_3	816	0.0015	96	48
$C_2Br_2La_2$	756	0.0015	52	74
AlH_4Na	750	0.0015	63	62
$I_2N_2Ti_2$	276	0.0016	18	28
CrS_2	42	0.0016	3	8
$AuCrTe_4$	396	0.0016	36	30
$Ba_2F_2I_2$	804	0.0016	65	69
Fe_2Se_2	958	0.0016	77	124

Table showing the first 50 lattice matching combinations with the smallest strain needed to build the supercell.

DySI (Pmmn)

Structural and electronic properties

**Formula**	DySI
**Spacegroup**	Pmmn
**Prototype**	FeOCl
**Parent 3D**	$Dy_2S_2I_2$
**Source DB**	ICSD
**DB ID**	79107
**DF2-C09 Binding energy [meV/ Å^2]**	10.58
**RVV10 Binding energy [meV/ Å^2]**	N/A
**Band gap (PBE) [eV]**	N/A

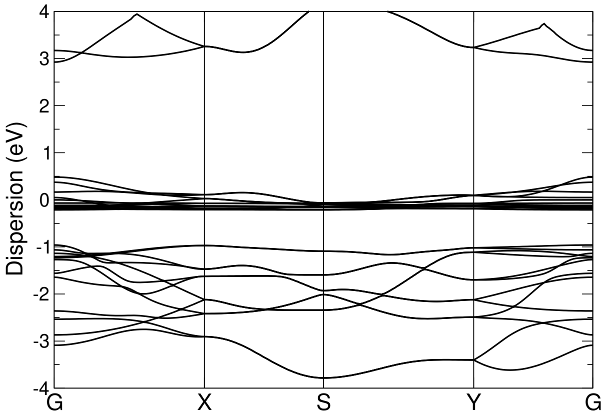

Band structure: Electronic band structure of DySI (Pmmn) in a representative window around the Fermi energy and along a high-symmetry path.

Optimized crystal structure

Structural parameters: relaxed cell (top) and atomic positions (bottom) of DySI (Pmmn) in Cartesian coordinates.

	x [Å]	y [Å]	z [Å]
$\mathbf{a_1}$	4.21294032	0.00000000	0.00000000
$\mathbf{a_2}$	0.00000000	5.31803808	0.00000000
$\mathbf{a_3}$	0.00000000	0.00000000	26.57681467

		x [Å]	y [Å]	z [Å]
●	Dy	0.00000000	0.00000000	14.42184091
●	Dy	2.10647016	2.65901904	12.15495128
●	S	2.10647016	0.00000000	12.67360051
●	S	0.00000000	2.65901904	13.90325768
●	I	2.10647016	0.00000000	16.64362541
●	I	0.00000000	2.65901904	9.93307812

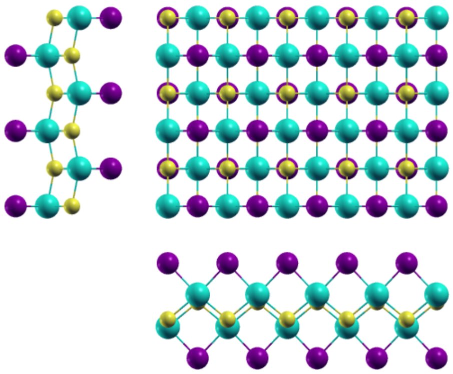

Orthographic projections: views of DySI (Pmmn) as seen from the x axis (left), the y axis (bottom) and the z axis (center).

Lattice matching - minimal size

Formula	N° atoms	strain	cell size 1	cell size 2
Tl	8	0.3754	1	2
Br_2Zn	9	0.1167	1	1
$SiTe_2$	9	0.1158	1	1
HgI_2	9	0.3014	1	1
Te_2Ti	9	0.1166	1	1
Ba_2Hg	9	0.2533	1	1
CNRb	9	0.2152	1	1
CKN	9	0.0121	1	1
$NiTe_2$	9	0.116	1	1
Se_2Zr	9	0.1158	1	1
CNNa	9	0.2867	1	1
Ba_2Cd	9	0.2568	1	1
$HfSe_2$	9	0.1166	1	1
$AgCuTe_2$	10	1.7842	1	1
Au_2Br_2	10	0.0599	1	1
Cl_2Y_2	10	0.117	1	1
O_2Pb_2	10	0.7041	1	1
As_4	10	0.292	1	1
Br_3Cs	10	0.3728	1	1
S_2	10	1.9532	1	2
Au_2Se_2	10	0.5403	1	1
CaClHO	10	0.1161	1	1
$AgClO_2$	10	0.1169	1	1
$SbSe_2Tl$	10	0.1751	1	1
Ag_2I_2	10	0.3031	1	1
Br_2CsF	10	0.3261	1	1
Gd	10	0.0322	1	4
Sn_2Te_2	10	0.3173	1	1
F_4Sn	11	0.2522	1	1
Hg_3N_2	11	0.4023	1	1
FKO_2Se	11	0.5351	1	1
$ClKO_3$	11	0.3806	1	1
$CuGeO_3$	11	0.1397	1	1
Cl_4Mn	11	0.0544	1	1
$Ba_2H_2I_2$	12	0.3226	1	1
CrS_2	12	0.1374	1	2
$Br_2Ho_2S_2$	12	0.0105	1	1
$I_2Lu_2Se_2$	12	0.5218	1	1
$Ho_2I_2S_2$	12	0.0016	1	1
AlH_4Na	12	0.0552	1	1
$GeTe_2$	12	1.921	1	2
$F_2I_2Sm_2$	12	0.2355	1	1
ReS_2	12	0.1404	1	2
Br_2Ca_3Si	12	0.2536	1	1
$Br_2S_2Y_2$	12	0.0131	1	1
$Gd_2I_2S_2$	12	0.0036	1	1
$C_2Br_2Gd_2$	12	0.1079	1	1
N_2W	12	0.1345	1	2
Cl_2Fe	12	0.3998	1	2
$I_2S_2Tb_2$	12	0.0014	1	1

Table showing the first 50 lattice matching combinations with the smallest number of atoms in the supercell.

Lattice matching - minimal strain

Formula	N° atoms	strain	cell size 1	cell size 2
$I_2N_2Zr_2$	822	0.0003	56	81
Sn	300	0.0005	35	90
$Cl_2Fe_2O_2$	366	0.0006	21	40
$Br_2N_2Zr_2$	630	0.0006	42	63
$Br_2O_2Yb_2$	690	0.0006	45	70
$Br_2H_2Zr_2$	744	0.0006	40	84
PTe_2Zr_2	740	0.0007	50	88
CrS_2	114	0.0008	8	22
N_3Na	420	0.0009	40	45
As_4	624	0.0009	56	72
Cl_2OOs	532	0.0009	42	70
Cu_2Te_2	672	0.0009	54	87
Fe_2Te_2	550	0.001	45	70
$Br_2O_2Tm_2$	732	0.001	48	74
PSn_2	492	0.001	42	80
Bi_2In_2	772	0.0011	76	79
Ge_2Te_2	54	0.0011	5	6
$SbSe_2Tl$	974	0.0011	97	98
As_2Ru_2	584	0.0011	48	74
$As_2Co_2Li_2$	846	0.0012	54	87
$Si_2Te_2Zr_2$	972	0.0012	61	101
Fe_2Se_2	770	0.0012	61	101
F_2Ni	669	0.0012	61	101
Ag_2Te_2	814	0.0012	67	103
Ca_2Cl_2	584	0.0013	48	74
$K_2O_2Tl_2$	738	0.0013	63	60
AsSb	476	0.0013	50	88
$I_2S_2Tl_2$	972	0.0013	61	101
S_2Sn	705	0.0013	60	115
$Cu_2Rb_2Te_2$	930	0.0014	76	79
Ca_2Cl_2	550	0.0014	45	70
S_2Zr	705	0.0014	60	115
Se_2Ta	519	0.0014	42	89
$I_2S_2Tb_2$	12	0.0014	1	1
In	469	0.0014	55	139
Se_4TiZr	492	0.0014	42	40
$NbTe_2$	705	0.0014	60	115
Cu_2Se_2	770	0.0015	61	101
$Cl_2H_2Sc_2$	786	0.0015	42	89
S_2Sn	492	0.0015	42	80
As_2Ru_2	550	0.0016	45	70
P_4	876	0.0016	74	108
$Br_2Er_2O_2$	732	0.0016	48	74
CdClHO	572	0.0016	42	80
$C_2Br_2La_2$	618	0.0016	42	61
$Hf_2I_2N_2$	732	0.0016	42	80
$Ho_2I_2S_2$	12	0.0016	1	1
$Ge_2Hf_2Te_2$	876	0.0016	56	90
$Ge_2Hf_2Te_2$	846	0.0016	54	87
CoI_2	564	0.0017	50	88

Table showing the first 50 lattice matching combinations with the smallest strain needed to build the supercell.

Er_2Br_2 (P-3m1)

Structural and electronic properties

**Formula**	Er_2Br_2
**Spacegroup**	P-3m1
**Prototype**	PtTe
**Parent 3D**	Br_2Er_2
**Source DB**	MPDS
**DB ID**	S542062
**DF2-C09 Binding energy [meV/ Å^2]**	11.66
**RVV10 Binding energy [meV/ Å^2]**	N/A
**Band gap (PBE) [eV]**	0.0

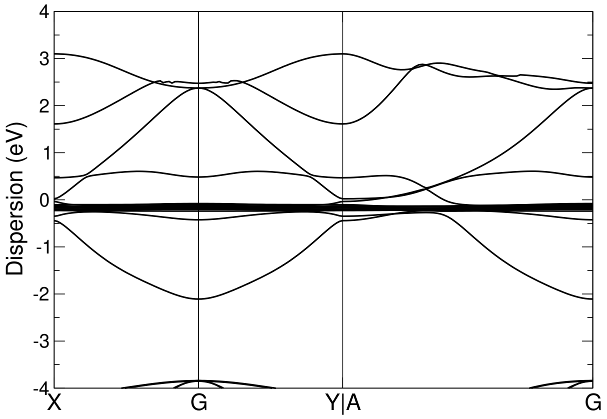

Band structure: Electronic band structure of Er_2Br_2 (P-3m1) in a representative window around the Fermi energy and along a high-symmetry path.

Optimized crystal structure

Structural parameters: relaxed cell (top) and atomic positions (bottom) of Er_2Br_2 (P-3m1) in Cartesian coordinates.

		x [Å]	y [Å]	z [Å]
$\mathbf{a_1}$		2.02050064	−3.49953016	0.00000000
$\mathbf{a_2}$		2.02043170	3.49949036	0.00000000
$\mathbf{a_3}$		0.00000000	0.00000000	25.22346404
		x [Å]	y [Å]	z [Å]
●	Er	1.01023311	-0.58325836	1.54233616
●	Br	1.01014045	1.74978871	3.30882095
●	Er	-1.01023311	0.58325836	-1.54233616
●	Br	1.01029125	1.74970164	-3.30882095

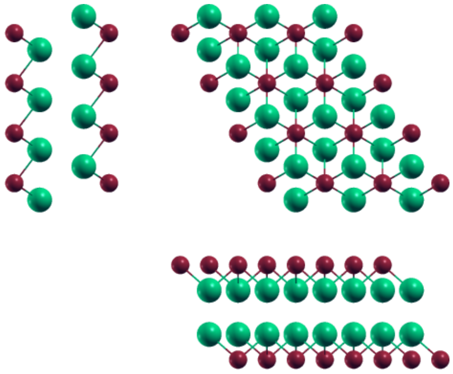

Orthographic projections: views of Er_2Br_2 (P-3m1) as seen from the x axis (left), the y axis (bottom) and the z axis (center).

Lattice matching - minimal size

Formula	N° atoms	strain	cell size 1	cell size 2
K	5	0.484	1	1
Ag_2	6	0.4984	1	1
As_2	6	0.2624	1	1
Sb_2	6	0.0074	1	1
CaCl	6	0.1255	1	1
Cl_2Zn	7	0.2609	1	1
$MoTe_2$	7	0.2545	1	1
PSn_2	7	0.2753	1	1
Ba_2Pt	7	0.4978	1	1
HfS_2	7	0.2662	1	1
CaI_2	7	0.4606	1	1
$HfTe_2$	7	0.0082	1	1
Te_2V	7	0.2571	1	1
$CuTe_2$	7	0.2657	1	1
S_2Zr	7	0.2741	1	1
I_2Yb	7	0.4543	1	1
Br_2Co	7	0.2618	1	1
Ca_2N	7	0.2631	1	1
$AuTe_2$	7	0.0006	1	1
Cl_2Zn	7	0.1358	1	1
$PdTe_2$	7	0.003	1	1
I_2Zn	7	0.0041	1	1
Te_2Zn	7	0.2543	1	1
Bi_2Pd	7	0.4238	1	1
Br_2Mn	7	0.2592	1	1
$CoTe_2$	7	0.2667	1	1
CdClO	7	0.2565	1	1
Ba_2N	7	0.0067	1	1
Se_2Ti	7	0.2493	1	1
Te_2Zr	7	0.0077	1	1
Te_2W	7	0.2547	1	1
I_2Tm	7	0.4577	1	1
S_2Sn	7	0.2745	1	1
$SnTe_2$	7	0.009	1	1
OTl_2	7	0.2567	1	1
Br_2Fe	7	0.2619	1	1
GeS_2	7	0.1185	1	1
$MnSe_2$	7	0.1254	1	1
DyI_2	7	0.4677	1	1
Br_2Ni	7	0.2691	1	1
CuO_2	7	0.0915	1	1
$NbTe_2$	7	0.2738	1	1
Cl_2Mg	7	0.2692	1	1
F_2Ni	7	0.1327	1	1
$PtTe_2$	7	0.001	1	1
Br_2Cd	7	0.0039	1	1
F_2Zn	7	0.1538	1	1
NaPSn	7	0.009	1	1
Fe_2Te_2	8	0.1455	1	1
Ca_2Cl_2	8	0.1459	1	1

Table showing the first 50 lattice matching combinations with the smallest number of atoms in the supercell.

Lattice matching - minimal strain

Formula	N° atoms	strain	cell size 1	cell size 2
Li_2Tl_2	164	0.0	25	16
Br_2Mn	499	0.0	64	81
$I_2S_2Sm_2$	634	0.0	73	57
Se_2Ti	388	0.0001	49	64
Cl_2Ni	291	0.0001	36	49
CdH_2O_2	593	0.0001	57	73
Br_2Ca_3Si	324	0.0001	45	24
LiO	114	0.0001	16	25
$CrTe_2$	343	0.0002	43	57
CaI_2	643	0.0002	100	81
$Br_2Hf_2N_2$	838	0.0002	73	91
$F_2Se_2Y_2$	294	0.0003	36	25
$Br_2Hf_2N_2$	742	0.0003	76	73
$Cl_2N_2Zr_2$	838	0.0003	73	91
K	349	0.0003	73	57
Cl_2Sc_2	400	0.0003	43	57
DyI_2	583	0.0003	91	73
Br_2Zr_2	452	0.0003	49	64
Cl_2NSc_2	280	0.0003	25	36
$NbSe_2$	291	0.0003	36	49
KS_2Ti	580	0.0003	64	81
$Dy_2I_2S_2$	802	0.0004	91	73
Se_2Ta	291	0.0004	36	49
O_2Pt	331	0.0004	37	61
Ga_2S_2	724	0.0005	81	100
Br_2Cr	343	0.0005	43	57
Br_2Gd_2	8	0.0005	1	1
Te_2W	447	0.0005	57	73
CaH_2O_2	747	0.0005	73	91
FeH_2O_2	116	0.0005	9	16
$AuTe_2$	7	0.0006	1	1
Ga_2S_2	724	0.0006	81	100
$CoTe_2$	624	0.0006	81	100
$Br_2H_2Zr_2$	514	0.0006	43	57
Sb_2Te_2	400	0.0006	57	43
As_2Sn_2	8	0.0006	1	1
Br_2Ca_3Si	708	0.0006	81	64
I_2La_2Sb	577	0.0007	73	57
I_2La_2Te	644	0.0007	81	64
$MoTe_2$	447	0.0007	57	73
$FeSe_2$	584	0.0007	65	108
CdClO	447	0.0007	57	73
NiO_2	496	0.0007	49	100
$I_2S_2Tb_2$	802	0.0007	91	73
Cu_2O_2	480	0.0008	62	58
I_2Pb	357	0.0008	57	43
Cl_2Mn	208	0.0008	25	36
Br_2Ti	343	0.0008	43	57
NbS_2	208	0.0008	25	36
Br_2Ca_3Si	708	0.0008	81	64

Table showing the first 50 lattice matching combinations with the smallest strain needed to build the supercell.

$Er_2O_2Br_2$ (P4/nmm)

Structural and electronic properties

**Formula**	$Er_2O_2Br_2$
**Spacegroup**	P4/nmm
**Prototype**	PbClF
**Parent 3D**	$Br_2Er_2O_2$
**Source DB**	MPDS
**DB ID**	S1903357
**DF2-C09 Binding energy [meV/ Å^2]**	15.32
**RVV10 Binding energy [meV/ Å^2]**	N/A
**Band gap (PBE) [eV]**	0.0

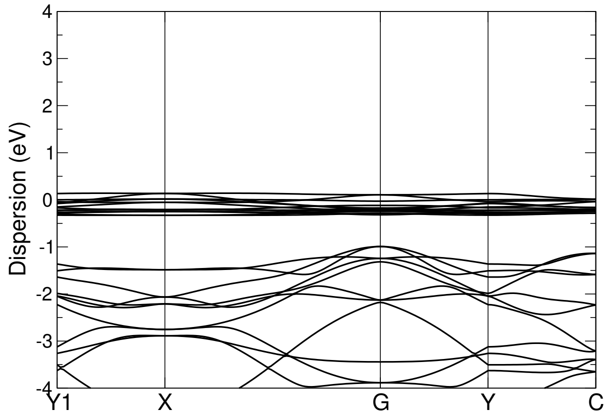

Band structure: Electronic band structure of $Er_2O_2Br_2$ (P4/nmm) in a representative window around the Fermi energy and along a high-symmetry path.

Optimized crystal structure

Structural parameters: relaxed cell (top) and atomic positions (bottom) of $Er_2O_2Br_2$ (P4/nmm) in Cartesian coordinates.

	x [Å]	y [Å]	z [Å]
$\mathbf{a_1}$	3.82241291	0.00032278	0.00000000
$\mathbf{a_2}$	0.00032278	3.82241291	0.00000000
$\mathbf{a_3}$	0.00000000	0.00000000	22.94455877

		x [Å]	y [Å]	z [Å]
●	Er	0.95548552	-0.95548552	1.14875081
●	Br	-0.95566008	-2.86707561	2.81215075
●	Er	-0.95580830	-2.86692739	-1.14875081
●	Br	0.95533730	-0.95533730	-2.81215075
•	O	-0.95569472	-0.95569472	0.00000000
•	O	0.95537194	-2.86671819	0.00000000

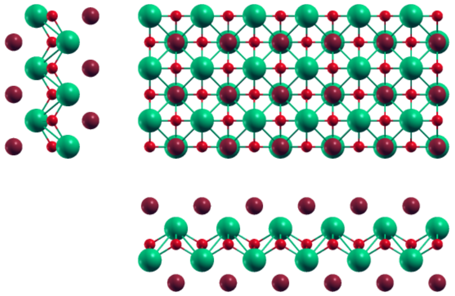

Orthographic projections: views of $Er_2O_2Br_2$ (P4/nmm) as seen from the x axis (left), the y axis (bottom) and the z axis (center).

Lattice matching - minimal size

Formula	N° atoms	strain	cell size 1	cell size 2
K	7	0.3992	1	1
K	7	0.1973	1	1
In	7	0.1098	1	1
InSe	8	0.1554	1	1
Bi_2	8	0.1614	1	1
Ag_2	8	0.772	1	1
PbTe	8	0.1572	1	1
Sb_2	8	0.1405	1	1
I_2Mg	9	0.1455	1	1
S_2V	9	0.1091	1	1
MoS_2	9	0.109	1	1
CdI_2	9	0.1589	1	1
Ba_2Pt	9	0.771	1	1
Br_2Ca	9	0.1602	1	1
HfS_2	9	0.1088	1	1
CaI_2	9	0.1853	1	1
Br_2La	9	0.1458	1	1
Br_2Cu	9	0.1107	1	1
Ca_2Si	9	0.7886	1	1
I_2Yb	9	0.1821	1	1
BiClTe	9	0.1593	1	1
$AuTe_2$	9	0.1341	1	1
BrCdI	9	0.1484	1	1
$PdTe_2$	9	0.1323	1	1
HgI_2	9	1.1729	1	1
I_2Zn	9	0.1378	1	1
BaF_2	9	0.1516	1	1
BiBrTe	9	0.1653	1	1
S_2W	9	0.109	1	1
GeI_2	9	0.1438	1	1
$CoTe_2$	9	0.1089	1	1
Ba_2N	9	0.1295	1	1
AsKSn	9	0.1502	1	1
$PbTe_2$	9	0.1475	1	1
Cl_2Cu	9	0.1012	1	1
I_2Tm	9	0.1838	1	1
$SnTe_2$	9	0.1418	1	1
GeI_2	9	0.1574	1	1
I_2Pb	9	0.7781	1	1
STl_2	9	0.1525	1	1
BiITe	9	0.1727	1	1
DyI_2	9	0.1889	1	1
CeI_2	9	0.0096	1	1
Se_2Yb	9	0.144	1	1
MoS_2	9	0.1089	1	1
$BiTe_2$	9	0.1443	1	1
GdI_2	9	0.1685	1	1
$PtTe_2$	9	0.1338	1	1
Br_2Cd	9	0.1316	1	1
O_2Pt	9	0.1101	1	1

Table showing the first 50 lattice matching combinations with the smallest number of atoms in the supercell.

Lattice matching - minimal strain

Formula	N° atoms	strain	cell size 1	cell size 2
$H_2I_2Sr_2$	882	0.0001	82	65
$AgTe_2$	258	0.0003	25	36
Mg_2	146	0.0003	16	25
Sn	445	0.0003	58	97
$Ag_2K_2Se_2$	510	0.0004	49	36
Pb_2Se_2	874	0.0004	97	73
Bi_2Pd	849	0.0005	85	113
Br_2Zr_2	548	0.0005	48	65
Ge_2Te_2	712	0.0007	78	61
Cl_2Fe	840	0.0007	81	118
$Ag_2K_2Te_2$	246	0.0007	25	16
C_4Ca_2	906	0.0007	81	70
Cl_4Cu_2	738	0.0008	89	34
Ca_2O_2	962	0.0008	85	113
$AgNO_2$	602	0.0008	63	56
$Br_2Ca_2H_2$	12	0.0008	1	1
$H_2I_2Sr_2$	870	0.0009	81	64
Cl_2Zr	840	0.0009	81	118
Se_2Ti	483	0.0009	48	65
$Cu_2Na_2Te_2$	690	0.0009	65	50
$Ba_2H_2I_2$	366	0.001	36	25
$CuGeO_3$	735	0.001	70	63
O_4PTl	876	0.001	85	61
$Ba_2F_2I_2$	876	0.001	85	61
$Ca_2Mn_2Si_2$	12	0.0011	1	1
HgO	536	0.0011	61	85
Cl_2Co	840	0.0011	81	118
$C_2I_2Y_2$	810	0.0012	67	68
$Gd_2I_2S_2$	690	0.0012	70	45
$Br_2Ho_2O_2$	12	0.0012	1	1
In	375	0.0013	49	81
CNRb	609	0.0013	77	49
K	983	0.0013	145	113
C_2Li_2	246	0.0014	25	24
S_2Ti	840	0.0014	81	118
O_2Pt	711	0.0015	64	109
$I_2S_2Tb_2$	732	0.0015	74	48
Se_2Ta	531	0.0015	52	73
$NbSe_2$	531	0.0015	52	73
Bi_2Se_2	602	0.0016	65	53
Au_2Br_2	914	0.0016	99	80
Cl_2Ni	531	0.0016	52	73
H_2Li_2Pd	221	0.0016	16	25
$As_2Mg_2Na_2$	870	0.0016	81	64
Cd_2I_3	980	0.0016	95	82
Cd_2I_3	919	0.0016	89	77
$Dy_2I_2S_2$	732	0.0016	74	48
AsI_2La_2	980	0.0016	95	82
$Br_2O_2Tm_2$	12	0.0017	1	1
$Cl_2Hg_2N_2$	810	0.0017	90	45

Table showing the first 50 lattice matching combinations with the smallest strain needed to build the supercell.

$Er_2O_2Cl_2$ (P-3m1)

Structural and electronic properties

**Formula**	$Er_2O_2Cl_2$
**Spacegroup**	P-3m1
**Prototype**	SmSI
**Parent 3D**	$Cl_2Er_2O_2$
**Source DB**	MPDS
**DB ID**	S1936405
**DF2-C09 Binding energy [meV/ Å^2]**	11.11
**RVV10 Binding energy [meV/ Å^2]**	N/A
**Band gap (PBE) [eV]**	0.0

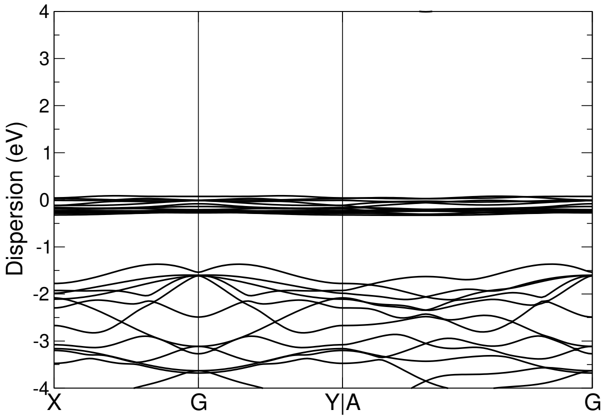

Band structure: Electronic band structure of $Er_2O_2Cl_2$ (P-3m1) in a representative window around the Fermi energy and along a high-symmetry path.

Optimized crystal structure

Structural parameters: relaxed cell (top) and atomic positions (bottom) of $Er_2O_2Cl_2$ (P-3m1) in Cartesian coordinates.

	x [Å]	y [Å]	z [Å]
$\mathbf{a_1}$	1.89814571	−3.28775394	0.00000000
$\mathbf{a_2}$	1.89820558	3.28778850	0.00000000
$\mathbf{a_3}$	0.00000000	0.00000000	24.51526075

		x [Å]	y [Å]	z [Å]
●	Er	-0.94908372	0.54795374	-1.42695337
●	Er	0.94908372	-0.54795374	1.42695337
●	Cl	0.94909540	1.64389852	-3.14836802
●	Cl	0.94911018	1.64388998	3.14836802
●	O	-0.94909243	0.54795877	0.84210185
●	O	0.94909243	-0.54795877	-0.84210185

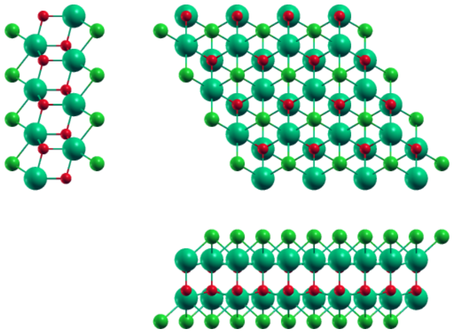

Orthographic projections: views of $Er_2O_2Cl_2$ (P-3m1) as seen from the x axis (left), the y axis (bottom) and the z axis (center).

Lattice matching - minimal size

Formula	N° atoms	strain	cell size 1	cell size 2
Tl	7	0.2491	1	1
InSe	8	0.4687	1	1
HgO	8	0.115	1	1
AsSb	8	0.0059	1	1
Bi_2	8	0.4835	1	1
PbTe	8	0.4732	1	1
CaCl	8	0.1489	1	1
Cl_2Mn	9	0.2568	1	1
CdI_2	9	0.4776	1	1
$AgTe_2$	9	0.1115	1	1
$MoSe_2$	9	0.2481	1	1
$ReSe_2$	9	0.2756	1	1
S_2Ta	9	0.2597	1	1
Br_2Zn	9	0.0043	1	1
Br_2Ca	9	0.4807	1	1
$SiTe_2$	9	0.0002	1	1
NSr_2	9	0.0076	1	1
PbS_2	9	0.0033	1	1
BiClTe	9	0.4785	1	1
BrCdI	9	0.4509	1	1
FeI_2	9	0.0089	1	1
S_2Ti	9	0.2667	1	1
Mg_3	9	0.4281	1	1
Te_2Ti	9	0.004	1	1
NbS_2	9	0.2591	1	1
CrI_2	9	0.0085	1	1
BaF_2	9	0.4591	1	1
BiBrTe	9	0.493	1	1
$RhTe_2$	9	0.0083	1	1
Bi_2Pd	9	0.1196	1	1
Cl_2Co	9	0.2662	1	1
Br_2V	9	0.2744	1	1
ClNZr	9	0.2713	1	1
Cl_2Fe	9	0.2652	1	1
Se_2V	9	0.2502	1	1
AsKSn	9	0.4555	1	1
$PbTe_2$	9	0.4485	1	1
$NiTe_2$	9	0.0008	1	1
I_2V	9	0.0011	1	1
GeI_2	9	0.4738	1	1
Se_2Zr	9	0.0002	1	1
STl_2	9	0.4615	1	1
CdO_2	9	0.266	1	1
CoI_2	9	0.0064	1	1
GeS_2	9	0.1382	1	1
$MnSe_2$	9	0.1488	1	1
Cl_2Zr	9	0.2657	1	1
Br_2Mg	9	0.0088	1	1
I_2Ti	9	0.0067	1	1
GdI_2	9	0.5006	1	1

Table showing the first 50 lattice matching combinations with the smallest number of atoms in the supercell.

Lattice matching - minimal strain

Formula	N° atoms	strain	cell size 1	cell size 2
As_2Li_2Nd	911	0.0	91	73
I_4Sr_2	198	0.0001	25	8
Cu_2Sr_2	580	0.0001	64	49
Tl	358	0.0001	49	64
STl_2	843	0.0001	100	81
Bi_2	552	0.0001	73	57
Se_2Zr	9	0.0002	1	1
NbS_2	627	0.0002	64	81
GeI_2Y_2	911	0.0002	91	73
CdI_2	678	0.0002	81	64
Bi_2STe_2	806	0.0002	81	64
Br_2Ca_3Si	366	0.0002	36	25
$SiTe_2$	9	0.0002	1	1
$Br_2F_2Sr_2$	678	0.0003	65	48
S_2Ta	627	0.0003	64	81
I_2La_2Te	341	0.0003	36	25
$Ga_2Gd_2I_2$	780	0.0003	73	57
Br_2La_2P	806	0.0003	81	64
$Cl_2Hg_2N_2$	882	0.0003	103	44
$LiNbS_2$	708	0.0004	64	81
$SbSe_2Tl$	132	0.0004	16	9
Br_2Hg_3	429	0.0005	64	9
In	186	0.0005	25	36
As_2O_3	845	0.0005	100	49
$Br_2F_2Pb_2$	678	0.0005	65	48
$LiOS_2Ti$	707	0.0005	57	73
$LiMnTe_2$	742	0.0006	81	64
$Ge_2I_2La_2$	510	0.0006	49	36
$Ga_2I_2Tb_2$	780	0.0006	73	57
Cl_2Fe	711	0.0006	73	91
S_2Ti	786	0.0006	81	100
CdI_2	678	0.0006	81	64
Bi_2Se_2Te	911	0.0006	91	73
$ClKO_3$	611	0.0006	81	25
$I_2La_2Si_2$	984	0.0007	91	73
$Br_2N_2Ti_2$	558	0.0007	48	45
Se_2W	486	0.0007	49	64
As_2Li_2Pr	911	0.0007	91	73
Bi_2SeTe_2	723	0.0007	73	57
InSe	692	0.0007	91	73
O_2Zn	171	0.0007	16	25
HN_3OZn	600	0.0007	43	57
Gd_2GeI_2	806	0.0007	81	64
Se_2V	486	0.0007	49	64
Nd	199	0.0008	25	49
BaF_2	843	0.0008	100	81
$NiTe_2$	9	0.0008	1	1
$MoSe_2$	486	0.0009	49	64
GeI_2	678	0.0009	81	64
Br_2Ca_3Si	846	0.0009	96	45

Table showing the first 50 lattice matching combinations with the smallest strain needed to build the supercell.

$Er_2O_2I_2$ (P4/nmm)

Structural and electronic properties

**Formula**	$Er_2O_2I_2$
**Spacegroup**	P4/nmm
**Prototype**	PbClF
**Parent 3D**	$Er_2I_2O_2$
**Source DB**	MPDS
**DB ID**	S383717
**DF2-C09 Binding energy [meV/ Å^2]**	15.11
**RVV10 Binding energy [meV/ Å^2]**	N/A
**Band gap (PBE) [eV]**	0.0

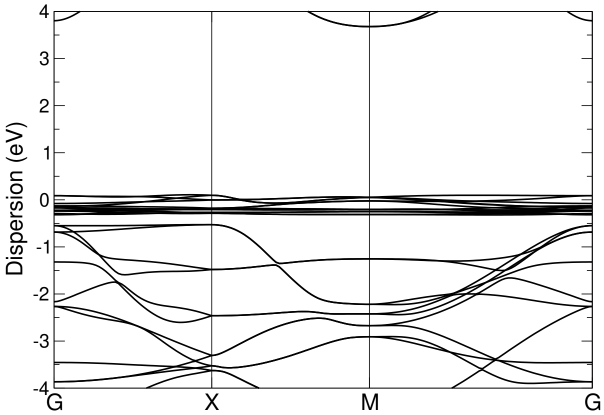

Band structure: Electronic band structure of $Er_2O_2I_2$ (P4/nmm) in a representative window around the Fermi energy and along a high-symmetry path.

Optimized crystal structure

Structural parameters: relaxed cell (top) and atomic positions (bottom) of $Er_2O_2I_2$ (P4/nmm) in Cartesian coordinates.

	x [Å]	y [Å]	z [Å]
$\mathbf{a_1}$	3.92899311	0.00000000	0.00000000
$\mathbf{a_2}$	0.00000000	3.92899311	0.00000000
$\mathbf{a_3}$	0.00000000	0.00000000	23.87698241

		x [Å]	y [Å]	z [Å]
●	Er	0.98224828	-0.98224828	1.07914474
●	I	-0.98224828	-2.94674483	3.03550709
●	Er	-0.98224828	-2.94674483	-1.07914474
●	I	0.98224828	-0.98224828	-3.03550709
●	O	-0.98224828	-0.98224828	0.00000000
●	O	0.98224828	-2.94674483	0.00000000

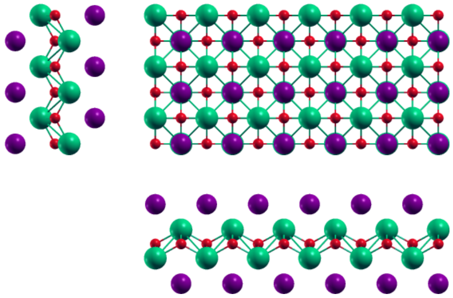

Orthographic projections: views of $Er_2O_2I_2$ (P4/nmm) as seen from the x axis (left), the y axis (bottom) and the z axis (center).

Lattice matching - minimal size

Formula	N° atoms	strain	cell size 1	cell size 2
K	7	0.1806	1	1
InSe	8	0.1431	1	1
Bi_2	8	0.1484	1	1
AgTl	8	0.02	1	1
Ag_2	8	0.1875	1	1
LiO	8	0.1103	1	1
P_2	8	0.1087	1	1
PbTe	8	0.1447	1	1
Sb_2	8	0.1304	1	1
I_2Mg	9	0.1346	1	1
CdI_2	9	0.1463	1	1
Nd	9	0.7456	1	3
Ba_2Pt	9	0.1871	1	1
Br_2Ca	9	0.1474	1	1
CaI_2	9	0.1697	1	1
I_2Pr	9	0.0027	1	1
Br_2La	9	0.1349	1	1
Br_2Cu	9	0.1043	1	1
Ca_2Si	9	0.193	1	1
I_2Yb	9	0.1668	1	1
BiClTe	9	0.1466	1	1
Cl_2Ti	9	0.1087	1	1
BrCdI	9	0.1371	1	1
HgI_2	9	0.4087	1	1
BaF_2	9	0.1398	1	1
BiBrTe	9	0.1518	1	1
$RhTe_2$	9	0.1087	1	1
GeI_2	9	0.1332	1	1
AsKSn	9	0.1386	1	1
$PbTe_2$	9	0.1363	1	1
I_2Nd	9	0.0018	1	1
Cl_2Cu	9	0.098	1	1
I_2Tm	9	0.1684	1	1
$SnTe_2$	9	0.1315	1	1
Cl_2V	9	0.1109	1	1
GeI_2	9	0.1449	1	1
I_2Pb	9	0.1895	1	1
STl_2	9	0.1406	1	1
BiITe	9	0.1584	1	1
GeS_2	9	0.2152	1	1
DyI_2	9	0.173	1	1
CeI_2	9	0.0035	1	1
Se_2Yb	9	0.1333	1	1
$BiTe_2$	9	0.1335	1	1
GdI_2	9	0.1547	1	1
$CrSe_2$	9	0.1112	1	1
I_2La	9	0.003	1	1
$CrSe_2$	9	0.1106	1	1
CdI_2	9	0.1459	1	1
F_2Zn	9	0.0066	1	1

Table showing the first 50 lattice matching combinations with the smallest number of atoms in the supercell.

Lattice matching - minimal strain

Formula	N° atoms	strain	cell size 1	cell size 2
Mg_2	456	0.0	49	81
$Br_2Eu_2F_2$	12	0.0	1	1
AlH_4Na	366	0.0001	36	25
Ho_2S_2	494	0.0001	49	50
$CuTe_2$	483	0.0002	48	65
HgI_2	540	0.0003	65	50
HfS_2	483	0.0003	48	65
Ag_2I_2	580	0.0003	64	49
Cu_2O_2	494	0.0003	49	50
Ag_2F_4	108	0.0003	12	6
Bi_2In_2	754	0.0003	85	61
$Bi_2Br_2O_2$	12	0.0004	1	1
$CoTe_2$	483	0.0006	48	65
FeH_2O_2	315	0.0006	20	39
Se_2Ta	840	0.0006	81	118
CaH_2O_2	613	0.0006	48	65
N_2W	237	0.0006	20	39
F_4Pb	817	0.0007	82	65
H_2Na_2Pd	800	0.0007	65	82
$Cu_2Rb_2Te_2$	876	0.0007	85	61
Ga_2S_2	548	0.0007	48	65
$I_2O_2Tm_2$	12	0.0007	1	1
$AgTe_2$	942	0.0007	89	136
H_4Ti	920	0.0007	65	106
$Cl_2N_2Zr_2$	678	0.0009	48	65
$Au_2K_2S_2$	78	0.0009	10	3
Br_2Ti	840	0.001	81	118
NbS_2	852	0.001	79	126
Ca_2O_2	706	0.0011	61	85
$AgBrO_2$	984	0.0011	96	102
Sn	70	0.0011	9	16
As_2O_3	973	0.0011	108	65
Br_2Cr	840	0.0013	81	118
$Ba_2H_2I_2$	510	0.0013	49	36
H_2Li_2Pd	920	0.0013	65	106
$I_2O_2Y_2$	12	0.0013	1	1
HgI_2	531	0.0014	64	49
S_2Ta	852	0.0014	79	126
$Br_2Hf_2N_2$	678	0.0014	48	65
F_4Pb	806	0.0014	81	64
H_2Na_2Pd	789	0.0014	64	81
$C_2Cl_2Y_2$	948	0.0014	72	86
Cl_2Sc_2	958	0.0014	81	118
Br_2Cu	837	0.0014	89	101
GeS_2	636	0.0015	65	82
LiO	602	0.0015	64	109
$CrTe_2$	840	0.0015	81	118
$HfLiS_2$	604	0.0015	52	73
$Br_2Nd_2O_2$	12	0.0015	1	1
$MoTe_2$	531	0.0015	52	73

Table showing the first 50 lattice matching combinations with the smallest strain needed to build the supercell.

$Er_2S_2Br_2$ (Pmm2)

Structural and electronic properties

**Formula**	$Er_2S_2Br_2$
**Spacegroup**	Pmm2
**Prototype**	FeOCl
**Parent 3D**	$Br_2Er_2S_2$
**Source DB**	MPDS
**DB ID**	S307276
**DF2-C09 Binding energy [meV/ Å^2]**	10.42
**RVV10 Binding energy [meV/ Å^2]**	N/A
**Band gap (PBE) [eV]**	0.0

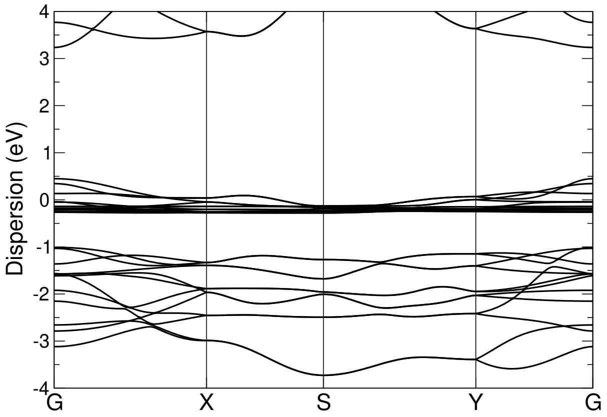

Band structure: Electronic band structure of $Er_2S_2Br_2$ (Pmm2) in a representative window around the Fermi energy and along a high-symmetry path.

Optimized crystal structure

Structural parameters: relaxed cell (top) and atomic positions (bottom) of $Er_2S_2Br_2$ (Pmm2) in Cartesian coordinates.

		x [Å]	y [Å]	z [Å]
$\mathbf{a_1}$		4.06377835	0.00000000	0.00000000
$\mathbf{a_2}$		0.00000000	5.28997400	0.00000000
$\mathbf{a_3}$		0.00000000	0.00000000	24.19069168
		x [Å]	y [Å]	z [Å]
●	Er	1.01608532	-1.32249350	-1.16754252
●	S	1.01583692	-3.96748050	-0.66610977
●	Br	-1.01571735	-1.32249350	-3.10789196
●	Er	-1.01608532	-3.96748050	1.16754252
●	S	-1.01583692	-1.32249350	0.66610977
●	Br	1.01571735	-3.96748050	3.10789196

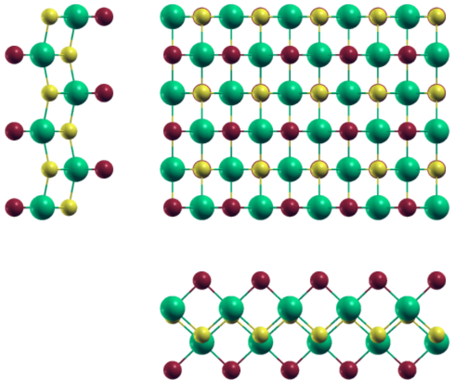

Orthographic projections: views of $Er_2S_2Br_2$ (Pmm2) as seen from the x axis (left), the y axis (bottom) and the z axis (center).

Lattice matching - minimal size

Formula	N° atoms	strain	cell size 1	cell size 2
AgTl	8	0.2591	1	1
In	8	0.1463	1	2
Gd	8	0.1315	1	2
Ba_2Hg	9	0.2748	1	1
CNRb	9	0.2271	1	1
CNNa	9	0.3088	1	1
Bi_2In_2	10	0.0633	1	1
Cu_2I_2	10	0.2599	1	1
O_2Pb_2	10	0.7616	1	1
As_4	10	0.3149	1	1
P_2	10	0.3734	1	2
O_2Sn_2	10	0.1689	1	1
Bi_2O_2	10	0.2571	1	1
PbS_2Sn	10	0.8126	1	1
$SbSe_2Tl$	10	0.4973	1	1
Br_2CsF	10	0.0626	1	1
Gd	10	0.1296	1	4
Sn_2Te_2	10	0.3445	1	1
F_4Sn	11	0.2736	1	1
FKO_2Se	11	1.1733	1	1
Cl_4Mn	11	0.0658	1	1
$Ba_2H_2I_2$	12	0.0619	1	1
CrS_2	12	0.1378	1	2
$Br_2Ho_2S_2$	12	0.0013	1	1
$I_2Lu_2Se_2$	12	0.5595	1	1
$Br_2F_2Sr_2$	12	0.2627	1	1
$Ho_2I_2S_2$	12	0.0109	1	1
Cu_4Te_2	12	0.1177	1	1
$Eu_2F_2I_2$	12	0.2564	1	1
AlH_4Na	12	0.3745	1	1
$GeTe_2$	12	0.6695	1	2
$Cl_2F_2Pb_2$	12	0.7458	1	1
$F_2I_2Sm_2$	12	0.7609	1	1
ReS_2	12	0.141	1	2
Br_2Ca_3Si	12	0.2751	1	1
Cl_2Ti	12	0.3737	1	2
$Ba_2Ge_2Mn_2$	12	0.7405	1	1
$Br_2S_2Y_2$	12	0.004	1	1
$Gd_2I_2S_2$	12	0.0164	1	1
$C_2Br_2Gd_2$	12	0.1078	1	1
N_2W	12	0.1346	1	2
$Br_2F_2Pb_2$	12	0.2623	1	1
$Cu_2Na_2Se_2$	12	0.7455	1	1
$Br_2Er_2Se_2$	12	0.0197	1	1
$I_2S_2Tb_2$	12	0.0141	1	1
Te_4TiZr	12	0.6698	1	1
$I_2S_2Yb_2$	12	0.0058	1	1
$Cu_2Rb_2Te_2$	12	0.0632	1	1
$Br_2Dy_2S_2$	12	0.0035	1	1
$Cl_2Ga_2Te_2$	12	0.0466	1	1

Table showing the first 50 lattice matching combinations with the smallest number of atoms in the supercell.

Lattice matching - minimal strain

Formula	N° atoms	strain	cell size 1	cell size 2
$LiMnTe_2$	848	0.0004	74	101
GeI_2	747	0.0004	74	101
K	194	0.0005	27	32
As_2CeLi_2	949	0.0005	74	101
CBr_2Lu_2	824	0.0005	54	100
$Br_2N_2Zr_2$	924	0.0005	54	100
PbTe	646	0.0005	74	101
In_2Se_2	48	0.0005	4	6
Bi_2STe_2	949	0.0006	74	101
I_2La_2Sb	322	0.0006	27	32
$I_2S_2Sm_2$	354	0.0006	27	32
AsSb	412	0.0006	44	74
As_2O_3	327	0.0007	32	27
BrNZr	291	0.0007	24	49
O_4PTl	738	0.0007	60	63
PTe_2Zr_2	634	0.0008	44	74
Te_2Zn	582	0.0008	49	96
Bi_2In_2	560	0.0008	56	56
Cl_2Hf_2	732	0.0008	50	108
O_2Sn_2	606	0.0009	47	81
CoI_2	486	0.0009	44	74
CdI_2	747	0.0009	74	101
$Cu_2Rb_2Te_2$	672	0.001	56	56
Br_2Hf_2	340	0.001	24	49
I_2Zn	42	0.001	4	6
Nd	75	0.001	8	27
IKO_3	414	0.001	49	24
$Ba_2F_2I_2$	738	0.001	60	63
$Ba_2F_2I_2$	726	0.001	59	62
Br_2La_2P	949	0.0011	74	101
As_4	896	0.0011	82	101
Bi_2STe_2	976	0.0011	76	104
O_4PTl	726	0.0011	59	62
Tl	171	0.0012	21	45
$RhTe_2$	681	0.0012	60	107
Hg_3N_2	821	0.0012	96	49
I_2Ti	486	0.0012	44	74
$NbTe_2$	624	0.0012	54	100
Ga_2S_3	774	0.0012	49	96
$Cl_2Fe_2O_2$	834	0.0012	49	90
Ag_2F_4	918	0.0012	90	63
C_4Ca_2	846	0.0012	62	79
Bi_2STe_2	949	0.0013	74	101
$Br_2Ho_2S_2$	12	0.0013	1	1
$Br_2Hf_2N_2$	726	0.0013	49	72
$AlLiTe_2$	784	0.0013	70	91
CNNa	552	0.0013	56	72
Mg_4	306	0.0014	21	45
CdI_2	747	0.0014	74	101
Cl_2OOs	696	0.0014	56	90

Table showing the first 50 lattice matching combinations with the smallest strain needed to build the supercell.

$Er_2S_2I_2$ (Pmm2)

Structural and electronic properties

**Formula**	$Er_2S_2I_2$
**Spacegroup**	Pmm2
**Prototype**	FeOCl
**Parent 3D**	$Er_2I_2S_2$
**Source DB**	MPDS
**DB ID**	S1937396
**DF2-C09 Binding energy [meV/ Å^2]**	11.36
**RVV10 Binding energy [meV/ Å^2]**	N/A
**Band gap (PBE) [eV]**	0.0

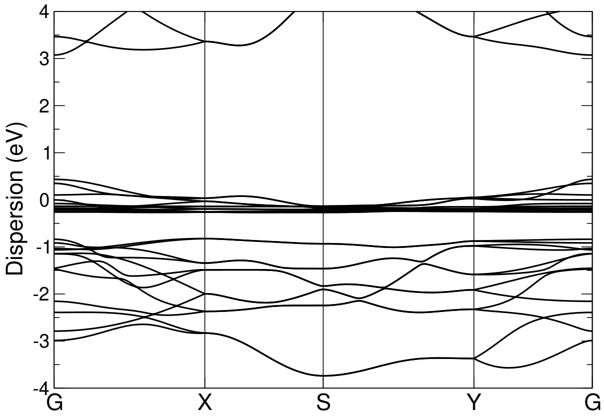

Band structure: Electronic band structure of $Er_2S_2I_2$ (Pmm2) in a representative window around the Fermi energy and along a high-symmetry path.

Optimized crystal structure

Structural parameters: relaxed cell (top) and atomic positions (bottom) of $Er_2S_2I_2$ (Pmm2) in Cartesian coordinates.

		x [Å]	y [Å]	z [Å]
$\mathbf{a_1}$		4.16967424	0.00000000	0.00000000
$\mathbf{a_2}$		0.00000000	5.30493940	0.00000000
$\mathbf{a_3}$		0.00000000	0.00000000	25.02562077
		x [Å]	y [Å]	z [Å]
●	Er	1.04244226	-1.32623485	-1.11433580
●	S	1.04243135	-3.97870455	-0.64347861
●	I	-1.04232476	-1.32623485	-3.33406727
●	Er	-1.04244226	-3.97870455	1.11433580
●	S	-1.04243135	-1.32623485	0.64347861
●	I	1.04232476	-3.97870455	3.33406727

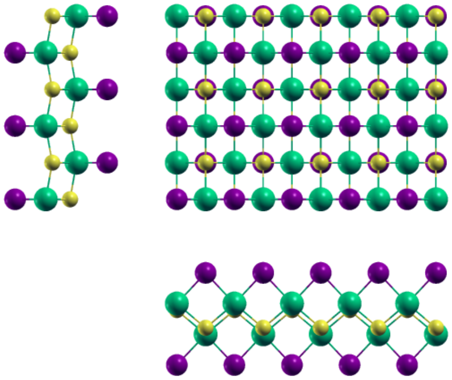

Orthographic projections: views of $Er_2S_2I_2$ (Pmm2) as seen from the x axis (left), the y axis (bottom) and the z axis (center).

Lattice matching - minimal size

Formula	N° atoms	strain	cell size 1	cell size 2
In	8	0.1458	1	2
Br_2Zn	9	0.1163	1	1
$AsSn_2$	9	0.1177	1	1
$SiTe_2$	9	0.1155	1	1
HgI_2	9	0.3087	1	1
Te_2Ti	9	0.1162	1	1
$RhTe_2$	9	0.1171	1	1
Ba_2Hg	9	0.2593	1	1
CNRb	9	0.2188	1	1
CKN	9	0.0108	1	1
$NiTe_2$	9	0.1156	1	1
$PtSe_2$	9	0.1176	1	1
CNNa	9	0.2929	1	1
Ba_2Cd	9	0.263	1	1
$HfSe_2$	9	0.1162	1	1
Bi_2In_2	10	0.0556	1	1
CdClHO	10	0.1173	1	1
Au_2Br_2	10	0.7872	1	1
Cl_2Y_2	10	0.1165	1	1
As_4	10	0.2985	1	1
Br_3Cs	10	0.3816	1	1
CaClHO	10	0.1157	1	1
P_2	10	0.3681	1	2
$AgClO_2$	10	0.1172	1	1
Ag_2I_2	10	0.3105	1	1
Ga_2Se_2	10	0.1166	1	1
Gd	10	0.0363	1	4
Sn_2Te_2	10	0.325	1	1
F_4Sn	11	0.2582	1	1
FKO_2Se	11	0.5464	1	1
F_4Nb	11	0.2503	1	1
$ClKO_3$	11	0.3895	1	1
Cl_4Mn	11	0.0572	1	1
$Ba_2H_2I_2$	12	0.3305	1	1
CrS_2	12	0.1378	1	2
$Br_2Ho_2S_2$	12	0.0072	1	1
$I_2Lu_2Se_2$	12	0.5326	1	1
$Ho_2I_2S_2$	12	0.0019	1	1
S_2Ta	12	0.3949	1	2
AlH_4Na	12	0.0582	1	1
$GeTe_2$	12	0.6391	1	2
Br_2Ca_3Si	12	0.2596	1	1
Cl_2Ti	12	0.3684	1	2
$I_2N_2Zr_2$	12	0.1169	1	1
NbS_2	12	0.3939	1	2
$Br_2S_2Y_2$	12	0.0102	1	1
$Gd_2I_2S_2$	12	0.0072	1	1
$C_2Br_2Gd_2$	12	0.1077	1	1
N_2W	12	0.1347	1	2
Pd_2S_4	12	0.9709	1	1

Table showing the first 50 lattice matching combinations with the smallest number of atoms in the supercell.

Lattice matching - minimal strain

Formula	N° atoms	strain	cell size 1	cell size 2
ClH_3O	651	0.0002	56	63
Cl_2Y_2	650	0.0004	49	89
$Ag_2K_2Te_2$	852	0.0005	72	70
$Ca_2Cl_2H_2$	876	0.0005	56	90
$Br_2O_2Sc_2$	276	0.0005	18	28
Cl_2Zn	606	0.0005	56	90
Bi_2In_2	708	0.0005	70	72
Ga_2Se_2	650	0.0006	49	89
$Br_2Hf_2N_2$	630	0.0007	42	63
$AsSe_2$	519	0.0008	42	89
$Cu_2Rb_2Te_2$	852	0.0008	70	72
Bi_2Se_2	988	0.0008	90	112
CBr_2Y_2	739	0.0008	49	89
I_2Y_2	956	0.0009	76	125
$NbSe_2$	519	0.0009	42	89
$Ag_2K_2Te_2$	840	0.0009	71	69
CNNa	477	0.0009	48	63
Hf_3Te_2	786	0.0009	56	90
Bi_2In_2	698	0.0009	69	71
$Cl_2H_2Zr_2$	474	0.0009	24	55
$ClKO_3$	779	0.001	89	49
Se_2Ta	519	0.001	42	89
$AgClO_2$	668	0.001	54	86
$Br_2Cr_2S_2$	354	0.001	24	35
$Cu_2Rb_2Te_2$	840	0.001	69	71
$NbSe_2$	519	0.001	42	89
$Br_2In_2O_2$	726	0.0011	49	72
$BrKO_3$	747	0.0011	87	45
As_4	708	0.0012	64	81
$Cl_2O_2Sc_2$	234	0.0012	15	24
$I_2N_2Zr_2$	918	0.0012	63	90
$AuCrTe_4$	354	0.0012	32	27
Te_2Zn	504	0.0013	42	84
Cu_2Se_2	876	0.0013	70	114
Mo_2Te_4	360	0.0013	30	30
CCl_2Gd_2	739	0.0014	49	89
Cl_2Ni	519	0.0014	42	89
Cl_2OOs	590	0.0014	47	77
O_2Sn_2	666	0.0014	51	90
F_2Ni	762	0.0014	70	114
Br_2Zn	561	0.0015	49	89
AlH_4Na	852	0.0015	71	71
Cu_2Te_2	754	0.0016	61	97
$I_2N_2Zr_2$	828	0.0016	49	89
I_2Y_2	932	0.0016	74	122
Ca_2O_2	280	0.0016	20	40
Hg_4O_2	846	0.0016	104	37
Cl_4Mn	781	0.0016	71	71
$Br_2N_2Zr_2$	684	0.0017	46	68
$As_2Co_2Li_2$	948	0.0017	61	97

Table showing the first 50 lattice matching combinations with the smallest strain needed to build the supercell.

$Er_2Se_2Br_2$ (Pmm2)

Structural and electronic properties

**Formula**	$Er_2Se_2Br_2$
**Spacegroup**	Pmm2
**Prototype**	FeOCl
**Parent 3D**	$Br_2Er_2Se_2$
**Source DB**	MPDS
**DB ID**	S1701716
**DF2-C09 Binding energy [meV/ Å^2]**	11.4
**RVV10 Binding energy [meV/ Å^2]**	N/A
**Band gap (PBE) [eV]**	0.0

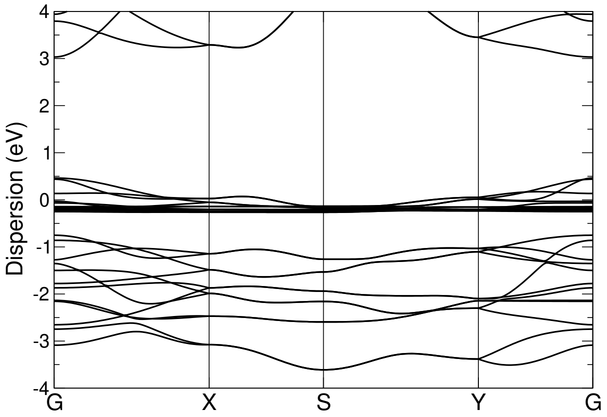

Band structure: Electronic band structure of $Er_2Se_2Br_2$ (Pmm2) in a representative window around the Fermi energy and along a high-symmetry path.

Optimized crystal structure

Structural parameters: relaxed cell (top) and atomic positions (bottom) of $Er_2Se_2Br_2$ (Pmm2) in Cartesian coordinates.

	x [Å]	y [Å]	z [Å]
$\mathbf{a_1}$	4.12400070	0.00000000	0.00000000
$\mathbf{a_2}$	0.00000000	5.58415994	0.00000000
$\mathbf{a_3}$	0.00000000	0.00000000	24.23451916

		x [Å]	y [Å]	z [Å]
●	Er	1.03115261	-1.39603999	-1.22935267
●	Se	1.03091124	-4.18811996	-0.78272038
●	Br	-1.03077807	-1.39603999	-3.13194790
●	Er	-1.03115261	-4.18811996	1.22935267
●	Se	-1.03091124	-1.39603999	0.78272038
●	Br	1.03077807	-4.18811996	3.13194790

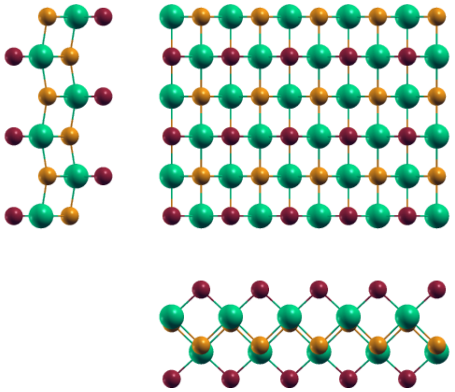

Orthographic projections: views of $Er_2Se_2Br_2$ (Pmm2) as seen from the x axis (left), the y axis (bottom) and the z axis (center).

Lattice matching - minimal size

Formula	N° atoms	strain	cell size 1	cell size 2
Tl	8	0.1448	1	2
In	8	0.1318	1	2
CNRb	9	0.2846	1	1
CKN	9	0.0263	1	1
CNNa	9	0.6158	1	1
I_2La	9	0.2281	1	1
Cu_2I_2	10	0.7422	1	1
Ag_2Br_2	10	0.2287	1	1
As_2Ir_2	10	0.2314	1	1
Au_2Se_2	10	0.2401	1	1
LiO	10	0.137	1	2
La_2S_2	10	0.2858	1	1
As_2Rh_2	10	0.2284	1	1
Se_2Sn_2	10	0.8631	1	1
F_4Pb	11	0.2987	1	1
$Br_2Ho_2S_2$	12	0.0172	1	1
S_2V	12	0.1333	1	2
MoS_2	12	0.1336	1	2
$Er_2I_2O_2$	12	0.2247	1	1
$I_2Lu_2Se_2$	12	0.0318	1	1
Cl_2Mn	12	0.3497	1	2
$MoSe_2$	12	0.1443	1	2
$Ho_2I_2S_2$	12	0.017	1	1
Cu_4Te_2	12	0.1186	1	1
$C_2I_2La_2$	12	1.1416	1	1
CaI_2	12	0.3363	1	2
$CuTe_2$	12	0.5356	1	2
PbS_2	12	0.609	1	2
$Cl_2Rh_2Te_2$	12	0.5983	1	1
$K_2O_2Tl_2$	12	0.5337	1	1
S_2Ti	12	0.1541	1	2
$I_2Se_2Tb_2$	12	0.0149	1	1
$Gd_2I_2Se_2$	12	0.0171	1	1
$Br_2S_2Y_2$	12	0.0152	1	1
S_2W	12	0.1337	1	2
Cl_2Co	12	0.1538	1	2
Pd_2S_4	12	0.8915	1	1
NbS_2	12	0.1468	1	2
Cl_2Fe	12	0.1532	1	2
S_2Ta	12	0.1462	1	2
Se_2V	12	0.1453	1	2
$I_2S_2Yb_2$	12	0.0165	1	1
$Br_2Dy_2S_2$	12	0.0161	1	1
$Er_2I_2Se_2$	12	0.0103	1	1
$Cl_2Ga_2Te_2$	12	0.0249	1	1
$I_2Se_2Tm_2$	12	0.0087	1	1
$Ag_2K_2Te_2$	12	0.0721	1	1
Cl_2V	12	0.1357	1	2
$Br_2Ga_2Te_2$	12	0.0269	1	1
Ca_4Cu_2	12	0.0227	1	1

Table showing the first 50 lattice matching combinations with the smallest number of atoms in the supercell.

Lattice matching - minimal strain

Formula	N° atoms	strain	cell size 1	cell size 2
NaO_4	440	0.0003	40	40
Br_2Co	801	0.0004	66	135
Br_2Fe	801	0.0004	66	135
Au_2Se_2	872	0.0005	88	86
As_2	666	0.0006	66	135
$AgNO_3$	440	0.0006	40	40
Cl_2Zn	801	0.0008	66	135
Cl_4Cu_2	288	0.0008	30	18
I_2Ni	543	0.0008	48	85
Cl_2Zn	198	0.001	18	30
Se_4TiZr	762	0.001	64	63
Ca_2N	801	0.001	66	135
Cl_2OOs	180	0.0011	14	24
$I_2Pr_2Si_2$	138	0.0012	9	14
FeI_2	543	0.0012	48	85
Ni_2Te_2	628	0.0012	48	85
Cl_2V	96	0.0012	7	18
S_2Sn	381	0.0012	32	63
Tl	357	0.0012	43	99
NSr_2	477	0.0012	42	75
PSn_2	381	0.0012	32	63
Mg_4	654	0.0012	43	99
OTl_2	795	0.0013	65	135
S_2Zr	381	0.0013	32	63
Se_2Sn	477	0.0013	42	75
CdClO	795	0.0013	65	135
Te_2V	795	0.0013	65	135
Hg_4O_2	402	0.0013	49	18
Br_2Mg	543	0.0013	48	85
Mg_6	546	0.0013	28	63
CdO_2	591	0.0013	46	105
CrI_2	477	0.0013	42	75
$Ca_2Cl_2H_2$	288	0.0014	18	30
CI_2Y_2	627	0.0014	42	75
$NbTe_2$	381	0.0014	32	63
$CdCl_2$	543	0.0014	48	85
Bi_2In_2	462	0.0014	45	48
K	633	0.0014	87	111
I_2Mn	543	0.0015	48	85
KS_2Ti	936	0.0015	66	135
MnO_2	99	0.0015	6	21
Br_2Mg	477	0.0015	42	75
$Cl_2Er_2H_2$	798	0.0016	48	85
FeI_2	477	0.0016	42	75
$I_2N_2Ti_2$	474	0.0016	30	49
$H_2Li_2O_2$	744	0.0016	44	80
$CrSe_2$	96	0.0016	7	18
I_2Ti	477	0.0016	42	75
C_2Li_2	504	0.0016	42	63
$Cl_2O_2Tm_2$	702	0.0016	41	76

Table showing the first 50 lattice matching combinations with the smallest strain needed to build the supercell.

$Er_2Se_2F_2$ (P-3m1)

Structural and electronic properties

**Formula**	$Er_2Se_2F_2$
**Spacegroup**	P-3m1
**Prototype**	SmSI
**Parent 3D**	$Er_2F_2Se_2$
**Source DB**	MPDS
**DB ID**	S307729
**DF2-C09 Binding energy [meV/ Å^2]**	16.2
**RVV10 Binding energy [meV/ Å^2]**	N/A
**Band gap (PBE) [eV]**	0.0

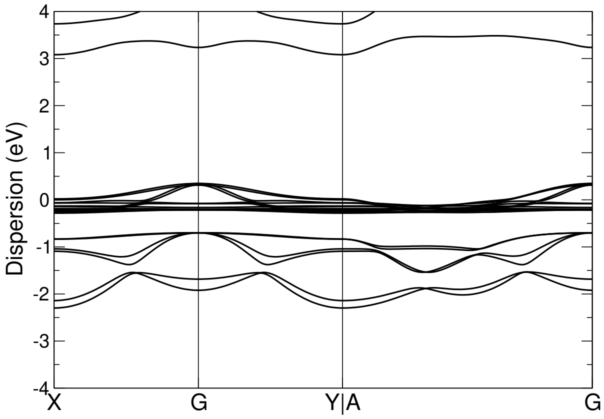

Band structure: Electronic band structure of $Er_2Se_2F_2$ (P-3m1) in a representative window around the Fermi energy and along a high-symmetry path.

Optimized crystal structure

Structural parameters: relaxed cell (top) and atomic positions (bottom) of $Er_2Se_2F_2$ (P-3m1) in Cartesian coordinates.

		x [Å]	y [Å]	z [Å]
$\mathbf{a_1}$		1.98429292	−3.43666158	0.00000000
$\mathbf{a_2}$		1.98408978	3.43654430	0.00000000
$\mathbf{a_3}$		0.00000000	0.00000000	25.11989189
		x [Å]	y [Å]	z [Å]
●	Er	0.99218348	-0.57283740	-1.70109915
●	Se	0.99206535	1.71826033	-3.29934984
•	F	0.99202141	4.00931524	-0.72617216
●	Er	2.97619921	0.57272012	1.70109915
●	Se	0.99202442	1.71828396	3.29934984
•	F	0.99206837	-0.57277094	0.72617216

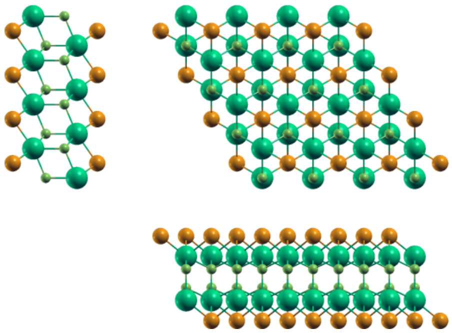

Orthographic projections: views of $Er_2Se_2F_2$ (P-3m1) as seen from the x axis (left), the y axis (bottom) and the z axis (center).

Lattice matching - minimal size

Formula	N° atoms	strain	cell size 1	cell size 2
Na	7	0.2632	1	1
GeTe	8	0.0088	1	1
Ag_2	8	2.9961	1	1
As_2	8	0.2747	1	1
S_2	8	0.0073	1	1
CaCl	8	0.1314	1	1
$IrTe_2$	9	0.0079	1	1
Cl_2Zn	9	0.2732	1	1
$MoTe_2$	9	0.2664	1	1
Ba_2Pt	9	2.9926	1	1
$ReSe_2$	9	0.2464	1	1
CaI_2	9	0.4824	1	1
$HfTe_2$	9	0.0002	1	1
Te_2V	9	0.2692	1	1
I_2Yb	9	0.4758	1	1
Br_2Co	9	0.2741	1	1
Ca_2N	9	0.2755	1	1
$AuTe_2$	9	0.0081	1	1
LiO_2	9	0.0713	1	1
Cl_2Zn	9	0.143	1	1
$PdTe_2$	9	0.0056	1	1
Te_2Zn	9	0.2662	1	1
Bi_2Pd	9	0.4433	1	1
Br_2Mn	9	0.2714	1	1
Cl_2Ni	9	0.2476	1	1
$CrTe_2$	9	0.2557	1	1
PtS_2	9	0.2647	1	1
CdClO	9	0.2685	1	1
Ba_2N	9	0.0018	1	1
Se_2Ti	9	0.2609	1	1
Br_2Ti	9	0.2549	1	1
Te_2Zr	9	0.0007	1	1
Te_2W	9	0.2667	1	1
$AsSe_2$	9	0.2495	1	1
I_2Tm	9	0.4793	1	1
OTl_2	9	0.2688	1	1
BiITe	9	0.4562	1	1
CdO_2	9	1.5541	1	1
$NbSe_2$	9	0.2481	1	1
Br_2Fe	9	0.2742	1	1
GeS_2	9	0.1233	1	1
$MnSe_2$	9	0.1313	1	1
Br_2Cr	9	0.2554	1	1
DyI_2	9	2.8419	1	1
Se_2Ta	9	0.2483	1	1
$NbSe_2$	9	0.2499	1	1
GdI_2	9	0.4472	1	1
F_2Ni	9	0.1396	1	1
Se_2Ta	9	0.2539	1	1
$PtTe_2$	9	0.0077	1	1

Table showing the first 50 lattice matching combinations with the smallest number of atoms in the supercell.

Lattice matching - minimal strain

Formula	N° atoms	strain	cell size 1	cell size 2
Ag_2	366	0.0	49	36
I_2Yb	678	0.0001	81	64
Br_2Cr	561	0.0001	57	73
Cl_2Sc_2	634	0.0001	57	73
H_2MgO_2	221	0.0002	16	25
$Br_2H_2Zr_2$	780	0.0002	57	73
$AsSe_2$	486	0.0002	49	64
$CrTe_2$	561	0.0002	57	73
CdH_2O_2	986	0.0002	81	100
Ba_2Cu_2	742	0.0002	81	64
$HfTe_2$	9	0.0002	1	1
$Gd_2I_2S_2$	678	0.0002	64	49
PtS_2	711	0.0002	73	91
In_2Se_3	11	0.0003	1	1
Cl_2Zr_2	486	0.0003	43	57
Ba_2Pt	402	0.0003	49	36
CaI_2	609	0.0003	73	57
NS_2Zr	802	0.0004	73	91
Br_2Ti	561	0.0004	57	73
$I_2S_2Sm_2$	600	0.0004	57	43
$Br_2Hf_2N_2$	624	0.0004	54	50
$Ge_2I_2La_2$	984	0.0004	91	73
Ga_2S_3	893	0.0004	73	91
Cl_2Fe	363	0.0004	36	49
ClH_3O	341	0.0004	36	25
$Cl_2Cr_2O_2$	906	0.0005	70	81
$NbSe_2$	486	0.0005	49	64
Pt_2Te_2	10	0.0005	1	1
Br_2Ca_3Si	678	0.0005	64	49
Sn_2Te_2	276	0.0005	32	21
Br_2V	429	0.0005	43	57
$H_2I_2Sr_2$	678	0.0006	65	48
CdClO	786	0.0006	81	100
$HNiO_2$	118	0.0006	9	16
Te_2W	786	0.0006	81	100
NaPSn	9	0.0007	1	1
CCl_2Sc_2	543	0.0007	43	57
$I_2Pr_2S_2$	510	0.0007	49	36
Te_2Zr	9	0.0007	1	1
K	385	0.0007	57	43
Se_2Ta	486	0.0007	49	64
Na	529	0.0008	73	91
CoH_2O_2	527	0.0008	37	61
Br_2Zr_2	708	0.0008	64	81
$MoTe_2$	786	0.0008	81	100
OTl_2	786	0.0008	81	100
Cl_2Zr	363	0.0008	36	49
$NbSe_2$	486	0.0008	49	64
Mg_2	722	0.0009	81	118
CNNa	834	0.0009	99	80

Table showing the first 50 lattice matching combinations with the smallest strain needed to build the supercell.

ErHCl (P-3m1)

Structural and electronic properties

**Formula**	ErHCl
**Spacegroup**	P-3m1
**Prototype**	SmSI
**Parent 3D**	$Er_2H_2Cl_2$
**Source DB**	COD
**DB ID**	1530725
**DF2-C09 Binding energy [meV/ Å^2]**	10.91
**RVV10 Binding energy [meV/ Å^2]**	17.19
**Band gap (PBE) [eV]**	N/A

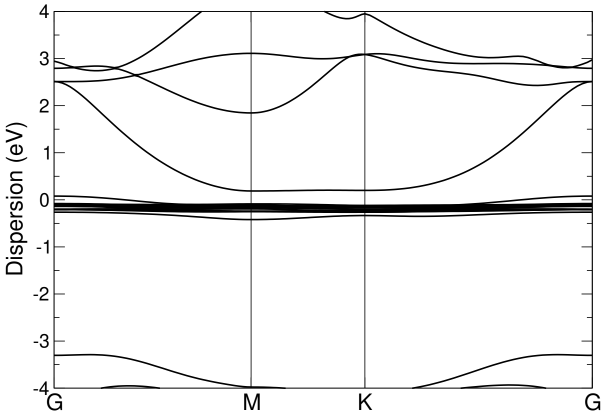

Band structure: Electronic band structure of ErHCl (P-3m1) in a representative window around the Fermi energy and along a high-symmetry path.

Optimized crystal structure

Structural parameters: relaxed cell (top) and atomic positions (bottom) of ErHCl (P-3m1) in Cartesian coordinates.

		x [Å]	y [Å]	z [Å]
$\mathbf{a_1}$		3.88641056	0.00000000	0.00000000
$\mathbf{a_2}$		−1.94320528	3.36573027	0.00000000
$\mathbf{a_3}$		0.00000000	0.00000000	26.11070302
		x [Å]	y [Å]	z [Å]
●	Er	1.94320528	1.12191009	11.59412418
·	H	1.94320528	1.12191009	13.83039162
●	Cl	0.00000000	0.00000000	10.01821820
●	Cl	0.00000000	0.00000000	16.09248482
●	Er	-0.00000000	2.24382018	14.51657884
·	H	-0.00000000	2.24382018	12.28031140

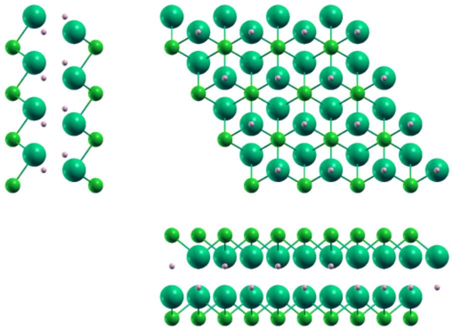

Orthographic projections: views of ErHCl (P-3m1) as seen from the x axis (left), the y axis (bottom) and the z axis (center).

Lattice matching - minimal size

Formula	N° atoms	strain	cell size 1	cell size 2
Tl	7	1.5366	1	1
HgO	8	0.4438	1	1
AsSb	8	0.0052	1	1
Bi_2	8	0.4555	1	1
GeTe	8	0.0008	1	1
S_2	8	0.0024	1	1
CaCl	8	0.139	1	1
$IrTe_2$	9	0.0018	1	1
$CdCl_2$	9	0.0002	1	1
CdI_2	9	0.4499	1	1
$AgTe_2$	9	0.4232	1	1
$MoSe_2$	9	1.5312	1	1
$ReSe_2$	9	0.2596	1	1
S_2Ta	9	1.5934	1	1
Br_2Ca	9	0.4529	1	1
CaI_2	9	2.9325	1	1
$InSe_2$	9	0.0001	1	1
$GeTe_2$	9	0.0012	1	1
I_2Mn	9	0.0001	1	1
NSr_2	9	0.0036	1	1
Ca_2Si	9	3.1905	1	1
I_2Yb	9	0.5017	1	1
PbS_2	9	0.0077	1	1
BiClTe	9	0.4508	1	1
LiO_2	9	0.0671	1	1
Cl_2Zn	9	0.1521	1	1
FeI_2	9	0.0023	1	1
I_2Ni	9	0.001	1	1
S_2Ti	9	1.6305	1	1
NbS_2	9	1.59	1	1
CrI_2	9	0.0027	1	1
BiBrTe	9	0.4645	1	1
Bi_2Pd	9	0.1143	1	1
N_2W	9	4.8643	1	1
Cl_2Ni	9	0.2609	1	1
Cl_2Co	9	1.6278	1	1
$CrTe_2$	9	0.2696	1	1
Br_2V	9	0.2585	1	1
ClNZr	9	0.2556	1	1
Cl_2Fe	9	0.25	1	1
S_2Ta	9	1.5523	1	1
Se_2V	9	1.5427	1	1
Se_2Ti	9	0.2752	1	1
Br_2Ti	9	0.2688	1	1
$AsSe_2$	9	0.2629	1	1
I_2Tm	9	2.9172	1	1
BiITe	9	0.4811	1	1
BrNZr	9	0.2648	1	1
$NbSe_2$	9	0.2615	1	1
CoI_2	9	0.0047	1	1

Table showing the first 50 lattice matching combinations with the smallest number of atoms in the supercell.

Lattice matching - minimal strain

Formula	N° atoms	strain	cell size 1	cell size 2
Se_2Ta	786	0.0	81	100
GeI_2La_2	557	0.0	57	43
Cu_2Sr_2	838	0.0	91	73
FeH_2O_2	527	0.0001	37	61
Br_2Hf_2	802	0.0001	73	91
I_2Mn	9	0.0001	1	1
ClNZr	561	0.0001	57	73
S_2Ta	429	0.0001	43	57
$InSe_2$	9	0.0001	1	1
$LiNbS_2$	486	0.0002	43	57
I_2Tm	471	0.0002	57	43
$CdCl_2$	9	0.0002	1	1
$F_2I_2Pb_2$	678	0.0002	65	48
$ReSe_2$	627	0.0002	64	81
$Br_2Cr_2O_2$	894	0.0003	73	76
N_3W_2	134	0.0003	9	16
NbS_2	429	0.0003	43	57
Ba_2Cd	534	0.0003	65	48
BrNZr	711	0.0004	73	91
$Gd_2I_2S_2$	510	0.0004	49	36
CNNa	642	0.0004	77	60
Ni_2Te_2	10	0.0004	1	1
H_2Si_2	10	0.0005	1	1
N_2W	537	0.0005	49	81
Sb_2Te_2	316	0.0005	36	25
Cl_2Fe	486	0.0005	49	64
Br_2V	627	0.0006	64	81
Se_2V	363	0.0006	36	49
$F_2Se_2Y_2$	246	0.0006	25	16
Br_2Ca_3Si	510	0.0006	49	36
$Br_2H_2Zr_2$	780	0.0007	57	73
$NbSe_2$	711	0.0007	73	91
Cl_2Cu	558	0.0007	61	64
S_2Ta	363	0.0008	36	49
$Ge_2I_2La_2$	678	0.0008	64	49
Ca_2Si	291	0.0008	36	25
$Ce_2I_2S_2$	366	0.0008	36	25
BiITe	609	0.0008	73	57
Br_2Ti	786	0.0008	81	100
GeTe	8	0.0008	1	1
$AlLiTe_2$	742	0.0009	81	64
Bi_2Te_3	723	0.0009	73	57
Cl_2Zr	486	0.0009	49	64
I_2La_2P	911	0.001	91	73
BiBrTe	765	0.001	91	73
$AsSe_2$	711	0.001	73	91
LiO	222	0.001	25	36
ReS_2	171	0.001	16	25
As_2O_3	419	0.001	49	25
I_2Ni	9	0.001	1	1

Table showing the first 50 lattice matching combinations with the smallest strain needed to build the supercell.

ErSCl (Pmmn)

Structural and electronic properties

**Formula**	ErSCl
**Spacegroup**	Pmmn
**Prototype**	FeOCl
**Parent 3D**	$Er_2S_2Cl_2$
**Source DB**	ICSD
**DB ID**	21009
**DF2-C09 Binding energy [meV/ Å^2]**	11.91
**RVV10 Binding energy [meV/ Å^2]**	20.29
**Band gap (PBE) [eV]**	0.2

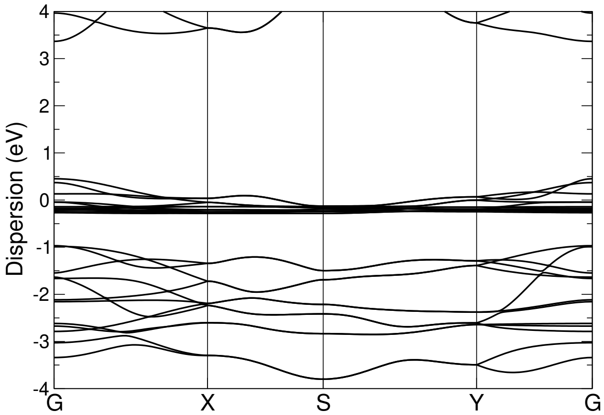

Band structure: Electronic band structure of ErSCl (Pmmn) in a representative window around the Fermi energy and along a high-symmetry path.

Optimized crystal structure

Structural parameters: relaxed cell (top) and atomic positions (bottom) of ErSCl (Pmmn) in Cartesian coordinates.

	x [Å]	y [Å]	z [Å]
$\mathbf{a_1}$	3.98426036	0.00000000	0.00000000
$\mathbf{a_2}$	0.00000000	5.28651622	0.00000000
$\mathbf{a_3}$	0.00000000	0.00000000	25.80628148

		x [Å]	y [Å]	z [Å]
●	Er	0.00000000	0.00000000	14.10208174
●	Er	1.99213018	2.64325811	11.70420322
●	S	1.99213018	0.00000000	12.21296180
●	S	0.00000000	2.64325811	13.59329093
●	Cl	1.99213018	0.00000000	15.84957715
●	Cl	0.00000000	2.64325811	9.95672106

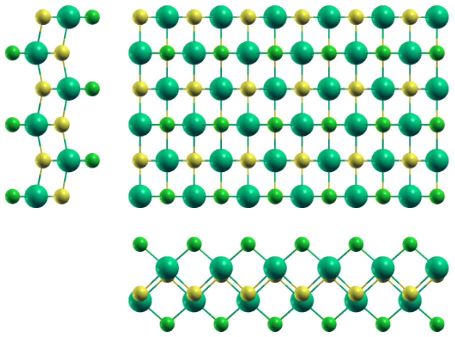

Orthographic projections: views of ErSCl (Pmmn) as seen from the x axis (left), the y axis (bottom) and the z axis (center).

Lattice matching - minimal size

Formula	N° atoms	strain	cell size 1	cell size 2
AgTl	8	0.2706	1	1
Br_2Cu	9	0.1196	1	1
Ba_2Hg	9	0.2873	1	1
CNRb	9	0.3225	1	1
CNNa	9	0.1519	1	1
Bi_2In_2	10	0.0704	1	1
As_4	10	0.3279	1	1
P_4	10	0.6289	1	1
LiO	10	0.3611	1	2
Au_2I_2	10	0.0646	1	1
PbS_2Sn	10	0.8477	1	1
$SbSe_2Tl$	10	0.5162	1	1
Br_2CsF	10	0.0694	1	1
Sn_2Te_2	10	0.0691	1	1
F_4Sn	11	0.286	1	1
FKO_2Se	11	1.1884	1	1
$Ba_2H_2I_2$	12	0.0685	1	1
CrS_2	12	0.1376	1	2
$Br_2Ho_2S_2$	12	0.008	1	1
$I_2Lu_2Se_2$	12	0.5807	1	1
$Ho_2I_2S_2$	12	0.018	1	1
Cu_4Te_2	12	0.1214	1	1
$C_2I_2La_2$	12	0.121	1	1
AlH_4Na	12	0.3914	1	1
$GeTe_2$	12	0.6932	1	2
$Cl_2F_2Pb_2$	12	0.7781	1	1
$Cl_2H_2Zr_2$	12	0.2823	1	1
ReS_2	12	0.1409	1	2
Br_2Ca_3Si	12	0.851	1	1
$Ba_2Ge_2Mn_2$	12	0.7726	1	1
$K_2O_2Tl_2$	12	0.5882	1	1
$Br_2S_2Y_2$	12	0.0064	1	1
BiBrTe	12	0.3517	1	2
$Gd_2I_2S_2$	12	0.0237	1	1
$C_2Br_2Gd_2$	12	0.1084	1	1
N_2W	12	0.1343	1	2
$Cu_2Na_2Se_2$	12	0.7779	1	1
$F_2I_2Yb_2$	12	0.7715	1	1
$I_2S_2Tb_2$	12	0.0213	1	1
Te_4TiZr	12	0.6935	1	1
$I_2S_2Yb_2$	12	0.0127	1	1
$Cu_2Rb_2Te_2$	12	0.0702	1	1
$Br_2Dy_2S_2$	12	0.0102	1	1
$Cl_2Ga_2Te_2$	12	0.557	1	1
Cl_2Cu	12	0.0953	1	2
$I_2Nd_2O_2$	12	0.2621	1	1
Cl_2V	12	0.3563	1	2
$Br_2Ga_2Te_2$	12	0.5666	1	1
Ca_4Cu_2	12	0.5556	1	1
O_4PSn	12	0.0669	1	1

Table showing the first 50 lattice matching combinations with the smallest number of atoms in the supercell.

Lattice matching - minimal strain

Formula	N° atoms	strain	cell size 1	cell size 2
PbTe	460	0.0007	53	71
As_2CeLi_2	673	0.0007	53	71
GeI_2	531	0.0008	53	71
GdI_2	591	0.0008	60	77
$LiMnTe_2$	602	0.0009	53	71
Br_2Hf_2	784	0.0009	56	112
I_2La_2P	745	0.0009	60	77
Bi_2Se_2	290	0.001	27	32
$NbTe_2$	411	0.001	36	65
C_2Li_2	460	0.001	40	55
S_2Zr	411	0.001	36	65
S_2Sn	411	0.0011	36	65
C_4Ca_2	660	0.0011	49	61
Bi_2STe_2	673	0.0011	53	71
NaO_4	624	0.0011	59	54
Bi_2STe_2	673	0.0011	53	71
Bi_2Pd	294	0.0012	25	48
$I_2N_2Ti_2$	360	0.0012	24	36
$As_2Cd_2K_2$	582	0.0012	48	49
FKO_2Se	712	0.0012	72	56
$Ag_2K_2Te_2$	624	0.0012	54	50
Bi_2In_2	486	0.0012	49	48
As_2Ir_2	34	0.0012	3	4
CrS_2	138	0.0012	10	26
$Ge_2Mn_2Sr_2$	42	0.0012	3	4
CNNa	645	0.0013	66	83
$AgTe_2$	147	0.0013	12	25
Ga_2S_3	876	0.0013	56	108
$Cu_2Rb_2Te_2$	582	0.0013	49	48
$AsLi_3$	626	0.0013	55	74
Cu_2F_4	42	0.0013	4	3
PSn_2	411	0.0014	36	65
$AgNO_3$	624	0.0014	59	54
$Br_2Hf_2N_2$	876	0.0014	60	86
Cl_2OOs	836	0.0014	68	107
InSe	478	0.0014	55	74
As_2Li_2Pr	700	0.0014	55	74
$I_2S_2Sm_2$	312	0.0015	24	28
CdI_2	531	0.0015	53	71
F_4Pb	570	0.0015	50	54
$Cl_2Fe_2O_2$	906	0.0015	54	97
Cu_4Te_2	282	0.0016	20	27
CBr_2Lu_2	541	0.0016	36	65
Te_4W_2	492	0.0016	42	40
Br_2La_2P	673	0.0016	53	71
$I_2La_2Si_2$	846	0.0016	60	81
$AsLi_3$	602	0.0016	53	71
$I_2Lu_2S_2$	12	0.0016	1	1
Ga_2Se_2	684	0.0016	54	90
$Br_2N_2Zr_2$	606	0.0016	36	65

Table showing the first 50 lattice matching combinations with the smallest strain needed to build the supercell.

ErSeI (Pmmn)

Structural and electronic properties

**Formula**	ErSeI
**Spacegroup**	Pmmn
**Prototype**	FeOCl
**Parent 3D**	$Er_2Se_2I_2$
**Source DB**	ICSD
**DB ID**	50194
**DF2-C09 Binding energy [meV/ Å^2]**	11.56
**RVV10 Binding energy [meV/ Å^2]**	18.61
**Band gap (PBE) [eV]**	N/A

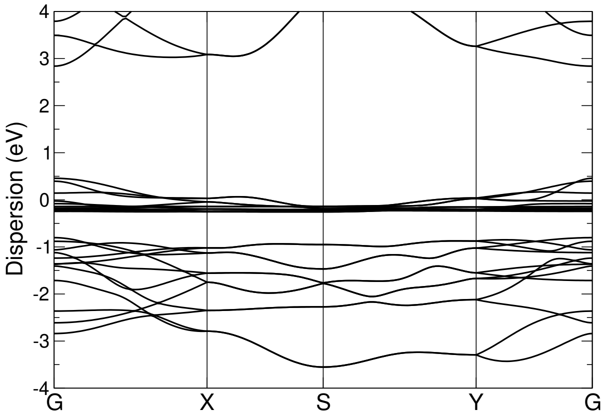

Band structure: Electronic band structure of ErSeI (Pmmn) in a representative window around the Fermi energy and along a high-symmetry path.

Optimized crystal structure

Structural parameters: relaxed cell (top) and atomic positions (bottom) of ErSeI (Pmmn) in Cartesian coordinates.

		x [Å]	y [Å]	z [Å]
$\mathbf{a_1}$		4.24971833	0.00000000	0.00000000
$\mathbf{a_2}$		0.00000000	5.59530767	0.00000000
$\mathbf{a_3}$		0.00000000	0.00000000	26.50880952
		x [Å]	y [Å]	z [Å]
●	Er	2.12485917	0.00000000	12.08509485
●	Se	2.12485917	2.79765383	12.49014880
●	I	0.00000000	0.00000000	9.91479230
●	Er	0.00000000	2.79765383	14.42363069
●	Se	0.00000000	0.00000000	14.01856445
●	I	2.12485917	2.79765383	16.59394385

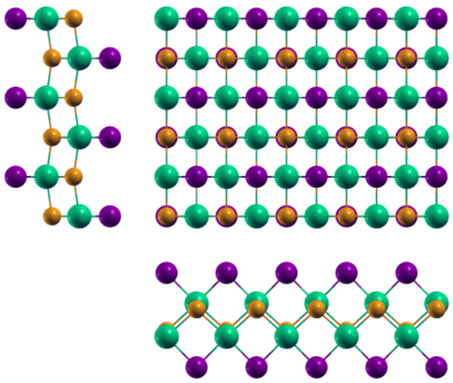

Orthographic projections: views of ErSeI (Pmmn) as seen from the x axis (left), the y axis (bottom) and the z axis (center).

Lattice matching - minimal size

Formula	N° atoms	strain	cell size 1	cell size 2
Tl	8	0.1443	1	2
CNRb	9	0.1972	1	1
In_2Se_2	10	0.086	1	1
Au_2Br_2	10	0.0701	1	1
Ge_2Te_2	10	0.8263	1	1
As_4	10	0.6135	1	1
Au_2Se_2	10	0.2195	1	1
LiO	10	0.137	1	2
Au_2I_2	10	0.769	1	1
P_2	10	0.1411	1	2
La_2S_2	10	0.8071	1	1
$SbSe_2Tl$	10	0.1732	1	1
Se_2Sn_2	10	0.8094	1	1
F_4Pb	11	0.279	1	1
KNO_3	11	0.3219	1	1
Cl_2Mn	12	0.3447	1	2
$MoSe_2$	12	0.1438	1	2
$ReSe_2$	12	0.3699	1	2
$Ho_2I_2S_2$	12	0.0172	1	1
$CuTe_2$	12	0.5065	1	2
PbS_2	12	0.5764	1	2
Cl_2Ti	12	0.1412	1	2
$K_2O_2Tl_2$	12	0.1872	1	1
$I_2Se_2Tb_2$	12	0.0045	1	1
$Gd_2I_2Se_2$	12	0.0066	1	1
$Gd_2I_2S_2$	12	0.0152	1	1
Pd_2S_4	12	0.3522	1	1
$Br_2Er_2Se_2$	12	0.0097	1	1
NbS_2	12	0.1462	1	2
Br_2V	12	0.3683	1	2
ClNZr	12	0.3642	1	2
S_2Ta	12	0.3382	1	2
Se_2V	12	0.1448	1	2
$I_2S_2Tb_2$	12	0.0159	1	1
$I_2Se_2Tm_2$	12	0.0016	1	1
$SnTe_2$	12	0.6898	1	2
Cl_2V	12	0.1357	1	2
Ca_4Cu_2	12	0.0217	1	1
$H_2I_2Sr_2$	12	0.7779	1	1
$Dy_2I_2S_2$	12	0.0164	1	1
Se_4TiZr	12	0.2282	1	1
$CrSe_2$	12	0.1352	1	2
$I_2Se_2Yb_2$	12	0.0025	1	1
Te_4W_2	12	0.5094	1	1
$AgClO_4$	12	0.065	1	1
$CrSe_2$	12	0.1363	1	2
$C_2Br_2La_2$	12	0.109	1	1
Cl_4Pd_2	12	0.0465	1	1
$Cl_2S_2Tl_2$	12	0.3818	1	1
$Ho_2I_2Se_2$	12	0.001	1	1

Table showing the first 50 lattice matching combinations with the smallest number of atoms in the supercell.

Lattice matching - minimal strain

Formula	N° atoms	strain	cell size 1	cell size 2
$F_2Se_2Y_2$	312	0.0005	24	28
$Dy_2I_2S_2$	846	0.0005	60	81
C_2Li_2	220	0.0006	18	28
Br_2Pr_2	768	0.0006	56	108
Cl_2La_2	684	0.0007	54	90
F_2Na	660	0.0007	56	108
DyI_2	603	0.0007	60	81
FeI_2	345	0.0009	30	55
Br_2Mg	345	0.0009	30	55
Cl_4Mg_2	978	0.0009	107	56
In_2Se_2	684	0.001	54	90
Au_2Br_2	866	0.001	77	101
$Ho_2I_2Se_2$	12	0.001	1	1
Ag_2Te_2	876	0.001	70	114
Au_2Br_2	832	0.001	74	97
CrI_2	345	0.001	30	55
$I_2S_2Tb_2$	846	0.0011	60	81
Ba_2Pt	591	0.0011	60	77
$GeTe_2$	345	0.0011	30	55
Au_2Br_2	394	0.0011	35	46
As_2Sn_2	622	0.0011	49	82
Ag_2	514	0.0011	60	77
Cl_2La_2	622	0.0012	49	82
CNRb	702	0.0012	77	80
$Cl_2S_2Tl_2$	660	0.0012	56	54
I_2Ni	345	0.0013	30	55
CdClHO	724	0.0013	52	103
Cu_2Te_2	180	0.0013	14	24
Au_2Br_2	360	0.0014	32	42
$Cl_2O_2Tm_2$	984	0.0014	56	108
Br_2Mn	681	0.0014	55	117
KS_2Ti	798	0.0015	55	117
$RhTe_2$	621	0.0015	52	103
$AgTe_2$	156	0.0015	12	28
In_2Se_2	906	0.0015	89	93
$K_2O_2Tl_2$	966	0.0015	80	81
$Cl_2S_2Tl_2$	648	0.0015	55	53
$I_2Pr_2S_2$	822	0.0016	60	77
In_2Se_2	702	0.0016	69	72
I_2Zn	615	0.0016	56	93
$CuTe_2$	660	0.0016	54	112
$I_2Se_2Tm_2$	12	0.0016	1	1
$I_2Nd_2S_2$	894	0.0016	65	84
CaI_2	675	0.0016	67	91
$As_2Co_2Li_2$	228	0.0016	14	24
Pd_2S_4	96	0.0017	9	7
$AgNO_2$	954	0.0017	81	117
CCl_2Gd_2	876	0.0017	56	108
Mg_6	240	0.0017	12	28
Ag_2Te_2	852	0.0017	68	111

Table showing the first 50 lattice matching combinations with the smallest strain needed to build the supercell.

$Eu_2H_2I_2$ (P4/nmm)

Structural and electronic properties

**Formula**	$Eu_2H_2I_2$
**Spacegroup**	P4/nmm
**Prototype**	PbClF
**Parent 3D**	$Eu_2H_2I_2$
**Source DB**	MPDS
**DB ID**	S1937757
**DF2-C09 Binding energy [meV/ Å^2]**	19.71
**RVV10 Binding energy [meV/ Å^2]**	N/A
**Band gap (PBE) [eV]**	0.0

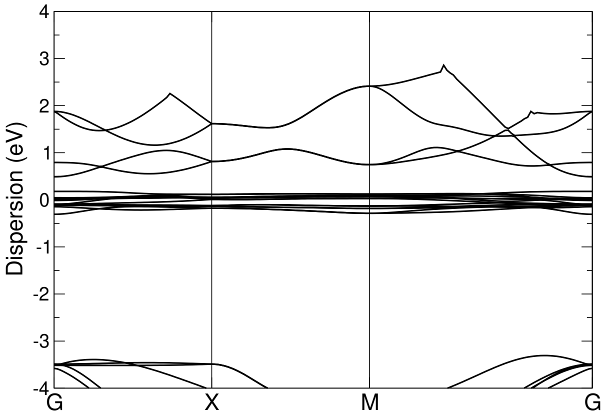

Band structure: Electronic band structure of $Eu_2H_2I_2$ (P4/nmm) in a representative window around the Fermi energy and along a high-symmetry path.

Optimized crystal structure

Structural parameters: relaxed cell (top) and atomic positions (bottom) of $Eu_2H_2I_2$ (P4/nmm) in Cartesian coordinates.

	x [Å]	y [Å]	z [Å]
$\mathbf{a_1}$	4.00916301	0.00000000	0.00000000
$\mathbf{a_2}$	0.00000000	4.00916301	0.00000000
$\mathbf{a_3}$	0.00000000	0.00000000	22.68086329

	x [Å]	y [Å]	z [Å]
Eu	1.00229075	-1.00229075	1.23056445
I	-1.00229075	-3.00687226	2.86412795
Eu	-1.00229075	-3.00687226	-1.23056445
H	-1.00229075	-1.00229075	0.00000000
H	1.00229075	-3.00687226	0.00000000
I	1.00229075	-1.00229075	-2.86412795

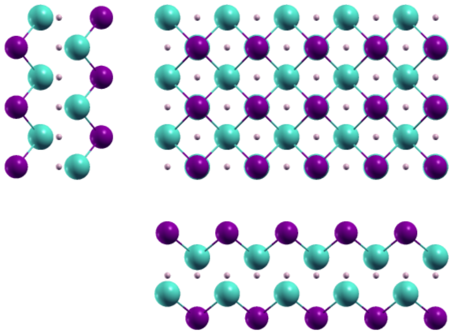

Orthographic projections: views of $Eu_2H_2I_2$ (P4/nmm) as seen from the x axis (left), the y axis (bottom) and the z axis (center).

Lattice matching - minimal size

Formula	N° atoms	strain	cell size 1	cell size 2
K	7	0.1693	1	1
Tl	7	0.1096	1	1
InSe	8	0.1352	1	1
Bi_2	8	0.1399	1	1
AgTl	8	0.0136	1	1
Ag_2	8	0.1757	1	1
P_2	8	0.1107	1	1
PbTe	8	0.1366	1	1
CaCl	8	0.2181	1	1
CdI_2	9	0.138	1	1
$MoSe_2$	9	0.1098	1	1
Ba_2Pt	9	0.1754	1	1
Br_2Ca	9	0.139	1	1
CaI_2	9	0.1593	1	1
$SiTe_2$	9	0.1084	1	1
Br_2Cu	9	0.1008	1	1
Ca_2Si	9	0.1808	1	1
I_2Yb	9	0.1566	1	1
BiClTe	9	0.1383	1	1
Cl_2Ti	9	0.1107	1	1
BrCdI	9	0.1298	1	1
HgI_2	9	0.3875	1	1
BaF_2	9	0.1322	1	1
BiBrTe	9	0.143	1	1
NbS_2	9	0.109	1	1
S_2Ta	9	0.1091	1	1
Se_2V	9	0.1094	1	1
AsKSn	9	0.1312	1	1
Cl_2Cu	9	0.0967	1	1
I_2Tm	9	0.158	1	1
I_2V	9	0.1087	1	1
GeI_2	9	0.1368	1	1
Se_2Zr	9	0.1085	1	1
I_2Pb	9	0.1776	1	1
STl_2	9	0.133	1	1
BiITe	9	0.149	1	1
GeS_2	9	0.5953	1	1
$MnSe_2$	9	0.218	1	1
DyI_2	9	0.1623	1	1
GdI_2	9	0.1455	1	1
I_2La	9	0.0065	1	1
CdI_2	9	0.1376	1	1
Sm	9	0.1637	1	3
I_2Pr	9	0.1383	1	1
Se_2W	9	0.1097	1	1
Bi_2Te_2	10	0.1973	1	1
Cu_2Sr_2	10	0.1439	1	1
Cl_2OOs	10	0.2246	1	1
$LiMnTe_2$	10	0.137	1	1
Cu_2Te_2	10	0.0565	1	1

Table showing the first 50 lattice matching combinations with the smallest number of atoms in the supercell.

Lattice matching - minimal strain

Formula	N° atoms	strain	cell size 1	cell size 2
GeS_2	486	0.0001	49	64
As_2Fe_2	560	0.0001	50	65
$TaTe_2$	483	0.0002	48	65
$I_2O_2Sm_2$	12	0.0003	1	1
$Hf_2Se_2Si_2$	690	0.0004	50	65
$H_2Li_2O_2$	882	0.0004	65	82
$F_2I_2Yb_2$	12	0.0004	1	1
HgO	806	0.0004	89	136
$H_2Li_2O_2$	870	0.0004	64	81
$Cu_2K_2Te_2$	690	0.0004	65	50
Co_2S_2	886	0.0004	81	100
$Hf_2I_2N_2$	678	0.0005	48	65
CdClHO	548	0.0006	48	65
$AsSn_2$	483	0.0006	48	65
Tl	859	0.0007	113	181
H_2Na_2Pd	614	0.0007	49	64
$Ba_2Ge_2Mn_2$	12	0.0007	1	1
Mg_6	246	0.0007	16	25
Te_2W	840	0.0007	81	118
$AgClO_2$	852	0.0007	80	93
Pb_2Se_2	912	0.0007	98	81
$MoTe_2$	840	0.0008	81	118
Mg_3	537	0.0009	49	81
As_2Fe_2	550	0.001	49	64
S_2Ti	852	0.001	79	126
Cl_2Co	852	0.0011	79	126
CaCl	686	0.0011	81	100
CdClO	840	0.0011	81	118
$HfLiS_2$	958	0.0012	81	118
$MnSe_2$	786	0.0012	81	100
O_4PTl	870	0.0012	81	64
Cl_2Zr	852	0.0012	79	126
$Ba_2F_2I_2$	870	0.0012	81	64
Cl_4Mn	474	0.0013	49	36
OTl_2	840	0.0013	81	118
$PtSe_2$	483	0.0013	48	65
$Eu_2I_2O_2$	12	0.0013	1	1
$AgClO_2$	468	0.0014	44	51
Cl_2Fe	852	0.0014	79	126
$AgClO_2$	404	0.0014	38	44
$Hf_2Se_2Si_2$	678	0.0015	49	64
$Ba_2H_2I_2$	678	0.0015	64	49
Hg_4O_2	906	0.0015	120	31
$Cu_2K_2Te_2$	678	0.0015	64	49
Te_2V	840	0.0015	81	118
Ga_2S_2	604	0.0016	52	73
CrI_2	603	0.0016	62	77
$CoTe_2$	531	0.0016	52	73
Br_2Mg	603	0.0016	62	77
AuI_4Li	732	0.0016	81	41

Table showing the first 50 lattice matching combinations with the smallest strain needed to build the supercell.

$Eu_2I_2F_2$ (P4/nmm)

Structural and electronic properties

**Formula**	$Eu_2I_2F_2$
**Spacegroup**	P4/nmm
**Prototype**	PbClF
**Parent 3D**	$Eu_2F_2I_2$
**Source DB**	MPDS
**DB ID**	S1800106
**DF2-C09 Binding energy [meV/ Å^2]**	16.64
**RVV10 Binding energy [meV/ Å^2]**	N/A
**Band gap (PBE) [eV]**	0.0

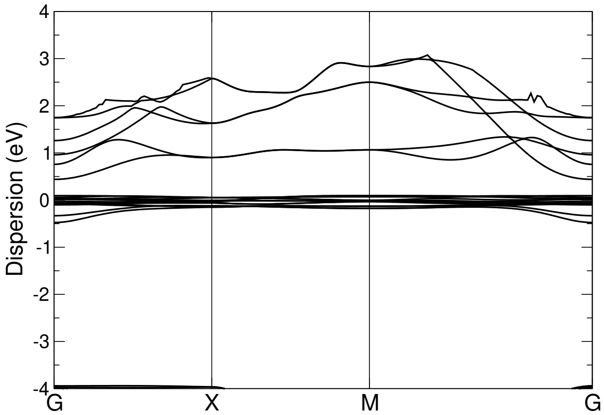

Band structure: Electronic band structure of $Eu_2I_2F_2$ (P4/nmm) in a representative window around the Fermi energy and along a high-symmetry path.

Optimized crystal structure

Structural parameters: relaxed cell (top) and atomic positions (bottom) of $Eu_2I_2F_2$ (P4/nmm) in Cartesian coordinates.

	x [Å]	y [Å]	z [Å]
$\mathbf{a_1}$	4.06997026	0.00000000	0.00000000
$\mathbf{a_2}$	0.00000000	4.06997026	0.00000000
$\mathbf{a_3}$	0.00000000	0.00000000	23.87445915

		x [Å]	y [Å]	z [Å]
●	Eu	1.01749256	-1.01749256	1.45923737
●	I	-1.01749256	-3.05247769	3.06595784
●	Eu	-1.01749256	-3.05247769	-1.45923737
●	I	1.01749256	-1.01749256	-3.06595784
●	F	-1.01749256	-1.01749256	0.00000000
●	F	1.01749256	-3.05247769	0.00000000

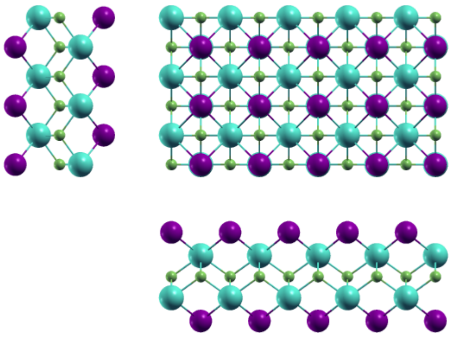

Orthographic projections: views of $Eu_2I_2F_2$ (P4/nmm) as seen from the x axis (left), the y axis (bottom) and the z axis (center).

Lattice matching - minimal size

Formula	N° atoms	strain	cell size 1	cell size 2
K	7	0.1615	1	1
Tl	7	0.1112	1	1
InSe	8	0.1299	1	1
Bi_2	8	0.1342	1	1
Ag_2	8	0.1675	1	1
PbTe	8	0.1311	1	1
CaCl	8	0.2102	1	1
Cl_2Mn	9	0.1099	1	1
CdI_2	9	0.1324	1	1
Nd	9	0.1744	1	3
$MoSe_2$	9	0.1114	1	1
Ba_2Pt	9	0.1672	1	1
S_2Ta	9	0.1094	1	1
Br_2Ca	9	0.1333	1	1
CaI_2	9	0.1521	1	1
Br_2Cu	9	0.0989	1	1
NSr_2	9	0.1085	1	1
Ca_2Si	9	0.1723	1	1
I_2Yb	9	0.1496	1	1
BiClTe	9	0.1327	1	1
FeI_2	9	0.1088	1	1
NbS_2	9	0.1095	1	1
CrI_2	9	0.1087	1	1
BiBrTe	9	0.137	1	1
NbS_2	9	0.1105	1	1
S_2Ta	9	0.1107	1	1
CKN	9	1.3075	1	1
Se_2V	9	0.111	1	1
I_2Tm	9	0.1509	1	1
GeI_2	9	0.1313	1	1
I_2Pb	9	0.1692	1	1
BiITe	9	0.1425	1	1
$MnSe_2$	9	0.2101	1	1
DyI_2	9	0.1549	1	1
Br_2Mg	9	0.1088	1	1
GdI_2	9	0.1393	1	1
CNNa	9	0.0692	1	1
CdI_2	9	0.1321	1	1
Se_2Sn	9	0.1085	1	1
I_2Pr	9	0.1327	1	1
Se_2W	9	0.1113	1	1
Bi_2Te_2	10	0.1879	1	1
Bi_2In_2	10	0.4079	1	1
Cu_2I_2	10	0.0026	1	1
Cu_2Sr_2	10	0.1378	1	1
Cl_2OOs	10	0.2163	1	1
$LiMnTe_2$	10	0.1315	1	1
$AgCuTe_2$	10	0.1981	1	1
$AsLi_3$	10	0.1301	1	1
O_2Sn_2	10	0.0316	1	1

Table showing the first 50 lattice matching combinations with the smallest number of atoms in the supercell.

Lattice matching - minimal strain

Formula	N° atoms	strain	cell size 1	cell size 2
$Br_2La_2O_2$	12	0.0	1	1
CCl_2Gd_2	613	0.0003	48	65
Tl	375	0.0003	49	81
CBr_2Y_2	613	0.0003	48	65
$H_2Li_2O_2$	690	0.0004	50	65
CoO_2	714	0.0004	54	130
$Cu_2K_2Te_2$	882	0.0004	82	65
Br_2Zn	483	0.0004	48	65
Mg_2	86	0.0005	9	16
$C_2Br_2Tb_2$	948	0.0005	72	86
Bi_2O_2	10	0.0005	1	1
Cl_4KTl	738	0.0005	89	34
$AgTe_2$	708	0.0005	65	106
Bi_2In_2	590	0.0006	65	50
Mg_4	618	0.0007	49	81
Te_2Ti	483	0.0007	48	65
$H_2Li_2O_2$	678	0.0007	49	64
$HfSe_2$	483	0.0007	48	65
O_2Pb_2	10	0.0008	1	1
$Br_2O_2Ti_2$	66	0.0008	5	6
$Al_2Cl_2O_2$	918	0.0008	63	90
Cl_2Y_2	548	0.0009	48	65
$C_2Br_2Gd_2$	948	0.0009	72	86
CaCl	968	0.0009	113	145
$Cu_2Rb_2Te_2$	690	0.0009	65	50
NiO_2	714	0.0009	54	130
$F_2I_2Sm_2$	12	0.0009	1	1
$FeSe_2$	297	0.001	25	49
NiO_2	633	0.001	48	115
Cl_4Mg_2	762	0.001	93	34
HfS_2	840	0.001	81	118
Cl_2Ni	852	0.001	79	126
Se_2Ta_4	366	0.001	25	36
Cl_2ORu	708	0.0011	64	81
$NbSe_2$	852	0.0011	79	126
Se_2Ta	852	0.0012	79	126
Fe_2Se_2	896	0.0012	82	101
$Cu_2K_2Te_2$	870	0.0012	81	64
Mg_3	945	0.0012	85	145
Cl_2OOs	886	0.0012	81	100
$Ge_2Se_2Zr_2$	882	0.0012	65	82
Ca_2Cl_2	718	0.0012	65	82
CoO_2	633	0.0012	48	115
$CoTe_2$	840	0.0013	81	118
Sm	387	0.0013	44	123
$ReSe_2$	852	0.0014	79	126
$LiOS_2Ti$	929	0.0014	64	109
C_4Ca_2	642	0.0014	54	53
Ga_2S_2	958	0.0014	81	118
CNRb	735	0.0014	90	65

Table showing the first 50 lattice matching combinations with the smallest strain needed to build the supercell.

EuOBr (P4/nmm)

Structural and electronic properties

**Formula**	EuOBr
**Spacegroup**	P4/nmm
**Prototype**	PbClF
**Parent 3D**	$Eu_2O_2Br_2$
**Source DB**	ICSD
**DB ID**	28531
**DF2-C09 Binding energy [meV/ Å^2]**	17.38
**RVV10 Binding energy [meV/ Å^2]**	N/A
**Band gap (PBE) [eV]**	N/A

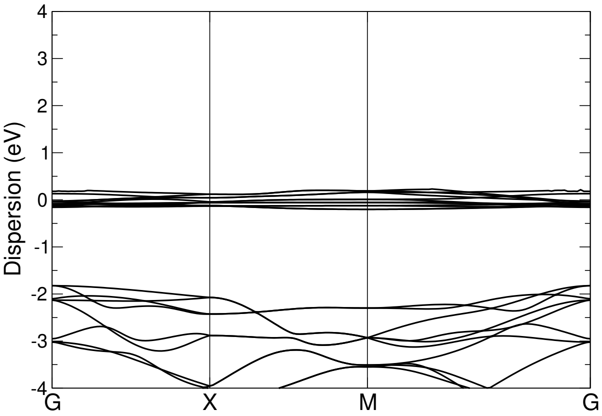

Band structure: Electronic band structure of EuOBr (P4/nmm) in a representative window around the Fermi energy and along a high-symmetry path.

Optimized crystal structure

Structural parameters: relaxed cell (top) and atomic positions (bottom) of EuOBr (P4/nmm) in Cartesian coordinates.

	x [Å]	y [Å]	z [Å]
$\mathbf{a_1}$	3.90047613	0.00000000	0.00000000
$\mathbf{a_2}$	0.00000000	3.90047613	0.00000000
$\mathbf{a_3}$	0.00000000	0.00000000	25.42586574

	x [Å]	y [Å]	z [Å]
Eu	0.00000000	1.95023806	13.88534255
Br	1.95023806	0.00000000	15.53246152
Eu	1.95023806	0.00000000	11.54052319
Br	0.00000000	1.95023806	9.89340422
O	1.95023806	1.95023806	12.71293287
O	0.00000000	0.00000000	12.71293287

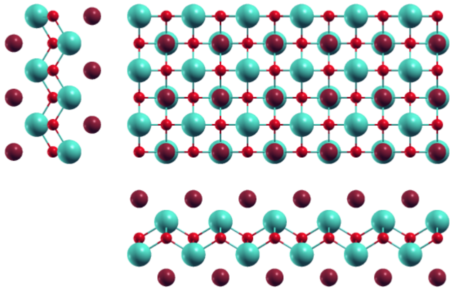

Orthographic projections: views of EuOBr (P4/nmm) as seen from the x axis (left), the y axis (bottom) and the z axis (center).

Lattice matching - minimal size

Formula	N° atoms	strain	cell size 1	cell size 2
K	7	0.3785	1	1
K	7	0.1849	1	1
InSe	8	0.1462	1	1
Bi_2	8	0.1517	1	1
AgTl	8	0.023	1	1
Ag_2	8	0.1919	1	1
LiO	8	0.1096	1	1
PbTe	8	0.1478	1	1
Sb_2	8	0.1329	1	1
I_2Mg	9	0.1373	1	1
S_2V	9	0.1112	1	1
MoS_2	9	0.111	1	1
CdI_2	9	0.1495	1	1
Ba_2Pt	9	0.1916	1	1
Br_2Ca	9	0.1506	1	1
CaI_2	9	0.1737	1	1
$AsSn_2$	9	0.1089	1	1
I_2Pr	9	0.0007	1	1
Br_2La	9	0.1376	1	1
Br_2Cu	9	0.1058	1	1
Ca_2Si	9	0.752	1	1
I_2Yb	9	0.1707	1	1
BiClTe	9	0.1498	1	1
BrCdI	9	0.1399	1	1
HgI_2	9	1.1208	1	1
I_2Zn	9	0.1305	1	1
BaF_2	9	0.1428	1	1
BiBrTe	9	0.1553	1	1
S_2W	9	0.111	1	1
GeI_2	9	0.1358	1	1
AsKSn	9	0.1415	1	1
$PbTe_2$	9	0.1391	1	1
I_2Nd	9	0.0016	1	1
Cl_2Cu	9	0.0987	1	1
I_2Tm	9	0.1723	1	1
$SnTe_2$	9	0.1341	1	1
Cl_2V	9	0.1101	1	1
GeI_2	9	0.1481	1	1
I_2Pb	9	0.194	1	1
STl_2	9	0.1436	1	1
$PtSe_2$	9	0.109	1	1
BiITe	9	0.1621	1	1
GeS_2	9	0.2191	1	1
DyI_2	9	0.177	1	1
CeI_2	9	0.0001	1	1
Se_2Yb	9	0.136	1	1
MoS_2	9	0.111	1	1
$BiTe_2$	9	0.1362	1	1
GdI_2	9	0.1582	1	1
$CrSe_2$	9	0.1104	1	1

Table showing the first 50 lattice matching combinations with the smallest number of atoms in the supercell.

Lattice matching - minimal strain

Formula	N° atoms	strain	cell size 1	cell size 2
CeI_2	9	0.0001	1	1
As_2Fe_2	896	0.0001	82	101
GeS_2	786	0.0002	81	100
Mg_2	602	0.0002	65	106
Cl_2Zn	483	0.0002	48	65
Se_2Sn_2	924	0.0003	100	81
Pd_2S_4	630	0.0003	70	35
Ni_2Se_2	10	0.0003	1	1
O_4PSn	510	0.0003	49	36
AuI_4Li	720	0.0005	81	39
Br_2Co	483	0.0005	48	65
Mg_3	171	0.0005	16	25
$Ag_2K_2Se_2$	678	0.0005	64	49
Br_2Fe	483	0.0006	48	65
NS_2Ta	276	0.0006	20	39
$AsSe_2$	840	0.0006	81	118
As_2Fe_2	886	0.0006	81	100
I_2Pr	9	0.0007	1	1
HgO	222	0.0007	25	36
$NbSe_2$	840	0.0008	81	118
In	655	0.0008	85	145
CCl_2Lu_2	613	0.0008	48	65
$Ba_2H_2I_2$	876	0.0008	85	61
Cl_2Cu	852	0.0009	88	108
As_2	418	0.0009	48	65
Au_2I_2	844	0.0009	92	73
Cl_4Mn	341	0.0009	36	25
KS_2Ti	548	0.001	48	65
$As_2Cd_2K_2$	510	0.001	49	36
H_2Na_2Pd	986	0.001	81	100
Se_2Ta	840	0.001	81	118
Br_2Ca_3Si	198	0.001	21	12
$I_2O_2Yb_2$	12	0.0011	1	1
Bi_2Pd	621	0.0011	61	85
Se_2W	852	0.0011	79	126
Pb_2Se_2	292	0.0011	32	25
$NbSe_2$	840	0.0011	81	118
Br_2CsF	754	0.0011	85	61
Tl	600	0.0011	79	126
$MoSe_2$	852	0.0011	79	126
Au_2I_2	834	0.0011	91	72
Tl	670	0.0012	89	136
K	688	0.0012	101	82
$H_2Na_2O_2$	678	0.0013	49	64
$C_2I_2Y_2$	768	0.0013	62	66
Br_2Mn	483	0.0013	48	65
BN	510	0.0014	44	123
$Br_2Cr_2S_2$	504	0.0014	42	42
Ir_2P_2	10	0.0014	1	1
P_4	710	0.0014	71	71

Table showing the first 50 lattice matching combinations with the smallest strain needed to build the supercell.

Fe_2TeSe (P4mm)

Structural and electronic properties

**Formula**	Fe_2TeSe
**Spacegroup**	P4mm
**Prototype**	Fe2SeTe
**Parent 3D**	Fe_2SeTe
**Source DB**	MPDS
**DB ID**	S1930815
**DF2-C09 Binding energy [meV/ Å^2]**	25.44
**RVV10 Binding energy [meV/ Å^2]**	N/A
**Band gap (PBE) [eV]**	0.0

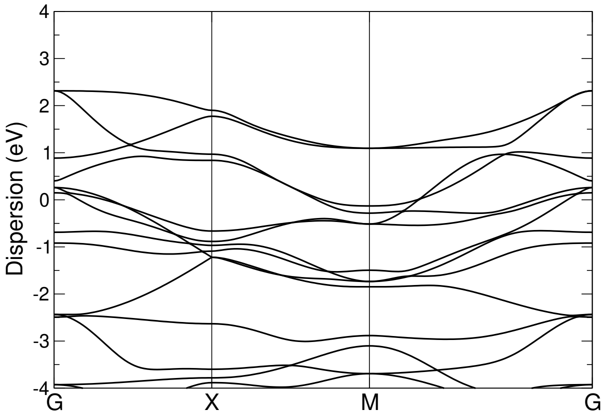

Band structure: Electronic band structure of Fe_2TeSe (P4mm) in a representative window around the Fermi energy and along a high-symmetry path.

Optimized crystal structure

Structural parameters: relaxed cell (top) and atomic positions (bottom) of Fe_2TeSe (P4mm) in Cartesian coordinates.

	x [Å]	y [Å]	z [Å]
$\mathbf{a_1}$	−3.75744305	0.00000000	0.00000000
$\mathbf{a_2}$	0.00000000	−3.75744305	0.00000000
$\mathbf{a_3}$	0.00000000	0.00000000	18.03839114

	x [Å]	y [Å]	z [Å]
Fe	0.00000000	-1.87872153	0.08040608
Fe	-1.87872153	0.00000000	0.08040608
Te	-1.87872153	-1.87872153	-1.56493295
Se	0.00000000	0.00000000	1.40412079

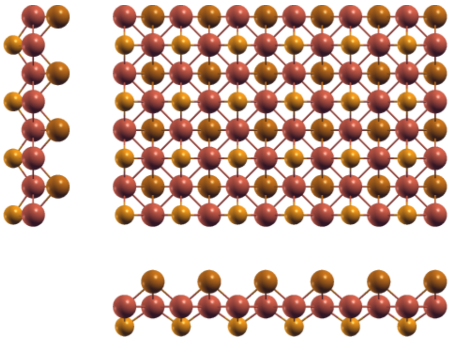

Orthographic projections: views of Fe_2TeSe (P4mm) as seen from the x axis (left), the y axis (bottom) and the z axis (center).

Lattice matching - minimal size

Formula	N° atoms	strain	cell size 1	cell size 2
K	5	1.1234	1	1
K	5	0.7823	1	1
InSe	6	0.1639	1	1
Bi_2	6	0.1704	1	1
AgTl	6	0.3654	1	1
PbTe	6	0.1659	1	1
Sb_2	6	0.1478	1	1
CrS_2	7	0.1113	1	1
I_2Mg	7	0.1532	1	1
CdI_2	7	0.1678	1	1
$MoTe_2$	7	0.1087	1	1
Br_2Ca	7	0.1692	1	1
CaI_2	7	0.1959	1	1
$HfTe_2$	7	0.1344	1	1
Br_2La	7	0.1536	1	1
Br_2Cu	7	0.1156	1	1
I_2Yb	7	0.1925	1	1
BiClTe	7	0.1682	1	1
ReS_2	7	0.1099	1	1
$AuTe_2$	7	0.1407	1	1
BrCdI	7	0.1564	1	1
Cl_2Zn	7	0.0057	1	1
$PdTe_2$	7	0.1387	1	1
I_2Zn	7	0.1448	1	1
BaF_2	7	0.1598	1	1
BiBrTe	7	0.1746	1	1
Bi_2Pd	7	0.2084	1	1
GeI_2	7	0.1514	1	1
Ba_2Hg	7	0.3843	1	1
PtS_2	7	0.1084	1	1
CdClO	7	0.109	1	1
Ba_2N	7	0.1356	1	1
AsKSn	7	0.1583	1	1
Te_2Zr	7	0.1348	1	1
Te_2W	7	0.1087	1	1
$PbTe_2$	7	0.1554	1	1
Cl_2Cu	7	0.1042	1	1
I_2Tm	7	0.1943	1	1
$SnTe_2$	7	0.1492	1	1
GeI_2	7	0.1661	1	1
STl_2	7	0.1608	1	1
OTl_2	7	0.1091	1	1
BiITe	7	0.1825	1	1
Se_2Yb	7	0.1516	1	1
$BiTe_2$	7	0.1519	1	1
GdI_2	7	0.178	1	1
$PtTe_2$	7	0.1404	1	1
Br_2Cd	7	0.1379	1	1
CdI_2	7	0.1673	1	1
Ba_2Cd	7	0.3899	1	1

Table showing the first 50 lattice matching combinations with the smallest number of atoms in the supercell.

Lattice matching - minimal strain

Formula	N° atoms	strain	cell size 1	cell size 2
$AgTe_2$	499	0.0	61	85
F_4Sn	805	0.0001	100	81
Cl_4KTl	292	0.0001	49	16
Se_2Ta_4	934	0.0002	82	101
$H_2I_2Sr_2$	550	0.0002	64	49
La_2S_2	444	0.0003	63	48
$Br_2H_2Yb_2$	10	0.0003	1	1
$As_2Fe_2Li_2$	10	0.0003	1	1
Se_2Ta_4	924	0.0003	81	100
Se_2Ta	387	0.0004	48	65
$C_2Br_2La_2$	114	0.0005	12	11
$NbSe_2$	387	0.0005	48	65
$AsSe_2$	387	0.0006	48	65
LiO_2	294	0.0006	36	50
Ge_2Te_2	872	0.0007	124	94
Cl_4Mn	180	0.0007	25	16
Gd	290	0.0007	46	106
AuI_4Li	702	0.0007	105	47
NbS_2	678	0.0007	81	118
Bi_2Pd	887	0.0008	113	145
$Br_2Lu_2O_2$	10	0.0008	1	1
C_4Ca_2	820	0.0008	91	76
$NbSe_2$	387	0.0008	48	65
Ba_2Hg	643	0.0009	100	81
Se_2Sn_2	400	0.0009	57	43
Cl_2Ni	387	0.0009	48	65
Se_2Sn_2	392	0.0009	56	42
$Ge_2Te_2Zr_2$	10	0.001	1	1
PbS_2Sn	580	0.001	81	64
$LiOS_2Ti$	914	0.001	81	118
$I_2La_2O_2$	896	0.001	101	82
Bi_2In_2	900	0.001	136	89
H_2NiO_2	946	0.001	79	126
$I_2Se_2Tb_2$	180	0.0011	24	14
Mg_3	208	0.0011	25	36
Br_2Ca_3Si	886	0.0012	100	81
$C_2Br_2Y_2$	744	0.0012	72	76
$FeSe_2$	523	0.0012	58	97
C_4Ca_2	712	0.0013	79	66
$AgClO_4$	618	0.0013	81	49
O_4PTl	294	0.0013	36	25
$AgCuTe_2$	440	0.0013	54	56
Sm	307	0.0014	48	115
$Ba_2F_2I_2$	294	0.0014	36	25
S_2Ta	678	0.0014	81	118
$AgClO_2$	744	0.0014	92	94
CoH_2O_2	801	0.0014	64	109
$F_2I_2Pb_2$	718	0.0014	82	65
Br_2Hf_2	452	0.0015	48	65
Cu_2Sr_2	768	0.0015	103	89

Table showing the first 50 lattice matching combinations with the smallest strain needed to build the supercell.

$FeBr_2$ (P-3m1)

Structural and electronic properties

**Formula**	$FeBr_2$
**Spacegroup**	P-3m1
**Prototype**	CdI2
**Parent 3D**	$FeBr_2$
**Source DB**	COD
**DB ID**	9009102
**DF2-C09 Binding energy [meV/ Å^2]**	15.51
**RVV10 Binding energy [meV/ Å^2]**	22.24
**Band gap (PBE) [eV]**	N/A

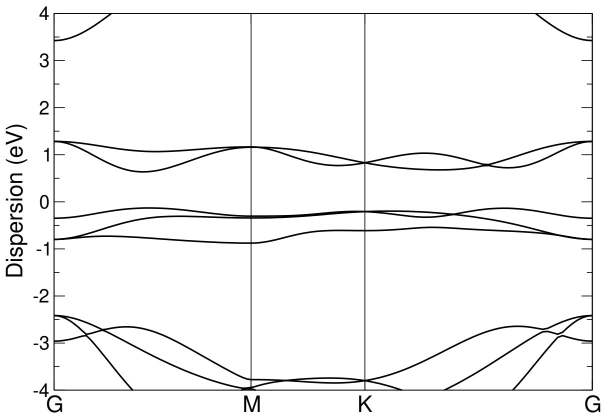

Band structure: Electronic band structure of $FeBr_2$ (P-3m1) in a representative window around the Fermi energy and along a high-symmetry path.

Optimized crystal structure

Structural parameters: relaxed cell (top) and atomic positions (bottom) of $FeBr_2$ (P-3m1) in Cartesian coordinates.

		x [Å]	y [Å]	z [Å]
$\mathbf{a_1}$		3.60591179	0.00000000	0.00000000
$\mathbf{a_2}$		−1.80295589	3.12281121	0.00000000
$\mathbf{a_3}$		0.00000000	0.00000000	22.64926017
		x [Å]	y [Å]	z [Å]
●	Br	-0.00000000	2.08187414	10.02682019
●	Fe	0.00000000	0.00000000	11.32463009
●	Br	1.80295589	1.04093707	12.62243999

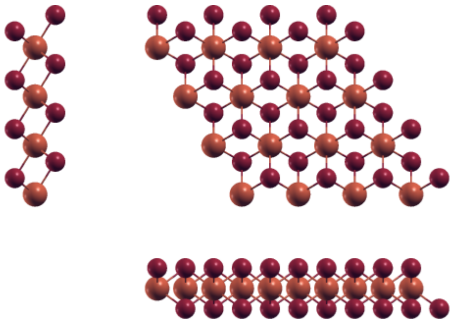

Orthographic projections: views of $FeBr_2$ (P-3m1) as seen from the x axis (left), the y axis (bottom) and the z axis (center).

Lattice matching - minimal size

Formula	N° atoms	strain	cell size 1	cell size 2
K	4	0.3113	1	1
Tl	4	0.1211	1	1
Sn	4	0.4232	1	1
Na	4	0.0075	1	1
In	4	0.4332	1	1
In	4	0.2503	1	1
HgO	5	0.1287	1	1
As_2	5	0.0004	1	1
LiO	5	0.2648	1	1
P_2	5	0.2759	1	1
Mg_2	5	0.1133	1	1
Sb_2	5	2.8442	1	1
Cl_2Zn	6	0.0007	1	1
I_2Mg	6	2.9172	1	1
S_2V	6	0.2546	1	1
MoS_2	6	0.2555	1	1
$MoTe_2$	6	0.0053	1	1
$AgTe_2$	6	0.1228	1	1
PSn_2	6	0.0094	1	1
HfS_2	6	0.0031	1	1
$HfTe_2$	6	0.4506	1	1
Te_2V	6	0.0034	1	1
$CuTe_2$	6	0.0027	1	1
S_2Zr	6	0.0087	1	1
Br_2La	6	2.9216	1	1
Br_2Co	6	0.0001	1	1
ReS_2	6	1.5597	1	1
Ca_2N	6	0.0009	1	1
$AuTe_2$	6	0.4699	1	1
$PdTe_2$	6	0.4637	1	1
Mg_3	6	0.1179	1	1
I_2Zn	6	0.4817	1	1
Te_2Zn	6	0.0054	1	1
S_2W	6	0.2556	1	1
Bi_2Pd	6	0.1358	1	1
GeI_2	6	0.5003	1	1
Br_2Mn	6	0.0019	1	1
PtS_2	6	0.0065	1	1
$CoTe_2$	6	0.0034	1	1
CdClO	6	0.0039	1	1
Ba_2N	6	0.4543	1	1
Se_2Ti	6	0.009	1	1
AsKSn	6	2.9833	1	1
Te_2Zr	6	0.4518	1	1
Te_2W	6	0.0051	1	1
Cl_2Cu	6	0.5809	1	1
S_2Sn	6	0.0089	1	1
$SnTe_2$	6	0.4943	1	1
Cl_2V	6	0.2612	1	1
I_2Pb	6	13.6558	1	1

Table showing the first 50 lattice matching combinations with the smallest number of atoms in the supercell.

Lattice matching - minimal strain

Formula	N° atoms	strain	cell size 1	cell size 2
$Ce_2I_2Si_2$	429	0.0	57	43
$I_2Pr_2S_2$	537	0.0	81	49
MoS_2	390	0.0001	57	73
NS_2Ta	219	0.0001	25	36
Ag_2	257	0.0001	61	37
Br_2Co	6	0.0001	1	1
Br_2PY_2	705	0.0001	100	81
$Dy_2I_2S_2$	171	0.0001	25	16
Br_2Cd	543	0.0001	100	81
S_2W	390	0.0001	57	73
Cl_2La_2	499	0.0001	81	64
BH_4Li	486	0.0001	64	49
$CrSe_2$	435	0.0002	64	81
Br_2Ho_2	565	0.0002	91	73
$Ga_2I_2Y_2$	363	0.0002	49	36
I_2Mg	300	0.0002	57	43
I_2Pr	339	0.0002	65	48
CCl_2Lu_2	8	0.0002	1	1
NaO_4	545	0.0003	100	49
Li_2Tl_2	247	0.0003	49	25
MoS_2	390	0.0003	57	73
FHOZn	516	0.0003	64	81
LiO	401	0.0003	73	91
Ba_2Pt	294	0.0003	61	37
Sb_2Se_2Te	386	0.0003	57	43
BiBrTe	183	0.0003	36	25
As_2	5	0.0004	1	1
$SnTe_2$	339	0.0004	64	49
$Br_2Er_2Se_2$	801	0.0004	135	66
Bi_2S_3	638	0.0004	91	73
BaF_2	255	0.0004	49	36
Cl_4Mn	217	0.0004	39	20
Nd	43	0.0004	9	16
$CeLi_2P_2$	437	0.0005	64	49
$I_2O_2Yb_2$	483	0.0005	65	48
Br_2La	300	0.0006	57	43
$Br_2Eu_2O_2$	483	0.0006	65	48
I_2Zn	390	0.0006	73	57
Bi_2Se_3	386	0.0006	57	43
CeI_2	339	0.0007	65	48
$I_2Pr_2S_2$	405	0.0007	61	37
S_2V	390	0.0007	57	73
Br_2Tb_2	565	0.0007	91	73
$I_2O_2Pr_2$	840	0.0007	118	81
Cl_2Zn	6	0.0007	1	1
CoH_2O_2	353	0.0008	36	49
O_2Pt	339	0.0008	49	64
DyI_2	123	0.0008	25	16
In	211	0.0008	49	64
$PtTe_2$	492	0.0008	91	73

Table showing the first 50 lattice matching combinations with the smallest strain needed to build the supercell.

$FeCl_2$ (P-3m1)

Structural and electronic properties

**Formula**	$FeCl_2$
**Spacegroup**	P-3m1
**Prototype**	CdI2
**Parent 3D**	$FeCl_2$
**Source DB**	COD
**DB ID**	9014952
**DF2-C09 Binding energy [meV/ Å^2]**	9.73
**RVV10 Binding energy [meV/ Å^2]**	15.2
**Band gap (PBE) [eV]**	0.88

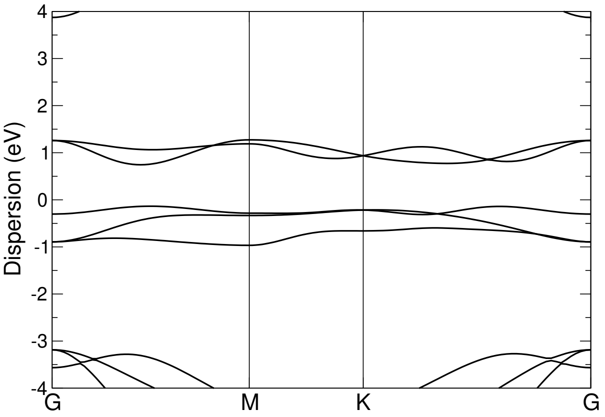

Band structure: Electronic band structure of $FeCl_2$ (P-3m1) in a representative window around the Fermi energy and along a high-symmetry path.

Optimized crystal structure

Structural parameters: relaxed cell (top) and atomic positions (bottom) of $FeCl_2$ (P-3m1) in Cartesian coordinates.

		x [Å]	y [Å]	z [Å]
$\mathbf{a_1}$		3.40451799	0.00000000	0.00000000
$\mathbf{a_2}$		−1.70225900	2.94839907	0.00000000
$\mathbf{a_3}$		0.00000000	0.00000000	22.46050431
		x [Å]	y [Å]	z [Å]
	Cl	1.70225900	0.98279969	12.43768770
	Fe	0.00000000	0.00000000	11.23025215
	Cl	0.00000000	1.96559938	10.02281661

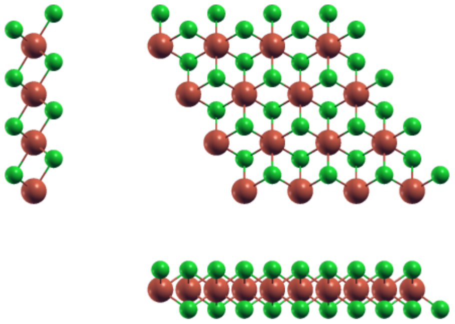

Orthographic projections: views of $FeCl_2$ (P-3m1) as seen from the x axis (left), the y axis (bottom) and the z axis (center).

Lattice matching - minimal size

Formula	N° atoms	strain	cell size 1	cell size 2
Tl	4	0.1402	1	1
Sn	4	0.1183	1	1
In	4	0.1206	1	1
HgO	5	0.1513	1	1
AsSb	5	0.4803	1	1
GeTe	5	0.4962	1	1
S_2	5	0.5004	1	1
Mg_2	5	0.1278	1	1
$IrTe_2$	6	0.4986	1	1
CrS_2	6	0.2674	1	1
$CdCl_2$	6	0.4934	1	1
Cl_2Mn	6	0.0059	1	1
$AgTe_2$	6	0.1428	1	1
$ReSe_2$	6	0.0072	1	1
S_2Ta	6	0.0039	1	1
Br_2Zn	6	0.4545	1	1
$InSe_2$	6	0.4944	1	1
$SiTe_2$	6	0.4648	1	1
$HfTe_2$	6	2.9941	1	1
I_2Mn	6	0.4937	1	1
NSr_2	6	0.4845	1	1
PbS_2	6	0.4737	1	1
FeI_2	6	2.8329	1	1
I_2Ni	6	2.8491	1	1
S_2Ti	6	0.0011	1	1
Mg_3	6	0.1354	1	1
Te_2Ti	6	0.4553	1	1
NbS_2	6	0.0043	1	1
CrI_2	6	0.4867	1	1
Ba_2Hg	6	0.3365	1	1
N_2W	6	0.2578	1	1
Cl_2Ni	6	0.0082	1	1
Cl_2Co	6	0.0007	1	1
NbS_2	6	0.0085	1	1
Br_2V	6	0.0064	1	1
ClNZr	6	0.0043	1	1
Ba_2N	6	3.0146	1	1
Te_2Zr	6	3.0007	1	1
$NiTe_2$	6	0.4633	1	1
Cl_2Cu	6	0.1087	1	1
I_2V	6	0.4682	1	1
Se_2Zr	6	0.4658	1	1
BiITe	6	13.689	1	1
CdO_2	6	0.0006	1	1
$NbSe_2$	6	0.0086	1	1
CoI_2	6	0.4814	1	1
O_2Zn	6	0.2627	1	1
Cl_2Zr	6	0.0004	1	1
$FeSe_2$	6	0.1152	1	1
Se_2Ta	6	0.0087	1	1

Table showing the first 50 lattice matching combinations with the smallest number of atoms in the supercell.

Lattice matching - minimal strain

Formula	N° atoms	strain	cell size 1	cell size 2
ClH_3O	272	0.0	49	25
H_2Si_2	388	0.0001	64	49
CI_2Y_2	504	0.0001	73	57
$Cl_2O_2Tm_2$	786	0.0001	100	81
CrS_2	543	0.0001	81	100
Br_2Ca_3Si	102	0.0001	16	9
FeH_2O_2	597	0.0001	64	81
In_2Se_3	327	0.0002	49	36
$Gd_2I_2S_2$	102	0.0002	16	9
$HfTe_2$	255	0.0002	49	36
Bi_2STe_2	155	0.0002	25	16
CaClHO	624	0.0002	100	81
Se_2Sn	390	0.0003	73	57
Te_2Zr	255	0.0003	49	36
GeTe	290	0.0003	64	49
Cu_2Se_2	387	0.0003	65	48
S_2Zn_2	343	0.0003	57	43
$AlLiTe_2$	439	0.0003	81	49
I_2Ti	390	0.0003	73	57
$F_2Lu_2Se_2$	258	0.0004	36	25
Cl_2Zr	6	0.0004	1	1
$InSe_2$	339	0.0004	64	49
Se_2Zr	492	0.0004	91	73
$Er_2F_2Se_2$	363	0.0004	49	36
NS_2Ta	463	0.0005	57	73
I_2V	492	0.0005	91	73
In_2S_3	437	0.0005	64	49
$I_2S_2Tl_2$	483	0.0005	65	48
NSr_2	390	0.0005	73	57
$F_2Se_2Yb_2$	429	0.0005	57	43
$Cl_2Er_2H_2$	486	0.0005	64	49
Cl_2ORu	635	0.0006	105	80
CdO_2	6	0.0006	1	1
PbTe	107	0.0006	25	16
$Cl_2Er_2O_2$	711	0.0006	91	73
Cl_2Gd_2	343	0.0006	57	43
$F_2Se_2Y_2$	471	0.0006	79	39
As_2CeLi_2	155	0.0006	25	16
I_2Mn	339	0.0006	64	49
P_2Sn_2	343	0.0007	57	43
CoI_2	390	0.0007	73	57
$Br_2Er_2O_2$	840	0.0007	118	81
F_2Na	543	0.0007	100	81
Cl_2Co	6	0.0007	1	1
$Cl_2O_2Yb_2$	786	0.0008	100	81
$CdCl_2$	339	0.0008	64	49
F_2Ni	339	0.0008	65	48
N_3W_2	353	0.0008	36	49
GeI_2	123	0.0008	25	16
$Si_2Te_2Zr_2$	483	0.0008	65	48

Table showing the first 50 lattice matching combinations with the smallest strain needed to build the supercell.

FeI_2 (P-3m1)

Structural and electronic properties

**Formula**	FeI_2
**Spacegroup**	P-3m1
**Prototype**	CdI2
**Parent 3D**	FeI_2
**Source DB**	COD
**DB ID**	9009103
**DF2-C09 Binding energy [meV/ Å^2]**	16.89
**RVV10 Binding energy [meV/ Å^2]**	23.26
**Band gap (PBE) [eV]**	N/A

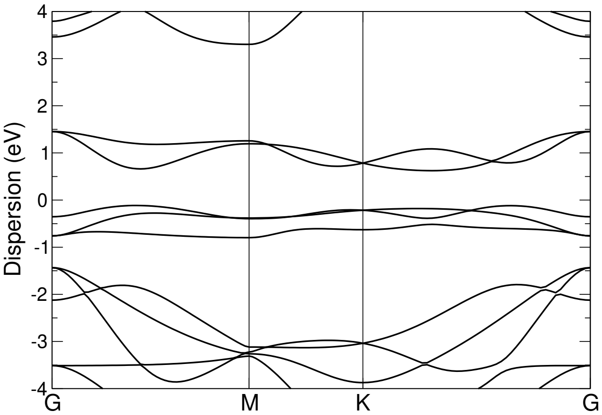

Band structure: Electronic band structure of FeI_2 (P-3m1) in a representative window around the Fermi energy and along a high-symmetry path.

Optimized crystal structure

Structural parameters: relaxed cell (top) and atomic positions (bottom) of FeI_2 (P-3m1) in Cartesian coordinates.

	x [Å]	y [Å]	z [Å]
$\mathbf{a_1}$	3.86738810	0.00000000	0.00000000
$\mathbf{a_2}$	−1.93369405	3.34925634	0.00000000
$\mathbf{a_3}$	0.00000000	0.00000000	22.81337588

	x [Å]	y [Å]	z [Å]
I	1.93369405	1.11641878	10.02692224
Fe	0.00000000	0.00000000	11.40668794
I	0.00000000	2.23283756	12.78645364

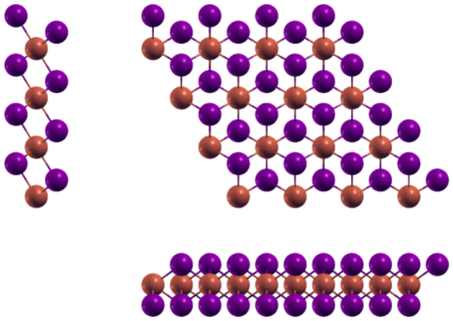

Orthographic projections: views of FeI_2 (P-3m1) as seen from the x axis (left), the y axis (bottom) and the z axis (center).

Lattice matching - minimal size

Formula	N° atoms	strain	cell size 1	cell size 2
Tl	4	0.4217	1	1
Tl	4	1.5532	1	1
HgO	5	0.1116	1	1
AsSb	5	0.0029	1	1
Bi_2	5	0.4613	1	1
GeTe	5	0.0032	1	1
S_2	5	0.0048	1	1
PbTe	5	0.4513	1	1
CaCl	5	0.141	1	1
$IrTe_2$	6	0.0041	1	1
$CdCl_2$	6	0.0021	1	1
Cl_2Mn	6	1.5951	1	1
CdI_2	6	0.4556	1	1
$AgTe_2$	6	0.4284	1	1
$MoSe_2$	6	1.5478	1	1
$ReSe_2$	6	0.2629	1	1
S_2Ta	6	0.2479	1	1
Br_2Ca	6	0.4586	1	1
CaI_2	6	2.963	1	1
$InSe_2$	6	0.0025	1	1
$GeTe_2$	6	0.0011	1	1
$SiTe_2$	6	0.0088	1	1
I_2Mn	6	0.0022	1	1
NSr_2	6	0.0013	1	1
I_2Yb	6	2.9298	1	1
PbS_2	6	0.0054	1	1
BiClTe	6	0.4564	1	1
LiO_2	6	0.0662	1	1
Cl_2Zn	6	0.1544	1	1
I_2Ni	6	0.0013	1	1
S_2Ti	6	0.2545	1	1
NbS_2	6	0.2473	1	1
CrI_2	6	0.0004	1	1
BiBrTe	6	0.4703	1	1
Bi_2Pd	6	0.1153	1	1
Cl_2Ni	6	0.2642	1	1
Cl_2Co	6	0.254	1	1
$CrTe_2$	6	0.273	1	1
Br_2V	6	0.2617	1	1
ClNZr	6	0.2588	1	1
Cl_2Fe	6	1.6399	1	1
Se_2V	6	1.5594	1	1
Br_2Ti	6	0.2721	1	1
$AsSe_2$	6	0.2662	1	1
I_2V	6	0.0075	1	1
Cl_2V	6	1.4458	1	1
GeI_2	6	0.4519	1	1
Se_2Zr	6	0.0084	1	1
BiITe	6	0.4872	1	1
CdO_2	6	0.2538	1	1

Table showing the first 50 lattice matching combinations with the smallest number of atoms in the supercell.

Lattice matching - minimal strain

Formula	N° atoms	strain	cell size 1	cell size 2
$CrSe_2$	183	0.0	25	36
Bi_2	462	0.0	100	81
Br_2Hf_2	643	0.0	81	100
Nd	247	0.0	49	100
Cu_4Te_2	711	0.0001	91	73
C	129	0.0001	16	81
Cl_2Ni	492	0.0001	73	91
Cd_2I_3	437	0.0001	64	49
$MoSe_2$	255	0.0001	36	49
Br_2Mg	6	0.0001	1	1
MnO_2	222	0.0001	25	49
AsI_2La_2	437	0.0002	64	49
Cl_2Hf_2	403	0.0002	49	64
$Br_2H_2Zr_2$	678	0.0002	64	81
NaO_4	93	0.0002	16	9
Hg_3N_2	368	0.0002	81	25
CoH_2O_2	173	0.0003	16	25
DyI_2	255	0.0003	49	36
Se_2W	255	0.0003	36	49
I_2La_2P	563	0.0003	81	64
$NbSe_2$	492	0.0003	73	91
ClNZr	435	0.0004	64	81
Cl_2Mn	300	0.0004	43	57
$AlLiTe_2$	447	0.0004	73	57
Br_2Ca_3Si	483	0.0004	65	48
BrNZr	543	0.0004	81	100
CrI_2	6	0.0004	1	1
$Dy_2I_2S_2$	363	0.0004	49	36
$Ga_2Gd_2I_2$	786	0.0004	100	81
Se_2Ta	492	0.0004	73	91
I_2Pb	183	0.0004	36	25
NS_2Ta	471	0.0004	49	81
$I_2Pr_2S_2$	258	0.0005	36	25
Br_3Cs	211	0.0005	49	16
Sb_2Te_3	638	0.0005	91	73
CCl_2Sc_2	597	0.0006	64	81
Ba_2Hg	339	0.0006	65	48
$AgNO_3$	93	0.0006	16	9
Bi_2SeTe_2	705	0.0006	100	81
GdI_2	435	0.0006	81	64
In	361	0.0006	81	118
$NbSe_2$	543	0.0007	81	100
$Ga_2I_2Tb_2$	786	0.0007	100	81
S_2Ti	390	0.0007	57	73
N_4	212	0.0007	24	35
Ca_4Cu_2	624	0.0007	102	53
$I_2S_2Tb_2$	363	0.0007	49	36
O_2Sn_2	357	0.0008	55	48
Cl_2NSc_2	414	0.0008	43	57
$Er_2I_2Se_2$	345	0.0009	55	30

Table showing the first 50 lattice matching combinations with the smallest strain needed to build the supercell.

FeO_2 (P-3m1)

Structural and electronic properties

**Formula**	FeO_2
**Spacegroup**	P-3m1
**Prototype**	CdI2
**Parent 3D**	FeO_2
**Source DB**	COD
**DB ID**	9009104
**DF2-C09 Binding energy [meV/ Å^2]**	18.96
**RVV10 Binding energy [meV/ Å^2]**	N/A
**Band gap (PBE) [eV]**	0.0

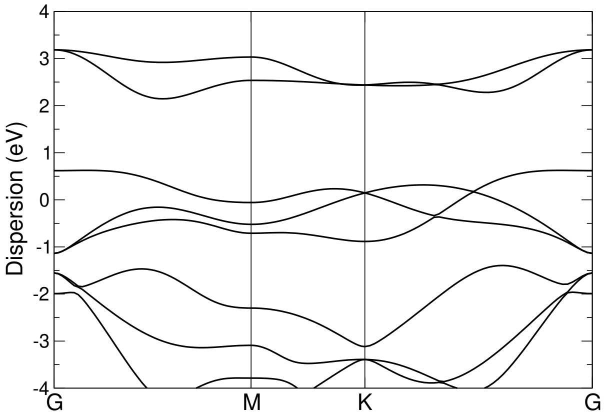

Band structure: Electronic band structure of FeO_2 (P-3m1) in a representative window around the Fermi energy and along a high-symmetry path.

Optimized crystal structure

Structural parameters: relaxed cell (top) and atomic positions (bottom) of FeO_2 (P-3m1) in Cartesian coordinates.

		x [Å]	y [Å]	z [Å]
$\mathbf{a_1}$		1.40825048	−2.43916139	0.00000000
$\mathbf{a_2}$		1.40825048	2.43916139	0.00000000
$\mathbf{a_3}$		0.00000000	0.00000000	15.75736792
		x [Å]	y [Å]	z [Å]
•	O	1.40825048	-0.81305380	-0.93799490
•	Fe	0.00000000	0.00000000	0.00000000
•	O	1.40825048	0.81305380	0.93799490

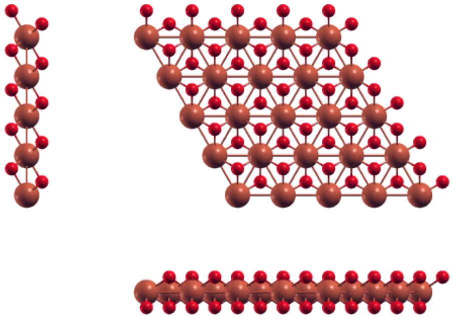

Orthographic projections: views of FeO_2 (P-3m1) as seen from the x axis (left), the y axis (bottom) and the z axis (center).

Lattice matching - minimal size

Formula	N° atoms	strain	cell size 1	cell size 2
In	4	0.4729	1	1
LiO	5	0.5004	1	1
BN	5	0.2608	1	1
C_2	5	0.2496	1	1
S_2V	6	0.4811	1	1
MoS_2	6	0.483	1	1
$CuTe_2$	6	13.6471	1	1
NiO_2	6	0.0013	1	1
ReS_2	6	0.4511	1	1
MnO_2	6	0.0091	1	1
S_2W	6	0.4831	1	1
Cl_2V	6	0.4937	1	1
MoS_2	6	0.4835	1	1
$CrSe_2$	6	0.4971	1	1
O_2Pt	6	0.469	1	1
N_2Re	6	0.0074	1	1
CoO_2	6	0.0008	1	1
Cl_2Hf_2	7	3.1902	1	1
O_2Sn_2	7	1.3901	1	1
CoH_2O_2	8	0.4486	1	1
H_2NiO_2	8	0.4866	1	1
H_2MgO_2	8	0.4788	1	1
$HfTe_2$	9	0.2203	2	1
CNRb	9	0.3	2	1
Ba_2N	9	0.2222	2	1
Te_2Zr	9	0.2209	2	1
Cl_2Cu	9	0.1203	2	1
Br_2Cd	9	0.226	2	1
NaPSn	9	0.2191	2	1
K	10	0.1235	3	1
$AgNO_2$	10	0.0676	2	1
Cu_2O_2	10	0.8144	2	1
$AgCuTe_2$	10	0.5237	2	1
Bi_2Se_2	10	1.934	2	1
Au_2I_2	10	0.6623	2	1
Ho_2S_2	10	0.3039	2	1
Pt_2Te_2	10	0.2193	2	1
Bi_2Te_2	11	6.1362	1	2
Br_2PY_2	11	0.2257	2	1
In_2Se_3	11	0.2203	2	1
$CuGeO_3$	11	0.8295	2	1
HgI_2	12	0.1355	3	1
Ba_2Hg	12	0.1156	3	1
CKN	12	0.1619	3	1
Cu_2O_4	12	0.4929	2	1
Cu_3Se_3	12	0.2219	2	1
$F_2Ho_2Se_2$	12	0.2243	2	1
$Er_2F_2Se_2$	12	0.2199	2	1
Ba_2Cd	12	0.1168	3	1
Bi_2In_2	13	0.1497	3	1

Table showing the first 50 lattice matching combinations with the smallest number of atoms in the supercell.

Lattice matching - minimal strain

Formula	N° atoms	strain	cell size 1	cell size 2
CS_2Ta_2	327	0.0	49	36
S_2W	390	0.0	73	57
KNO_3	192	0.0	49	9
$Cl_2N_2Zr_2$	537	0.0	81	49
CBr_2Y_2	93	0.0001	16	9
Cl_2Ti	255	0.0001	49	36
MoS_2	390	0.0001	73	57
Br_2Ho_2	496	0.0001	100	49
MoS_2	390	0.0001	73	57
Bi_2S_3	545	0.0001	100	49
Br_2Cd	354	0.0001	79	39
$Br_2Hf_2N_2$	405	0.0001	61	37
$Dy_2I_2S_2$	342	0.0002	64	25
$F_2Se_2Tm_2$	297	0.0002	49	25
P_2	219	0.0002	49	36
CaH_2O_2	488	0.0003	81	49
C_2	275	0.0003	49	64
Br_2PY_2	432	0.0003	79	39
$LiMnSe_2$	247	0.0004	49	25
$LiNbS_2$	208	0.0004	36	25
Br_2Tb_2	496	0.0004	100	49
$Hf_2Se_2Si_2$	411	0.0004	65	36
S_2Ta	183	0.0005	36	25
Cl_2Y_2	84	0.0005	16	9
Ca_2N	294	0.0005	61	37
In_2Te_3	47	0.0005	9	4
$Fe_2Li_2P_2$	237	0.0005	39	20
Sb_2SeTe_2	47	0.0005	9	4
La_2S_2	121	0.0006	27	10
$CrSe_2$	339	0.0006	64	49
STl_2	39	0.0006	9	4
Cl_2V	339	0.0006	64	49
CCl_2Gd_2	93	0.0006	16	9
$Cl_2N_2Zr_2$	405	0.0007	61	37
$CuTe_2$	390	0.0007	81	49
CNb_2S_2	327	0.0007	49	36
Cl_2Hf_2	208	0.0007	36	25
CoO_2	6	0.0008	1	1
Br_2Zn	75	0.0008	16	9
$PdTe_2$	354	0.0008	79	39
O_2Pt	492	0.0008	91	73
S_2V	390	0.0008	73	57
$I_2Se_2Yb_2$	228	0.0008	48	14
$I_2Se_2Tm_2$	228	0.0009	48	14
DyI_2	267	0.0009	64	25
$Hf_2Si_2Te_2$	237	0.0009	39	20
Mg_2	291	0.0009	65	48
NbS_2	183	0.0009	36	25
Bi_2Se_2Te	47	0.0009	9	4
As_2Fe_2	339	0.0009	65	36

Table showing the first 50 lattice matching combinations with the smallest strain needed to build the supercell.

FeO_2 (Pmmn)

Structural and electronic properties

**Formula**	FeO_2
**Spacegroup**	Pmmn
**Prototype**	FeO2
**Parent 3D**	Fe_2O_4
**Source DB**	COD
**DB ID**	9015156
**DF2-C09 Binding energy [meV/ Å^2]**	16.3
**RVV10 Binding energy [meV/ Å^2]**	29.81
**Band gap (PBE) [eV]**	N/A

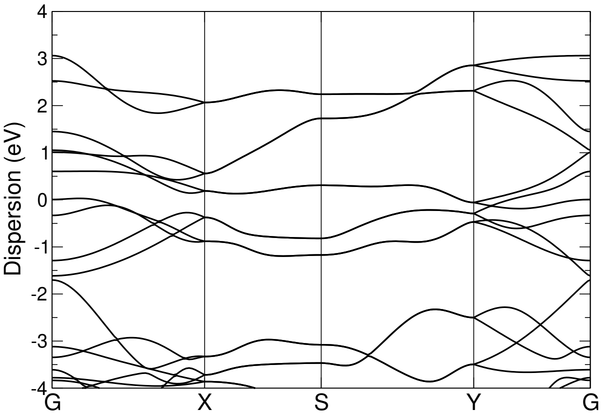

Band structure: Electronic band structure of FeO_2 (Pmmn) in a representative window around the Fermi energy and along a high-symmetry path.

Optimized crystal structure

Structural parameters: relaxed cell (top) and atomic positions (bottom) of FeO_2 (Pmmn) in Cartesian coordinates.

	x [Å]	y [Å]	z [Å]
$\mathbf{a_1}$	2.81565578	0.00000000	0.00000000
$\mathbf{a_2}$	0.00000000	3.68453870	0.00000000
$\mathbf{a_3}$	0.00000000	0.00000000	24.00362860

	x [Å]	y [Å]	z [Å]
Fe	1.40782789	0.92113551	11.01485776
O	1.40782789	2.76340542	11.52339117
O	1.40782789	2.76340645	14.02077597
Fe	0.00000000	2.76340590	12.98877084
O	0.00000000	0.92113599	12.48023743
O	0.00000000	0.92113495	9.98285263

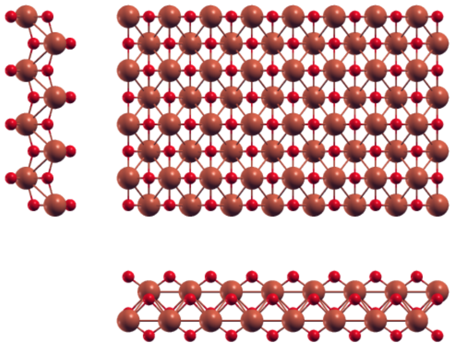

Orthographic projections: views of FeO_2 (Pmmn) as seen from the x axis (left), the y axis (bottom) and the z axis (center).

Lattice matching - minimal size

Formula	N° atoms	strain	cell size 1	cell size 2
InSe	8	0.2998	1	1
Nd	8	0.6798	1	2
HgO	8	0.0665	1	1
Bi_2	8	0.311	1	1
PbTe	8	0.3032	1	1
CdI_2	9	0.3065	1	1
$AgTe_2$	9	0.3494	1	1
Br_2Ca	9	0.3089	1	1
Br_2La	9	0.2814	1	1
BiClTe	9	0.3072	1	1
BrCdI	9	0.2865	1	1
LiO_2	9	0.1052	1	1
Cl_2Zn	9	0.9319	1	1
BaF_2	9	0.8025	1	1
BiBrTe	9	0.3181	1	1
Bi_2Pd	9	0.3871	1	1
AsKSn	9	0.7968	1	1
$PbTe_2$	9	0.2847	1	1
GeI_2	9	0.3037	1	1
STl_2	9	0.8064	1	1
$FeSe_2$	9	0.2779	1	1
GdI_2	9	0.3238	1	1
F_2Ni	9	0.3691	1	1
CdI_2	9	0.3057	1	1
I_2Pr	9	0.3074	1	1
Cu_2Sr_2	10	0.3201	1	1
Cl_2OOs	10	0.3565	1	1
$LiMnTe_2$	10	0.3042	1	1
Cu_2Te_2	10	0.296	1	1
$AgCuTe_2$	10	0.5874	1	1
$AsLi_3$	10	0.3004	1	1
Cl_2OV	10	0.2787	1	1
Fe_2Se_2	10	0.3679	1	1
Cl_2ORu	10	0.3448	1	1
As_2Co_2	10	0.3577	1	1
$AgBrO_2$	10	0.2289	1	1
$MnNaTe_2$	10	0.2858	1	1
Cu_2I_2	10	0.2825	1	1
Fe_2S_2	10	0.3436	1	1
Cu_2Se_2	10	0.3703	1	1
$AgClO_2$	10	0.2121	1	1
Gd_2I_2	10	0.282	1	1
Co_2Se_2	10	0.3605	1	1
Ca_2Cl_2	10	0.3563	1	1
SSb_2Te_2	11	0.2864	1	1
As_2Li_2Nd	11	0.2986	1	1
Bi_2STe_2	11	0.3016	1	1
Hf_3Te_2	11	0.9271	1	1
As_2CeLi_2	11	0.3033	1	1
H_2Li_2Pt	11	0.3465	1	1

Table showing the first 50 lattice matching combinations with the smallest number of atoms in the supercell.

Lattice matching - minimal strain

Formula	N° atoms	strain	cell size 1	cell size 2
Ge_2Se_2	414	0.0004	49	30
$Br_2N_2Zr_2$	822	0.0005	81	56
$C_2Br_2La_2$	402	0.0005	40	27
$HNiO_2$	766	0.0005	67	91
$AgCuTe_2$	580	0.0006	64	49
Tl	380	0.0007	54	56
Mg_4	548	0.0008	54	56
$I_2N_2Zr_2$	360	0.0008	36	24
PSn_2	831	0.0009	96	85
HgO	892	0.0009	112	110
AgTl	458	0.001	63	40
C_2Li_2	950	0.001	109	74
O_2Zn	693	0.0011	70	91
S_2Sn	831	0.0011	96	85
S_2Zr	831	0.0013	96	85
$F_2I_2Pb_2$	228	0.0013	24	14
C_4Ca_2	618	0.0013	64	39
C_2	518	0.0014	52	103
$AgTe_2$	996	0.0014	110	112
FeH_2O_2	875	0.0014	70	91
$NbTe_2$	831	0.0014	96	85
Ba_2Cd	186	0.0014	24	14
$Al_2Cl_2O_2$	102	0.0015	9	8
$Br_2S_2Yb_2$	894	0.0015	100	49
As_4	814	0.0015	97	58
Br_2Fe	780	0.0016	89	82
Br_2Co	780	0.0016	89	82
Tl	581	0.0016	82	89
CCl_2Lu_2	944	0.0017	89	82
As_2	698	0.0017	89	82
C_4Ca_2	582	0.0017	60	37
As_2Li_2Nd	56	0.0017	6	4
Br_2Zr_2	246	0.0017	25	24
CNRb	147	0.0017	20	9
GeI_2Y_2	56	0.0017	6	4
NiO_2	42	0.0017	4	6
Se_2W	843	0.0018	91	99
Cl_2Zn	780	0.0018	89	82
Br_2Pr_2	300	0.0018	32	27
S_2V	258	0.0018	27	32
S_2	720	0.0018	95	75
Se_2V	759	0.0018	82	89
Br_3Cs	676	0.0018	96	25
O_2Zn	642	0.0018	65	84
$MoSe_2$	843	0.0018	91	99
H_2MgO_2	322	0.0018	27	32
$HfSe_2$	273	0.0018	32	27
Se_2W	759	0.0018	82	89
Te_2Ti	273	0.0018	32	27
Se_2Ti	222	0.0018	25	24

Table showing the first 50 lattice matching combinations with the smallest strain needed to build the supercell.

FeOCl (Pmmn)

Structural and electronic properties

**Formula**	FeOCl
**Spacegroup**	Pmmn
**Prototype**	FeOCl
**Parent 3D**	$Fe_2O_2Cl_2$
**Source DB**	COD
**DB ID**	1010645
**DF2-C09 Binding energy [meV/ Å^2]**	14.17
**RVV10 Binding energy [meV/ Å^2]**	22.8
**Band gap (PBE) [eV]**	N/A

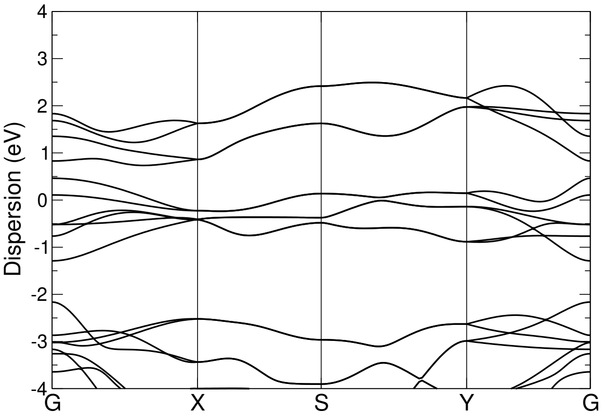

Band structure: Electronic band structure of FeOCl (Pmmn) in a representative window around the Fermi energy and along a high-symmetry path.

Optimized crystal structure

Structural parameters: relaxed cell (top) and atomic positions (bottom) of FeOCl (Pmmn) in Cartesian coordinates.

		x [Å]	y [Å]	z [Å]
$\mathbf{a_1}$		3.15863862	0.00000000	0.00000000
$\mathbf{a_2}$		0.00000000	3.71638917	0.00000000
$\mathbf{a_3}$		0.00000000	0.00000000	24.81404145
		x [Å]	y [Å]	z [Å]
●	Fe	0.00000000	1.85819458	11.59487608
●	Cl	1.57931931	1.85819458	10.02721996
●	O	0.00000000	0.00000000	11.98502132
●	Fe	1.57931931	0.00000000	13.21916713
●	Cl	0.00000000	0.00000000	14.78682016
●	O	1.57931931	1.85819458	12.82901756

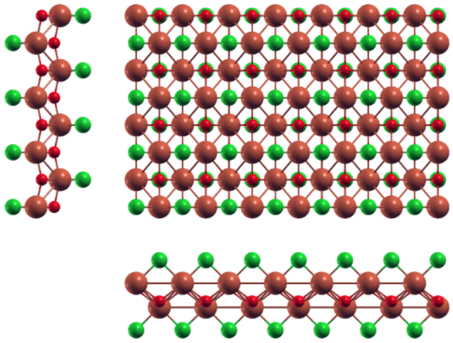

Orthographic projections: views of FeOCl (Pmmn) as seen from the x axis (left), the y axis (bottom) and the z axis (center).

Lattice matching - minimal size

Formula	N° atoms	strain	cell size 1	cell size 2
Tl	7	0.0459	1	1
HgO	8	0.2841	1	1
AsSb	8	0.5831	1	1
Bi_2	8	0.2401	1	1
CaCl	8	0.3026	1	1
CdI_2	9	0.2365	1	1
$AgTe_2$	9	0.0445	1	1
Br_2Zn	9	0.553	1	1
Br_2Ca	9	0.2384	1	1
BiClTe	9	0.237	1	1
LiO_2	9	0.092	1	1
Cl_2Zn	9	0.0635	1	1
Mg_3	9	0.2573	1	1
Te_2Ti	9	0.5539	1	1
BiBrTe	9	0.2459	1	1
Bi_2Pd	9	0.2993	1	1
BiITe	9	0.2568	1	1
CoI_2	9	0.5844	1	1
GeS_2	9	0.2823	1	1
$MnSe_2$	9	0.3024	1	1
GdI_2	9	0.2506	1	1
CNNa	9	1.777	1	1
F_2Ni	9	0.0597	1	1
CdI_2	9	0.2358	1	1
I_2Pr	9	0.2372	1	1
$HfSe_2$	9	0.5539	1	1
Cu_2Sr_2	10	0.2476	1	1
Cl_2OOs	10	0.0521	1	1
$AgCuTe_2$	10	0.4699	1	1
O_2Sn_2	10	0.3362	1	1
Cl_2Y_2	10	0.5495	1	1
Ca_2O_2	10	0.3015	1	1
$AlLiTe_2$	10	0.2549	1	1
Fe_2Se_2	10	0.0592	1	1
Cl_2ORu	10	0.0467	1	1
As_2Co_2	10	0.3114	1	1
Cu_2Te_2	10	0.0661	1	1
$AgBrO_2$	10	0.1669	1	1
Ge_2S_2	10	1.2087	1	1
C_2Li_2	10	0.2745	1	1
Mg_4	10	0.0461	1	1
Fe_2S_2	10	0.299	1	1
Co_2S_2	10	0.3037	1	1
As_2Fe_2	10	0.2836	1	1
O_2Sn_2	10	0.9004	1	1
Cu_2Se_2	10	0.0602	1	1
$AgClO_2$	10	0.1546	1	1
Fe_2SeTe	10	0.0693	1	1
O_2Sn_2	10	0.2294	1	1
Co_2Se_2	10	0.3138	1	1

Table showing the first 50 lattice matching combinations with the smallest number of atoms in the supercell.

Lattice matching - minimal strain

Formula	N° atoms	strain	cell size 1	cell size 2
AsSb	940	0.0004	120	110
MnO_2	714	0.0004	63	112
$Dy_2I_2S_2$	366	0.0006	40	21
Au_2I_2	608	0.0008	72	44
Cl_2OOs	76	0.0008	8	7
$Br_2H_2Zr_2$	618	0.0009	48	55
C	127	0.0009	12	55
$I_2Se_2Yb_2$	108	0.0009	12	6
Ga_2Se_2	784	0.0009	82	73
$IrTe_2$	711	0.0009	82	73
CCl_2Sc_2	563	0.0009	48	55
CNNa	987	0.0009	122	85
$AgNO_3$	923	0.001	108	55
NaO_4	923	0.001	108	55
N_2W	765	0.001	73	109
S_2	638	0.001	82	73
ClNZr	453	0.0011	48	55
NS_2Ta	792	0.0011	66	99
$Br_2N_2Zr_2$	858	0.0011	80	63
Ni_2SbTe_2	857	0.0011	82	73
Cl_2Zr_2	508	0.0011	48	55
$Br_2Er_2S_2$	834	0.0012	90	49
In_2Se_2	218	0.0012	27	14
In_2S_3	857	0.0012	82	73
N_2Re	219	0.0013	20	33
C_4Ca_2	906	0.0013	89	62
Ge_2Te_2	204	0.0014	24	15
GeTe	638	0.0014	82	73
$Ho_2I_2S_2$	366	0.0014	40	21
Te_2Zn	414	0.0015	45	48
$MoTe_2$	414	0.0015	45	48
$Cl_2Er_2S_2$	906	0.0015	97	54
Te_2W	414	0.0015	45	48
Cl_2Mn	834	0.0015	87	104
NbS_2	804	0.0015	84	100
Ca_4Cu_2	282	0.0015	32	15
$HfLiS_2$	462	0.0015	45	48
I_2V	423	0.0015	48	45
$Br_2Hf_2N_2$	972	0.0016	90	72
S_2Ta	804	0.0016	84	100
$LiNbS_2$	904	0.0016	84	100
Se_2Zr	423	0.0016	48	45
S_2Sn_2	592	0.0016	68	46
Ge_2S_2	820	0.0016	92	67
CdH_2O_2	510	0.0016	45	48
$Cl_2Ho_2O_2$	558	0.0017	48	45
$Cl_2Er_2O_2$	558	0.0017	48	45
$I_2Se_2Tm_2$	108	0.0017	12	6
H_2Si_2	738	0.0017	77	69
$InSe_2$	669	0.0017	77	69

Table showing the first 50 lattice matching combinations with the smallest strain needed to build the supercell.

FeS (P4/nmm)

Structural and electronic properties

**Formula**	FeS
**Spacegroup**	P4/nmm
**Prototype**	FeSe
**Parent 3D**	Fe_2S_2
**Source DB**	COD
**DB ID**	9011800
**DF2-C09 Binding energy [meV/ Å^2]**	23.28
**RVV10 Binding energy [meV/ Å^2]**	29.4
**Band gap (PBE) [eV]**	N/A

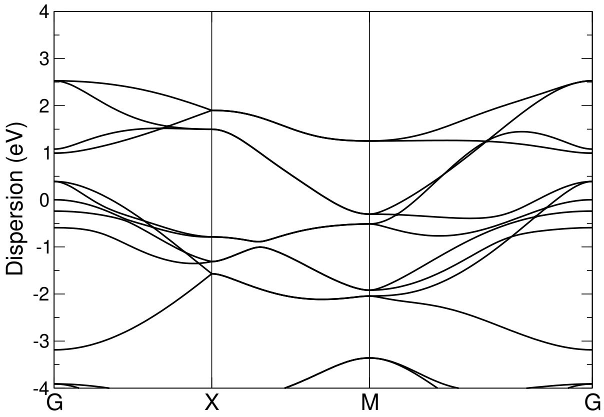

Band structure: Electronic band structure of FeS (P4/nmm) in a representative window around the Fermi energy and along a high-symmetry path.

Optimized crystal structure

Structural parameters: relaxed cell (top) and atomic positions (bottom) of FeS (P4/nmm) in Cartesian coordinates.

	x [Å]	y [Å]	z [Å]
$\mathbf{a_1}$	3.58435132	0.00000000	0.00000000
$\mathbf{a_2}$	0.00000000	3.58435132	0.00000000
$\mathbf{a_3}$	0.00000000	0.00000000	22.47169967

		x [Å]	y [Å]	z [Å]
●	S	0.00000000	1.79217566	12.45947983
●	Fe	0.00000000	0.00000000	11.23584984
●	Fe	1.79217566	1.79217566	11.23584984
●	S	1.79217566	0.00000000	10.01221984

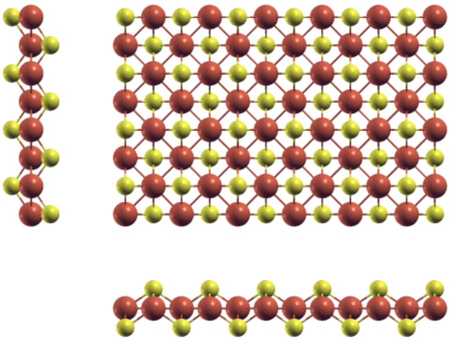

Orthographic projections: views of FeS (P4/nmm) as seen from the x axis (left), the y axis (bottom) and the z axis (center).

Lattice matching - minimal size

Formula	N° atoms	strain	cell size 1	cell size 2
Tl	5	0.209	1	1
InSe	6	0.1906	1	1
AsSb	6	0.1401	1	1
Bi_2	6	0.7539	1	1
GeTe	6	0.1454	1	1
AgTl	6	0.4137	1	1
S_2	6	0.1468	1	1
PbTe	6	0.1929	1	1
Mg_2	6	0.5588	1	1
Sb_2	6	0.171	1	1
CaCl	6	0.0021	1	1
$IrTe_2$	7	0.1462	1	1
I_2Mg	7	0.1777	1	1
$CdCl_2$	7	0.1444	1	1
CdI_2	7	0.1952	1	1
$AgTe_2$	7	0.2123	1	1
Br_2Zn	7	0.132	1	1
Br_2Ca	7	0.1969	1	1
$InSe_2$	7	0.1447	1	1
$GeTe_2$	7	0.1435	1	1
$SiTe_2$	7	0.1351	1	1
$HfTe_2$	7	0.1542	1	1
I_2Mn	7	0.1445	1	1
Br_2La	7	0.1781	1	1
NSr_2	7	0.1414	1	1
PbS_2	7	0.1379	1	1
BiClTe	7	0.1957	1	1
$AuTe_2$	7	0.1622	1	1
BrCdI	7	0.1815	1	1
LiO_2	7	0.353	1	1
$PdTe_2$	7	0.1596	1	1
FeI_2	7	0.1426	1	1
I_2Ni	7	0.1437	1	1
S_2Ti	7	0.1091	1	1
Mg_3	7	0.5893	1	1
Te_2Ti	7	0.1322	1	1
CrI_2	7	0.1421	1	1
I_2Zn	7	0.1673	1	1
BaF_2	7	0.1857	1	1
BiBrTe	7	0.7676	1	1
GeI_2	7	0.1755	1	1
Ba_2Hg	7	1.1633	1	1
Cl_2Co	7	0.109	1	1
Cl_2Fe	7	0.1088	1	1
Ba_2N	7	0.1557	1	1
AsKSn	7	0.1839	1	1
Te_2Zr	7	0.1547	1	1
$PbTe_2$	7	0.1803	1	1
$NiTe_2$	7	0.1346	1	1
$SnTe_2$	7	0.1728	1	1

Table showing the first 50 lattice matching combinations with the smallest number of atoms in the supercell.

Lattice matching - minimal strain

Formula	N° atoms	strain	cell size 1	cell size 2
As_2Ir_2	732	0.0	101	82
$F_2I_2Tm_2$	718	0.0	82	65
H_4Ti	389	0.0001	36	49
Bi_2In_2	620	0.0001	97	58
$Cu_2Na_2Se_2$	718	0.0001	82	65
$Bi_2I_2O_2$	718	0.0001	82	65
$Br_2H_2Sr_2$	550	0.0001	64	49
Ba_2Hg	304	0.0001	49	36
Tl	597	0.0001	113	145
$Cl_2F_2Pb_2$	718	0.0002	82	65
Cl_2Rb_2	424	0.0002	81	25
$I_2Nd_2O_2$	708	0.0003	81	64
$Cu_2Se_2Tl_2$	560	0.0003	65	50
HgI_2	148	0.0003	25	16
Br_2Ca_3Si	412	0.0004	49	36
$AgTe_2$	499	0.0004	64	81
$Cu_2Rb_2Te_2$	736	0.0004	97	58
$Ca_2H_2I_2$	886	0.0005	100	81
Cu_2I_2	452	0.0005	64	49
As_2Ir_2	724	0.0005	100	81
MnO_2	197	0.0006	20	39
$Cl_2F_2Pb_2$	708	0.0006	81	64
Bi_2Se_2	172	0.0006	25	18
H_2Li_2Pd	389	0.0006	36	49
F_4Sn	376	0.0007	49	36
Br_2CsF	520	0.0007	81	49
$Bi_2I_2O_2$	708	0.0007	81	64
$Cu_2Na_2Se_2$	708	0.0007	81	64
Ag_2I_2	164	0.0007	25	16
I_2Pb	715	0.0007	118	81
MoS_2	678	0.0008	81	118
Ge_2Se_2	472	0.0008	67	51
$AgBrO_2$	136	0.0008	18	16
$F_2I_2Tm_2$	708	0.0008	81	64
$Ag_2K_2Te_2$	118	0.0008	16	9
$Ge_2Mn_2Sr_2$	896	0.0009	101	82
S_2W	678	0.0009	81	118
$MoSe_2$	387	0.0009	48	65
In	136	0.0009	25	36
MoS_2	678	0.0009	81	118
F_4Nb	877	0.0009	113	85
La_2S_2	488	0.0009	72	50
H_2NiO_2	914	0.001	81	118
$H_2I_2Sr_2$	294	0.001	36	25
Se_2W	387	0.001	48	65
$Cu_2K_2Te_2$	814	0.001	106	65
CKN	406	0.0011	70	42
Sb_2Te_2	796	0.0012	118	81
$Br_2F_2Pb_2$	550	0.0012	64	49
$Ba_2H_2I_2$	618	0.0013	81	49

Table showing the first 50 lattice matching combinations with the smallest strain needed to build the supercell.

FeSe (P4/nmm)

Structural and electronic properties

**Formula**	FeSe
**Spacegroup**	P4/nmm
**Prototype**	FeSe
**Parent 3D**	Fe_2Se_2
**Source DB**	ICSD
**DB ID**	290411
**DF2-C09 Binding energy [meV/ Å^2]**	22.61
**RVV10 Binding energy [meV/ Å^2]**	29.3
**Band gap (PBE) [eV]**	N/A

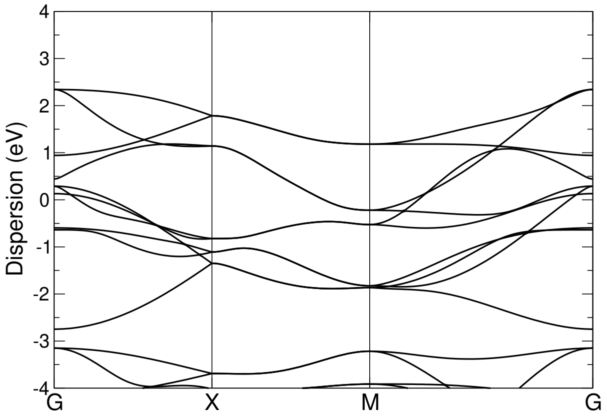

Band structure: Electronic band structure of FeSe (P4/nmm) in a representative window around the Fermi energy and along a high-symmetry path.

Optimized crystal structure

Structural parameters: relaxed cell (top) and atomic positions (bottom) of FeSe (P4/nmm) in Cartesian coordinates.

	x [Å]	y [Å]	z [Å]
$\mathbf{a_1}$	3.67632350	0.00000000	0.00000000
$\mathbf{a_2}$	0.00000000	3.67632350	0.00000000
$\mathbf{a_3}$	0.00000000	0.00000000	22.82656596

		x [Å]	y [Å]	z [Å]
●	Se	0.00000000	1.83816175	12.79947343
●	Fe	0.00000000	0.00000000	11.41328298
●	Fe	1.83816175	1.83816175	11.41328298
●	Se	1.83816175	0.00000000	10.02709253

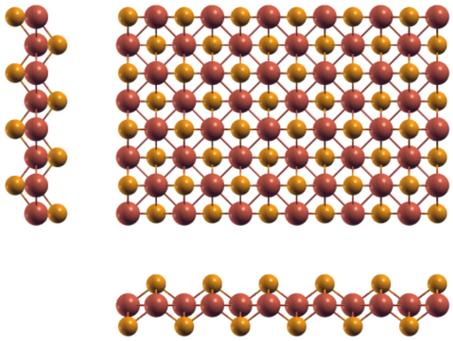

Orthographic projections: views of FeSe (P4/nmm) as seen from the x axis (left), the y axis (bottom) and the z axis (center).

Lattice matching - minimal size

Formula	N° atoms	strain	cell size 1	cell size 2
InSe	6	0.1757	1	1
HgO	6	0.2091	1	1
AsSb	6	0.1307	1	1
Bi_2	6	0.1827	1	1
GeTe	6	0.1352	1	1
AgTl	6	0.3871	1	1
S_2	6	0.1365	1	1
PbTe	6	0.1778	1	1
Sb_2	6	0.1579	1	1
$IrTe_2$	7	0.136	1	1
CrS_2	7	0.109	1	1
I_2Mg	7	0.1639	1	1
$CdCl_2$	7	0.1344	1	1
CdI_2	7	0.1799	1	1
Br_2Ca	7	0.1814	1	1
CaI_2	7	0.7866	1	1
$InSe_2$	7	0.1347	1	1
$GeTe_2$	7	0.1336	1	1
$HfTe_2$	7	0.1429	1	1
I_2Mn	7	0.1345	1	1
Br_2La	7	0.1643	1	1
Br_2Cu	7	0.1229	1	1
NSr_2	7	0.1319	1	1
I_2Yb	7	0.7768	1	1
BiClTe	7	0.1803	1	1
$AuTe_2$	7	0.1501	1	1
BrCdI	7	0.1674	1	1
Cl_2Zn	7	0.0046	1	1
$PdTe_2$	7	0.1478	1	1
FeI_2	7	0.1328	1	1
I_2Ni	7	0.1338	1	1
CrI_2	7	0.1325	1	1
I_2Zn	7	0.1546	1	1
BaF_2	7	0.1712	1	1
BiBrTe	7	0.1873	1	1
Bi_2Pd	7	0.2199	1	1
GeI_2	7	0.1619	1	1
Ba_2Hg	7	0.407	1	1
N_2W	7	0.1105	1	1
Ba_2N	7	0.1443	1	1
AsKSn	7	0.1695	1	1
Te_2Zr	7	0.1434	1	1
$PbTe_2$	7	0.1663	1	1
Cl_2Cu	7	0.109	1	1
I_2Tm	7	0.782	1	1
$SnTe_2$	7	0.1595	1	1
GeI_2	7	0.1781	1	1
STl_2	7	0.1723	1	1
BiITe	7	0.1959	1	1
BrNZr	7	0.1085	1	1

Table showing the first 50 lattice matching combinations with the smallest number of atoms in the supercell.

Lattice matching - minimal strain

Formula	N° atoms	strain	cell size 1	cell size 2
$Cu_2Se_2Tl_2$	886	0.0001	100	81
$Ba_2H_2I_2$	196	0.0001	25	16
Bi_2Pd	631	0.0001	82	101
HgO	742	0.0002	113	145
$H_2I_2Sr_2$	412	0.0003	49	36
Bi_2Pd	624	0.0004	81	100
Cl_2Cu	334	0.0004	46	50
Cl_4Mn	749	0.0005	106	65
F_4Pb	269	0.0005	36	25
F_4Nb	653	0.0005	82	65
Ba_2Cd	403	0.0005	64	49
$Si_2Te_2Zr_2$	10	0.0005	1	1
F_2Ni	7	0.0005	1	1
N_3Na	692	0.0006	103	70
Bi_2O_2	732	0.0006	101	82
ClH_3O	877	0.0006	118	81
$F_2I_2Pb_2$	550	0.0007	64	49
H_2Li_2Pd	280	0.0007	25	36
Br_2Ca_3Si	560	0.0007	65	50
$AgTe_2$	679	0.0008	85	113
Ag_2F_4	530	0.0008	80	35
$I_2S_2Tm_2$	876	0.0008	114	70
NiO_2	197	0.0008	20	39
Cu_2I_2	724	0.0008	100	81
$C_2Br_2Y_2$	686	0.0009	68	69
$I_2S_2Tl_2$	10	0.0009	1	1
$Br_2Dy_2S_2$	982	0.0009	127	79
Ba_2Hg	410	0.001	65	50
$AgCuTe_2$	448	0.001	56	56
Ho_2S_2	68	0.0011	9	8
$Cl_2Rh_2Te_2$	66	0.0011	9	5
Cu_2Se_2	8	0.0011	1	1
AlH_4Na	618	0.0011	81	49
$Cu_2Na_2Te_2$	706	0.0011	85	61
$Br_2La_2O_2$	896	0.0011	101	82
$Eu_2F_2I_2$	896	0.0012	101	82
In	492	0.0012	89	136
Bi_2O_2	724	0.0012	100	81
Ca_2O_2	732	0.0012	82	101
Mg_2	172	0.0012	25	36
H_2Li_2Pt	742	0.0012	68	94
$Dy_2I_2S_2$	770	0.0012	101	61
$Br_2H_2Sr_2$	886	0.0012	100	81
H_4Ti	280	0.0013	25	36
F_4Nb	644	0.0013	81	64
CNb_2S_2	914	0.0013	81	118
$FeSe_2$	995	0.0013	113	181
CoO_2	197	0.0013	20	39
Cl_2Hf_2	452	0.0013	48	65
CNRb	323	0.0014	56	33

Table showing the first 50 lattice matching combinations with the smallest strain needed to build the supercell.

FeTe (P4/nmm)

Structural and electronic properties

**Formula**	FeTe
**Spacegroup**	P4/nmm
**Prototype**	FeSe
**Parent 3D**	Fe_2Te_2
**Source DB**	ICSD
**DB ID**	169974
**DF2-C09 Binding energy [meV/ Å^2]**	26.63
**RVV10 Binding energy [meV/ Å^2]**	31.58
**Band gap (PBE) [eV]**	N/A

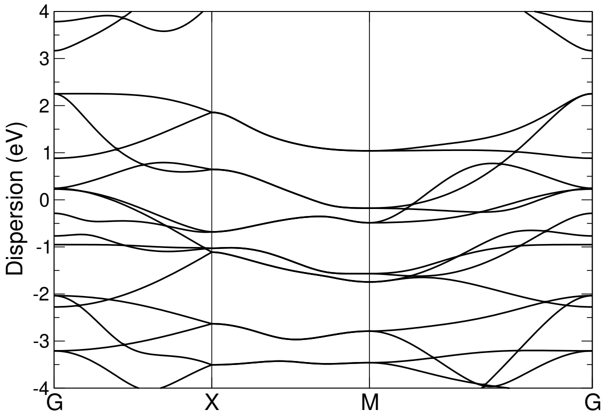

Band structure: Electronic band structure of FeTe (P4/nmm) in a representative window around the Fermi energy and along a high-symmetry path.

Optimized crystal structure

Structural parameters: relaxed cell (top) and atomic positions (bottom) of FeTe (P4/nmm) in Cartesian coordinates.

		x [Å]	y [Å]	z [Å]
$\mathbf{a_1}$		3.80212619	0.00000000	0.00000000
$\mathbf{a_2}$		0.00000000	3.80212619	0.00000000
$\mathbf{a_3}$		0.00000000	0.00000000	23.27024298
		x [Å]	y [Å]	z [Å]
●	Te	1.90106309	0.00000000	13.26119042
●	Fe	0.00000000	0.00000000	11.63512149
●	Fe	1.90106309	1.90106309	11.63512149
●	Te	0.00000000	1.90106309	10.00905256

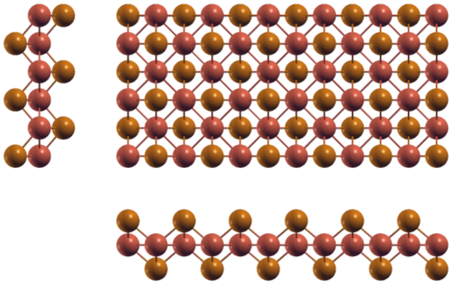

Orthographic projections: views of FeTe (P4/nmm) as seen from the x axis (left), the y axis (bottom) and the z axis (center).

Lattice matching - minimal size

Formula	N° atoms	strain	cell size 1	cell size 2
K	5	0.405	1	1
In	5	0.1093	1	1
InSe	6	0.158	1	1
HgO	6	0.5601	1	1
Bi_2	6	0.1642	1	1
Ag_2	6	0.7819	1	1
As_2	6	0.1087	1	1
PbTe	6	0.1598	1	1
Sb_2	6	0.1427	1	1
Cl_2Zn	7	0.1085	1	1
I_2Mg	7	0.1478	1	1
CdI_2	7	0.1617	1	1
Ba_2Pt	7	0.7809	1	1
Br_2Ca	7	0.163	1	1
CaI_2	7	0.1885	1	1
$HfTe_2$	7	0.1303	1	1
Br_2La	7	0.1482	1	1
Br_2Cu	7	0.1121	1	1
I_2Yb	7	0.1853	1	1
Br_2Co	7	0.1086	1	1
BiClTe	7	0.162	1	1
ReS_2	7	0.1112	1	1
Ca_2N	7	0.1088	1	1
$AuTe_2$	7	0.1361	1	1
BrCdI	7	0.1508	1	1
$PdTe_2$	7	0.1342	1	1
I_2Zn	7	0.1399	1	1
BaF_2	7	0.1541	1	1
BiBrTe	7	0.1681	1	1
Bi_2Pd	7	0.5884	1	1
GeI_2	7	0.1461	1	1
Ba_2N	7	0.1314	1	1
AsKSn	7	0.1527	1	1
Te_2Zr	7	0.1306	1	1
$PbTe_2$	7	0.1499	1	1
Cl_2Cu	7	0.102	1	1
I_2Tm	7	0.187	1	1
$SnTe_2$	7	0.1441	1	1
GeI_2	7	0.1601	1	1
I_2Pb	7	0.788	1	1
STl_2	7	0.155	1	1
BiITe	7	0.1757	1	1
Br_2Fe	7	0.1086	1	1
DyI_2	7	0.1922	1	1
Se_2Yb	7	0.1463	1	1
$BiTe_2$	7	0.1466	1	1
GdI_2	7	0.1714	1	1
$PtTe_2$	7	0.1358	1	1
Br_2Cd	7	0.1335	1	1
O_2Pt	7	0.1096	1	1

Table showing the first 50 lattice matching combinations with the smallest number of atoms in the supercell.

Lattice matching - minimal strain

Formula	N° atoms	strain	cell size 1	cell size 2
Ho_2S_2	696	0.0	89	85
Ag_2I_2	584	0.0001	85	61
Se_2Ta_4	752	0.0001	65	82
O_2Zn	339	0.0002	36	65
$FeSe_2$	775	0.0003	85	145
H_2MnO_2	275	0.0004	20	39
$CrTe_2$	387	0.0004	48	65
$Br_2O_2Yb_2$	10	0.0004	1	1
Ca_2Cl_2	8	0.0004	1	1
$I_2S_2Tb_2$	550	0.0005	70	45
$Cu_2K_2Te_2$	294	0.0005	36	25
Cl_2Sc_2	452	0.0005	48	65
Hg_3S_2	562	0.0005	103	30
$Cu_2Na_2Te_2$	550	0.0005	64	49
Sn	277	0.0006	49	81
As_2Ru_2	8	0.0006	1	1
Se_2Ta_4	742	0.0006	64	81
In	366	0.0006	65	106
Br_2Cr	387	0.0007	48	65
Tl	136	0.0008	25	36
$Br_2H_2Zr_2$	582	0.0008	48	65
Ca_2O_2	452	0.0008	49	64
$Br_2O_2Tm_2$	10	0.0009	1	1
$Dy_2I_2S_2$	550	0.001	70	45
Br_2Ti	387	0.001	48	65
$Ho_2I_2S_2$	584	0.001	74	48
Bi_2Se_2	472	0.001	65	53
Cl_4Mn	989	0.001	136	89
Hg_3S_2	486	0.0011	89	26
$AgClO_4$	814	0.0011	106	65
Mg_4	244	0.0011	25	36
HgI_2	523	0.0011	85	61
LiO	568	0.0011	79	126
K	693	0.0012	145	113
$LiNbS_2$	796	0.0013	81	118
$Cl_2Hg_2N_2$	564	0.0013	81	40
S_2Ta	678	0.0013	81	118
O_4PSn	294	0.0013	36	25
$F_2I_2Pb_2$	896	0.0014	101	82
$C_2I_2Y_2$	676	0.0014	67	68
$CuGeO_3$	791	0.0014	94	83
Cu_2F_4	346	0.0014	49	25
Au_2I_2	428	0.0015	61	46
Hg_3S_2	431	0.0015	79	23
Ba_2Cd	650	0.0015	101	82
BiITe	679	0.0015	103	89
$Cl_2H_2Sc_2$	582	0.0015	48	65
Bi_2Te_3	857	0.0015	103	89
Br_2V	427	0.0015	52	73
BiITe	587	0.0016	89	77

Table showing the first 50 lattice matching combinations with the smallest strain needed to build the supercell.

Ga_2S_3 (P-3m1)

Structural and electronic properties

**Formula**	Ga_2S_3
**Spacegroup**	P-3m1
**Prototype**	As
**Parent 3D**	Ga_2S_3
**Source DB**	MPDS
**DB ID**	S1831448
**DF2-C09 Binding energy [meV/ Å^2]**	20.66
**RVV10 Binding energy [meV/ Å^2]**	N/A
**Band gap (PBE) [eV]**	0.56

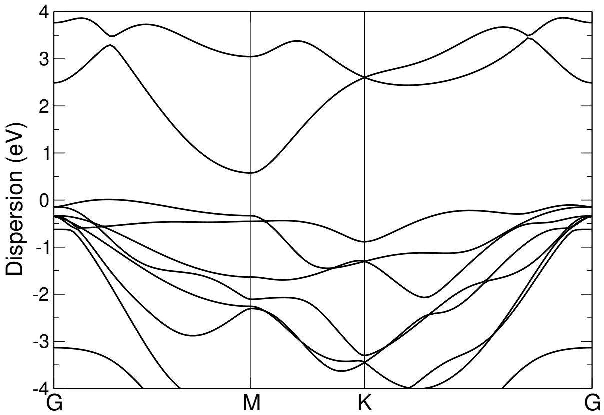

Band structure: Electronic band structure of Ga_2S_3 (P-3m1) in a representative window around the Fermi energy and along a high-symmetry path.

Optimized crystal structure

Structural parameters: relaxed cell (top) and atomic positions (bottom) of Ga_2S_3 (P-3m1) in Cartesian coordinates.

	x [Å]	y [Å]	z [Å]
$\mathbf{a_1}$	1.77551643	−3.07528467	0.00000000
$\mathbf{a_2}$	1.77551643	3.07528467	0.00000000
$\mathbf{a_3}$	0.00000000	0.00000000	23.36996352

	x [Å]	y [Å]	z [Å]
Ga	0.88775821	0.51254744	-13.42479898
S	2.66327464	-0.51254744	-14.57673682
Ga	2.66327464	-0.51254744	-9.94516454
S	0.88775821	-1.53764233	-11.68498176
S	0.88775821	0.51254744	-8.79322670

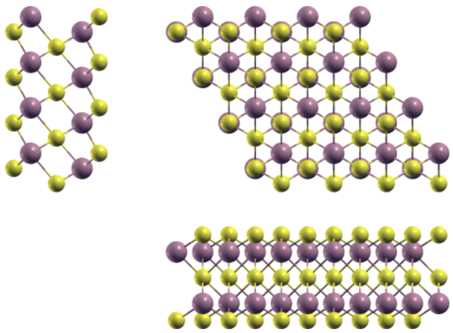

Orthographic projections: views of Ga_2S_3 (P-3m1) as seen from the x axis (left), the y axis (bottom) and the z axis (center).

Lattice matching - minimal size

Formula	N° atoms	strain	cell size 1	cell size 2
Tl	6	0.1254	1	1
Na	6	0.0004	1	1
In	6	0.1117	1	1
In	6	0.2601	1	1
Gd	6	0.1139	1	1
HgO	7	0.134	1	1
As_2	7	0.0077	1	1
S_2	7	0.4495	1	1
LiO	7	0.2753	1	1
Mg_2	7	0.1164	1	1
$IrTe_2$	8	0.4479	1	1
Cl_2Zn	8	0.0066	1	1
S_2V	8	0.2646	1	1
MoS_2	8	0.2656	1	1
$MoTe_2$	8	0.0019	1	1
$AgTe_2$	8	0.1274	1	1
$HfTe_2$	8	0.4686	1	1
Te_2V	8	0.0039	1	1
Br_2Co	8	0.0073	1	1
ReS_2	8	0.2483	1	1
Ca_2N	8	0.0083	1	1
$PdTe_2$	8	0.4822	1	1
MnO_2	8	4.874	1	1
Mg_3	8	0.1218	1	1
I_2Zn	8	0.5008	1	1
Te_2Zn	8	0.0017	1	1
S_2W	8	0.2657	1	1
Bi_2Pd	8	0.1418	1	1
Br_2Mn	8	0.0054	1	1
$CrTe_2$	8	0.0057	1	1
PtS_2	8	0.0007	1	1
CdClO	8	0.0034	1	1
Ba_2N	8	0.4725	1	1
Se_2Ti	8	0.002	1	1
Br_2Ti	8	0.0062	1	1
Te_2Zr	8	0.4698	1	1
Te_2W	8	0.0021	1	1
$PbTe_2$	8	3.0421	1	1
Cl_2V	8	0.2716	1	1
OTl_2	8	0.0035	1	1
BrNZr	8	0.0089	1	1
Br_2Fe	8	0.0073	1	1
O_2Zn	8	1.5446	1	1
Br_2Cr	8	0.0059	1	1
$FeSe_2$	8	0.4248	1	1
CuO_2	8	2.75	1	1
MoS_2	8	0.2659	1	1
$CrSe_2$	8	0.2698	1	1
Se_2Ta	8	0.007	1	1
Br_2Cd	8	0.4798	1	1

Table showing the first 50 lattice matching combinations with the smallest number of atoms in the supercell.

Lattice matching - minimal strain

Formula	N° atoms	strain	cell size 1	cell size 2
$F_2Lu_2Se_2$	543	0.0	57	43
As_2CeLi_2	305	0.0	36	25
H_2NiO_2	905	0.0	81	100
PbTe	230	0.0	36	25
Pt_2Te_2	747	0.0001	91	73
MnO_2	488	0.0001	49	81
NS_2Zr	9	0.0001	1	1
$BiTe_2$	353	0.0001	49	36
Br_2CsF	275	0.0002	39	20
Br_2Tb_2	593	0.0002	73	57
S_2V	638	0.0002	73	91
Sb_2Se_2Te	425	0.0002	49	36
GeI_2	255	0.0002	36	25
$Br_2Dy_2O_2$	613	0.0002	65	48
$Br_2O_2Y_2$	613	0.0002	65	48
$F_2Ho_2Se_2$	789	0.0002	81	64
NaPSn	674	0.0002	91	73
IKO_3	25	0.0002	4	1
Li_2Tl_2	551	0.0003	79	39
Cu_2S_2	517	0.0003	65	48
O_2Zn	327	0.0003	36	49
Cl_2La_2	516	0.0003	64	49
F_2Tl_2	517	0.0003	65	48
Na	6	0.0004	1	1
$PdTe_2$	536	0.0004	73	57
Se_2Yb	353	0.0004	49	36
$Er_2F_2Se_2$	893	0.0004	91	73
Bi_2S_3	650	0.0004	73	57
P_2Rh_2	517	0.0005	65	48
$Ge_2I_2La_2$	221	0.0005	25	16
$LiMnTe_2$	280	0.0005	36	25
$F_2Se_2Tm_2$	986	0.0005	100	81
S_2Sn_2	840	0.0006	112	70
In	401	0.0006	64	81
Br_2Ca_3Si	527	0.0006	61	37
GeI_2	353	0.0006	49	36
$LiMnSe_2$	824	0.0006	100	81
Sb_2Se_2Te	425	0.0007	49	36
$HfTe_2$	674	0.0007	91	73
PtS_2	8	0.0007	1	1
H_2MgO_2	820	0.0007	73	91
MnO_2	368	0.0007	37	61
In_2Se_3	820	0.0007	91	73
Br_2Ho_2	593	0.0007	73	57
I_2La_2Te	490	0.0007	61	37
ReS_2	437	0.0007	49	64
$I_2S_2Sm_2$	699	0.0007	81	49
C_2Li_2	495	0.0008	63	45
O_2Sn_2	517	0.0008	65	48
P_4	307	0.0008	39	28

Table showing the first 50 lattice matching combinations with the smallest strain needed to build the supercell.

Ga_2Se_2 (P3m1)

Structural and electronic properties

**Formula**	Ga_2Se_2
**Spacegroup**	P3m1
**Prototype**	GaSe
**Parent 3D**	Ga_2Se_2
**Source DB**	MPDS
**DB ID**	S1251699
**DF2-C09 Binding energy [meV/ Å^2]**	15.81
**RVV10 Binding energy [meV/ Å^2]**	N/A
**Band gap (PBE) [eV]**	0.96

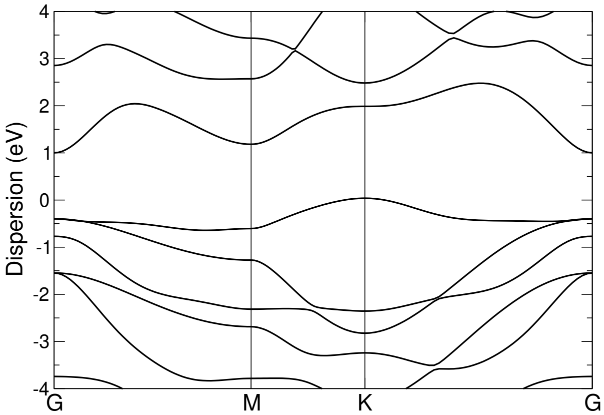

Band structure: Electronic band structure of Ga_2Se_2 (P3m1) in a representative window around the Fermi energy and along a high-symmetry path.

Optimized crystal structure

Structural parameters: relaxed cell (top) and atomic positions (bottom) of Ga_2Se_2 (P3m1) in Cartesian coordinates.

	x [Å]	y [Å]	z [Å]
$\mathbf{a_1}$	−1.95118191	−3.37954620	0.00000000
$\mathbf{a_2}$	3.90236381	0.00000000	0.00000000
$\mathbf{a_3}$	0.00000000	0.00000000	21.96800772
	x [Å]	y [Å]	z [Å]
Ga	0.00000000	0.00000000	-1.27889418
Se	0.00000000	-2.25303080	-2.39637580
Ga	0.00000000	-2.25303080	2.57637952
Se	0.00000000	0.00000000	1.09889046

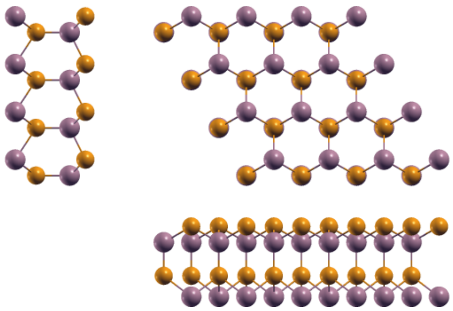

Orthographic projections: views of Ga_2Se_2 (P3m1) as seen from the x axis (left), the y axis (bottom) and the z axis (center).

Lattice matching - minimal size

Formula	N° atoms	strain	cell size 1	cell size 2
Na	5	0.2747	1	1
AsSb	6	0.007	1	1
Bi_2	6	0.4508	1	1
GeTe	6	0.0011	1	1
S_2	6	0.0005	1	1
CaCl	6	0.1374	1	1
$IrTe_2$	7	0.0002	1	1
$CdCl_2$	7	0.0021	1	1
$ReSe_2$	7	0.257	1	1
Br_2Ca	7	0.4481	1	1
$InSe_2$	7	0.0018	1	1
$GeTe_2$	7	0.0031	1	1
$HfTe_2$	7	0.0083	1	1
I_2Mn	7	0.002	1	1
NSr_2	7	0.0055	1	1
I_2Yb	7	0.4965	1	1
LiO_2	7	0.0679	1	1
Cl_2Zn	7	0.1503	1	1
FeI_2	7	0.0042	1	1
I_2Ni	7	0.003	1	1
S_2Ti	7	0.2488	1	1
CrI_2	7	0.0046	1	1
BiBrTe	7	0.4596	1	1
Bi_2Pd	7	0.1136	1	1
Cl_2Ni	7	0.2582	1	1
Cl_2Co	7	0.2484	1	1
$CrTe_2$	7	0.2668	1	1
NbS_2	7	1.5446	1	1
Br_2V	7	0.2559	1	1
Cl_2Fe	7	0.2474	1	1
Se_2Ti	7	0.2723	1	1
Br_2Ti	7	0.266	1	1
Te_2Zr	7	0.0088	1	1
$AsSe_2$	7	0.2602	1	1
I_2Tm	7	0.5001	1	1
BiITe	7	0.4761	1	1
CdO_2	7	0.2482	1	1
BrNZr	7	0.2621	1	1
$NbSe_2$	7	0.2588	1	1
CoI_2	7	0.0066	1	1
GeS_2	7	0.1284	1	1
$MnSe_2$	7	0.1374	1	1
Br_2Cr	7	0.2664	1	1
Cl_2Zr	7	0.2479	1	1
Se_2Ta	7	0.2589	1	1
Br_2Mg	7	0.0043	1	1
I_2Ti	7	0.0063	1	1
$NbSe_2$	7	0.2606	1	1
GdI_2	7	0.4668	1	1
F_2Ni	7	0.1465	1	1

Table showing the first 50 lattice matching combinations with the smallest number of atoms in the supercell.

Lattice matching - minimal strain

Formula	N° atoms	strain	cell size 1	cell size 2
I_2La_2Te	376	0.0	49	36
Bi_2Te_3	644	0.0	81	64
$BrKO_3$	449	0.0	81	25
GdI_2	583	0.0	91	73
$Cl_2H_2Zr_2$	438	0.0001	36	49
BiITe	516	0.0001	81	64
H_2MnO_2	116	0.0002	9	16
$IrTe_2$	7	0.0002	1	1
Br_2Ca_3Si	412	0.0002	49	36
AsI_2La_2	577	0.0002	73	57
Ba_2Cu_2	452	0.0002	64	49
Cd_2I_3	577	0.0003	73	57
Se_2Ta	499	0.0003	64	81
Br_2V	447	0.0003	57	73
S_2Ti	388	0.0003	49	64
NbS_2	291	0.0003	36	49
$ClKO_3$	276	0.0004	49	16
$NbSe_2$	499	0.0004	64	81
O_2Zn	331	0.0004	37	61
Se_2Ta	565	0.0004	73	91
Cu_2Sr_2	724	0.0004	100	81
I_2Yb	403	0.0004	64	49
S_2	6	0.0005	1	1
Cl_2Zr_2	520	0.0005	57	73
$CrTe_2$	624	0.0005	81	100
CaI_2	357	0.0005	57	43
Cl_2Hf_2	400	0.0006	43	57
BiBrTe	643	0.0006	100	81
$Cl_2H_2Sc_2$	838	0.0006	73	91
Cl_2Sc_2	724	0.0006	81	100
Ni_2SbTe_2	9	0.0006	1	1
Cl_2Co	388	0.0007	49	64
$AsSe_2$	499	0.0007	64	81
C_2F_2	52	0.0007	4	9
Cl_2Ni	499	0.0008	64	81
$Gd_2I_2Se_2$	724	0.0008	100	54
CdO_2	388	0.0008	49	64
Br_2Cr	624	0.0008	81	100
$LiOS_2Ti$	389	0.0008	36	49
CCl_2Sc_2	593	0.0009	57	73
In_2S_3	9	0.0009	1	1
$Cl_2Fe_2O_2$	784	0.0009	73	82
$Br_2H_2Zr_2$	924	0.0009	81	100
$NbSe_2$	499	0.0009	64	81
$I_2N_2Ti_2$	924	0.0009	96	90
I_2La_2P	729	0.001	91	73
Cl_2Zr	388	0.001	49	64
$I_2Se_2Tb_2$	400	0.001	55	30
$AlLiTe_2$	580	0.0011	81	64
GeTe	6	0.0011	1	1

Table showing the first 50 lattice matching combinations with the smallest strain needed to build the supercell.

$Ga_2Te_2Br_2$ (Pm)

Structural and electronic properties

**Formula**	$Ga_2Te_2Br_2$
**Spacegroup**	Pm
**Prototype**	GaTeCl
**Parent 3D**	$Br_4Ga_4Te_4$
**Source DB**	MPDS
**DB ID**	S1718665
**DF2-C09 Binding energy [meV/ Å^2]**	14.28
**RVV10 Binding energy [meV/ Å^2]**	N/A
**Band gap (PBE) [eV]**	2.14

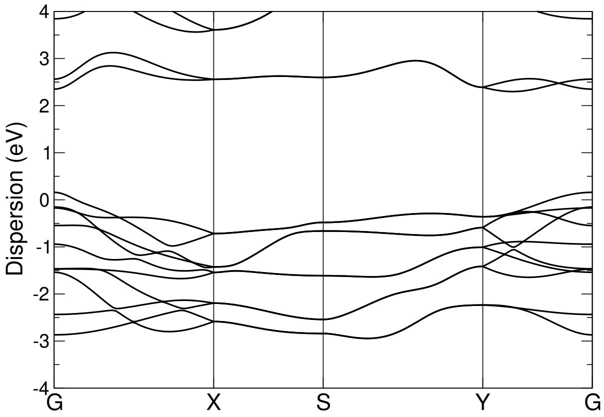

Band structure: Electronic band structure of $Ga_2Te_2Br_2$ (Pm) in a representative window around the Fermi energy and along a high-symmetry path.

Optimized crystal structure

Structural parameters: relaxed cell (top) and atomic positions (bottom) of $Ga_2Te_2Br_2$ (Pm) in Cartesian coordinates.

	x [Å]	y [Å]	z [Å]
$\mathbf{a_1}$	−4.13143239	0.00000000	0.00000000
$\mathbf{a_2}$	0.00000000	−6.01839903	0.00000000
$\mathbf{a_3}$	0.00000000	0.00000000	22.63417135

		x [Å]	y [Å]	z [Å]
●	Ga	-2.06571619	-2.87166671	0.93886482
●	Ga	0.00000000	-5.88086623	-0.93886482
●	Te	-2.06571619	-1.54154122	-1.36035773
●	Te	0.00000000	-4.55074074	1.36035773
●	Br	-2.06571619	-1.50045622	2.84437876
●	Br	0.00000000	-4.50965574	-2.84437876

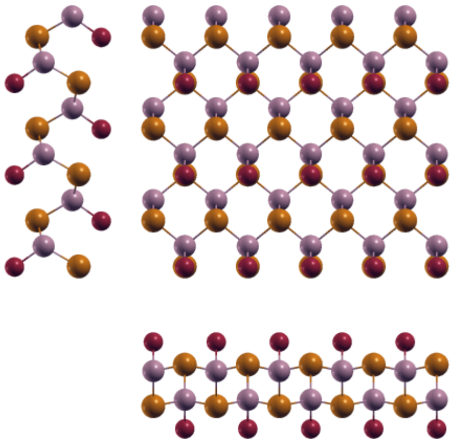

Orthographic projections: views of $Ga_2Te_2Br_2$ (Pm) as seen from the x axis (left), the y axis (bottom) and the z axis (center).

Lattice matching - minimal size

Formula	N° atoms	strain	cell size 1	cell size 2
GeTe	8	0.1221	1	1
Tl	8	0.2877	1	2
Na	8	0.1428	1	2
$IrTe_2$	9	0.1219	1	1
$CdCl_2$	9	0.1222	1	1
$InSe_2$	9	0.1222	1	1
I_2Mn	9	0.1222	1	1
NSr_2	9	0.1228	1	1
FeI_2	9	0.1226	1	1
I_2Ni	9	0.1224	1	1
CrI_2	9	0.1226	1	1
Br_2Mg	9	0.1226	1	1
Se_2Sn	9	0.1228	1	1
H_2Si_2	10	0.1221	1	1
Au_2Br_2	10	0.2587	1	1
S_2Zn_2	10	0.1217	1	1
P_2Sn_2	10	0.1217	1	1
$LiMnSe_2$	10	0.1213	1	1
Ga_2Se_2	10	0.1219	1	1
As_2	10	0.1488	1	2
S_2	10	0.612	1	2
Au_2Se_2	10	0.1578	1	1
Ni_2Te_2	10	0.1223	1	1
Cl_2Tb_2	10	0.1214	1	1
Hg_3N_2	11	0.3866	1	1
FKO_2Se	11	0.0292	1	1
$ClKO_3$	11	0.366	1	1
In_2S_3	11	0.122	1	1
Ni_2SbTe_2	11	0.1218	1	1
$BrKO_3$	11	0.397	1	1
Cl_2Zn	12	0.1479	1	2
$Br_2Ho_2S_2$	12	0.3803	1	1
$I_2Lu_2Se_2$	12	0.0043	1	1
Cl_2Mn	12	0.1279	1	2
$MoTe_2$	12	0.1444	1	2
PSn_2	12	0.1562	1	2
$Ho_2I_2S_2$	12	0.3889	1	1
Br_2Zn	12	0.1661	1	2
HfS_2	12	0.151	1	2
$AsSn_2$	12	0.1596	1	2
$GeTe_2$	12	0.6007	1	2
Te_2V	12	0.1459	1	2
$CuTe_2$	12	0.1507	1	2
S_2Zr	12	0.1555	1	2
Br_2Co	12	0.1484	1	2
$Cl_2Rh_2Te_2$	12	0.1643	1	1
Ca_2N	12	0.1492	1	2
S_2Ti	12	0.1314	1	2
Te_2Ti	12	0.1664	1	2
$Br_2S_2Y_2$	12	0.3858	1	1

Table showing the first 50 lattice matching combinations with the smallest number of atoms in the supercell.

Lattice matching - minimal strain

Formula	N° atoms	strain	cell size 1	cell size 2
Sb_2Te_3	809	0.0007	59	91
Cu_4Te_2	900	0.0008	59	91
$I_2Pr_2S_2$	504	0.0009	36	48
I_2Pb	360	0.0009	36	48
Cu_4Te_2	852	0.001	56	86
BiBrTe	594	0.0011	56	86
Co_2S_2	286	0.0011	21	40
$AgCuTe_2$	654	0.0011	49	90
Bi_2In_2	990	0.0012	93	108
$F_2Se_2Y_2$	960	0.0013	72	88
Cu_4Te_2	822	0.0013	54	83
Ag_2	312	0.0013	36	48
Cl_2Zn	789	0.0014	69	125
Sb_2Te_3	766	0.0014	56	86
Bi_2SeTe_2	809	0.0015	59	91
BiBrTe	540	0.0015	51	78
Br_2CsF	990	0.0015	93	108
Ba_2Pt	360	0.0016	36	48
Sb_2Te_3	739	0.0016	54	83
Au_2I_2	960	0.0016	86	111
Cl_2OOs	776	0.0017	58	107
Cu_2Sr_2	618	0.0017	51	78
CaCl	206	0.0017	21	40
Cu_2F_4	756	0.0017	67	59
$MnSe_2$	246	0.0018	21	40
$C_2Cl_2Y_2$	438	0.0019	25	48
Hg_3N_2	726	0.0019	81	48
CrS_2	792	0.0019	52	160
Ho_2S_2	252	0.0019	20	33
Cl_4Mn	689	0.0019	59	67
Bi_2	536	0.002	59	91
$AuCrTe_4$	738	0.002	63	60
Sb_2Te_2	408	0.002	36	48
Se_2Ta_4	762	0.002	40	87
Ge_2S_2	814	0.002	67	103
Hg_3N_2	619	0.002	69	41
$HNiO_2$	738	0.0021	39	126
Au_2I_2	892	0.0021	80	103
C_2Li_2	500	0.0021	40	65
Cu_4Te_2	774	0.0021	51	78
Gd	201	0.0022	20	81
Sm	214	0.0022	21	88
$Ce_2I_2Si_2$	96	0.0022	6	10
Sb_2Se_2Te	86	0.0022	6	10
$ReSe_2$	66	0.0022	5	12
$I_2Nd_2S_2$	282	0.0022	20	27
I_2Mg	66	0.0022	6	10
CNNa	729	0.0022	70	103
Bi_2Te_2	690	0.0022	63	78
I_2La_2P	696	0.0022	51	78

Table showing the first 50 lattice matching combinations with the smallest strain needed to build the supercell.

GaGeTe (P-3m1)

Structural and electronic properties

**Formula**	GaGeTe
**Spacegroup**	P-3m1
**Prototype**	GaGeTe
**Parent 3D**	$Ga_2Ge_2Te_2$
**Source DB**	ICSD
**DB ID**	35386
**DF2-C09 Binding energy [meV/ Å^2]**	16.71
**RVV10 Binding energy [meV/ Å^2]**	22.0
**Band gap (PBE) [eV]**	0.76

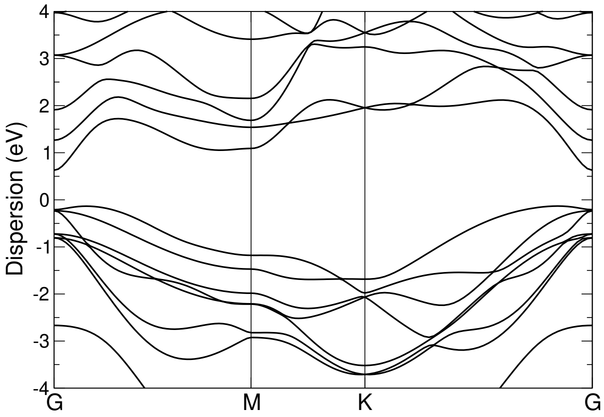

Band structure: Electronic band structure of GaGeTe (P-3m1) in a representative window around the Fermi energy and along a high-symmetry path.

Optimized crystal structure

Structural parameters: relaxed cell (top) and atomic positions (bottom) of GaGeTe (P-3m1) in Cartesian coordinates.

	x [Å]	y [Å]	z [Å]
$\mathbf{a_1}$	4.10978936	0.00000000	0.00000000
$\mathbf{a_2}$	−2.05489468	3.55918199	0.00000000
$\mathbf{a_3}$	0.00000000	0.00000000	28.35702162

		x [Å]	y [Å]	z [Å]
●	Te	-0.00000000	2.37278799	18.33103598
●	Ga	2.05489468	1.18639400	11.30564355
●	Ge	2.05489468	1.18639400	13.77599984
●	Ga	0.00000000	0.00000000	17.05137807
●	Ge	0.00000000	0.00000000	14.58102178
●	Te	-0.00000000	2.37278799	10.02598564

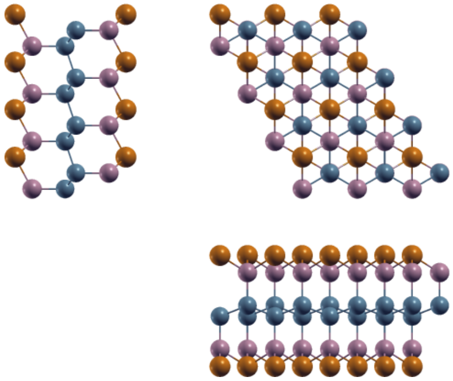

Orthographic projections: views of GaGeTe (P-3m1) as seen from the x axis (left), the y axis (bottom) and the z axis (center).

Lattice matching - minimal size

Formula	N° atoms	strain	cell size 1	cell size 2
K	7	0.206	1	1
K	7	0.4636	1	1
Ag_2	8	0.4775	1	1
As_2	8	1.6307	1	1
Sb_2	8	0.0007	1	1
CaCl	8	0.1207	1	1
Cl_2Zn	9	0.2501	1	1
I_2Mg	9	0.005	1	1
MoS_2	9	4.8445	1	1
$MoTe_2$	9	1.5883	1	1
PSn_2	9	0.2637	1	1
Ba_2Pt	9	0.4769	1	1
HfS_2	9	0.2551	1	1
$AsSn_2$	9	0.2691	1	1
Te_2V	9	0.2465	1	1
I_2Pr	9	0.1499	1	1
$CuTe_2$	9	0.2546	1	1
S_2Zr	9	0.2626	1	1
Br_2La	9	0.0054	1	1
Ca_2Si	9	2.8364	1	1
Br_2Co	9	1.6275	1	1
Ca_2N	9	1.6347	1	1
$AuTe_2$	9	0.0084	1	1
BrCdI	9	0.0083	1	1
Cl_2Zn	9	0.1298	1	1
I_2Zn	9	0.0039	1	1
$RhTe_2$	9	0.2732	1	1
S_2W	9	4.8459	1	1
GeI_2	9	0.0032	1	1
Br_2Mn	9	0.2485	1	1
$CrTe_2$	9	1.5329	1	1
$CoTe_2$	9	0.2556	1	1
CdClO	9	1.5989	1	1
Se_2Ti	9	1.56	1	1
Br_2Ti	9	1.5288	1	1
Te_2W	9	1.5895	1	1
$PbTe_2$	9	0.0073	1	1
I_2Nd	9	0.1508	1	1
S_2Sn	9	0.263	1	1
$SnTe_2$	9	0.0009	1	1
I_2Pb	9	0.4816	1	1
$PtSe_2$	9	0.27	1	1
OTl_2	9	1.6003	1	1
Br_2Fe	9	1.628	1	1
GeS_2	9	0.1147	1	1
$TaTe_2$	9	0.2683	1	1
$MnSe_2$	9	0.1206	1	1
DyI_2	9	0.4479	1	1
Br_2Ni	9	0.2578	1	1
CeI_2	9	0.1492	1	1

Table showing the first 50 lattice matching combinations with the smallest number of atoms in the supercell.

Lattice matching - minimal strain

Formula	N° atoms	strain	cell size 1	cell size 2
C_2	104	0.0	9	25
CdH_2O_2	543	0.0	43	57
H_2NiO_2	699	0.0001	49	81
$CoTe_2$	561	0.0001	57	73
CdClHO	886	0.0001	81	100
Li_2P_2Pr	11	0.0001	1	1
KS_2Ti	550	0.0002	49	64
Ga_2S_2	634	0.0002	57	73
KNO_3	509	0.0002	64	25
HfS_2	561	0.0003	57	73
CS_2Ta_2	221	0.0003	16	25
Ba_2Pt	678	0.0003	81	64
Cl_2Ti	171	0.0004	16	25
Te_2W	429	0.0004	43	57
Bi_2Te_2	438	0.0004	49	36
PSn_2	711	0.0005	73	91
Ge_2Se_2	634	0.0005	67	58
$TaTe_2$	786	0.0005	81	100
P_2	146	0.0005	16	25
$MoTe_2$	429	0.0006	43	57
Br_2Mn	486	0.0006	49	64
Ag_2	614	0.0006	81	64
Cl_2Zn	486	0.0006	49	64
$CuTe_2$	561	0.0006	57	73
I_2Pb	609	0.0006	73	57
HN_3OZn	246	0.0006	16	25
Sb_2	8	0.0007	1	1
H_2NiO_2	527	0.0007	37	61
$Cl_2H_2Lu_2$	870	0.0007	64	81
Te_2Zn	429	0.0007	43	57
P_4	964	0.0007	98	94
$Cl_2Hg_2N_2$	270	0.0008	30	15
Sb_2Te_2	666	0.0008	73	57
$CrSe_2$	405	0.0008	37	61
K_2PtSe_2	304	0.0008	39	14
$Br_2N_2Zr_2$	870	0.0009	64	81
$SnTe_2$	9	0.0009	1	1
CdClO	429	0.0009	43	57
Br_2La_2	10	0.0009	1	1
CBr_2Lu_2	789	0.0009	64	81
FHOZn	466	0.0009	37	61
I_2La_2Sb	911	0.0009	91	73
S_2Sn	711	0.001	73	91
K	681	0.001	100	81
Ag_2Te_2	640	0.001	64	64
$HfLiS_2$	486	0.001	43	57
Cl_2Mg	627	0.001	64	81
CNb_2S_2	221	0.001	16	25
Tl	604	0.001	81	118
$AsSn_2$	786	0.001	81	100

Table showing the first 50 lattice matching combinations with the smallest strain needed to build the supercell.

GaS (P-3m1)

Structural and electronic properties

**Formula**	GaS
**Spacegroup**	P-3m1
**Prototype**	GaS (P-3m1)
**Parent 3D**	Ga_2S_2
**Source DB**	ICSD
**DB ID**	40824
**DF2-C09 Binding energy [meV/ Å^2]**	16.05
**RVV10 Binding energy [meV/ Å^2]**	20.81
**Band gap (PBE) [eV]**	2.21

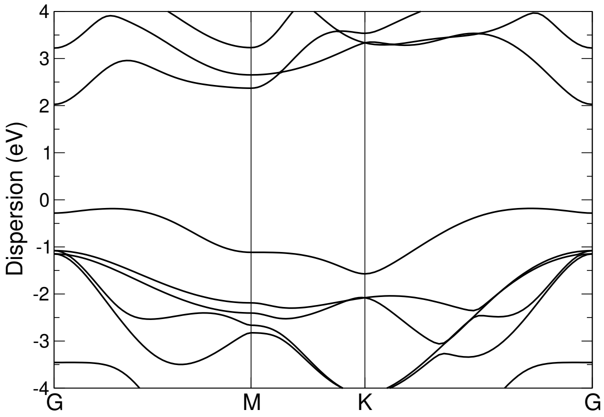

Band structure: Electronic band structure of GaS (P-3m1) in a representative window around the Fermi energy and along a high-symmetry path.

Optimized crystal structure

Structural parameters: relaxed cell (top) and atomic positions (bottom) of GaS (P-3m1) in Cartesian coordinates.

	x [Å]	y [Å]	z [Å]
$\mathbf{a_1}$	3.64133231	0.00000000	0.00000000
$\mathbf{a_2}$	−1.82066616	3.15348628	0.00000000
$\mathbf{a_3}$	0.00000000	0.00000000	24.64142092

		x [Å]	y [Å]	z [Å]
●	Ga	0.00000000	2.10232419	13.55350159
●	S	1.82066616	1.05116209	10.00816632
●	S	0.00000000	0.00000000	14.63325580
●	Ga	0.00000000	2.10232419	11.08791812

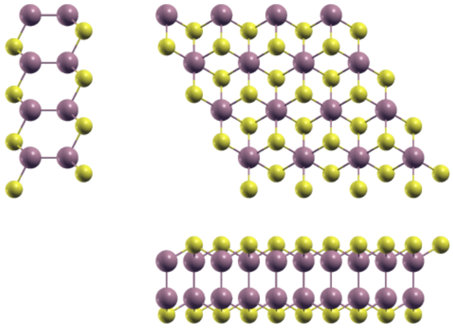

Orthographic projections: views of GaS (P-3m1) as seen from the x axis (left), the y axis (bottom) and the z axis (center).

Lattice matching - minimal size

Formula	N° atoms	strain	cell size 1	cell size 2
Tl	5	0.1186	1	1
In	5	0.4227	1	1
In	5	1.5898	1	1
InSe	6	2.9921	1	1
HgO	6	0.1256	1	1
As_2	6	0.0042	1	1
LiO	6	0.2583	1	1
P_2	6	0.2691	1	1
PbTe	6	3.0157	1	1
Mg_2	6	0.1116	1	1
Sb_2	6	0.4782	1	1
Cl_2Zn	7	0.0052	1	1
I_2Mg	7	0.493	1	1
S_2V	7	0.2484	1	1
MoS_2	7	0.2494	1	1
CdI_2	7	3.039	1	1
$AgTe_2$	7	0.1202	1	1
PSn_2	7	0.0047	1	1
$MoSe_2$	7	0.2757	1	1
HfS_2	7	0.0015	1	1
$AsSn_2$	7	0.0086	1	1
Te_2V	7	0.0079	1	1
$CuTe_2$	7	0.0019	1	1
S_2Zr	7	0.0039	1	1
Br_2La	7	0.4939	1	1
Br_2Cu	7	1.0253	1	1
NiO_2	7	4.847	1	1
Br_2Co	7	0.0046	1	1
BiClTe	7	3.0438	1	1
ReS_2	7	1.5266	1	1
Ca_2N	7	0.0037	1	1
Cl_2Ti	7	0.2693	1	1
$AuTe_2$	7	0.4583	1	1
BrCdI	7	0.5014	1	1
$PdTe_2$	7	0.4523	1	1
Mg_3	7	0.1157	1	1
I_2Zn	7	0.4698	1	1
S_2W	7	0.2495	1	1
Bi_2Pd	7	0.1322	1	1
GeI_2	7	2.834	1	1
Br_2Mn	7	0.0064	1	1
$CoTe_2$	7	0.0012	1	1
CdClO	7	0.0083	1	1
AsKSn	7	2.9225	1	1
$PbTe_2$	7	0.4987	1	1
S_2Sn	7	0.0042	1	1
$SnTe_2$	7	0.4822	1	1
Cl_2V	7	0.2548	1	1
GeI_2	7	3.0189	1	1
$PtSe_2$	7	0.0092	1	1

Table showing the first 50 lattice matching combinations with the smallest number of atoms in the supercell.

Lattice matching - minimal strain

Formula	N° atoms	strain	cell size 1	cell size 2
N_3W_2	189	0.0	16	25
Cu_2I_2	452	0.0	64	49
Br_2Gd_2Ge	501	0.0	64	49
In_2Se_2	656	0.0	91	73
As_2Sn_2	724	0.0	100	81
$AsLi_3$	340	0.0	49	36
Br_2Gd_2	724	0.0	100	81
MoS_2	388	0.0001	49	64
I_2La_2	400	0.0001	57	43
I_2La_2Te	180	0.0001	25	16
BH_4Li	634	0.0001	73	57
S_2W	388	0.0002	49	64
$Br_2Nd_2O_2$	548	0.0003	65	48
Gd_2I_2	452	0.0003	64	49
Br_2Ca_3Si	196	0.0003	25	16
MoS_2	388	0.0003	49	64
$F_2Se_2Y_2$	118	0.0003	16	9
PTe_2Ti_2	9	0.0003	1	1
InSe	268	0.0003	49	36
In	229	0.0004	43	57
As_2Li_2Pr	376	0.0004	49	36
$SnTe_2$	463	0.0004	73	57
$Ce_2I_2S_2$	466	0.0004	61	37
$I_2La_2Si_2$	412	0.0004	49	36
Ca_2Si	355	0.0004	61	37
$I_2O_2Y_2$	548	0.0004	65	48
Cl_2V	447	0.0005	57	73
Br_2Ni	7	0.0005	1	1
Br_2OV	628	0.0005	82	75
Bi_2Se_3	501	0.0005	64	49
CNb_2S_2	824	0.0005	81	100
Cl_2Mg	7	0.0005	1	1
$CeLi_2P_2$	577	0.0006	73	57
Br_2La	403	0.0006	64	49
Br_2Er_2	724	0.0006	100	81
Bi_2STe_2	376	0.0006	49	36
$H_2I_2Yb_2$	548	0.0006	65	48
AsKSn	357	0.0006	57	43
Br_2La_2	580	0.0006	81	64
S_2V	388	0.0006	49	64
Cu_2I_2	796	0.0007	118	81
LiO	418	0.0007	64	81
$CrSe_2$	447	0.0008	57	73
$Cl_2H_2Lu_2$	10	0.0008	1	1
Cl_2OV	568	0.0008	72	70
C_2F_2	296	0.0008	25	49
Sb_2	452	0.0009	81	64
$Br_2H_2Sr_2$	958	0.0009	118	81
I_2Mg	403	0.0009	64	49
$Cu_2Se_2Tl_2$	958	0.0009	118	81

Table showing the first 50 lattice matching combinations with the smallest strain needed to build the supercell.

GaS (P-6m2)

Structural and electronic properties

**Formula**	GaS
**Spacegroup**	P-6m2
**Prototype**	GaS
**Parent 3D**	Ga_4S_4
**Source DB**	ICSD
**DB ID**	635254
**DF2-C09 Binding energy [meV/ Å^2]**	15.78
**RVV10 Binding energy [meV/ Å^2]**	20.59
**Band gap (PBE) [eV]**	2.34

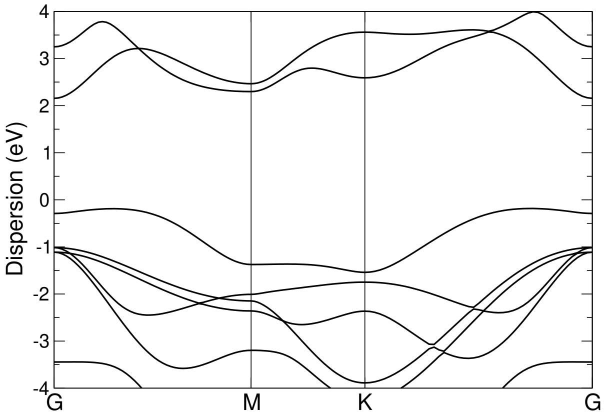

Band structure: Electronic band structure of GaS (P-6m2) in a representative window around the Fermi energy and along a high-symmetry path.

Optimized crystal structure

Structural parameters: relaxed cell (top) and atomic positions (bottom) of GaS (P-6m2) in Cartesian coordinates.

	x [Å]	y [Å]	z [Å]
$\mathbf{a_1}$	3.63321511	0.00000000	0.00000000
$\mathbf{a_2}$	−1.81660755	3.14645658	0.00000000
$\mathbf{a_3}$	0.00000000	0.00000000	24.62270720

		x [Å]	y [Å]	z [Å]
●	Ga	1.81660755	1.04881886	13.54668585
●	Ga	1.81660755	1.04881886	11.07602135
●	S	0.00000000	2.09763772	14.63030522
●	S	0.00000000	2.09763772	9.99240198

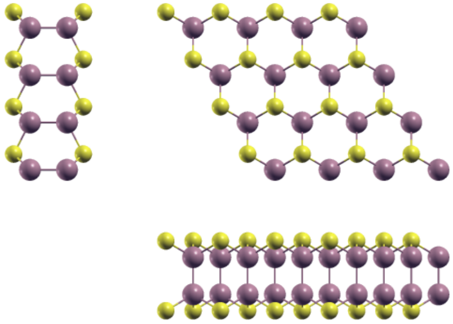

Orthographic projections: views of GaS (P-6m2) as seen from the x axis (left), the y axis (bottom) and the z axis (center).

Lattice matching - minimal size

Formula	N° atoms	strain	cell size 1	cell size 2
Tl	5	0.1191	1	1
In	5	0.4251	1	1
In	5	1.5976	1	1
InSe	6	3.0062	1	1
HgO	6	0.1263	1	1
As_2	6	0.0032	1	1
LiO	6	0.2597	1	1
P_2	6	0.2706	1	1
PbTe	6	3.0299	1	1
Mg_2	6	0.112	1	1
Sb_2	6	0.4809	1	1
Cl_2Zn	7	0.0042	1	1
I_2Mg	7	0.4958	1	1
S_2V	7	0.2498	1	1
MoS_2	7	1.6269	1	1
$MoTe_2$	7	0.0087	1	1
$AgTe_2$	7	0.1208	1	1
PSn_2	7	0.0058	1	1
HfS_2	7	0.0005	1	1
Te_2V	7	0.0069	1	1
$CuTe_2$	7	0.0008	1	1
S_2Zr	7	0.005	1	1
Br_2La	7	0.4967	1	1
Br_2Cu	7	1.0305	1	1
NiO_2	7	4.8694	1	1
Ca_2Si	7	13.6045	1	1
Br_2Co	7	0.0036	1	1
ReS_2	7	1.5341	1	1
Ca_2N	7	0.0027	1	1
Cl_2Ti	7	0.2708	1	1
$AuTe_2$	7	0.4609	1	1
$PdTe_2$	7	0.4549	1	1
Mg_3	7	0.1162	1	1
I_2Zn	7	0.4725	1	1
Te_2Zn	7	0.0089	1	1
S_2W	7	1.6274	1	1
Bi_2Pd	7	0.133	1	1
GeI_2	7	2.8474	1	1
Br_2Mn	7	0.0054	1	1
$CoTe_2$	7	0.0001	1	1
CdClO	7	0.0073	1	1
AsKSn	7	2.9363	1	1
Te_2W	7	0.0086	1	1
$PbTe_2$	7	0.5016	1	1
S_2Sn	7	0.0053	1	1
$SnTe_2$	7	0.485	1	1
Cl_2V	7	0.2563	1	1
GeI_2	7	3.0331	1	1
STl_2	7	2.9677	1	1
OTl_2	7	0.0072	1	1

Table showing the first 50 lattice matching combinations with the smallest number of atoms in the supercell.

Lattice matching - minimal strain

Formula	N° atoms	strain	cell size 1	cell size 2
Ca_2Si	471	0.0	81	49
$Ce_2I_2S_2$	618	0.0	81	49
As_2Li_2Nd	376	0.0	49	36
$Ce_2I_2Si_2$	550	0.0001	64	49
FeH_2O_2	280	0.0001	25	36
$AuTe_2$	643	0.0001	100	81
Cl_2La_2	656	0.0001	91	73
$F_2Lu_2Se_2$	708	0.0001	81	64
$CoTe_2$	7	0.0001	1	1
I_2Mg	403	0.0001	64	49
GeI_2Y_2	376	0.0002	49	36
Br_2Ca_3Si	196	0.0002	25	16
BrCdI	357	0.0002	57	43
$Ga_2Ge_2Te_2$	634	0.0002	73	57
SSb_2Te_2	443	0.0003	57	43
NiO_2	439	0.0003	49	81
$Br_2O_2V_2$	624	0.0003	66	60
$Bi_2Br_2O_2$	548	0.0003	65	48
Li_2P_2Pr	577	0.0003	73	57
LiO	418	0.0003	64	81
GdI_2	219	0.0004	36	25
S_2V	388	0.0004	49	64
$Gd_2I_2S_2$	196	0.0004	25	16
Sb_2Se_2Te	501	0.0004	64	49
$Au_2K_2S_2$	480	0.0004	90	20
Cl_2OV	568	0.0004	72	70
HfS_2	7	0.0005	1	1
Br_2Er_2	724	0.0005	100	81
H_2MgO_2	516	0.0005	49	64
Gd	305	0.0005	52	97
Br_2La	403	0.0005	64	49
$PtTe_2$	643	0.0005	100	81
FHOZn	520	0.0005	57	73
Bi_2Se_3	501	0.0006	64	49
Cl_2V	447	0.0006	57	73
$MnNaTe_2$	400	0.0006	57	43
$CrSe_2$	447	0.0006	57	73
AlH_4Na	276	0.0006	39	20
Ca_2Si	355	0.0006	61	37
$I_2La_2Si_2$	412	0.0006	49	36
$Ce_2I_2S_2$	466	0.0006	61	37
Bi_2Se_2Te	376	0.0007	49	36
Sb_2Te_2	392	0.0007	61	37
$SnTe_2$	463	0.0007	73	57
$I_2O_2Y_2$	548	0.0007	65	48
As_2Li_2Pr	376	0.0007	49	36
$Er_2I_2O_2$	548	0.0007	65	48
In	229	0.0007	43	57
InSe	268	0.0007	49	36
$AgNO_3$	511	0.0007	79	39

Table showing the first 50 lattice matching combinations with the smallest strain needed to build the supercell.

GaSe (P-6m2)

Structural and electronic properties

**Formula**	GaSe
**Spacegroup**	P-6m2
**Prototype**	GaS
**Parent 3D**	Ga_4Se_4
**Source DB**	COD
**DB ID**	9008968
**DF2-C09 Binding energy [meV/ Å^2]**	15.15
**RVV10 Binding energy [meV/ Å^2]**	20.4
**Band gap (PBE) [eV]**	1.78

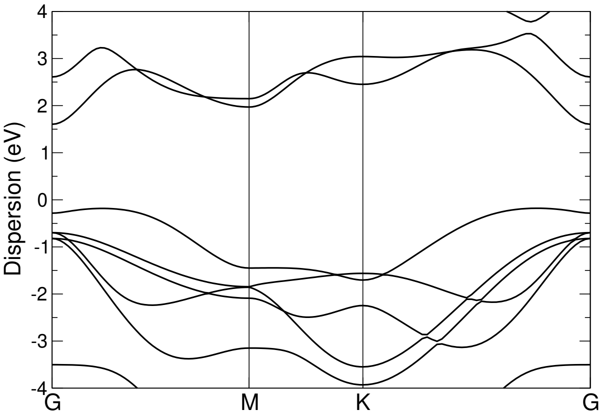

Band structure: Electronic band structure of GaSe (P-6m2) in a representative window around the Fermi energy and along a high-symmetry path.

Optimized crystal structure

Structural parameters: relaxed cell (top) and atomic positions (bottom) of GaSe (P-6m2) in Cartesian coordinates.

	x [Å]	y [Å]	z [Å]
$\mathbf{a_1}$	3.81795280	0.00000000	0.00000000
$\mathbf{a_2}$	−1.90897640	3.30644411	0.00000000
$\mathbf{a_3}$	0.00000000	0.00000000	24.79287445

		x [Å]	y [Å]	z [Å]
●	Ga	0.00000000	2.20429607	11.16226528
●	Ga	0.00000000	2.20429607	13.63060917
●	Se	1.90897640	1.10214804	9.98946647
●	Se	1.90897640	1.10214804	14.80340798

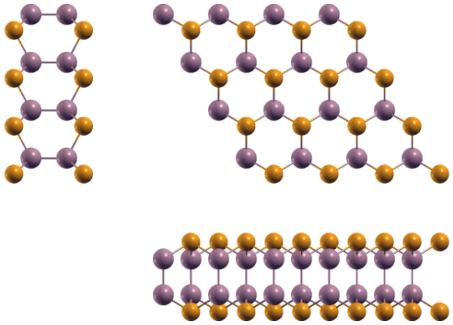

Orthographic projections: views of GaSe (P-6m2) as seen from the x axis (left), the y axis (bottom) and the z axis (center).

Lattice matching - minimal size

Formula	N° atoms	strain	cell size 1	cell size 2
Tl	5	1.5976	1	1
InSe	6	0.462	1	1
Nd	6	0.2239	1	2
HgO	6	0.1139	1	1
AsSb	6	0.0032	1	1
Bi_2	6	0.4766	1	1
GeTe	6	0.0094	1	1
P_2	6	1.5591	1	1
PbTe	6	0.4664	1	1
CaCl	6	0.1464	1	1
$CdCl_2$	7	0.0083	1	1
Cl_2Mn	7	1.6406	1	1
CdI_2	7	0.4708	1	1
$AgTe_2$	7	0.4423	1	1
$MoSe_2$	7	1.592	1	1
$ReSe_2$	7	0.2716	1	1
S_2Ta	7	0.256	1	1
Br_2Zn	7	0.0069	1	1
Br_2Ca	7	0.4739	1	1
CaI_2	7	3.0444	1	1
$InSe_2$	7	0.0087	1	1
$GeTe_2$	7	0.0073	1	1
$SiTe_2$	7	0.0029	1	1
I_2Mn	7	0.0084	1	1
NSr_2	7	0.0048	1	1
I_2Yb	7	3.0102	1	1
PbS_2	7	0.0006	1	1
BiClTe	7	0.4717	1	1
Cl_2Ti	7	1.5602	1	1
FeI_2	7	0.0061	1	1
I_2Ni	7	0.0075	1	1
S_2Ti	7	0.2629	1	1
Mg_3	7	0.4221	1	1
Te_2Ti	7	0.0066	1	1
NbS_2	7	0.2554	1	1
CrI_2	7	0.0058	1	1
BaF_2	7	0.4525	1	1
BiBrTe	7	0.4859	1	1
Bi_2Pd	7	0.1182	1	1
Cl_2Ni	7	0.2729	1	1
Cl_2Co	7	0.2624	1	1
NbS_2	7	0.2496	1	1
Br_2V	7	0.2704	1	1
ClNZr	7	0.2674	1	1
Cl_2Fe	7	0.2614	1	1
S_2Ta	7	0.2485	1	1
Se_2V	7	0.2467	1	1
AsKSn	7	0.449	1	1
$AsSe_2$	7	0.2751	1	1
$NiTe_2$	7	0.0035	1	1

Table showing the first 50 lattice matching combinations with the smallest number of atoms in the supercell.

Lattice matching - minimal strain

Formula	N° atoms	strain	cell size 1	cell size 2
S_2V	208	0.0	25	36
GeI_2	583	0.0	91	73
$MoSe_2$	343	0.0	43	57
NbS_2	447	0.0001	57	73
Se_2W	343	0.0001	43	57
ClNZr	624	0.0001	81	100
As_2CeLi_2	729	0.0002	91	73
$Ga_2Gd_2I_2$	708	0.0002	81	64
PbTe	510	0.0002	91	73
$HNiO_2$	392	0.0002	37	61
I_2La_2Sb	269	0.0002	36	25
Bi_2	452	0.0002	81	64
Cu_4Te_2	634	0.0002	73	57
$I_2La_2Si_2$	886	0.0003	100	81
NbS_2	388	0.0003	49	64
As_2Li_2Pr	805	0.0003	100	81
InSe	562	0.0003	100	81
As_2Li_2Nd	805	0.0003	100	81
$LiMnTe_2$	656	0.0003	91	73
Br_2Y_2	8	0.0004	1	1
S_2Ta	447	0.0004	57	73
$Br_2H_2Zr_2$	924	0.0004	81	100
As_2O_3	511	0.0004	79	39
$Ga_2I_2Tb_2$	708	0.0005	81	64
$LiNbS_2$	520	0.0005	57	73
N_2Re	84	0.0005	9	16
$Cl_2O_2Y_2$	10	0.0005	1	1
$Cl_2H_2Zr_2$	580	0.0005	49	64
BiITe	357	0.0005	57	43
GeI_2Y_2	805	0.0006	100	81
K	169	0.0006	36	25
PbS_2	7	0.0006	1	1
S_2Ta	388	0.0006	49	64
ClH_3O	180	0.0006	25	16
Bi_2Te_3	443	0.0006	57	43
Cl_2ORu	572	0.0006	73	70
$AsLi_3$	724	0.0006	100	81
Bi_2STe_2	729	0.0007	91	73
Tl	229	0.0007	43	57
GdI_2	403	0.0007	64	49
$H_2I_2Sr_2$	958	0.0008	118	81
MoS_2	208	0.0008	25	36
CCl_2Sc_2	824	0.0008	81	100
S_2W	208	0.0008	25	36
Sb_2Te_3	577	0.0008	73	57
Bi_2SeTe_2	644	0.0008	81	64
Br_2Ca	516	0.0008	81	64
Ba_2Cu_2	340	0.0008	49	36
Ca_4Cu_2	696	0.0008	99	50
H_2MgO_2	280	0.0009	25	36

Table showing the first 50 lattice matching combinations with the smallest strain needed to build the supercell.

GaTe (P-6m2)

Structural and electronic properties

**Formula**	GaTe
**Spacegroup**	P-6m2
**Prototype**	GaS
**Parent 3D**	Ga_4Te_4
**Source DB**	ICSD
**DB ID**	43328
**DF2-C09 Binding energy [meV/ Å^2]**	15.71
**RVV10 Binding energy [meV/ Å^2]**	20.61
**Band gap (PBE) [eV]**	1.4

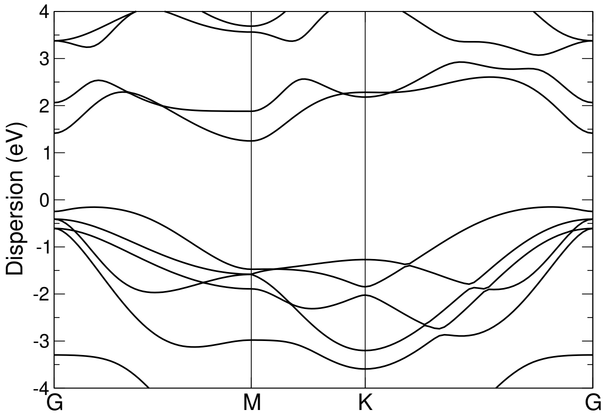

Band structure: Electronic band structure of GaTe (P-6m2) in a representative window around the Fermi energy and along a high-symmetry path.

Optimized crystal structure

Structural parameters: relaxed cell (top) and atomic positions (bottom) of GaTe (P-6m2) in Cartesian coordinates.

		x [Å]	y [Å]	z [Å]
$\mathbf{a_1}$		4.13535057	0.00000000	0.00000000
$\mathbf{a_2}$		−2.06767528	3.58131865	0.00000000
$\mathbf{a_3}$		0.00000000	0.00000000	25.06293218
		x [Å]	y [Å]	z [Å]
●	Ga	2.06767528	1.19377288	11.29704469
●	Ga	2.06767528	1.19377288	13.76588749
●	Te	0.00000000	2.38754576	10.02533380
●	Te	0.00000000	2.38754576	15.03759838

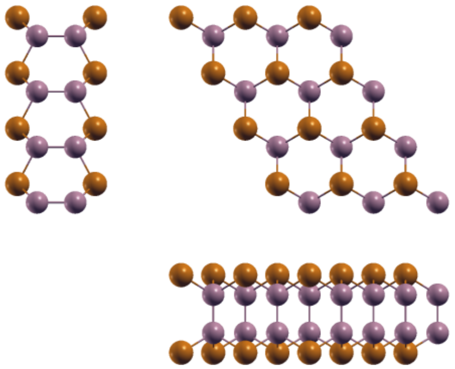

Orthographic projections: views of GaTe (P-6m2) as seen from the x axis (left), the y axis (bottom) and the z axis (center).

Lattice matching - minimal size

Formula	N° atoms	strain	cell size 1	cell size 2
K	5	0.4563	1	1
Na	5	1.5505	1	1
AgTl	6	0.1653	1	1
Ag_2	6	0.4701	1	1
As_2	6	0.2476	1	1
Sb_2	6	0.0036	1	1
CaCl	6	0.1191	1	1
Cl_2Zn	7	0.2462	1	1
I_2Mg	7	0.0021	1	1
PSn_2	7	0.2596	1	1
Ba_2Pt	7	0.4694	1	1
Br_2Zn	7	0.2749	1	1
HfS_2	7	1.6293	1	1
$AsSn_2$	7	0.2649	1	1
Te_2V	7	1.5809	1	1
I_2Pr	7	0.1471	1	1
S_2Zr	7	0.2585	1	1
Br_2La	7	0.0024	1	1
Ca_2Si	7	0.481	1	1
Br_2Co	7	0.247	1	1
Ca_2N	7	0.2483	1	1
BrCdI	7	0.0053	1	1
Cl_2Zn	7	0.1278	1	1
Te_2Ti	7	0.2754	1	1
I_2Zn	7	0.0068	1	1
BaF_2	7	0.0087	1	1
$RhTe_2$	7	0.2689	1	1
GeI_2	7	0.0002	1	1
Br_2Mn	7	1.5922	1	1
PtS_2	7	1.558	1	1
$CoTe_2$	7	1.6317	1	1
Se_2Ti	7	1.5389	1	1
AsKSn	7	0.0072	1	1
$PbTe_2$	7	0.0043	1	1
I_2Nd	7	0.148	1	1
S_2Sn	7	0.2589	1	1
$SnTe_2$	7	0.002	1	1
Sn	7	0.6342	1	3
Cl_2V	7	4.8694	1	1
I_2Pb	7	0.4741	1	1
$PtSe_2$	7	0.2658	1	1
Br_2Fe	7	0.2471	1	1
GeS_2	7	0.1135	1	1
$TaTe_2$	7	0.2641	1	1
$MnSe_2$	7	0.1191	1	1
Br_2Ni	7	0.2538	1	1
CeI_2	7	0.1464	1	1
CuO_2	7	0.1752	1	1
$NbTe_2$	7	0.2582	1	1
Se_2Yb	7	0.0005	1	1

Table showing the first 50 lattice matching combinations with the smallest number of atoms in the supercell.

Lattice matching - minimal strain

Formula	N° atoms	strain	cell size 1	cell size 2
Ba_2Ni_3	9	0.0	1	1
Br_2Mn	343	0.0	43	57
$I_2Pr_2Si_2$	10	0.0001	1	1
$Br_2Hf_2N_2$	580	0.0001	49	64
ReS_2	84	0.0001	9	16
CdClHO	724	0.0002	81	100
$TaTe_2$	565	0.0002	73	91
Cl_2Zr_2	244	0.0002	25	36
PSn_2	499	0.0002	64	81
HgI_2	404	0.0002	65	48
GeI_2	7	0.0002	1	1
$AsCuLi_2$	8	0.0003	1	1
Cl_2V	439	0.0003	49	81
S_2Sn	499	0.0003	64	81
$CrSe_2$	331	0.0003	37	61
KS_2Ti	400	0.0004	43	57
$AsSn_2$	565	0.0004	73	91
$Cl_2N_2Zr_2$	580	0.0004	49	64
Se_2Yb	7	0.0005	1	1
Na	193	0.0005	36	49
ClH_3O	443	0.0005	57	43
$AgNO_3$	180	0.0005	25	16
S_2Zr	499	0.0006	64	81
CCl_2Sc_2	280	0.0006	25	36
Br_2V	208	0.0006	25	36
Sb_2Te_2	580	0.0006	81	64
CaH_2O_2	516	0.0006	49	64
In	368	0.0007	65	108
$AgBrO_2$	332	0.0007	41	42
Br_2HLa	8	0.0007	1	1
$BiTe_2$	7	0.0007	1	1
$Hf_2I_2N_2$	838	0.0007	73	91
$Br_2N_2Zr_2$	666	0.0007	57	73
CdClHO	656	0.0007	73	91
Ca_2N	388	0.0007	49	64
I_2Pb	516	0.0008	81	64
$NbTe_2$	499	0.0008	64	81
CBr_2Lu_2	593	0.0008	57	73
MnO_2	43	0.0008	4	9
Te_4W_2	672	0.0008	84	56
$Cl_2H_2Lu_2$	666	0.0008	57	73
Ho_2S_2	316	0.0008	40	39
Au_2I_2	744	0.0008	105	81
$Ce_2I_2S_2$	634	0.0009	73	57
Ga_2S_3	389	0.0009	36	49
Ca_2Si	463	0.0009	73	57
CoH_2O_2	116	0.0009	9	16
Cl_2V	331	0.0009	37	61
NS_2Zr	340	0.0009	36	49
$RhTe_2$	624	0.0009	81	100

Table showing the first 50 lattice matching combinations with the smallest strain needed to build the supercell.

GaTeCl ($Pmn2_1$)

Structural and electronic properties

**Formula**	GaTeCl
**Spacegroup**	$Pmn2_1$
**Prototype**	GaTeCl
**Parent 3D**	$Ga_4Te_4Cl_4$
**Source DB**	COD
**DB ID**	7221395
**DF2-C09 Binding energy [meV/ Å^2]**	14.27
**RVV10 Binding energy [meV/ Å^2]**	20.83
**Band gap (PBE) [eV]**	2.28

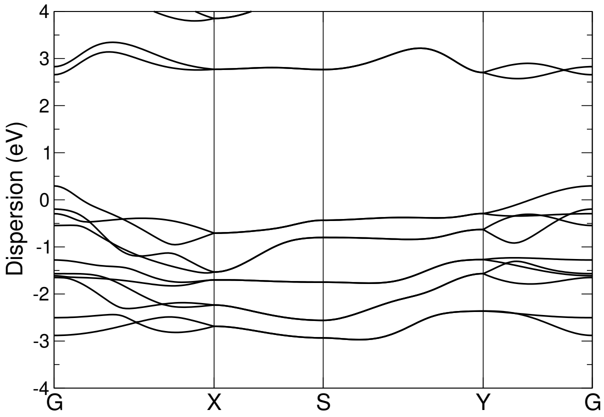

Band structure: Electronic band structure of GaTeCl ($Pmn2_1$) in a representative window around the Fermi energy and along a high-symmetry path.

Optimized crystal structure

Structural parameters: relaxed cell (top) and atomic positions (bottom) of GaTeCl ($Pmn2_1$) in Cartesian coordinates.

	x [Å]	y [Å]	z [Å]
$\mathbf{a_1}$	4.08110504	0.00000000	0.00000000
$\mathbf{a_2}$	0.00000000	5.98359074	0.00000000
$\mathbf{a_3}$	0.00000000	0.00000000	25.09508195

		x [Å]	y [Å]	z [Å]
●	Ga	0.00000000	0.09177691	13.46761273
●	Ga	2.04055252	3.08355260	11.62748338
●	Te	0.00000000	1.38859714	11.15643054
●	Te	2.04055252	4.38041954	13.93864351
●	Cl	0.00000000	1.43384525	15.20722649
●	Cl	2.04055252	4.42562506	9.88787038

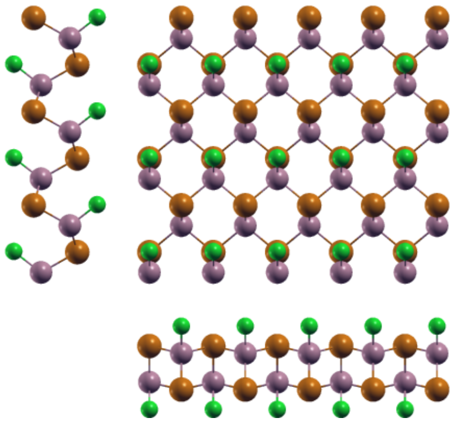

Orthographic projections: views of GaTeCl ($Pmn2_1$) as seen from the x axis (left), the y axis (bottom) and the z axis (center).

Lattice matching - minimal size

Formula	N° atoms	strain	cell size 1	cell size 2
AsSb	8	0.1226	1	1
GeTe	8	0.1217	1	1
Tl	8	0.2927	1	2
Na	8	0.145	1	2
$IrTe_2$	9	0.1216	1	1
$CdCl_2$	9	0.1218	1	1
$InSe_2$	9	0.1218	1	1
I_2Mn	9	0.1218	1	1
NSr_2	9	0.1223	1	1
FeI_2	9	0.1221	1	1
I_2Ni	9	0.122	1	1
CrI_2	9	0.1222	1	1
CoI_2	9	0.1225	1	1
Br_2Mg	9	0.1222	1	1
CNNa	9	0.9555	1	1
Se_2Sn	9	0.1224	1	1
Ba_2Cd	9	0.2672	1	1
H_2Si_2	10	0.1218	1	1
Au_2Br_2	10	0.2663	1	1
As_4	10	0.9648	1	1
Br_3Cs	10	0.3701	1	1
As_2	10	0.1512	1	2
S_2	10	0.6282	1	2
P_2	10	0.2845	1	2
Ni_2Te_2	10	0.1219	1	1
In	10	0.2585	1	4
I_3Sn	10	0.3892	1	1
Hg_3N_2	11	0.3988	1	1
PTe_2Zr_2	11	0.1227	1	1
FKO_2Se	11	0.0342	1	1
$ClKO_3$	11	0.3776	1	1
In_2S_3	11	0.1217	1	1
$BrKO_3$	11	0.4094	1	1
Cl_2Zn	12	0.1504	1	2
$Br_2Ho_2S_2$	12	0.0362	1	1
$I_2Lu_2Se_2$	12	0.0072	1	1
Cl_2Mn	12	0.1292	1	2
$MoTe_2$	12	0.1467	1	2
PSn_2	12	0.159	1	2
$MoSe_2$	12	0.2915	1	2
$ReSe_2$	12	0.1365	1	2
$Ho_2I_2S_2$	12	0.4001	1	1
S_2Ta	12	0.1302	1	2
HfS_2	12	0.1535	1	2
$AsSn_2$	12	0.1625	1	2
$GeTe_2$	12	0.6166	1	2
Te_2V	12	0.1482	1	2
$CuTe_2$	12	0.1532	1	2
S_2Zr	12	0.1583	1	2
Br_2Cu	12	0.1306	1	2

Table showing the first 50 lattice matching combinations with the smallest number of atoms in the supercell.

Lattice matching - minimal strain

Formula	N° atoms	strain	cell size 1	cell size 2
$H_2Na_2O_2$	372	0.0001	20	42
$Br_2O_2Sc_2$	228	0.0005	14	24
Bi_2	462	0.001	51	78
Cl_2OV	172	0.0011	12	25
Br_2Ca	540	0.0012	51	78
N_3Na	436	0.0012	40	49
AsI_2La_2	779	0.0012	59	85
AsI_2La_2	752	0.0012	57	82
Cl_4Mn	969	0.0012	84	93
$I_2Nd_2S_2$	504	0.0012	36	48
CNNa	768	0.0012	74	108
CuO_2	273	0.0013	20	51
$Cl_2O_2Sc_2$	198	0.0013	12	21
$Ag_2K_2Te_2$	348	0.0013	28	30
K_2PtS_2	578	0.0013	63	40
$HNiO_2$	452	0.0013	24	77
Fe_2S_2	286	0.0014	21	40
Bi_2SeTe_2	696	0.0014	51	78
Bi_2SeTe_2	626	0.0015	46	70
$Br_2Ho_2S_2$	102	0.0015	8	9
Bi_2In_2	622	0.0016	59	67
Bi_2	416	0.0017	46	70
$Cu_2Rb_2Te_2$	756	0.0017	59	67
I_2Pr	540	0.0017	51	78
Bi_2Te_2	916	0.0018	84	103
Sb_2Te_3	626	0.0018	46	70
BiClTe	594	0.0018	56	86
BiClTe	540	0.0018	51	78
CaCl	880	0.0018	90	170
Sm	283	0.0019	28	115
Br_2CsF	912	0.0019	86	99
CdI_2	594	0.0019	56	86
$Br_2Dy_2S_2$	102	0.0019	8	9
AuI_4Li	996	0.002	94	72
Cl_2ORu	776	0.002	58	107
CuO_2	192	0.002	14	36
O_2Sn_2	528	0.002	38	75
C_2Li_2	348	0.002	28	45
$F_2Se_2Y_2$	858	0.002	65	78
Br_2La_2P	766	0.0021	56	86
$Cl_2N_2Zr_2$	972	0.0021	61	101
I_2La_2Sb	456	0.0021	36	48
CdI_2	540	0.0021	51	78
Cu_4Te_2	696	0.0021	46	70
CdI_2	594	0.0022	56	86
$H_2Li_2O_2$	366	0.0022	21	40
Fe_2Se_2	914	0.0022	69	125
CNRb	669	0.0022	73	77
Cl_2OOs	528	0.0022	40	72
Cu_2Te_2	108	0.0023	8	15

Table showing the first 50 lattice matching combinations with the smallest strain needed to build the supercell.

Gd (Pmmm)

Structural and electronic properties

**Formula**	Gd
**Spacegroup**	Pmmm
**Prototype**	Sn
**Parent 3D**	Gd
**Source DB**	COD
**DB ID**	1526591
**DF2-C09 Binding energy [meV/ Å^2]**	0.38
**RVV10 Binding energy [meV/ Å^2]**	N/A
**Band gap (PBE) [eV]**	0.0

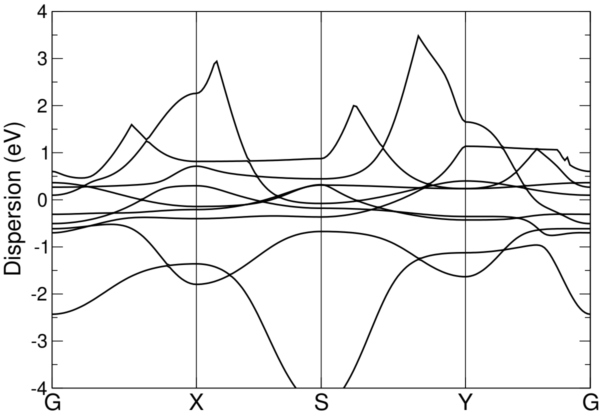

Band structure: Electronic band structure of Gd (Pmmm) in a representative window around the Fermi energy and along a high-symmetry path.

Optimized crystal structure

Structural parameters: relaxed cell (top) and atomic positions (bottom) of Gd (Pmmm) in Cartesian coordinates.

	x [Å]	y [Å]	z [Å]
$\mathbf{a_1}$	2.30073702	0.00000000	0.00000000
$\mathbf{a_2}$	0.00000000	2.65870454	0.00000000
$\mathbf{a_3}$	0.00000000	0.00000000	12.00000000

		x [Å]	y [Å]	z [Å]
●	Gd	0.00000000	-1.32935227	0.00000000

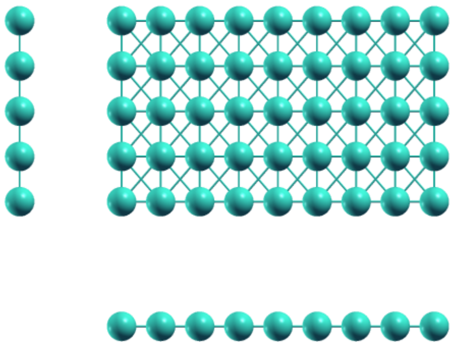

Orthographic projections: views of Gd (Pmmm) as seen from the x axis (left), the y axis (bottom) and the z axis (center).

Lattice matching - minimal size

Formula	N° atoms	strain	cell size 1	cell size 2
Na	2	0.3586	1	1
In	3	3.4061	1	2
$MoTe_2$	4	0.3637	1	1
CrO_2	4	0.5752	1	1
$AuTe_2$	4	1.6782	1	1
LiO_2	4	0.321	1	1
$PdTe_2$	4	1.6593	1	1
Te_2Zn	4	0.3633	1	1
N_2W	4	0.2184	1	1
$CrTe_2$	4	0.3468	1	1
PtS_2	4	0.3609	1	1
Se_2Ti	4	0.355	1	1
Br_2Ti	4	0.3455	1	1
Br_2Cr	4	0.3461	1	1
$FeSe_2$	4	1.0643	1	1
$PtTe_2$	4	1.6751	1	1
Br_2Cd	4	1.6522	1	1
NS_2Zr	5	0.3595	1	1
$HNiO_2$	5	0.2077	1	1
Br_2Tb_2	5	1.6638	1	1
Br_2Zr_2	5	0.3542	1	1
Bi_2Se_2	5	3.415	1	1
Cl_2Cu	5	0.1401	2	1
As_2	5	2.1269	1	2
Br_2Ho_2	5	1.6677	1	1
Na	5	0.1347	4	1
O_2Sn_2	5	5.4162	1	1
Cl_2Sc_2	5	0.3466	1	1
$HfLiS_2$	5	0.3626	1	1
Br_2PY_2	6	1.6508	1	1
BiBrTe	6	0.1256	3	1
N_2W	6	0.1547	3	1
Bi_2S_3	6	1.6658	1	1
GdI_2	6	0.1274	3	1
Ga_2S_3	6	0.3594	1	1
Cl_2Zn	7	2.1168	1	2
$MoTe_2$	7	0.1345	4	1
PSn_2	7	2.2152	1	2
Cu_2Sr_2	7	0.1262	3	1
$Br_2H_2Zr_2$	7	0.3459	1	1
HfS_2	7	2.1537	1	2
$CuTe_2$	7	2.1501	1	2
S_2Zr	7	2.2075	1	2
Br_2Co	7	2.1228	1	2
CrO_2	7	0.1747	4	1
Ca_2N	7	2.132	1	2
$AlLiTe_2$	7	0.129	3	1
Te_2Zn	7	0.1345	4	1
Br_2Mn	7	0.2021	4	1
PtS_2	7	0.1346	4	1

Table showing the first 50 lattice matching combinations with the smallest number of atoms in the supercell.

Lattice matching - minimal strain

Formula	N° atoms	strain	cell size 1	cell size 2
Br_2Zr_2	248	0.0002	76	43
HfS_2	253	0.0002	97	52
MnO_2	204	0.0002	54	50
C_2F_2	377	0.0002	73	76
$F_2Ho_2Se_2$	314	0.0002	86	38
PTe_2Zr_2	85	0.0003	25	12
Br_2V	75	0.0003	27	16
$CoTe_2$	253	0.0004	97	52
$F_2Lu_2Se_2$	226	0.0004	64	27
$CuTe_2$	253	0.0004	97	52
Se_2Ti	205	0.0004	76	43
Ga_2S_2	305	0.0005	97	52
Sn	153	0.0005	90	63
$I_2Nd_2S_2$	18	0.0005	6	2
$F_2Se_2Tm_2$	402	0.0006	108	49
$ReSe_2$	75	0.0006	27	16
$As_2Fe_2Li_2$	382	0.0006	106	46
$Br_2H_2Yb_2$	382	0.0006	106	46
$LiMnSe_2$	304	0.0007	108	49
PSn_2	123	0.0007	48	25
Fe_2SeTe	290	0.0007	106	46
CaH_2O_2	357	0.0009	97	52
Nd	149	0.0009	76	73
CrS_2	13	0.0009	4	3
Br_2PY_2	276	0.0009	86	38
AsSb	49	0.001	25	12
$Br_2Cr_2O_2$	427	0.0011	109	53
Mg_4	242	0.0011	70	43
Cl_2Zr_2	91	0.0011	27	16
$Cl_2N_2Zr_2$	409	0.0011	97	52
Br_2Cd	200	0.0011	86	38
S_2Sn	123	0.0012	48	25
Br_2La_2	172	0.0012	64	27
$I_2S_2Yb_2$	48	0.0012	18	5
I_2Y_2	304	0.0013	108	49
$I_2S_2Tm_2$	48	0.0013	18	5
$Br_2Lu_2O_2$	382	0.0013	106	46
Au_2Br_2	244	0.0013	104	35
Tl	113	0.0013	70	43
CoI_2	61	0.0014	25	12
Sb_2	118	0.0014	64	27
S_2Zr	123	0.0014	48	25
Cl_2Ni	75	0.0015	27	16
CCl_2Sc_2	107	0.0015	27	16
$GeNi_3Te_2$	402	0.0015	108	49
Cl_2Cu	126	0.0015	51	25
$Ge_2Te_2Zr_2$	382	0.0015	106	46
Ga_2S_2	305	0.0015	97	52
$Br_2Cr_2O_2$	394	0.0015	100	49
$Br_2Hf_2N_2$	409	0.0016	97	52

Table showing the first 50 lattice matching combinations with the smallest strain needed to build the supercell.

Gd_2Br_2 (P-3m1)

Structural and electronic properties

**Formula**	Gd_2Br_2
**Spacegroup**	P-3m1
**Prototype**	PtTe
**Parent 3D**	Br_2Gd_2
**Source DB**	ICSD
**DB ID**	47226
**DF2-C09 Binding energy [meV/ Å^2]**	12.2
**RVV10 Binding energy [meV/ Å^2]**	N/A
**Band gap (PBE) [eV]**	0.0

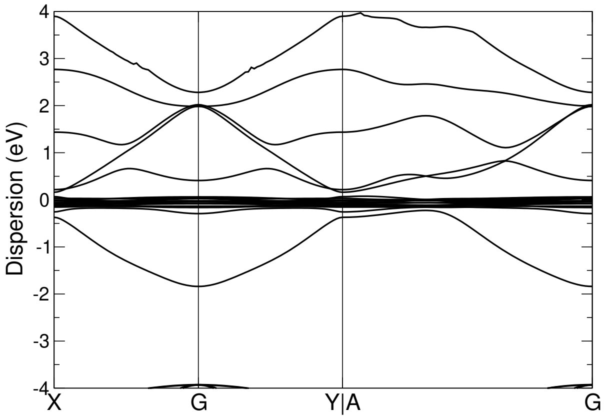

Band structure: Electronic band structure of Gd_2Br_2 (P-3m1) in a representative window around the Fermi energy and along a high-symmetry path.

Optimized crystal structure

Structural parameters: relaxed cell (top) and atomic positions (bottom) of Gd_2Br_2 (P-3m1) in Cartesian coordinates.

	x [Å]	y [Å]	z [Å]
$\mathbf{a_1}$	2.02282383	−3.50363365	0.00000000
$\mathbf{a_2}$	2.02263009	3.50352179	0.00000000
$\mathbf{a_3}$	0.00000000	0.00000000	25.43955707

	x [Å]	y [Å]	z [Å]
Gd	1.01121508	2.91958106	1.54836132
Br	1.01140166	0.58393302	3.36130675
Gd	1.01141501	0.58394073	-1.54836132
Br	1.01122843	2.91958877	-3.36130675

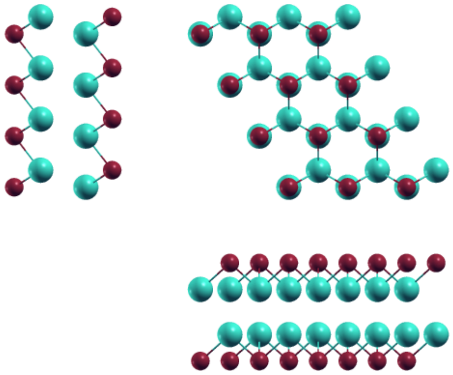

Orthographic projections: views of Gd_2Br_2 (P-3m1) as seen from the x axis (left), the y axis (bottom) and the z axis (center).

Lattice matching - minimal size

Formula	N° atoms	strain	cell size 1	cell size 2
K	5	0.4826	1	1
Ag_2	6	0.497	1	1
As_2	6	0.2616	1	1
Sb_2	6	0.0069	1	1
CaCl	6	0.1251	1	1
Cl_2Zn	7	0.2602	1	1
$MoTe_2$	7	0.2538	1	1
PSn_2	7	0.2745	1	1
Ba_2Pt	7	0.4963	1	1
HfS_2	7	0.2655	1	1
CaI_2	7	0.4593	1	1
$HfTe_2$	7	0.0087	1	1
Te_2V	7	0.2564	1	1
$CuTe_2$	7	0.265	1	1
S_2Zr	7	0.2733	1	1
I_2Yb	7	0.453	1	1
Br_2Co	7	0.261	1	1
Ca_2N	7	0.2624	1	1
$AuTe_2$	7	0.0011	1	1
Cl_2Zn	7	0.1354	1	1
$PdTe_2$	7	0.0035	1	1
I_2Zn	7	0.0035	1	1
Te_2Zn	7	0.2536	1	1
Bi_2Pd	7	0.4226	1	1
Br_2Mn	7	0.2585	1	1
$CoTe_2$	7	0.2659	1	1
CdClO	7	0.2557	1	1
Ba_2N	7	0.0072	1	1
Se_2Ti	7	0.2486	1	1
Te_2Zr	7	0.0082	1	1
Te_2W	7	0.254	1	1
I_2Tm	7	0.4564	1	1
S_2Sn	7	0.2737	1	1
$SnTe_2$	7	0.0085	1	1
I_2Pb	7	0.5012	1	1
OTl_2	7	0.256	1	1
Br_2Fe	7	0.2611	1	1
GeS_2	7	0.1182	1	1
$MnSe_2$	7	0.1251	1	1
DyI_2	7	0.4663	1	1
Br_2Ni	7	0.2683	1	1
CuO_2	7	0.0917	1	1
$NbTe_2$	7	0.273	1	1
Cl_2Mg	7	0.2684	1	1
F_2Ni	7	0.1323	1	1
$PtTe_2$	7	0.0015	1	1
Br_2Cd	7	0.0044	1	1
F_2Zn	7	0.1533	1	1
Fe_2Te_2	8	0.145	1	1
Ca_2Cl_2	8	0.1454	1	1

Table showing the first 50 lattice matching combinations with the smallest number of atoms in the supercell.

Lattice matching - minimal strain

Formula	N° atoms	strain	cell size 1	cell size 2
CaH_2O_2	747	0.0	73	91
Ga_2S_2	724	0.0	81	100
Sb_2Te_2	400	0.0001	57	43
As_2Sn_2	8	0.0001	1	1
O_2Pt	331	0.0001	37	61
Se_2Ta	291	0.0001	36	49
I_2La_2Sb	577	0.0001	73	57
I_2La_2Te	644	0.0002	81	64
KS_2Ti	580	0.0002	64	81
CdClO	447	0.0002	57	73
$NbSe_2$	291	0.0002	36	49
$I_2S_2Tb_2$	802	0.0002	91	73
DyI_2	583	0.0002	91	73
K	349	0.0002	73	57
$Cl_2N_2Zr_2$	838	0.0002	73	91
$F_2Se_2Y_2$	294	0.0003	36	25
NbS_2	208	0.0003	25	36
Br_2Ca_3Si	708	0.0003	81	64
Ba_2Pt	403	0.0003	64	49
PTe_2Ti_2	824	0.0004	81	100
OTl_2	447	0.0004	57	73
$Cl_2Hf_2N_2$	666	0.0004	57	73
Br_2Ca_3Si	324	0.0004	45	24
$CuTe_2$	565	0.0004	73	91
Se_2Ti	388	0.0005	49	64
Br_2Ni	624	0.0005	81	100
Li_2Tl_2	164	0.0005	25	16
Br_2Er_2	8	0.0005	1	1
Cl_2Mg	624	0.0006	81	100
Br_2Mn	499	0.0006	64	81
$I_2S_2Sm_2$	634	0.0006	73	57
Ag_2	354	0.0006	64	49
Cl_2Zn	499	0.0006	64	81
Cl_2Ni	291	0.0006	36	49
CdH_2O_2	593	0.0006	57	73
$SbSe_2Tl$	492	0.0007	75	48
LiO	114	0.0007	16	25
Te_2V	447	0.0007	57	73
S_2Ta	208	0.0007	25	36
$CrTe_2$	343	0.0008	43	57
CaI_2	643	0.0008	100	81
$Br_2Hf_2N_2$	838	0.0008	73	91
$LiNbS_2$	244	0.0008	25	36
$Br_2Hf_2N_2$	742	0.0008	76	73
HfS_2	565	0.0008	73	91
$AsSe_2$	291	0.0008	36	49
$Br_2N_2Ti_2$	468	0.0008	45	48
Cl_2Sc_2	400	0.0008	43	57
Br_2Zr_2	452	0.0009	49	64
Cl_2NSc_2	280	0.0009	25	36

Table showing the first 50 lattice matching combinations with the smallest strain needed to build the supercell.

$Gd_2C_2Br_2$ (C2/m)

Structural and electronic properties

**Formula**	$Gd_2C_2Br_2$
**Spacegroup**	C2/m
**Prototype**	Gd2C2Br2
**Parent 3D**	$C_2Br_2Gd_2$
**Source DB**	MPDS
**DB ID**	S1703258
**DF2-C09 Binding energy [meV/ Å^2]**	11.67
**RVV10 Binding energy [meV/ Å^2]**	N/A
**Band gap (PBE) [eV]**	0.0

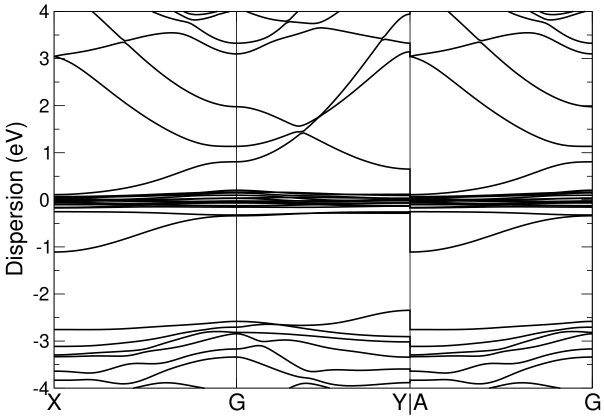

Band structure: Electronic band structure of $Gd_2C_2Br_2$ (C2/m) in a representative window around the Fermi energy and along a high-symmetry path.

Optimized crystal structure

Structural parameters: relaxed cell (top) and atomic positions (bottom) of $Gd_2C_2Br_2$ (C2/m) in Cartesian coordinates.

	x [Å]	y [Å]	z [Å]
$\mathbf{a_1}$	0.00000000	3.87989008	0.00000000
$\mathbf{a_2}$	3.58254843	1.93994504	0.00000000
$\mathbf{a_3}$	0.00000000	0.00000000	25.16182328

		x [Å]	y [Å]	z [Å]
●	Gd	0.91298216	1.93994504	1.48536761
●	C	-0.55630871	-0.00000000	0.34944289
●	Br	-1.47839711	1.93994504	3.31513348
●	Gd	-0.91298216	1.93994504	-1.48536761
●	C	0.55630871	0.00000000	-0.34944289
●	Br	1.47839711	1.93994504	-3.31513348

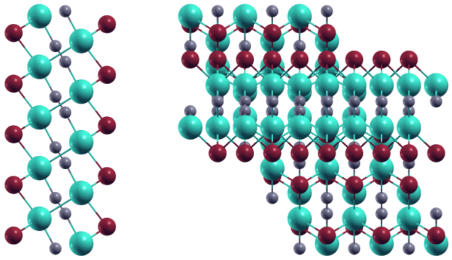

Orthographic projections: views of $Gd_2C_2Br_2$ (C2/m) as seen from the x axis (left), the y axis (bottom) and the z axis (center).

Lattice matching - minimal size

Formula	N° atoms	strain	cell size 1	cell size 2
In	7	0.1883	1	1
HgO	8	0.3895	1	1
AgTl	8	0.1745	1	1
CaCl	8	0.1205	1	1
Sm	8	0.1626	1	2
Br_2Cu	9	0.2811	1	1
Cl_2Zn	9	0.1308	1	1
GeS_2	9	0.1137	1	1
$MnSe_2$	9	0.1205	1	1
CNNa	9	0.2166	1	1
F_2Ni	9	0.1277	1	1
O_2Pt	9	0.1871	1	1
Cu_2I_2	10	0.1777	1	1
Cl_2OOs	10	0.1274	1	1
Cu_2O_2	10	0.9745	1	1
$AgCuTe_2$	10	0.1554	1	1
O_2Sn_2	10	0.1517	1	1
Au_2Br_2	10	0.2139	1	1
Cl_2OV	10	0.1132	1	1
Fe_2Se_2	10	0.1273	1	1
Cl_2ORu	10	0.1237	1	1
As_2Co_2	10	0.1238	1	1
Cu_2Te_2	10	0.1328	1	1
O_2Pb_2	10	0.1736	1	1
Ge_2S_2	10	0.1828	1	1
As_4	10	0.2212	1	1
Br_2OV	10	0.1175	1	1
Au_2Se_2	10	1.5707	1	1
Fe_2S_2	10	0.1192	1	1
LiO	10	0.2925	1	2
BN	10	0.1485	1	2
Au_2I_2	10	0.2516	1	1
Co_2S_2	10	0.1209	1	1
As_2Fe_2	10	0.1141	1	1
Ge_2Se_2	10	0.1867	1	1
Cu_2Se_2	10	0.1281	1	1
Bi_2O_2	10	0.1752	1	1
C_2	10	0.1436	1	2
Co_2Se_2	10	0.1247	1	1
Ca_2Cl_2	10	0.1233	1	1
Hf_3Te_2	11	0.1299	1	1
H_2Na_2Pd	11	0.1133	1	1
FKO_2Se	11	2.2482	1	1
F_4Nb	11	0.1824	1	1
NaO_4	11	0.6405	1	1
$AgNO_3$	11	0.6432	1	1
$Br_2In_2O_2$	12	0.1592	1	1
CrS_2	12	0.2508	1	2
$Br_2Ho_2S_2$	12	0.2888	1	1
S_2V	12	0.2797	1	2

Table showing the first 50 lattice matching combinations with the smallest number of atoms in the supercell.

Lattice matching - minimal strain

Formula	N° atoms	strain	cell size 1	cell size 2
CoO_2	759	0.0002	63	127
I_2Tm	168	0.0003	20	16
$C_2Br_2Tb_2$	12	0.0004	1	1
Te_2V	156	0.0005	16	20
FHOZn	526	0.0005	43	67
NiO_2	759	0.0005	63	127
$AgNO_3$	613	0.0005	68	41
GeI_2La_2	200	0.0005	20	16
O_2Zn	594	0.0005	53	92
$CrSe_2$	459	0.0005	43	67
NaO_4	613	0.0006	68	41
As_2Li_2Pr	962	0.0006	92	82
InSe	716	0.0006	92	82
$Cu_2Se_2Tl_2$	858	0.0006	78	65
CaClHO	860	0.0007	82	92
Bi_2O_2	804	0.0007	86	72
$Cl_2Hf_2N_2$	216	0.0007	16	20
OTl_2	156	0.0008	16	20
Cl_2Cu	654	0.0008	70	78
$AsLi_3$	880	0.0008	92	82
As_2Li_2Nd	962	0.0008	92	82
Cl_2V	459	0.0008	43	67
$Br_2La_2O_2$	948	0.0009	86	72
F_2Na	768	0.0009	82	92
$Eu_2F_2I_2$	948	0.0009	86	72
CaI_2	168	0.0009	20	16
CdClO	156	0.0009	16	20
GeI_2Y_2	962	0.0009	92	82
C_4Ca_2	744	0.001	68	56
Br_2PY_2	638	0.001	58	58
Br_2Cd	522	0.0011	58	58
Cl_2ORu	194	0.0011	19	20
Br_2Mn	156	0.0011	16	20
$I_2Se_2Tb_2$	738	0.0011	78	45
$NiTe_2$	768	0.0011	82	92
Cu_2I_2	728	0.0011	78	65
Cl_2OV	718	0.0011	67	79
HgI_2	930	0.0012	115	80
In_2Te_3	923	0.0012	88	79
Sb_2SeTe_2	923	0.0012	88	79
$SiTe_2$	738	0.0012	79	88
Se_2Zr	738	0.0012	79	88
$As_2Mg_2Na_2$	630	0.0013	60	45
Bi_2O_2	728	0.0013	78	65
Bi_2STe_2	962	0.0013	92	82
H_2NiO_2	555	0.0013	40	63
$Ga_2I_2Tb_2$	708	0.0013	63	55
MoS_2	429	0.0013	40	63
Bi_2Se_2Te	923	0.0013	88	79
$Br_2Hf_2N_2$	198	0.0013	17	16

Table showing the first 50 lattice matching combinations with the smallest strain needed to build the supercell.

Gd_2CCl_2 (P-3m1)

Structural and electronic properties

**Formula**	Gd_2CCl_2
**Spacegroup**	P-3m1
**Prototype**	Bi2Te2S
**Parent 3D**	CCl_2Gd_2
**Source DB**	MPDS
**DB ID**	S1400313
**DF2-C09 Binding energy [meV/ Å^2]**	11.74
**RVV10 Binding energy [meV/ Å^2]**	N/A
**Band gap (PBE) [eV]**	0.0

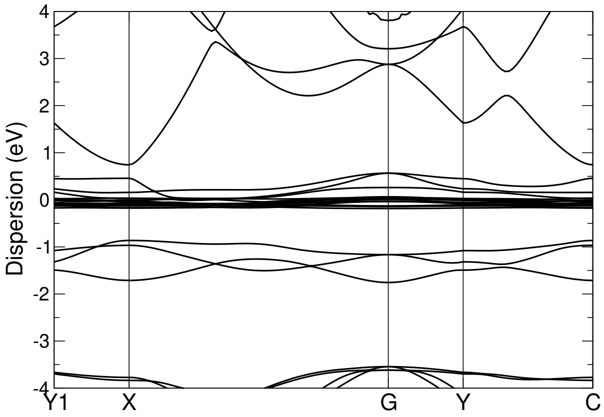

Band structure: Electronic band structure of Gd_2CCl_2 (P-3m1) in a representative window around the Fermi energy and along a high-symmetry path.

Optimized crystal structure

Structural parameters: relaxed cell (top) and atomic positions (bottom) of Gd_2CCl_2 (P-3m1) in Cartesian coordinates.

	x [Å]	y [Å]	z [Å]
$\mathbf{a_1}$	1.88021065	−3.25667340	0.00000000
$\mathbf{a_2}$	1.88021065	3.25667340	0.00000000
$\mathbf{a_3}$	0.00000000	0.00000000	24.26496732

		x [Å]	y [Å]	z [Å]
●	Gd	0.00000000	-1.08555145	1.36960413
●	Gd	0.00000000	1.08555145	-1.36960413
•	C	-1.88021065	0.00000000	0.00000000
•	Cl	0.00000000	-1.08554347	-3.08786818
•	Cl	0.00000000	1.08554347	3.08786818

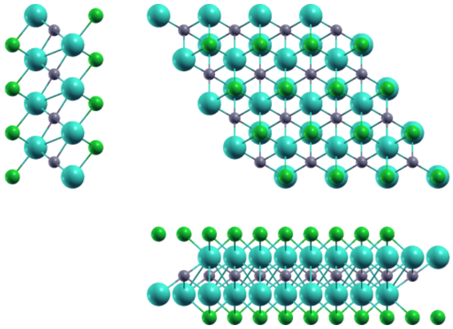

Orthographic projections: views of Gd_2CCl_2 (P-3m1) as seen from the x axis (left), the y axis (bottom) and the z axis (center).

Lattice matching - minimal size

Formula	N° atoms	strain	cell size 1	cell size 2
Tl	6	0.112	1	1
Tl	6	0.2551	1	1
InSe	7	0.4802	1	1
HgO	7	0.1171	1	1
Bi_2	7	0.4953	1	1
P_2	7	0.2481	1	1
PbTe	7	0.4848	1	1
CaCl	7	0.1532	1	1
I_2Mg	8	0.4542	1	1
Cl_2Mn	8	0.2631	1	1
$AgTe_2$	8	0.1131	1	1
$MoSe_2$	8	0.2541	1	1
S_2Ta	8	0.266	1	1
Br_2Zn	8	0.0001	1	1
$AsSn_2$	8	0.0067	1	1
$SiTe_2$	8	0.0043	1	1
Br_2La	8	0.4551	1	1
PbS_2	8	0.0079	1	1
Cl_2Ti	8	0.2483	1	1
BrCdI	8	0.462	1	1
S_2Ti	8	0.2732	1	1
Te_2Ti	8	0.0004	1	1
NbS_2	8	0.2654	1	1
BaF_2	8	0.4703	1	1
$RhTe_2$	8	0.0039	1	1
Bi_2Pd	8	0.1221	1	1
GeI_2	8	0.4497	1	1
Cl_2Co	8	0.2727	1	1
NbS_2	8	0.2593	1	1
Cl_2Fe	8	0.2717	1	1
S_2Ta	8	0.2581	1	1
Se_2V	8	0.2563	1	1
AsKSn	8	0.4667	1	1
$PbTe_2$	8	0.4596	1	1
$NiTe_2$	8	0.0037	1	1
I_2V	8	0.0056	1	1
GeI_2	8	0.4854	1	1
Se_2Zr	8	0.0047	1	1
STl_2	8	0.4728	1	1
$PtSe_2$	8	0.006	1	1
CdO_2	8	0.2725	1	1
GeS_2	8	0.142	1	1
$TaTe_2$	8	0.0072	1	1
$MnSe_2$	8	0.1531	1	1
Cl_2Zr	8	0.2722	1	1
Se_2Yb	8	0.4503	1	1
$BiTe_2$	8	0.4509	1	1
F_2Na	8	0.0021	1	1
$HfSe_2$	8	0.0004	1	1
Se_2W	8	0.2543	1	1

Table showing the first 50 lattice matching combinations with the smallest number of atoms in the supercell.

Lattice matching - minimal strain

Formula	N° atoms	strain	cell size 1	cell size 2
NbS_2	563	0.0	64	81
Bi_2	418	0.0	64	49
$CrSe_2$	327	0.0	36	49
$MnNaTe_2$	824	0.0001	100	81
AsKSn	674	0.0001	91	73
BiBrTe	414	0.0001	57	43
Sb_2SeTe_2	725	0.0001	81	64
Cl_2Hf_2	805	0.0001	81	100
CoO_2	93	0.0001	9	16
Br_2Zn	8	0.0001	1	1
H_2MnO_2	490	0.0001	37	61
In_2Te_3	725	0.0002	81	64
H_2Li_2Pt	90	0.0002	8	10
Bi_2STe_2	650	0.0002	73	57
DyI_2	255	0.0002	36	25
Tl	358	0.0003	57	73
Bi_2Se_2Te	725	0.0003	81	64
$Cl_2H_2Zr_2$	806	0.0003	64	81
HN_3OZn	629	0.0003	49	64
SSb_2Te_2	905	0.0003	100	81
$I_2Se_2Tm_2$	876	0.0003	108	56
$Br_2La_2O_2$	613	0.0003	65	48
Bi_2O_2	517	0.0003	65	48
Bi_2Te_3	425	0.0003	49	36
$Eu_2F_2I_2$	613	0.0003	65	48
BrCdI	743	0.0003	100	81
BiITe	353	0.0004	49	36
Cl_2NSc_2	820	0.0004	73	91
Te_2Ti	8	0.0004	1	1
BN	38	0.0004	4	9
$HfSe_2$	8	0.0004	1	1
$Dy_2I_2S_2$	330	0.0004	36	25
$Ga_2Gd_2I_2$	614	0.0005	64	49
Br_3Cs	505	0.0005	81	25
NS_2Ta	180	0.0005	16	25
Bi_2SeTe_2	565	0.0006	64	49
CBr_2Y_2	10	0.0006	1	1
I_2La_2	747	0.0006	91	73
Se_2V	504	0.0006	57	73
PbTe	479	0.0006	73	57
KNO_3	325	0.0006	49	16
As_2CeLi_2	650	0.0006	73	57
FeO_2	93	0.0006	9	16
CS_2Ta_2	565	0.0006	49	64
NiO_2	93	0.0006	9	16
$PbTe_2$	743	0.0006	100	81
$I_2S_2Tb_2$	330	0.0007	36	25
Cl_2Ti	437	0.0007	49	64
GeI_2Y_2	725	0.0007	81	64
NbS_2	638	0.0007	73	91

Table showing the first 50 lattice matching combinations with the smallest strain needed to build the supercell.

Gd_2Cl_2 (P-3m1)

Structural and electronic properties

**Formula**	Gd_2Cl_2
**Spacegroup**	P-3m1
**Prototype**	PtTe
**Parent 3D**	Cl_2Gd_2
**Source DB**	MPDS
**DB ID**	S542059
**DF2-C09 Binding energy [meV/ Å^2]**	11.4
**RVV10 Binding energy [meV/ Å^2]**	N/A
**Band gap (PBE) [eV]**	0.0

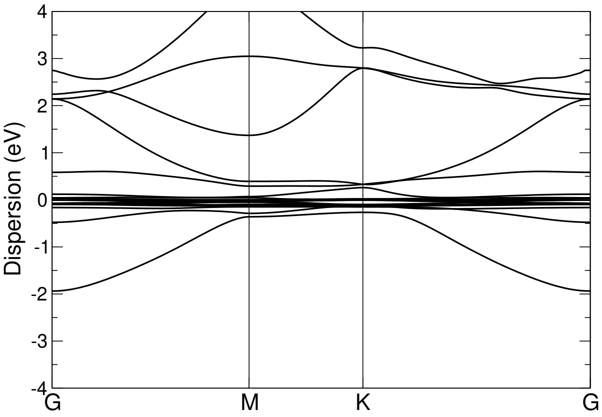

Band structure: Electronic band structure of Gd_2Cl_2 (P-3m1) in a representative window around the Fermi energy and along a high-symmetry path.

Optimized crystal structure

Structural parameters: relaxed cell (top) and atomic positions (bottom) of Gd_2Cl_2 (P-3m1) in Cartesian coordinates.

	x [Å]	y [Å]	z [Å]
$\mathbf{a_1}$	1.96237203	−3.39892806	0.00000000
$\mathbf{a_2}$	1.96237203	3.39892806	0.00000000
$\mathbf{a_3}$	0.00000000	0.00000000	24.64624902

		x [Å]	y [Å]	z [Å]
●	Gd	1.96237203	1.13297602	1.53605912
●	Cl	0.00000000	0.00000000	3.17660965
●	Gd	1.96237203	-1.13297602	-1.53605912
●	Cl	0.00000000	0.00000000	-3.17660965

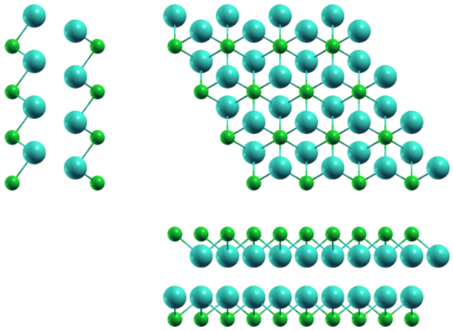

Orthographic projections: views of Gd_2Cl_2 (P-3m1) as seen from the x axis (left), the y axis (bottom) and the z axis (center).

Lattice matching - minimal size

Formula	N° atoms	strain	cell size 1	cell size 2
Na	5	0.2707	1	1
HgO	6	0.4331	1	1
GeTe	6	0.0038	1	1
S_2	6	0.0022	1	1
CaCl	6	0.1353	1	1
$IrTe_2$	7	0.0029	1	1
$CdCl_2$	7	0.0048	1	1
$MoTe_2$	7	0.274	1	1
$ReSe_2$	7	0.2533	1	1
CaI_2	7	0.4961	1	1
$InSe_2$	7	0.0044	1	1
$GeTe_2$	7	0.0058	1	1
$HfTe_2$	7	0.0055	1	1
I_2Mn	7	0.0047	1	1
NSr_2	7	0.0081	1	1
LiO_2	7	0.069	1	1
Cl_2Zn	7	0.1477	1	1
FeI_2	7	0.0068	1	1
I_2Ni	7	0.0056	1	1
CrI_2	7	0.0072	1	1
Te_2Zn	7	0.2738	1	1
BiBrTe	7	0.453	1	1
Bi_2Pd	7	0.1125	1	1
Cl_2Ni	7	0.2545	1	1
$CrTe_2$	7	0.263	1	1
PtS_2	7	0.2722	1	1
ClNZr	7	0.2494	1	1
Ba_2N	7	0.0071	1	1
Se_2Ti	7	0.2684	1	1
Br_2Ti	7	0.2622	1	1
Te_2Zr	7	0.006	1	1
Te_2W	7	0.2743	1	1
$AsSe_2$	7	0.2565	1	1
I_2Tm	7	0.493	1	1
BiITe	7	0.4693	1	1
CdO_2	7	1.5923	1	1
BrNZr	7	0.2583	1	1
$NbSe_2$	7	0.2551	1	1
CoI_2	7	0.0092	1	1
GeS_2	7	0.1266	1	1
$MnSe_2$	7	0.1352	1	1
Br_2Cr	7	0.2625	1	1
DyI_2	7	2.909	1	1
Se_2Ta	7	0.2552	1	1
Br_2Mg	7	0.007	1	1
I_2Ti	7	0.0089	1	1
$NbSe_2$	7	0.2569	1	1
GdI_2	7	0.46	1	1
F_2Ni	7	0.1441	1	1
Se_2Ta	7	0.2611	1	1

Table showing the first 50 lattice matching combinations with the smallest number of atoms in the supercell.

Lattice matching - minimal strain

Formula	N° atoms	strain	cell size 1	cell size 2
$I_2S_2Tb_2$	486	0.0	57	43
CdO_2	343	0.0	43	57
I_2La_2Sb	376	0.0001	49	36
Cl_2Co	343	0.0001	43	57
ClNZr	388	0.0001	49	64
Cl_2NSc_2	389	0.0001	36	49
Br_2Zr_2	724	0.0001	81	100
Se_2Ta	447	0.0002	57	73
CrO_2	496	0.0002	49	100
$AlLiTe_2$	656	0.0002	91	73
Cl_2Zr	343	0.0002	43	57
CaI_2	403	0.0002	64	49
$NbSe_2$	447	0.0003	57	73
K	232	0.0003	49	36
AsI_2La_2	644	0.0003	81	64
$C_2Cl_2Y_2$	788	0.0003	77	80
Cl_2Mn	291	0.0003	36	49
Cd_2I_3	644	0.0004	81	64
DyI_2	357	0.0004	57	43
$I_2N_2Ti_2$	612	0.0004	63	60
GdI_2	643	0.0005	100	81
Sm	25	0.0005	4	9
S_2Ti	343	0.0005	43	57
Li_2Tl_2	520	0.0005	81	49
Se_2Ti	624	0.0005	81	100
Cl_2Fe	343	0.0006	43	57
$I_2S_2Sm_2$	412	0.0006	49	36
MnO_2	393	0.0007	39	79
$Br_2H_2Zr_2$	580	0.0007	49	64
Cl_2Ni	447	0.0007	57	73
BrNZr	499	0.0007	64	81
Cl_2Tb_2	8	0.0007	1	1
$Ge_2I_2La_2$	634	0.0008	73	57
$AsSe_2$	447	0.0008	57	73
$AgNO_2$	876	0.0008	121	98
Bi_2Te_3	729	0.0009	91	73
S_2Zn_2	8	0.0009	1	1
I_2Tm	403	0.0009	64	49
BiITe	583	0.0009	91	73
$CrTe_2$	565	0.001	73	91
$Cl_2N_2Zr_2$	936	0.001	99	90
CrS_2	331	0.001	37	61
Cl_2Sc_2	656	0.001	73	91
$NbSe_2$	447	0.001	57	73
$Cl_2N_2Ti_2$	356	0.001	35	36
CCl_2Sc_2	516	0.0011	49	64
$Dy_2I_2S_2$	486	0.0011	57	43
Li_2Tl_2	392	0.0011	61	37
Br_2Hf_2	580	0.0011	64	81
$F_2Se_2Yb_2$	10	0.0011	1	1

Table showing the first 50 lattice matching combinations with the smallest strain needed to build the supercell.

$Gd_2Ga_2I_2$ (P-3m1)

Structural and electronic properties

**Formula**	$Gd_2Ga_2I_2$
**Spacegroup**	P-3m1
**Prototype**	SmSI
**Parent 3D**	$Ga_2Gd_2I_2$
**Source DB**	MPDS
**DB ID**	S1623208
**DF2-C09 Binding energy [meV/ Å^2]**	13.05
**RVV10 Binding energy [meV/ Å^2]**	N/A
**Band gap (PBE) [eV]**	0.0

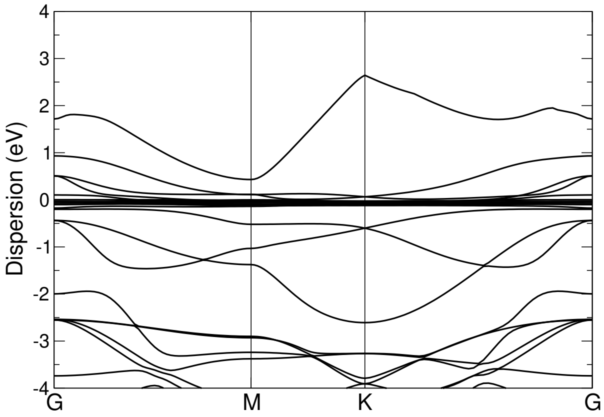

Band structure: Electronic band structure of $Gd_2Ga_2I_2$ (P-3m1) in a representative window around the Fermi energy and along a high-symmetry path.

Optimized crystal structure

Structural parameters: relaxed cell (top) and atomic positions (bottom) of $Gd_2Ga_2I_2$ (P-3m1) in Cartesian coordinates.

	x [Å]	y [Å]	z [Å]
$\mathbf{a_1}$	2.14677675	−3.71832641	0.00000000
$\mathbf{a_2}$	2.14677675	3.71832641	0.00000000
$\mathbf{a_3}$	0.00000000	0.00000000	28.05212442

	x [Å]	y [Å]	z [Å]
Gd	-1.07338838	-1.85916320	-11.92625783
Ga	1.07338838	-0.61972107	-13.77028086
I	1.07338838	-3.09860534	-9.95423393
Gd	-1.07338838	-1.85916320	-16.12586658
Ga	1.07338838	-3.09860534	-14.28184356
I	1.07338838	-0.61972107	-18.09789049

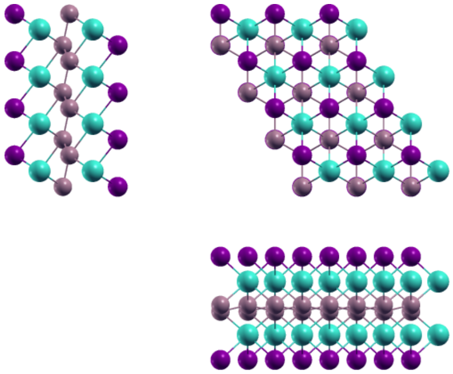

Orthographic projections: views of $Gd_2Ga_2I_2$ (P-3m1) as seen from the x axis (left), the y axis (bottom) and the z axis (center).

Lattice matching - minimal size

Formula	N° atoms	strain	cell size 1	cell size 2
InSe	8	0.0053	1	1
AsSb	8	0.264	1	1
Bi_2	8	0.0004	1	1
GeTe	8	0.2728	1	1
AgTl	8	0.1474	1	1
S_2	8	0.2751	1	1
PbTe	8	0.0036	1	1
CaCl	8	0.1115	1	1
$IrTe_2$	9	0.2741	1	1
$CdCl_2$	9	0.2712	1	1
CdI_2	9	0.0019	1	1
Br_2Zn	9	0.25	1	1
Br_2Ca	9	0.0006	1	1
$InSe_2$	9	0.2717	1	1
$GeTe_2$	9	0.2697	1	1
$SiTe_2$	9	0.2555	1	1
I_2Pr	9	0.1323	1	1
I_2Mn	9	0.2714	1	1
NSr_2	9	0.2663	1	1
PbS_2	9	0.2604	1	1
BiClTe	9	0.0015	1	1
Cl_2Zn	9	0.1175	1	1
FeI_2	9	0.2682	1	1
I_2Ni	9	0.27	1	1
Te_2Ti	9	0.2504	1	1
CrI_2	9	0.2675	1	1
BaF_2	9	0.009	1	1
BiBrTe	9	0.004	1	1
I_2Nd	9	0.133	1	1
$NiTe_2$	9	0.2547	1	1
Cl_2Cu	9	0.0693	1	1
I_2V	9	0.2574	1	1
GeI_2	9	0.0033	1	1
Se_2Zr	9	0.2561	1	1
STl_2	9	0.0081	1	1
CoI_2	9	0.2646	1	1
$MnSe_2$	9	0.1114	1	1
CeI_2	9	0.1318	1	1
Br_2Mg	9	0.268	1	1
I_2Ti	9	0.2651	1	1
GdI_2	9	0.007	1	1
F_2Ni	9	0.1156	1	1
I_2La	9	0.1367	1	1
F_2Na	9	1.6375	1	1
CdI_2	9	0.0023	1	1
Se_2Sn	9	0.266	1	1
F_2Zn	9	0.1295	1	1
I_2Pr	9	0.0014	1	1
$HfSe_2$	9	0.2504	1	1
H_2Si_2	10	0.2722	1	1

Table showing the first 50 lattice matching combinations with the smallest number of atoms in the supercell.

Lattice matching - minimal strain

Formula	N° atoms	strain	cell size 1	cell size 2
$RhTe_2$	429	0.0	43	57
NS_2Ta	694	0.0	49	100
$Cl_2Hf_2N_2$	366	0.0	25	36
ClNZr	171	0.0	16	25
OTl_2	258	0.0	25	36
$SiTe_2$	561	0.0001	57	73
CrI_2	786	0.0001	81	100
$Cl_2H_2Zr_2$	588	0.0001	37	61
CBr_2Y_2	614	0.0001	49	64
Ga_2Se_2	708	0.0002	64	81
NaO_4	341	0.0002	36	25
NbS_2	405	0.0002	37	61
CoI_2	711	0.0002	73	91
CdClO	258	0.0002	25	36
AsSb	620	0.0002	73	91
Br_2Mg	786	0.0003	81	100
Te_2V	258	0.0003	25	36
$Ga_2I_2Tb_2$	12	0.0003	1	1
$Cl_2Er_2O_2$	780	0.0003	57	73
N_2Re	51	0.0003	4	9
H_2Li_2Pt	694	0.0003	49	80
$Cl_2O_2Y_2$	870	0.0003	64	81
PSn_2	363	0.0004	36	49
FeI_2	786	0.0004	81	100
S_2Ta	537	0.0004	49	81
$CrSe_2$	102	0.0004	9	16
Bi_2	8	0.0004	1	1
N_2W	471	0.0004	39	79
C	49	0.0004	4	25
CCl_2Gd_2	614	0.0005	49	64
Se_2Zr	561	0.0005	57	73
Bi_2Te_2	838	0.0005	91	73
Br_2Y_2	708	0.0005	64	81
CrS_2	297	0.0005	25	49
$NiTe_2$	561	0.0005	57	73
I_2Ti	711	0.0005	73	91
Br_2Zn	486	0.0006	49	64
$Cl_2Rh_2Te_2$	954	0.0006	96	63
$Br_2H_2Zr_2$	246	0.0006	16	25
$Au_2K_2Se_2$	354	0.0006	45	14
Br_2Ca	9	0.0006	1	1
Bi_2Pd	840	0.0007	81	118
Cl_2Y_2	550	0.0007	49	64
$F_2Se_2Y_2$	780	0.0007	73	57
PbS_2	627	0.0008	64	81
CdClHO	486	0.0008	43	57
Se_4TiZr	726	0.0008	72	49
Cl_2V	102	0.0008	9	16
I_4Zr_2	882	0.0008	91	56
S_2Sn	363	0.0009	36	49

Table showing the first 50 lattice matching combinations with the smallest strain needed to build the supercell.

Gd_2GeBr_2 (P-3m1)

Structural and electronic properties

**Formula**	Gd_2GeBr_2
**Spacegroup**	P-3m1
**Prototype**	Bi2Te2S
**Parent 3D**	Br_2Gd_2Ge
**Source DB**	MPDS
**DB ID**	S1923103
**DF2-C09 Binding energy [meV/ Å^2]**	10.92
**RVV10 Binding energy [meV/ Å^2]**	N/A
**Band gap (PBE) [eV]**	0.0

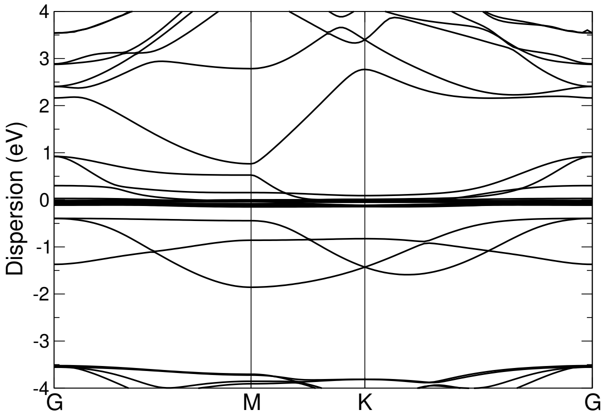

Band structure: Electronic band structure of Gd_2GeBr_2 (P-3m1) in a representative window around the Fermi energy and along a high-symmetry path.

Optimized crystal structure

Structural parameters: relaxed cell (top) and atomic positions (bottom) of Gd_2GeBr_2 (P-3m1) in Cartesian coordinates.

	x [Å]	y [Å]	z [Å]
$\mathbf{a_1}$	2.08093594	−3.60431379	0.00000000
$\mathbf{a_2}$	2.08093594	3.60431379	0.00000000
$\mathbf{a_3}$	0.00000000	0.00000000	25.70756555

		x [Å]	y [Å]	z [Å]
●	Gd	0.00000000	-1.20149195	-1.74217511
●	Br	0.00000000	1.20146289	-3.46184871
●	Gd	0.00000000	1.20149195	1.74217511
●	Ge	-2.08093594	0.00000000	0.00000000
●	Br	0.00000000	-1.20146289	3.46184871

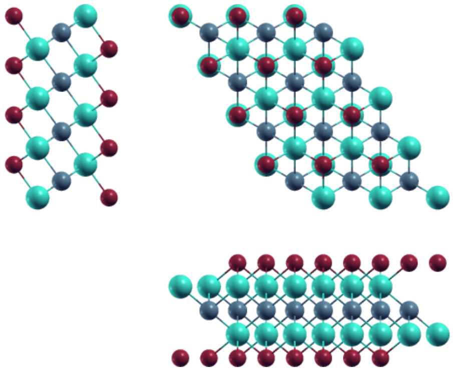

Orthographic projections: views of Gd_2GeBr_2 (P-3m1) as seen from the x axis (left), the y axis (bottom) and the z axis (center).

Lattice matching - minimal size

Formula	N° atoms	strain	cell size 1	cell size 2
K	6	0.4489	1	1
InSe	7	0.0095	1	1
AgTl	7	0.162	1	1
Ag_2	7	0.4625	1	1
Sb_2	7	0.0065	1	1
CaCl	7	0.1176	1	1
I_2Mg	8	0.0009	1	1
PSn_2	8	0.2554	1	1
Ba_2Pt	8	0.4618	1	1
Br_2Zn	8	0.2705	1	1
HfS_2	8	0.2472	1	1
$AsSn_2$	8	0.2606	1	1
I_2Pr	8	0.1444	1	1
$CuTe_2$	8	0.2467	1	1
S_2Zr	8	0.2544	1	1
Br_2La	8	0.0006	1	1
Ca_2Si	8	0.4732	1	1
BrCdI	8	0.0022	1	1
Cl_2Zn	8	0.1258	1	1
Te_2Ti	8	0.2709	1	1
BaF_2	8	0.0056	1	1
Te_2Zn	8	1.5438	1	1
$RhTe_2$	8	0.2646	1	1
GeI_2	8	0.0028	1	1
$CoTe_2$	8	0.2476	1	1
AsKSn	8	0.0041	1	1
$PbTe_2$	8	0.0012	1	1
I_2Nd	8	0.1453	1	1
$NiTe_2$	8	0.2757	1	1
S_2Sn	8	0.2547	1	1
$SnTe_2$	8	0.005	1	1
Sn	8	0.6243	1	3
I_2Pb	8	0.4664	1	1
STl_2	8	0.0065	1	1
$PtSe_2$	8	0.2615	1	1
GeS_2	8	0.1124	1	1
$TaTe_2$	8	0.2598	1	1
$MnSe_2$	8	0.1175	1	1
Br_2Ni	8	0.2498	1	1
CeI_2	8	0.1437	1	1
$NbTe_2$	8	0.2541	1	1
In	8	0.6389	1	3
Se_2Yb	8	0.0026	1	1
Cl_2Mg	8	0.2499	1	1
$BiTe_2$	8	0.0023	1	1
F_2Ni	8	0.1233	1	1
I_2La	8	0.1497	1	1
F_2Na	8	0.2734	1	1
F_2Zn	8	0.1409	1	1
$HfSe_2$	8	0.2709	1	1

Table showing the first 50 lattice matching combinations with the smallest number of atoms in the supercell.

Lattice matching - minimal strain

Formula	N° atoms	strain	cell size 1	cell size 2
Cu_2I_2	9	0.0	1	1
Ga_2Se_2	805	0.0	81	100
PSn_2	504	0.0	57	73
Ga_2S_2	501	0.0	49	64
N_3W_2	745	0.0	49	100
$HNiO_2$	321	0.0001	25	49
Te_2W	327	0.0001	36	49
$Br_2Hf_2N_2$	534	0.0002	48	49
$Hf_2I_2N_2$	806	0.0002	64	81
CdClHO	644	0.0002	64	81
$RhTe_2$	638	0.0002	73	91
I_2Pb	674	0.0002	91	73
Se_2Ta	233	0.0002	25	36
Ba_2Pt	743	0.0003	100	81
$MoTe_2$	327	0.0003	36	49
Ca_2N	386	0.0003	43	57
PTe_2Ti_2	565	0.0003	49	64
$NbSe_2$	233	0.0003	25	36
Gd_2I_2	9	0.0003	1	1
CdH_2O_2	425	0.0003	36	49
Cl_2OV	111	0.0003	11	14
$Br_2Hf_2N_2$	557	0.0003	43	57
Se_2V	155	0.0003	16	25
$F_2Se_2Y_2$	461	0.0004	49	36
$TaTe_2$	563	0.0004	64	81
Br_2Ni	437	0.0004	49	64
Te_2Zn	327	0.0004	36	49
Cl_2Mg	437	0.0005	49	64
Ag_2	662	0.0005	100	81
S_2Sn	504	0.0005	57	73
Bi_2Se_3	10	0.0005	1	1
CdClHO	729	0.0006	73	91
H_2MnO_2	590	0.0006	39	79
Br_2La	8	0.0006	1	1
$Br_2N_2Ti_2$	857	0.0007	73	82
Bi_2Te_2	457	0.0007	57	43
$HfLiS_2$	376	0.0007	36	49
Cl_2Ni	233	0.0007	25	36
Cl_2Y_2	805	0.0007	81	100
$AsSe_2$	233	0.0007	25	36
LiO	407	0.0008	49	81
$Cl_2H_2Lu_2$	629	0.0008	49	64
S_2Zr	504	0.0008	57	73
Au_2Se_2	475	0.0008	63	40
C_2F_2	381	0.0008	25	64
As_2	329	0.0008	43	57
$Cl_2N_2Zr_2$	557	0.0009	43	57
CCl_2Lu_2	500	0.0009	43	57
I_2Mg	8	0.0009	1	1
$AsSn_2$	563	0.001	64	81

Table showing the first 50 lattice matching combinations with the smallest strain needed to build the supercell.

Gd_2GeI_2 (P-3m1)

Structural and electronic properties

**Formula**	Gd_2GeI_2
**Spacegroup**	P-3m1
**Prototype**	Bi2Te2S
**Parent 3D**	Gd_2GeI_2
**Source DB**	MPDS
**DB ID**	S1923102
**DF2-C09 Binding energy [meV/ Å^2]**	13.14
**RVV10 Binding energy [meV/ Å^2]**	N/A
**Band gap (PBE) [eV]**	0.0

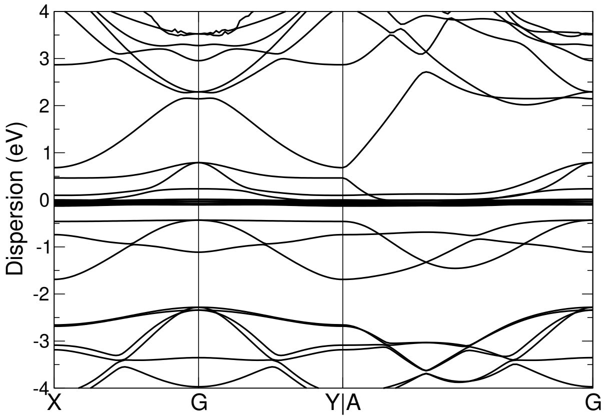

Band structure: Electronic band structure of Gd_2GeI_2 (P-3m1) in a representative window around the Fermi energy and along a high-symmetry path.

Optimized crystal structure

Structural parameters: relaxed cell (top) and atomic positions (bottom) of Gd_2GeI_2 (P-3m1) in Cartesian coordinates.

	x [Å]	y [Å]	z [Å]
$\mathbf{a_1}$	2.13847369	−3.70452110	0.00000000
$\mathbf{a_2}$	2.13897254	3.70480912	0.00000000
$\mathbf{a_3}$	0.00000000	0.00000000	26.51041937

		x [Å]	y [Å]	z [Å]
●	Gd	-1.06944068	0.61744186	-1.67691336
●	I	1.06998255	-0.61775472	-3.65663700
●	Gd	1.06944068	-0.61744186	1.67691336
●	I	-1.06998255	0.61775472	3.65663700
●	Ge	1.06948627	1.85240456	0.00000000

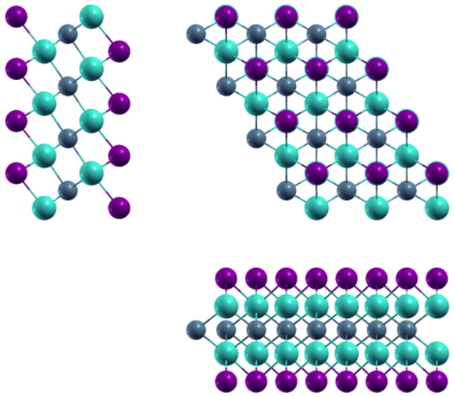

Orthographic projections: views of Gd_2GeI_2 (P-3m1) as seen from the x axis (left), the y axis (bottom) and the z axis (center).

Lattice matching - minimal size

Formula	N° atoms	strain	cell size 1	cell size 2
InSe	7	0.0036	1	1
AsSb	7	0.2664	1	1
Bi_2	7	0.0022	1	1
GeTe	7	0.2754	1	1
AgTl	7	0.149	1	1
PbTe	7	0.0018	1	1
CaCl	7	0.1121	1	1
$CdCl_2$	8	0.2738	1	1
CdI_2	8	0.0001	1	1
Br_2Ca	8	0.0011	1	1
$InSe_2$	8	0.2744	1	1
$GeTe_2$	8	0.2722	1	1
$SiTe_2$	8	0.258	1	1
I_2Pr	8	0.1337	1	1
I_2Mn	8	0.274	1	1
NSr_2	8	0.2689	1	1
PbS_2	8	0.2628	1	1
BiClTe	8	0.0002	1	1
Cl_2Zn	8	0.1183	1	1
FeI_2	8	0.2707	1	1
I_2Ni	8	0.2726	1	1
CrI_2	8	0.27	1	1
BaF_2	8	0.0073	1	1
BiBrTe	8	0.0058	1	1
$RhTe_2$	8	0.247	1	1
AsKSn	8	0.0087	1	1
I_2Nd	8	0.1344	1	1
$NiTe_2$	8	0.2571	1	1
Cl_2Cu	8	0.0687	1	1
I_2V	8	0.2598	1	1
GeI_2	8	0.0016	1	1
Se_2Zr	8	0.2585	1	1
STl_2	8	0.0064	1	1
CoI_2	8	0.2672	1	1
GeS_2	8	0.4246	1	1
$TaTe_2$	8	1.5803	1	1
$MnSe_2$	8	0.1121	1	1
CeI_2	8	0.1331	1	1
Br_2Mg	8	0.2705	1	1
I_2Ti	8	0.2676	1	1
GdI_2	8	0.0088	1	1
F_2Ni	8	0.1164	1	1
I_2La	8	0.1382	1	1
F_2Na	8	0.255	1	1
CdI_2	8	0.0006	1	1
Se_2Sn	8	0.2685	1	1
F_2Zn	8	0.1307	1	1
I_2Pr	8	0.0003	1	1
H_2Si_2	9	0.2748	1	1
Bi_2Te_2	9	0.4725	1	1

Table showing the first 50 lattice matching combinations with the smallest number of atoms in the supercell.

Lattice matching - minimal strain

Formula	N° atoms	strain	cell size 1	cell size 2
Se_2V	488	0.0	49	81
I_2Ti	705	0.0	81	100
NS_2Ta	511	0.0	39	79
Te_2Zn	233	0.0001	25	36
$MoTe_2$	233	0.0001	25	36
CdI_2	8	0.0001	1	1
FHOZn	109	0.0002	9	16
$Cu_2Rb_2Te_2$	613	0.0002	65	48
BiClTe	8	0.0002	1	1
Te_2W	233	0.0003	25	36
Bi_2In_2	517	0.0003	65	48
$CrSe_2$	93	0.0003	9	16
CoI_2	705	0.0003	81	100
I_2Pr	8	0.0003	1	1
F_2Na	504	0.0003	57	73
I_2V	563	0.0004	64	81
$HfLiS_2$	269	0.0004	25	36
Ga_2Se_2	501	0.0004	49	64
$Cl_2O_2Yb_2$	723	0.0004	57	73
$PtSe_2$	386	0.0004	43	57
Br_2La_2P	10	0.0004	1	1
$NbTe_2$	327	0.0004	36	49
CI_2Y_2	905	0.0005	81	100
$Cl_2O_2Tm_2$	723	0.0005	57	73
$Br_2Hf_2N_2$	574	0.0005	50	54
CdI_2	8	0.0006	1	1
Se_2Zr	563	0.0006	64	81
CaClHO	577	0.0006	57	73
C_4Ca_2	954	0.0006	90	84
Se_2V	368	0.0006	37	61
Se_2Sn	705	0.0006	81	100
$I_2N_2Zr_2$	629	0.0006	49	64
S_2Zr	327	0.0006	36	49
CdH_2O_2	305	0.0007	25	36
$Cl_2Ho_2O_2$	806	0.0007	64	81
S_2Ta	368	0.0007	37	61
$Cl_2Er_2O_2$	806	0.0007	64	81
AsSb	605	0.0008	81	100
CBr_2Lu_2	425	0.0008	36	49
PTe_2Zr_2	820	0.0008	73	91
Tl	326	0.0008	49	81
$Br_2N_2Zr_2$	474	0.0009	36	49
NSr_2	705	0.0009	81	100
S_2Sn	327	0.0009	36	49
Cl_2V	93	0.0009	9	16
ClH_3O	820	0.0009	91	73
$FeSe_2$	743	0.0009	70	131
Bi_2STe_2	10	0.001	1	1
$SiTe_2$	563	0.001	64	81
CdClHO	443	0.001	43	57

Table showing the first 50 lattice matching combinations with the smallest strain needed to build the supercell.

Gd_2I_2 (P-3m1)

Structural and electronic properties

**Formula**	Gd_2I_2
**Spacegroup**	P-3m1
**Prototype**	PtTe
**Parent 3D**	Gd_2I_2
**Source DB**	ICSD
**DB ID**	61035
**DF2-C09 Binding energy [meV/ Å^2]**	14.83
**RVV10 Binding energy [meV/ Å^2]**	N/A
**Band gap (PBE) [eV]**	0.0

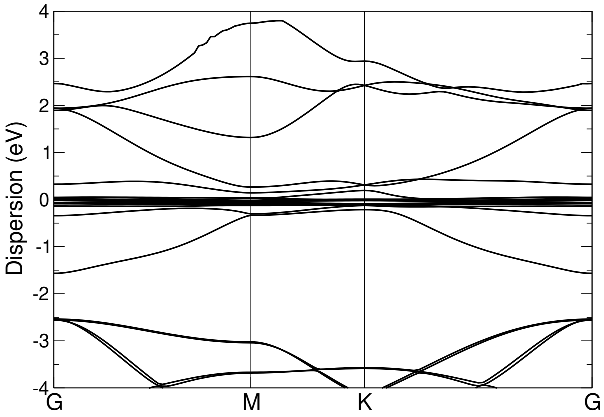

Band structure: Electronic band structure of Gd_2I_2 (P-3m1) in a representative window around the Fermi energy and along a high-symmetry path.

Optimized crystal structure

Structural parameters: relaxed cell (top) and atomic positions (bottom) of Gd_2I_2 (P-3m1) in Cartesian coordinates.

	x [Å]	y [Å]	z [Å]
$\mathbf{a_1}$	2.07957398	−3.60192398	0.00000000
$\mathbf{a_2}$	2.07957068	3.60192207	0.00000000
$\mathbf{a_3}$	0.00000000	0.00000000	26.25260863

		x [Å]	y [Å]	z [Å]
●	Gd	1.03978883	-0.60032236	1.53568140
●	Gd	3.11935583	0.60032045	-1.53568140
●	I	1.03979299	1.80095662	3.60674693
●	I	1.03977769	1.80096545	-3.60674693

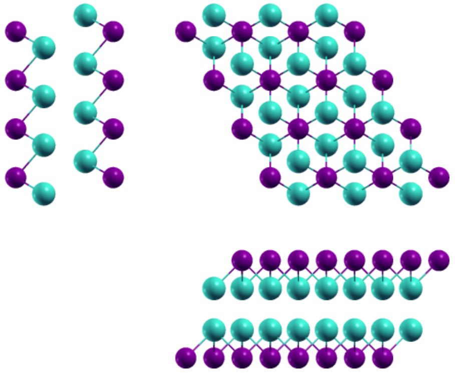

Orthographic projections: views of Gd_2I_2 (P-3m1) as seen from the x axis (left), the y axis (bottom) and the z axis (center).

Lattice matching - minimal size

Formula	N° atoms	strain	cell size 1	cell size 2
K	5	0.4497	1	1
AgTl	6	0.1624	1	1
Ag_2	6	0.4632	1	1
Sb_2	6	0.0062	1	1
CaCl	6	0.1177	1	1
I_2Mg	7	0.0006	1	1
PSn_2	7	0.2559	1	1
Ba_2Pt	7	0.4626	1	1
Br_2Zn	7	0.2709	1	1
HfS_2	7	0.2476	1	1
$AsSn_2$	7	0.2611	1	1
I_2Pr	7	0.1447	1	1
$CuTe_2$	7	0.2471	1	1
S_2Zr	7	0.2548	1	1
Br_2La	7	0.0003	1	1
Ca_2Si	7	0.474	1	1
BrCdI	7	0.0025	1	1
Cl_2Zn	7	0.126	1	1
Te_2Ti	7	0.2714	1	1
BaF_2	7	0.0059	1	1
Te_2Zn	7	1.5461	1	1
$RhTe_2$	7	0.265	1	1
GeI_2	7	0.0025	1	1
$CoTe_2$	7	0.248	1	1
AsKSn	7	0.0044	1	1
$PbTe_2$	7	0.0015	1	1
I_2Nd	7	0.1455	1	1
S_2Sn	7	0.2552	1	1
$SnTe_2$	7	0.0047	1	1
Sn	7	0.6253	1	3
I_2Pb	7	0.4672	1	1
STl_2	7	0.0069	1	1
$PtSe_2$	7	0.262	1	1
GeS_2	7	0.1125	1	1
$TaTe_2$	7	0.2603	1	1
$MnSe_2$	7	0.1177	1	1
Br_2Ni	7	0.2502	1	1
CeI_2	7	0.144	1	1
$NbTe_2$	7	0.2545	1	1
In	7	0.6399	1	3
Se_2Yb	7	0.0022	1	1
Cl_2Mg	7	0.2503	1	1
$BiTe_2$	7	0.002	1	1
F_2Ni	7	0.1235	1	1
I_2La	7	0.15	1	1
F_2Na	7	0.2738	1	1
F_2Zn	7	0.1412	1	1
$HfSe_2$	7	0.2714	1	1
Bi_2Te_2	8	2.9277	1	1
Fe_2Te_2	8	0.134	1	1

Table showing the first 50 lattice matching combinations with the smallest number of atoms in the supercell.

Lattice matching - minimal strain

Formula	N° atoms	strain	cell size 1	cell size 2
$NbSe_2$	208	0.0	25	36
Ca_2N	343	0.0	43	57
Se_2V	139	0.0	16	25
$MoTe_2$	291	0.0001	36	49
$F_2Se_2Y_2$	412	0.0001	49	36
Se_2Ta	208	0.0001	25	36
I_2Pb	583	0.0001	91	73
Te_2Zn	291	0.0001	36	49
CdClHO	580	0.0001	64	81
$Hf_2I_2N_2$	742	0.0001	64	81
Cl_2OV	100	0.0002	11	14
S_2Sn	447	0.0002	57	73
Te_2W	291	0.0002	36	49
$HNiO_2$	296	0.0002	25	49
Bi_2Se_3	9	0.0002	1	1
CdClHO	656	0.0003	73	91
N_3W_2	696	0.0003	49	100
Ga_2S_2	452	0.0003	49	64
PSn_2	447	0.0003	57	73
Br_2La	7	0.0003	1	1
Cu_2I_2	8	0.0003	1	1
Br_2Gd_2Ge	9	0.0003	1	1
Ga_2Se_2	724	0.0003	81	100
$Br_2N_2Ti_2$	784	0.0004	73	82
$HfLiS_2$	340	0.0004	36	49
Cl_2Ni	208	0.0004	25	36
LiO	358	0.0005	49	81
S_2Zr	447	0.0005	57	73
Au_2Se_2	412	0.0005	63	40
$Br_2Hf_2N_2$	486	0.0005	48	49
As_2	286	0.0005	43	57
$RhTe_2$	565	0.0005	73	91
Ba_2Pt	643	0.0006	100	81
PTe_2Ti_2	516	0.0006	49	64
$I_2N_2Ti_2$	936	0.0006	90	96
CCl_2Lu_2	457	0.0006	43	57
I_2Mg	7	0.0006	1	1
CdH_2O_2	389	0.0006	36	49
$Br_2Hf_2N_2$	514	0.0007	43	57
$I_2N_2Zr_2$	924	0.0007	81	100
$NbTe_2$	447	0.0007	57	73
$TaTe_2$	499	0.0007	64	81
I_4Sr_2	806	0.0007	128	49
Br_2Ni	388	0.0007	49	64
$I_2Pr_2S_2$	802	0.0007	91	73
$Ce_2I_2S_2$	708	0.0008	81	64
Ca_2Si	516	0.0008	81	64
Ga_2S_2	452	0.0008	49	64
Cl_2Mg	388	0.0008	49	64
Ag_2	562	0.0008	100	81

Table showing the first 50 lattice matching combinations with the smallest strain needed to build the supercell.

$Gd_2O_2Br_2$ (P4/nmm)

Structural and electronic properties

**Formula**	$Gd_2O_2Br_2$
**Spacegroup**	P4/nmm
**Prototype**	PbClF
**Parent 3D**	$Br_2Gd_2O_2$
**Source DB**	MPDS
**DB ID**	S1903363
**DF2-C09 Binding energy [meV/ Å^2]**	16.3
**RVV10 Binding energy [meV/ Å^2]**	N/A
**Band gap (PBE) [eV]**	0.0

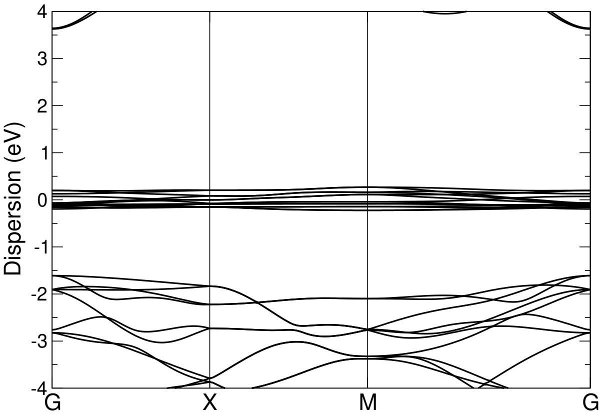

Band structure: Electronic band structure of $Gd_2O_2Br_2$ (P4/nmm) in a representative window around the Fermi energy and along a high-symmetry path.

Optimized crystal structure

Structural parameters: relaxed cell (top) and atomic positions (bottom) of $Gd_2O_2Br_2$ (P4/nmm) in Cartesian coordinates.

	x [Å]	y [Å]	z [Å]
$\mathbf{a_1}$	3.87566840	0.00000000	0.00000000
$\mathbf{a_2}$	0.00000000	3.87566840	0.00000000
$\mathbf{a_3}$	0.00000000	0.00000000	22.90171599

	x [Å]	y [Å]	z [Å]
Gd	0.96891710	-0.96891710	1.16164120
Br	-0.96891710	-2.90675130	2.81296922
Gd	-0.96891710	-2.90675130	-1.16164120
Br	0.96891710	-0.96891710	-2.81296922
O	0.96891710	-2.90675130	0.00000000
O	-0.96891710	-0.96891710	0.00000000

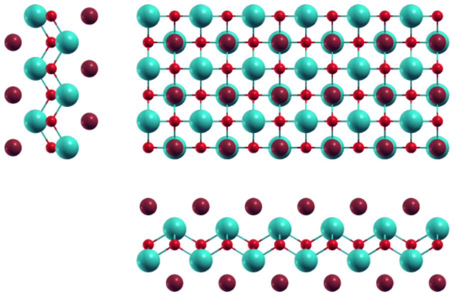

Orthographic projections: views of $Gd_2O_2Br_2$ (P4/nmm) as seen from the x axis (left), the y axis (bottom) and the z axis (center).

Lattice matching - minimal size

Formula	N° atoms	strain	cell size 1	cell size 2
K	7	0.385	1	1
K	7	0.1887	1	1
In	7	0.1113	1	1
InSe	8	0.149	1	1
Bi_2	8	0.1547	1	1
AgTl	8	0.0258	1	1
Ag_2	8	0.1959	1	1
LiO	8	0.109	1	1
PbTe	8	0.1507	1	1
Sb_2	8	0.1352	1	1
I_2Mg	9	0.1398	1	1
S_2V	9	0.1105	1	1
MoS_2	9	0.1104	1	1
CdI_2	9	0.1524	1	1
Nd	9	0.77	1	3
PSn_2	9	0.1087	1	1
Ba_2Pt	9	0.1956	1	1
Br_2Ca	9	0.1536	1	1
CaI_2	9	0.1773	1	1
I_2Pr	9	0.0037	1	1
S_2Zr	9	0.1085	1	1
Br_2La	9	0.1401	1	1
Br_2Cu	9	0.1072	1	1
I_2Yb	9	0.1742	1	1
BiClTe	9	0.1527	1	1
$AuTe_2$	9	0.1294	1	1
BrCdI	9	0.1425	1	1
I_2Zn	9	0.1327	1	1
BaF_2	9	0.1455	1	1
BiBrTe	9	0.1583	1	1
S_2W	9	0.1103	1	1
Bi_2Pd	9	0.5618	1	1
GeI_2	9	0.1383	1	1
AsKSn	9	0.1442	1	1
$PbTe_2$	9	0.1416	1	1
I_2Nd	9	0.0047	1	1
Cl_2Cu	9	0.0994	1	1
I_2Tm	9	0.1759	1	1
S_2Sn	9	0.1086	1	1
$SnTe_2$	9	0.1364	1	1
Cl_2V	9	0.1095	1	1
GeI_2	9	0.1509	1	1
I_2Pb	9	0.7532	1	1
STl_2	9	0.1463	1	1
BiITe	9	0.1653	1	1
DyI_2	9	0.1807	1	1
CeI_2	9	0.0029	1	1
$NbTe_2$	9	0.1084	1	1
Se_2Yb	9	0.1385	1	1
MoS_2	9	0.1103	1	1

Table showing the first 50 lattice matching combinations with the smallest number of atoms in the supercell.

Lattice matching - minimal strain

Formula	N° atoms	strain	cell size 1	cell size 2
$Ba_2F_2I_2$	510	0.0	49	36
$AgClO_4$	246	0.0	25	16
Ca_2O_2	412	0.0002	36	49
Te_2V	483	0.0002	48	65
$Cl_2Hf_2N_2$	678	0.0002	48	65
OTl_2	483	0.0002	48	65
Pd_2S_4	708	0.0003	79	39
F_2Zn	9	0.0003	1	1
$Cu_2Na_2Te_2$	882	0.0003	82	65
Cl_2Rb_2	186	0.0003	25	9
CdClO	483	0.0004	48	65
Cl_4Cu_2	534	0.0005	64	25
O_4PTl	510	0.0006	49	36
Br_2V	840	0.0006	81	118
$Br_2Ca_2F_2$	12	0.0006	1	1
$I_2Lu_2O_2$	12	0.0007	1	1
$Cl_2S_2Tl_2$	780	0.0007	81	49
$Br_2F_2Tm_2$	12	0.0007	1	1
$Cu_2K_2Te_2$	876	0.0008	85	61
$Ca_2Ge_2Mn_2$	12	0.0009	1	1
HgI_2	933	0.0009	113	85
$Br_2O_2V_2$	66	0.0009	5	6
Se_2Ta_4	678	0.001	49	64
Cl_2Zr_2	958	0.001	81	118
$FeSe_2$	102	0.001	9	16
$ReSe_2$	840	0.001	81	118
$Br_2F_2Yb_2$	12	0.0011	1	1
$Cu_2Na_2Te_2$	870	0.0011	81	64
LiO_2	510	0.0011	49	72
$Cl_2O_2V_2$	54	0.0012	4	5
CdH_2O_2	613	0.0012	48	65
K	681	0.0012	100	81
F_4Pb	640	0.0013	65	50
$Bi_2Cl_2O_2$	12	0.0014	1	1
Bi_2Pd	363	0.0015	36	49
H_2Na_2Pd	997	0.0015	82	101
MoS_2	711	0.0015	64	109
I_2Tm	885	0.0015	103	89
S_2W	711	0.0015	64	109
MoS_2	711	0.0015	64	109
I_2Tm	765	0.0015	89	77
H_2NiO_2	929	0.0016	64	109
GeI_2La_2	919	0.0016	89	77
$I_2N_2Zr_2$	834	0.0016	62	77
Br_2Cu_2	10	0.0016	1	1
$HNiO_2$	276	0.0016	20	39
Te_2W	483	0.0016	48	65
$PtTe_2$	975	0.0016	106	113
CaI_2	885	0.0016	103	89
Bi_2In_2	316	0.0016	36	25

Table showing the first 50 lattice matching combinations with the smallest strain needed to build the supercell.

$Gd_2S_2I_2$ (Pmm2)

Structural and electronic properties

**Formula**	$Gd_2S_2I_2$
**Spacegroup**	Pmm2
**Prototype**	FeOCl
**Parent 3D**	$Gd_2I_2S_2$
**Source DB**	ICSD
**DB ID**	416462
**DF2-C09 Binding energy [meV/ Å^2]**	10.1
**RVV10 Binding energy [meV/ Å^2]**	N/A
**Band gap (PBE) [eV]**	0.0

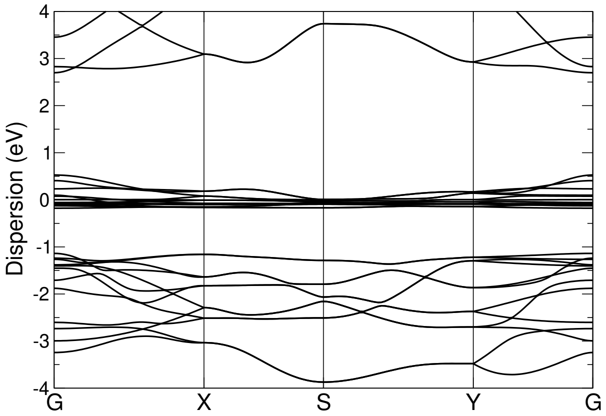

Band structure: Electronic band structure of $Gd_2S_2I_2$ (Pmm2) in a representative window around the Fermi energy and along a high-symmetry path.

Optimized crystal structure

Structural parameters: relaxed cell (top) and atomic positions (bottom) of $Gd_2S_2I_2$ (Pmm2) in Cartesian coordinates.

	x [Å]	y [Å]	z [Å]
$\mathbf{a_1}$	4.25588736	0.00000000	0.00000000
$\mathbf{a_2}$	0.00000000	5.33500670	0.00000000
$\mathbf{a_3}$	0.00000000	0.00000000	25.30258659

		x [Å]	y [Å]	z [Å]
●	Gd	1.06396284	-4.00125503	-1.15533983
●	S	1.06406640	-1.33375168	-0.58825954
●	I	-1.06383228	-4.00125503	-3.38178946
●	Gd	-1.06396284	-1.33375168	1.15533983
●	S	-1.06406640	-4.00125503	0.58825954
●	I	1.06383228	-1.33375168	3.38178946

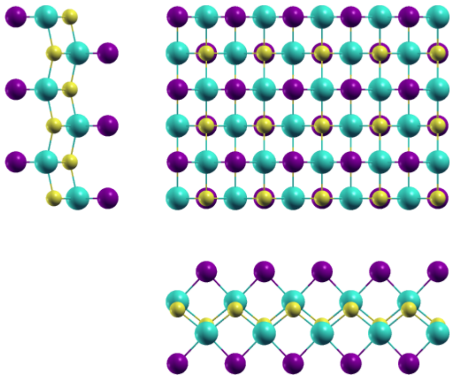

Orthographic projections: views of $Gd_2S_2I_2$ (Pmm2) as seen from the x axis (left), the y axis (bottom) and the z axis (center).

Lattice matching - minimal size

Formula	N° atoms	strain	cell size 1	cell size 2
Ba_2Hg	9	0.7373	1	1
CNRb	9	0.2115	1	1
Ba_2Cd	9	0.2509	1	1
Bi_2In_2	10	0.322	1	1
In_2Se_2	10	0.096	1	1
$AgCuTe_2$	10	1.7615	1	1
Au_2Br_2	10	0.0605	1	1
As_4	10	0.2857	1	1
Br_3Cs	10	0.3642	1	1
S_2	10	0.6262	1	2
Au_2I_2	10	0.7746	1	1
Bi_2O_2	10	0.693	1	1
$AgClO_2$	10	0.1168	1	1
PbS_2Sn	10	0.2499	1	1
$SbSe_2Tl$	10	0.1935	1	1
Br_2CsF	10	0.3184	1	1
Gd	10	0.0282	1	4
Hg_3N_2	11	0.3931	1	1
FKO_2Se	11	0.524	1	1
$ClKO_3$	11	0.3718	1	1
Cl_4Mn	11	0.0521	1	1
$Ba_2H_2I_2$	12	0.3151	1	1
CrS_2	12	0.137	1	2
$Br_2Ho_2S_2$	12	0.0138	1	1
$I_2Lu_2Se_2$	12	0.5111	1	1
$Ho_2I_2S_2$	12	0.0051	1	1
$Eu_2F_2I_2$	12	0.6912	1	1
AlH_4Na	12	0.0526	1	1
ReS_2	12	0.1398	1	2
Br_2Ca_3Si	12	0.2478	1	1
$I_2Se_2Tb_2$	12	0.0188	1	1
$Gd_2I_2Se_2$	12	0.0202	1	1
$Br_2S_2Y_2$	12	0.0161	1	1
Ba_2Hg	12	0.4639	1	2
N_2W	12	0.1342	1	2
$I_2S_2Tb_2$	12	0.0021	1	1
$I_2S_2Yb_2$	12	0.0097	1	1
$Cu_2Rb_2Te_2$	12	0.3214	1	1
$Br_2Dy_2S_2$	12	0.0118	1	1
$Er_2I_2Se_2$	12	0.0167	1	1
$Cl_2Ga_2Te_2$	12	0.4898	1	1
$I_2Se_2Tm_2$	12	0.0163	1	1
$Ag_2K_2Te_2$	12	0.0543	1	1
$Br_2Ga_2Te_2$	12	0.4982	1	1
Ca_4Cu_2	12	0.4879	1	1
$Br_2Lu_2S_2$	12	0.0227	1	1
$Br_2S_2Yb_2$	12	0.0173	1	1
$Ba_2F_2I_2$	12	0.302	1	1
$Br_2Er_2S_2$	12	0.015	1	1
O_4PTl	12	0.3018	1	1

Table showing the first 50 lattice matching combinations with the smallest number of atoms in the supercell.

Lattice matching - minimal strain

Formula	N° atoms	strain	cell size 1	cell size 2
CdClHO	820	0.0003	60	115
$Cl_2N_2Zr_2$	534	0.0004	35	54
C_2Li_2	322	0.0005	27	40
Au_2Br_2	396	0.0006	36	45
$Se_2Si_2Zr_2$	168	0.0007	10	18
Cu_2Te_2	614	0.0008	49	80
As_2O_3	553	0.0009	53	47
$K_2O_2Tl_2$	660	0.0009	56	54
Hf_3Te_2	871	0.001	61	101
$Br_2In_2O_2$	630	0.001	42	63
$Br_2Cr_2O_2$	588	0.0011	35	63
$As_2Co_2Li_2$	774	0.0011	49	80
Mg_6	174	0.0011	9	20
$AsSn_2$	705	0.0011	60	115
$Ca_2Cl_2H_2$	972	0.0011	61	101
Au_2Br_2	352	0.0012	32	40
NSr_2	564	0.0012	50	88
S_2Ti	255	0.0012	20	45
$Br_2Er_2O_2$	690	0.0012	45	70
CNNa	765	0.0012	76	103
$ClKO_3$	705	0.0012	80	45
PTe_2Zr_2	670	0.0013	45	80
Li_2Tl_2	738	0.0013	73	75
Se_2Sn	564	0.0013	50	88
AsSb	430	0.0013	45	80
CrI_2	564	0.0013	50	88
Cl_2Co	255	0.0013	20	45
$AgClO_2$	766	0.0014	61	100
Pb_2Se_2	694	0.0014	65	76
CdO_2	255	0.0014	20	45
Br_2Mg	564	0.0014	50	88
Cl_2Zr	255	0.0015	20	45
Cl_2Zn	669	0.0015	61	101
FeI_2	564	0.0015	50	88
CoI_2	510	0.0015	45	80
Ag_2Te_2	722	0.0015	59	92
$Er_2F_2Se_2$	960	0.0015	60	100
$Br_2O_2Tm_2$	660	0.0015	43	67
$Ge_2Hf_2Te_2$	774	0.0016	49	80
$Br_2Ca_2H_2$	690	0.0016	45	70
$Er_2F_2Se_2$	930	0.0017	58	97
$Br_2Cr_2S_2$	942	0.0017	63	94
K_2PtSe_2	897	0.0017	102	57
CoH_2O_2	158	0.0017	8	22
ClH_3O	956	0.0017	81	94
I_2Ti	510	0.0017	45	80
As_2Ru_2	526	0.0017	43	67
Ca_2O_2	498	0.0017	35	72
$Br_2O_2Yb_2$	618	0.0017	40	63
Cl_2Fe	255	0.0017	20	45

Table showing the first 50 lattice matching combinations with the smallest strain needed to build the supercell.

$Gd_2S_2I_2$ (P-3m1)

Structural and electronic properties

**Formula**	$Gd_2S_2I_2$
**Spacegroup**	P-3m1
**Prototype**	SmSI
**Parent 3D**	$Gd_2I_2S_2$
**Source DB**	MPDS
**DB ID**	S1703873
**DF2-C09 Binding energy [meV/ Å^2]**	11.93
**RVV10 Binding energy [meV/ Å^2]**	N/A
**Band gap (PBE) [eV]**	0.0

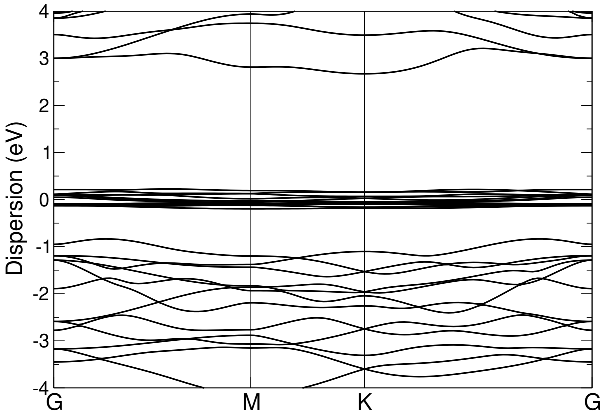

Band structure: Electronic band structure of $Gd_2S_2I_2$ (P-3m1) in a representative window around the Fermi energy and along a high-symmetry path.

Optimized crystal structure

Structural parameters: relaxed cell (top) and atomic positions (bottom) of $Gd_2S_2I_2$ (P-3m1) in Cartesian coordinates.

	x [Å]	y [Å]	z [Å]
$\mathbf{a_1}$	2.26878361	−3.92964848	0.00000000
$\mathbf{a_2}$	2.26878361	3.92964848	0.00000000
$\mathbf{a_3}$	0.00000000	0.00000000	26.83695490

		x [Å]	y [Å]	z [Å]
	Gd	3.40317541	0.65494141	-1.75536137
	Gd	1.13439180	-0.65494141	1.75536137
	S	3.40317541	0.65494141	1.02772528
	S	1.13439180	-0.65494141	-1.02772528
	I	1.13439180	1.96482424	-3.72664231
	I	1.13439180	1.96482424	3.72664231

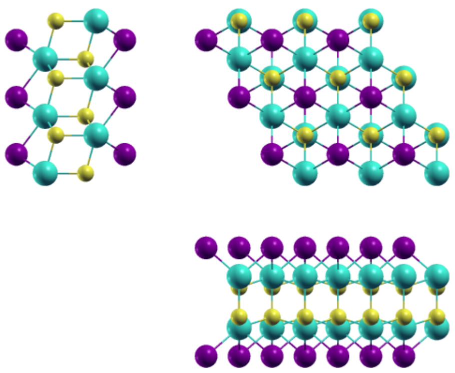

Orthographic projections: views of $Gd_2S_2I_2$ (P-3m1) as seen from the x axis (left), the y axis (bottom) and the z axis (center).

Lattice matching - minimal size

Formula	N° atoms	strain	cell size 1	cell size 2
K	7	0.1504	1	1
K	7	0.004	1	1
S_2	8	1.5617	1	1
Sb_2	8	0.271	1	1
Ba_2Pt	9	0.0094	1	1
CaI_2	9	0.0051	1	1
$GeTe_2$	9	1.5356	1	1
$HfTe_2$	9	0.2492	1	1
I_2Pr	9	0.1165	1	1
I_2Yb	9	0.0076	1	1
$AuTe_2$	9	0.2597	1	1
Cl_2Zn	9	0.4218	1	1
$PdTe_2$	9	0.2564	1	1
I_2Zn	9	0.2662	1	1
Ba_2Hg	9	0.1372	1	1
Te_2Zr	9	0.2499	1	1
I_2Nd	9	0.1169	1	1
Cl_2Cu	9	0.1939	1	1
I_2Tm	9	0.0063	1	1
$SnTe_2$	9	0.2732	1	1
DyI_2	9	0.0024	1	1
CeI_2	9	0.1161	1	1
CuO_2	9	1.6249	1	1
$PtTe_2$	9	0.2592	1	1
Br_2Cd	9	0.2551	1	1
I_2La	9	0.1193	1	1
F_2Zn	9	0.1147	1	1
Ba_2Cd	9	0.1393	1	1
NaPSn	9	0.2481	1	1
Fe_2Te_2	10	0.1113	1	1
Li_2Tl_2	10	0.4634	1	1
Ca_2Cl_2	10	0.1115	1	1
Cu_2I_2	10	0.1293	1	1
Cl_2OOs	10	0.4017	1	1
Cu_2Te_2	10	0.1128	1	1
Ir_2P_2	10	0.1168	1	1
Ag_2Br_2	10	0.1196	1	1
Br_2Er_2	10	0.2605	1	1
O_2Sn_2	10	0.1154	1	1
Cu_2S_2	10	0.1134	1	1
Au_2Br_2	10	0.1366	1	1
Ge_2Te_2	10	0.1518	1	1
Br_2Tb_2	10	0.2572	1	1
Br_2Cu_2	10	0.1141	1	1
As_2Ir_2	10	0.1209	1	1
Cu_2Te_2	10	0.4277	1	1
O_2Pb_2	10	0.1271	1	1
Cl_2La_2	10	0.2629	1	1
Br_2Gd_2	10	0.2613	1	1
O_2Sn_2	10	0.1136	1	1

Table showing the first 50 lattice matching combinations with the smallest number of atoms in the supercell.

Lattice matching - minimal strain

Formula	N° atoms	strain	cell size 1	cell size 2
MoS_2	471	0.0	39	79
$F_2Se_2Tm_2$	600	0.0	43	57
$HfTe_2$	486	0.0	49	64
In_2Se_3	614	0.0	49	64
HfS_2	171	0.0001	16	25
H_2MgO_2	794	0.0001	49	100
$PtTe_2$	627	0.0001	64	81
Br_2Zr_2	466	0.0001	37	61
H_2Si_2	412	0.0001	36	49
$LiMnSe_2$	486	0.0001	43	57
S_2W	471	0.0001	39	79
Cl_2Fe	102	0.0002	9	16
$InSe_2$	363	0.0002	36	49
MoS_2	471	0.0002	39	79
In_2Se_2	802	0.0002	73	91
$Er_2F_2Se_2$	678	0.0002	49	64
Br_2Cd	561	0.0003	57	73
$CoTe_2$	171	0.0003	16	25
Br_2Ca_3Si	12	0.0003	1	1
$Cl_2O_2Tm_2$	366	0.0003	25	36
Se_2Ti	405	0.0003	37	61
$AuTe_2$	627	0.0003	64	81
FeH_2O_2	69	0.0003	4	9
$Cl_2Er_2H_2$	510	0.0004	36	49
Ga_2S_2	196	0.0004	16	25
$CuTe_2$	171	0.0004	16	25
CaClHO	294	0.0004	25	36
Br_2PY_2	707	0.0004	57	73
I_2Mn	363	0.0005	36	49
Te_2Zr	486	0.0005	49	64
GeTe	314	0.0005	36	49
F_2Na	258	0.0005	25	36
Se_2Sn_2	814	0.0005	83	79
Cl_2Zr	102	0.0006	9	16
$Cl_2O_2Yb_2$	366	0.0006	25	36
$CdCl_2$	363	0.0006	36	49
$I_2Se_2Tb_2$	948	0.0007	91	67
$PdTe_2$	561	0.0007	57	73
In_2S_3	461	0.0007	36	49
I_2Y_2	486	0.0007	43	57
Pt_2Te_2	550	0.0007	49	64
CdO_2	102	0.0008	9	16
Ni_2Te_2	412	0.0008	36	49
$C_2Br_2La_2$	750	0.0008	58	67
S_2V	594	0.0008	49	100
CaH_2O_2	221	0.0009	16	25
N_2W	51	0.0009	4	9
Sn	314	0.0009	39	80
Br_2Er_2	708	0.0009	64	81
Li_2Tl_2	924	0.0009	100	81

Table showing the first 50 lattice matching combinations with the smallest strain needed to build the supercell.

$Gd_2Se_2I_2$ (Pmm2)

Structural and electronic properties

**Formula**	$Gd_2Se_2I_2$
**Spacegroup**	Pmm2
**Prototype**	FeOCl
**Parent 3D**	$Gd_2I_2Se_2$
**Source DB**	MPDS
**DB ID**	S376100
**DF2-C09 Binding energy [meV/ Å^2]**	10.67
**RVV10 Binding energy [meV/ Å^2]**	N/A
**Band gap (PBE) [eV]**	0.0

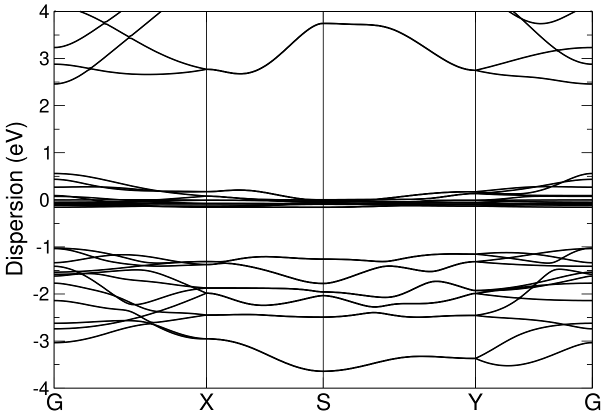

Band structure: Electronic band structure of $Gd_2Se_2I_2$ (Pmm2) in a representative window around the Fermi energy and along a high-symmetry path.

Optimized crystal structure

Structural parameters: relaxed cell (top) and atomic positions (bottom) of $Gd_2Se_2I_2$ (Pmm2) in Cartesian coordinates.

	x [Å]	y [Å]	z [Å]
$\mathbf{a_1}$	4.32631264	0.00000000	0.00000000
$\mathbf{a_2}$	0.00000000	5.63707413	0.00000000
$\mathbf{a_3}$	0.00000000	0.00000000	25.24382960

	x [Å]	y [Å]	z [Å]
I	1.08144273	-4.22780560	-3.38557103
Gd	-1.08163682	-4.22780560	-1.20000556
Gd	1.08163682	-1.40926853	1.20000556
Se	-1.08150899	-1.40926853	-0.72047475
Se	1.08150899	-4.22780560	0.72047475
I	-1.08144273	-1.40926853	3.38557103

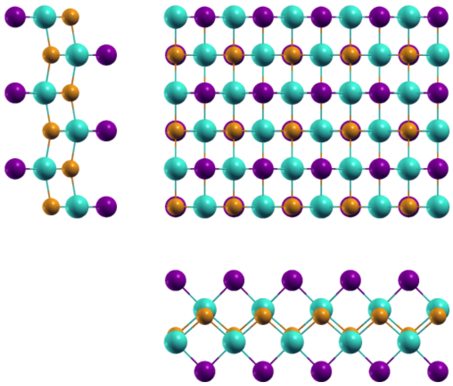

Orthographic projections: views of $Gd_2Se_2I_2$ (Pmm2) as seen from the x axis (left), the y axis (bottom) and the z axis (center).

Lattice matching - minimal size

Formula	N° atoms	strain	cell size 1	cell size 2
K	7	0.758	1	1
K	8	0.4556	1	2
Tl	8	0.1423	1	2
In_2Se_2	10	0.0845	1	1
Au_2Br_2	10	0.2552	1	1
Ge_2Te_2	10	0.261	1	1
P_2Rh_2	10	1.9312	1	1
Au_2Se_2	10	0.4951	1	1
F_2Tl_2	10	1.9322	1	1
Au_2I_2	10	0.0672	1	1
P_2	10	0.1393	1	2
$SbSe_2Tl$	10	0.1693	1	1
Se_2Sn_2	10	0.2586	1	1
KNO_3	11	0.3077	1	1
Ga_2S_3	11	0.2692	1	1
MoS_2	12	0.1327	1	2
Cl_2Mn	12	0.1458	1	2
$MoSe_2$	12	0.1418	1	2
S_2Ta	12	0.1471	1	2
$CuTe_2$	12	0.4881	1	2
Br_2Cu	12	0.5531	1	2
$Cu_2Na_2Te_2$	12	0.2588	1	1
Cl_2Ti	12	0.1394	1	2
$K_2O_2Tl_2$	12	0.2006	1	1
S_2Ti	12	0.3502	1	2
$I_2Se_2Tb_2$	12	0.002	1	1
NbS_2	12	0.1468	1	2
S_2W	12	0.1327	1	2
$Gd_2I_2S_2$	12	0.0182	1	1
Cl_2Co	12	0.3496	1	2
Pd_2S_4	12	0.3409	1	1
$CrTe_2$	12	0.3757	1	2
$Br_2Er_2Se_2$	12	0.0155	1	1
NbS_2	12	0.1441	1	2
CNRb	12	0.7475	1	2
Cl_2Fe	12	0.3482	1	2
S_2Ta	12	0.1436	1	2
Se_2V	12	0.1428	1	2
Br_2Ti	12	0.3746	1	2
$Er_2I_2Se_2$	12	0.0063	1	1
$I_2Se_2Tm_2$	12	0.0079	1	1
$SnTe_2$	12	0.6654	1	2
$Ag_2K_2Se_2$	12	0.2762	1	1
AuI_4Li	12	0.1119	1	1
BrNZr	12	0.3691	1	2
$TaTe_2$	12	0.5136	1	2
Br_2Cr	12	0.3752	1	2
Cl_2Zr	12	0.3489	1	2
$H_2I_2Sr_2$	12	0.7476	1	1
I_2Ti	12	0.5654	1	2

Table showing the first 50 lattice matching combinations with the smallest number of atoms in the supercell.

Lattice matching - minimal strain

Formula	N° atoms	strain	cell size 1	cell size 2
$Br_2H_2Zr_2$	750	0.0006	37	88
$AuCrTe_4$	348	0.0006	30	28
$F_2Lu_2Se_2$	708	0.0007	44	74
Sb_2Te_2	784	0.0007	70	91
$Br_2N_2Ti_2$	834	0.0007	49	90
$IrTe_2$	624	0.0007	54	100
Nd	246	0.0007	25	96
Hg_3S_2	930	0.0007	110	54
$PtSe_2$	291	0.0007	24	49
BiBrTe	42	0.0008	4	6
Ga_2Se_2	724	0.0008	54	100
In_2S_3	824	0.0009	54	100
$Cl_2Er_2O_2$	870	0.001	49	96
Cu_2Sr_2	48	0.001	4	6
P_4	730	0.001	59	94
GeTe	524	0.001	54	100
I_2Pb	693	0.0011	70	91
S_2	524	0.0011	54	100
$AsSn_2$	291	0.0011	24	49
I_2La_2Te	949	0.0011	74	101
$C_2I_2Y_2$	924	0.0012	57	97
$Cl_2O_2Tm_2$	870	0.0012	49	96
I_2La_2Te	857	0.0012	67	91
Ni_2SbTe_2	824	0.0012	54	100
Br_2Ca_3Si	948	0.0012	67	91
Br_2La_2	560	0.0013	44	74
C_2F_2	272	0.0013	12	50
H_2Si_2	724	0.0013	54	100
As_2O_3	270	0.0013	25	24
Te_2Zr	681	0.0014	60	107
CdClHO	340	0.0014	24	49
In_2Se_3	895	0.0014	60	107
$HfTe_2$	681	0.0014	60	107
$C_2I_2Y_2$	876	0.0014	54	92
Sb_2	412	0.0015	44	74
$Cl_2O_2Yb_2$	930	0.0015	52	103
Ni_2Se_2	982	0.0015	79	127
$HfSe_2$	621	0.0015	52	103
Te_2Ti	621	0.0016	52	103
$InSe_2$	624	0.0016	54	100
CeI_2	855	0.0016	79	127
$TaTe_2$	291	0.0016	24	49
P_2Sn_2	762	0.0016	57	105
$AgBrO_2$	698	0.0016	55	92
S_2Zn_2	400	0.0017	30	55
$Cl_2O_2Ti_2$	258	0.0017	15	28
I_2La	780	0.0017	73	114
$C_2Br_2Gd_2$	792	0.0017	48	84
$C_2Br_2Tb_2$	792	0.0017	48	84
$I_2N_2Ti_2$	870	0.0017	53	92

Table showing the first 50 lattice matching combinations with the smallest strain needed to build the supercell.

GdI_2 (P-6m2)

Structural and electronic properties

**Formula**	GdI_2
**Spacegroup**	P-6m2
**Prototype**	MoS2
**Parent 3D**	Gd_2I_4
**Source DB**	MPDS
**DB ID**	S1300639
**DF2-C09 Binding energy [meV/ Å^2]**	11.45
**RVV10 Binding energy [meV/ Å^2]**	N/A
**Band gap (PBE) [eV]**	0.0

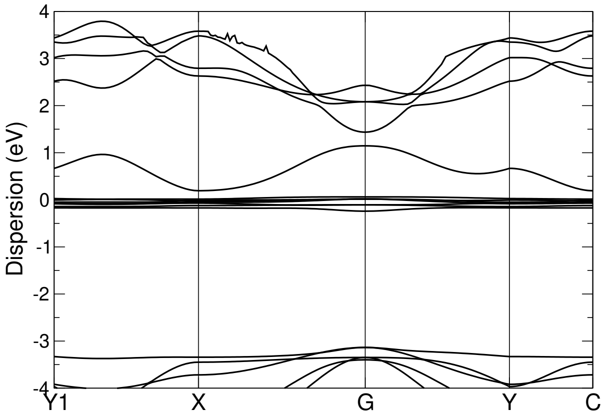

Band structure: Electronic band structure of GdI_2 (P-6m2) in a representative window around the Fermi energy and along a high-symmetry path.

Optimized crystal structure

Structural parameters: relaxed cell (top) and atomic positions (bottom) of GdI_2 (P-6m2) in Cartesian coordinates.

	x [Å]	y [Å]	z [Å]
$\mathbf{a_1}$	2.17828927	−3.77290770	0.00000000
$\mathbf{a_2}$	4.35657855	0.00000000	0.00000000
$\mathbf{a_3}$	0.00000000	0.00000000	19.93856518

	x [Å]	y [Å]	z [Å]
Gd	-2.17828927	-1.25763590	0.00000000
I	-0.00000000	-2.51527180	-1.97960314
I	-0.00000000	-2.51527180	1.97960314

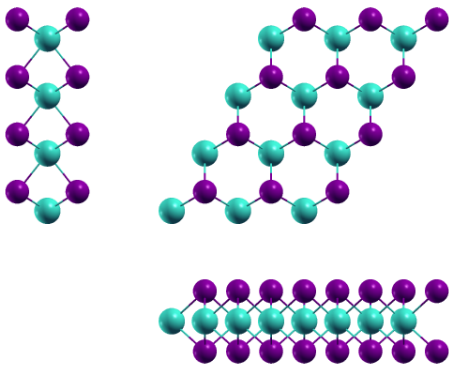

Orthographic projections: views of GdI_2 (P-6m2) as seen from the x axis (left), the y axis (bottom) and the z axis (center).

Lattice matching - minimal size

Formula	N° atoms	strain	cell size 1	cell size 2
AsSb	5	0.2545	1	1
Bi_2	5	0.0064	1	1
GeTe	5	0.2628	1	1
S_2	5	0.2651	1	1
CaCl	5	0.4326	1	1
$IrTe_2$	6	0.2641	1	1
$CdCl_2$	6	0.2613	1	1
CdI_2	6	0.0086	1	1
Br_2Ca	6	0.0074	1	1
$InSe_2$	6	0.2619	1	1
$AsSn_2$	6	1.5223	1	1
$GeTe_2$	6	0.2599	1	1
$SiTe_2$	6	0.2464	1	1
I_2Pr	6	0.1274	1	1
I_2Mn	6	0.2615	1	1
NSr_2	6	0.2567	1	1
BiClTe	6	0.0082	1	1
Cl_2Zn	6	0.1143	1	1
FeI_2	6	0.2584	1	1
I_2Ni	6	0.2602	1	1
CrI_2	6	0.2578	1	1
BiBrTe	6	0.0028	1	1
$RhTe_2$	6	1.5423	1	1
Ba_2Hg	6	0.1546	1	1
I_2Nd	6	0.1281	1	1
$NiTe_2$	6	1.5976	1	1
Cl_2Cu	6	0.0718	1	1
I_2V	6	0.2481	1	1
Se_2Zr	6	0.2469	1	1
$PtSe_2$	6	1.5268	1	1
BiITe	6	0.0037	1	1
CoI_2	6	0.2551	1	1
$MnSe_2$	6	0.4324	1	1
CeI_2	6	0.1269	1	1
Br_2Mg	6	0.2583	1	1
I_2Ti	6	0.2555	1	1
F_2Ni	6	0.1127	1	1
I_2La	6	0.1315	1	1
CdI_2	6	0.009	1	1
Se_2Sn	6	0.2564	1	1
F_2Zn	6	0.1249	1	1
Gd	6	0.1735	1	3
NaPSn	6	0.2749	1	1
I_2Pr	6	0.0082	1	1
H_2Si_2	7	0.2623	1	1
Bi_2Te_2	7	0.4511	1	1
Fe_2Te_2	7	0.1197	1	1
Ca_2Cl_2	7	0.12	1	1
Cu_2I_2	7	0.1447	1	1
Cl_2Gd_2	7	0.2683	1	1

Table showing the first 50 lattice matching combinations with the smallest number of atoms in the supercell.

Lattice matching - minimal strain

Formula	N° atoms	strain	cell size 1	cell size 2
Sm	52	0.0	9	25
$Cl_2O_2Tm_2$	471	0.0	43	57
I_2Ti	390	0.0	57	73
Ga_2Se_2	583	0.0	73	91
CaClHO	357	0.0001	43	57
HfS_2	183	0.0001	25	36
$IrTe_2$	492	0.0001	73	91
$Cl_2O_2Y_2$	531	0.0002	49	64
$CoTe_2$	183	0.0002	25	36
BrNZr	123	0.0002	16	25
AlH_4Na	483	0.0003	65	48
CoI_2	390	0.0003	57	73
CrS_2	354	0.0004	39	79
Ga_2S_2	219	0.0004	25	36
S_2Zn_2	643	0.0004	81	100
Cl_2Hf_2	471	0.0005	49	81
Cl_2Gd_2	643	0.0005	81	100
$I_2N_2Zr_2$	402	0.0005	36	49
$GeTe_2$	435	0.0005	64	81
$CuTe_2$	183	0.0005	25	36
CI_2Y_2	536	0.0005	57	73
$Cl_2Ho_2O_2$	531	0.0005	49	64
K_2PtTe_2	187	0.0005	39	14
S_2	401	0.0005	73	91
C_4Ca_2	309	0.0006	35	34
FeI_2	435	0.0006	64	81
$RhTe_2$	255	0.0006	36	49
I_2Ni	435	0.0007	64	81
Se_2Sn	390	0.0007	57	73
Br_2Hf_2	148	0.0007	16	25
Au_2Se_2	348	0.0007	60	42
$F_2Se_2Yb_2$	843	0.0007	81	100
Ni_2SbTe_2	674	0.0007	73	91
$NiTe_2$	300	0.0007	43	57
AsSb	317	0.0007	57	73
Ga_2Se_2	403	0.0007	49	64
Br_2Mg	435	0.0008	64	81
P_2Sn_2	643	0.0008	81	100
$I_2Lu_2S_2$	591	0.0008	77	60
$Cl_2Er_2S_2$	591	0.0008	77	60
In_2S_3	674	0.0008	73	91
I_2V	339	0.0008	49	64
F_2Na	300	0.0008	43	57
Hg_3N_2	317	0.0008	64	25
As_4	575	0.0009	85	80
NSr_2	390	0.0009	57	73
$Cl_2O_2Yb_2$	471	0.0009	43	57
CaH_2O_2	255	0.0009	25	36
I_2La_2P	8	0.0009	1	1
$Br_2S_2Yb_2$	693	0.001	91	70

Table showing the first 50 lattice matching combinations with the smallest strain needed to build the supercell.

GeI_2 (P-3m1)

Structural and electronic properties

**Formula**	GeI_2
**Spacegroup**	P-3m1
**Prototype**	CdI2
**Parent 3D**	Ge_3I_6
**Source DB**	ICSD
**DB ID**	23176
**DF2-C09 Binding energy [meV/ Å^2]**	12.09
**RVV10 Binding energy [meV/ Å^2]**	17.61
**Band gap (PBE) [eV]**	2.14

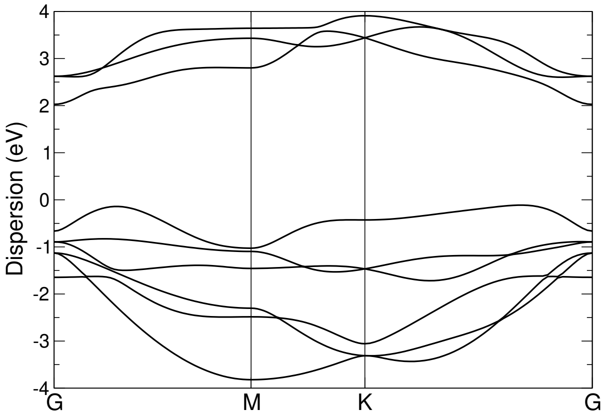

Band structure: Electronic band structure of GeI_2 (P-3m1) in a representative window around the Fermi energy and along a high-symmetry path.

Optimized crystal structure

Structural parameters: relaxed cell (top) and atomic positions (bottom) of GeI_2 (P-3m1) in Cartesian coordinates.

	x [Å]	y [Å]	z [Å]
$\mathbf{a_1}$	4.26308426	0.00000000	0.00000000
$\mathbf{a_2}$	−2.13154213	3.69193927	0.00000000
$\mathbf{a_3}$	0.00000000	0.00000000	23.54706005

		x [Å]	y [Å]	z [Å]
●	I	0.00000000	2.46129285	10.00756483
●	Ge	0.00000000	0.00000000	11.77353002
●	I	2.13154213	1.23064642	13.53949521

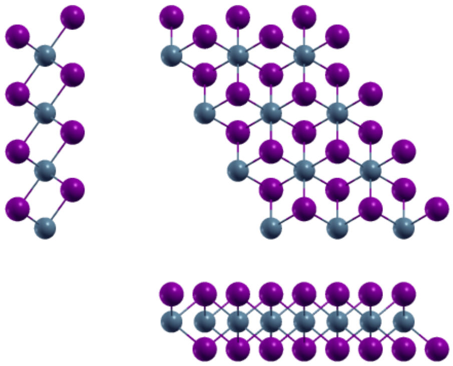

Orthographic projections: views of GeI_2 (P-3m1) as seen from the x axis (left), the y axis (bottom) and the z axis (center).

Lattice matching - minimal size

Formula	N° atoms	strain	cell size 1	cell size 2
InSe	5	0.002	1	1
AsSb	5	0.2688	1	1
Bi_2	5	0.0038	1	1
AgTl	5	0.1505	1	1
PbTe	5	0.0002	1	1
CaCl	5	0.1127	1	1
CdI_2	6	0.0015	1	1
$MoSe_2$	6	4.8754	1	1
Br_2Zn	6	0.2545	1	1
Br_2Ca	6	0.0027	1	1
HfS_2	6	1.5243	1	1
$AsSn_2$	6	1.5964	1	1
$GeTe_2$	6	0.2747	1	1
$SiTe_2$	6	0.2602	1	1
I_2Pr	6	0.1349	1	1
S_2Zr	6	1.5631	1	1
Br_2Cu	6	0.711	1	1
NSr_2	6	0.2712	1	1
PbS_2	6	0.2651	1	1
BiClTe	6	0.0019	1	1
BrCdI	6	0.009	1	1
Cl_2Zn	6	0.1192	1	1
FeI_2	6	0.2731	1	1
I_2Ni	6	0.275	1	1
HgI_2	6	0.2058	1	1
Te_2Ti	6	0.2549	1	1
CrI_2	6	0.2724	1	1
BaF_2	6	0.0058	1	1
BiBrTe	6	0.0075	1	1
$RhTe_2$	6	0.2491	1	1
$CoTe_2$	6	1.5266	1	1
AsKSn	6	0.0072	1	1
I_2Nd	6	0.1356	1	1
$NiTe_2$	6	0.2593	1	1
Cl_2Cu	6	0.0682	1	1
S_2Sn	6	1.565	1	1
I_2V	6	0.262	1	1
Se_2Zr	6	0.2607	1	1
STl_2	6	0.0048	1	1
$PtSe_2$	6	0.2462	1	1
CoI_2	6	0.2694	1	1
GeS_2	6	0.4283	1	1
$MnSe_2$	6	0.1126	1	1
Br_2Ni	6	1.5384	1	1
CeI_2	6	0.1343	1	1
Br_2Mg	6	0.2729	1	1
I_2Ti	6	0.2699	1	1
$NbTe_2$	6	1.5616	1	1
Cl_2Mg	6	1.5388	1	1
F_2Ni	6	0.1171	1	1

Table showing the first 50 lattice matching combinations with the smallest number of atoms in the supercell.

Lattice matching - minimal strain

Formula	N° atoms	strain	cell size 1	cell size 2
$MoSe_2$	390	0.0	49	81
$NiTe_2$	435	0.0	64	81
IKO_3	120	0.0	25	9
Ga_2Se_2	583	0.0	73	91
$TaTe_2$	300	0.0	43	57
Cl_2Co	123	0.0001	16	25
Tl	172	0.0001	37	61
$RhTe_2$	339	0.0001	49	64
PTe_2Zr_2	743	0.0001	81	100
NS_2Zr	219	0.0001	25	36
Se_2W	390	0.0002	49	81
H_2NiO_2	107	0.0002	9	16
As_2CeLi_2	8	0.0002	1	1
Ga_2S_3	255	0.0002	25	36
$ClKO_3$	317	0.0002	64	25
PbTe	5	0.0002	1	1
CdO_2	123	0.0002	16	25
S_2Ti	123	0.0003	16	25
Br_2CsF	387	0.0003	65	48
Bi_2Te_2	499	0.0003	81	64
$LiMnTe_2$	7	0.0003	1	1
Br_2Y_2	583	0.0003	73	91
$Br_2Er_2S_2$	747	0.0004	101	74
$HfSe_2$	390	0.0004	57	73
Te_2Ti	390	0.0004	57	73
Cl_2Zr	123	0.0004	16	25
Au_2Br_2	478	0.0004	74	64
Se_2W	294	0.0005	37	61
PtS_2	183	0.0005	25	36
Br_2Pr_2	463	0.0005	57	73
O_2Zn	222	0.0005	25	49
$AsSn_2$	300	0.0005	43	57
PbS_2	492	0.0005	73	91
$Cl_2O_2Y_2$	765	0.0006	73	91
Na	111	0.0006	25	36
CaClHO	516	0.0006	64	81
$Hf_2I_2N_2$	471	0.0006	43	57
CdClHO	357	0.0006	43	57
$MoSe_2$	294	0.0006	37	61
$SiTe_2$	435	0.0006	64	81
Bi_2STe_2	8	0.0006	1	1
Cu_2O_4	852	0.0007	78	103
ClH_3O	638	0.0007	91	73
Br_2Zn	390	0.0007	57	73
$Cl_2O_2Tm_2$	678	0.0007	64	81
$Br_2N_2Zr_2$	402	0.0007	36	49
$Cl_2Er_2S_2$	531	0.0008	71	53
CBr_2Lu_2	353	0.0008	36	49
Cl_2Fe	123	0.0008	16	25
$Cl_2H_2Lu_2$	402	0.0008	36	49

Table showing the first 50 lattice matching combinations with the smallest strain needed to build the supercell.

GeI_2 (P-6m2)

Structural and electronic properties

**Formula**	GeI_2
**Spacegroup**	P-6m2
**Prototype**	MoS2
**Parent 3D**	GeI_2
**Source DB**	COD
**DB ID**	1010319
**DF2-C09 Binding energy [meV/ Å^2]**	8.81
**RVV10 Binding energy [meV/ Å^2]**	13.33
**Band gap (PBE) [eV]**	2.04

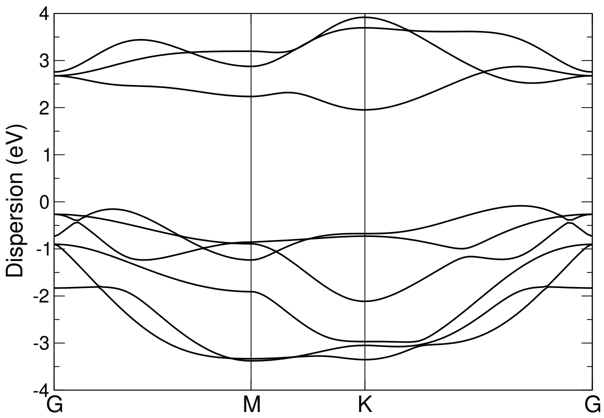

Band structure: Electronic band structure of GeI_2 (P-6m2) in a representative window around the Fermi energy and along a high-symmetry path.

Optimized crystal structure

Structural parameters: relaxed cell (top) and atomic positions (bottom) of GeI_2 (P-6m2) in Cartesian coordinates.

	x [Å]	y [Å]	z [Å]
$\mathbf{a_1}$	4.13721237	0.00000000	0.00000000
$\mathbf{a_2}$	−2.06860618	3.58293101	0.00000000
$\mathbf{a_3}$	0.00000000	0.00000000	23.87131395

		x [Å]	y [Å]	z [Å]
●	I	2.06860618	1.19431034	13.86267248
●	Ge	0.00000000	0.00000000	11.93565697
●	I	2.06860618	1.19431034	10.00864147

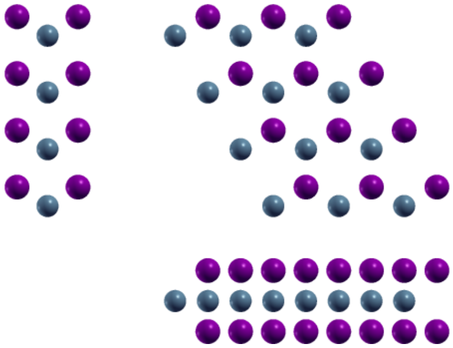

Orthographic projections: views of GeI_2 (P-6m2) as seen from the x axis (left), the y axis (bottom) and the z axis (center).

Lattice matching - minimal size

Formula	N° atoms	strain	cell size 1	cell size 2
K	4	0.4558	1	1
Na	4	1.549	1	1
AgTl	5	0.1651	1	1
Ag_2	5	0.4695	1	1
As_2	5	0.2473	1	1
Sb_2	5	0.0038	1	1
CaCl	5	0.119	1	1
Cl_2Zn	6	1.5994	1	1
I_2Mg	6	0.0019	1	1
$MoTe_2$	6	1.5653	1	1
PSn_2	6	0.2593	1	1
Ba_2Pt	6	0.4689	1	1
Br_2Zn	6	0.2746	1	1
HfS_2	6	1.6277	1	1
$AsSn_2$	6	0.2646	1	1
I_2Pr	6	0.1469	1	1
$CuTe_2$	6	0.2504	1	1
S_2Zr	6	0.2582	1	1
Br_2La	6	0.0022	1	1
Ca_2Si	6	0.4804	1	1
Br_2Co	6	0.2467	1	1
Ca_2N	6	0.248	1	1
BrCdI	6	0.005	1	1
Cl_2Zn	6	0.1277	1	1
Te_2Ti	6	0.275	1	1
I_2Zn	6	0.007	1	1
BaF_2	6	0.0084	1	1
$RhTe_2$	6	0.2686	1	1
Br_2Mn	6	1.5906	1	1
PtS_2	6	1.5564	1	1
$CoTe_2$	6	1.6301	1	1
Se_2Ti	6	1.5374	1	1
AsKSn	6	0.007	1	1
Te_2W	6	1.5665	1	1
$PbTe_2$	6	0.004	1	1
I_2Nd	6	0.1478	1	1
S_2Sn	6	0.2586	1	1
$SnTe_2$	6	0.0022	1	1
Sn	6	0.6335	1	3
Cl_2V	6	4.8648	1	1
I_2Pb	6	0.4735	1	1
STl_2	6	0.0094	1	1
$PtSe_2$	6	0.2655	1	1
Br_2Fe	6	0.2468	1	1
GeS_2	6	0.1134	1	1
$TaTe_2$	6	0.2638	1	1
$MnSe_2$	6	0.119	1	1
Br_2Ni	6	0.2536	1	1
CeI_2	6	0.1462	1	1
CuO_2	6	0.175	1	1

Table showing the first 50 lattice matching combinations with the smallest number of atoms in the supercell.

Lattice matching - minimal strain

Formula	N° atoms	strain	cell size 1	cell size 2
PSn_2	435	0.0	64	81
CdClHO	643	0.0	81	100
ReS_2	75	0.0001	9	16
$CrSe_2$	294	0.0001	37	61
KS_2Ti	357	0.0002	43	57
$AsSn_2$	492	0.0002	73	91
$Cl_2N_2Zr_2$	531	0.0002	49	64
Ba_2Ni_3	8	0.0002	1	1
Ga_2Te_2	7	0.0002	1	1
Se_2Yb	6	0.0002	1	1
Br_2Mn	300	0.0002	43	57
Na	157	0.0003	36	49
ClH_3O	386	0.0003	57	43
$I_2Pr_2Si_2$	9	0.0003	1	1
$Br_2Hf_2N_2$	531	0.0003	49	64
HgI_2	339	0.0004	65	48
Br_2V	183	0.0004	25	36
$TaTe_2$	492	0.0004	73	91
Cl_2Zr_2	219	0.0004	25	36
Sb_2Te_2	499	0.0004	81	64
CaH_2O_2	467	0.0004	49	64
$AsCuLi_2$	7	0.0005	1	1
Cl_2V	390	0.0005	49	81
$BiTe_2$	6	0.0005	1	1
S_2Sn	435	0.0005	64	81
$Br_2N_2Zr_2$	609	0.0005	57	73
CBr_2Lu_2	536	0.0006	57	73
MnO_2	39	0.0006	4	9
$AgBrO_2$	291	0.0006	41	42
Ga_2S_3	353	0.0006	36	49
NS_2Zr	304	0.0007	36	49
$RhTe_2$	543	0.0007	81	100
Ag_2I_2	387	0.0007	65	48
$AgNO_3$	155	0.0008	25	16
S_2Zr	435	0.0008	64	81
Ba_2Pt	492	0.0008	91	73
CCl_2Sc_2	255	0.0008	25	36
$C_2Cl_2Y_2$	576	0.0008	58	67
$PtSe_2$	492	0.0008	73	91
Sb_2Se_2Te	8	0.0008	1	1
$MoSe_2$	123	0.0009	16	25
Au_2I_2	639	0.0009	105	81
$CuTe_2$	339	0.0009	49	64
In	303	0.0009	65	108
Br_2HLa	7	0.0009	1	1
$Hf_2I_2N_2$	765	0.0009	73	91
CdClHO	583	0.001	73	91
Ca_2N	339	0.001	49	64
Cl_2Zn	300	0.001	43	57
I_2Pb	435	0.001	81	64

Table showing the first 50 lattice matching combinations with the smallest strain needed to build the supercell.

GeS (Pmn2_1)

Structural and electronic properties

**Formula**	GeS
**Spacegroup**	Pmn2_1
**Prototype**	GeS
**Parent 3D**	Ge_4S_4
**Source DB**	COD
**DB ID**	9008784
**DF2-C09 Binding energy [meV/ Å^2]**	37.26
**RVV10 Binding energy [meV/ Å^2]**	34.84
**Band gap (PBE) [eV]**	1.65

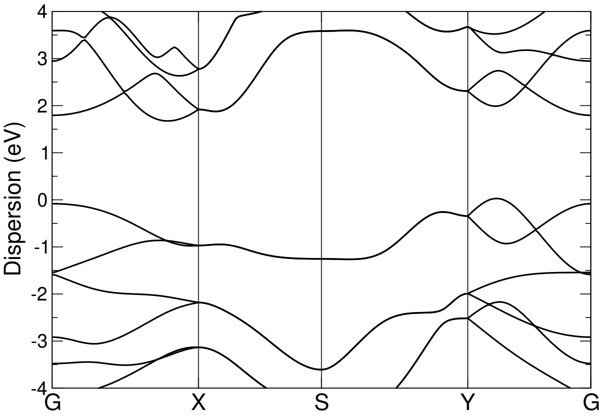

Band structure: Electronic band structure of GeS (Pmn2_1) in a representative window around the Fermi energy and along a high-symmetry path.

Optimized crystal structure

Structural parameters: relaxed cell (top) and atomic positions (bottom) of GeS (Pmn2_1) in Cartesian coordinates.

		x [Å]	y [Å]	z [Å]
$\mathbf{a_1}$		3.67799801	0.00000000	0.00000000
$\mathbf{a_2}$		0.00000000	4.37455914	0.00000000
$\mathbf{a_3}$		0.00000000	0.00000000	22.68057985
		x [Å]	y [Å]	z [Å]
●	Ge	0.91949950	3.83522480	12.61715616
●	Ge	2.75849851	1.64884706	10.06347544
●	S	2.75849851	2.18701602	12.42302720
●	S	0.91949950	4.37515572	10.25750375

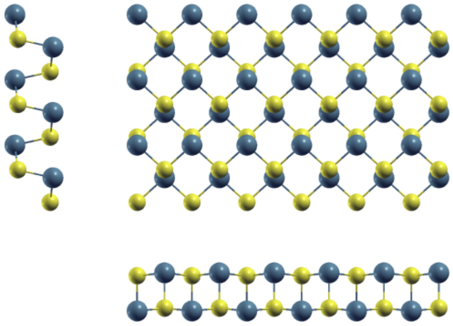

Orthographic projections: views of GeS (Pmn2_1) as seen from the x axis (left), the y axis (bottom) and the z axis (center).

Lattice matching - minimal size

Formula	N° atoms	strain	cell size 1	cell size 2
K	5	0.0632	1	1
Nd	7	0.1934	1	3
I_2Pr	7	0.3081	1	1
LiO_2	7	0.1431	1	1
Cl_2Zn	7	0.2698	1	1
Ba_2Hg	7	0.2952	1	1
CNRb	7	1.0274	1	1
CKN	7	1.1965	1	1
I_2Nd	7	0.3097	1	1
CeI_2	7	0.3068	1	1
F_2Ni	7	0.0487	1	1
I_2La	7	0.318	1	1
F_2Zn	7	0.3014	1	1
Ba_2Cd	7	0.2997	1	1
Fe_2Te_2	8	0.2871	1	1
Li_2Tl_2	8	0.2446	1	1
Ca_2Cl_2	8	0.2878	1	1
Cu_2I_2	8	0.2782	1	1
Cl_2OOs	8	0.2651	1	1
Cu_2Te_2	8	0.1729	1	1
Ir_2P_2	8	0.3093	1	1
Ag_2Br_2	8	0.3189	1	1
$AgNO_2$	8	0.1492	1	1
$AgCuTe_2$	8	0.1755	1	1
O_2Sn_2	8	0.0246	1	1
S_2Sn_2	8	0.0364	1	1
Cu_2S_2	8	0.2961	1	1
Au_2Br_2	8	0.3006	1	1
$AlLiTe_2$	8	0.5427	1	1
Ge_2Te_2	8	0.0513	1	1
Br_2Cu_2	8	0.2991	1	1
Fe_2Se_2	8	0.049	1	1
Cl_2ORu	8	0.2578	1	1
N_3Na	8	0.3814	1	1
As_4	8	0.0273	1	1
P_4	8	0.1991	1	1
O_2Sn_2	8	0.297	1	1
P_2Rh_2	8	0.295	1	1
F_2Tl_2	8	0.2952	1	1
Au_2I_2	8	0.0731	1	1
O_2Sn_2	8	0.0664	1	1
Ge_2Se_2	8	0.0251	1	1
Cu_2Se_2	8	0.0484	1	1
Ag_2Te_2	8	0.2958	1	1
As_2Ru_2	8	0.2881	1	1
Bi_2O_2	8	0.2749	1	1
$AgClO_2$	8	0.1005	1	1
La_2S_2	8	0.0538	1	1
Ni_2Se_2	8	0.3064	1	1
As_2Rh_2	8	0.3184	1	1

Table showing the first 50 lattice matching combinations with the smallest number of atoms in the supercell.

Lattice matching - minimal strain

Formula	N° atoms	strain	cell size 1	cell size 2
CrO_2	421	0.0001	37	91
AsI_2La_2	368	0.0004	42	40
Cd_2I_3	368	0.0004	42	40
$Ga_2I_2Y_2$	412	0.0004	40	42
CoO_2	528	0.0005	48	112
CNRb	854	0.0005	140	98
BaF_2	286	0.0006	40	42
NiO_2	528	0.0006	48	112
H_2Li_2Pd	440	0.0008	35	60
O_2Pt	288	0.0008	30	56
$GeNi_3Te_2$	890	0.0008	80	95
$AgTe_2$	447	0.0009	51	81
Se_2Sn_2	52	0.0009	7	6
In	176	0.0009	30	56
$I_2Se_2Tb_2$	504	0.0009	63	42
N_2Re	661	0.001	61	139
KNO_3	688	0.001	112	48
$AgNO_2$	696	0.001	88	86
$I_2Sb_2Te_2$	706	0.001	100	51
AsKSn	286	0.001	40	42
NaPSn	605	0.0011	80	95
Mg_2	260	0.0012	35	60
Cu_2F_4	558	0.0013	75	43
H_4Ti	440	0.0013	35	60
Pt_2Te_2	700	0.0013	80	95
$AgBrO_2$	760	0.0013	90	100
In_2Se_2	820	0.0013	120	85
Br_2Zr_2	796	0.0014	80	119
$I_2Pr_2Si_2$	852	0.0014	81	88
NaPSn	574	0.0014	76	90
$Cl_2N_2Ti_2$	838	0.0014	73	91
Li_2Tl_2	684	0.0014	99	72
Se_2Ti	677	0.0014	80	119
$Cl_2N_2Ti_2$	828	0.0014	72	90
$GeNi_3Te_2$	844	0.0014	76	90
Pt_2Te_2	664	0.0014	76	90
STl_2	286	0.0015	40	42
CaI_2	595	0.0015	88	81
I_2La_2	328	0.0015	40	42
NbS_2	588	0.0015	66	108
Cl_2NSc_2	804	0.0015	66	108
$C_2I_2Y_2$	870	0.0016	81	91
N_2W	423	0.0016	42	85
$Er_2F_2Se_2$	844	0.0016	76	90
$Cl_2Fe_2O_2$	820	0.0017	67	92
Li_2Tl_2	656	0.0017	95	69
$AlLiTe_2$	828	0.0017	105	102
$Ga_2Ge_2Te_2$	636	0.0017	60	66
Li_2P_2Pr	570	0.0017	60	66
Li_2Tl_2	560	0.0017	81	59

Table showing the first 50 lattice matching combinations with the smallest strain needed to build the supercell.

GeS_2 (P-4m2)

Structural and electronic properties

**Formula**	GeS_2
**Spacegroup**	P-4m2
**Prototype**	HgI2
**Parent 3D**	Ge_2S_4
**Source DB**	MPDS
**DB ID**	S1831556
**DF2-C09 Binding energy [meV/ Å^2]**	17.62
**RVV10 Binding energy [meV/ Å^2]**	N/A
**Band gap (PBE) [eV]**	1.37

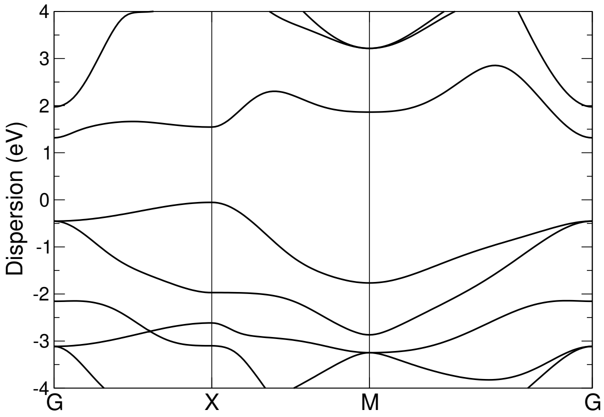

Band structure: Electronic band structure of GeS_2 (P-4m2) in a representative window around the Fermi energy and along a high-symmetry path.

Optimized crystal structure

Structural parameters: relaxed cell (top) and atomic positions (bottom) of GeS_2 (P-4m2) in Cartesian coordinates.

	x [Å]	y [Å]	z [Å]
$\mathbf{a_1}$	3.50889379	0.00000000	0.00000000
$\mathbf{a_2}$	0.00000000	3.50889379	0.00000000
$\mathbf{a_3}$	0.00000000	0.00000000	17.71474278

		x [Å]	y [Å]	z [Å]
●	Ge	0.00000000	0.00000000	0.00000000
●	S	1.75444690	0.00000000	1.42532262
●	S	0.00000000	-1.75444690	-1.42532262

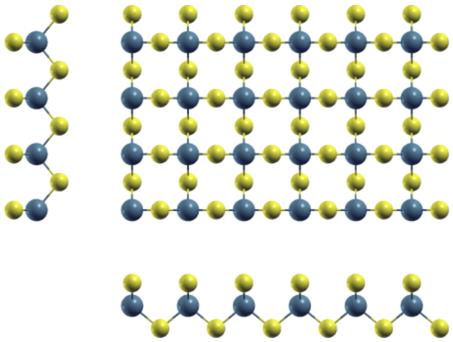

Orthographic projections: views of GeS_2 (P-4m2) as seen from the x axis (left), the y axis (bottom) and the z axis (center).

Lattice matching - minimal size

Formula	N° atoms	strain	cell size 1	cell size 2
Tl	4	0.2203	1	1
Tl	4	0.1084	1	1
InSe	5	0.7702	1	1
AsSb	5	0.1491	1	1
K	5	1.6879	1	2
GeTe	5	0.155	1	1
S_2	5	0.1566	1	1
PbTe	5	0.777	1	1
Mg_2	5	0.5882	1	1
Sb_2	5	0.1831	1	1
$IrTe_2$	6	0.1559	1	1
I_2Mg	6	0.1903	1	1
$CdCl_2$	6	0.1539	1	1
CdI_2	6	0.7837	1	1
PSn_2	6	0.1315	1	1
Br_2Zn	6	0.1399	1	1
Br_2Ca	6	0.7885	1	1
$InSe_2$	6	0.1543	1	1
$AsSn_2$	6	0.1343	1	1
$GeTe_2$	6	0.1529	1	1
$SiTe_2$	6	0.1435	1	1
$HfTe_2$	6	0.1647	1	1
I_2Pr	6	0.3842	1	1
I_2Mn	6	0.154	1	1
S_2Zr	6	0.1309	1	1
Br_2La	6	0.1907	1	1
NSr_2	6	0.1506	1	1
PbS_2	6	0.1466	1	1
BiClTe	6	0.7851	1	1
$AuTe_2$	6	0.1735	1	1
BrCdI	6	0.1944	1	1
$PdTe_2$	6	0.1707	1	1
FeI_2	6	0.1518	1	1
I_2Ni	6	0.1531	1	1
Mg_3	6	0.2136	1	1
Te_2Ti	6	0.1402	1	1
CrI_2	6	0.1514	1	1
I_2Zn	6	0.179	1	1
$RhTe_2$	6	0.1366	1	1
GeI_2	6	0.1879	1	1
S_2Ta	6	0.1089	1	1
Ba_2N	6	0.1664	1	1
Se_2V	6	0.1086	1	1
AsKSn	6	0.1969	1	1
Te_2Zr	6	0.1653	1	1
$PbTe_2$	6	0.1931	1	1
I_2Nd	6	0.3863	1	1
$NiTe_2$	6	0.1429	1	1
S_2Sn	6	0.1311	1	1
$SnTe_2$	6	0.185	1	1

Table showing the first 50 lattice matching combinations with the smallest number of atoms in the supercell.

Lattice matching - minimal strain

Formula	N° atoms	strain	cell size 1	cell size 2
Mg_3	441	0.0	65	82
$Br_2Nd_2O_2$	636	0.0001	82	65
$H_2I_2Yb_2$	627	0.0001	81	64
Mg_4	650	0.0001	82	101
Ni_2Se_2	624	0.0001	100	81
$Eu_2H_2I_2$	486	0.0001	64	49
CeI_2	543	0.0001	100	81
$I_2O_2Y_2$	636	0.0001	82	65
Cu_2I_2	291	0.0002	49	36
$Br_2Eu_2O_2$	786	0.0002	100	81
$Br_2H_2Sr_2$	363	0.0002	49	36
H_4Ti	467	0.0002	49	64
Tl	347	0.0002	82	101
$F_2I_2Yb_2$	486	0.0003	64	49
$Cl_2S_2Tl_2$	891	0.0003	149	74
$Eu_2I_2O_2$	495	0.0003	65	50
H_2Li_2Pd	467	0.0003	49	64
$As_2Cd_2K_2$	639	0.0004	97	58
$F_2I_2Pb_2$	258	0.0004	36	25
As_2Ir_2	887	0.0004	145	113
HgI_2	513	0.0004	106	65
Mg_4	643	0.0005	81	100
$I_2O_2Sm_2$	486	0.0005	64	49
Ba_2Cd	183	0.0005	36	25
Br_2Ca_3Si	840	0.0006	118	81
$Ba_2Ge_2Mn_2$	486	0.0006	64	49
Ho_2S_2	407	0.0006	65	53
I_2La_2Te	759	0.0007	118	81
Ag_2I_2	578	0.0007	106	65
$Br_2Nd_2O_2$	627	0.0007	81	64
I_2La	435	0.0007	81	64
Mg_3	435	0.0007	64	81
I_2Nd	978	0.0008	181	145
$I_2O_2Pr_2$	849	0.0008	113	85
Tl	343	0.0008	81	100
H_2Na_2Pd	8	0.0008	1	1
$K_2Mn_2Sb_2$	495	0.0008	65	50
$Br_2Ce_2O_2$	495	0.0008	65	50
$Bi_2Cl_2O_2$	795	0.0008	101	82
$F_2Se_2Y_2$	714	0.0008	108	65
As_2Fe_2	7	0.0009	1	1
Pb_2Se_2	708	0.0009	128	81
I_2Pr	543	0.0009	100	81
NaO_4	743	0.0009	131	70
$I_2O_2Y_2$	627	0.0009	81	64
$AgNO_3$	743	0.0009	131	70
As_2Rh_2	499	0.001	81	64
Te_4W_2	57	0.001	9	5
Bi_2Se_4	282	0.001	54	20
$Bi_2Br_2O_2$	636	0.0011	82	65

Table showing the first 50 lattice matching combinations with the smallest strain needed to build the supercell.

GeSe ($Pmn2_1$)

Structural and electronic properties

**Formula**	GeSe
**Spacegroup**	$Pmn2_1$
**Prototype**	GeS
**Parent 3D**	Ge_4Se_4
**Source DB**	COD
**DB ID**	1528768
**DF2-C09 Binding energy [meV/ Å^2]**	35.97
**RVV10 Binding energy [meV/ Å^2]**	33.9
**Band gap (PBE) [eV]**	1.05

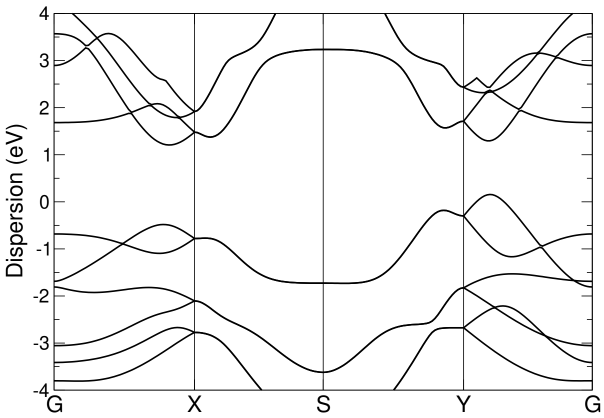

Band structure: Electronic band structure of GeSe ($Pmn2_1$) in a representative window around the Fermi energy and along a high-symmetry path.

Optimized crystal structure

Structural parameters: relaxed cell (top) and atomic positions (bottom) of GeSe ($Pmn2_1$) in Cartesian coordinates.

	x [Å]	y [Å]	z [Å]
$\mathbf{a_1}$	3.93789224	0.00000012	0.00000000
$\mathbf{a_2}$	0.00000014	4.29653405	0.00000000
$\mathbf{a_3}$	0.00000000	0.00000000	22.79413914

		x [Å]	y [Å]	z [Å]
●	Ge	0.98620063	1.72888979	10.18720195
●	Ge	2.95507606	3.87715546	12.60694003
●	Se	0.98609613	2.09843874	12.70273733
●	Se	2.95518213	4.24670631	10.09139896

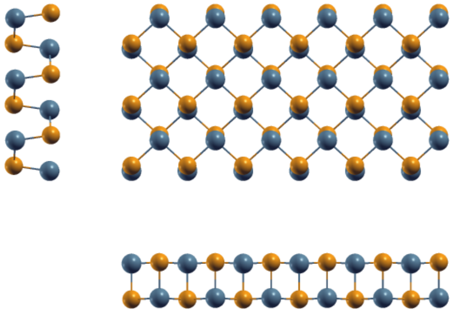

Orthographic projections: views of GeSe ($Pmn2_1$) as seen from the x axis (left), the y axis (bottom) and the z axis (center).

Lattice matching - minimal size

Formula	N° atoms	strain	cell size 1	cell size 2
K	5	0.0337	1	1
AgTl	6	0.0106	1	1
Nd	7	0.1712	1	3
I_2Pr	7	0.029	1	1
Br_2La	7	0.1223	1	1
Br_2Cu	7	0.1069	1	1
Ca_2Si	7	0.6266	1	1
BrCdI	7	0.1239	1	1
Cl_2Zn	7	0.2354	1	1
HgI_2	7	0.3443	1	1
$PbTe_2$	7	0.1233	1	1
I_2Nd	7	0.0284	1	1
Cl_2Cu	7	0.1035	1	1
I_2Pb	7	0.618	1	1
CeI_2	7	0.0295	1	1
I_2La	7	0.0256	1	1
F_2Zn	7	0.0317	1	1
Bi_2Te_2	8	0.1843	1	1
Fe_2Te_2	8	0.2502	1	1
Li_2Tl_2	8	0.2173	1	1
Ca_2Cl_2	8	0.2508	1	1
Cl_2OOs	8	0.232	1	1
Ir_2P_2	8	0.0286	1	1
Ag_2Br_2	8	0.0253	1	1
$AgNO_2$	8	0.1147	1	1
S_2Sn_2	8	0.0101	1	1
Cu_2S_2	8	0.258	1	1
Au_2Br_2	8	0.0296	1	1
Br_2Cu_2	8	0.2606	1	1
N_3Na	8	0.0679	1	1
As_2Ir_2	8	0.024	1	1
Cu_2Te_2	8	0.2387	1	1
Ge_2S_2	8	0.0221	1	1
$MnNaTe_2$	8	0.1237	1	1
Bi_2Se_2	8	0.0254	1	1
O_2Sn_2	8	0.2588	1	1
Cu_2I_2	8	0.1226	1	1
P_2Rh_2	8	0.2571	1	1
F_2Tl_2	8	0.2573	1	1
BN	8	0.1455	1	2
Au_2I_2	8	0.0448	1	1
Sb_2Te_2	8	0.6225	1	1
O_2Sn_2	8	1.655	1	1
Ag_2Te_2	8	0.2586	1	1
As_2Ru_2	8	0.2511	1	1
C_2	8	0.1423	1	2
Gd_2I_2	8	0.1225	1	1
Ni_2Se_2	8	0.0297	1	1
As_2Rh_2	8	0.0254	1	1
Fe_2SeTe	8	0.2428	1	1

Table showing the first 50 lattice matching combinations with the smallest number of atoms in the supercell.

Lattice matching - minimal strain

Formula	N° atoms	strain	cell size 1	cell size 2
Fe_2O_4	414	0.0004	30	49
Br_2Mn	581	0.0004	68	103
$Ga_2Ge_2Te_2$	634	0.0005	58	67
AlH_4Na	574	0.0005	67	51
Li_2P_2Pr	567	0.0005	58	67
KS_2Ti	684	0.0006	68	103
$Dy_2I_2S_2$	782	0.0006	80	77
H_2Li_2Pd	455	0.0006	35	63
$AsLi_3$	700	0.0007	84	91
$Hf_2Se_2Si_2$	938	0.0007	77	105
InSe	518	0.0007	84	91
As_2Li_2Pr	791	0.0007	84	91
$I_2La_2Si_2$	882	0.0007	84	91
Fe_2S_2	472	0.0008	51	67
$MoSe_2$	299	0.0008	32	57
MnO_2	820	0.0009	70	180
BH_4Li	634	0.0009	58	67
Se_2W	299	0.001	32	57
CNRb	354	0.001	57	42
Bi_2STe_2	791	0.001	84	91
H_4Ti	455	0.001	35	63
Sb_2	366	0.001	58	67
As_2Li_2Nd	791	0.0011	84	91
As_2Fe_2	728	0.0011	77	105
DyI_2	551	0.0011	80	77
C_4Ca_2	1000	0.0012	100	100
$AlLiTe_2$	920	0.0012	114	116
Br_2Ca_3Si	408	0.0012	42	40
GeI_2Y_2	791	0.0012	84	91
Br_2La_2	500	0.0013	58	67
Cl_2Zn	581	0.0013	68	103
CrO_2	820	0.0014	70	180
O_2Sn_2	964	0.0014	102	139
BH_4Li	578	0.0014	53	61
Tl	185	0.0015	32	57
Ho_2S_2	532	0.0015	63	70
$MnSe_2$	405	0.0015	51	67
$I_2S_2Tb_2$	782	0.0015	80	77
BiITe	804	0.0015	114	116
Te_2V	581	0.0015	68	103
$CeLi_2P_2$	567	0.0016	58	67
$CeLi_2P_2$	517	0.0016	53	61
O_2Sn_2	964	0.0016	102	139
O_2Sn_2	584	0.0016	62	84
CaCl	338	0.0016	51	67
Cl_2ORu	556	0.0016	61	78
Mg_2	266	0.0016	35	63
Cl_4Mg_2	336	0.0016	54	20
Bi_2Se_2Te	791	0.0017	84	91
CoO_2	467	0.0017	41	101

Table showing the first 50 lattice matching combinations with the smallest strain needed to build the supercell.

Hf_2Br_2 (P-3m1)

Structural and electronic properties

**Formula**	Hf_2Br_2
**Spacegroup**	P-3m1
**Prototype**	PtTe
**Parent 3D**	Br_2Hf_2
**Source DB**	MPDS
**DB ID**	S546529
**DF2-C09 Binding energy [meV/ Å^2]**	15.75
**RVV10 Binding energy [meV/ Å^2]**	N/A
**Band gap (PBE) [eV]**	0.0

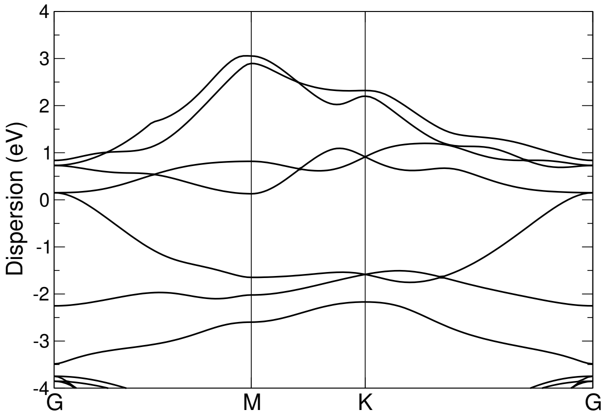

Band structure: Electronic band structure of Hf_2Br_2 (P-3m1) in a representative window around the Fermi energy and along a high-symmetry path.

Optimized crystal structure

Structural parameters: relaxed cell (top) and atomic positions (bottom) of Hf_2Br_2 (P-3m1) in Cartesian coordinates.

	x [Å]	y [Å]	z [Å]
$\mathbf{a_1}$	1.74017947	−3.01407926	0.00000000
$\mathbf{a_2}$	1.74017947	3.01407926	0.00000000
$\mathbf{a_3}$	0.00000000	0.00000000	24.08473971

		x [Å]	y [Å]	z [Å]
●	Hf	1.74017947	1.00469309	-1.13206949
●	Br	0.00000000	0.00000000	-3.03920179
●	Hf	1.74017947	-1.00469309	1.13206949
●	Br	0.00000000	0.00000000	3.03920179

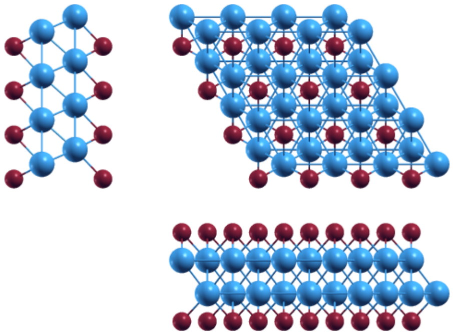

Orthographic projections: views of Hf_2Br_2 (P-3m1) as seen from the x axis (left), the y axis (bottom) and the z axis (center).

Lattice matching - minimal size

Formula	N° atoms	strain	cell size 1	cell size 2
Nd	5	4.8751	1	1
Tl	5	0.132	1	1
Sn	5	0.1136	1	1
Na	5	0.0093	1	1
In	5	0.1154	1	1
In	5	0.2737	1	1
HgO	6	0.1417	1	1
AsSb	6	0.4541	1	1
GeTe	6	0.4692	1	1
S_2	6	0.4732	1	1
Mg_2	6	0.1213	1	1
$IrTe_2$	7	0.4715	1	1
$CdCl_2$	7	0.4665	1	1
$AgTe_2$	7	0.1343	1	1
$ReSe_2$	7	0.0032	1	1
$InSe_2$	7	0.4674	1	1
$GeTe_2$	7	0.464	1	1
$HfTe_2$	7	0.4931	1	1
I_2Mn	7	0.4668	1	1
NSr_2	7	0.4581	1	1
PbS_2	7	0.4477	1	1
ReS_2	7	0.2612	1	1
LiO_2	7	2.6906	1	1
FeI_2	7	0.4613	1	1
I_2Ni	7	0.4645	1	1
S_2Ti	7	0.0091	1	1
Mg_3	7	0.1278	1	1
CrI_2	7	0.4602	1	1
Bi_2Pd	7	0.1504	1	1
Cl_2Ni	7	0.0023	1	1
$CrTe_2$	7	0.0038	1	1
Br_2V	7	0.004	1	1
ClNZr	7	0.0061	1	1
Ba_2N	7	0.4972	1	1
Se_2Ti	7	0.0076	1	1
Br_2Ti	7	0.0032	1	1
Te_2Zr	7	0.4944	1	1
$AsSe_2$	7	0.0009	1	1
BrNZr	7	0.0004	1	1
$NbSe_2$	7	0.0019	1	1
CoI_2	7	0.4551	1	1
O_2Zn	7	0.2485	1	1
Br_2Cr	7	0.0035	1	1
$FeSe_2$	7	0.1112	1	1
Se_2Ta	7	0.0018	1	1
Br_2Mg	7	0.4609	1	1
CuO_2	7	2.8693	1	1
I_2Ti	7	0.456	1	1
$NbSe_2$	7	0.0006	1	1
Se_2Ta	7	0.0024	1	1

Table showing the first 50 lattice matching combinations with the smallest number of atoms in the supercell.

Lattice matching - minimal strain

Formula	N° atoms	strain	cell size 1	cell size 2
Nd	277	0.0	49	81
$F_2Se_2Yb_2$	708	0.0	81	64
I_2Mn	583	0.0	91	73
FeI_2	643	0.0	100	81
Br_2Cd	357	0.0	57	43
GeI_2La_2	569	0.0001	81	49
$Cl_2Er_2H_2$	802	0.0001	91	73
Br_2Mg	643	0.0001	100	81
Br_2PY_2	443	0.0001	57	43
$MnNaTe_2$	244	0.0001	36	25
P_2Sn_2	580	0.0001	81	64
$CdCl_2$	583	0.0002	91	73
$LiMnSe_2$	520	0.0002	73	57
Hg_3N_2	21	0.0002	4	1
$Ba_2F_2I_2$	276	0.0002	39	20
I_2Tm	471	0.0002	81	49
S_2Zn_2	580	0.0002	81	64
$InSe_2$	583	0.0002	91	73
I_2La_2P	180	0.0003	25	16
$Cu_2Na_2Te_2$	476	0.0003	65	36
$F_2Se_2Tm_2$	634	0.0003	73	57
CoH_2O_2	661	0.0003	64	81
CrI_2	643	0.0003	100	81
Ni_2Te_2	656	0.0004	91	73
Te_2Zr	403	0.0004	64	49
Cu_3Se_3	550	0.0004	64	49
$I_2Pr_2S_2$	118	0.0004	16	9
BrNZr	7	0.0004	1	1
I_2Y_2	520	0.0004	73	57
$PbTe_2$	219	0.0004	36	25
I_2Pb	91	0.0004	16	9
SSb_2Te_2	269	0.0005	36	25
$Ge_2Te_2Zr_2$	548	0.0005	65	48
I_2Yb	355	0.0005	61	37
H_2Si_2	656	0.0005	91	73
N_2W	343	0.0005	43	57
BrCdI	219	0.0005	36	25
O_2Zn	388	0.0006	49	64
GeI_2La_2	429	0.0006	61	37
Cl_2La_2	340	0.0006	49	36
$NbSe_2$	7	0.0006	1	1
Nd	209	0.0006	37	61
In_2Se_2	340	0.0006	49	36
Sm	52	0.0007	9	16
GdI_2	148	0.0007	25	16
Ba_2N	403	0.0007	64	49
FeH_2O_2	457	0.0007	43	57
Br_2OV	44	0.0007	6	5
S_2Sn_2	448	0.0007	70	42
Ba_2Cu_2	392	0.0007	61	37

Table showing the first 50 lattice matching combinations with the smallest strain needed to build the supercell.

Hf_2Cl_2 (P-3m1)

Structural and electronic properties

**Formula**	Hf_2Cl_2
**Spacegroup**	P-3m1
**Prototype**	PtTe
**Parent 3D**	Cl_2Hf_2
**Source DB**	MPDS
**DB ID**	S541493
**DF2-C09 Binding energy [meV/ Å^2]**	14.78
**RVV10 Binding energy [meV/ Å^2]**	N/A
**Band gap (PBE) [eV]**	0.0

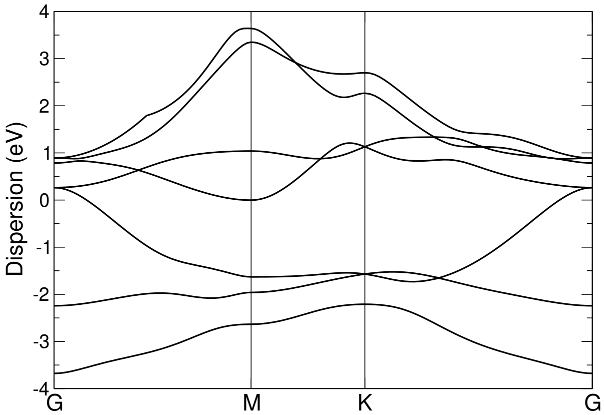

Band structure: Electronic band structure of Hf_2Cl_2 (P-3m1) in a representative window around the Fermi energy and along a high-symmetry path.

Optimized crystal structure

Structural parameters: relaxed cell (top) and atomic positions (bottom) of Hf_2Cl_2 (P-3m1) in Cartesian coordinates.

		x [Å]	y [Å]	z [Å]
$\mathbf{a_1}$		1.69257795	−2.93163100	0.00000000
$\mathbf{a_2}$		1.69257795	2.93163100	0.00000000
$\mathbf{a_3}$		0.00000000	0.00000000	23.57152332
		x [Å]	y [Å]	z [Å]
●	Hf	0.00000000	0.97721033	-1.15091374
●	Cl	1.69257795	0.00000000	-2.90825262
●	Hf	0.00000000	-0.97721033	1.15091374
●	Cl	1.69257795	0.00000000	2.90825262

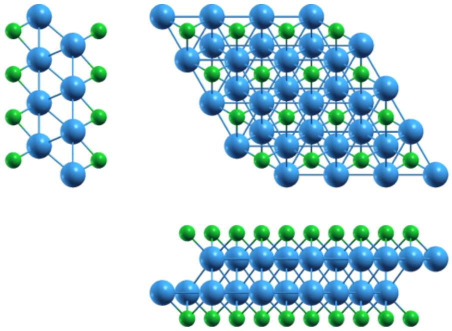

Orthographic projections: views of Hf_2Cl_2 (P-3m1) as seen from the x axis (left), the y axis (bottom) and the z axis (center).

Lattice matching - minimal size

Formula	N° atoms	strain	cell size 1	cell size 2
Tl	5	0.1426	1	1
Tl	5	0.0088	1	1
Sn	5	0.1197	1	1
In	5	0.1222	1	1
HgO	6	0.1539	1	1
AsSb	6	0.4873	1	1
S_2	6	2.9283	1	1
Mg_2	6	0.1297	1	1
CrS_2	7	0.2713	1	1
$CdCl_2$	7	0.5005	1	1
Cl_2Mn	7	0.0033	1	1
$AgTe_2$	7	0.1452	1	1
S_2Ta	7	0.0012	1	1
Br_2Zn	7	0.4612	1	1
$InSe_2$	7	0.5016	1	1
FeO_2	7	1.449	1	1
$GeTe_2$	7	0.4979	1	1
$SiTe_2$	7	0.4716	1	1
I_2Mn	7	0.5009	1	1
PbS_2	7	0.4806	1	1
LiO_2	7	0.3172	1	1
FeI_2	7	0.495	1	1
I_2Ni	7	0.4984	1	1
S_2Ti	7	0.0038	1	1
Mg_3	7	0.1376	1	1
Te_2Ti	7	0.462	1	1
NbS_2	7	0.0016	1	1
CrI_2	7	0.4938	1	1
$RhTe_2$	7	0.4512	1	1
N_2W	7	0.2616	1	1
Cl_2Co	7	0.0034	1	1
NbS_2	7	0.0059	1	1
CNRb	7	3.8549	1	1
Br_2V	7	0.0092	1	1
ClNZr	7	0.007	1	1
Cl_2Fe	7	0.0027	1	1
S_2Ta	7	0.0067	1	1
Se_2V	7	0.008	1	1
$NiTe_2$	7	0.4701	1	1
Cl_2Cu	7	0.1117	1	1
I_2V	7	0.475	1	1
Se_2Zr	7	0.4726	1	1
CdO_2	7	0.0033	1	1
O_2Zn	7	0.2665	1	1
Cl_2Zr	7	0.0031	1	1
$FeSe_2$	7	0.1164	1	1
Br_2Mg	7	0.4946	1	1
I_2Ti	7	2.84	1	1
F_2Na	7	0.4661	1	1
$HfSe_2$	7	0.462	1	1

Table showing the first 50 lattice matching combinations with the smallest number of atoms in the supercell.

Lattice matching - minimal strain

Formula	N° atoms	strain	cell size 1	cell size 2
CoO_2	208	0.0	25	36
Br_2Zn	643	0.0	100	81
$Cl_2Ho_2O_2$	708	0.0	81	64
CCl_2Gd_2	805	0.0001	100	81
DyI_2	91	0.0001	16	9
Bi_2Se_2Te	180	0.0002	25	16
FeI_2	403	0.0002	64	49
I_2La_2P	429	0.0002	61	37
Sb_2SeTe_2	180	0.0002	25	16
Nd	89	0.0002	16	25
In_2Te_3	180	0.0003	25	16
H_2Li_2Pt	725	0.0003	80	81
In_2S_3	443	0.0003	57	43
F_2Na	583	0.0003	91	73
Br_2Mg	403	0.0003	64	49
Te_2Ti	643	0.0003	100	81
$HfSe_2$	643	0.0003	100	81
$Fe_2Li_2P_2$	548	0.0003	65	48
Br_3Cs	20	0.0004	4	1
$Cl_2O_2Yb_2$	802	0.0004	91	73
I_2V	516	0.0004	81	64
$IrTe_2$	357	0.0004	57	43
$Al_2Cl_2O_2$	684	0.0004	75	64
In_2Se_2	244	0.0004	36	25
Bi_2Mn_2	124	0.0004	17	14
GdI_2	471	0.0005	81	49
GeTe	314	0.0005	57	43
$Cl_2O_2Tm_2$	802	0.0005	91	73
NiO_2	208	0.0005	25	36
CrI_2	403	0.0006	64	49
$Dy_2I_2S_2$	118	0.0006	16	9
$I_2S_2Tb_2$	118	0.0006	16	9
CNRb	477	0.0006	90	39
NS_2Ta	580	0.0006	64	81
Ga_2Se_2	400	0.0006	57	43
GeI_2Y_2	180	0.0006	25	16
CaClHO	656	0.0006	91	73
CBr_2Y_2	805	0.0007	100	81
$Cl_2O_2Y_2$	708	0.0007	81	64
I_4Sr_2	542	0.0007	98	25
FeH_2O_2	747	0.0007	73	91
FeO_2	208	0.0007	25	36
Cu_2Sr_2	392	0.0007	61	37
O_2Zn	624	0.0008	81	100
Cl_2La_2	244	0.0008	36	25
$Br_2Er_2S_2$	732	0.0008	108	50
As_2Li_2Nd	180	0.0009	25	16
$Br_2Lu_2S_2$	432	0.0009	63	30
F_4Pb	256	0.0009	39	20
$Br_2Lu_2S_2$	432	0.0009	63	30

Table showing the first 50 lattice matching combinations with the smallest strain needed to build the supercell.

Hf_3Te_2 (P4/mmm)

Structural and electronic properties

**Formula**	Hf_3Te_2
**Spacegroup**	P4/mmm
**Prototype**	Hf3Te2
**Parent 3D**	Hf_3Te_2
**Source DB**	ICSD
**DB ID**	75936
**DF2-C09 Binding energy [meV/ Å^2]**	31.69
**RVV10 Binding energy [meV/ Å^2]**	33.05
**Band gap (PBE) [eV]**	N/A

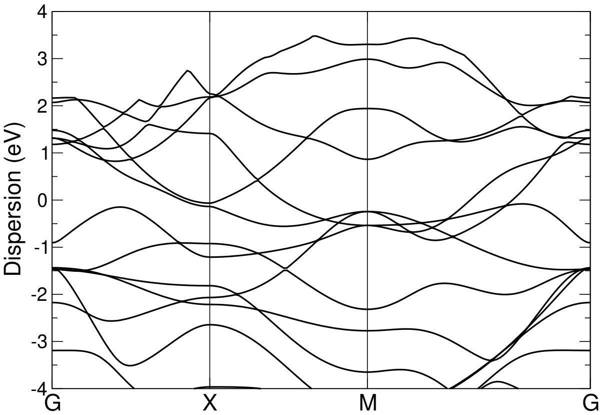

Band structure: Electronic band structure of Hf_3Te_2 (P4/mmm) in a representative window around the Fermi energy and along a high-symmetry path.

Optimized crystal structure

Structural parameters: relaxed cell (top) and atomic positions (bottom) of Hf_3Te_2 (P4/mmm) in Cartesian coordinates.

	x [Å]	y [Å]	z [Å]
$\mathbf{a_1}$	3.70370675	0.00000000	0.00000000
$\mathbf{a_2}$	0.00000000	3.70370675	0.00000000
$\mathbf{a_3}$	0.00000000	0.00000000	26.02973979

	x [Å]	y [Å]	z [Å]
Te	0.00000000	0.00000000	9.96160423
Hf	1.85185338	1.85185338	11.31743795
Hf	1.85185338	1.85185338	14.71230183
Hf	0.00000000	0.00000000	13.01486989
Te	0.00000000	0.00000000	16.06813556

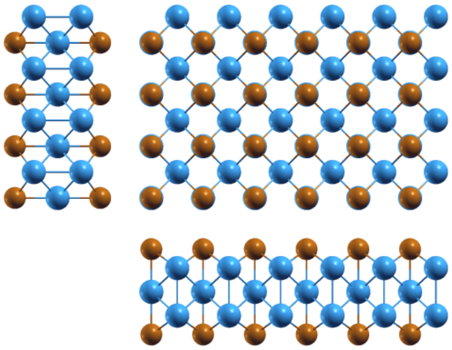

Orthographic projections: views of Hf_3Te_2 (P4/mmm) as seen from the x axis (left), the y axis (bottom) and the z axis (center).

Lattice matching - minimal size

Formula	N° atoms	strain	cell size 1	cell size 2
K	6	1.1606	1	1
Tl	6	0.5612	1	1
InSe	7	0.1716	1	1
HgO	7	0.5967	1	1
Bi_2	7	0.1784	1	1
GeTe	7	0.1326	1	1
AgTl	7	0.3796	1	1
S_2	7	0.1337	1	1
PbTe	7	0.1736	1	1
Sb_2	7	0.1543	1	1
$IrTe_2$	8	0.1332	1	1
CrS_2	8	0.1097	1	1
I_2Mg	8	0.1602	1	1
$CdCl_2$	8	0.1318	1	1
CdI_2	8	0.1757	1	1
Br_2Ca	8	0.1771	1	1
CaI_2	8	0.773	1	1
$InSe_2$	8	0.132	1	1
$GeTe_2$	8	0.131	1	1
$HfTe_2$	8	0.1399	1	1
I_2Mn	8	0.1319	1	1
Br_2La	8	0.1605	1	1
Br_2Cu	8	0.1203	1	1
NSr_2	8	0.1293	1	1
BiClTe	8	0.1761	1	1
$AuTe_2$	8	0.1468	1	1
BrCdI	8	0.1635	1	1
LiO_2	8	0.325	1	1
Cl_2Zn	8	0.0011	1	1
$PdTe_2$	8	0.1445	1	1
FeI_2	8	0.1303	1	1
I_2Ni	8	0.1312	1	1
CrI_2	8	0.1299	1	1
I_2Zn	8	0.1511	1	1
BaF_2	8	0.1672	1	1
BiBrTe	8	0.1829	1	1
Bi_2Pd	8	0.2159	1	1
GeI_2	8	0.1582	1	1
Ba_2Hg	8	0.3992	1	1
N_2W	8	0.1113	1	1
$CrTe_2$	8	0.1085	1	1
Ba_2N	8	0.1412	1	1
AsKSn	8	0.1656	1	1
Te_2Zr	8	0.1403	1	1
$PbTe_2$	8	0.1625	1	1
Cl_2Cu	8	0.1072	1	1
I_2Tm	8	0.7685	1	1
$SnTe_2$	8	0.1559	1	1
GeI_2	8	0.1739	1	1
STl_2	8	0.1683	1	1

Table showing the first 50 lattice matching combinations with the smallest number of atoms in the supercell.

Lattice matching - minimal strain

Formula	N° atoms	strain	cell size 1	cell size 2
$I_2La_2O_2$	800	0.0001	82	65
$Br_2F_2Sr_2$	997	0.0001	101	82
$Cu_2Rb_2Te_2$	221	0.0002	25	16
K	281	0.0004	49	36
$Br_2F_2Pb_2$	997	0.0004	101	82
Cu_2F_4	863	0.0004	109	53
HgO	380	0.0004	50	65
Bi_2In_2	189	0.0005	25	16
$Ca_2Cl_2H_2$	11	0.0006	1	1
$I_2La_2O_2$	789	0.0007	81	64
$Br_2F_2Sr_2$	986	0.0007	100	81
$As_2Mg_2Na_2$	461	0.0007	49	36
HgO	373	0.0007	49	64
Bi_2Te_2	914	0.0008	118	81
$Ag_2K_2Te_2$	699	0.0009	81	49
$Er_2I_2S_2$	786	0.0009	90	56
$Br_2F_2Pb_2$	986	0.0009	100	81
$Gd_2I_2S_2$	871	0.001	101	61
F_4Sn	725	0.001	81	64
O_2Pt	773	0.0011	79	126
Cl_2Zn	8	0.0011	1	1
F_4Nb	905	0.0011	100	81
S_2Ti	435	0.0011	48	65
$Br_2Ho_2S_2$	943	0.0011	107	68
$AgNO_2$	300	0.0012	36	30
Se_2Sn_2	778	0.0012	98	72
Cl_4KTl	896	0.0013	130	41
$AgTe_2$	327	0.0013	36	49
$AgClO_2$	770	0.0014	86	85
AlH_4Na	920	0.0014	106	65
Cu_2F_4	794	0.0014	100	49
I_2Pr	782	0.0014	103	89
Cl_2Co	435	0.0014	48	65
BiClTe	782	0.0014	103	89
$I_2S_2Yb_2$	851	0.0014	97	61
$Br_2Dy_2S_2$	878	0.0015	100	63
S_2Sn_2	634	0.0015	78	61
Sn	105	0.0015	16	25
$AgClO_2$	725	0.0015	81	80
Br_2CsF	189	0.0015	25	16
CdI_2	782	0.0015	103	89
BiClTe	676	0.0016	89	77
Cl_2Mn	479	0.0016	52	73
I_2Pr	676	0.0016	89	77
CdI_2	676	0.0016	89	77
CdO_2	435	0.0016	48	65
Br_2Ca	782	0.0016	103	89
CdH_2O_2	695	0.0016	62	77
$I_2S_2Tm_2$	786	0.0016	90	56
Se_2Sn	869	0.0016	106	113

Table showing the first 50 lattice matching combinations with the smallest strain needed to build the supercell.

HfNBr (P-3m1)

Structural and electronic properties

**Formula**	HfNBr
**Spacegroup**	P-3m1
**Prototype**	SmSI
**Parent 3D**	$Hf_2N_2Br_2$
**Source DB**	ICSD
**DB ID**	190383
**DF2-C09 Binding energy [meV/ Å^2]**	13.88
**RVV10 Binding energy [meV/ Å^2]**	21.17
**Band gap (PBE) [eV]**	2.04

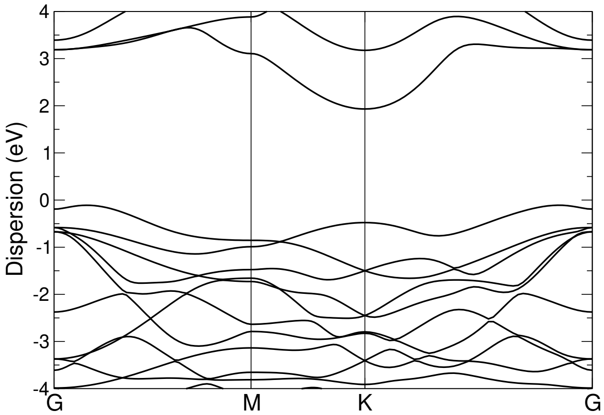

Band structure: Electronic band structure of HfNBr (P-3m1) in a representative window around the Fermi energy and along a high-symmetry path.

Optimized crystal structure

Structural parameters: relaxed cell (top) and atomic positions (bottom) of HfNBr (P-3m1) in Cartesian coordinates.

		x [Å]	y [Å]	z [Å]
$\mathbf{a_1}$		3.61744257	0.00000000	0.00000000
$\mathbf{a_2}$		−1.80872129	3.13279716	0.00000000
$\mathbf{a_3}$		0.00000000	0.00000000	26.62035798
		x [Å]	y [Å]	z [Å]
●	Hf	1.80872129	1.04426572	14.60004149
•	N	1.80872129	1.04426572	12.44473159
●	Br	1.80872129	3.13279716	16.59191156
●	Br	1.80872129	3.13279716	10.02844641
●	Hf	-0.00000000	2.08853144	12.02031648
•	N	-0.00000000	2.08853144	14.17562638

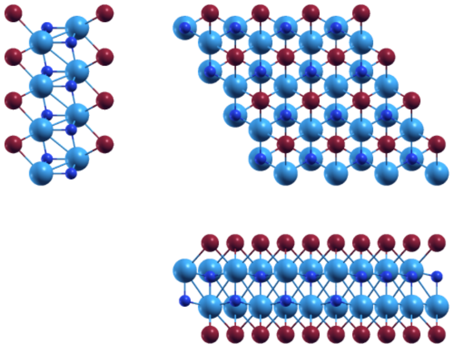

Orthographic projections: views of HfNBr (P-3m1) as seen from the x axis (left), the y axis (bottom) and the z axis (center).

Lattice matching - minimal size

Formula	N° atoms	strain	cell size 1	cell size 2
Tl	7	0.1202	1	1
Na	7	0.0089	1	1
In	7	0.4297	1	1
In	7	0.2483	1	1
InSe	8	3.0338	1	1
HgO	8	0.1277	1	1
As_2	8	0.0011	1	1
LiO	8	0.2626	1	1
P_2	8	0.2736	1	1
Mg_2	8	0.1127	1	1
Sb_2	8	0.4863	1	1
Cl_2Zn	9	0.0022	1	1
I_2Mg	9	0.5013	1	1
S_2V	9	1.6368	1	1
MoS_2	9	0.2535	1	1
$MoTe_2$	9	0.0067	1	1
$AgTe_2$	9	0.1219	1	1
PSn_2	9	0.0079	1	1
HfS_2	9	0.0016	1	1
Te_2V	9	0.0049	1	1
$CuTe_2$	9	0.0012	1	1
S_2Zr	9	0.0071	1	1
Br_2La	9	2.902	1	1
Br_2Co	9	0.0016	1	1
ReS_2	9	1.5488	1	1
Ca_2N	9	0.0006	1	1
Cl_2Ti	9	0.2738	1	1
$AuTe_2$	9	0.4661	1	1
$PdTe_2$	9	0.46	1	1
Mg_3	9	0.1172	1	1
I_2Zn	9	0.4778	1	1
Te_2Zn	9	0.0069	1	1
S_2W	9	0.2536	1	1
Bi_2Pd	9	0.1346	1	1
GeI_2	9	0.4963	1	1
Br_2Mn	9	0.0034	1	1
PtS_2	9	0.0079	1	1
$CoTe_2$	9	0.0019	1	1
CdClO	9	0.0053	1	1
Ba_2N	9	0.4506	1	1
AsKSn	9	2.9633	1	1
Te_2Zr	9	0.4481	1	1
Te_2W	9	0.0066	1	1
S_2Sn	9	0.0074	1	1
Cl_2V	9	0.2591	1	1
STl_2	9	2.995	1	1
OTl_2	9	0.0052	1	1
Br_2Fe	9	0.0015	1	1
Br_2Ni	9	0.0036	1	1
$NbTe_2$	9	0.0069	1	1

Table showing the first 50 lattice matching combinations with the smallest number of atoms in the supercell.

Lattice matching - minimal strain

Formula	N° atoms	strain	cell size 1	cell size 2
I_2Pb	477	0.0	61	37
$I_2Pr_2Si_2$	678	0.0	64	49
H_2NiO_2	707	0.0001	57	73
Br_2Tb_2	924	0.0001	100	81
STl_2	402	0.0001	49	36
Ga_2Te_2	580	0.0001	64	49
$AsCuLi_2$	580	0.0001	64	49
FeO_2	405	0.0001	37	61
Ba_2Ni_3	629	0.0001	64	49
Cu_2Sr_2	316	0.0001	36	25
Cl_2V	627	0.0001	64	81
Br_2Er_2	838	0.0002	91	73
As_2O_3	74	0.0003	9	4
ReS_2	363	0.0003	36	49
$I_2S_2Tb_2$	246	0.0003	25	16
$AuTe_2$	765	0.0003	91	73
Br_2Gd_2Ge	557	0.0003	57	43
GeI_2	531	0.0003	64	49
Cu_2I_2	514	0.0003	57	43
$AgNO_3$	845	0.0004	100	49
In_2Se_2	742	0.0004	81	64
I_2Nd	534	0.0004	65	48
$PdTe_2$	843	0.0005	100	81
$Br_2O_2Sm_2$	678	0.0005	65	48
NaO_4	669	0.0005	79	39
$Cl_2N_2Zr_2$	12	0.0005	1	1
$Cl_2O_2Ti_2$	906	0.0006	81	70
Se_2Yb	531	0.0006	64	49
Br_2HLa	580	0.0006	64	49
Br_2Ho_2	924	0.0006	100	81
CoO_2	405	0.0006	37	61
$F_2Lu_2Se_2$	780	0.0006	73	57
Ca_2N	9	0.0006	1	1
Ir_2P_2	582	0.0006	65	48
Gd_2I_2	514	0.0007	57	43
$I_2O_2Tm_2$	678	0.0007	65	48
I_2Zn	678	0.0007	81	64
$PtTe_2$	765	0.0007	91	73
In	358	0.0007	49	64
DyI_2	198	0.0007	25	16
CoH_2O_2	461	0.0008	36	49
La_2S_2	414	0.0008	49	30
Sb_2Te_2	682	0.0008	81	49
N_2W	258	0.0008	25	36
I_2La_2P	341	0.0008	36	25
Br_2Gd_2	838	0.0008	91	73
CaH_2O_2	11	0.0008	1	1
$BiTe_2$	531	0.0008	64	49
$I_2Pr_2S_2$	588	0.0009	61	37
$PbTe_2$	471	0.0009	57	43

Table showing the first 50 lattice matching combinations with the smallest strain needed to build the supercell.

HfNBr (Pmmn)

Structural and electronic properties

**Formula**	HfNBr
**Spacegroup**	Pmmn
**Prototype**	FeOCl
**Parent 3D**	$Hf_2N_2Br_2$
**Source DB**	COD
**DB ID**	1532008
**DF2-C09 Binding energy [meV/ Å^2]**	10.67
**RVV10 Binding energy [meV/ Å^2]**	18.17
**Band gap (PBE) [eV]**	2.17

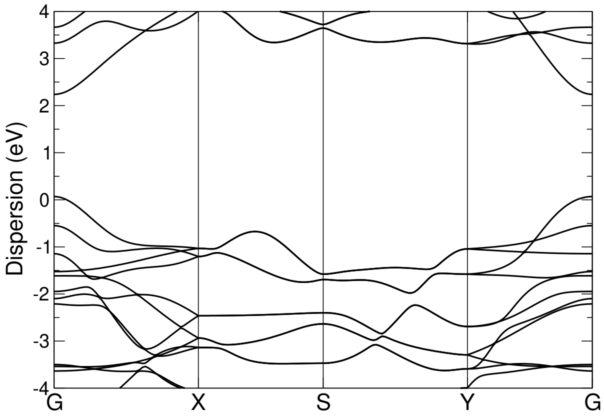

Band structure: Electronic band structure of HfNBr (Pmmn) in a representative window around the Fermi energy and along a high-symmetry path.

Optimized crystal structure

Structural parameters: relaxed cell (top) and atomic positions (bottom) of HfNBr (Pmmn) in Cartesian coordinates.

	x [Å]	y [Å]	z [Å]
$\mathbf{a_1}$	3.56841909	0.00000000	0.00000000
$\mathbf{a_2}$	0.00000000	4.12089793	0.00000000
$\mathbf{a_3}$	0.00000000	0.00000000	25.92259759

		x [Å]	y [Å]	z [Å]
●	Hf	0.00000000	2.06044896	12.10335080
●	Br	1.78420954	2.06044896	10.01285281
●	N	0.00000000	0.00000000	12.65618029
●	Hf	1.78420954	0.00000000	13.81924669
●	Br	0.00000000	0.00000000	15.90975656
●	N	1.78420954	2.06044896	13.26642336

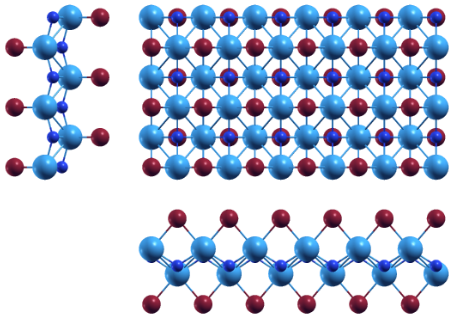

Orthographic projections: views of HfNBr (Pmmn) as seen from the x axis (left), the y axis (bottom) and the z axis (center).

Lattice matching - minimal size

Formula	N° atoms	strain	cell size 1	cell size 2
K	7	0.203	1	1
InSe	8	0.57	1	1
HgO	8	0.6259	1	1
AgTl	8	0.0371	1	1
Tl	8	1.2507	1	2
PbTe	8	0.5753	1	1
CaCl	8	0.0396	1	1
Ca_2Si	9	0.2168	1	1
I_2Yb	9	0.644	1	1
Cl_2Zn	9	0.2957	1	1
BiBrTe	9	0.5983	1	1
CNRb	9	1.1599	1	1
Cl_2Cu	9	0.4215	1	1
GeI_2	9	0.576	1	1
$MnSe_2$	9	0.0396	1	1
CNNa	9	0.0517	1	1
F_2Ni	9	0.2892	1	1
I_2La	9	0.2896	1	1
Ba_2Cd	9	0.3424	1	1
Cu_2I_2	10	0.0526	1	1
Cu_2Sr_2	10	0.6015	1	1
Cl_2OOs	10	0.0315	1	1
$LiMnTe_2$	10	0.5769	1	1
Ag_2Br_2	10	0.2904	1	1
$AgCuTe_2$	10	0.2016	1	1
$AsLi_3$	10	0.5709	1	1
S_2Sn_2	10	0.3206	1	1
Cl_2OV	10	0.2553	1	1
Fe_2Se_2	10	0.2883	1	1
Cl_2ORu	10	0.0338	1	1
As_2Co_2	10	0.2805	1	1
N_3Na	10	1.1209	1	1
As_2Ir_2	10	0.2944	1	1
Cu_2Te_2	10	0.3	1	1
O_2Pb_2	10	0.0497	1	1
As_4	10	0.4714	1	1
P_4	10	0.4281	1	1
Br_2OV	10	0.2676	1	1
Fe_2S_2	10	0.0406	1	1
Co_2S_2	10	0.0393	1	1
O_2Sn_2	10	0.056	1	1
Ge_2Se_2	10	0.3015	1	1
Cu_2Se_2	10	0.2901	1	1
Bi_2O_2	10	0.0508	1	1
$AgClO_2$	10	0.1071	1	1
C_2	10	0.1492	1	2
La_2S_2	10	0.3548	1	1
PbS_2Sn	10	0.0738	1	1
As_2Rh_2	10	0.29	1	1
O_2Sn_2	10	0.0559	1	1

Table showing the first 50 lattice matching combinations with the smallest number of atoms in the supercell.

Lattice matching - minimal strain

Formula	N° atoms	strain	cell size 1	cell size 2
NbS_2	549	0.0001	52	79
Pt_2Te_2	516	0.0001	50	54
I_2V	609	0.0002	64	75
CdH_2O_2	38	0.0002	3	4
Br_2Gd_2Ge	534	0.0002	49	48
Cu_2I_2	486	0.0002	49	48
$Cl_2Ho_2O_2$	834	0.0002	64	75
$Cl_2H_2Zr_2$	786	0.0002	52	79
I_2Pr	474	0.0002	54	50
$Br_2Ho_2S_2$	726	0.0003	72	49
$HNiO_2$	342	0.0003	25	48
Te_2W	30	0.0003	3	4
BiClTe	474	0.0003	54	50
Br_2Er_2	742	0.0003	73	76
NaPSn	462	0.0003	50	54
$AuTe_2$	666	0.0003	73	76
I_2Zn	435	0.0003	48	49
$Er_2F_2Se_2$	624	0.0004	50	54
CS_3Tl_2	216	0.0004	28	8
$MoTe_2$	30	0.0004	3	4
CNb_2S_2	862	0.0005	62	98
$Ga_2I_2Y_2$	894	0.0005	76	73
Gd_2I_2	486	0.0005	49	48
$Al_2Cl_2O_2$	870	0.0005	64	81
Gd_2GeI_2	574	0.0005	54	50
H_2MgO_2	231	0.0006	16	27
Br_2Ca	474	0.0006	54	50
Te_2Zn	30	0.0006	3	4
$HfTe_2$	462	0.0006	50	54
CdI_2	474	0.0006	54	50
In_2Se_3	570	0.0006	50	54
$FeSe_2$	594	0.0007	53	92
CNNa	69	0.0007	8	7
$Er_2I_2S_2$	630	0.0007	63	42
$PtTe_2$	666	0.0007	73	76
$AgClO_2$	850	0.0007	83	88
BaF_2	675	0.0007	76	73
Bi_2Se_3	534	0.0007	49	48
AsKSn	675	0.0007	76	73
Br_2La	438	0.0008	49	48
ClH_3O	39	0.0008	4	3
Br_2Gd_2	742	0.0008	73	76
$GeNi_3Te_2$	624	0.0009	50	54
$HfLiS_2$	34	0.0009	3	4
Ho_2S_2	396	0.0009	40	39
$Cl_2O_2Y_2$	834	0.0009	64	75
As_2Sn_2	742	0.0009	73	76
$AgBrO_2$	824	0.0009	82	83
$C_2Br_2Tb_2$	198	0.0009	16	17
S_2Ta	549	0.0009	52	79

Table showing the first 50 lattice matching combinations with the smallest strain needed to build the supercell.

HfNCl (P-3m1)

Structural and electronic properties

**Formula**	HfNCl
**Spacegroup**	P-3m1
**Prototype**	SmSI
**Parent 3D**	$Hf_2N_2Cl_2$
**Source DB**	ICSD
**DB ID**	261539
**DF2-C09 Binding energy [meV/ Å^2]**	12.76
**RVV10 Binding energy [meV/ Å^2]**	20.13
**Band gap (PBE) [eV]**	2.39

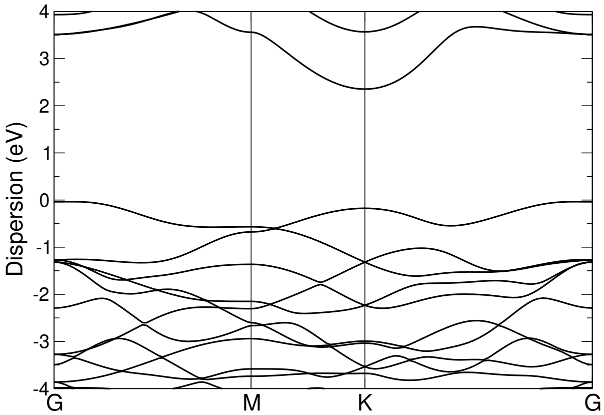

Band structure: Electronic band structure of HfNCl (P-3m1) in a representative window around the Fermi energy and along a high-symmetry path.

Optimized crystal structure

Structural parameters: relaxed cell (top) and atomic positions (bottom) of HfNCl (P-3m1) in Cartesian coordinates.

		x [Å]	y [Å]	z [Å]
$\mathbf{a_1}$		3.57775728	0.00000000	0.00000000
$\mathbf{a_2}$		−1.78887864	3.09842869	0.00000000
$\mathbf{a_3}$		0.00000000	0.00000000	26.29365290
		x [Å]	y [Å]	z [Å]
●	Hf	0.00000000	2.06561913	14.44831767
•	N	0.00000000	2.06561913	12.28532692
•	Cl	1.78887864	3.09842869	10.02649087
•	Cl	1.78887864	3.09842869	16.26716203
●	Hf	1.78887864	1.03280956	11.84533523
•	N	1.78887864	1.03280956	14.00832597

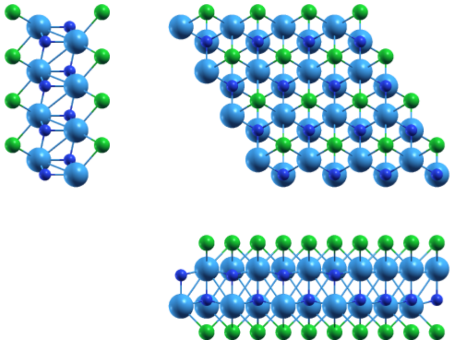

Orthographic projections: views of HfNCl (P-3m1) as seen from the x axis (left), the y axis (bottom) and the z axis (center).

Lattice matching - minimal size

Formula	N° atoms	strain	cell size 1	cell size 2
K	7	0.3188	1	1
Tl	7	0.1232	1	1
Sn	7	0.4315	1	1
Na	7	0.0039	1	1
In	7	0.2552	1	1
HgO	8	0.1313	1	1
As_2	8	0.0041	1	1
LiO	8	0.2701	1	1
Mg_2	8	0.1148	1	1
Sb_2	8	0.5	1	1
CrS_2	9	1.5432	1	1
Cl_2Zn	9	0.003	1	1
I_2Mg	9	2.9658	1	1
S_2V	9	0.2596	1	1
MoS_2	9	0.2606	1	1
$MoTe_2$	9	0.0016	1	1
$AgTe_2$	9	0.1251	1	1
HfS_2	9	0.0069	1	1
$HfTe_2$	9	0.4597	1	1
Te_2V	9	0.0003	1	1
$CuTe_2$	9	0.0065	1	1
Br_2La	9	2.9703	1	1
Br_2Co	9	0.0037	1	1
ReS_2	9	1.5867	1	1
Ca_2N	9	0.0046	1	1
$AuTe_2$	9	0.4794	1	1
$PdTe_2$	9	0.4731	1	1
Mg_3	9	0.1199	1	1
I_2Zn	9	2.8496	1	1
Te_2Zn	9	0.0018	1	1
S_2W	9	0.2607	1	1
Bi_2Pd	9	0.1388	1	1
Br_2Mn	9	0.0018	1	1
$CrTe_2$	9	0.0091	1	1
PtS_2	9	0.0028	1	1
$CoTe_2$	9	0.0072	1	1
CdClO	9	0.0002	1	1
Ba_2N	9	0.4635	1	1
Se_2Ti	9	0.0054	1	1
AsKSn	9	3.0329	1	1
Te_2Zr	9	0.4609	1	1
Te_2W	9	0.0015	1	1
Cl_2Cu	9	0.5921	1	1
Cl_2V	9	0.2664	1	1
OTl_2	9	0.0	1	1
Br_2Fe	9	0.0037	1	1
Br_2Ni	9	0.0089	1	1
MoS_2	9	0.2609	1	1
Cl_2Mg	9	0.009	1	1
$CrSe_2$	9	0.2647	1	1

Table showing the first 50 lattice matching combinations with the smallest number of atoms in the supercell.

Lattice matching - minimal strain

Formula	N° atoms	strain	cell size 1	cell size 2
OTl_2	9	0.0	1	1
$Ga_2Gd_2I_2$	366	0.0	36	25
$F_2Lu_2Se_2$	678	0.0	64	49
Br_2Ho_2	742	0.0001	81	64
BH_4Li	600	0.0001	57	43
Te_2Zr	843	0.0001	100	81
In	415	0.0002	57	73
$PbTe_2$	402	0.0002	49	36
CdClO	9	0.0002	1	1
$SnTe_2$	471	0.0002	57	43
GeI_2La_2	230	0.0002	25	16
F_2Zn	534	0.0002	65	48
$Br_2Gd_2O_2$	678	0.0002	65	48
S_2V	627	0.0002	64	81
$Ga_2I_2Tb_2$	366	0.0003	36	25
$F_2Ho_2Se_2$	984	0.0003	91	73
N_2Re	171	0.0003	16	25
$CrSe_2$	711	0.0003	73	91
Te_2V	9	0.0003	1	1
As_2Sn_2	666	0.0003	73	57
Bi_2S_3	806	0.0003	81	64
Br_2Gd_2	666	0.0004	73	57
ClH_3O	141	0.0004	16	9
FHOZn	802	0.0004	73	91
Au_2I_2	864	0.0004	104	60
$MnNaTe_2$	438	0.0004	49	36
$CrSe_2$	786	0.0005	81	100
Bi_2	266	0.0005	36	25
I_2Tm	198	0.0005	25	16
CrS_2	363	0.0005	36	49
$Br_2Ca_2F_2$	678	0.0005	65	48
Br_2Tb_2	742	0.0005	81	64
$C_2Br_2Tb_2$	216	0.0006	20	16
$HfTe_2$	843	0.0006	100	81
Br_2Ca	291	0.0006	36	25
H_2MgO_2	789	0.0006	64	81
$CeLi_2P_2$	557	0.0007	57	43
$C_2Br_2Gd_2$	216	0.0007	20	16
SSb_2Te_2	474	0.0008	49	36
ReS_2	429	0.0008	43	57
Cl_2La_2	666	0.0008	73	57
Cl_2V	786	0.0008	81	100
BrCdI	402	0.0008	49	36
$I_2Lu_2O_2$	678	0.0008	65	48
$I_2Nd_2S_2$	780	0.0008	81	49
BN	392	0.0008	39	79
$Br_2F_2Tm_2$	678	0.0009	65	48
I_2Yb	198	0.0009	25	16
$PtTe_2$	678	0.0009	81	64
$Br_2F_2Yb_2$	678	0.0009	65	48

Table showing the first 50 lattice matching combinations with the smallest strain needed to build the supercell.

HfNI (P-3m1)

Structural and electronic properties

**Formula**	HfNI
**Spacegroup**	P-3m1
**Prototype**	SmSI
**Parent 3D**	$Hf_2N_2I_2$
**Source DB**	ICSD
**DB ID**	190384
**DF2-C09 Binding energy [meV/ Å^2]**	16.4
**RVV10 Binding energy [meV/ Å^2]**	23.23
**Band gap (PBE) [eV]**	0.99

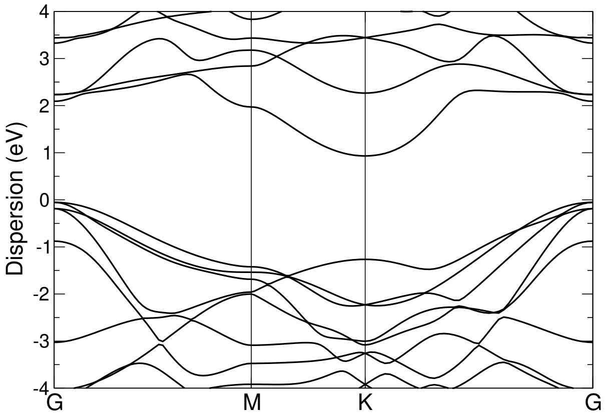

Band structure: Electronic band structure of HfNI (P-3m1) in a representative window around the Fermi energy and along a high-symmetry path.

Optimized crystal structure

Structural parameters: relaxed cell (top) and atomic positions (bottom) of HfNI (P-3m1) in Cartesian coordinates.

		x [Å]	y [Å]	z [Å]
$\mathbf{a_1}$		3.69808501	0.00000000	0.00000000
$\mathbf{a_2}$		−1.84904250	3.20263556	0.00000000
$\mathbf{a_3}$		0.00000000	0.00000000	27.01415029
		x [Å]	y [Å]	z [Å]
●	Hf	1.84904250	1.06754519	14.77138372
•	N	1.84904250	1.06754519	12.63661924
●	I	0.00000000	0.00000000	16.98535995
●	I	0.00000000	0.00000000	10.02879034
●	Hf	0.00000000	2.13509037	12.24276657
•	N	0.00000000	2.13509037	14.37753105

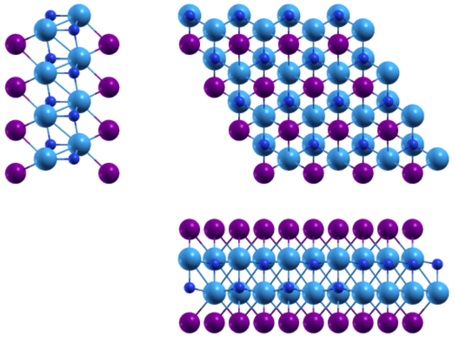

Orthographic projections: views of HfNI (P-3m1) as seen from the x axis (left), the y axis (bottom) and the z axis (center).

Lattice matching - minimal size

Formula	N° atoms	strain	cell size 1	cell size 2
Tl	7	0.1151	1	1
Tl	7	0.2661	1	1
In	7	1.5368	1	1
InSe	8	0.501	1	1
HgO	8	0.1212	1	1
Bi_2	8	2.972	1	1
LiO	8	0.2485	1	1
P_2	8	0.2587	1	1
PbTe	8	2.919	1	1
Mg_2	8	0.4325	1	1
Sb_2	8	0.4597	1	1
Sm	8	0.222	1	2
I_2Mg	9	0.474	1	1
S_2V	9	1.5598	1	1
MoS_2	9	1.5651	1	1
Cl_2Mn	9	0.2745	1	1
$AgTe_2$	9	0.1165	1	1
PSn_2	9	0.0026	1	1
$MoSe_2$	9	0.2651	1	1
Br_2Zn	9	0.0081	1	1
HfS_2	9	0.0086	1	1
$AsSn_2$	9	0.0011	1	1
I_2Pr	9	0.2096	1	1
$CuTe_2$	9	0.009	1	1
S_2Zr	9	0.0034	1	1
Br_2La	9	0.4749	1	1
Br_2Cu	9	0.9897	1	1
Cl_2Ti	9	0.2589	1	1
BrCdI	9	0.4821	1	1
HgI_2	9	0.322	1	1
Mg_3	9	0.1127	1	1
Te_2Ti	9	0.0085	1	1
I_2Zn	9	0.4516	1	1
$RhTe_2$	9	0.004	1	1
S_2W	9	1.5656	1	1
Bi_2Pd	9	0.1271	1	1
GeI_2	9	0.4693	1	1
NbS_2	9	0.2705	1	1
$CoTe_2$	9	0.0083	1	1
S_2Ta	9	0.2693	1	1
Se_2V	9	0.2673	1	1
AsKSn	9	0.487	1	1
$PbTe_2$	9	0.4796	1	1
S_2Sn	9	0.0031	1	1
$SnTe_2$	9	0.4636	1	1
Cl_2V	9	1.5951	1	1
GeI_2	9	2.9221	1	1
STl_2	9	0.4933	1	1
$PtSe_2$	9	0.0018	1	1
GeS_2	9	0.1491	1	1

Table showing the first 50 lattice matching combinations with the smallest number of atoms in the supercell.

Lattice matching - minimal strain

Formula	N° atoms	strain	cell size 1	cell size 2
CdClHO	10	0.0	1	1
Br_2HLa	838	0.0	91	73
C_2	42	0.0001	4	9
Gd_2I_2	742	0.0001	81	64
Sb_2Te_3	474	0.0001	49	36
Se_2V	786	0.0002	81	100
Cu_2I_2	742	0.0002	81	64
Br_2Gd_2Ge	806	0.0002	81	64
CS_2Ta_2	789	0.0002	64	81
$I_2O_2Sm_2$	678	0.0002	65	48
Cl_2Ti	627	0.0003	64	81
In_2Te_3	629	0.0003	64	49
$Ge_2I_2La_2$	366	0.0003	36	25
PbTe	428	0.0004	57	43
Sb_2SeTe_2	629	0.0004	64	49
Bi_2Se_3	806	0.0004	81	64
Cl_2V	429	0.0004	43	57
$CeLi_2P_2$	911	0.0004	91	73
As_2CeLi_2	557	0.0004	57	43
P_2	546	0.0004	64	81
Ba_2Pt	198	0.0004	25	16
BrCdI	609	0.0004	73	57
Br_2La	678	0.0004	81	64
Cu_4Te_2	510	0.0004	49	36
Bi_2STe_2	557	0.0005	57	43
$AsCuLi_2$	838	0.0005	91	73
SSb_2Te_2	723	0.0005	73	57
$MoSe_2$	711	0.0005	73	91
Se_2Sn_2	810	0.0005	95	60
$Eu_2H_2I_2$	678	0.0005	65	48
$TaTe_2$	9	0.0006	1	1
Sb_2	762	0.0006	100	81
GeI_2	471	0.0006	57	43
LiO	422	0.0006	49	64
$I_2Pr_2Si_2$	984	0.0006	91	73
Se_2W	711	0.0007	73	91
Ag_2	182	0.0007	25	16
FHOZn	486	0.0007	43	57
Bi_2Se_2Te	629	0.0007	64	49
Ga_2Te_2	838	0.0007	91	73
HN_3OZn	870	0.0007	64	81
Ba_2Ni_3	911	0.0007	91	73
$Al_2Cl_2O_2$	582	0.0008	48	49
I_2Mg	678	0.0008	81	64
$Eu_2I_2O_2$	678	0.0008	65	48
STl_2	531	0.0008	64	49
Br_2La_2	924	0.0008	100	81
$MnNaTe_2$	666	0.0008	73	57
$CrSe_2$	429	0.0008	43	57
$Cl_2Hg_2N_2$	438	0.0009	52	21

Table showing the first 50 lattice matching combinations with the smallest strain needed to build the supercell.

HfS_2 (P-3m1)

Structural and electronic properties

**Formula**	HfS_2
**Spacegroup**	P-3m1
**Prototype**	CdI2
**Parent 3D**	HfS_2
**Source DB**	ICSD
**DB ID**	638847
**DF2-C09 Binding energy [meV/ Å^2]**	18.15
**RVV10 Binding energy [meV/ Å^2]**	23.21
**Band gap (PBE) [eV]**	1.29

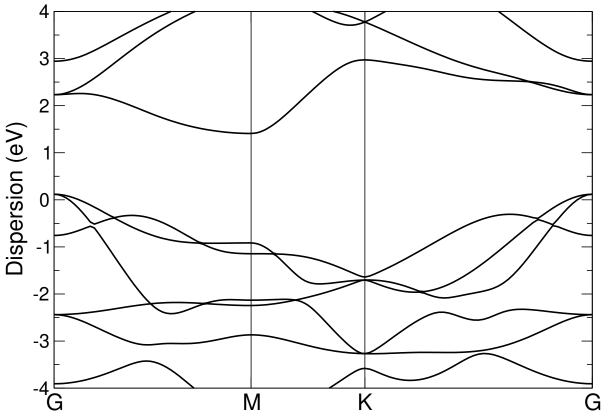

Band structure: Electronic band structure of HfS_2 (P-3m1) in a representative window around the Fermi energy and along a high-symmetry path.

Optimized crystal structure

Structural parameters: relaxed cell (top) and atomic positions (bottom) of HfS_2 (P-3m1) in Cartesian coordinates.

	x [Å]	y [Å]	z [Å]
$\mathbf{a_1}$	3.62964944	0.00000000	0.00000000
$\mathbf{a_2}$	−1.81482472	3.14336862	0.00000000
$\mathbf{a_3}$	0.00000000	0.00000000	22.90293634

		x [Å]	y [Å]	z [Å]
●	S	-0.00000000	2.09557908	9.99752915
●	Hf	0.00000000	0.00000000	11.45146817
●	S	1.81482472	1.04778954	12.90540720

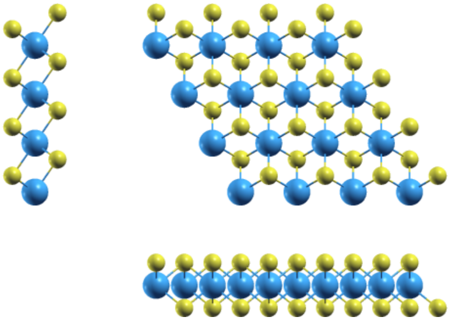

Orthographic projections: views of HfS_2 (P-3m1) as seen from the x axis (left), the y axis (bottom) and the z axis (center).

Lattice matching - minimal size

Formula	N° atoms	strain	cell size 1	cell size 2
Tl	4	0.1194	1	1
In	4	0.4261	1	1
In	4	0.2462	1	1
InSe	5	3.0124	1	1
HgO	5	0.1266	1	1
As_2	5	0.0027	1	1
LiO	5	0.2604	1	1
P_2	5	0.2713	1	1
PbTe	5	3.0362	1	1
Mg_2	5	0.1121	1	1
Sb_2	5	0.4821	1	1
Cl_2Zn	6	0.0037	1	1
I_2Mg	6	0.497	1	1
S_2V	6	0.2504	1	1
MoS_2	6	1.6304	1	1
$MoTe_2$	6	0.0083	1	1
$AgTe_2$	6	0.121	1	1
PSn_2	6	0.0062	1	1
Te_2V	6	0.0064	1	1
$CuTe_2$	6	0.0004	1	1
S_2Zr	6	0.0055	1	1
Br_2La	6	0.4979	1	1
Br_2Cu	6	1.0329	1	1
NiO_2	6	4.8793	1	1
Ca_2Si	6	13.6316	1	1
Br_2Co	6	0.0031	1	1
ReS_2	6	1.5374	1	1
Ca_2N	6	0.0022	1	1
Cl_2Ti	6	0.2715	1	1
$AuTe_2$	6	0.4621	1	1
$PdTe_2$	6	0.456	1	1
Mg_3	6	0.1164	1	1
I_2Zn	6	0.4737	1	1
Te_2Zn	6	0.0084	1	1
S_2W	6	1.6309	1	1
Bi_2Pd	6	0.1334	1	1
GeI_2	6	2.8533	1	1
N_2W	6	1.4485	1	1
Br_2Mn	6	0.0049	1	1
$CoTe_2$	6	0.0003	1	1
CdClO	6	0.0069	1	1
Te_2W	6	0.0081	1	1
S_2Sn	6	0.0057	1	1
$SnTe_2$	6	0.4862	1	1
Cl_2V	6	0.2569	1	1
GeI_2	6	3.0393	1	1
STl_2	6	2.9739	1	1
OTl_2	6	0.0067	1	1
Br_2Fe	6	0.0031	1	1
$TaTe_2$	6	0.0095	1	1

Table showing the first 50 lattice matching combinations with the smallest number of atoms in the supercell.

Lattice matching - minimal strain

Formula	N° atoms	strain	cell size 1	cell size 2
H_2MgO_2	467	0.0	49	64
$PtTe_2$	543	0.0	100	81
FHOZn	463	0.0	57	73
Sb_2Se_2Te	437	0.0001	64	49
$Gd_2I_2S_2$	171	0.0001	25	16
GdI_2	183	0.0001	36	25
$MnNaTe_2$	343	0.0001	57	43
$CrSe_2$	390	0.0001	57	73
Li_2P_2Pr	504	0.0001	73	57
Gd	253	0.0002	52	97
Bi_2Se_2Te	327	0.0002	49	36
NiO_2	390	0.0002	49	81
Sb_2Te_2	331	0.0002	61	37
SSb_2Te_2	386	0.0002	57	43
AlH_4Na	237	0.0002	39	20
$Br_2O_2V_2$	558	0.0002	66	60
$AgNO_3$	432	0.0002	79	39
$Ga_2Ge_2Te_2$	561	0.0003	73	57
BrCdI	300	0.0003	57	43
$Bi_2Br_2O_2$	483	0.0003	65	48
$Er_2I_2O_2$	483	0.0003	65	48
GeI_2Y_2	327	0.0003	49	36
$Br_2Eu_2F_2$	483	0.0003	65	48
Br_2Ca_3Si	171	0.0003	25	16
$CoTe_2$	6	0.0003	1	1
Cl_2La_2	565	0.0003	91	73
CoO_2	390	0.0003	49	81
$CuTe_2$	6	0.0004	1	1
O_2Pt	300	0.0004	43	57
$AuTe_2$	543	0.0004	100	81
FeH_2O_2	255	0.0004	25	36
$Ce_2I_2Si_2$	486	0.0004	64	49
Sb_2Se_2Te	437	0.0004	64	49
Sb_2	333	0.0004	73	57
NiO_2	294	0.0004	37	61
Ga_2S_2	7	0.0005	1	1
Ca_2Si	390	0.0005	81	49
$Ce_2I_2S_2$	537	0.0005	81	49
$CrSe_2$	435	0.0005	64	81
As_2Li_2Nd	327	0.0005	49	36
$Al_2Cl_2O_2$	435	0.0005	49	48
Sb_2SeTe_2	327	0.0006	49	36
$F_2Lu_2Se_2$	627	0.0006	81	64
I_2Mg	339	0.0006	64	49
In_2Te_3	327	0.0006	49	36
Br_2La_2	447	0.0007	73	57
Bi_2O_2	678	0.0007	118	81
As_2Sn_2	565	0.0007	91	73
$PbTe_2$	300	0.0007	57	43
$BiTe_2$	339	0.0007	64	49

Table showing the first 50 lattice matching combinations with the smallest strain needed to build the supercell.

$HfSe_2$ (P-3m1)

Structural and electronic properties

**Formula**	$HfSe_2$
**Spacegroup**	P-3m1
**Prototype**	CdI2
**Parent 3D**	$HfSe_2$
**Source DB**	ICSD
**DB ID**	182678
**DF2-C09 Binding energy [meV/ Å^2]**	18.84
**RVV10 Binding energy [meV/ Å^2]**	23.91
**Band gap (PBE) [eV]**	0.64

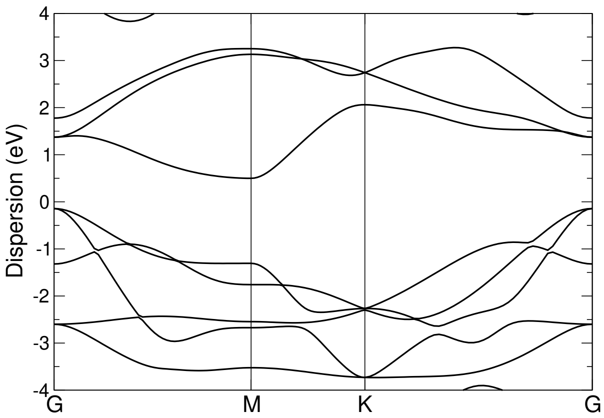

Band structure: Electronic band structure of $HfSe_2$ (P-3m1) in a representative window around the Fermi energy and along a high-symmetry path.

Optimized crystal structure

Structural parameters: relaxed cell (top) and atomic positions (bottom) of $HfSe_2$ (P-3m1) in Cartesian coordinates.

		x [Å]	y [Å]	z [Å]
$\mathbf{a_1}$		3.76395668	0.00000000	0.00000000
$\mathbf{a_2}$		−1.88197834	3.25968211	0.00000000
$\mathbf{a_3}$		0.00000000	0.00000000	23.17133296
		x [Å]	y [Å]	z [Å]
●	Se	1.88197834	1.08656070	13.15919737
●	Hf	0.00000000	0.00000000	11.58566648
●	Se	0.00000000	2.17312140	10.01213559

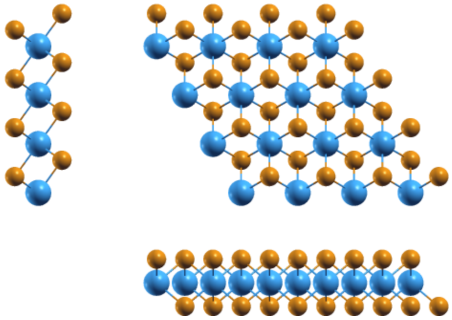

Orthographic projections: views of $HfSe_2$ (P-3m1) as seen from the x axis (left), the y axis (bottom) and the z axis (center).

Lattice matching - minimal size

Formula	N° atoms	strain	cell size 1	cell size 2
Tl	4	0.1118	1	1
Tl	4	0.2545	1	1
InSe	5	0.479	1	1
HgO	5	0.1169	1	1
Bi_2	5	0.4942	1	1
P_2	5	0.2475	1	1
PbTe	5	0.4836	1	1
CaCl	5	0.1528	1	1
I_2Mg	6	0.4531	1	1
Cl_2Mn	6	0.2624	1	1
CdI_2	6	2.8341	1	1
$AgTe_2$	6	0.1129	1	1
$MoSe_2$	6	0.2535	1	1
S_2Ta	6	0.2654	1	1
Br_2Zn	6	0.0003	1	1
Br_2Ca	6	2.8496	1	1
$AsSn_2$	6	0.0071	1	1
$SiTe_2$	6	0.0038	1	1
Br_2La	6	0.454	1	1
PbS_2	6	0.0074	1	1
BiClTe	6	2.8386	1	1
Cl_2Ti	6	0.2477	1	1
BrCdI	6	0.4609	1	1
S_2Ti	6	0.2726	1	1
Te_2Ti	6	0.0	1	1
NbS_2	6	0.2647	1	1
BaF_2	6	0.4692	1	1
$RhTe_2$	6	0.0043	1	1
Bi_2Pd	6	0.1219	1	1
GeI_2	6	0.4486	1	1
Cl_2Co	6	0.2721	1	1
NbS_2	6	0.2587	1	1
Cl_2Fe	6	0.271	1	1
S_2Ta	6	0.2575	1	1
Se_2V	6	0.2557	1	1
AsKSn	6	0.4656	1	1
$PbTe_2$	6	0.4585	1	1
$NiTe_2$	6	0.0032	1	1
I_2V	6	0.0052	1	1
Cl_2V	6	1.5346	1	1
GeI_2	6	0.4842	1	1
Se_2Zr	6	0.0042	1	1
STl_2	6	0.4716	1	1
$PtSe_2$	6	0.0065	1	1
BiITe	6	2.9959	1	1
CdO_2	6	0.2719	1	1
GeS_2	6	0.1416	1	1
$TaTe_2$	6	0.0076	1	1
$MnSe_2$	6	0.1527	1	1
Cl_2Zr	6	0.2716	1	1

Table showing the first 50 lattice matching combinations with the smallest number of atoms in the supercell.

Lattice matching - minimal strain

Formula	N° atoms	strain	cell size 1	cell size 2
BN	30	0.0	4	9
Te_2Ti	6	0.0	1	1
Br_3Cs	343	0.0	81	25
H_2MnO_2	552	0.0001	49	81
BiITe	255	0.0001	49	36
BrCdI	543	0.0001	100	81
Bi_2SeTe_2	437	0.0001	64	49
Bi_2Te_3	327	0.0001	49	36
Se_2V	390	0.0002	57	73
PbTe	333	0.0002	73	57
SSb_2Te_2	705	0.0002	100	81
HN_3OZn	531	0.0002	49	64
$Cl_2H_2Zr_2$	678	0.0002	64	81
Bi_2Se_2Te	563	0.0002	81	64
As_2CeLi_2	504	0.0002	73	57
NiO_2	75	0.0002	9	16
DyI_2	183	0.0002	36	25
$I_2S_2Tb_2$	258	0.0002	36	25
Bi_2O_2	387	0.0003	65	48
GeI_2Y_2	563	0.0003	81	64
NbS_2	492	0.0003	73	91
Br_2Zn	6	0.0003	1	1
CoO_2	75	0.0003	9	16
Cl_2Hf_2	643	0.0003	81	100
BiBrTe	300	0.0004	57	43
GeI_2	390	0.0004	73	57
CCl_2Gd_2	8	0.0004	1	1
NbS_2	435	0.0004	64	81
Bi_2	290	0.0005	64	49
$CrSe_2$	255	0.0005	36	49
$MnNaTe_2$	624	0.0005	100	81
AsKSn	492	0.0005	91	73
As_2Li_2Nd	563	0.0005	81	64
Sb_2SeTe_2	563	0.0005	81	64
C_4Ca_2	831	0.0006	113	82
H_2MnO_2	416	0.0006	37	61
In_2Te_3	563	0.0006	81	64
H_2Li_2Pt	74	0.0006	8	10
Cu_2Sr_2	343	0.0007	57	43
Bi_2STe_2	504	0.0007	73	57
$Br_2O_2Ti_2$	375	0.0007	45	40
$LiMnTe_2$	447	0.0007	73	57
Tl	244	0.0007	57	73
$Ga_2I_2Y_2$	711	0.0007	91	73
$Br_2La_2O_2$	483	0.0007	65	48
$LiOS_2Ti$	597	0.0007	64	81
$Eu_2F_2I_2$	483	0.0007	65	48
$Br_2Cr_2O_2$	435	0.0007	49	48
S_2Ta	492	0.0008	73	91
LiO	206	0.0008	36	49

Table showing the first 50 lattice matching combinations with the smallest strain needed to build the supercell.

HfSiTe (P4/nmm)

Structural and electronic properties

**Formula**	HfSiTe
**Spacegroup**	P4/nmm
**Prototype**	PbClF
**Parent 3D**	$Hf_2Si_2Te_2$
**Source DB**	ICSD
**DB ID**	25737
**DF2-C09 Binding energy [meV/ Å^2]**	35.12
**RVV10 Binding energy [meV/ Å^2]**	32.85
**Band gap (PBE) [eV]**	N/A

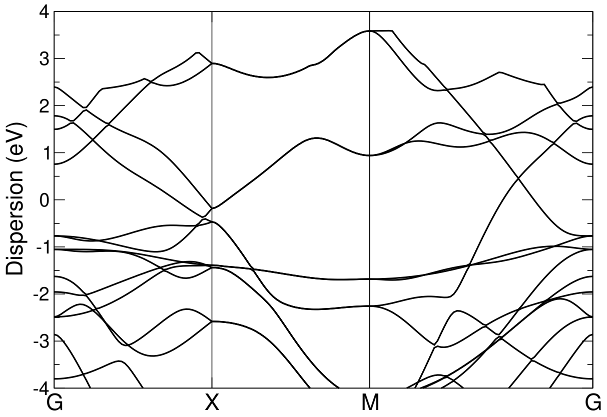

Band structure: Electronic band structure of HfSiTe (P4/nmm) in a representative window around the Fermi energy and along a high-symmetry path.

Optimized crystal structure

Structural parameters: relaxed cell (top) and atomic positions (bottom) of HfSiTe (P4/nmm) in Cartesian coordinates.

	x [Å]	y [Å]	z [Å]
$\mathbf{a_1}$	3.65335566	0.00000000	0.00000000
$\mathbf{a_2}$	0.00000000	3.65335566	0.00000000
$\mathbf{a_3}$	0.00000000	0.00000000	26.79297394

		x [Å]	y [Å]	z [Å]
●	Hf	1.82667783	0.00000000	11.29080159
●	Te	0.00000000	1.82667783	9.87649920
●	Hf	0.00000000	1.82667783	15.50217235
●	Si	1.82667783	1.82667783	13.39648697
●	Si	0.00000000	0.00000000	13.39648697
●	Te	1.82667783	0.00000000	16.91647474

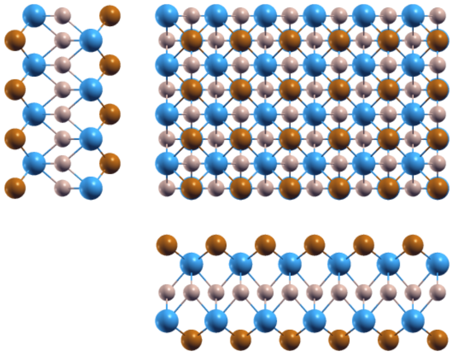

Orthographic projections: views of HfSiTe (P4/nmm) as seen from the x axis (left), the y axis (bottom) and the z axis (center).

Lattice matching - minimal size

Formula	N° atoms	strain	cell size 1	cell size 2
InSe	8	0.1793	1	1
HgO	8	0.2123	1	1
AsSb	8	0.1329	1	1
Bi_2	8	0.1865	1	1
GeTe	8	0.1376	1	1
AgTl	8	0.3935	1	1
S_2	8	0.1389	1	1
PbTe	8	0.1814	1	1
Sb_2	8	0.161	1	1
CaCl	8	0.0068	1	1
$IrTe_2$	9	0.1384	1	1
I_2Mg	9	0.1672	1	1
$CdCl_2$	9	0.1367	1	1
CdI_2	9	0.1836	1	1
$AgTe_2$	9	0.589	1	1
Br_2Ca	9	0.1851	1	1
$InSe_2$	9	0.137	1	1
$GeTe_2$	9	0.136	1	1
$HfTe_2$	9	0.1456	1	1
I_2Mn	9	0.1368	1	1
Br_2La	9	0.1676	1	1
Br_2Cu	9	0.1253	1	1
NSr_2	9	0.1341	1	1
I_2Yb	9	0.7883	1	1
PbS_2	9	0.1309	1	1
BiClTe	9	0.184	1	1
$AuTe_2$	9	0.1529	1	1
BrCdI	9	0.1708	1	1
Cl_2Zn	9	0.0076	1	1
$PdTe_2$	9	0.1506	1	1
FeI_2	9	0.1351	1	1
I_2Ni	9	0.1361	1	1
Mg_3	9	0.5628	1	1
CrI_2	9	0.1347	1	1
I_2Zn	9	0.1576	1	1
BaF_2	9	0.1746	1	1
BiBrTe	9	0.1911	1	1
GeI_2	9	0.1651	1	1
Ba_2Hg	9	1.1143	1	1
N_2W	9	0.1098	1	1
Cl_2Ni	9	0.1086	1	1
Ba_2N	9	0.147	1	1
AsKSn	9	0.173	1	1
Te_2Zr	9	0.146	1	1
$PbTe_2$	9	0.1696	1	1
$AsSe_2$	9	0.1089	1	1
$SnTe_2$	9	0.1627	1	1
I_2V	9	0.1294	1	1
GeI_2	9	0.1817	1	1
STl_2	9	0.1758	1	1

Table showing the first 50 lattice matching combinations with the smallest number of atoms in the supercell.

Lattice matching - minimal strain

Formula	N° atoms	strain	cell size 1	cell size 2
$Cu_2K_2Te_2$	246	0.0001	25	16
$Br_2F_2Sr_2$	870	0.0001	81	64
F_4Sn	629	0.0001	64	49
Cl_4KTl	636	0.0001	81	25
NbS_2	483	0.0002	48	65
$As_2Mg_2Na_2$	876	0.0003	85	61
Mg_6	690	0.0003	50	65
Sn	670	0.0003	89	136
HgO	546	0.0003	64	81
$Br_2F_2Pb_2$	870	0.0003	81	64
S_2Ta	483	0.0005	48	65
O_2Pb_2	924	0.0005	100	81
$I_2La_2O_2$	690	0.0005	65	50
$LiNbS_2$	548	0.0005	48	65
K	571	0.0006	85	61
Co_2Se_2	10	0.0006	1	1
Ba_2Cd	933	0.0007	113	85
Cl_4Mn	731	0.0007	81	49
Au_2Br_2	438	0.0008	49	36
Cr_2O_4	54	0.0008	4	5
Tl	623	0.0008	85	113
Mg_6	678	0.0009	49	64
Li_2Tl_2	908	0.0009	108	65
Ba_2Hg	531	0.0009	64	49
FeO_2	237	0.0009	20	39
$Br_2H_2Sr_2$	882	0.0009	82	65
AlH_4Na	930	0.0009	97	58
Bi_2Se_2	874	0.0009	97	73
CKN	441	0.0011	56	35
Br_2Ca_3Si	678	0.0011	64	49
Mg_4	962	0.0011	85	113
Mg_3	363	0.0011	36	49
Ag_2F_4	816	0.0012	95	41
Cl_2NSc_2	613	0.0012	48	65
$I_2Lu_2Se_2$	414	0.0012	45	24
Cu_2I_2	752	0.0013	82	65
Ge_2Te_2	734	0.0013	83	59
$Fe_2Li_2P_2$	12	0.0013	1	1
ReS_2	852	0.0014	79	126
Cu_4Te_2	876	0.0015	79	67
NS_2Ta	820	0.0015	64	109
Bi_2Mn_2	376	0.0015	36	40
STl_2	885	0.0015	103	89
STl_2	765	0.0016	89	77
$MoSe_2$	531	0.0016	52	73
Br_2Ca_3Si	360	0.0016	40	20
$FeSe_2$	171	0.0016	16	25
$I_2Sb_2Te_2$	768	0.0016	90	38
Se_2Sn_2	372	0.0016	42	30
Cu_2O_2	86	0.0016	9	8

Table showing the first 50 lattice matching combinations with the smallest strain needed to build the supercell.

$HfTe_2$ (P-3m1)

Structural and electronic properties

**Formula**	$HfTe_2$
**Spacegroup**	P-3m1
**Prototype**	CdI2
**Parent 3D**	$HfTe_2$
**Source DB**	ICSD
**DB ID**	603713
**DF2-C09 Binding energy [meV/ Å^2]**	23.42
**RVV10 Binding energy [meV/ Å^2]**	26.37
**Band gap (PBE) [eV]**	N/A

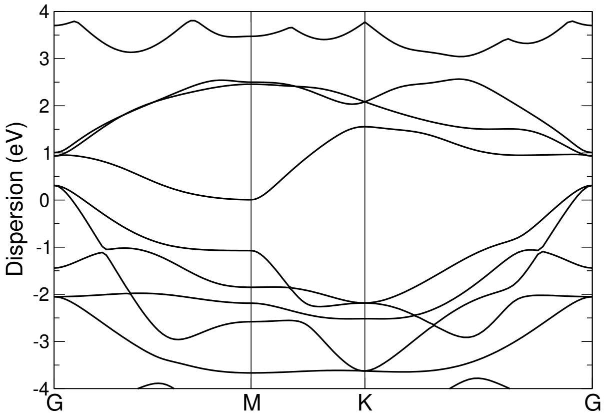

Band structure: Electronic band structure of $HfTe_2$ (P-3m1) in a representative window around the Fermi energy and along a high-symmetry path.

Optimized crystal structure

Structural parameters: relaxed cell (top) and atomic positions (bottom) of $HfTe_2$ (P-3m1) in Cartesian coordinates.

	x [Å]	y [Å]	z [Å]
$\mathbf{a_1}$	3.97029911	0.00000000	0.00000000
$\mathbf{a_2}$	−1.98514955	3.43837989	0.00000000
$\mathbf{a_3}$	0.00000000	0.00000000	23.56272401

		x [Å]	y [Å]	z [Å]
●	Te	1.98514955	1.14612663	10.01027097
●	Hf	0.00000000	0.00000000	11.78136200
●	Te	-0.00000000	2.29225326	13.55245303

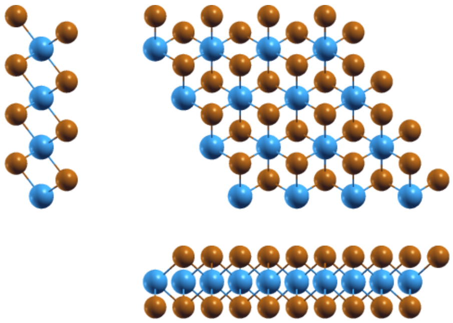

Orthographic projections: views of $HfTe_2$ (P-3m1) as seen from the x axis (left), the y axis (bottom) and the z axis (center).

Lattice matching - minimal size

Formula	N° atoms	strain	cell size 1	cell size 2
K	4	2.9208	1	1
Na	4	0.2629	1	1
GeTe	5	0.009	1	1
As_2	5	0.2744	1	1
S_2	5	0.0075	1	1
CaCl	5	0.1312	1	1
$IrTe_2$	6	0.0082	1	1
Cl_2Zn	6	0.2729	1	1
$MoTe_2$	6	0.2661	1	1
$ReSe_2$	6	1.6004	1	1
CaI_2	6	0.4818	1	1
Te_2V	6	0.2689	1	1
I_2Yb	6	0.4752	1	1
Br_2Co	6	0.2738	1	1
Ca_2N	6	0.2752	1	1
$AuTe_2$	6	0.0079	1	1
Cl_2Zn	6	0.1428	1	1
$PdTe_2$	6	0.0054	1	1
S_2Ti	6	1.5561	1	1
Te_2Zn	6	0.2658	1	1
Bi_2Pd	6	0.4427	1	1
Ba_2Hg	6	0.2079	1	1
Br_2Mn	6	0.2711	1	1
Cl_2Ni	6	0.2473	1	1
Cl_2Co	6	1.5535	1	1
$CrTe_2$	6	0.2554	1	1
PtS_2	6	0.2643	1	1
Br_2V	6	1.5944	1	1
Cl_2Fe	6	1.5483	1	1
CdClO	6	0.2682	1	1
Ba_2N	6	0.0015	1	1
Se_2Ti	6	0.2606	1	1
Br_2Ti	6	0.2546	1	1
Te_2Zr	6	0.0005	1	1
Te_2W	6	0.2663	1	1
$AsSe_2$	6	0.2492	1	1
I_2Tm	6	0.4787	1	1
I_2Pb	6	3.0142	1	1
OTl_2	6	0.2684	1	1
BiITe	6	0.4556	1	1
BrNZr	6	1.6279	1	1
$NbSe_2$	6	0.2478	1	1
Br_2Fe	6	0.2739	1	1
GeS_2	6	0.1231	1	1
$MnSe_2$	6	0.1311	1	1
Br_2Cr	6	0.2551	1	1
Cl_2Zr	6	1.5511	1	1
Se_2Ta	6	0.248	1	1
$NbSe_2$	6	0.2496	1	1
F_2Ni	6	0.1393	1	1

Table showing the first 50 lattice matching combinations with the smallest number of atoms in the supercell.

Lattice matching - minimal strain

Formula	N° atoms	strain	cell size 1	cell size 2
$Gd_2I_2S_2$	486	0.0	64	49
PtS_2	492	0.0	73	91
In_2Se_3	8	0.0	1	1
$CrTe_2$	390	0.0	57	73
$AsSe_2$	339	0.0	49	64
H_2MgO_2	173	0.0001	16	25
Cl_2Sc_2	463	0.0001	57	73
$I_2S_2Sm_2$	429	0.0001	57	43
$Ge_2I_2La_2$	711	0.0002	91	73
Cl_2Fe	255	0.0002	36	49
ClH_3O	233	0.0002	36	25
$NbSe_2$	339	0.0002	49	64
$Er_2F_2Se_2$	9	0.0002	1	1
Ag_2	219	0.0003	49	36
Br_2V	300	0.0003	43	57
Br_2Ca_3Si	486	0.0003	64	49
I_2Yb	435	0.0003	81	64
Ho_2S_2	476	0.0003	72	65
Br_2Cr	390	0.0003	57	73
CdClO	543	0.0004	81	100
$Br_2H_2Zr_2$	609	0.0004	57	73
$I_2Pr_2S_2$	363	0.0004	49	36
CdH_2O_2	743	0.0004	81	100
Ba_2Cu_2	499	0.0005	81	64
Sn_2Te_2	180	0.0005	32	21
Te_2Zr	6	0.0005	1	1
K	214	0.0005	57	43
Cl_2Zr_2	357	0.0005	43	57
Ba_2Pt	255	0.0005	49	36
CoH_2O_2	416	0.0005	37	61
CaI_2	390	0.0006	73	57
Br_2Zr_2	516	0.0006	64	81
OTl_2	543	0.0006	81	100
Cl_2Zr	255	0.0006	36	49
$Cl_2Hf_2N_2$	843	0.0006	81	100
NS_2Zr	583	0.0006	73	91
Br_2Ti	390	0.0006	57	73
$Br_2Hf_2N_2$	462	0.0006	54	50
Ga_2S_3	674	0.0007	73	91
Hg_3N_2	227	0.0007	49	16
$Cl_2Cr_2O_2$	696	0.0007	70	81
$Ag_2K_2Se_2$	840	0.0007	118	81
Pt_2Te_2	7	0.0007	1	1
AsI_2La_2	705	0.0007	100	81
Cd_2I_3	705	0.0008	100	81
CdO_2	255	0.0008	36	49
$HfLiS_2$	583	0.0008	73	91
GeI_2La_2	563	0.0008	81	64
S_2V	123	0.0008	16	25
$H_2I_2Sr_2$	483	0.0008	65	48

Table showing the first 50 lattice matching combinations with the smallest strain needed to build the supercell.

HgI_2 (P-4m2)

Structural and electronic properties

**Formula**	HgI_2
**Spacegroup**	P-4m2
**Prototype**	HgI2
**Parent 3D**	Hg_2I_4
**Source DB**	COD
**DB ID**	9014434
**DF2-C09 Binding energy [meV/ Å^2]**	11.51
**RVV10 Binding energy [meV/ Å^2]**	18.8
**Band gap (PBE) [eV]**	1.79

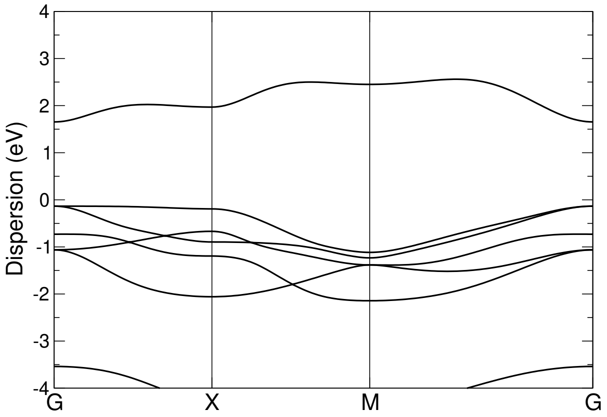

Band structure: Electronic band structure of HgI_2 (P-4m2) in a representative window around the Fermi energy and along a high-symmetry path.

Optimized crystal structure

Structural parameters: relaxed cell (top) and atomic positions (bottom) of HgI_2 (P-4m2) in Cartesian coordinates.

	x [Å]	y [Å]	z [Å]
$\mathbf{a_1}$	4.47733085	0.00000000	0.00000000
$\mathbf{a_2}$	0.00000000	4.47733085	0.00000000
$\mathbf{a_3}$	0.00000000	0.00000000	23.57439385

		x [Å]	y [Å]	z [Å]
●	I	2.23866543	0.00000000	13.56942965
●	Hg	2.23866543	2.23866543	11.78719693
●	I	0.00000000	2.23866543	10.00496420

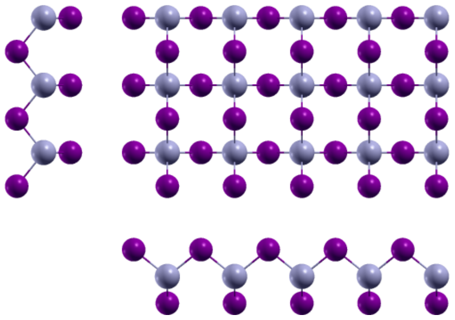

Orthographic projections: views of HgI_2 (P-4m2) as seen from the x axis (left), the y axis (bottom) and the z axis (center).

Lattice matching - minimal size

Formula	N° atoms	strain	cell size 1	cell size 2
InSe	5	0.1086	1	1
K	5	0.976	1	2
AgTl	5	0.2149	1	1
PbTe	5	0.109	1	1
Sm	5	0.149	1	2
PSn_2	6	0.1105	1	1
$AsSn_2$	6	0.1096	1	1
I_2Pr	6	0.5907	1	1
S_2Zr	6	0.1106	1	1
Ca_2Si	6	0.13	1	1
$RhTe_2$	6	0.1091	1	1
CKN	6	0.3703	1	1
I_2Nd	6	0.5936	1	1
Cl_2Cu	6	0.1413	1	1
S_2Sn	6	0.1106	1	1
GeI_2	6	0.1091	1	1
$PtSe_2$	6	0.1095	1	1
Br_2Ni	6	0.1114	1	1
CeI_2	6	0.5884	1	1
$NbTe_2$	6	0.1107	1	1
Cl_2Mg	6	0.1114	1	1
I_2La	6	0.2093	1	1
Bi_2Te_2	7	0.1399	1	1
Li_2Tl_2	7	0.1632	1	1
CdClHO	7	0.1099	1	1
Ir_2P_2	7	0.593	1	1
Ag_2Br_2	7	0.2099	1	1
CdClHO	7	0.1092	1	1
$AsLi_3$	7	0.1087	1	1
S_2Sn_2	7	0.2314	1	1
Cu_2S_2	7	0.5696	1	1
Ge_2Te_2	7	0.0199	1	1
As_2Ir_2	7	0.2125	1	1
P_2Rh_2	7	0.5676	1	1
F_2Tl_2	7	0.568	1	1
Au_2I_2	7	0.0183	1	1
Ge_2Se_2	7	0.2192	1	1
Ag_2Te_2	7	0.5765	1	1
Ni_2Se_2	7	0.5877	1	1
As_2Rh_2	7	0.2096	1	1
Ag_2I_2	7	0.001	1	1
Sn_2Te_2	7	0.0091	1	1
As_2O_3	8	0.7743	1	1
F_4Pb	8	0.0074	1	1
As_2Li_2Nd	8	0.1084	1	1
Bi_2STe_2	8	0.1088	1	1
As_2CeLi_2	8	0.109	1	1
K	8	0.2333	2	2
As_2Li_2Pr	8	0.1086	1	1
PTe_2Ti_2	8	0.1115	1	1

Table showing the first 50 lattice matching combinations with the smallest number of atoms in the supercell.

Lattice matching - minimal strain

Formula	N° atoms	strain	cell size 1	cell size 2
$Ca_2H_2I_2$	687	0.0	65	82
$Ca_2Ge_2Mn_2$	933	0.0	85	113
$I_2Nd_2O_2$	852	0.0	82	101
$Cl_2S_2Tl_2$	786	0.0001	100	81
I_2La	774	0.0001	113	145
Cu_2Te_2	219	0.0002	25	36
$I_2Pr_2Si_2$	534	0.0002	48	65
Cl_2Zr_2	216	0.0002	20	39
Ga_2Te_2	404	0.0002	48	65
$Br_2F_2Tm_2$	933	0.0002	85	113
Ba_2Ni_3	469	0.0002	48	65
As_2Ir_2	516	0.0002	64	81
$Br_2Eu_2F_2$	540	0.0002	50	65
$AsCuLi_2$	404	0.0002	48	65
$Er_2I_2O_2$	540	0.0003	50	65
$I_2Lu_2O_2$	933	0.0003	85	113
$Cl_2F_2Pb_2$	843	0.0003	81	100
Mg_3	486	0.0003	53	109
ReS_2	552	0.0003	54	130
Fe_2S_2	148	0.0003	16	25
$Bi_2I_2O_2$	843	0.0003	81	100
$Cu_2Na_2Se_2$	843	0.0003	81	100
Bi_2Se_2	60	0.0004	8	9
GeI_2	339	0.0004	48	65
GeS_2	513	0.0004	65	106
As_2Rh_2	919	0.0004	113	145
H_2Na_2Pd	725	0.0004	65	106
I_2Nd	339	0.0004	49	64
$F_2I_2Tm_2$	843	0.0005	81	100
Cl_2Cu	243	0.0005	31	50
$I_2O_2Tm_2$	540	0.0005	50	65
$Br_2O_2Sm_2$	531	0.0005	49	64
CCl_2Sc_2	255	0.0005	20	39
$Bi_2Cl_2O_2$	933	0.0005	85	113
P_2Rh_2	304	0.0005	36	49
In	21	0.0006	4	9
Se_2Yb	339	0.0006	48	65
$I_2Nd_2O_2$	843	0.0006	81	100
$As_2Co_2Li_2$	291	0.0006	25	36
$Cl_2O_2Sc_2$	654	0.0006	56	81
$I_2O_2Tm_2$	531	0.0006	49	64
Br_2HLa	404	0.0006	48	65
$Br_2Ho_2O_2$	402	0.0006	36	49
Ir_2P_2	403	0.0006	49	64
Mg_6	369	0.0006	25	49
Ag_2Br_2	919	0.0007	113	145
$Bi_2Br_2O_2$	540	0.0007	50	65
F_2Tl_2	304	0.0007	36	49
O_2Sn_2	539	0.0007	57	92
$Br_2O_2Yb_2$	693	0.0007	61	85

Table showing the first 50 lattice matching combinations with the smallest strain needed to build the supercell.

Ho_2Br_2 (P-3m1)

Structural and electronic properties

**Formula**	Ho_2Br_2
**Spacegroup**	P-3m1
**Prototype**	PtTe
**Parent 3D**	Br_2Ho_2
**Source DB**	MPDS
**DB ID**	S542057
**DF2-C09 Binding energy [meV/ Å^2]**	11.82
**RVV10 Binding energy [meV/ Å^2]**	N/A
**Band gap (PBE) [eV]**	0.0

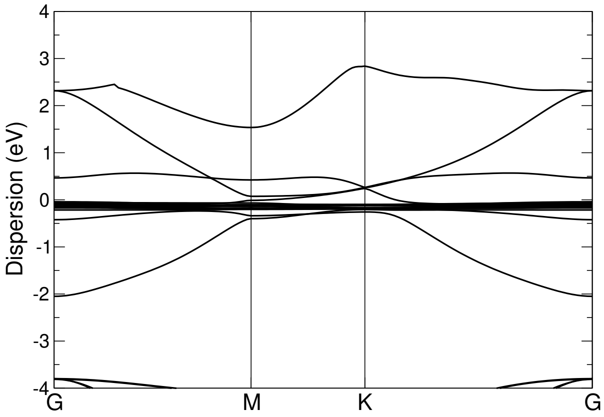

Band structure: Electronic band structure of Ho_2Br_2 (P-3m1) in a representative window around the Fermi energy and along a high-symmetry path.

Optimized crystal structure

Structural parameters: relaxed cell (top) and atomic positions (bottom) of Ho_2Br_2 (P-3m1) in Cartesian coordinates.

		x [Å]	y [Å]	z [Å]
$\mathbf{a_1}$		2.01226630	−3.48534748	0.00000000
$\mathbf{a_2}$		2.01226630	3.48534748	0.00000000
$\mathbf{a_3}$		0.00000000	0.00000000	25.29393139
		x [Å]	y [Å]	z [Å]
●	Ho	2.01226630	-1.16178249	-1.54769168
●	Br	-0.00000000	0.00000000	-3.33410375
●	Ho	2.01226630	1.16178249	1.54769168
●	Br	-0.00000000	-0.00000000	3.33410375

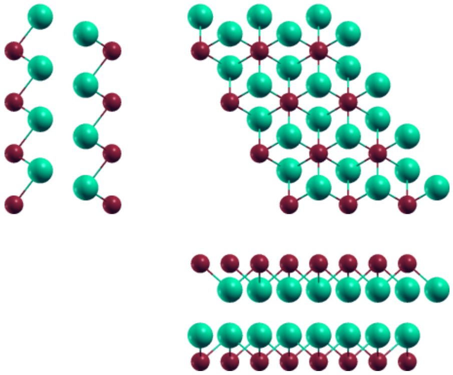

Orthographic projections: views of Ho_2Br_2 (P-3m1) as seen from the x axis (left), the y axis (bottom) and the z axis (center).

Lattice matching - minimal size

Formula	N° atoms	strain	cell size 1	cell size 2
Na	5	0.254	1	1
Gd	5	0.2366	1	1
Ag_2	6	2.9088	1	1
As_2	6	0.2651	1	1
Sb_2	6	0.0094	1	1
CaCl	6	0.1267	1	1
Cl_2Zn	7	0.2636	1	1
$MoTe_2$	7	0.2571	1	1
Ba_2Pt	7	2.9054	1	1
HfS_2	7	0.269	1	1
CaI_2	7	0.4654	1	1
$HfTe_2$	7	0.0063	1	1
Te_2V	7	0.2598	1	1
$CuTe_2$	7	0.2685	1	1
I_2Yb	7	0.4591	1	1
Br_2Co	7	0.2645	1	1
Ca_2N	7	0.2658	1	1
$AuTe_2$	7	0.0014	1	1
Cl_2Zn	7	0.1374	1	1
$PdTe_2$	7	0.0011	1	1
I_2Zn	7	0.006	1	1
Te_2Zn	7	0.2569	1	1
Bi_2Pd	7	0.4281	1	1
Br_2Mn	7	0.2619	1	1
$CrTe_2$	7	0.2469	1	1
PtS_2	7	0.2554	1	1
$CoTe_2$	7	0.2695	1	1
CdClO	7	0.2591	1	1
Ba_2N	7	0.0048	1	1
Te_2Zr	7	0.0058	1	1
Te_2W	7	0.2573	1	1
I_2Tm	7	0.4625	1	1
OTl_2	7	0.2594	1	1
Br_2Fe	7	0.2646	1	1
GeS_2	7	0.1195	1	1
$MnSe_2$	7	0.1267	1	1
Br_2Cr	7	0.2465	1	1
DyI_2	7	0.4726	1	1
Br_2Ni	7	0.2719	1	1
CuO_2	7	0.0909	1	1
Cl_2Mg	7	0.272	1	1
F_2Ni	7	0.1342	1	1
$PtTe_2$	7	0.001	1	1
Br_2Cd	7	0.002	1	1
NaPSn	7	0.0072	1	1
Fe_2Te_2	8	0.1472	1	1
Ca_2Cl_2	8	0.1476	1	1
Cl_2OOs	8	0.1331	1	1
NS_2Zr	8	0.2546	1	1
Br_2Er_2	8	0.0019	1	1

Table showing the first 50 lattice matching combinations with the smallest number of atoms in the supercell.

Lattice matching - minimal strain

Formula	N° atoms	strain	cell size 1	cell size 2
PtS_2	447	0.0	57	73
GeI_3Rb	120	0.0	25	4
OTl_2	499	0.0	64	81
$Cl_2Hf_2N_2$	742	0.0001	64	81
$Cl_2N_2Zr_2$	924	0.0001	81	100
Br_2Co	565	0.0001	73	91
FeO_2	496	0.0001	49	100
Br_2V	291	0.0001	36	49
CdClO	499	0.0001	64	81
Br_2Ca_3Si	634	0.0002	73	57
N_2W	84	0.0002	9	16
Br_2Fe	565	0.0002	73	91
CaH_2O_2	824	0.0002	81	100
I_2La_2Te	577	0.0002	73	57
Bi_2S_3	9	0.0002	1	1
GeI_2La_2	805	0.0003	100	81
C_4Ca_2	628	0.0003	70	58
$I_2Pr_2S_2$	486	0.0003	57	43
Te_2V	499	0.0003	64	81
Se_2Ta	343	0.0003	43	57
$AgNO_3$	429	0.0004	61	37
CCl_2Lu_2	747	0.0004	73	91
$LiOS_2Ti$	280	0.0004	25	36
Ag_2	314	0.0004	57	43
$C_2Br_2La_2$	644	0.0004	68	62
Br_2Tb_2	8	0.0005	1	1
$CrSe_2$	139	0.0005	16	25
Cl_2Zn	565	0.0005	73	91
I_2Tm	643	0.0005	100	81
$Au_2K_2Se_2$	722	0.0005	128	35
As_2	474	0.0005	73	91
PbS_2Sn	708	0.0005	99	78
Ag_2Te_2	572	0.0006	73	70
$Cl_2H_2Sc_2$	514	0.0006	43	57
NiO_2	393	0.0006	39	79
CaI_2	583	0.0006	91	73
$Br_2Hf_2N_2$	924	0.0006	81	100
$CuTe_2$	624	0.0006	81	100
NS_2Zr	520	0.0006	57	73
CoO_2	496	0.0006	49	100
Cl_2Zr_2	340	0.0007	36	49
$Br_2O_2Sc_2$	634	0.0007	64	63
Ba_2Pt	357	0.0007	57	43
Ga_2S_3	593	0.0007	57	73
Cl_2V	139	0.0007	16	25
$HfLiS_2$	520	0.0008	57	73
I_2Yb	643	0.0008	100	81
$I_2Nd_2S_2$	550	0.0009	64	49
$I_2S_2Tb_2$	708	0.0009	81	64
$Cu_2Na_2Te_2$	548	0.0009	65	48

Table showing the first 50 lattice matching combinations with the smallest strain needed to build the supercell.

$Ho_2O_2Br_2$ (P4/nmm)

Structural and electronic properties

**Formula**	$Ho_2O_2Br_2$
**Spacegroup**	P4/nmm
**Prototype**	PbClF
**Parent 3D**	$Br_2Ho_2O_2$
**Source DB**	MPDS
**DB ID**	S1903366
**DF2-C09 Binding energy [meV/ Å^2]**	15.49
**RVV10 Binding energy [meV/ Å^2]**	N/A
**Band gap (PBE) [eV]**	0.0

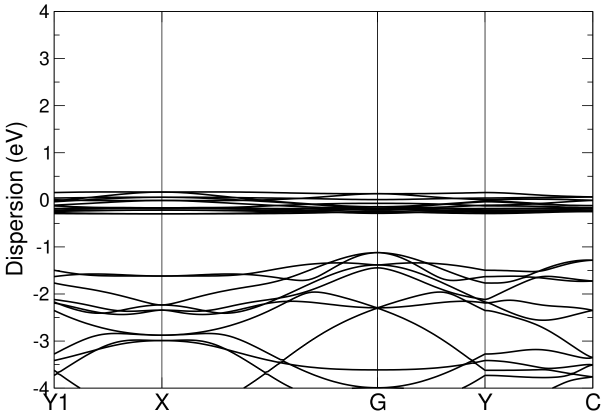

Band structure: Electronic band structure of $Ho_2O_2Br_2$ (P4/nmm) in a representative window around the Fermi energy and along a high-symmetry path.

Optimized crystal structure

Structural parameters: relaxed cell (top) and atomic positions (bottom) of $Ho_2O_2Br_2$ (P4/nmm) in Cartesian coordinates.

		x [Å]	y [Å]	z [Å]
$\mathbf{a_1}$		3.83250167	0.00030170	0.00000000
$\mathbf{a_2}$		0.00030170	3.83250167	0.00000000
$\mathbf{a_3}$		0.00000000	0.00000000	22.92232254
		x [Å]	y [Å]	z [Å]
●	Ho	0.95802039	-0.95802039	1.14900720
●	Br	-0.95818432	-2.87461905	2.81051089
●	Ho	-0.95832209	-2.87448128	-1.14900720
●	Br	0.95788262	-0.95788262	-2.81051089
•	O	-0.95820912	-0.95820912	0.00000000
•	O	0.95790742	-2.87429255	0.00000000

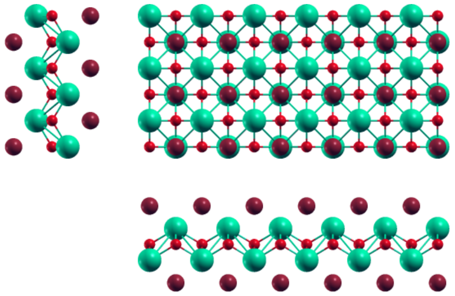

Orthographic projections: views of $Ho_2O_2Br_2$ (P4/nmm) as seen from the x axis (left), the y axis (bottom) and the z axis (center).

Lattice matching - minimal size

Formula	N° atoms	strain	cell size 1	cell size 2
K	7	0.3965	1	1
K	7	0.1956	1	1
In	7	0.1101	1	1
InSe	8	0.1541	1	1
Bi_2	8	0.1601	1	1
Ag_2	8	0.7672	1	1
PbTe	8	0.1559	1	1
Sb_2	8	0.1394	1	1
I_2Mg	9	0.1444	1	1
S_2V	9	0.1094	1	1
MoS_2	9	0.1092	1	1
CdI_2	9	0.1577	1	1
Ba_2Pt	9	0.7662	1	1
Br_2Ca	9	0.1589	1	1
HfS_2	9	0.1085	1	1
CaI_2	9	0.1837	1	1
I_2Pr	9	0.0091	1	1
Br_2La	9	0.1447	1	1
Br_2Cu	9	0.11	1	1
Ca_2Si	9	0.7837	1	1
I_2Yb	9	0.1806	1	1
BiClTe	9	0.158	1	1
$AuTe_2$	9	0.1332	1	1
BrCdI	9	0.1472	1	1
$PdTe_2$	9	0.1313	1	1
HgI_2	9	1.166	1	1
I_2Zn	9	0.1368	1	1
BaF_2	9	0.1504	1	1
BiBrTe	9	0.1639	1	1
S_2W	9	0.1092	1	1
GeI_2	9	0.1427	1	1
$CoTe_2$	9	0.1086	1	1
AsKSn	9	0.149	1	1
$PbTe_2$	9	0.1463	1	1
Cl_2Cu	9	0.1008	1	1
I_2Tm	9	0.1822	1	1
$SnTe_2$	9	0.1408	1	1
GeI_2	9	0.1561	1	1
I_2Pb	9	0.7733	1	1
STl_2	9	0.1513	1	1
BiITe	9	0.1713	1	1
DyI_2	9	0.1873	1	1
Br_2Ni	9	0.109	1	1
CeI_2	9	0.0083	1	1
Se_2Yb	9	0.1429	1	1
MoS_2	9	0.1092	1	1
Cl_2Mg	9	0.109	1	1
$BiTe_2$	9	0.1432	1	1
GdI_2	9	0.1671	1	1
$PtTe_2$	9	0.1329	1	1

Table showing the first 50 lattice matching combinations with the smallest number of atoms in the supercell.

Lattice matching - minimal strain

Formula	N° atoms	strain	cell size 1	cell size 2
In	375	0.0001	49	81
$Ca_2Mn_2Si_2$	12	0.0002	1	1
$Ba_2F_2I_2$	876	0.0002	85	61
HgO	536	0.0002	61	85
$Ba_2H_2I_2$	366	0.0003	36	25
C_2Li_2	246	0.0003	25	24
H_2Li_2Pd	221	0.0004	16	25
$As_2Mg_2Na_2$	870	0.0004	81	64
Se_2Ti	483	0.0004	48	65
$Br_2Ca_2H_2$	12	0.0004	1	1
Ca_2O_2	962	0.0004	85	113
Cl_4Cu_2	738	0.0005	89	34
Bi_2Se_2	912	0.0005	98	81
HgI_2	402	0.0006	49	36
S_2Ti	840	0.0007	81	118
K	550	0.0007	81	64
O_4PTl	876	0.0007	85	61
Cl_2Co	840	0.0007	81	118
$CuGeO_3$	735	0.0007	70	63
Br_2Zr_2	548	0.0008	48	65
$Ag_2K_2Se_2$	510	0.0009	49	36
H_4Ti	221	0.0009	16	25
Cl_2Zr	840	0.0009	81	118
C_4Ca_2	906	0.001	81	70
Au_2Br_2	914	0.001	99	80
$C_2I_2La_2$	138	0.0011	12	11
C_4Ca_2	774	0.0011	69	60
P_2Rh_2	10	0.0012	1	1
Cl_2Fe	840	0.0012	81	118
$Br_2Er_2O_2$	12	0.0012	1	1
Na	353	0.0013	48	65
Mg_6	366	0.0013	25	36
F_2Tl_2	10	0.0013	1	1
$H_2Na_2O_2$	882	0.0013	65	82
$H_2I_2Sr_2$	882	0.0014	82	65
C_2	510	0.0014	44	123
Mo_2Te_4	450	0.0014	45	30
$H_2Na_2O_2$	870	0.0014	64	81
Ge_2Te_2	712	0.0015	78	61
$NbSe_2$	531	0.0015	52	73
$AgNO_2$	602	0.0015	63	56
$AgTe_2$	258	0.0015	25	36
$AsSe_2$	531	0.0016	52	73
In	493	0.0016	64	109
Mg_2	146	0.0016	16	25
Sn	445	0.0016	58	97
Pb_2Se_2	874	0.0016	97	73
Ga_2S_3	613	0.0016	48	65
O_2Pt	711	0.0016	64	109
Br_2CsF	316	0.0016	36	25

Table showing the first 50 lattice matching combinations with the smallest strain needed to build the supercell.

$Ho_2O_2Cl_2$ (P-3m1)

Structural and electronic properties

**Formula**	$Ho_2O_2Cl_2$
**Spacegroup**	P-3m1
**Prototype**	SmSI
**Parent 3D**	$Cl_2Ho_2O_2$
**Source DB**	MPDS
**DB ID**	S1936402
**DF2-C09 Binding energy [meV/ Å^2]**	11.01
**RVV10 Binding energy [meV/ Å^2]**	N/A
**Band gap (PBE) [eV]**	0.0

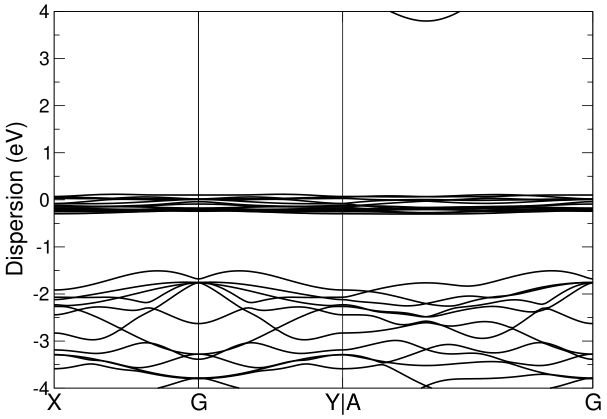

Band structure: Electronic band structure of $Ho_2O_2Cl_2$ (P-3m1) in a representative window around the Fermi energy and along a high-symmetry path.

Optimized crystal structure

Structural parameters: relaxed cell (top) and atomic positions (bottom) of $Ho_2O_2Cl_2$ (P-3m1) in Cartesian coordinates.

	x [Å]	y [Å]	z [Å]
$\mathbf{a_1}$	1.90399125	−3.29786347	0.00000000
$\mathbf{a_2}$	1.90403792	3.29789042	0.00000000
$\mathbf{a_3}$	0.00000000	0.00000000	24.53336819

		x [Å]	y [Å]	z [Å]
●	Ho	0.95198084	-2.74822001	-13.69502652
●	Ho	0.95201041	-0.54964347	-10.83834167
●	Cl	0.95202727	1.64894041	-9.11592084
●	Cl	0.95201065	1.64895001	-15.41744735
●	O	0.95198117	-2.74822020	-11.42527620
●	O	0.95201008	-0.54964328	-13.10809199

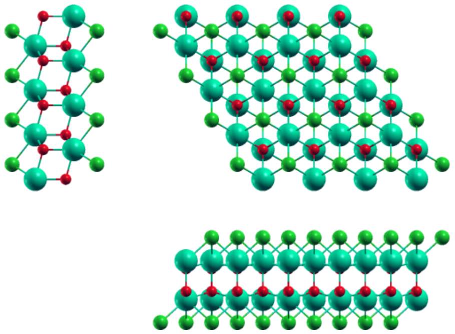

Orthographic projections: views of $Ho_2O_2Cl_2$ (P-3m1) as seen from the x axis (left), the y axis (bottom) and the z axis (center).

Lattice matching - minimal size

Formula	N° atoms	strain	cell size 1	cell size 2
Tl	7	0.2472	1	1
InSe	8	0.4651	1	1
Nd	8	0.2255	1	2
HgO	8	0.1144	1	1
AsSb	8	0.0044	1	1
Bi_2	8	0.4798	1	1
PbTe	8	0.4695	1	1
CaCl	8	0.1475	1	1
$CdCl_2$	9	0.0096	1	1
Cl_2Mn	9	0.2549	1	1
CdI_2	9	0.4739	1	1
$AgTe_2$	9	0.111	1	1
$MoSe_2$	9	0.2462	1	1
$ReSe_2$	9	0.2734	1	1
S_2Ta	9	0.2577	1	1
Br_2Zn	9	0.0057	1	1
Br_2Ca	9	0.477	1	1
$GeTe_2$	9	0.0085	1	1
$SiTe_2$	9	0.0017	1	1
NSr_2	9	0.0061	1	1
PbS_2	9	0.0018	1	1
BiClTe	9	0.4748	1	1
BrCdI	9	0.4474	1	1
FeI_2	9	0.0074	1	1
I_2Ni	9	0.0087	1	1
S_2Ti	9	0.2646	1	1
Mg_3	9	0.4248	1	1
Te_2Ti	9	0.0054	1	1
NbS_2	9	0.2571	1	1
CrI_2	9	0.007	1	1
BaF_2	9	0.4555	1	1
Bi_2Pd	9	0.1189	1	1
Cl_2Ni	9	0.2748	1	1
Cl_2Co	9	0.2641	1	1
Br_2V	9	0.2722	1	1
ClNZr	9	0.2692	1	1
Cl_2Fe	9	0.2631	1	1
S_2Ta	9	0.2501	1	1
Se_2V	9	0.2483	1	1
AsKSn	9	0.452	1	1
$NiTe_2$	9	0.0023	1	1
I_2V	9	0.0004	1	1
GeI_2	9	0.4701	1	1
Se_2Zr	9	0.0013	1	1
STl_2	9	0.4579	1	1
CdO_2	9	0.2639	1	1
$NbSe_2$	9	0.2754	1	1
CoI_2	9	0.0049	1	1
GeS_2	9	0.137	1	1
$MnSe_2$	9	0.1474	1	1

Table showing the first 50 lattice matching combinations with the smallest number of atoms in the supercell.

Lattice matching - minimal strain

Formula	N° atoms	strain	cell size 1	cell size 2
Cl_2NSc_2	707	0.0	57	73
Cl_2Hf_2	708	0.0	64	81
Cu_2O_2	606	0.0001	65	54
Cl_2Co	711	0.0001	73	91
$Br_2Hf_2N_2$	834	0.0002	75	64
F_4Nb	630	0.0002	65	48
Bi_2STe_2	911	0.0002	91	73
S_2Ti	711	0.0002	73	91
CdO_2	711	0.0003	73	91
$I_2S_2Sm_2$	366	0.0003	36	25
I_2V	9	0.0004	1	1
H_2MgO_2	330	0.0004	25	36
Ca_4Cu_2	894	0.0004	99	50
I_2Pr	678	0.0004	81	64
Br_2Ca	678	0.0004	81	64
Sb_2Te_3	723	0.0004	73	57
I_2La_2P	629	0.0004	64	49
$AsLi_3$	838	0.0004	91	73
$Al_2Cl_2O_2$	624	0.0004	50	54
$AlLiTe_2$	514	0.0005	57	43
Cl_2Mn	561	0.0005	57	73
Cl_2Zr	711	0.0005	73	91
BiClTe	678	0.0005	81	64
$Br_2Cr_2S_2$	846	0.0005	77	64
GdI_2	531	0.0005	64	49
Bi_2Te_3	557	0.0006	57	43
ClH_3O	230	0.0006	25	16
S_2Ta	486	0.0006	49	64
K	241	0.0007	36	25
BiITe	471	0.0007	57	43
Se_2V	486	0.0007	49	64
$Cl_2O_2Y_2$	12	0.0007	1	1
Gd_2GeI_2	806	0.0007	81	64
O_2Zn	171	0.0007	16	25
InSe	692	0.0007	91	73
Bi_2SeTe_2	723	0.0007	73	57
N_2Re	102	0.0007	9	16
As_2Li_2Pr	911	0.0008	91	73
$Ga_2I_2Tb_2$	870	0.0008	81	64
$I_2La_2Si_2$	984	0.0008	91	73
As_2O_3	669	0.0008	79	39
CdI_2	678	0.0008	81	64
Cl_2Fe	711	0.0009	73	91
$Ge_2I_2La_2$	510	0.0009	49	36
La_2S_2	348	0.0009	40	27
$Ag_2K_2Te_2$	606	0.0009	65	36
Br_2OV	640	0.0009	64	64
Br_2Hg_3	429	0.001	64	9
Cu_4Te_2	780	0.001	73	57
$Cl_2N_2Ti_2$	426	0.001	36	35

Table showing the first 50 lattice matching combinations with the smallest strain needed to build the supercell.

$Ho_2S_2Br_2$ (Pmm2)

Structural and electronic properties

**Formula**	$Ho_2S_2Br_2$
**Spacegroup**	Pmm2
**Prototype**	FeOCl
**Parent 3D**	$Br_2Ho_2S_2$
**Source DB**	MPDS
**DB ID**	S307277
**DF2-C09 Binding energy [meV/ Å^2]**	10.39
**RVV10 Binding energy [meV/ Å^2]**	N/A
**Band gap (PBE) [eV]**	0.0

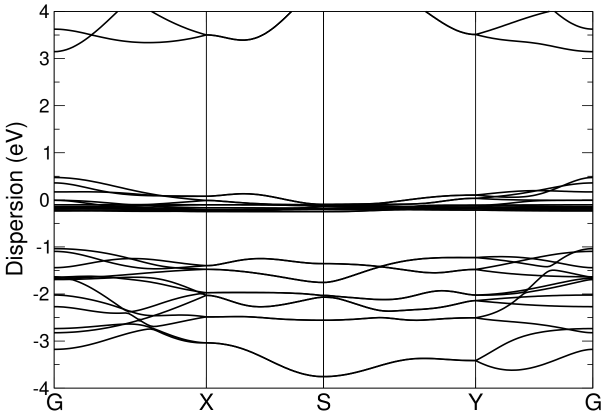

Band structure: Electronic band structure of $Ho_2S_2Br_2$ (Pmm2) in a representative window around the Fermi energy and along a high-symmetry path.

Optimized crystal structure

Structural parameters: relaxed cell (top) and atomic positions (bottom) of $Ho_2S_2Br_2$ (Pmm2) in Cartesian coordinates.

		x [Å]	y [Å]	z [Å]
$\mathbf{a_1}$		4.07895474	0.00000000	0.00000000
$\mathbf{a_2}$		0.00000000	5.29439293	0.00000000
$\mathbf{a_3}$		0.00000000	0.00000000	24.21494472
		x [Å]	y [Å]	z [Å]
●	Ho	1.01984437	-1.32359823	-1.17447756
●	S	1.01965684	-3.97079470	-0.65504702
●	Br	-1.01955559	-1.32359823	-3.11926310
●	Ho	-1.01984437	-3.97079470	1.17447756
●	S	-1.01965684	-1.32359823	0.65504702
●	Br	1.01955559	-3.97079470	3.11926310

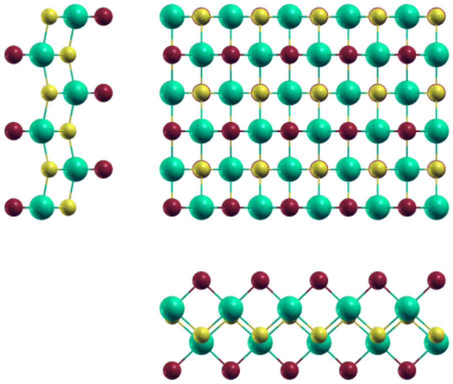

Orthographic projections: views of $Ho_2S_2Br_2$ (Pmm2) as seen from the x axis (left), the y axis (bottom) and the z axis (center).

Lattice matching - minimal size

Formula	N° atoms	strain	cell size 1	cell size 2
AgTl	8	0.257	1	1
In	8	0.1461	1	2
Gd	8	0.1317	1	2
Ba_2Hg	9	0.2724	1	1
CNRb	9	0.3141	1	1
CNNa	9	0.3063	1	1
Bi_2In_2	10	0.0622	1	1
Cu_2I_2	10	0.2577	1	1
Cu_2Te_2	10	1.7878	1	1
O_2Pb_2	10	0.7554	1	1
As_4	10	0.3124	1	1
P_2	10	0.3722	1	2
O_2Sn_2	10	0.1683	1	1
Bi_2O_2	10	0.2549	1	1
PbS_2Sn	10	0.8061	1	1
$SbSe_2Tl$	10	0.4936	1	1
Br_2CsF	10	0.0615	1	1
Gd	10	0.0455	1	4
Sn_2Te_2	10	0.3416	1	1
F_4Sn	11	0.2712	1	1
FKO_2Se	11	0.5704	1	1
Cl_4Mn	11	0.0646	1	1
$Ba_2H_2I_2$	12	0.0609	1	1
S_2V	12	0.1482	1	2
$I_2Lu_2Se_2$	12	0.5554	1	1
$Br_2F_2Sr_2$	12	0.2604	1	1
$Ho_2I_2S_2$	12	0.0096	1	1
Cu_4Te_2	12	0.1171	1	1
$Eu_2F_2I_2$	12	0.2542	1	1
AlH_4Na	12	0.0658	1	1
$GeTe_2$	12	0.6648	1	2
$Cl_2F_2Pb_2$	12	0.7397	1	1
$F_2I_2Sm_2$	12	0.7547	1	1
ReS_2	12	0.1409	1	2
Br_2Ca_3Si	12	0.2728	1	1
Cl_2Ti	12	0.3725	1	2
$Br_2S_2Y_2$	12	0.0043	1	1
$Gd_2I_2S_2$	12	0.015	1	1
$C_2Br_2Gd_2$	12	0.1078	1	1
N_2W	12	0.1345	1	2
$Br_2F_2Pb_2$	12	0.2601	1	1
$Cu_2Na_2Se_2$	12	0.7395	1	1
$Br_2Er_2Se_2$	12	0.0191	1	1
$I_2S_2Tb_2$	12	0.0127	1	1
Te_4TiZr	12	0.6651	1	1
$I_2S_2Yb_2$	12	0.0045	1	1
$Cu_2Rb_2Te_2$	12	0.0621	1	1
$Br_2Dy_2S_2$	12	0.0022	1	1
$Cl_2Ga_2Te_2$	12	0.0462	1	1
$I_2Nd_2O_2$	12	0.7429	1	1

Table showing the first 50 lattice matching combinations with the smallest number of atoms in the supercell.

Lattice matching - minimal strain

Formula	N° atoms	strain	cell size 1	cell size 2
$NbTe_2$	624	0.0002	54	100
$Br_2Hf_2N_2$	726	0.0003	49	72
I_2Zn	42	0.0003	4	6
S_2Zr	624	0.0003	54	100
Ag_2F_4	918	0.0004	90	63
Se_2Ta	291	0.0005	24	49
Te_2Zn	582	0.0005	49	96
S_2Sn	624	0.0005	54	100
As_2O_3	327	0.0007	32	27
$Cl_2H_2Sc_2$	438	0.0007	24	49
$As_2Cd_2K_2$	738	0.0007	60	63
Se_4TiZr	624	0.0008	54	50
I_2La_2Sb	322	0.0008	27	32
Br_2La_2P	949	0.0008	74	101
CdI_2	747	0.0008	74	101
CrS_2	42	0.0008	3	8
CdI_2	747	0.0008	74	101
Bi_2Se_2	912	0.0009	84	102
CNNa	552	0.0009	56	72
PTe_2Zr_2	564	0.0009	39	66
$Ag_2K_2Te_2$	738	0.0009	63	60
C_4Ca_2	846	0.001	62	79
Ga_2Se_2	788	0.001	60	107
Bi_2STe_2	949	0.001	74	101
BiClTe	747	0.001	74	101
$Br_2O_2Sc_2$	318	0.001	21	32
PSn_2	624	0.001	54	100
I_2Pr	747	0.0011	74	101
Hf_3Te_2	943	0.0011	68	107
AsSb	366	0.0011	39	66
I_2Ti	486	0.0011	44	74
K	194	0.0011	27	32
Cl_2OOs	696	0.0012	56	90
CI_2Y_2	634	0.0012	44	74
CBr_2Lu_2	824	0.0012	54	100
CoI_2	486	0.0012	44	74
Br_2Ti	291	0.0012	24	49
$LiMnTe_2$	872	0.0012	76	104
$LiMnTe_2$	848	0.0012	74	101
$Br_2N_2Zr_2$	924	0.0013	54	100
Se_2Sn	486	0.0013	44	74
$Br_2Er_2S_2$	12	0.0013	1	1
Cl_2OOs	672	0.0013	54	87
$F_2Lu_2Se_2$	60	0.0013	4	6
In_2Se_2	48	0.0013	4	6
GeI_2	768	0.0013	76	104
$AgTe_2$	261	0.0013	21	45
Br_2Zr_2	672	0.0014	48	96
NSr_2	486	0.0014	44	74
Te_4W_2	426	0.0014	36	35

Table showing the first 50 lattice matching combinations with the smallest strain needed to build the supercell.

$Ho_2Se_2F_2$ (P-3m1)

Structural and electronic properties

**Formula**	$Ho_2Se_2F_2$
**Spacegroup**	P-3m1
**Prototype**	SmSI
**Parent 3D**	$F_2Ho_2Se_2$
**Source DB**	MPDS
**DB ID**	S307730
**DF2-C09 Binding energy [meV/ Å^2]**	16.28
**RVV10 Binding energy [meV/ Å^2]**	N/A
**Band gap (PBE) [eV]**	0.0

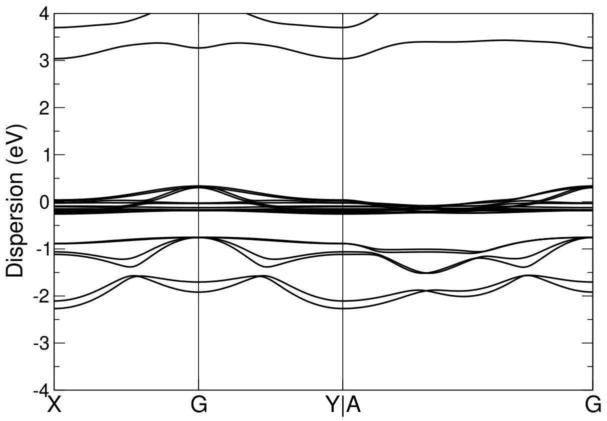

Band structure: Electronic band structure of $Ho_2Se_2F_2$ (P-3m1) in a representative window around the Fermi energy and along a high-symmetry path.

Optimized crystal structure

Structural parameters: relaxed cell (top) and atomic positions (bottom) of $Ho_2Se_2F_2$ (P-3m1) in Cartesian coordinates.

		x [Å]	y [Å]	z [Å]
$\mathbf{a_1}$		1.99846434	−3.46144177	0.00000000
$\mathbf{a_2}$		1.99836804	3.46138617	0.00000000
$\mathbf{a_3}$		0.00000000	0.00000000	25.04985692
		x [Å]	y [Å]	z [Å]
●	Ho	0.99918548	0.57688001	1.70195087
●	Se	0.99924619	-1.73071279	3.27643241
●	F	-0.99922314	-0.57690175	0.72726051
●	Ho	-0.99918548	-0.57688001	-1.70195087
●	Se	0.99921815	-1.73072898	-3.27643241
●	F	0.99922314	0.57690175	-0.72726051

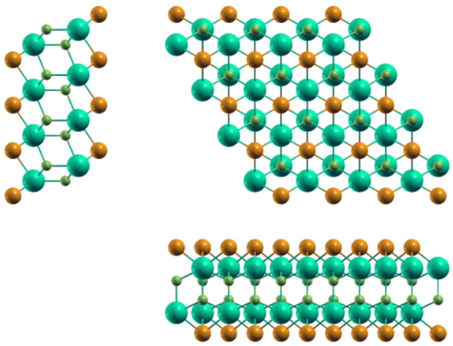

Orthographic projections: views of $Ho_2Se_2F_2$ (P-3m1) as seen from the x axis (left), the y axis (bottom) and the z axis (center).

Lattice matching - minimal size

Formula	N° atoms	strain	cell size 1	cell size 2
K	7	0.4976	1	1
Na	7	0.2585	1	1
Gd	7	0.2402	1	1
As_2	8	0.2698	1	1
CaCl	8	0.1289	1	1
Cl_2Zn	9	0.2682	1	1
$MoTe_2$	9	0.2616	1	1
HfS_2	9	0.2738	1	1
CaI_2	9	0.4736	1	1
$HfTe_2$	9	0.0031	1	1
Te_2V	9	0.2644	1	1
$CuTe_2$	9	0.2732	1	1
I_2Yb	9	0.4672	1	1
Br_2Co	9	0.2691	1	1
Ca_2N	9	0.2705	1	1
$AuTe_2$	9	0.0047	1	1
Cl_2Zn	9	0.1401	1	1
$PdTe_2$	9	0.0022	1	1
I_2Zn	9	0.0094	1	1
Te_2Zn	9	0.2614	1	1
Br_2Mn	9	0.2665	1	1
PtS_2	9	0.2599	1	1
$CoTe_2$	9	0.2743	1	1
CdClO	9	0.2637	1	1
Ba_2N	9	0.0016	1	1
Se_2Ti	9	0.2563	1	1
Br_2Ti	9	0.2504	1	1
Te_2Zr	9	0.0026	1	1
Te_2W	9	0.2619	1	1
I_2Tm	9	0.4706	1	1
OTl_2	9	0.2639	1	1
BiITe	9	0.4479	1	1
CdO_2	9	1.53	1	1
BrNZr	9	0.2468	1	1
Br_2Fe	9	0.2692	1	1
GeS_2	9	0.1213	1	1
$MnSe_2$	9	0.1289	1	1
Br_2Cr	9	0.2508	1	1
DyI_2	9	0.4809	1	1
CuO_2	9	0.0899	1	1
F_2Ni	9	0.1368	1	1
Se_2Ta	9	0.2494	1	1
$PtTe_2$	9	0.0042	1	1
Br_2Cd	9	0.0012	1	1
NaPSn	9	0.004	1	1
Fe_2Te_2	10	0.1503	1	1
Ca_2Cl_2	10	0.1507	1	1
Cl_2Gd_2	10	0.0084	1	1
Cl_2OOs	10	0.1356	1	1
NS_2Zr	10	0.2591	1	1

Table showing the first 50 lattice matching combinations with the smallest number of atoms in the supercell.

Lattice matching - minimal strain

Formula	N° atoms	strain	cell size 1	cell size 2
Te_2V	711	0.0	73	91
Ba_2Cu_2	838	0.0	91	73
Se_2Ta	486	0.0001	49	64
O_2Sn_2	750	0.0001	77	72
I_2Yb	765	0.0001	91	73
NS_2Zr	708	0.0002	64	81
Br_2Zr_2	634	0.0002	57	73
H_2NiO_2	221	0.0002	16	25
Ga_2S_3	789	0.0002	64	81
Gd	314	0.0002	38	86
Bi_2Te_2	316	0.0003	36	25
$Cl_2Hf_2N_2$	984	0.0003	73	91
OTl_2	711	0.0003	73	91
$AsSe_2$	429	0.0003	43	57
$Br_2O_2Ti_2$	672	0.0003	56	56
$Br_2O_2V_2$	756	0.0003	60	66
$Cl_2H_2Sc_2$	678	0.0003	49	64
KS_2Ti	886	0.0003	81	100
PtS_2	627	0.0004	64	81
$I_2S_2Tb_2$	780	0.0005	73	57
Sb_2Te_2	438	0.0005	49	36
Cl_2Zn	786	0.0005	81	100
CdClO	711	0.0005	73	91
$I_2S_2Sm_2$	678	0.0005	64	49
Se_2V	258	0.0005	25	36
$NbSe_2$	429	0.0006	43	57
Na	465	0.0006	64	81
Se_2Ti	561	0.0006	57	73
$I_2Nd_2S_2$	600	0.0006	57	43
Se_2Ta	429	0.0006	43	57
$NbSe_2$	429	0.0007	43	57
Br_2Mn	786	0.0007	81	100
S_2Ta	258	0.0008	25	36
K	433	0.0008	64	49
Ca_2Si	402	0.0008	49	36
$Ce_2I_2S_2$	510	0.0008	49	36
DyI_2	609	0.0009	73	57
Br_2Ti	486	0.0009	49	64
CaI_2	678	0.0009	81	64
N_2Re	297	0.0009	25	49
ReS_2	537	0.0009	49	81
Te_4W_2	750	0.001	77	48
Br_2PY_2	11	0.0011	1	1
$Br_2H_2Zr_2$	678	0.0011	49	64
$Cl_2N_2Zr_2$	558	0.0011	48	45
Br_2Co	786	0.0011	81	100
$C_2Br_2Tb_2$	696	0.0011	58	58
Br_2Fe	786	0.0011	81	100
Cl_2Ni	429	0.0012	43	57
K	438	0.0012	65	48

Table showing the first 50 lattice matching combinations with the smallest strain needed to build the supercell.

$Ho_2Se_2I_2$ (Pmm2)

Structural and electronic properties

**Formula**	$Ho_2Se_2I_2$
**Spacegroup**	Pmm2
**Prototype**	FeOCl
**Parent 3D**	$Ho_2I_2Se_2$
**Source DB**	MPDS
**DB ID**	S376104
**DF2-C09 Binding energy [meV/ Å^2]**	11.4
**RVV10 Binding energy [meV/ Å^2]**	N/A
**Band gap (PBE) [eV]**	0.0

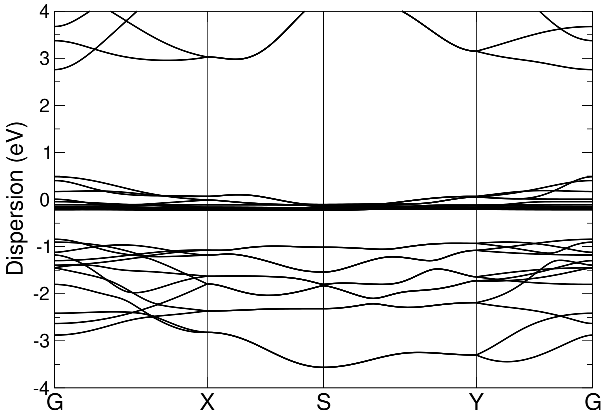

Band structure: Electronic band structure of $Ho_2Se_2I_2$ (Pmm2) in a representative window around the Fermi energy and along a high-symmetry path.

Optimized crystal structure

Structural parameters: relaxed cell (top) and atomic positions (bottom) of $Ho_2Se_2I_2$ (Pmm2) in Cartesian coordinates.

	x [Å]	y [Å]	z [Å]
$\mathbf{a_1}$	−4.26208868	0.00000000	0.00000000
$\mathbf{a_2}$	0.00000000	−5.59985158	0.00000000
$\mathbf{a_3}$	0.00000000	0.00000000	25.05819484

		x [Å]	y [Å]	z [Å]
●	I	-1.06528119	-1.39996289	-3.35302798
●	Ho	1.06566235	-1.39996289	-1.17774023
●	Ho	-1.06566235	1.39996289	1.17774023
●	Se	1.06541064	1.39996289	-0.75307577
●	Se	-1.06541064	-1.39996289	0.75307577
●	I	1.06528119	1.39996289	3.35302798

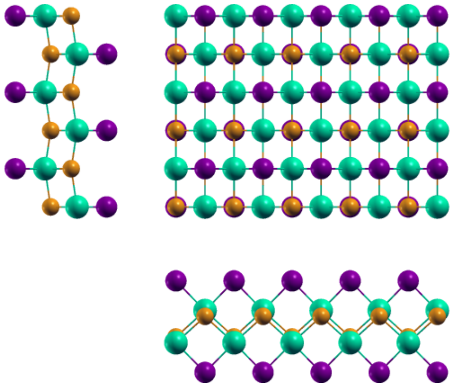

Orthographic projections: views of $Ho_2Se_2I_2$ (Pmm2) as seen from the x axis (left), the y axis (bottom) and the z axis (center).

Lattice matching - minimal size

Formula	N° atoms	strain	cell size 1	cell size 2
Tl	8	0.144	1	2
CNRb	9	0.1962	1	1
In_2Se_2	10	0.0858	1	1
Au_2Br_2	10	0.0702	1	1
Ge_2Te_2	10	0.2702	1	1
As_4	10	0.6094	1	1
Au_2Se_2	10	0.2176	1	1
LiO	10	0.1368	1	2
P_2	10	0.1409	1	2
La_2S_2	10	0.802	1	1
$SbSe_2Tl$	10	0.1727	1	1
Se_2Sn_2	10	0.8043	1	1
F_4Pb	11	0.2771	1	1
KNO_3	11	0.3197	1	1
Cl_2Mn	12	0.1478	1	2
$MoSe_2$	12	0.1436	1	2
$ReSe_2$	12	0.3689	1	2
$Ho_2I_2S_2$	12	0.0177	1	1
S_2Ta	12	0.3477	1	2
$CuTe_2$	12	0.5036	1	2
PbS_2	12	0.5731	1	2
Cl_2Ti	12	0.141	1	2
$K_2O_2Tl_2$	12	0.1867	1	1
$I_2Se_2Tb_2$	12	0.0035	1	1
$Gd_2I_2Se_2$	12	0.0055	1	1
NbS_2	12	0.3468	1	2
$Gd_2I_2S_2$	12	0.0154	1	1
Cl_2Ni	12	0.3707	1	2
Pd_2S_4	12	0.3506	1	1
$Br_2Er_2Se_2$	12	0.0107	1	1
Br_2V	12	0.3673	1	2
ClNZr	12	0.3632	1	2
Se_2V	12	0.1446	1	2
$I_2S_2Tb_2$	12	0.0163	1	1
$Er_2I_2Se_2$	12	0.001	1	1
$I_2Se_2Tm_2$	12	0.0026	1	1
$SnTe_2$	12	0.6859	1	2
Cl_2V	12	0.1356	1	2
Ca_4Cu_2	12	0.0216	1	1
AuI_4Li	12	0.116	1	1
$NbSe_2$	12	0.3716	1	2
$H_2I_2Sr_2$	12	0.7729	1	1
$Dy_2I_2S_2$	12	0.0168	1	1
Se_4TiZr	12	0.2263	1	1
$I_2Se_2Yb_2$	12	0.0035	1	1
Mo_2Te_4	12	0.5138	1	1
$AgClO_4$	12	0.0642	1	1
$CrSe_2$	12	0.1362	1	2
O_2Pt	12	0.1315	1	2
$C_2Br_2La_2$	12	0.109	1	1

Table showing the first 50 lattice matching combinations with the smallest number of atoms in the supercell.

Lattice matching - minimal strain

Formula	N° atoms	strain	cell size 1	cell size 2
$Cl_2S_2Tl_2$	660	0.0003	56	54
Cu_2Te_2	180	0.0003	14	24
I_2Ni	345	0.0003	30	55
$GeTe_2$	345	0.0003	30	55
Au_2Br_2	394	0.0004	35	46
$K_2O_2Tl_2$	966	0.0005	80	81
C_2Li_2	220	0.0006	18	28
$As_2Co_2Li_2$	228	0.0006	14	24
CNRb	702	0.0006	77	80
Mg_6	240	0.0007	12	28
Cl_2La_2	622	0.0007	49	82
Ni_2Te_2	400	0.0008	30	55
$RhTe_2$	621	0.0008	52	103
In_2Se_2	684	0.0009	54	90
$I_2S_2Tb_2$	846	0.0009	60	81
$CdCl_2$	345	0.0009	30	55
DyI_2	603	0.001	60	81
Au_2Se_2	634	0.001	63	64
$Dy_2I_2S_2$	948	0.001	67	91
$Er_2I_2Se_2$	12	0.001	1	1
Au_2Br_2	360	0.001	32	42
I_2Mn	345	0.0011	30	55
Bi_2Se_2	660	0.0011	58	78
F_2Na	660	0.0011	56	108
In_2Se_2	622	0.0011	49	82
$Cl_2O_2Tm_2$	984	0.0011	56	108
$Cl_2Er_2H_2$	510	0.0012	30	55
$Ge_2Hf_2Te_2$	228	0.0012	14	24
CdClHO	724	0.0012	52	103
FeI_2	345	0.0012	30	55
Cl_4Mg_2	978	0.0012	107	56
$I_2N_2Zr_2$	930	0.0013	52	103
$Dy_2I_2S_2$	876	0.0013	62	84
I_2Zn	615	0.0013	56	93
$InSe_2$	345	0.0013	30	55
H_2Li_2Pt	783	0.0013	43	105
$F_2Se_2Y_2$	312	0.0013	24	28
Br_2Mg	345	0.0014	30	55
$Dy_2I_2S_2$	846	0.0014	60	81
I_2Zn	594	0.0014	54	90
Hg_3S_2	942	0.0015	112	54
Pb_2Se_2	876	0.0015	80	99
As_2Sn_2	622	0.0015	49	82
Ag_2Te_2	852	0.0015	68	111
$I_2Nd_2S_2$	966	0.0015	70	91
$Cl_2S_2Tl_2$	648	0.0015	55	53
Br_2Pr_2	768	0.0015	56	108
In_2Se_2	886	0.0015	87	91
CdClHO	732	0.0016	52	105
$Hf_2I_2N_2$	942	0.0016	52	105

Table showing the first 50 lattice matching combinations with the smallest strain needed to build the supercell.

In_2Se_3 (C2)

Structural and electronic properties

**Formula**	In_2Se_3
**Spacegroup**	C2
**Prototype**	In2Se3
**Parent 3D**	In_2Se_3
**Source DB**	ICSD
**DB ID**	602266
**DF2-C09 Binding energy [meV/ Å^2]**	16.09
**RVV10 Binding energy [meV/ Å^2]**	23.49
**Band gap (PBE) [eV]**	0.99

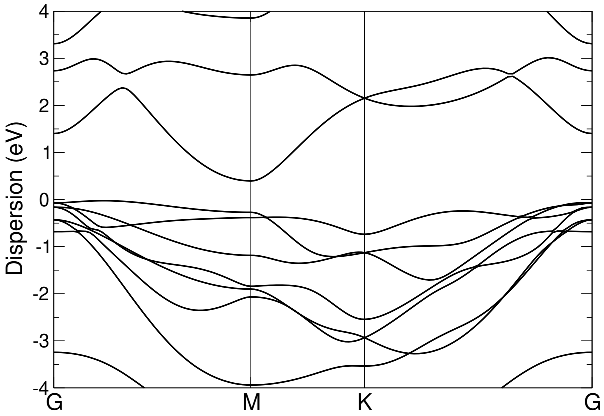

Band structure: Electronic band structure of In_2Se_3 (C2) in a representative window around the Fermi energy and along a high-symmetry path.

Optimized crystal structure

Structural parameters: relaxed cell (top) and atomic positions (bottom) of In_2Se_3 (C2) in Cartesian coordinates.

	x [Å]	y [Å]	z [Å]
$\mathbf{a_1}$	3.97052013	0.00000000	0.00000000
$\mathbf{a_2}$	−1.98526006	3.43857130	0.00000000
$\mathbf{a_3}$	0.00000000	0.00000000	26.38816686

		x [Å]	y [Å]	z [Å]
●	In	0.00000000	2.29238086	11.29089911
●	Se	1.98526006	1.14619043	9.91528256
●	In	1.98526006	1.14619043	15.09726775
●	Se	-0.00000000	-0.00000000	13.19408343
●	Se	0.00000000	2.29238086	16.47288430

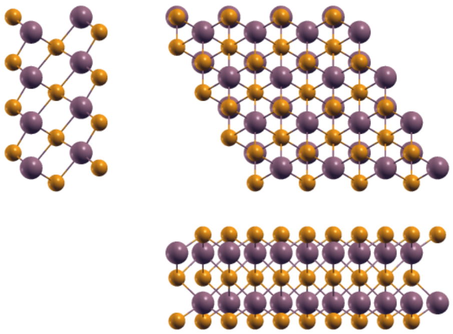

Orthographic projections: views of In_2Se_3 (C2) as seen from the x axis (left), the y axis (bottom) and the z axis (center).

Lattice matching - minimal size

Formula	N° atoms	strain	cell size 1	cell size 2
K	6	2.9205	1	1
Na	6	0.2628	1	1
GeTe	7	0.0091	1	1
As_2	7	0.2744	1	1
S_2	7	0.0076	1	1
CaCl	7	0.1311	1	1
$IrTe_2$	8	0.0082	1	1
Cl_2Zn	8	0.2728	1	1
$MoTe_2$	8	0.2661	1	1
$ReSe_2$	8	1.6002	1	1
CaI_2	8	0.4817	1	1
$HfTe_2$	8	0.0	1	1
Te_2V	8	0.2688	1	1
I_2Yb	8	0.4752	1	1
Br_2Co	8	0.2737	1	1
Ca_2N	8	0.2751	1	1
$AuTe_2$	8	0.0079	1	1
Cl_2Zn	8	0.1428	1	1
$PdTe_2$	8	0.0053	1	1
S_2Ti	8	1.5559	1	1
Te_2Zn	8	0.2658	1	1
Bi_2Pd	8	0.4427	1	1
Ba_2Hg	8	0.2079	1	1
Br_2Mn	8	0.2711	1	1
Cl_2Ni	8	0.2473	1	1
Cl_2Co	8	1.5533	1	1
$CrTe_2$	8	0.2554	1	1
PtS_2	8	0.2643	1	1
Br_2V	8	1.5942	1	1
Cl_2Fe	8	1.5481	1	1
CdClO	8	0.2681	1	1
Ba_2N	8	0.0015	1	1
Se_2Ti	8	0.2606	1	1
Br_2Ti	8	0.2546	1	1
Te_2Zr	8	0.0005	1	1
Te_2W	8	0.2663	1	1
$AsSe_2$	8	0.2492	1	1
I_2Tm	8	0.4787	1	1
I_2Pb	8	3.0139	1	1
OTl_2	8	0.2684	1	1
BiITe	8	0.4556	1	1
BrNZr	8	1.6278	1	1
$NbSe_2$	8	0.2478	1	1
Br_2Fe	8	0.2738	1	1
GeS_2	8	0.1231	1	1
$MnSe_2$	8	0.1311	1	1
Br_2Cr	8	0.255	1	1
Cl_2Zr	8	1.5509	1	1
Se_2Ta	8	0.2479	1	1
$NbSe_2$	8	0.2495	1	1

Table showing the first 50 lattice matching combinations with the smallest number of atoms in the supercell.

Lattice matching - minimal strain

Formula	N° atoms	strain	cell size 1	cell size 2
PtS_2	638	0.0	73	91
$Gd_2I_2S_2$	614	0.0	64	49
$HfTe_2$	8	0.0	1	1
$CrTe_2$	504	0.0001	57	73
$AsSe_2$	437	0.0001	49	64
H_2MgO_2	205	0.0001	16	25
$I_2S_2Sm_2$	543	0.0001	57	43
Cl_2Sc_2	577	0.0001	57	73
$Ge_2I_2La_2$	893	0.0001	91	73
Cl_2Fe	327	0.0002	36	49
ClH_3O	305	0.0002	36	25
$NbSe_2$	437	0.0002	49	64
Br_2V	386	0.0003	43	57
Br_2Ca_3Si	614	0.0003	64	49
$Er_2F_2Se_2$	11	0.0003	1	1
Ho_2S_2	620	0.0003	72	65
Ag_2	317	0.0003	49	36
I_2Yb	597	0.0003	81	64
Br_2Cr	504	0.0003	57	73
CdClO	705	0.0004	81	100
$I_2Pr_2S_2$	461	0.0004	49	36
$Br_2H_2Zr_2$	723	0.0005	57	73
Te_2Zr	8	0.0005	1	1
K	328	0.0005	57	43
CdH_2O_2	905	0.0005	81	100
Sn_2Te_2	244	0.0005	32	21
Ba_2Cu_2	661	0.0005	81	64
CoH_2O_2	490	0.0005	37	61
Br_2Zr_2	644	0.0005	64	81
Cl_2Zr_2	443	0.0005	43	57
OTl_2	705	0.0006	81	100
Ba_2Pt	353	0.0006	49	36
Cl_2Zr	327	0.0006	36	49
CaI_2	536	0.0006	73	57
NS_2Zr	729	0.0006	73	91
Br_2Ti	504	0.0006	57	73
$Br_2Hf_2N_2$	570	0.0006	54	50
Hg_3N_2	325	0.0006	49	16
Ga_2S_3	820	0.0007	73	91
$Cl_2Cr_2O_2$	836	0.0007	70	81
AsI_2La_2	905	0.0007	100	81
Pt_2Te_2	9	0.0007	1	1
Cd_2I_3	905	0.0007	100	81
CdO_2	327	0.0008	36	49
$HfLiS_2$	729	0.0008	73	91
GeI_2La_2	725	0.0008	81	64
S_2V	155	0.0008	16	25
I_2La_2Sb	500	0.0008	57	43
Br_2Hf_2	501	0.0008	49	64
$H_2I_2Sr_2$	613	0.0009	65	48

Table showing the first 50 lattice matching combinations with the smallest strain needed to build the supercell.

InOBr (Pmmn)

Structural and electronic properties

**Formula**	InOBr
**Spacegroup**	Pmmn
**Prototype**	FeOCl
**Parent 3D**	$In_2O_2Br_2$
**Source DB**	ICSD
**DB ID**	24059
**DF2-C09 Binding energy [meV/ Å^2]**	9.6
**RVV10 Binding energy [meV/ Å^2]**	17.3
**Band gap (PBE) [eV]**	2.34

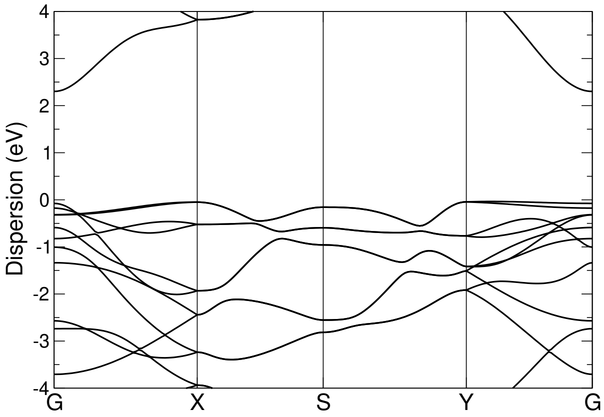

Band structure: Electronic band structure of InOBr (Pmmn) in a representative window around the Fermi energy and along a high-symmetry path.

Optimized crystal structure

Structural parameters: relaxed cell (top) and atomic positions (bottom) of InOBr (Pmmn) in Cartesian coordinates.

		x [Å]	y [Å]	z [Å]
$\mathbf{a_1}$		3.64237699	0.00000000	0.00000000
$\mathbf{a_2}$		0.00000000	4.13802081	0.00000000
$\mathbf{a_3}$		0.00000000	0.00000000	26.05067855
		x [Å]	y [Å]	z [Å]
●	In	1.82118850	2.06901040	12.02402668
●	Br	0.00000000	2.06901040	10.00972343
●	O	1.82118850	0.00000000	12.69162247
●	In	0.00000000	0.00000000	14.02664676
●	Br	1.82118850	0.00000000	16.04096781
●	O	0.00000000	2.06901040	13.35904929

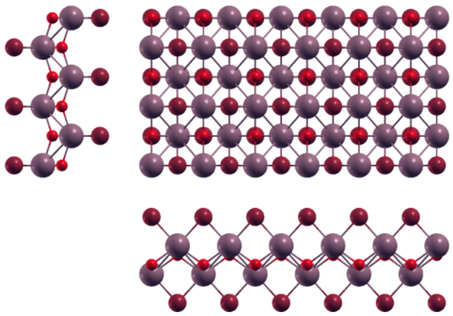

Orthographic projections: views of InOBr (Pmmn) as seen from the x axis (left), the y axis (bottom) and the z axis (center).

Lattice matching - minimal size

Formula	N° atoms	strain	cell size 1	cell size 2
K	7	0.3579	1	1
AgTl	8	0.0289	1	1
Ag_2	8	0.2009	1	1
CaCl	8	0.0407	1	1
Ba_2Pt	9	0.2005	1	1
Br_2Cu	9	0.466	1	1
Ca_2Si	9	0.2068	1	1
Cl_2Zn	9	0.2824	1	1
BiBrTe	9	0.5859	1	1
Ba_2Hg	9	0.0533	1	1
I_2Pb	9	0.203	1	1
GeS_2	9	0.2442	1	1
$MnSe_2$	9	0.0408	1	1
GdI_2	9	0.5946	1	1
CNNa	9	0.4448	1	1
F_2Ni	9	0.035	1	1
Ba_2Cd	9	0.3335	1	1
Cu_2I_2	10	0.0438	1	1
Cu_2Sr_2	10	0.589	1	1
Cl_2OOs	10	0.0323	1	1
Cu_2Te_2	10	0.1936	1	1
$AgCuTe_2$	10	0.0366	1	1
O_2Sn_2	10	0.0082	1	1
S_2Sn_2	10	0.3126	1	1
Cl_2OV	10	0.6326	1	1
Ge_2Te_2	10	0.3441	1	1
Fe_2Se_2	10	0.0353	1	1
Cl_2ORu	10	0.2699	1	1
As_2Co_2	10	0.0378	1	1
Cu_2Te_2	10	0.2864	1	1
O_2Pb_2	10	0.3038	1	1
$AgBrO_2$	10	0.108	1	1
Ge_2S_2	10	0.0199	1	1
As_4	10	0.4525	1	1
P_4	10	0.1175	1	1
Br_2OV	10	0.2558	1	1
Fe_2S_2	10	0.2578	1	1
Sb_2Te_2	10	0.205	1	1
Co_2S_2	10	0.0403	1	1
As_2Fe_2	10	0.2453	1	1
O_2Sn_2	10	0.0567	1	1
Cu_2Se_2	10	0.0347	1	1
Bi_2O_2	10	0.0422	1	1
$AgClO_2$	10	0.1026	1	1
La_2S_2	10	0.3458	1	1
PbS_2Sn	10	0.0641	1	1
$SbSe_2Tl$	10	0.7153	1	1
O_2Sn_2	10	0.0566	1	1
Co_2Se_2	10	0.0371	1	1
Ca_2Cl_2	10	0.0382	1	1

Table showing the first 50 lattice matching combinations with the smallest number of atoms in the supercell.

Lattice matching - minimal strain

Formula	N° atoms	strain	cell size 1	cell size 2
MoS_2	312	0.0001	28	48
S_2W	312	0.0002	28	48
$SiTe_2$	963	0.0003	100	121
MoS_2	312	0.0003	28	48
$NiTe_2$	963	0.0004	100	121
HN_3OZn	798	0.0005	51	82
O_2Sn_2	770	0.0005	71	86
Se_2Zr	963	0.0007	100	121
O_2Sn_2	770	0.0007	71	86
S_2V	312	0.0007	28	48
KNO_3	817	0.0007	102	41
CS_2Ta_2	716	0.0007	51	82
Cl_2Ti	552	0.0008	51	82
O_2Sn_2	358	0.0008	33	40
$Br_2Dy_2S_2$	822	0.0009	81	56
P_2	470	0.0009	51	82
P_4	976	0.0009	98	97
O_2Sn_2	358	0.0009	33	40
$PbTe_2$	504	0.001	56	56
$Gd_2I_2S_2$	630	0.0011	63	42
$Er_2I_2S_2$	726	0.0011	72	49
HgO	814	0.0012	92	131
$MnNaTe_2$	560	0.0012	56	56
Se_2Zr	894	0.0013	93	112
I_2V	894	0.0013	93	112
Cl_4Mn	727	0.0013	77	53
H_2MgO_2	585	0.0013	40	69
CNb_2S_2	716	0.0014	51	82
N_2Re	669	0.0014	54	115
PTe_2Zr_2	955	0.0014	80	95
$Ge_2Se_2Zr_2$	90	0.0014	7	8
Ca_2Cl_2	74	0.0014	7	8
$AgClO_4$	276	0.0014	28	18
BH_4Li	996	0.0014	82	84
SSb_2Te_2	616	0.0014	56	56
H_2NiO_2	408	0.0015	28	48
STl_2	750	0.0015	84	82
Ga_2Se_2	860	0.0015	80	95
Cl_2La_2	860	0.0015	84	89
Bi_2	702	0.0015	89	84
$CeLi_2P_2$	912	0.0015	82	84
$SiTe_2$	894	0.0015	93	112
BrCdI	504	0.0015	56	56
Cl_2NSc_2	615	0.0015	45	69
$I_2S_2Tb_2$	630	0.0015	63	42
Br_2Gd_2Ge	616	0.0015	56	56
Cu_2I_2	560	0.0015	56	56
NbS_2	477	0.0015	45	69
H_2MgO_2	408	0.0015	28	48
Sn_2Te_2	444	0.0016	50	36

Table showing the first 50 lattice matching combinations with the smallest strain needed to build the supercell.

InSe (C2/m)

Structural and electronic properties

**Formula**	InSe
**Spacegroup**	C2/m
**Prototype**	InSe
**Parent 3D**	In_2Se_2
**Source DB**	ICSD
**DB ID**	32714
**DF2-C09 Binding energy [meV/ Å^2]**	32.7
**RVV10 Binding energy [meV/ Å^2]**	33.7
**Band gap (PBE) [eV]**	1.68

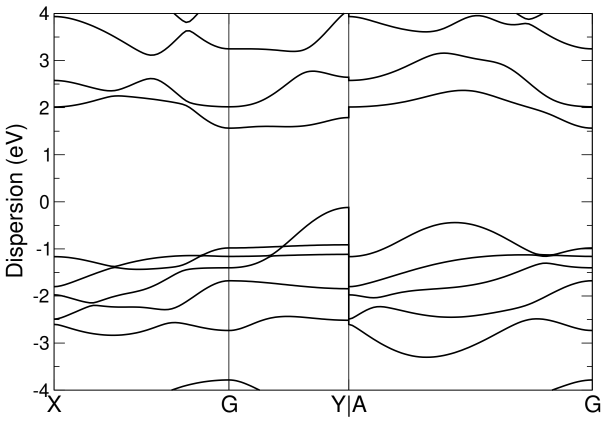

Band structure: Electronic band structure of InSe (C2/m) in a representative window around the Fermi energy and along a high-symmetry path.

Optimized crystal structure

Structural parameters: relaxed cell (top) and atomic positions (bottom) of InSe (C2/m) in Cartesian coordinates.

	x [Å]	y [Å]	z [Å]
$\mathbf{a_1}$	4.07548980	−0.00000245	0.00000000
$\mathbf{a_2}$	−2.03774839	5.57970901	0.00000000
$\mathbf{a_3}$	0.00000000	0.00000000	23.12995708

		x [Å]	y [Å]	z [Å]
●	Se	2.03775669	0.91972294	9.84821982
●	In	2.03773239	2.71705145	11.90501800
●	In	2.03775148	5.43330932	11.22493662
●	Se	-0.00001255	1.65095750	13.28173972

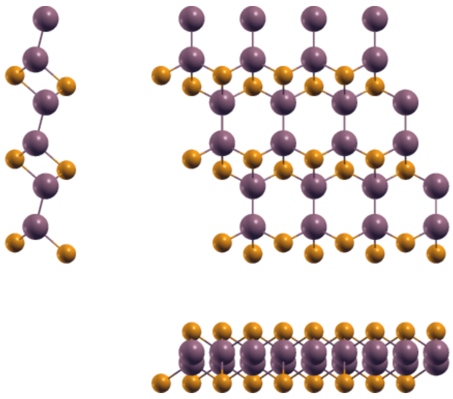

Orthographic projections: views of InSe (C2/m) as seen from the x axis (left), the y axis (bottom) and the z axis (center).

Lattice matching - minimal size

Formula	N° atoms	strain	cell size 1	cell size 2
K	5	0.0933	1	1
Tl	6	0.0839	1	2
In	6	0.0845	1	2
CaI_2	7	0.0928	1	1
Li_2Tl_2	8	0.6158	1	1
Cu_2O_2	8	0.099	1	1
LiO	8	0.0836	1	2
Au_2I_2	8	0.1005	1	1
P_2	8	0.0835	1	2
$SbSe_2Tl$	8	0.0857	1	1
I_2La_2Te	9	0.0931	1	1
S_2V	10	0.0841	1	2
MoS_2	10	0.0841	1	2
Cl_2Mn	10	0.2377	1	2
$MoSe_2$	10	0.0838	1	2
$CuTe_2$	10	0.5476	1	2
ReS_2	10	0.404	1	2
Cl_2Ti	10	0.0836	1	2
S_2Ti	10	0.2471	1	2
$I_2Se_2Tb_2$	10	0.1016	1	1
$Gd_2I_2Se_2$	10	0.1033	1	1
S_2W	10	0.084	1	2
$Gd_2I_2S_2$	10	0.0956	1	1
Cl_2Ni	10	0.2568	1	2
Cl_2Co	10	0.2466	1	2
Pd_2S_4	10	0.2076	1	1
$CrTe_2$	10	0.2657	1	2
NbS_2	10	0.2342	1	2
ClNZr	10	0.2514	1	2
Cl_2Fe	10	0.2456	1	2
S_2Ta	10	0.0841	1	2
Se_2V	10	0.084	1	2
$I_2S_2Tb_2$	10	0.0941	1	1
Br_2Ti	10	0.2648	1	2
$AsSe_2$	10	0.2589	1	2
$Er_2I_2Se_2$	10	0.098	1	1
$I_2Se_2Tm_2$	10	0.0968	1	1
Cl_2V	10	0.0837	1	2
CdO_2	10	0.4699	1	2
AuI_4Li	10	0.2105	1	1
BrNZr	10	0.2608	1	2
$NbSe_2$	10	0.2574	1	2
O_2Zn	10	0.3535	1	2
Br_2Cr	10	0.2653	1	2
Cl_2Zr	10	0.2461	1	2
Se_2Ta	10	0.2575	1	2
$NbSe_2$	10	0.2593	1	2
MoS_2	10	0.084	1	2
$I_2S_2Sm_2$	10	0.0933	1	1
$CrSe_2$	10	0.0838	1	2

Table showing the first 50 lattice matching combinations with the smallest number of atoms in the supercell.

Lattice matching - minimal strain

Formula	N° atoms	strain	cell size 1	cell size 2
$CrSe_2$	220	0.0004	19	48
$Br_2O_2Sc_2$	476	0.0004	35	56
Cl_2OOs	172	0.0005	16	27
Bi_2	274	0.0006	40	57
Br_2Ca	331	0.0006	40	57
Cl_4Pd_2	870	0.0006	96	81
Se_4TiZr	708	0.0006	72	70
Sb_2Te_2	752	0.0007	85	103
$Cl_2Rh_2Te_2$	914	0.0009	95	89
Li_2Tl_2	916	0.0009	113	116
Au_2Se_2	780	0.0011	99	96
HgI_2	326	0.0011	44	50
Bi_2SeTe_2	399	0.0011	36	51
$AgNO_3$	859	0.0011	96	95
$Cl_2S_2Tl_2$	838	0.0011	88	81
$Ba_2H_2I_2$	650	0.0012	62	67
Li_2Tl_2	600	0.0012	74	76
$Cl_2Fe_2O_2$	218	0.0012	14	27
CNNa	459	0.0013	57	77
$I_2N_2Zr_2$	918	0.0013	60	113
C_2Li_2	328	0.0013	33	49
$RhTe_2$	579	0.0013	60	113
Ge_2S_2	820	0.0013	85	120
I_2Pr	331	0.0014	40	57
Sn_2Te_2	560	0.0014	67	73
Bi_2	246	0.0014	36	51
$K_2O_2Tl_2$	692	0.0014	71	68
$Ag_2K_2Te_2$	420	0.0014	42	42
Sb_2Te_3	399	0.0014	36	51
Cl_2V	650	0.0014	56	142
Te_4W_2	822	0.0014	81	83
$Ce_2I_2S_2$	718	0.0014	64	77
$F_2Lu_2Se_2$	94	0.0014	7	11
Cl_2V	220	0.0014	19	48
$Br_2H_2Zr_2$	606	0.0014	36	77
Te_2Zn	712	0.0014	70	144
BiClTe	331	0.0014	40	57
CNRb	627	0.0014	90	89
Cl_2V	128	0.0015	11	28
$Ag_2K_2Se_2$	476	0.0015	44	50
N_2Re	560	0.0015	41	132
$Er_2I_2Se_2$	906	0.0015	93	89
NaO_4	679	0.0015	76	75
Cl_4Pd_2	860	0.0015	95	80
$AgTe_2$	301	0.0015	28	63
CdI_2	365	0.0015	44	63
$AgNO_3$	679	0.0016	76	75
$Ho_2I_2Se_2$	886	0.0016	91	87
FHOZn	156	0.0016	11	28
$Er_2I_2Se_2$	702	0.0016	72	69

Table showing the first 50 lattice matching combinations with the smallest strain needed to build the supercell.

InSe (P-6m2)

Structural and electronic properties

**Formula**	InSe
**Spacegroup**	P-6m2
**Prototype**	GaS
**Parent 3D**	In_4Se_4
**Source DB**	COD
**DB ID**	9008967
**DF2-C09 Binding energy [meV/ Å^2]**	14.91
**RVV10 Binding energy [meV/ Å^2]**	20.82
**Band gap (PBE) [eV]**	1.48

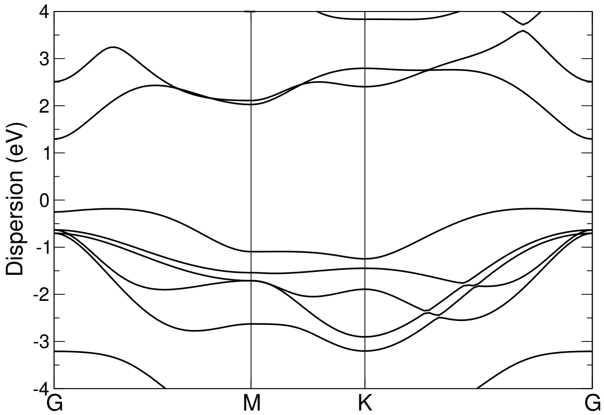

Band structure: Electronic band structure of InSe (P-6m2) in a representative window around the Fermi energy and along a high-symmetry path.

Optimized crystal structure

Structural parameters: relaxed cell (top) and atomic positions (bottom) of InSe (P-6m2) in Cartesian coordinates.

		x [Å]	y [Å]	z [Å]
$\mathbf{a_1}$		4.06593457	0.00000000	0.00000000
$\mathbf{a_2}$		−2.03296728	3.52120263	0.00000000
$\mathbf{a_3}$		0.00000000	0.00000000	25.37667800
		x [Å]	y [Å]	z [Å]
●	In	2.03296728	1.17373421	14.09907617
●	In	2.03296728	1.17373421	11.27760183
●	Se	-0.00000000	2.34746842	15.38725313
●	Se	-0.00000000	2.34746842	9.98942487

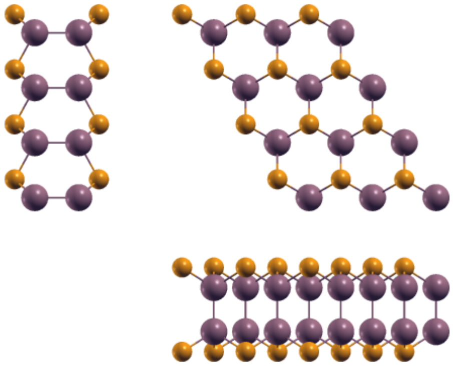

Orthographic projections: views of InSe (P-6m2) as seen from the x axis (left), the y axis (bottom) and the z axis (center).

Lattice matching - minimal size

Formula	N° atoms	strain	cell size 1	cell size 2
K	5	0.4765	1	1
Na	5	0.2476	1	1
In	5	4.8687	1	1
As_2	6	0.2583	1	1
Sb_2	6	0.0044	1	1
CaCl	6	0.1236	1	1
Cl_2Zn	7	0.2569	1	1
$MoTe_2$	7	0.2506	1	1
PSn_2	7	0.271	1	1
HfS_2	7	0.2621	1	1
CaI_2	7	0.4534	1	1
Te_2V	7	1.6405	1	1
$CuTe_2$	7	0.2616	1	1
S_2Zr	7	0.2699	1	1
Ca_2Si	7	2.9013	1	1
I_2Yb	7	0.4472	1	1
Br_2Co	7	0.2577	1	1
Ca_2N	7	0.259	1	1
$AuTe_2$	7	0.0034	1	1
Cl_2Zn	7	0.1336	1	1
$PdTe_2$	7	0.0058	1	1
I_2Zn	7	0.0011	1	1
Te_2Zn	7	0.2504	1	1
GeI_2	7	0.0083	1	1
Br_2Mn	7	0.2552	1	1
Cl_2Ni	7	1.5255	1	1
PtS_2	7	0.249	1	1
$CoTe_2$	7	0.2626	1	1
CdClO	7	1.6368	1	1
Se_2Ti	7	1.5971	1	1
Br_2Ti	7	1.5652	1	1
Te_2W	7	1.6272	1	1
I_2Tm	7	0.4505	1	1
S_2Sn	7	0.2702	1	1
$SnTe_2$	7	0.006	1	1
I_2Pb	7	0.4949	1	1
OTl_2	7	1.6382	1	1
BrNZr	7	1.5453	1	1
$NbSe_2$	7	1.5285	1	1
Br_2Fe	7	0.2578	1	1
GeS_2	7	0.117	1	1
$TaTe_2$	7	0.2757	1	1
$MnSe_2$	7	0.1236	1	1
DyI_2	7	0.4604	1	1
Br_2Ni	7	0.2649	1	1
CeI_2	7	0.1541	1	1
Se_2Ta	7	1.5291	1	1
$NbTe_2$	7	0.2696	1	1
Se_2Yb	7	0.0086	1	1
$NbSe_2$	7	1.5378	1	1

Table showing the first 50 lattice matching combinations with the smallest number of atoms in the supercell.

Lattice matching - minimal strain

Formula	N° atoms	strain	cell size 1	cell size 2
Ga_2S_2	656	0.0	73	91
$Br_2N_2Zr_2$	924	0.0001	81	100
Br_3Cs	136	0.0001	25	9
Br_2Ca_3Si	802	0.0001	91	73
$I_2S_2Tb_2$	886	0.0001	100	81
CBr_2Lu_2	824	0.0001	81	100
Br_2Mn	447	0.0002	57	73
$I_2S_2Sm_2$	708	0.0002	81	64
K	388	0.0002	81	64
Ca_2N	499	0.0002	64	81
I_2Pb	403	0.0002	64	49
PtS_2	388	0.0002	49	64
$Gd_2I_2S_2$	802	0.0002	91	73
BrNZr	291	0.0002	36	49
KS_2Ti	520	0.0002	57	73
Br_2Zr_2	400	0.0002	43	57
I_4Sr_2	304	0.0003	49	18
PTe_2Ti_2	747	0.0003	73	91
In	277	0.0003	49	81
DyI_2	643	0.0003	100	81
Br_2Ni	565	0.0004	73	91
$Br_2Hf_2N_2$	742	0.0004	64	81
Cl_2Hf_2	244	0.0004	25	36
F_4Pb	500	0.0004	65	48
Cl_2Mg	565	0.0005	73	91
I_2La_2Sb	644	0.0005	81	64
$Br_2Er_2S_2$	48	0.0005	6	4
$HfLiS_2$	452	0.0006	49	64
N_2Re	393	0.0006	39	79
$Cu_2K_2Te_2$	958	0.0006	118	81
Se_2Ti	343	0.0006	43	57
Br_2Hf_2	340	0.0006	36	49
Nd	25	0.0006	4	9
IKO_3	276	0.0006	49	16
$I_2Nd_2S_2$	634	0.0007	73	57
As_2O_3	109	0.0007	16	9
As_2	418	0.0007	64	81
$Cl_2H_2Lu_2$	838	0.0008	73	91
NS_2Zr	452	0.0008	49	64
CCl_2Lu_2	661	0.0008	64	81
Te_2Zn	388	0.0008	49	64
$Cl_2O_2Ti_2$	338	0.0009	32	35
Ga_2S_3	516	0.0009	49	64
$Ho_2I_2Se_2$	684	0.0009	90	54
In	209	0.0009	37	61
$Cl_2N_2Zr_2$	742	0.001	64	81
$Er_2I_2Se_2$	684	0.001	90	54
$Dy_2I_2S_2$	886	0.001	100	81
$Ce_2I_2S_2$	486	0.001	57	43
Ca_2Si	357	0.001	57	43

Table showing the first 50 lattice matching combinations with the smallest strain needed to build the supercell.

$K_2Cd_2As_2$ (P4/nmm)

Structural and electronic properties

**Formula**	$K_2Cd_2As_2$
**Spacegroup**	P4/nmm
**Prototype**	PbClF
**Parent 3D**	$As_2Cd_2K_2$
**Source DB**	MPDS
**DB ID**	S1713895
**DF2-C09 Binding energy [meV/ Å^2]**	24.61
**RVV10 Binding energy [meV/ Å^2]**	N/A
**Band gap (PBE) [eV]**	0.21

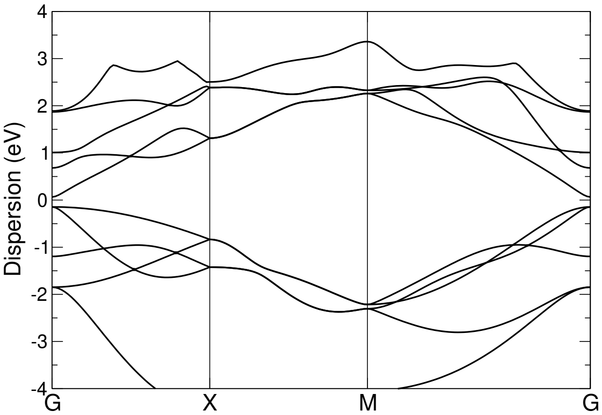

Band structure: Electronic band structure of $K_2Cd_2As_2$ (P4/nmm) in a representative window around the Fermi energy and along a high-symmetry path.

Optimized crystal structure

Structural parameters: relaxed cell (top) and atomic positions (bottom) of $K_2Cd_2As_2$ (P4/nmm) in Cartesian coordinates.

		x [Å]	y [Å]	z [Å]
$\mathbf{a_1}$		4.54114153	0.00000000	0.00000000
$\mathbf{a_2}$		0.00000000	4.54114153	0.00000000
$\mathbf{a_3}$		0.00000000	0.00000000	23.01281737
		x [Å]	y [Å]	z [Å]
●	K	-1.13528538	-3.40585615	2.81601185
●	As	1.13528538	-1.13528538	1.69585829
●	K	1.13528538	-1.13528538	-2.81601185
●	Cd	-1.13528538	-1.13528538	0.00000000
●	Cd	1.13528538	-3.40585615	0.00000000
●	As	-1.13528538	-3.40585615	-1.69585829

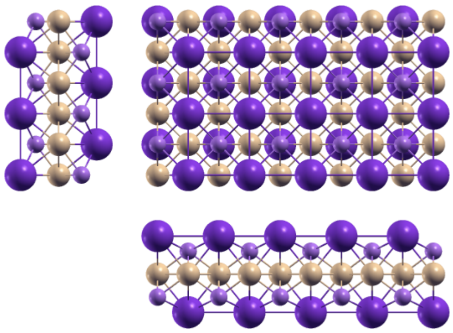

Orthographic projections: views of $K_2Cd_2As_2$ (P4/nmm) as seen from the x axis (left), the y axis (bottom) and the z axis (center).

Lattice matching - minimal size

Formula	N° atoms	strain	cell size 1	cell size 2
Bi_2	8	0.1084	1	1
AgTl	8	0.2075	1	1
Sm	8	0.1465	1	2
Br_2Zn	9	0.1096	1	1
$AsSn_2$	9	0.1111	1	1
$SiTe_2$	9	0.1088	1	1
HgI_2	9	0.0066	1	1
Te_2Ti	9	0.1095	1	1
$RhTe_2$	9	0.1105	1	1
CNRb	9	0.0475	1	1
CKN	9	0.3569	1	1
$NiTe_2$	9	0.1089	1	1
Cl_2Cu	9	0.1391	1	1
Se_2Zr	9	0.1087	1	1
$PtSe_2$	9	0.111	1	1
CeI_2	9	0.5687	1	1
CNNa	9	0.5178	1	1
I_2La	9	0.5878	1	1
F_2Zn	9	0.5594	1	1
$HfSe_2$	9	0.1095	1	1
Bi_2Te_2	10	0.1345	1	1
Li_2Tl_2	10	0.1562	1	1
Cu_2I_2	10	0.2201	1	1
CdClHO	10	0.1114	1	1
Ag_2Br_2	10	0.5894	1	1
CdClHO	10	0.1107	1	1
S_2Sn_2	10	0.2232	1	1
Cl_2Y_2	10	0.1099	1	1
Ag_2	10	0.9703	1	2
As_2Ir_2	10	0.5966	1	1
O_2Pb_2	10	0.2162	1	1
CaClHO	10	0.109	1	1
Au_2I_2	10	0.0219	1	1
Ge_2Se_2	10	0.2116	1	1
Bi_2O_2	10	0.2177	1	1
$AgClO_2$	10	0.116	1	1
Ni_2Se_2	10	0.568	1	1
As_2Rh_2	10	0.5886	1	1
Ag_2I_2	10	0.0056	1	1
Br_2CsF	10	0.0077	1	1
Ga_2Se_2	10	0.11	1	1
Sn_2Te_2	10	0.0028	1	1
As_2O_3	11	0.1965	1	1
Sb_2Te_3	11	0.1088	1	1
NaO_4	11	0.1663	1	1
$AgNO_3$	11	0.1672	1	1
ClH_3O	11	0.132	1	1
Bi_2SeTe_2	11	0.1085	1	1
$Ba_2H_2I_2$	12	0.0057	1	1
Ba_2Pt	12	0.969	1	2

Table showing the first 50 lattice matching combinations with the smallest number of atoms in the supercell.

Lattice matching - minimal strain

Formula	N° atoms	strain	cell size 1	cell size 2
Ca_2Cl_2	196	0.0	16	25
$Ge_2Se_2Zr_2$	246	0.0	16	25
Cu_2I_2	896	0.0	82	101
$Br_2O_2Y_2$	876	0.0	61	85
Cu_2S_2	706	0.0001	61	85
$Br_2Dy_2O_2$	876	0.0001	61	85
I_2La_2	548	0.0002	48	65
$I_2Nd_2O_2$	870	0.0002	64	81
$Ge_2Mn_2Sr_2$	678	0.0003	49	64
$I_2N_2Ti_2$	726	0.0003	49	72
HgO	248	0.0003	25	49
GeS_2	639	0.0004	58	97
$Ca_2H_2I_2$	690	0.0004	50	65
F_2Tl_2	706	0.0005	61	85
Tl	427	0.0005	53	109
As_2Fe_2	736	0.0005	58	97
Se_2Ta	237	0.0005	20	39
As_2Ir_2	550	0.0005	49	64
AsKSn	483	0.0005	48	65
Br_2Ca_3Si	96	0.0005	9	7
Cu_2I_2	886	0.0006	81	100
$Bi_2Cl_2O_2$	510	0.0006	36	49
P_2Rh_2	706	0.0006	61	85
O_2Sn_2	706	0.0006	61	85
$CuTe_2$	411	0.0006	36	65
Ni_2Se_2	412	0.0007	36	49
$AgTe_2$	594	0.0007	49	100
As_2Co_2	196	0.0007	16	25
$Br_2Ho_2S_2$	738	0.0007	63	60
$Cl_2H_2Sc_2$	354	0.0007	20	39
As_2Sn_2	958	0.0007	81	118
O_2Pt	714	0.0008	54	130
Cu_2F_4	510	0.0008	49	36
Mg_4	754	0.0008	53	109
$Ca_2Cl_2F_2$	366	0.0008	25	36
CeI_2	363	0.0009	36	49
F_2Ni	942	0.0009	89	136
$Br_2Eu_2O_2$	510	0.001	36	49
CrI_2	852	0.001	79	126
O_4PSn	12	0.001	1	1
$Br_2S_2Yb_2$	660	0.001	56	54
$Hf_2Se_2Si_2$	930	0.001	58	97
$AuTe_2$	840	0.001	81	118
O_2Pt	633	0.001	48	115
$I_2O_2Pr_2$	882	0.0011	65	82
Br_2Mg	852	0.0011	79	126
$Cl_2F_2Pb_2$	870	0.0011	64	81
FeI_2	852	0.0011	79	126
$SbSe_2Tl$	438	0.0011	45	42
H_2Na_2Pd	833	0.0011	58	97

Table showing the first 50 lattice matching combinations with the smallest strain needed to build the supercell.

KAgSe (P4/nmm)

Structural and electronic properties

**Formula**	KAgSe
**Spacegroup**	P4/nmm
**Prototype**	PbClF
**Parent 3D**	$K_2Ag_2Se_2$
**Source DB**	COD
**DB ID**	1509424
**DF2-C09 Binding energy [meV/ Å^2]**	24.88
**RVV10 Binding energy [meV/ Å^2]**	31.9
**Band gap (PBE) [eV]**	0.56

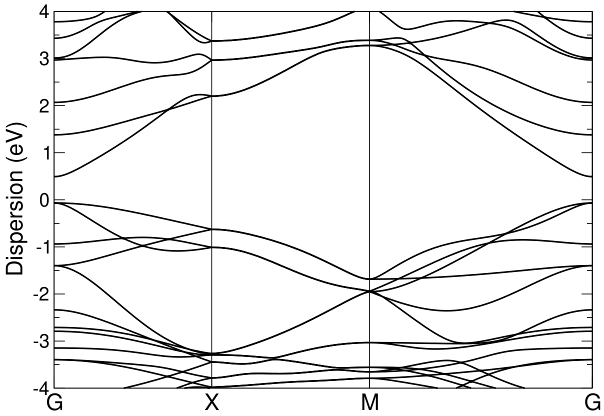

Band structure: Electronic band structure of KAgSe (P4/nmm) in a representative window around the Fermi energy and along a high-symmetry path.

Optimized crystal structure

Structural parameters: relaxed cell (top) and atomic positions (bottom) of KAgSe (P4/nmm) in Cartesian coordinates.

	x [Å]	y [Å]	z [Å]
$\mathbf{a_1}$	4.46279445	0.00000000	0.00000000
$\mathbf{a_2}$	0.00000000	4.46279445	0.00000000
$\mathbf{a_3}$	0.00000000	0.00000000	25.19012658

	x [Å]	y [Å]	z [Å]
K	2.23139723	0.00000000	15.25664379
Se	0.00000000	2.23139723	14.32420405
K	0.00000000	2.23139723	9.93348279
Ag	0.00000000	0.00000000	12.59506329
Ag	2.23139723	2.23139723	12.59506329
Se	2.23139723	0.00000000	10.86592254

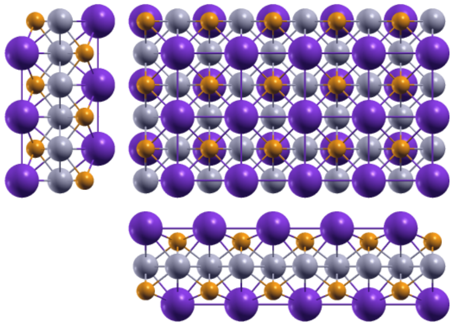

Orthographic projections: views of KAgSe (P4/nmm) as seen from the x axis (left), the y axis (bottom) and the z axis (center).

Lattice matching - minimal size

Formula	N° atoms	strain	cell size 1	cell size 2
InSe	8	0.109	1	1
K	8	0.9833	1	2
AgTl	8	0.2167	1	1
Nd	9	0.1324	1	3
PSn_2	9	0.1101	1	1
$AsSn_2$	9	0.1093	1	1
I_2Pr	9	0.5954	1	1
S_2Zr	9	0.1103	1	1
Ca_2Si	9	0.1311	1	1
HgI_2	9	0.0015	1	1
$RhTe_2$	9	0.1088	1	1
$CoTe_2$	9	0.1115	1	1
CKN	9	0.3734	1	1
I_2Nd	9	0.5983	1	1
Cl_2Cu	9	0.1418	1	1
S_2Sn	9	0.1102	1	1
$PtSe_2$	9	0.1092	1	1
Br_2Ni	9	0.1111	1	1
CeI_2	9	0.593	1	1
$NbTe_2$	9	0.1103	1	1
Cl_2Mg	9	0.1111	1	1
I_2La	9	0.6129	1	1
F_2Zn	9	0.5834	1	1
Bi_2Te_2	10	0.1412	1	1
Li_2Tl_2	10	0.1649	1	1
Ca_2Cl_2	10	0.5591	1	1
CdClHO	10	0.1096	1	1
Ir_2P_2	10	0.5977	1	1
Ag_2Br_2	10	0.2116	1	1
CdClHO	10	0.1089	1	1
$AsLi_3$	10	0.109	1	1
Ge_2Te_2	10	0.0188	1	1
As_2Ir_2	10	0.2142	1	1
Ga_2S_2	10	0.1114	1	1
Au_2I_2	10	0.0177	1	1
Sb_2Te_2	10	0.1301	1	1
Ge_2Se_2	10	0.2211	1	1
Ag_2Te_2	10	0.5811	1	1
As_2Ru_2	10	0.5597	1	1
La_2S_2	10	0.0179	1	1
Ni_2Se_2	10	0.5923	1	1
As_2Rh_2	10	0.2113	1	1
Ag_2I_2	10	0.0026	1	1
Se_2Sn_2	10	0.0145	1	1
Sn_2Te_2	10	0.0106	1	1
Ga_2S_2	10	0.1112	1	1
As_2O_3	11	0.7803	1	1
F_4Pb	11	0.0059	1	1
As_2Li_2Nd	11	0.1088	1	1
Bi_2Se_2Te	11	0.1086	1	1

Table showing the first 50 lattice matching combinations with the smallest number of atoms in the supercell.

Lattice matching - minimal strain

Formula	N° atoms	strain	cell size 1	cell size 2
Mg_3	594	0.0	49	100
I_2Nd	495	0.0	50	65
$Br_2O_2Sm_2$	690	0.0001	50	65
I_3Sn	814	0.0001	97	58
I_2Pr	486	0.0001	49	64
ClNZr	237	0.0002	20	39
Ir_2P_2	560	0.0002	50	65
BH_4Li	678	0.0002	48	65
$Ge_2Mn_2Sr_2$	882	0.0003	65	82
F_2Zn	849	0.0003	85	113
$Br_2Er_2O_2$	510	0.0004	36	49
$Ca_2Cl_2F_2$	876	0.0004	61	85
$SnTe_2$	483	0.0004	48	65
$Ge_2Mn_2Sr_2$	870	0.0005	64	81
$H_2Li_2O_2$	246	0.0005	16	25
$Br_2Ca_2H_2$	510	0.0005	36	49
$I_2O_2Yb_2$	678	0.0005	49	64
$Br_2Eu_2O_2$	678	0.0005	49	64
As_2Ir_2	718	0.0005	65	82
$CeLi_2P_2$	613	0.0006	48	65
$Br_2O_2Pr_2$	870	0.0006	64	81
CeI_2	486	0.0006	49	64
$AgTe_2$	297	0.0006	25	49
Te_2Zr	840	0.0006	81	118
Te_2Zn	411	0.0007	36	65
$Br_2H_2Zr_2$	354	0.0007	20	39
$Ca_2Mn_2Si_2$	510	0.0007	36	49
$HfTe_2$	840	0.0007	81	118
CoH_2O_2	974	0.0008	54	130
Mo_2Te_4	798	0.0008	70	63
Ni_2Se_2	550	0.0009	49	64
Cl_2Zn	258	0.0009	25	36
Ir_2P_2	550	0.0009	49	64
$Br_2Ho_2O_2$	510	0.0009	36	49
Br_2HLa	548	0.0009	48	65
C_2Li_2	784	0.001	70	91
In	33	0.001	4	9
Br_2Cu_2	962	0.001	85	113
CoH_2O_2	863	0.001	48	115
Se_2Zr	852	0.001	79	126
$Br_2O_2Sm_2$	678	0.001	49	64
$I_2O_2Tm_2$	690	0.001	50	65
Ag_2Br_2	708	0.0011	64	81
CCl_2Sc_2	315	0.0011	20	39
$Cl_2O_2V_2$	972	0.0011	61	101
KNO_3	684	0.0011	79	42
$SiTe_2$	852	0.0011	79	126
I_2Nd	486	0.0011	49	64
H_2Na_2Pd	920	0.0011	65	106
ReS_2	633	0.0012	48	115

Table showing the first 50 lattice matching combinations with the smallest strain needed to build the supercell.

$KTlCl_4$ (P-4)

Structural and electronic properties

**Formula**	$KTlCl_4$
**Spacegroup**	P-4
**Prototype**	CaWO4
**Parent 3D**	$K_2Tl_2Cl_8$
**Source DB**	COD
**DB ID**	1527421
**DF2-C09 Binding energy [meV/ Å^2]**	N/A
**RVV10 Binding energy [meV/ Å^2]**	26.76
**Band gap (PBE) [eV]**	2.84

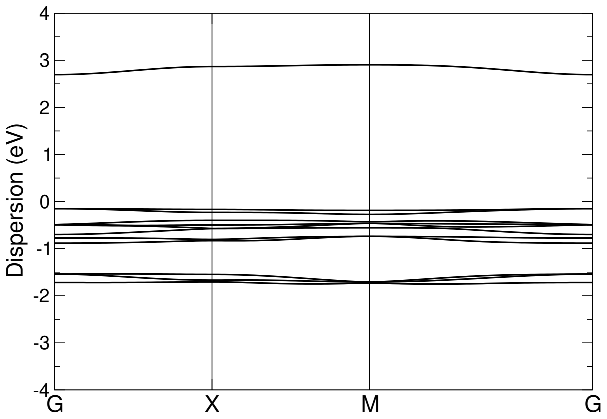

Band structure: Electronic band structure of $KTlCl_4$ (P-4) in a representative window around the Fermi energy and along a high-symmetry path.

Optimized crystal structure

Structural parameters: relaxed cell (top) and atomic positions (bottom) of $KTlCl_4$ (P-4) in Cartesian coordinates.

		x [Å]	y [Å]	z [Å]
$\mathbf{a_1}$		6.57732732	0.00000000	0.00000000
$\mathbf{a_2}$		0.00000000	6.57732732	0.00000000
$\mathbf{a_3}$		0.00000000	0.00000000	22.77898021
		x [Å]	y [Å]	z [Å]
●	Tl	3.28866366	3.28866366	11.38949011
●	Cl	5.06491558	2.33330678	12.82723796
●	Cl	4.24402054	5.06491558	9.95174225
●	K	0.00000000	0.00000000	11.38949011
●	Cl	1.51241174	4.24402054	12.82723796
●	Cl	2.33330678	1.51241174	9.95174225

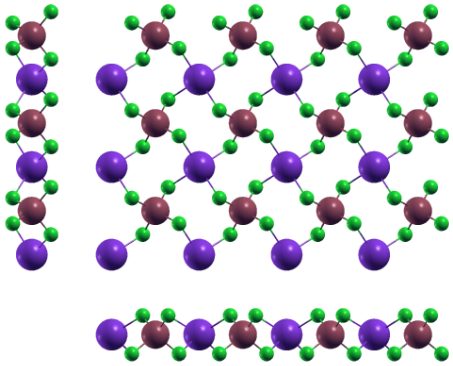

Orthographic projections: views of $KTlCl_4$ (P-4) as seen from the x axis (left), the y axis (bottom) and the z axis (center).

Lattice matching - minimal size

Formula	N° atoms	strain	cell size 1	cell size 2
Cl_2Rb_2	10	0.0087	1	1
AsSb	10	0.1491	1	2
GeTe	10	0.1517	1	2
I_3Sn	10	0.5986	1	1
Hg_3N_2	11	0.1348	1	1
IKO_3	11	0.1431	1	1
Hg_3S_2	11	0.1702	1	1
$BrKO_3$	11	0.1384	1	1
$CdCl_2$	12	0.1513	1	2
Br_2Zn	12	0.1455	1	2
$InSe_2$	12	0.1514	1	2
$AsSn_2$	12	0.1434	1	2
$SiTe_2$	12	0.1469	1	2
I_2Mn	12	0.1513	1	2
Hg_4O_2	12	0.2314	1	1
$Cl_2Rh_2Te_2$	12	0.1463	1	1
FeI_2	12	0.1503	1	2
I_2Ni	12	0.1509	1	2
Bi_2	12	0.1648	1	3
Te_2Ti	12	0.1456	1	2
$RhTe_2$	12	0.1442	1	2
Ag_2	12	0.7841	1	3
Cl_4Mg_2	12	0.1196	1	1
$NiTe_2$	12	0.1466	1	2
Cl_2Cu	12	0.1883	1	2
I_2V	12	0.1473	1	2
Se_2Zr	12	0.147	1	2
$PtSe_2$	12	0.1436	1	2
Br_2Mg	12	0.1503	1	2
PbTe	12	0.1605	1	3
$HfSe_2$	12	0.1456	1	2
H_2Si_2	14	0.1516	1	2
HgO	14	0.0061	1	4
CdClHO	14	0.144	1	2
Cl_2Y_2	14	0.1451	1	2
Ga_2Se_2	14	0.1479	1	2
P_4	14	0.1662	1	2
As_2	14	0.1498	1	4
CaClHO	14	0.1464	1	2
Ni_2Te_2	14	0.1512	1	2
$AgClO_2$	14	0.1412	1	2
CdI_2	15	0.1623	1	3
Ba_2Pt	15	0.7831	1	3
Br_2Ca	15	0.1636	1	3
BiClTe	15	0.1627	1	3
$PbTe_2$	15	0.1504	1	3
$SnTe_2$	15	0.1446	1	3
GeI_2	15	0.1607	1	3
DyI_2	15	0.193	1	3
CdI_2	15	0.1618	1	3

Table showing the first 50 lattice matching combinations with the smallest number of atoms in the supercell.

Lattice matching - minimal strain

Formula	N° atoms	strain	cell size 1	cell size 2
$Hf_2Si_2Te_2$	636	0.0001	25	81
I_3Sn	560	0.0001	50	65
Fe_2SeTe	292	0.0001	16	49
$Br_2F_2Sr_2$	534	0.0002	25	64
$H_2I_2Yb_2$	204	0.0002	9	25
O_2Pb_2	560	0.0002	34	89
I_2La_2Te	974	0.0003	54	130
$F_2I_2Sm_2$	738	0.0004	34	89
$Br_2H_2Yb_2$	390	0.0004	16	49
$Br_2F_2Pb_2$	534	0.0004	25	64
$As_2Fe_2Li_2$	390	0.0004	16	49
$Eu_2F_2I_2$	738	0.0005	34	89
Br_2Hg_3	675	0.0005	80	39
$Br_2La_2O_2$	738	0.0006	34	89
$Br_2Nd_2O_2$	204	0.0006	9	25
$Br_2Lu_2O_2$	390	0.0006	16	49
Co_2Se_2	474	0.0007	25	81
Cr_2O_4	606	0.0007	20	81
$I_2O_2Y_2$	204	0.0008	9	25
O_2Zn	267	0.0008	12	65
$Ba_2H_2I_2$	894	0.0008	49	100
Cl_4Mn	395	0.0008	25	49
Br_2Cu	465	0.0009	30	95
I_2La	129	0.0009	9	25
$Ge_2Te_2Zr_2$	390	0.0009	16	49
Li_2Tl_2	276	0.001	20	39
Bi_2O_2	560	0.0011	34	89
Br_2CsF	694	0.0011	49	100
Cu_2Se_2	766	0.0011	41	130
As_2Rh_2	154	0.0011	9	25
$I_2S_2Sm_2$	978	0.0012	48	115
I_3Sn	550	0.0012	49	64
$Fe_2Li_2P_2$	636	0.0012	25	81
Hf_3Te_2	896	0.0013	41	130
In_2Te_3	879	0.0013	44	123
Sb_2SeTe_2	879	0.0013	44	123
Ag_2Br_2	154	0.0014	9	25
K	403	0.0014	48	115
Bi_2Mn_2	408	0.0014	20	72
Bi_2Se_2Te	879	0.0014	44	123
CKN	519	0.0015	43	87
FeH_2O_2	397	0.0015	12	65
$I_2O_2Pr_2$	738	0.0015	34	89
STl_2	633	0.0016	44	123
F_4Nb	470	0.0016	25	64
Br_2La_2	608	0.0016	34	101
Br_2Ca_3Si	978	0.0016	48	115
Sb_2	406	0.0016	34	101
$FeSe_2$	339	0.0016	16	81
$Cl_2N_2Ti_2$	78	0.0017	3	10

Table showing the first 50 lattice matching combinations with the smallest strain needed to build the supercell.

KTlO ($P2_1/m$)

Structural and electronic properties

**Formula**	KTlO
**Spacegroup**	$P2_1/m$
**Prototype**	RhTeCl
**Parent 3D**	$K_2Tl_2O_2$
**Source DB**	COD
**DB ID**	1528008
**DF2-C09 Binding energy [meV/ Å^2]**	27.36
**RVV10 Binding energy [meV/ Å^2]**	33.3
**Band gap (PBE) [eV]**	1.54

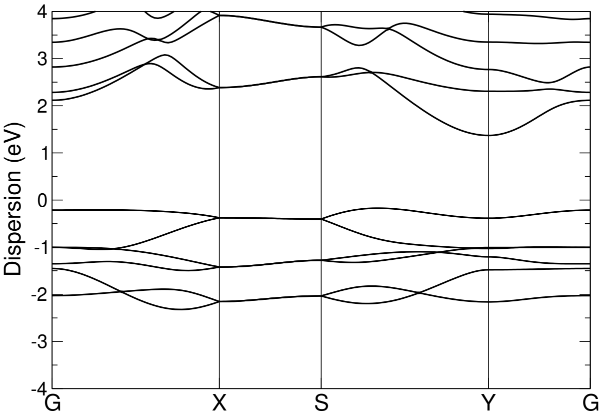

Band structure: Electronic band structure of KTlO ($P2_1/m$) in a representative window around the Fermi energy and along a high-symmetry path.

Optimized crystal structure

Structural parameters: relaxed cell (top) and atomic positions (bottom) of KTlO ($P2_1/m$) in Cartesian coordinates.

	x [Å]	y [Å]	z [Å]
$\mathbf{a_1}$	3.79333809	0.00000000	0.00000000
$\mathbf{a_2}$	0.00000000	6.22789093	0.00000000
$\mathbf{a_3}$	0.00000000	0.00000000	22.92933309

		x [Å]	y [Å]	z [Å]
●	K	1.89666904	5.14023489	9.99121699
●	Tl	1.89666904	0.98297122	12.85195911
•	O	1.89666904	2.96153977	11.55784546
●	K	0.00000000	4.19459421	12.93811062
●	Tl	0.00000000	2.12396123	10.07737845
•	O	0.00000000	0.14538891	11.37148863

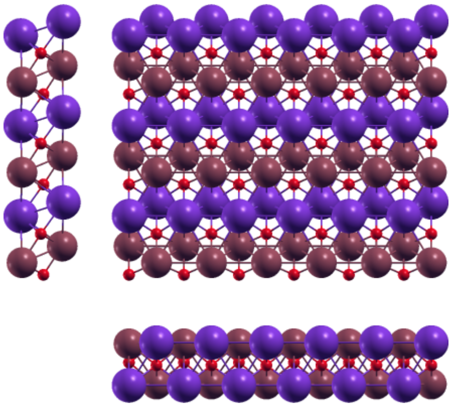

Orthographic projections: views of KTlO ($P2_1/m$) as seen from the x axis (left), the y axis (bottom) and the z axis (center).

Lattice matching - minimal size

Formula	N° atoms	strain	cell size 1	cell size 2
Na	8	0.0213	1	2
In	8	0.2341	1	2
$AuTe_2$	9	0.4778	1	1
I_2Zn	9	0.4893	1	1
$PtTe_2$	9	0.4768	1	1
$AgNO_2$	10	1.1898	1	1
AsSb	10	0.0242	1	2
Br_2Tb_2	10	0.4732	1	1
Ge_2S_2	10	0.2973	1	1
Cl_2La_2	10	0.4834	1	1
As_2	10	0.0159	1	2
Au_2Se_2	10	0.0059	1	1
In_2Se_2	10	0.4865	1	1
$SbSe_2Tl$	10	0.0196	1	1
As_2Sn_2	10	0.4808	1	1
FKO_2Se	11	0.2243	1	1
Bi_2S_3	11	0.4738	1	1
$CuGeO_3$	11	0.4137	1	1
Cl_2Zn	12	0.0165	1	2
Cl_2Mn	12	0.1215	1	2
$MoTe_2$	12	0.0196	1	2
$ReSe_2$	12	0.1285	1	2
S_2Ta	12	0.1225	1	2
Br_2Zn	12	0.016	1	2
HfS_2	12	0.0144	1	2
$SiTe_2$	12	0.0189	1	2
Te_2V	12	0.0183	1	2
$CuTe_2$	12	0.0146	1	2
PbS_2	12	0.0219	1	2
Br_2Co	12	0.0161	1	2
Cl_4Cu_2	12	0.5006	1	1
Ca_2N	12	0.0156	1	2
S_2Ti	12	0.125	1	2
$I_2Se_2Tb_2$	12	0.2176	1	1
Te_2Ti	12	0.0162	1	2
$Gd_2I_2Se_2$	12	0.2191	1	1
NbS_2	12	0.1222	1	2
$Br_2S_2Y_2$	12	0.4568	1	1
Te_2Zn	12	0.0198	1	2
I_4Zr_2	12	0.0383	1	1
$RhTe_2$	12	0.0138	1	2
Br_2Mn	12	0.0173	1	2
Cl_2Ni	12	0.129	1	2
Cl_2Co	12	0.1248	1	2
$Br_2Er_2Se_2$	12	0.5011	1	1
PtS_2	12	0.0205	1	2
NbS_2	12	0.1202	1	2
$CoTe_2$	12	0.0143	1	2
Br_2V	12	0.128	1	2
ClNZr	12	0.1268	1	2

Table showing the first 50 lattice matching combinations with the smallest number of atoms in the supercell.

Lattice matching - minimal strain

Formula	N° atoms	strain	cell size 1	cell size 2
$Ho_2I_2Se_2$	966	0.0005	81	80
I_2La_2Sb	837	0.0007	67	87
CuO_2	606	0.0008	45	112
$Gd_2I_2S_2$	660	0.0009	54	56
Ge_2Te_2	552	0.001	50	63
C_4Ca_2	720	0.001	50	70
Bi_2Te_2	924	0.0011	86	102
$I_2S_2Tm_2$	870	0.0011	70	75
Li_2Tl_2	288	0.0012	28	30
BiBrTe	249	0.0013	24	35
$Dy_2I_2S_2$	738	0.0013	60	63
$Cl_2H_2Zr_2$	972	0.0013	47	115
$AuTe_2$	816	0.0013	74	124
$PtTe_2$	816	0.0013	74	124
Nd	146	0.0013	15	56
Cu_4Te_2	354	0.0013	24	35
$Cu_2Na_2Te_2$	756	0.0013	56	70
In_2Se_2	692	0.0014	68	71
As_2Sn_2	874	0.0014	69	115
PbS_2Sn	204	0.0014	18	24
$H_2Na_2O_2$	948	0.0015	52	106
Li_2P_2Pr	732	0.0015	52	84
$Ga_2Ge_2Te_2$	816	0.0015	52	84
Br_2La_2	700	0.0015	56	91
$Er_2I_2Se_2$	966	0.0015	81	80
$I_2S_2Tb_2$	738	0.0016	60	63
$Ho_2I_2Se_2$	942	0.0016	79	78
Sb_2	518	0.0016	56	91
H_2MgO_2	743	0.0016	38	103
Sb_2	480	0.0016	52	84
K	538	0.0017	74	94
$CeLi_2P_2$	673	0.0017	48	77
AsI_2La_2	726	0.0017	56	78
I_2La_2	852	0.0017	70	108
Sb_2Te_3	319	0.0017	24	35
BH_4Li	750	0.0017	48	77
$F_2Lu_2Se_2$	882	0.0017	56	91
$C_2I_2Y_2$	810	0.0017	51	84
Br_2La_2	648	0.0017	52	84
Br_2HLa	596	0.0018	48	77
$SnTe_2$	564	0.0018	52	84
$AuTe_2$	759	0.0018	69	115
Bi_2Te_2	708	0.0018	66	78
$CrSe_2$	615	0.0018	44	117
$Br_2O_2V_2$	516	0.0018	30	56
$SnTe_2$	519	0.0018	48	77
GeI_3Rb	411	0.0018	56	15
Cl_2La_2	874	0.0018	69	115
FHOZn	732	0.0018	44	117
$Cl_2Fe_2O_2$	270	0.0018	15	30

Table showing the first 50 lattice matching combinations with the smallest strain needed to build the supercell.

La_2AsI_2 (P-3m1)

Structural and electronic properties

**Formula**	La_2AsI_2
**Spacegroup**	P-3m1
**Prototype**	Bi2Te2S
**Parent 3D**	AsI_2La_2
**Source DB**	MPDS
**DB ID**	S1713663
**DF2-C09 Binding energy [meV/ Å^2]**	11.67
**RVV10 Binding energy [meV/ Å^2]**	N/A
**Band gap (PBE) [eV]**	0.0

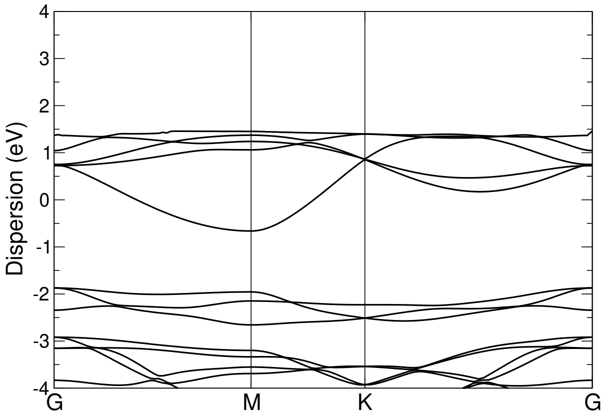

Band structure: Electronic band structure of La_2AsI_2 (P-3m1) in a representative window around the Fermi energy and along a high-symmetry path.

Optimized crystal structure

Structural parameters: relaxed cell (top) and atomic positions (bottom) of La_2AsI_2 (P-3m1) in Cartesian coordinates.

	x [Å]	y [Å]	z [Å]
$\mathbf{a_1}$	2.20919497	−3.82643793	0.00000000
$\mathbf{a_2}$	2.20919497	3.82643793	0.00000000
$\mathbf{a_3}$	0.00000000	0.00000000	27.01343761

		x [Å]	y [Å]	z [Å]
●	La	2.20919497	-1.27547931	-1.66011905
●	I	2.20919497	1.27547931	-3.75824166
●	La	2.20919497	1.27547931	1.66011905
●	As	0.00000000	0.00000000	0.00000000
●	I	2.20919497	-1.27547931	3.75824166

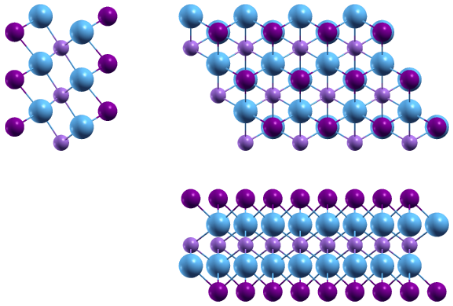

Orthographic projections: views of La_2AsI_2 (P-3m1) as seen from the x axis (left), the y axis (bottom) and the z axis (center).

Lattice matching - minimal size

Formula	N° atoms	strain	cell size 1	cell size 2
GeTe	7	0.2537	1	1
AgTl	7	0.1358	1	1
S_2	7	0.2558	1	1
$IrTe_2$	8	0.2549	1	1
CaI_2	8	0.0075	1	1
$GeTe_2$	8	1.6277	1	1
$HfTe_2$	8	0.2665	1	1
I_2Pr	8	0.1232	1	1
NSr_2	8	0.2478	1	1
I_2Yb	8	0.0049	1	1
Tl	8	0.6379	1	3
LiO_2	8	1.6114	1	1
Cl_2Zn	8	0.1118	1	1
$PdTe_2$	8	0.2743	1	1
FeI_2	8	0.2495	1	1
CrI_2	8	0.2489	1	1
Ba_2Hg	8	0.1481	1	1
Ba_2N	8	0.2687	1	1
Te_2Zr	8	0.2672	1	1
I_2Nd	8	0.1238	1	1
I_2Tm	8	0.0063	1	1
BiITe	8	0.0029	1	1
CoI_2	8	0.2462	1	1
CeI_2	8	0.1228	1	1
Br_2Mg	8	0.2493	1	1
I_2Ti	8	0.2467	1	1
GdI_2	8	0.0065	1	1
Br_2Cd	8	0.2729	1	1
I_2La	8	0.1268	1	1
F_2Na	8	1.5379	1	1
Se_2Sn	8	0.2475	1	1
F_2Zn	8	0.1209	1	1
Ba_2Cd	8	0.1506	1	1
NaPSn	8	0.2652	1	1
Fe_2Te_2	9	0.1164	1	1
Li_2Tl_2	9	0.4958	1	1
Ca_2Cl_2	9	0.1166	1	1
Cu_2I_2	9	0.139	1	1
Cl_2Gd_2	9	0.2588	1	1
Cu_2Sr_2	9	0.0083	1	1
Cl_2OOs	9	0.1101	1	1
Br_2Pr_2	9	1.5323	1	1
Ir_2P_2	9	0.1237	1	1
Ag_2Br_2	9	0.1272	1	1
Cu_2O_2	9	0.2294	1	1
Cu_2S_2	9	0.1192	1	1
Au_2Br_2	9	0.1482	1	1
$AlLiTe_2$	9	0.0041	1	1
Ge_2Te_2	9	0.1648	1	1
Br_2Tb_2	9	0.2752	1	1

Table showing the first 50 lattice matching combinations with the smallest number of atoms in the supercell.

Lattice matching - minimal strain

Formula	N° atoms	strain	cell size 1	cell size 2
MnO_2	317	0.0	25	64
Br_2Mg	437	0.0	49	64
Cd_2I_3	10	0.0	1	1
PTe_2Zr_2	500	0.0	43	57
NaO_4	425	0.0	49	36
$GeNi_3Te_2$	911	0.0	73	91
CCl_2Sc_2	490	0.0001	37	61
Li_2Tl_2	516	0.0001	64	49
FeI_2	437	0.0002	49	64
Br_2Zr_2	180	0.0002	16	25
ClNZr	488	0.0002	49	81
Se_2Ti	155	0.0002	16	25
Ga_2Se_2	577	0.0002	57	73
CrI_2	437	0.0002	49	64
S_2	431	0.0002	57	73
Te_2Zr	705	0.0002	81	100
$Br_2H_2Zr_2$	551	0.0003	37	61
$MoSe_2$	93	0.0003	9	16
$NiTe_2$	327	0.0003	36	49
ReS_2	432	0.0003	39	79
CaClHO	376	0.0003	36	49
Cl_2Gd_2	644	0.0003	64	81
Ge_2S_2	368	0.0004	40	42
Cl_2Tb_2	644	0.0004	64	81
Ni_2SbTe_2	650	0.0004	57	73
$IrTe_2$	504	0.0004	57	73
CoH_2O_2	745	0.0004	49	100
$Cl_2O_2Tm_2$	474	0.0004	36	49
Se_2W	93	0.0005	9	16
Cl_2Zr_2	429	0.0005	37	61
PSn_2	233	0.0006	25	36
S_2Sn_2	879	0.0006	99	96
$C_2Br_2La_2$	718	0.0006	62	68
NaPSn	638	0.0006	73	91
In	174	0.0006	25	49
In_2Se_3	905	0.0007	81	100
$Br_2S_2Y_2$	745	0.0007	77	60
$HfTe_2$	705	0.0007	81	100
AsSb	329	0.0007	43	57
$AgNO_3$	425	0.0008	49	36
Ba_2N	705	0.0008	81	100
Pt_2Te_2	729	0.0008	73	91
ClNZr	368	0.0008	37	61
Mo_2Te_4	527	0.0009	55	42
O_2Pt	272	0.0009	25	49
$SiTe_2$	327	0.0009	36	49
I_2Y_2	644	0.0009	64	81
$AgTe_2$	649	0.001	65	108
Se_4TiZr	233	0.001	25	18
Tl	61	0.001	9	16

Table showing the first 50 lattice matching combinations with the smallest strain needed to build the supercell.

$La_2C_2Br_2$ (C2/m)

Structural and electronic properties

**Formula**	$La_2C_2Br_2$
**Spacegroup**	C2/m
**Prototype**	Gd2C2Br2
**Parent 3D**	$C_2Br_2La_2$
**Source DB**	MPDS
**DB ID**	S1936152
**DF2-C09 Binding energy [meV/ Å^2]**	10.46
**RVV10 Binding energy [meV/ Å^2]**	N/A
**Band gap (PBE) [eV]**	0.0

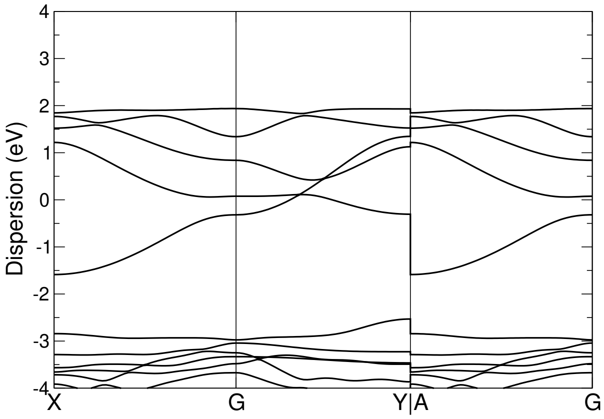

Band structure: Electronic band structure of $La_2C_2Br_2$ (C2/m) in a representative window around the Fermi energy and along a high-symmetry path.

Optimized crystal structure

Structural parameters: relaxed cell (top) and atomic positions (bottom) of $La_2C_2Br_2$ (C2/m) in Cartesian coordinates.

	x [Å]	y [Å]	z [Å]
$\mathbf{a_1}$	0.00001562	4.10030689	0.00000000
$\mathbf{a_2}$	3.75248722	2.05016760	0.00000000
$\mathbf{a_3}$	0.00000000	0.00000000	25.84537508

	x [Å]	y [Å]	z [Å]
La	0.89490173	1.02545567	1.53833741
C	2.44874152	3.07513941	0.33281271
Br	-0.30922897	3.07553285	3.46829035
La	-0.89488612	3.07485121	-1.53833741
C	1.30376131	3.07533508	-0.33281271
Br	0.30924458	1.02477404	-3.46829035

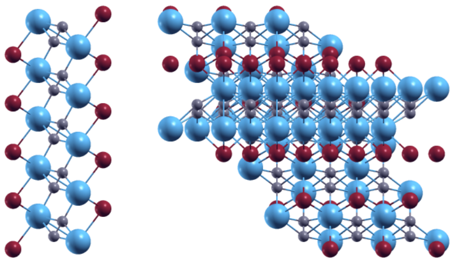

Orthographic projections: views of $La_2C_2Br_2$ (C2/m) as seen from the x axis (left), the y axis (bottom) and the z axis (center).

Lattice matching - minimal size

Formula	N° atoms	strain	cell size 1	cell size 2
K	7	0.1806	1	1
P_2	8	0.1846	1	1
CaCl	8	0.1093	1	1
Sm	8	0.1476	1	2
Nd	9	0.4465	1	3
$MoSe_2$	9	0.1882	1	1
I_2Pr	9	0.1318	1	1
Cl_2Ti	9	0.1848	1	1
Cl_2Zn	9	0.1159	1	1
I_2Nd	9	0.1326	1	1
$MnSe_2$	9	0.1092	1	1
CeI_2	9	0.1312	1	1
CNNa	9	0.1855	1	1
F_2Ni	9	0.1138	1	1
F_2Zn	9	0.1288	1	1
Se_2W	9	0.1883	1	1
Fe_2Te_2	10	0.1226	1	1
Ca_2Cl_2	10	0.1229	1	1
Cl_2OOs	10	0.1137	1	1
Ir_2P_2	10	0.1325	1	1
$AgNO_2$	10	0.8297	1	1
AsSb	10	0.4572	1	2
S_2Sn_2	10	0.1647	1	1
Cu_2S_2	10	0.1265	1	1
Ge_2Te_2	10	0.1841	1	1
Br_2Cu_2	10	0.1278	1	1
Fe_2Se_2	10	0.1136	1	1
Cl_2ORu	10	0.1114	1	1
As_2Co_2	10	0.1113	1	1
Cu_2Te_2	10	0.1173	1	1
Ge_2S_2	10	0.1566	1	1
As_4	10	0.189	1	1
Br_2OV	10	0.1077	1	1
O_2Sn_2	10	0.1269	1	1
P_2Rh_2	10	0.126	1	1
Au_2Se_2	10	0.3961	1	1
Fe_2S_2	10	0.1085	1	1
F_2Tl_2	10	0.1261	1	1
Au_2I_2	10	0.2161	1	1
Co_2S_2	10	0.1095	1	1
Ge_2Se_2	10	0.1593	1	1
Cu_2Se_2	10	0.1141	1	1
Ag_2Te_2	10	0.1253	1	1
As_2Ru_2	10	0.1231	1	1
La_2S_2	10	0.1808	1	1
Ni_2Se_2	10	0.1311	1	1
$SbSe_2Tl$	10	0.3311	1	1
Fe_2SeTe	10	0.1191	1	1
Se_2Sn_2	10	0.1835	1	1
Co_2Se_2	10	0.1119	1	1

Table showing the first 50 lattice matching combinations with the smallest number of atoms in the supercell.

Lattice matching - minimal strain

Formula	N° atoms	strain	cell size 1	cell size 2
Cl_4Mn	760	0.0003	80	56
CKN	408	0.0003	50	36
$AgNO_2$	468	0.0004	48	45
$Br_2H_2Yb_2$	138	0.0004	11	12
$As_2Fe_2Li_2$	138	0.0004	11	12
In	249	0.0004	32	57
Br_2Ho_2	644	0.0004	62	68
Fe_2SeTe	114	0.0005	11	12
Bi_2S_3	712	0.0005	62	68
Fe_2O_4	402	0.0005	27	40
O_2Sn_2	700	0.0006	64	79
S_2Zn_2	616	0.0006	58	67
N_4	738	0.0006	57	99
AsI_2La_2	718	0.0006	68	62
Cd_2I_3	718	0.0006	68	62
Br_2Tb_2	644	0.0007	62	68
S_2Ti	687	0.0007	65	99
CBr_2Y_2	234	0.0007	19	24
$Gd_2I_2S_2$	750	0.0008	67	58
$Dy_2I_2S_2$	708	0.0009	63	55
$Ga_2Gd_2I_2$	942	0.0009	80	77
P_2Sn_2	616	0.0009	58	67
DyI_2	543	0.0009	63	55
Cl_2Co	687	0.001	65	99
$PtTe_2$	576	0.001	62	68
$Ga_2I_2Tb_2$	942	0.001	80	77
Br_2Ca_3Si	750	0.0011	67	58
$Br_2Lu_2O_2$	138	0.0011	11	12
CdO_2	687	0.0011	65	99
Cl_2Tb_2	616	0.0011	58	67
$AgTe_2$	507	0.0011	48	73
STl_2	522	0.0012	58	58
BaF_2	522	0.0012	58	58
$I_2S_2Tb_2$	708	0.0012	63	55
$PdTe_2$	576	0.0012	62	68
$Br_2O_2V_2$	708	0.0012	53	65
Se_2Sn_2	576	0.0012	62	51
Bi_2Pd	816	0.0012	80	112
O_2Pt	363	0.0012	32	57
Cl_2Zr	687	0.0012	65	99
$AgClO_4$	726	0.0013	73	48
Ag_2Te_2	194	0.0013	19	20
Ag_2Te_2	174	0.0013	17	18
$Ga_2I_2Y_2$	696	0.0013	58	58
$Ge_2Te_2Zr_2$	138	0.0013	11	12
CCl_2Gd_2	234	0.0013	19	24
Ca_2O_2	928	0.0014	80	112
I_4Zr_2	258	0.0014	27	16
$AuTe_2$	576	0.0014	62	68
HgO	544	0.0014	61	89

Table showing the first 50 lattice matching combinations with the smallest strain needed to build the supercell.

$La_2C_2I_2$ (C2/m)

Structural and electronic properties

**Formula**	$La_2C_2I_2$
**Spacegroup**	C2/m
**Prototype**	Gd2C2Br2
**Parent 3D**	$C_2I_2La_2$
**Source DB**	MPDS
**DB ID**	S1936154
**DF2-C09 Binding energy [meV/ Å^2]**	12.09
**RVV10 Binding energy [meV/ Å^2]**	N/A
**Band gap (PBE) [eV]**	0.01

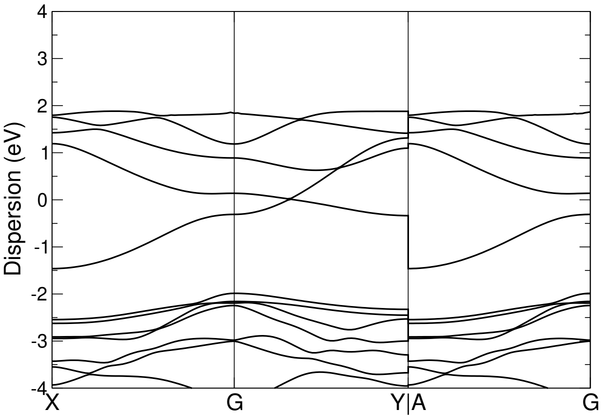

Band structure: Electronic band structure of $La_2C_2I_2$ (C2/m) in a representative window around the Fermi energy and along a high-symmetry path.

Optimized crystal structure

Structural parameters: relaxed cell (top) and atomic positions (bottom) of $La_2C_2I_2$ (C2/m) in Cartesian coordinates.

		x [Å]	y [Å]	z [Å]
$\mathbf{a_1}$		0.00014328	4.17920649	0.00000000
$\mathbf{a_2}$		−3.84467079	2.08947103	0.00000000
$\mathbf{a_3}$		0.00000000	0.00000000	26.66244041
		x [Å]	y [Å]	z [Å]
●	La	-0.88952839	1.04472433	-1.49381301
●	C	-2.51159234	3.13417138	-0.29837708
●	I	-3.47199081	1.04470452	-3.67354222
●	La	-2.95514240	1.04474670	1.49381301
●	C	-1.33293517	3.13450614	0.29837708
●	I	-0.37267998	1.04476652	3.67354222

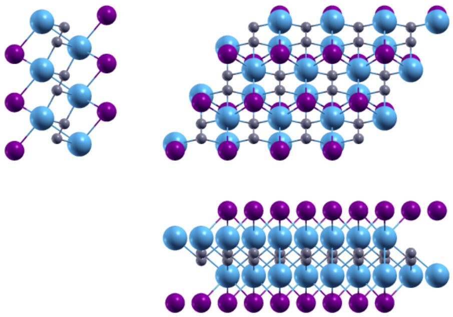

Orthographic projections: views of $La_2C_2I_2$ (C2/m) as seen from the x axis (left), the y axis (bottom) and the z axis (center).

Lattice matching - minimal size

Formula	N° atoms	strain	cell size 1	cell size 2
Cl_2Mn	9	0.1847	1	1
Nd	9	0.4209	1	3
S_2Ta	9	0.1863	1	1
I_2Pr	9	0.1237	1	1
Cl_2Zn	9	0.1105	1	1
HgI_2	9	0.1888	1	1
NbS_2	9	0.186	1	1
NbS_2	9	0.1827	1	1
S_2Ta	9	0.182	1	1
I_2Nd	9	0.1244	1	1
GeS_2	9	0.3959	1	1
CeI_2	9	0.1232	1	1
F_2Ni	9	0.1089	1	1
I_2La	9	0.1278	1	1
F_2Zn	9	0.1211	1	1
Fe_2Te_2	10	0.116	1	1
Ca_2Cl_2	10	0.1162	1	1
Cl_2OOs	10	0.109	1	1
Ir_2P_2	10	0.1243	1	1
Ag_2Br_2	10	0.1282	1	1
$AgNO_2$	10	0.786	1	1
Cu_2S_2	10	0.1192	1	1
Ge_2Te_2	10	0.1709	1	1
GeTe	10	0.4474	1	2
Br_2Cu_2	10	0.1203	1	1
Fe_2Se_2	10	0.1087	1	1
Cl_2ORu	10	0.1073	1	1
As_2Ir_2	10	0.13	1	1
Cu_2Te_2	10	0.1116	1	1
Ge_2S_2	10	0.1467	1	1
As_4	10	0.1769	1	1
O_2Sn_2	10	0.1195	1	1
P_2Rh_2	10	0.1188	1	1
S_2	10	1.5647	1	2
F_2Tl_2	10	0.1188	1	1
As_2Fe_2	10	0.3977	1	1
Cu_2Se_2	10	0.1091	1	1
Ag_2Te_2	10	0.1184	1	1
As_2Ru_2	10	0.1163	1	1
$LiNbS_2$	10	0.1864	1	1
La_2S_2	10	0.1678	1	1
Ni_2Se_2	10	0.1231	1	1
$SbSe_2Tl$	10	0.3116	1	1
As_2Rh_2	10	0.128	1	1
Fe_2SeTe	10	0.1131	1	1
Se_2Sn_2	10	0.1702	1	1
F_4Pb	11	0.1791	1	1
Hf_3Te_2	11	0.1101	1	1
H_2Na_2Pd	11	0.3943	1	1
Cl_2NSc_2	11	0.1851	1	1

Table showing the first 50 lattice matching combinations with the smallest number of atoms in the supercell.

Lattice matching - minimal strain

Formula	N° atoms	strain	cell size 1	cell size 2
$I_2Sb_2Te_2$	570	0.0002	63	32
$ClKO_3$	983	0.0004	123	49
$I_2Nd_2S_2$	708	0.0004	63	55
Bi_2SeTe_2	638	0.0006	58	58
Sb_2Te_3	638	0.0007	58	58
$C_2Cl_2Y_2$	984	0.0008	73	91
$I_2Pr_2S_2$	882	0.0009	79	68
CI_2Y_2	196	0.001	16	20
P_2Rh_2	114	0.001	11	12
Br_2V	459	0.001	43	67
Se_2Sn	156	0.001	16	20
I_2Ti	156	0.001	16	20
F_2Tl_2	114	0.0011	11	12
$Br_2Ho_2O_2$	138	0.0011	11	12
FHOZn	528	0.0011	40	72
NSr_2	156	0.0011	16	20
Bi_2	464	0.0011	58	58
BrNZr	687	0.0011	65	99
Cl_2V	456	0.0011	40	72
$CrSe_2$	456	0.0011	40	72
Ag_2	518	0.0012	67	58
Br_2Cd	549	0.0012	58	67
CoI_2	156	0.0012	16	20
$Ca_2Mn_2Si_2$	138	0.0012	11	12
Ba_2Pt	576	0.0012	67	58
Se_2Ta	759	0.0012	72	109
Br_2Ti	759	0.0012	72	109
Cu_4Te_2	696	0.0012	58	58
K_2PdSe_2	40	0.0012	5	2
Se_2Ta	687	0.0013	65	99
$Ba_2H_2I_2$	696	0.0013	66	50
NbS_2	450	0.0013	41	68
$ReSe_2$	459	0.0013	43	67
N_4	438	0.0013	33	60
Br_2CsF	596	0.0013	66	50
Cl_2Zr_2	526	0.0013	43	67
Br_2Cr	759	0.0014	72	109
$Br_2Ca_2H_2$	138	0.0014	11	12
Br_2Ti	717	0.0014	68	103
$I_2Pr_2S_2$	750	0.0014	67	58
$Br_2Dy_2O_2$	138	0.0014	11	12
$CuGeO_3$	776	0.0014	71	70
I_3Sn	262	0.0014	33	16
$Br_2O_2Y_2$	138	0.0014	11	12
Cu_2S_2	982	0.0014	95	103
O_2Sn_2	982	0.0014	95	103
In_2Se_2	860	0.0014	82	92
$Cl_2H_2Sc_2$	984	0.0015	65	99
Cu_2S_2	114	0.0015	11	12
AsSb	136	0.0015	16	20

Table showing the first 50 lattice matching combinations with the smallest strain needed to build the supercell.

La_2GeI_2 (P-3m1)

Structural and electronic properties

**Formula**	La_2GeI_2
**Spacegroup**	P-3m1
**Prototype**	Bi2Te2S
**Parent 3D**	La_2GeI_2
**Source DB**	ICSD
**DB ID**	414170
**DF2-C09 Binding energy [meV/ Å^2]**	10.95
**RVV10 Binding energy [meV/ Å^2]**	16.62
**Band gap (PBE) [eV]**	0.37

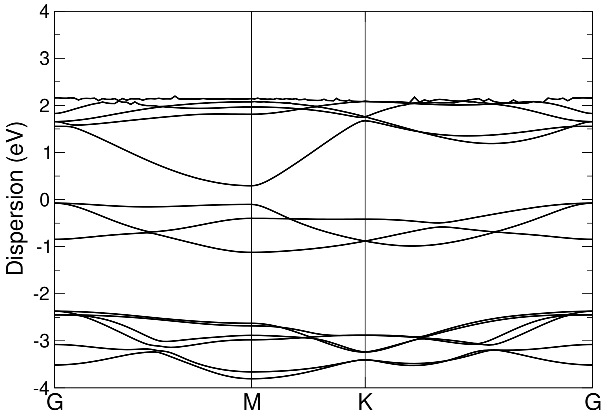

Band structure: Electronic band structure of La_2GeI_2 (P-3m1) in a representative window around the Fermi energy and along a high-symmetry path.

Optimized crystal structure

Structural parameters: relaxed cell (top) and atomic positions (bottom) of La_2GeI_2 (P-3m1) in Cartesian coordinates.

		x [Å]	y [Å]	z [Å]
$\mathbf{a_1}$		4.47414799	0.00000000	0.00000000
$\mathbf{a_2}$		−2.23707400	3.87472582	0.00000000
$\mathbf{a_3}$		0.00000000	0.00000000	27.49786594
		x [Å]	y [Å]	z [Å]
●	La	2.23707400	1.29157527	15.45781978
●	La	0.00000000	2.58315055	12.04004617
●	Ge	0.00000000	0.00000000	13.74893297
●	I	2.23707400	1.29157527	9.97377270
●	I	0.00000000	2.58315055	17.52409324

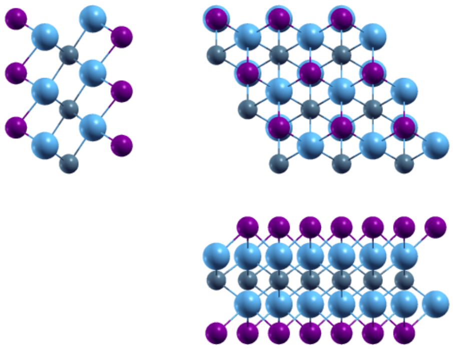

Orthographic projections: views of La_2GeI_2 (P-3m1) as seen from the x axis (left), the y axis (bottom) and the z axis (center).

Lattice matching - minimal size

Formula	N° atoms	strain	cell size 1	cell size 2
GeTe	7	1.5989	1	1
AgTl	7	0.1313	1	1
S_2	7	0.2479	1	1
In	7	0.2216	1	2
$IrTe_2$	8	0.247	1	1
$CdCl_2$	8	1.591	1	1
CaI_2	8	0.0014	1	1
$InSe_2$	8	1.5938	1	1
$HfTe_2$	8	0.2582	1	1
I_2Pr	8	0.1198	1	1
I_2Mn	8	1.5919	1	1
Br_2Cu	8	0.6338	1	1
NSr_2	8	1.5664	1	1
I_2Yb	8	0.0011	1	1
$AuTe_2$	8	0.2692	1	1
$PdTe_2$	8	0.2657	1	1
I_2Ni	8	1.585	1	1
I_2Zn	8	0.2759	1	1
Ba_2Hg	8	0.1428	1	1
Ba_2N	8	0.2603	1	1
Te_2Zr	8	0.2589	1	1
I_2Nd	8	0.1204	1	1
I_2Tm	8	0.0003	1	1
I_2V	8	1.5207	1	1
BiITe	8	0.0087	1	1
CoI_2	8	1.5577	1	1
DyI_2	8	0.0043	1	1
CeI_2	8	0.1194	1	1
$NbSe_2$	8	4.8631	1	1
F_2Ni	8	0.4277	1	1
$PtTe_2$	8	0.2686	1	1
Br_2Cd	8	0.2643	1	1
I_2La	8	0.1231	1	1
Se_2Sn	8	1.5646	1	1
F_2Zn	8	0.1178	1	1
Ba_2Cd	8	0.1451	1	1
NaPSn	8	0.257	1	1
H_2Si_2	9	1.5961	1	1
Fe_2Te_2	9	0.1138	1	1
Li_2Tl_2	9	0.4803	1	1
Ca_2Cl_2	9	0.114	1	1
Cu_2I_2	9	0.1342	1	1
Cl_2OOs	9	0.1085	1	1
Ir_2P_2	9	0.1202	1	1
Ag_2Br_2	9	0.1234	1	1
Br_2Er_2	9	0.27	1	1
O_2Sn_2	9	0.4478	1	1
Cu_2S_2	9	0.1162	1	1
Au_2Br_2	9	0.1425	1	1
Ge_2Te_2	9	0.1585	1	1

Table showing the first 50 lattice matching combinations with the smallest number of atoms in the supercell.

Lattice matching - minimal strain

Formula	N° atoms	strain	cell size 1	cell size 2
Br_2Cd	638	0.0	73	91
H_2NiO_2	370	0.0	25	49
$Cl_2Er_2H_2$	557	0.0	43	57
$F_2Se_2Yb_2$	629	0.0001	49	64
Br_2Hf_2	569	0.0001	49	81
I_2Mn	386	0.0001	43	57
Te_2V	155	0.0001	16	25
P_2Sn_2	501	0.0001	49	64
$RhTe_2$	233	0.0001	25	36
BrNZr	368	0.0001	37	61
Br_2PY_2	820	0.0002	73	91
$CdCl_2$	386	0.0002	43	57
$InSe_2$	386	0.0002	43	57
$AgClO_4$	613	0.0002	65	48
$Cl_2Hf_2N_2$	230	0.0002	16	25
OTl_2	155	0.0002	16	25
Br_2Ho_2	805	0.0003	81	100
I_2Tm	8	0.0003	1	1
NaO_4	500	0.0003	57	43
S_2Zn_2	501	0.0003	49	64
GeI_3Rb	485	0.0003	81	16
Te_2Zr	563	0.0003	64	81
Bi_2Se_2	356	0.0003	40	39
PTe_2Zr_2	425	0.0004	36	49
Ni_2Te_2	443	0.0004	43	57
CdClO	155	0.0004	16	25
$C_2Br_2Tb_2$	200	0.0004	16	20
Cl_2Rb_2	866	0.0004	130	54
Cu_3Se_3	806	0.0005	64	81
$GeNi_3Te_2$	723	0.0005	57	73
H_2Si_2	443	0.0005	43	57
Bi_2S_3	905	0.0005	81	100
$C_2Br_2Gd_2$	200	0.0005	16	20
$C_2Br_2Y_2$	679	0.0006	53	69
Br_2Hf_2	429	0.0006	37	61
$NbSe_2$	488	0.0006	49	81
CdClHO	269	0.0007	25	36
$LiOS_2Ti$	125	0.0007	9	16
$PtTe_2$	705	0.0007	81	100
IO_3Tl	65	0.0007	9	4
Pd_2S_4	842	0.0007	100	57
Ba_2N	563	0.0007	64	81
Br_2Tb_2	805	0.0008	81	100
In_2Se_3	725	0.0008	64	81
$Br_2O_2V_2$	173	0.0008	13	18
$HfTe_2$	563	0.0008	64	81
$AsSe_2$	488	0.0008	49	81
Cl_2Mn	93	0.0009	9	16
Ni_2SbTe_2	565	0.0009	49	64
GeTe	329	0.0009	43	57

Table showing the first 50 lattice matching combinations with the smallest strain needed to build the supercell.

La_2I_2 (P-3m1)

Structural and electronic properties

**Formula**	La_2I_2
**Spacegroup**	P-3m1
**Prototype**	PtTe
**Parent 3D**	I_2La_2
**Source DB**	MPDS
**DB ID**	S1301547
**DF2-C09 Binding energy [meV/ Å^2]**	13.75
**RVV10 Binding energy [meV/ Å^2]**	N/A
**Band gap (PBE) [eV]**	0.0

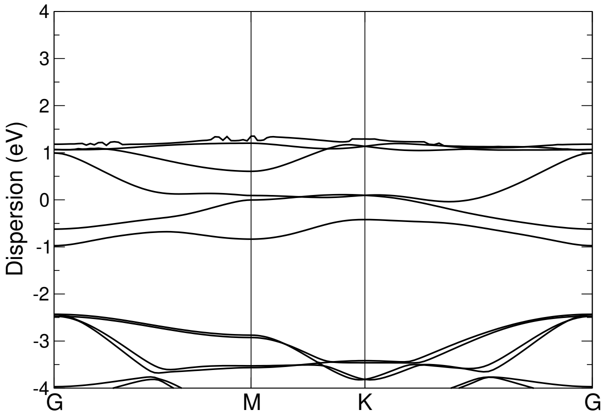

Band structure: Electronic band structure of La_2I_2 (P-3m1) in a representative window around the Fermi energy and along a high-symmetry path.

Optimized crystal structure

Structural parameters: relaxed cell (top) and atomic positions (bottom) of La_2I_2 (P-3m1) in Cartesian coordinates.

		x [Å]	y [Å]	z [Å]
$\mathbf{a_1}$		2.09665784	−3.63151791	0.00000000
$\mathbf{a_2}$		2.09666566	3.63152242	0.00000000
$\mathbf{a_3}$		0.00000000	0.00000000	26.67023833
		x [Å]	y [Å]	z [Å]
●	La	1.04834333	0.60526131	-1.49238475
●	I	1.04833922	-1.81575301	-3.69692898
●	La	3.14498016	-0.60525679	1.49238475
●	I	1.04831862	-1.81576490	3.69692898

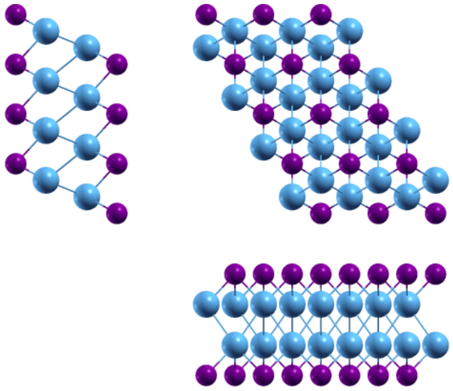

Orthographic projections: views of La_2I_2 (P-3m1) as seen from the x axis (left), the y axis (bottom) and the z axis (center).

Lattice matching - minimal size

Formula	N° atoms	strain	cell size 1	cell size 2
InSe	6	0.0059	1	1
AgTl	6	0.1583	1	1
Ag_2	6	0.4536	1	1
PbTe	6	0.0077	1	1
CaCl	6	0.1159	1	1
I_2Mg	7	0.0045	1	1
CdI_2	7	0.0094	1	1
PSn_2	7	0.2507	1	1
Ba_2Pt	7	0.453	1	1
Br_2Zn	7	0.2653	1	1
$AsSn_2$	7	0.2557	1	1
$SiTe_2$	7	0.2713	1	1
I_2Pr	7	0.1413	1	1
S_2Zr	7	0.2496	1	1
Br_2La	7	0.0041	1	1
Ca_2Si	7	0.4642	1	1
BrCdI	7	0.0014	1	1
Cl_2Zn	7	0.1236	1	1
Te_2Ti	7	0.2658	1	1
BaF_2	7	0.002	1	1
$RhTe_2$	7	0.2596	1	1
GeI_2	7	0.0063	1	1
AsKSn	7	0.0005	1	1
$PbTe_2$	7	0.0023	1	1
I_2Nd	7	0.1421	1	1
$NiTe_2$	7	0.2704	1	1
Cl_2Cu	7	0.0659	1	1
S_2Sn	7	0.25	1	1
$SnTe_2$	7	0.0085	1	1
I_2V	7	0.2733	1	1
GeI_2	7	0.0079	1	1
Se_2Zr	7	0.2719	1	1
I_2Pb	7	0.4575	1	1
STl_2	7	0.0029	1	1
$PtSe_2$	7	0.2566	1	1
GeS_2	7	0.1111	1	1
$TaTe_2$	7	0.2549	1	1
$MnSe_2$	7	0.1158	1	1
CeI_2	7	0.1406	1	1
$NbTe_2$	7	0.2494	1	1
In	7	0.6271	1	3
Se_2Yb	7	0.006	1	1
$BiTe_2$	7	0.0058	1	1
F_2Ni	7	0.1212	1	1
I_2La	7	0.1463	1	1
F_2Na	7	0.2682	1	1
CdI_2	7	0.009	1	1
F_2Zn	7	0.1379	1	1
$HfSe_2$	7	0.2658	1	1
Bi_2Te_2	8	0.4971	1	1

Table showing the first 50 lattice matching combinations with the smallest number of atoms in the supercell.

Lattice matching - minimal strain

Formula	N° atoms	strain	cell size 1	cell size 2
CBr_2Y_2	747	0.0	73	91
KS_2Ti	340	0.0	36	49
Sb_2Te_2	724	0.0001	100	81
$NbTe_2$	388	0.0001	49	64
Ga_2S_2	400	0.0001	43	57
$As_2Cd_2K_2$	548	0.0002	65	48
$AsSn_2$	447	0.0002	57	73
$RhTe_2$	499	0.0002	64	81
PTe_2Ti_2	457	0.0002	43	57
S_2Zr	388	0.0003	49	64
$Cl_2O_2Yb_2$	924	0.0003	81	100
Br_2Mn	291	0.0004	36	49
Br_2Ni	343	0.0004	43	57
$TaTe_2$	447	0.0004	57	73
Br_2Pr_2	724	0.0004	81	100
F_2Na	624	0.0004	81	100
Cl_2Mg	343	0.0004	43	57
O_2Sn_2	568	0.0005	70	72
Se_2Ta	208	0.0005	25	36
AsKSn	7	0.0005	1	1
Cl_2Y_2	656	0.0005	73	91
CdClHO	580	0.0005	64	81
$LiOS_2Ti$	189	0.0005	16	25
S_2Sn	388	0.0006	49	64
CCl_2Gd_2	747	0.0006	73	91
Bi_2Te_2	452	0.0006	64	49
Ag_2F_4	452	0.0007	65	32
Br_2Zn	565	0.0007	73	91
$Cl_2H_2Sc_2$	316	0.0007	25	36
$Cl_2H_2Lu_2$	514	0.0007	43	57
Se_4TiZr	388	0.0007	49	32
Br_2Cu	559	0.0007	76	85
O_2Pt	84	0.0008	9	16
$Cl_2O_2Ti_2$	766	0.0008	70	81
Cl_2Zn	291	0.0008	36	49
IO_3Tl	381	0.0008	64	25
$PtSe_2$	447	0.0008	57	73
$Br_2O_2Ti_2$	636	0.0009	60	66
$Hf_2I_2N_2$	666	0.0009	57	73
CdClHO	520	0.0009	57	73
Cl_2Mn	139	0.001	16	25
Te_2Ti	565	0.001	73	91
As_4	608	0.001	81	71
$HfSe_2$	565	0.001	73	91
$Ce_2I_2S_2$	802	0.0011	91	73
Ca_2Si	583	0.0011	91	73
O_4PSn	548	0.0011	65	48
PSn_2	388	0.0011	49	64
CBr_2Lu_2	516	0.0011	49	64
AgTl	386	0.0011	66	61

Table showing the first 50 lattice matching combinations with the smallest strain needed to build the supercell.

La_2PBr_2 (P-3m1)

Structural and electronic properties

**Formula**	La_2PBr_2
**Spacegroup**	P-3m1
**Prototype**	Bi2Te2S
**Parent 3D**	La_2PBr_2
**Source DB**	ICSD
**DB ID**	418009
**DF2-C09 Binding energy [meV/ Å^2]**	10.55
**RVV10 Binding energy [meV/ Å^2]**	16.75
**Band gap (PBE) [eV]**	N/A

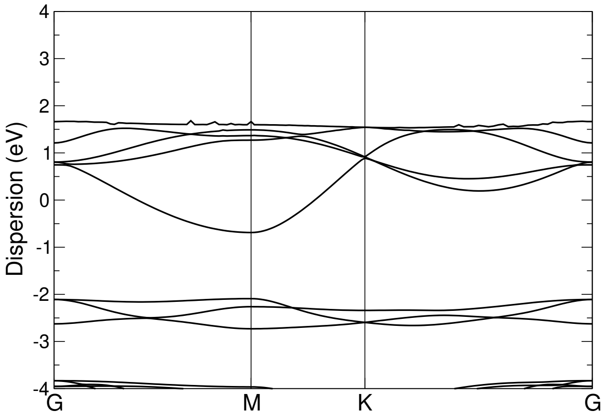

Band structure: Electronic band structure of La_2PBr_2 (P-3m1) in a representative window around the Fermi energy and along a high-symmetry path.

Optimized crystal structure

Structural parameters: relaxed cell (top) and atomic positions (bottom) of La_2PBr_2 (P-3m1) in Cartesian coordinates.

	x [Å]	y [Å]	z [Å]
$\mathbf{a_1}$	4.27374487	0.00000000	0.00000000
$\mathbf{a_2}$	−2.13687244	3.70117163	0.00000000
$\mathbf{a_3}$	0.00000000	0.00000000	27.00181070

		x [Å]	y [Å]	z [Å]
●	La	2.13687244	1.23372388	15.13545095
●	Br	0.00000000	2.46744775	17.01218779
●	La	0.00000000	2.46744775	11.86635975
●	P	0.00000000	0.00000000	13.50090535
●	Br	2.13687244	1.23372388	9.98962291

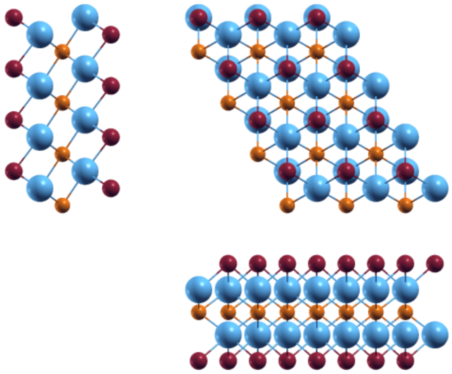

Orthographic projections: views of La_2PBr_2 (P-3m1) as seen from the x axis (left), the y axis (bottom) and the z axis (center).

Lattice matching - minimal size

Formula	N° atoms	strain	cell size 1	cell size 2
Tl	6	4.8662	1	1
InSe	7	0.0032	1	1
AsSb	7	0.2671	1	1
Bi_2	7	0.0026	1	1
PbTe	7	0.0014	1	1
CaCl	7	0.1122	1	1
$CdCl_2$	8	0.2744	1	1
CdI_2	8	0.0003	1	1
PSn_2	8	1.5601	1	1
$MoSe_2$	8	4.8503	1	1
Br_2Zn	8	1.6391	1	1
Br_2Ca	8	0.0015	1	1
$InSe_2$	8	0.275	1	1
$AsSn_2$	8	1.5877	1	1
$GeTe_2$	8	0.2729	1	1
$SiTe_2$	8	0.2585	1	1
I_2Pr	8	0.134	1	1
I_2Mn	8	0.2746	1	1
S_2Zr	8	1.5546	1	1
Br_2Cu	8	0.7068	1	1
NSr_2	8	0.2695	1	1
PbS_2	8	0.2634	1	1
BiClTe	8	0.0007	1	1
Cl_2Zn	8	0.1185	1	1
FeI_2	8	0.2713	1	1
I_2Ni	8	0.2732	1	1
Te_2Ti	8	0.2533	1	1
CrI_2	8	0.2707	1	1
BaF_2	8	0.0069	1	1
BiBrTe	8	0.0063	1	1
$RhTe_2$	8	0.2475	1	1
PtS_2	8	1.4492	1	1
AsKSn	8	0.0083	1	1
I_2Nd	8	0.1347	1	1
$NiTe_2$	8	0.2577	1	1
Cl_2Cu	8	0.0685	1	1
S_2Sn	8	1.5565	1	1
I_2V	8	0.2604	1	1
GeI_2	8	0.0012	1	1
Se_2Zr	8	0.2591	1	1
STl_2	8	0.006	1	1
$PtSe_2$	8	1.5924	1	1
CoI_2	8	0.2677	1	1
GeS_2	8	0.4257	1	1
$MnSe_2$	8	0.1122	1	1
Br_2Ni	8	1.53	1	1
CeI_2	8	0.1334	1	1
Br_2Mg	8	0.2711	1	1
I_2Ti	8	0.2682	1	1
$NbTe_2$	8	1.5531	1	1

Table showing the first 50 lattice matching combinations with the smallest number of atoms in the supercell.

Lattice matching - minimal strain

Formula	N° atoms	strain	cell size 1	cell size 2
$PtSe_2$	386	0.0	43	57
$Cl_2O_2Yb_2$	723	0.0	57	73
$HfLiS_2$	269	0.0001	25	36
F_2Na	504	0.0001	57	73
CoI_2	705	0.0001	81	100
CdI_2	8	0.0001	1	1
Se_2Zr	563	0.0001	64	81
$CrSe_2$	93	0.0002	9	16
Se_2V	368	0.0002	37	61
$I_2N_2Zr_2$	629	0.0002	49	64
FHOZn	109	0.0003	9	16
CdI_2	8	0.0003	1	1
$Cl_2Er_2O_2$	806	0.0003	64	81
AsSb	605	0.0003	81	100
Te_2Zn	233	0.0003	25	36
$Cu_2Rb_2Te_2$	613	0.0004	65	48
CBr_2Lu_2	425	0.0004	36	49
NS_2Ta	511	0.0004	39	79
Tl	326	0.0004	49	81
Gd_2GeI_2	10	0.0004	1	1
$Br_2N_2Zr_2$	474	0.0004	36	49
I_2Ti	705	0.0004	81	100
ClH_3O	820	0.0005	91	73
$MoTe_2$	233	0.0005	25	36
Bi_2STe_2	10	0.0005	1	1
$SiTe_2$	563	0.0006	64	81
PbS_2	638	0.0006	73	91
$AsSn_2$	386	0.0006	43	57
BiClTe	8	0.0007	1	1
Bi_2In_2	517	0.0007	65	48
Te_2W	233	0.0007	25	36
Br_2Pr_2	577	0.0007	57	73
PtS_2	233	0.0007	25	36
I_2Pr	8	0.0008	1	1
I_2V	563	0.0008	64	81
Ga_2Se_2	501	0.0008	49	64
$Br_2Ho_2S_2$	949	0.0008	101	74
$NbTe_2$	327	0.0009	36	49
Br_2Y_2	729	0.0009	73	91
$LiMnTe_2$	9	0.0009	1	1
CI_2Y_2	905	0.0009	81	100
Bi_2Te_2	661	0.0009	81	64
$Cl_2O_2Tm_2$	723	0.0009	57	73
Cu_2O_4	830	0.0009	64	85
S_2Ti	155	0.0009	16	25
Pd_2S_4	561	0.0009	69	36
$Br_2Hf_2N_2$	574	0.0009	50	54
Au_2Br_2	644	0.001	76	66
Pb_2Se_2	710	0.001	86	70
$ClKO_3$	445	0.001	64	25

Table showing the first 50 lattice matching combinations with the smallest strain needed to build the supercell.

La_2PI_2 (P-3m1)

Structural and electronic properties

**Formula**	La_2PI_2
**Spacegroup**	P-3m1
**Prototype**	Bi2Te2S
**Parent 3D**	La_2PI_2
**Source DB**	ICSD
**DB ID**	418010
**DF2-C09 Binding energy [meV/ Å^2]**	11.93
**RVV10 Binding energy [meV/ Å^2]**	17.85
**Band gap (PBE) [eV]**	N/A

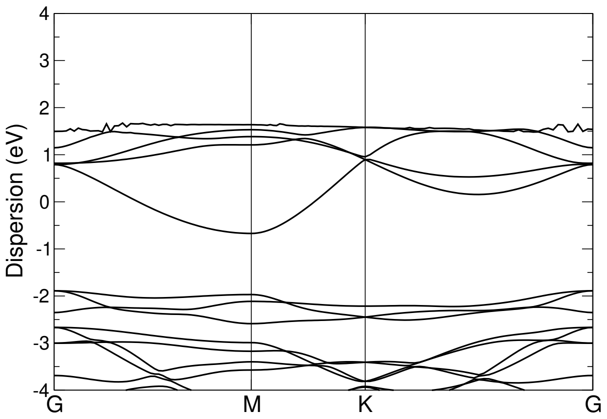

Band structure: Electronic band structure of La_2PI_2 (P-3m1) in a representative window around the Fermi energy and along a high-symmetry path.

Optimized crystal structure

Structural parameters: relaxed cell (top) and atomic positions (bottom) of La_2PI_2 (P-3m1) in Cartesian coordinates.

	x [Å]	y [Å]	z [Å]
$\mathbf{a_1}$	4.34806502	0.00000000	0.00000000
$\mathbf{a_2}$	−2.17403251	3.76553477	0.00000000
$\mathbf{a_3}$	0.00000000	0.00000000	27.47532337

		x [Å]	y [Å]	z [Å]
●	La	-0.00000000	2.51035651	15.34584271
●	I	2.17403251	1.25517826	17.48219743
●	La	2.17403251	1.25517826	12.12948066
●	P	0.00000000	0.00000000	13.73766169
●	I	-0.00000000	2.51035651	9.99312595

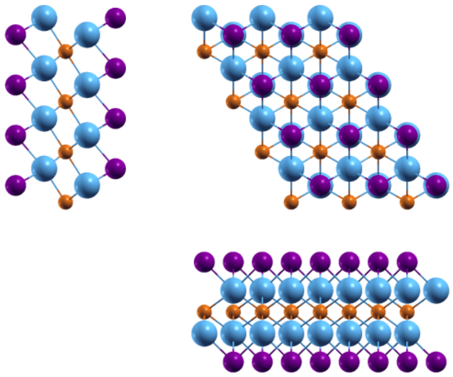

Orthographic projections: views of La_2PI_2 (P-3m1) as seen from the x axis (left), the y axis (bottom) and the z axis (center).

Lattice matching - minimal size

Formula	N° atoms	strain	cell size 1	cell size 2
AsSb	7	0.2557	1	1
Bi_2	7	0.0055	1	1
GeTe	7	0.2641	1	1
S_2	7	0.2664	1	1
CaCl	7	0.4347	1	1
$IrTe_2$	8	0.2654	1	1
$CdCl_2$	8	0.2626	1	1
CdI_2	8	0.0077	1	1
S_2Ta	8	4.8596	1	1
Br_2Ca	8	0.0065	1	1
$InSe_2$	8	0.2632	1	1
$AsSn_2$	8	1.5288	1	1
$GeTe_2$	8	0.2612	1	1
$SiTe_2$	8	0.2476	1	1
I_2Pr	8	0.1281	1	1
I_2Mn	8	0.2628	1	1
NSr_2	8	0.2579	1	1
BiClTe	8	0.0073	1	1
Cl_2Zn	8	0.1147	1	1
FeI_2	8	0.2597	1	1
I_2Ni	8	0.2615	1	1
Te_2Ti	8	1.5806	1	1
NbS_2	8	4.8499	1	1
CrI_2	8	0.2591	1	1
BiBrTe	8	0.0019	1	1
$RhTe_2$	8	1.5489	1	1
I_2Nd	8	0.1287	1	1
$NiTe_2$	8	0.2468	1	1
Cl_2Cu	8	0.0715	1	1
I_2V	8	0.2494	1	1
GeI_2	8	0.0091	1	1
Se_2Zr	8	0.2481	1	1
$PtSe_2$	8	1.5334	1	1
BiITe	8	0.0046	1	1
CoI_2	8	0.2563	1	1
$MnSe_2$	8	0.4345	1	1
CeI_2	8	0.1276	1	1
Br_2Mg	8	0.2595	1	1
I_2Ti	8	0.2568	1	1
GdI_2	8	0.0009	1	1
F_2Ni	8	0.1131	1	1
I_2La	8	0.1321	1	1
CdI_2	8	0.0081	1	1
Se_2Sn	8	0.2576	1	1
F_2Zn	8	0.1254	1	1
Gd	8	0.1746	1	3
I_2Pr	8	0.0073	1	1
$HfSe_2$	8	1.5808	1	1
H_2Si_2	9	0.2636	1	1
Bi_2Te_2	9	0.4533	1	1

Table showing the first 50 lattice matching combinations with the smallest number of atoms in the supercell.

Lattice matching - minimal strain

Formula	N° atoms	strain	cell size 1	cell size 2
CaH_2O_2	305	0.0	25	36
$Cl_2O_2Yb_2$	557	0.0	43	57
Hg_3N_2	445	0.0001	64	25
F_2Na	386	0.0001	43	57
I_2V	437	0.0001	49	64
In_2S_3	820	0.0001	73	91
CrI_2	563	0.0001	64	81
P_2Sn_2	805	0.0001	81	100
GeTe	547	0.0001	73	91
Br_2Mg	563	0.0002	64	81
Cl_2Hf_2	429	0.0002	37	61
AsSb	431	0.0002	57	73
$Cl_2N_2Zr_2$	341	0.0002	25	36
Br_2Hf_2	180	0.0003	16	25
$F_2Se_2Y_2$	893	0.0003	91	73
$RhTe_2$	327	0.0003	36	49
FeI_2	563	0.0003	64	81
O_2Zn	545	0.0003	49	100
$NbSe_2$	155	0.0004	16	25
$Cl_2Ho_2O_2$	629	0.0004	49	64
$CuTe_2$	233	0.0004	25	36
S_2Zn_2	805	0.0005	81	100
CdClHO	376	0.0005	36	49
H_2Si_2	729	0.0005	73	91
ReS_2	272	0.0005	25	49
PTe_2Zr_2	650	0.0005	57	73
As_2O_3	205	0.0005	25	16
Br_2Hg_3	290	0.0005	49	9
C_2F_2	145	0.0006	9	25
CoI_2	504	0.0006	57	73
$AsSe_2$	155	0.0006	16	25
$LiNbS_2$	569	0.0007	49	81
K_2PtTe_2	265	0.0007	39	14
Ni_2SbTe_2	905	0.0007	81	100
BrNZr	155	0.0007	16	25
Br_2Pr_2	443	0.0007	43	57
AlH_4Na	613	0.0007	65	48
S_2Ta	488	0.0007	49	81
$Br_2Hf_2N_2$	341	0.0008	25	36
$Br_2S_2Yb_2$	875	0.0008	91	70
$IrTe_2$	638	0.0008	73	91
HfS_2	233	0.0008	25	36
$InSe_2$	638	0.0008	73	91
S_2	605	0.0008	81	100
Se_2Zr	437	0.0008	49	64
$Cl_2O_2Tm_2$	557	0.0009	43	57
As_4	745	0.0009	85	80
Cu_2Sr_2	9	0.0009	1	1
Sm	70	0.0009	9	25
GdI_2	8	0.0009	1	1

Table showing the first 50 lattice matching combinations with the smallest strain needed to build the supercell.

La_2S_2 (Pmm2)

Structural and electronic properties

**Formula**	La_2S_2
**Spacegroup**	Pmm2
**Prototype**	LaS
**Parent 3D**	La_2S_2
**Source DB**	ICSD
**DB ID**	280987
**DF2-C09 Binding energy [meV/ Å^2]**	0.33
**RVV10 Binding energy [meV/ Å^2]**	N/A
**Band gap (PBE) [eV]**	0.0

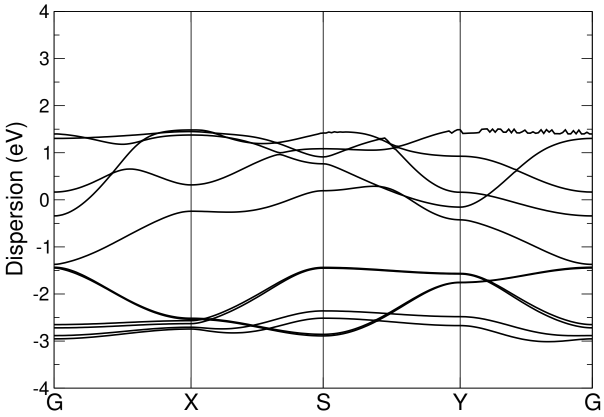

Band structure: Electronic band structure of La_2S_2 (Pmm2) in a representative window around the Fermi energy and along a high-symmetry path.

Optimized crystal structure

Structural parameters: relaxed cell (top) and atomic positions (bottom) of La_2S_2 (Pmm2) in Cartesian coordinates.

	x [Å]	y [Å]	z [Å]
$\mathbf{a_1}$	4.23002016	0.00000000	0.00000000
$\mathbf{a_2}$	0.00000000	4.38559753	0.00000000
$\mathbf{a_3}$	0.00000000	0.00000000	25.78681726

	x [Å]	y [Å]	z [Å]
La	0.00000000	-2.19279877	2.05657898
La	0.00000000	-2.19279877	-2.05657898
S	0.00000000	-4.38559753	3.46816882
S	0.00000000	-4.38559753	-3.46816882

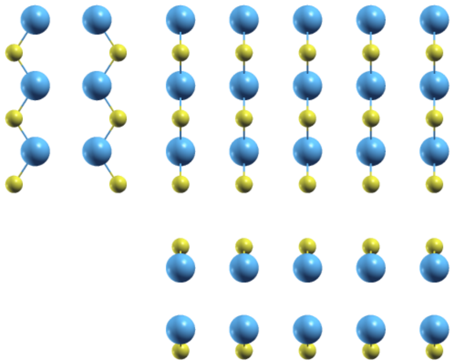

Orthographic projections: views of La_2S_2 (Pmm2) as seen from the x axis (left), the y axis (bottom) and the z axis (center).

Lattice matching - minimal size

Formula	N° atoms	strain	cell size 1	cell size 2
K	5	0.0087	1	1
K	5	0.1368	1	1
Na	5	0.1102	1	1
Sm	6	0.1519	1	2
Nd	7	0.1467	1	3
Ba_2Pt	7	0.1411	1	1
CaI_2	7	0.1299	1	1
I_2Pr	7	0.2293	1	1
Ca_2Si	7	0.1451	1	1
I_2Yb	7	0.1281	1	1
Ba_2Hg	7	0.0155	1	1
$CrTe_2$	7	0.1115	1	1
PtS_2	7	0.11	1	1
CNRb	7	0.3044	1	1
Se_2Ti	7	0.1106	1	1
Br_2Ti	7	0.1116	1	1
I_2Nd	7	0.2305	1	1
I_2Tm	7	0.1291	1	1
Br_2Cr	7	0.1115	1	1
DyI_2	7	0.1319	1	1
CeI_2	7	0.2284	1	1
F_2Ni	7	0.58	1	1
Se_2Ta	7	0.1118	1	1
I_2La	7	0.2365	1	1
F_2Zn	7	0.2245	1	1
Ba_2Cd	7	0.0135	1	1
Bi_2Te_2	8	0.1574	1	1
Fe_2Te_2	8	0.2144	1	1
Li_2Tl_2	8	0.1851	1	1
Ca_2Cl_2	8	0.2149	1	1
Cl_2OOs	8	0.5621	1	1
NS_2Zr	8	0.1101	1	1
Ir_2P_2	8	0.2302	1	1
Ag_2Br_2	8	0.2372	1	1
O_2Sn_2	8	0.1979	1	1
Cu_2S_2	8	0.2208	1	1
Au_2Br_2	8	0.0114	1	1
Ge_2Te_2	8	0.0049	1	1
Br_2Cu_2	8	0.2229	1	1
Fe_2Se_2	8	0.5784	1	1
N_3Na	8	0.3323	1	1
Cu_2Te_2	8	0.5999	1	1
Ge_2S_2	8	0.0407	1	1
Br_2Zr_2	8	0.1107	1	1
O_2Sn_2	8	0.2214	1	1
P_2Rh_2	8	0.22	1	1
Fe_2S_2	8	0.5441	1	1
F_2Tl_2	8	0.2202	1	1
BN	8	0.144	1	2
Sb_2Te_2	8	0.144	1	1

Table showing the first 50 lattice matching combinations with the smallest number of atoms in the supercell.

Lattice matching - minimal strain

Formula	N° atoms	strain	cell size 1	cell size 2
Fe_2SeTe	444	0.0003	48	63
Cd_2I_3	778	0.0003	82	90
$Br_2Dy_2O_2$	818	0.0004	71	89
$Br_2O_2Y_2$	818	0.0004	71	89
Cu_2S_2	640	0.0004	71	89
Ag_2	384	0.0004	64	64
Ba_2Pt	448	0.0004	64	64
$Ge_2Te_2Zr_2$	580	0.0005	49	64
$Br_2Lu_2O_2$	570	0.0005	48	63
$Br_2H_2Yb_2$	570	0.0005	48	63
$Cl_2N_2Zr_2$	414	0.0006	30	49
FeO_2	121	0.0006	10	27
$As_2Fe_2Li_2$	570	0.0006	48	63
CaH_2O_2	365	0.0006	30	49
F_2Tl_2	640	0.0006	71	89
Br_2Hg_3	121	0.0006	24	5
I_2V	228	0.0006	27	40
$LiNbS_2$	92	0.0007	8	15
P_2Rh_2	640	0.0007	71	89
In_2Te_3	50	0.0007	5	6
$Ge_2Te_2Zr_2$	570	0.0007	48	63
O_2Sn_2	640	0.0007	71	89
S_2Ta	77	0.0007	8	15
CBr_2Lu_2	503	0.0008	42	67
Sb_2SeTe_2	50	0.0008	5	6
$Br_2Hf_2N_2$	414	0.0008	30	49
$Br_2N_2Zr_2$	570	0.0008	42	67
$Br_2Cr_2O_2$	592	0.0008	46	68
Se_2Zr	228	0.0008	27	40
STl_2	38	0.0009	5	6
$Cl_2Ho_2O_2$	348	0.0009	27	40
$NbTe_2$	369	0.0009	42	67
$CuTe_2$	267	0.0009	30	49
Cl_2Hf_2	92	0.0009	8	15
CoO_2	121	0.0009	10	27
$I_2Pr_2S_2$	640	0.0009	64	64
Fe_2S_2	488	0.0009	50	72
Ba_2N	685	0.0009	85	115
$Cl_2Er_2O_2$	348	0.0009	27	40
Te_2Zr	685	0.001	85	115
Bi_2Se_2Te	50	0.001	5	6
S_2Zr	369	0.0011	42	67
Br_2Ti	794	0.0011	86	150
Br_2Cr	794	0.0011	86	150
NbS_2	77	0.0011	8	15
$Ca_2Cl_2F_2$	580	0.0011	49	64
$SbSe_2Tl$	264	0.0011	36	30
As_2Fe_2	200	0.0011	20	30
Cl_2Sc_2	944	0.0011	86	150
$SiTe_2$	228	0.0011	27	40

Table showing the first 50 lattice matching combinations with the smallest strain needed to build the supercell.

La_2SbI_2 (P-3m1)

Structural and electronic properties

**Formula**	La_2SbI_2
**Spacegroup**	P-3m1
**Prototype**	Bi2Te2S
**Parent 3D**	I_2La_2Sb
**Source DB**	MPDS
**DB ID**	S1713664
**DF2-C09 Binding energy [meV/ Å^2]**	10.92
**RVV10 Binding energy [meV/ Å^2]**	N/A
**Band gap (PBE) [eV]**	0.0

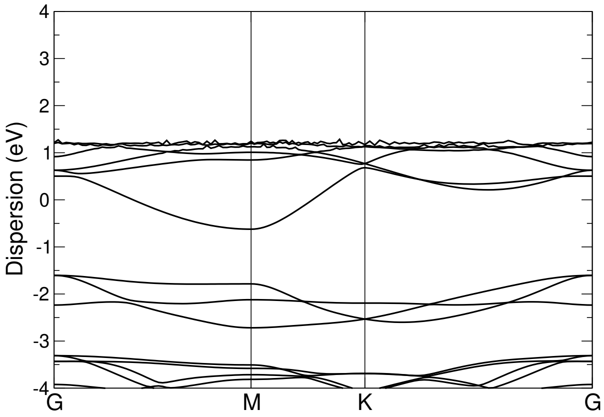

Band structure: Electronic band structure of La_2SbI_2 (P-3m1) in a representative window around the Fermi energy and along a high-symmetry path.

Optimized crystal structure

Structural parameters: relaxed cell (top) and atomic positions (bottom) of La_2SbI_2 (P-3m1) in Cartesian coordinates.

	x [Å]	y [Å]	z [Å]
$\mathbf{a_1}$	2.28972568	−3.96592120	0.00000000
$\mathbf{a_2}$	2.28972568	3.96592120	0.00000000
$\mathbf{a_3}$	0.00000000	0.00000000	27.22912391

		x [Å]	y [Å]	z [Å]
●	La	2.28972568	-1.32197373	1.79845927
●	I	2.28972568	1.32197373	3.81275517
●	La	2.28972568	1.32197373	-1.79845927
●	Sb	0.00000000	0.00000000	0.00000000
●	I	2.28972568	-1.32197373	-3.81275517

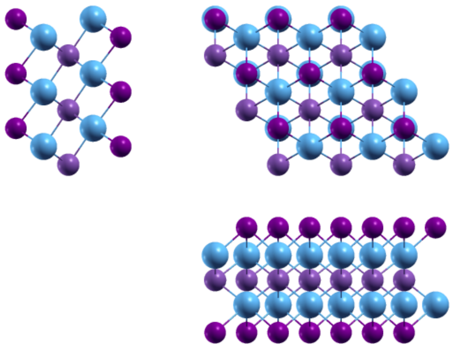

Orthographic projections: views of La_2SbI_2 (P-3m1) as seen from the x axis (left), the y axis (bottom) and the z axis (center).

Lattice matching - minimal size

Formula	N° atoms	strain	cell size 1	cell size 2
K	6	0.1463	1	1
K	6	0.0004	1	1
AgTl	7	0.124	1	1
Ag_2	7	0.0052	1	1
S_2	7	1.5305	1	1
Sb_2	7	0.2647	1	1
I_2Mg	8	0.2729	1	1
Ba_2Pt	8	0.0049	1	1
I_2Pr	8	0.1145	1	1
Br_2La	8	0.2734	1	1
Ca_2Si	8	0.0096	1	1
$AuTe_2$	8	0.2538	1	1
$PdTe_2$	8	0.2505	1	1
I_2Zn	8	0.2601	1	1
GeI_2	8	0.2702	1	1
Ba_2Hg	8	0.1338	1	1
I_2Nd	8	0.115	1	1
Cl_2Cu	8	0.1901	1	1
$SnTe_2$	8	0.2669	1	1
I_2Pb	8	0.0068	1	1
DyI_2	8	0.0066	1	1
CeI_2	8	0.1142	1	1
CuO_2	8	1.5926	1	1
Se_2Yb	8	0.2705	1	1
$BiTe_2$	8	0.2709	1	1
$PtTe_2$	8	0.2533	1	1
Br_2Cd	8	0.2493	1	1
I_2La	8	0.1171	1	1
F_2Zn	8	0.1129	1	1
Ba_2Cd	8	0.1358	1	1
Li_2Tl_2	9	0.4526	1	1
Cu_2I_2	9	0.1264	1	1
Cl_2Gd_2	9	1.5465	1	1
Cu_2Te_2	9	0.1121	1	1
Ir_2P_2	9	0.1149	1	1
Ag_2Br_2	9	0.1174	1	1
Br_2Er_2	9	0.2546	1	1
O_2Sn_2	9	0.1137	1	1
Cu_2S_2	9	0.1118	1	1
Au_2Br_2	9	0.133	1	1
Ge_2Te_2	9	0.1477	1	1
Br_2Cu_2	9	0.1124	1	1
As_2Ir_2	9	0.1186	1	1
O_2Pb_2	9	0.1243	1	1
$AgBrO_2$	9	0.2991	1	1
Cl_2La_2	9	0.2569	1	1
Br_2Gd_2	9	0.2553	1	1
O_2Sn_2	9	0.112	1	1
$AsCuLi_2$	9	0.2695	1	1
Cu_2I_2	9	0.2743	1	1

Table showing the first 50 lattice matching combinations with the smallest number of atoms in the supercell.

Lattice matching - minimal strain

Formula	N° atoms	strain	cell size 1	cell size 2
Te_2W	368	0.0	37	61
$HfLiS_2$	569	0.0	49	81
As_2Sn_2	577	0.0	57	73
Br_2La_2	729	0.0	73	91
Br_2Cd	437	0.0	49	64
Cl_2Gd_2	376	0.0001	36	49
ClNZr	93	0.0001	9	16
$CrSe_2$	545	0.0001	49	100
CrO_2	120	0.0001	9	25
Br_2Gd_2	577	0.0001	57	73
Br_2PY_2	565	0.0001	49	64
$MoTe_2$	368	0.0002	37	61
Ga_2Se_2	269	0.0002	25	36
NaO_4	725	0.0002	81	64
FHOZn	645	0.0002	49	100
Sb_2	547	0.0003	73	91
N_2Re	317	0.0003	25	64
C_4Ca_2	960	0.0003	84	90
$Cl_2O_2Y_2$	341	0.0003	25	36
Te_2Zn	488	0.0003	49	81
Te_2Zn	368	0.0003	37	61
K	6	0.0004	1	1
Te_2Zr	386	0.0004	43	57
CBr_2Lu_2	205	0.0004	16	25
Cu_3Se_3	557	0.0004	43	57
CdH_2O_2	490	0.0004	37	61
Cl_2V	432	0.0005	39	79
$SnTe_2$	705	0.0005	81	100
$Br_2N_2Zr_2$	230	0.0005	16	25
$CeLi_2P_2$	905	0.0005	81	100
C	109	0.0005	9	64
$I_2Lu_2S_2$	284	0.0005	28	24
Sm	129	0.0005	16	49
In_2Se_2	644	0.0005	64	81
Br_2Y_2	269	0.0006	25	36
I_2Zn	563	0.0006	64	81
$Br_2Er_2S_2$	322	0.0006	32	27
$HfLiS_2$	429	0.0006	37	61
$Br_2H_2Zr_2$	141	0.0006	9	16
Ca_4Cu_2	309	0.0006	33	24
Cl_2Tb_2	376	0.0007	36	49
Br_2Er_2	577	0.0007	57	73
$K_2O_2Tl_2$	837	0.0007	87	67
Ba_2N	386	0.0007	43	57
$I_2S_2Sm_2$	11	0.0007	1	1
PtS_2	488	0.0008	49	81
PbS_2	233	0.0008	25	36
$Br_2Ho_2S_2$	322	0.0008	32	27
$CrSe_2$	432	0.0008	39	79
Bi_2Pd	649	0.0008	65	108

Table showing the first 50 lattice matching combinations with the smallest strain needed to build the supercell.

$La_2Si_2I_2$ (P-3m1)

Structural and electronic properties

**Formula**	$La_2Si_2I_2$
**Spacegroup**	P-3m1
**Prototype**	SmSI
**Parent 3D**	$I_2La_2Si_2$
**Source DB**	MPDS
**DB ID**	S1902101
**DF2-C09 Binding energy [meV/ Å^2]**	13.29
**RVV10 Binding energy [meV/ Å^2]**	N/A
**Band gap (PBE) [eV]**	0.0

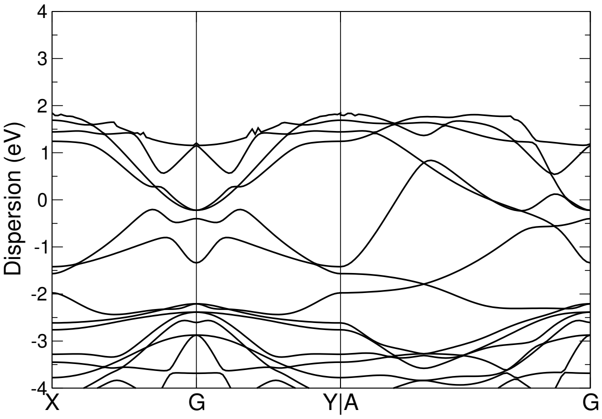

Band structure: Electronic band structure of $La_2Si_2I_2$ (P-3m1) in a representative window around the Fermi energy and along a high-symmetry path.

Optimized crystal structure

Structural parameters: relaxed cell (top) and atomic positions (bottom) of $La_2Si_2I_2$ (P-3m1) in Cartesian coordinates.

		x [Å]	y [Å]	z [Å]
$\mathbf{a_1}$		2.12228288	−3.67584474	0.00000000
$\mathbf{a_2}$		2.12223349	3.67581622	0.00000000
$\mathbf{a_3}$		0.00000000	0.00000000	28.89413565
		x [Å]	y [Å]	z [Å]
●	La	-1.06111668	-1.83790815	-2.05643731
●	Si	1.06112093	-3.06318260	-0.06728849
●	I	1.06113725	-0.61264787	-4.24092537
●	La	-1.06111680	-1.83790808	2.05643731
●	Si	1.06116195	-0.61266214	0.06728849
●	I	1.06114563	-3.06319686	4.24092537

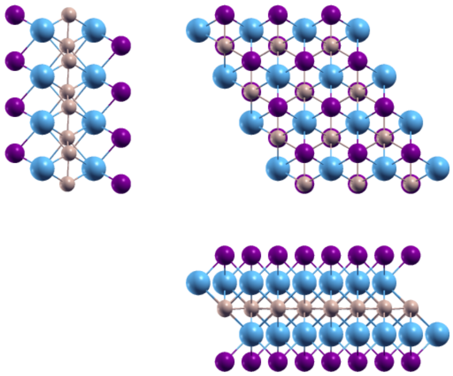

Orthographic projections: views of $La_2Si_2I_2$ (P-3m1) as seen from the x axis (left), the y axis (bottom) and the z axis (center).

Lattice matching - minimal size

Formula	N° atoms	strain	cell size 1	cell size 2
InSe	8	0.0001	1	1
AsSb	8	0.2718	1	1
Bi_2	8	0.0059	1	1
AgTl	8	0.1525	1	1
PbTe	8	0.0018	1	1
CaCl	8	0.1135	1	1
CdI_2	9	0.0036	1	1
Br_2Zn	9	0.2573	1	1
Br_2Ca	9	0.0048	1	1
$AsSn_2$	9	0.2481	1	1
$SiTe_2$	9	0.2631	1	1
I_2Pr	9	0.1365	1	1
$CuTe_2$	9	1.5364	1	1
NSr_2	9	0.2742	1	1
Ca_2Si	9	0.45	1	1
PbS_2	9	0.2681	1	1
BiClTe	9	0.0039	1	1
BrCdI	9	0.007	1	1
Cl_2Zn	9	0.1203	1	1
Te_2Ti	9	0.2577	1	1
CrI_2	9	0.2755	1	1
BaF_2	9	0.0037	1	1
BiBrTe	9	0.0096	1	1
AsKSn	9	0.0051	1	1
$PbTe_2$	9	0.0079	1	1
I_2Nd	9	0.1373	1	1
$NiTe_2$	9	0.2622	1	1
Cl_2Cu	9	0.0675	1	1
I_2V	9	0.265	1	1
GeI_2	9	0.0021	1	1
Se_2Zr	9	0.2636	1	1
STl_2	9	0.0028	1	1
$PtSe_2$	9	0.2489	1	1
CoI_2	9	0.2725	1	1
GeS_2	9	0.433	1	1
$TaTe_2$	9	0.2473	1	1
$MnSe_2$	9	0.1134	1	1
CeI_2	9	0.1359	1	1
Br_2Mg	9	0.2759	1	1
I_2Ti	9	0.273	1	1
F_2Ni	9	0.1182	1	1
I_2La	9	0.1413	1	1
F_2Na	9	0.26	1	1
CdI_2	9	0.0031	1	1
Se_2Sn	9	0.2739	1	1
F_2Zn	9	0.1334	1	1
I_2Pr	9	0.004	1	1
$HfSe_2$	9	0.2578	1	1
Bi_2Te_2	10	0.4821	1	1
Fe_2Te_2	10	0.1272	1	1

Table showing the first 50 lattice matching combinations with the smallest number of atoms in the supercell.

Lattice matching - minimal strain

Formula	N° atoms	strain	cell size 1	cell size 2
As_2Li_2Pr	11	0.0	1	1
InSe	8	0.0001	1	1
$F_2Se_2Y_2$	678	0.0001	64	49
Br_2Y_2	886	0.0001	81	100
Se_2Ti	258	0.0001	25	36
Cl_2Y_2	634	0.0001	57	73
$HNiO_2$	694	0.0002	49	100
$Ba_2H_2I_2$	678	0.0002	65	48
Cu_2F_4	606	0.0002	65	36
S_2V	102	0.0002	9	16
$PtSe_2$	486	0.0002	49	64
Br_2Pr_2	708	0.0002	64	81
Ga_2Se_2	886	0.0003	81	100
PbS_2	786	0.0003	81	100
$AsLi_3$	10	0.0004	1	1
Ga_2S_2	412	0.0004	36	49
Bi_2Te_2	666	0.0004	73	57
I_2V	711	0.0004	73	91
$Cl_2O_2Yb_2$	870	0.0005	64	81
$Br_2O_2Ti_2$	510	0.0005	40	45
Se_2Zr	711	0.0005	73	91
Br_2Zr_2	294	0.0005	25	36
MoS_2	102	0.0005	9	16
F_2Na	627	0.0005	64	81
S_2W	102	0.0006	9	16
Ga_2Se_2	634	0.0006	57	73
As_2Li_2Nd	11	0.0006	1	1
Ga_2S_2	412	0.0006	36	49
H_2Li_2Pd	930	0.0006	65	108
$Cl_2Er_2O_2$	984	0.0006	73	91
CBr_2Y_2	707	0.0007	57	73
Ge_2Se_2	882	0.0007	91	84
MoS_2	102	0.0007	9	16
FeH_2O_2	395	0.0007	25	49
PTe_2Ti_2	461	0.0007	36	49
$CoTe_2$	363	0.0008	36	49
$Cl_2Ho_2O_2$	984	0.0008	73	91
GeI_2Y_2	11	0.0008	1	1
ClH_3O	806	0.0009	81	64
Br_2Ni	363	0.0009	36	49
$AsSn_2$	486	0.0009	49	64
$SiTe_2$	711	0.0009	73	91
NiO_2	51	0.0009	4	9
Te_4W_2	870	0.0009	85	60
Cl_2Mg	363	0.0009	36	49
Bi_2STe_2	11	0.001	1	1
$I_2Lu_2S_2$	846	0.001	81	60
$Br_2Lu_2S_2$	744	0.0011	71	53
Br_3Cs	484	0.0011	64	25
HfS_2	363	0.0011	36	49

Table showing the first 50 lattice matching combinations with the smallest strain needed to build the supercell.

La_2TeI_2 (P-3m1)

Structural and electronic properties

**Formula**	La_2TeI_2
**Spacegroup**	P-3m1
**Prototype**	Bi2Te2S
**Parent 3D**	La_2TeI_2
**Source DB**	ICSD
**DB ID**	240698
**DF2-C09 Binding energy [meV/ Å^2]**	11.48
**RVV10 Binding energy [meV/ Å^2]**	17.48
**Band gap (PBE) [eV]**	N/A

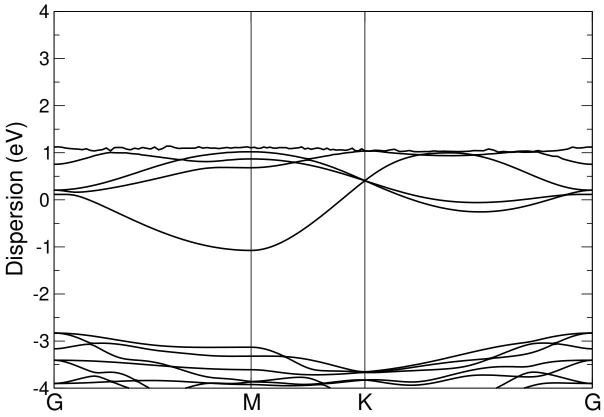

Band structure: Electronic band structure of La_2TeI_2 (P-3m1) in a representative window around the Fermi energy and along a high-symmetry path.

Optimized crystal structure

Structural parameters: relaxed cell (top) and atomic positions (bottom) of La_2TeI_2 (P-3m1) in Cartesian coordinates.

		x [Å]	y [Å]	z [Å]
$\mathbf{a_1}$		4.55276743	0.00000000	0.00000000
$\mathbf{a_2}$		−2.27638371	3.94281225	0.00000000
$\mathbf{a_3}$		0.00000000	0.00000000	27.62712781
		x [Å]	y [Å]	z [Å]
●	La	2.27638371	1.31427075	12.04396994
●	I	2.27638371	1.31427075	17.61307947
●	Te	0.00000000	0.00000000	13.81356391
●	La	0.00000000	2.62854150	15.58315788
●	I	0.00000000	2.62854150	10.01404835

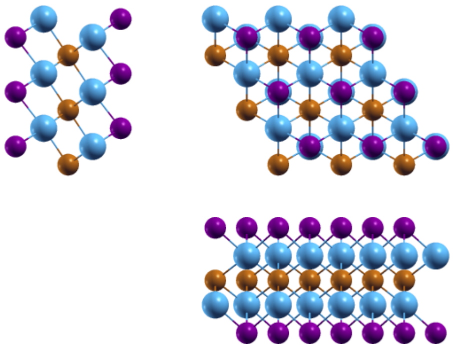

Orthographic projections: views of La_2TeI_2 (P-3m1) as seen from the x axis (left), the y axis (bottom) and the z axis (center).

Lattice matching - minimal size

Formula	N° atoms	strain	cell size 1	cell size 2
K	6	0.1489	1	1
K	6	0.0024	1	1
GeTe	7	1.5391	1	1
AgTl	7	0.1257	1	1
Ag_2	7	0.008	1	1
Sb_2	7	0.2687	1	1
$IrTe_2$	8	1.5456	1	1
$CdCl_2$	8	1.5314	1	1
Ba_2Pt	8	0.0077	1	1
CaI_2	8	0.0067	1	1
$InSe_2$	8	1.5341	1	1
$HfTe_2$	8	0.2472	1	1
I_2Pr	8	0.1157	1	1
I_2Mn	8	1.5323	1	1
I_2Yb	8	0.0091	1	1
$AuTe_2$	8	0.2576	1	1
$PdTe_2$	8	0.2542	1	1
I_2Ni	8	1.5257	1	1
I_2Zn	8	0.264	1	1
GeI_2	8	0.2743	1	1
Ba_2Hg	8	0.1359	1	1
Ba_2N	8	0.2492	1	1
Se_2Ti	8	4.8617	1	1
Te_2Zr	8	0.2478	1	1
I_2Nd	8	0.1162	1	1
Cl_2Cu	8	0.1925	1	1
I_2Tm	8	0.0078	1	1
$SnTe_2$	8	0.2709	1	1
DyI_2	8	0.0039	1	1
CeI_2	8	0.1154	1	1
Se_2Yb	8	0.2746	1	1
$BiTe_2$	8	0.275	1	1
$PtTe_2$	8	0.257	1	1
Br_2Cd	8	1.6395	1	1
I_2La	8	0.1185	1	1
F_2Zn	8	0.114	1	1
Ba_2Cd	8	0.138	1	1
NaPSn	8	1.5998	1	1
H_2Si_2	9	1.5363	1	1
Fe_2Te_2	9	0.1108	1	1
Li_2Tl_2	9	0.4594	1	1
Ca_2Cl_2	9	0.111	1	1
Cu_2I_2	9	0.1282	1	1
In_2Se_2	9	0.1704	1	1
Cu_2Te_2	9	0.1125	1	1
Ir_2P_2	9	0.1161	1	1
Ag_2Br_2	9	0.1188	1	1
Br_2Er_2	9	0.2583	1	1
O_2Sn_2	9	0.1148	1	1
S_2Sn_2	9	0.1362	1	1

Table showing the first 50 lattice matching combinations with the smallest number of atoms in the supercell.

Lattice matching - minimal strain

Formula	N° atoms	strain	cell size 1	cell size 2
Ga_2Se_2	376	0.0	36	49
MoS_2	545	0.0	49	100
S_2W	545	0.0	49	100
$SiTe_2$	233	0.0001	25	36
Ba_2N	437	0.0001	49	64
As_2Sn_2	644	0.0001	64	81
Bi_2S_3	650	0.0001	57	73
Ga_2S_2	180	0.0001	16	25
Br_2Gd_2	644	0.0002	64	81
Br_2Ho_2	577	0.0002	57	73
NaO_4	650	0.0002	73	57
MoS_2	545	0.0002	49	100
$IrTe_2$	327	0.0002	36	49
Br_2Ca_3Si	11	0.0002	1	1
PTe_2Ti_2	205	0.0002	16	25
I_2Zn	638	0.0002	73	91
$Cl_2Er_2O_2$	341	0.0003	25	36
Cl_4KTl	974	0.0003	130	54
Cu_3Se_3	629	0.0003	49	64
S_2Ti	93	0.0003	9	16
Br_2Tb_2	577	0.0003	57	73
Na	246	0.0003	37	61
Br_2Ni	155	0.0003	16	25
$ClKO_3$	65	0.0004	9	4
Cl_2Mg	155	0.0004	16	25
$GeNi_3Te_2$	557	0.0004	43	57
H_2NiO_2	590	0.0004	39	79
Se_2Zr	233	0.0005	25	36
S_2	278	0.0005	36	49
Br_2La_2	805	0.0005	81	100
$NiTe_2$	233	0.0005	25	36
AuI_4Li	681	0.0006	81	46
Ni_2SbTe_2	425	0.0006	36	49
Se_2Ti	488	0.0006	49	81
GeS_2	759	0.0007	81	118
Cl_2Co	93	0.0007	9	16
Li_2Tl_2	824	0.0007	100	81
Br_2Er_2	644	0.0007	64	81
Ga_2S_3	490	0.0007	37	61
$Cl_2H_2Lu_2$	230	0.0007	16	25
C_2F_2	276	0.0007	16	49
Sb_2	605	0.0007	81	100
Sn	275	0.0007	39	80
S_2V	545	0.0008	49	100
NS_2Zr	429	0.0008	37	61
CdO_2	93	0.0008	9	16
In_2S_3	425	0.0009	36	49
$PdTe_2$	504	0.0009	57	73
As_2Fe_2	877	0.0009	81	118
O_2Sn_2	904	0.001	84	121

Table showing the first 50 lattice matching combinations with the smallest strain needed to build the supercell.

$LaBr_2$ (P-6m2)

Structural and electronic properties

**Formula**	$LaBr_2$
**Spacegroup**	P-6m2
**Prototype**	MoS2
**Parent 3D**	La_2Br_4
**Source DB**	ICSD
**DB ID**	65481
**DF2-C09 Binding energy [meV/ Å^2]**	11.15
**RVV10 Binding energy [meV/ Å^2]**	16.81
**Band gap (PBE) [eV]**	0.62

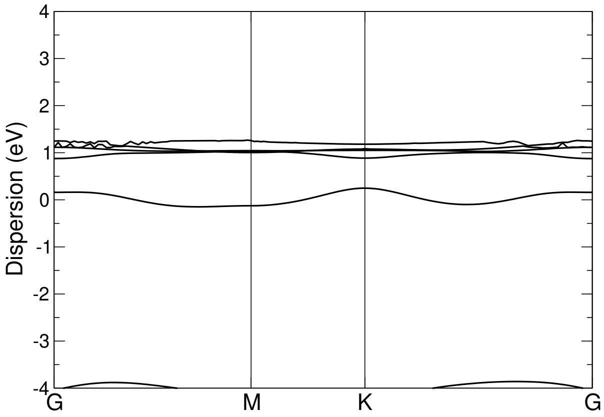

Band structure: Electronic band structure of $LaBr_2$ (P-6m2) in a representative window around the Fermi energy and along a high-symmetry path.

Optimized crystal structure

Structural parameters: relaxed cell (top) and atomic positions (bottom) of $LaBr_2$ (P-6m2) in Cartesian coordinates.

		x [Å]	y [Å]	z [Å]
$\mathbf{a_1}$		4.15651452	0.00000000	0.00000000
$\mathbf{a_2}$		−2.07825726	3.59964717	0.00000000
$\mathbf{a_3}$		0.00000000	0.00000000	23.83572073
		x [Å]	y [Å]	z [Å]
●	La	0.00000000	0.00000000	11.91786036
●	Br	2.07825726	1.19988239	13.82221810
●	Br	2.07825726	1.19988239	10.01350263

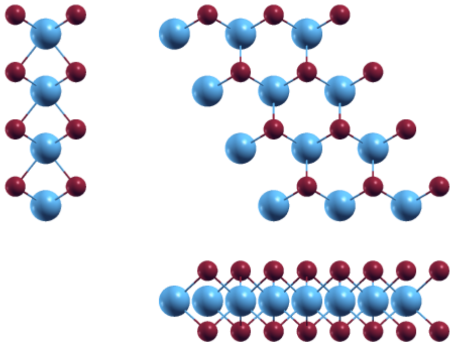

Orthographic projections: views of $LaBr_2$ (P-6m2) as seen from the x axis (left), the y axis (bottom) and the z axis (center).

Lattice matching - minimal size

Formula	N° atoms	strain	cell size 1	cell size 2
K	4	0.4504	1	1
Na	4	1.5333	1	1
AgTl	5	0.1627	1	1
Ag_2	5	0.464	1	1
As_2	5	1.5909	1	1
Sb_2	5	0.0059	1	1
CaCl	5	0.1179	1	1
Cl_2Zn	6	1.5832	1	1
I_2Mg	6	0.0003	1	1
$MoTe_2$	6	1.5494	1	1
PSn_2	6	0.2563	1	1
Ba_2Pt	6	0.4633	1	1
$ReSe_2$	6	1.4472	1	1
Br_2Zn	6	0.2714	1	1
HfS_2	6	0.248	1	1
$AsSn_2$	6	0.2615	1	1
Te_2V	6	1.5633	1	1
I_2Pr	6	0.1449	1	1
$CuTe_2$	6	0.2475	1	1
S_2Zr	6	0.2552	1	1
Ca_2Si	6	0.4748	1	1
Br_2Co	6	1.5878	1	1
Ca_2N	6	1.5948	1	1
BrCdI	6	0.0028	1	1
Cl_2Zn	6	0.1262	1	1
Te_2Ti	6	0.2718	1	1
I_2Zn	6	0.0091	1	1
BaF_2	6	0.0062	1	1
$RhTe_2$	6	0.2655	1	1
GeI_2	6	0.0022	1	1
PtS_2	6	1.5406	1	1
$CoTe_2$	6	0.2484	1	1
CdClO	6	1.5598	1	1
Se_2Ti	6	1.5218	1	1
AsKSn	6	0.0047	1	1
Te_2W	6	1.5506	1	1
$PbTe_2$	6	0.0018	1	1
I_2Nd	6	0.1458	1	1
S_2Sn	6	0.2556	1	1
$SnTe_2$	6	0.0044	1	1
Sn	6	0.6263	1	3
I_2Pb	6	0.4679	1	1
STl_2	6	0.0072	1	1
$PtSe_2$	6	0.2624	1	1
OTl_2	6	1.5611	1	1
Br_2Fe	6	1.5883	1	1
GeS_2	6	0.1126	1	1
$TaTe_2$	6	0.2607	1	1
$MnSe_2$	6	0.1178	1	1
Br_2Ni	6	0.2506	1	1

Table showing the first 50 lattice matching combinations with the smallest number of atoms in the supercell.

Lattice matching - minimal strain

Formula	N° atoms	strain	cell size 1	cell size 2
CdClHO	583	0.0	73	91
Bi_2Se_3	8	0.0001	1	1
S_2Sn	390	0.0001	57	73
$HfLiS_2$	304	0.0001	36	49
Cl_2Ni	183	0.0001	25	36
$Br_2N_2Ti_2$	711	0.0001	73	82
LiO	309	0.0002	49	81
S_2Zr	390	0.0002	57	73
Te_2Zn	255	0.0002	36	49
Au_2Se_2	349	0.0002	63	40
As_2	243	0.0002	43	57
$F_2Se_2Y_2$	363	0.0002	49	36
Se_2V	123	0.0003	16	25
Gd_2I_2	7	0.0003	1	1
$NbSe_2$	183	0.0003	25	36
CCl_2Lu_2	414	0.0003	43	57
Ca_2N	300	0.0003	43	57
I_2Mg	6	0.0003	1	1
$MoTe_2$	255	0.0004	36	49
$I_2N_2Zr_2$	843	0.0004	81	100
$NbTe_2$	390	0.0004	57	73
Cl_2OV	89	0.0004	11	14
Se_2Ta	183	0.0004	25	36
I_2Pb	492	0.0004	91	73
$I_2N_2Ti_2$	846	0.0004	90	96
CdClHO	516	0.0004	64	81
$Hf_2I_2N_2$	678	0.0004	64	81
$I_2Pr_2S_2$	711	0.0004	91	73
$Ce_2I_2S_2$	627	0.0005	81	64
Ca_2Si	435	0.0005	81	64
Ga_2S_2	403	0.0005	49	64
Te_2W	255	0.0005	36	49
$HNiO_2$	271	0.0005	25	49
Br_2Fe	300	0.0006	43	57
$Ce_2I_2Si_2$	9	0.0006	1	1
N_3W_2	647	0.0006	49	100
Ga_2S_2	403	0.0006	49	64
PSn_2	390	0.0006	57	73
Tl	73	0.0006	16	25
C_2Li_2	276	0.0006	40	39
Cu_2I_2	7	0.0006	1	1
Br_2Gd_2Ge	8	0.0006	1	1
Br_2Co	300	0.0006	43	57
$CoTe_2$	339	0.0006	49	64
Ga_2Se_2	643	0.0006	81	100
Mg_6	951	0.0007	81	118
$Br_2Hf_2N_2$	438	0.0008	48	49
LiO	233	0.0008	37	61
$RhTe_2$	492	0.0008	73	91
Ba_2Pt	543	0.0009	100	81

Table showing the first 50 lattice matching combinations with the smallest strain needed to build the supercell.

LaCl (P-3m1)

Structural and electronic properties

**Formula**	LaCl
**Spacegroup**	P-3m1
**Prototype**	ZrCl
**Parent 3D**	La_2Cl_2
**Source DB**	ICSD
**DB ID**	24410
**DF2-C09 Binding energy [meV/ Å^2]**	11.04
**RVV10 Binding energy [meV/ Å^2]**	16.83
**Band gap (PBE) [eV]**	0.01

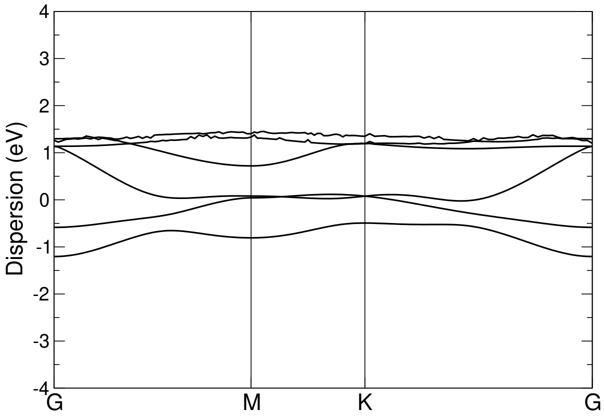

Band structure: Electronic band structure of LaCl (P-3m1) in a representative window around the Fermi energy and along a high-symmetry path.

Optimized crystal structure

Structural parameters: relaxed cell (top) and atomic positions (bottom) of LaCl (P-3m1) in Cartesian coordinates.

		x [Å]	y [Å]	z [Å]
$\mathbf{a_1}$		4.05547926	0.00000000	0.00000000
$\mathbf{a_2}$		−2.02773963	3.51214806	0.00000000
$\mathbf{a_3}$		0.00000000	0.00000000	26.57420659
		x [Å]	y [Å]	z [Å]
●	La	2.02773963	1.17071602	14.77661346
●	Cl	0.00000000	-0.00000000	10.02837205
●	Cl	0.00000000	-0.00000000	16.54583454
●	La	0.00000000	2.34143204	11.79759313

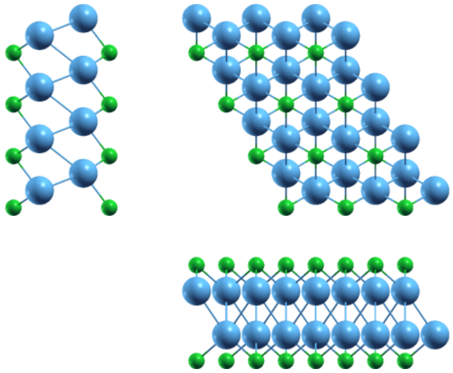

Orthographic projections: views of LaCl (P-3m1) as seen from the x axis (left), the y axis (bottom) and the z axis (center).

Lattice matching - minimal size

Formula	N° atoms	strain	cell size 1	cell size 2
K	5	0.4796	1	1
Na	5	0.2492	1	1
Ag_2	6	0.4939	1	1
As_2	6	0.26	1	1
Sb_2	6	0.0057	1	1
CaCl	6	0.1244	1	1
Cl_2Zn	7	0.2585	1	1
$MoTe_2$	7	1.6351	1	1
PSn_2	7	0.2728	1	1
Ba_2Pt	7	0.4932	1	1
$ReSe_2$	7	1.5277	1	1
HfS_2	7	0.2638	1	1
CaI_2	7	0.4564	1	1
Te_2V	7	0.2548	1	1
$CuTe_2$	7	0.2633	1	1
S_2Zr	7	0.2716	1	1
Ca_2Si	7	2.9171	1	1
I_2Yb	7	0.4502	1	1
Br_2Co	7	0.2594	1	1
Ca_2N	7	0.2607	1	1
$AuTe_2$	7	0.0022	1	1
Cl_2Zn	7	0.1345	1	1
$PdTe_2$	7	0.0046	1	1
NbS_2	7	1.4481	1	1
I_2Zn	7	0.0023	1	1
GeI_2	7	0.0096	1	1
Br_2Mn	7	0.2569	1	1
Cl_2Ni	7	1.5341	1	1
PtS_2	7	0.2506	1	1
$CoTe_2$	7	0.2643	1	1
Br_2V	7	1.5219	1	1
CdClO	7	0.2542	1	1
Ba_2N	7	0.0083	1	1
Se_2Ti	7	0.2471	1	1
Te_2W	7	1.6364	1	1
I_2Tm	7	0.4535	1	1
S_2Sn	7	0.272	1	1
$SnTe_2$	7	0.0073	1	1
I_2Pb	7	0.4981	1	1
OTl_2	7	0.2544	1	1
BrNZr	7	1.5541	1	1
$NbSe_2$	7	1.5371	1	1
Br_2Fe	7	0.2595	1	1
GeS_2	7	0.1176	1	1
$MnSe_2$	7	0.1243	1	1
DyI_2	7	0.4634	1	1
Br_2Ni	7	0.2667	1	1
Se_2Ta	7	1.5378	1	1
$NbTe_2$	7	0.2713	1	1
$NbSe_2$	7	1.5465	1	1

Table showing the first 50 lattice matching combinations with the smallest number of atoms in the supercell.

Lattice matching - minimal strain

Formula	N° atoms	strain	cell size 1	cell size 2
O_2Zn	84	0.0	9	16
$CoTe_2$	565	0.0	73	91
Na	260	0.0	49	64
$NbSe_2$	291	0.0001	36	49
Br_2Co	499	0.0001	64	81
Ga_2S_2	656	0.0001	73	91
Br_2Fe	499	0.0001	64	81
Br_2Ca_3Si	738	0.0002	84	67
$I_2Pr_2S_2$	550	0.0002	64	49
Ca_2Si	357	0.0002	57	43
$Ce_2I_2S_2$	486	0.0002	57	43
$Dy_2I_2S_2$	886	0.0002	100	81
Br_2Hg_3	120	0.0002	25	4
In	209	0.0003	37	61
$Cl_2H_2Lu_2$	924	0.0003	81	100
Ga_2S_3	516	0.0003	49	64
$AsSe_2$	291	0.0003	36	49
HfS_2	565	0.0003	73	91
$LiNbS_2$	244	0.0003	25	36
CCl_2Lu_2	661	0.0004	64	81
NS_2Zr	452	0.0004	49	64
S_2Ta	208	0.0004	25	36
Te_2V	447	0.0005	57	73
As_2	418	0.0005	64	81
Cl_2Zn	499	0.0005	64	81
Ag_2	354	0.0006	64	49
IKO_3	276	0.0006	49	16
Nd	25	0.0006	4	9
Br_2Hf_2	340	0.0006	36	49
Cl_2Mg	624	0.0006	81	100
O_2Pt	439	0.0006	49	81
Se_2Sn_2	668	0.0006	95	72
Br_2Ni	624	0.0006	81	100
$Er_2I_2Se_2$	684	0.0007	90	54
$SbSe_2Tl$	492	0.0007	75	48
$Ho_2I_2Se_2$	622	0.0007	82	49
$CuTe_2$	565	0.0007	73	91
Mg_3	678	0.0008	81	118
$Cl_2Hf_2N_2$	666	0.0008	57	73
OTl_2	447	0.0008	57	73
Cl_2Hf_2	244	0.0008	25	36
PTe_2Ti_2	824	0.0008	81	100
Ba_2Pt	403	0.0008	64	49
O_4PSn	958	0.0008	118	81
NbS_2	208	0.0009	25	36
Br_2Ca_3Si	708	0.0009	81	64
DyI_2	643	0.0009	100	81
$Cl_2N_2Ti_2$	974	0.0009	92	101
$Br_2N_2Ti_2$	468	0.0009	45	48
$I_2S_2Tb_2$	802	0.0009	91	73

Table showing the first 50 lattice matching combinations with the smallest strain needed to build the supercell.

LaGeI (P-3m1)

Structural and electronic properties

**Formula**	LaGeI
**Spacegroup**	P-3m1
**Prototype**	Ce2Si2I2
**Parent 3D**	$La_2Ge_2I_2$
**Source DB**	ICSD
**DB ID**	59801
**DF2-C09 Binding energy [meV/ Å^2]**	11.98
**RVV10 Binding energy [meV/ Å^2]**	17.89
**Band gap (PBE) [eV]**	N/A

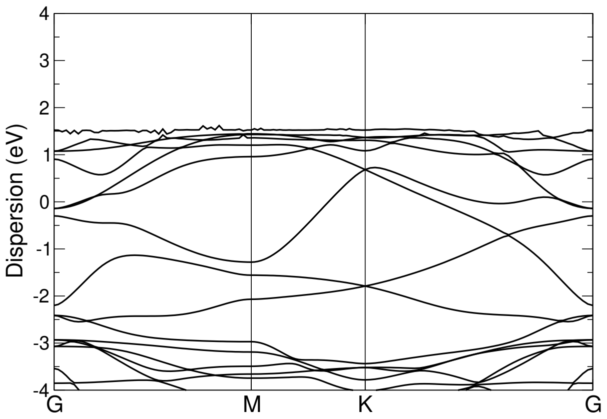

Band structure: Electronic band structure of LaGeI (P-3m1) in a representative window around the Fermi energy and along a high-symmetry path.

Optimized crystal structure

Structural parameters: relaxed cell (top) and atomic positions (bottom) of LaGeI (P-3m1) in Cartesian coordinates.

	x [Å]	y [Å]	z [Å]
$\mathbf{a_1}$	4.43445741	0.00000000	0.00000000
$\mathbf{a_2}$	−2.21722870	3.84035277	0.00000000
$\mathbf{a_3}$	0.00000000	0.00000000	28.31243107

	x [Å]	y [Å]	z [Å]
La	0.00000000	0.00000000	12.11070867
Ge	2.21722870	1.28011759	14.02493304
I	0.00000000	2.56023518	10.01063186
La	0.00000000	0.00000000	16.20172241
Ge	0.00000000	2.56023518	14.28749803
I	2.21722870	1.28011759	18.30179921

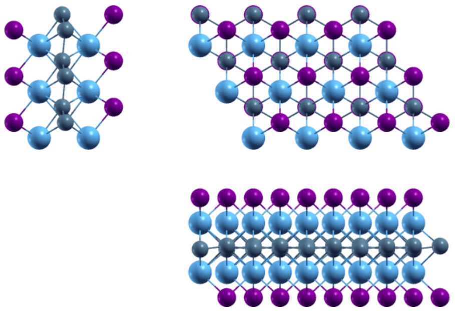

Orthographic projections: views of LaGeI (P-3m1) as seen from the x axis (left), the y axis (bottom) and the z axis (center).

Lattice matching - minimal size

Formula	N° atoms	strain	cell size 1	cell size 2
GeTe	8	1.6304	1	1
AgTl	8	0.1345	1	1
S_2	8	0.2535	1	1
$IrTe_2$	9	1.6372	1	1
$CdCl_2$	9	0.25	1	1
CaI_2	9	0.0057	1	1
$InSe_2$	9	0.2505	1	1
$GeTe_2$	9	0.2486	1	1
$SiTe_2$	9	1.5411	1	1
$HfTe_2$	9	0.2641	1	1
I_2Pr	9	0.1222	1	1
I_2Mn	9	0.2501	1	1
Br_2Cu	9	0.6475	1	1
NSr_2	9	1.5972	1	1
I_2Yb	9	0.0031	1	1
Tl	9	0.6322	1	3
$AuTe_2$	9	0.2754	1	1
Cl_2Zn	9	0.1112	1	1
$PdTe_2$	9	0.2718	1	1
FeI_2	9	0.2472	1	1
I_2Ni	9	0.2489	1	1
CrI_2	9	0.2466	1	1
Ba_2Hg	9	0.1466	1	1
Br_2V	9	4.8794	1	1
Ba_2N	9	0.2663	1	1
Te_2Zr	9	0.2648	1	1
I_2Nd	9	0.1228	1	1
$NiTe_2$	9	1.5368	1	1
I_2Tm	9	0.0045	1	1
I_2V	9	1.5507	1	1
Se_2Zr	9	1.544	1	1
BiITe	9	0.0046	1	1
CoI_2	9	1.5884	1	1
DyI_2	9	0.0086	1	1
CeI_2	9	0.1217	1	1
Br_2Mg	9	0.247	1	1
GdI_2	9	0.0082	1	1
$PtTe_2$	9	0.2748	1	1
Br_2Cd	9	0.2704	1	1
I_2La	9	0.1257	1	1
Se_2Sn	9	1.5954	1	1
F_2Zn	9	0.12	1	1
Ba_2Cd	9	0.149	1	1
NaPSn	9	0.2628	1	1
H_2Si_2	10	1.6275	1	1
Fe_2Te_2	10	0.1156	1	1
Li_2Tl_2	10	2.8493	1	1
Ca_2Cl_2	10	0.1158	1	1
Cu_2I_2	10	0.1376	1	1
Cl_2Gd_2	10	0.2565	1	1

Table showing the first 50 lattice matching combinations with the smallest number of atoms in the supercell.

Lattice matching - minimal strain

Formula	N° atoms	strain	cell size 1	cell size 2
$F_2Se_2Tm_2$	870	0.0001	64	81
Na	121	0.0001	16	25
In_2Se_3	893	0.0001	73	91
S_2Zn_2	634	0.0002	57	73
$HfTe_2$	711	0.0002	73	91
Se_2V	102	0.0002	9	16
$LiMnSe_2$	708	0.0002	64	81
I_2Ti	429	0.0002	43	57
Br_2V	537	0.0002	49	81
CI_2Y_2	543	0.0002	43	57
ReS_2	594	0.0003	49	100
I_2Ni	486	0.0003	49	64
Te_2Zr	711	0.0003	73	91
Ni_2Te_2	550	0.0003	49	64
CdClHO	294	0.0003	25	36
$Hf_2I_2N_2$	366	0.0003	25	36
$ReSe_2$	405	0.0004	37	61
$F_2Se_2Yb_2$	780	0.0004	57	73
Se_2Zr	363	0.0004	36	49
$Er_2F_2Se_2$	984	0.0004	73	91
Se_2Sn	429	0.0004	43	57
Br_2V	405	0.0004	37	61
C_2	312	0.0005	25	81
Ga_2S_3	221	0.0005	16	25
$GeTe_2$	486	0.0005	49	64
P_2Sn_2	634	0.0005	57	73
NS_2Zr	196	0.0005	16	25
I_2V	363	0.0005	36	49
CoI_2	429	0.0005	43	57
$CdCl_2$	486	0.0005	49	64
$Cl_2Er_2O_2$	510	0.0006	36	49
Cl_2Zr_2	618	0.0006	49	81
I_3Sn	314	0.0006	39	20
I_2Mn	486	0.0007	49	64
NSr_2	429	0.0007	43	57
Tl	70	0.0007	9	16
IKO_3	509	0.0007	64	25
H_2Li_2Pt	778	0.0007	53	92
N_3Na	914	0.0007	97	83
Cl_2Gd_2	634	0.0008	57	73
$Cl_2Er_2H_2$	678	0.0008	49	64
I_2Y_2	708	0.0008	64	81
$SiTe_2$	363	0.0008	36	49
$Cl_2Ho_2O_2$	510	0.0009	36	49
Pt_2Te_2	802	0.0009	73	91
$TaTe_2$	258	0.0009	25	36
$InSe_2$	486	0.0009	49	64
Ba_2N	786	0.0009	81	100
$AgNO_3$	474	0.0009	49	36
AsSb	372	0.001	43	57

Table showing the first 50 lattice matching combinations with the smallest strain needed to build the supercell.

$LaHBr_2$ (P-6m2)

Structural and electronic properties

**Formula**	$LaHBr_2$
**Spacegroup**	P-6m2
**Prototype**	AsCuLi2
**Parent 3D**	$Br_4H_2La_2$
**Source DB**	MPDS
**DB ID**	S1707575
**DF2-C09 Binding energy [meV/ Å^2]**	10.58
**RVV10 Binding energy [meV/ Å^2]**	N/A
**Band gap (PBE) [eV]**	3.78

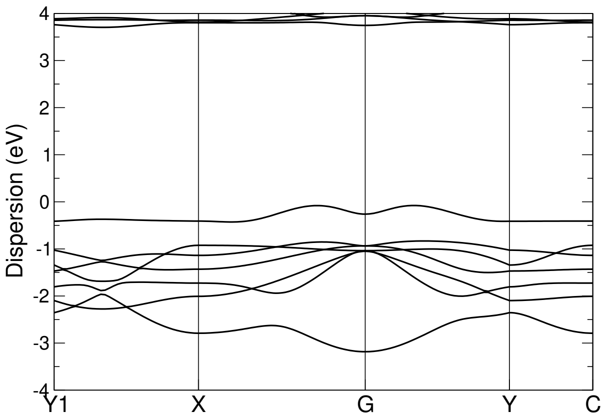

Band structure: Electronic band structure of $LaHBr_2$ (P-6m2) in a representative window around the Fermi energy and along a high-symmetry path.

Optimized crystal structure

Structural parameters: relaxed cell (top) and atomic positions (bottom) of $LaHBr_2$ (P-6m2) in Cartesian coordinates.

		x [Å]	y [Å]	z [Å]
$\mathbf{a_1}$		2.06454777	−3.57590164	0.00000000
$\mathbf{a_2}$		4.12909555	0.00000000	0.00000000
$\mathbf{a_3}$		0.00000000	0.00000000	19.78006031
		x [Å]	y [Å]	z [Å]
	La	0.00000000	-0.00000000	0.00000000
	H	-2.06454777	-1.19196721	0.00000000
	Br	0.00000000	-2.38393443	-1.95443406
	Br	0.00000000	-2.38393443	1.95443406

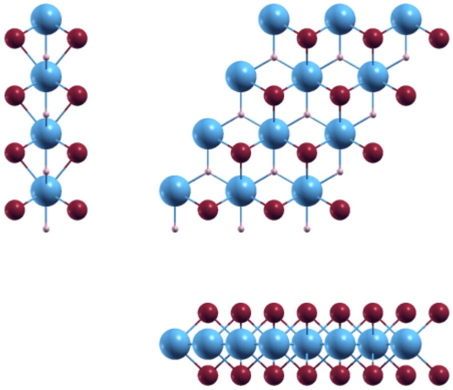

Orthographic projections: views of $LaHBr_2$ (P-6m2) as seen from the x axis (left), the y axis (bottom) and the z axis (center).

Lattice matching - minimal size

Formula	N° atoms	strain	cell size 1	cell size 2
K	5	0.4581	1	1
Na	5	1.5557	1	1
AgTl	6	0.1661	1	1
Ag_2	6	0.4719	1	1
As_2	6	0.2485	1	1
Sb_2	6	0.0029	1	1
CaCl	6	0.1195	1	1
Cl_2Zn	7	0.2472	1	1
I_2Mg	7	0.0028	1	1
PSn_2	7	0.2606	1	1
Ba_2Pt	7	0.4712	1	1
Br_2Zn	7	0.276	1	1
HfS_2	7	1.6347	1	1
$AsSn_2$	7	0.2659	1	1
Te_2V	7	1.5862	1	1
I_2Pr	7	0.1478	1	1
S_2Zr	7	0.2595	1	1
Br_2La	7	0.0031	1	1
Ca_2Si	7	0.4828	1	1
Br_2Co	7	0.248	1	1
Ca_2N	7	0.2492	1	1
BrCdI	7	0.006	1	1
Cl_2Zn	7	0.1283	1	1
I_2Zn	7	0.0061	1	1
BaF_2	7	0.0094	1	1
$RhTe_2$	7	0.27	1	1
GeI_2	7	0.0009	1	1
Br_2Mn	7	1.5974	1	1
PtS_2	7	1.5631	1	1
$CoTe_2$	7	1.6371	1	1
ClNZr	7	1.4486	1	1
CdClO	7	1.5826	1	1
Se_2Ti	7	1.5441	1	1
AsKSn	7	0.0079	1	1
$PbTe_2$	7	0.005	1	1
I_2Nd	7	0.1487	1	1
S_2Sn	7	0.2599	1	1
$SnTe_2$	7	0.0013	1	1
Sn	7	0.6366	1	3
I_2Pb	7	0.4759	1	1
$PtSe_2$	7	0.2668	1	1
OTl_2	7	1.5839	1	1
Br_2Fe	7	0.248	1	1
GeS_2	7	0.1138	1	1
$TaTe_2$	7	0.2651	1	1
$MnSe_2$	7	0.1194	1	1
Br_2Ni	7	0.2548	1	1
CeI_2	7	0.1471	1	1
CuO_2	7	0.1757	1	1
$NbTe_2$	7	0.2592	1	1

Table showing the first 50 lattice matching combinations with the smallest number of atoms in the supercell.

Lattice matching - minimal strain

Formula	N° atoms	strain	cell size 1	cell size 2
$Hf_2I_2N_2$	838	0.0	73	91
CdClHO	656	0.0	73	91
Ca_2N	388	0.0	49	64
I_2Pb	516	0.0	81	64
$NbTe_2$	499	0.0001	64	81
$Cl_2H_2Lu_2$	666	0.0001	57	73
CCl_2Sc_2	280	0.0001	25	36
$Ce_2I_2S_2$	634	0.0001	73	57
S_2Zr	499	0.0002	64	81
Ca_2Si	463	0.0002	73	57
CoH_2O_2	116	0.0002	9	16
$AgNO_3$	180	0.0002	25	16
In	368	0.0002	65	108
Cl_2V	331	0.0002	37	61
Ho_2S_2	316	0.0002	40	39
$I_2Nd_2S_2$	802	0.0002	91	73
$Br_2H_2Zr_2$	316	0.0002	25	36
$CeLi_2P_2$	9	0.0004	1	1
Se_2Ti	291	0.0004	36	49
Te_4W_2	672	0.0004	84	56
S_2Sn	499	0.0004	64	81
Cl_2Mg	447	0.0004	57	73
$AsCuLi_2$	8	0.0005	1	1
Br_2Ni	447	0.0005	57	73
Cl_2Zr_2	244	0.0005	25	36
$PtSe_2$	624	0.0005	81	100
$TaTe_2$	565	0.0005	73	91
As_2	324	0.0006	49	64
$Br_2Hf_2N_2$	580	0.0006	49	64
PTe_2Ti_2	593	0.0006	57	73
$I_2Pr_2Si_2$	10	0.0006	1	1
$AgTe_2$	678	0.0006	81	118
HgI_2	404	0.0006	65	48
FHOZn	520	0.0006	49	81
Cu_2Te_2	740	0.0006	87	98
NaO_4	180	0.0006	25	16
Br_2Mn	343	0.0007	43	57
CCl_2Lu_2	516	0.0007	49	64
Bi_2In_2	796	0.0007	118	81
Ga_2Te_2	8	0.0007	1	1
Ba_2Ni_3	9	0.0007	1	1
CrO_2	43	0.0007	4	9
$CrSe_2$	439	0.0008	49	81
Br_2Zr_2	340	0.0008	36	49
I_4Sr_2	714	0.0008	114	43
ClNZr	208	0.0008	25	36
Te_2V	343	0.0008	43	57
ReS_2	84	0.0009	9	16
I_2La_2Sb	805	0.0009	100	81
$I_2N_2Ti_2$	618	0.0009	60	63

Table showing the first 50 lattice matching combinations with the smallest strain needed to build the supercell.

LaI_2 (P4/mmm)

Structural and electronic properties

**Formula**	LaI_2
**Spacegroup**	P4/mmm
**Prototype**	Zr2Cu
**Parent 3D**	LaI_2
**Source DB**	COD
**DB ID**	1529708
**DF2-C09 Binding energy [meV/ Å^2]**	15.63
**RVV10 Binding energy [meV/ Å^2]**	21.19
**Band gap (PBE) [eV]**	N/A

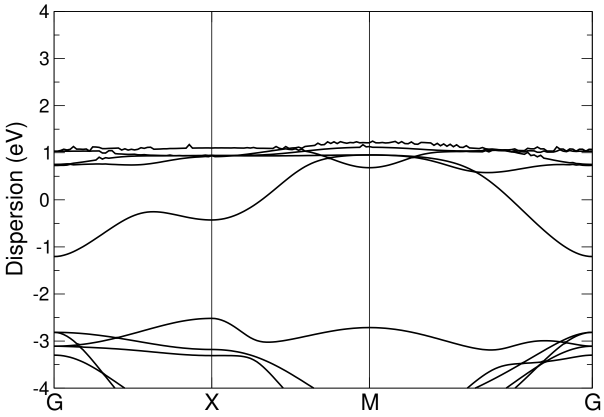

Band structure: Electronic band structure of LaI_2 (P4/mmm) in a representative window around the Fermi energy and along a high-symmetry path.

Optimized crystal structure

Structural parameters: relaxed cell (top) and atomic positions (bottom) of LaI_2 (P4/mmm) in Cartesian coordinates.

		x [Å]	y [Å]	z [Å]
$\mathbf{a_1}$		3.95359285	0.00000000	0.00000000
$\mathbf{a_2}$		0.00000000	3.95359285	0.00000000
$\mathbf{a_3}$		0.00000000	0.00000000	23.84174384
		x [Å]	y [Å]	z [Å]
●	I	1.97679643	1.97679643	10.01994011
●	I	1.97679643	1.97679643	13.82180372
●	La	0.00000000	0.00000000	11.92087192

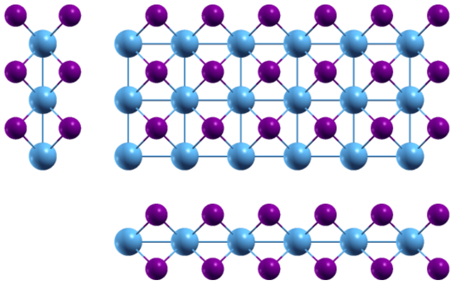

Orthographic projections: views of LaI_2 (P4/mmm) as seen from the x axis (left), the y axis (bottom) and the z axis (center).

Lattice matching - minimal size

Formula	N° atoms	strain	cell size 1	cell size 2
K	4	0.177	1	1
InSe	5	0.1406	1	1
Bi_2	5	0.1457	1	1
AgTl	5	0.0177	1	1
Ag_2	5	0.1837	1	1
LiO	5	0.111	1	1
P_2	5	0.1093	1	1
PbTe	5	0.1421	1	1
I_2Mg	6	0.1323	1	1
CdI_2	6	0.1436	1	1
Nd	6	0.1914	1	3
Ba_2Pt	6	0.1834	1	1
Br_2Zn	6	0.109	1	1
Br_2Ca	6	0.1447	1	1
CaI_2	6	0.1664	1	1
I_2Pr	6	0.0056	1	1
Br_2La	6	0.1326	1	1
Br_2Cu	6	0.1031	1	1
Ca_2Si	6	0.1892	1	1
I_2Yb	6	0.1636	1	1
BiClTe	6	0.1439	1	1
Cl_2Ti	6	0.1093	1	1
BrCdI	6	0.1347	1	1
HgI_2	6	0.402	1	1
Te_2Ti	6	0.1091	1	1
BaF_2	6	0.1374	1	1
BiBrTe	6	0.149	1	1
GeI_2	6	0.131	1	1
AsKSn	6	0.1362	1	1
$PbTe_2$	6	0.134	1	1
I_2Nd	6	0.0047	1	1
Cl_2Cu	6	0.0975	1	1
I_2Tm	6	0.1651	1	1
$SnTe_2$	6	0.1294	1	1
GeI_2	6	0.1423	1	1
I_2Pb	6	0.1857	1	1
STl_2	6	0.1382	1	1
BiITe	6	0.1554	1	1
GeS_2	6	0.2119	1	1
DyI_2	6	0.1696	1	1
CeI_2	6	0.0064	1	1
Se_2Yb	6	0.1312	1	1
$BiTe_2$	6	0.1313	1	1
GdI_2	6	0.1517	1	1
$CrSe_2$	6	0.1113	1	1
CdI_2	6	0.1432	1	1
I_2Pr	6	0.144	1	1
$HfSe_2$	6	0.1091	1	1
Bi_2Te_2	7	0.7762	1	1
Bi_2In_2	7	1.1751	1	1

Table showing the first 50 lattice matching combinations with the smallest number of atoms in the supercell.

Lattice matching - minimal strain

Formula	N° atoms	strain	cell size 1	cell size 2
Mg_3	882	0.0	113	181
Tl	73	0.0001	16	25
As_2Fe_2	516	0.0001	64	81
HgI_2	774	0.0001	145	113
$Hf_2Se_2Si_2$	687	0.0001	65	82
O_2Zn	177	0.0002	20	39
Mg_2	368	0.0002	58	97
Br_2CsF	291	0.0002	49	36
As_2Rh_2	7	0.0003	1	1
Mg_4	148	0.0003	16	25
$Ba_2F_2I_2$	486	0.0004	64	49
$Cl_2H_2Lu_2$	534	0.0004	48	65
H_4Ti	552	0.0005	49	81
Ag_2Br_2	7	0.0005	1	1
O_4PTl	486	0.0006	64	49
$H_2Li_2O_2$	852	0.0006	82	101
$Hf_2Se_2Si_2$	678	0.0006	64	81
$H_2I_2Yb_2$	9	0.0006	1	1
Se_2Ta_4	402	0.0006	36	49
GeS_2	435	0.0007	64	81
Cl_2Mg	339	0.0007	48	65
Br_2Ni	339	0.0008	48	65
$Br_2Lu_2S_2$	30	0.0009	4	3
Cl_4KTl	129	0.0009	25	9
O_2Sn_2	818	0.0009	102	128
$Br_2Lu_2S_2$	30	0.0009	4	3
$Cu_2K_2Te_2$	849	0.0009	113	85
Ag_2I_2	887	0.0009	145	113
PTe_2Ti_2	469	0.0009	48	65
O_4PSn	849	0.0009	113	85
Bi_2Se_4	990	0.0009	170	80
$Br_2O_2Pr_2$	9	0.001	1	1
Ho_2S_2	754	0.001	106	109
O_2Sn_2	818	0.001	102	128
H_2Li_2Pd	552	0.001	49	81
Bi_2Se_4	186	0.001	32	15
Cl_2NSc_2	867	0.001	79	126
Cl_2Mn	615	0.0011	79	126
P_4	249	0.0011	35	36
$H_2Li_2O_2$	843	0.0011	81	100
$Se_2Si_2Zr_2$	843	0.0012	81	100
$Br_2N_2Zr_2$	534	0.0012	48	65
Ga_2S_2	404	0.0012	48	65
CBr_2Lu_2	469	0.0012	48	65
C_4Ca_2	750	0.0013	88	81
Te_4W_2	462	0.0013	64	45
$Br_2Nd_2O_2$	9	0.0014	1	1
$I_2S_2Sm_2$	843	0.0014	103	89
Br_2Cu	90	0.0015	14	16
F_4Pb	705	0.0015	100	81

Table showing the first 50 lattice matching combinations with the smallest strain needed to build the supercell.

LaOBr (P4/nmm)

Structural and electronic properties

**Formula**	LaOBr
**Spacegroup**	P4/nmm
**Prototype**	PbClF
**Parent 3D**	$La_2O_2Br_2$
**Source DB**	COD
**DB ID**	1530049
**DF2-C09 Binding energy [meV/ Å^2]**	29.9
**RVV10 Binding energy [meV/ Å^2]**	36.2
**Band gap (PBE) [eV]**	4.03

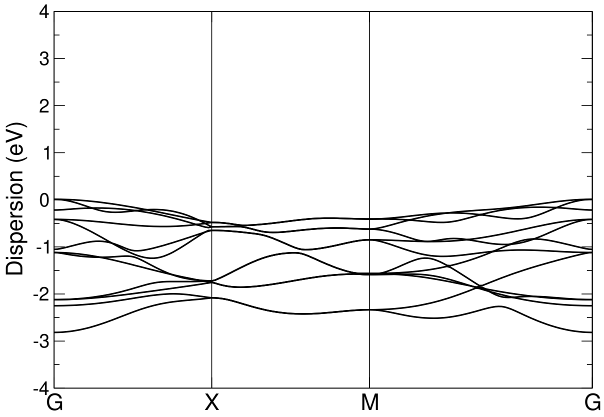

Band structure: Electronic band structure of LaOBr (P4/nmm) in a representative window around the Fermi energy and along a high-symmetry path.

Optimized crystal structure

Structural parameters: relaxed cell (top) and atomic positions (bottom) of LaOBr (P4/nmm) in Cartesian coordinates.

	x [Å]	y [Å]	z [Å]
$\mathbf{a_1}$	4.07011932	0.00000000	0.00000000
$\mathbf{a_2}$	0.00000000	4.07011932	0.00000000
$\mathbf{a_3}$	0.00000000	0.00000000	25.47219649

		x [Å]	y [Å]	z [Å]
●	La	2.03505966	0.00000000	13.98562723
●	Br	0.00000000	2.03505966	15.62743225
●	La	0.00000000	2.03505966	11.48656926
●	Br	2.03505966	0.00000000	9.84476423
•	O	0.00000000	0.00000000	12.73609824
•	O	2.03505966	2.03505966	12.73609824

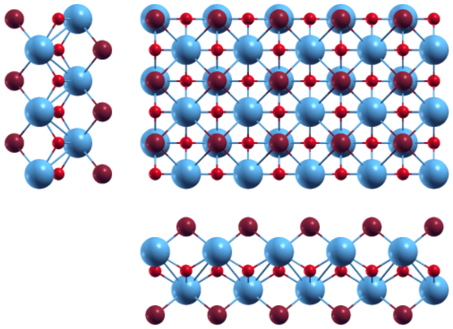

Orthographic projections: views of LaOBr (P4/nmm) as seen from the x axis (left), the y axis (bottom) and the z axis (center).

Lattice matching - minimal size

Formula	N° atoms	strain	cell size 1	cell size 2
K	7	0.1614	1	1
Tl	7	0.1112	1	1
InSe	8	0.1299	1	1
Bi_2	8	0.1341	1	1
Ag_2	8	0.1674	1	1
PbTe	8	0.1311	1	1
CaCl	8	0.2102	1	1
Cl_2Mn	9	0.1099	1	1
CdI_2	9	0.1324	1	1
Nd	9	0.1743	1	3
$MoSe_2$	9	0.1114	1	1
Ba_2Pt	9	0.1671	1	1
S_2Ta	9	0.1094	1	1
Br_2Ca	9	0.1333	1	1
CaI_2	9	0.152	1	1
Br_2Cu	9	0.0989	1	1
NSr_2	9	0.1085	1	1
Ca_2Si	9	0.1723	1	1
I_2Yb	9	0.1496	1	1
BiClTe	9	0.1327	1	1
FeI_2	9	0.1088	1	1
NbS_2	9	0.1095	1	1
CrI_2	9	0.1087	1	1
BiBrTe	9	0.137	1	1
NbS_2	9	0.1105	1	1
S_2Ta	9	0.1107	1	1
Se_2V	9	0.111	1	1
I_2Tm	9	0.1509	1	1
GeI_2	9	0.1313	1	1
I_2Pb	9	0.1692	1	1
BiITe	9	0.1425	1	1
$MnSe_2$	9	0.2101	1	1
DyI_2	9	0.1548	1	1
Br_2Mg	9	0.1088	1	1
GdI_2	9	0.1393	1	1
CNNa	9	0.0692	1	1
CdI_2	9	0.1321	1	1
Se_2Sn	9	0.1085	1	1
I_2Pr	9	0.1327	1	1
Se_2W	9	0.1113	1	1
Bi_2Te_2	10	0.1879	1	1
Bi_2In_2	10	0.4079	1	1
Cu_2I_2	10	0.0025	1	1
Cu_2Sr_2	10	0.1378	1	1
Cl_2OOs	10	0.2162	1	1
$LiMnTe_2$	10	0.1315	1	1
$AgCuTe_2$	10	0.1981	1	1
$AsLi_3$	10	0.1301	1	1
O_2Sn_2	10	0.0316	1	1
S_2Sn_2	10	0.0193	1	1

Table showing the first 50 lattice matching combinations with the smallest number of atoms in the supercell.

Lattice matching - minimal strain

Formula	N° atoms	strain	cell size 1	cell size 2
$Eu_2F_2I_2$	12	0.0	1	1
CCl_2Gd_2	613	0.0003	48	65
Tl	375	0.0004	49	81
CBr_2Y_2	613	0.0004	48	65
$H_2Li_2O_2$	690	0.0004	50	65
CoO_2	714	0.0004	54	130
Br_2Zn	483	0.0004	48	65
$Cu_2K_2Te_2$	882	0.0004	82	65
Mg_2	86	0.0005	9	16
Bi_2O_2	10	0.0005	1	1
$C_2Br_2Tb_2$	948	0.0005	72	86
Cl_4KTl	738	0.0006	89	34
$AgTe_2$	708	0.0006	65	106
Bi_2In_2	590	0.0006	65	50
Mg_4	618	0.0007	49	81
Te_2Ti	483	0.0007	48	65
$H_2Li_2O_2$	678	0.0007	49	64
$HfSe_2$	483	0.0007	48	65
$Al_2Cl_2O_2$	918	0.0008	63	90
$Br_2O_2Ti_2$	66	0.0008	5	6
O_2Pb_2	10	0.0008	1	1
Cl_2Y_2	548	0.0009	48	65
$C_2Br_2Gd_2$	948	0.0009	72	86
CaCl	968	0.0009	113	145
NiO_2	714	0.0009	54	130
$FeSe_2$	297	0.0009	25	49
$Cu_2Rb_2Te_2$	690	0.001	65	50
$F_2I_2Sm_2$	12	0.001	1	1
Cl_4Mg_2	762	0.001	93	34
NiO_2	633	0.001	48	115
HfS_2	840	0.001	81	118
Cl_2Ni	852	0.001	79	126
Se_2Ta_4	366	0.0011	25	36
Cl_2ORu	708	0.0011	64	81
$NbSe_2$	852	0.0011	79	126
Se_2Ta	852	0.0012	79	126
Fe_2Se_2	896	0.0012	82	101
Mg_3	945	0.0012	85	145
$Ge_2Se_2Zr_2$	882	0.0012	65	82
Ca_2Cl_2	718	0.0012	65	82
Cl_2OOs	886	0.0012	81	100
$Cu_2K_2Te_2$	870	0.0012	81	64
CoO_2	633	0.0012	48	115
$CoTe_2$	840	0.0013	81	118
Sm	387	0.0014	44	123
$ReSe_2$	852	0.0014	79	126
C_4Ca_2	642	0.0014	54	53
Ga_2S_2	958	0.0014	81	118
$LiOS_2Ti$	929	0.0014	64	109
CNRb	735	0.0014	90	65

Table showing the first 50 lattice matching combinations with the smallest strain needed to build the supercell.

LaOI (P4/nmm)

Structural and electronic properties

**Formula**	LaOI
**Spacegroup**	P4/nmm
**Prototype**	PbClF
**Parent 3D**	$La_2O_2I_2$
**Source DB**	COD
**DB ID**	9009171
**DF2-C09 Binding energy [meV/ Å^2]**	14.81
**RVV10 Binding energy [meV/ Å^2]**	21.34
**Band gap (PBE) [eV]**	3.41

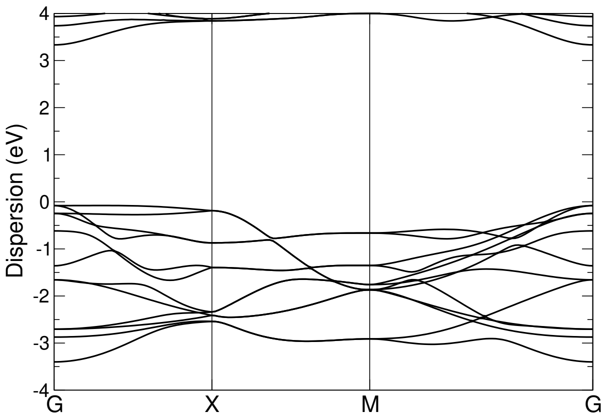

Band structure: Electronic band structure of LaOI (P4/nmm) in a representative window around the Fermi energy and along a high-symmetry path.

Optimized crystal structure

Structural parameters: relaxed cell (top) and atomic positions (bottom) of LaOI (P4/nmm) in Cartesian coordinates.

		x [Å]	y [Å]	z [Å]
$\mathbf{a_1}$		4.16089323	0.00000000	0.00000000
$\mathbf{a_2}$		0.00000000	4.16089323	0.00000000
$\mathbf{a_3}$		0.00000000	0.00000000	26.17512528
		x [Å]	y [Å]	z [Å]
●	La	0.00000000	2.08044661	14.28657600
●	I	2.08044661	0.00000000	16.21987343
●	La	2.08044661	0.00000000	11.88854928
●	I	0.00000000	2.08044661	9.95525185
●	O	0.00000000	0.00000000	13.08756264
●	O	2.08044661	2.08044661	13.08756264

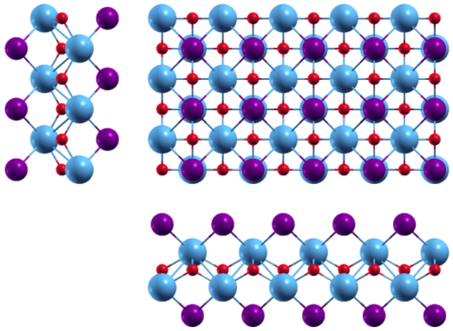

Orthographic projections: views of LaOI (P4/nmm) as seen from the x axis (left), the y axis (bottom) and the z axis (center).

Lattice matching - minimal size

Formula	N° atoms	strain	cell size 1	cell size 2
K	7	0.1508	1	1
AgTl	8	0.0169	1	1
Ag_2	8	0.1563	1	1
Nd	9	0.1626	1	3
Ba_2Pt	9	0.156	1	1
$ReSe_2$	9	0.1093	1	1
CaI_2	9	0.1424	1	1
Br_2Cu	9	0.349	1	1
Ca_2Si	9	0.1607	1	1
I_2Yb	9	0.1402	1	1
Cl_2Zn	9	0.2146	1	1
S_2Ti	9	0.1106	1	1
Ba_2Hg	9	0.0025	1	1
Cl_2Ni	9	0.1091	1	1
Cl_2Co	9	0.1107	1	1
Br_2V	9	0.1095	1	1
ClNZr	9	0.1099	1	1
Cl_2Fe	9	0.1109	1	1
$AsSe_2$	9	0.1089	1	1
I_2Tm	9	0.1414	1	1
I_2Pb	9	0.1579	1	1
BiITe	9	0.1339	1	1
$NbSe_2$	9	0.1091	1	1
DyI_2	9	0.1449	1	1
Cl_2Zr	9	0.1108	1	1
Se_2Ta	9	0.109	1	1
$NbSe_2$	9	0.1088	1	1
GdI_2	9	0.1312	1	1
CNNa	9	0.3506	1	1
F_2Ni	9	0.2102	1	1
Ba_2Cd	9	0.0052	1	1
Bi_2Te_2	10	0.175	1	1
Li_2Tl_2	10	0.7764	1	1
Bi_2In_2	10	0.3848	1	1
InSe	10	0.972	1	2
Cu_2I_2	10	0.0077	1	1
Cu_2Sr_2	10	0.1299	1	1
Cl_2OOs	10	0.2122	1	1
Bi_2Mn_2	10	0.1643	1	1
$AgCuTe_2$	10	0.1878	1	1
O_2Sn_2	10	0.2217	1	1
Au_2Br_2	10	0.0135	1	1
$AlLiTe_2$	10	0.1331	1	1
Ge_2Te_2	10	0.0236	1	1
Fe_2Se_2	10	0.2096	1	1
Cl_2ORu	10	0.6043	1	1
As_2Co_2	10	0.5939	1	1
N_3Na	10	0.371	1	1
Cu_2Te_2	10	0.2176	1	1
Ge_2S_2	10	0.2389	1	1

Table showing the first 50 lattice matching combinations with the smallest number of atoms in the supercell.

Lattice matching - minimal strain

Formula	N° atoms	strain	cell size 1	cell size 2
$H_2Li_2O_2$	510	0.0	36	49
$FeSe_2$	594	0.0001	49	100
Co_2Se_2	560	0.0001	50	65
Hf_3Te_2	800	0.0001	65	82
AsSb	418	0.0002	48	65
Br_2CsF	934	0.0003	101	82
As_2Co_2	550	0.0003	49	64
$AgTe_2$	945	0.0003	85	145
H_2Li_2Pt	697	0.0004	47	83
$Ca_2Cl_2H_2$	882	0.0004	65	82
Co_2S_2	962	0.0005	85	113
Mg_3	102	0.0005	9	16
$Hf_2Si_2Te_2$	690	0.0005	50	65
PTe_2Zr_2	613	0.0006	48	65
Cl_2OV	832	0.0006	70	103
CoI_2	483	0.0006	48	65
Hf_3Te_2	789	0.0007	64	81
$CrSe_2$	237	0.0007	20	39
$AsSn_2$	840	0.0007	81	118
$Hf_2Se_2Si_2$	876	0.0008	61	85
FHOZn	276	0.0008	20	39
Br_2CsF	924	0.0009	100	81
H_2NiO_2	315	0.0009	20	39
Cl_4Mn	806	0.0009	81	64
Cl_2Zn	636	0.0009	65	82
I_2Ti	483	0.001	48	65
$AgClO_4$	510	0.001	49	36
Ca_2Cl_2	550	0.001	49	64
$Ge_2Se_2Zr_2$	678	0.001	49	64
CdClHO	958	0.001	81	118
Co_2Se_2	550	0.001	49	64
Fe_2SeTe	896	0.001	82	101
Ca_2O_2	196	0.0011	16	25
Pb_2Se_2	86	0.0011	9	8
CaCl	736	0.0011	85	113
$PtSe_2$	840	0.0012	81	118
Bi_2In_2	924	0.0012	100	81
Se_2Ti	852	0.0012	79	126
$Ca_2Cl_2H_2$	870	0.0012	64	81
$MnSe_2$	849	0.0012	85	113
As_2Fe_2	706	0.0013	61	85
K_2PdS_2	330	0.0013	40	18
HgO	456	0.0014	49	81
C_4Ca_2	642	0.0014	53	54
Br_2Zr_2	978	0.0014	79	126
CI_2Y_2	613	0.0014	48	65
CNNa	918	0.0014	101	104
In	199	0.0015	25	49
Na	600	0.0015	79	126
$Ag_2K_2Te_2$	678	0.0015	64	49

Table showing the first 50 lattice matching combinations with the smallest strain needed to build the supercell.

$LiAlTe_2$ (P3m1)

Structural and electronic properties

**Formula**	$LiAlTe_2$
**Spacegroup**	P3m1
**Prototype**	LiMnSe2
**Parent 3D**	$LiAlTe_2$
**Source DB**	COD
**DB ID**	4321120
**DF2-C09 Binding energy [meV/ Å^2]**	19.57
**RVV10 Binding energy [meV/ Å^2]**	22.1
**Band gap (PBE) [eV]**	0.92

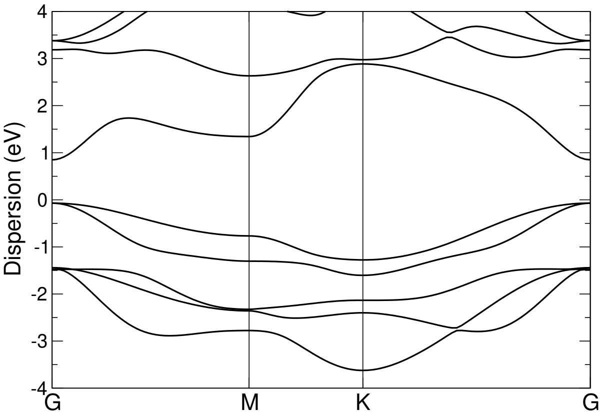

Band structure: Electronic band structure of $LiAlTe_2$ (P3m1) in a representative window around the Fermi energy and along a high-symmetry path.

Optimized crystal structure

Structural parameters: relaxed cell (top) and atomic positions (bottom) of $LiAlTe_2$ (P3m1) in Cartesian coordinates.

	x [Å]	y [Å]	z [Å]
$\mathbf{a_1}$	4.38014646	0.00000000	0.00000000
$\mathbf{a_2}$	−2.19007323	3.79331810	0.00000000
$\mathbf{a_3}$	0.00000000	0.00000000	24.42601368

		x [Å]	y [Å]	z [Å]
●	Li	0.00000000	0.00000000	14.20292508
●	Te	2.19007323	1.26443937	13.81425280
●	Al	2.19007323	1.26443937	11.26864802
●	Te	0.00000000	2.52887874	10.11053313

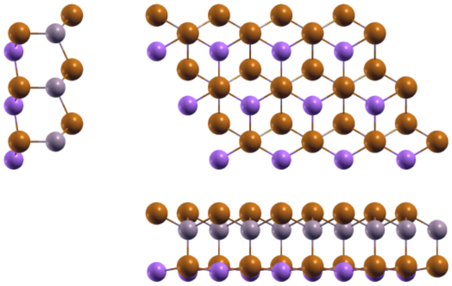

Orthographic projections: views of $LiAlTe_2$ (P3m1) as seen from the x axis (left), the y axis (bottom) and the z axis (center).

Lattice matching - minimal size

Formula	N° atoms	strain	cell size 1	cell size 2
Bi_2	6	0.0088	1	1
GeTe	6	0.2593	1	1
S_2	6	0.2615	1	1
CaCl	6	0.4268	1	1
$IrTe_2$	7	0.2605	1	1
$CdCl_2$	7	0.2578	1	1
Br_2Zn	7	1.5532	1	1
$InSe_2$	7	0.2583	1	1
$GeTe_2$	7	0.2564	1	1
$SiTe_2$	7	1.5832	1	1
$HfTe_2$	7	0.2725	1	1
I_2Pr	7	0.1258	1	1
I_2Mn	7	0.258	1	1
NSr_2	7	1.6408	1	1
I_2Yb	7	0.0091	1	1
PbS_2	7	0.2476	1	1
Cl_2Zn	7	0.1133	1	1
FeI_2	7	0.255	1	1
I_2Ni	7	0.2567	1	1
Te_2Ti	7	1.5554	1	1
CrI_2	7	0.2543	1	1
BiBrTe	7	0.0053	1	1
$RhTe_2$	7	1.5242	1	1
Ba_2Hg	7	0.1521	1	1
Cl_2Fe	7	4.8686	1	1
Ba_2N	7	0.2748	1	1
Te_2Zr	7	0.2732	1	1
I_2Nd	7	0.1264	1	1
I_2V	7	1.5931	1	1
Se_2Zr	7	1.5863	1	1
BiITe	7	0.0011	1	1
CoI_2	7	1.6318	1	1
$MnSe_2$	7	0.4266	1	1
Br_2Ni	7	1.4495	1	1
CeI_2	7	0.1253	1	1
Cl_2Zr	7	4.8769	1	1
Br_2Mg	7	0.2548	1	1
GdI_2	7	0.0025	1	1
F_2Ni	7	0.1118	1	1
I_2La	7	0.1296	1	1
Se_2Sn	7	1.639	1	1
F_2Zn	7	0.1233	1	1
Gd	7	0.1707	1	3
Ba_2Cd	7	0.1547	1	1
NaPSn	7	0.2712	1	1
$HfSe_2$	7	1.5555	1	1
H_2Si_2	8	0.2588	1	1
Fe_2Te_2	8	0.1184	1	1
Li_2Tl_2	8	2.9243	1	1
Ca_2Cl_2	8	0.1186	1	1

Table showing the first 50 lattice matching combinations with the smallest number of atoms in the supercell.

Lattice matching - minimal strain

Formula	N° atoms	strain	cell size 1	cell size 2
GeTe	418	0.0	64	81
Cl_2Ti	84	0.0	9	16
Br_2Ti	139	0.0	16	25
Cl_2Zr	439	0.0001	49	81
I_2V	343	0.0001	43	57
$F_2Se_2Tm_2$	924	0.0001	81	100
CS_2Ta_2	116	0.0001	9	16
P_2	68	0.0001	9	16
S_2Ti	331	0.0001	37	61
$Br_2H_2Zr_2$	214	0.0001	16	25
KNO_3	56	0.0002	9	4
CBr_2Y_2	389	0.0002	36	49
In_2S_3	661	0.0002	64	81
Cl_2Gd_2	656	0.0002	73	91
Cl_2Co	331	0.0002	37	61
Tl	368	0.0002	65	108
$LiMnSe_2$	724	0.0002	81	100
Br_2Cr	139	0.0002	16	25
Mg_4	692	0.0003	65	108
CdO_2	439	0.0003	49	81
Cl_2Fe	439	0.0003	49	81
$Cl_2H_2Lu_2$	316	0.0003	25	36
CdO_2	331	0.0004	37	61
Cl_2Y_2	340	0.0004	36	49
FeI_2	447	0.0004	57	73
H_2Si_2	580	0.0004	64	81
CrS_2	496	0.0004	49	100
$Cl_2Ho_2O_2$	514	0.0005	43	57
Cl_2Sc_2	164	0.0005	16	25
Te_4W_2	340	0.0005	40	30
Br_2Mg	447	0.0005	57	73
$CrTe_2$	139	0.0005	16	25
Cl_2Zr	331	0.0006	37	61
$Cl_2H_2Sc_2$	214	0.0006	16	25
Cl_2Mg	208	0.0006	25	36
CNb_2S_2	116	0.0006	9	16
PTe_2Zr_2	516	0.0007	49	64
Br_2Ni	208	0.0007	25	36
$InSe_2$	499	0.0007	64	81
S_2Zn_2	656	0.0007	73	91
$GeTe_2$	447	0.0007	57	73
CCl_2Gd_2	389	0.0008	36	49
CrI_2	447	0.0008	57	73
Li_2Tl_2	400	0.0008	57	43
$Cl_2Hg_2N_2$	846	0.0008	114	65
PTe_2Ti_2	280	0.0008	25	36
Se_2Ta	139	0.0008	16	25
Se_2Zr	343	0.0008	43	57
I_2Y_2	724	0.0008	81	100
$Cl_2Er_2H_2$	742	0.0009	64	81

Table showing the first 50 lattice matching combinations with the smallest strain needed to build the supercell.

$LiAuI_4$ (P-1)

Structural and electronic properties

**Formula**	$LiAuI_4$
**Spacegroup**	P-1
**Prototype**	LiAuI4
**Parent 3D**	$Li_2Au_2I_8$
**Source DB**	COD
**DB ID**	1510187
**DF2-C09 Binding energy [meV/ Å^2]**	17.06
**RVV10 Binding energy [meV/ Å^2]**	23.9
**Band gap (PBE) [eV]**	0.97

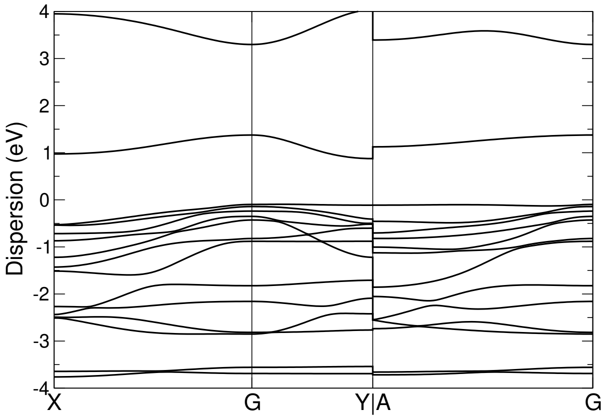

Band structure: Electronic band structure of $LiAuI_4$ (P-1) in a representative window around the Fermi energy and along a high-symmetry path.

Optimized crystal structure

Structural parameters: relaxed cell (top) and atomic positions (bottom) of $LiAuI_4$ (P-1) in Cartesian coordinates.

		x [Å]	y [Å]	z [Å]
$\mathbf{a_1}$		4.40480518	0.23969168	0.00000000
$\mathbf{a_2}$		0.40852322	7.20354322	0.00000000
$\mathbf{a_3}$		0.00000000	0.00000000	23.65881569
		x [Å]	y [Å]	z [Å]
●	Li	0.01781494	0.30999455	11.82933950
●	Au	2.42436616	4.03151387	11.82943267
●	I	3.82563340	2.68445214	13.65291087
●	I	1.02301929	5.37833378	10.00582743
●	I	2.50327441	6.19979988	13.38802203
●	I	2.34543976	1.86318678	10.27091458

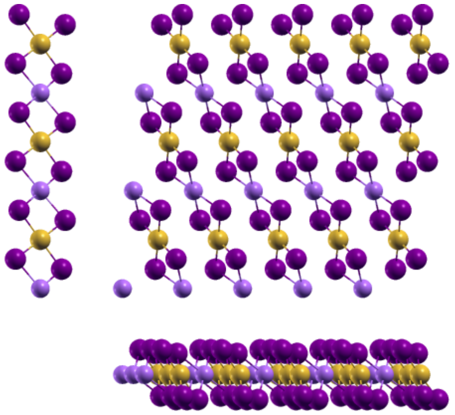

Orthographic projections: views of $LiAuI_4$ (P-1) as seen from the x axis (left), the y axis (bottom) and the z axis (center).

Lattice matching - minimal size

Formula	N° atoms	strain	cell size 1	cell size 2
K	8	0.0499	1	2
Ag_2	8	0.2114	1	1
S_2	8	0.1948	1	1
Na	8	0.1261	1	2
Ba_2Pt	9	0.2111	1	1
$GeTe_2$	9	0.1922	1	1
Ca_2Si	9	0.2156	1	1
I_2Yb	9	0.3956	1	1
PbS_2	9	0.1877	1	1
LiO_2	9	0.1961	1	1
DyI_2	9	0.2005	1	1
I_2Ti	9	0.19	1	1
I_2La	9	0.2105	1	1
F_2Na	9	0.1842	1	1
Sm	9	0.1452	1	3
Cl_2Gd_2	10	0.1964	1	1
In_2Se_2	10	0.0968	1	1
Br_2Pr_2	10	0.1837	1	1
Ag_2Br_2	10	0.2109	1	1
Pb_2Se_2	10	0.1714	1	1
Ag_2	10	0.0553	1	2
Au_2I_2	10	0.1069	1	1
Br_2Y_2	10	0.1876	1	1
Sb_2Te_2	10	0.2143	1	1
Ba_2Cu_2	10	0.3953	1	1
As_2Rh_2	10	0.2107	1	1
CaCl	10	0.0584	1	2
I_3Sn	10	0.4429	1	1
I_2Y_2	10	0.1974	1	1
F_4Pb	11	0.105	1	1
AsI_2La_2	11	0.1921	1	1
CI_2Y_2	11	0.1902	1	1
FKO_2Se	11	0.1842	1	1
CBr_2Y_2	11	0.1826	1	1
CCl_2Gd_2	11	0.1829	1	1
$AgNO_3$	11	0.2735	1	1
I_2La_2Sb	11	0.2065	1	1
Hg_3S_2	11	1.6051	1	1
Cl_2Zn	12	0.1282	1	2
$Br_2Ho_2S_2$	12	0.9758	1	1
$MoTe_2$	12	0.1268	1	2
$Gd_2I_2S_2$	12	0.2026	1	1
Ba_2Pt	12	0.055	1	2
CaI_2	12	0.0419	1	2
$HfTe_2$	12	0.0399	1	2
Te_2V	12	0.1273	1	2
AsSb	12	0.2832	1	3
$Cu_2Na_2Te_2$	12	0.1065	1	1
I_2Yb	12	0.0399	1	2
$Cl_2H_2Zr_2$	12	0.6966	1	1

Table showing the first 50 lattice matching combinations with the smallest number of atoms in the supercell.

Lattice matching - minimal strain

Formula	N° atoms	strain	cell size 1	cell size 2
$Br_2Lu_2O_2$	912	0.0003	47	105
$Br_2Cr_2S_2$	816	0.0004	44	92
Br_2Ca_3Si	762	0.0004	46	81
$Ge_2Te_2Zr_2$	912	0.0004	47	105
$Br_2Eu_2O_2$	720	0.0005	39	81
$I_2N_2Zr_2$	672	0.0005	31	81
CeI_2	477	0.0005	39	81
F_4Nb	658	0.0005	43	80
I_2Pr	477	0.0006	39	81
I_2La_2Te	681	0.0006	46	81
Ni_2Se_2	558	0.0007	39	81
Fe_2SeTe	702	0.0007	47	105
$Br_2Cr_2S_2$	408	0.0008	22	46
Br_2Ca_3Si	780	0.0009	47	83
$I_2O_2Yb_2$	720	0.0009	39	81
$Gd_2I_2S_2$	798	0.0009	48	85
$Br_2H_2Yb_2$	912	0.001	47	105
Ga_2Se_2	510	0.001	31	81
$As_2Fe_2Li_2$	912	0.001	47	105
Br_2CsF	788	0.0011	66	98
Bi_2Mn_2	692	0.0011	42	110
$Gd_2I_2S_2$	780	0.0011	47	83
I_2La_2Te	445	0.0011	30	53
Br_2Ca_3Si	498	0.0012	30	53
$SbSe_2Tl$	362	0.0012	31	44
Br_2Ca_3Si	498	0.0012	30	53
Ir_2P_2	558	0.0012	39	81
Se_4TiZr	648	0.0012	46	62
Br_2Ca_3Si	480	0.0013	29	51
$RhTe_2$	429	0.0013	31	81
Cl_2Rb_2	688	0.0013	76	58
H_2NiO_2	882	0.0013	37	132
$Gd_2I_2S_2$	498	0.0013	30	53
CKN	564	0.0014	54	80
$ClKO_3$	506	0.0014	51	40
$Eu_2I_2O_2$	732	0.0014	41	81
$Br_2O_2Sm_2$	720	0.0014	39	81
I_2La_2Te	429	0.0014	29	51
As_2Ir_2	504	0.0014	36	72
P_2Rh_2	612	0.0014	42	90
I_2La_2Te	252	0.0014	17	30
In	258	0.0014	27	96
F_2Tl_2	612	0.0014	42	90
I_2Nd	477	0.0014	39	81
Cu_2F_4	612	0.0014	48	54
Br_2Ca_3Si	282	0.0014	17	30
$I_2O_2Sm_2$	732	0.0014	41	81
$AgClO_2$	664	0.0015	44	100
$Br_2O_2Y_2$	792	0.0015	42	90
$Br_2Dy_2O_2$	792	0.0015	42	90

Table showing the first 50 lattice matching combinations with the smallest strain needed to build the supercell.

$LiBH_4$ (C2)

Structural and electronic properties

**Formula**	$LiBH_4$
**Spacegroup**	C2
**Prototype**	LiBH4
**Parent 3D**	$Li_2B_2H_8$
**Source DB**	ICSD
**DB ID**	245569
**DF2-C09 Binding energy [meV/ Å^2]**	20.99
**RVV10 Binding energy [meV/ Å^2]**	24.29
**Band gap (PBE) [eV]**	6.39

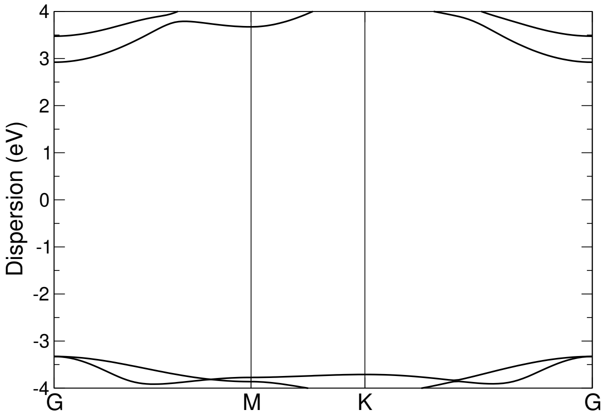

Band structure: Electronic band structure of $LiBH_4$ (C2) in a representative window around the Fermi energy and along a high-symmetry path.

Optimized crystal structure

Structural parameters: relaxed cell (top) and atomic positions (bottom) of $LiBH_4$ (C2) in Cartesian coordinates.

	x [Å]	y [Å]	z [Å]
$\mathbf{a_1}$	4.11981953	0.00000000	0.00000000
$\mathbf{a_2}$	−2.05990976	3.56786837	0.00000000
$\mathbf{a_3}$	0.00000000	0.00000000	21.62248245

		x [Å]	y [Å]	z [Å]
	Li	2.05990976	1.18928946	11.16423803
	B	0.00000000	2.37857891	11.12060660
	H	0.00000000	2.37857891	9.89800200
	H	-0.98732513	2.94861134	11.56034891
	H	0.00000000	1.23851405	11.56034891
	H	0.98732513	2.94861134	11.56034891

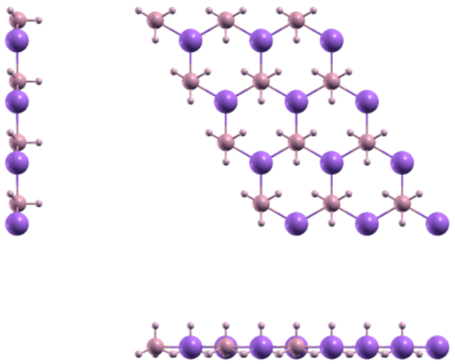

Orthographic projections: views of $LiBH_4$ (C2) as seen from the x axis (left), the y axis (bottom) and the z axis (center).

Lattice matching - minimal size

Formula	N° atoms	strain	cell size 1	cell size 2
K	7	0.4607	1	1
Na	7	1.5634	1	1
Ag_2	8	0.4746	1	1
As_2	8	0.2499	1	1
Sb_2	8	0.0018	1	1
CaCl	8	0.1201	1	1
Cl_2Zn	9	0.2485	1	1
I_2Mg	9	0.0039	1	1
PSn_2	9	0.2621	1	1
Ba_2Pt	9	0.4739	1	1
HfS_2	9	0.2536	1	1
$AsSn_2$	9	0.2674	1	1
Te_2V	9	1.594	1	1
I_2Pr	9	0.1488	1	1
S_2Zr	9	0.261	1	1
Br_2La	9	0.0042	1	1
Ca_2Si	9	0.4856	1	1
Br_2Co	9	0.2493	1	1
Ca_2N	9	0.2506	1	1
BrCdI	9	0.0071	1	1
Cl_2Zn	9	0.129	1	1
I_2Zn	9	0.005	1	1
$RhTe_2$	9	0.2715	1	1
GeI_2	9	0.002	1	1
Br_2Mn	9	0.247	1	1
$CrTe_2$	9	1.5247	1	1
$CoTe_2$	9	0.254	1	1
CdClO	9	1.5904	1	1
Se_2Ti	9	1.5517	1	1
Br_2Ti	9	1.5206	1	1
AsKSn	9	0.009	1	1
Te_2W	9	1.581	1	1
$PbTe_2$	9	0.0061	1	1
I_2Nd	9	0.1497	1	1
S_2Sn	9	0.2614	1	1
$SnTe_2$	9	0.0003	1	1
Sn	9	0.6402	1	3
I_2Pb	9	0.4786	1	1
$PtSe_2$	9	0.2684	1	1
OTl_2	9	1.5918	1	1
Br_2Fe	9	0.2494	1	1
GeS_2	9	0.1142	1	1
$TaTe_2$	9	0.2666	1	1
$MnSe_2$	9	0.12	1	1
Br_2Ni	9	0.2563	1	1
CeI_2	9	0.1481	1	1
CuO_2	9	0.1765	1	1
$NbTe_2$	9	0.2607	1	1
Se_2Yb	9	0.0022	1	1
Cl_2Mg	9	0.2563	1	1

Table showing the first 50 lattice matching combinations with the smallest number of atoms in the supercell.

Lattice matching - minimal strain

Formula	N° atoms	strain	cell size 1	cell size 2
$Cl_2Hf_2N_2$	600	0.0001	43	57
Br_2Co	486	0.0001	49	64
OTl_2	429	0.0001	43	57
$AsSn_2$	786	0.0001	81	100
Ga_2S_2	634	0.0001	57	73
Br_2Fe	486	0.0001	49	64
$I_2Pr_2S_2$	870	0.0002	81	64
K	681	0.0002	100	81
FHOZn	466	0.0002	37	61
Te_2V	429	0.0002	43	57
$Ag_2K_2Se_2$	678	0.0002	65	48
CBr_2Lu_2	789	0.0002	64	81
ClNZr	258	0.0003	25	36
CdClO	429	0.0003	43	57
$SnTe_2$	9	0.0003	1	1
Br_2Zr_2	412	0.0003	36	49
I_4Sr_2	942	0.0003	114	43
$Br_2N_2Zr_2$	870	0.0003	64	81
$CrSe_2$	537	0.0003	49	81
CrO_2	51	0.0003	4	9
$CrSe_2$	405	0.0003	37	61
CCl_2Lu_2	614	0.0004	49	64
Sb_2Te_2	666	0.0004	73	57
NaO_4	230	0.0004	25	16
PTe_2Ti_2	707	0.0004	57	73
As_2	422	0.0005	49	64
$I_2N_2Ti_2$	738	0.0005	60	63
HN_3OZn	246	0.0005	16	25
$PtSe_2$	786	0.0005	81	100
Cl_2Zn	486	0.0005	49	64
Ag_2	614	0.0006	81	64
Br_2Ni	561	0.0006	57	73
Cl_2Mg	561	0.0006	57	73
Se_2Ti	363	0.0007	36	49
$CeLi_2P_2$	11	0.0007	1	1
$TaTe_2$	786	0.0007	81	100
Bi_2Te_2	438	0.0007	49	36
Ba_2Pt	678	0.0008	81	64
$Br_2H_2Zr_2$	366	0.0008	25	36
$I_2Nd_2S_2$	984	0.0008	91	73
Cl_2V	405	0.0009	37	61
Cu_2Te_2	914	0.0009	87	98
CoH_2O_2	134	0.0009	9	16
Ge_2Se_2	634	0.0009	67	58
Ca_2Si	609	0.0009	73	57
$Ce_2I_2S_2$	780	0.0009	73	57
Sn_2Te_2	874	0.0009	99	70
Ga_2S_2	634	0.0009	57	73
$Cl_2H_2Lu_2$	780	0.0009	57	73
Ho_2S_2	396	0.001	40	39

Table showing the first 50 lattice matching combinations with the smallest strain needed to build the supercell.

LiOH (P4/nmm)

Structural and electronic properties

**Formula**	LiOH
**Spacegroup**	P4/nmm
**Prototype**	LiOH
**Parent 3D**	$Li_2(OH)_2$
**Source DB**	COD
**DB ID**	9008958
**DF2-C09 Binding energy [meV/ Å^2]**	12.69
**RVV10 Binding energy [meV/ Å^2]**	19.74
**Band gap (PBE) [eV]**	3.94

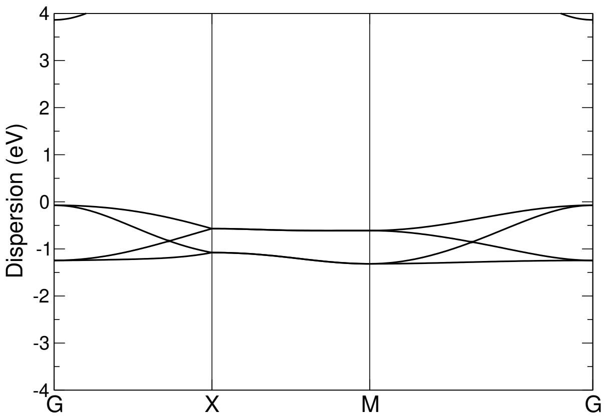

Band structure: Electronic band structure of LiOH (P4/nmm) in a representative window around the Fermi energy and along a high-symmetry path.

Optimized crystal structure

Structural parameters: relaxed cell (top) and atomic positions (bottom) of LiOH (P4/nmm) in Cartesian coordinates.

	x [Å]	y [Å]	z [Å]
$\mathbf{a_1}$	3.56669494	0.00000000	0.00000000
$\mathbf{a_2}$	0.00000000	3.56669494	0.00000000
$\mathbf{a_3}$	0.00000000	0.00000000	23.64880301

	x [Å]	y [Å]	z [Å]
H	0.00000000	1.78334747	13.63171789
O	0.00000000	1.78334747	12.66525294
Li	0.00000000	0.00000000	11.82440150
Li	1.78334747	1.78334747	11.82440150
H	1.78334747	0.00000000	10.01708512
O	1.78334747	0.00000000	10.98355007

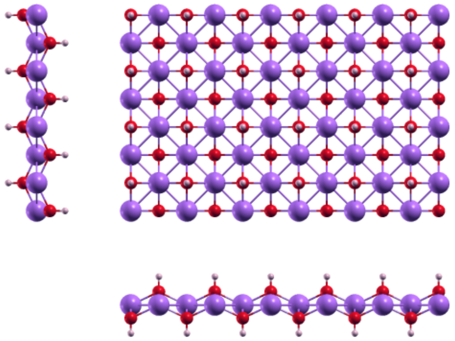

Orthographic projections: views of LiOH (P4/nmm) as seen from the x axis (left), the y axis (bottom) and the z axis (center).

Lattice matching - minimal size

Formula	N° atoms	strain	cell size 1	cell size 2
Tl	7	0.2116	1	1
InSe	8	0.1937	1	1
AsSb	8	0.142	1	1
GeTe	8	0.1475	1	1
S_2	8	0.149	1	1
PbTe	8	0.196	1	1
Mg_2	8	0.5655	1	1
Sb_2	8	0.1737	1	1
CaCl	8	0.0044	1	1
$IrTe_2$	9	0.1484	1	1
I_2Mg	9	0.1805	1	1
$CdCl_2$	9	0.1465	1	1
CdI_2	9	0.754	1	1
$AgTe_2$	9	0.2149	1	1
S_2Ta	9	0.1084	1	1
Br_2Zn	9	0.1337	1	1
$InSe_2$	9	0.1469	1	1
$GeTe_2$	9	0.1456	1	1
$SiTe_2$	9	0.1369	1	1
$HfTe_2$	9	0.1565	1	1
I_2Mn	9	0.1466	1	1
Br_2La	9	0.1809	1	1
NSr_2	9	0.1435	1	1
PbS_2	9	0.1398	1	1
$AuTe_2$	9	0.1648	1	1
BrCdI	9	0.1844	1	1
$PdTe_2$	9	0.1621	1	1
FeI_2	9	0.1446	1	1
I_2Ni	9	0.1458	1	1
Mg_3	9	0.5964	1	1
Te_2Ti	9	0.1339	1	1
CrI_2	9	0.1442	1	1
I_2Zn	9	0.1699	1	1
BaF_2	9	0.1887	1	1
BiBrTe	9	0.7766	1	1
$RhTe_2$	9	0.1307	1	1
GeI_2	9	0.1783	1	1
Ba_2Hg	9	1.1763	1	1
Ba_2N	9	0.1581	1	1
AsKSn	9	0.1868	1	1
Te_2Zr	9	0.157	1	1
$PbTe_2$	9	0.1832	1	1
$NiTe_2$	9	0.1365	1	1
$SnTe_2$	9	0.1755	1	1
I_2V	9	0.138	1	1
GeI_2	9	0.1963	1	1
Se_2Zr	9	0.1373	1	1
STl_2	9	0.1899	1	1
CoI_2	9	0.1424	1	1
GeS_2	9	0.0076	1	1

Table showing the first 50 lattice matching combinations with the smallest number of atoms in the supercell.

Lattice matching - minimal strain

Formula	N° atoms	strain	cell size 1	cell size 2
$F_2I_2Yb_2$	870	0.0	81	64
$I_2O_2Sm_2$	882	0.0	82	65
$I_2La_2O_2$	510	0.0	49	36
Ag_2Br_2	934	0.0001	101	82
I_3Sn	670	0.0001	89	34
$Cu_2Na_2Te_2$	612	0.0001	61	41
Bi_2O_2	580	0.0002	64	49
Mg_2	314	0.0002	36	49
Br_2CsF	814	0.0002	97	58
HN_3OZn	678	0.0002	48	65
$Ba_2Ge_2Mn_2$	870	0.0003	81	64
As_2Rh_2	934	0.0003	101	82
$F_2I_2Pb_2$	876	0.0003	85	61
$Eu_2H_2I_2$	882	0.0004	82	65
O_2Pb_2	590	0.0004	65	50
$Eu_2F_2I_2$	690	0.0004	65	50
$Eu_2H_2I_2$	870	0.0004	81	64
$Br_2La_2O_2$	690	0.0004	65	50
Ba_2Cd	693	0.0004	85	61
Mg_3	486	0.0005	49	64
$Ag_2K_2Se_2$	246	0.0005	25	16
PbS_2Sn	372	0.0006	42	30
$F_2I_2Sm_2$	690	0.0006	65	50
I_2La	852	0.0006	101	82
Ag_2Br_2	924	0.0006	100	81
Ba_2Pt	951	0.0006	118	81
CrO_2	237	0.0007	20	39
$Br_2La_2O_2$	678	0.0007	64	49
Ag_2	870	0.0007	118	81
$Eu_2F_2I_2$	678	0.0007	64	49
$I_2O_2Sm_2$	870	0.0007	81	64
C_2	584	0.0008	54	130
As_2Rh_2	924	0.0008	100	81
$Eu_2I_2O_2$	882	0.0009	82	65
$Cu_2Se_2Tl_2$	678	0.0009	64	49
Tl	465	0.0011	64	81
$Ba_2H_2I_2$	780	0.0011	81	49
CNRb	843	0.0011	110	61
CS_2Ta_2	613	0.0011	48	65
I_2La	843	0.0011	100	81
AgTl	536	0.0011	71	55
Pb_2Se_2	422	0.0011	49	32
S_2V	840	0.0011	81	118
$AgTe_2$	636	0.0012	65	82
Cl_2Ti	483	0.0012	48	65
$H_2I_2Sr_2$	366	0.0013	36	25
P_2	418	0.0013	48	65
Mg_4	708	0.0014	64	81
$K_2Mn_2Sb_2$	882	0.0014	82	65
$Br_2Ce_2O_2$	882	0.0014	82	65

Table showing the first 50 lattice matching combinations with the smallest strain needed to build the supercell.

LuHCl (P-3m1)

Structural and electronic properties

**Formula**	LuHCl
**Spacegroup**	P-3m1
**Prototype**	SmSI
**Parent 3D**	$Lu_2H_2Cl_2$
**Source DB**	ICSD
**DB ID**	62226
**DF2-C09 Binding energy [meV/ Å^2]**	12.65
**RVV10 Binding energy [meV/ Å^2]**	19.42
**Band gap (PBE) [eV]**	N/A

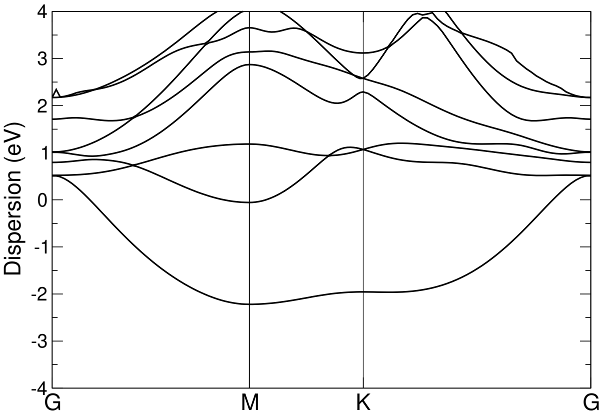

Band structure: Electronic band structure of LuHCl (P-3m1) in a representative window around the Fermi energy and along a high-symmetry path.

Optimized crystal structure

Structural parameters: relaxed cell (top) and atomic positions (bottom) of LuHCl (P-3m1) in Cartesian coordinates.

	x [Å]	y [Å]	z [Å]
$\mathbf{a_1}$	3.64768723	0.00000000	0.00000000
$\mathbf{a_2}$	−1.82384362	3.15898981	0.00000000
$\mathbf{a_3}$	0.00000000	0.00000000	26.03828895

	x [Å]	y [Å]	z [Å]
Lu	-0.00000000	2.10599321	14.33536270
H	-0.00000000	2.10599321	12.20614330
Cl	-0.00000000	0.00000000	16.02290737
Cl	-0.00000000	0.00000000	10.01538158
Lu	1.82384362	1.05299660	11.70292625
H	1.82384362	1.05299660	13.83214565

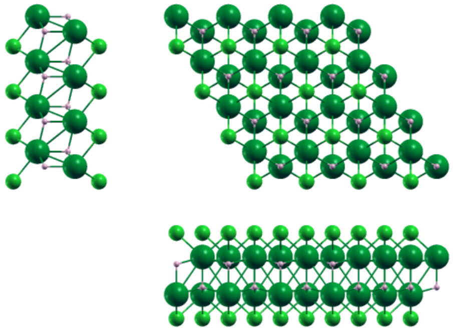

Orthographic projections: views of LuHCl (P-3m1) as seen from the x axis (left), the y axis (bottom) and the z axis (center).

Lattice matching - minimal size

Formula	N° atoms	strain	cell size 1	cell size 2
Tl	7	0.1182	1	1
Tl	7	0.2756	1	1
In	7	1.5837	1	1
InSe	8	2.9811	1	1
HgO	8	0.1251	1	1
As_2	8	0.005	1	1
LiO	8	0.2572	1	1
P_2	8	0.2679	1	1
PbTe	8	3.0047	1	1
Mg_2	8	0.1113	1	1
Sb_2	8	0.4761	1	1
Cl_2Zn	9	0.006	1	1
I_2Mg	9	2.8472	1	1
S_2V	9	0.2473	1	1
MoS_2	9	0.2483	1	1
CdI_2	9	3.0278	1	1
$AgTe_2$	9	0.1197	1	1
PSn_2	9	0.0039	1	1
$MoSe_2$	9	0.2745	1	1
Br_2Ca	9	3.0444	1	1
HfS_2	9	0.0023	1	1
$AsSn_2$	9	0.0077	1	1
Te_2V	9	0.0087	1	1
$CuTe_2$	9	0.0027	1	1
S_2Zr	9	0.0031	1	1
Br_2La	9	2.8515	1	1
Br_2Cu	9	1.0212	1	1
Br_2Co	9	0.0054	1	1
BiClTe	9	3.0327	1	1
ReS_2	9	1.5208	1	1
Ca_2N	9	0.0045	1	1
Cl_2Ti	9	0.2681	1	1
$AuTe_2$	9	0.4563	1	1
BrCdI	9	0.4992	1	1
$PdTe_2$	9	0.4503	1	1
HgI_2	9	0.3355	1	1
Mg_3	9	0.1154	1	1
I_2Zn	9	0.4677	1	1
S_2W	9	0.2484	1	1
Bi_2Pd	9	0.1316	1	1
GeI_2	9	0.486	1	1
Br_2Mn	9	0.0072	1	1
$CoTe_2$	9	0.002	1	1
CdClO	9	0.0091	1	1
AsKSn	9	2.9118	1	1
$PbTe_2$	9	0.4965	1	1
S_2Sn	9	0.0034	1	1
$SnTe_2$	9	0.4801	1	1
Cl_2V	9	0.2537	1	1
GeI_2	9	3.0078	1	1

Table showing the first 50 lattice matching combinations with the smallest number of atoms in the supercell.

Lattice matching - minimal strain

Formula	N° atoms	strain	cell size 1	cell size 2
C_2F_2	346	0.0	25	49
$CrSe_2$	561	0.0	57	73
Sb_2	614	0.0	81	64
Br_2HLa	666	0.0001	73	57
P_2	686	0.0002	81	100
Br_2La_2	742	0.0002	81	64
AsKSn	471	0.0002	57	43
Bi_2STe_2	474	0.0002	49	36
$CeLi_2P_2$	723	0.0003	73	57
Cl_2La_2	924	0.0003	100	81
$H_2I_2Yb_2$	678	0.0003	65	48
Cl_2Mg	9	0.0003	1	1
O_2Zn	258	0.0003	25	36
CNb_2S_2	986	0.0003	81	100
$AlLiTe_2$	316	0.0003	36	25
I_2Zn	765	0.0003	91	73
Cl_2Ti	786	0.0003	81	100
Br_2Ni	9	0.0004	1	1
$PbTe_2$	531	0.0004	64	49
CS_2Ta_2	986	0.0004	81	100
I_2La	534	0.0004	65	48
KNO_3	611	0.0005	81	25
PTe_2Ti_2	11	0.0005	1	1
MoS_2	486	0.0005	49	64
$AsCuLi_2$	666	0.0006	73	57
PbTe	366	0.0006	49	36
Li_2P_2Pr	806	0.0006	81	64
Br_2Ca_3Si	246	0.0006	25	16
As_2CeLi_2	474	0.0006	49	36
H_2NiO_2	614	0.0006	49	64
S_2W	486	0.0007	49	64
As_2Rh_2	582	0.0007	65	48
$Ga_2Ge_2Te_2$	870	0.0007	81	64
I_2La_2Te	230	0.0007	25	16
I_2La_2	514	0.0007	57	43
Cl_4Pd_2	120	0.0007	14	6
MoS_2	486	0.0007	49	64
$I_2Pr_2Si_2$	780	0.0007	73	57
As_2Sn_2	924	0.0008	100	81
In_2Se_2	838	0.0008	91	73
Br_2Gd_2Ge	629	0.0008	64	49
Cu_2I_2	580	0.0008	64	49
Bi_2Se_4	420	0.0008	52	18
Ga_2S_2	10	0.0008	1	1
N_3W_2	221	0.0008	16	25
GeI_2	402	0.0008	49	36
Ga_2Te_2	666	0.0008	73	57
Ba_2Ni_3	723	0.0009	73	57
Br_2Gd_2	924	0.0009	100	81
$AsLi_3$	438	0.0009	49	36

Table showing the first 50 lattice matching combinations with the smallest strain needed to build the supercell.

$Mg(OH)_2$ (P-3m1)

Structural and electronic properties

**Formula**	$Mg(OH)_2$
**Spacegroup**	P-3m1
**Prototype**	Mg(OH)2
**Parent 3D**	$Mg(OH)_2$
**Source DB**	COD
**DB ID**	9006332
**DF2-C09 Binding energy [meV/ Å^2]**	29.98
**RVV10 Binding energy [meV/ Å^2]**	36.55
**Band gap (PBE) [eV]**	3.33

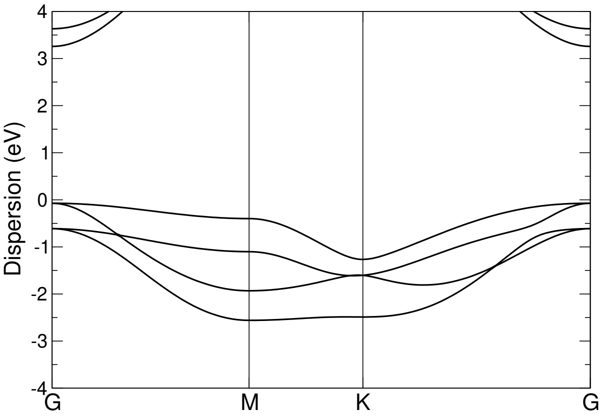

Band structure: Electronic band structure of $Mg(OH)_2$ (P-3m1) in a representative window around the Fermi energy and along a high-symmetry path.

Optimized crystal structure

Structural parameters: relaxed cell (top) and atomic positions (bottom) of $Mg(OH)_2$ (P-3m1) in Cartesian coordinates.

	x [Å]	y [Å]	z [Å]
$\mathbf{a_1}$	3.17587888	−0.00000000	0.00000000
$\mathbf{a_2}$	−1.58793944	2.75039179	0.00000000
$\mathbf{a_3}$	0.00000000	0.00000000	24.02879956

		x [Å]	y [Å]	z [Å]
•	H	0.00000000	1.83359453	10.01182624
•	O	0.00000000	1.83359453	10.97611685
•	Mg	-0.00000000	-0.00000000	12.01439978
•	H	1.58793944	0.91679726	14.01697332
•	O	1.58793944	0.91679726	13.05268271

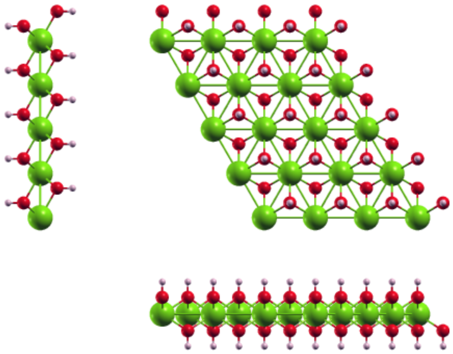

Orthographic projections: views of $Mg(OH)_2$ (P-3m1) as seen from the x axis (left), the y axis (bottom) and the z axis (center).

Lattice matching - minimal size

Formula	N° atoms	strain	cell size 1	cell size 2
Sn	6	0.1406	1	1
Na	6	0.4677	1	1
In	6	0.1445	1	1
In	6	0.0023	1	1
As_2	7	2.8345	1	1
LiO	7	0.0084	1	1
C_2	7	4.8583	1	1
Cl_2Zn	8	0.4855	1	1
S_2V	8	0.0009	1	1
MoS_2	8	0.0016	1	1
$MoTe_2$	8	0.4735	1	1
HfS_2	8	0.4954	1	1
FeO_2	8	0.2578	1	1
$AsSn_2$	8	2.9998	1	1
Te_2V	8	0.4784	1	1
$CuTe_2$	8	0.4945	1	1
NiO_2	8	0.2596	1	1
Br_2Co	8	0.4871	1	1
Ca_2N	8	2.8412	1	1
Te_2Zn	8	0.473	1	1
$RhTe_2$	8	3.0377	1	1
S_2W	8	0.0017	1	1
Br_2Mn	8	0.4824	1	1
$CrTe_2$	8	0.4543	1	1
PtS_2	8	0.4703	1	1
$CoTe_2$	8	0.4963	1	1
CdClO	8	0.4772	1	1
Se_2Ti	8	0.4637	1	1
Br_2Ti	8	0.4529	1	1
Te_2W	8	0.4739	1	1
Cl_2V	8	0.0058	1	1
$PtSe_2$	8	3.0084	1	1
OTl_2	8	0.4776	1	1
Br_2Fe	8	0.4873	1	1
Br_2Cr	8	0.4537	1	1
Br_2Ni	8	0.5007	1	1
CeI_2	8	0.3359	1	1
$FeSe_2$	8	0.1353	1	1
MoS_2	8	0.0018	1	1
Cl_2Mg	8	0.5009	1	1
$CrSe_2$	8	0.0045	1	1
Se_2Ta	8	0.451	1	1
$CrSe_2$	8	0.0071	1	1
O_2Pt	8	0.0038	1	1
N_2Re	8	0.2681	1	1
F_2Zn	8	0.3292	1	1
CoO_2	8	0.2588	1	1
Fe_2Te_2	9	0.3112	1	1
Ca_2Cl_2	9	0.312	1	1
CdClHO	9	2.9845	1	1

Table showing the first 50 lattice matching combinations with the smallest number of atoms in the supercell.

Lattice matching - minimal strain

Formula	N° atoms	strain	cell size 1	cell size 2
HfS_2	467	0.0	64	49
$HfTe_2$	173	0.0001	25	16
KS_2Ti	593	0.0001	73	57
$Gd_2I_2S_2$	794	0.0001	100	49
In_2Se_3	205	0.0001	25	16
$I_2S_2Tb_2$	629	0.0001	79	39
Bi_2Se_2Te	125	0.0002	16	9
$Er_2F_2Se_2$	221	0.0002	25	16
NiO_2	563	0.0002	64	81
$AsSn_2$	353	0.0002	49	36
ClH_3O	65	0.0003	9	4
I_2Zn	416	0.0003	61	37
GeI_2Y_2	125	0.0003	16	9
F_4Nb	295	0.0003	39	20
Br_2Mn	536	0.0003	73	57
Na	528	0.0003	91	73
Br_2Ca_3Si	794	0.0003	100	49
$Cl_2O_2Y_2$	330	0.0003	36	25
$CoTe_2$	467	0.0003	64	49
CoO_2	563	0.0003	64	81
$CuTe_2$	467	0.0004	64	49
$TaTe_2$	353	0.0004	49	36
N_2Re	705	0.0004	81	100
$Cl_2Ho_2O_2$	330	0.0004	36	25
CdH_2O_2	725	0.0004	81	64
$Br_2N_2Zr_2$	543	0.0004	57	43
CdClO	597	0.0004	81	64
Ga_2S_2	516	0.0005	64	49
Sn_2Te_2	668	0.0005	100	42
CBr_2Lu_2	500	0.0005	57	43
$Al_2Cl_2O_2$	38	0.0005	4	3
As_2Li_2Nd	125	0.0005	16	9
MnO_2	386	0.0005	43	57
Te_2Zr	173	0.0005	25	16
Sb_2SeTe_2	125	0.0005	16	9
DyI_2	512	0.0006	79	39
$Br_2Hf_2N_2$	231	0.0006	27	16
$F_2Lu_2Se_2$	699	0.0006	81	49
In_2Te_3	125	0.0006	16	9
Br_2Zr_2	824	0.0006	100	81
OTl_2	597	0.0006	81	64
$Cl_2Hf_2N_2$	789	0.0006	81	64
Pt_2Te_2	189	0.0007	25	16
Ga_2S_3	820	0.0007	91	73
I_2V	255	0.0007	36	25
NS_2Zr	747	0.0007	91	73
C_2	407	0.0008	49	81
CaH_2O_2	565	0.0008	64	49
Te_2W	597	0.0008	81	64
NaPSn	173	0.0008	25	16

Table showing the first 50 lattice matching combinations with the smallest strain needed to build the supercell.

Mg_2 (P4/nmm)

Structural and electronic properties

**Formula**	Mg_2
**Spacegroup**	P4/nmm
**Prototype**	Mg
**Parent 3D**	Mg_6Xe_4
**Source DB**	ICSD
**DB ID**	670330
**DF2-C09 Binding energy [meV/ Å^2]**	10.66
**RVV10 Binding energy [meV/ Å^2]**	N/A
**Band gap (PBE) [eV]**	0.0

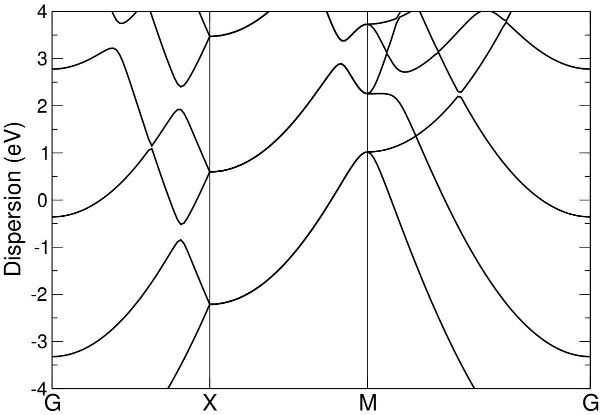

Band structure: Electronic band structure of Mg_2 (P4/nmm) in a representative window around the Fermi energy and along a high-symmetry path.

Optimized crystal structure

Structural parameters: relaxed cell (top) and atomic positions (bottom) of Mg_2 (P4/nmm) in Cartesian coordinates.

	x [Å]	y [Å]	z [Å]
$\mathbf{a_1}$	3.05582014	0.00000000	0.00000000
$\mathbf{a_2}$	0.00000000	3.05582014	0.00000000
$\mathbf{a_3}$	0.00000000	0.00000000	15.61693113

		x [Å]	y [Å]	z [Å]
●	Mg	0.76395503	-0.76395503	-1.16511431
●	Mg	-0.76395503	-2.29186510	1.16511431

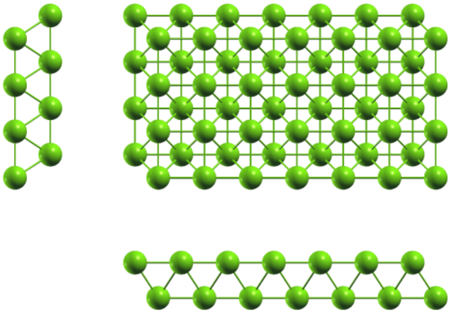

Orthographic projections: views of Mg_2 (P4/nmm) as seen from the x axis (left), the y axis (bottom) and the z axis (center).

Lattice matching - minimal size

Formula	N° atoms	strain	cell size 1	cell size 2
Tl	3	0.1456	1	1
Na	3	0.1789	1	1
As_2	4	0.1888	1	1
LiO	4	0.1347	1	1
BN	4	0.1105	1	1
P_2	4	0.141	1	1
Cl_2Zn	5	0.1875	1	1
S_2V	5	0.1293	1	1
MoS_2	5	0.1298	1	1
Cl_2Mn	5	0.151	1	1
$MoTe_2$	5	0.1816	1	1
$MoSe_2$	5	0.1449	1	1
$ReSe_2$	5	0.1645	1	1
S_2Ta	5	0.153	1	1
HfS_2	5	0.1924	1	1
$AsSn_2$	5	0.7751	1	1
Te_2V	5	0.184	1	1
$CuTe_2$	5	0.1919	1	1
Br_2Co	5	0.1883	1	1
Ca_2N	5	0.1895	1	1
Cl_2Ti	5	0.1411	1	1
S_2Ti	5	0.1581	1	1
NbS_2	5	0.1526	1	1
Te_2Zn	5	0.1814	1	1
$RhTe_2$	5	0.7861	1	1
S_2W	5	0.1299	1	1
Br_2Mn	5	0.186	1	1
Cl_2Ni	5	0.1655	1	1
Cl_2Co	5	0.1577	1	1
$CrTe_2$	5	0.1725	1	1
PtS_2	5	0.1801	1	1
NbS_2	5	0.1484	1	1
$CoTe_2$	5	0.1928	1	1
Br_2V	5	0.1636	1	1
ClNZr	5	0.1614	1	1
Cl_2Fe	5	0.1569	1	1
CdClO	5	0.1834	1	1
S_2Ta	5	0.1476	1	1
Se_2V	5	0.1464	1	1
Se_2Ti	5	0.1769	1	1
Br_2Ti	5	0.1718	1	1
Te_2W	5	0.1818	1	1
$AsSe_2$	5	0.1671	1	1
Cl_2V	5	0.1328	1	1
$PtSe_2$	5	0.7776	1	1
OTl_2	5	0.1837	1	1
CdO_2	5	0.1575	1	1
BrNZr	5	0.1686	1	1
$NbSe_2$	5	0.166	1	1
Br_2Fe	5	0.1884	1	1

Table showing the first 50 lattice matching combinations with the smallest number of atoms in the supercell.

Lattice matching - minimal strain

Formula	N° atoms	strain	cell size 1	cell size 2
$Ca_2Cl_2F_2$	806	0.0	136	89
$Er_2I_2O_2$	456	0.0	81	49
Bi_2O_2	68	0.0	16	9
$Br_2Eu_2F_2$	456	0.0	81	49
Ho_2S_2	362	0.0001	81	50
Bi_2Se_2Te	541	0.0002	108	65
$H_2Li_2O_2$	314	0.0002	49	36
I_2La	368	0.0002	97	58
CoO_2	291	0.0002	48	65
$Br_2Eu_2O_2$	602	0.0002	106	65
Co_2S_2	414	0.0003	85	61
CeI_2	407	0.0003	106	65
Cu_2O_2	362	0.0003	81	50
$Br_2Ce_2O_2$	800	0.0003	145	85
$K_2Mn_2Sb_2$	800	0.0003	145	85
$Br_2Er_2O_2$	146	0.0003	25	16
Sb_2SeTe_2	541	0.0004	108	65
$Bi_2Br_2O_2$	456	0.0004	81	49
$Fe_2Li_2P_2$	222	0.0004	36	25
NiO_2	291	0.0004	48	65
$H_2I_2Yb_2$	542	0.0004	97	58
In_2Te_3	541	0.0004	108	65
Ag_2Te_2	342	0.0004	75	48
I_2Pr	407	0.0005	106	65
$Br_2O_2Ti_2$	242	0.0005	40	27
As_2Rh_2	426	0.0005	97	58
$Br_2La_2O_2$	86	0.0005	16	9
GeI_2Y_2	541	0.0005	108	65
$Eu_2F_2I_2$	86	0.0005	16	9
Ni_2Se_2	472	0.0005	106	65
$Ca_2H_2I_2$	800	0.0006	145	85
$Hf_2Se_2Si_2$	736	0.0006	113	85
Br_2Cu_2	814	0.0006	181	113
NaPSn	479	0.0006	118	81
Pt_2Te_2	560	0.0006	118	81
$Cl_2O_2Sc_2$	826	0.0006	137	92
F_2Zn	701	0.0007	181	113
Ag_2Br_2	426	0.0007	97	58
$I_2O_2Tm_2$	456	0.0007	81	49
As_2Li_2Nd	541	0.0007	108	65
$I_2O_2Yb_2$	602	0.0008	106	65
$Eu_2I_2O_2$	800	0.0008	145	85
$Er_2F_2Se_2$	722	0.0009	118	81
FeO_2	291	0.0009	48	65
$GeNi_3Te_2$	722	0.0009	118	81
CaI_2	472	0.0009	131	70
CaCl	292	0.0009	85	61
Se_2Ta_4	694	0.001	101	82
$MnSe_2$	353	0.001	85	61
$HfTe_2$	479	0.001	118	81

Table showing the first 50 lattice matching combinations with the smallest strain needed to build the supercell.

Mg_2Cl_4 (P2/m)

Structural and electronic properties

**Formula**	Mg_2Cl_4
**Spacegroup**	P2/m
**Prototype**	Cl2Mg
**Parent 3D**	Cl_4Mg_2
**Source DB**	None
**DB ID**	None
**DF2-C09 Binding energy [meV/ Å^2]**	2.82
**RVV10 Binding energy [meV/ Å^2]**	N/A
**Band gap (PBE) [eV]**	4.52

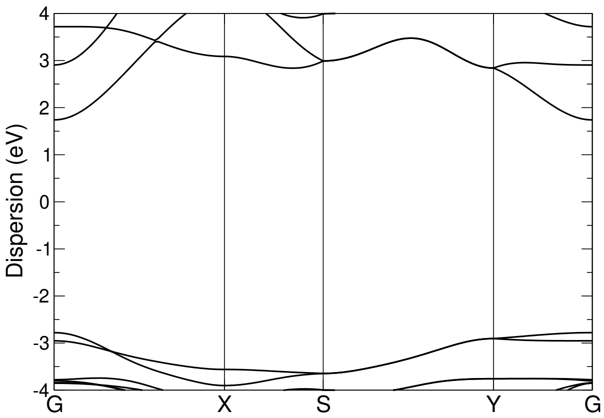

Band structure: Electronic band structure of Mg_2Cl_4 (P2/m) in a representative window around the Fermi energy and along a high-symmetry path.

Optimized crystal structure

Structural parameters: relaxed cell (top) and atomic positions (bottom) of Mg_2Cl_4 (P2/m) in Cartesian coordinates.

	x [Å]	y [Å]	z [Å]
$\mathbf{a_1}$	0.00000000	−3.66093990	0.00000000
$\mathbf{a_2}$	6.31263199	0.00000000	0.00000000
$\mathbf{a_3}$	0.00000000	0.00000000	19.14814024

		x [Å]	y [Å]	z [Å]
●	Cl	1.57815800	0.00000000	1.12058637
●	Cl	4.73447400	1.83046995	1.76820365
●	Mg	3.15631600	1.83046995	0.00000000
●	Mg	0.00000000	1.83046995	0.00000000
●	Cl	4.73447400	0.00000000	-1.12058637
●	Cl	1.57815800	1.83046995	-1.76820365

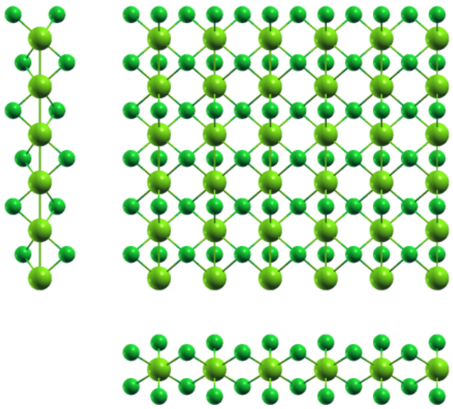

Orthographic projections: views of Mg_2Cl_4 (P2/m) as seen from the x axis (left), the y axis (bottom) and the z axis (center).

Lattice matching - minimal size

Formula	N° atoms	strain	cell size 1	cell size 2
Tl	8	0.0422	1	2
Bi_2	8	0.1443	1	1
PbTe	8	0.1425	1	1
CdI_2	9	0.1433	1	1
Br_2Ca	9	0.1438	1	1
Ca_2Si	9	0.7372	1	1
BiClTe	9	0.1434	1	1
BiBrTe	9	0.1461	1	1
GeI_2	9	0.1371	1	1
Cl_2Cu	9	0.1588	1	1
GeI_2	9	0.1426	1	1
I_2Pb	9	0.7274	1	1
BiITe	9	0.1497	1	1
GdI_2	9	0.1476	1	1
CdI_2	9	0.1431	1	1
I_2Pr	9	0.1435	1	1
Cu_2Sr_2	10	0.1467	1	1
$LiMnTe_2$	10	0.1427	1	1
HgO	10	0.1253	1	2
AsSb	10	0.5971	1	2
$AlLiTe_2$	10	0.149	1	1
Cl_2ORu	10	0.2356	1	1
Bi_2Se_2	10	0.1907	1	1
$AsCuLi_2$	10	0.1369	1	1
As_2	10	1.1785	1	2
Ga_2Te_2	10	0.137	1	1
Sb_2	10	0.2111	1	2
Ba_2Ni_3	11	0.137	1	1
Bi_2Te_3	11	0.1496	1	1
As_2CeLi_2	11	0.1425	1	1
I_2La_2P	11	0.1471	1	1
Bi_2STe_2	11	0.1429	1	1
Sb_2Te_3	11	0.1452	1	1
Bi_2SeTe_2	11	0.1446	1	1
Br_2La_2P	11	0.1431	1	1
Cl_2Zn	12	1.1727	1	2
Cl_2Mn	12	0.4289	1	2
$MoTe_2$	12	1.147	1	2
$AgTe_2$	12	0.0407	1	2
Br_2Zn	12	0.5664	1	2
HfS_2	12	0.5189	1	2
$AsSn_2$	12	0.5466	1	2
Cu_4Te_2	12	0.1455	1	1
$SiTe_2$	12	0.5787	1	2
Te_2V	12	1.1576	1	2
Hg_4O_2	12	1.0676	1	1
NSr_2	12	0.6022	1	2
I_2Yb	12	0.8556	1	2
Br_2Co	12	1.1761	1	2
$Se_2Si_2Zr_2$	12	0.2259	1	1

Table showing the first 50 lattice matching combinations with the smallest number of atoms in the supercell.

Lattice matching - minimal strain

Formula	N° atoms	strain	cell size 1	cell size 2
$SbSe_2Tl$	244	0.0007	24	25
$Cl_2Er_2O_2$	462	0.0011	27	50
Sb_2Te_2	808	0.0011	74	91
NbS_2	417	0.0011	32	75
STl_2	21	0.0011	2	3
$SiTe_2$	312	0.0011	27	50
Se_2Zr	312	0.0011	27	50
$RhTe_2$	447	0.0011	38	73
$Br_2Hf_2N_2$	438	0.0011	24	49
I_4Sr_2	258	0.0011	27	16
Au_2I_2	594	0.0011	55	66
S_2Ta	417	0.0011	32	75
Ca_2N	291	0.0011	24	49
$BrKO_3$	435	0.0011	50	27
$LiNbS_2$	492	0.0011	32	75
$F_2Se_2Y_2$	486	0.0011	38	43
In	26	0.0011	3	8
CdClHO	520	0.0012	38	73
As_2O_3	569	0.0012	54	49
Br_3Cs	932	0.0013	112	65
K_2O_4	672	0.0013	70	42
FeO_2	129	0.0013	8	27
Nd	260	0.0013	27	98
BaF_2	21	0.0013	2	3
$NiTe_2$	312	0.0013	27	50
HgI_2	870	0.0013	92	106
$Cl_2N_2Zr_2$	438	0.0013	24	49
As_2	242	0.0014	24	49
F_4Pb	502	0.0014	42	50
$CrTe_2$	312	0.0014	25	54
Hg_3S_2	625	0.0014	75	35
Li_2Tl_2	244	0.0014	24	25
CdClHO	678	0.0014	49	96
$Hf_2I_2N_2$	870	0.0014	49	96
Cl_2Sc_2	366	0.0014	25	54
Ho_2S_2	254	0.0014	21	32
CCl_2Lu_2	389	0.0014	24	49
$Ga_2I_2Y_2$	30	0.0015	2	3
AlH_4Na	330	0.0015	27	28
CaH_2O_2	389	0.0015	24	49
I_2V	312	0.0016	27	50
Br_2Cr	312	0.0016	25	54
Br_2Fe	291	0.0016	24	49
Ag_2I_2	976	0.0016	92	106
N_3W_2	676	0.0016	31	98
In_2Te_3	27	0.0016	2	3
Cl_2NSc_2	567	0.0017	32	75
Br_2Co	291	0.0017	24	49
$Br_2H_2Zr_2$	474	0.0017	25	54
S_2Ti	552	0.0017	43	98

Table showing the first 50 lattice matching combinations with the smallest strain needed to build the supercell.

Mg_2Cl_4 (P-1)

Structural and electronic properties

**Formula**	Mg_2Cl_4
**Spacegroup**	P-1
**Prototype**	Cl2Mg
**Parent 3D**	Cl_4Mg_2
**Source DB**	ICSD
**DB ID**	51247
**DF2-C09 Binding energy [meV/ Å^2]**	2.82
**RVV10 Binding energy [meV/ Å^2]**	N/A
**Band gap (PBE) [eV]**	5.44

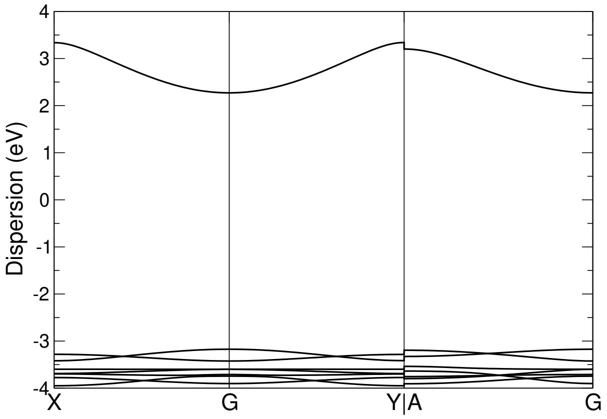

Band structure: Electronic band structure of Mg_2Cl_4 (P-1) in a representative window around the Fermi energy and along a high-symmetry path.

Optimized crystal structure

Structural parameters: relaxed cell (top) and atomic positions (bottom) of Mg_2Cl_4 (P-1) in Cartesian coordinates.

	x [Å]	y [Å]	z [Å]
$\mathbf{a_1}$	7.06635537	0.00114611	0.00000000
$\mathbf{a_2}$	2.95807344	6.42321804	0.00000000
$\mathbf{a_3}$	0.00000000	0.00000000	18.93739989

	x [Å]	y [Å]	z [Å]
Mg	2.75444052	-7.29100225	-0.00178167
Mg	-1.60423203	-5.55428771	0.00178167
Cl	-1.47903672	-3.21160902	0.00000000
Cl	0.57510425	-6.42264498	0.00000000
Cl	-2.95807404	-6.42602813	1.73853036
Cl	-2.95807284	-6.42040795	-1.73853036

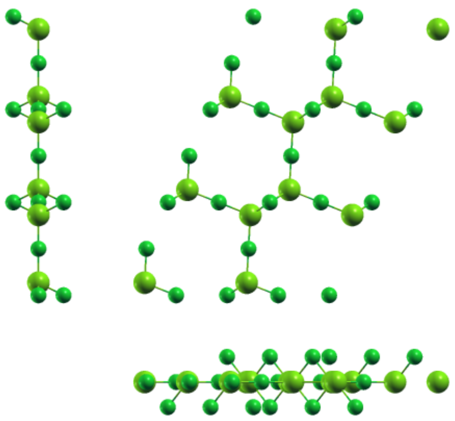

Orthographic projections: views of Mg_2Cl_4 (P-1) as seen from the x axis (left), the y axis (bottom) and the z axis (center).

Lattice matching - minimal size

Formula	N° atoms	strain	cell size 1	cell size 2
Br_2Cu	9	0.2569	1	1
Bi_2Te_2	10	0.1431	1	1
Cl_2Rb_2	10	0.0997	1	1
HgO	10	0.134	1	2
Tl	10	0.0373	1	4
Au_2Se_2	10	0.1238	1	1
LiO	10	0.1705	1	2
Na	10	0.0248	1	4
Fe_2SeTe	10	0.1699	1	1
Hg_3N_2	11	0.0281	1	1
IKO_3	11	0.0247	1	1
Hg_3S_2	11	0.0392	1	1
$BrKO_3$	11	0.0262	1	1
$Ge_2Hf_2Te_2$	12	0.1692	1	1
Cu_4Te_2	12	0.1475	1	1
Hg_4O_2	12	0.2112	1	1
Cl_4Cu_2	12	0.0932	1	1
$As_2Fe_2Li_2$	12	0.1698	1	1
$Br_2Lu_2O_2$	12	0.1702	1	1
$Br_2H_2Yb_2$	12	0.1698	1	1
$Ca_2Cl_2F_2$	12	0.1709	1	1
Se_4TiZr	12	0.1253	1	1
Mo_2Te_4	12	0.1295	1	1
Te_4W_2	12	0.1286	1	1
$Ge_2Te_2Zr_2$	12	0.1703	1	1
Cl_4KTl	12	0.1042	1	1
Cl_4Pd_2	12	0.1212	1	1
Cu_2O_2	14	0.2121	1	2
HgO	14	0.032	1	4
CdClHO	14	0.1291	1	2
AsSb	14	0.1428	1	4
Pb_2Se_2	14	0.0938	1	2
Bi_2	14	0.162	2	1
As_2	14	0.0243	1	4
Au_2Se_2	14	0.0314	1	2
Sb_2Te_2	14	0.3304	1	2
$AgClO_2$	14	0.2213	1	2
FHOZn	14	0.169	1	2
K	15	0.1048	2	3
CdI_2	15	0.162	2	1
Br_2Ca	15	0.162	2	1
BiClTe	15	0.162	2	1
GdI_2	15	0.1622	2	1
I_2Pr	15	0.162	2	1
Cu_2Sr_2	16	0.1622	2	1
$LiMnTe_2$	16	0.162	2	1
IO_3Tl	16	0.4255	1	2
S_2	16	0.1614	2	2
KNO_3	16	0.4001	1	2
I_2La_2P	17	0.1622	2	1

Table showing the first 50 lattice matching combinations with the smallest number of atoms in the supercell.

Lattice matching - minimal strain

Formula	N° atoms	strain	cell size 1	cell size 2
CKN	642	0.0008	52	110
$Cl_2N_2Ti_2$	840	0.0008	31	109
Br_2Cu	624	0.0009	39	130
$AsSn_2$	786	0.0009	45	172
Cu_2O_2	756	0.0009	42	126
CdClHO	958	0.0009	45	172
Bi_2O_2	576	0.0009	34	93
$Er_2I_2Se_2$	978	0.0009	56	107
$PtSe_2$	174	0.0009	10	38
$HNiO_2$	476	0.0009	16	95
$Br_2La_2O_2$	762	0.001	34	93
$Eu_2F_2I_2$	762	0.001	34	93
Ba_2Cu_2	596	0.0011	36	95
CdClHO	746	0.0011	35	134
$Ge_2Hf_2Te_2$	942	0.0011	37	120
I_2Yb	501	0.0012	36	95
Sb_2Se_2Te	955	0.0012	45	137
$Ho_2I_2Se_2$	978	0.0012	56	107
$AsSn_2$	174	0.0012	10	38
Bi_2Se_2	826	0.0012	51	130
Ag_2I_2	734	0.0013	49	110
I_4Sr_2	924	0.0013	71	83
$As_2Co_2Li_2$	942	0.0013	37	120
Sb_2Se_2Te	955	0.0013	45	137
I_2Mg	681	0.0013	45	137
Cl_2Gd_2	882	0.0014	45	153
Mg_6	780	0.0014	24	106
O_2Sn_2	328	0.0014	18	55
$BiTe_2$	681	0.0015	45	137
O_2Pb_2	576	0.0015	34	93
Br_2La	681	0.0015	45	137
I_3Sn	926	0.0015	81	110
Mg_6	456	0.0015	14	62
CdClHO	212	0.0015	10	38
Bi_2Se_3	955	0.0015	45	137
$AgClO_2$	818	0.0015	43	140
Cu_2Te_2	702	0.0015	37	120
$Cu_2Se_2Tl_2$	762	0.0016	34	93
$Br_2O_2Tb_2$	438	0.0016	18	55
I_4Sr_2	792	0.0016	61	71
$F_2I_2Sm_2$	762	0.0016	34	93
As_2Li_2Nd	247	0.0016	12	35
Se_2Yb	681	0.0016	45	137
BH_4Li	858	0.0016	35	108
Cu_2S_2	328	0.0016	18	55
GeI_2Y_2	247	0.0016	12	35
Ge_2Se_2	336	0.0016	20	54
LiO_2	579	0.0017	30	133
$Br_2O_2Y_2$	438	0.0017	18	55
$AgClO_2$	496	0.0017	26	85

Table showing the first 50 lattice matching combinations with the smallest strain needed to build the supercell.

Mg_3 (P4/mmm)

Structural and electronic properties

**Formula**	Mg_3
**Spacegroup**	P4/mmm
**Prototype**	Mg
**Parent 3D**	Mg_3Xe_2
**Source DB**	ICSD
**DB ID**	670329
**DF2-C09 Binding energy [meV/ Å^2]**	18.31
**RVV10 Binding energy [meV/ Å^2]**	N/A
**Band gap (PBE) [eV]**	0.0

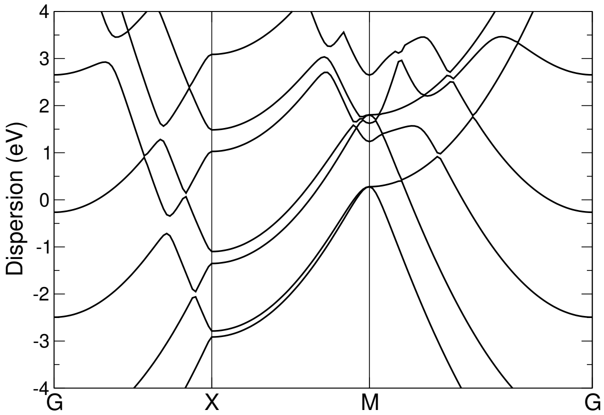

Band structure: Electronic band structure of Mg_3 (P4/mmm) in a representative window around the Fermi energy and along a high-symmetry path.

Optimized crystal structure

Structural parameters: relaxed cell (top) and atomic positions (bottom) of Mg_3 (P4/mmm) in Cartesian coordinates.

		x [Å]	y [Å]	z [Å]
$\mathbf{a_1}$		3.12394891	0.00000000	0.00000000
$\mathbf{a_2}$		0.00000000	3.12394891	0.00000000
$\mathbf{a_3}$		0.00000000	0.00000000	18.77350378
		x [Å]	y [Å]	z [Å]
●	Mg	0.00000000	-1.56197445	2.24101353
●	Mg	0.00000000	-1.56197445	-2.24101353
●	Mg	1.56197445	-3.12394891	0.00000000

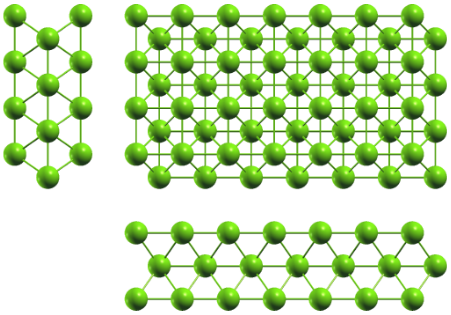

Orthographic projections: views of Mg_3 (P4/mmm) as seen from the x axis (left), the y axis (bottom) and the z axis (center).

Lattice matching - minimal size

Formula	N° atoms	strain	cell size 1	cell size 2
Tl	4	0.006	1	1
Tl	4	0.1367	1	1
Na	4	0.1667	1	1
K	5	2.1726	1	2
As_2	5	0.1759	1	1
P_2	5	0.1326	1	1
CaCl	5	1.1307	1	1
Cl_2Zn	6	0.1747	1	1
Cl_2Mn	6	0.1415	1	1
$MoTe_2$	6	0.1693	1	1
$AgTe_2$	6	0.0091	1	1
PSn_2	6	0.1869	1	1
$MoSe_2$	6	0.1361	1	1
$ReSe_2$	6	0.1536	1	1
S_2Ta	6	0.1433	1	1
HfS_2	6	0.1792	1	1
$AsSn_2$	6	0.1918	1	1
$SiTe_2$	6	0.7774	1	1
Te_2V	6	0.1715	1	1
$CuTe_2$	6	0.1788	1	1
S_2Zr	6	0.1859	1	1
Br_2Co	6	0.1754	1	1
Ca_2N	6	0.1765	1	1
Cl_2Ti	6	0.1327	1	1
S_2Ti	6	0.1478	1	1
NbS_2	6	0.1429	1	1
Te_2Zn	6	0.1691	1	1
$RhTe_2$	6	0.1955	1	1
Br_2Mn	6	0.1733	1	1
Cl_2Ni	6	0.1545	1	1
Cl_2Co	6	0.1475	1	1
$CrTe_2$	6	0.1609	1	1
PtS_2	6	0.1679	1	1
NbS_2	6	0.1392	1	1
$CoTe_2$	6	0.1796	1	1
Br_2V	6	0.1528	1	1
ClNZr	6	0.1508	1	1
Cl_2Fe	6	0.1468	1	1
CdClO	6	0.1709	1	1
S_2Ta	6	0.1385	1	1
Se_2V	6	0.1374	1	1
Se_2Ti	6	0.1649	1	1
Br_2Ti	6	0.1602	1	1
Te_2W	6	0.1695	1	1
$AsSe_2$	6	0.156	1	1
$NiTe_2$	6	0.7751	1	1
Cl_2Cu	6	0.181	1	1
S_2Sn	6	0.1863	1	1
I_2V	6	0.7826	1	1
Se_2Zr	6	0.779	1	1

Table showing the first 50 lattice matching combinations with the smallest number of atoms in the supercell.

Lattice matching - minimal strain

Formula	N° atoms	strain	cell size 1	cell size 2
$Ag_2K_2Se_2$	594	0.0	100	49
I_2La	882	0.0	181	113
GeS_2	441	0.0	82	65
Br_2Cu_2	764	0.0001	136	89
As_2Fe_2	499	0.0001	81	64
I_2Pr	123	0.0001	25	16
$Ba_2Ge_2Mn_2$	537	0.0002	81	49
CaCl	509	0.0002	113	85
As_2Rh_2	995	0.0003	181	113
HgI_2	486	0.0003	109	53
$Br_2O_2Tb_2$	942	0.0003	136	89
$MnSe_2$	594	0.0003	113	85
$Ca_2H_2I_2$	708	0.0003	106	65
Cu_2Se_2	499	0.0003	85	61
BiBrTe	519	0.0004	108	65
O_2Sn_2	681	0.0004	111	87
Cl_4Mn	47	0.0004	9	4
Co_2S_2	679	0.0004	113	85
$H_2Li_2O_2$	486	0.0005	64	49
$Cu_2Se_2Tl_2$	945	0.0005	145	85
$F_2I_2Yb_2$	537	0.0005	81	49
$I_2La_2O_2$	102	0.0005	16	9
Ag_2Br_2	995	0.0005	181	113
$I_2O_2Yb_2$	171	0.0005	25	16
Co_2Se_2	291	0.0005	49	36
$Br_2Eu_2O_2$	171	0.0005	25	16
$Br_2Ce_2O_2$	708	0.0005	106	65
$K_2Mn_2Sb_2$	708	0.0005	106	65
O_2Sn_2	681	0.0006	111	87
$I_2S_2Tl_2$	621	0.0006	85	61
$Hf_2Se_2Si_2$	627	0.0006	81	64
$I_2Nd_2O_2$	639	0.0006	97	58
CeI_2	123	0.0006	25	16
$Br_2F_2Yb_2$	942	0.0006	136	89
Bi_2O_2	775	0.0006	145	85
GeS_2	435	0.0007	81	64
Ag_2I_2	539	0.0008	109	53
Cl_2La_2	678	0.0008	118	81
H_2Na_2Pd	571	0.0008	82	65
$As_2Fe_2Li_2$	258	0.0008	36	25
$Br_2H_2Yb_2$	258	0.0008	36	25
As_2Co_2	291	0.0008	49	36
Ni_2Se_2	139	0.0009	25	16
$Eu_2H_2I_2$	537	0.0009	81	49
As_2Sn_2	678	0.0009	118	81
Ir_2P_2	139	0.0009	25	16
Bi_2Te_2	396	0.0009	80	39
F_2Ni	438	0.0009	85	61
$Si_2Te_2Zr_2$	621	0.0009	85	61
Br_2Gd_2	678	0.0009	118	81

Table showing the first 50 lattice matching combinations with the smallest strain needed to build the supercell.

Mg_4 (P4/nmm)

Structural and electronic properties

**Formula**	Mg_4
**Spacegroup**	P4/nmm
**Prototype**	Mg
**Parent 3D**	Mg_3Xe_2
**Source DB**	ICSD
**DB ID**	670329
**DF2-C09 Binding energy [meV/ Å^2]**	18.31
**RVV10 Binding energy [meV/ Å^2]**	N/A
**Band gap (PBE) [eV]**	0.0

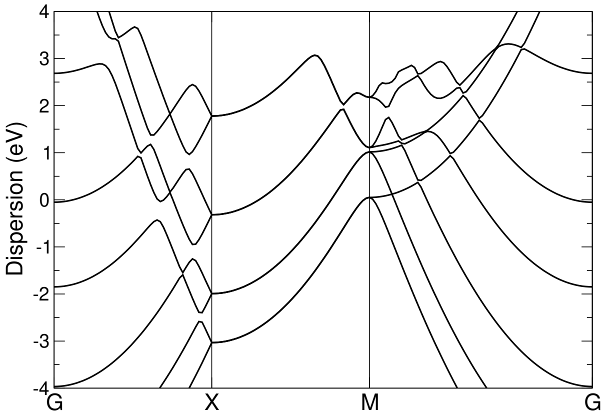

Band structure: Electronic band structure of Mg_4 (P4/nmm) in a representative window around the Fermi energy and along a high-symmetry path.

Optimized crystal structure

Structural parameters: relaxed cell (top) and atomic positions (bottom) of Mg_4 (P4/nmm) in Cartesian coordinates.

	x [Å]	y [Å]	z [Å]
$\mathbf{a_1}$	3.16104107	0.00000000	0.00000000
$\mathbf{a_2}$	0.00000000	3.16104107	0.00000000
$\mathbf{a_3}$	0.00000000	0.00000000	22.08014679

	x [Å]	y [Å]	z [Å]
Mg	0.79026027	-0.79026027	3.31639214
Mg	-0.79026027	-2.37078080	-3.31639214
Mg	0.79026027	-0.79026027	-1.10429245
Mg	-0.79026027	-2.37078080	1.10429245

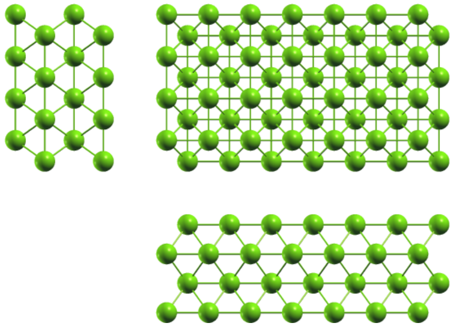

Orthographic projections: views of Mg_4 (P4/nmm) as seen from the x axis (left), the y axis (bottom) and the z axis (center).

Lattice matching - minimal size

Formula	N° atoms	strain	cell size 1	cell size 2
Tl	5	0.0003	1	1
Tl	5	0.1324	1	1
Na	5	0.1607	1	1
Sm	5	0.1098	1	1
AsSb	6	0.7794	1	1
As_2	6	0.1694	1	1
CaCl	6	0.408	1	1
Cl_2Zn	7	0.1682	1	1
Cl_2Mn	7	0.1368	1	1
$MoTe_2$	7	0.1631	1	1
$AgTe_2$	7	0.0033	1	1
PSn_2	7	0.1799	1	1
$MoSe_2$	7	0.1318	1	1
$ReSe_2$	7	0.1482	1	1
S_2Ta	7	0.1385	1	1
Br_2Zn	7	0.1935	1	1
HfS_2	7	0.1726	1	1
$AsSn_2$	7	0.1846	1	1
Te_2V	7	0.1652	1	1
$CuTe_2$	7	0.1721	1	1
S_2Zr	7	0.179	1	1
NSr_2	7	0.7858	1	1
PbS_2	7	0.7694	1	1
Br_2Co	7	0.1689	1	1
Ca_2N	7	0.17	1	1
LiO_2	7	0.1245	1	1
S_2Ti	7	0.1427	1	1
Mg_3	7	0.0055	1	1
Te_2Ti	7	0.1939	1	1
NbS_2	7	0.1381	1	1
CrI_2	7	0.7891	1	1
Te_2Zn	7	0.1629	1	1
$RhTe_2$	7	0.1882	1	1
Br_2Mn	7	0.1669	1	1
Cl_2Ni	7	0.1491	1	1
Cl_2Co	7	0.1424	1	1
$CrTe_2$	7	0.1551	1	1
PtS_2	7	0.1618	1	1
NbS_2	7	0.1347	1	1
$CoTe_2$	7	0.1729	1	1
Br_2V	7	0.1475	1	1
ClNZr	7	0.1456	1	1
Cl_2Fe	7	0.1418	1	1
CdClO	7	0.1646	1	1
S_2Ta	7	0.134	1	1
Se_2V	7	0.133	1	1
Se_2Ti	7	0.159	1	1
Br_2Ti	7	0.1545	1	1
Te_2W	7	0.1633	1	1
$AsSe_2$	7	0.1505	1	1

Table showing the first 50 lattice matching combinations with the smallest number of atoms in the supercell.

Lattice matching - minimal strain

Formula	N° atoms	strain	cell size 1	cell size 2
GeS_2	650	0.0001	101	82
O_2Pb_2	520	0.0001	81	49
Co_2S_2	460	0.0001	65	50
$Se_2Si_2Zr_2$	718	0.0001	82	65
Cu_2Te_2	584	0.0001	85	61
I_2Pr	811	0.0002	136	89
Ag_2I_2	892	0.0002	149	74
$I_2Nd_2O_2$	814	0.0002	106	65
$AlLiTe_2$	692	0.0003	108	65
I_2La	148	0.0003	25	16
$F_2I_2Sm_2$	618	0.0003	81	49
Tl	5	0.0003	1	1
Cu_2Se_2	340	0.0004	49	36
$H_2I_2Yb_2$	196	0.0004	25	16
As_2Fe_2	724	0.0004	100	81
$Cu_2Se_2Tl_2$	736	0.0004	97	58
GeS_2	643	0.0005	100	81
Cu_2I_2	620	0.0005	97	58
Co_2Se_2	792	0.0005	113	85
CaCl	360	0.0005	65	50
As_2Rh_2	164	0.0005	25	16
$As_2Co_2Li_2$	706	0.0006	85	61
Ir_2P_2	900	0.0006	136	89
$I_2S_2Tl_2$	412	0.0006	49	36
CuO_2	429	0.0006	60	63
$MnSe_2$	410	0.0006	65	50
$Ba_2F_2I_2$	694	0.0007	100	49
Br_2OV	640	0.0007	89	71
$Br_2O_2Yb_2$	294	0.0007	36	25
$Eu_2F_2I_2$	618	0.0007	81	49
Br_2La_2	796	0.0007	118	81
$Br_2La_2O_2$	618	0.0007	81	49
O_4PTl	694	0.0007	100	49
H_2Na_2Pd	814	0.0007	101	82
Fe_2O_4	548	0.0008	56	54
I_2Nd	811	0.0008	136	89
Sb_2	634	0.0008	118	81
Ag_2Br_2	164	0.0008	25	16
$F_2I_2Pb_2$	118	0.0008	16	9
$As_2Cd_2K_2$	754	0.0008	109	53
I_2Pb	734	0.0008	131	70
As_2Co_2	792	0.0008	113	85
HgI_2	818	0.0009	149	74
$Br_2H_2Sr_2$	736	0.0009	97	58
$Se_2Si_2Zr_2$	708	0.0009	81	64
$Hf_2Se_2Si_2$	886	0.0009	100	81
F_2Ni	304	0.0009	49	36
$Si_2Te_2Zr_2$	412	0.0009	49	36
CeI_2	811	0.0009	136	89
Ba_2Cd	91	0.0009	16	9

Table showing the first 50 lattice matching combinations with the smallest strain needed to build the supercell.

Mg_6 (P4/nmm)

Structural and electronic properties

**Formula**	Mg_6
**Spacegroup**	P4/nmm
**Prototype**	Mg
**Parent 3D**	Mg_6Xe_2
**Source DB**	ICSD
**DB ID**	670327
**DF2-C09 Binding energy [meV/ Å^2]**	12.96
**RVV10 Binding energy [meV/ Å^2]**	N/A
**Band gap (PBE) [eV]**	0.0

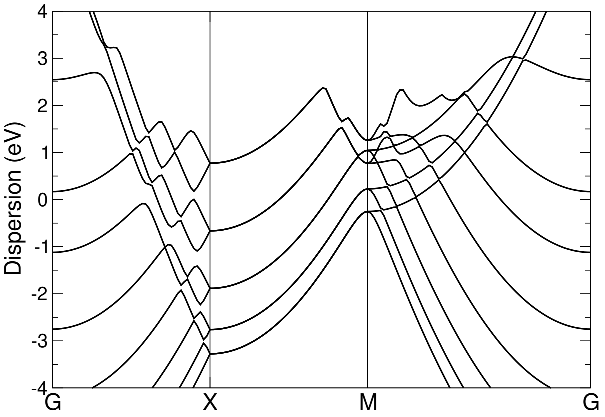

Band structure: Electronic band structure of Mg_6 (P4/nmm) in a representative window around the Fermi energy and along a high-symmetry path.

Optimized crystal structure

Structural parameters: relaxed cell (top) and atomic positions (bottom) of Mg_6 (P4/nmm) in Cartesian coordinates.

	x [Å]	y [Å]	z [Å]
$\mathbf{a_1}$	3.20247103	0.00000000	0.00000000
$\mathbf{a_2}$	0.00000000	3.20247103	0.00000000
$\mathbf{a_3}$	0.00000000	0.00000000	29.75389953

		x [Å]	y [Å]	z [Å]
●	Mg	0.80061776	-0.80061776	-5.41314191
●	Mg	-0.80061776	-2.40185327	5.41314191
●	Mg	0.80061776	-0.80061776	3.25796502
●	Mg	-0.80061776	-2.40185327	-3.25796502
●	Mg	0.80061776	-0.80061776	-1.08874934
●	Mg	-0.80061776	-2.40185327	1.08874934

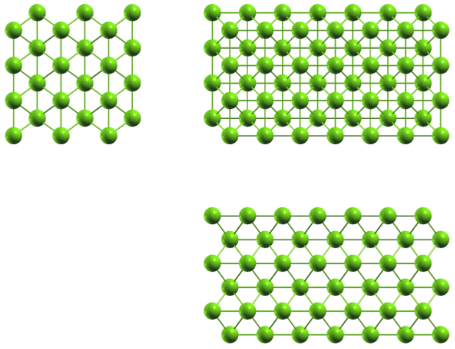

Orthographic projections: views of Mg_6 (P4/nmm) as seen from the x axis (left), the y axis (bottom) and the z axis (center).

Lattice matching - minimal size

Formula	N° atoms	strain	cell size 1	cell size 2
Tl	7	0.0057	1	1
Na	7	0.1543	1	1
Sm	7	0.1111	1	1
HgO	8	0.0064	1	1
GeTe	8	0.7791	1	1
As_2	8	0.1626	1	1
S_2	8	0.7852	1	1
CaCl	8	0.3943	1	1
$IrTe_2$	9	0.7826	1	1
Cl_2Zn	9	0.1615	1	1
$CdCl_2$	9	0.7749	1	1
Cl_2Mn	9	0.1321	1	1
$MoTe_2$	9	0.1566	1	1
$AgTe_2$	9	0.0028	1	1
PSn_2	9	0.1726	1	1
$ReSe_2$	9	0.1427	1	1
S_2Ta	9	0.1336	1	1
Br_2Zn	9	0.1856	1	1
HfS_2	9	0.1656	1	1
$InSe_2$	9	0.7764	1	1
$AsSn_2$	9	0.1771	1	1
$GeTe_2$	9	0.7711	1	1
$SiTe_2$	9	0.1909	1	1
Te_2V	9	0.1586	1	1
I_2Mn	9	0.7754	1	1
$CuTe_2$	9	0.1652	1	1
S_2Zr	9	0.1717	1	1
PbS_2	9	0.1955	1	1
Br_2Co	9	0.1621	1	1
Ca_2N	9	0.1632	1	1
LiO_2	9	0.1198	1	1
FeI_2	9	0.7669	1	1
I_2Ni	9	0.7718	1	1
S_2Ti	9	0.1375	1	1
Te_2Ti	9	0.1859	1	1
NbS_2	9	0.1333	1	1
Te_2Zn	9	0.1564	1	1
$RhTe_2$	9	0.1805	1	1
Br_2Mn	9	0.1602	1	1
Cl_2Ni	9	0.1435	1	1
Cl_2Co	9	0.1373	1	1
$CrTe_2$	9	0.1491	1	1
PtS_2	9	0.1554	1	1
NbS_2	9	0.1301	1	1
$CoTe_2$	9	0.1659	1	1
Br_2V	9	0.142	1	1
ClNZr	9	0.1402	1	1
Cl_2Fe	9	0.1367	1	1
CdClO	9	0.1581	1	1
S_2Ta	9	0.1295	1	1

Table showing the first 50 lattice matching combinations with the smallest number of atoms in the supercell.

Lattice matching - minimal strain

Formula	N° atoms	strain	cell size 1	cell size 2
F_2Tl_2	316	0.0	36	25
I_2Pr	489	0.0	61	41
$As_2Co_2Li_2$	510	0.0	49	36
P_2Rh_2	316	0.0001	36	25
$Hf_2Si_2Te_2$	690	0.0003	65	50
Cu_2I_2	896	0.0003	106	65
Co_2S_2	742	0.0003	81	64
$MnSe_2$	687	0.0003	82	65
Co_2Se_2	590	0.0003	65	50
C_2	198	0.0004	20	39
$Ca_2Cl_2F_2$	876	0.0004	85	61
CaCl	614	0.0004	81	64
$Ba_2H_2I_2$	972	0.0004	109	53
$I_2O_2Sm_2$	246	0.0004	25	16
Ag_2I_2	394	0.0004	49	25
$Br_2Dy_2O_2$	366	0.0004	36	25
$MnSe_2$	678	0.0004	81	64
I_3Sn	586	0.0004	81	25
$Br_2O_2Y_2$	366	0.0005	36	25
Cu_2Te_2	438	0.0005	49	36
$Fe_2Li_2P_2$	678	0.0005	64	49
$Ge_2Hf_2Te_2$	510	0.0005	49	36
Cu_2S_2	316	0.0005	36	25
$Eu_2I_2O_2$	246	0.0006	25	16
I_2Mg	951	0.0006	118	81
HgI_2	369	0.0006	49	25
$Ho_2I_2Se_2$	240	0.0007	28	12
Br_2La	951	0.0007	118	81
$Eu_2H_2I_2$	246	0.0007	25	16
$Cu_2K_2Te_2$	894	0.0008	100	49
F_4Nb	731	0.0008	81	49
$Hf_2Si_2Te_2$	678	0.0009	64	49
$Br_2F_2Sr_2$	780	0.0009	81	49
$Br_2Hf_2N_2$	918	0.001	90	63
Ho_2S_2	628	0.0011	72	49
$K_2Mn_2Sb_2$	246	0.0011	25	16
$Br_2Ce_2O_2$	246	0.0011	25	16
$F_2I_2Yb_2$	246	0.0011	25	16
$Gd_2I_2S_2$	174	0.0011	20	9
O_2Sn_2	316	0.0011	36	25
$Br_2F_2Pb_2$	780	0.0012	81	49
C_2Li_2	52	0.0012	6	4
$Br_2Hf_2N_2$	876	0.0012	86	60
$Br_2Ho_2O_2$	366	0.0013	36	25
$Br_2Er_2Se_2$	546	0.0013	63	28
Cl_4Mg_2	780	0.0014	106	24
$Ba_2Ge_2Mn_2$	246	0.0014	25	16
Co_2Se_2	580	0.0014	64	49
$Ca_2Mn_2Si_2$	366	0.0015	36	25
$BiTe_2$	951	0.0015	118	81

Table showing the first 50 lattice matching combinations with the smallest strain needed to build the supercell.

$MgBr_2$ (P-3m1)

Structural and electronic properties

**Formula**	$MgBr_2$
**Spacegroup**	P-3m1
**Prototype**	CdI2
**Parent 3D**	$MgBr_2$
**Source DB**	COD
**DB ID**	9009107
**DF2-C09 Binding energy [meV/ Å^2]**	10.2
**RVV10 Binding energy [meV/ Å^2]**	15.29
**Band gap (PBE) [eV]**	4.78

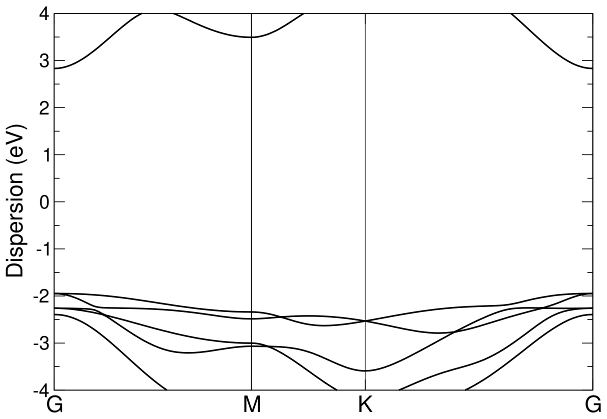

Band structure: Electronic band structure of $MgBr_2$ (P-3m1) in a representative window around the Fermi energy and along a high-symmetry path.

Optimized crystal structure

Structural parameters: relaxed cell (top) and atomic positions (bottom) of $MgBr_2$ (P-3m1) in Cartesian coordinates.

	x [Å]	y [Å]	z [Å]
$\mathbf{a_1}$	3.86630519	0.00000000	0.00000000
$\mathbf{a_2}$	−1.93315259	3.34831851	0.00000000
$\mathbf{a_3}$	0.00000000	0.00000000	23.03816395

		x [Å]	y [Å]	z [Å]
●	Br	0.00000000	2.23221234	13.03171210
●	Mg	-0.00000000	-0.00000000	11.51908197
●	Br	1.93315259	1.11610617	10.00645185

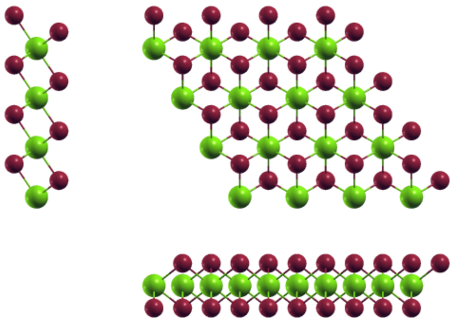

Orthographic projections: views of $MgBr_2$ (P-3m1) as seen from the x axis (left), the y axis (bottom) and the z axis (center).

Lattice matching - minimal size

Formula	N° atoms	strain	cell size 1	cell size 2
Tl	4	0.422	1	1
Tl	4	1.5542	1	1
InSe	5	0.4473	1	1
HgO	5	0.1116	1	1
AsSb	5	0.0028	1	1
Bi_2	5	0.4616	1	1
GeTe	5	0.0033	1	1
S_2	5	0.0049	1	1
PbTe	5	0.4516	1	1
CaCl	5	0.1411	1	1
$IrTe_2$	6	0.0042	1	1
$CdCl_2$	6	0.0022	1	1
Cl_2Mn	6	1.5961	1	1
CdI_2	6	0.4559	1	1
$AgTe_2$	6	0.4287	1	1
$MoSe_2$	6	1.5488	1	1
$ReSe_2$	6	0.2631	1	1
S_2Ta	6	0.2481	1	1
Br_2Ca	6	0.4589	1	1
CaI_2	6	2.9648	1	1
$InSe_2$	6	0.0026	1	1
$GeTe_2$	6	0.0012	1	1
$SiTe_2$	6	0.0087	1	1
I_2Mn	6	0.0023	1	1
NSr_2	6	0.0011	1	1
I_2Yb	6	2.9315	1	1
PbS_2	6	0.0053	1	1
BiClTe	6	0.4568	1	1
LiO_2	6	0.0661	1	1
Cl_2Zn	6	0.1546	1	1
FeI_2	6	0.0001	1	1
I_2Ni	6	0.0014	1	1
S_2Ti	6	0.2547	1	1
NbS_2	6	0.2475	1	1
CrI_2	6	0.0003	1	1
BiBrTe	6	0.4706	1	1
Bi_2Pd	6	0.1154	1	1
Cl_2Ni	6	0.2643	1	1
Cl_2Co	6	0.2542	1	1
$CrTe_2$	6	0.2732	1	1
Br_2V	6	0.2619	1	1
ClNZr	6	0.259	1	1
Cl_2Fe	6	1.6409	1	1
Se_2V	6	1.5603	1	1
Br_2Ti	6	0.2723	1	1
$AsSe_2$	6	0.2664	1	1
I_2V	6	0.0074	1	1
Cl_2V	6	1.4467	1	1
GeI_2	6	0.4522	1	1
Se_2Zr	6	0.0083	1	1

Table showing the first 50 lattice matching combinations with the smallest number of atoms in the supercell.

Lattice matching - minimal strain

Formula	N° atoms	strain	cell size 1	cell size 2
Cd_2I_3	437	0.0	64	49
MnO_2	222	0.0	25	49
Cl_2Ni	492	0.0	73	91
AsI_2La_2	437	0.0	64	49
NaO_4	93	0.0001	16	9
Nd	247	0.0001	49	100
Br_2Hf_2	643	0.0001	81	100
Hg_3N_2	368	0.0001	81	25
FeI_2	6	0.0001	1	1
I_2La_2P	563	0.0002	81	64
$CrSe_2$	183	0.0002	25	36
Bi_2	462	0.0002	100	81
Cu_4Te_2	711	0.0002	91	73
C	129	0.0002	16	81
ClNZr	435	0.0002	64	81
$Dy_2I_2S_2$	363	0.0003	49	36
CrI_2	6	0.0003	1	1
$Ga_2Gd_2I_2$	786	0.0003	100	81
$MoSe_2$	255	0.0003	36	49
Br_2Ca_3Si	483	0.0003	65	48
NS_2Ta	471	0.0003	49	81
Cl_2Hf_2	403	0.0003	49	64
$I_2Pr_2S_2$	258	0.0003	36	25
$Br_2H_2Zr_2$	678	0.0003	64	81
Sb_2Te_3	638	0.0004	91	73
CoH_2O_2	173	0.0004	16	25
DyI_2	255	0.0004	49	36
Se_2W	255	0.0004	36	49
$NbSe_2$	492	0.0004	73	91
Ba_2Hg	339	0.0005	65	48
Cl_2Mn	300	0.0005	43	57
$AlLiTe_2$	447	0.0005	73	57
BrNZr	543	0.0005	81	100
Se_2Ta	492	0.0005	73	91
I_2Pb	183	0.0005	36	25
$NbSe_2$	543	0.0005	81	100
$Ga_2I_2Tb_2$	786	0.0005	100	81
S_2Ti	390	0.0006	57	73
N_4	212	0.0006	24	35
In	361	0.0006	81	118
Br_3Cs	211	0.0007	49	16
O_2Sn_2	357	0.0007	55	48
Ca_4Cu_2	624	0.0007	102	53
CCl_2Sc_2	597	0.0007	64	81
$AgNO_3$	93	0.0007	16	9
Bi_2SeTe_2	705	0.0008	100	81
GdI_2	435	0.0008	81	64
$AsSe_2$	543	0.0008	81	100
$LiNbS_2$	403	0.0008	49	64
$I_2S_2Tb_2$	363	0.0009	49	36

Table showing the first 50 lattice matching combinations with the smallest strain needed to build the supercell.

$MgCl_2$ (P-3m1)

Structural and electronic properties

**Formula**	$MgCl_2$
**Spacegroup**	P-3m1
**Prototype**	CdI2
**Parent 3D**	$MgCl_2$
**Source DB**	COD
**DB ID**	8103685
**DF2-C09 Binding energy [meV/ Å^2]**	12.35
**RVV10 Binding energy [meV/ Å^2]**	17.69
**Band gap (PBE) [eV]**	6.03

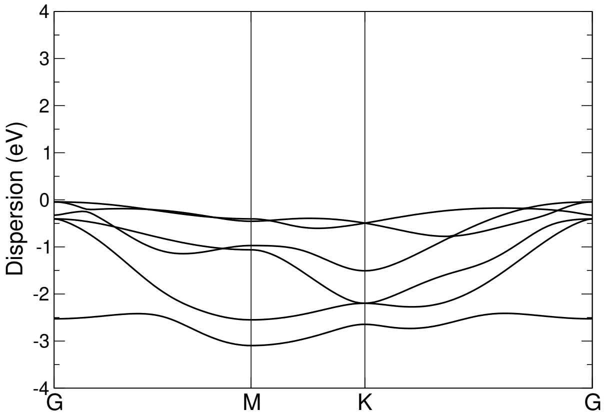

Band structure: Electronic band structure of $MgCl_2$ (P-3m1) in a representative window around the Fermi energy and along a high-symmetry path.

Optimized crystal structure

Structural parameters: relaxed cell (top) and atomic positions (bottom) of $MgCl_2$ (P-3m1) in Cartesian coordinates.

	x [Å]	y [Å]	z [Å]
$\mathbf{a_1}$	3.64533158	0.00000000	0.00000000
$\mathbf{a_2}$	−1.82266579	3.15694975	0.00000000
$\mathbf{a_3}$	0.00000000	0.00000000	22.72622643

	x [Å]	y [Å]	z [Å]
Mg	1.82266579	1.05231658	11.36311397
Cl	1.82266579	3.15694975	12.74506878
Cl	0.00000000	2.10463317	9.98115690

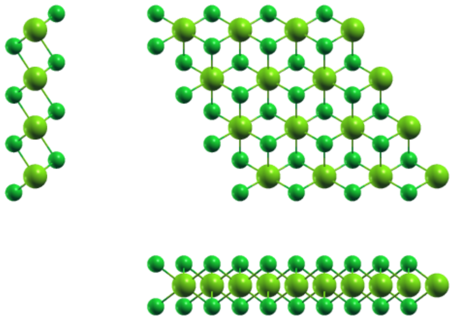

Orthographic projections: views of $MgCl_2$ (P-3m1) as seen from the x axis (left), the y axis (bottom) and the z axis (center).

Lattice matching - minimal size

Formula	N° atoms	strain	cell size 1	cell size 2
Tl	4	0.1183	1	1
In	4	0.4216	1	1
In	4	1.586	1	1
InSe	5	2.9852	1	1
HgO	5	0.1253	1	1
As_2	5	0.0047	1	1
LiO	5	0.2576	1	1
P_2	5	0.2683	1	1
PbTe	5	3.0088	1	1
Mg_2	5	0.1114	1	1
Sb_2	5	0.4769	1	1
Cl_2Zn	6	0.0057	1	1
I_2Mg	6	2.8511	1	1
S_2V	6	0.2477	1	1
MoS_2	6	0.2487	1	1
CdI_2	6	3.0319	1	1
$AgTe_2$	6	0.1199	1	1
PSn_2	6	0.0042	1	1
$MoSe_2$	6	0.2749	1	1
HfS_2	6	0.002	1	1
$AsSn_2$	6	0.008	1	1
Te_2V	6	0.0084	1	1
$CuTe_2$	6	0.0024	1	1
S_2Zr	6	0.0034	1	1
Br_2La	6	2.8554	1	1
Br_2Cu	6	1.0228	1	1
Br_2Co	6	0.0051	1	1
BiClTe	6	3.0368	1	1
ReS_2	6	1.523	1	1
Ca_2N	6	0.0042	1	1
Cl_2Ti	6	0.2685	1	1
$AuTe_2$	6	0.457	1	1
BrCdI	6	0.5	1	1
$PdTe_2$	6	0.451	1	1
HgI_2	6	0.3362	1	1
Mg_3	6	0.1155	1	1
I_2Zn	6	0.4685	1	1
S_2W	6	0.2488	1	1
Bi_2Pd	6	0.1318	1	1
GeI_2	6	0.4868	1	1
Br_2Mn	6	0.0069	1	1
$CoTe_2$	6	0.0017	1	1
CdClO	6	0.0088	1	1
AsKSn	6	2.9157	1	1
$PbTe_2$	6	0.4974	1	1
S_2Sn	6	0.0037	1	1
$SnTe_2$	6	0.4809	1	1
Cl_2V	6	0.2541	1	1
GeI_2	6	3.0119	1	1
$PtSe_2$	6	0.0087	1	1

Table showing the first 50 lattice matching combinations with the smallest number of atoms in the supercell.

Lattice matching - minimal strain

Formula	N° atoms	strain	cell size 1	cell size 2
CNb_2S_2	743	0.0	81	100
$CeLi_2P_2$	504	0.0	73	57
Br_2Ni	6	0.0001	1	1
Bi_2STe_2	327	0.0001	49	36
AsKSn	300	0.0001	57	43
Br_2La_2	499	0.0001	81	64
$H_2I_2Yb_2$	483	0.0002	65	48
PTe_2Ti_2	8	0.0002	1	1
MoS_2	339	0.0002	49	64
$CrSe_2$	390	0.0003	57	73
$Cl_2H_2Lu_2$	9	0.0003	1	1
C_2F_2	271	0.0003	25	49
Br_2Ca_3Si	171	0.0003	25	16
Sb_2	371	0.0003	81	64
S_2W	339	0.0004	49	64
I_2La_2Te	155	0.0004	25	16
I_2La_2	343	0.0004	57	43
Br_2HLa	447	0.0004	73	57
MoS_2	339	0.0004	49	64
As_2Sn_2	624	0.0005	100	81
In_2Se_2	565	0.0005	91	73
Br_2Gd_2Ge	437	0.0005	64	49
Cu_2I_2	388	0.0005	64	49
P_2	443	0.0005	81	100
Ga_2S_2	7	0.0005	1	1
N_3W_2	173	0.0005	16	25
Br_2Gd_2	624	0.0006	100	81
$AsLi_3$	291	0.0006	49	36
Cl_2La_2	624	0.0006	100	81
O_2Zn	183	0.0006	25	36
$AlLiTe_2$	208	0.0006	36	25
BH_4Li	561	0.0006	73	57
$Br_2H_2Sr_2$	840	0.0006	118	81
I_2Zn	492	0.0006	91	73
Cl_2Ti	543	0.0007	81	100
Cu_2I_2	678	0.0007	118	81
Br_2OV	546	0.0007	82	75
$PbTe_2$	339	0.0007	64	49
I_2La	339	0.0007	65	48
CS_2Ta_2	743	0.0007	81	100
$Br_2Nd_2O_2$	483	0.0007	65	48
KNO_3	368	0.0008	81	25
Cl_4Pd_2	78	0.0008	14	6
Gd_2I_2	388	0.0008	64	49
$F_2Se_2Y_2$	102	0.0008	16	9
InSe	219	0.0009	49	36
In	186	0.0009	43	57
Bi_2Se_4	264	0.0009	52	18
$AsCuLi_2$	447	0.0009	73	57
As_2Li_2Pr	327	0.0009	49	36

Table showing the first 50 lattice matching combinations with the smallest strain needed to build the supercell.

MgI_2 (P-3m1)

Structural and electronic properties

**Formula**	MgI_2
**Spacegroup**	P-3m1
**Prototype**	CdI2
**Parent 3D**	MgI_2
**Source DB**	COD
**DB ID**	9009108
**DF2-C09 Binding energy [meV/ Å^2]**	10.76
**RVV10 Binding energy [meV/ Å^2]**	16.16
**Band gap (PBE) [eV]**	3.6

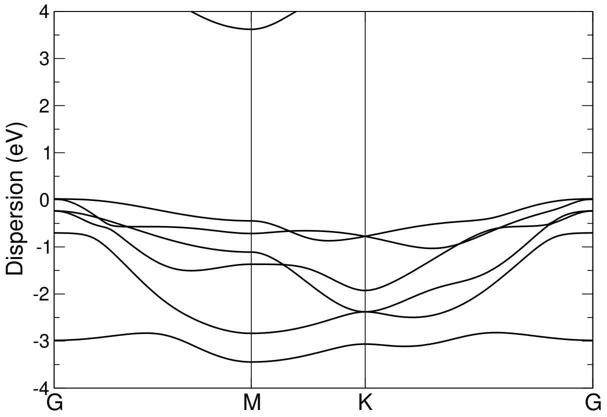

Band structure: Electronic band structure of MgI_2 (P-3m1) in a representative window around the Fermi energy and along a high-symmetry path.

Optimized crystal structure

Structural parameters: relaxed cell (top) and atomic positions (bottom) of MgI_2 (P-3m1) in Cartesian coordinates.

	x [Å]	y [Å]	z [Å]
$\mathbf{a_1}$	4.15353501	0.00000000	0.00000000
$\mathbf{a_2}$	−2.07676750	3.59706683	0.00000000
$\mathbf{a_3}$	0.00000000	0.00000000	23.37429551

		x [Å]	y [Å]	z [Å]
●	I	-0.00000000	2.39804456	9.98447167
●	Mg	0.00000000	0.00000000	11.68714775
●	I	2.07676750	1.19902228	13.38982383

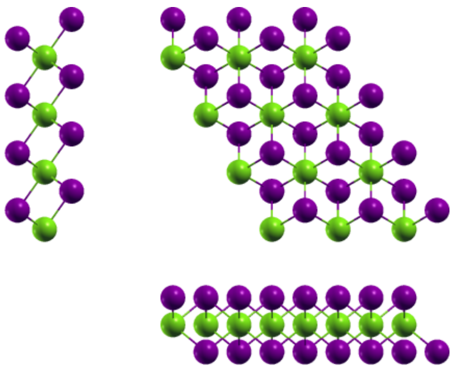

Orthographic projections: views of MgI_2 (P-3m1) as seen from the x axis (left), the y axis (bottom) and the z axis (center).

Lattice matching - minimal size

Formula	N° atoms	strain	cell size 1	cell size 2
K	4	0.4513	1	1
Na	4	1.5357	1	1
AgTl	5	0.1631	1	1
Ag_2	5	0.4648	1	1
As_2	5	1.5934	1	1
Sb_2	5	0.0056	1	1
CaCl	5	0.118	1	1
Cl_2Zn	6	1.5857	1	1
$MoTe_2$	6	1.5519	1	1
PSn_2	6	0.2567	1	1
Ba_2Pt	6	0.4642	1	1
$ReSe_2$	6	1.4495	1	1
Br_2Zn	6	0.2719	1	1
HfS_2	6	0.2485	1	1
$AsSn_2$	6	0.262	1	1
Te_2V	6	1.5658	1	1
I_2Pr	6	0.1452	1	1
$CuTe_2$	6	0.248	1	1
S_2Zr	6	0.2557	1	1
Br_2La	6	0.0003	1	1
Ca_2Si	6	0.4756	1	1
Br_2Co	6	1.5903	1	1
Ca_2N	6	1.5973	1	1
BrCdI	6	0.0032	1	1
Cl_2Zn	6	0.1264	1	1
Te_2Ti	6	0.2723	1	1
I_2Zn	6	0.0088	1	1
BaF_2	6	0.0065	1	1
$RhTe_2$	6	0.266	1	1
GeI_2	6	0.0018	1	1
PtS_2	6	1.543	1	1
$CoTe_2$	6	0.2489	1	1
CdClO	6	1.5623	1	1
Se_2Ti	6	1.5242	1	1
AsKSn	6	0.0051	1	1
Te_2W	6	1.5531	1	1
$PbTe_2$	6	0.0022	1	1
I_2Nd	6	0.1461	1	1
S_2Sn	6	0.256	1	1
$SnTe_2$	6	0.0041	1	1
Sn	6	0.6274	1	3
I_2Pb	6	0.4688	1	1
STl_2	6	0.0075	1	1
$PtSe_2$	6	0.2629	1	1
OTl_2	6	1.5636	1	1
Br_2Fe	6	1.5908	1	1
GeS_2	6	0.1127	1	1
$TaTe_2$	6	0.2611	1	1
$MnSe_2$	6	0.118	1	1
Br_2Ni	6	1.6286	1	1

Table showing the first 50 lattice matching combinations with the smallest number of atoms in the supercell.

Lattice matching - minimal strain

Formula	N° atoms	strain	cell size 1	cell size 2
CCl_2Lu_2	414	0.0	43	57
$I_2N_2Zr_2$	843	0.0	81	100
$NbTe_2$	390	0.0	57	73
$I_2Pr_2S_2$	711	0.0001	91	73
$Ce_2I_2S_2$	627	0.0001	81	64
Ca_2Si	435	0.0001	81	64
As_2	243	0.0001	43	57
Ga_2S_2	403	0.0001	49	64
S_2Zr	390	0.0002	57	73
LiO	309	0.0002	49	81
Au_2Se_2	349	0.0002	63	40
Br_2Fe	300	0.0002	43	57
$Ce_2I_2Si_2$	9	0.0002	1	1
Cl_2Ni	183	0.0002	25	36
$HfLiS_2$	304	0.0002	36	49
Tl	73	0.0003	16	25
C_2Li_2	276	0.0003	40	39
$Br_2N_2Ti_2$	711	0.0003	73	82
Br_2Co	300	0.0003	43	57
$CoTe_2$	339	0.0003	49	64
Br_2La	6	0.0003	1	1
CdClHO	583	0.0004	73	91
$I_2N_2Ti_2$	846	0.0004	90	96
Bi_2Se_3	8	0.0004	1	1
S_2Sn	390	0.0004	57	73
LiO	233	0.0005	37	61
Te_2Zn	255	0.0005	36	49
PtS_2	255	0.0005	36	49
Sb_2Se_2Te	8	0.0006	1	1
$F_2Se_2Y_2$	363	0.0006	49	36
Se_2V	123	0.0006	16	25
HfS_2	339	0.0006	49	64
Mg_6	951	0.0006	81	118
Gd_2I_2	7	0.0006	1	1
$NbSe_2$	183	0.0007	25	36
Ca_2N	300	0.0007	43	57
$ReSe_2$	183	0.0007	25	36
$MoTe_2$	255	0.0007	36	49
Cl_2OV	89	0.0007	11	14
$I_2Nd_2S_2$	786	0.0007	100	81
Se_2Ta	183	0.0008	25	36
I_2Pb	492	0.0008	91	73
CdClHO	516	0.0008	64	81
$Hf_2I_2N_2$	678	0.0008	64	81
Ag_2	419	0.0008	91	73
Se_2W	123	0.0008	16	25
N_3W_2	512	0.0009	39	79
Te_2W	255	0.0009	36	49
$HNiO_2$	271	0.0009	25	49
Cl_2Zn	300	0.0009	43	57

Table showing the first 50 lattice matching combinations with the smallest strain needed to build the supercell.

$MnBr_2$ (C2/m)

Structural and electronic properties

**Formula**	$MnBr_2$
**Spacegroup**	C2/m
**Prototype**	CdI2
**Parent 3D**	$MnBr_2$
**Source DB**	ICSD
**DB ID**	67500
**DF2-C09 Binding energy [meV/ Å^2]**	15.04
**RVV10 Binding energy [meV/ Å^2]**	21.62
**Band gap (PBE) [eV]**	1.85

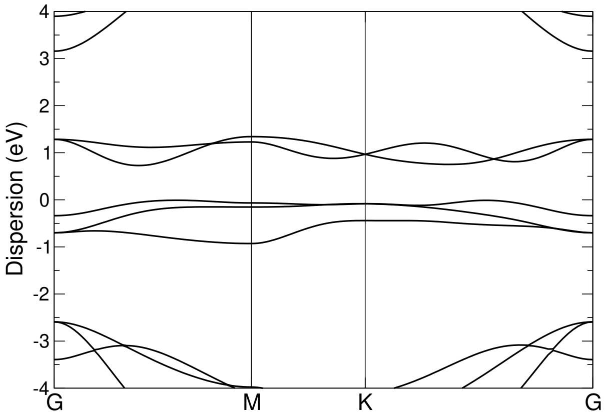

Band structure: Electronic band structure of $MnBr_2$ (C2/m) in a representative window around the Fermi energy and along a high-symmetry path.

Optimized crystal structure

Structural parameters: relaxed cell (top) and atomic positions (bottom) of $MnBr_2$ (C2/m) in Cartesian coordinates.

		x [Å]	y [Å]	z [Å]
$\mathbf{a_1}$		3.59157104	0.00000000	0.00000000
$\mathbf{a_2}$		−1.79578552	3.11039176	0.00000000
$\mathbf{a_3}$		0.00000000	0.00000000	22.74343183
		x [Å]	y [Å]	z [Å]
●	Br	1.79578552	1.03679725	9.99338748
●	Mn	0.00000000	0.00000000	11.37171592
●	Br	0.00000000	2.07359451	12.75004435

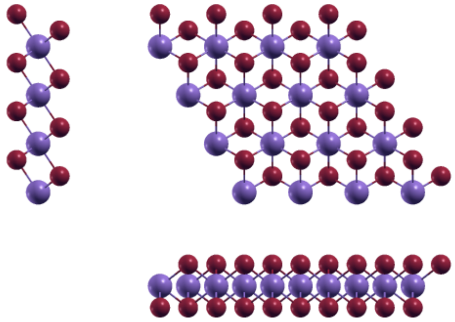

Orthographic projections: views of $MnBr_2$ (C2/m) as seen from the x axis (left), the y axis (bottom) and the z axis (center).

Lattice matching - minimal size

Formula	N° atoms	strain	cell size 1	cell size 2
K	4	0.3151	1	1
Tl	4	0.1222	1	1
Sn	4	0.4274	1	1
Na	4	0.0056	1	1
In	4	1.6383	1	1
HgO	5	0.13	1	1
As_2	5	0.0023	1	1
LiO	5	0.2674	1	1
Mg_2	5	0.114	1	1
Sb_2	5	0.4952	1	1
CrS_2	6	1.5302	1	1
Cl_2Zn	6	0.0012	1	1
S_2V	6	0.2571	1	1
MoS_2	6	0.2581	1	1
$MoTe_2$	6	0.0034	1	1
$AgTe_2$	6	0.124	1	1
HfS_2	6	0.005	1	1
$HfTe_2$	6	0.4552	1	1
Te_2V	6	0.0015	1	1
$CuTe_2$	6	0.0047	1	1
Br_2Co	6	0.0018	1	1
Ca_2N	6	0.0028	1	1
$AuTe_2$	6	0.4747	1	1
$PdTe_2$	6	0.4685	1	1
Mg_3	6	0.1189	1	1
I_2Zn	6	0.4866	1	1
Te_2Zn	6	0.0036	1	1
S_2W	6	0.2582	1	1
Bi_2Pd	6	0.1373	1	1
GeI_2	6	2.9175	1	1
PtS_2	6	0.0046	1	1
$CoTe_2$	6	0.0054	1	1
CdClO	6	0.002	1	1
Ba_2N	6	0.459	1	1
Se_2Ti	6	0.0072	1	1
AsKSn	6	3.0084	1	1
Te_2Zr	6	0.4564	1	1
Te_2W	6	0.0033	1	1
Cl_2Cu	6	0.5866	1	1
$SnTe_2$	6	0.4993	1	1
Cl_2V	6	0.2638	1	1
STl_2	6	3.0406	1	1
OTl_2	6	0.0018	1	1
Br_2Fe	6	0.0019	1	1
Br_2Ni	6	0.0071	1	1
Se_2Yb	6	2.9206	1	1
MoS_2	6	0.2584	1	1
Cl_2Mg	6	0.0071	1	1
$BiTe_2$	6	2.924	1	1
$CrSe_2$	6	0.2622	1	1

Table showing the first 50 lattice matching combinations with the smallest number of atoms in the supercell.

Lattice matching - minimal strain

Formula	N° atoms	strain	cell size 1	cell size 2
Ga_2Te_2	343	0.0	57	43
Ba_2Ni_3	386	0.0	57	43
Br_2Er_2	499	0.0	81	64
$I_2Pr_2Si_2$	429	0.0001	57	43
Sb_2	290	0.0001	64	49
LiO	443	0.0001	81	100
In_2Se_2	447	0.0002	73	57
CaI_2	123	0.0002	25	16
Br_2Ca_3Si	615	0.0002	91	57
$AsCuLi_2$	343	0.0002	57	43
GeI_2	300	0.0002	57	43
$Bi_2Cl_2O_2$	483	0.0003	65	48
Br_2Cd	492	0.0003	91	73
Br_2La_2	388	0.0003	64	49
H_2MgO_2	536	0.0003	57	73
Cl_2V	492	0.0003	73	91
Sb_2Te_3	233	0.0004	36	25
I_2La_2	291	0.0004	49	36
$I_2Nd_2S_2$	405	0.0004	61	37
Ge_2Se_2	581	0.0004	103	68
KS_2Ti	7	0.0004	1	1
$Al_2Cl_2O_2$	666	0.0004	76	73
Se_2Yb	300	0.0005	57	43
Li_2P_2Pr	437	0.0005	64	49
Br_2PY_2	638	0.0005	91	73
$AuTe_2$	435	0.0005	81	64
H_2NiO_2	597	0.0005	64	81
Br_2Gd_2	499	0.0006	81	64
Se_2Sn_2	398	0.0006	74	44
$Ga_2Ge_2Te_2$	486	0.0006	64	49
$PdTe_2$	492	0.0006	91	73
$I_2Nd_2O_2$	840	0.0006	118	81
MoS_2	435	0.0006	64	81
As_2Sn_2	499	0.0007	81	64
Br_2HLa	343	0.0007	57	43
$F_2Ho_2Se_2$	786	0.0007	100	81
$BiTe_2$	300	0.0007	57	43
Bi_2SeTe_2	233	0.0008	36	25
S_2W	435	0.0008	64	81
$Ca_2Ge_2Mn_2$	483	0.0008	65	48
MoS_2	435	0.0009	64	81
AsKSn	255	0.0009	49	36
Ba_2N	543	0.0009	100	81
BN	347	0.0009	49	100
Cu_4Te_2	429	0.0009	59	42
CNRb	348	0.0009	78	38
$PtTe_2$	435	0.0009	81	64
$CrSe_2$	492	0.0009	73	91
Ba_2Pt	390	0.0009	81	49
Ag_2Te_2	635	0.0009	105	80

Table showing the first 50 lattice matching combinations with the smallest strain needed to build the supercell.

$MnCl_2$ (C2/m)

Structural and electronic properties

**Formula**	$MnCl_2$
**Spacegroup**	C2/m
**Prototype**	CdI2
**Parent 3D**	$MnCl_2$
**Source DB**	COD
**DB ID**	9009130
**DF2-C09 Binding energy [meV/ Å^2]**	14.84
**RVV10 Binding energy [meV/ Å^2]**	21.51
**Band gap (PBE) [eV]**	2.06

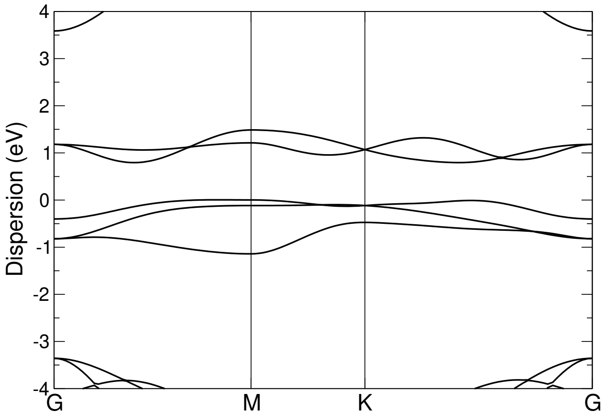

Band structure: Electronic band structure of $MnCl_2$ (C2/m) in a representative window around the Fermi energy and along a high-symmetry path.

Optimized crystal structure

Structural parameters: relaxed cell (top) and atomic positions (bottom) of $MnCl_2$ (C2/m) in Cartesian coordinates.

	x [Å]	y [Å]	z [Å]
$\mathbf{a_1}$	3.36171469	0.00000000	0.00000000
$\mathbf{a_2}$	−1.68085734	2.91133032	0.00000000
$\mathbf{a_3}$	0.00000000	0.00000000	22.63117628

	x [Å]	y [Å]	z [Å]
Cl	1.68085734	0.97044344	10.00616581
Mn	0.00000000	1.94088688	11.31558814
Cl	0.00000000	0.00000000	12.62501047

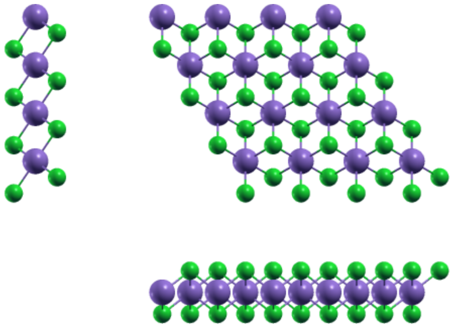

Orthographic projections: views of $MnCl_2$ (C2/m) as seen from the x axis (left), the y axis (bottom) and the z axis (center).

Lattice matching - minimal size

Formula	N° atoms	strain	cell size 1	cell size 2
Tl	4	0.1455	1	1
Tl	4	0.0057	1	1
Sn	4	0.1215	1	1
In	4	0.1241	1	1
Sm	4	4.8721	1	1
AsSb	5	0.496	1	1
Mg_2	5	0.132	1	1
$IrTe_2$	6	2.9628	1	1
$AgTe_2$	6	0.1483	1	1
$MoSe_2$	6	0.0064	1	1
S_2Ta	6	0.0021	1	1
Br_2Zn	6	0.4695	1	1
$AsSn_2$	6	0.4523	1	1
$SiTe_2$	6	0.48	1	1
NSr_2	6	0.5003	1	1
FeI_2	6	2.9095	1	1
I_2Ni	6	2.9261	1	1
S_2Ti	6	0.0071	1	1
Mg_3	6	0.1403	1	1
Te_2Ti	6	0.4702	1	1
NbS_2	6	0.0016	1	1
$RhTe_2$	6	0.4592	1	1
N_2W	6	0.2662	1	1
Cl_2Co	6	0.0068	1	1
NbS_2	6	0.0027	1	1
Cl_2Fe	6	0.006	1	1
S_2Ta	6	0.0035	1	1
Se_2V	6	0.0048	1	1
$NiTe_2$	6	0.4785	1	1
Cl_2Cu	6	0.1154	1	1
I_2V	6	0.4835	1	1
Se_2Zr	6	0.4811	1	1
$PtSe_2$	6	0.4539	1	1
CdO_2	6	0.0066	1	1
CoI_2	6	0.4971	1	1
O_2Zn	6	0.2712	1	1
$TaTe_2$	6	0.4509	1	1
Cl_2Zr	6	0.0064	1	1
$FeSe_2$	6	0.118	1	1
Br_2Mg	6	2.9078	1	1
I_2Ti	6	0.498	1	1
F_2Na	6	0.4745	1	1
N_2Re	6	1.5224	1	1
Se_2Sn	6	0.4997	1	1
$HfSe_2$	6	0.4703	1	1
Se_2W	6	0.0062	1	1
Cu_2I_2	7	0.3272	1	1
CdClHO	7	0.4495	1	1
Br_2Pr_2	7	0.4725	1	1
$HNiO_2$	7	0.2558	1	1

Table showing the first 50 lattice matching combinations with the smallest number of atoms in the supercell.

Lattice matching - minimal strain

Formula	N° atoms	strain	cell size 1	cell size 2
I_2V	390	0.0001	73	57
Sm	228	0.0001	49	81
IO_3Tl	17	0.0001	4	1
$PtTe_2$	183	0.0002	36	25
AsSb	290	0.0002	64	49
Cu_4Te_2	405	0.0002	61	37
$Cl_2O_2Tm_2$	627	0.0002	81	64
Cl_2Y_2	565	0.0002	91	73
$AuTe_2$	183	0.0002	36	25
$Cl_2N_2Zr_2$	693	0.0002	99	66
FeH_2O_2	743	0.0003	81	100
$HNiO_2$	463	0.0003	57	73
CBr_2Y_2	638	0.0003	91	73
Cl_2Gd_2	291	0.0003	49	36
$I_2N_2Zr_2$	786	0.0003	100	81
CaClHO	499	0.0003	81	64
Bi_2Te_2	496	0.0003	100	49
As_2Co_2	387	0.0003	65	48
Sb_2Te_3	368	0.0004	61	37
FeI_2	300	0.0004	57	43
$Cl_2Ho_2O_2$	561	0.0005	73	57
Cl_2NSc_2	8	0.0005	1	1
AsKSn	123	0.0005	25	16
PTe_2Zr_2	437	0.0005	64	49
Br_2Mg	300	0.0005	57	43
CaI_2	75	0.0006	16	9
S_2Zn_2	291	0.0006	49	36
I_2Tm	75	0.0006	16	9
F_2Na	435	0.0006	81	64
CoI_2	339	0.0006	64	49
$Ca_2Cl_2F_2$	840	0.0007	118	81
$Cl_2O_2Yb_2$	627	0.0007	81	64
$GeTe_2$	300	0.0007	57	43
$Ga_2I_2Y_2$	171	0.0007	25	16
CrI_2	300	0.0008	57	43
Sm	172	0.0008	37	61
$RhTe_2$	543	0.0008	100	81
$Br_2N_2Zr_2$	477	0.0008	69	45
Te_4TiZr	600	0.0008	114	43
Br_2Er_2	208	0.0008	36	25
$F_2Se_2Yb_2$	363	0.0008	49	36
Li_2Tl_2	43	0.0008	9	4
Se_2Zr	390	0.0008	73	57
GeI_2La_2	93	0.0009	16	9
NS_2Ta	583	0.0009	73	91
BiBrTe	390	0.0009	81	49
CCl_2Gd_2	638	0.0009	91	73
I_2Ni	300	0.0009	57	43
$AgClO_2$	519	0.0009	89	63
Ga_2Se_2	565	0.0009	91	73

Table showing the first 50 lattice matching combinations with the smallest strain needed to build the supercell.

MnI_2 (C2/m)

Structural and electronic properties

**Formula**	MnI_2
**Spacegroup**	C2/m
**Prototype**	CdI2
**Parent 3D**	MnI_2
**Source DB**	COD
**DB ID**	9009110
**DF2-C09 Binding energy [meV/ Å^2]**	17.03
**RVV10 Binding energy [meV/ Å^2]**	23.29
**Band gap (PBE) [eV]**	1.4

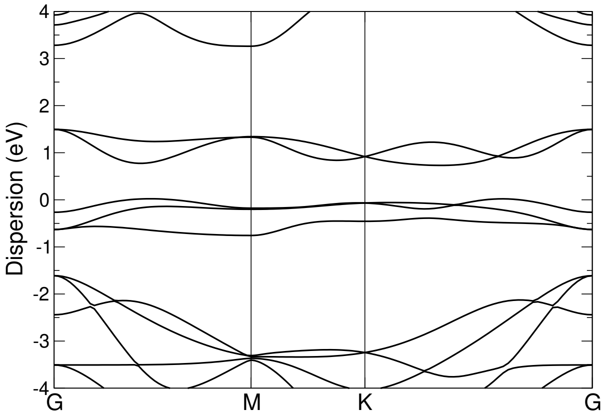

Band structure: Electronic band structure of MnI_2 (C2/m) in a representative window around the Fermi energy and along a high-symmetry path.

Optimized crystal structure

Structural parameters: relaxed cell (top) and atomic positions (bottom) of MnI_2 (C2/m) in Cartesian coordinates.

	x [Å]	y [Å]	z [Å]
$\mathbf{a_1}$	3.88550918	0.00000000	0.00000000
$\mathbf{a_2}$	−1.94275459	3.36494965	0.00000000
$\mathbf{a_3}$	0.00000000	0.00000000	22.93641140

		x [Å]	y [Å]	z [Å]
●	I	0.00000000	2.24329977	10.02535583
●	Mn	0.00000000	0.00000000	11.46820570
●	I	1.94275459	1.12164988	12.91105557

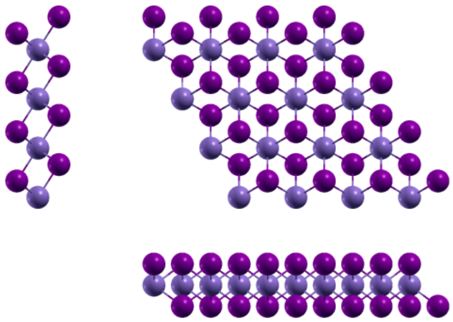

Orthographic projections: views of MnI_2 (C2/m) as seen from the x axis (left), the y axis (bottom) and the z axis (center).

Lattice matching - minimal size

Formula	N° atoms	strain	cell size 1	cell size 2
Tl	4	1.5374	1	1
HgO	5	0.1109	1	1
AsSb	5	0.0051	1	1
Bi_2	5	0.4558	1	1
GeTe	5	0.001	1	1
S_2	5	0.0025	1	1
CaCl	5	0.1391	1	1
$IrTe_2$	6	0.0019	1	1
$CdCl_2$	6	0.0001	1	1
CdI_2	6	0.4501	1	1
$AgTe_2$	6	0.4234	1	1
$MoSe_2$	6	1.532	1	1
$ReSe_2$	6	0.2598	1	1
S_2Ta	6	1.5942	1	1
Br_2Ca	6	0.4531	1	1
CaI_2	6	2.934	1	1
$InSe_2$	6	0.0003	1	1
$GeTe_2$	6	0.0011	1	1
NSr_2	6	0.0035	1	1
Ca_2Si	6	3.1921	1	1
I_2Yb	6	2.901	1	1
PbS_2	6	0.0076	1	1
BiClTe	6	0.451	1	1
LiO_2	6	0.067	1	1
Cl_2Zn	6	0.1523	1	1
FeI_2	6	0.0022	1	1
I_2Ni	6	0.0009	1	1
S_2Ti	6	1.6313	1	1
NbS_2	6	1.5908	1	1
CrI_2	6	0.0026	1	1
BiBrTe	6	0.4647	1	1
Bi_2Pd	6	0.1144	1	1
N_2W	6	4.8666	1	1
Cl_2Ni	6	0.261	1	1
Cl_2Co	6	1.6287	1	1
$CrTe_2$	6	0.2698	1	1
Br_2V	6	0.2587	1	1
ClNZr	6	0.2558	1	1
Cl_2Fe	6	0.2501	1	1
S_2Ta	6	1.5531	1	1
Se_2V	6	1.5435	1	1
Se_2Ti	6	0.2753	1	1
Br_2Ti	6	0.2689	1	1
$AsSe_2$	6	0.2631	1	1
I_2Tm	6	2.9186	1	1
BiITe	6	0.4814	1	1
BrNZr	6	0.265	1	1
$NbSe_2$	6	0.2616	1	1
CoI_2	6	0.0046	1	1
GeS_2	6	0.1298	1	1

Table showing the first 50 lattice matching combinations with the smallest number of atoms in the supercell.

Lattice matching - minimal strain

Formula	N° atoms	strain	cell size 1	cell size 2
Br_2Hf_2	583	0.0	73	91
GeI_2La_2	386	0.0001	57	43
$Cl_2Er_2H_2$	9	0.0001	1	1
$CdCl_2$	6	0.0001	1	1
Cu_4Te_2	786	0.0001	100	81
Se_2Ta	543	0.0002	81	100
Cu_2Sr_2	565	0.0002	91	73
FeH_2O_2	416	0.0002	37	61
N_3W_2	107	0.0002	9	16
$F_2I_2Pb_2$	483	0.0002	65	48
NbS_2	300	0.0002	43	57
ClNZr	390	0.0002	57	73
Ba_2Cd	339	0.0002	65	48
S_2Ta	300	0.0003	43	57
$InSe_2$	6	0.0003	1	1
$Br_2Cr_2O_2$	675	0.0003	73	76
$LiNbS_2$	357	0.0003	43	57
Ni_2Te_2	7	0.0003	1	1
I_2Tm	300	0.0003	57	43
$ReSe_2$	435	0.0004	64	81
$Cl_2H_2Sc_2$	843	0.0004	81	100
N_2W	390	0.0004	49	81
Sb_2Te_2	208	0.0004	36	25
Sb_2Te_3	705	0.0004	100	81
Br_2V	435	0.0005	64	81
BrNZr	492	0.0005	73	91
$Gd_2I_2S_2$	363	0.0005	49	36
Se_2V	255	0.0005	36	49
$F_2Se_2Y_2$	171	0.0005	25	16
CNNa	411	0.0005	77	60
H_2Si_2	7	0.0006	1	1
$NbSe_2$	492	0.0006	73	91
$Ge_2I_2La_2$	486	0.0006	64	49
Cl_2Fe	339	0.0007	49	64
Cl_2Cu	375	0.0007	61	64
BiITe	390	0.0007	73	57
Br_2Ca_3Si	363	0.0007	49	36
Bi_2Te_3	504	0.0008	73	57
$Br_2H_2Zr_2$	609	0.0008	57	73
BiBrTe	492	0.0009	91	73
S_2Ta	255	0.0009	36	49
$AsSe_2$	492	0.0009	73	91
Ca_2Si	183	0.0009	36	25
$Ce_2I_2S_2$	258	0.0009	36	25
LiO	147	0.0009	25	36
ReS_2	123	0.0009	16	25
As_2O_3	272	0.0009	49	25
Br_2Ti	543	0.0009	81	100
I_2Ni	6	0.0009	1	1
GeTe	5	0.001	1	1

Table showing the first 50 lattice matching combinations with the smallest strain needed to build the supercell.

MnO_2 (P-3m1)

Structural and electronic properties

**Formula**	MnO_2
**Spacegroup**	P-3m1
**Prototype**	CdI2
**Parent 3D**	MnO_2
**Source DB**	ICSD
**DB ID**	53991
**DF2-C09 Binding energy [meV/ Å^2]**	18.2
**RVV10 Binding energy [meV/ Å^2]**	N/A
**Band gap (PBE) [eV]**	0.0

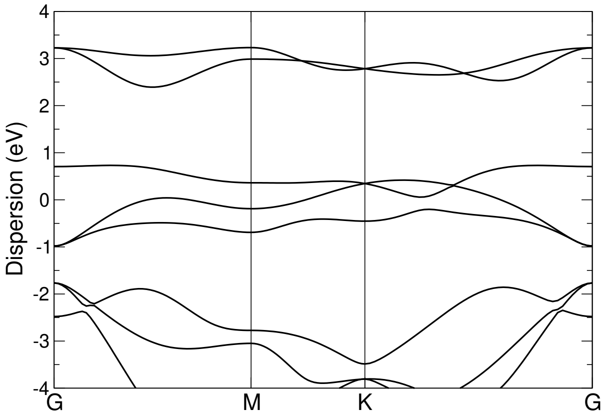

Band structure: Electronic band structure of MnO_2 (P-3m1) in a representative window around the Fermi energy and along a high-symmetry path.

Optimized crystal structure

Structural parameters: relaxed cell (top) and atomic positions (bottom) of MnO_2 (P-3m1) in Cartesian coordinates.

	x [Å]	y [Å]	z [Å]
$\mathbf{a_1}$	1.38079096	−2.39158518	0.00000000
$\mathbf{a_2}$	1.38079096	2.39158518	0.00000000
$\mathbf{a_3}$	0.00000000	0.00000000	16.05318921

		x [Å]	y [Å]	z [Å]
•	O	0.00000000	0.79719430	1.01057489
•	Mn	-1.38079096	0.00000000	0.00000000
•	O	0.00000000	-0.79719430	-1.01057489

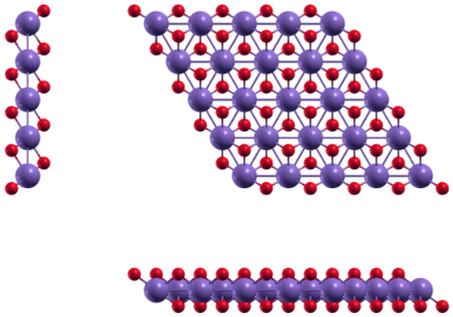

Orthographic projections: views of MnO_2 (P-3m1) as seen from the x axis (left), the y axis (bottom) and the z axis (center).

Lattice matching - minimal size

Formula	N° atoms	strain	cell size 1	cell size 2
Nd	4	0.0092	1	1
In	4	0.4971	1	1
BN	5	0.2742	1	1
C_2	5	0.2623	1	1
CrS_2	6	0.4592	1	1
FeO_2	6	0.0095	1	1
ReS_2	6	0.4743	1	1
CrO_2	6	0.0022	1	1
Cl_2Cu	6	0.6881	1	1
O_2Zn	6	0.451	1	1
O_2Pt	6	0.493	1	1
CoH_2O_2	8	0.4717	1	1
GeTe	8	0.2203	2	1
S_2	8	0.2223	2	1
Ga_2S_3	8	13.6076	1	1
$IrTe_2$	9	0.2215	2	1
$CdCl_2$	9	0.219	2	1
$InSe_2$	9	0.2194	2	1
I_2Mn	9	0.2191	2	1
Ca_2Si	9	6.0454	1	2
H_2Si_2	10	0.2198	2	1
K	10	0.1296	3	1
Cl_2Gd_2	10	0.2253	2	1
Cu_2O_2	10	0.8526	2	1
$AgCuTe_2$	10	0.5496	2	1
S_2Zn_2	10	0.2241	2	1
P_2Sn_2	10	0.2236	2	1
Ga_2Se_2	10	0.2217	2	1
Ni_2Te_2	10	0.2187	2	1
Ho_2S_2	10	0.3206	2	1
AgTl	11	0.1137	3	1
$CuGeO_3$	11	0.8685	2	1
In_2S_3	11	0.2206	2	1
Ni_2SbTe_2	11	0.2226	2	1
HgI_2	12	0.1433	3	1
Bi_2Pd	12	0.1567	3	1
Ba_2Hg	12	0.1203	3	1
CKN	12	0.1717	3	1
I_2Nd	12	0.4221	3	1
Cu_2O_4	12	0.5173	2	1
O_2Zn	12	0.1978	2	2
I_2La	12	0.4328	3	1
$F_2Se_2Yb_2$	12	0.2238	2	1
$Cl_2Er_2H_2$	12	0.2193	2	1
Ir_2P_2	13	0.4217	3	1
Bi_2Mn_2	13	0.1173	3	1
Ag_2Br_2	13	0.434	3	1
O_2Sn_2	13	0.4354	3	1
S_2Sn_2	13	0.1206	3	1
Au_2Br_2	13	0.1186	3	1

Table showing the first 50 lattice matching combinations with the smallest number of atoms in the supercell.

Lattice matching - minimal strain

Formula	N° atoms	strain	cell size 1	cell size 2
Br_2Mg	222	0.0	49	25
AsI_2La_2	317	0.0	64	25
Cd_2I_3	317	0.0	64	25
Cl_2Tb_2	393	0.0	79	39
Ga_2S_3	488	0.0001	81	49
$BiTe_2$	39	0.0001	9	4
NS_2Zr	439	0.0001	81	49
FeI_2	222	0.0001	49	25
$CrSe_2$	255	0.0002	49	36
Gd	204	0.0002	50	54
Br_2V	123	0.0002	25	16
CrI_2	222	0.0002	49	25
Sb_2Se_2Te	47	0.0003	9	4
$MoSe_2$	183	0.0003	36	25
Na	292	0.0003	81	49
Na	220	0.0003	61	37
S_2V	300	0.0003	57	43
Se_2Yb	39	0.0003	9	4
$Cl_2Fe_2O_2$	714	0.0004	112	63
Se_2W	183	0.0004	36	25
$F_2Se_2Tm_2$	594	0.0005	100	49
H_2MgO_2	386	0.0005	57	43
$I_2Nd_2S_2$	129	0.0005	25	9
I_2Y_2	393	0.0006	79	39
GeI_2	39	0.0006	9	4
$LiMnSe_2$	496	0.0006	100	49
PSn_2	75	0.0006	16	9
$ReSe_2$	123	0.0006	25	16
Fe_2S_2	197	0.0006	39	20
In	241	0.0006	64	49
ReS_2	435	0.0007	81	64
Cl_2Gd_2	393	0.0007	79	39
Ga_2S_3	368	0.0007	61	37
Sb_2Se_2Te	47	0.0007	9	4
PtS_2	390	0.0007	81	49
NS_2Zr	331	0.0007	61	37
Ba_2Ni_3	47	0.0008	9	4
CrS_2	543	0.0008	100	81
Ga_2Te_2	43	0.0008	9	4
$Au_2K_2Se_2$	264	0.0008	70	9
Ge_2Se_2	820	0.0009	180	70
$I_2Pr_2Si_2$	51	0.0009	9	4
O_2Pt	339	0.0009	64	49
$C_2I_2Y_2$	225	0.0009	39	18
Cl_2Zr_2	139	0.001	25	16
Se_4TiZr	150	0.001	32	9
Mo_2Te_4	192	0.001	40	12
Tl	133	0.001	36	25
$Ce_2I_2Si_2$	51	0.0011	9	4
$AsCuLi_2$	43	0.0011	9	4

Table showing the first 50 lattice matching combinations with the smallest strain needed to build the supercell.

MoS_2 (P-3m1)

Structural and electronic properties

**Formula**	MoS_2
**Spacegroup**	P-3m1
**Prototype**	CdI2
**Parent 3D**	MoS_2
**Source DB**	ICSD
**DB ID**	26622
**DF2-C09 Binding energy [meV/ Å^2]**	28.65
**RVV10 Binding energy [meV/ Å^2]**	33.51
**Band gap (PBE) [eV]**	N/A

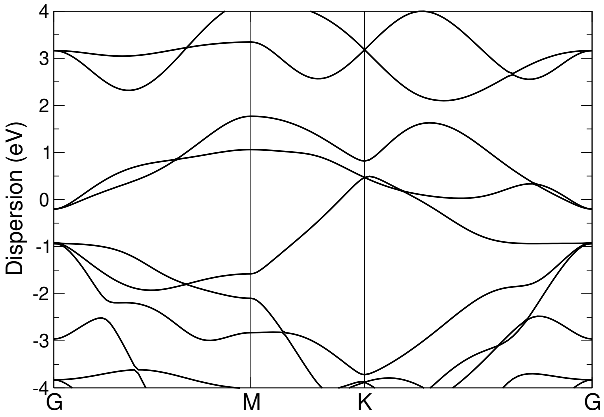

Band structure: Electronic band structure of MoS_2 (P-3m1) in a representative window around the Fermi energy and along a high-symmetry path.

Optimized crystal structure

Structural parameters: relaxed cell (top) and atomic positions (bottom) of MoS_2 (P-3m1) in Cartesian coordinates.

	x [Å]	y [Å]	z [Å]
$\mathbf{a_1}$	3.18673750	0.00000000	0.00000000
$\mathbf{a_2}$	−1.59336875	2.75979563	0.00000000
$\mathbf{a_3}$	0.00000000	0.00000000	23.18923690

	x [Å]	y [Å]	z [Å]
Mo	0.00000000	1.83986375	11.59461514
S	0.00000000	0.00000000	13.18330340
S	1.59336875	0.91993188	10.00593681

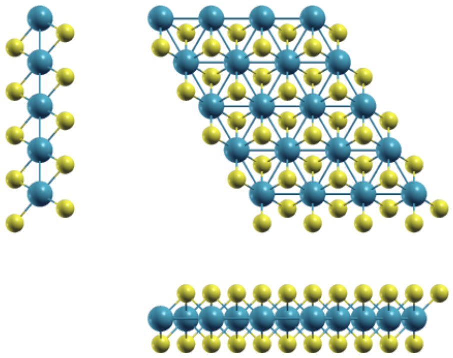

Orthographic projections: views of MoS_2 (P-3m1) as seen from the x axis (left), the y axis (bottom) and the z axis (center).

Lattice matching - minimal size

Formula	N° atoms	strain	cell size 1	cell size 2
Sn	4	0.1393	1	1
Na	4	0.4637	1	1
In	4	0.1431	1	1
In	4	0.0039	1	1
As_2	5	0.484	1	1
LiO	5	0.0067	1	1
Mg_2	5	0.154	1	1
Sb_2	5	13.6492	1	1
Cl_2Zn	6	0.4813	1	1
S_2V	6	0.0007	1	1
$MoTe_2$	6	0.4694	1	1
PSn_2	6	2.9285	1	1
HfS_2	6	2.8488	1	1
FeO_2	6	0.2556	1	1
$AsSn_2$	6	2.9783	1	1
Te_2V	6	0.4743	1	1
I_2Pr	6	0.3341	1	1
S_2Zr	6	2.9185	1	1
NiO_2	6	0.2574	1	1
Br_2Co	6	0.4829	1	1
Ca_2N	6	0.4854	1	1
Te_2Zn	6	0.4689	1	1
$RhTe_2$	6	3.0159	1	1
S_2W	6	0.0001	1	1
Br_2Mn	6	0.4782	1	1
$CrTe_2$	6	0.4504	1	1
PtS_2	6	0.4663	1	1
$CoTe_2$	6	2.853	1	1
CdClO	6	0.473	1	1
Se_2Ti	6	0.4597	1	1
Br_2Ti	6	0.4489	1	1
Te_2W	6	0.4698	1	1
I_2Nd	6	0.3362	1	1
S_2Sn	6	2.922	1	1
Cl_2V	6	0.0041	1	1
$PtSe_2$	6	2.9869	1	1
OTl_2	6	0.4735	1	1
Br_2Fe	6	0.4831	1	1
Br_2Cr	6	0.4497	1	1
Br_2Ni	6	0.4964	1	1
CeI_2	6	0.3325	1	1
$FeSe_2$	6	0.134	1	1
$NbTe_2$	6	2.9158	1	1
MoS_2	6	0.0002	1	1
Cl_2Mg	6	0.4966	1	1
$CrSe_2$	6	0.0029	1	1
$CrSe_2$	6	0.0054	1	1
O_2Pt	6	0.0054	1	1
N_2Re	6	0.2658	1	1
F_2Zn	6	0.3258	1	1

Table showing the first 50 lattice matching combinations with the smallest number of atoms in the supercell.

Lattice matching - minimal strain

Formula	N° atoms	strain	cell size 1	cell size 2
Ba_2N	123	0.0	25	16
I_2La_2Te	545	0.0	100	49
Br_2Fe	390	0.0001	73	57
S_2W	6	0.0001	1	1
Ba_2Cu_2	247	0.0001	49	25
Ga_2S_2	388	0.0001	64	49
FeO_2	390	0.0001	57	73
$NbTe_2$	300	0.0001	57	43
Br_2Co	390	0.0001	73	57
$AsLi_3$	84	0.0001	16	9
$Br_2In_2O_2$	312	0.0001	48	28
CCl_2Lu_2	504	0.0002	73	57
PbS_2	183	0.0002	36	25
$Gd_2I_2S_2$	471	0.0002	79	39
MoS_2	6	0.0002	1	1
Br_2Ca_3Si	594	0.0002	100	49
PTe_2Ti_2	437	0.0002	64	49
I_2Yb	222	0.0002	49	25
PtS_2	492	0.0002	91	73
Cu_3Se_3	171	0.0003	25	16
As_2	333	0.0003	73	57
S_2Zr	300	0.0003	57	43
Br_2Ni	339	0.0004	64	49
$F_2Lu_2Se_2$	405	0.0004	61	37
Br_2Y_2	208	0.0004	36	25
InSe	66	0.0004	16	9
Cl_2Mg	339	0.0004	64	49
As_2Li_2Pr	93	0.0005	16	9
Br_2Ca_3Si	471	0.0005	79	39
$I_2La_2Si_2$	102	0.0005	16	9
Bi_2STe_2	93	0.0005	16	9
$HfLiS_2$	565	0.0005	91	73
Br_2La_2	439	0.0005	81	49
$Ba_2H_2I_2$	714	0.0006	130	54
S_2Sn	300	0.0006	57	43
Se_2Ti	543	0.0006	100	81
CdClHO	291	0.0007	49	36
Te_2V	435	0.0007	81	64
S_2V	6	0.0007	1	1
$PtSe_2$	255	0.0007	49	36
$Cl_2H_2Lu_2$	486	0.0007	64	49
Cl_2Zn	390	0.0007	73	57
Se_4TiZr	600	0.0007	114	43
Ga_2Se_2	208	0.0008	36	25
$Cl_2N_2Zr_2$	396	0.0008	60	36
Sb_2	341	0.0008	81	49
Te_2Zn	492	0.0008	91	73
Ca_2N	390	0.0008	73	57
$C_2I_2Y_2$	435	0.0008	65	40
CoO_2	390	0.0008	57	73

Table showing the first 50 lattice matching combinations with the smallest strain needed to build the supercell.

MoS_2 (P-6m2)

Structural and electronic properties

**Formula**	MoS_2
**Spacegroup**	P-6m2
**Prototype**	MoS2
**Parent 3D**	Mo_2S_4
**Source DB**	ICSD
**DB ID**	644259
**DF2-C09 Binding energy [meV/ Å^2]**	21.56
**RVV10 Binding energy [meV/ Å^2]**	28.76
**Band gap (PBE) [eV]**	1.62

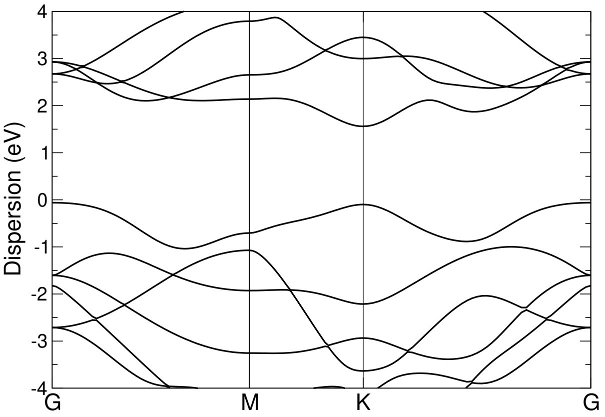

Band structure: Electronic band structure of MoS_2 (P-6m2) in a representative window around the Fermi energy and along a high-symmetry path.

Optimized crystal structure

Structural parameters: relaxed cell (top) and atomic positions (bottom) of MoS_2 (P-6m2) in Cartesian coordinates.

		x [Å]	y [Å]	z [Å]
$\mathbf{a_1}$		3.18817952	0.00000000	0.00000000
$\mathbf{a_2}$		−1.59408976	2.76104446	0.00000000
$\mathbf{a_3}$		0.00000000	0.00000000	23.15470209
		x [Å]	y [Å]	z [Å]
●	Mo	0.00000000	1.84069630	11.57735104
●	S	1.59408976	0.92034815	10.01882599
●	S	1.59408976	0.92034815	13.13587609

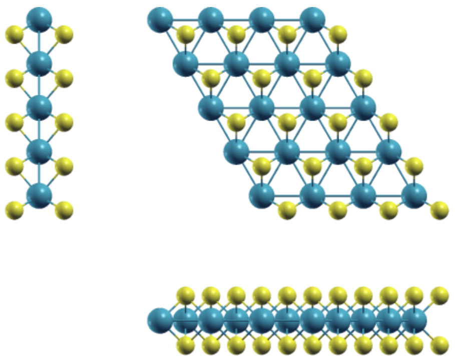

Orthographic projections: views of MoS_2 (P-6m2) as seen from the x axis (left), the y axis (bottom) and the z axis (center).

Lattice matching - minimal size

Formula	N° atoms	strain	cell size 1	cell size 2
Sn	4	0.1391	1	1
Na	4	0.4631	1	1
In	4	0.1429	1	1
In	4	0.0041	1	1
As_2	5	0.4834	1	1
LiO	5	0.0065	1	1
Mg_2	5	0.1538	1	1
Sb_2	5	13.6367	1	1
Cl_2Zn	6	0.4807	1	1
S_2V	6	0.0009	1	1
MoS_2	6	0.0002	1	1
$MoTe_2$	6	0.4688	1	1
PSn_2	6	2.9257	1	1
HfS_2	6	2.8461	1	1
FeO_2	6	0.2553	1	1
$AsSn_2$	6	2.9755	1	1
Te_2V	6	0.4737	1	1
I_2Pr	6	0.3337	1	1
S_2Zr	6	2.9158	1	1
NiO_2	6	0.2571	1	1
Br_2Co	6	0.4823	1	1
Ca_2N	6	0.4848	1	1
Te_2Zn	6	0.4684	1	1
$RhTe_2$	6	3.013	1	1
S_2W	6	0.0001	1	1
Br_2Mn	6	0.4776	1	1
$CrTe_2$	6	0.4498	1	1
PtS_2	6	0.4657	1	1
$CoTe_2$	6	2.8502	1	1
CdClO	6	0.4725	1	1
Se_2Ti	6	0.4591	1	1
Br_2Ti	6	0.4484	1	1
Te_2W	6	0.4692	1	1
I_2Nd	6	0.3357	1	1
S_2Sn	6	2.9192	1	1
Cl_2V	6	0.0039	1	1
$PtSe_2$	6	2.984	1	1
OTl_2	6	0.473	1	1
Br_2Fe	6	0.4825	1	1
Br_2Cr	6	0.4492	1	1
Br_2Ni	6	0.4959	1	1
CeI_2	6	0.3321	1	1
$FeSe_2$	6	0.1339	1	1
$NbTe_2$	6	2.913	1	1
Cl_2Mg	6	0.496	1	1
$CrSe_2$	6	0.0027	1	1
$CrSe_2$	6	0.0052	1	1
O_2Pt	6	0.0056	1	1
N_2Re	6	0.2655	1	1
F_2Zn	6	0.3254	1	1

Table showing the first 50 lattice matching combinations with the smallest number of atoms in the supercell.

Lattice matching - minimal strain

Formula	N° atoms	strain	cell size 1	cell size 2
$Gd_2I_2S_2$	471	0.0	79	39
PTe_2Ti_2	437	0.0	64	49
I_2Yb	222	0.0	49	25
CCl_2Lu_2	504	0.0	73	57
As_2	333	0.0001	73	57
S_2Zr	300	0.0001	57	43
$NbTe_2$	300	0.0001	57	43
FeO_2	390	0.0001	57	73
Ba_2Cu_2	247	0.0001	49	25
S_2W	6	0.0001	1	1
Br_2Ni	339	0.0002	64	49
Br_2Ca_3Si	594	0.0002	100	49
I_2La_2Te	545	0.0002	100	49
MoS_2	6	0.0002	1	1
Cl_2Mg	339	0.0002	64	49
Ba_2N	123	0.0002	25	16
Br_2Ca_3Si	471	0.0003	79	39
Br_2Fe	390	0.0003	73	57
Bi_2STe_2	93	0.0003	16	9
Ga_2S_2	388	0.0003	64	49
$Br_2In_2O_2$	312	0.0003	48	28
$HfLiS_2$	565	0.0003	91	73
Br_2La_2	439	0.0003	81	49
Br_2Co	390	0.0003	73	57
$AsLi_3$	84	0.0003	16	9
S_2Sn	300	0.0004	57	43
PbS_2	183	0.0004	36	25
CdClHO	291	0.0005	49	36
PtS_2	492	0.0005	91	73
Cu_3Se_3	171	0.0005	25	16
$Cl_2H_2Lu_2$	486	0.0005	64	49
Se_4TiZr	600	0.0005	114	43
Sb_2	341	0.0006	81	49
$F_2Lu_2Se_2$	405	0.0006	61	37
Te_2Zn	492	0.0006	91	73
$Cl_2N_2Zr_2$	396	0.0006	60	36
Ca_2N	390	0.0006	73	57
Br_2Y_2	208	0.0006	36	25
CoO_2	390	0.0006	57	73
Br_2Mn	435	0.0006	81	64
InSe	66	0.0006	16	9
As_2Li_2Pr	93	0.0007	16	9
$I_2La_2Si_2$	102	0.0007	16	9
$C_2I_2Y_2$	435	0.0007	65	40
$Ba_2H_2I_2$	714	0.0008	130	54
Fe_2S_2	678	0.0008	118	81
$MoTe_2$	492	0.0008	91	73
Na	381	0.0008	100	81
Se_2Ti	543	0.0008	100	81
N_2Re	492	0.0008	73	91

Table showing the first 50 lattice matching combinations with the smallest strain needed to build the supercell.

$MoSe_2$ (P-6m2)

Structural and electronic properties

**Formula**	$MoSe_2$
**Spacegroup**	P-6m2
**Prototype**	MoS2
**Parent 3D**	Mo_2Se_4
**Source DB**	ICSD
**DB ID**	644346
**DF2-C09 Binding energy [meV/ Å^2]**	20.45
**RVV10 Binding energy [meV/ Å^2]**	28.43
**Band gap (PBE) [eV]**	1.48

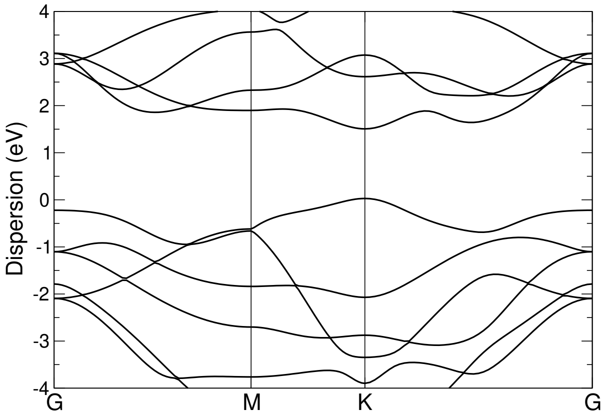

Band structure: Electronic band structure of $MoSe_2$ (P-6m2) in a representative window around the Fermi energy and along a high-symmetry path.

Optimized crystal structure

Structural parameters: relaxed cell (top) and atomic positions (bottom) of $MoSe_2$ (P-6m2) in Cartesian coordinates.

	x [Å]	y [Å]	z [Å]
$\mathbf{a_1}$	3.31579398	0.00000000	0.00000000
$\mathbf{a_2}$	−1.65789699	2.87156182	0.00000000
$\mathbf{a_3}$	0.00000000	0.00000000	23.35864962

		x [Å]	y [Å]	z [Å]
●	Mo	-0.00000000	1.91437455	11.67966487
●	Se	1.65789699	2.87156182	13.35090103
●	Se	1.65789699	2.87156182	10.00842655

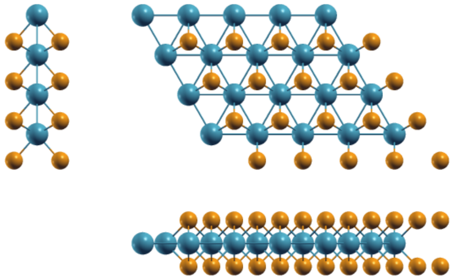

Orthographic projections: views of $MoSe_2$ (P-6m2) as seen from the x axis (left), the y axis (bottom) and the z axis (center).

Lattice matching - minimal size

Formula	N° atoms	strain	cell size 1	cell size 2
Tl	4	0.1516	1	1
Tl	4	0.0007	1	1
Sn	4	0.1254	1	1
In	4	0.1284	1	1
GeTe	5	3.0375	1	1
P_2	5	0.0045	1	1
PbTe	5	13.5901	1	1
Mg_2	5	0.1371	1	1
$CdCl_2$	6	3.0231	1	1
Cl_2Mn	6	0.0066	1	1
$AgTe_2$	6	0.1546	1	1
PSn_2	6	0.459	1	1
S_2Ta	6	0.0087	1	1
Br_2Zn	6	0.4862	1	1
$InSe_2$	6	3.0281	1	1
$AsSn_2$	6	0.4685	1	1
$SiTe_2$	6	0.497	1	1
I_2Mn	6	3.0247	1	1
S_2Zr	6	0.4571	1	1
NSr_2	6	2.978	1	1
NiO_2	6	1.5256	1	1
Cl_2Ti	6	0.0043	1	1
FeI_2	6	2.9951	1	1
I_2Ni	6	3.0121	1	1
Mg_3	6	0.146	1	1
Te_2Ti	6	0.4869	1	1
NbS_2	6	0.0082	1	1
$RhTe_2$	6	0.4756	1	1
N_2W	6	0.2757	1	1
NbS_2	6	0.0038	1	1
S_2Ta	6	0.003	1	1
Se_2V	6	0.0016	1	1
$NiTe_2$	6	0.4955	1	1
Cl_2Cu	6	0.1231	1	1
S_2Sn	6	0.4578	1	1
I_2V	6	0.5006	1	1
GeI_2	6	13.6038	1	1
Se_2Zr	6	0.4981	1	1
$PtSe_2$	6	0.4701	1	1
CoI_2	6	2.9623	1	1
$TaTe_2$	6	0.467	1	1
Br_2Ni	6	0.4487	1	1
$FeSe_2$	6	0.1215	1	1
Br_2Mg	6	2.9933	1	1
$NbTe_2$	6	0.4566	1	1
Cl_2Mg	6	0.4488	1	1
Se_2Sn	6	2.9748	1	1
CoO_2	6	1.5218	1	1
$HfSe_2$	6	0.487	1	1
Se_2W	6	0.0002	1	1

Table showing the first 50 lattice matching combinations with the smallest number of atoms in the supercell.

Lattice matching - minimal strain

Formula	N° atoms	strain	cell size 1	cell size 2
GeI_2	390	0.0	81	49
$NiTe_2$	339	0.0	64	49
Sb_2Se_2Te	155	0.0	25	16
$TaTe_2$	492	0.0	91	73
Ga_2Se_2	343	0.0	57	43
Cl_2Y_2	447	0.0001	73	57
FeI_2	255	0.0001	49	36
$RhTe_2$	435	0.0001	81	64
Se_2W	6	0.0002	1	1
As_2CeLi_2	488	0.0002	81	49
PbTe	341	0.0002	81	49
Cu_3Se_3	258	0.0002	36	25
Cd_2I_3	93	0.0003	16	9
Br_2Mg	255	0.0003	49	36
MnO_2	183	0.0003	25	36
I_2Pb	222	0.0003	49	25
AsI_2La_2	93	0.0003	16	9
$LiMnTe_2$	439	0.0003	81	49
H_2MnO_2	536	0.0003	57	73
$BiTe_2$	123	0.0004	25	16
$HNiO_2$	583	0.0004	73	91
PbTe	257	0.0004	61	37
Br_2Y_2	343	0.0004	57	43
As_2CeLi_2	368	0.0004	61	37
Bi_2STe_2	368	0.0004	61	37
CBr_2Y_2	504	0.0004	73	57
Sb_2Se_2Te	155	0.0004	25	16
$Cl_2O_2Y_2$	429	0.0005	57	43
Ba_2N	183	0.0005	36	25
$Hf_2I_2N_2$	711	0.0005	91	73
CrI_2	255	0.0005	49	36
CdClHO	565	0.0005	91	73
Te_2Zr	183	0.0005	36	25
$I_2Pr_2S_2$	297	0.0006	49	25
CaClHO	388	0.0006	64	49
$SiTe_2$	339	0.0006	64	49
Se_2Yb	123	0.0006	25	16
$AsSn_2$	492	0.0006	91	73
GeI_2	294	0.0006	61	37
PbS_2	300	0.0006	57	43
Bi_2STe_2	488	0.0006	81	49
S_2Sn_2	342	0.0007	66	36
$As_2Mg_2Na_2$	237	0.0007	39	20
$Cl_2O_2Tm_2$	486	0.0007	64	49
Tl	4	0.0007	1	1
$Ce_2I_2Si_2$	171	0.0008	25	16
N_4	204	0.0008	28	30
Ga_2Se_2	447	0.0008	73	57
Ge_2Se_2	299	0.0008	57	32
GeI_2	123	0.0009	25	16

Table showing the first 50 lattice matching combinations with the smallest strain needed to build the supercell.

$MoTe_2$ ($P2_1/m$)

Structural and electronic properties

**Formula**	$MoTe_2$
**Spacegroup**	$P2_1/m$
**Prototype**	WTe2
**Parent 3D**	Mo_4Te_8
**Source DB**	COD
**DB ID**	2310356
**DF2-C09 Binding energy [meV/ Å^2]**	24.52
**RVV10 Binding energy [meV/ Å^2]**	30.09
**Band gap (PBE) [eV]**	N/A

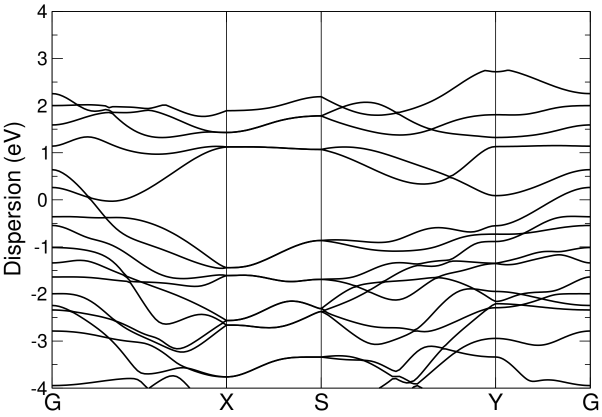

Band structure: Electronic band structure of $MoTe_2$ ($P2_1/m$) in a representative window around the Fermi energy and along a high-symmetry path.

Optimized crystal structure

Structural parameters: relaxed cell (top) and atomic positions (bottom) of $MoTe_2$ ($P2_1/m$) in Cartesian coordinates.

	x [Å]	y [Å]	z [Å]
$\mathbf{a_1}$	3.46243803	0.00000000	0.00000000
$\mathbf{a_2}$	0.00000000	6.37782584	0.00000000
$\mathbf{a_3}$	0.00000000	0.00000000	24.19479066

		x [Å]	y [Å]	z [Å]
●	Te	2.59682852	3.26052109	14.17931777
●	Te	0.86560951	2.25522269	10.01546730
●	Te	0.86560951	0.01242713	13.58753627
●	Te	2.59682852	5.50328147	10.60725761
●	Mo	2.59682852	1.60532603	12.00055619
●	Mo	0.86560951	3.91030803	12.19423683

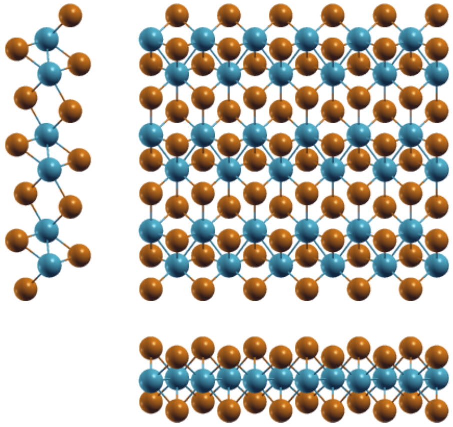

Orthographic projections: views of $MoTe_2$ ($P2_1/m$) as seen from the x axis (left), the y axis (bottom) and the z axis (center).

Lattice matching - minimal size

Formula	N° atoms	strain	cell size 1	cell size 2
Tl	8	0.0277	1	2
LiO	8	0.34	1	1
Cl_2V	9	0.3357	1	1
$CrSe_2$	9	0.3378	1	1
HgO	10	0.0212	1	2
Br_2Er_2	10	0.9852	1	1
AsSb	10	0.1791	1	2
GeTe	10	0.1866	1	2
Ag_2	10	1.0533	1	2
Br_2OV	10	0.2561	1	1
Bi_2Se_2	10	0.2074	1	1
S_2	10	0.1885	1	2
Au_2Se_2	10	0.0281	1	1
Br_2Ho_2	10	0.9762	1	1
Mg_2	10	0.1091	1	2
CaCl	10	0.3408	1	2
$CuGeO_3$	11	0.7764	1	1
$IrTe_2$	12	0.1877	1	2
$CdCl_2$	12	0.1852	1	2
$AgTe_2$	12	0.0258	1	2
PSn_2	12	0.0213	1	2
Ba_2Pt	12	1.0519	1	2
$ReSe_2$	12	0.0198	1	2
Cu_4Te_2	12	0.1283	1	1
HfS_2	12	0.0172	1	2
$InSe_2$	12	0.1857	1	2
$AsSn_2$	12	0.0245	1	2
$GeTe_2$	12	0.184	1	2
I_2Mn	12	0.1854	1	2
$CuTe_2$	12	0.017	1	2
S_2Zr	12	0.0208	1	2
NSr_2	12	0.181	1	2
PbS_2	12	0.176	1	2
$Br_2Cr_2S_2$	12	0.3604	1	1
Ca_2N	12	0.016	1	2
FeI_2	12	0.1826	1	2
I_2Ni	12	0.1842	1	2
S_2Ti	12	0.024	1	2
Mg_3	12	0.1137	1	2
$Br_2S_2Y_2$	12	0.2515	1	1
CrI_2	12	0.182	1	2
$RhTe_2$	12	0.027	1	2
Cl_2Ni	12	0.0193	1	2
$CrTe_2$	12	0.016	1	2
$Br_2Er_2Se_2$	12	0.6046	1	1
$CoTe_2$	12	0.0173	1	2
Br_2V	12	0.0204	1	2
ClNZr	12	0.0218	1	2
Cl_4Mg_2	12	0.5741	1	1
Te_4TiZr	12	0.1829	1	1

Table showing the first 50 lattice matching combinations with the smallest number of atoms in the supercell.

Lattice matching - minimal strain

Formula	N° atoms	strain	cell size 1	cell size 2
Ir_2P_2	824	0.0005	70	101
I_2Nd	723	0.0006	70	101
O_2Pt	411	0.0007	30	77
$I_2Nd_2S_2$	264	0.0007	20	24
$Ag_2K_2Se_2$	798	0.0008	63	70
AsI_2La_2	527	0.0009	42	55
Cd_2I_3	527	0.0009	42	55
I_2Pr	723	0.0009	70	101
$CuGeO_3$	461	0.0009	36	49
$Er_2F_2Se_2$	882	0.001	56	91
I_2Y_2	780	0.001	62	102
MnO_2	192	0.001	12	40
N_4	96	0.001	6	15
LiO	512	0.0011	47	115
$F_2Se_2Tm_2$	984	0.0011	62	102
F_4Pb	968	0.0011	83	94
$Cl_2Cr_2O_2$	414	0.0012	24	45
$CrSe_2$	627	0.0012	47	115
$I_2S_2Tm_2$	360	0.0012	30	30
$Er_2I_2S_2$	360	0.0013	30	30
I_3Sn	838	0.0013	97	64
$Br_2Ho_2O_2$	450	0.0014	30	45
Gd_2I_2	644	0.0014	54	80
P_2Rh_2	360	0.0014	30	45
In	257	0.0014	30	77
$Ca_2Mn_2Si_2$	450	0.0014	30	45
F_2Tl_2	360	0.0015	30	45
$F_2Se_2Tm_2$	966	0.0015	61	100
$Br_2Ca_2H_2$	450	0.0016	30	45
CeI_2	723	0.0016	70	101
K_2PtSe_2	960	0.0016	110	60
Br_2Gd_2Ge	724	0.0017	54	80
Br_2La_2	786	0.0017	65	99
Au_2Br_2	348	0.0017	32	39
Ag_2I_2	862	0.0017	83	91
C_4Ca_2	318	0.0017	23	30
CNRb	798	0.0017	90	86
$Ge_2Mn_2Sr_2$	576	0.0017	40	56
$Br_2Dy_2O_2$	450	0.0017	30	45
I_3Sn	390	0.0017	45	30
$Br_2O_2Y_2$	450	0.0017	30	45
$I_2Pr_2Si_2$	804	0.0018	54	80
$Ge_2I_2La_2$	744	0.0018	54	70
Ag_2	888	0.0018	106	126
C_2	144	0.0018	10	42
Ni_2Se_2	824	0.0018	70	101
I_2Y_2	766	0.0018	61	100
Cu_2S_2	360	0.0018	30	45
Sb_2	588	0.0018	65	99
Br_2Ca_3Si	606	0.0018	45	56

Table showing the first 50 lattice matching combinations with the smallest strain needed to build the supercell.

$MoTe_2$ (P-6m2)

Structural and electronic properties

**Formula**	$MoTe_2$
**Spacegroup**	P-6m2
**Prototype**	MoS2
**Parent 3D**	Mo_2Te_4
**Source DB**	COD
**DB ID**	9009147
**DF2-C09 Binding energy [meV/ Å^2]**	25.21
**RVV10 Binding energy [meV/ Å^2]**	30.39
**Band gap (PBE) [eV]**	1.07

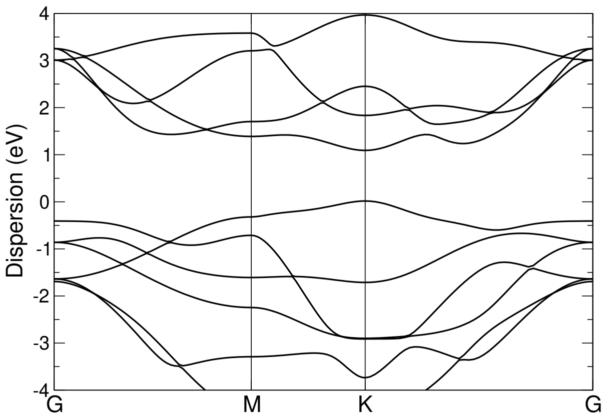

Band structure: Electronic band structure of $MoTe_2$ (P-6m2) in a representative window around the Fermi energy and along a high-symmetry path.

Optimized crystal structure

Structural parameters: relaxed cell (top) and atomic positions (bottom) of $MoTe_2$ (P-6m2) in Cartesian coordinates.

		x [Å]	y [Å]	z [Å]
$\mathbf{a_1}$		3.56539293	0.00000000	0.00000000
$\mathbf{a_2}$		−1.78269647	3.08772086	0.00000000
$\mathbf{a_3}$		0.00000000	0.00000000	23.64017947
		x [Å]	y [Å]	z [Å]
●	Te	1.78269647	1.02924029	10.00768461
●	Te	1.78269647	1.02924029	13.63265216
●	Mo	0.00000000	2.05848057	11.82017038

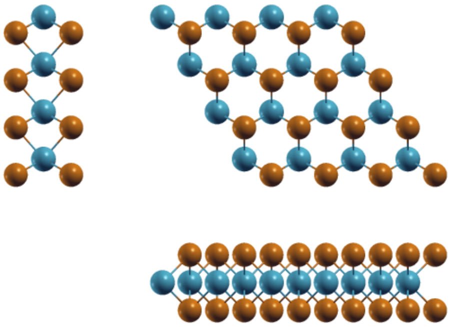

Orthographic projections: views of $MoTe_2$ (P-6m2) as seen from the x axis (left), the y axis (bottom) and the z axis (center).

Lattice matching - minimal size

Formula	N° atoms	strain	cell size 1	cell size 2
K	4	0.3222	1	1
Tl	4	0.1242	1	1
Na	4	0.0022	1	1
In	4	0.111	1	1
In	4	0.2575	1	1
Gd	4	0.1143	1	1
HgO	5	0.1325	1	1
As_2	5	0.0058	1	1
LiO	5	0.2725	1	1
Mg_2	5	0.1155	1	1
Sb_2	5	2.9128	1	1
CrS_2	6	1.555	1	1
Cl_2Zn	6	0.0047	1	1
I_2Mg	6	2.9875	1	1
S_2V	6	0.2619	1	1
MoS_2	6	0.2629	1	1
$AgTe_2$	6	0.1261	1	1
Br_2Ca	6	3.1939	1	1
HfS_2	6	0.0086	1	1
$HfTe_2$	6	0.4638	1	1
Te_2V	6	0.0019	1	1
$CuTe_2$	6	0.0082	1	1
Br_2La	6	2.992	1	1
Br_2Cu	6	1.0757	1	1
Br_2Co	6	0.0053	1	1
ReS_2	6	1.5988	1	1
Ca_2N	6	0.0063	1	1
$AuTe_2$	6	0.4836	1	1
$PdTe_2$	6	0.4773	1	1
Mg_3	6	0.1208	1	1
I_2Zn	6	0.4957	1	1
Te_2Zn	6	0.0002	1	1
S_2W	6	0.263	1	1
Bi_2Pd	6	0.1401	1	1
GeI_2	6	2.9628	1	1
Br_2Mn	6	0.0035	1	1
$CrTe_2$	6	0.0075	1	1
PtS_2	6	0.0012	1	1
$CoTe_2$	6	0.0089	1	1
CdClO	6	0.0014	1	1
Ba_2N	6	0.4676	1	1
Se_2Ti	6	0.0038	1	1
Br_2Ti	6	0.0081	1	1
Te_2Zr	6	0.465	1	1
Te_2W	6	0.0002	1	1
Cl_2Cu	6	0.5971	1	1
Cl_2V	6	0.2688	1	1
OTl_2	6	0.0016	1	1
Br_2Fe	6	0.0054	1	1
Br_2Cr	6	0.0078	1	1

Table showing the first 50 lattice matching combinations with the smallest number of atoms in the supercell.

Lattice matching - minimal strain

Formula	N° atoms	strain	cell size 1	cell size 2
Cu_3Se_3	711	0.0	91	73
Gd_2I_2	291	0.0001	49	36
O_2Pt	390	0.0001	57	73
I_2Zn	339	0.0001	64	49
Gd_2GeI_2	233	0.0001	36	25
Sb_2	257	0.0001	57	43
$AuTe_2$	390	0.0001	73	57
NaPSn	543	0.0001	100	81
BiClTe	183	0.0001	36	25
I_2La_2Sb	368	0.0002	61	37
CoH_2O_2	414	0.0002	43	57
Te_2W	6	0.0002	1	1
$HNiO_2$	219	0.0002	25	36
Te_2Zn	6	0.0002	1	1
K	220	0.0002	61	37
Cl_2OV	519	0.0002	77	72
CdI_2	183	0.0002	36	25
Bi_2In_2	197	0.0002	39	20
I_2Pr	183	0.0002	36	25
Br_2Cu_2	387	0.0002	65	48
$PtTe_2$	390	0.0003	73	57
FHOZn	643	0.0003	81	100
Cu_2I_2	291	0.0003	49	36
Br_2Gd_2Ge	327	0.0003	49	36
$Cu_2Rb_2Te_2$	237	0.0003	39	20
Bi_2Se_3	327	0.0003	49	36
Ba_2N	492	0.0003	91	73
$Br_2O_2Tb_2$	483	0.0003	65	48
Pt_2Te_2	624	0.0003	100	81
Br_2La_2	343	0.0004	57	43
Br_2La	255	0.0004	49	36
$CrSe_2$	543	0.0004	81	100
H_2NiO_2	674	0.0004	73	91
$Br_2Hf_2N_2$	30	0.0004	4	3
Br_2Cd	435	0.0004	81	64
Li_2P_2Pr	386	0.0004	57	43
$GeNi_3Te_2$	786	0.0004	100	81
$HfLiS_2$	7	0.0005	1	1
$PdTe_2$	435	0.0005	81	64
Br_2La_2P	233	0.0005	36	25
$Ga_2Ge_2Te_2$	429	0.0006	57	43
$I_2S_2Sm_2$	405	0.0006	61	37
Ba_2Cu_2	139	0.0006	25	16
CdH_2O_2	8	0.0006	1	1
Cu_2O_2	479	0.0006	81	59
Br_2PY_2	563	0.0006	81	64
CdI_2	183	0.0006	36	25
$I_2O_2Sm_2$	840	0.0007	118	81
I_2Mg	255	0.0007	49	36
Br_2Er_2	447	0.0007	73	57

Table showing the first 50 lattice matching combinations with the smallest strain needed to build the supercell.

N_4 ($Pmn2_1$)

Structural and electronic properties

**Formula**	N_4
**Spacegroup**	$Pmn2_1$
**Prototype**	N
**Parent 3D**	N_8
**Source DB**	ICSD
**DB ID**	671899
**DF2-C09 Binding energy [meV/ Å^2]**	17.58
**RVV10 Binding energy [meV/ Å^2]**	N/A
**Band gap (PBE) [eV]**	0.86

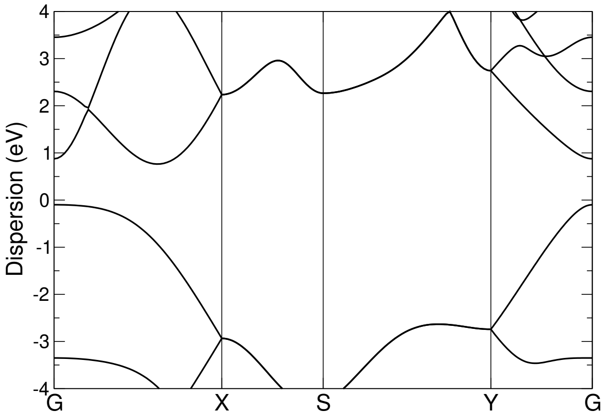

Band structure: Electronic band structure of N_4 ($Pmn2_1$) in a representative window around the Fermi energy and along a high-symmetry path.

Optimized crystal structure

Structural parameters: relaxed cell (top) and atomic positions (bottom) of N_4 ($Pmn2_1$) in Cartesian coordinates.

	x [Å]	y [Å]	z [Å]
$\mathbf{a_1}$	2.31551874	0.00000000	0.00000000
$\mathbf{a_2}$	0.00000000	3.82772593	0.00000000
$\mathbf{a_3}$	0.00000000	0.00000000	13.75280590

		x [Å]	y [Å]	z [Å]
•	N	1.73663906	-3.61024633	-0.43631588
•	N	0.57887969	-1.69638337	0.43631588
•	N	1.73663906	-2.13134256	-0.43631588
•	N	0.57887969	-4.04520553	0.43631588

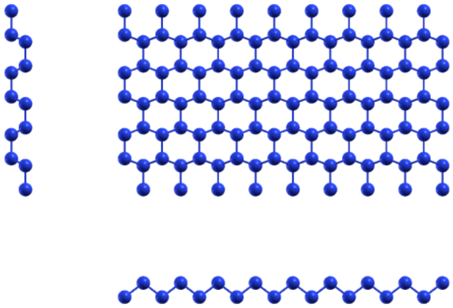

Orthographic projections: views of N_4 ($Pmn2_1$) as seen from the x axis (left), the y axis (bottom) and the z axis (center).

Lattice matching - minimal size

Formula	N° atoms	strain	cell size 1	cell size 2
GeTe	6	1.3858	1	1
C_2	6	0.4782	1	1
$IrTe_2$	7	1.3917	1	1
$CdCl_2$	7	1.3789	1	1
$ReSe_2$	7	2.832	1	1
$InSe_2$	7	1.3813	1	1
I_2Mn	7	1.3796	1	1
Cl_2Ni	7	2.8435	1	1
$AsSe_2$	7	2.8621	1	1
$NbSe_2$	7	2.8489	1	1
Se_2Ta	7	2.8501	1	1
H_2Si_2	8	1.3833	1	1
S_2Zn_2	8	1.4047	1	1
P_2Sn_2	8	1.4023	1	1
Ga_2Se_2	8	1.3929	1	1
BN	8	0.4744	1	2
K	9	0.2805	2	1
K	9	0.0126	2	1
In_2S_3	9	1.3871	1	1
Ni_2SbTe_2	9	1.3971	1	1
AsSb	10	0.2398	2	1
Cr_2O_4	10	0.5976	1	1
LiO_2	10	1.5611	1	2
GeTe	10	0.2475	2	1
Ag_2	10	0.0162	2	1
$Br_2Cr_2O_2$	10	0.3582	1	1
Cu_2O_4	10	0.8043	1	1
$Cl_2N_2Ti_2$	10	0.3557	1	1
$Ca_2Cl_2F_2$	10	0.4956	1	1
$Br_2O_2V_2$	10	0.3442	1	1
S_2	10	0.2495	2	1
$C_2I_2Y_2$	10	0.7811	1	1
$Cl_2Er_2H_2$	10	1.3803	1	1
$IrTe_2$	11	0.2487	2	1
I_2Mg	11	0.1229	2	1
$CdCl_2$	11	0.2461	2	1
Ba_2Pt	11	0.016	2	1
CaI_2	11	0.0114	2	1
$InSe_2$	11	0.2466	2	1
$GeTe_2$	11	0.2449	2	1
$SiTe_2$	11	0.2325	2	1
$HfTe_2$	11	0.2599	2	1
I_2Mn	11	0.2463	2	1
Br_2La	11	0.123	2	1
Ca_2Si	11	0.0197	2	1
I_2Yb	11	0.0123	2	1
PbS_2	11	0.2367	2	1
BrCdI	11	0.1245	2	1
FeI_2	11	0.2435	2	1
I_2Ni	11	0.2451	2	1

Table showing the first 50 lattice matching combinations with the smallest number of atoms in the supercell.

Lattice matching - minimal strain

Formula	N° atoms	strain	cell size 1	cell size 2
$NiTe_2$	663	0.0001	108	77
LiO_2	562	0.0003	85	74
CrI_2	212	0.0005	35	24
CaClHO	740	0.0005	108	77
$CuGeO_3$	605	0.0006	90	49
S_2Sn_2	380	0.0006	63	32
$C_2Br_2La_2$	738	0.0006	99	57
Br_2Mg	212	0.0006	35	24
$Cl_2O_2Tm_2$	894	0.0006	108	77
In	315	0.0007	63	63
$SiTe_2$	663	0.0007	108	77
FeI_2	212	0.0007	35	24
Au_2I_2	708	0.0007	121	56
$MoSe_2$	204	0.0008	30	28
$Br_2S_2Y_2$	316	0.0009	49	20
ClNZr	456	0.0009	69	60
NSr_2	212	0.0009	35	24
CoH_2O_2	262	0.0009	28	30
Se_2W	204	0.0009	30	28
$Cl_2Er_2O_2$	894	0.001	108	77
Mo_2Te_4	96	0.001	15	6
Bi_2Se_2	756	0.0011	126	63
Br_2Cu	476	0.0011	80	52
$AgClO_2$	532	0.0011	81	52
Se_2Zr	663	0.0011	108	77
Se_2Sn	212	0.0011	35	24
Te_4TiZr	212	0.0011	35	12
$Cl_2Cr_2O_2$	34	0.0012	4	3
$C_2I_2La_2$	438	0.0013	60	33
CI_2Y_2	260	0.0013	35	24
Cl_2Tb_2	592	0.0013	89	59
$C_2I_2Y_2$	624	0.0014	81	50
F_2Na	663	0.0014	108	77
Tl	148	0.0015	30	28
CdClHO	280	0.0015	40	30
$Hf_2I_2N_2$	340	0.0015	40	30
$Cl_2O_2Yb_2$	894	0.0015	108	77
P_4	380	0.0016	60	35
Sn	467	0.0016	93	95
$GeTe_2$	212	0.0017	35	24
Na	443	0.0017	92	75
I_2Ti	212	0.0017	35	24
Cu_2O_4	510	0.0017	60	45
CKN	189	0.0017	36	15
Sn	391	0.0017	78	79
C_2F_2	560	0.0017	56	84
$TaTe_2$	250	0.0018	40	30
Na	385	0.0018	80	65
O_2Sn_2	396	0.0018	58	41
Au_2Br_2	656	0.0018	110	54

Table showing the first 50 lattice matching combinations with the smallest strain needed to build the supercell.

Na_2PdH_2 (P4/mmm)

Structural and electronic properties

**Formula**	Na_2PdH_2
**Spacegroup**	P4/mmm
**Prototype**	Na2HgO2
**Parent 3D**	Na_2PdH_2
**Source DB**	ICSD
**DB ID**	68071
**DF2-C09 Binding energy [meV/ Å^2]**	29.8
**RVV10 Binding energy [meV/ Å^2]**	N/A
**Band gap (PBE) [eV]**	N/A

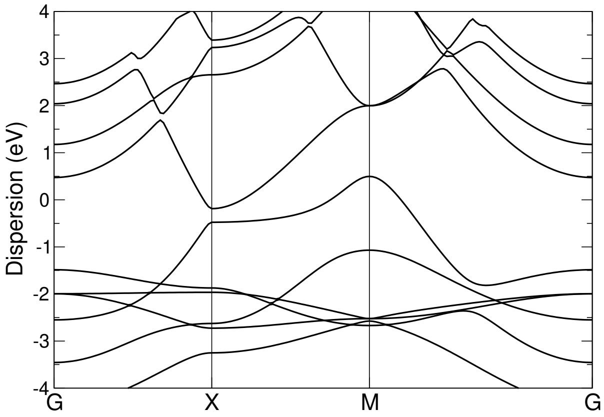

Band structure: Electronic band structure of Na_2PdH_2 (P4/mmm) in a representative window around the Fermi energy and along a high-symmetry path.

Optimized crystal structure

Structural parameters: relaxed cell (top) and atomic positions (bottom) of Na_2PdH_2 (P4/mmm) in Cartesian coordinates.

	x [Å]	y [Å]	z [Å]
$\mathbf{a_1}$	3.50295963	0.00000000	0.00000000
$\mathbf{a_2}$	0.00000000	3.50295963	0.00000000
$\mathbf{a_3}$	0.00000000	0.00000000	23.37825231

		x [Å]	y [Å]	z [Å]
•	H	0.00000000	0.00000000	13.38993280
●	Na	1.75147981	1.75147981	10.09839698
●	Na	1.75147981	1.75147981	13.27985533
•	H	0.00000000	3.50295963	9.98831950
●	Pd	0.00000000	3.50295963	11.68912615

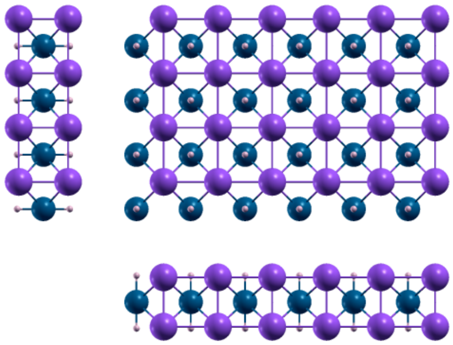

Orthographic projections: views of Na_2PdH_2 (P4/mmm) as seen from the x axis (left), the y axis (bottom) and the z axis (center).

Lattice matching - minimal size

Formula	N° atoms	strain	cell size 1	cell size 2
Tl	6	0.2212	1	1
Tl	6	0.1086	1	1
InSe	7	0.7732	1	1
AsSb	7	0.1498	1	1
K	7	1.6941	1	2
GeTe	7	0.1558	1	1
S_2	7	0.1574	1	1
PbTe	7	0.7801	1	1
Mg_2	7	0.5906	1	1
Sb_2	7	0.1841	1	1
$IrTe_2$	8	0.1567	1	1
I_2Mg	8	0.1913	1	1
$CdCl_2$	8	0.1547	1	1
CdI_2	8	0.7868	1	1
PSn_2	8	0.1321	1	1
$MoSe_2$	8	0.1084	1	1
Br_2Zn	8	0.1406	1	1
$InSe_2$	8	0.1551	1	1
$AsSn_2$	8	0.135	1	1
$GeTe_2$	8	0.1537	1	1
$SiTe_2$	8	0.1442	1	1
$HfTe_2$	8	0.1656	1	1
I_2Pr	8	0.3859	1	1
I_2Mn	8	0.1548	1	1
S_2Zr	8	0.1315	1	1
Br_2La	8	0.1918	1	1
NSr_2	8	0.1514	1	1
PbS_2	8	0.1474	1	1
BiClTe	8	0.7882	1	1
$AuTe_2$	8	0.1745	1	1
BrCdI	8	0.1955	1	1
$PdTe_2$	8	0.1716	1	1
FeI_2	8	0.1526	1	1
I_2Ni	8	0.1539	1	1
Mg_3	8	0.2145	1	1
Te_2Ti	8	0.1408	1	1
CrI_2	8	0.1522	1	1
I_2Zn	8	0.18	1	1
$RhTe_2$	8	0.1372	1	1
GeI_2	8	0.1889	1	1
Ba_2N	8	0.1673	1	1
Se_2V	8	0.1088	1	1
AsKSn	8	0.7531	1	1
Te_2Zr	8	0.1661	1	1
$PbTe_2$	8	0.1942	1	1
I_2Nd	8	0.388	1	1
$NiTe_2$	8	0.1437	1	1
S_2Sn	8	0.1317	1	1
$SnTe_2$	8	0.186	1	1
I_2V	8	0.1454	1	1

Table showing the first 50 lattice matching combinations with the smallest number of atoms in the supercell.

Lattice matching - minimal strain

Formula	N° atoms	strain	cell size 1	cell size 2
$K_2Mn_2Sb_2$	625	0.0	65	50
$Br_2Ce_2O_2$	625	0.0	65	50
$Bi_2Cl_2O_2$	997	0.0	101	82
Pb_2Se_2	964	0.0001	128	81
H_4Ti	575	0.0001	50	65
$Br_2Nd_2O_2$	789	0.0001	81	64
$I_2O_2Y_2$	789	0.0001	81	64
$F_2Se_2Y_2$	930	0.0002	108	65
$Bi_2Br_2O_2$	800	0.0003	82	65
$Cu_2Se_2Tl_2$	461	0.0003	49	36
Ba_2Cd	255	0.0003	36	25
$I_2O_2Sm_2$	614	0.0003	64	49
HgI_2	725	0.0004	106	65
$F_2I_2Pb_2$	330	0.0004	36	25
LiO	370	0.0005	48	65
H_2Li_2Pd	565	0.0005	49	64
$Eu_2I_2O_2$	625	0.0005	65	50
$Bi_2Cl_2O_2$	986	0.0006	100	81
$Ca_2Ge_2Mn_2$	997	0.0006	101	82
$Eu_2I_2O_2$	614	0.0006	64	49
Cu_2I_2	389	0.0006	49	36
$Er_2I_2O_2$	800	0.0007	82	65
$Eu_2H_2I_2$	614	0.0007	64	49
Ni_2Se_2	824	0.0007	100	81
$Br_2Eu_2F_2$	800	0.0007	82	65
Mg_4	814	0.0007	82	101
$Br_2F_2Tm_2$	997	0.0007	101	82
Mg_3	571	0.0008	65	82
GeS_2	8	0.0008	1	1
$I_2Lu_2O_2$	997	0.0008	101	82
Pb_2Se_2	489	0.0008	65	41
$Ba_2F_2I_2$	833	0.0008	97	58
$H_2I_2Yb_2$	789	0.0009	81	64
$Ca_2H_2I_2$	625	0.0009	65	50
CeI_2	743	0.0009	100	81
$FeSe_2$	233	0.001	25	36
$Br_2Eu_2O_2$	986	0.001	100	81
$Br_2H_2Sr_2$	461	0.001	49	36
$Bi_2Br_2O_2$	789	0.001	81	64
H_4Ti	565	0.001	49	64
Tl	511	0.001	82	101
$AgNO_3$	605	0.001	79	42
$F_2I_2Yb_2$	614	0.0011	64	49
$K_2Mn_2Sb_2$	614	0.0011	64	49
$Ag_2K_2Se_2$	920	0.0011	106	65
$Br_2Ce_2O_2$	614	0.0011	64	49
$Ca_2Ge_2Mn_2$	986	0.0011	100	81
$As_2Cd_2K_2$	833	0.0012	97	58
O_4PTl	833	0.0012	97	58
I_2La_2Te	995	0.0012	118	81

Table showing the first 50 lattice matching combinations with the smallest strain needed to build the supercell.

$NaAlH_4$ (P-4)

Structural and electronic properties

**Formula**	$NaAlH_4$
**Spacegroup**	P-4
**Prototype**	CaWO4
**Parent 3D**	$Al_2H_8Na_2$
**Source DB**	COD
**DB ID**	1533929
**DF2-C09 Binding energy [meV/ Å^2]**	28.11
**RVV10 Binding energy [meV/ Å^2]**	N/A
**Band gap (PBE) [eV]**	5.1

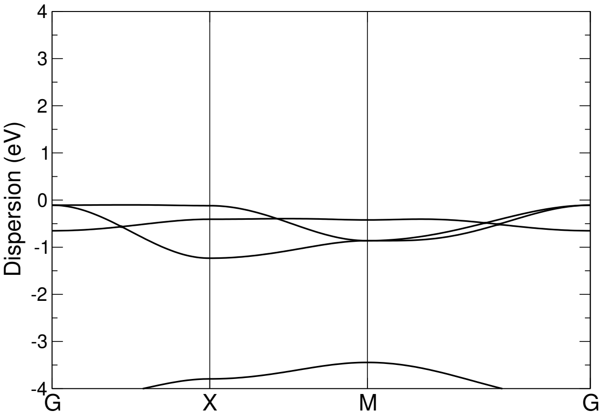

Band structure: Electronic band structure of $NaAlH_4$ (P-4) in a representative window around the Fermi energy and along a high-symmetry path.

Optimized crystal structure

Structural parameters: relaxed cell (top) and atomic positions (bottom) of $NaAlH_4$ (P-4) in Cartesian coordinates.

	x [Å]	y [Å]	z [Å]
$\mathbf{a_1}$	4.71554254	0.00000000	0.00000000
$\mathbf{a_2}$	0.00000000	4.71554254	0.00000000
$\mathbf{a_3}$	0.00000000	0.00000000	15.60216614

	x [Å]	y [Å]	z [Å]
Na	0.00000000	0.00000000	0.00000000
Al	2.35777127	-2.35777127	0.00000000
H	1.42421388	-1.42409466	0.96439493
H	3.29132866	-3.29144788	0.96439493
H	1.42409466	-3.29132866	-0.96439493
H	3.29144788	-1.42421388	-0.96439493

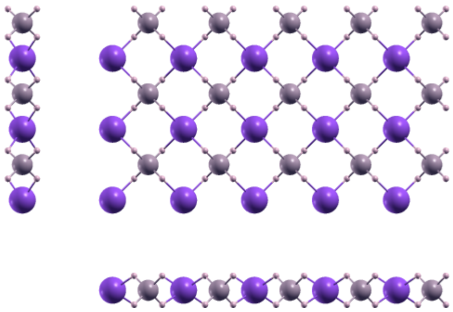

Orthographic projections: views of $NaAlH_4$ (P-4) as seen from the x axis (left), the y axis (bottom) and the z axis (center).

Lattice matching - minimal size

Formula	N° atoms	strain	cell size 1	cell size 2
AsSb	8	0.1113	1	1
GeTe	8	0.1099	1	1
Gd	8	0.1434	1	2
$IrTe_2$	9	0.1097	1	1
$CdCl_2$	9	0.1102	1	1
CaI_2	9	0.109	1	1
$InSe_2$	9	0.1101	1	1
I_2Mn	9	0.1101	1	1
Br_2Cu	9	0.1035	1	1
NSr_2	9	0.1109	1	1
I_2Yb	9	0.1084	1	1
FeI_2	9	0.1106	1	1
I_2Ni	9	0.1103	1	1
CrI_2	9	0.1107	1	1
Ba_2Hg	9	0.2117	1	1
CNRb	9	0.0361	1	1
CKN	9	0.2474	1	1
I_2Tm	9	0.1087	1	1
CoI_2	9	0.1112	1	1
Br_2Mg	9	0.1107	1	1
CNNa	9	0.4721	1	1
Se_2Sn	9	0.111	1	1
Ba_2Cd	9	0.2146	1	1
H_2Si_2	10	0.11	1	1
Li_2Tl_2	10	0.1396	1	1
Bi_2In_2	10	0.008	1	1
Cu_2I_2	10	0.5832	1	1
Nd	10	0.053	1	4
Cl_2OOs	10	1.6722	1	1
Au_2Br_2	10	0.23	1	1
Ge_2Te_2	10	0.2218	1	1
N_3Na	10	0.0308	1	1
S_2Zn_2	10	0.1093	1	1
As_4	10	0.1886	1	1
P_4	10	0.1619	1	1
P_2Sn_2	10	0.1094	1	1
$LiMnSe_2$	10	0.1088	1	1
Ga_2Se_2	10	0.1097	1	1
Au_2I_2	10	0.6483	1	1
Ni_2Te_2	10	0.1102	1	1
La_2S_2	10	0.2225	1	1
PbS_2Sn	10	0.2122	1	1
Ba_2Cu_2	10	0.1084	1	1
Cl_2Tb_2	10	0.109	1	1
Sm	10	0.1546	1	4
As_2O_3	11	0.174	1	1
F_4Sn	11	0.6124	1	1
GeI_2La_2	11	0.1087	1	1
PTe_2Zr_2	11	0.1115	1	1
F_4Nb	11	0.5945	1	1

Table showing the first 50 lattice matching combinations with the smallest number of atoms in the supercell.

Lattice matching - minimal strain

Formula	N° atoms	strain	cell size 1	cell size 2
Ho_2S_2	582	0.0001	49	72
$Er_2I_2O_2$	366	0.0001	25	36
$K_2Mn_2Sb_2$	876	0.0001	61	85
$Br_2Ce_2O_2$	876	0.0001	61	85
$Br_2Eu_2F_2$	366	0.0001	25	36
F_4Nb	614	0.0002	49	64
Cu_2F_4	870	0.0002	81	64
Br_2Pr_2	476	0.0002	36	65
HfS_2	237	0.0002	20	39
$FeSe_2$	471	0.0003	34	89
Cu_2I_2	962	0.0003	85	113
$Ho_2I_2S_2$	864	0.0003	72	72
GdI_2	483	0.0003	48	65
$CuTe_2$	237	0.0003	20	39
Co_2Se_2	736	0.0003	58	97
$Bi_2Br_2O_2$	366	0.0003	25	36
O_2Sn_2	504	0.0003	42	63
Ag_2F_4	516	0.0004	50	36
$Eu_2I_2O_2$	876	0.0004	61	85
$CoTe_2$	237	0.0005	20	39
$Fe_2Li_2P_2$	780	0.0005	49	81
Ge_2Se_2	574	0.0005	51	67
$I_2S_2Yb_2$	762	0.0006	63	64
S_2Sn_2	678	0.0006	61	78
Ga_2S_2	276	0.0006	20	39
AsKSn	840	0.0006	81	118
$Ca_2Cl_2F_2$	246	0.0006	16	25
CaH_2O_2	315	0.0007	20	39
I_2La_2P	613	0.0007	48	65
Br_2Ca_3Si	870	0.0007	64	81
F_2Na	411	0.0008	36	65
$I_2O_2Tm_2$	366	0.0008	25	36
$I_2Nd_2O_2$	510	0.0008	36	49
$Ge_2Te_2Zr_2$	246	0.0009	16	25
Ba_2Cd	636	0.0009	65	82
$Hf_2Si_2Te_2$	930	0.0009	58	97
$Cl_2N_2Zr_2$	354	0.0009	20	39
Cl_2Cu	546	0.0009	48	86
Ba_2Hg	627	0.001	64	81
$Ca_2H_2I_2$	876	0.001	61	85
Cu_2Se_2	814	0.001	65	106
$F_2I_2Pb_2$	882	0.001	65	82
Sn	214	0.001	25	64
As_2Co_2	736	0.0011	58	97
$PdTe_2$	852	0.0011	79	126
$Br_2Lu_2O_2$	246	0.0011	16	25
Fe_2Se_2	618	0.0011	49	81
$I_2S_2Tb_2$	954	0.0011	80	79
$Ho_2I_2S_2$	852	0.0011	71	71
Br_2Cd	852	0.0012	79	126

Table showing the first 50 lattice matching combinations with the smallest strain needed to build the supercell.

NaCN (Pmm2)

Structural and electronic properties

**Formula**	NaCN
**Spacegroup**	Pmm2
**Prototype**	NaCN
**Parent 3D**	$Na_2C_2N_2$
**Source DB**	ICSD
**DB ID**	77172
**DF2-C09 Binding energy [meV/ Å^2]**	20.79
**RVV10 Binding energy [meV/ Å^2]**	26.15
**Band gap (PBE) [eV]**	4.83

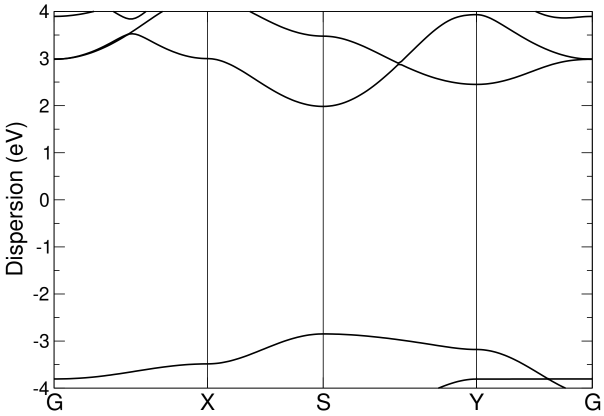

Band structure: Electronic band structure of NaCN (Pmm2) in a representative window around the Fermi energy and along a high-symmetry path.

Optimized crystal structure

Structural parameters: relaxed cell (top) and atomic positions (bottom) of NaCN (Pmm2) in Cartesian coordinates.

	x [Å]	y [Å]	z [Å]
$\mathbf{a_1}$	3.56439363	0.00000000	0.00000000
$\mathbf{a_2}$	0.00000000	4.71733012	0.00000000
$\mathbf{a_3}$	0.00000000	0.00000000	20.00000000

	x [Å]	y [Å]	z [Å]
Na	0.00000000	1.34876629	10.00000000
C	1.78219681	4.16073082	10.00000000
N	1.78219681	2.98480655	10.00000000

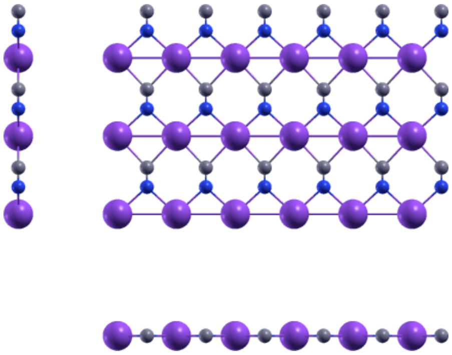

Orthographic projections: views of NaCN (Pmm2) as seen from the x axis (left), the y axis (bottom) and the z axis (center).

Lattice matching - minimal size

Formula	N° atoms	strain	cell size 1	cell size 2
AgTl	5	0.0782	1	1
CaCl	5	0.2606	1	1
Br_2Cu	6	0.6629	1	1
Cl_2Zn	6	0.2816	1	1
CNRb	6	0.4349	1	1
$MnSe_2$	6	0.2605	1	1
Ba_2Cd	6	0.3888	1	1
Cu_2I_2	7	0.0672	1	1
Cl_2OOs	7	0.276	1	1
$AgCuTe_2$	7	0.3222	1	1
S_2Sn_2	7	0.254	1	1
Au_2Br_2	7	0.3883	1	1
GeTe	7	0.3589	1	2
Cl_2ORu	7	0.2685	1	1
Cu_2Te_2	7	0.2856	1	1
O_2Pb_2	7	0.0659	1	1
As_4	7	0.0123	1	1
Fe_2S_2	7	0.7692	1	1
Co_2S_2	7	0.2615	1	1
O_2Sn_2	7	0.0786	1	1
Bi_2O_2	7	0.0664	1	1
Sn_2Te_2	7	0.3397	1	1
O_2Sn_2	7	0.0786	1	1
As_2O_3	8	0.3066	1	1
Hf_3Te_2	8	0.28	1	1
F_4Nb	8	0.0688	1	1
Cl_4Mn	8	0.9092	1	1
$Br_2In_2O_2$	9	0.336	1	1
$IrTe_2$	9	0.3609	1	2
$Br_2Ho_2S_2$	9	0.5763	1	1
$CdCl_2$	9	0.3566	1	2
$Ge_2Hf_2Te_2$	9	0.8495	1	1
$Br_2F_2Sr_2$	9	0.068	1	1
$Ho_2I_2S_2$	9	0.5883	1	1
Cu_4Te_2	9	0.5977	1	1
$Cl_2O_2Sc_2$	9	0.3039	1	1
CaI_2	9	1.0578	1	2
$InSe_2$	9	0.3574	1	2
$Eu_2F_2I_2$	9	0.0662	1	1
AlH_4Na	9	0.9208	1	1
$Br_2N_2Zr_2$	9	0.3348	1	1
$I_2N_2Zr_2$	9	0.3433	1	1
I_2Mn	9	0.3569	1	2
$F_2I_2Sm_2$	9	0.0658	1	1
NiO_2	9	0.3422	1	2
Ca_2Si	9	2.4396	1	2
$Cl_2N_2Zr_2$	9	0.0378	1	1
Tl	9	0.0355	2	3
$Br_2O_2Sc_2$	9	0.3087	1	1
FeI_2	9	0.3522	1	2

Table showing the first 50 lattice matching combinations with the smallest number of atoms in the supercell.

Lattice matching - minimal strain

Formula	N° atoms	strain	cell size 1	cell size 2
H_2Si_2	488	0.0002	60	77
$InSe_2$	411	0.0003	60	77
$Al_2Cl_2O_2$	654	0.0003	56	81
$Cl_2Er_2H_2$	642	0.0004	60	77
GeTe	334	0.0005	60	77
I_2Mn	411	0.0005	60	77
$CdCl_2$	411	0.0006	60	77
$Ca_2H_2I_2$	972	0.0006	104	110
$Br_2Hf_2N_2$	69	0.0007	7	8
In_2S_3	565	0.0007	60	77
I_2Zn	156	0.0008	24	28
Ni_2Te_2	488	0.0008	60	77
$Er_2F_2Se_2$	834	0.0009	80	99
$Br_2Ho_2S_2$	552	0.0009	72	56
$Er_2I_2S_2$	477	0.0009	63	48
$Br_2Lu_2S_2$	720	0.0009	92	74
$Cl_2Fe_2O_2$	987	0.001	85	122
$I_2Lu_2S_2$	645	0.001	83	66
ClH_3O	204	0.001	28	24
$Br_2Lu_2S_2$	720	0.001	92	74
$Br_2Ce_2O_2$	972	0.001	104	110
$K_2Mn_2Sb_2$	972	0.001	104	110
CS_3Tl_2	243	0.001	49	16
S_2Ti	432	0.0011	54	90
$I_2S_2Tm_2$	477	0.0011	63	48
$AgCuTe_2$	590	0.0011	74	92
$SiTe_2$	423	0.0011	60	81
$NiTe_2$	423	0.0011	60	81
Cl_2Co	432	0.0011	54	90
$Cl_2Er_2O_2$	666	0.0012	60	81
$Gd_2I_2S_2$	765	0.0012	103	76
Cl_2ORu	57	0.0012	7	9
$Cl_2Ho_2O_2$	585	0.0012	53	71
$Cl_2Ga_2Te_2$	768	0.0012	108	74
I_2V	372	0.0012	53	71
$Cl_2Er_2S_2$	645	0.0012	83	66
Se_2Zr	423	0.0012	60	81
$F_2Lu_2Se_2$	240	0.0012	24	28
As_2Ir_2	752	0.0013	104	110
In_2Se_2	459	0.0013	77	57
$F_2I_2Pb_2$	954	0.0013	110	104
Br_2Cu	495	0.0013	74	91
Cl_2Zr	432	0.0013	54	90
$Br_2Er_2S_2$	552	0.0013	72	56
$C_2Br_2Tb_2$	144	0.0014	14	17
$IrTe_2$	411	0.0014	60	77
Ba_2Cd	642	0.0014	110	104
CaClHO	504	0.0014	60	81
$I_2La_2O_2$	918	0.0014	104	101
$Cu_2Se_2Tl_2$	933	0.0014	103	104

Table showing the first 50 lattice matching combinations with the smallest strain needed to build the supercell.

NaOH (Pmmn)

Structural and electronic properties

**Formula**	NaOH
**Spacegroup**	Pmmn
**Prototype**	KOH
**Parent 3D**	$Na_2(OH)_2$
**Source DB**	ICSD
**DB ID**	26833
**DF2-C09 Binding energy [meV/ Å^2]**	12.89
**RVV10 Binding energy [meV/ Å^2]**	23.56
**Band gap (PBE) [eV]**	2.78

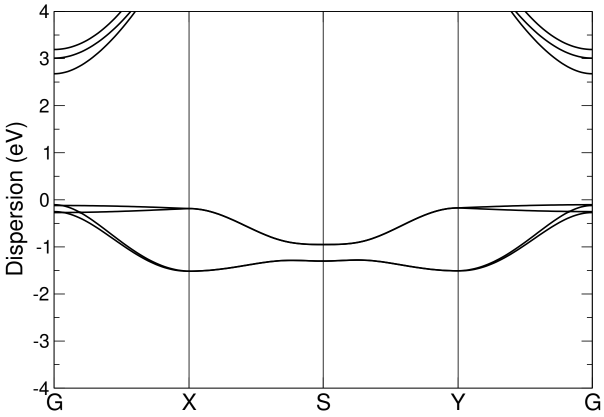

Band structure: Electronic band structure of NaOH (Pmmn) in a representative window around the Fermi energy and along a high-symmetry path.

Optimized crystal structure

Structural parameters: relaxed cell (top) and atomic positions (bottom) of NaOH (Pmmn) in Cartesian coordinates.

	x [Å]	y [Å]	z [Å]
$\mathbf{a_1}$	3.40158188	0.00000000	0.00000000
$\mathbf{a_2}$	0.00000000	3.42044482	0.00000000
$\mathbf{a_3}$	0.00000000	0.00000000	24.60736639

	x [Å]	y [Å]	z [Å]
Na	0.85046925	1.71022241	13.31798927
H	0.85040796	1.71022241	9.99987618
O	0.85032884	1.71022241	10.96889519
Na	2.55113148	3.42044482	11.28937585
H	2.55119223	3.42044482	14.60747332
O	2.55128373	0.00000000	13.63848937

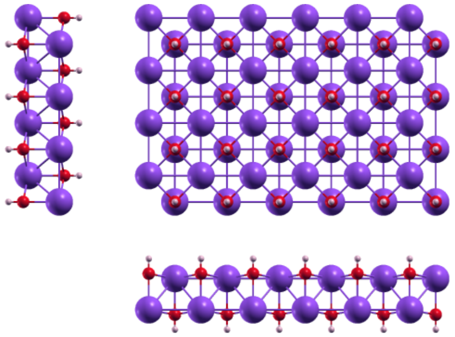

Orthographic projections: views of NaOH (Pmmn) as seen from the x axis (left), the y axis (bottom) and the z axis (center).

Lattice matching - minimal size

Formula	N° atoms	strain	cell size 1	cell size 2
Sn	7	0.583	1	1
Na	7	0.129	1	1
In	7	0.5906	1	1
AsSb	8	0.1626	1	1
GeTe	8	0.1693	1	1
As_2	8	0.1349	1	1
S_2	8	0.1711	1	1
Mg_2	8	0.2161	1	1
$IrTe_2$	9	0.1704	1	1
Cl_2Zn	9	0.1341	1	1
I_2Mg	9	0.7792	1	1
$CdCl_2$	9	0.1681	1	1
$MoTe_2$	9	0.1306	1	1
PSn_2	9	0.1422	1	1
Br_2Zn	9	0.1521	1	1
HfS_2	9	0.137	1	1
$InSe_2$	9	0.1685	1	1
$AsSn_2$	9	0.1456	1	1
$GeTe_2$	9	0.167	1	1
$SiTe_2$	9	0.1562	1	1
$HfTe_2$	9	0.1803	1	1
Te_2V	9	0.132	1	1
I_2Pr	9	1.1097	1	1
I_2Mn	9	0.1682	1	1
$CuTe_2$	9	0.1368	1	1
S_2Zr	9	0.1416	1	1
Br_2La	9	0.7805	1	1
NSr_2	9	0.1644	1	1
NiO_2	9	0.1096	1	1
PbS_2	9	0.1598	1	1
Br_2Co	9	0.1345	1	1
Ca_2N	9	0.1353	1	1
$AuTe_2$	9	0.1901	1	1
LiO_2	9	0.1027	1	1
$PdTe_2$	9	0.1869	1	1
FeI_2	9	0.1658	1	1
I_2Ni	9	0.1672	1	1
Te_2Ti	9	0.1524	1	1
CrI_2	9	0.1653	1	1
I_2Zn	9	0.1961	1	1
Te_2Zn	9	0.1305	1	1
$RhTe_2$	9	0.1482	1	1
GeI_2	9	0.772	1	1
Br_2Mn	9	0.1332	1	1
PtS_2	9	0.1297	1	1
$CoTe_2$	9	0.1373	1	1
CdClO	9	0.1317	1	1
Ba_2N	9	0.1821	1	1
Te_2Zr	9	0.1809	1	1
Te_2W	9	0.1307	1	1

Table showing the first 50 lattice matching combinations with the smallest number of atoms in the supercell.

Lattice matching - minimal strain

Formula	N° atoms	strain	cell size 1	cell size 2
$Cl_2Ga_2Te_2$	372	0.0001	42	20
$CuGeO_3$	536	0.0003	56	40
Na	749	0.0005	106	113
Bi_2Se_4	906	0.0009	112	39
PtS_2	975	0.0009	106	113
K	901	0.001	136	85
O_4PSn	150	0.001	16	9
C_2	416	0.0011	40	88
$GeNi_3Te_2$	156	0.0011	14	12
NaPSn	120	0.0012	14	12
$HNiO_2$	254	0.0012	21	32
$I_2S_2Tb_2$	318	0.0013	32	21
Pt_2Te_2	132	0.0013	14	12
$Ca_2Mn_2Si_2$	882	0.0013	82	65
$Cu_2Na_2Se_2$	876	0.0013	85	61
$Bi_2I_2O_2$	876	0.0013	85	61
Ni_2Se_2	580	0.0013	64	49
$F_2I_2Tm_2$	876	0.0013	85	61
$Cl_2F_2Pb_2$	876	0.0013	85	61
Cu_2I_2	316	0.0013	36	25
Au_2Br_2	428	0.0013	50	32
$Br_2Ho_2O_2$	882	0.0013	82	65
As_2Ir_2	438	0.0013	49	36
$Bi_2Cl_2O_2$	690	0.0013	65	50
CeI_2	531	0.0013	64	49
H_2Li_2Pd	986	0.0013	81	100
$Br_2Ca_2H_2$	882	0.0013	82	65
H_4Ti	997	0.0013	82	101
BiITe	873	0.0013	108	75
$Br_2Eu_2O_2$	678	0.0013	64	49
$Br_2H_2Sr_2$	366	0.0013	36	25
H_4Ti	986	0.0013	81	100
PtS_2	900	0.0013	98	104
PtS_2	891	0.0014	97	103
$Ag_2K_2Te_2$	444	0.0014	49	25
F_4Pb	872	0.0014	97	58
In	358	0.0014	49	64
$Br_2Ho_2O_2$	870	0.0014	81	64
P_2Rh_2	742	0.0014	81	64
Sn_2Te_2	132	0.0015	16	9
$K_2O_2Tl_2$	948	0.0015	106	52
O_2Pt	483	0.0015	48	65
$As_2Cd_2K_2$	150	0.0015	16	9
F_2Tl_2	742	0.0015	81	64
Sn	623	0.0015	85	113
$Ca_2Ge_2Mn_2$	690	0.0015	65	50
$HfLiS_2$	994	0.0015	97	103
$Ca_2Mn_2Si_2$	870	0.0015	81	64
In	411	0.0015	56	75
DyI_2	255	0.0015	32	21

Table showing the first 50 lattice matching combinations with the smallest strain needed to build the supercell.

Nb_2CS_2 (P-3m1)

Structural and electronic properties

**Formula**	Nb_2CS_2
**Spacegroup**	P-3m1
**Prototype**	Bi2Te2S
**Parent 3D**	Nb_2CS_2
**Source DB**	COD
**DB ID**	1532143
**DF2-C09 Binding energy [meV/ Å^2]**	24.69
**RVV10 Binding energy [meV/ Å^2]**	31.85
**Band gap (PBE) [eV]**	N/A

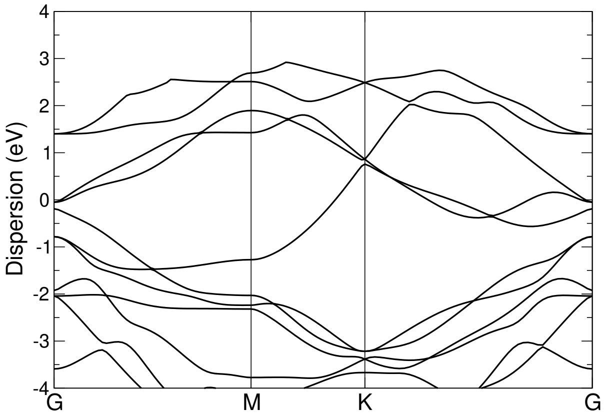

Band structure: Electronic band structure of Nb_2CS_2 (P-3m1) in a representative window around the Fermi energy and along a high-symmetry path.

Optimized crystal structure

Structural parameters: relaxed cell (top) and atomic positions (bottom) of Nb_2CS_2 (P-3m1) in Cartesian coordinates.

		x [Å]	y [Å]	z [Å]
$\mathbf{a_1}$		3.28075411	0.00000000	0.00000000
$\mathbf{a_2}$		−1.64037705	2.84121640	0.00000000
$\mathbf{a_3}$		0.00000000	0.00000000	25.65930162
		x [Å]	y [Å]	z [Å]
●	Nb	1.64037705	0.94707213	11.63553630
●	S	-0.00000000	1.89414427	9.99720564
●	Nb	-0.00000000	1.89414427	14.02376532
●	C	0.00000000	0.00000000	12.82965081
●	S	1.64037705	0.94707213	15.66209597

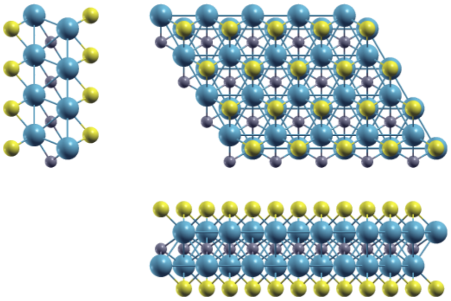

Orthographic projections: views of Nb_2CS_2 (P-3m1) as seen from the x axis (left), the y axis (bottom) and the z axis (center).

Lattice matching - minimal size

Formula	N° atoms	strain	cell size 1	cell size 2
Tl	6	0.0058	1	1
Sn	6	0.1288	1	1
In	6	0.1319	1	1
As_2	7	0.4494	1	1
LiO	7	0.007	1	1
P_2	7	0.0005	1	1
Mg_2	7	0.1412	1	1
PSn_2	8	0.4716	1	1
$MoSe_2$	8	0.0051	1	1
Br_2Zn	8	0.4994	1	1
HfS_2	8	0.4561	1	1
$AsSn_2$	8	0.4813	1	1
$CuTe_2$	8	0.4552	1	1
S_2Zr	8	0.4697	1	1
NSr_2	8	3.0454	1	1
NiO_2	8	1.5616	1	1
Br_2Co	8	0.4484	1	1
Ca_2N	8	0.4507	1	1
Cl_2Ti	8	0.0007	1	1
Mg_3	8	0.1507	1	1
Te_2Ti	8	0.5002	1	1
$RhTe_2$	8	2.8366	1	1
NbS_2	8	0.009	1	1
$CoTe_2$	8	0.4569	1	1
S_2Ta	8	0.0081	1	1
Se_2V	8	0.0067	1	1
$NiTe_2$	8	2.9345	1	1
S_2Sn	8	0.4703	1	1
STl_2	8	13.6114	1	1
$PtSe_2$	8	0.483	1	1
CoI_2	8	3.0293	1	1
Br_2Fe	8	0.4485	1	1
$TaTe_2$	8	0.4798	1	1
Br_2Ni	8	0.461	1	1
$FeSe_2$	8	0.1245	1	1
$NbTe_2$	8	0.4691	1	1
Cl_2Mg	8	0.4612	1	1
I_2La	8	0.3175	1	1
$CrSe_2$	8	0.0082	1	1
N_2Re	8	0.2471	1	1
Se_2Sn	8	3.0421	1	1
CoO_2	8	1.5577	1	1
$HfSe_2$	8	0.5003	1	1
Se_2W	8	0.0052	1	1
CdClHO	9	0.4783	1	1
$HNiO_2$	9	0.2721	1	1
Ag_2Br_2	9	0.3185	1	1
CdClHO	9	0.4867	1	1
Cl_2Y_2	9	0.4961	1	1
As_2Ir_2	9	0.3234	1	1

Table showing the first 50 lattice matching combinations with the smallest number of atoms in the supercell.

Lattice matching - minimal strain

Formula	N° atoms	strain	cell size 1	cell size 2
Cl_2Mg	743	0.0	100	81
F_2Na	414	0.0	57	43
Br_2Ni	743	0.0	100	81
$Cl_2O_2Yb_2$	543	0.0001	57	43
$PtSe_2$	536	0.0001	73	57
Br_2La_2	189	0.0001	25	16
STl_2	552	0.0001	81	49
PTe_2Ti_2	905	0.0002	100	81
BaF_2	416	0.0002	61	37
Ca_2Si	512	0.0002	79	39
$Ce_2I_2S_2$	629	0.0002	79	39
I_2Y_2	280	0.0002	36	25
Cl_2Y_2	516	0.0003	64	49
$Cl_2H_2Lu_2$	986	0.0003	100	81
CBr_2Lu_2	820	0.0003	91	73
Sb_2	157	0.0003	25	16
Au_2Br_2	825	0.0004	117	60
Cu_2O_4	495	0.0004	51	40
$LiMnSe_2$	280	0.0004	36	25
$Br_2N_2Zr_2$	893	0.0004	91	73
$Ga_2I_2Y_2$	527	0.0004	61	37
Ga_2Se_2	516	0.0004	64	49
$Br_2Hf_2N_2$	862	0.0005	98	62
$Se_2Si_2Zr_2$	613	0.0005	65	48
P_2	7	0.0005	1	1
Ga_2S_2	824	0.0005	100	81
N_3W_2	725	0.0005	64	81
$F_2Se_2Tm_2$	330	0.0005	36	25
PbS_2	353	0.0006	49	36
Cu_2Se_2	914	0.0006	118	81
$AlLiTe_2$	116	0.0006	16	9
Cl_2Ti	8	0.0007	1	1
K_2O_4	213	0.0007	33	8
$AsSn_2$	536	0.0007	73	57
Cl_2Tb_2	280	0.0007	36	25
CS_2Ta_2	10	0.0007	1	1
FeO_2	327	0.0007	36	49
STl_2	416	0.0008	61	37
KNO_3	25	0.0008	4	1
Br_2Pr_2	457	0.0008	57	43
$Cl_2O_2Tm_2$	543	0.0008	57	43
CBr_2Y_2	565	0.0008	64	49
Br_2Y_2	389	0.0008	49	36
F_2Ni	833	0.0008	118	81
$NbTe_2$	674	0.0009	91	73
Li_2P_2Pr	205	0.0009	25	16
CdClHO	661	0.0009	81	64
$Hf_2I_2N_2$	789	0.0009	81	64
CaClHO	457	0.0009	57	43
$Ga_2Ge_2Te_2$	221	0.001	25	16

Table showing the first 50 lattice matching combinations with the smallest strain needed to build the supercell.

NbF_4 (P4/mmm)

Structural and electronic properties

**Formula**	NbF_4
**Spacegroup**	P4/mmm
**Prototype**	SnF4
**Parent 3D**	NbF_4
**Source DB**	ICSD
**DB ID**	25768
**DF2-C09 Binding energy [meV/ Å^2]**	15.15
**RVV10 Binding energy [meV/ Å^2]**	29.07
**Band gap (PBE) [eV]**	N/A

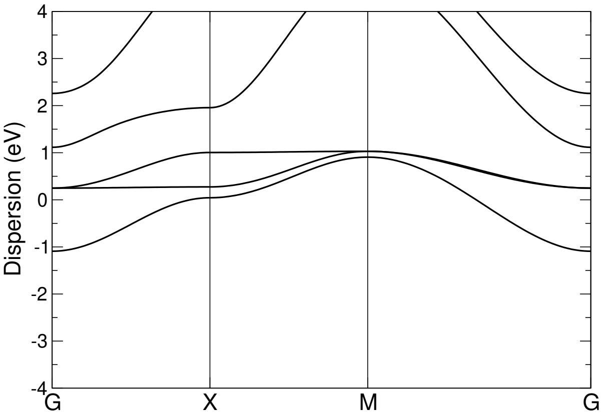

Band structure: Electronic band structure of NbF_4 (P4/mmm) in a representative window around the Fermi energy and along a high-symmetry path.

Optimized crystal structure

Structural parameters: relaxed cell (top) and atomic positions (bottom) of NbF_4 (P4/mmm) in Cartesian coordinates.

	x [Å]	y [Å]	z [Å]
$\mathbf{a_1}$	4.12470603	0.00000000	0.00000000
$\mathbf{a_2}$	0.00000000	4.12470603	0.00000000
$\mathbf{a_3}$	0.00000000	0.00000000	23.77635710

	x [Å]	y [Å]	z [Å]
F	2.06235301	2.06235301	13.76422298
Nb	2.06235301	2.06235301	11.88817855
F	2.06235301	2.06235301	10.01213412
F	2.06235301	4.12470603	11.88817855
F	4.12470603	2.06235301	11.88817855

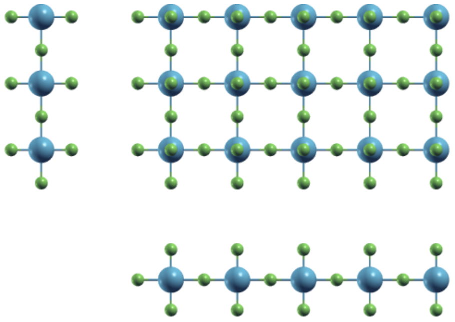

Orthographic projections: views of NbF_4 (P4/mmm) as seen from the x axis (left), the y axis (bottom) and the z axis (center).

Lattice matching - minimal size

Formula	N° atoms	strain	cell size 1	cell size 2
K	6	0.1549	1	1
Bi_2	7	0.1295	1	1
AgTl	7	0.0145	1	1
Ag_2	7	0.1606	1	1
CaCl	7	0.5913	1	1
Cl_2Mn	8	0.1113	1	1
Ba_2Pt	8	0.1603	1	1
S_2Ta	8	0.1108	1	1
CaI_2	8	0.1461	1	1
Br_2Cu	8	0.0977	1	1
Ca_2Si	8	0.1652	1	1
I_2Yb	8	0.1438	1	1
Cl_2Zn	8	0.2194	1	1
S_2Ti	8	0.1097	1	1
NbS_2	8	0.1109	1	1
BiBrTe	8	0.1321	1	1
Ba_2Hg	8	0.0067	1	1
Cl_2Co	8	0.1098	1	1
ClNZr	8	0.1091	1	1
Cl_2Fe	8	0.11	1	1
I_2Tm	8	0.145	1	1
I_2Pb	8	0.1623	1	1
BiITe	8	0.1372	1	1
$MnSe_2$	8	0.5911	1	1
DyI_2	8	0.1487	1	1
Cl_2Zr	8	0.1099	1	1
GdI_2	8	0.1343	1	1
CNNa	8	0.0665	1	1
F_2Ni	8	0.2148	1	1
Ba_2Cd	8	0.0094	1	1
Bi_2Te_2	9	0.18	1	1
Bi_2In_2	9	0.3938	1	1
Cu_2I_2	9	0.0037	1	1
Cu_2Sr_2	9	0.1329	1	1
Cl_2OOs	9	0.2092	1	1
Cu_2Te_2	9	0.1862	1	1
Bi_2Mn_2	9	0.1672	1	1
O_2Sn_2	9	0.2268	1	1
S_2Sn_2	9	0.0154	1	1
Au_2Br_2	9	0.0175	1	1
$AlLiTe_2$	9	0.1363	1	1
Fe_2Se_2	9	0.2142	1	1
Cl_2ORu	9	0.2112	1	1
As_2Co_2	9	0.2088	1	1
N_3Na	9	0.3796	1	1
O_2Pb_2	9	0.007	1	1
S_2Zn_2	9	0.1088	1	1
Ge_2S_2	9	0.2445	1	1
As_4	9	0.0612	1	1
Cl_2Zr_2	9	0.1088	1	1

Table showing the first 50 lattice matching combinations with the smallest number of atoms in the supercell.

Lattice matching - minimal strain

Formula	N° atoms	strain	cell size 1	cell size 2
Cl_2Zn	705	0.0	81	100
$Cu_2Rb_2Te_2$	800	0.0002	82	65
AlH_4Na	614	0.0002	64	49
Bi_2In_2	670	0.0002	82	65
$Cl_2Ho_2O_2$	630	0.0002	48	65
Ho_2S_2	76	0.0002	8	9
Cu_2O_2	76	0.0003	8	9
Cl_2OOs	644	0.0003	64	81
Bi_2Se_2	785	0.0003	89	85
H_2MgO_2	295	0.0003	20	39
Cu_2F_4	699	0.0003	81	49
$Fe_2Li_2P_2$	806	0.0004	64	81
I_3Sn	345	0.0004	49	25
I_2V	435	0.0005	48	65
Fe_2Se_2	653	0.0005	65	82
AuI_4Li	658	0.0005	80	43
Co_2S_2	501	0.0005	49	64
Cl_4Cu_2	69	0.0006	9	4
Bi_2In_2	661	0.0006	81	64
S_2Zr	759	0.0006	81	118
$Cl_2O_2Y_2$	630	0.0006	48	65
S_2Sn	759	0.0006	81	118
N_2Re	660	0.0007	54	130
$I_2S_2Yb_2$	87	0.0007	9	7
$NbTe_2$	759	0.0007	81	118
$C_2Cl_2Y_2$	646	0.0007	50	66
Tl	570	0.0008	85	145
Mg_6	731	0.0008	49	81
$AgTe_2$	581	0.0009	58	97
$Cu_2Rb_2Te_2$	789	0.0009	81	64
PSn_2	759	0.0009	81	118
Fe_2S_2	877	0.0009	85	113
Cl_2OOs	635	0.001	63	80
$FeSe_2$	817	0.001	74	149
$CrTe_2$	773	0.0011	79	126
$Si_2Te_2Zr_2$	817	0.0011	65	82
F_2Ni	571	0.0011	65	82
Cl_2Sc_2	899	0.0011	79	126
$Cu_2K_2Te_2$	997	0.0011	101	82
N_2Re	585	0.0011	48	115
Hf_3Te_2	905	0.0011	81	100
C_2Li_2	577	0.0011	61	68
Ga_2Se_2	500	0.0011	48	65
Br_2Cr	773	0.0012	79	126
S_2V	217	0.0012	20	39
H_2Na_2Pd	730	0.0012	61	85
CaCl	373	0.0012	49	64
LiO_2	551	0.0012	55	92
Sn	174	0.0012	25	49
$MnSe_2$	437	0.0013	49	64

Table showing the first 50 lattice matching combinations with the smallest strain needed to build the supercell.

NbS_2 (P-3m1)

Structural and electronic properties

**Formula**	NbS_2
**Spacegroup**	P-3m1
**Prototype**	CdI2
**Parent 3D**	NbS_2
**Source DB**	ICSD
**DB ID**	24755
**DF2-C09 Binding energy [meV/ Å^2]**	23.15
**RVV10 Binding energy [meV/ Å^2]**	27.99
**Band gap (PBE) [eV]**	N/A

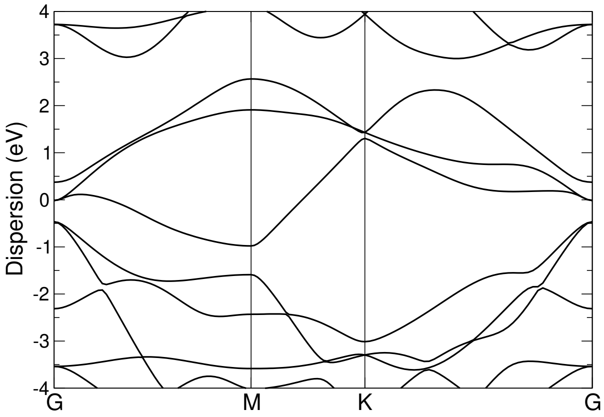

Band structure: Electronic band structure of NbS_2 (P-3m1) in a representative window around the Fermi energy and along a high-symmetry path.

Optimized crystal structure

Structural parameters: relaxed cell (top) and atomic positions (bottom) of NbS_2 (P-3m1) in Cartesian coordinates.

	x [Å]	y [Å]	z [Å]
$\mathbf{a_1}$	3.37329862	0.00000000	0.00000000
$\mathbf{a_2}$	−1.68664931	2.92136230	0.00000000
$\mathbf{a_3}$	0.00000000	0.00000000	23.10244315

		x [Å]	y [Å]	z [Å]
●	S	1.68664931	0.97378743	13.09442176
●	Nb	0.00000000	0.00000000	11.55122158
●	S	-0.00000000	1.94757487	10.00802139

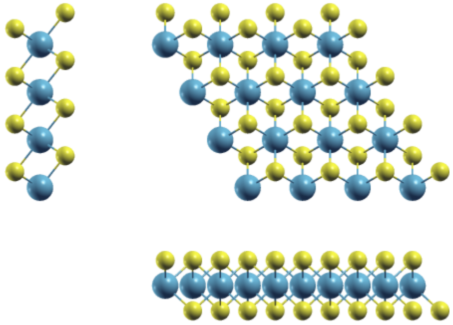

Orthographic projections: views of NbS_2 (P-3m1) as seen from the x axis (left), the y axis (bottom) and the z axis (center).

Lattice matching - minimal size

Formula	N° atoms	strain	cell size 1	cell size 2
Tl	4	0.144	1	1
Tl	4	0.0072	1	1
Sn	4	0.1206	1	1
In	4	0.1231	1	1
GeTe	5	2.9295	1	1
Mg_2	5	0.1308	1	1
CrS_2	6	0.2738	1	1
$CdCl_2$	6	2.9155	1	1
Cl_2Mn	6	0.0016	1	1
$AgTe_2$	6	0.1467	1	1
$MoSe_2$	6	0.008	1	1
S_2Ta	6	0.0005	1	1
Br_2Zn	6	0.4654	1	1
$InSe_2$	6	2.9204	1	1
$AsSn_2$	6	0.4483	1	1
$SiTe_2$	6	0.4758	1	1
I_2Mn	6	2.9171	1	1
NSr_2	6	0.496	1	1
PbS_2	6	0.4849	1	1
FeI_2	6	0.4994	1	1
I_2Ni	6	2.905	1	1
S_2Ti	6	0.0055	1	1
Mg_3	6	0.1389	1	1
Te_2Ti	6	0.4661	1	1
CrI_2	6	0.4982	1	1
$RhTe_2$	6	0.4552	1	1
N_2W	6	0.2639	1	1
Cl_2Co	6	0.0051	1	1
NbS_2	6	0.0043	1	1
ClNZr	6	0.0087	1	1
Cl_2Fe	6	0.0044	1	1
S_2Ta	6	0.0051	1	1
Se_2V	6	0.0064	1	1
$NiTe_2$	6	0.4743	1	1
Cl_2Cu	6	0.1136	1	1
I_2V	6	0.4793	1	1
Se_2Zr	6	0.4769	1	1
$PtSe_2$	6	0.4499	1	1
CdO_2	6	0.005	1	1
CoI_2	6	0.4928	1	1
O_2Zn	6	0.2689	1	1
Cl_2Zr	6	0.0048	1	1
$FeSe_2$	6	0.1172	1	1
Br_2Mg	6	0.499	1	1
I_2Ti	6	0.4937	1	1
F_2Na	6	0.4703	1	1
Se_2Sn	6	0.4953	1	1
NaPSn	6	3.0406	1	1
$HfSe_2$	6	0.4662	1	1
Se_2W	6	0.0078	1	1

Table showing the first 50 lattice matching combinations with the smallest number of atoms in the supercell.

Lattice matching - minimal strain

Formula	N° atoms	strain	cell size 1	cell size 2
Se_2Sn	339	0.0	64	49
I_2Y_2	291	0.0	49	36
Ga_2Se_2	447	0.0001	73	57
$SiTe_2$	435	0.0001	81	64
$CdCl_2$	300	0.0001	57	43
BiBrTe	294	0.0001	61	37
Ni_2Te_2	343	0.0001	57	43
$Cl_2Er_2O_2$	627	0.0002	81	64
$Hf_2Si_2Te_2$	483	0.0002	65	48
As_2Sn_2	208	0.0002	36	25
CI_2Y_2	437	0.0002	64	49
I_2Mn	300	0.0002	57	43
Bi_2Te_2	393	0.0002	79	39
NSr_2	339	0.0002	64	49
Cu_2Sr_2	439	0.0003	81	49
Ga_2Se_2	624	0.0003	100	81
Br_2Gd_2	208	0.0003	36	25
N_2W	492	0.0003	73	91
STl_2	123	0.0003	25	16
$HfSe_2$	492	0.0003	91	73
Te_2Ti	492	0.0003	91	73
$Cl_2Er_2H_2$	429	0.0003	57	43
H_2MnO_2	414	0.0003	43	57
Se_2Zr	435	0.0003	81	64
Br_2Y_2	447	0.0004	73	57
Cl_2Y_2	624	0.0004	100	81
S_2Ta	6	0.0005	1	1
$InSe_2$	300	0.0005	57	43
$Cl_2O_2Y_2$	561	0.0005	73	57
$SbSe_2Tl$	86	0.0005	18	8
PbS_2Sn	782	0.0005	150	83
$LiNbS_2$	7	0.0005	1	1
$LiMnSe_2$	291	0.0006	49	36
Cl_2Tb_2	291	0.0006	49	36
Br_2Pr_2	565	0.0006	91	73
Co_2Se_2	387	0.0006	65	48
Br_2Zn	492	0.0006	91	73
I_2Ti	339	0.0006	64	49
PbS_2	390	0.0006	73	57
Br_2Hg_3	32	0.0006	9	1
BaF_2	123	0.0007	25	16
$NiTe_2$	435	0.0007	81	64
$F_2Se_2Tm_2$	363	0.0007	49	36
I_2Ni	300	0.0007	57	43
CCl_2Gd_2	638	0.0007	91	73
H_2Si_2	343	0.0008	57	43
Cl_2OOs	599	0.0008	101	74
Li_2Tl_2	43	0.0008	9	4
Br_2Er_2	208	0.0008	36	25
Cl_2La_2	208	0.0009	36	25

Table showing the first 50 lattice matching combinations with the smallest strain needed to build the supercell.

NbS_2 (P-6m2)

Structural and electronic properties

**Formula**	NbS_2
**Spacegroup**	P-6m2
**Prototype**	MoS2
**Parent 3D**	NbS_2
**Source DB**	ICSD
**DB ID**	237034
**DF2-C09 Binding energy [meV/ Å^2]**	24.34
**RVV10 Binding energy [meV/ Å^2]**	29.0
**Band gap (PBE) [eV]**	N/A

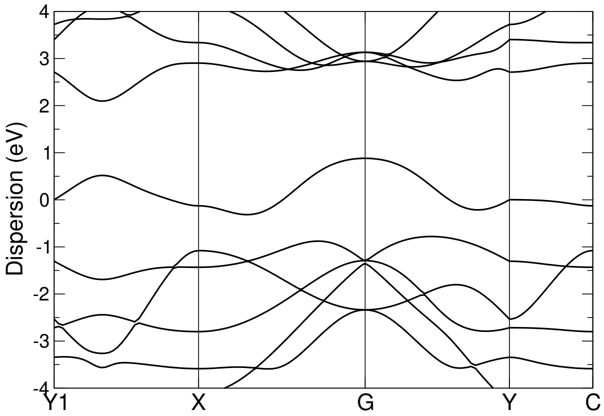

Band structure: Electronic band structure of NbS_2 (P-6m2) in a representative window around the Fermi energy and along a high-symmetry path.

Optimized crystal structure

Structural parameters: relaxed cell (top) and atomic positions (bottom) of NbS_2 (P-6m2) in Cartesian coordinates.

		x [Å]	y [Å]	z [Å]
$\mathbf{a_1}$		3.34259995	−0.00008796	0.00000000
$\mathbf{a_2}$		1.67149725	2.89466258	0.00000000
$\mathbf{a_3}$		0.00000000	0.00000000	23.14326909
		x [Å]	y [Å]	z [Å]
●	Nb	4.61216779	2.66254588	11.57163454
●	S	1.26946882	0.73284822	10.00669861
●	S	1.26946882	0.73284822	13.13657047

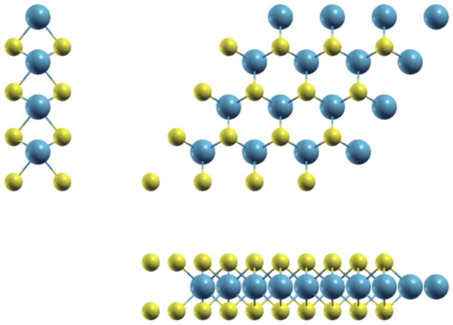

Orthographic projections: views of NbS_2 (P-6m2) as seen from the x axis (left), the y axis (bottom) and the z axis (center).

Lattice matching - minimal size

Formula	N° atoms	strain	cell size 1	cell size 2
Tl	4	0.148	1	1
Tl	4	0.003	1	1
Sn	4	0.1231	1	1
In	4	0.1258	1	1
AsSb	5	2.9069	1	1
P_2	5	0.0081	1	1
Mg_2	5	0.1341	1	1
Cl_2Mn	6	0.0027	1	1
$AgTe_2$	6	0.1508	1	1
PSn_2	6	0.4497	1	1
$MoSe_2$	6	0.0038	1	1
S_2Ta	6	0.0048	1	1
Br_2Zn	6	0.4763	1	1
$AsSn_2$	6	0.459	1	1
$SiTe_2$	6	0.487	1	1
S_2Zr	6	0.4478	1	1
PbS_2	6	0.4962	1	1
Cl_2Ti	6	0.008	1	1
Mg_3	6	0.1426	1	1
Te_2Ti	6	0.4771	1	1
NbS_2	6	0.0044	1	1
$RhTe_2$	6	0.466	1	1
N_2W	6	0.2701	1	1
Cl_2Co	6	0.0096	1	1
Cl_2Fe	6	0.0088	1	1
S_2Ta	6	0.0008	1	1
Se_2V	6	0.0022	1	1
$NiTe_2$	6	0.4854	1	1
Cl_2Cu	6	0.1185	1	1
S_2Sn	6	0.4485	1	1
$PtSe_2$	6	0.4606	1	1
CdO_2	6	0.0094	1	1
O_2Zn	6	0.2752	1	1
$TaTe_2$	6	0.4575	1	1
Cl_2Zr	6	0.0092	1	1
$FeSe_2$	6	0.1194	1	1
$NbTe_2$	6	0.4473	1	1
F_2Na	6	0.4814	1	1
$HfSe_2$	6	0.4771	1	1
Se_2W	6	0.0036	1	1
Cu_2I_2	7	0.3329	1	1
CdClHO	7	0.4562	1	1
Br_2Pr_2	7	0.4794	1	1
$HNiO_2$	7	0.2595	1	1
Cl_2Hf_2	7	0.006	1	1
Bi_2Mn_2	7	0.2399	1	1
CdClHO	7	0.4641	1	1
Cl_2Y_2	7	0.4731	1	1
O_2Pb_2	7	0.326	1	1
As_4	7	0.384	1	1

Table showing the first 50 lattice matching combinations with the smallest number of atoms in the supercell.

Lattice matching - minimal strain

Formula	N° atoms	strain	cell size 1	cell size 2
CCl_2Gd_2	563	0.0	81	64
Bi_2	341	0.0	81	49
$MnNaTe_2$	139	0.0001	25	16
Cl_2Cu	720	0.0001	135	105
$Br_2Hf_2N_2$	549	0.0001	79	52
Br_2Y_2	388	0.0001	64	49
$Ga_2I_2Tb_2$	405	0.0001	61	37
$Cl_2O_2Tm_2$	561	0.0001	73	57
Br_2Zn	435	0.0001	81	64
$IrTe_2$	255	0.0001	49	36
CoI_2	300	0.0001	57	43
$HNiO_2$	516	0.0002	64	81
$Ga_2Gd_2I_2$	405	0.0002	61	37
I_2Ti	300	0.0002	57	43
H_2Li_2Pt	643	0.0002	81	80
$PtSe_2$	543	0.0002	100	81
CaClHO	447	0.0002	73	57
Ga_2Se_2	388	0.0003	64	49
$Cl_2H_2Zr_2$	9	0.0003	1	1
SSb_2Te_2	155	0.0003	25	16
PbS_2	339	0.0003	64	49
Ga_2Se_2	291	0.0003	49	36
BrCdI	123	0.0003	25	16
$RhTe_2$	492	0.0004	91	73
C_2F_2	355	0.0004	37	61
Br_2Cd	183	0.0004	36	25
Te_2Ti	435	0.0004	81	64
BN	59	0.0004	9	16
$HfSe_2$	435	0.0004	81	64
Br_2Ca	294	0.0005	61	37
NS_2Ta	643	0.0005	81	100
$PdTe_2$	183	0.0005	36	25
H_2MnO_2	467	0.0005	49	64
In_2S_3	327	0.0006	49	36
CBr_2Y_2	563	0.0006	81	64
Bi_2SeTe_2	488	0.0006	81	49
Ba_2Cu_2	84	0.0006	16	9
AsSb	257	0.0006	57	43
Br_2PY_2	233	0.0006	36	25
Bi_2	257	0.0006	61	37
CI_2Y_2	386	0.0006	57	43
$PbTe_2$	123	0.0006	25	16
$As_2Fe_2Li_2$	840	0.0006	118	81
$Br_2H_2Yb_2$	840	0.0006	118	81
F_2Na	390	0.0007	73	57
$Cu_2Na_2Te_2$	237	0.0007	39	20
N_2Re	255	0.0007	36	49
Fe_2SeTe	678	0.0007	118	81
I_2Yb	75	0.0007	16	9
$I_2N_2Zr_2$	711	0.0007	91	73

Table showing the first 50 lattice matching combinations with the smallest strain needed to build the supercell.

$NbSe_2$ (P-6m2)

Structural and electronic properties

**Formula**	$NbSe_2$
**Spacegroup**	P-6m2
**Prototype**	MoS2
**Parent 3D**	Nb_2Se_4
**Source DB**	COD
**DB ID**	9014575
**DF2-C09 Binding energy [meV/ Å^2]**	23.51
**RVV10 Binding energy [meV/ Å^2]**	28.78
**Band gap (PBE) [eV]**	N/A

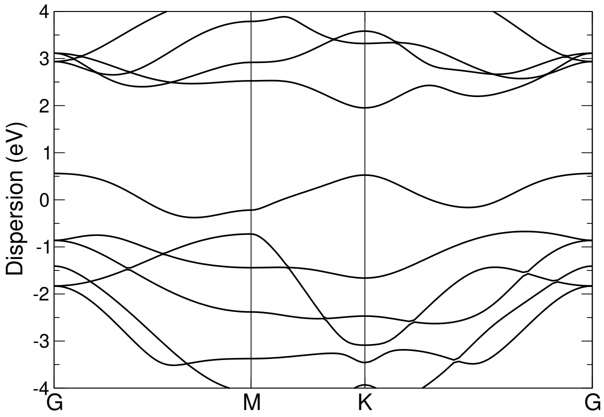

Band structure: Electronic band structure of $NbSe_2$ (P-6m2) in a representative window around the Fermi energy and along a high-symmetry path.

Optimized crystal structure

Structural parameters: relaxed cell (top) and atomic positions (bottom) of $NbSe_2$ (P-6m2) in Cartesian coordinates.

		x [Å]	y [Å]	z [Å]
$\mathbf{a_1}$		3.46608415	0.00000000	0.00000000
$\mathbf{a_2}$		−1.73304207	3.00171692	0.00000000
$\mathbf{a_3}$		0.00000000	0.00000000	23.35992895
		x [Å]	y [Å]	z [Å]
●	Nb	0.00000000	2.00114462	11.67996447
●	Se	1.73304207	1.00057231	13.36256072
●	Se	1.73304207	1.00057231	9.99736823

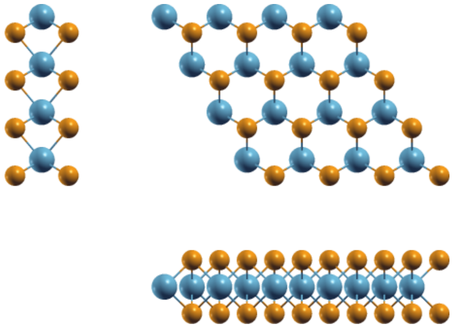

Orthographic projections: views of $NbSe_2$ (P-6m2) as seen from the x axis (left), the y axis (bottom) and the z axis (center).

Lattice matching - minimal size

Formula	N° atoms	strain	cell size 1	cell size 2
Tl	4	0.1334	1	1
Sn	4	0.1144	1	1
In	4	0.1163	1	1
HgO	5	0.1434	1	1
AsSb	5	0.4589	1	1
GeTe	5	0.4741	1	1
S_2	5	0.4781	1	1
Mg_2	5	0.1224	1	1
$IrTe_2$	6	0.4765	1	1
CrS_2	6	0.2556	1	1
$CdCl_2$	6	0.4714	1	1
$AgTe_2$	6	0.1358	1	1
$ReSe_2$	6	0.0013	1	1
$InSe_2$	6	0.4724	1	1
$GeTe_2$	6	0.4689	1	1
$HfTe_2$	6	0.4983	1	1
I_2Mn	6	0.4717	1	1
NSr_2	6	0.4629	1	1
I_2Yb	6	13.65	1	1
PbS_2	6	0.4525	1	1
ReS_2	6	0.2639	1	1
$AuTe_2$	6	2.9848	1	1
FeI_2	6	0.4661	1	1
I_2Ni	6	0.4693	1	1
S_2Ti	6	0.0073	1	1
Mg_3	6	0.1291	1	1
CrI_2	6	0.465	1	1
Bi_2Pd	6	0.1523	1	1
Ba_2Hg	6	0.3189	1	1
N_2W	6	0.2465	1	1
Cl_2Ni	6	0.0004	1	1
Cl_2Co	6	0.0076	1	1
$CrTe_2$	6	0.0058	1	1
Br_2V	6	0.0021	1	1
ClNZr	6	0.0042	1	1
Cl_2Fe	6	0.0083	1	1
Ba_2N	6	2.9029	1	1
Br_2Ti	6	0.0052	1	1
Te_2Zr	6	0.4996	1	1
$AsSe_2$	6	0.001	1	1
I_2V	6	0.4472	1	1
CdO_2	6	0.0077	1	1
BrNZr	6	0.0024	1	1
CoI_2	6	0.46	1	1
Br_2Cr	6	0.0055	1	1
Cl_2Zr	6	0.0079	1	1
$FeSe_2$	6	0.1118	1	1
Se_2Ta	6	0.0001	1	1
Br_2Mg	6	0.4658	1	1
I_2Ti	6	0.4608	1	1

Table showing the first 50 lattice matching combinations with the smallest number of atoms in the supercell.

Lattice matching - minimal strain

Formula	N° atoms	strain	cell size 1	cell size 2
Gd_2I_2	208	0.0	36	25
$F_2Se_2Y_2$	297	0.0001	49	25
CrS_2	390	0.0001	57	73
Se_2Ta	6	0.0001	1	1
I_2Ti	543	0.0001	100	81
$IrTe_2$	435	0.0002	81	64
NaPSn	339	0.0002	64	49
$HNiO_2$	304	0.0002	36	49
Br_2Gd_2	291	0.0002	49	36
Bi_2Se_3	233	0.0002	36	25
$As_2Fe_2Li_2$	483	0.0003	65	48
BiBrTe	123	0.0003	25	16
Cl_2Gd_2	447	0.0003	73	57
$Br_2H_2Yb_2$	483	0.0003	65	48
CI_2Y_2	705	0.0003	100	81
Cu_2I_2	208	0.0003	36	25
Br_2Gd_2Ge	233	0.0003	36	25
As_2Sn_2	291	0.0003	49	36
ReS_2	492	0.0003	73	91
FeI_2	492	0.0003	91	73
Br_2La	183	0.0003	36	25
Br_2Er_2	291	0.0003	49	36
Pt_2Te_2	388	0.0004	64	49
Ga_2Se_2	499	0.0004	81	64
$GeNi_3Te_2$	486	0.0004	64	49
Cl_2Ni	6	0.0004	1	1
Br_2Mg	492	0.0004	91	73
Se_2Sn	543	0.0005	100	81
CoI_2	543	0.0005	100	81
Fe_2SeTe	387	0.0005	65	48
In_2S_3	563	0.0005	81	64
Ba_2Pt	75	0.0005	16	9
Ba_2Cu_2	439	0.0006	81	49
S_2Zn_2	447	0.0006	73	57
I_2Mg	183	0.0007	36	25
CrI_2	492	0.0007	91	73
NSr_2	543	0.0007	100	81
GeTe	371	0.0007	81	64
$F_2Ho_2Se_2$	429	0.0007	57	43
Cu_2Sr_2	139	0.0008	25	16
I_2Yb	390	0.0008	81	49
$GeTe_2$	492	0.0008	91	73
NS_2Ta	357	0.0008	43	57
Ag_2	66	0.0008	16	9
S_2	371	0.0008	81	64
$Er_2F_2Se_2$	486	0.0008	64	49
Ni_2Se_2	678	0.0008	118	81
$AgCuTe_2$	553	0.0009	91	70
$F_2Se_2Yb_2$	561	0.0009	73	57
$Ce_2I_2Si_2$	258	0.0009	36	25

Table showing the first 50 lattice matching combinations with the smallest strain needed to build the supercell.

$NbSe_2$ (P-3m1)

Structural and electronic properties

**Formula**	$NbSe_2$
**Spacegroup**	P-3m1
**Prototype**	CdI2
**Parent 3D**	$NbSe_2$
**Source DB**	ICSD
**DB ID**	76576
**DF2-C09 Binding energy [meV/ Å^2]**	27.14
**RVV10 Binding energy [meV/ Å^2]**	32.01
**Band gap (PBE) [eV]**	N/A

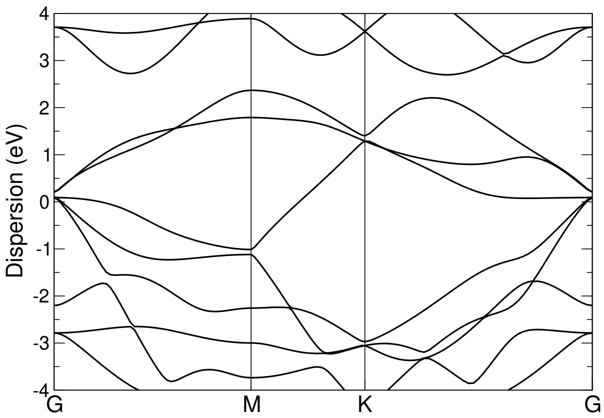

Band structure: Electronic band structure of $NbSe_2$ (P-3m1) in a representative window around the Fermi energy and along a high-symmetry path.

Optimized crystal structure

Structural parameters: relaxed cell (top) and atomic positions (bottom) of $NbSe_2$ (P-3m1) in Cartesian coordinates.

	x [Å]	y [Å]	z [Å]
$\mathbf{a_1}$	3.47568271	0.00000000	0.00000000
$\mathbf{a_2}$	−1.73784135	3.01002952	0.00000000
$\mathbf{a_3}$	0.00000000	0.00000000	23.35794018

		x [Å]	y [Å]	z [Å]
●	Se	0.00000000	2.00668635	10.00406506
●	Nb	0.00000000	0.00000000	11.67897009
●	Se	1.73784135	1.00334317	13.35387512

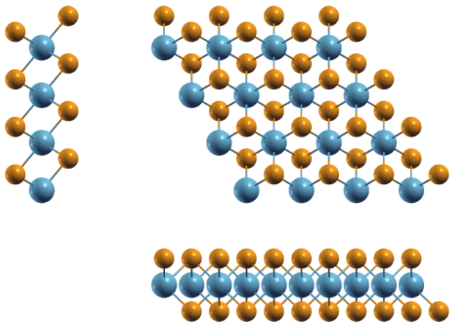

Orthographic projections: views of $NbSe_2$ (P-3m1) as seen from the x axis (left), the y axis (bottom) and the z axis (center).

Lattice matching - minimal size

Formula	N° atoms	strain	cell size 1	cell size 2
Tl	4	0.1325	1	1
Sn	4	0.1138	1	1
In	4	0.1157	1	1
In	4	0.2747	1	1
HgO	5	0.1423	1	1
AsSb	5	0.4556	1	1
GeTe	5	0.4708	1	1
S_2	5	0.4748	1	1
Mg_2	5	0.1217	1	1
$IrTe_2$	6	0.4731	1	1
CrS_2	6	0.2538	1	1
$CdCl_2$	6	0.4681	1	1
$AgTe_2$	6	0.1347	1	1
$ReSe_2$	6	0.0026	1	1
$InSe_2$	6	0.469	1	1
$GeTe_2$	6	0.4656	1	1
$HfTe_2$	6	0.4948	1	1
I_2Mn	6	0.4684	1	1
NSr_2	6	0.4597	1	1
PbS_2	6	0.4493	1	1
ReS_2	6	0.2621	1	1
$AuTe_2$	6	2.9675	1	1
$PdTe_2$	6	2.9353	1	1
FeI_2	6	0.4629	1	1
I_2Ni	6	0.4661	1	1
S_2Ti	6	0.0085	1	1
Mg_3	6	0.1282	1	1
CrI_2	6	0.4617	1	1
I_2Zn	6	3.0289	1	1
Bi_2Pd	6	0.151	1	1
Ba_2Hg	6	0.3162	1	1
N_2W	6	1.5931	1	1
Cl_2Ni	6	0.0017	1	1
Cl_2Co	6	0.0089	1	1
$CrTe_2$	6	0.0044	1	1
Br_2V	6	0.0034	1	1
ClNZr	6	0.0055	1	1
Ba_2N	6	0.4989	1	1
Se_2Ti	6	0.0083	1	1
Br_2Ti	6	0.0038	1	1
Te_2Zr	6	0.4961	1	1
$AsSe_2$	6	0.0003	1	1
I_2Tm	6	13.6529	1	1
CdO_2	6	0.009	1	1
BrNZr	6	0.0011	1	1
$NbSe_2$	6	0.0013	1	1
CoI_2	6	0.4567	1	1
O_2Zn	6	0.2494	1	1
Br_2Cr	6	0.0041	1	1
Cl_2Zr	6	0.0092	1	1

Table showing the first 50 lattice matching combinations with the smallest number of atoms in the supercell.

Lattice matching - minimal strain

Formula	N° atoms	strain	cell size 1	cell size 2
Nd	172	0.0	37	61
Cl_2La_2	291	0.0001	49	36
Ba_2Cu_2	331	0.0001	61	37
O_2Zn	339	0.0001	49	64
S_2Sn_2	378	0.0001	70	42
N_2W	300	0.0001	43	57
I_2Yb	294	0.0001	61	37
$Br_2Lu_2O_2$	483	0.0002	65	48
Br_2OV	38	0.0002	6	5
$PbTe_2$	183	0.0002	36	25
In_2Se_3	437	0.0002	64	49
I_2Y_2	447	0.0002	73	57
$I_2Pr_2S_2$	102	0.0002	16	9
C_2F_2	91	0.0002	9	16
$HfTe_2$	339	0.0002	64	49
$Ge_2Te_2Zr_2$	483	0.0002	65	48
Te_2Zr	339	0.0003	64	49
$AsSe_2$	6	0.0003	1	1
Ni_2Te_2	565	0.0003	91	73
CrI_2	543	0.0003	100	81
Ni_2SbTe_2	563	0.0003	81	64
Cl_2Tb_2	447	0.0003	73	57
I_2Ni	492	0.0003	91	73
I_2La_2P	155	0.0004	25	16
Hg_3N_2	17	0.0004	4	1
$Er_2F_2Se_2$	486	0.0005	64	49
$AgCuTe_2$	553	0.0005	91	70
S_2	371	0.0005	81	64
$CdCl_2$	492	0.0005	91	73
P_2Sn_2	499	0.0005	81	64
Br_2PY_2	386	0.0005	57	43
Ag_2	66	0.0005	16	9
$GeTe_2$	492	0.0005	91	73
Cu_2Sr_2	139	0.0005	25	16
Br_2Mg	543	0.0005	100	81
$F_2Ho_2Se_2$	429	0.0006	57	43
GeI_2La_2	488	0.0006	81	49
$Ba_2F_2I_2$	237	0.0006	39	20
I_2Mn	492	0.0006	91	73
NSr_2	543	0.0006	100	81
$F_2Se_2Yb_2$	627	0.0006	81	64
Br_2Hf_2	7	0.0006	1	1
FeI_2	543	0.0007	100	81
Br_2Cd	300	0.0007	57	43
I_2Pr	597	0.0007	118	81
O_2Sn_2	688	0.0007	108	91
$Cl_2Er_2H_2$	711	0.0007	91	73
$Br_2Eu_2O_2$	840	0.0008	118	81
Ba_2Pt	75	0.0008	16	9
$MnNaTe_2$	208	0.0008	36	25

Table showing the first 50 lattice matching combinations with the smallest strain needed to build the supercell.

$NbTe_2$ (P-3m1)

Structural and electronic properties

**Formula**	$NbTe_2$
**Spacegroup**	P-3m1
**Prototype**	CdI2
**Parent 3D**	$NbTe_2$
**Source DB**	ICSD
**DB ID**	645529
**DF2-C09 Binding energy [meV/ Å^2]**	28.27
**RVV10 Binding energy [meV/ Å^2]**	31.83
**Band gap (PBE) [eV]**	N/A

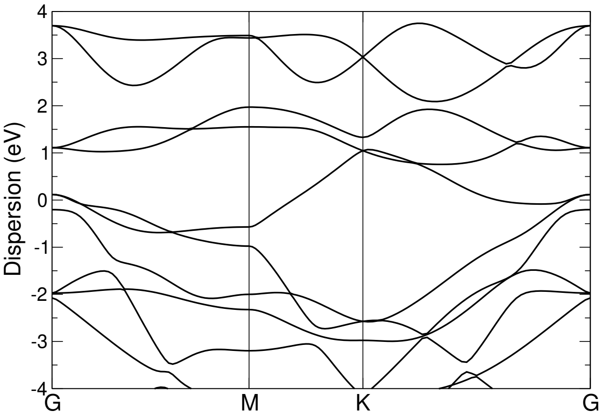

Band structure: Electronic band structure of $NbTe_2$ (P-3m1) in a representative window around the Fermi energy and along a high-symmetry path.

Optimized crystal structure

Structural parameters: relaxed cell (top) and atomic positions (bottom) of $NbTe_2$ (P-3m1) in Cartesian coordinates.

	x [Å]	y [Å]	z [Å]
$\mathbf{a_1}$	3.66986029	0.00000000	0.00000000
$\mathbf{a_2}$	−1.83493015	3.17819224	0.00000000
$\mathbf{a_3}$	0.00000000	0.00000000	23.66028845

		x [Å]	y [Å]	z [Å]
●	Te	1.83493015	1.05939741	13.68666895
●	Nb	0.00000000	0.00000000	11.83014422
●	Te	0.00000000	2.11879483	9.97361949

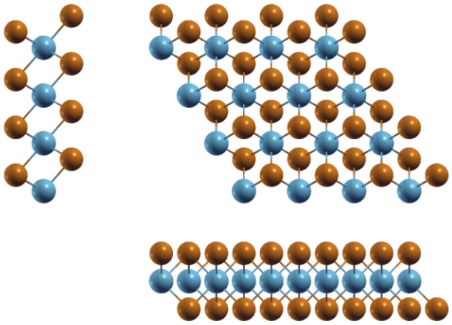

Orthographic projections: views of $NbTe_2$ (P-3m1) as seen from the x axis (left), the y axis (bottom) and the z axis (center).

Lattice matching - minimal size

Formula	N° atoms	strain	cell size 1	cell size 2
Tl	4	0.1168	1	1
Tl	4	0.2714	1	1
In	4	1.5628	1	1
HgO	5	0.1233	1	1
Bi_2	5	3.0203	1	1
As_2	5	0.0078	1	1
LiO	5	0.2533	1	1
P_2	5	0.2638	1	1
PbTe	5	2.9666	1	1
Sb_2	5	0.4688	1	1
Cl_2Zn	6	0.0088	1	1
I_2Mg	6	0.4833	1	1
S_2V	6	1.5862	1	1
MoS_2	6	1.5916	1	1
CdI_2	6	2.9894	1	1
$AgTe_2$	6	0.1182	1	1
PSn_2	6	0.001	1	1
$MoSe_2$	6	0.2703	1	1
Br_2Ca	6	3.0058	1	1
HfS_2	6	0.0051	1	1
$AsSn_2$	6	0.0048	1	1
$CuTe_2$	6	0.0055	1	1
S_2Zr	6	0.0002	1	1
Br_2La	6	0.4842	1	1
Br_2Co	6	0.0082	1	1
BiClTe	6	2.9942	1	1
Ca_2N	6	0.0073	1	1
Cl_2Ti	6	0.264	1	1
$AuTe_2$	6	0.4493	1	1
HgI_2	6	0.3295	1	1
Mg_3	6	0.1141	1	1
I_2Zn	6	0.4606	1	1
BaF_2	6	0.5004	1	1
$RhTe_2$	6	0.0077	1	1
S_2W	6	1.592	1	1
Bi_2Pd	6	0.1295	1	1
GeI_2	6	0.4785	1	1
NbS_2	6	0.2759	1	1
$CoTe_2$	6	0.0048	1	1
S_2Ta	6	0.2746	1	1
Se_2V	6	0.2726	1	1
AsKSn	6	0.4965	1	1
S_2Sn	6	0.0005	1	1
$SnTe_2$	6	0.4727	1	1
Cl_2V	6	0.2499	1	1
GeI_2	6	2.9697	1	1
STl_2	6	2.9056	1	1
$PtSe_2$	6	0.0054	1	1
Br_2Fe	6	0.0081	1	1
GeS_2	6	0.1526	1	1

Table showing the first 50 lattice matching combinations with the smallest number of atoms in the supercell.

Lattice matching - minimal strain

Formula	N° atoms	strain	cell size 1	cell size 2
S_2W	300	0.0	43	57
I_2Mg	390	0.0	73	57
Br_2HLa	499	0.0001	81	64
I_2La_2	388	0.0001	64	49
I_2Pr	255	0.0001	49	36
MoS_2	300	0.0001	43	57
MoS_2	300	0.0001	43	57
CS_2Ta_2	674	0.0001	73	91
$Ce_2I_2Si_2$	561	0.0002	73	57
BiClTe	255	0.0002	49	36
$Br_2Ho_2S_2$	624	0.0002	100	54
S_2Zr	6	0.0002	1	1
Cl_2Ti	492	0.0002	73	91
I_2Zn	543	0.0002	100	81
$Ag_2K_2Te_2$	237	0.0002	39	20
$CeLi_2P_2$	563	0.0003	81	64
P_2	401	0.0004	73	91
Br_2La	390	0.0004	73	57
N_2Re	294	0.0004	37	61
In_2Te_3	386	0.0004	57	43
AsKSn	339	0.0004	64	49
Gd_2GeI_2	327	0.0004	49	36
$Ge_2Mn_2Sr_2$	483	0.0004	65	48
$I_2S_2Yb_2$	279	0.0005	45	24
Bi_2Se_3	504	0.0005	73	57
S_2Sn	6	0.0005	1	1
As_2Ir_2	387	0.0005	65	48
Sb_2SeTe_2	386	0.0005	57	43
Cl_2V	339	0.0005	49	64
Sb_2Se_2Te	504	0.0005	73	57
$AsCuLi_2$	499	0.0005	81	64
Br_2La_2	565	0.0005	91	73
CdI_2	255	0.0005	49	36
FHOZn	403	0.0006	49	64
Se_4TiZr	12	0.0006	2	1
O_2Pt	255	0.0007	36	49
STl_2	300	0.0007	57	43
Br_2Ca	255	0.0007	49	36
$I_2Pr_2Si_2$	627	0.0007	81	64
Gd_2I_2	447	0.0007	73	57
CrO_2	75	0.0007	9	16
$Br_2Cr_2O_2$	462	0.0007	54	50
F_4Nb	759	0.0007	118	81
$CrSe_2$	339	0.0007	49	64
$Ho_2I_2S_2$	492	0.0007	80	42
Cu_2F_4	714	0.0007	130	54
Sb_2	419	0.0007	91	73
Ga_2Te_2	499	0.0008	81	64
HN_3OZn	765	0.0008	73	91
CrS_2	183	0.0008	25	36

Table showing the first 50 lattice matching combinations with the smallest strain needed to build the supercell.

$Nd_2O_2I_2$ (P4/nmm)

Structural and electronic properties

**Formula**	$Nd_2O_2I_2$
**Spacegroup**	P4/nmm
**Prototype**	PbClF
**Parent 3D**	$I_2Nd_2O_2$
**Source DB**	MPDS
**DB ID**	S1140364
**DF2-C09 Binding energy [meV/ Å^2]**	14.99
**RVV10 Binding energy [meV/ Å^2]**	N/A
**Band gap (PBE) [eV]**	0.0

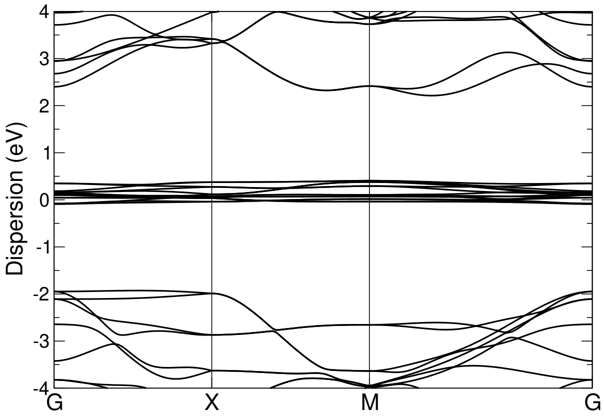

Band structure: Electronic band structure of $Nd_2O_2I_2$ (P4/nmm) in a representative window around the Fermi energy and along a high-symmetry path.

Optimized crystal structure

Structural parameters: relaxed cell (top) and atomic positions (bottom) of $Nd_2O_2I_2$ (P4/nmm) in Cartesian coordinates.

		x [Å]	y [Å]	z [Å]
$\mathbf{a_1}$		4.03461492	0.00000000	0.00000000
$\mathbf{a_2}$		0.00000000	4.03461492	0.00000000
$\mathbf{a_3}$		0.00000000	0.00000000	23.96913430
		x [Å]	y [Å]	z [Å]
	Nd	1.00865373	-1.00865373	1.15318201
	I	-1.00865373	-3.02596119	3.05704980
	Nd	-1.00865373	-3.02596119	-1.15318201
	I	1.00865373	-1.00865373	-3.05704980
	O	-1.00865373	-1.00865373	0.00000000
	O	1.00865373	-3.02596119	0.00000000

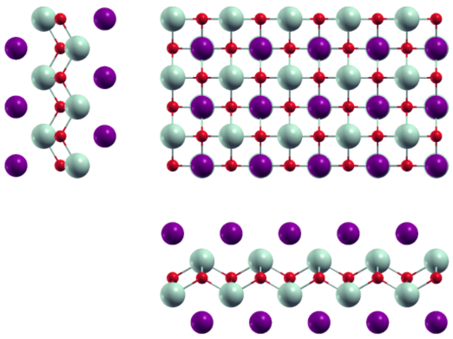

Orthographic projections: views of $Nd_2O_2I_2$ (P4/nmm) as seen from the x axis (left), the y axis (bottom) and the z axis (center).

Lattice matching - minimal size

Formula	N° atoms	strain	cell size 1	cell size 2
K	7	0.1659	1	1
Tl	7	0.1102	1	1
InSe	8	0.1329	1	1
Bi_2	8	0.1374	1	1
Ag_2	8	0.1722	1	1
P_2	8	0.1115	1	1
PbTe	8	0.1342	1	1
CaCl	8	0.2147	1	1
Cl_2Mn	9	0.109	1	1
CdI_2	9	0.1356	1	1
Nd	9	0.1793	1	3
$MoSe_2$	9	0.1104	1	1
Ba_2Pt	9	0.1719	1	1
Br_2Ca	9	0.1365	1	1
CaI_2	9	0.1562	1	1
Br_2Cu	9	0.1	1	1
Ca_2Si	9	0.1772	1	1
I_2Yb	9	0.1536	1	1
BiClTe	9	0.1359	1	1
Cl_2Ti	9	0.1114	1	1
HgI_2	9	0.381	1	1
NbS_2	9	0.1087	1	1
BaF_2	9	0.1301	1	1
BiBrTe	9	0.1404	1	1
NbS_2	9	0.1096	1	1
S_2Ta	9	0.1098	1	1
CKN	9	0.4843	1	1
Se_2V	9	0.11	1	1
Cl_2Cu	9	0.0965	1	1
I_2Tm	9	0.155	1	1
GeI_2	9	0.1344	1	1
I_2Pb	9	0.174	1	1
STl_2	9	0.1308	1	1
BiITe	9	0.1462	1	1
GeS_2	9	0.5863	1	1
$MnSe_2$	9	0.2147	1	1
DyI_2	9	0.1591	1	1
GdI_2	9	0.1429	1	1
CNNa	9	0.0713	1	1
CdI_2	9	0.1352	1	1
Sm	9	0.1605	1	3
I_2Pr	9	0.1359	1	1
Se_2W	9	0.1104	1	1
Bi_2Te_2	10	0.1933	1	1
Bi_2In_2	10	1.1225	1	1
Cu_2I_2	10	0.0068	1	1
Cu_2Sr_2	10	0.1413	1	1
Cl_2OOs	10	0.221	1	1
$LiMnTe_2$	10	0.1346	1	1
Cu_2Te_2	10	0.0579	1	1

Table showing the first 50 lattice matching combinations with the smallest number of atoms in the supercell.

Lattice matching - minimal strain

Formula	N° atoms	strain	cell size 1	cell size 2
HgI_2	852	0.0	101	82
Cl_2Rb_2	484	0.0001	64	25
Ca_2Cl_2	886	0.0002	81	100
$As_2Cd_2K_2$	870	0.0002	81	64
Mg_4	814	0.0002	65	106
$AgClO_4$	366	0.0003	36	25
Fe_2S_2	708	0.0003	64	81
CdClHO	548	0.0004	48	65
As_2Co_2	896	0.0004	82	101
ReS_2	237	0.0004	20	39
Br_2CsF	580	0.0004	64	49
$Ba_2H_2I_2$	690	0.0004	65	50
Ag_2I_2	924	0.0005	100	81
$RhTe_2$	483	0.0005	48	65
I_3Sn	796	0.0005	100	49
Tl	496	0.0006	65	106
HgI_2	843	0.0006	100	81
Mg_3	639	0.0006	58	97
Br_2Mn	840	0.0006	81	118
FKO_2Se	270	0.0007	30	18
KS_2Ti	958	0.0007	81	118
$Ag_2K_2Te_2$	876	0.0008	85	61
AlH_4Na	510	0.0009	49	36
$Cl_2F_2Pb_2$	12	0.0009	1	1
As_2Co_2	886	0.0009	81	100
$Bi_2I_2O_2$	12	0.0009	1	1
$Cu_2Na_2Se_2$	12	0.0009	1	1
$MnSe_2$	636	0.001	65	82
$Ba_2F_2I_2$	882	0.001	82	65
CaCl	554	0.001	65	82
$F_2I_2Tm_2$	12	0.0011	1	1
ClNZr	852	0.0011	79	126
O_4PSn	870	0.0012	81	64
Cl_2Zn	840	0.0013	81	118
O_4PTl	882	0.0013	82	65
GeS_2	849	0.0013	85	113
CoH_2O_2	315	0.0014	20	39
Tl	493	0.0014	64	109
O_2Sn_2	740	0.0014	66	86
$Se_2Si_2Zr_2$	690	0.0014	50	65
$Ba_2H_2I_2$	678	0.0015	64	49
$Hf_2Se_2Si_2$	678	0.0015	49	64
$Br_2N_2Zr_2$	750	0.0015	52	73
$I_2N_2Zr_2$	678	0.0016	48	65
Se_2W	711	0.0016	64	109
Se_2V	711	0.0016	64	109
H_2Si_2	680	0.0016	62	77
$H_2Na_2O_2$	876	0.0016	61	85
Ca_2Si	885	0.0016	103	89
$InSe_2$	603	0.0016	62	77

Table showing the first 50 lattice matching combinations with the smallest strain needed to build the supercell.

$Nd_2S_2I_2$ (P-3m1)

Structural and electronic properties

**Formula**	$Nd_2S_2I_2$
**Spacegroup**	P-3m1
**Prototype**	SmSI
**Parent 3D**	$I_2Nd_2S_2$
**Source DB**	MPDS
**DB ID**	S1703863
**DF2-C09 Binding energy [meV/ Å^2]**	10.95
**RVV10 Binding energy [meV/ Å^2]**	N/A
**Band gap (PBE) [eV]**	0.0

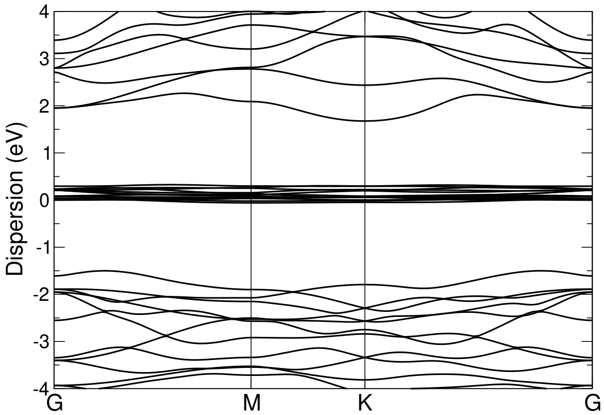

Band structure: Electronic band structure of $Nd_2S_2I_2$ (P-3m1) in a representative window around the Fermi energy and along a high-symmetry path.

Optimized crystal structure

Structural parameters: relaxed cell (top) and atomic positions (bottom) of $Nd_2S_2I_2$ (P-3m1) in Cartesian coordinates.

	x [Å]	y [Å]	z [Å]
$\mathbf{a_1}$	2.30398082	−3.99061185	0.00000000
$\mathbf{a_2}$	2.30398082	3.99061185	0.00000000
$\mathbf{a_3}$	0.00000000	0.00000000	26.93278978

		x [Å]	y [Å]	z [Å]
●	Nd	-1.15199041	-0.66510197	1.76441262
●	Nd	1.15199041	0.66510197	-1.76441262
●	S	-1.15199041	-0.66510197	-1.03971429
●	S	1.15199041	0.66510197	1.03971429
●	I	1.15199041	-1.99530592	3.75708236
●	I	1.15199041	-1.99530592	-3.75708236

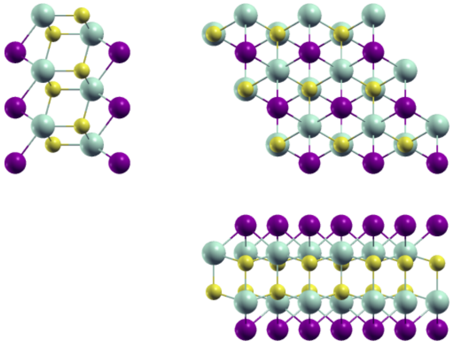

Orthographic projections: views of $Nd_2S_2I_2$ (P-3m1) as seen from the x axis (left), the y axis (bottom) and the z axis (center).

Lattice matching - minimal size

Formula	N° atoms	strain	cell size 1	cell size 2
K	7	0.1437	1	1
K	7	0.0033	1	1
AgTl	8	0.1223	1	1
Ag_2	8	0.0022	1	1
Sb_2	8	0.2606	1	1
I_2Mg	9	0.2686	1	1
Ba_2Pt	9	0.0019	1	1
I_2Pr	9	0.1133	1	1
Br_2La	9	0.2691	1	1
Ca_2Si	9	0.0065	1	1
$AuTe_2$	9	0.2499	1	1
BrCdI	9	0.2732	1	1
$PdTe_2$	9	0.2467	1	1
I_2Zn	9	0.2561	1	1
GeI_2	9	0.266	1	1
Ba_2Hg	9	0.1316	1	1
$PbTe_2$	9	0.2718	1	1
I_2Nd	9	0.1137	1	1
$SnTe_2$	9	0.2628	1	1
I_2Pb	9	0.0038	1	1
CeI_2	9	0.113	1	1
Se_2Yb	9	0.2663	1	1
$BiTe_2$	9	0.2667	1	1
$PtTe_2$	9	0.2494	1	1
I_2La	9	0.1158	1	1
F_2Zn	9	0.1119	1	1
Ba_2Cd	9	0.1335	1	1
Fe_2Te_2	10	0.431	1	1
Ca_2Cl_2	10	0.432	1	1
Cu_2I_2	10	0.1245	1	1
Cl_2Gd_2	10	1.5256	1	1
In_2Se_2	10	2.1443	1	1
Cu_2Te_2	10	0.1116	1	1
Ir_2P_2	10	0.1136	1	1
Ag_2Br_2	10	0.116	1	1
Br_2Er_2	10	0.2506	1	1
O_2Sn_2	10	0.1127	1	1
S_2Sn_2	10	0.1319	1	1
Cu_2S_2	10	0.4439	1	1
Au_2Br_2	10	0.1307	1	1
Br_2Tb_2	10	0.2475	1	1
Br_2Cu_2	10	0.1114	1	1
As_2Ir_2	10	0.1171	1	1
O_2Pb_2	10	0.1225	1	1
$AgBrO_2$	10	0.2944	1	1
Br_2Gd_2	10	1.6302	1	1
$MnNaTe_2$	10	0.2726	1	1
O_2Sn_2	10	0.111	1	1
$AsCuLi_2$	10	0.2653	1	1
Cu_2I_2	10	0.27	1	1

Table showing the first 50 lattice matching combinations with the smallest number of atoms in the supercell.

Lattice matching - minimal strain

Formula	N° atoms	strain	cell size 1	cell size 2
$ReSe_2$	102	0.0001	9	16
$PtTe_2$	486	0.0001	49	64
HN_3OZn	444	0.0001	25	49
Sb_2Se_2Te	986	0.0002	81	100
$CeLi_2P_2$	893	0.0002	73	91
Br_2HLa	802	0.0002	73	91
PTe_2Zr_2	330	0.0002	25	36
Sb_2Se_2Te	986	0.0003	81	100
$CrSe_2$	594	0.0004	49	100
Br_2Mn	405	0.0004	37	61
Br_2PY_2	543	0.0004	43	57
$C_2I_2La_2$	708	0.0004	55	63
$I_2Se_2Yb_2$	822	0.0004	77	60
I_2Zn	561	0.0004	57	73
AsSb	222	0.0005	25	36
$AuTe_2$	486	0.0005	49	64
$I_2Se_2Tm_2$	822	0.0005	77	60
Gd	18	0.0005	2	6
Te_2V	537	0.0005	49	81
MnO_2	129	0.0005	9	25
Br_2Cd	429	0.0006	43	57
$BiTe_2$	786	0.0006	81	100
$F_2Ho_2Se_2$	600	0.0006	43	57
I_3Sn	534	0.0007	65	36
In_2Se_2	634	0.0007	57	73
$AsCuLi_2$	802	0.0007	73	91
Br_2La_2	708	0.0007	64	81
I_2Mg	786	0.0007	81	100
Mo_2Te_4	264	0.0007	24	20
KNO_3	845	0.0007	100	49
Br_2V	102	0.0008	9	16
KS_2Ti	466	0.0008	37	61
CS_2Ta_2	395	0.0008	25	49
$Cl_2Hf_2N_2$	780	0.0008	49	81
OTl_2	537	0.0008	49	81
BH_4Li	984	0.0008	73	91
Ca_2O_2	822	0.0008	65	108
$I_2Pr_2Si_2$	984	0.0008	73	91
LiO	392	0.0009	39	79
$F_2Lu_2Se_2$	870	0.0009	64	81
Cl_2Ti	297	0.0009	25	49
Br_2Ho_2	550	0.0009	49	64
NaO_4	911	0.0009	91	73
CoI_2	258	0.0009	25	36
Se_2Yb	786	0.0009	81	100
Ge_2Te_2	526	0.0009	53	52
Sb_2	546	0.0009	64	81
Ga_2Te_2	802	0.0009	73	91
Ba_2Ni_3	893	0.001	73	91
Cl_2Ni	102	0.001	9	16

Table showing the first 50 lattice matching combinations with the smallest strain needed to build the supercell.

NdI_2 (P4/mmm)

Structural and electronic properties

**Formula**	NdI_2
**Spacegroup**	P4/mmm
**Prototype**	Zr2Cu
**Parent 3D**	NdI_2
**Source DB**	ICSD
**DB ID**	72190
**DF2-C09 Binding energy [meV/ Å^2]**	16.39
**RVV10 Binding energy [meV/ Å^2]**	21.88
**Band gap (PBE) [eV]**	N/A

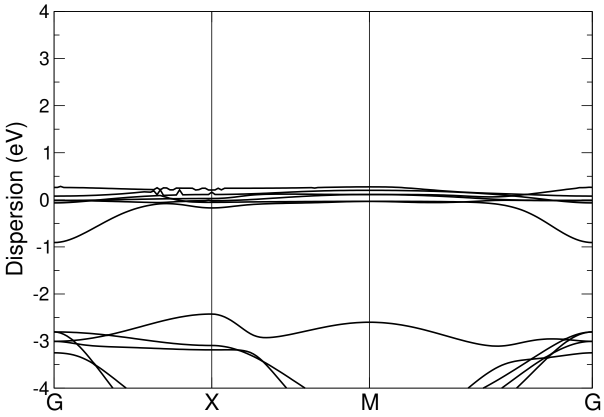

Band structure: Electronic band structure of NdI_2 (P4/mmm) in a representative window around the Fermi energy and along a high-symmetry path.

Optimized crystal structure

Structural parameters: relaxed cell (top) and atomic positions (bottom) of NdI_2 (P4/mmm) in Cartesian coordinates.

	x [Å]	y [Å]	z [Å]
$\mathbf{a_1}$	3.91402839	0.00000000	0.00000000
$\mathbf{a_2}$	0.00000000	3.91402839	0.00000000
$\mathbf{a_3}$	0.00000000	0.00000000	23.75933831

		x [Å]	y [Å]	z [Å]
●	I	1.95701420	1.95701420	10.01834663
●	I	1.95701420	1.95701420	13.74099168
●	Nd	0.00000000	3.91402839	11.87966916

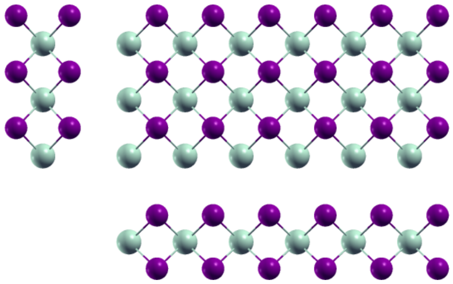

Orthographic projections: views of NdI_2 (P4/mmm) as seen from the x axis (left), the y axis (bottom) and the z axis (center).

Lattice matching - minimal size

Formula	N° atoms	strain	cell size 1	cell size 2
K	4	0.1828	1	1
InSe	5	0.1447	1	1
Bi_2	5	0.1501	1	1
AgTl	5	0.0215	1	1
Ag_2	5	0.1898	1	1
LiO	5	0.1099	1	1
PbTe	5	0.1463	1	1
Sb_2	5	0.1317	1	1
I_2Mg	6	0.136	1	1
MoS_2	6	0.1114	1	1
CdI_2	6	0.1479	1	1
Nd	6	0.7523	1	3
Ba_2Pt	6	0.1895	1	1
Br_2Ca	6	0.1491	1	1
CaI_2	6	0.1718	1	1
$AsSn_2$	6	0.1085	1	1
I_2Pr	6	0.001	1	1
Br_2La	6	0.1363	1	1
Br_2Cu	6	0.1051	1	1
Ca_2Si	6	0.1954	1	1
I_2Yb	6	0.1689	1	1
BiClTe	6	0.1483	1	1
BrCdI	6	0.1385	1	1
HgI_2	6	1.112	1	1
I_2Zn	6	0.1293	1	1
BaF_2	6	0.1413	1	1
BiBrTe	6	0.1536	1	1
S_2W	6	0.1114	1	1
GeI_2	6	0.1345	1	1
AsKSn	6	0.1401	1	1
$PbTe_2$	6	0.1377	1	1
Cl_2Cu	6	0.0983	1	1
I_2Tm	6	0.1704	1	1
$SnTe_2$	6	0.1328	1	1
Cl_2V	6	0.1105	1	1
GeI_2	6	0.1465	1	1
I_2Pb	6	0.1919	1	1
STl_2	6	0.1422	1	1
$PtSe_2$	6	0.1086	1	1
BiITe	6	0.1603	1	1
GeS_2	6	0.2173	1	1
DyI_2	6	0.1751	1	1
CeI_2	6	0.0017	1	1
Se_2Yb	6	0.1347	1	1
MoS_2	6	0.1114	1	1
$BiTe_2$	6	0.1349	1	1
GdI_2	6	0.1565	1	1
$CrSe_2$	6	0.1107	1	1
I_2La	6	0.0048	1	1
$CrSe_2$	6	0.1102	1	1

Table showing the first 50 lattice matching combinations with the smallest number of atoms in the supercell.

Lattice matching - minimal strain

Formula	N° atoms	strain	cell size 1	cell size 2
$Ag_2K_2Se_2$	495	0.0	65	50
$Br_2O_2Sm_2$	9	0.0001	1	1
$Cu_2K_2Te_2$	363	0.0001	49	36
$Cu_2Na_2Te_2$	786	0.0001	100	81
Ir_2P_2	7	0.0002	1	1
Ca_2N	339	0.0003	48	65
F_4Pb	563	0.0004	81	64
HgI_2	339	0.0004	64	49
$Br_2Hf_2N_2$	534	0.0004	48	65
Tl	403	0.0005	89	136
$Hf_2Se_2Si_2$	843	0.0005	81	100
H_2Li_2Pd	725	0.0005	65	106
$C_2Cl_2Y_2$	801	0.0006	79	94
Br_2CsF	499	0.0006	85	61
$AgClO_4$	942	0.0006	136	89
Bi_2Pd	438	0.0006	61	85
Mo_2Te_4	723	0.0006	101	70
$I_2O_2Yb_2$	9	0.0006	1	1
C_2Li_2	588	0.0006	84	84
BrNZr	597	0.0006	81	118
As_2O_3	649	0.0007	108	65
Cl_4Mn	233	0.0007	36	25
Ca_2O_2	523	0.0007	61	85
As_2	274	0.0008	48	65
GeS_2	978	0.0008	145	181
Cl_2Cu	588	0.0008	88	108
Mg_4	811	0.0008	89	136
$AgBrO_2$	717	0.0008	99	105
$AgBrO_2$	636	0.0008	88	93
CCl_2Lu_2	469	0.0009	48	65
$C_2Cl_2Y_2$	732	0.0009	72	86
O_4PTl	849	0.0009	113	85
$Ba_2F_2I_2$	849	0.001	113	85
I_2Pr	6	0.001	1	1
$Cl_2N_2Zr_2$	534	0.001	48	65
As_2Fe_2	643	0.001	81	100
O_2Sn_2	799	0.001	101	124
NS_2Ta	216	0.001	20	39
H_4Ti	725	0.0011	65	106
$I_2O_2Tm_2$	9	0.0011	1	1
$Ag_2K_2Se_2$	486	0.0011	64	49
Mg_3	123	0.0011	16	25
Br_2Fe	339	0.0011	48	65
$Cu_2Rb_2Te_2$	621	0.0011	85	61
Se_2V	615	0.0011	79	126
Br_2Co	339	0.0012	48	65
N_2W	177	0.0012	20	39
CaH_2O_2	469	0.0012	48	65
Cl_2Cu	315	0.0012	47	58
$NbSe_2$	597	0.0014	81	118

Table showing the first 50 lattice matching combinations with the smallest strain needed to build the supercell.

NdOBr (C2/m)

Structural and electronic properties

**Formula**	NdOBr
**Spacegroup**	C2/m
**Prototype**	PbClF
**Parent 3D**	$Nd_2O_2Br_2$
**Source DB**	COD
**DB ID**	9009172
**DF2-C09 Binding energy [meV/ Å^2]**	21.77
**RVV10 Binding energy [meV/ Å^2]**	29.82
**Band gap (PBE) [eV]**	0.0

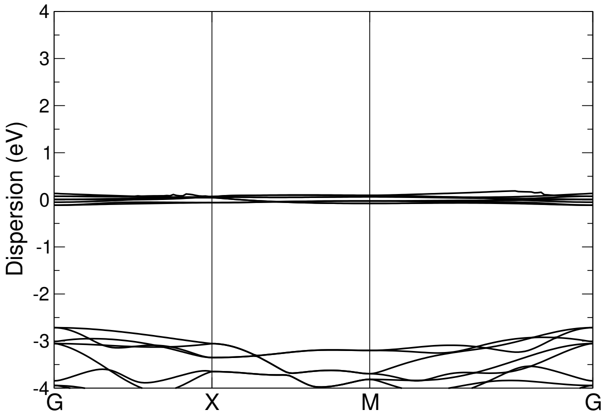

Band structure: Electronic band structure of NdOBr (C2/m) in a representative window around the Fermi energy and along a high-symmetry path.

Optimized crystal structure

Structural parameters: relaxed cell (top) and atomic positions (bottom) of NdOBr (C2/m) in Cartesian coordinates.

	x [Å]	y [Å]	z [Å]
$\mathbf{a_1}$	3.94164508	0.00000000	0.00000000
$\mathbf{a_2}$	0.00000000	3.94164508	0.00000000
$\mathbf{a_3}$	0.00000000	0.00000000	25.44806045

	x [Å]	y [Å]	z [Å]
Nd	0.00000000	1.97082254	13.92436886
Br	1.97082254	0.00000000	15.56932514
Nd	1.97082254	0.00000000	11.52369159
Br	0.00000000	1.97082254	9.87873531
O	0.00000000	0.00000000	12.72403023
O	1.97082254	1.97082254	12.72403023

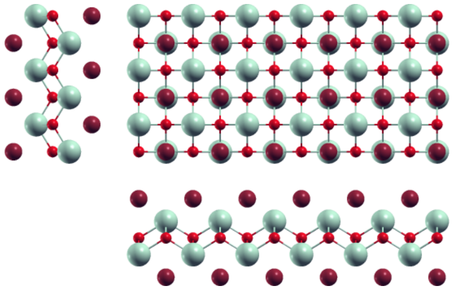

Orthographic projections: views of NdOBr (C2/m) as seen from the x axis (left), the y axis (bottom) and the z axis (center).

Lattice matching - minimal size

Formula	N° atoms	strain	cell size 1	cell size 2
K	7	0.1787	1	1
InSe	8	0.1418	1	1
Bi_2	8	0.147	1	1
AgTl	8	0.0188	1	1
Ag_2	8	0.1855	1	1
LiO	8	0.1107	1	1
P_2	8	0.109	1	1
PbTe	8	0.1433	1	1
Sb_2	8	0.1293	1	1
I_2Mg	9	0.1334	1	1
CdI_2	9	0.1449	1	1
Nd	9	0.7399	1	3
Ba_2Pt	9	0.1852	1	1
Br_2Ca	9	0.146	1	1
CaI_2	9	0.168	1	1
I_2Pr	9	0.0042	1	1
Br_2La	9	0.1337	1	1
Br_2Cu	9	0.1037	1	1
Ca_2Si	9	0.191	1	1
I_2Yb	9	0.1652	1	1
BiClTe	9	0.1452	1	1
Cl_2Ti	9	0.109	1	1
BrCdI	9	0.1359	1	1
HgI_2	9	0.4052	1	1
BaF_2	9	0.1385	1	1
BiBrTe	9	0.1504	1	1
GeI_2	9	0.132	1	1
AsKSn	9	0.1374	1	1
$PbTe_2$	9	0.1351	1	1
I_2Nd	9	0.0033	1	1
Cl_2Cu	9	0.0977	1	1
I_2Tm	9	0.1667	1	1
$SnTe_2$	9	0.1304	1	1
Cl_2V	9	0.1112	1	1
GeI_2	9	0.1435	1	1
I_2Pb	9	0.1875	1	1
STl_2	9	0.1393	1	1
BiITe	9	0.1569	1	1
GeS_2	9	0.2135	1	1
DyI_2	9	0.1712	1	1
CeI_2	9	0.005	1	1
Se_2Yb	9	0.1322	1	1
$BiTe_2$	9	0.1324	1	1
GdI_2	9	0.1532	1	1
I_2La	9	0.0014	1	1
$CrSe_2$	9	0.1109	1	1
CdI_2	9	0.1445	1	1
F_2Zn	9	0.0081	1	1
I_2Pr	9	0.1453	1	1
Bi_2Te_2	10	0.7817	1	1

Table showing the first 50 lattice matching combinations with the smallest number of atoms in the supercell.

Lattice matching - minimal strain

Formula	N° atoms	strain	cell size 1	cell size 2
GeS_2	636	0.0001	65	82
H_2Na_2Pd	789	0.0001	64	81
$I_2O_2Y_2$	12	0.0002	1	1
$Ba_2H_2I_2$	510	0.0002	49	36
Ga_2S_2	548	0.0003	48	65
H_2Li_2Pd	699	0.0004	49	81
Sn	70	0.0004	9	16
PTe_2Ti_2	613	0.0006	48	65
Cl_4KTl	204	0.0006	25	9
$CrTe_2$	840	0.0006	81	118
Cl_2Sc_2	958	0.0007	81	118
Br_2Ni	483	0.0007	48	65
GeS_2	627	0.0007	64	81
Ag_2I_2	590	0.0007	65	50
Cl_2Mg	483	0.0007	48	65
$AgTe_2$	942	0.0008	89	136
Se_2Ta_4	510	0.0008	36	49
$H_2I_2Yb_2$	12	0.0008	1	1
Br_2Cr	840	0.0008	81	118
As_2Fe_2	718	0.0008	65	82
Ga_2S_2	548	0.0009	48	65
H_4Ti	699	0.0009	49	81
FeH_2O_2	315	0.0009	20	39
Br_2Ti	840	0.001	81	118
$CoTe_2$	483	0.001	48	65
$Cl_2H_2Lu_2$	678	0.001	48	65
Br_2Cu	969	0.0011	103	117
$Bi_2Br_2O_2$	12	0.0011	1	1
Br_2Cu	894	0.0011	95	108
Mg_4	196	0.0012	16	25
Te_4W_2	654	0.0012	64	45
$Br_2N_2Ti_2$	78	0.0012	6	7
Mg_2	542	0.0012	58	97
Br_2Cu	819	0.0013	87	99
$Hf_2Se_2Si_2$	882	0.0013	65	82
HfS_2	483	0.0013	48	65
O_2Zn	237	0.0013	20	39
Br_2Cu	762	0.0014	81	92
Ho_2S_2	494	0.0014	49	50
I_2La	9	0.0014	1	1
AlH_4Na	366	0.0014	36	25
Br_2Cu	687	0.0015	73	83
Bi_2Se_4	282	0.0015	32	15
Tl	121	0.0015	16	25
$Er_2I_2O_2$	12	0.0015	1	1
Mg_2	456	0.0015	49	81
Cl_2Mn	852	0.0015	79	126
CdClO	531	0.0015	52	73
$Cl_2N_2Ti_2$	66	0.0015	5	6
$Br_2Eu_2F_2$	12	0.0015	1	1

Table showing the first 50 lattice matching combinations with the smallest strain needed to build the supercell.

$NiBr_2$ (P-3m1)

Structural and electronic properties

**Formula**	$NiBr_2$
**Spacegroup**	P-3m1
**Prototype**	CdI2
**Parent 3D**	$NiBr_2$
**Source DB**	COD
**DB ID**	9008013
**DF2-C09 Binding energy [meV/ Å^2]**	18.06
**RVV10 Binding energy [meV/ Å^2]**	24.35
**Band gap (PBE) [eV]**	0.75

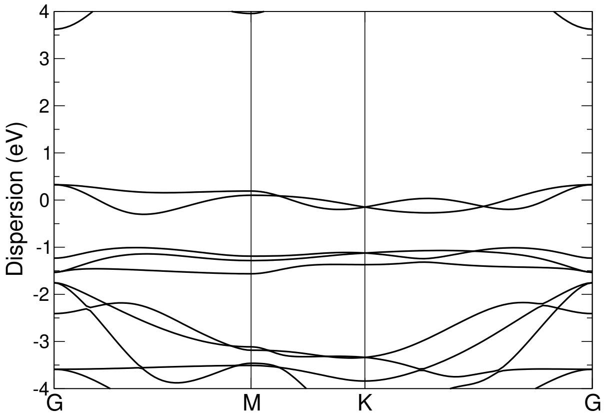

Band structure: Electronic band structure of $NiBr_2$ (P-3m1) in a representative window around the Fermi energy and along a high-symmetry path.

Optimized crystal structure

Structural parameters: relaxed cell (top) and atomic positions (bottom) of $NiBr_2$ (P-3m1) in Cartesian coordinates.

	x [Å]	y [Å]	z [Å]
$\mathbf{a_1}$	3.64491612	0.00000000	0.00000000
$\mathbf{a_2}$	−1.82245806	3.15658995	0.00000000
$\mathbf{a_3}$	0.00000000	0.00000000	22.83712824

		x [Å]	y [Å]	z [Å]
●	Br	1.82245806	1.05219665	10.02429647
●	Ni	-0.00000000	2.10439330	11.41856412
●	Br	1.82245806	3.15658995	12.81283177

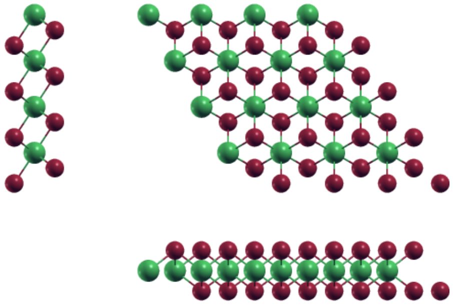

Orthographic projections: views of $NiBr_2$ (P-3m1) as seen from the x axis (left), the y axis (bottom) and the z axis (center).

Lattice matching - minimal size

Formula	N° atoms	strain	cell size 1	cell size 2
Tl	4	0.1184	1	1
In	4	0.4217	1	1
In	4	1.5864	1	1
InSe	5	2.9859	1	1
HgO	5	0.1253	1	1
As_2	5	0.0047	1	1
LiO	5	0.2577	1	1
P_2	5	0.2684	1	1
PbTe	5	3.0095	1	1
Mg_2	5	0.1114	1	1
Sb_2	5	0.477	1	1
Cl_2Zn	6	0.0057	1	1
I_2Mg	6	2.8518	1	1
S_2V	6	0.2478	1	1
MoS_2	6	0.2488	1	1
CdI_2	6	3.0327	1	1
$AgTe_2$	6	0.1199	1	1
PSn_2	6	0.0042	1	1
$MoSe_2$	6	0.275	1	1
HfS_2	6	0.002	1	1
$AsSn_2$	6	0.0081	1	1
Te_2V	6	0.0083	1	1
$CuTe_2$	6	0.0023	1	1
S_2Zr	6	0.0034	1	1
Br_2La	6	0.4927	1	1
Br_2Cu	6	1.023	1	1
Br_2Co	6	0.0051	1	1
BiClTe	6	3.0375	1	1
ReS_2	6	1.5233	1	1
Ca_2N	6	0.0041	1	1
Cl_2Ti	6	0.2686	1	1
$AuTe_2$	6	0.4572	1	1
BrCdI	6	0.5001	1	1
$PdTe_2$	6	0.4512	1	1
HgI_2	6	0.3363	1	1
Mg_3	6	0.1155	1	1
I_2Zn	6	0.4687	1	1
S_2W	6	0.2488	1	1
Bi_2Pd	6	0.1319	1	1
GeI_2	6	0.4869	1	1
Br_2Mn	6	0.0068	1	1
$CoTe_2$	6	0.0016	1	1
CdClO	6	0.0088	1	1
AsKSn	6	2.9164	1	1
$PbTe_2$	6	0.4975	1	1
S_2Sn	6	0.0037	1	1
$SnTe_2$	6	0.481	1	1
Cl_2V	6	0.2542	1	1
GeI_2	6	3.0126	1	1
$PtSe_2$	6	0.0088	1	1

Table showing the first 50 lattice matching combinations with the smallest number of atoms in the supercell.

Lattice matching - minimal strain

Formula	N° atoms	strain	cell size 1	cell size 2
CNb_2S_2	743	0.0	81	100
Cl_2Mg	6	0.0001	1	1
$CeLi_2P_2$	504	0.0001	73	57
Bi_2STe_2	327	0.0001	49	36
PTe_2Ti_2	8	0.0001	1	1
AsKSn	300	0.0002	57	43
Br_2La_2	499	0.0002	81	64
MoS_2	339	0.0002	49	64
$H_2I_2Yb_2$	483	0.0002	65	48
Br_2Ca_3Si	171	0.0003	25	16
S_2W	339	0.0003	49	64
$CrSe_2$	390	0.0003	57	73
I_2La_2Te	155	0.0003	25	16
$Cl_2H_2Lu_2$	9	0.0004	1	1
I_2La_2	343	0.0004	57	43
C_2F_2	271	0.0004	25	49
MoS_2	339	0.0004	49	64
Sb_2	371	0.0004	81	64
As_2Sn_2	624	0.0004	100	81
In_2Se_2	565	0.0004	91	73
Br_2Gd_2Ge	437	0.0004	64	49
Cu_2I_2	388	0.0004	64	49
Ga_2S_2	7	0.0005	1	1
N_3W_2	173	0.0005	16	25
Br_2HLa	447	0.0005	73	57
Br_2Gd_2	624	0.0005	100	81
$AsLi_3$	291	0.0005	49	36
P_2	443	0.0006	81	100
BH_4Li	561	0.0006	73	57
Cl_2La_2	624	0.0006	100	81
$Br_2H_2Sr_2$	840	0.0007	118	81
O_2Zn	183	0.0007	25	36
Cu_2I_2	678	0.0007	118	81
$AlLiTe_2$	208	0.0007	36	25
Br_2OV	546	0.0007	82	75
$Br_2Nd_2O_2$	483	0.0007	65	48
I_2Zn	492	0.0007	91	73
Cl_2Ti	543	0.0007	81	100
Gd_2I_2	388	0.0007	64	49
$PbTe_2$	339	0.0008	64	49
I_2La	339	0.0008	65	48
CS_2Ta_2	743	0.0008	81	100
$F_2Se_2Y_2$	102	0.0008	16	9
Cl_4Pd_2	78	0.0008	14	6
InSe	219	0.0008	49	36
In	186	0.0008	43	57
KNO_3	368	0.0008	81	25
As_2Li_2Pr	327	0.0008	49	36
$SnTe_2$	390	0.0008	73	57
$Ce_2I_2S_2$	405	0.0009	61	37

Table showing the first 50 lattice matching combinations with the smallest strain needed to build the supercell.

$NiCl_2$ (P-3m1)

Structural and electronic properties

**Formula**	$NiCl_2$
**Spacegroup**	P-3m1
**Prototype**	CdI2
**Parent 3D**	$NiCl_2$
**Source DB**	COD
**DB ID**	9009132
**DF2-C09 Binding energy [meV/ Å^2]**	16.34
**RVV10 Binding energy [meV/ Å^2]**	22.6
**Band gap (PBE) [eV]**	1.1

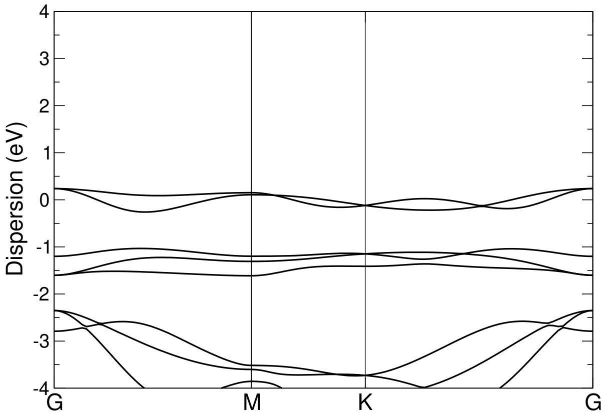

Band structure: Electronic band structure of $NiCl_2$ (P-3m1) in a representative window around the Fermi energy and along a high-symmetry path.

Optimized crystal structure

Structural parameters: relaxed cell (top) and atomic positions (bottom) of $NiCl_2$ (P-3m1) in Cartesian coordinates.

	x [Å]	y [Å]	z [Å]
$\mathbf{a_1}$	3.46298895	0.00000000	0.00000000
$\mathbf{a_2}$	−1.73149447	2.99903640	0.00000000
$\mathbf{a_3}$	0.00000000	0.00000000	22.64034135

		x [Å]	y [Å]	z [Å]
●	Ni	1.73149447	0.99967880	11.32017339
●	Cl	0.00000000	0.00000000	10.02437466
●	Cl	0.00000000	1.99935760	12.61596397

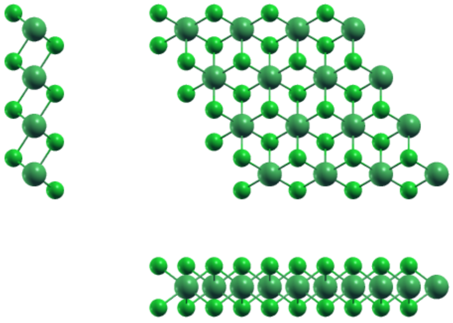

Orthographic projections: views of $NiCl_2$ (P-3m1) as seen from the x axis (left), the y axis (bottom) and the z axis (center).

Lattice matching - minimal size

Formula	N° atoms	strain	cell size 1	cell size 2
Tl	4	0.1338	1	1
Sn	4	0.1145	1	1
In	4	0.1165	1	1
HgO	5	0.1438	1	1
AsSb	5	0.4599	1	1
GeTe	5	0.4752	1	1
S_2	5	0.4792	1	1
Mg_2	5	0.1227	1	1
$IrTe_2$	6	0.4775	1	1
CrS_2	6	0.2561	1	1
$CdCl_2$	6	0.4725	1	1
$AgTe_2$	6	0.1361	1	1
$ReSe_2$	6	0.0009	1	1
$InSe_2$	6	0.4734	1	1
$GeTe_2$	6	0.47	1	1
$HfTe_2$	6	0.4994	1	1
I_2Mn	6	0.4728	1	1
NSr_2	6	0.464	1	1
I_2Yb	6	13.6747	1	1
PbS_2	6	0.4535	1	1
ReS_2	6	0.2645	1	1
$AuTe_2$	6	2.9904	1	1
FeI_2	6	0.4672	1	1
I_2Ni	6	0.4704	1	1
S_2Ti	6	0.0069	1	1
Mg_3	6	0.1294	1	1
CrI_2	6	0.4661	1	1
Bi_2Pd	6	0.1527	1	1
Ba_2Hg	6	0.3197	1	1
N_2W	6	0.2471	1	1
Cl_2Co	6	0.0072	1	1
$CrTe_2$	6	0.0062	1	1
Br_2V	6	0.0017	1	1
ClNZr	6	0.0038	1	1
Cl_2Fe	6	0.0079	1	1
Ba_2N	6	2.9084	1	1
Br_2Ti	6	0.0056	1	1
Te_2Zr	6	0.5007	1	1
$AsSe_2$	6	0.0015	1	1
I_2V	6	0.4482	1	1
CdO_2	6	0.0073	1	1
BrNZr	6	0.0028	1	1
$NbSe_2$	6	0.0004	1	1
CoI_2	6	0.461	1	1
Br_2Cr	6	0.0059	1	1
Cl_2Zr	6	0.0075	1	1
$FeSe_2$	6	0.112	1	1
Se_2Ta	6	0.0005	1	1
Br_2Mg	6	0.4669	1	1
I_2Ti	6	0.4619	1	1

Table showing the first 50 lattice matching combinations with the smallest number of atoms in the supercell.

Lattice matching - minimal strain

Formula	N° atoms	strain	cell size 1	cell size 2
$GeNi_3Te_2$	486	0.0	64	49
Br_2Mg	492	0.0	91	73
CoI_2	543	0.0001	100	81
Br_2Er_2	291	0.0001	49	36
In_2S_3	563	0.0001	81	64
Br_2La	183	0.0001	36	25
FeI_2	492	0.0001	91	73
ReS_2	492	0.0001	73	91
BiBrTe	123	0.0002	25	16
Bi_2Se_3	233	0.0002	36	25
S_2Zn_2	447	0.0002	73	57
I_2Mg	183	0.0002	36	25
CrI_2	492	0.0003	91	73
I_2Ti	543	0.0003	100	81
GeTe	371	0.0003	81	64
$F_2Se_2Y_2$	297	0.0003	49	25
NS_2Ta	357	0.0004	43	57
Gd_2I_2	208	0.0004	36	25
$NbSe_2$	6	0.0004	1	1
$F_2Se_2Yb_2$	561	0.0004	73	57
$Ce_2I_2Si_2$	258	0.0005	36	25
FeH_2O_2	467	0.0005	49	64
Ba_2N	300	0.0005	57	43
$AuTe_2$	255	0.0005	49	36
C_2Li_2	548	0.0005	96	65
CrS_2	390	0.0005	57	73
AsSb	462	0.0005	100	81
Se_2Ta	6	0.0005	1	1
P_2Sn_2	447	0.0006	73	57
$IrTe_2$	435	0.0006	81	64
NaPSn	339	0.0006	64	49
$HNiO_2$	304	0.0006	36	49
Br_2Gd_2	291	0.0006	49	36
$As_2Fe_2Li_2$	483	0.0006	65	48
$Br_2H_2Yb_2$	483	0.0007	65	48
$Bi_2Cl_2O_2$	840	0.0007	118	81
H_2Si_2	499	0.0007	81	64
Cl_2Gd_2	447	0.0007	73	57
CI_2Y_2	705	0.0007	100	81
Cu_3Se_3	429	0.0007	57	43
Cu_2I_2	208	0.0007	36	25
Br_2Gd_2Ge	233	0.0007	36	25
As_2Sn_2	291	0.0007	49	36
Pt_2Te_2	388	0.0008	64	49
Ga_2Se_2	499	0.0008	81	64
Sb_2Se_2Te	233	0.0008	36	25
$PtTe_2$	255	0.0009	49	36
Se_2Sn	543	0.0009	100	81
CoH_2O_2	674	0.0009	73	91
$ReSe_2$	6	0.0009	1	1

Table showing the first 50 lattice matching combinations with the smallest strain needed to build the supercell.

NiI_2 (P-3m1)

Structural and electronic properties

**Formula**	NiI_2
**Spacegroup**	P-3m1
**Prototype**	CdI2
**Parent 3D**	NiI_2
**Source DB**	COD
**DB ID**	9009133
**DF2-C09 Binding energy [meV/ Å^2]**	21.51
**RVV10 Binding energy [meV/ Å^2]**	26.86
**Band gap (PBE) [eV]**	0.32

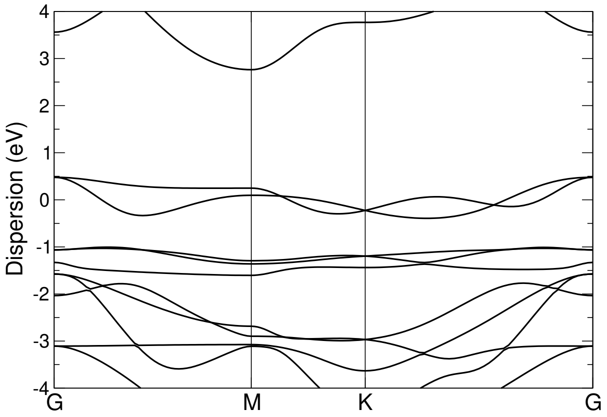

Band structure: Electronic band structure of NiI_2 (P-3m1) in a representative window around the Fermi energy and along a high-symmetry path.

Optimized crystal structure

Structural parameters: relaxed cell (top) and atomic positions (bottom) of NiI_2 (P-3m1) in Cartesian coordinates.

	x [Å]	y [Å]	z [Å]
$\mathbf{a_1}$	3.87785107	−0.00000000	0.00000000
$\mathbf{a_2}$	−1.93892554	3.35831754	0.00000000
$\mathbf{a_3}$	0.00000000	0.00000000	23.05482196

		x [Å]	y [Å]	z [Å]
●	I	1.93892554	1.11943918	10.02000308
●	I	1.93892554	3.35831754	13.03481888
●	Ni	0.00000000	2.23887836	11.52741098

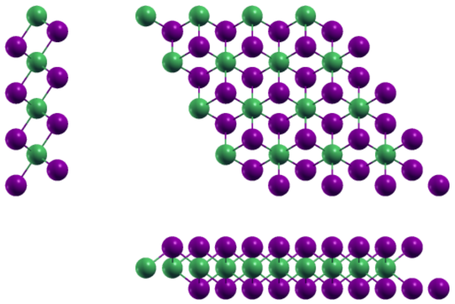

Orthographic projections: views of NiI_2 (P-3m1) as seen from the x axis (left), the y axis (bottom) and the z axis (center).

Lattice matching - minimal size

Formula	N° atoms	strain	cell size 1	cell size 2
Tl	4	1.544	1	1
HgO	5	0.1112	1	1
AsSb	5	0.0041	1	1
Bi_2	5	0.4581	1	1
GeTe	5	0.0019	1	1
S_2	5	0.0035	1	1
PbTe	5	0.4482	1	1
CaCl	5	0.1399	1	1
$IrTe_2$	6	0.0028	1	1
$CdCl_2$	6	0.0008	1	1
Cl_2Mn	6	1.5857	1	1
CdI_2	6	0.4524	1	1
$AgTe_2$	6	0.4255	1	1
$MoSe_2$	6	1.5387	1	1
$ReSe_2$	6	0.2611	1	1
S_2Ta	6	0.2462	1	1
Br_2Ca	6	0.4554	1	1
$InSe_2$	6	0.0012	1	1
$GeTe_2$	6	0.0002	1	1
I_2Mn	6	0.0009	1	1
NSr_2	6	0.0025	1	1
I_2Yb	6	2.9131	1	1
PbS_2	6	0.0067	1	1
BiClTe	6	0.4533	1	1
LiO_2	6	0.0667	1	1
Cl_2Zn	6	0.1532	1	1
FeI_2	6	0.0013	1	1
S_2Ti	6	1.6383	1	1
NbS_2	6	1.5977	1	1
CrI_2	6	0.0017	1	1
BiBrTe	6	0.4671	1	1
Bi_2Pd	6	0.1148	1	1
Cl_2Ni	6	0.2624	1	1
Cl_2Co	6	1.6357	1	1
$CrTe_2$	6	0.2711	1	1
Br_2V	6	0.26	1	1
ClNZr	6	0.2571	1	1
Cl_2Fe	6	1.6302	1	1
S_2Ta	6	1.5598	1	1
Se_2V	6	1.5502	1	1
Br_2Ti	6	0.2703	1	1
$AsSe_2$	6	0.2644	1	1
I_2Tm	6	2.9308	1	1
I_2V	6	0.0088	1	1
GeI_2	6	0.4488	1	1
BiITe	6	0.4838	1	1
BrNZr	6	0.2663	1	1
$NbSe_2$	6	0.263	1	1
CoI_2	6	0.0037	1	1
GeS_2	6	0.1305	1	1

Table showing the first 50 lattice matching combinations with the smallest number of atoms in the supercell.

Lattice matching - minimal strain

Formula	N° atoms	strain	cell size 1	cell size 2
As_2O_3	272	0.0	49	25
ReS_2	123	0.0	16	25
LiO	147	0.0	25	36
$AsSe_2$	492	0.0001	73	91
BiBrTe	492	0.0001	91	73
I_2Yb	300	0.0001	57	43
N_2W	294	0.0001	37	61
Bi_2Te_3	504	0.0002	73	57
$GeTe_2$	6	0.0002	1	1
BiITe	390	0.0002	73	57
Ba_2Cu_2	343	0.0003	57	43
$Ge_2I_2La_2$	486	0.0003	64	49
Cl_2Zr_2	516	0.0003	64	81
$Ho_2I_2Se_2$	345	0.0003	55	30
$NbSe_2$	492	0.0003	73	91
Tl	157	0.0004	36	49
Cl_2NSc_2	414	0.0004	43	57
$F_2Se_2Y_2$	171	0.0004	25	16
Se_2V	255	0.0005	36	49
Br_2V	435	0.0005	64	81
Sb_2Te_3	705	0.0005	100	81
Sb_2Te_2	208	0.0005	36	25
$I_2S_2Tb_2$	363	0.0006	49	36
Au_2I_2	548	0.0006	96	65
Ni_2Te_2	7	0.0006	1	1
GdI_2	435	0.0006	81	64
Bi_2SeTe_2	705	0.0007	100	81
$AgNO_3$	93	0.0007	16	9
CCl_2Sc_2	597	0.0007	64	81
NbS_2	300	0.0007	43	57
Br_3Cs	211	0.0007	49	16
N_3W_2	107	0.0008	9	16
Ba_2Cd	339	0.0008	65	48
Cl_2Cu	375	0.0008	61	64
$CdCl_2$	6	0.0008	1	1
$Br_2Er_2Se_2$	543	0.0008	85	48
I_2Pb	183	0.0009	36	25
Se_2Ta	492	0.0009	73	91
$Cu_2Na_2Te_2$	840	0.0009	118	81
BrNZr	543	0.0009	81	100
$AlLiTe_2$	447	0.0009	73	57
Cl_2Mn	300	0.0009	43	57
$F_2I_2Pb_2$	483	0.0009	65	48
I_2Mn	6	0.0009	1	1
$NbSe_2$	492	0.001	73	91
Br_2Hf_2	583	0.001	73	91
Se_2W	255	0.001	36	49
DyI_2	255	0.001	49	36
CoH_2O_2	173	0.001	16	25
GeI_2La_2	386	0.001	57	43

Table showing the first 50 lattice matching combinations with the smallest strain needed to build the supercell.

NiO_2 (P-3m1)

Structural and electronic properties

**Formula**	NiO_2
**Spacegroup**	P-3m1
**Prototype**	CdI2
**Parent 3D**	NiO_2
**Source DB**	COD
**DB ID**	1522025
**DF2-C09 Binding energy [meV/ Å^2]**	16.57
**RVV10 Binding energy [meV/ Å^2]**	27.64
**Band gap (PBE) [eV]**	1.32

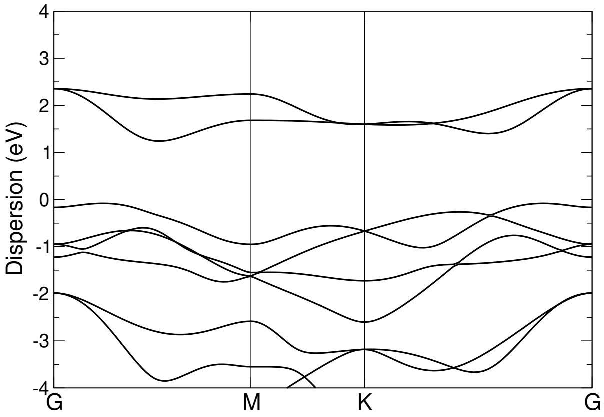

Band structure: Electronic band structure of NiO_2 (P-3m1) in a representative window around the Fermi energy and along a high-symmetry path.

Optimized crystal structure

Structural parameters: relaxed cell (top) and atomic positions (bottom) of NiO_2 (P-3m1) in Cartesian coordinates.

	x [Å]	y [Å]	z [Å]
$\mathbf{a_1}$	2.82420335	0.00000000	0.00000000
$\mathbf{a_2}$	−1.41210167	2.44583184	0.00000000
$\mathbf{a_3}$	0.00000000	0.00000000	21.88245306

	x [Å]	y [Å]	z [Å]
O	1.41210167	0.81527728	10.00062912
Ni	0.00000000	0.00000000	10.94122653
O	-0.00000000	1.63055456	11.88182393

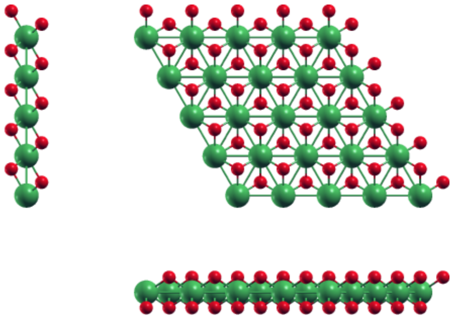

Orthographic projections: views of NiO_2 (P-3m1) as seen from the x axis (left), the y axis (bottom) and the z axis (center).

Lattice matching - minimal size

Formula	N° atoms	strain	cell size 1	cell size 2
In	4	0.2092	1	1
In	4	0.4696	1	1
LiO	5	0.497	1	1
BN	5	0.259	1	1
P_2	5	2.9764	1	1
C_2	5	0.2479	1	1
S_2V	6	0.4777	1	1
MoS_2	6	0.4796	1	1
$MoSe_2$	6	3.0369	1	1
HfS_2	6	13.5931	1	1
FeO_2	6	0.0013	1	1
ReS_2	6	0.4479	1	1
Cl_2Ti	6	2.9785	1	1
S_2W	6	0.4798	1	1
$CoTe_2$	6	13.6121	1	1
Cl_2V	6	2.8447	1	1
MoS_2	6	0.4802	1	1
$CrSe_2$	6	0.4872	1	1
$CrSe_2$	6	0.4936	1	1
O_2Pt	6	0.4657	1	1
N_2Re	6	0.0061	1	1
CoO_2	6	0.0005	1	1
Se_2W	6	3.0392	1	1
Ga_2S_2	7	13.6202	1	1
O_2Sn_2	7	1.3817	1	1
Ga_2S_2	7	13.6819	1	1
CS_2Ta_2	8	2.9796	1	1
H_2NiO_2	8	0.4832	1	1
H_2MgO_2	8	0.4755	1	1
CNb_2S_2	8	2.9697	1	1
$HfTe_2$	9	0.2186	2	1
Br_2Cu	9	5.2917	1	2
$PdTe_2$	9	0.2255	2	1
$H_2Na_2O_2$	9	0.3196	1	1
Se_2Ta_4	9	0.312	1	1
CNRb	9	0.2975	2	1
Ba_2N	9	0.2206	2	1
Te_2Zr	9	0.2192	2	1
CNNa	9	0.5611	2	1
Br_2Cd	9	0.2243	2	1
K	10	0.1228	3	1
$AgNO_2$	10	0.2166	2	1
Ho_2S_2	10	0.3017	2	1
Bi_2Te_2	11	6.1019	1	2
Bi_2Mn_2	11	1.8135	1	2
Br_2PY_2	11	0.224	2	1
In_2Se_3	11	0.2187	2	1
$C_2Br_2Gd_2$	12	0.9094	2	1
$Ag_2K_2Te_2$	12	0.5185	2	1
Cu_3Se_3	12	0.2202	2	1

Table showing the first 50 lattice matching combinations with the smallest number of atoms in the supercell.

Lattice matching - minimal strain

Formula	N° atoms	strain	cell size 1	cell size 2
H_2NiO_2	504	0.0	73	57
$I_2S_2Tb_2$	342	0.0	64	25
$CuTe_2$	294	0.0001	61	37
GeI_2Y_2	47	0.0001	9	4
Br_2Tb_2	393	0.0001	79	39
$CoTe_2$	390	0.0001	81	49
$AuTe_2$	447	0.0002	100	49
HfS_2	390	0.0002	81	49
H_2MgO_2	563	0.0002	81	64
$HfSe_2$	75	0.0002	16	9
BN	354	0.0002	64	81
$PtTe_2$	447	0.0002	100	49
Te_2Ti	75	0.0002	16	9
Ga_2S_2	439	0.0003	81	49
As_2Li_2Nd	47	0.0003	9	4
Bi_2S_3	432	0.0003	79	39
HN_3OZn	363	0.0004	49	36
CaH_2O_2	368	0.0004	61	37
Bi_2Se_2Te	47	0.0004	9	4
DyI_2	267	0.0004	64	25
Br_2Zr_2	139	0.0004	25	16
Bi_2O_2	606	0.0004	130	54
Mg_2	291	0.0004	65	48
HfS_2	294	0.0004	61	37
$GeNi_3Te_2$	297	0.0005	49	25
$C_2Br_2Gd_2$	759	0.0005	127	63
$Br_2O_2Ti_2$	360	0.0005	60	30
O_2Pt	492	0.0005	91	73
$PdTe_2$	354	0.0005	79	39
Br_2Zn	75	0.0005	16	9
CoO_2	6	0.0005	1	1
Cl_2Hf_2	208	0.0005	36	25
Br_2Ho_2	393	0.0006	79	39
LiO	290	0.0006	64	49
Ge_2S_2	528	0.0006	112	48
$Cl_2N_2Zr_2$	405	0.0006	61	37
$Al_2Cl_2O_2$	177	0.0006	27	16
CCl_2Gd_2	93	0.0006	16	9
Br_2Pr_2	84	0.0007	16	9
$CrSe_2$	339	0.0007	64	49
S_2V	435	0.0007	81	64
CNRb	453	0.0007	116	35
Br_2Er_2	496	0.0007	100	49
Sb_2SeTe_2	47	0.0007	9	4
$Cu_2Se_2Tl_2$	714	0.0008	130	54
$CoTe_2$	294	0.0008	61	37
Se_2Ti	123	0.0008	25	16
In_2Te_3	47	0.0008	9	4
Fe_2Se_2	197	0.0008	39	20
$C_2Br_2Tb_2$	759	0.0008	127	63

Table showing the first 50 lattice matching combinations with the smallest strain needed to build the supercell.

OH_3Cl (P3m1)

Structural and electronic properties

**Formula**	OH_3Cl
**Spacegroup**	P3m1
**Prototype**	KNO3
**Parent 3D**	ClH_3O
**Source DB**	MPDS
**DB ID**	S1010916
**DF2-C09 Binding energy [meV/ Å^2]**	7.54
**RVV10 Binding energy [meV/ Å^2]**	N/A
**Band gap (PBE) [eV]**	5.92

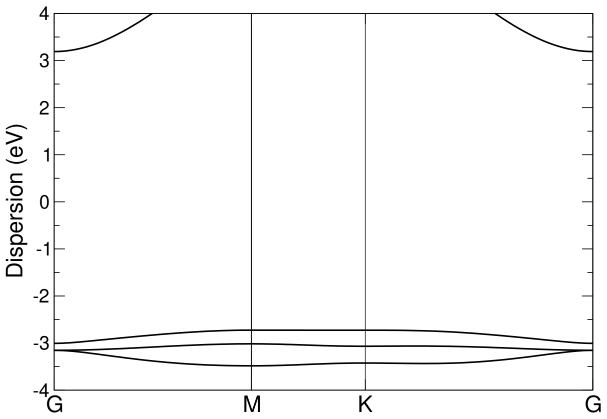

Band structure: Electronic band structure of OH_3Cl (P3m1) in a representative window around the Fermi energy and along a high-symmetry path.

Optimized crystal structure

Structural parameters: relaxed cell (top) and atomic positions (bottom) of OH_3Cl (P3m1) in Cartesian coordinates.

	x [Å]	y [Å]	z [Å]
$\mathbf{a_1}$	−2.38318667	−4.12780040	0.00000000
$\mathbf{a_2}$	4.76637334	0.00000000	0.00000000
$\mathbf{a_3}$	0.00000000	0.00000000	13.95200696

	x [Å]	y [Å]	z [Å]
H	1.54337301	-3.64293376	0.05895302
H	-1.54337301	-3.64293376	0.05895302
H	0.00000000	-0.96973328	0.05895302
Cl	0.00000000	-2.75186693	-0.59143213
O	0.00000000	0.00000000	0.41457306

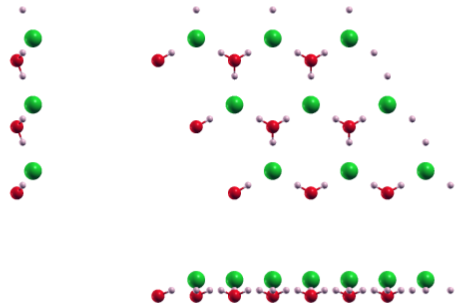

Orthographic projections: views of OH_3Cl (P3m1) as seen from the x axis (left), the y axis (bottom) and the z axis (center).

Lattice matching - minimal size

Formula	N° atoms	strain	cell size 1	cell size 2
K	6	0.1308	1	1
InSe	7	0.2606	1	1
Bi_2	7	0.2688	1	1
AgTl	7	0.1144	1	1
PbTe	7	0.2631	1	1
I_2Mg	8	0.2467	1	1
CdI_2	8	0.2655	1	1
Br_2Ca	8	0.2673	1	1
I_2Pr	8	0.4237	1	1
Br_2La	8	0.2472	1	1
BiClTe	8	0.266	1	1
BrCdI	8	1.6273	1	1
HgI_2	8	0.1448	1	1
BaF_2	8	0.2553	1	1
BiBrTe	8	0.2741	1	1
Ba_2Hg	8	0.1212	1	1
AsKSn	8	0.2534	1	1
$PbTe_2$	8	0.2495	1	1
I_2Nd	8	0.4259	1	1
Cl_2Cu	8	0.2901	1	1
GeI_2	8	0.2634	1	1
STl_2	8	0.2566	1	1
CeI_2	8	0.422	1	1
CdI_2	8	0.2649	1	1
Ba_2Cd	8	0.1227	1	1
I_2Pr	8	0.2661	1	1
Bi_2Te_2	9	0.0032	1	1
Cu_2I_2	9	0.1159	1	1
Cu_2Sr_2	9	0.2756	1	1
$LiMnTe_2$	9	0.2638	1	1
Ir_2P_2	9	0.4254	1	1
$AgCuTe_2$	9	0.1144	1	1
$AsLi_3$	9	0.261	1	1
S_2Sn_2	9	0.1215	1	1
Au_2Br_2	9	0.1196	1	1
Ge_2Te_2	9	0.132	1	1
As_2Ir_2	9	0.4434	1	1
O_2Pb_2	9	0.1144	1	1
$AgBrO_2$	9	0.2704	1	1
$MnNaTe_2$	9	0.2503	1	1
Cu_2I_2	9	0.248	1	1
Ge_2Se_2	9	0.4545	1	1
Bi_2O_2	9	0.115	1	1
$AgClO_2$	9	0.2521	1	1
La_2S_2	9	0.1304	1	1
PbS_2Sn	9	0.1239	1	1
Gd_2I_2	9	0.2476	1	1
Ni_2Se_2	9	0.4215	1	1
$SbSe_2Tl$	9	0.9565	1	1
Ag_2I_2	9	0.1457	1	1

Table showing the first 50 lattice matching combinations with the smallest number of atoms in the supercell.

Lattice matching - minimal strain

Formula	N° atoms	strain	cell size 1	cell size 2
Cl_2Fe	272	0.0	25	49
S_2Ta	545	0.0	49	100
$PtSe_2$	368	0.0	37	61
Bi_2STe_2	820	0.0	73	91
GeI_2Y_2	725	0.0	64	81
$AsSn_2$	488	0.0	49	81
Se_2Yb	386	0.0001	43	57
Cl_4Cu_2	315	0.0001	39	20
$Cl_2O_2Y_2$	230	0.0001	16	25
BaF_2	504	0.0001	57	73
N_2Re	120	0.0001	9	25
$Er_2I_2S_2$	651	0.0002	63	56
In_2Se_3	305	0.0002	25	36
CdClO	93	0.0002	9	16
$BiTe_2$	386	0.0002	43	57
$HfTe_2$	233	0.0002	25	36
$PbTe_2$	437	0.0002	49	64
Br_2Ca	705	0.0002	81	100
H_2MgO_2	65	0.0003	4	9
As_2Li_2Nd	725	0.0003	64	81
$LiOS_2Ti$	590	0.0003	39	79
Te_2Zr	233	0.0003	25	36
GeI_2	386	0.0003	43	57
$Ga_2I_2Y_2$	723	0.0003	57	73
$F_2Lu_2Se_2$	474	0.0003	36	49
$LiMnTe_2$	729	0.0003	73	91
OTl_2	93	0.0004	9	16
Cl_2Zr	272	0.0004	25	49
$Cl_2Hf_2N_2$	141	0.0004	9	16
CdI_2	638	0.0004	73	91
Bi_2Se_2Te	725	0.0004	64	81
$Er_2F_2Se_2$	341	0.0004	25	36
Ag_2F_4	970	0.0005	110	70
Ba_2Ni_3	500	0.0005	43	57
Ga_2Te_2	443	0.0005	43	57
Br_2La_2P	820	0.0005	73	91
Sb_2Se_2Te	500	0.0005	43	57
Br_3Cs	551	0.0006	79	39
CdO_2	272	0.0006	25	49
Ga_2Se_2	180	0.0006	16	25
$I_2Pr_2Si_2$	557	0.0006	43	57
$TaTe_2$	488	0.0006	49	81
$Cl_2H_2Zr_2$	669	0.0006	39	79
$Cl_2Ho_2O_2$	230	0.0006	16	25
S_2V	47	0.0006	4	9
CdH_2O_2	125	0.0006	9	16
Fe_2Se_2	877	0.0006	81	118
GeI_2	638	0.0007	73	91
$AsSn_2$	368	0.0007	37	61
$Al_2Cl_2O_2$	242	0.0007	16	27

Table showing the first 50 lattice matching combinations with the smallest strain needed to build the supercell.

OLuBr (P4/nmm)

Structural and electronic properties

**Formula**	OLuBr
**Spacegroup**	P4/nmm
**Prototype**	PbClF
**Parent 3D**	$O_2Lu_2Br_2$
**Source DB**	COD
**DB ID**	2214421
**DF2-C09 Binding energy [meV/ Å^2]**	15.35
**RVV10 Binding energy [meV/ Å^2]**	23.01
**Band gap (PBE) [eV]**	4.39

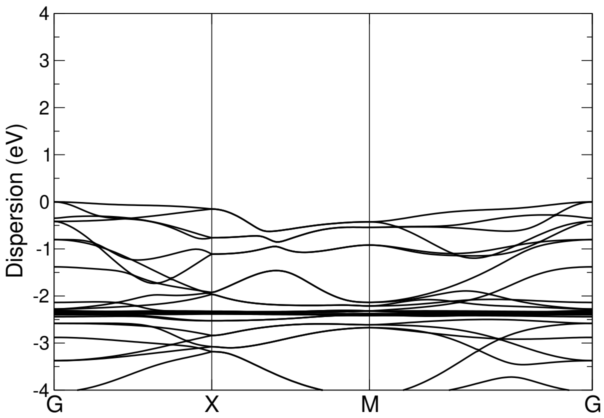

Band structure: Electronic band structure of OLuBr (P4/nmm) in a representative window around the Fermi energy and along a high-symmetry path.

Optimized crystal structure

Structural parameters: relaxed cell (top) and atomic positions (bottom) of OLuBr (P4/nmm) in Cartesian coordinates.

		x [Å]	y [Å]	z [Å]
$\mathbf{a_1}$		3.76350250	0.00000000	0.00000000
$\mathbf{a_2}$		0.00000000	3.76350250	0.00000000
$\mathbf{a_3}$		0.00000000	0.00000000	25.46999632
		x [Å]	y [Å]	z [Å]
●	Lu	1.88175125	0.00000000	13.86814878
●	Lu	0.00000000	1.88175125	11.60184754
●	Br	0.00000000	1.88175125	15.56073371
●	Br	1.88175125	0.00000000	9.90926261
•	O	0.00000000	0.00000000	12.73499816
•	O	1.88175125	1.88175125	12.73499816

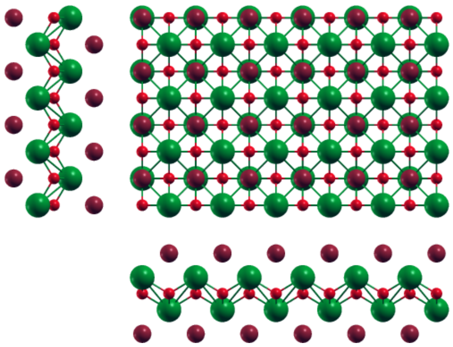

Orthographic projections: views of OLuBr (P4/nmm) as seen from the x axis (left), the y axis (bottom) and the z axis (center).

Lattice matching - minimal size

Formula	N° atoms	strain	cell size 1	cell size 2
K	7	1.1193	1	1
K	7	0.7793	1	1
InSe	8	0.1631	1	1
Bi_2	8	0.1695	1	1
AgTl	8	0.3639	1	1
PbTe	8	0.165	1	1
Sb_2	8	0.1471	1	1
CrS_2	9	0.1115	1	1
I_2Mg	9	0.1525	1	1
CdI_2	9	0.1669	1	1
$MoTe_2$	9	0.1085	1	1
Br_2Ca	9	0.1683	1	1
CaI_2	9	0.1949	1	1
$HfTe_2$	9	0.1338	1	1
Te_2V	9	0.109	1	1
Br_2La	9	0.1528	1	1
Br_2Cu	9	0.1151	1	1
I_2Yb	9	0.1915	1	1
BiClTe	9	0.1673	1	1
ReS_2	9	0.1101	1	1
$AuTe_2$	9	0.1401	1	1
BrCdI	9	0.1556	1	1
Cl_2Zn	9	0.0064	1	1
$PdTe_2$	9	0.1381	1	1
I_2Zn	9	0.1441	1	1
BaF_2	9	0.159	1	1
BiBrTe	9	0.1737	1	1
Bi_2Pd	9	0.2076	1	1
GeI_2	9	0.1507	1	1
Ba_2Hg	9	0.3826	1	1
CdClO	9	0.1088	1	1
Ba_2N	9	0.135	1	1
AsKSn	9	0.1575	1	1
Te_2Zr	9	0.1342	1	1
Te_2W	9	0.1085	1	1
$PbTe_2$	9	0.1546	1	1
Cl_2Cu	9	0.1038	1	1
I_2Tm	9	0.1933	1	1
$SnTe_2$	9	0.1485	1	1
GeI_2	9	0.1653	1	1
STl_2	9	0.16	1	1
OTl_2	9	0.1089	1	1
BiITe	9	0.1816	1	1
DyI_2	9	0.7549	1	1
Se_2Yb	9	0.1509	1	1
$BiTe_2$	9	0.1511	1	1
GdI_2	9	0.1771	1	1
$PtTe_2$	9	0.1398	1	1
Br_2Cd	9	0.1373	1	1
CdI_2	9	0.1665	1	1

Table showing the first 50 lattice matching combinations with the smallest number of atoms in the supercell.

Lattice matching - minimal strain

Formula	N° atoms	strain	cell size 1	cell size 2
$H_2I_2Sr_2$	690	0.0001	65	50
Ba_2Hg	843	0.0001	100	81
$NbSe_2$	483	0.0002	48	65
$Ge_2Te_2Zr_2$	12	0.0002	1	1
$AsSe_2$	483	0.0003	48	65
AuI_4Li	912	0.0003	105	47
$FeSe_2$	639	0.0005	58	97
La_2S_2	570	0.0005	63	48
$AgClO_4$	780	0.0005	81	49
$Ba_2F_2I_2$	366	0.0006	36	25
Cl_4KTl	390	0.0006	49	16
O_4PTl	366	0.0007	36	25
LiO_2	366	0.0007	36	50
Se_2Sn_2	514	0.0007	57	43
Br_2Hf_2	548	0.0007	48	65
$AgTe_2$	621	0.0007	61	85
Fe_2SeTe	10	0.0008	1	1
Sm	403	0.001	48	115
$H_2I_2Sr_2$	678	0.001	64	49
$C_2Br_2Y_2$	990	0.001	80	85
$Br_2H_2Yb_2$	12	0.001	1	1
Sn	496	0.001	65	106
$As_2Fe_2Li_2$	12	0.0011	1	1
$C_2Br_2La_2$	138	0.0011	12	11
AlH_4Na	246	0.0011	25	16
BrNZr	483	0.0011	48	65
$AgClO_2$	928	0.0011	92	94
Se_2Ta	483	0.0012	48	65
C_4Ca_2	870	0.0012	79	66
$CrSe_2$	852	0.0012	79	126
In	859	0.0012	113	181
$NbSe_2$	483	0.0013	48	65
FHOZn	978	0.0013	79	126
Gd	382	0.0013	46	106
NbS_2	840	0.0013	81	118
$C_2Br_2Y_2$	978	0.0013	79	84
$I_2Se_2Tb_2$	228	0.0014	24	14
$C_2Br_2Y_2$	756	0.0014	61	65
Cl_4Mn	230	0.0015	25	16
CoH_2O_2	929	0.0015	64	109
ReS_2	711	0.0015	64	109
$As_2Mg_2Na_2$	678	0.0015	64	49
S_2Ti	531	0.0015	52	73
$Br_2Cr_2S_2$	558	0.0015	48	45
Au_2I_2	968	0.0015	108	80
I_2La_2P	919	0.0015	89	77
Bi_2Pd	495	0.0016	50	65
GdI_2	885	0.0016	103	89
Cl_2Co	531	0.0016	52	73
HfS_2	603	0.0016	62	77

Table showing the first 50 lattice matching combinations with the smallest strain needed to build the supercell.

OLuI (P4/nmm)

Structural and electronic properties

**Formula**	OLuI
**Spacegroup**	P4/nmm
**Prototype**	PbClF
**Parent 3D**	$O_2Lu_2I_2$
**Source DB**	COD
**DB ID**	2216822
**DF2-C09 Binding energy [meV/ Å^2]**	15.54
**RVV10 Binding energy [meV/ Å^2]**	22.04
**Band gap (PBE) [eV]**	3.29

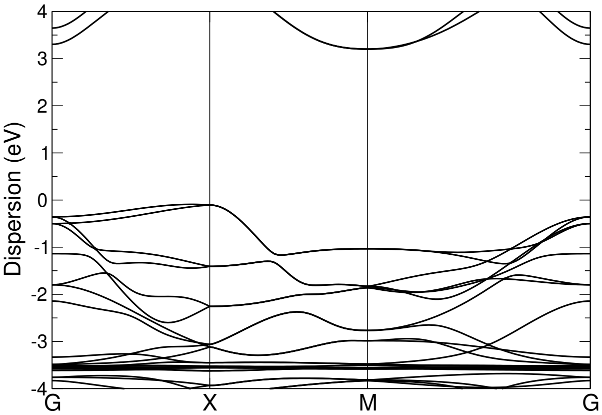

Band structure: Electronic band structure of OLuI (P4/nmm) in a representative window around the Fermi energy and along a high-symmetry path.

Optimized crystal structure

Structural parameters: relaxed cell (top) and atomic positions (bottom) of OLuI (P4/nmm) in Cartesian coordinates.

		x [Å]	y [Å]	z [Å]
$\mathbf{a_1}$		3.88102349	0.00000000	0.00000000
$\mathbf{a_2}$		0.00000000	3.88102349	0.00000000
$\mathbf{a_3}$		0.00000000	0.00000000	25.98722526
		x [Å]	y [Å]	z [Å]
●	Lu	1.94051174	0.00000000	14.06974504
●	Lu	0.00000000	1.94051174	11.91748021
●	I	0.00000000	1.94051174	15.98933563
●	I	1.94051174	0.00000000	9.99788963
•	O	0.00000000	0.00000000	12.99361263
•	O	1.94051174	1.94051174	12.99361263

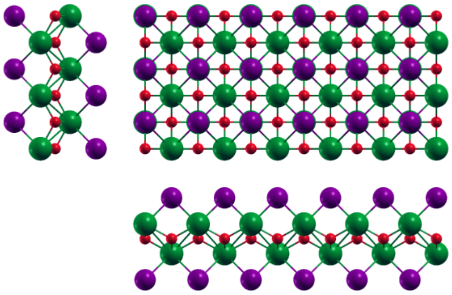

Orthographic projections: views of OLuI (P4/nmm) as seen from the x axis (left), the y axis (bottom) and the z axis (center).

Lattice matching - minimal size

Formula	N° atoms	strain	cell size 1	cell size 2
K	7	0.3836	1	1
K	7	0.1879	1	1
In	7	0.1114	1	1
InSe	8	0.1484	1	1
Bi_2	8	0.154	1	1
AgTl	8	0.0252	1	1
Ag_2	8	0.1951	1	1
LiO	8	0.1091	1	1
PbTe	8	0.1501	1	1
Sb_2	8	0.1347	1	1
I_2Mg	9	0.1393	1	1
S_2V	9	0.1107	1	1
MoS_2	9	0.1105	1	1
CdI_2	9	0.1517	1	1
Nd	9	0.7675	1	3
PSn_2	9	0.1085	1	1
Ba_2Pt	9	0.1947	1	1
Br_2Ca	9	0.1529	1	1
CaI_2	9	0.1765	1	1
I_2Pr	9	0.0031	1	1
Br_2La	9	0.1395	1	1
Br_2Cu	9	0.1069	1	1
I_2Yb	9	0.1735	1	1
BiClTe	9	0.1521	1	1
BrCdI	9	0.1419	1	1
HgI_2	9	1.1335	1	1
I_2Zn	9	0.1322	1	1
BaF_2	9	0.1449	1	1
BiBrTe	9	0.1577	1	1
S_2W	9	0.1105	1	1
Bi_2Pd	9	0.56	1	1
GeI_2	9	0.1377	1	1
AsKSn	9	0.1436	1	1
$PbTe_2$	9	0.1411	1	1
I_2Nd	9	0.004	1	1
Cl_2Cu	9	0.0992	1	1
I_2Tm	9	0.1751	1	1
S_2Sn	9	0.1084	1	1
$SnTe_2$	9	0.1359	1	1
Cl_2V	9	0.1096	1	1
GeI_2	9	0.1503	1	1
I_2Pb	9	0.1972	1	1
STl_2	9	0.1457	1	1
BiITe	9	0.1646	1	1
DyI_2	9	0.1799	1	1
CeI_2	9	0.0023	1	1
Se_2Yb	9	0.1379	1	1
MoS_2	9	0.1104	1	1
$BiTe_2$	9	0.1381	1	1
GdI_2	9	0.1607	1	1

Table showing the first 50 lattice matching combinations with the smallest number of atoms in the supercell.

Lattice matching - minimal strain

Formula	N° atoms	strain	cell size 1	cell size 2
$Br_2F_2Tm_2$	12	0.0001	1	1
$Ca_2Ge_2Mn_2$	12	0.0002	1	1
HgI_2	933	0.0003	113	85
$FeSe_2$	102	0.0004	9	16
$Br_2O_2V_2$	66	0.0005	5	6
Te_2V	483	0.0005	48	65
K	681	0.0006	100	81
$Ba_2F_2I_2$	510	0.0006	49	36
$ReSe_2$	840	0.0006	81	118
$Br_2Gd_2O_2$	12	0.0007	1	1
$AgClO_4$	246	0.0007	25	16
Pd_2S_4	708	0.0008	79	39
$Bi_2Cl_2O_2$	12	0.0008	1	1
Ca_2O_2	412	0.0008	36	49
H_2Na_2Pd	997	0.0008	82	101
$Cl_2Hf_2N_2$	678	0.0008	48	65
OTl_2	483	0.0008	48	65
Br_2V	840	0.0009	81	118
F_2Zn	9	0.0009	1	1
O_4PTl	510	0.001	49	36
$Cu_2Na_2Te_2$	882	0.001	82	65
Cl_2Rb_2	186	0.001	25	9
CdClO	483	0.001	48	65
Br_2Mn	483	0.001	48	65
$Br_2Cr_2S_2$	504	0.0011	42	42
Cl_4Cu_2	534	0.0012	64	25
Cl_2Ni	840	0.0012	81	118
Pb_2Se_2	292	0.0013	32	25
$Br_2Ca_2F_2$	12	0.0013	1	1
$Cl_2S_2Tl_2$	780	0.0013	81	49
H_2Na_2Pd	986	0.0014	81	100
$As_2Cd_2K_2$	510	0.0014	49	36
H_2NiO_2	929	0.0014	64	109
KS_2Ti	548	0.0014	48	65
CaI_2	885	0.0014	103	89
$Cu_2K_2Te_2$	876	0.0014	85	61
$C_2I_2Y_2$	864	0.0015	70	74
$Ba_2H_2I_2$	876	0.0015	85	61
$C_2I_2Y_2$	666	0.0015	54	57
$Cl_2O_2V_2$	54	0.0016	4	5
Cl_2Zr_2	958	0.0016	81	118
In	655	0.0016	85	145
Br_2Ca_3Si	198	0.0016	21	12
$NbSe_2$	840	0.0016	81	118
GeS_2	795	0.0016	82	101
CaI_2	765	0.0016	89	77
Se_2Ta_4	678	0.0016	49	64
CuO_2	678	0.0016	63	100
$H_2Na_2O_2$	690	0.0016	50	65
$AuTe_2$	975	0.0017	106	113

Table showing the first 50 lattice matching combinations with the smallest strain needed to build the supercell.

$OsOCl_2$ (Pmmm)

Structural and electronic properties

**Formula**	$OsOCl_2$
**Spacegroup**	Pmmm
**Prototype**	NbOI2
**Parent 3D**	$OsOCl_2$
**Source DB**	ICSD
**DB ID**	83884
**DF2-C09 Binding energy [meV/ Å^2]**	12.87
**RVV10 Binding energy [meV/ Å^2]**	21.99
**Band gap (PBE) [eV]**	N/A

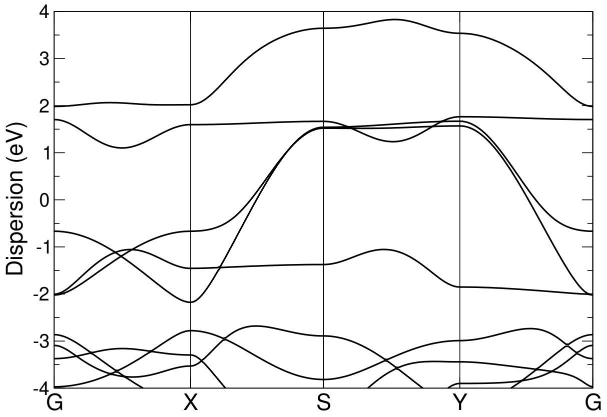

Band structure: Electronic band structure of $OsOCl_2$ (Pmmm) in a representative window around the Fermi energy and along a high-symmetry path.

Optimized crystal structure

Structural parameters: relaxed cell (top) and atomic positions (bottom) of $OsOCl_2$ (Pmmm) in Cartesian coordinates.

	x [Å]	y [Å]	z [Å]
$\mathbf{a_1}$	3.61860820	0.00000000	0.00000000
$\mathbf{a_2}$	0.00000000	3.71659461	0.00000000
$\mathbf{a_3}$	0.00000000	0.00000000	23.13966656

		x [Å]	y [Å]	z [Å]
●	Os	0.00000000	3.71659461	11.56983328
●	Cl	1.80930410	0.00000000	9.98501721
●	O	3.61860820	1.85829730	11.56983328
●	Cl	1.80930410	0.00000000	13.15464935

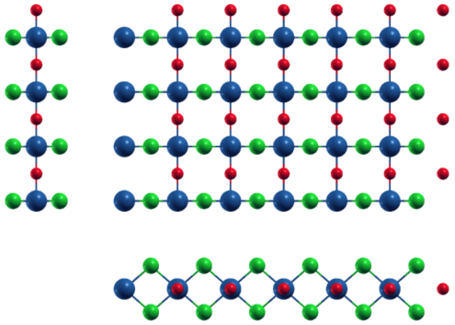

Orthographic projections: views of $OsOCl_2$ (Pmmm) as seen from the x axis (left), the y axis (bottom) and the z axis (center).

Lattice matching - minimal size

Formula	N° atoms	strain	cell size 1	cell size 2
K	5	1.2125	1	1
InSe	6	0.1773	1	1
HgO	6	0.2068	1	1
AsSb	6	0.1317	1	1
Bi_2	6	0.1845	1	1
GeTe	6	0.1364	1	1
AgTl	6	0.3959	1	1
S_2	6	0.1376	1	1
PbTe	6	0.1795	1	1
Sb_2	6	0.1594	1	1
CaCl	6	0.0104	1	1
$IrTe_2$	7	0.1371	1	1
I_2Mg	7	0.1655	1	1
$CdCl_2$	7	0.1355	1	1
CdI_2	7	0.1816	1	1
$AgTe_2$	7	0.5974	1	1
Br_2Ca	7	0.1831	1	1
CaI_2	7	0.2122	1	1
$InSe_2$	7	0.1358	1	1
$GeTe_2$	7	0.1347	1	1
$SiTe_2$	7	0.1274	1	1
$HfTe_2$	7	0.1442	1	1
I_2Mn	7	0.1356	1	1
Br_2La	7	0.1658	1	1
Br_2Cu	7	0.1277	1	1
NSr_2	7	0.1329	1	1
I_2Yb	7	0.2085	1	1
PbS_2	7	0.1299	1	1
BiClTe	7	0.182	1	1
$AuTe_2$	7	0.1514	1	1
BrCdI	7	0.169	1	1
LiO_2	7	0.3257	1	1
Cl_2Zn	7	0.0087	1	1
$PdTe_2$	7	0.1491	1	1
FeI_2	7	0.1339	1	1
I_2Ni	7	0.1349	1	1
Mg_3	7	0.5709	1	1
CrI_2	7	0.1335	1	1
I_2Zn	7	0.156	1	1
BaF_2	7	0.1728	1	1
BiBrTe	7	0.189	1	1
Bi_2Pd	7	0.2254	1	1
GeI_2	7	0.1634	1	1
Ba_2Hg	7	0.4168	1	1
N_2W	7	0.1103	1	1
Ba_2N	7	0.1456	1	1
AsKSn	7	0.1711	1	1
Te_2Zr	7	0.1446	1	1
$PbTe_2$	7	0.1679	1	1
$NiTe_2$	7	0.127	1	1

Table showing the first 50 lattice matching combinations with the smallest number of atoms in the supercell.

Lattice matching - minimal strain

Formula	N° atoms	strain	cell size 1	cell size 2
F_4Nb	644	0.0003	81	64
In_2Se_2	172	0.0005	27	16
Cl_2V	204	0.0005	24	36
$I_2S_2Yb_2$	614	0.0005	80	49
ClNZr	573	0.0007	72	95
$I_2Se_2Yb_2$	156	0.0007	21	12
NbS_2	599	0.0008	74	101
$Cl_2Fe_2O_2$	76	0.0008	7	8
$CrSe_2$	204	0.0008	24	36
$Dy_2I_2S_2$	532	0.0009	70	42
S_2Ta	599	0.0009	74	101
CCl_2Sc_2	845	0.0009	80	105
Se_2Sn_2	584	0.0009	85	61
$AgNO_3$	415	0.001	60	35
F_4Nb	635	0.001	80	63
$LiNbS_2$	700	0.001	74	101
AsSb	482	0.001	79	83
NaO_4	415	0.001	60	35
$I_2Se_2Tm_2$	156	0.001	21	12
PbS_2Sn	872	0.001	124	94
$Br_2N_2Zr_2$	94	0.001	10	9
CoI_2	565	0.0011	79	83
Bi_2O_2	724	0.0011	100	81
NSr_2	499	0.0011	70	73
$Br_2Er_2Se_2$	180	0.0011	24	14
$I_2S_2Tb_2$	532	0.0011	70	42
H_2Li_2Pt	380	0.0011	35	48
Cl_2Zr_2	740	0.0011	80	105
NS_2Ta	380	0.0011	35	60
Se_2Sn	499	0.0011	70	73
$Br_2Ho_2S_2$	696	0.0011	90	56
$I_2S_2Tm_2$	614	0.0012	80	49
$Br_2La_2O_2$	886	0.0012	100	81
AlH_4Na	618	0.0012	81	49
$Eu_2F_2I_2$	886	0.0012	100	81
I_2Ti	565	0.0012	79	83
$Cu_2Na_2Te_2$	348	0.0012	42	30
CI_2Y_2	645	0.0012	70	73
CrI_2	499	0.0012	70	73
$F_2Se_2Yb_2$	756	0.0013	75	76
$Br_2Ho_2S_2$	672	0.0013	87	54
PTe_2Zr_2	731	0.0013	79	83
$Br_2Er_2S_2$	696	0.0014	90	56
$HNiO_2$	628	0.0014	57	100
Cl_2NSc_2	801	0.0014	74	101
HgO	412	0.0014	63	80
Br_2Mg	499	0.0014	70	73
$I_2S_2Tm_2$	590	0.0014	77	47
$Cl_2Er_2S_2$	836	0.0014	107	68
$C_2Br_2Y_2$	996	0.0014	99	100

Table showing the first 50 lattice matching combinations with the smallest strain needed to build the supercell.

P (Pmna)

Structural and electronic properties

**Formula**	P
**Spacegroup**	Pmna
**Prototype**	P(black)
**Parent 3D**	P_4
**Source DB**	COD
**DB ID**	9012486
**DF2-C09 Binding energy [meV/ Å^2]**	38.43
**RVV10 Binding energy [meV/ Å^2]**	30.66
**Band gap (PBE) [eV]**	0.89

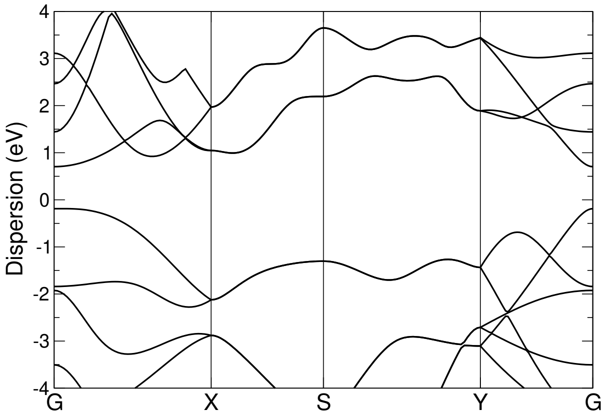

Band structure: Electronic band structure of P (Pmna) in a representative window around the Fermi energy and along a high-symmetry path.

Optimized crystal structure

Structural parameters: relaxed cell (top) and atomic positions (bottom) of P (Pmna) in Cartesian coordinates.

	x [Å]	y [Å]	z [Å]
$\mathbf{a_1}$	3.30546570	0.00000000	0.00000000
$\mathbf{a_2}$	0.00000000	4.61434267	0.00000000
$\mathbf{a_3}$	0.00000000	0.00000000	22.18331037

		x [Å]	y [Å]	z [Å]
●	P	1.65273285	4.20634859	10.03655911
●	P	1.65273285	0.40799408	12.14675126
●	P	3.30546570	2.71516516	10.03655911
●	P	3.30546570	1.89917751	12.14675127

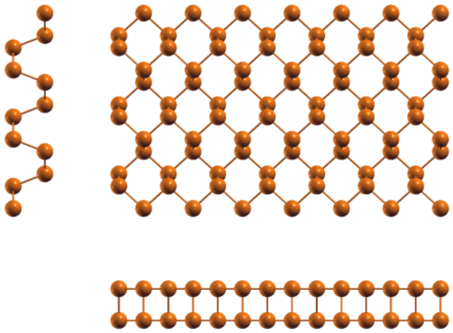

Orthographic projections: views of P (Pmna) as seen from the x axis (left), the y axis (bottom) and the z axis (center).

Lattice matching - minimal size

Formula	N° atoms	strain	cell size 1	cell size 2
HgO	6	0.2399	1	1
Sm	6	0.1314	1	2
I_2Pr	7	0.0812	1	1
Br_2Cu	7	0.7142	1	1
I_2Nd	7	0.0817	1	1
CeI_2	7	0.0808	1	1
F_2Zn	7	0.0793	1	1
Bi_2In_2	8	0.406	1	1
Nd	8	0.3659	1	4
Cu_2Te_2	8	0.3492	1	1
Ir_2P_2	8	0.0816	1	1
$AgNO_2$	8	0.0233	1	1
O_2Sn_2	8	0.1026	1	1
Ge_2Te_2	8	0.3179	1	1
Br_2Cu_2	8	0.0787	1	1
As_2Co_2	8	0.939	1	1
Ge_2S_2	8	0.2307	1	1
As_4	8	0.2435	1	1
C_2Li_2	8	0.1937	1	1
O_2Sn_2	8	0.0807	1	1
Ho_2S_2	8	1.2137	1	1
Ni_2Se_2	8	0.0807	1	1
$SbSe_2Tl$	8	2.3909	1	1
Co_2Se_2	8	0.9453	1	1
Ca_2Cl_2	8	0.9359	1	1
As_2O_3	9	0.3688	1	1
$CuGeO_3$	9	0.633	1	1
Cl_4Mn	9	0.419	1	1
$Br_2In_2O_2$	10	0.1135	1	1
$Er_2I_2O_2$	10	0.0827	1	1
$Cl_2O_2Sc_2$	10	0.3609	1	1
AlH_4Na	10	0.4252	1	1
$Br_2N_2Zr_2$	10	0.1147	1	1
$I_2N_2Zr_2$	10	0.1104	1	1
$Cl_2N_2Zr_2$	10	0.3928	1	1
$Br_2Cr_2S_2$	10	0.0066	1	1
$Br_2O_2Sc_2$	10	0.367	1	1
BrCdI	10	3.9674	1	2
$Ca_2Ge_2Mn_2$	10	0.0798	1	1
$Br_2F_2Tm_2$	10	0.0798	1	1
$I_2Lu_2O_2$	10	0.0797	1	1
$Ge_2Se_2Zr_2$	10	0.9359	1	1
Ba_2Hg	10	0.7567	1	2
$Br_2O_2Tb_2$	10	0.0786	1	1
$I_2O_2Yb_2$	10	0.0814	1	1
$Bi_2Cl_2O_2$	10	0.0801	1	1
$Cu_2Rb_2Te_2$	10	0.4052	1	1
$Br_2F_2Yb_2$	10	0.079	1	1
$Br_2Eu_2O_2$	10	0.0809	1	1
$Br_2O_2V_2$	10	0.3257	1	1

Table showing the first 50 lattice matching combinations with the smallest number of atoms in the supercell.

Lattice matching - minimal strain

Formula	N° atoms	strain	cell size 1	cell size 2
PtS_2	229	0.0005	28	39
$I_2S_2Sm_2$	978	0.0005	108	91
O_2Sn_2	668	0.0006	75	92
K	523	0.0007	108	91
$Br_2O_2Pr_2$	354	0.0007	36	35
O_2Sn_2	668	0.0007	75	92
O_2Sn_2	284	0.0007	35	36
O_2Sn_2	568	0.0007	70	72
CdClHO	572	0.0007	63	80
$Ga_2Ge_2Te_2$	964	0.0007	94	98
$SbSe_2Tl$	728	0.0007	108	74
Li_2P_2Pr	866	0.0008	94	98
Ag_2Br_2	284	0.0008	36	35
Bi_2STe_2	638	0.0008	72	70
NS_2Zr	268	0.0008	28	39
$AgBrO_2$	632	0.0008	77	81
$Ho_2I_2S_2$	876	0.0008	108	74
Cu_2S_2	284	0.0008	35	36
Ga_2S_3	307	0.0008	28	39
$Br_2O_2Y_2$	356	0.0008	35	36
I_3Sn	204	0.0008	35	16
$Br_2Dy_2O_2$	356	0.0009	35	36
$HfSe_2$	541	0.0009	70	87
Te_2Ti	541	0.0009	70	87
PbTe	428	0.0009	72	70
As_2CeLi_2	638	0.0009	72	70
As_2Rh_2	284	0.0009	36	35
Br_2CsF	48	0.0009	7	5
$Br_2Lu_2S_2$	402	0.0009	48	35
$Br_2In_2O_2$	976	0.0009	97	98
I_2La_2Sb	887	0.0009	108	91
Br_2Zn	541	0.0009	70	87
$RhTe_2$	492	0.0009	63	80
$HfLiS_2$	268	0.0009	28	39
BH_4Li	808	0.001	79	82
$Br_2Lu_2S_2$	402	0.001	48	35
$SnTe_2$	562	0.001	79	82
Sb_2	572	0.001	94	98
GeI_2	498	0.001	72	70
$Gd_2I_2Se_2$	730	0.001	94	59
NS_2Ta	520	0.0011	44	86
O_2Zn	465	0.0011	48	91
I_2La	249	0.0011	36	35
Bi_2O_2	936	0.0011	122	112
Na	151	0.0011	28	39
Ca_2Si	558	0.0011	87	70
$Cl_2Rh_2Te_2$	778	0.0011	100	63
$AsLi_3$	568	0.0012	72	70
$I_2Se_2Tb_2$	730	0.0012	94	59
F_2Tl_2	284	0.0012	35	36

Table showing the first 50 lattice matching combinations with the smallest strain needed to build the supercell.

P (P-3m1)

Structural and electronic properties

**Formula**	P
**Spacegroup**	P-3m1
**Prototype**	As
**Parent 3D**	P_2
**Source DB**	ICSD
**DB ID**	647901
**DF2-C09 Binding energy [meV/ Å^2]**	51.57
**RVV10 Binding energy [meV/ Å^2]**	20.63
**Band gap (PBE) [eV]**	1.91

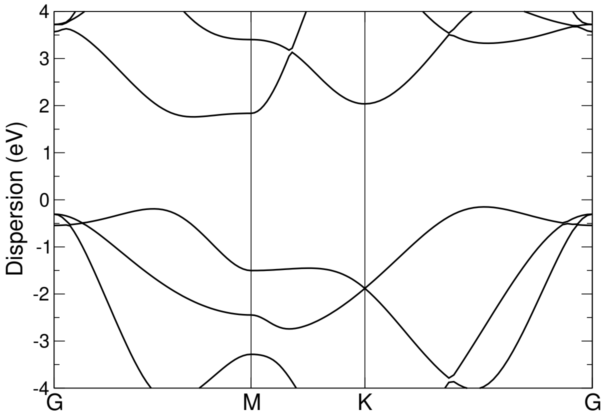

Band structure: Electronic band structure of P (P-3m1) in a representative window around the Fermi energy and along a high-symmetry path.

Optimized crystal structure

Structural parameters: relaxed cell (top) and atomic positions (bottom) of P (P-3m1) in Cartesian coordinates.

		x [Å]	y [Å]	z [Å]
$\mathbf{a_1}$		3.28427516	0.00000000	0.00000000
$\mathbf{a_2}$		−1.64213758	2.84426572	0.00000000
$\mathbf{a_3}$		0.00000000	0.00000000	21.25099823
		x [Å]	y [Å]	z [Å]
●	P	1.64213758	0.94808857	10.00621958
●	P	-0.00000000	1.89617715	11.24477865

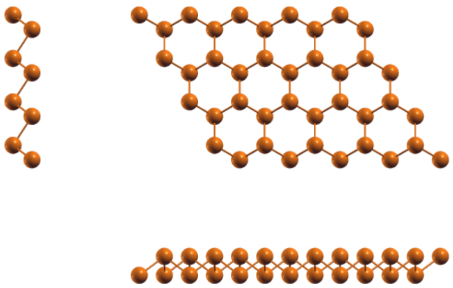

Orthographic projections: views of P (P-3m1) as seen from the x axis (left), the y axis (bottom) and the z axis (center).

Lattice matching - minimal size

Formula	N° atoms	strain	cell size 1	cell size 2
Tl	3	0.0053	1	1
Sn	3	0.1284	1	1
In	3	0.1316	1	1
As_2	4	0.4482	1	1
LiO	4	0.0075	1	1
Mg_2	4	0.1408	1	1
PSn_2	5	0.4703	1	1
$MoSe_2$	5	0.0045	1	1
Br_2Zn	5	0.4981	1	1
HfS_2	5	0.4549	1	1
$AsSn_2$	5	0.48	1	1
$SiTe_2$	5	2.9357	1	1
$CuTe_2$	5	0.454	1	1
S_2Zr	5	0.4684	1	1
NSr_2	5	3.0385	1	1
NiO_2	5	1.5579	1	1
Br_2Co	5	0.4471	1	1
Ca_2N	5	0.4495	1	1
Cl_2Ti	5	0.0002	1	1
Mg_3	5	0.1503	1	1
Te_2Ti	5	0.4989	1	1
$RhTe_2$	5	0.4873	1	1
NbS_2	5	0.0084	1	1
$CoTe_2$	5	0.4557	1	1
S_2Ta	5	0.0076	1	1
Se_2V	5	0.0062	1	1
$NiTe_2$	5	2.9279	1	1
S_2Sn	5	0.469	1	1
$PtSe_2$	5	0.4817	1	1
CoI_2	5	3.0224	1	1
Br_2Fe	5	0.4473	1	1
$TaTe_2$	5	0.4785	1	1
Br_2Ni	5	0.4598	1	1
$FeSe_2$	5	0.1242	1	1
$NbTe_2$	5	0.4679	1	1
Cl_2Mg	5	0.4599	1	1
I_2La	5	0.3164	1	1
$CrSe_2$	5	0.0087	1	1
N_2Re	5	0.2464	1	1
Se_2Sn	5	3.0353	1	1
CoO_2	5	1.554	1	1
$HfSe_2$	5	0.4989	1	1
Se_2W	5	0.0047	1	1
CdClHO	6	0.477	1	1
Br_2Pr_2	6	0.5013	1	1
$HNiO_2$	6	0.2714	1	1
Ag_2Br_2	6	0.3175	1	1
CdClHO	6	0.4853	1	1
Cl_2Y_2	6	0.4948	1	1
Ag_2	6	0.2202	2	1

Table showing the first 50 lattice matching combinations with the smallest number of atoms in the supercell.

Lattice matching - minimal strain

Formula	N° atoms	strain	cell size 1	cell size 2
$F_2Se_2Tm_2$	222	0.0	36	25
$LiMnSe_2$	172	0.0001	36	25
$AlLiTe_2$	68	0.0001	16	9
Cl_2Ti	5	0.0002	1	1
Sb_2	82	0.0002	25	16
$Cl_2H_2Lu_2$	686	0.0002	100	81
Cu_2O_4	342	0.0002	51	40
CS_2Ta_2	7	0.0002	1	1
FeO_2	219	0.0002	36	49
Cl_2Y_2	324	0.0002	64	49
STl_2	233	0.0002	61	37
KNO_3	13	0.0003	4	1
$Cl_2O_2Tm_2$	372	0.0003	57	43
$Ce_2I_2S_2$	392	0.0003	79	39
Ca_2Si	275	0.0003	79	39
CBr_2Y_2	373	0.0003	64	49
$NbTe_2$	401	0.0004	91	73
Br_2La_2	114	0.0004	25	16
Li_2P_2Pr	130	0.0004	25	16
CdClHO	418	0.0004	81	64
$Hf_2I_2N_2$	546	0.0004	81	64
CaClHO	286	0.0004	57	43
Cl_2Mg	443	0.0005	100	81
CNb_2S_2	7	0.0005	1	1
$Ga_2Ge_2Te_2$	146	0.0005	25	16
F_2Na	243	0.0005	57	43
C_2	50	0.0005	9	16
Br_2Ni	443	0.0006	100	81
$Cl_2O_2Yb_2$	372	0.0006	57	43
S_2Zr	401	0.0006	91	73
$PtSe_2$	317	0.0006	73	57
Au_2Br_2	474	0.0007	117	60
PTe_2Ti_2	605	0.0007	100	81
BaF_2	233	0.0007	61	37
In_2Te_3	407	0.0007	81	49
I_2Y_2	172	0.0007	36	25
$Cl_2Hg_2N_2$	294	0.0007	75	24
PTe_2Zr_2	278	0.0008	49	36
Sb_2SeTe_2	407	0.0008	81	49
Cu_2Se_2	560	0.0008	118	81
CdClHO	374	0.0008	73	57
CBr_2Lu_2	547	0.0008	91	73
S_2Sn	401	0.0009	91	73
CCl_2Gd_2	373	0.0009	64	49
$Br_2N_2Zr_2$	620	0.0009	91	73
$Br_2In_2O_2$	470	0.0009	82	51
$Ga_2I_2Y_2$	344	0.0009	61	37
Ga_2Se_2	324	0.001	64	49
$Br_2Hf_2N_2$	568	0.001	98	62
$I_2S_2Tl_2$	722	0.001	118	81

Table showing the first 50 lattice matching combinations with the smallest strain needed to build the supercell.

PbBrF (P4/nmm)

Structural and electronic properties

**Formula**	PbBrF
**Spacegroup**	P4/nmm
**Prototype**	PbClF
**Parent 3D**	$Pb_2Br_2F_2$
**Source DB**	COD
**DB ID**	9009175
**DF2-C09 Binding energy [meV/ Å^2]**	23.68
**RVV10 Binding energy [meV/ Å^2]**	30.51
**Band gap (PBE) [eV]**	3.03

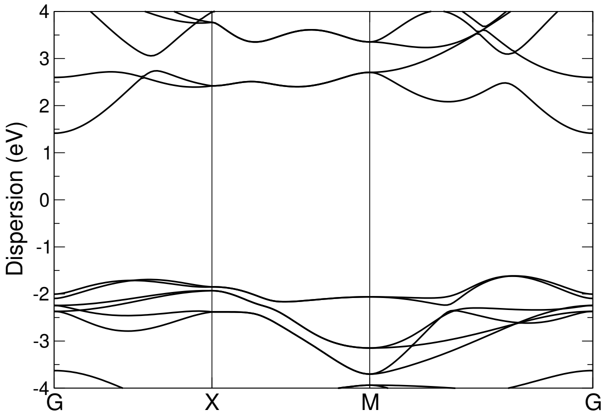

Band structure: Electronic band structure of PbBrF (P4/nmm) in a representative window around the Fermi energy and along a high-symmetry path.

Optimized crystal structure

Structural parameters: relaxed cell (top) and atomic positions (bottom) of PbBrF (P4/nmm) in Cartesian coordinates.

		x [Å]	y [Å]	z [Å]
$\mathbf{a_1}$		4.10708954	0.00000000	0.00000000
$\mathbf{a_2}$		0.00000000	4.10708954	0.00000000
$\mathbf{a_3}$		0.00000000	0.00000000	25.40318847
		x [Å]	y [Å]	z [Å]
●	Pb	0.00000000	2.05354477	14.23597005
●	Br	2.05354477	0.00000000	15.61705627
●	Pb	2.05354477	0.00000000	11.16721842
●	Br	0.00000000	2.05354477	9.78613220
●	F	0.00000000	0.00000000	12.70159423
●	F	2.05354477	2.05354477	12.70159423

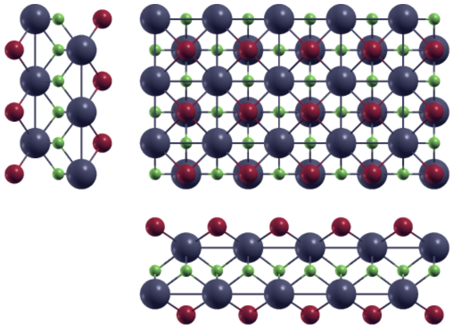

Orthographic projections: views of PbBrF (P4/nmm) as seen from the x axis (left), the y axis (bottom) and the z axis (center).

Lattice matching - minimal size

Formula	N° atoms	strain	cell size 1	cell size 2
K	7	0.157	1	1
Bi_2	8	0.131	1	1
GeTe	8	0.1086	1	1
AgTl	8	0.0136	1	1
Ag_2	8	0.1627	1	1
CaCl	8	0.5975	1	1
$IrTe_2$	9	0.1088	1	1
Cl_2Mn	9	0.1108	1	1
CdI_2	9	0.1293	1	1
Ba_2Pt	9	0.1625	1	1
S_2Ta	9	0.1104	1	1
Br_2Ca	9	0.1302	1	1
CaI_2	9	0.148	1	1
$InSe_2$	9	0.1084	1	1
Br_2Cu	9	0.098	1	1
Ca_2Si	9	0.1674	1	1
I_2Yb	9	0.1456	1	1
BiClTe	9	0.1296	1	1
S_2Ti	9	0.1093	1	1
NbS_2	9	0.1105	1	1
BiBrTe	9	0.1336	1	1
Ba_2Hg	9	0.0088	1	1
Cl_2Co	9	0.1094	1	1
NbS_2	9	0.1115	1	1
Cl_2Fe	9	0.1095	1	1
I_2Tm	9	0.1469	1	1
I_2Pb	9	0.1645	1	1
BiITe	9	0.1388	1	1
GeS_2	9	0.5616	1	1
$MnSe_2$	9	0.5972	1	1
DyI_2	9	0.1506	1	1
Cl_2Zr	9	0.1094	1	1
GdI_2	9	0.1358	1	1
CNNa	9	0.0673	1	1
F_2Ni	9	0.2171	1	1
I_2Pr	9	0.1296	1	1
H_2Si_2	10	0.1085	1	1
Bi_2Te_2	10	0.1825	1	1
Bi_2In_2	10	0.3982	1	1
Cu_2I_2	10	0.0017	1	1
Cu_2Sr_2	10	0.1344	1	1
Cl_2OOs	10	0.2114	1	1
Bi_2Mn_2	10	0.1687	1	1
$AgCuTe_2$	10	0.1938	1	1
O_2Sn_2	10	0.0344	1	1
S_2Sn_2	10	0.0165	1	1
$AlLiTe_2$	10	0.1379	1	1
Fe_2Se_2	10	0.2164	1	1
Cl_2ORu	10	0.2134	1	1
As_2Co_2	10	0.6128	1	1

Table showing the first 50 lattice matching combinations with the smallest number of atoms in the supercell.

Lattice matching - minimal strain

Formula	N° atoms	strain	cell size 1	cell size 2
In	159	0.0002	20	39
Br_2CsF	752	0.0002	82	65
$Hf_2Se_2Si_2$	510	0.0002	36	49
Co_2Se_2	708	0.0003	64	81
$Br_2F_2Sr_2$	12	0.0003	1	1
CaCl	430	0.0003	50	65
$SiTe_2$	483	0.0003	48	65
$Hf_2Si_2Te_2$	870	0.0003	64	81
$MnSe_2$	495	0.0004	50	65
Co_2S_2	560	0.0004	50	65
Bi_2Mn_2	58	0.0004	5	7
Hf_3Te_2	997	0.0004	82	101
$AgClO_4$	876	0.0004	85	61
Cu_2F_4	930	0.0004	97	58
Cl_4Mn	629	0.0004	64	49
$NiTe_2$	483	0.0004	48	65
Cl_4KTl	534	0.0004	64	25
$Cl_2Er_2O_2$	678	0.0005	48	65
Br_2CsF	742	0.0006	81	64
Se_2Zr	483	0.0007	48	65
As_2Fe_2	412	0.0007	36	49
$MnSe_2$	486	0.0007	49	64
CaCl	422	0.0008	49	64
$Fe_2Li_2P_2$	882	0.0009	65	82
Hf_3Te_2	986	0.0009	81	100
CaClHO	548	0.001	48	65
$Cu_2Rb_2Te_2$	870	0.0011	81	64
$Cl_2O_2Tm_2$	678	0.0011	48	65
$AgTe_2$	639	0.0011	58	97
Se_2Ta	852	0.0011	79	126
Mg_6	780	0.0012	49	81
Fe_2S_2	550	0.0012	49	64
Br_2OV	942	0.0013	83	111
H_4Ti	134	0.0013	9	16
$Br_2H_2Sr_2$	12	0.0013	1	1
Bi_2In_2	742	0.0014	81	64
Cl_2Zn	795	0.0014	82	101
Cu_2Se_2	886	0.0015	81	100
As_4	702	0.0015	71	69
$C_2Cl_2Y_2$	696	0.0015	50	66
Cr_2O_4	618	0.0015	40	63
Co_2S_2	550	0.0015	49	64
CdClHO	604	0.0015	52	73
Bi_2Pd	942	0.0015	89	136
GeS_2	363	0.0015	36	49
$LiNbS_2$	820	0.0015	64	109
O_2Pt	237	0.0016	20	39
Cl_2Mg	840	0.0016	81	118
S_2Ta	711	0.0016	64	109
I_2V	483	0.0016	48	65

Table showing the first 50 lattice matching combinations with the smallest strain needed to build the supercell.

PbClF (P4/nmm)

Structural and electronic properties

**Formula**	PbClF
**Spacegroup**	P4/nmm
**Prototype**	PbClF
**Parent 3D**	$Pb_2Cl_2F_2$
**Source DB**	COD
**DB ID**	1011046
**DF2-C09 Binding energy [meV/ Å^2]**	29.74
**RVV10 Binding energy [meV/ Å^2]**	37.34
**Band gap (PBE) [eV]**	3.52

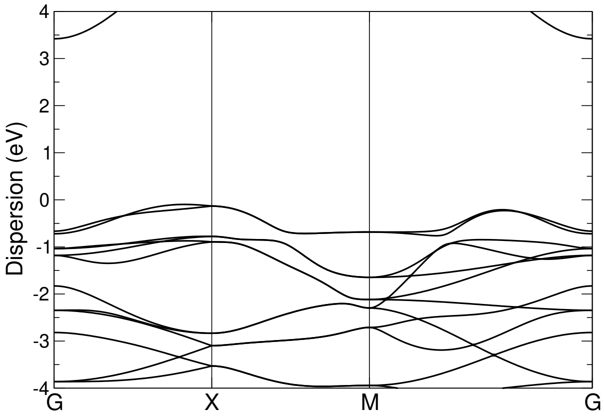

Band structure: Electronic band structure of PbClF (P4/nmm) in a representative window around the Fermi energy and along a high-symmetry path.

Optimized crystal structure

Structural parameters: relaxed cell (top) and atomic positions (bottom) of PbClF (P4/nmm) in Cartesian coordinates.

	x [Å]	y [Å]	z [Å]
$\mathbf{a_1}$	4.02719915	0.00000000	0.00000000
$\mathbf{a_2}$	0.00000000	4.02719915	0.00000000
$\mathbf{a_3}$	0.00000000	0.00000000	25.15948268

		x [Å]	y [Å]	z [Å]
	Pb	0.00000000	2.01359958	14.14127857
	Cl	2.01359958	0.00000000	15.31434150
	Pb	2.01359958	0.00000000	11.01820410
	Cl	0.00000000	2.01359958	9.84514117
	F	2.01359958	2.01359958	12.57974134
	F	0.00000000	0.00000000	12.57974134

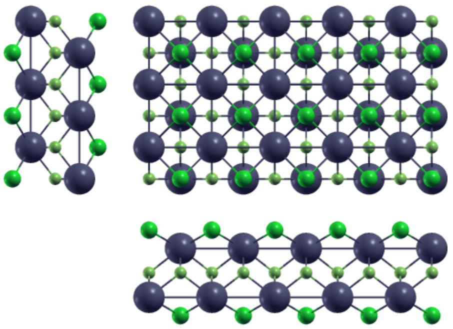

Orthographic projections: views of PbClF (P4/nmm) as seen from the x axis (left), the y axis (bottom) and the z axis (center).

Lattice matching - minimal size

Formula	N° atoms	strain	cell size 1	cell size 2
K	7	0.1669	1	1
Tl	7	0.11	1	1
InSe	8	0.1335	1	1
Bi_2	8	0.1381	1	1
Ag_2	8	0.1732	1	1
P_2	8	0.1112	1	1
PbTe	8	0.1349	1	1
CaCl	8	0.2157	1	1
Cl_2Mn	9	0.1089	1	1
CdI_2	9	0.1363	1	1
Nd	9	0.1804	1	3
$MoSe_2$	9	0.1102	1	1
Ba_2Pt	9	0.1729	1	1
Br_2Ca	9	0.1372	1	1
CaI_2	9	0.1571	1	1
Br_2Cu	9	0.1002	1	1
Ca_2Si	9	0.1782	1	1
I_2Yb	9	0.1545	1	1
BiClTe	9	0.1365	1	1
Cl_2Ti	9	0.1112	1	1
HgI_2	9	0.3829	1	1
BaF_2	9	0.1307	1	1
BiBrTe	9	0.1411	1	1
NbS_2	9	0.1094	1	1
S_2Ta	9	0.1096	1	1
CKN	9	0.4866	1	1
Se_2V	9	0.1099	1	1
AsKSn	9	0.1297	1	1
Cl_2Cu	9	0.0966	1	1
I_2Tm	9	0.1559	1	1
GeI_2	9	0.1351	1	1
I_2Pb	9	0.175	1	1
STl_2	9	0.1314	1	1
BiITe	9	0.147	1	1
GeS_2	9	0.5889	1	1
$MnSe_2$	9	0.2156	1	1
DyI_2	9	0.16	1	1
GdI_2	9	0.1436	1	1
I_2La	9	0.0085	1	1
CdI_2	9	0.1359	1	1
Sm	9	0.1614	1	3
I_2Pr	9	0.1366	1	1
Se_2W	9	0.1102	1	1
Bi_2Te_2	10	0.1944	1	1
Bi_2In_2	10	1.1272	1	1
Cu_2I_2	10	0.0077	1	1
Cu_2Sr_2	10	0.142	1	1
Cl_2OOs	10	0.2221	1	1
$LiMnTe_2$	10	0.1353	1	1
Cu_2Te_2	10	0.0575	1	1

Table showing the first 50 lattice matching combinations with the smallest number of atoms in the supercell.

Lattice matching - minimal strain

Formula	N° atoms	strain	cell size 1	cell size 2
$Bi_2I_2O_2$	12	0.0001	1	1
$Cu_2Na_2Se_2$	12	0.0001	1	1
$Ba_2F_2I_2$	882	0.0001	82	65
Fe_2S_2	718	0.0002	65	82
$F_2I_2Tm_2$	12	0.0002	1	1
HgI_2	843	0.0003	100	81
I_3Sn	866	0.0004	109	53
$Ba_2H_2I_2$	690	0.0005	65	50
CoH_2O_2	315	0.0005	20	39
Ca_2Cl_2	896	0.0005	82	101
ReS_2	237	0.0006	20	39
CdClHO	548	0.0006	48	65
Fe_2S_2	708	0.0006	64	81
O_4PTl	882	0.0006	82	65
$Ba_2H_2I_2$	678	0.0006	64	49
$Hf_2Se_2Si_2$	678	0.0006	49	64
Cl_2Rb_2	484	0.0008	64	25
Cl_4Mn	474	0.0009	49	36
$I_2Nd_2O_2$	12	0.0009	1	1
$PtSe_2$	483	0.0009	48	65
$Ba_2F_2I_2$	870	0.0009	81	64
Te_2V	840	0.0009	81	118
Br_2Mn	840	0.001	81	118
Ca_2Cl_2	886	0.0011	81	100
$As_2Cd_2K_2$	870	0.0011	81	64
Mg_4	814	0.0011	65	106
$AgClO_4$	366	0.0011	36	25
As_2Fe_2	550	0.0011	49	64
$AgClO_2$	384	0.0012	36	42
FKO_2Se	270	0.0012	30	18
OTl_2	840	0.0012	81	118
O_2Sn_2	740	0.0012	66	86
O_4PTl	870	0.0012	81	64
As_2Co_2	896	0.0012	82	101
Mg_3	537	0.0013	49	81
Br_2CsF	580	0.0013	64	49
$H_2Na_2O_2$	876	0.0013	61	85
Ag_2I_2	924	0.0013	100	81
$RhTe_2$	483	0.0013	48	65
CdClO	840	0.0014	81	118
KS_2Ti	958	0.0014	81	118
I_3Sn	796	0.0014	100	49
$MoSe_2$	711	0.0014	64	109
Se_2W	711	0.0014	64	109
$Ba_2Ge_2Mn_2$	12	0.0014	1	1
Tl	496	0.0014	65	106
CNNa	489	0.0015	55	53
$AsSn_2$	483	0.0015	48	65
ClNZr	852	0.0016	79	126
Tl	493	0.0016	64	109

Table showing the first 50 lattice matching combinations with the smallest strain needed to build the supercell.

PbF_4 (P4/mmm)

Structural and electronic properties

**Formula**	PbF_4
**Spacegroup**	P4/mmm
**Prototype**	SnF4
**Parent 3D**	PbF_4
**Source DB**	COD
**DB ID**	1528583
**DF2-C09 Binding energy [meV/ Å^2]**	15.66
**RVV10 Binding energy [meV/ Å^2]**	28.95
**Band gap (PBE) [eV]**	2.49

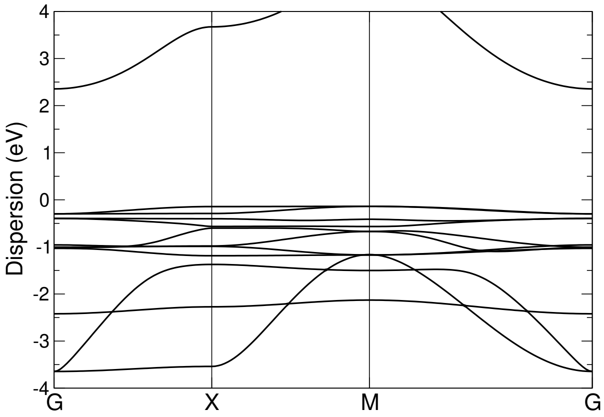

Band structure: Electronic band structure of PbF_4 (P4/mmm) in a representative window around the Fermi energy and along a high-symmetry path.

Optimized crystal structure

Structural parameters: relaxed cell (top) and atomic positions (bottom) of PbF_4 (P4/mmm) in Cartesian coordinates.

		x [Å]	y [Å]	z [Å]
$\mathbf{a_1}$		4.40688125	0.00000000	0.00000000
$\mathbf{a_2}$		0.00000000	4.40688125	0.00000000
$\mathbf{a_3}$		0.00000000	0.00000000	24.07510781
		x [Å]	y [Å]	z [Å]
•	F	2.20344063	2.20344063	10.05200076
●	Pb	2.20344063	2.20344063	12.03755390
•	F	2.20344063	2.20344063	14.02310705
•	F	0.00000000	2.20344063	12.03755390
•	F	2.20344063	0.00000000	12.03755390

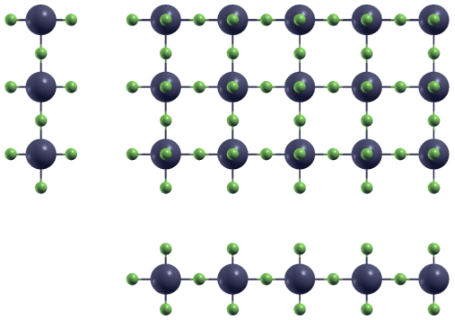

Orthographic projections: views of PbF_4 (P4/mmm) as seen from the x axis (left), the y axis (bottom) and the z axis (center).

Lattice matching - minimal size

Formula	N° atoms	strain	cell size 1	cell size 2
AgTl	7	0.2237	1	1
Ag_2	7	0.1323	1	1
As_2	7	0.1108	1	1
Cl_2Zn	8	0.111	1	1
Nd	8	0.137	1	3
PSn_2	8	0.1089	1	1
Ba_2Pt	8	0.1321	1	1
HfS_2	8	0.1102	1	1
I_2Pr	8	0.2113	1	1
S_2Zr	8	0.109	1	1
Ca_2Si	8	0.1356	1	1
Br_2Co	8	0.1109	1	1
Ca_2N	8	0.1107	1	1
BrCdI	8	0.1087	1	1
HgI_2	8	0.0076	1	1
Br_2Mn	8	0.1113	1	1
$CoTe_2$	8	0.1101	1	1
CKN	8	0.3859	1	1
I_2Nd	8	0.2123	1	1
Cl_2Cu	8	0.1441	1	1
S_2Sn	8	0.109	1	1
I_2Pb	8	0.1335	1	1
Br_2Fe	8	0.1109	1	1
Br_2Ni	8	0.1097	1	1
CeI_2	8	0.2104	1	1
$NbTe_2$	8	0.1091	1	1
Cl_2Mg	8	0.1097	1	1
I_2La	8	0.2177	1	1
F_2Zn	8	0.207	1	1
Bi_2Te_2	9	0.1465	1	1
Li_2Tl_2	9	0.1716	1	1
Cl_2OOs	9	0.5395	1	1
Ir_2P_2	9	0.2121	1	1
Ag_2Br_2	9	0.2183	1	1
Cu_2S_2	9	0.5918	1	1
Au_2Br_2	9	0.0162	1	1
Ge_2Te_2	9	0.0153	1	1
Br_2Cu_2	9	0.5973	1	1
As_2Ir_2	9	0.221	1	1
$AgBrO_2$	9	0.1234	1	1
Ge_2S_2	9	0.0506	1	1
$MnNaTe_2$	9	0.1086	1	1
O_2Sn_2	9	0.5935	1	1
KS_2Ti	9	0.1112	1	1
Ga_2S_2	9	0.1101	1	1
P_2Rh_2	9	0.5898	1	1
F_2Tl_2	9	0.5901	1	1
Sb_2Te_2	9	0.1346	1	1
Ge_2Se_2	9	0.2283	1	1
Ag_2Te_2	9	0.599	1	1

Table showing the first 50 lattice matching combinations with the smallest number of atoms in the supercell.

Lattice matching - minimal strain

Formula	N° atoms	strain	cell size 1	cell size 2
$Ca_2Cl_2F_2$	474	0.0	36	49
$I_2O_2Tm_2$	817	0.0001	65	82
Cu_2Te_2	645	0.0001	61	85
H_4Ti	810	0.0002	53	109
$Br_2F_2Yb_2$	640	0.0002	50	65
Br_2Cu_2	510	0.0003	50	65
$As_2Co_2Li_2$	815	0.0003	61	85
$Br_2O_2Tb_2$	629	0.0004	49	64
I_2Nd	563	0.0004	64	81
In_2Se_2	500	0.0004	48	65
$Br_2O_2Sm_2$	806	0.0004	64	81
O_2Sn_2	501	0.0005	49	64
Fe_2Se_2	269	0.0005	25	36
Br_3Cs	556	0.0005	80	39
Ho_2S_2	253	0.0005	25	32
$I_2N_2Ti_2$	480	0.0006	36	50
Ir_2P_2	644	0.0006	64	81
$Br_2Eu_2F_2$	817	0.0006	65	82
$Br_2Ca_2F_2$	640	0.0006	50	65
$Er_2I_2O_2$	817	0.0007	65	82
$I_2O_2Tm_2$	806	0.0007	64	81
H_2Li_2Pd	810	0.0007	53	109
$Br_2Lu_2S_2$	570	0.0007	54	50
I_2Zn	435	0.0007	48	65
$Br_2Lu_2S_2$	570	0.0007	54	50
Br_2Cu_2	501	0.0008	49	64
Br_2Cu	861	0.0008	93	132
As_2Ir_2	814	0.0009	82	101
Cl_2Hf_2	256	0.0009	20	39
$Hf_2Se_2Si_2$	230	0.0009	16	25
$Ge_2Hf_2Te_2$	815	0.0009	61	85
Cu_2O_2	253	0.0009	25	32
Bi_2Pd	93	0.0009	9	16
$I_2O_2Yb_2$	806	0.001	64	81
Ag_2Br_2	805	0.001	81	100
S_2Zn_2	877	0.001	81	118
F_2Zn	445	0.001	50	65
$Si_2Te_2Zr_2$	341	0.001	25	36
Cu_2S_2	501	0.001	49	64
F_2Ni	233	0.0011	25	36
$Bi_2Br_2O_2$	817	0.0011	65	82
Cl_2Y_2	899	0.0011	79	126
Cl_2Tb_2	877	0.0011	81	118
$Fe_2Li_2P_2$	341	0.0011	25	36
Mo_2Te_4	968	0.0011	94	83
CrS_2	585	0.0011	48	115
Ga_2Se_2	899	0.0011	79	126
$Br_2O_2Y_2$	629	0.0011	49	64
CeI_2	1000	0.0011	113	145
$Br_2Dy_2O_2$	629	0.0012	49	64

Table showing the first 50 lattice matching combinations with the smallest strain needed to build the supercell.

PbI_2 (P-3m1)

Structural and electronic properties

**Formula**	PbI_2
**Spacegroup**	P-3m1
**Prototype**	CdI2
**Parent 3D**	Pb_2I_4
**Source DB**	COD
**DB ID**	9009143
**DF2-C09 Binding energy [meV/ Å^2]**	8.55
**RVV10 Binding energy [meV/ Å^2]**	15.84
**Band gap (PBE) [eV]**	2.54

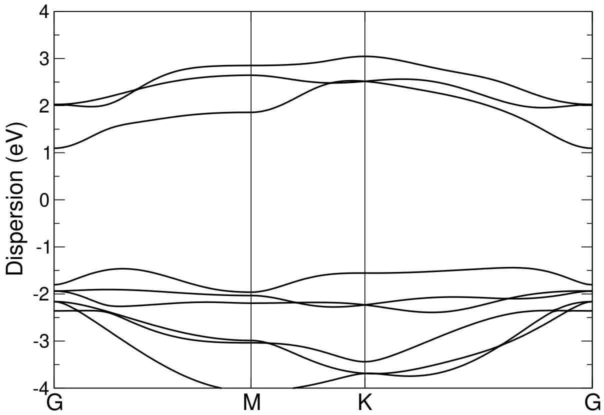

Band structure: Electronic band structure of PbI_2 (P-3m1) in a representative window around the Fermi energy and along a high-symmetry path.

Optimized crystal structure

Structural parameters: relaxed cell (top) and atomic positions (bottom) of PbI_2 (P-3m1) in Cartesian coordinates.

	x [Å]	y [Å]	z [Å]
$\mathbf{a_1}$	4.64483455	0.00000000	0.00000000
$\mathbf{a_2}$	−2.32241728	4.02254472	0.00000000
$\mathbf{a_3}$	0.00000000	0.00000000	23.90178579

		x [Å]	y [Å]	z [Å]
●	I	2.32241728	1.34084824	13.82769331
●	Pb	2.32241728	4.02254472	11.95089290
●	I	-0.00000000	2.68169648	10.07409249

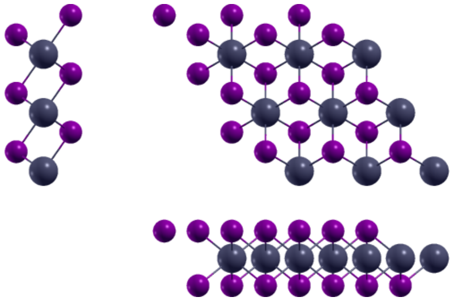

Orthographic projections: views of PbI_2 (P-3m1) as seen from the x axis (left), the y axis (bottom) and the z axis (center).

Lattice matching - minimal size

Formula	N° atoms	strain	cell size 1	cell size 2
K	4	0.1404	1	1
K	4	0.0069	1	1
AgTl	5	0.1202	1	1
Ag_2	5	0.0016	1	1
As_2	5	4.8642	1	1
Sb_2	5	0.2554	1	1
I_2Mg	6	0.2633	1	1
Ba_2Pt	6	0.0018	1	1
$HfTe_2$	6	1.5376	1	1
I_2Pr	6	0.1119	1	1
Br_2La	6	0.2637	1	1
Ca_2Si	6	0.0027	1	1
Br_2Co	6	4.855	1	1
Ca_2N	6	4.8754	1	1
$AuTe_2$	6	1.5939	1	1
BrCdI	6	0.2677	1	1
I_2Zn	6	1.6282	1	1
BaF_2	6	0.2726	1	1
GeI_2	6	0.2606	1	1
Ba_2Hg	6	0.1289	1	1
Ba_2N	6	1.5485	1	1
AsKSn	6	0.2705	1	1
Te_2Zr	6	1.5411	1	1
$PbTe_2$	6	0.2663	1	1
I_2Nd	6	0.1123	1	1
Cl_2Cu	6	0.3092	1	1
$SnTe_2$	6	0.2575	1	1
STl_2	6	0.274	1	1
Br_2Fe	6	4.8565	1	1
CeI_2	6	0.1117	1	1
Se_2Yb	6	0.261	1	1
$BiTe_2$	6	0.2613	1	1
$PtTe_2$	6	1.591	1	1
I_2La	6	0.1141	1	1
F_2Zn	6	0.4425	1	1
Ba_2Cd	6	0.1307	1	1
NaPSn	6	1.5312	1	1
Fe_2Te_2	7	0.4225	1	1
Ca_2Cl_2	7	0.4235	1	1
Cu_2I_2	7	0.1222	1	1
Nd	7	0.1633	2	1
Cu_2Te_2	7	0.1112	1	1
Ir_2P_2	7	0.1122	1	1
Ag_2Br_2	7	0.1143	1	1
Tl	7	0.1549	2	1
O_2Sn_2	7	0.1115	1	1
Cu_2S_2	7	0.4351	1	1
Au_2Br_2	7	0.1278	1	1
Br_2Tb_2	7	1.5803	1	1
As_2Ir_2	7	0.1154	1	1

Table showing the first 50 lattice matching combinations with the smallest number of atoms in the supercell.

Lattice matching - minimal strain

Formula	N° atoms	strain	cell size 1	cell size 2
$Br_2Hf_2N_2$	477	0.0	37	61
Ca_2N	390	0.0	49	81
BrNZr	75	0.0	9	16
Cu_3Se_3	402	0.0	36	49
Br_2HLa	516	0.0	64	81
SSb_2Te_2	743	0.0	81	100
Sb_2	317	0.0001	57	73
BrCdI	543	0.0001	81	100
Se_2W	222	0.0001	25	49
Gd_2I_2	583	0.0001	73	91
CoH_2O_2	57	0.0001	4	9
$PtTe_2$	300	0.0002	43	57
Cu_2I_2	583	0.0002	73	91
Br_2Gd_2Ge	674	0.0002	73	91
In_2Se_2	403	0.0002	49	64
$AgNO_3$	705	0.0002	100	81
$AuTe_2$	300	0.0002	43	57
Ba_2N	255	0.0002	36	49
$MoSe_2$	222	0.0003	25	49
Br_2La_2	463	0.0003	57	73
$MnNaTe_2$	643	0.0003	81	100
Bi_2Se_3	674	0.0003	73	91
$CeLi_2P_2$	597	0.0004	64	81
FeI_2	183	0.0004	25	36
Br_2La	492	0.0004	73	91
Br_2Hf_2	91	0.0004	9	16
Tl	124	0.0005	25	49
$PtSe_2$	123	0.0005	16	25
$AsCuLi_2$	516	0.0005	64	81
Li_2P_2Pr	536	0.0005	57	73
As_2	309	0.0005	49	81
$Cl_2N_2Zr_2$	477	0.0005	37	61
Br_2Mg	183	0.0005	25	36
Au_2I_2	552	0.0006	80	78
NaO_4	705	0.0006	100	81
Ca_2N	294	0.0006	37	61
$Ga_2Ge_2Te_2$	609	0.0006	57	73
Hg_3N_2	47	0.0006	9	4
CCl_2Lu_2	552	0.0007	49	81
$I_2Pr_2Si_2$	678	0.0007	64	81
$GeTe_2$	183	0.0007	25	36
Fe_2S_2	715	0.0007	81	118
I_2Mg	492	0.0008	73	91
Ga_2Te_2	516	0.0008	64	81
Ba_2Ni_3	597	0.0008	64	81
CaH_2O_2	416	0.0008	37	61
Br_2Er_2	357	0.0008	43	57
CrI_2	183	0.0008	25	36
Te_4TiZr	183	0.0008	25	18
Te_2Zr	255	0.0008	36	49

Table showing the first 50 lattice matching combinations with the smallest strain needed to build the supercell.

PbIF (P4/nmm)

Structural and electronic properties

**Formula**	PbIF
**Spacegroup**	P4/nmm
**Prototype**	PbClF
**Parent 3D**	$Pb_2I_2F_2$
**Source DB**	COD
**DB ID**	9007903
**DF2-C09 Binding energy [meV/ Å^2]**	15.39
**RVV10 Binding energy [meV/ Å^2]**	21.48
**Band gap (PBE) [eV]**	2.33

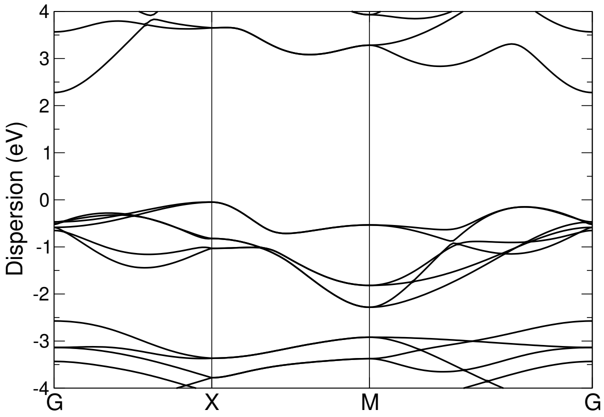

Band structure: Electronic band structure of PbIF (P4/nmm) in a representative window around the Fermi energy and along a high-symmetry path.

Optimized crystal structure

Structural parameters: relaxed cell (top) and atomic positions (bottom) of PbIF (P4/nmm) in Cartesian coordinates.

		x [Å]	y [Å]	z [Å]
$\mathbf{a_1}$		4.20740819	0.00000000	0.00000000
$\mathbf{a_2}$		0.00000000	4.20740819	0.00000000
$\mathbf{a_3}$		0.00000000	0.00000000	26.06708143
		x [Å]	y [Å]	z [Å]
●	Pb	0.00000000	2.10370409	14.53094578
●	I	2.10370409	0.00000000	16.18770312
●	Pb	2.10370409	0.00000000	11.53613565
●	I	0.00000000	2.10370409	9.87937831
●	F	2.10370409	2.10370409	13.03354071
●	F	0.00000000	0.00000000	13.03354071

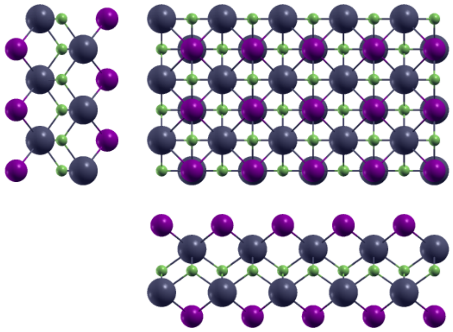

Orthographic projections: views of PbIF (P4/nmm) as seen from the x axis (left), the y axis (bottom) and the z axis (center).

Lattice matching - minimal size

Formula	N° atoms	strain	cell size 1	cell size 2
K	7	0.1459	1	1
AgTl	8	0.0205	1	1
Ag_2	8	0.1511	1	1
CaCl	8	0.5637	1	1
Ba_2Pt	9	0.1508	1	1
$ReSe_2$	9	0.1104	1	1
CaI_2	9	0.138	1	1
Ca_2Si	9	0.1553	1	1
I_2Yb	9	0.1359	1	1
Cl_2Zn	9	0.2089	1	1
Ba_2Hg	9	0.0027	1	1
Cl_2Ni	9	0.1102	1	1
$CrTe_2$	9	0.109	1	1
Br_2V	9	0.1106	1	1
ClNZr	9	0.1111	1	1
Ba_2N	9	0.1084	1	1
Br_2Ti	9	0.1091	1	1
$AsSe_2$	9	0.1099	1	1
I_2Tm	9	0.137	1	1
I_2Pb	9	0.1526	1	1
BiITe	9	0.13	1	1
BrNZr	9	0.1096	1	1
$NbSe_2$	9	0.1102	1	1
$MnSe_2$	9	0.5634	1	1
Br_2Cr	9	0.109	1	1
DyI_2	9	0.1403	1	1
Se_2Ta	9	0.1101	1	1
$NbSe_2$	9	0.1099	1	1
CNNa	9	0.3406	1	1
F_2Ni	9	0.5946	1	1
Se_2Ta	9	0.1092	1	1
Ba_2Cd	9	0.0001	1	1
Bi_2Te_2	10	0.1689	1	1
Cl_2OOs	10	0.6033	1	1
$AgCuTe_2$	10	0.1831	1	1
O_2Sn_2	10	0.2155	1	1
Au_2Br_2	10	0.0091	1	1
Bi_2	10	0.9746	1	2
Ge_2Te_2	10	0.0192	1	1
Fe_2Se_2	10	0.5929	1	1
Cl_2ORu	10	0.5884	1	1
N_3Na	10	0.0389	1	1
Cu_2Te_2	10	0.2117	1	1
Ge_2S_2	10	0.232	1	1
As_4	10	0.0575	1	1
Cl_2Zr_2	10	0.1108	1	1
Au_2I_2	10	0.0272	1	1
Na	10	0.8535	1	4
Sb_2Te_2	10	0.154	1	1
Co_2S_2	10	0.5656	1	1

Table showing the first 50 lattice matching combinations with the smallest number of atoms in the supercell.

Lattice matching - minimal strain

Formula	N° atoms	strain	cell size 1	cell size 2
Co_2Se_2	962	0.0	85	113
$Ge_2Hf_2Te_2$	870	0.0001	64	81
F_2Ni	486	0.0001	49	64
$Si_2Te_2Zr_2$	678	0.0001	49	64
Ba_2Cd	9	0.0001	1	1
Co_2S_2	412	0.0002	36	49
I_2Mn	483	0.0002	48	65
$CdCl_2$	483	0.0002	48	65
$I_2S_2Tl_2$	678	0.0002	49	64
N_3W_2	974	0.0002	54	130
$Cl_2Er_2H_2$	678	0.0002	48	65
$H_2Li_2O_2$	876	0.0003	61	85
Ni_2Te_2	548	0.0003	48	65
$InSe_2$	483	0.0003	48	65
GeS_2	258	0.0004	25	36
H_2Na_2Pd	330	0.0004	25	36
Cu_2Se_2	550	0.0004	49	64
$As_2Co_2Li_2$	870	0.0005	64	81
Cl_2OV	504	0.0006	42	63
Ga_2Se_2	958	0.0006	81	118
H_2Si_2	548	0.0006	48	65
Fe_2Se_2	550	0.0007	49	64
Au_2Se_2	216	0.0008	24	18
Mg_4	118	0.0008	9	16
CaCl	314	0.0008	36	49
LiO	198	0.0009	20	39
$Cl_2S_2Tl_2$	876	0.0009	85	61
I_2Ni	483	0.0009	48	65
$MnSe_2$	363	0.0009	36	49
Cu_2Te_2	708	0.0009	64	81
Cl_2Y_2	958	0.001	81	118
GeTe	418	0.001	48	65
AlH_4Na	882	0.001	82	65
Te_2V	852	0.001	79	126
Te_4W_2	162	0.001	15	12
OTl_2	852	0.0011	79	126
$GeTe_2$	483	0.0011	48	65
Ag_2F_4	330	0.0011	35	20
$As_2Fe_2Li_2$	882	0.0011	65	82
CdClO	852	0.0011	79	126
Tl	70	0.0011	9	16
HgO	542	0.0011	58	97
$Br_2H_2Yb_2$	882	0.0012	65	82
$AgBrO_2$	872	0.0012	80	98
Cr_2O_4	972	0.0012	61	101
In_2S_3	613	0.0012	48	65
As_2Fe_2	294	0.0012	25	36
CNNa	954	0.0013	104	110
Fe_2O_4	228	0.0013	14	24
Sn	394	0.0014	49	100

Table showing the first 50 lattice matching combinations with the smallest strain needed to build the supercell.

PbO (P4/nmm)

Structural and electronic properties

**Formula**	PbO
**Spacegroup**	P4/nmm
**Prototype**	FeSe
**Parent 3D**	$Ag_2Pb_2O_2Br_2$
**Source DB**	ICSD
**DB ID**	33913
**DF2-C09 Binding energy [meV/ Å^2]**	7.84
**RVV10 Binding energy [meV/ Å^2]**	14.32
**Band gap (PBE) [eV]**	2.45

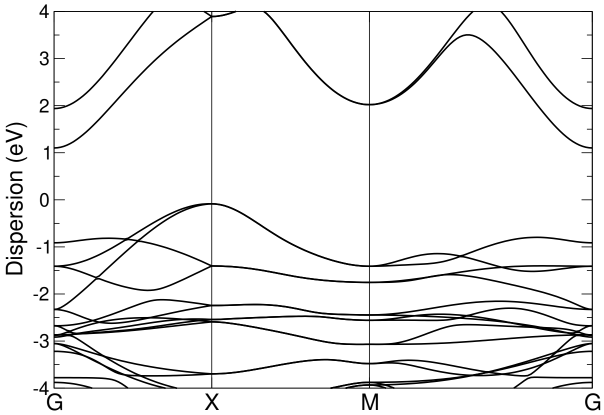

Band structure: Electronic band structure of PbO (P4/nmm) in a representative window around the Fermi energy and along a high-symmetry path.

Optimized crystal structure

Structural parameters: relaxed cell (top) and atomic positions (bottom) of PbO (P4/nmm) in Cartesian coordinates.

	x [Å]	y [Å]	z [Å]
$\mathbf{a_1}$	3.95797861	0.00000000	0.00000000
$\mathbf{a_2}$	−0.00000000	3.95797861	0.00000000
$\mathbf{a_3}$	0.00000000	0.00000000	23.27354698

		x [Å]	y [Å]	z [Å]
●	Ag	1.97898930	0.00000000	10.47019933
●	Ag	-0.00000000	1.97898930	12.80334765
●	Br	1.97898930	0.00000000	13.34766873
●	Br	-0.00000000	1.97898930	9.92587825

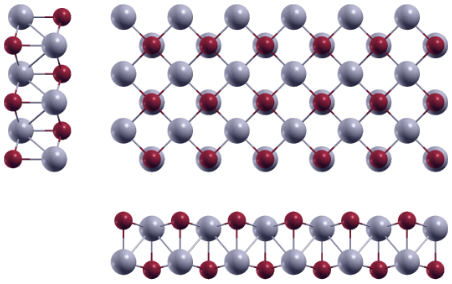

Orthographic projections: views of PbO (P4/nmm) as seen from the x axis (left), the y axis (bottom) and the z axis (center).

Lattice matching - minimal size

Formula	N° atoms	strain	cell size 1	cell size 2
K	5	0.1764	1	1
InSe	6	0.1401	1	1
Bi_2	6	0.1452	1	1
AgTl	6	0.0173	1	1
Ag_2	6	0.1831	1	1
LiO	6	0.1111	1	1
P_2	6	0.1094	1	1
PbTe	6	0.1416	1	1
I_2Mg	7	0.132	1	1
CdI_2	7	0.1431	1	1
Nd	7	0.1907	1	3
Ba_2Pt	7	0.1828	1	1
Br_2Zn	7	0.1089	1	1
Br_2Ca	7	0.1442	1	1
CaI_2	7	0.1658	1	1
I_2Pr	7	0.0061	1	1
Br_2La	7	0.1322	1	1
Br_2Cu	7	0.1029	1	1
Ca_2Si	7	0.1885	1	1
I_2Yb	7	0.163	1	1
BiClTe	7	0.1435	1	1
Cl_2Ti	7	0.1094	1	1
BrCdI	7	0.1343	1	1
HgI_2	7	0.4009	1	1
Te_2Ti	7	0.1089	1	1
BaF_2	7	0.137	1	1
BiBrTe	7	0.1485	1	1
GeI_2	7	0.1306	1	1
AsKSn	7	0.1358	1	1
$PbTe_2$	7	0.1336	1	1
I_2Nd	7	0.0052	1	1
Cl_2Cu	7	0.0974	1	1
I_2Tm	7	0.1645	1	1
GeI_2	7	0.1418	1	1
I_2Pb	7	0.1851	1	1
STl_2	7	0.1377	1	1
BiITe	7	0.1549	1	1
GeS_2	7	0.2114	1	1
DyI_2	7	0.169	1	1
CeI_2	7	0.0069	1	1
Se_2Yb	7	0.1308	1	1
$BiTe_2$	7	0.131	1	1
GdI_2	7	0.1512	1	1
I_2La	7	0.0005	1	1
$CrSe_2$	7	0.1114	1	1
CdI_2	7	0.1428	1	1
I_2Pr	7	0.1435	1	1
$HfSe_2$	7	0.1089	1	1
Bi_2Te_2	8	0.7742	1	1
Bi_2In_2	8	1.1722	1	1

Table showing the first 50 lattice matching combinations with the smallest number of atoms in the supercell.

Lattice matching - minimal strain

Formula	N° atoms	strain	cell size 1	cell size 2
$H_2Li_2O_2$	934	0.0001	82	101
$Hf_2Se_2Si_2$	742	0.0001	64	81
$Ba_2F_2I_2$	550	0.0001	64	49
As_2Rh_2	8	0.0003	1	1
Br_2CsF	340	0.0003	49	36
$Br_2Lu_2S_2$	34	0.0004	4	3
$Cu_2K_2Te_2$	962	0.0004	113	85
As_2Fe_2	580	0.0004	64	81
$Br_2Lu_2S_2$	34	0.0004	4	3
$Br_2O_2Pr_2$	10	0.0005	1	1
Tl	89	0.0005	16	25
Mg_3	995	0.0005	113	181
I_2La	7	0.0005	1	1
$H_2Li_2O_2$	924	0.0006	81	100
O_4PTl	550	0.0006	64	49
O_2Zn	197	0.0006	20	39
HgI_2	919	0.0006	145	113
$Br_2N_2Zr_2$	582	0.0007	48	65
CBr_2Lu_2	517	0.0007	48	65
Mg_2	426	0.0007	58	97
P_4	284	0.0008	35	36
Mg_4	164	0.0008	16	25
$Cl_2H_2Lu_2$	582	0.0009	48	65
O_2Sn_2	920	0.001	102	128
Bi_2Se_4	942	0.001	138	65
F_4Pb	805	0.001	100	81
Bi_2Se_4	914	0.001	134	63
H_4Ti	601	0.001	49	81
$Ag_2K_2Se_2$	708	0.0011	81	64
Cl_2NSc_2	946	0.0011	79	126
Br_2OV	640	0.0011	71	89
O_2Sn_2	920	0.0012	102	128
Bi_2Se_4	246	0.0012	36	17
$H_2I_2Yb_2$	10	0.0012	1	1
Se_2Ta_4	438	0.0012	36	49
H_2Li_2Pd	717	0.0012	58	97
Cl_2Mg	387	0.0012	48	65
GeS_2	499	0.0012	64	81
Br_2Ni	387	0.0013	48	65
Bi_2Se_4	218	0.0013	32	15
NbS_2	694	0.0013	79	126
Cl_2Mn	694	0.0013	79	126
Cl_4KTl	154	0.0014	25	9
$Cu_2Rb_2Te_2$	412	0.0014	49	36
K	501	0.0014	103	89
PTe_2Ti_2	517	0.0014	48	65
Br_2Zr_2	796	0.0014	81	118
O_4PSn	962	0.0014	113	85
$I_2S_2Sm_2$	946	0.0015	103	89
Ho_2S_2	860	0.0015	106	109

Table showing the first 50 lattice matching combinations with the smallest strain needed to build the supercell.

PbTe (P3m1)

Structural and electronic properties

**Formula**	PbTe
**Spacegroup**	P3m1
**Prototype**	GeTe
**Parent 3D**	$Pb_2Bi_2Te_5$
**Source DB**	ICSD
**DB ID**	42708
**DF2-C09 Binding energy [meV/ Å^2]**	27.49
**RVV10 Binding energy [meV/ Å^2]**	32.98
**Band gap (PBE) [eV]**	1.64

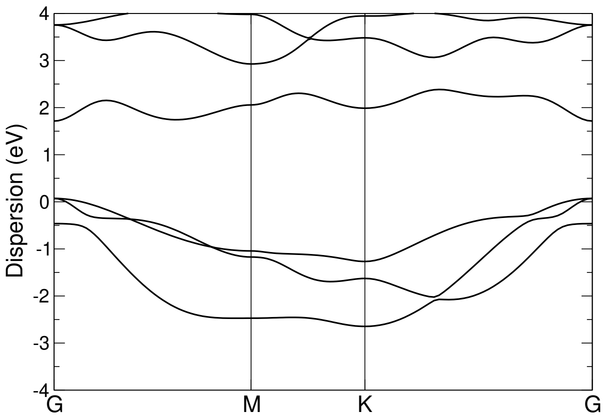

Band structure: Electronic band structure of PbTe (P3m1) in a representative window around the Fermi energy and along a high-symmetry path.

Optimized crystal structure

Structural parameters: relaxed cell (top) and atomic positions (bottom) of PbTe (P3m1) in Cartesian coordinates.

	x [Å]	y [Å]	z [Å]
$\mathbf{a_1}$	4.26096939	0.00000000	0.00000000
$\mathbf{a_2}$	−2.13048470	3.69010774	0.00000000
$\mathbf{a_3}$	0.00000000	0.00000000	21.61397501

		x [Å]	y [Å]	z [Å]
●	Te	0.00000000	2.46007183	11.68016541
●	Pb	0.00000000	0.00000000	9.93380960

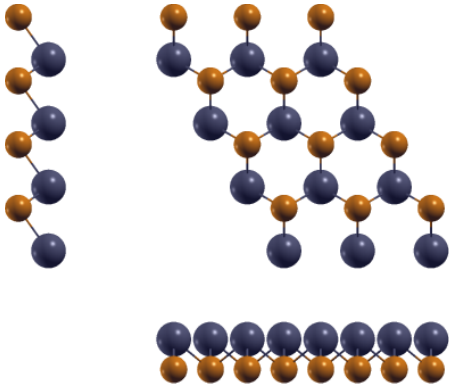

Orthographic projections: views of PbTe (P3m1) as seen from the x axis (left), the y axis (bottom) and the z axis (center).

Lattice matching - minimal size

Formula	N° atoms	strain	cell size 1	cell size 2
InSe	4	0.0018	1	1
AsSb	4	0.2692	1	1
Bi_2	4	0.0041	1	1
AgTl	4	0.1508	1	1
CaCl	4	0.1127	1	1
CdI_2	5	0.0017	1	1
$MoSe_2$	5	4.8804	1	1
Br_2Zn	5	0.2548	1	1
Br_2Ca	5	0.003	1	1
HfS_2	5	1.526	1	1
$AsSn_2$	5	1.5981	1	1
$GeTe_2$	5	0.275	1	1
$SiTe_2$	5	0.2605	1	1
I_2Pr	5	0.1351	1	1
S_2Zr	5	1.5648	1	1
Br_2Cu	5	0.7119	1	1
NSr_2	5	0.2715	1	1
PbS_2	5	0.2654	1	1
BiClTe	5	0.0021	1	1
BrCdI	5	0.0087	1	1
Cl_2Zn	5	0.1193	1	1
FeI_2	5	0.2734	1	1
I_2Ni	5	0.2753	1	1
HgI_2	5	0.2061	1	1
Te_2Ti	5	0.2552	1	1
CrI_2	5	0.2727	1	1
BaF_2	5	0.0055	1	1
BiBrTe	5	0.0077	1	1
$RhTe_2$	5	0.2494	1	1
$CoTe_2$	5	1.5283	1	1
AsKSn	5	0.0069	1	1
I_2Nd	5	0.1358	1	1
$NiTe_2$	5	0.2597	1	1
Cl_2Cu	5	0.0681	1	1
I_2V	5	0.2624	1	1
GeI_2	5	0.0002	1	1
Se_2Zr	5	0.2611	1	1
STl_2	5	0.0046	1	1
$PtSe_2$	5	0.2465	1	1
CoI_2	5	0.2698	1	1
GeS_2	5	0.4289	1	1
$MnSe_2$	5	0.1127	1	1
Br_2Ni	5	1.5401	1	1
CeI_2	5	0.1345	1	1
Br_2Mg	5	0.2732	1	1
I_2Ti	5	0.2703	1	1
$NbTe_2$	5	1.5633	1	1
Cl_2Mg	5	1.5405	1	1
F_2Ni	5	0.1173	1	1
I_2La	5	0.1397	1	1

Table showing the first 50 lattice matching combinations with the smallest number of atoms in the supercell.

Lattice matching - minimal strain

Formula	N° atoms	strain	cell size 1	cell size 2
CdO_2	107	0.0	16	25
Ga_2S_3	230	0.0	25	36
As_2CeLi_2	7	0.0	1	1
H_2NiO_2	98	0.0	9	16
NS_2Zr	194	0.0001	25	36
$RhTe_2$	290	0.0001	49	64
Cl_2Co	107	0.0001	16	25
$HfSe_2$	333	0.0002	57	73
Te_2Ti	333	0.0002	57	73
Br_2CsF	322	0.0002	65	48
$TaTe_2$	257	0.0002	43	57
Ga_2Se_2	510	0.0002	73	91
Cl_2Zr	107	0.0002	16	25
Se_2W	257	0.0002	37	61
GeI_2	5	0.0002	1	1
$MoSe_2$	341	0.0002	49	81
$NiTe_2$	371	0.0003	64	81
IKO_3	95	0.0003	25	9
Au_2Br_2	404	0.0003	74	64
O_2Zn	197	0.0003	25	49
$Cl_2O_2Y_2$	692	0.0003	73	91
Na	86	0.0003	25	36
CaClHO	452	0.0004	64	81
$Hf_2I_2N_2$	428	0.0004	43	57
Tl	135	0.0004	37	61
CdClHO	314	0.0004	43	57
PTe_2Zr_2	662	0.0004	81	100
$MoSe_2$	257	0.0004	37	61
$ClKO_3$	253	0.0004	64	25
Br_2Zn	333	0.0005	57	73
$Cl_2O_2Tm_2$	614	0.0005	64	81
S_2Ti	107	0.0005	16	25
Bi_2Te_2	418	0.0005	81	64
$Br_2Er_2S_2$	646	0.0005	101	74
$LiMnTe_2$	6	0.0005	1	1
Br_2Y_2	510	0.0005	73	91
Cu_2O_4	774	0.0006	78	103
Cl_2Fe	107	0.0006	16	25
$Cl_2H_2Lu_2$	366	0.0006	36	49
CCl_2Gd_2	479	0.0006	57	73
CdClHO	354	0.0007	49	64
$Br_2S_2Yb_2$	584	0.0007	91	67
H_4Ti	670	0.0007	65	108
PtS_2	158	0.0007	25	36
Br_2Pr_2	406	0.0007	57	73
$Cl_2Er_2S_2$	460	0.0007	71	53
Cu_4Te_2	472	0.0007	59	59
$AsSn_2$	257	0.0008	43	57
PbS_2	419	0.0008	73	91
Bi_2STe_2	7	0.0008	1	1

Table showing the first 50 lattice matching combinations with the smallest strain needed to build the supercell.

$PdCl_2$ ($P2_1/c$)

Structural and electronic properties

**Formula**	$PdCl_2$
**Spacegroup**	$P2_1/c$
**Prototype**	PdCl2
**Parent 3D**	Pd_2Cl_4
**Source DB**	ICSD
**DB ID**	421221
**DF2-C09 Binding energy [meV/ Å^2]**	14.02
**RVV10 Binding energy [meV/ Å^2]**	21.28
**Band gap (PBE) [eV]**	0.91

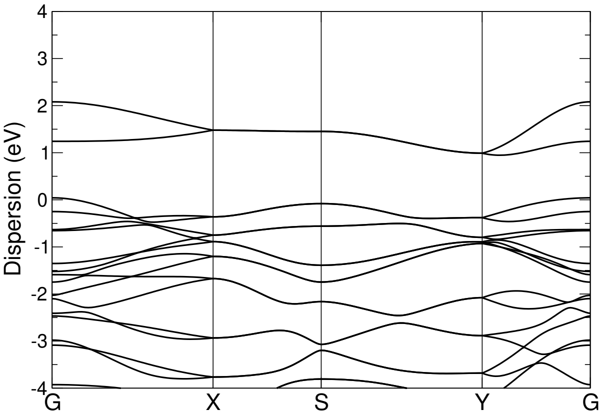

Band structure: Electronic band structure of $PdCl_2$ ($P2_1/c$) in a representative window around the Fermi energy and along a high-symmetry path.

Optimized crystal structure

Structural parameters: relaxed cell (top) and atomic positions (bottom) of $PdCl_2$ ($P2_1/c$) in Cartesian coordinates.

	x [Å]	y [Å]	z [Å]
$\mathbf{a_1}$	4.24897133	−0.00001406	0.00000000
$\mathbf{a_2}$	−0.00001979	6.32738160	0.00000000
$\mathbf{a_3}$	0.00000000	0.00000000	22.69370559

		x [Å]	y [Å]	z [Å]
●	Cl	2.88378759	6.45443522	10.06585823
●	Cl	3.48952668	3.29046197	10.06572858
●	Pd	0.00012382	1.50550120	11.34687582
●	Pd	2.12460296	4.66900533	11.34682980
●	Cl	0.75937335	6.04745282	12.62786128
●	Cl	1.36507935	2.88399689	12.62796308

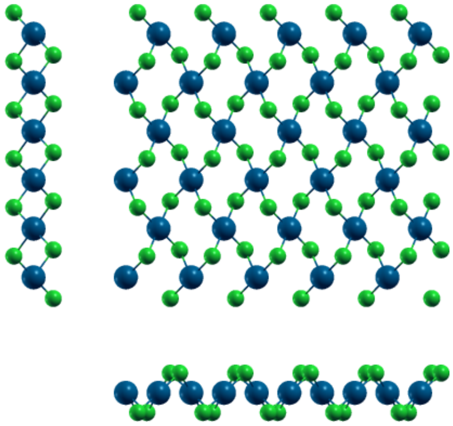

Orthographic projections: views of $PdCl_2$ ($P2_1/c$) as seen from the x axis (left), the y axis (bottom) and the z axis (center).

Lattice matching - minimal size

Formula	N° atoms	strain	cell size 1	cell size 2
HgI_2	9	0.2814	1	1
HgO	10	0.127	1	2
AsSb	10	0.1549	1	2
Ge_2Te_2	10	0.2627	1	1
Ag_2	10	0.8474	1	2
As_2	10	0.1331	1	2
Au_2Se_2	10	0.0376	1	1
$SbSe_2Tl$	10	0.1355	1	1
Ag_2I_2	10	0.283	1	1
Cl_2Zn	12	0.1325	1	2
$CdCl_2$	12	0.1594	1	2
PSn_2	12	0.0419	1	2
Ba_2Pt	12	0.8464	1	2
$ReSe_2$	12	0.2828	1	2
Br_2Zn	12	0.1464	1	2
$InSe_2$	12	0.1597	1	2
$AsSn_2$	12	0.1413	1	2
$SiTe_2$	12	0.1497	1	2
Te_2V	12	0.131	1	2
I_2Mn	12	0.1595	1	2
$CuTe_2$	12	0.0453	1	2
S_2Zr	12	0.0423	1	2
NSr_2	12	0.1563	1	2
Br_2Co	12	0.1328	1	2
$Cl_2Rh_2Te_2$	12	0.1447	1	1
Ca_2N	12	0.1334	1	2
$K_2O_2Tl_2$	12	0.0342	1	1
FeI_2	12	0.1575	1	2
I_2Ni	12	0.1586	1	2
$I_2Se_2Tb_2$	12	0.407	1	1
Te_2Ti	12	0.1466	1	2
$Gd_2I_2Se_2$	12	0.4105	1	1
$Br_2S_2Y_2$	12	0.3561	1	1
CrI_2	12	0.1571	1	2
$RhTe_2$	12	0.1433	1	2
$Gd_2I_2S_2$	12	0.3659	1	1
$C_2Br_2Gd_2$	12	0.4326	1	1
Br_2Mn	12	0.1318	1	2
$CrTe_2$	12	0.1262	1	2
$Br_2Er_2Se_2$	12	0.3918	1	1
Br_2V	12	0.2816	1	2
ClNZr	12	0.2785	1	2
CdClO	12	0.1307	1	2
Cl_4Mg_2	12	0.3963	1	1
$I_2S_2Tb_2$	12	0.363	1	1
Se_2Ti	12	0.1279	1	2
Br_2Ti	12	0.1259	1	2
$PbTe_2$	12	0.6626	1	2
$NiTe_2$	12	0.1492	1	2
$Er_2I_2Se_2$	12	0.0363	1	1

Table showing the first 50 lattice matching combinations with the smallest number of atoms in the supercell.

Lattice matching - minimal strain

Formula	N° atoms	strain	cell size 1	cell size 2
In_2Se_2	870	0.0006	81	96
$Cl_2H_2Lu_2$	120	0.0007	6	14
Cu_2O_2	944	0.0008	72	128
Cl_2Mg	78	0.0008	6	14
O_2Zn	402	0.0008	25	84
Br_2Ni	78	0.0008	6	14
PTe_2Ti_2	106	0.0009	6	14
CdO_2	393	0.0009	28	75
$Br_2Cr_2O_2$	396	0.001	21	45
Ga_2S_2	92	0.0011	6	14
$AgClO_2$	576	0.0013	42	81
$Ce_2I_2S_2$	726	0.0013	50	71
Ca_2Si	513	0.0013	50	71
$F_2Se_2Y_2$	222	0.0013	16	21
$I_2La_2Si_2$	114	0.0014	7	12
In_2Se_2	860	0.0015	80	95
Br_2Ca_3Si	618	0.0016	41	62
In_2Se_2	796	0.0016	74	88
$AgNO_2$	966	0.0016	77	126
$I_2N_2Ti_2$	438	0.0016	25	48
$Ca_2Cl_2F_2$	414	0.0017	24	45
H_2MnO_2	866	0.0017	36	130
Bi_2Se_2	652	0.0017	54	82
$AuCrTe_4$	852	0.0017	70	72
$AuCrTe_4$	828	0.0017	68	70
$Br_2O_2Tm_2$	462	0.0017	27	50
$Er_2I_2Se_2$	102	0.0017	8	9
Br_2Ca_3Si	588	0.0017	39	59
As_2Ru_2	362	0.0017	27	50
$Br_2N_2Zr_2$	120	0.0018	6	14
$Ho_2I_2Se_2$	102	0.0018	8	9
$Ge_2Se_2Zr_2$	438	0.0018	24	49
Ca_2Cl_2	340	0.0018	24	49
Ca_2Cl_2	362	0.0018	27	50
CBr_2Lu_2	106	0.0018	6	14
Pd_2S_4	756	0.0018	67	59
Ag_2Te_2	814	0.0018	61	112
Li_2Tl_2	834	0.0018	77	93
Pd_2S_4	714	0.0019	63	56
Te_4W_2	930	0.0019	70	85
$I_2S_2Tb_2$	618	0.0019	41	62
Cl_2Hf_2	404	0.0019	24	65
$Ag_2K_2Te_2$	354	0.0019	27	32
$Br_2O_2Yb_2$	414	0.0019	24	45
Fe_2Te_2	362	0.002	27	50
Ga_2S_2	92	0.002	6	14
Cu_2Se_2	626	0.002	45	89
O_2Sn_2	584	0.002	44	80
Te_4W_2	876	0.002	66	80
FeO_2	177	0.002	10	39

Table showing the first 50 lattice matching combinations with the smallest strain needed to build the supercell.

PdS_2 ($P2_1/c$)

Structural and electronic properties

**Formula**	PdS_2
**Spacegroup**	$P2_1/c$
**Prototype**	PdS2
**Parent 3D**	Pd_4S_8
**Source DB**	COD
**DB ID**	2310589
**DF2-C09 Binding energy [meV/ Å^2]**	27.08
**RVV10 Binding energy [meV/ Å^2]**	31.51
**Band gap (PBE) [eV]**	1.21

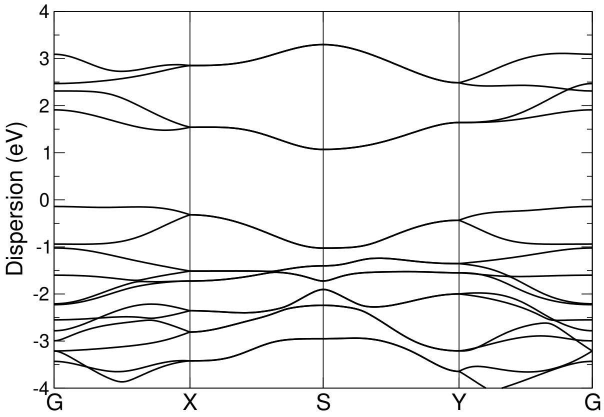

Band structure: Electronic band structure of PdS_2 ($P2_1/c$) in a representative window around the Fermi energy and along a high-symmetry path.

Optimized crystal structure

Structural parameters: relaxed cell (top) and atomic positions (bottom) of PdS_2 ($P2_1/c$) in Cartesian coordinates.

	x [Å]	y [Å]	z [Å]
$\mathbf{a_1}$	5.46285818	0.00000000	0.00000000
$\mathbf{a_2}$	0.00000000	5.56730796	0.00000000
$\mathbf{a_3}$	0.00000000	0.00000000	21.24510231

		x [Å]	y [Å]	z [Å]
●	Pd	2.73142909	0.00000000	10.62255116
●	Pd	0.00000000	2.78365398	10.62255116
•	S	0.57041189	0.62086748	9.98455773
•	S	3.30184098	2.16278650	11.26054458
•	S	4.89244628	4.94644048	11.26054458
•	S	2.16101719	3.40452146	9.98455773

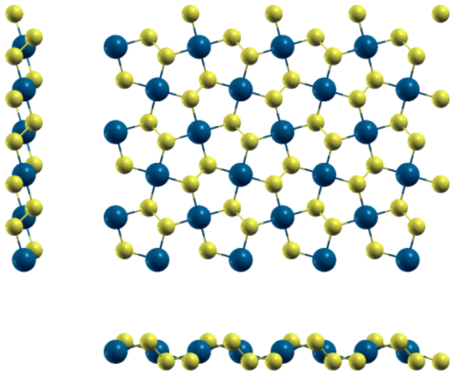

Orthographic projections: views of PdS_2 ($P2_1/c$) as seen from the x axis (left), the y axis (bottom) and the z axis (center).

Lattice matching - minimal size

Formula	N° atoms	strain	cell size 1	cell size 2
K	7	0.1095	1	1
In	8	0.1436	1	2
CaI_2	9	0.1115	1	1
I_2Yb	9	0.1121	1	1
I_2Tm	9	0.1118	1	1
Nd	10	0.2138	1	4
In_2Se_2	10	0.1007	1	1
Cl_2Rb_2	10	1.189	1	1
Tl	10	0.2014	1	4
LiO	10	0.1471	1	2
Au_2I_2	10	0.5096	1	1
Ba_2Cu_2	10	0.1122	1	1
GeI_2La_2	11	0.1119	1	1
Cl_4Mn	11	0.5647	1	1
I_2La_2Te	11	0.11	1	1
S_2V	12	0.1445	1	2
MoS_2	12	0.1448	1	2
AlH_4Na	12	0.5722	1	1
Cl_4Cu_2	12	0.389	1	1
$I_2Se_2Tb_2$	12	0.1875	1	1
$Gd_2I_2Se_2$	12	0.1895	1	1
S_2W	12	0.1448	1	2
$Br_2Er_2Se_2$	12	0.4574	1	1
CNRb	12	0.4904	1	2
Br_2Ca_3Si	12	0.1099	1	1
$Er_2I_2Se_2$	12	0.1835	1	1
$I_2Se_2Tm_2$	12	0.1822	1	1
$Ag_2K_2Te_2$	12	0.5885	1	1
Cl_2V	12	0.1462	1	2
As_2	12	0.1657	1	3
S_2	12	0.8061	1	3
Br_2Ca_3Si	12	0.1102	1	1
$H_2I_2Sr_2$	12	1.6684	1	1
MoS_2	12	0.1449	1	2
$I_2S_2Sm_2$	12	0.1095	1	1
$CrSe_2$	12	0.1458	1	2
$I_2Se_2Yb_2$	12	0.1814	1	1
$AgClO_4$	12	0.6104	1	1
$CrSe_2$	12	0.1467	1	2
O_2Pt	12	0.1431	1	2
$Cl_2S_2Tl_2$	12	0.2237	1	1
$Ho_2I_2Se_2$	12	0.1844	1	1
$Er_2I_2S_2$	12	0.4539	1	1
Li_2Tl_2	14	0.4309	1	2
Cu_2O_2	14	0.0049	1	2
Pb_2Se_2	14	0.3942	1	2
Ho_2S_2	14	0.0046	1	2
FHOZn	14	0.1458	1	2
Cl_2Zn	15	0.1645	1	3
HfS_2	15	0.1688	1	3

Table showing the first 50 lattice matching combinations with the smallest number of atoms in the supercell.

Lattice matching - minimal strain

Formula	N° atoms	strain	cell size 1	cell size 2
NSr_2	471	0.0001	36	85
Se_2Sn	471	0.0002	36	85
$Br_2Gd_2O_2$	708	0.0003	39	79
F_2Zn	471	0.0003	39	79
CeI_2	420	0.0003	35	70
$Br_2Eu_2O_2$	630	0.0003	35	70
Ni_2Se_2	490	0.0004	35	70
I_2Pr	423	0.0006	36	69
BiClTe	423	0.0006	36	69
$Br_2Ca_2F_2$	708	0.0006	39	79
$Ag_2K_2Te_2$	588	0.0006	42	56
CdI_2	423	0.0007	36	69
GeI_2La_2	842	0.0007	57	100
I_2Tm	642	0.0008	57	100
$I_2Lu_2O_2$	708	0.0008	39	79
$Ce_2I_2S_2$	750	0.0008	48	77
I_2Pr	420	0.0008	35	70
$Br_2F_2Tm_2$	708	0.0008	39	79
CrI_2	471	0.0009	36	85
Br_2La_2P	561	0.0009	36	69
Br_2Ca	423	0.001	36	69
$Br_2F_2Yb_2$	708	0.001	39	79
$Ca_2Ge_2Mn_2$	708	0.001	39	79
$I_2S_2Sm_2$	708	0.001	44	74
Cl_2Tb_2	756	0.001	50	114
CdI_2	423	0.001	36	69
NaO_4	809	0.0011	64	85
S_2Zn_2	756	0.0012	50	114
CoI_2	471	0.0012	36	85
CNRb	966	0.0012	97	128
K	338	0.0012	44	74
Br_2Mg	471	0.0012	36	85
$I_2O_2Yb_2$	630	0.0012	35	70
CdO_2	663	0.0013	44	133
I_2Yb	642	0.0013	57	100
FeI_2	471	0.0013	36	85
Pb_2Se_2	894	0.0014	73	114
Bi_2STe_2	561	0.0014	36	69
P_2Sn_2	756	0.0014	50	114
Ba_2Cu_2	742	0.0015	57	100
$Bi_2Cl_2O_2$	630	0.0015	35	70
Li_2Tl_2	804	0.0015	70	96
Br_2Cu_2	550	0.0015	39	79
$AgNO_3$	907	0.0015	72	95
Ir_2P_2	490	0.0016	35	70
$Au_2K_2Se_2$	642	0.0016	67	40
AsSb	386	0.0016	36	85
CaI_2	642	0.0016	57	100
C_4Ca_2	888	0.0016	53	95
Hg_4O_2	438	0.0016	49	24

Table showing the first 50 lattice matching combinations with the smallest strain needed to build the supercell.

Pr_2Br_2 (P-3m1)

Structural and electronic properties

**Formula**	Pr_2Br_2
**Spacegroup**	P-3m1
**Prototype**	PtTe
**Parent 3D**	Br_2Pr_2
**Source DB**	MPDS
**DB ID**	S542061
**DF2-C09 Binding energy [meV/ Å^2]**	14.15
**RVV10 Binding energy [meV/ Å^2]**	N/A
**Band gap (PBE) [eV]**	0.0

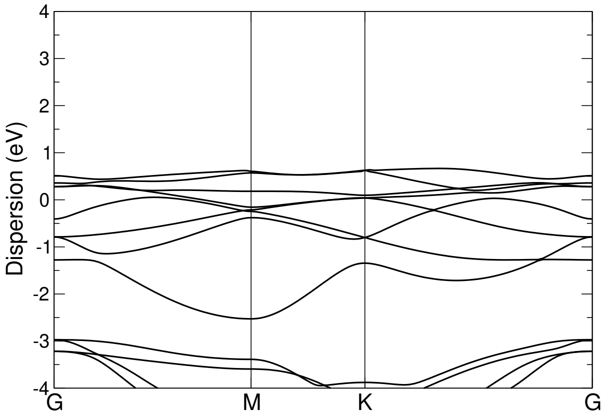

Band structure: Electronic band structure of Pr_2Br_2 (P-3m1) in a representative window around the Fermi energy and along a high-symmetry path.

Optimized crystal structure

Structural parameters: relaxed cell (top) and atomic positions (bottom) of Pr_2Br_2 (P-3m1) in Cartesian coordinates.

		x [Å]	y [Å]	z [Å]
$\mathbf{a_1}$		1.88544991	−3.26569504	0.00000000
$\mathbf{a_2}$		1.88544991	3.26569504	0.00000000
$\mathbf{a_3}$		0.00000000	0.00000000	22.94417887
		x [Å]	y [Å]	z [Å]
●	Pr	0.00000000	1.08856501	0.75625964
●	Br	1.88544991	0.00000000	2.74872141
●	Pr	0.00000000	-1.08856501	-0.75625964
●	Br	1.88544991	0.00000000	-2.74872141

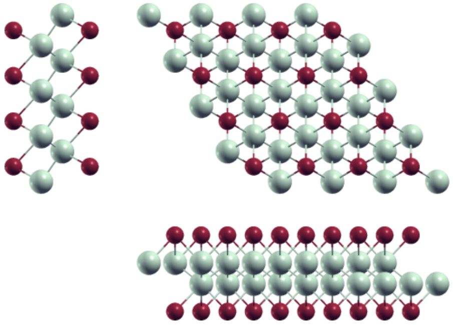

Orthographic projections: views of Pr_2Br_2 (P-3m1) as seen from the x axis (left), the y axis (bottom) and the z axis (center).

Lattice matching - minimal size

Formula	N° atoms	strain	cell size 1	cell size 2
Tl	5	0.1115	1	1
Tl	5	0.2533	1	1
InSe	6	0.4768	1	1
HgO	6	0.1164	1	1
AsSb	6	0.0092	1	1
P_2	6	0.2464	1	1
PbTe	6	0.4814	1	1
CaCl	6	0.1519	1	1
I_2Mg	7	0.451	1	1
Cl_2Mn	7	0.2612	1	1
CdI_2	7	0.4858	1	1
$AgTe_2$	7	0.1126	1	1
S_2Ta	7	0.2642	1	1
Br_2Zn	7	0.0012	1	1
$AsSn_2$	7	0.0079	1	1
$SiTe_2$	7	0.0029	1	1
Br_2La	7	0.4518	1	1
PbS_2	7	0.0065	1	1
BiClTe	7	0.4868	1	1
Cl_2Ti	7	0.2466	1	1
BrCdI	7	0.4587	1	1
S_2Ti	7	0.2713	1	1
Te_2Ti	7	0.0009	1	1
NbS_2	7	0.2635	1	1
BaF_2	7	0.467	1	1
BiBrTe	7	0.5015	1	1
$RhTe_2$	7	0.0052	1	1
Bi_2Pd	7	0.1214	1	1
Cl_2Co	7	0.2708	1	1
NbS_2	7	0.2575	1	1
ClNZr	7	0.276	1	1
Cl_2Fe	7	0.2697	1	1
S_2Ta	7	0.2563	1	1
Se_2V	7	0.2545	1	1
AsKSn	7	0.4634	1	1
$PbTe_2$	7	0.4563	1	1
$NiTe_2$	7	0.0023	1	1
I_2V	7	0.0043	1	1
GeI_2	7	0.482	1	1
Se_2Zr	7	0.0034	1	1
STl_2	7	0.4694	1	1
$PtSe_2$	7	0.0073	1	1
CdO_2	7	0.2706	1	1
GeS_2	7	0.1408	1	1
$TaTe_2$	7	0.0085	1	1
$MnSe_2$	7	0.1519	1	1
Cl_2Zr	7	0.2703	1	1
$BiTe_2$	7	0.4477	1	1
F_2Na	7	0.0008	1	1
CdI_2	7	0.4847	1	1

Table showing the first 50 lattice matching combinations with the smallest number of atoms in the supercell.

Lattice matching - minimal strain

Formula	N° atoms	strain	cell size 1	cell size 2
$LiNbS_2$	656	0.0	73	91
BaF_2	583	0.0001	91	73
LiO	242	0.0001	36	49
S_2Ta	565	0.0001	73	91
$LiOS_2Ti$	661	0.0002	64	81
Bi_2STe_2	577	0.0002	73	57
$Ga_2I_2Y_2$	802	0.0002	91	73
$LiMnTe_2$	520	0.0002	73	57
AlH_4Na	476	0.0002	65	36
$Cu_2Se_2Tl_2$	548	0.0002	65	48
Cu_2Sr_2	400	0.0002	57	43
$Br_2O_2Ti_2$	420	0.0002	45	40
GeI_3Rb	301	0.0002	64	9
$I_2La_2Si_2$	708	0.0002	81	64
As_2Li_2Pr	644	0.0003	81	64
$Br_2Cr_2O_2$	484	0.0003	49	48
InSe	452	0.0003	81	64
$F_2Se_2Y_2$	618	0.0003	81	49
As_2Li_2Nd	644	0.0004	81	64
Sb_2Te_3	501	0.0004	64	49
I_2La_2	724	0.0004	100	81
N_2W	139	0.0004	16	25
GeI_2	463	0.0005	73	57
CdI_2	463	0.0006	73	57
NbS_2	565	0.0006	73	91
GeI_2Y_2	644	0.0006	81	64
$Er_2I_2Se_2$	768	0.0006	108	56
$AsLi_3$	580	0.0006	81	64
S_2Ta	447	0.0006	57	73
$I_2S_2Tb_2$	294	0.0006	36	25
NiO_2	84	0.0007	9	16
As_2CeLi_2	577	0.0007	73	57
Br_2La_2P	577	0.0007	73	57
I_2La_2P	443	0.0007	57	43
PbTe	406	0.0007	73	57
Se_2V	447	0.0007	57	73
$Cl_2O_2Yb_2$	10	0.0007	1	1
Bi_2SeTe_2	501	0.0008	64	49
Ho_2S_2	624	0.0008	86	70
F_2Na	7	0.0008	1	1
CNb_2S_2	457	0.0008	43	57
H_2MnO_2	601	0.0008	49	81
Br_3Cs	424	0.0008	81	25
$HfSe_2$	7	0.0009	1	1
BN	34	0.0009	4	9
Te_2Ti	7	0.0009	1	1
AsKSn	643	0.0009	100	81
BiITe	304	0.0009	49	36
$F_2Se_2Y_2$	466	0.001	61	37
Cu_4Te_2	550	0.001	64	49

Table showing the first 50 lattice matching combinations with the smallest strain needed to build the supercell.

$Pr_2S_2I_2$ (P-3m1)

Structural and electronic properties

**Formula**	$Pr_2S_2I_2$
**Spacegroup**	P-3m1
**Prototype**	SmSI
**Parent 3D**	$I_2Pr_2S_2$
**Source DB**	MPDS
**DB ID**	S1703862
**DF2-C09 Binding energy [meV/ Å^2]**	10.7
**RVV10 Binding energy [meV/ Å^2]**	N/A
**Band gap (PBE) [eV]**	0.0

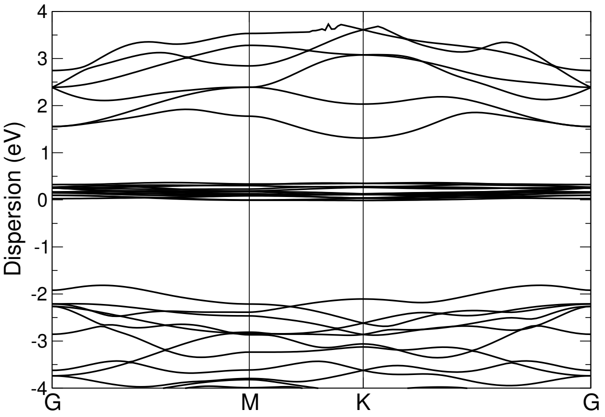

Band structure: Electronic band structure of $Pr_2S_2I_2$ (P-3m1) in a representative window around the Fermi energy and along a high-symmetry path.

Optimized crystal structure

Structural parameters: relaxed cell (top) and atomic positions (bottom) of $Pr_2S_2I_2$ (P-3m1) in Cartesian coordinates.

	x [Å]	y [Å]	z [Å]
$\mathbf{a_1}$	2.31820132	−4.01524247	0.00000000
$\mathbf{a_2}$	2.31820132	4.01524247	0.00000000
$\mathbf{a_3}$	0.00000000	0.00000000	26.98331198

	x [Å]	y [Å]	z [Å]
Pr	3.47730198	0.66920708	-1.77131964
Pr	1.15910066	-0.66920708	1.77131964
S	3.47730198	0.66920708	1.04552339
S	1.15910066	-0.66920708	-1.04552339
I	1.15910066	2.00762123	-3.77151254
I	1.15910066	2.00762123	3.77151254

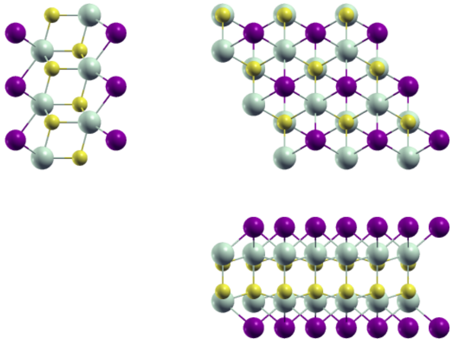

Orthographic projections: views of $Pr_2S_2I_2$ (P-3m1) as seen from the x axis (left), the y axis (bottom) and the z axis (center).

Lattice matching - minimal size

Formula	N° atoms	strain	cell size 1	cell size 2
K	7	0.1412	1	1
K	7	0.0061	1	1
AgTl	8	0.1206	1	1
Ag_2	8	0.0007	1	1
Sb_2	8	0.2566	1	1
I_2Mg	9	0.2645	1	1
Ba_2Pt	9	0.001	1	1
I_2Pr	9	0.1122	1	1
Br_2La	9	0.265	1	1
Ca_2Si	9	0.0036	1	1
BrCdI	9	0.269	1	1
BaF_2	9	0.2738	1	1
GeI_2	9	0.2618	1	1
Ba_2Hg	9	0.1295	1	1
AsKSn	9	0.2717	1	1
$PbTe_2$	9	0.2676	1	1
I_2Nd	9	0.1126	1	1
Cl_2Cu	9	0.3106	1	1
$SnTe_2$	9	0.2587	1	1
I_2Pb	9	0.0009	1	1
STl_2	9	0.2753	1	1
CeI_2	9	0.112	1	1
Se_2Yb	9	0.2622	1	1
$BiTe_2$	9	0.2626	1	1
I_2La	9	0.1145	1	1
F_2Zn	9	0.1109	1	1
Ba_2Cd	9	0.1314	1	1
Fe_2Te_2	10	0.4244	1	1
Ca_2Cl_2	10	0.4254	1	1
Cu_2I_2	10	0.1227	1	1
Cu_2Te_2	10	0.1113	1	1
Ir_2P_2	10	0.1125	1	1
Ag_2Br_2	10	0.1147	1	1
Br_2Er_2	10	0.2468	1	1
O_2Sn_2	10	0.1117	1	1
Au_2Br_2	10	0.1284	1	1
As_2Ir_2	10	0.1158	1	1
O_2Pb_2	10	0.1208	1	1
$AgBrO_2$	10	0.2899	1	1
Cl_2La_2	10	0.249	1	1
Br_2Gd_2	10	0.2475	1	1
$MnNaTe_2$	10	0.2684	1	1
$AsCuLi_2$	10	0.2612	1	1
Cu_2I_2	10	0.2658	1	1
I_2La_2	10	0.271	1	1
Br_2Ho_2	10	1.5903	1	1
Sb_2Te_2	10	0.0023	1	1
P_2	10	0.2233	1	2
O_2Sn_2	10	0.1304	1	1
Ag_2Te_2	10	0.1092	1	1

Table showing the first 50 lattice matching combinations with the smallest number of atoms in the supercell.

Lattice matching - minimal strain

Formula	N° atoms	strain	cell size 1	cell size 2
Br_2Fe	537	0.0	49	81
$PbTe_2$	786	0.0	81	100
Te_2Zr	363	0.0	36	49
Br_2Co	537	0.0001	49	81
CrI_2	258	0.0001	25	36
I_2Mg	711	0.0001	73	91
$Ce_2I_2Si_2$	984	0.0001	73	91
Cl_2La_2	550	0.0002	49	64
BH_4Li	870	0.0002	64	81
$I_2Se_2Tb_2$	966	0.0002	91	70
CCl_2Lu_2	699	0.0002	49	81
$NbSe_2$	102	0.0002	9	16
Hg_3N_2	74	0.0002	9	4
Ca_2N	405	0.0002	37	61
$AsSn_2$	171	0.0003	16	25
Br_2Ho_2	486	0.0003	43	57
As_2	344	0.0003	37	61
Br_2OV	322	0.0003	27	40
Br_2Mg	258	0.0003	25	36
$PtSe_2$	171	0.0004	16	25
Br_2Hf_2	118	0.0004	9	16
In_2Se_3	461	0.0004	36	49
CCl_2Lu_2	527	0.0004	37	61
$SnTe_2$	627	0.0004	64	81
Br_2La	711	0.0004	73	91
$HfTe_2$	363	0.0004	36	49
Sb_2Se_2Te	893	0.0005	73	91
FeI_2	258	0.0005	25	36
$AsSe_2$	102	0.0005	9	16
$CeLi_2P_2$	789	0.0005	64	81
Bi_2Se_3	893	0.0005	73	91
Bi_2S_3	543	0.0005	43	57
$MnNaTe_2$	886	0.0006	81	100
Br_2La_2	634	0.0006	57	73
$MoSe_2$	297	0.0006	25	49
Br_2Fe	405	0.0007	37	61
$PtTe_2$	429	0.0007	43	57
$Er_2F_2Se_2$	510	0.0007	36	49
Cl_2Zn	537	0.0007	49	81
Ag_2	8	0.0007	1	1
Br_2Co	405	0.0007	37	61
CoH_2O_2	69	0.0007	4	9
Gd_2I_2	802	0.0007	73	91
Se_2W	297	0.0007	25	49
Br_2Tb_2	486	0.0008	43	57
Sb_2	488	0.0008	57	73
$AgBrO_2$	746	0.0008	67	86
NSr_2	258	0.0008	25	36
Cu_3Se_3	510	0.0008	36	49
BrNZr	102	0.0008	9	16

Table showing the first 50 lattice matching combinations with the smallest strain needed to build the supercell.

$Pr_2Si_2I_2$ (P-3m1)

Structural and electronic properties

**Formula**	$Pr_2Si_2I_2$
**Spacegroup**	P-3m1
**Prototype**	SmSI
**Parent 3D**	$I_2Pr_2Si_2$
**Source DB**	MPDS
**DB ID**	S1902103
**DF2-C09 Binding energy [meV/ Å^2]**	14.52
**RVV10 Binding energy [meV/ Å^2]**	N/A
**Band gap (PBE) [eV]**	0.0

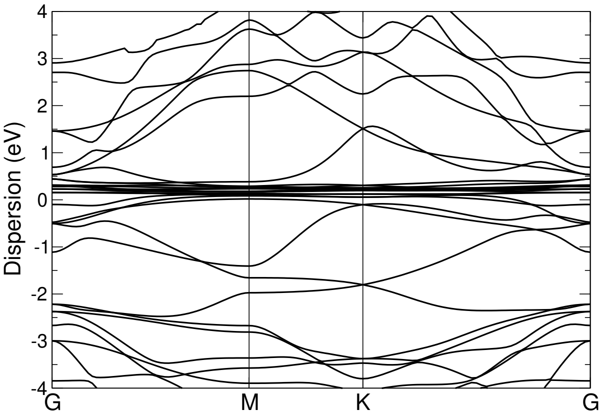

Band structure: Electronic band structure of $Pr_2Si_2I_2$ (P-3m1) in a representative window around the Fermi energy and along a high-symmetry path.

Optimized crystal structure

Structural parameters: relaxed cell (top) and atomic positions (bottom) of $Pr_2Si_2I_2$ (P-3m1) in Cartesian coordinates.

	x [Å]	y [Å]	z [Å]
$\mathbf{a_1}$	2.06728022	−3.58063437	0.00000000
$\mathbf{a_2}$	2.06728022	3.58063437	0.00000000
$\mathbf{a_3}$	0.00000000	0.00000000	28.18734617

		x [Å]	y [Å]	z [Å]
●	Pr	0.00000000	0.00000000	-1.93293999
●	Si	2.06728022	-1.19354479	-0.06822329
●	I	2.06728022	1.19354479	-4.07750817
●	Pr	0.00000000	0.00000000	1.93293999
●	Si	2.06728022	1.19354479	0.06822329
●	I	2.06728022	-1.19354479	4.07750817

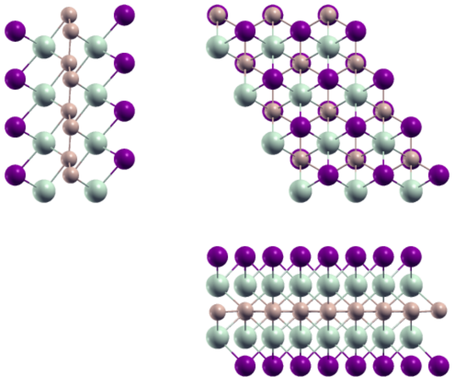

Orthographic projections: views of $Pr_2Si_2I_2$ (P-3m1) as seen from the x axis (left), the y axis (bottom) and the z axis (center).

Lattice matching - minimal size

Formula	N° atoms	strain	cell size 1	cell size 2
K	7	0.4566	1	1
AgTl	8	0.1654	1	1
Ag_2	8	0.4703	1	1
As_2	8	0.2477	1	1
Sb_2	8	0.0035	1	1
CaCl	8	0.1192	1	1
Cl_2Zn	9	0.2463	1	1
I_2Mg	9	0.0022	1	1
PSn_2	9	0.2597	1	1
Ba_2Pt	9	0.4696	1	1
Br_2Zn	9	0.275	1	1
$AsSn_2$	9	0.265	1	1
I_2Pr	9	0.1472	1	1
$CuTe_2$	9	1.6272	1	1
S_2Zr	9	0.2586	1	1
Br_2La	9	0.0025	1	1
Ca_2Si	9	0.4812	1	1
Br_2Co	9	0.2471	1	1
Ca_2N	9	0.2484	1	1
BrCdI	9	0.0053	1	1
Cl_2Zn	9	0.1279	1	1
Te_2Ti	9	0.2755	1	1
I_2Zn	9	0.0067	1	1
BaF_2	9	0.0088	1	1
Te_2Zn	9	1.5663	1	1
$RhTe_2$	9	0.2691	1	1
GeI_2	9	0.0003	1	1
AsKSn	9	0.0073	1	1
$PbTe_2$	9	0.0043	1	1
I_2Nd	9	0.1481	1	1
S_2Sn	9	0.259	1	1
$SnTe_2$	9	0.0019	1	1
Sn	9	0.6345	1	3
I_2Pb	9	0.4743	1	1
$PtSe_2$	9	0.2659	1	1
Br_2Fe	9	0.2472	1	1
GeS_2	9	0.1136	1	1
$TaTe_2$	9	0.2642	1	1
$MnSe_2$	9	0.1191	1	1
Br_2Ni	9	0.254	1	1
CeI_2	9	0.1465	1	1
CuO_2	9	0.1752	1	1
$NbTe_2$	9	0.2584	1	1
Se_2Yb	9	0.0005	1	1
Cl_2Mg	9	0.254	1	1
$BiTe_2$	9	0.0008	1	1
F_2Ni	9	0.1252	1	1
I_2La	9	0.1527	1	1
F_2Zn	9	0.1436	1	1
$HfSe_2$	9	0.2755	1	1

Table showing the first 50 lattice matching combinations with the smallest number of atoms in the supercell.

Lattice matching - minimal strain

Formula	N° atoms	strain	cell size 1	cell size 2
$Br_2Hf_2N_2$	678	0.0	49	64
Br_2Mn	429	0.0001	43	57
Ga_2Te_2	10	0.0001	1	1
$TaTe_2$	711	0.0001	73	91
Ba_2Ni_3	11	0.0001	1	1
Cl_2Zr_2	294	0.0001	25	36
$AsCuLi_2$	10	0.0002	1	1
HgI_2	534	0.0002	65	48
Cl_2V	537	0.0002	49	81
S_2Sn	627	0.0002	64	81
ReS_2	102	0.0002	9	16
CdClHO	886	0.0003	81	100
PSn_2	627	0.0003	64	81
GeI_2	9	0.0003	1	1
$CrSe_2$	405	0.0004	37	61
$AgNO_3$	230	0.0005	25	16
KS_2Ti	486	0.0005	43	57
S_2Zr	627	0.0005	64	81
$AsSn_2$	711	0.0005	73	91
$Cl_2N_2Zr_2$	678	0.0005	49	64
CCl_2Sc_2	330	0.0005	25	36
Se_2Yb	9	0.0005	1	1
Na	265	0.0006	36	49
In	498	0.0006	65	108
ClH_3O	557	0.0006	57	43
Br_2HLa	10	0.0006	1	1
$Hf_2I_2N_2$	984	0.0006	73	91
CdClHO	802	0.0007	73	91
Ca_2N	486	0.0007	49	64
I_2Pb	678	0.0007	81	64
$NbTe_2$	627	0.0007	64	81
Br_2V	258	0.0007	25	36
Sb_2Te_2	742	0.0007	81	64
CaH_2O_2	614	0.0007	49	64
$Cl_2H_2Lu_2$	780	0.0007	57	73
Ho_2S_2	396	0.0007	40	39
$AgBrO_2$	414	0.0008	41	42
$Ce_2I_2S_2$	780	0.0008	73	57
Ca_2Si	609	0.0008	73	57
CoH_2O_2	134	0.0008	9	16
$BiTe_2$	9	0.0008	1	1
Cl_2V	405	0.0008	37	61
$Br_2N_2Zr_2$	780	0.0008	57	73
$I_2Nd_2S_2$	984	0.0008	91	73
Au_2I_2	954	0.0009	105	81
$Br_2H_2Zr_2$	366	0.0009	25	36
CBr_2Lu_2	707	0.0009	57	73
MnO_2	51	0.0009	4	9
$AgTe_2$	840	0.0009	81	118
Ga_2S_3	461	0.0009	36	49

Table showing the first 50 lattice matching combinations with the smallest strain needed to build the supercell.

PrI_2 (P-6m2)

Structural and electronic properties

**Formula**	PrI_2
**Spacegroup**	P-6m2
**Prototype**	MoS2
**Parent 3D**	I_4Pr_2
**Source DB**	MPDS
**DB ID**	S1300558
**DF2-C09 Binding energy [meV/ Å^2]**	13.49
**RVV10 Binding energy [meV/ Å^2]**	N/A
**Band gap (PBE) [eV]**	0.0

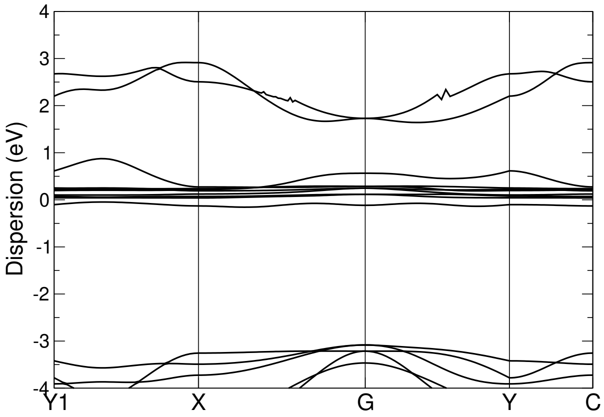

Band structure: Electronic band structure of PrI_2 (P-6m2) in a representative window around the Fermi energy and along a high-symmetry path.

Optimized crystal structure

Structural parameters: relaxed cell (top) and atomic positions (bottom) of PrI_2 (P-6m2) in Cartesian coordinates.

	x [Å]	y [Å]	z [Å]
$\mathbf{a_1}$	2.14028708	−3.70708596	0.00000000
$\mathbf{a_2}$	4.28057415	0.00000000	0.00000000
$\mathbf{a_3}$	0.00000000	0.00000000	20.25327946

		x [Å]	y [Å]	z [Å]
●	Pr	0.00000000	-2.47139064	0.00000000
●	I	-2.14028708	-1.23569532	-2.04634125
●	I	-2.14028708	-1.23569532	2.04634125

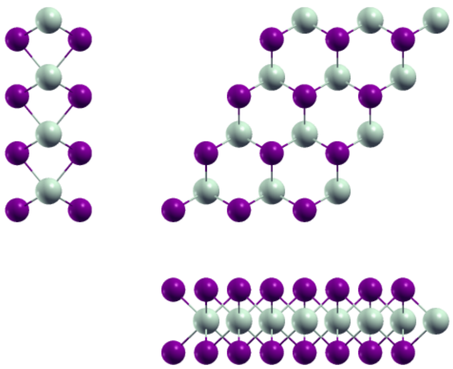

Orthographic projections: views of PrI_2 (P-6m2) as seen from the x axis (left), the y axis (bottom) and the z axis (center).

Lattice matching - minimal size

Formula	N° atoms	strain	cell size 1	cell size 2
Tl	4	4.8502	1	1
InSe	5	0.0039	1	1
AsSb	5	0.266	1	1
Bi_2	5	0.0019	1	1
GeTe	5	0.2749	1	1
AgTl	5	0.1487	1	1
PbTe	5	0.0022	1	1
CaCl	5	0.1119	1	1
$CdCl_2$	6	0.2733	1	1
CdI_2	6	0.0004	1	1
PSn_2	6	1.5546	1	1
Br_2Zn	6	1.6334	1	1
Br_2Ca	6	0.0008	1	1
$InSe_2$	6	0.2739	1	1
$AsSn_2$	6	1.5821	1	1
$GeTe_2$	6	0.2718	1	1
$SiTe_2$	6	0.2575	1	1
I_2Pr	6	0.1334	1	1
I_2Mn	6	0.2735	1	1
S_2Zr	6	1.5491	1	1
NSr_2	6	0.2684	1	1
PbS_2	6	0.2624	1	1
BiClTe	6	0.0001	1	1
Cl_2Zn	6	0.1182	1	1
FeI_2	6	0.2702	1	1
I_2Ni	6	0.2721	1	1
Te_2Ti	6	1.6356	1	1
CrI_2	6	0.2696	1	1
BaF_2	6	0.0076	1	1
BiBrTe	6	0.0055	1	1
$RhTe_2$	6	0.2466	1	1
Se_2V	6	4.8684	1	1
AsKSn	6	0.009	1	1
I_2Nd	6	0.1341	1	1
$NiTe_2$	6	0.2567	1	1
Cl_2Cu	6	0.0688	1	1
S_2Sn	6	1.551	1	1
I_2V	6	0.2593	1	1
GeI_2	6	0.0019	1	1
Se_2Zr	6	0.2581	1	1
STl_2	6	0.0067	1	1
$PtSe_2$	6	1.5869	1	1
CoI_2	6	0.2667	1	1
GeS_2	6	0.424	1	1
$MnSe_2$	6	0.1119	1	1
Br_2Ni	6	1.5247	1	1
CeI_2	6	0.1328	1	1
Br_2Mg	6	0.27	1	1
I_2Ti	6	0.2671	1	1
$NbTe_2$	6	1.5476	1	1

Table showing the first 50 lattice matching combinations with the smallest number of atoms in the supercell.

Lattice matching - minimal strain

Formula	N° atoms	strain	cell size 1	cell size 2
I_2V	435	0.0	64	81
Ga_2Se_2	403	0.0	49	64
Te_2W	183	0.0001	25	36
BiClTe	6	0.0001	1	1
$NbTe_2$	255	0.0001	36	49
CI_2Y_2	743	0.0001	81	100
$Cl_2O_2Tm_2$	609	0.0001	57	73
$Br_2Hf_2N_2$	474	0.0002	50	54
Bi_2In_2	387	0.0002	65	48
$MoTe_2$	183	0.0002	25	36
CaClHO	463	0.0003	57	73
Se_2Sn	543	0.0003	81	100
I_2Ti	543	0.0003	81	100
S_2Zr	255	0.0003	36	49
Se_2V	390	0.0003	49	81
Gd_2GeI_2	8	0.0003	1	1
NS_2Ta	433	0.0004	39	79
CdH_2O_2	255	0.0004	25	36
$Cl_2Ho_2O_2$	678	0.0004	64	81
S_2Ta	294	0.0004	37	61
Te_2Zn	183	0.0004	25	36
CdI_2	6	0.0004	1	1
$Cu_2Rb_2Te_2$	483	0.0005	65	48
FHOZn	91	0.0005	9	16
PTe_2Zr_2	674	0.0005	73	91
NSr_2	543	0.0005	81	100
S_2Sn	255	0.0006	36	49
Pd_2S_4	423	0.0006	69	36
$CrSe_2$	75	0.0006	9	16
Cl_2V	75	0.0006	9	16
CoI_2	543	0.0006	81	100
CdClHO	357	0.0007	43	57
F_2Na	390	0.0007	57	73
$HfLiS_2$	219	0.0007	25	36
$Cl_2O_2Yb_2$	609	0.0007	57	73
$F_2Se_2Y_2$	561	0.0007	73	57
$PtSe_2$	300	0.0007	43	57
Br_2La_2P	8	0.0008	1	1
Cl_2Y_2	403	0.0008	49	64
Br_2Ca	6	0.0008	1	1
$FeSe_2$	603	0.0008	70	131
CdI_2	6	0.0009	1	1
$NiTe_2$	390	0.0009	57	73
CrS_2	222	0.0009	25	49
Se_2Zr	435	0.0009	64	81
Se_2V	294	0.001	37	61
$I_2N_2Zr_2$	531	0.001	49	64
ClH_3O	705	0.001	100	81
Cl_4Cu_2	714	0.001	130	54
$Br_2Ho_2S_2$	747	0.0011	101	74

Table showing the first 50 lattice matching combinations with the smallest strain needed to build the supercell.

PrI_2 (P4/mmm)

Structural and electronic properties

**Formula**	PrI_2
**Spacegroup**	P4/mmm
**Prototype**	Zr2Cu
**Parent 3D**	I_2Pr
**Source DB**	MPDS
**DB ID**	S1300557
**DF2-C09 Binding energy [meV/ Å^2]**	16.8
**RVV10 Binding energy [meV/ Å^2]**	N/A
**Band gap (PBE) [eV]**	0.0

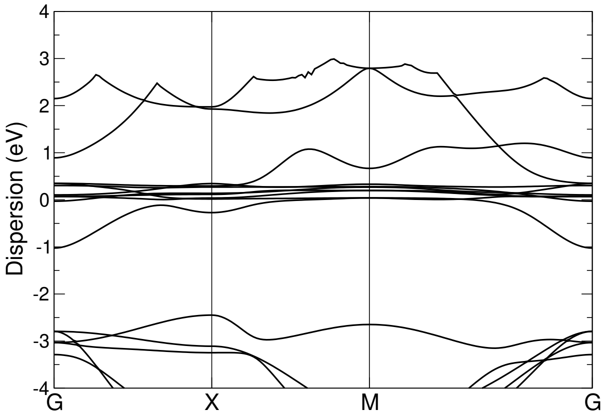

Band structure: Electronic band structure of PrI_2 (P4/mmm) in a representative window around the Fermi energy and along a high-symmetry path.

Optimized crystal structure

Structural parameters: relaxed cell (top) and atomic positions (bottom) of PrI_2 (P4/mmm) in Cartesian coordinates.

		x [Å]	y [Å]	z [Å]
$\mathbf{a_1}$		3.90606025	0.00000000	0.00000000
$\mathbf{a_2}$		0.00000000	3.90606025	0.00000000
$\mathbf{a_3}$		0.00000000	0.00000000	19.37975479
		x [Å]	y [Å]	z [Å]
●	I	0.00000000	0.00000000	-1.87002978
●	Pr	1.95303012	-1.95303012	0.00000000
●	I	0.00000000	0.00000000	1.87002978

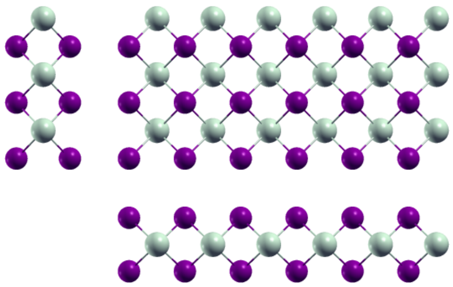

Orthographic projections: views of PrI_2 (P4/mmm) as seen from the x axis (left), the y axis (bottom) and the z axis (center).

Lattice matching - minimal size

Formula	N° atoms	strain	cell size 1	cell size 2
K	4	0.184	1	1
InSe	5	0.1456	1	1
Bi_2	5	0.151	1	1
AgTl	5	0.0224	1	1
Ag_2	5	0.191	1	1
LiO	5	0.1097	1	1
PbTe	5	0.1472	1	1
Sb_2	5	0.1324	1	1
I_2Mg	6	0.1368	1	1
S_2V	6	0.1114	1	1
MoS_2	6	0.1112	1	1
CdI_2	6	0.1488	1	1
Ba_2Pt	6	0.1907	1	1
Br_2Ca	6	0.15	1	1
CaI_2	6	0.1729	1	1
$AsSn_2$	6	0.1087	1	1
Br_2La	6	0.137	1	1
Br_2Cu	6	0.1055	1	1
Ca_2Si	6	0.1967	1	1
I_2Yb	6	0.17	1	1
BiClTe	6	0.1492	1	1
BrCdI	6	0.1393	1	1
HgI_2	6	1.1171	1	1
I_2Zn	6	0.13	1	1
BaF_2	6	0.1422	1	1
BiBrTe	6	0.1546	1	1
S_2W	6	0.1112	1	1
GeI_2	6	0.1353	1	1
AsKSn	6	0.1409	1	1
$PbTe_2$	6	0.1385	1	1
I_2Nd	6	0.001	1	1
Cl_2Cu	6	0.0985	1	1
I_2Tm	6	0.1715	1	1
$SnTe_2$	6	0.1335	1	1
Cl_2V	6	0.1103	1	1
GeI_2	6	0.1474	1	1
I_2Pb	6	0.1931	1	1
STl_2	6	0.143	1	1
$PtSe_2$	6	0.1089	1	1
BiITe	6	0.1613	1	1
GeS_2	6	0.2184	1	1
DyI_2	6	0.1762	1	1
CeI_2	6	0.0008	1	1
Se_2Yb	6	0.1355	1	1
MoS_2	6	0.1111	1	1
$BiTe_2$	6	0.1357	1	1
GdI_2	6	0.1575	1	1
$CrSe_2$	6	0.1105	1	1
I_2La	6	0.0058	1	1
$CrSe_2$	6	0.11	1	1

Table showing the first 50 lattice matching combinations with the smallest number of atoms in the supercell.

Lattice matching - minimal strain

Formula	N° atoms	strain	cell size 1	cell size 2
Mg_6	489	0.0	41	61
As_2Fe_2	643	0.0	81	100
$Hf_2Se_2Si_2$	852	0.0001	82	101
$Ag_2K_2Se_2$	486	0.0001	64	49
Mg_3	123	0.0001	16	25
Mg_4	811	0.0002	89	136
NS_2Ta	216	0.0002	20	39
CCl_2Lu_2	469	0.0002	48	65
Br_2Fe	339	0.0002	48	65
Cl_4Mn	233	0.0003	36	25
Br_2Co	339	0.0003	48	65
Cl_2Cu	588	0.0003	88	108
As_2	274	0.0003	48	65
$I_2O_2Yb_2$	9	0.0004	1	1
Bi_2Pd	438	0.0004	61	85
Br_2CsF	499	0.0004	85	61
Mg_2	407	0.0004	65	106
$Hf_2Se_2Si_2$	843	0.0005	81	100
Se_2Ta_4	933	0.0005	85	113
Tl	403	0.0005	89	136
AuI_4Li	477	0.0006	81	39
$Br_2Eu_2O_2$	9	0.0007	1	1
$NbSe_2$	597	0.0007	81	118
Ir_2P_2	7	0.0008	1	1
$C_2Cl_2Y_2$	801	0.0008	79	94
CeI_2	6	0.0008	1	1
Ca_2N	339	0.0008	48	65
O_4PSn	363	0.0008	49	36
$AsSe_2$	597	0.0008	81	118
Cl_2Zn	339	0.0008	48	65
Pd_2S_4	420	0.0008	70	35
GeS_2	543	0.0009	81	100
$Br_2O_2Sm_2$	9	0.0009	1	1
Mo_2Te_4	723	0.0009	101	70
Se_2Sn_2	624	0.0009	100	81
Au_2I_2	568	0.001	92	73
I_2Nd	6	0.001	1	1
C_2Li_2	588	0.001	84	84
Ni_2Se_2	7	0.001	1	1
BrNZr	597	0.001	81	118
O_2Sn_2	799	0.001	101	124
$Cu_2K_2Te_2$	363	0.0011	49	36
$Cu_2Na_2Te_2$	786	0.0011	100	81
$AgBrO_2$	636	0.0011	88	93
Tl	363	0.0011	79	126
$Cl_2S_2Tl_2$	708	0.0011	106	65
Se_2V	615	0.0012	79	126
$C_2I_2Y_2$	582	0.0012	62	66
P_4	497	0.0013	71	71
BN	378	0.0013	44	123

Table showing the first 50 lattice matching combinations with the smallest strain needed to build the supercell.

PrOBr (P4/nmm)

Structural and electronic properties

**Formula**	PrOBr
**Spacegroup**	P4/nmm
**Prototype**	PbClF
**Parent 3D**	$Pr_2O_2Br_2$
**Source DB**	COD
**DB ID**	2232654
**DF2-C09 Binding energy [meV/ Å^2]**	24.06
**RVV10 Binding energy [meV/ Å^2]**	31.39
**Band gap (PBE) [eV]**	N/A

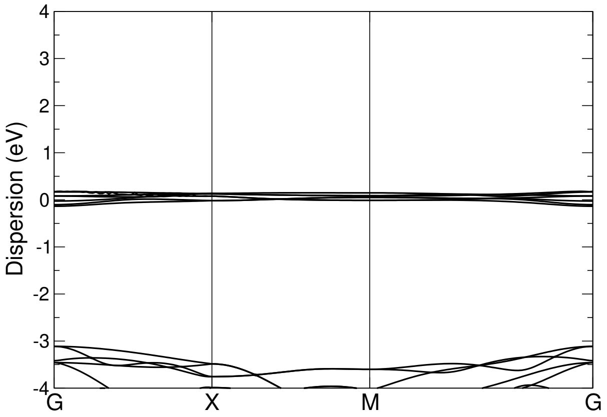

Band structure: Electronic band structure of PrOBr (P4/nmm) in a representative window around the Fermi energy and along a high-symmetry path.

Optimized crystal structure

Structural parameters: relaxed cell (top) and atomic positions (bottom) of PrOBr (P4/nmm) in Cartesian coordinates.

	x [Å]	y [Å]	z [Å]
$\mathbf{a_1}$	3.96185464	0.00000000	0.00000000
$\mathbf{a_2}$	0.00000000	3.96185464	0.00000000
$\mathbf{a_3}$	0.00000000	0.00000000	25.33672527

	x [Å]	y [Å]	z [Å]
Pr	1.98092732	0.00000000	13.88571410
Br	0.00000000	1.98092732	15.53518468
Pr	0.00000000	1.98092732	11.45101117
Br	1.98092732	0.00000000	9.80154060
O	0.00000000	0.00000000	12.66836264
O	1.98092732	1.98092732	12.66836264

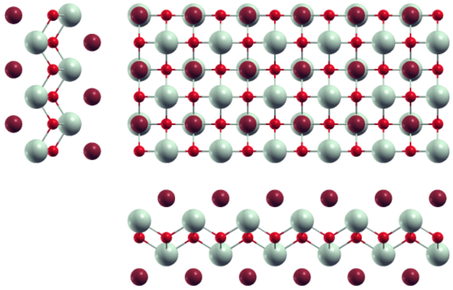

Orthographic projections: views of PrOBr (P4/nmm) as seen from the x axis (left), the y axis (bottom) and the z axis (center).

Lattice matching - minimal size

Formula	N° atoms	strain	cell size 1	cell size 2
K	7	0.1758	1	1
InSe	8	0.1397	1	1
Bi_2	8	0.1448	1	1
AgTl	8	0.0169	1	1
Ag_2	8	0.1825	1	1
LiO	8	0.1112	1	1
P_2	8	0.1095	1	1
PbTe	8	0.1412	1	1
I_2Mg	9	0.1316	1	1
CdI_2	9	0.1427	1	1
Nd	9	0.1901	1	3
Ba_2Pt	9	0.1822	1	1
Br_2Zn	9	0.1087	1	1
Br_2Ca	9	0.1438	1	1
CaI_2	9	0.1653	1	1
I_2Pr	9	0.0066	1	1
Br_2La	9	0.1319	1	1
Br_2Cu	9	0.1028	1	1
Ca_2Si	9	0.1879	1	1
I_2Yb	9	0.1625	1	1
BiClTe	9	0.143	1	1
Cl_2Ti	9	0.1095	1	1
BrCdI	9	0.134	1	1
HgI_2	9	0.3998	1	1
Te_2Ti	9	0.1088	1	1
BaF_2	9	0.1366	1	1
BiBrTe	9	0.1481	1	1
GeI_2	9	0.1303	1	1
AsKSn	9	0.1354	1	1
$PbTe_2$	9	0.1332	1	1
I_2Nd	9	0.0057	1	1
Cl_2Cu	9	0.0974	1	1
I_2Tm	9	0.164	1	1
GeI_2	9	0.1414	1	1
I_2Pb	9	0.1845	1	1
STl_2	9	0.1373	1	1
BiITe	9	0.1544	1	1
GeS_2	9	0.6125	1	1
DyI_2	9	0.1684	1	1
CeI_2	9	0.0073	1	1
Se_2Yb	9	0.1304	1	1
$BiTe_2$	9	0.1306	1	1
GdI_2	9	0.1508	1	1
I_2La	9	0.001	1	1
$CrSe_2$	9	0.1115	1	1
CdI_2	9	0.1423	1	1
I_2Pr	9	0.1431	1	1
$HfSe_2$	9	0.1088	1	1
Bi_2Te_2	10	0.7724	1	1
Bi_2In_2	10	1.1696	1	1

Table showing the first 50 lattice matching combinations with the smallest number of atoms in the supercell.

Lattice matching - minimal strain

Formula	N° atoms	strain	cell size 1	cell size 2
$Br_2N_2Zr_2$	678	0.0003	48	65
CBr_2Lu_2	613	0.0003	48	65
$Br_2Lu_2S_2$	42	0.0003	4	3
$Br_2Lu_2S_2$	42	0.0004	4	3
$Hf_2Se_2Si_2$	870	0.0004	64	81
Ag_2Br_2	10	0.0005	1	1
$Ba_2F_2I_2$	690	0.0005	65	50
$Ag_2K_2Se_2$	870	0.0006	81	64
$Ba_2F_2I_2$	678	0.0006	64	49
O_4PTl	690	0.0006	65	50
P_4	354	0.0007	35	36
Br_2OV	782	0.0007	71	89
As_2Rh_2	10	0.0007	1	1
H_2Li_2Pd	833	0.0007	58	97
Br_2CsF	438	0.0007	49	36
As_2Fe_2	708	0.0009	64	81
Bi_2Se_4	318	0.0009	36	17
Tl	121	0.0009	16	25
$Cu_2Rb_2Te_2$	510	0.0009	49	36
O_4PTl	678	0.001	64	49
I_2La	9	0.001	1	1
Br_2Zr_2	958	0.001	81	118
$Ge_2Mn_2Sr_2$	12	0.0011	1	1
$I_2N_2Ti_2$	102	0.0011	8	9
NbS_2	852	0.0011	79	126
O_2Zn	237	0.0011	20	39
Mg_2	542	0.0012	58	97
Mg_4	196	0.0013	16	25
Bi_2In_2	438	0.0013	49	36
H_4Ti	833	0.0013	58	97
S_2Ta	852	0.0013	79	126
Cl_4Mn	815	0.0013	85	61
$LiNbS_2$	978	0.0013	79	126
Se_2Ti	840	0.0014	81	118
$As_2Cd_2K_2$	678	0.0014	64	49
$Cl_2H_2Lu_2$	678	0.0014	48	65
O_2Sn_2	840	0.0014	76	96
Bi_2Pd	258	0.0015	25	36
H_4Ti	699	0.0015	49	81
K	707	0.0015	103	89
$NbTe_2$	483	0.0015	48	65
Br_2Mn	531	0.0015	52	73
KS_2Ti	604	0.0015	52	73
K	611	0.0016	89	77
In	70	0.0016	9	16
$I_2S_2Sm_2$	996	0.0016	89	77
Ga_2Se_2	680	0.0016	62	77
$H_2I_2Yb_2$	12	0.0016	1	1
Se_2Ta_4	510	0.0016	36	49
Bi_2Se_4	282	0.0016	32	15

Table showing the first 50 lattice matching combinations with the smallest strain needed to build the supercell.

PtO_2 (P-3m1)

Structural and electronic properties

**Formula**	PtO_2
**Spacegroup**	P-3m1
**Prototype**	CdI2
**Parent 3D**	Pt_2O_4
**Source DB**	ICSD
**DB ID**	24923
**DF2-C09 Binding energy [meV/ Å^2]**	19.29
**RVV10 Binding energy [meV/ Å^2]**	32.25
**Band gap (PBE) [eV]**	1.69

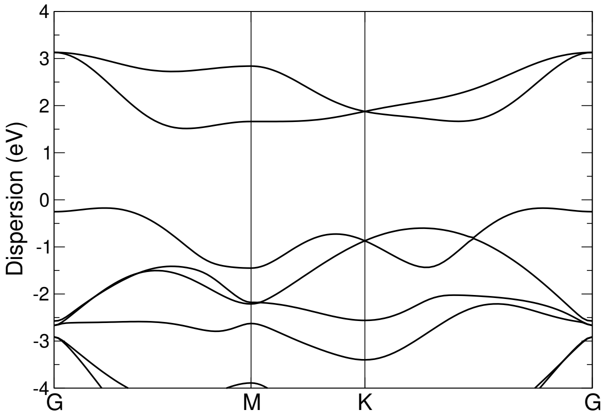

Band structure: Electronic band structure of PtO_2 (P-3m1) in a representative window around the Fermi energy and along a high-symmetry path.

Optimized crystal structure

Structural parameters: relaxed cell (top) and atomic positions (bottom) of PtO_2 (P-3m1) in Cartesian coordinates.

		x [Å]	y [Å]	z [Å]
$\mathbf{a_1}$		3.15008804	0.00000000	0.00000000
$\mathbf{a_2}$		−1.57504402	2.72805627	0.00000000
$\mathbf{a_3}$		0.00000000	0.00000000	21.89270767
		x [Å]	y [Å]	z [Å]
•	O	1.57504402	0.90935209	11.89074023
•	Pt	0.00000000	0.00000000	10.94635383
•	O	0.00000000	1.81870418	10.00196743

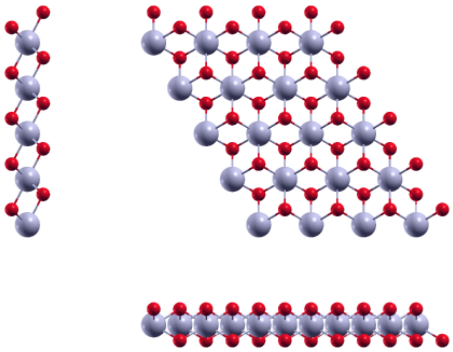

Orthographic projections: views of PtO_2 (P-3m1) as seen from the x axis (left), the y axis (bottom) and the z axis (center).

Lattice matching - minimal size

Formula	N° atoms	strain	cell size 1	cell size 2
Sn	4	0.144	1	1
Na	4	0.4775	1	1
In	4	0.148	1	1
In	4	0.0015	1	1
As_2	5	0.4984	1	1
Cl_2Zn	6	0.4956	1	1
S_2V	6	0.0048	1	1
MoS_2	6	0.0055	1	1
$MoTe_2$	6	0.4834	1	1
PSn_2	6	3.0007	1	1
HfS_2	6	2.9192	1	1
FeO_2	6	0.2632	1	1
Te_2V	6	2.8355	1	1
S_2Zr	6	2.9905	1	1
NiO_2	6	0.265	1	1
Br_2Co	6	0.4973	1	1
ReS_2	6	0.0071	1	1
CrO_2	6	0.2475	1	1
Ca_2N	6	0.4998	1	1
MnO_2	6	0.2504	1	1
Te_2Zn	6	0.4829	1	1
S_2W	6	0.0056	1	1
Bi_2Pd	6	0.2065	1	1
Br_2Mn	6	2.855	1	1
Cl_2Ni	6	0.4488	1	1
$CrTe_2$	6	0.4639	1	1
PtS_2	6	0.4802	1	1
$CoTe_2$	6	2.9234	1	1
CdClO	6	0.4871	1	1
Se_2Ti	6	0.4734	1	1
Br_2Ti	6	0.4624	1	1
Te_2W	6	0.4838	1	1
$AsSe_2$	6	0.4523	1	1
$NiTe_2$	6	3.1964	1	1
S_2Sn	6	2.994	1	1
OTl_2	6	2.8317	1	1
BrNZr	6	0.4556	1	1
$NbSe_2$	6	0.4498	1	1
Br_2Fe	6	0.4974	1	1
Br_2Cr	6	0.4632	1	1
$FeSe_2$	6	0.1384	1	1
Se_2Ta	6	0.45	1	1
$NbTe_2$	6	2.9878	1	1
$NbSe_2$	6	0.453	1	1
MoS_2	6	0.0057	1	1
$CrSe_2$	6	0.0085	1	1
Se_2Ta	6	0.4605	1	1
N_2Re	6	0.2738	1	1
CoO_2	6	0.2642	1	1
Fe_2Te_2	7	0.319	1	1

Table showing the first 50 lattice matching combinations with the smallest number of atoms in the supercell.

Lattice matching - minimal strain

Formula	N° atoms	strain	cell size 1	cell size 2
$CuTe_2$	300	0.0	57	43
$Cl_2H_2Sc_2$	786	0.0	100	81
$MoTe_2$	390	0.0001	73	57
CoO_2	492	0.0001	73	91
Cl_2Zn	339	0.0001	64	49
Br_2Gd_2	331	0.0001	61	37
Te_2Zn	390	0.0001	73	57
As_2Sn_2	331	0.0002	61	37
S_2Sn	255	0.0002	49	36
Te_2W	390	0.0002	73	57
AsKSn	75	0.0002	16	9
Cu_2I_2	197	0.0002	39	20
Se_4TiZr	255	0.0003	49	18
Se_2Ta	543	0.0003	100	81
$Br_2H_2Sr_2$	237	0.0003	39	20
PSn_2	255	0.0003	49	36
I_2Y_2	139	0.0003	25	16
$LiMnSe_2$	139	0.0003	25	16
HfS_2	300	0.0004	57	43
F_2Na	183	0.0004	36	25
$HfLiS_2$	447	0.0004	73	57
$F_2Se_2Tm_2$	171	0.0004	25	16
$Cl_2O_2Yb_2$	258	0.0004	36	25
Br_2Er_2	331	0.0004	61	37
CaH_2O_2	386	0.0004	57	43
$Cl_2O_2Tm_2$	258	0.0004	36	25
S_2Zr	255	0.0005	49	36
CaI_2	354	0.0005	79	39
NiO_2	492	0.0005	73	91
Br_2Ti	543	0.0005	100	81
O_4PSn	714	0.0005	130	54
CaClHO	208	0.0006	36	25
Na	307	0.0006	81	64
Cl_2La_2	439	0.0006	81	49
CdH_2O_2	504	0.0007	73	57
$NbTe_2$	255	0.0007	49	36
$Br_2H_2Zr_2$	786	0.0007	100	81
$Cl_2N_2Zr_2$	429	0.0007	57	43
$CoTe_2$	300	0.0007	57	43
Br_2Co	339	0.0007	64	49
Mo_2Te_4	411	0.0007	77	30
I_2Tm	354	0.0007	79	39
KS_2Ti	388	0.0007	64	49
Br_2Fe	339	0.0008	64	49
I_2La_2	84	0.0008	16	9
$As_2Cd_2K_2$	714	0.0008	130	54
Ge_2S_2	288	0.0008	56	30
Br_2Cr	543	0.0008	100	81
FeO_2	492	0.0008	73	91
Cl_2Tb_2	139	0.0008	25	16

Table showing the first 50 lattice matching combinations with the smallest strain needed to build the supercell.

PtS_2 (P-3m1)

Structural and electronic properties

**Formula**	PtS_2
**Spacegroup**	P-3m1
**Prototype**	CdI2
**Parent 3D**	PtS_2
**Source DB**	COD
**DB ID**	5910085
**DF2-C09 Binding energy [meV/ Å^2]**	27.31
**RVV10 Binding energy [meV/ Å^2]**	33.0
**Band gap (PBE) [eV]**	1.78

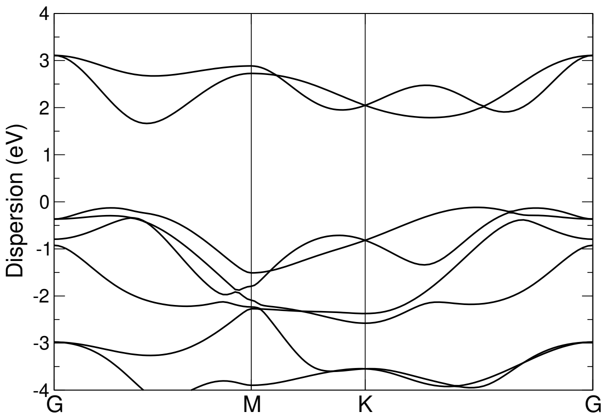

Band structure: Electronic band structure of PtS_2 (P-3m1) in a representative window around the Fermi energy and along a high-symmetry path.

Optimized crystal structure

Structural parameters: relaxed cell (top) and atomic positions (bottom) of PtS_2 (P-3m1) in Cartesian coordinates.

		x [Å]	y [Å]	z [Å]
$\mathbf{a_1}$		3.55611114	−0.00000000	0.00000000
$\mathbf{a_2}$		−1.77805557	3.07968259	0.00000000
$\mathbf{a_3}$		0.00000000	0.00000000	22.47156614
		x [Å]	y [Å]	z [Å]
●	S	-0.00000000	2.05312172	9.99934757
●	Pt	0.00000000	-0.00000000	11.23578307
●	S	1.77805557	1.02656086	12.47221856

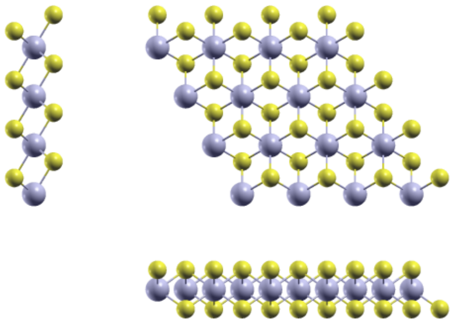

Orthographic projections: views of PtS_2 (P-3m1) as seen from the x axis (left), the y axis (bottom) and the z axis (center).

Lattice matching - minimal size

Formula	N° atoms	strain	cell size 1	cell size 2
K	4	0.3247	1	1
Tl	4	0.125	1	1
K	4	13.6272	1	1
Na	4	0.001	1	1
In	4	0.1114	1	1
In	4	0.2592	1	1
Gd	4	0.114	1	1
HgO	5	0.1335	1	1
As_2	5	0.007	1	1
S_2	5	0.4479	1	1
LiO	5	0.2743	1	1
Mg_2	5	0.1161	1	1
Sb_2	5	2.9289	1	1
CrS_2	6	1.5639	1	1
Cl_2Zn	6	0.0059	1	1
I_2Mg	6	3.004	1	1
S_2V	6	0.2636	1	1
MoS_2	6	0.2647	1	1
CdI_2	6	3.194	1	1
$MoTe_2$	6	0.0012	1	1
$AgTe_2$	6	0.127	1	1
$HfTe_2$	6	0.4669	1	1
Te_2V	6	0.0032	1	1
$CuTe_2$	6	0.0095	1	1
Br_2La	6	3.0085	1	1
Br_2Cu	6	1.082	1	1
Br_2Co	6	0.0066	1	1
ReS_2	6	0.2474	1	1
Ca_2N	6	0.0076	1	1
$AuTe_2$	6	0.4868	1	1
$PdTe_2$	6	0.4804	1	1
Mg_3	6	0.1215	1	1
I_2Zn	6	0.499	1	1
Te_2Zn	6	0.0011	1	1
S_2W	6	0.2648	1	1
Bi_2Pd	6	0.1412	1	1
GeI_2	6	2.9792	1	1
Br_2Mn	6	0.0047	1	1
$CrTe_2$	6	0.0063	1	1
CdClO	6	0.0027	1	1
Ba_2N	6	0.4707	1	1
Se_2Ti	6	0.0026	1	1
Br_2Ti	6	0.0069	1	1
Te_2Zr	6	0.4681	1	1
Te_2W	6	0.0014	1	1
Cl_2V	6	0.2706	1	1
OTl_2	6	0.0029	1	1
Br_2Fe	6	0.0066	1	1
Br_2Cr	6	0.0066	1	1
$FeSe_2$	6	0.4233	1	1

Table showing the first 50 lattice matching combinations with the smallest number of atoms in the supercell.

Lattice matching - minimal strain

Formula	N° atoms	strain	cell size 1	cell size 2
$HfTe_2$	492	0.0	91	73
In_2Se_3	638	0.0	91	73
Br_2Ho_2	447	0.0	73	57
Sb_2Se_2Te	327	0.0	49	36
$I_2S_2Sm_2$	537	0.0	81	49
In	273	0.0001	64	81
$LiMnTe_2$	208	0.0002	36	25
Bi_2STe_2	233	0.0002	36	25
In_2Se_2	388	0.0002	64	49
O_2Sn_2	387	0.0002	65	48
Bi_2S_3	504	0.0002	73	57
$Er_2F_2Se_2$	711	0.0002	91	73
MoS_2	492	0.0003	73	91
S_2W	492	0.0003	73	91
$Ce_2I_2Si_2$	363	0.0003	49	36
K	292	0.0004	81	49
IKO_3	17	0.0004	4	1
$F_2Ho_2Se_2$	627	0.0004	81	64
I_4Sr_2	150	0.0005	32	9
MoS_2	492	0.0005	73	91
GeI_2	183	0.0005	36	25
Cu_2S_2	387	0.0005	65	48
Sb_2Se_2Te	327	0.0005	49	36
S_2V	492	0.0005	73	91
Br_2Tb_2	447	0.0005	73	57
Te_2Zr	492	0.0005	91	73
P_4	229	0.0005	39	28
I_2Mg	255	0.0005	49	36
$Br_2O_2Y_2$	483	0.0006	65	48
C_4Ca_2	744	0.0006	108	70
CdI_2	183	0.0006	36	25
$Br_2Dy_2O_2$	483	0.0006	65	48
NS_2Zr	7	0.0006	1	1
Br_2PY_2	563	0.0006	81	64
$K_2Mn_2Sb_2$	840	0.0006	118	81
$Br_2Ce_2O_2$	840	0.0006	118	81
H_2NiO_2	743	0.0007	81	100
As_2CeLi_2	233	0.0007	36	25
$F_2Lu_2Se_2$	429	0.0007	57	43
Ga_2S_3	8	0.0007	1	1
Br_2CsF	197	0.0007	39	20
$I_2S_2Sm_2$	405	0.0007	61	37
PbTe	158	0.0007	36	25
Br_2La_2P	233	0.0007	36	25
Pt_2Te_2	565	0.0007	91	73
MnO_2	390	0.0007	49	81
I_2La_2Sb	488	0.0008	81	49
$HfLiS_2$	7	0.0008	1	1
$GeNi_3Te_2$	786	0.0008	100	81
$Eu_2I_2O_2$	840	0.0008	118	81

Table showing the first 50 lattice matching combinations with the smallest strain needed to build the supercell.

$PtSe_2$ (P-3m1)

Structural and electronic properties

**Formula**	$PtSe_2$
**Spacegroup**	P-3m1
**Prototype**	CdI2
**Parent 3D**	$PtSe_2$
**Source DB**	COD
**DB ID**	9009117
**DF2-C09 Binding energy [meV/ Å^2]**	29.63
**RVV10 Binding energy [meV/ Å^2]**	35.16
**Band gap (PBE) [eV]**	1.34

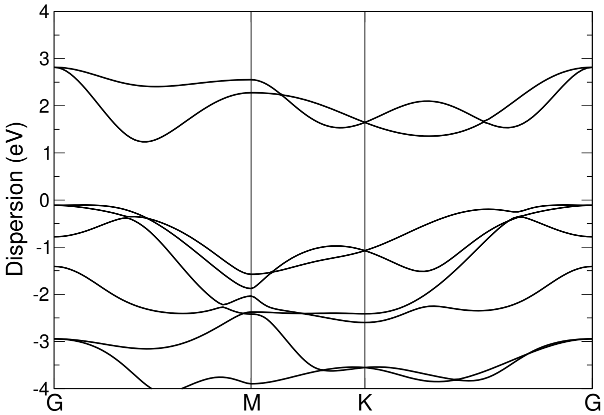

Band structure: Electronic band structure of $PtSe_2$ (P-3m1) in a representative window around the Fermi energy and along a high-symmetry path.

Optimized crystal structure

Structural parameters: relaxed cell (top) and atomic positions (bottom) of $PtSe_2$ (P-3m1) in Cartesian coordinates.

	x [Å]	y [Å]	z [Å]
$\mathbf{a_1}$	3.71203994	0.00000000	0.00000000
$\mathbf{a_2}$	−1.85601997	3.21472089	0.00000000
$\mathbf{a_3}$	0.00000000	0.00000000	22.55693476

		x [Å]	y [Å]	z [Å]
●	Se	0.00000000	2.14314726	12.61133017
●	Pt	-0.00000000	-0.00000000	11.27846738
●	Se	1.85601997	1.07157363	9.94560459

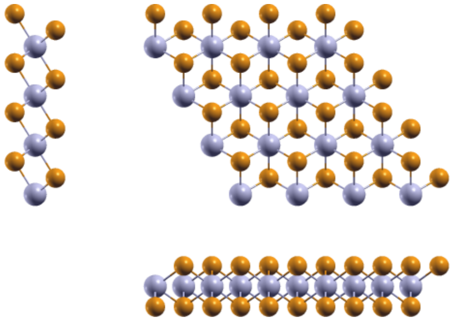

Orthographic projections: views of $PtSe_2$ (P-3m1) as seen from the x axis (left), the y axis (bottom) and the z axis (center).

Lattice matching - minimal size

Formula	N° atoms	strain	cell size 1	cell size 2
Tl	4	0.1144	1	1
Tl	4	0.2636	1	1
In	4	1.5242	1	1
InSe	5	0.4962	1	1
HgO	5	0.1202	1	1
P_2	5	0.2563	1	1
PbTe	5	0.5009	1	1
Mg_2	5	0.4285	1	1
Sb_2	5	0.4553	1	1
Sm	5	0.2197	1	2
I_2Mg	6	0.4695	1	1
S_2V	6	1.547	1	1
MoS_2	6	1.5522	1	1
Cl_2Mn	6	0.2719	1	1
CdI_2	6	2.9183	1	1
$AgTe_2$	6	0.1157	1	1
PSn_2	6	0.0044	1	1
$MoSe_2$	6	0.2625	1	1
S_2Ta	6	0.275	1	1
Br_2Zn	6	0.0063	1	1
Br_2Ca	6	2.9343	1	1
$AsSn_2$	6	0.0006	1	1
I_2Pr	6	0.2071	1	1
S_2Zr	6	0.0051	1	1
Br_2La	6	0.4704	1	1
Br_2Cu	6	0.9812	1	1
I_2Yb	6	3.1936	1	1
BiClTe	6	2.923	1	1
Cl_2Ti	6	0.2565	1	1
BrCdI	6	0.4775	1	1
HgI_2	6	0.3183	1	1
Mg_3	6	0.1121	1	1
Te_2Ti	6	0.0066	1	1
NbS_2	6	0.2743	1	1
I_2Zn	6	0.4473	1	1
BaF_2	6	0.4861	1	1
$RhTe_2$	6	0.0022	1	1
S_2W	6	1.5527	1	1
Bi_2Pd	6	0.1259	1	1
GeI_2	6	0.4648	1	1
NbS_2	6	0.268	1	1
S_2Ta	6	0.2667	1	1
Se_2V	6	0.2648	1	1
AsKSn	6	0.4824	1	1
$PbTe_2$	6	0.475	1	1
I_2Nd	6	0.2084	1	1
S_2Sn	6	0.0049	1	1
$SnTe_2$	6	0.4592	1	1
Cl_2V	6	1.582	1	1
GeI_2	6	0.5016	1	1

Table showing the first 50 lattice matching combinations with the smallest number of atoms in the supercell.

Lattice matching - minimal strain

Formula	N° atoms	strain	cell size 1	cell size 2
Br_2La_2P	386	0.0	57	43
S_2V	255	0.0	36	49
Sb_2Se_2Te	638	0.0	91	73
ClH_3O	368	0.0	61	37
BiBrTe	255	0.0	49	36
CNb_2S_2	536	0.0001	57	73
$CeLi_2P_2$	705	0.0001	100	81
CdI_2	300	0.0001	57	43
$CrSe_2$	300	0.0002	43	57
$MnNaTe_2$	499	0.0002	81	64
$I_2La_2Si_2$	486	0.0002	64	49
NbS_2	543	0.0002	81	100
As_2Li_2Pr	437	0.0003	64	49
CdI_2	300	0.0003	57	43
InSe	290	0.0003	64	49
AsKSn	390	0.0003	73	57
$BiTe_2$	492	0.0003	91	73
Se_2V	492	0.0003	73	91
CoH_2O_2	255	0.0004	25	36
As_2Li_2Nd	437	0.0004	64	49
$I_2Pr_2S_2$	171	0.0004	25	16
$PbTe_2$	435	0.0004	81	64
$HNiO_2$	148	0.0004	16	25
Gd_2GeI_2	386	0.0004	57	43
Sb_2Se_2Te	638	0.0005	91	73
C_2F_2	433	0.0005	39	79
I_2Pb	123	0.0005	25	16
Tl	310	0.0005	73	91
$Cl_2H_2Zr_2$	843	0.0005	81	100
Br_2HLa	624	0.0005	100	81
SSb_2Te_2	563	0.0005	81	64
BH_4Li	786	0.0005	100	81
Bi_2STe_2	386	0.0005	57	43
BrCdI	435	0.0006	81	64
Se_2Yb	492	0.0006	91	73
GeI_2Y_2	437	0.0006	64	49
$Ba_2Ge_2Mn_2$	483	0.0006	65	48
P_2	317	0.0006	57	73
$AsLi_3$	388	0.0006	64	49
S_2Ta	543	0.0006	81	100
$AsSn_2$	6	0.0006	1	1
BiClTe	300	0.0007	57	43
$F_2I_2Tm_2$	483	0.0007	65	48
MoS_2	255	0.0007	36	49
I_2Pr	300	0.0007	57	43
$Gd_2I_2Se_2$	291	0.0008	49	24
Cl_2Ti	390	0.0008	57	73
$SnTe_2$	543	0.0008	100	81
S_2W	255	0.0008	36	49
$Ce_2I_2Si_2$	711	0.0008	91	73

Table showing the first 50 lattice matching combinations with the smallest strain needed to build the supercell.

RbCl (P4/nmm)

Structural and electronic properties

**Formula**	RbCl
**Spacegroup**	P4/nmm
**Prototype**	FeSe
**Parent 3D**	$Rb_2Ni_2C_4N_4Cl_2$
**Source DB**	ICSD
**DB ID**	380471
**DF2-C09 Binding energy [meV/ Å^2]**	14.43
**RVV10 Binding energy [meV/ Å^2]**	18.67
**Band gap (PBE) [eV]**	4.61

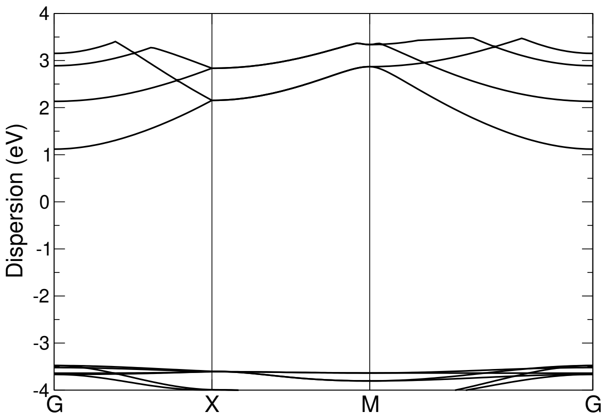

Band structure: Electronic band structure of RbCl (P4/nmm) in a representative window around the Fermi energy and along a high-symmetry path.

Optimized crystal structure

Structural parameters: relaxed cell (top) and atomic positions (bottom) of RbCl (P4/nmm) in Cartesian coordinates.

	x [Å]	y [Å]	z [Å]
$\mathbf{a_1}$	6.45465690	0.00000000	0.00000000
$\mathbf{a_2}$	0.00000000	6.45465690	0.00000000
$\mathbf{a_3}$	0.00000000	0.00000000	20.03971303

		x [Å]	y [Å]	z [Å]
●	Cl	0.00000000	0.00000000	10.03476412
●	Rb	0.00000000	3.22732845	10.01985651
●	Rb	3.22732845	0.00000000	10.01985651
●	Cl	3.22732845	3.22732845	10.00494890

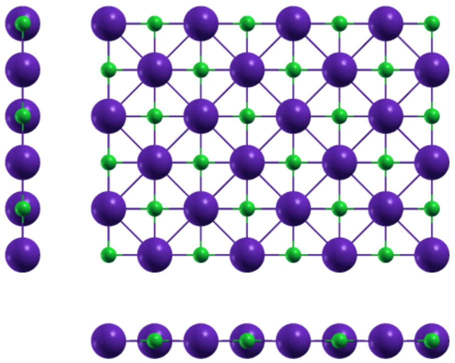

Orthographic projections: views of RbCl (P4/nmm) as seen from the x axis (left), the y axis (bottom) and the z axis (center).

Lattice matching - minimal size

Formula	N° atoms	strain	cell size 1	cell size 2
Br_3Cs	8	0.1321	1	1
Na	8	0.1507	1	4
I_3Sn	8	0.2157	1	1
IO_3Tl	9	0.1293	1	1
Hg_3N_2	9	0.1422	1	1
$ClKO_3$	9	0.1346	1	1
IKO_3	9	0.1513	1	1
Hg_3S_2	9	0.1809	1	1
$BrKO_3$	9	0.1461	1	1
Br_2Zn	10	0.1486	1	2
$AsSn_2$	10	0.1461	1	2
$SiTe_2$	10	0.1503	1	2
Hg_4O_2	10	0.2461	1	1
Te_2Ti	10	0.1487	1	2
$RhTe_2$	10	0.1471	1	2
Pd_2S_4	10	0.5711	1	1
$CoTe_2$	10	0.1432	1	2
Cl_4Mg_2	10	0.1273	1	1
Cl_2Cu	10	0.1956	1	2
I_2V	10	0.1509	1	2
Se_2Zr	10	0.1505	1	2
$PtSe_2$	10	0.1463	1	2
Br_2Ni	10	0.1437	1	2
Cl_2Mg	10	0.1437	1	2
Cl_4KTl	10	0.009	1	1
$HfSe_2$	10	0.1487	1	2
CdClHO	12	0.1457	1	2
$AgNO_2$	12	0.4895	1	2
HgO	12	0.0027	1	4
CdClHO	12	0.1468	1	2
Cl_2Y_2	12	0.1481	1	2
Ga_2Se_2	12	0.1516	1	2
Ga_2S_2	12	0.1433	1	2
Ga_2Se_2	12	0.1479	1	2
Ga_2S_2	12	0.1436	1	2
BrCdI	13	0.1604	1	3
BaF_2	13	0.164	1	3
AsKSn	13	0.1624	1	3
$SnTe_2$	13	0.153	1	3
STl_2	13	0.165	1	3
DyI_2	13	0.7727	1	3
Hg_3S_2	13	0.1518	2	1
Hg_4O_2	14	0.0993	2	1
PTe_2Ti_2	14	0.1436	1	2
AuI_4Li	14	0.1437	2	1
$MoTe_2$	16	0.1529	1	4
$AgTe_2$	16	0.0064	1	4
Cl_2Gd_2	16	0.1332	1	3
$Au_2K_2S_2$	16	1.1129	1	2
$C_2Br_2Y_2$	16	0.168	1	2

Table showing the first 50 lattice matching combinations with the smallest number of atoms in the supercell.

Lattice matching - minimal strain

Formula	N° atoms	strain	cell size 1	cell size 2
$I_2Nd_2O_2$	484	0.0001	25	64
F_2Zn	111	0.0001	9	25
Fe_2S_2	424	0.0002	25	81
$Br_2Ca_2F_2$	186	0.0003	9	25
$AgClO_4$	132	0.0003	9	16
$Br_2Gd_2O_2$	186	0.0003	9	25
$Ca_2H_2I_2$	670	0.0004	34	89
$Ba_2F_2I_2$	796	0.0004	49	100
Cu_2Se_2	260	0.0004	16	49
GeI_2La_2	866	0.0004	54	130
Br_2CsF	296	0.0004	25	49
$Br_2Ce_2O_2$	670	0.0005	34	89
$K_2Mn_2Sb_2$	670	0.0005	34	89
O_4PTl	796	0.0006	49	100
$I_2S_2Tl_2$	358	0.0007	16	49
I_2Tm	606	0.0007	54	130
I_2Yb	606	0.0007	54	130
$Br_2F_2Yb_2$	186	0.0007	9	25
$Cl_2F_2Pb_2$	484	0.0008	25	64
$Bi_2I_2O_2$	484	0.0009	25	64
Ba_2Cu_2	736	0.0009	54	130
$Cu_2Na_2Se_2$	484	0.0009	25	64
K_2PtTe_2	300	0.0009	35	32
F_2Ni	211	0.001	16	49
$Si_2Te_2Zr_2$	358	0.001	16	49
$F_2I_2Tm_2$	484	0.001	25	64
$I_2Lu_2O_2$	186	0.001	9	25
$Eu_2I_2O_2$	670	0.001	34	89
$Ge_2Se_2Zr_2$	944	0.001	41	130
Ca_2Cl_2	684	0.001	41	130
$Br_2F_2Tm_2$	186	0.0011	9	25
I_2Tm	537	0.0011	48	115
CaI_2	537	0.0012	48	115
$H_2I_2Sr_2$	70	0.0012	4	9
GeI_2La_2	767	0.0012	48	115
$Ca_2Ge_2Mn_2$	186	0.0012	9	25
$Br_2O_2V_2$	72	0.0012	3	10
Br_2Cu_2	136	0.0013	9	25
$HNiO_2$	308	0.0013	12	65
$As_2Mg_2Na_2$	70	0.0013	4	9
Sb_2Se_2Te	791	0.0013	44	123
AuI_4Li	688	0.0013	58	76
$Ce_2I_2Si_2$	914	0.0013	44	123
As_2Ir_2	492	0.0014	34	89
Sb_2Se_2Te	791	0.0014	44	123
I_4Sr_2	820	0.0014	79	84
I_2Mg	545	0.0014	44	123
$Cl_2S_2Tl_2$	814	0.0014	58	97
Ag_2I_2	648	0.0014	53	109
$Ba_2H_2I_2$	394	0.0015	25	49

Table showing the first 50 lattice matching combinations with the smallest strain needed to build the supercell.

$ReSe_2$ (P-6m2)

Structural and electronic properties

**Formula**	$ReSe_2$
**Spacegroup**	P-6m2
**Prototype**	MoS2
**Parent 3D**	Re_4Se_8
**Source DB**	ICSD
**DB ID**	650091
**DF2-C09 Binding energy [meV/ Å^2]**	28.29
**RVV10 Binding energy [meV/ Å^2]**	30.28
**Band gap (PBE) [eV]**	N/A

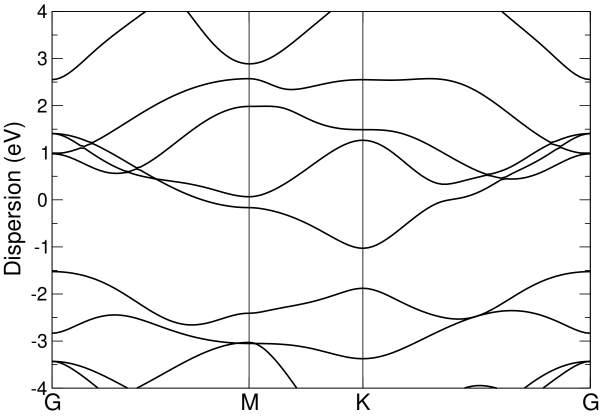

Band structure: Electronic band structure of $ReSe_2$ (P-6m2) in a representative window around the Fermi energy and along a high-symmetry path.

Optimized crystal structure

Structural parameters: relaxed cell (top) and atomic positions (bottom) of $ReSe_2$ (P-6m2) in Cartesian coordinates.

		x [Å]	y [Å]	z [Å]
$\mathbf{a_1}$		3.45634959	0.00000000	0.00000000
$\mathbf{a_2}$		−1.72817479	2.99328655	0.00000000
$\mathbf{a_3}$		0.00000000	0.00000000	23.15151602
		x [Å]	y [Å]	z [Å]
●	Re	0.00000000	0.00000000	11.57575801
●	Se	1.72817479	0.99776218	10.03078381
●	Se	1.72817479	0.99776218	13.12073222

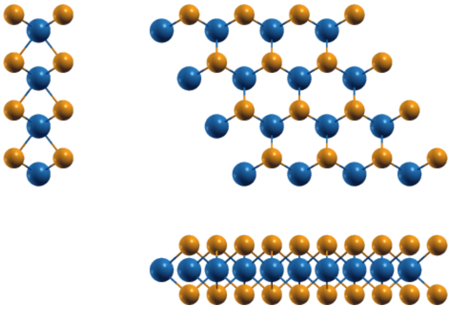

Orthographic projections: views of $ReSe_2$ (P-6m2) as seen from the x axis (left), the y axis (bottom) and the z axis (center).

Lattice matching - minimal size

Formula	N° atoms	strain	cell size 1	cell size 2
Tl	4	0.1345	1	1
Sn	4	0.1149	1	1
In	4	0.1169	1	1
HgO	5	0.1446	1	1
AsSb	5	0.4622	1	1
GeTe	5	0.4775	1	1
S_2	5	0.4816	1	1
Mg_2	5	0.1232	1	1
$IrTe_2$	6	0.4799	1	1
CrS_2	6	0.2574	1	1
I_2Mg	6	3.1891	1	1
$CdCl_2$	6	0.4748	1	1
$AgTe_2$	6	0.1368	1	1
$InSe_2$	6	0.4758	1	1
$GeTe_2$	6	0.4723	1	1
$SiTe_2$	6	0.4472	1	1
$HfTe_2$	6	2.9003	1	1
I_2Mn	6	0.4751	1	1
Br_2La	6	3.1939	1	1
NSr_2	6	0.4663	1	1
PbS_2	6	0.4557	1	1
ReS_2	6	0.2658	1	1
$AuTe_2$	6	3.0026	1	1
$PdTe_2$	6	2.97	1	1
FeI_2	6	0.4695	1	1
I_2Ni	6	0.4727	1	1
S_2Ti	6	0.006	1	1
Mg_3	6	0.1301	1	1
CrI_2	6	0.4684	1	1
Bi_2Pd	6	0.1536	1	1
Ba_2Hg	6	0.3216	1	1
N_2W	6	0.2483	1	1
Cl_2Ni	6	0.0009	1	1
Cl_2Co	6	0.0063	1	1
$CrTe_2$	6	0.0071	1	1
Br_2V	6	0.0008	1	1
ClNZr	6	0.0029	1	1
Cl_2Fe	6	0.007	1	1
Ba_2N	6	2.9202	1	1
Br_2Ti	6	0.0065	1	1
Te_2Zr	6	2.9067	1	1
$AsSe_2$	6	0.0024	1	1
I_2V	6	0.4504	1	1
Se_2Zr	6	0.4482	1	1
CdO_2	6	0.0064	1	1
BrNZr	6	0.0037	1	1
$NbSe_2$	6	0.0013	1	1
CoI_2	6	0.4633	1	1
Br_2Cr	6	0.0068	1	1
Cl_2Zr	6	0.0066	1	1

Table showing the first 50 lattice matching combinations with the smallest number of atoms in the supercell.

Lattice matching - minimal strain

Formula	N° atoms	strain	cell size 1	cell size 2
CoH_2O_2	674	0.0	73	91
$PtTe_2$	255	0.0	49	36
$I_2Nd_2S_2$	102	0.0001	16	9
$InSe_2$	435	0.0001	81	64
Sb_2Se_2Te	233	0.0001	36	25
Cu_3Se_3	429	0.0002	57	43
H_2Si_2	499	0.0002	81	64
Cu_4Te_2	171	0.0002	25	16
$Cl_2Er_2H_2$	627	0.0002	81	64
NSr_2	492	0.0003	91	73
PTe_2Zr_2	705	0.0003	100	81
P_2Sn_2	447	0.0003	73	57
Sb_2Se_2Te	233	0.0003	36	25
I_2Mn	435	0.0003	81	64
$Ge_2Hf_2Te_2$	483	0.0004	65	48
$Ge_2I_2La_2$	405	0.0004	61	37
AsSb	462	0.0004	100	81
C_2Li_2	548	0.0004	96	65
$AuTe_2$	255	0.0004	49	36
Ba_2N	300	0.0004	57	43
FeH_2O_2	467	0.0004	49	64
$Ce_2I_2Si_2$	258	0.0005	36	25
$F_2Se_2Yb_2$	561	0.0005	73	57
Ni_2SbTe_2	504	0.0005	73	57
$CdCl_2$	435	0.0005	81	64
Ag_2I_2	197	0.0005	39	20
Se_2Sn	492	0.0005	91	73
NS_2Ta	357	0.0005	43	57
C_2	173	0.0005	25	49
Gd	75	0.0006	16	27
Te_2Zr	300	0.0006	57	43
MnO_2	123	0.0006	16	25
GeTe	371	0.0006	81	64
S_2	333	0.0006	73	57
$Ca_2Ge_2Mn_2$	840	0.0006	118	81
$I_2Lu_2O_2$	840	0.0006	118	81
CrI_2	492	0.0006	91	73
$Br_2F_2Tm_2$	840	0.0006	118	81
CI_2Y_2	638	0.0007	91	73
Ni_2Te_2	499	0.0007	81	64
I_2Mg	183	0.0007	36	25
$BiTe_2$	183	0.0007	36	25
S_2Zn_2	447	0.0007	73	57
N_2W	339	0.0008	49	64
Sb_2Te_3	155	0.0008	25	16
In_2S_3	563	0.0008	81	64
Br_2V	6	0.0008	1	1
CoI_2	543	0.0008	100	81
Br_2Mg	492	0.0009	91	73
$Bi_2Cl_2O_2$	840	0.0009	118	81

Table showing the first 50 lattice matching combinations with the smallest strain needed to build the supercell.

RhTeCl ($P2_1/m$)

Structural and electronic properties

**Formula**	RhTeCl
**Spacegroup**	$P2_1/m$
**Prototype**	RhTeCl
**Parent 3D**	$Rh_2Te_2Cl_2$
**Source DB**	ICSD
**DB ID**	56853
**DF2-C09 Binding energy [meV/ Å^2]**	19.17
**RVV10 Binding energy [meV/ Å^2]**	27.12
**Band gap (PBE) [eV]**	1.13

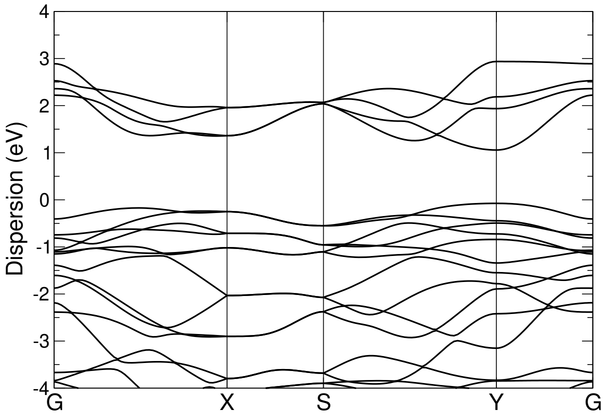

Band structure: Electronic band structure of RhTeCl ($P2_1/m$) in a representative window around the Fermi energy and along a high-symmetry path.

Optimized crystal structure

Structural parameters: relaxed cell (top) and atomic positions (bottom) of RhTeCl ($P2_1/m$) in Cartesian coordinates.

	x [Å]	y [Å]	z [Å]
$\mathbf{a_1}$	3.68417431	0.00000000	0.00000000
$\mathbf{a_2}$	0.00000000	6.60102736	0.00000000
$\mathbf{a_3}$	0.00000000	0.00000000	22.65480374

	x [Å]	y [Å]	z [Å]
Te	1.84208716	4.90209607	9.97855968
Rh	1.84208716	0.51446312	11.33676713
Cl	1.84208716	2.60089135	12.72649446
Te	0.00000000	5.90953954	12.67624003
Rh	0.00000000	3.69617671	11.31801859
Cl	0.00000000	1.60967424	9.92833135

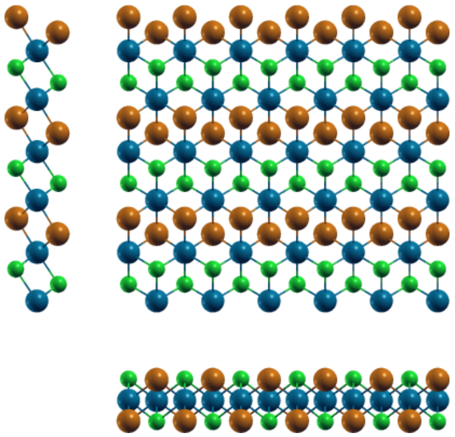

Orthographic projections: views of RhTeCl ($P2_1/m$) as seen from the x axis (left), the y axis (bottom) and the z axis (center).

Lattice matching - minimal size

Formula	N° atoms	strain	cell size 1	cell size 2
Tl	8	0.27	1	2
Cl_2Zn	9	0.2456	1	1
Cl_2OOs	10	0.2402	1	1
HgO	10	0.1141	1	2
AsSb	10	0.015	1	2
Pb_2Se_2	10	0.2018	1	1
Bi_2	10	0.5225	1	2
Cu_2Te_2	10	0.2487	1	1
Au_2Se_2	10	0.0159	1	1
$SbSe_2Tl$	10	0.5447	1	1
Sb_2	10	0.1912	1	2
Hf_3Te_2	11	0.2443	1	1
$CdCl_2$	12	0.0197	1	2
$I_2Lu_2Se_2$	12	0.175	1	1
$AgTe_2$	12	0.1103	1	2
PSn_2	12	0.0115	1	2
$MoSe_2$	12	0.269	1	2
$Ge_2Hf_2Te_2$	12	0.25	1	1
Br_2Zn	12	0.0083	1	2
Br_2Ca	12	0.5196	1	2
HfS_2	12	0.0162	1	2
$AsSn_2$	12	0.0092	1	2
$GeTe_2$	12	0.0187	1	2
$SiTe_2$	12	0.0102	1	2
$CuTe_2$	12	0.0165	1	2
S_2Zr	12	0.012	1	2
Br_2Cu	12	0.1324	1	2
NSr_2	12	0.0165	1	2
PbS_2	12	0.0128	1	2
FeI_2	12	0.0177	1	2
I_2Ni	12	0.0189	1	2
$As_2Co_2Li_2$	12	0.2493	1	1
Te_2Ti	12	0.0084	1	2
CrI_2	12	0.0173	1	2
$RhTe_2$	12	0.0082	1	2
Bi_2Pd	12	0.032	1	2
GeI_2	12	0.1962	1	2
$C_2Br_2Gd_2$	12	0.1364	1	1
$Br_2Er_2Se_2$	12	0.5237	1	1
$CoTe_2$	12	0.016	1	2
Se_2V	12	0.2713	1	2
Te_4TiZr	12	0.0179	1	1
$PbTe_2$	12	0.4851	1	2
$NiTe_2$	12	0.0099	1	2
$Cl_2Ga_2Te_2$	12	0.2917	1	1
Cl_2Cu	12	0.113	1	2
S_2Sn	12	0.0119	1	2
$SnTe_2$	12	0.1932	1	2
I_2V	12	0.0111	1	2
$Br_2Ga_2Te_2$	12	0.1749	1	1

Table showing the first 50 lattice matching combinations with the smallest number of atoms in the supercell.

Lattice matching - minimal strain

Formula	N° atoms	strain	cell size 1	cell size 2
$Ga_2Gd_2I_2$	954	0.0006	63	96
$Ga_2I_2Tb_2$	954	0.0006	63	96
N_2Re	342	0.0006	21	72
$Cl_2N_2Ti_2$	414	0.0006	24	45
Br_2CsF	370	0.0008	35	40
In_2Se_2	914	0.0009	89	95
As_4	58	0.0009	5	7
Fe_2Se_2	66	0.0011	5	9
$I_2S_2Tm_2$	456	0.0011	36	40
P_4	778	0.0011	63	100
$SnTe_2$	657	0.0011	60	99
F_4Pb	147	0.0012	12	15
Bi_2Pd	114	0.0012	9	20
$I_2Pr_2Si_2$	954	0.0012	60	99
$Si_2Te_2Zr_2$	84	0.0013	5	9
F_2Ni	57	0.0013	5	9
$ClKO_3$	894	0.0014	99	60
STl_2	429	0.0014	40	63
$Al_2Cl_2O_2$	558	0.0014	30	63
Ca_4Cu_2	852	0.0014	72	70
Bi_2Mn_2	564	0.0014	40	81
I_4Sr_2	438	0.0014	45	28
Ho_2S_2	186	0.0014	15	24
$I_2S_2Tl_2$	84	0.0015	5	9
$Br_2Ho_2S_2$	510	0.0015	40	45
AlH_4Na	564	0.0015	45	49
GdI_2	543	0.0015	52	77
$Au_2K_2Se_2$	372	0.0015	42	20
In_2Te_3	555	0.0015	40	63
Cu_2O_2	186	0.0015	15	24
CNb_2S_2	459	0.0016	24	63
Sb_2SeTe_2	555	0.0016	40	63
Cl_2Hf_2	696	0.0016	44	108
F_4Nb	590	0.0016	45	64
Cu_2Se_2	66	0.0016	5	9
$I_2S_2Yb_2$	456	0.0016	36	40
I_2La_2P	697	0.0017	52	77
Bi_2Mn_2	550	0.0017	39	79
$LiNbS_2$	696	0.0017	44	108
Br_3Cs	744	0.0017	88	54
CrS_2	300	0.0017	20	60
S_2Ta	588	0.0017	44	108
Gd_2GeI_2	858	0.0018	63	96
Br_2Ca_3Si	888	0.0018	63	85
Bi_2Se_2Te	555	0.0018	40	63
Gd_2I_2	676	0.0018	54	88
Ag_2F_4	858	0.0018	80	63
$Br_2F_2Sr_2$	732	0.0018	50	72
Bi_2Se_2	698	0.0018	61	83
$Br_2F_2Pb_2$	732	0.0019	50	72

Table showing the first 50 lattice matching combinations with the smallest strain needed to build the supercell.

$RuOCl_2$ (Pmmm)

Structural and electronic properties

**Formula**	$RuOCl_2$
**Spacegroup**	Pmmm
**Prototype**	NbOI2
**Parent 3D**	$RuOCl_2$
**Source DB**	ICSD
**DB ID**	83883
**DF2-C09 Binding energy [meV/ Å^2]**	12.99
**RVV10 Binding energy [meV/ Å^2]**	21.84
**Band gap (PBE) [eV]**	N/A

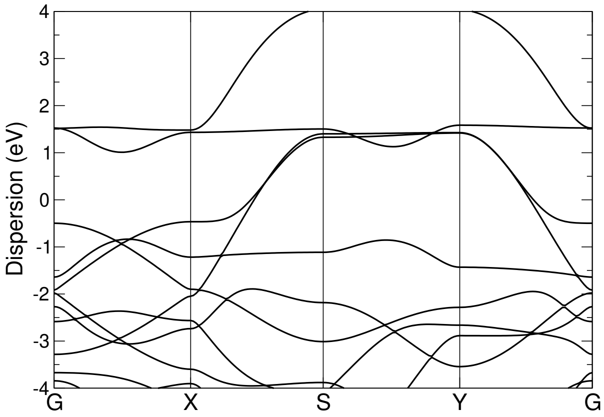

Band structure: Electronic band structure of $RuOCl_2$ (Pmmm) in a representative window around the Fermi energy and along a high-symmetry path.

Optimized crystal structure

Structural parameters: relaxed cell (top) and atomic positions (bottom) of $RuOCl_2$ (Pmmm) in Cartesian coordinates.

	x [Å]	y [Å]	z [Å]
$\mathbf{a_1}$	3.57286232	0.00000000	0.00000000
$\mathbf{a_2}$	0.00000000	3.67936414	0.00000000
$\mathbf{a_3}$	0.00000000	0.00000000	23.20066007

		x [Å]	y [Å]	z [Å]
●	Cl	3.57286232	1.83968207	9.98524469
●	Ru	1.78643116	1.83968207	11.60033003
●	Cl	0.00000000	1.83968207	13.21541538
●	O	1.78643116	0.00000000	11.60033003

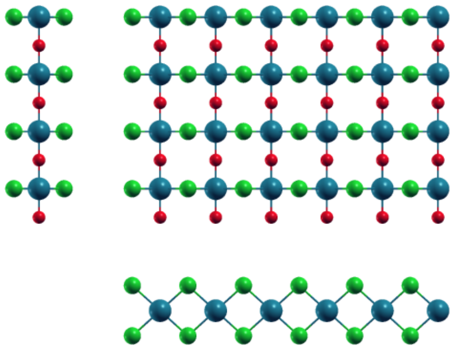

Orthographic projections: views of $RuOCl_2$ (Pmmm) as seen from the x axis (left), the y axis (bottom) and the z axis (center).

Lattice matching - minimal size

Formula	N° atoms	strain	cell size 1	cell size 2
Tl	5	0.6061	1	1
InSe	6	0.184	1	1
HgO	6	0.221	1	1
AsSb	6	0.1359	1	1
GeTe	6	0.1409	1	1
AgTl	6	0.4086	1	1
S_2	6	0.1422	1	1
PbTe	6	0.1862	1	1
Sb_2	6	0.1652	1	1
CaCl	6	0.0076	1	1
$IrTe_2$	7	0.1416	1	1
I_2Mg	7	0.1716	1	1
$CdCl_2$	7	0.1399	1	1
CdI_2	7	0.1884	1	1
$AgTe_2$	7	0.2026	1	1
Br_2Zn	7	0.1283	1	1
Br_2Ca	7	0.19	1	1
$InSe_2$	7	0.1403	1	1
$GeTe_2$	7	0.1391	1	1
$SiTe_2$	7	0.1312	1	1
$HfTe_2$	7	0.1492	1	1
I_2Mn	7	0.14	1	1
Br_2La	7	0.172	1	1
Br_2Cu	7	0.1326	1	1
NSr_2	7	0.1372	1	1
I_2Yb	7	0.7874	1	1
PbS_2	7	0.1339	1	1
BiClTe	7	0.1889	1	1
$AuTe_2$	7	0.1568	1	1
BrCdI	7	0.1753	1	1
Cl_2Zn	7	0.0136	1	1
$PdTe_2$	7	0.1544	1	1
FeI_2	7	0.1382	1	1
I_2Ni	7	0.1393	1	1
Mg_3	7	0.5882	1	1
Te_2Ti	7	0.1285	1	1
CrI_2	7	0.1378	1	1
I_2Zn	7	0.1616	1	1
BaF_2	7	0.1792	1	1
BiBrTe	7	0.1962	1	1
$RhTe_2$	7	0.1256	1	1
Bi_2Pd	7	0.2324	1	1
GeI_2	7	0.1695	1	1
Ba_2Hg	7	1.161	1	1
Ba_2N	7	0.1506	1	1
AsKSn	7	0.1775	1	1
Te_2Zr	7	0.1496	1	1
$PbTe_2$	7	0.1741	1	1
$NiTe_2$	7	0.1308	1	1
I_2Tm	7	0.7927	1	1

Table showing the first 50 lattice matching combinations with the smallest number of atoms in the supercell.

Lattice matching - minimal strain

Formula	N° atoms	strain	cell size 1	cell size 2
$Ba_2H_2I_2$	348	0.0003	45	28
$Cu_2Se_2Tl_2$	698	0.0003	80	63
$H_2I_2Sr_2$	348	0.0004	42	30
$Cl_2O_2Y_2$	718	0.0005	70	73
Cl_2Fe	635	0.0006	80	105
$HNiO_2$	380	0.0006	35	60
Ga_2Se_2	572	0.0006	70	73
Bi_2O_2	580	0.0006	81	64
H_2Li_2Pd	330	0.0007	30	42
$C_2Br_2Tb_2$	194	0.0008	20	19
Cu_2I_2	572	0.0008	80	63
S_2V	204	0.0008	24	36
Br_2Cu	593	0.0009	86	83
Br_2Y_2	572	0.0009	70	73
$LiOS_2Ti$	915	0.0009	85	115
$Br_2Dy_2S_2$	770	0.0009	101	61
$C_2Br_2Y_2$	864	0.0009	87	86
ClH_3O	264	0.0009	36	24
$Ho_2I_2S_2$	664	0.0009	88	52
$Cu_2Na_2Te_2$	588	0.0009	72	50
Cl_2Zr	635	0.0009	80	105
$AgCuTe_2$	568	0.001	72	70
$Cl_2Ho_2O_2$	718	0.001	70	73
Au_2I_2	472	0.001	70	48
PbS_2	499	0.0011	70	73
$C_2Br_2Gd_2$	194	0.0011	20	19
$Br_2La_2O_2$	708	0.0011	81	64
NbS_2	654	0.0011	81	110
H_2MgO_2	276	0.0011	24	36
$Al_2Cl_2O_2$	86	0.0011	8	9
$Eu_2F_2I_2$	708	0.0011	81	64
MoS_2	204	0.0011	24	36
$Br_2H_2Sr_2$	698	0.0012	80	63
H_4Ti	330	0.0012	30	42
S_2W	204	0.0012	24	36
CNNa	57	0.0012	9	7
Cl_2Co	635	0.0013	80	105
CNRb	480	0.0013	84	48
MoS_2	204	0.0013	24	36
Bi_2O_2	572	0.0013	80	63
$As_2Cd_2K_2$	588	0.0013	75	48
Mg_2	204	0.0013	30	42
I_2V	499	0.0013	70	73
PbS_2Sn	376	0.0014	54	40
S_2Ta	599	0.0014	74	101
CKN	286	0.0014	49	30
$Ba_2F_2I_2$	588	0.0014	75	48
$C_2Br_2Tb_2$	174	0.0015	18	17
Cl_2Rb_2	332	0.0015	63	20
CNRb	389	0.0015	68	39

Table showing the first 50 lattice matching combinations with the smallest strain needed to build the supercell.

$S_2Lu_2Br_2$ (Pmm2)

Structural and electronic properties

**Formula**	$S_2Lu_2Br_2$
**Spacegroup**	Pmm2
**Prototype**	FeOCl
**Parent 3D**	$Br_2Lu_2S_2$
**Source DB**	MPDS
**DB ID**	S307274
**DF2-C09 Binding energy [meV/ Å^2]**	9.03
**RVV10 Binding energy [meV/ Å^2]**	N/A
**Band gap (PBE) [eV]**	2.93

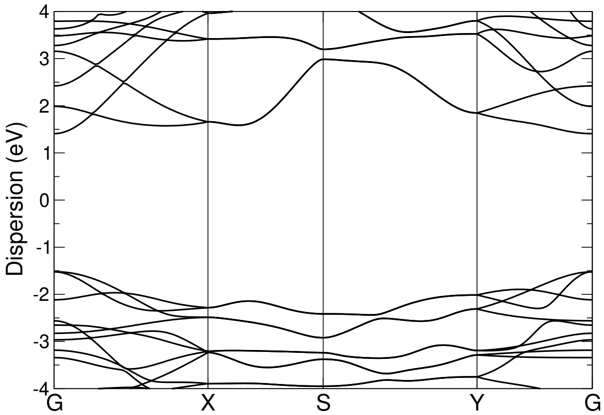

Band structure: Electronic band structure of $S_2Lu_2Br_2$ (Pmm2) in a representative window around the Fermi energy and along a high-symmetry path.

Optimized crystal structure

Structural parameters: relaxed cell (top) and atomic positions (bottom) of $S_2Lu_2Br_2$ (Pmm2) in Cartesian coordinates.

	x [Å]	y [Å]	z [Å]
$\mathbf{a_1}$	3.95835233	0.00000000	0.00000000
$\mathbf{a_2}$	0.00000000	5.28331379	0.00000000
$\mathbf{a_3}$	0.00000000	0.00000000	24.03880272

		x [Å]	y [Å]	z [Å]
●	Lu	-0.98963416	-1.32082845	1.11363224
●	Lu	0.98963416	-3.96248534	-1.11363224
●	S	-0.98950304	-3.96248534	0.71113592
●	S	0.98950304	-1.32082845	-0.71113592
●	Br	-0.98951467	-3.96248534	-3.06238110
●	Br	0.98951467	-1.32082845	3.06238110

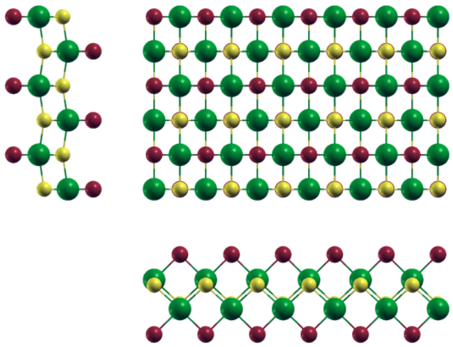

Orthographic projections: views of $S_2Lu_2Br_2$ (Pmm2) as seen from the x axis (left), the y axis (bottom) and the z axis (center).

Lattice matching - minimal size

Formula	N° atoms	strain	cell size 1	cell size 2
AgTl	8	0.2745	1	1
Br_2Cu	9	0.1205	1	1
Ba_2Hg	9	0.862	1	1
CNRb	9	0.325	1	1
CNNa	9	0.3258	1	1
Bi_2In_2	10	0.0728	1	1
Bi_2	10	0.3457	1	2
Cu_2Te_2	10	0.2201	1	1
As_4	10	0.3325	1	1
P_4	10	0.6322	1	1
Br_2OV	10	1.9827	1	1
Au_2I_2	10	0.066	1	1
PbS_2Sn	10	0.8596	1	1
$SbSe_2Tl$	10	0.5227	1	1
Br_2CsF	10	0.0717	1	1
Sn_2Te_2	10	0.0712	1	1
F_4Sn	11	0.2903	1	1
FKO_2Se	11	1.1691	1	1
$Ba_2H_2I_2$	12	0.0708	1	1
CrS_2	12	0.1377	1	2
$Br_2Ho_2S_2$	12	0.0103	1	1
S_2V	12	0.3483	1	2
MoS_2	12	0.3497	1	2
$I_2Lu_2Se_2$	12	0.588	1	1
$Ge_2Hf_2Te_2$	12	0.2212	1	1
$Ho_2I_2S_2$	12	0.0205	1	1
Cu_4Te_2	12	0.1227	1	1
$C_2I_2La_2$	12	0.1223	1	1
Br_2Ca	12	0.3435	1	2
AlH_4Na	12	0.3973	1	1
$GeTe_2$	12	0.7015	1	2
$Cl_2F_2Pb_2$	12	0.7892	1	1
BiClTe	12	0.3417	1	2
ReS_2	12	0.1411	1	2
$Br_2Cr_2S_2$	12	0.6082	1	1
Br_2Ca_3Si	12	0.863	1	1
$Ba_2Ge_2Mn_2$	12	0.7836	1	1
$K_2O_2Tl_2$	12	0.5954	1	1
$As_2Co_2Li_2$	12	0.2206	1	1
$Br_2S_2Y_2$	12	0.0084	1	1
BiBrTe	12	0.3533	1	2
S_2W	12	0.1494	1	2
$Gd_2I_2S_2$	12	0.0262	1	1
N_2W	12	0.1343	1	2
$Cu_2Na_2Se_2$	12	0.7889	1	1
$I_2S_2Tb_2$	12	0.0238	1	1
Te_4TiZr	12	0.7017	1	1
$I_2S_2Yb_2$	12	0.0151	1	1
$Cu_2Rb_2Te_2$	12	0.0726	1	1
$Br_2Dy_2S_2$	12	0.0126	1	1

Table showing the first 50 lattice matching combinations with the smallest number of atoms in the supercell.

Lattice matching - minimal strain

Formula	N° atoms	strain	cell size 1	cell size 2
$Br_2Lu_2S_2$	12	0.0001	1	1
$Br_2O_2Pr_2$	42	0.0003	3	4
$Hf_2Se_2Si_2$	258	0.0003	16	27
Ag_2Br_2	34	0.0004	3	4
$Ba_2F_2I_2$	582	0.0005	48	49
Te_2Zn	738	0.0005	63	120
Br_3Cs	972	0.0005	120	63
$Br_2N_2Zr_2$	606	0.0006	36	65
CBr_2Lu_2	541	0.0006	36	65
As_2Rh_2	34	0.0006	3	4
Br_2CsF	486	0.0006	49	48
C_4Ca_2	966	0.0006	72	89
O_4PTl	582	0.0007	48	49
As_2Fe_2	204	0.0007	16	27
F_4Pb	570	0.0007	50	54
Tl	97	0.0008	12	25
I_2La	30	0.0008	3	4
Cl_2Hf_2	432	0.0009	30	63
P_4	402	0.0009	35	48
CNNa	720	0.001	74	92
InSe	460	0.0011	53	71
As_2Li_2Pr	673	0.0011	53	71
$I_2La_2Si_2$	744	0.0011	53	71
IKO_3	528	0.0011	63	30
$AsLi_3$	602	0.0011	53	71
$Br_2Hf_2N_2$	918	0.0011	63	90
Mg_4	172	0.0011	12	25
$Cu_2Rb_2Te_2$	582	0.0011	49	48
$Ge_2Mn_2Sr_2$	42	0.0013	3	4
As_2Li_2Nd	673	0.0013	53	71
$Cl_2H_2Lu_2$	606	0.0013	36	65
$I_2N_2Ti_2$	360	0.0013	24	36
Ga_2S_3	978	0.0013	63	120
Bi_2STe_2	673	0.0014	53	71
HgI_2	849	0.0014	93	97
GeI_2Y_2	673	0.0014	53	71
Bi_2In_2	486	0.0015	49	48
$H_2I_2Yb_2$	42	0.0015	3	4
S_2Ti	744	0.0015	61	126
Pb_2Se_2	754	0.0015	73	79
CNNa	702	0.0015	72	90
$C_2Br_2Tb_2$	990	0.0015	66	99
GeS_2	177	0.0016	16	27
$C_2Br_2Gd_2$	990	0.0016	66	99
Cl_2Mg	411	0.0016	36	65
$As_2Cd_2K_2$	582	0.0016	48	49
Br_2Ni	411	0.0016	36	65
Bi_2Pd	294	0.0016	25	48
Bi_2Se_4	78	0.0017	8	5
Cl_4KTl	222	0.0017	25	12

Table showing the first 50 lattice matching combinations with the smallest strain needed to build the supercell.

$S_2Lu_2I_2$ (Pmmn)

Structural and electronic properties

**Formula**	$S_2Lu_2I_2$
**Spacegroup**	Pmmn
**Prototype**	FeOCl
**Parent 3D**	$I_2Lu_2S_2$
**Source DB**	MPDS
**DB ID**	S1937399
**DF2-C09 Binding energy [meV/ Å^2]**	11.12
**RVV10 Binding energy [meV/ Å^2]**	N/A
**Band gap (PBE) [eV]**	2.55

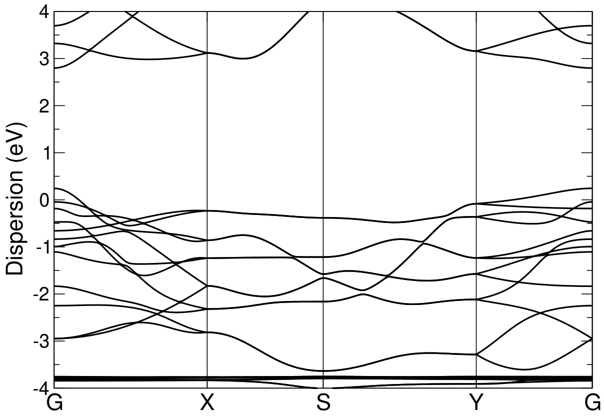

Band structure: Electronic band structure of $S_2Lu_2I_2$ (Pmmn) in a representative window around the Fermi energy and along a high-symmetry path.

Optimized crystal structure

Structural parameters: relaxed cell (top) and atomic positions (bottom) of $S_2Lu_2I_2$ (Pmmn) in Cartesian coordinates.

		x [Å]	y [Å]	z [Å]
$\mathbf{a_1}$		4.00335192	0.00000000	0.00000000
$\mathbf{a_2}$		0.00000000	5.28184844	0.00000000
$\mathbf{a_3}$		0.00000000	0.00000000	24.89276428
		x [Å]	y [Å]	z [Å]
●	Lu	1.00077011	-1.32046211	-1.11353934
●	S	1.00081420	-3.96138633	-0.66017671
●	I	-1.00089087	-1.32046211	-3.34131492
●	Lu	-1.00077011	-3.96138633	1.11353934
●	S	-1.00081420	-1.32046211	0.66017671
●	I	1.00089087	-3.96138633	3.34131492

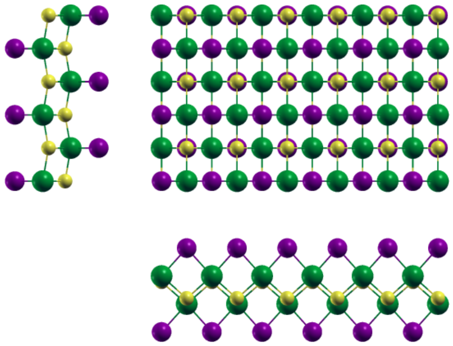

Orthographic projections: views of $S_2Lu_2I_2$ (Pmmn) as seen from the x axis (left), the y axis (bottom) and the z axis (center).

Lattice matching - minimal size

Formula	N° atoms	strain	cell size 1	cell size 2
AgTl	8	0.2678	1	1
In	8	0.1467	1	2
Br_2Cu	9	0.119	1	1
Ba_2Hg	9	0.2843	1	1
CNNa	9	0.1518	1	1
Bi_2In_2	10	0.0684	1	1
Ge_2Te_2	10	0.3166	1	1
As_4	10	0.3249	1	1
LiO	10	0.3613	1	2
Au_2I_2	10	0.0634	1	1
O_2Sn_2	10	0.1707	1	1
PbS_2Sn	10	0.8392	1	1
$SbSe_2Tl$	10	0.5118	1	1
Br_2CsF	10	0.0675	1	1
Sn_2Te_2	10	0.0673	1	1
F_4Sn	11	0.283	1	1
FKO_2Se	11	1.1867	1	1
Cl_4Mn	11	0.382	1	1
$Ba_2H_2I_2$	12	0.0667	1	1
CrS_2	12	0.1379	1	2
$Br_2Ho_2S_2$	12	0.0064	1	1
$I_2Lu_2Se_2$	12	0.5759	1	1
$Ho_2I_2S_2$	12	0.0163	1	1
Cu_4Te_2	12	0.1204	1	1
$C_2I_2La_2$	12	0.12	1	1
AlH_4Na	12	0.3874	1	1
$GeTe_2$	12	0.6878	1	2
$Cl_2F_2Pb_2$	12	0.2582	1	1
$Cl_2H_2Zr_2$	12	0.2796	1	1
ReS_2	12	0.1413	1	2
Br_2Ca_3Si	12	0.8426	1	1
$Ba_2Ge_2Mn_2$	12	0.7649	1	1
$K_2O_2Tl_2$	12	0.5834	1	1
$Br_2S_2Y_2$	12	0.0054	1	1
$Gd_2I_2S_2$	12	0.022	1	1
$C_2Br_2Gd_2$	12	0.1082	1	1
N_2W	12	0.1346	1	2
$Cu_2Na_2Se_2$	12	0.2581	1	1
$F_2I_2Yb_2$	12	0.7637	1	1
$I_2S_2Tb_2$	12	0.0196	1	1
Te_4TiZr	12	0.6881	1	1
$I_2S_2Yb_2$	12	0.011	1	1
$Cu_2Rb_2Te_2$	12	0.0683	1	1
$Br_2Dy_2S_2$	12	0.0086	1	1
$Cl_2Ga_2Te_2$	12	0.5523	1	1
Cl_2Cu	12	0.0951	1	2
$I_2Nd_2O_2$	12	0.2594	1	1
$Br_2Ga_2Te_2$	12	0.5618	1	1
Ca_4Cu_2	12	0.5509	1	1
O_4PSn	12	0.0652	1	1

Table showing the first 50 lattice matching combinations with the smallest number of atoms in the supercell.

Lattice matching - minimal strain

Formula	N° atoms	strain	cell size 1	cell size 2
K	172	0.0001	24	28
$I_2S_2Sm_2$	312	0.0002	24	28
$Br_2Cr_2S_2$	432	0.0005	30	42
I_2La_2Sb	284	0.0005	24	28
Bi_2STe_2	765	0.0005	60	81
Te_4W_2	492	0.0006	42	40
$AsLi_3$	684	0.0007	60	81
Br_3Cs	886	0.0008	109	58
LiO_2	330	0.0008	27	56
Te_2Zn	660	0.0008	56	108
GdI_2	591	0.0008	60	77
O_4PTl	660	0.0009	54	56
Ga_2S_3	876	0.0009	56	108
CNNa	645	0.001	66	83
InSe	522	0.001	60	81
As_2Li_2Pr	765	0.001	60	81
PbTe	522	0.001	60	81
$I_2La_2Si_2$	846	0.001	60	81
HgI_2	954	0.0011	104	110
As_2CeLi_2	765	0.0011	60	81
$Br_2H_2Zr_2$	996	0.0011	54	112
C_2Li_2	460	0.0011	40	55
PTe_2Zr_2	801	0.0012	56	93
GeI_2	603	0.0012	60	81
Ga_2Se_2	684	0.0013	54	90
GeS_2	156	0.0013	14	24
As_2Fe_2	180	0.0013	14	24
$Cl_2H_2Lu_2$	510	0.0013	30	55
As_2Li_2Nd	792	0.0013	62	84
Cl_4Mn	595	0.0013	55	53
O_4PTl	648	0.0014	53	55
PTe_2Zr_2	774	0.0014	54	90
Cl_2Mg	345	0.0014	30	55
$Br_2Hf_2N_2$	822	0.0014	56	81
$Ba_2F_2I_2$	660	0.0014	54	56
Br_2Ni	345	0.0014	30	55
GeI_2Y_2	792	0.0015	62	84
PTe_2Ti_2	455	0.0015	30	55
$Ba_2F_2I_2$	648	0.0015	53	55
Ga_2Se_2	622	0.0015	49	82
$LiMnTe_2$	684	0.0015	60	81
$Hf_2Se_2Si_2$	228	0.0015	14	24
As_2Li_2Nd	765	0.0016	60	81
I_2La_2P	745	0.0016	60	77
$Cl_2Er_2S_2$	12	0.0016	1	1
AsSb	522	0.0016	56	93
$Cl_2O_2Y_2$	786	0.0016	49	82
Ga_2S_2	400	0.0016	30	55
CNNa	627	0.0017	64	81
$Ba_2H_2I_2$	660	0.0017	55	55

Table showing the first 50 lattice matching combinations with the smallest strain needed to build the supercell.

Sb (P-3m1)

Structural and electronic properties

**Formula**	Sb
**Spacegroup**	P-3m1
**Prototype**	As
**Parent 3D**	Sb_6Te_6
**Source DB**	ICSD
**DB ID**	20459
**DF2-C09 Binding energy [meV/ Å^2]**	29.59
**RVV10 Binding energy [meV/ Å^2]**	33.07
**Band gap (PBE) [eV]**	1.23

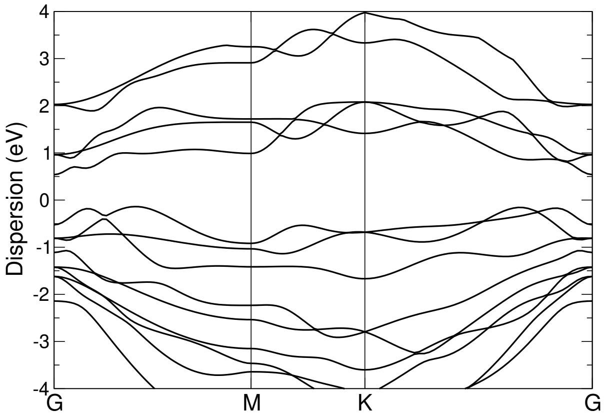

Band structure: Electronic band structure of Sb (P-3m1) in a representative window around the Fermi energy and along a high-symmetry path.

Optimized crystal structure

Structural parameters: relaxed cell (top) and atomic positions (bottom) of Sb (P-3m1) in Cartesian coordinates.

	x [Å]	y [Å]	z [Å]
$\mathbf{a_1}$	4.31311481	0.00000000	0.00000000
$\mathbf{a_2}$	−2.15655741	3.73526700	0.00000000
$\mathbf{a_3}$	0.00000000	0.00000000	27.49254885

		x [Å]	y [Å]	z [Å]
●	Sb	0.00000000	2.49017800	11.74391041
●	Te	0.00000000	2.49017800	17.45328686
●	Sb	0.00000000	0.00000000	15.74863844
●	Te	0.00000000	0.00000000	10.03926200
●	Te	2.15655741	1.24508900	13.74627443

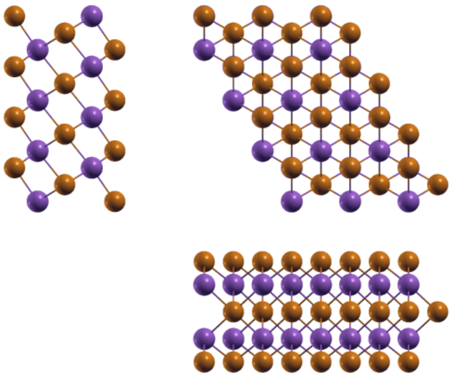

Orthographic projections: views of Sb (P-3m1) as seen from the x axis (left), the y axis (bottom) and the z axis (center).

Lattice matching - minimal size

Formula	N° atoms	strain	cell size 1	cell size 2
InSe	7	0.0074	1	1
AsSb	7	0.261	1	1
Bi_2	7	0.0017	1	1
GeTe	7	0.2696	1	1
S_2	7	0.2719	1	1
PbTe	7	0.0057	1	1
CaCl	7	0.4435	1	1
$IrTe_2$	8	0.2709	1	1
$CdCl_2$	8	0.2681	1	1
CdI_2	8	0.004	1	1
PSn_2	8	1.529	1	1
Br_2Zn	8	0.2472	1	1
Br_2Ca	8	0.0028	1	1
$InSe_2$	8	0.2686	1	1
$AsSn_2$	8	1.5561	1	1
$GeTe_2$	8	0.2666	1	1
$SiTe_2$	8	1.6375	1	1
I_2Pr	8	0.1307	1	1
I_2Mn	8	0.2682	1	1
S_2Zr	8	1.5236	1	1
Br_2Cu	8	0.6916	1	1
NSr_2	8	0.2633	1	1
PbS_2	8	0.2574	1	1
BiClTe	8	0.0036	1	1
Cl_2Zn	8	0.1164	1	1
FeI_2	8	0.2651	1	1
I_2Ni	8	0.2669	1	1
Te_2Ti	8	0.2476	1	1
CrI_2	8	0.2644	1	1
BiBrTe	8	0.0019	1	1
I_2Nd	8	0.1314	1	1
$NiTe_2$	8	1.633	1	1
Cl_2Cu	8	0.07	1	1
S_2Sn	8	1.5255	1	1
I_2V	8	0.2544	1	1
GeI_2	8	0.0054	1	1
Se_2Zr	8	1.6406	1	1
$PtSe_2$	8	1.5608	1	1
BiITe	8	0.0086	1	1
CoI_2	8	0.2616	1	1
$MnSe_2$	8	0.4433	1	1
CeI_2	8	0.1302	1	1
Br_2Mg	8	0.2649	1	1
I_2Ti	8	0.2621	1	1
$NbTe_2$	8	1.5221	1	1
GdI_2	8	0.0048	1	1
F_2Ni	8	0.1146	1	1
I_2La	8	0.135	1	1
F_2Na	8	0.2498	1	1
CdI_2	8	0.0044	1	1

Table showing the first 50 lattice matching combinations with the smallest number of atoms in the supercell.

Lattice matching - minimal strain

Formula	N° atoms	strain	cell size 1	cell size 2
Br_2V	155	0.0	16	25
KS_2Ti	269	0.0	25	36
Ga_2Se_2	443	0.0	43	57
N_2W	545	0.0	49	100
$F_2Se_2Y_2$	789	0.0001	81	64
Ni_2Te_2	805	0.0001	81	100
CdClHO	376	0.0001	36	49
GeI_3Rb	290	0.0001	49	9
$Hf_2I_2N_2$	474	0.0001	36	49
CrI_2	638	0.0002	73	91
C_2	178	0.0003	16	49
$Cl_2O_2Y_2$	723	0.0003	57	73
$CdCl_2$	705	0.0003	81	100
$Cl_2O_2Yb_2$	629	0.0003	49	64
Br_2Mn	233	0.0004	25	36
Cl_2Mn	368	0.0004	37	61
Br_2Pr_2	501	0.0004	49	64
Br_2Mg	638	0.0004	73	91
$Cl_2Ho_2O_2$	723	0.0004	57	73
F_2Na	437	0.0004	49	64
I_2Mn	705	0.0005	81	100
LiO	77	0.0005	9	16
I_2Ni	705	0.0005	81	100
PTe_2Zr_2	725	0.0005	64	81
FeH_2O_2	590	0.0005	39	79
FeI_2	638	0.0005	73	91
$LiOS_2Ti$	650	0.0005	49	81
Cu_4Te_2	11	0.0006	1	1
Bi_2Te_2	824	0.0006	100	81
$C_2I_2La_2$	638	0.0007	58	58
$GeTe_2$	705	0.0007	81	100
Cl_2Y_2	443	0.0007	43	57
$TaTe_2$	327	0.0007	36	49
$InSe_2$	705	0.0007	81	100
$Br_2Ga_2Te_2$	809	0.0007	91	59
Bi_2Se_2	620	0.0007	72	65
NSr_2	638	0.0007	73	91
I_2V	504	0.0007	57	73
Cl_2Zr_2	180	0.0008	16	25
$ReSe_2$	155	0.0008	16	25
Ga_2Se_2	577	0.0008	57	73
Cl_2NSc_2	490	0.0008	37	61
Cl_2Zn	233	0.0008	25	36
Ca_2O_2	877	0.0009	81	118
AgTl	369	0.0009	53	52
Se_2Sn	638	0.001	73	91
H_2Si_2	805	0.001	81	100
$I_2N_2Zr_2$	557	0.001	43	57
Bi_2SeTe_2	10	0.0011	1	1
$AgNO_3$	305	0.0011	36	25

Table showing the first 50 lattice matching combinations with the smallest strain needed to build the supercell.

Sb_2Te_2S (P-3m1)

Structural and electronic properties

**Formula**	Sb_2Te_2S
**Spacegroup**	P-3m1
**Prototype**	Bi2Te2S
**Parent 3D**	SSb_2Te_2
**Source DB**	MPDS
**DB ID**	S1716597
**DF2-C09 Binding energy [meV/ Å^2]**	22.39
**RVV10 Binding energy [meV/ Å^2]**	N/A
**Band gap (PBE) [eV]**	0.76

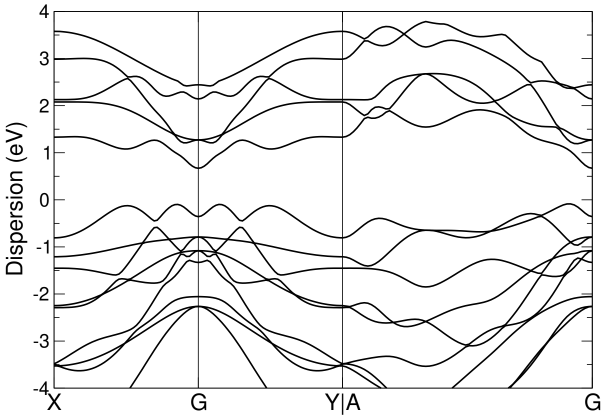

Band structure: Electronic band structure of Sb_2Te_2S (P-3m1) in a representative window around the Fermi energy and along a high-symmetry path.

Optimized crystal structure

Structural parameters: relaxed cell (top) and atomic positions (bottom) of Sb_2Te_2S (P-3m1) in Cartesian coordinates.

	x [Å]	y [Å]	z [Å]
$\mathbf{a_1}$	2.09036016	−3.62063851	0.00000000
$\mathbf{a_2}$	2.09038484	3.62065276	0.00000000
$\mathbf{a_3}$	0.00000000	0.00000000	25.68293785

		x [Å]	y [Å]	z [Å]
●	Sb	-1.04518208	0.60343616	-1.69016700
●	Te	1.04519016	-0.60344082	-3.44984812
●	Sb	1.04518208	-0.60343616	1.69016700
●	Te	-1.04519016	0.60344082	3.44984812
●	S	1.04519242	1.81032638	0.00000000

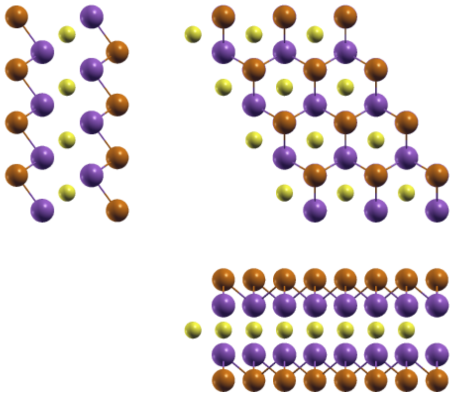

Orthographic projections: views of Sb_2Te_2S (P-3m1) as seen from the x axis (left), the y axis (bottom) and the z axis (center).

Lattice matching - minimal size

Formula	N° atoms	strain	cell size 1	cell size 2
InSe	7	0.0073	1	1
AgTl	7	0.1598	1	1
Ag_2	7	0.4571	1	1
PbTe	7	0.0091	1	1
Sb_2	7	0.0086	1	1
CaCl	7	0.1165	1	1
I_2Mg	8	0.0031	1	1
Ba_2Pt	8	0.4565	1	1
Br_2Zn	8	0.2674	1	1
$AsSn_2$	8	0.2577	1	1
$SiTe_2$	8	0.2734	1	1
I_2Pr	8	0.1425	1	1
$CuTe_2$	8	1.5882	1	1
Br_2La	8	0.0027	1	1
Ca_2Si	8	0.4678	1	1
BrCdI	8	0.0001	1	1
Cl_2Zn	8	0.1245	1	1
Te_2Ti	8	0.2678	1	1
BaF_2	8	0.0034	1	1
Te_2Zn	8	1.5286	1	1
$RhTe_2$	8	0.2616	1	1
GeI_2	8	0.0049	1	1
AsKSn	8	0.0019	1	1
$PbTe_2$	8	0.0009	1	1
I_2Nd	8	0.1433	1	1
$NiTe_2$	8	0.2725	1	1
Cl_2Cu	8	0.0655	1	1
$SnTe_2$	8	0.0071	1	1
I_2V	8	0.2754	1	1
GeI_2	8	0.0094	1	1
Se_2Zr	8	0.274	1	1
I_2Pb	8	0.4611	1	1
STl_2	8	0.0044	1	1
$PtSe_2$	8	0.2586	1	1
GeS_2	8	0.1116	1	1
$TaTe_2$	8	0.2569	1	1
$MnSe_2$	8	0.1165	1	1
Br_2Ni	8	0.247	1	1
CeI_2	8	0.1418	1	1
In	8	0.6318	1	3
Se_2Yb	8	0.0047	1	1
Cl_2Mg	8	0.2471	1	1
$BiTe_2$	8	0.0044	1	1
F_2Ni	8	0.122	1	1
I_2La	8	0.1477	1	1
F_2Na	8	0.2702	1	1
F_2Zn	8	0.1391	1	1
$HfSe_2$	8	0.2678	1	1
Bi_2Te_2	9	0.5009	1	1
Fe_2Te_2	9	0.1322	1	1

Table showing the first 50 lattice matching combinations with the smallest number of atoms in the supercell.

Lattice matching - minimal strain

Formula	N° atoms	strain	cell size 1	cell size 2
$Cl_2H_2Zr_2$	230	0.0	16	25
I_2Pb	743	0.0	100	81
BrCdI	8	0.0001	1	1
BrNZr	233	0.0001	25	36
$CoTe_2$	386	0.0001	43	57
Te_2Ti	705	0.0001	81	100
Br_2Zn	705	0.0002	81	100
BN	95	0.0002	9	25
$HfSe_2$	705	0.0002	81	100
Sm	189	0.0002	25	64
Ga_2Se_2	729	0.0002	73	91
HfS_2	386	0.0002	43	57
H_2MnO_2	745	0.0002	49	100
$Br_2N_2Zr_2$	629	0.0002	49	64
Ga_2S_2	443	0.0003	43	57
CCl_2Gd_2	905	0.0003	81	100
CBr_2Lu_2	565	0.0003	49	64
NbS_2	155	0.0003	16	25
$Cl_2O_2Sc_2$	635	0.0003	55	60
$MnNaTe_2$	9	0.0003	1	1
Ca_2Si	674	0.0004	91	73
$Ce_2I_2S_2$	893	0.0004	91	73
Te_2V	327	0.0005	36	49
CdClHO	577	0.0005	57	73
AgTl	452	0.0005	66	61
$Hf_2I_2N_2$	723	0.0005	57	73
Br_2Hf_2	269	0.0005	25	36
$PtSe_2$	563	0.0005	64	81
$CuTe_2$	386	0.0006	43	57
$Ba_2F_2I_2$	613	0.0006	65	48
Hg_3N_2	170	0.0007	25	9
$Cl_2Hf_2N_2$	474	0.0008	36	49
OTl_2	327	0.0008	36	49
$I_2N_2Zr_2$	911	0.0008	73	91
CBr_2Y_2	905	0.0008	81	100
$LiOS_2Ti$	205	0.0009	16	25
CdClHO	644	0.0009	64	81
Cl_2Y_2	729	0.0009	73	91
$I_2Pr_2S_2$	986	0.0009	100	81
$PbTe_2$	8	0.0009	1	1
Sb_2Te_2	747	0.0009	91	73
CdClO	327	0.001	36	49
$Br_2Cr_2S_2$	440	0.001	40	40
$Cl_2O_2Ti_2$	836	0.001	70	81
CaH_2O_2	500	0.001	43	57
Cu_2Te_2	674	0.001	70	81
Br_2Pr_2	805	0.001	81	100
O_4PTl	613	0.001	65	48
$TaTe_2$	504	0.001	57	73
Ag_2F_4	517	0.001	65	32

Table showing the first 50 lattice matching combinations with the smallest strain needed to build the supercell.

Sb_2Te_2Se (P-3m1)

Structural and electronic properties

**Formula**	Sb_2Te_2Se
**Spacegroup**	P-3m1
**Prototype**	Bi2Te2S
**Parent 3D**	Sb_2Te_2Se
**Source DB**	COD
**DB ID**	9007591
**DF2-C09 Binding energy [meV/ Å^2]**	23.46
**RVV10 Binding energy [meV/ Å^2]**	28.13
**Band gap (PBE) [eV]**	0.65

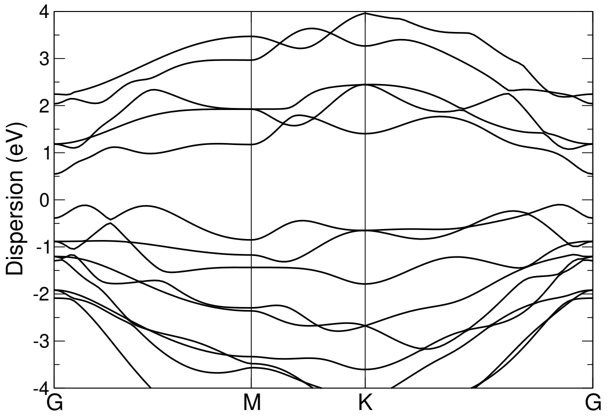

Band structure: Electronic band structure of Sb_2Te_2Se (P-3m1) in a representative window around the Fermi energy and along a high-symmetry path.

Optimized crystal structure

Structural parameters: relaxed cell (top) and atomic positions (bottom) of Sb_2Te_2Se (P-3m1) in Cartesian coordinates.

	x [Å]	y [Å]	z [Å]
$\mathbf{a_1}$	4.22959327	0.00000000	0.00000000
$\mathbf{a_2}$	−2.11479664	3.66293522	0.00000000
$\mathbf{a_3}$	0.00000000	0.00000000	27.08482530

		x [Å]	y [Å]	z [Å]
●	Sb	0.00000000	2.44195681	11.72941984
●	Te	0.00000000	2.44195681	17.09814209
●	Sb	0.00000000	0.00000000	15.35540545
●	Te	-2.11479664	3.66293522	9.98668320
●	Se	2.11479664	1.22097841	13.54241265

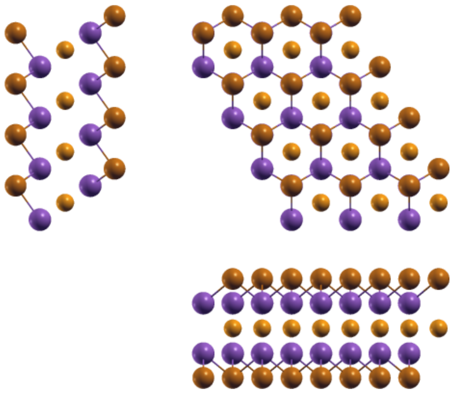

Orthographic projections: views of Sb_2Te_2Se (P-3m1) as seen from the x axis (left), the y axis (bottom) and the z axis (center).

Lattice matching - minimal size

Formula	N° atoms	strain	cell size 1	cell size 2
InSe	7	0.0017	1	1
AsSb	7	0.2743	1	1
Bi_2	7	0.0076	1	1
AgTl	7	0.1542	1	1
As_2	7	1.5313	1	1
P_2	7	4.8587	1	1
PbTe	7	0.0035	1	1
CaCl	7	0.1141	1	1
Cl_2Zn	8	1.5239	1	1
I_2Mg	8	0.0084	1	1
CdI_2	8	0.0053	1	1
PSn_2	8	1.596	1	1
Br_2Zn	8	0.2596	1	1
Br_2Ca	8	0.0065	1	1
HfS_2	8	1.5509	1	1
$AsSn_2$	8	0.2503	1	1
$SiTe_2$	8	0.2654	1	1
I_2Pr	8	0.1379	1	1
S_2Zr	8	1.5903	1	1
Br_2La	8	0.0081	1	1
Br_2Cu	8	0.7244	1	1
Ca_2Si	8	0.4541	1	1
PbS_2	8	0.2705	1	1
Br_2Co	8	1.5283	1	1
BiClTe	8	0.0056	1	1
Ca_2N	8	1.5351	1	1
Cl_2Ti	8	4.862	1	1
BrCdI	8	0.0054	1	1
Cl_2Zn	8	0.1212	1	1
Te_2Ti	8	0.26	1	1
BaF_2	8	0.0021	1	1
$RhTe_2$	8	0.254	1	1
$CoTe_2$	8	1.5533	1	1
AsKSn	8	0.0035	1	1
$PbTe_2$	8	0.0063	1	1
I_2Nd	8	0.1387	1	1
$NiTe_2$	8	0.2646	1	1
Cl_2Cu	8	0.067	1	1
S_2Sn	8	1.5923	1	1
I_2V	8	0.2673	1	1
GeI_2	8	0.0037	1	1
Se_2Zr	8	0.266	1	1
I_2Pb	8	0.4476	1	1
STl_2	8	0.0012	1	1
$PtSe_2$	8	1.629	1	1
CoI_2	8	0.2749	1	1
Br_2Fe	8	1.5288	1	1
$TaTe_2$	8	0.2495	1	1
$MnSe_2$	8	0.1141	1	1
Br_2Ni	8	1.5652	1	1

Table showing the first 50 lattice matching combinations with the smallest number of atoms in the supercell.

Lattice matching - minimal strain

Formula	N° atoms	strain	cell size 1	cell size 2
S_2Sn	386	0.0	43	57
$I_2N_2Zr_2$	723	0.0001	57	73
In_2Te_3	10	0.0001	1	1
$HNiO_2$	511	0.0001	39	79
CCl_2Gd_2	725	0.0001	64	81
As_2O_3	490	0.0001	61	37
$NiTe_2$	638	0.0002	73	91
Cu_2O_2	355	0.0002	39	40
I_2V	705	0.0002	81	100
$TaTe_2$	437	0.0002	49	64
$CuTe_2$	327	0.0002	36	49
Cl_2Hf_2	180	0.0002	16	25
CoO_2	47	0.0002	4	9
Br_2Zn	563	0.0002	64	81
HN_3OZn	551	0.0003	37	61
CaH_2O_2	425	0.0003	36	49
N_2W	272	0.0003	25	49
S_2Zr	386	0.0003	43	57
$Hf_2I_2N_2$	629	0.0004	49	64
Bi_2Se_2Te	10	0.0004	1	1
CdClHO	501	0.0004	49	64
Mg_2	541	0.0004	65	108
CaClHO	729	0.0004	73	91
CBr_2Y_2	725	0.0005	64	81
$NbTe_2$	386	0.0005	43	57
PSn_2	386	0.0005	43	57
$Cl_2N_2Zr_2$	474	0.0005	36	49
$Al_2Cl_2O_2$	39	0.0005	3	4
Ho_2S_2	355	0.0005	39	40
Te_2Ti	563	0.0005	64	81
FeO_2	47	0.0005	4	9
CS_2Ta_2	650	0.0005	49	81
$HfSe_2$	563	0.0005	64	81
H_2MgO_2	125	0.0005	9	16
HfS_2	327	0.0006	36	49
Br_3Cs	420	0.0006	64	25
$Cl_2O_2Tm_2$	911	0.0006	73	91
Cl_2Ti	488	0.0006	49	81
Sn_2Te_2	599	0.0007	75	56
NiO_2	47	0.0007	4	9
La_2S_2	50	0.0008	6	5
$SiTe_2$	638	0.0008	73	91
P_2	407	0.0008	49	81
$AsSn_2$	437	0.0008	49	64
ClH_3O	725	0.0008	81	64
GeI_2Y_2	10	0.0008	1	1
$CoTe_2$	327	0.0009	36	49
$LiNbS_2$	180	0.0009	16	25
FeH_2O_2	370	0.0009	25	49
As_4	539	0.0009	63	56

Table showing the first 50 lattice matching combinations with the smallest strain needed to build the supercell.

Sb_2Te_3 (P-3m1)

Structural and electronic properties

**Formula**	Sb_2Te_3
**Spacegroup**	P-3m1
**Prototype**	Bi2Te3
**Parent 3D**	Sb_2Te_3
**Source DB**	COD
**DB ID**	9007590
**DF2-C09 Binding energy [meV/ Å^2]**	25.22
**RVV10 Binding energy [meV/ Å^2]**	30.03
**Band gap (PBE) [eV]**	0.68

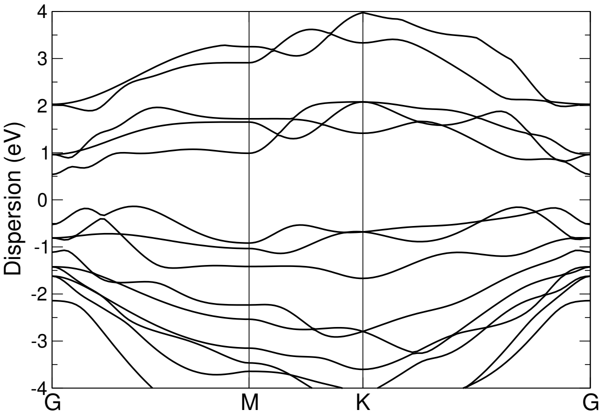

Band structure: Electronic band structure of Sb_2Te_3 (P-3m1) in a representative window around the Fermi energy and along a high-symmetry path.

Optimized crystal structure

Structural parameters: relaxed cell (top) and atomic positions (bottom) of Sb_2Te_3 (P-3m1) in Cartesian coordinates.

	x [Å]	y [Å]	z [Å]
$\mathbf{a_1}$	4.31311481	0.00000000	0.00000000
$\mathbf{a_2}$	−2.15655741	3.73526700	0.00000000
$\mathbf{a_3}$	0.00000000	0.00000000	27.49254885

		x [Å]	y [Å]	z [Å]
●	Sb	0.00000000	2.49017800	11.74391041
●	Te	0.00000000	2.49017800	17.45328686
●	Sb	0.00000000	0.00000000	15.74863844
●	Te	0.00000000	0.00000000	10.03926200
●	Te	2.15655741	1.24508900	13.74627443

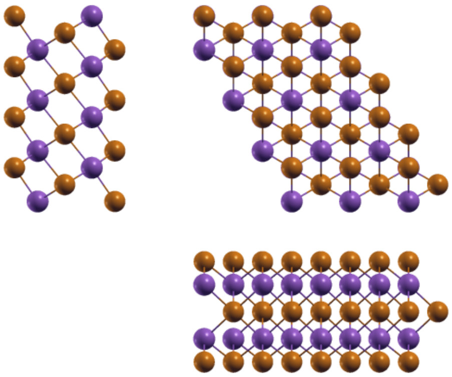

Orthographic projections: views of Sb_2Te_3 (P-3m1) as seen from the x axis (left), the y axis (bottom) and the z axis (center).

Lattice matching - minimal size

Formula	N° atoms	strain	cell size 1	cell size 2
InSe	7	0.0074	1	1
AsSb	7	0.261	1	1
Bi_2	7	0.0017	1	1
GeTe	7	0.2696	1	1
S_2	7	0.2719	1	1
PbTe	7	0.0057	1	1
CaCl	7	0.4435	1	1
$IrTe_2$	8	0.2709	1	1
$CdCl_2$	8	0.2681	1	1
CdI_2	8	0.004	1	1
PSn_2	8	1.529	1	1
Br_2Zn	8	0.2472	1	1
Br_2Ca	8	0.0028	1	1
$InSe_2$	8	0.2686	1	1
$AsSn_2$	8	1.5561	1	1
$GeTe_2$	8	0.2666	1	1
$SiTe_2$	8	1.6375	1	1
I_2Pr	8	0.1307	1	1
I_2Mn	8	0.2682	1	1
S_2Zr	8	1.5236	1	1
Br_2Cu	8	0.6916	1	1
NSr_2	8	0.2633	1	1
PbS_2	8	0.2574	1	1
BiClTe	8	0.0036	1	1
Cl_2Zn	8	0.1164	1	1
FeI_2	8	0.2651	1	1
I_2Ni	8	0.2669	1	1
Te_2Ti	8	0.2476	1	1
CrI_2	8	0.2644	1	1
BiBrTe	8	0.0019	1	1
I_2Nd	8	0.1314	1	1
$NiTe_2$	8	1.633	1	1
Cl_2Cu	8	0.07	1	1
S_2Sn	8	1.5255	1	1
I_2V	8	0.2544	1	1
GeI_2	8	0.0054	1	1
Se_2Zr	8	1.6406	1	1
$PtSe_2$	8	1.5608	1	1
BiITe	8	0.0086	1	1
CoI_2	8	0.2616	1	1
$MnSe_2$	8	0.4433	1	1
CeI_2	8	0.1302	1	1
Br_2Mg	8	0.2649	1	1
I_2Ti	8	0.2621	1	1
$NbTe_2$	8	1.5221	1	1
GdI_2	8	0.0048	1	1
F_2Ni	8	0.1146	1	1
I_2La	8	0.135	1	1
F_2Na	8	0.2498	1	1
CdI_2	8	0.0044	1	1

Table showing the first 50 lattice matching combinations with the smallest number of atoms in the supercell.

Lattice matching - minimal strain

Formula	N° atoms	strain	cell size 1	cell size 2
Br_2V	155	0.0	16	25
KS_2Ti	269	0.0	25	36
Ga_2Se_2	443	0.0	43	57
N_2W	545	0.0	49	100
$F_2Se_2Y_2$	789	0.0001	81	64
Ni_2Te_2	805	0.0001	81	100
CdClHO	376	0.0001	36	49
GeI_3Rb	290	0.0001	49	9
$Hf_2I_2N_2$	474	0.0001	36	49
CrI_2	638	0.0002	73	91
C_2	178	0.0003	16	49
$Cl_2O_2Y_2$	723	0.0003	57	73
$CdCl_2$	705	0.0003	81	100
$Cl_2O_2Yb_2$	629	0.0003	49	64
Br_2Mn	233	0.0004	25	36
Cl_2Mn	368	0.0004	37	61
Br_2Pr_2	501	0.0004	49	64
Br_2Mg	638	0.0004	73	91
$Cl_2Ho_2O_2$	723	0.0004	57	73
F_2Na	437	0.0004	49	64
I_2Mn	705	0.0005	81	100
LiO	77	0.0005	9	16
I_2Ni	705	0.0005	81	100
PTe_2Zr_2	725	0.0005	64	81
FeH_2O_2	590	0.0005	39	79
FeI_2	638	0.0005	73	91
$LiOS_2Ti$	650	0.0005	49	81
Cu_4Te_2	11	0.0006	1	1
Bi_2Te_2	824	0.0006	100	81
$C_2I_2La_2$	638	0.0007	58	58
$GeTe_2$	705	0.0007	81	100
Cl_2Y_2	443	0.0007	43	57
$TaTe_2$	327	0.0007	36	49
$InSe_2$	705	0.0007	81	100
$Br_2Ga_2Te_2$	809	0.0007	91	59
Bi_2Se_2	620	0.0007	72	65
NSr_2	638	0.0007	73	91
I_2V	504	0.0007	57	73
Cl_2Zr_2	180	0.0008	16	25
$ReSe_2$	155	0.0008	16	25
Ga_2Se_2	577	0.0008	57	73
Cl_2NSc_2	490	0.0008	37	61
Cl_2Zn	233	0.0008	25	36
Ca_2O_2	877	0.0009	81	118
AgTl	369	0.0009	53	52
Se_2Sn	638	0.001	73	91
H_2Si_2	805	0.001	81	100
$I_2N_2Zr_2$	557	0.001	43	57
Bi_2SeTe_2	10	0.0011	1	1
$AgNO_3$	305	0.0011	36	25

Table showing the first 50 lattice matching combinations with the smallest strain needed to build the supercell.

Sb_2TeSe_2 (P-3m1)

Structural and electronic properties

**Formula**	Sb_2TeSe_2
**Spacegroup**	P-3m1
**Prototype**	Bi2Te2S
**Parent 3D**	Sb_2TeSe_2
**Source DB**	ICSD
**DB ID**	651529
**DF2-C09 Binding energy [meV/ Å^2]**	22.52
**RVV10 Binding energy [meV/ Å^2]**	28.78
**Band gap (PBE) [eV]**	0.76

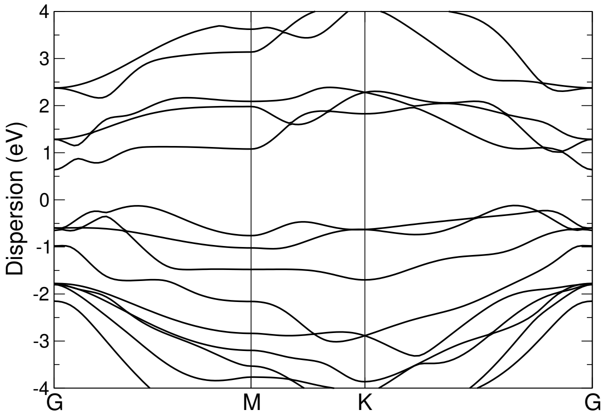

Band structure: Electronic band structure of Sb_2TeSe_2 (P-3m1) in a representative window around the Fermi energy and along a high-symmetry path.

Optimized crystal structure

Structural parameters: relaxed cell (top) and atomic positions (bottom) of Sb_2TeSe_2 (P-3m1) in Cartesian coordinates.

		x [Å]	y [Å]	z [Å]
$\mathbf{a_1}$		4.14862460	0.00000000	0.00000000
$\mathbf{a_2}$		−2.07431230	3.59281429	0.00000000
$\mathbf{a_3}$		0.00000000	0.00000000	27.27327826
		x [Å]	y [Å]	z [Å]
●	Sb	2.07431230	1.19760476	15.71944173
●	Se	2.07431230	1.19760476	10.04833035
●	Sb	0.00000000	0.00000000	11.55383653
●	Te	0.00000000	2.39520953	13.63663913
●	Se	0.00000000	0.00000000	17.22494791

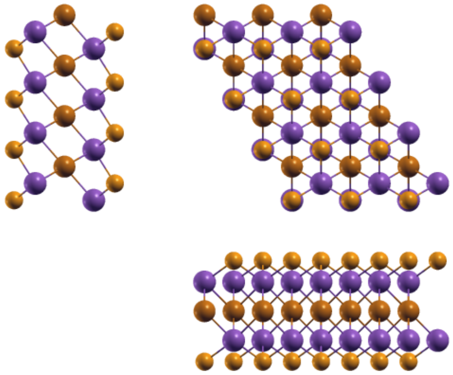

Orthographic projections: views of Sb_2TeSe_2 (P-3m1) as seen from the x axis (left), the y axis (bottom) and the z axis (center).

Lattice matching - minimal size

Formula	N° atoms	strain	cell size 1	cell size 2
K	6	0.4526	1	1
Na	6	1.5397	1	1
AgTl	7	0.1637	1	1
Ag_2	7	0.4662	1	1
As_2	7	1.5976	1	1
Sb_2	7	0.0051	1	1
CaCl	7	0.1183	1	1
Cl_2Zn	8	1.5898	1	1
I_2Mg	8	0.0006	1	1
$MoTe_2$	8	1.5559	1	1
PSn_2	8	0.2575	1	1
Ba_2Pt	8	0.4656	1	1
Br_2Zn	8	0.2727	1	1
HfS_2	8	0.2492	1	1
$AsSn_2$	8	0.2627	1	1
I_2Pr	8	0.1458	1	1
$CuTe_2$	8	0.2487	1	1
S_2Zr	8	0.2564	1	1
Br_2La	8	0.0009	1	1
Ca_2Si	8	0.4771	1	1
Br_2Co	8	1.5944	1	1
Ca_2N	8	0.2463	1	1
BrCdI	8	0.0037	1	1
Cl_2Zn	8	0.1268	1	1
Te_2Ti	8	0.2731	1	1
I_2Zn	8	0.0082	1	1
BaF_2	8	0.0071	1	1
$RhTe_2$	8	0.2668	1	1
GeI_2	8	0.0013	1	1
Br_2Mn	8	1.581	1	1
PtS_2	8	1.547	1	1
$CoTe_2$	8	0.2496	1	1
Br_2V	8	1.4478	1	1
CdClO	8	1.5663	1	1
Se_2Ti	8	1.5282	1	1
AsKSn	8	0.0056	1	1
Te_2W	8	1.5571	1	1
$PbTe_2$	8	0.0027	1	1
I_2Nd	8	0.1466	1	1
S_2Sn	8	0.2568	1	1
$SnTe_2$	8	0.0035	1	1
Sn	8	0.6292	1	3
I_2Pb	8	0.4702	1	1
STl_2	8	0.0081	1	1
$PtSe_2$	8	0.2637	1	1
Br_2Fe	8	1.5949	1	1
GeS_2	8	0.1129	1	1
$TaTe_2$	8	0.2619	1	1
$MnSe_2$	8	0.1183	1	1
Br_2Ni	8	1.6328	1	1

Table showing the first 50 lattice matching combinations with the smallest number of atoms in the supercell.

Lattice matching - minimal strain

Formula	N° atoms	strain	cell size 1	cell size 2
PtS_2	327	0.0	36	49
HfS_2	437	0.0001	49	64
LiO	307	0.0001	37	61
$ReSe_2$	233	0.0001	25	36
$I_2Nd_2S_2$	986	0.0002	100	81
Ag_2	601	0.0003	91	73
$CoTe_2$	437	0.0003	49	64
Se_2W	155	0.0003	16	25
Br_2Co	386	0.0003	43	57
N_3W_2	590	0.0003	39	79
Tl	105	0.0003	16	25
C_2Li_2	356	0.0003	40	39
$Ce_2I_2Si_2$	11	0.0003	1	1
Br_2Fe	386	0.0003	43	57
Cl_2Zn	386	0.0003	43	57
Ga_2S_2	501	0.0004	49	64
Ca_2Si	597	0.0004	81	64
$CuTe_2$	437	0.0004	49	64
$Ce_2I_2S_2$	789	0.0004	81	64
$MoSe_2$	155	0.0004	16	25
$I_2Pr_2S_2$	893	0.0005	91	73
Sb_2Se_2Te	10	0.0005	1	1
$PtSe_2$	638	0.0005	73	91
$AgBrO_2$	665	0.0005	73	75
$NbTe_2$	504	0.0005	57	73
Ba_2Pt	674	0.0005	91	73
$CrSe_2$	488	0.0005	49	81
I_2Mg	8	0.0006	1	1
CCl_2Lu_2	500	0.0006	43	57
$RhTe_2$	705	0.0006	81	100
NS_2Zr	376	0.0006	36	49
Ag_2I_2	517	0.0006	65	48
Ga_2S_3	425	0.0007	36	49
As_2	329	0.0007	43	57
MnO_2	47	0.0007	4	9
CBr_2Lu_2	650	0.0007	57	73
S_2Zr	504	0.0007	57	73
Au_2Se_2	475	0.0007	63	40
$Br_2N_2Zr_2$	723	0.0008	57	73
Cl_2Ni	233	0.0008	25	36
$HfLiS_2$	376	0.0008	36	49
$BiTe_2$	8	0.0008	1	1
$Br_2N_2Ti_2$	857	0.0008	73	82
CaH_2O_2	565	0.0009	49	64
Sb_2Te_2	661	0.0009	81	64
Br_2La	8	0.0009	1	1
Br_2V	233	0.0009	25	36
CdClHO	729	0.0009	73	91
Bi_2Se_3	10	0.001	1	1
S_2Sn	504	0.001	57	73

Table showing the first 50 lattice matching combinations with the smallest strain needed to build the supercell.

Sb_2TeSe_2 (P3m1)

Structural and electronic properties

**Formula**	Sb_2TeSe_2
**Spacegroup**	P3m1
**Prototype**	Cu5FeS4(hR5)
**Parent 3D**	Sb_2TeSe_2
**Source DB**	COD
**DB ID**	1008844
**DF2-C09 Binding energy [meV/ Å^2]**	24.32
**RVV10 Binding energy [meV/ Å^2]**	29.88
**Band gap (PBE) [eV]**	0.72

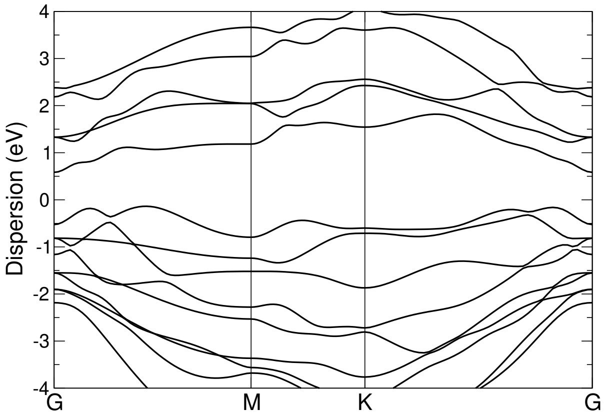

Band structure: Electronic band structure of Sb_2TeSe_2 (P3m1) in a representative window around the Fermi energy and along a high-symmetry path.

Optimized crystal structure

Structural parameters: relaxed cell (top) and atomic positions (bottom) of Sb_2TeSe_2 (P3m1) in Cartesian coordinates.

	x [Å]	y [Å]	z [Å]
$\mathbf{a_1}$	4.14460170	0.00000000	0.00000000
$\mathbf{a_2}$	−2.07230085	3.58933036	0.00000000
$\mathbf{a_3}$	0.00000000	0.00000000	27.08676926

	x [Å]	y [Å]	z [Å]
Se	2.07230085	1.19644345	13.41656565
Sb	0.00000000	0.00000000	15.26294491
Se	0.00000000	0.00000000	10.03970597
Sb	0.00000000	2.39288690	11.54225832
Te	0.00000000	2.39288690	17.05230560

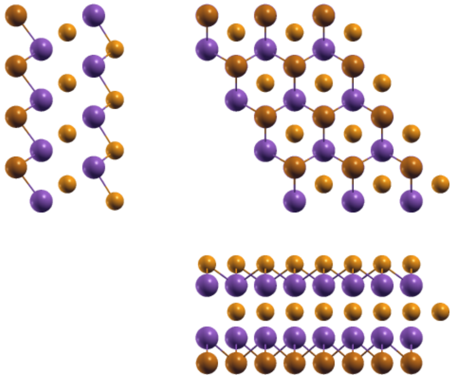

Orthographic projections: views of Sb_2TeSe_2 (P3m1) as seen from the x axis (left), the y axis (bottom) and the z axis (center).

Lattice matching - minimal size

Formula	N° atoms	strain	cell size 1	cell size 2
K	6	0.4537	1	1
Na	6	1.5429	1	1
AgTl	7	0.1642	1	1
Ag_2	7	0.4674	1	1
As_2	7	0.2462	1	1
Sb_2	7	0.0046	1	1
CaCl	7	0.1186	1	1
Cl_2Zn	8	1.5932	1	1
I_2Mg	8	0.001	1	1
$MoTe_2$	8	1.5592	1	1
PSn_2	8	0.2581	1	1
Ba_2Pt	8	0.4667	1	1
Br_2Zn	8	0.2733	1	1
HfS_2	8	0.2498	1	1
$AsSn_2$	8	0.2634	1	1
I_2Pr	8	0.1462	1	1
$CuTe_2$	8	0.2493	1	1
S_2Zr	8	0.2571	1	1
Br_2La	8	0.0014	1	1
Ca_2Si	8	0.4782	1	1
Br_2Co	8	1.5978	1	1
Ca_2N	8	0.2469	1	1
BrCdI	8	0.0042	1	1
Cl_2Zn	8	0.1271	1	1
Te_2Ti	8	0.2738	1	1
I_2Zn	8	0.0078	1	1
BaF_2	8	0.0076	1	1
$RhTe_2$	8	0.2674	1	1
GeI_2	8	0.0008	1	1
Br_2Mn	8	1.5844	1	1
PtS_2	8	1.5503	1	1
$CoTe_2$	8	0.2502	1	1
Se_2Ti	8	1.5314	1	1
AsKSn	8	0.0061	1	1
Te_2W	8	1.5604	1	1
$PbTe_2$	8	0.0032	1	1
I_2Nd	8	0.1471	1	1
S_2Sn	8	0.2574	1	1
$SnTe_2$	8	0.0031	1	1
Sn	8	0.6307	1	3
Cl_2V	8	4.8469	1	1
I_2Pb	8	0.4714	1	1
STl_2	8	0.0086	1	1
$PtSe_2$	8	0.2643	1	1
Br_2Fe	8	1.5983	1	1
GeS_2	8	0.1131	1	1
$TaTe_2$	8	0.2626	1	1
$MnSe_2$	8	0.1185	1	1
Br_2Ni	8	1.6363	1	1
CeI_2	8	0.1455	1	1

Table showing the first 50 lattice matching combinations with the smallest number of atoms in the supercell.

Lattice matching - minimal strain

Formula	N° atoms	strain	cell size 1	cell size 2
$PtSe_2$	638	0.0	73	91
$MoSe_2$	155	0.0	16	25
$CuTe_2$	437	0.0	49	64
Ba_2Pt	674	0.0001	91	73
$CrSe_2$	488	0.0001	49	81
Cl_2Zn	386	0.0001	43	57
$RhTe_2$	705	0.0001	81	100
NS_2Zr	376	0.0001	36	49
N_3W_2	590	0.0002	39	79
Se_2W	155	0.0002	16	25
Ag_2	601	0.0002	91	73
Ga_2S_3	425	0.0002	36	49
Ag_2I_2	517	0.0002	65	48
$AgBrO_2$	674	0.0002	74	76
MnO_2	47	0.0003	4	9
CBr_2Lu_2	650	0.0003	57	73
$I_2Nd_2S_2$	986	0.0003	100	81
$AgBrO_2$	665	0.0003	73	75
$Br_2N_2Zr_2$	723	0.0003	57	73
$BiTe_2$	8	0.0003	1	1
$ReSe_2$	233	0.0003	25	36
CaH_2O_2	565	0.0004	49	64
HfS_2	437	0.0004	49	64
Sb_2Te_2	661	0.0004	81	64
Sb_2Se_2Te	10	0.0005	1	1
Br_2V	233	0.0005	25	36
PtS_2	327	0.0005	36	49
ClH_3O	500	0.0005	57	43
Na	229	0.0006	36	49
LiO	307	0.0006	37	61
Se_2Yb	8	0.0006	1	1
$Cl_2N_2Zr_2$	629	0.0006	49	64
$AsSn_2$	638	0.0007	73	91
KS_2Ti	443	0.0007	43	57
$CrSe_2$	368	0.0007	37	61
$AgBrO_2$	656	0.0007	72	74
$CoTe_2$	437	0.0007	49	64
Br_2Co	386	0.0007	43	57
Tl	105	0.0008	16	25
C_2Li_2	356	0.0008	40	39
$Ce_2I_2Si_2$	11	0.0008	1	1
Br_2Fe	386	0.0008	43	57
$C_2Cl_2Y_2$	692	0.0008	58	67
$AgBrO_2$	373	0.0008	41	42
GeI_2	8	0.0008	1	1
PSn_2	563	0.0009	64	81
Ga_2S_2	501	0.0009	49	64
Ca_2Si	597	0.0009	81	64
$Ce_2I_2S_2$	789	0.0009	81	64
CdClHO	805	0.0009	81	100

Table showing the first 50 lattice matching combinations with the smallest strain needed to build the supercell.

SbTeI (C2/m)

Structural and electronic properties

**Formula**	SbTeI
**Spacegroup**	C2/m
**Prototype**	SbTeI
**Parent 3D**	$Sb_2Te_2I_2$
**Source DB**	COD
**DB ID**	1008205
**DF2-C09 Binding energy [meV/ Å^2]**	17.67
**RVV10 Binding energy [meV/ Å^2]**	N/A
**Band gap (PBE) [eV]**	0.87

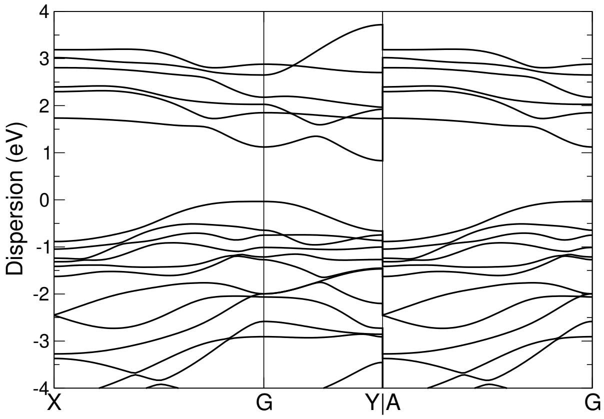

Band structure: Electronic band structure of SbTeI (C2/m) in a representative window around the Fermi energy and along a high-symmetry path.

Optimized crystal structure

Structural parameters: relaxed cell (top) and atomic positions (bottom) of SbTeI (C2/m) in Cartesian coordinates.

	x [Å]	y [Å]	z [Å]
$\mathbf{a_1}$	4.32306746	0.00042175	0.00000000
$\mathbf{a_2}$	2.16053073	7.31150964	0.00000000
$\mathbf{a_3}$	0.00000000	0.00000000	24.03963856

		x [Å]	y [Å]	z [Å]
●	Sb	4.32384389	4.99758114	11.98720753
●	Sb	4.32428461	1.28604950	12.05242755
●	Te	4.32341674	7.35113027	10.36118804
●	Te	2.16216682	6.24358276	13.67845276
●	I	2.16246792	3.86889625	9.90651214
●	I	2.16258252	2.41429512	14.13312766

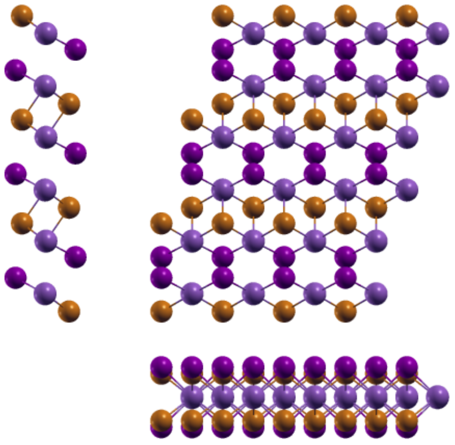

Orthographic projections: views of SbTeI (C2/m) as seen from the x axis (left), the y axis (bottom) and the z axis (center).

Lattice matching - minimal size

Formula	N° atoms	strain	cell size 1	cell size 2
K	8	0.0926	1	2
O_2Zn	9	0.1643	1	1
Ag_2	10	0.767	1	2
S_2	10	0.5096	1	2
Ge_2Se_2	10	0.7667	1	1
Sb_2	10	0.0651	1	2
CaCl	10	0.0705	1	2
FeH_2O_2	11	0.1639	1	1
I_2Mg	12	0.0661	1	2
Ba_2Pt	12	0.766	1	2
$GeTe_2$	12	0.5001	1	2
$HfTe_2$	12	0.0647	1	2
Hg_4O_2	12	0.8503	1	1
Br_2La	12	0.0662	1	2
$AuTe_2$	12	0.0644	1	2
BrCdI	12	0.0669	1	2
Cl_2Zn	12	0.0694	1	2
$PdTe_2$	12	0.0644	1	2
I_2Zn	12	0.0647	1	2
GeI_2	12	0.0657	1	2
Ba_2N	12	0.0646	1	2
Te_2Zr	12	0.0646	1	2
$SnTe_2$	12	0.0653	1	2
$MnSe_2$	12	0.0705	1	2
DyI_2	12	0.7229	1	2
Se_2Yb	12	0.0657	1	2
$BiTe_2$	12	0.0658	1	2
F_2Ni	12	0.0694	1	2
$PtTe_2$	12	0.0644	1	2
Br_2Cd	12	0.0644	1	2
F_2Na	12	0.4692	1	2
$AuCrTe_4$	12	0.0678	1	1
NaPSn	12	0.0648	1	2
Fe_2Te_2	14	0.0704	1	2
Ca_2Cl_2	14	0.0704	1	2
Cl_2Gd_2	14	0.5156	1	2
Cl_2OOs	14	0.0716	1	2
Br_2Pr_2	14	0.4673	1	2
Br_2Er_2	14	0.0644	1	2
Br_2Tb_2	14	0.0644	1	2
Fe_2Se_2	14	0.0695	1	2
Cl_2ORu	14	0.0721	1	2
As_2Co_2	14	0.0698	1	2
Cu_2Te_2	14	0.0694	1	2
Cl_2La_2	14	0.0645	1	2
Br_2Gd_2	14	0.0644	1	2
Br_2OV	14	0.0744	1	2
$AsCuLi_2$	14	0.0656	1	2
Cu_2I_2	14	0.0663	1	2
Fe_2S_2	14	0.0709	1	2

Table showing the first 50 lattice matching combinations with the smallest number of atoms in the supercell.

Lattice matching - minimal strain

Formula	N° atoms	strain	cell size 1	cell size 2
$C_2I_2La_2$	570	0.0002	32	63
K_2PdS_2	605	0.0002	60	49
HgO	144	0.0003	12	36
$CuGeO_3$	518	0.0005	33	64
$BrKO_3$	630	0.0005	65	48
$LiNbS_2$	676	0.0006	36	115
Cu_2F_4	828	0.0006	65	73
S_2Ta	561	0.0007	36	115
CoO_2	297	0.0008	15	69
Ge_2S_2	706	0.001	51	100
Mg_3	612	0.001	39	126
$SbSe_2Tl$	328	0.001	28	40
NbS_2	561	0.001	36	115
ClH_3O	953	0.0011	68	109
Li_2Tl_2	492	0.0011	42	60
$AgClO_2$	424	0.0011	28	64
NbS_2	438	0.0011	28	90
Cl_2NSc_2	618	0.0012	28	90
$Fe_2Li_2P_2$	342	0.0012	17	40
K_2PdSe_2	795	0.0013	80	63
NiO_2	297	0.0013	15	69
Hg_4O_2	768	0.0014	85	43
S_2Ta	438	0.0014	28	90
$LiNbS_2$	528	0.0015	28	90
CNRb	792	0.0015	78	108
Co_2Se_2	850	0.0015	55	130
Cl_2Mn	438	0.0015	28	90
As_2O_3	550	0.0016	45	56
$Hf_2Si_2Te_2$	768	0.0016	38	90
IO_3Tl	561	0.0016	56	45
Co_2Se_2	588	0.0017	38	90
$Er_2F_2Se_2$	816	0.0017	41	95
Bi_2Te_2	492	0.0018	40	63
Te_4W_2	990	0.0018	68	97
$Hf_2Si_2Te_2$	666	0.0018	33	78
$Br_2S_2Y_2$	900	0.0019	61	89
$Cl_2N_2Zr_2$	948	0.0019	50	108
Fe_2Se_2	262	0.0019	17	40
CNRb	507	0.0019	50	69
$Hf_2Si_2Te_2$	342	0.0019	17	40
Cl_4Mg_2	792	0.002	78	54
Br_2Pr_2	912	0.002	56	144
I_2Ti	537	0.002	40	99
Co_2Se_2	510	0.0021	33	78
$C_2Br_2La_2$	936	0.0021	51	105
$Cl_2Hg_2N_2$	774	0.0022	62	67
Cu_2O_2	302	0.0022	21	44
Bi_2Se_4	516	0.0022	44	42
O_2Sn_2	798	0.0023	55	117
Au_2Se_2	888	0.0023	78	105

Table showing the first 50 lattice matching combinations with the smallest strain needed to build the supercell.

Sc_2CCl_2 (P-3m1)

Structural and electronic properties

**Formula**	Sc_2CCl_2
**Spacegroup**	P-3m1
**Prototype**	Bi2Te2S
**Parent 3D**	Sc_2CCl_2
**Source DB**	ICSD
**DB ID**	59124
**DF2-C09 Binding energy [meV/ Å^2]**	13.23
**RVV10 Binding energy [meV/ Å^2]**	19.94
**Band gap (PBE) [eV]**	0.88

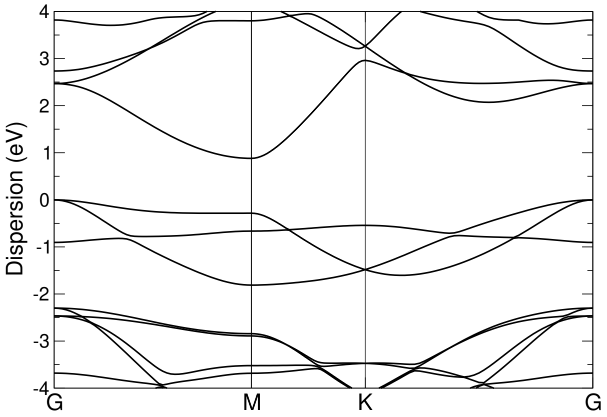

Band structure: Electronic band structure of Sc_2CCl_2 (P-3m1) in a representative window around the Fermi energy and along a high-symmetry path.

Optimized crystal structure

Structural parameters: relaxed cell (top) and atomic positions (bottom) of Sc_2CCl_2 (P-3m1) in Cartesian coordinates.

		x [Å]	y [Å]	z [Å]
$\mathbf{a_1}$		3.44183358	0.00000000	0.00000000
$\mathbf{a_2}$		−1.72091679	2.98071532	0.00000000
$\mathbf{a_3}$		0.00000000	0.00000000	25.77438891
		x [Å]	y [Å]	z [Å]
	Sc	0.00000000	1.98714355	11.65355423
	Sc	1.72091679	0.99357177	14.12083468
	C	0.00000000	0.00000000	12.88719445
	Cl	0.00000000	1.98714355	15.79128105
	Cl	1.72091679	0.99357177	9.98310786

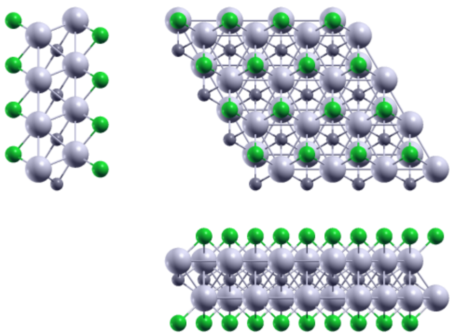

Orthographic projections: views of Sc_2CCl_2 (P-3m1) as seen from the x axis (left), the y axis (bottom) and the z axis (center).

Lattice matching - minimal size

Formula	N° atoms	strain	cell size 1	cell size 2
Tl	6	0.136	1	1
Sn	6	0.1158	1	1
In	6	0.1179	1	1
HgO	7	0.1464	1	1
AsSb	7	0.4672	1	1
GeTe	7	0.4827	1	1
S_2	7	0.4868	1	1
Mg_2	7	0.1244	1	1
$IrTe_2$	8	0.485	1	1
CrS_2	8	0.2601	1	1
$CdCl_2$	8	0.4799	1	1
$AgTe_2$	8	0.1384	1	1
$ReSe_2$	8	0.002	1	1
S_2Ta	8	0.0089	1	1
$InSe_2$	8	0.4809	1	1
$GeTe_2$	8	0.4773	1	1
$SiTe_2$	8	0.452	1	1
$HfTe_2$	8	2.9262	1	1
I_2Mn	8	0.4802	1	1
NSr_2	8	0.4713	1	1
PbS_2	8	0.4607	1	1
ReS_2	8	0.2686	1	1
$AuTe_2$	8	3.0293	1	1
LiO_2	8	0.3035	1	1
$PdTe_2$	8	2.9965	1	1
FeI_2	8	0.4745	1	1
I_2Ni	8	0.4778	1	1
S_2Ti	8	0.004	1	1
Mg_3	8	0.1315	1	1
CrI_2	8	0.4734	1	1
GeI_2	8	3.191	1	1
Ba_2Hg	8	0.3257	1	1
N_2W	8	1.6276	1	1
Cl_2Ni	8	0.0029	1	1
Cl_2Co	8	0.0044	1	1
$CrTe_2$	8	0.0092	1	1
Br_2V	8	0.0012	1	1
ClNZr	8	0.0009	1	1
Cl_2Fe	8	0.0051	1	1
Br_2Ti	8	0.0086	1	1
Te_2Zr	8	2.9326	1	1
$AsSe_2$	8	0.0044	1	1
$NiTe_2$	8	0.4506	1	1
Cl_2Cu	8	0.1033	1	1
I_2V	8	0.4553	1	1
Se_2Zr	8	0.453	1	1
CdO_2	8	0.0045	1	1
BrNZr	8	0.0057	1	1
$NbSe_2$	8	0.0033	1	1
CoI_2	8	0.4683	1	1

Table showing the first 50 lattice matching combinations with the smallest number of atoms in the supercell.

Lattice matching - minimal strain

Formula	N° atoms	strain	cell size 1	cell size 2
In_2S_3	650	0.0	73	57
NaPSn	414	0.0	57	43
$PdTe_2$	353	0.0	49	36
$AgNO_3$	65	0.0	9	4
Bi_2SeTe_2	205	0.0	25	16
O_2Zn	504	0.0001	57	73
Cd_2I_3	490	0.0001	61	37
AsI_2La_2	490	0.0001	61	37
AsSb	601	0.0001	91	73
Br_2HLa	280	0.0001	36	25
Pt_2Te_2	457	0.0002	57	43
I_2Y_2	516	0.0002	64	49
PbS_2	743	0.0002	100	81
GeTe	479	0.0002	73	57
CoH_2O_2	905	0.0003	81	100
$C_2Br_2Y_2$	663	0.0003	69	53
$AsCuLi_2$	280	0.0003	36	25
Cl_2Tb_2	516	0.0003	64	49
$Br_2H_2Zr_2$	11	0.0004	1	1
Cl_2Zr_2	9	0.0004	1	1
Br_2Y_2	824	0.0004	100	81
$I_2Pr_2Si_2$	330	0.0005	36	25
C	9	0.0005	1	4
HgI_2	255	0.0005	39	20
$CeLi_2P_2$	305	0.0005	36	25
$GeTe_2$	597	0.0005	81	64
Bi_2	157	0.0005	25	16
CoI_2	674	0.0005	91	73
Br_2Tb_2	389	0.0006	49	36
FeI_2	597	0.0006	81	64
H_2Si_2	593	0.0006	73	57
Ga_2Te_2	280	0.0006	36	25
$GeNi_3Te_2$	543	0.0006	57	43
PTe_2Zr_2	820	0.0006	91	73
CrS_2	563	0.0006	64	81
Ba_2Ni_3	305	0.0006	36	25
$Cl_2S_2Tl_2$	974	0.0006	130	54
$Er_2F_2Se_2$	543	0.0007	57	43
Cu_2Te_2	517	0.0007	65	48
$IrTe_2$	536	0.0007	73	57
Br_2Mg	597	0.0007	81	64
I_2Ni	597	0.0007	81	64
Cl_2OV	467	0.0007	55	48
ReS_2	705	0.0007	81	100
Br_2Cu_2	914	0.0008	118	81
Te_4TiZr	597	0.0008	81	32
NaO_4	65	0.0008	9	4
Ga_2Se_2	824	0.0008	100	81
$LiMnSe_2$	516	0.0008	64	49
GeI_2	255	0.0008	36	25

Table showing the first 50 lattice matching combinations with the smallest strain needed to build the supercell.

Sc_2NCl_2 (P-3m1)

Structural and electronic properties

**Formula**	Sc_2NCl_2
**Spacegroup**	P-3m1
**Prototype**	Bi2Te2S
**Parent 3D**	Sc_2NCl_2
**Source DB**	ICSD
**DB ID**	59125
**DF2-C09 Binding energy [meV/ Å^2]**	14.4
**RVV10 Binding energy [meV/ Å^2]**	21.25
**Band gap (PBE) [eV]**	N/A

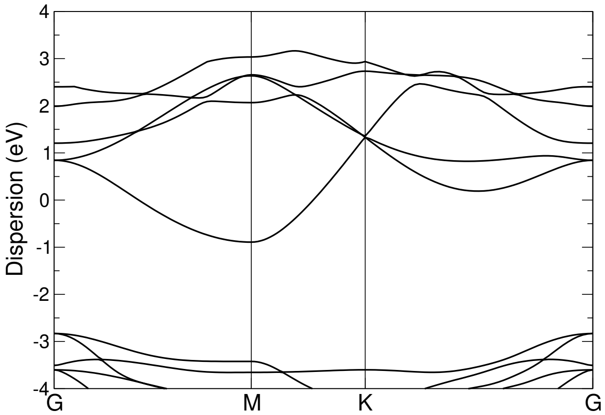

Band structure: Electronic band structure of Sc_2NCl_2 (P-3m1) in a representative window around the Fermi energy and along a high-symmetry path.

Optimized crystal structure

Structural parameters: relaxed cell (top) and atomic positions (bottom) of Sc_2NCl_2 (P-3m1) in Cartesian coordinates.

		x [Å]	y [Å]	z [Å]
$\mathbf{a_1}$		3.36507662	0.00000000	0.00000000
$\mathbf{a_2}$		−1.68253831	2.91424184	0.00000000
$\mathbf{a_3}$		0.00000000	0.00000000	25.71228735
		x [Å]	y [Å]	z [Å]
	Sc	0.00000000	1.94282790	11.66969692
	Sc	1.68253831	0.97141395	14.04259042
	N	0.00000000	0.00000000	12.85614367
	Cl	0.00000000	1.94282790	15.72975671
	Cl	1.68253831	0.97141395	9.98253063

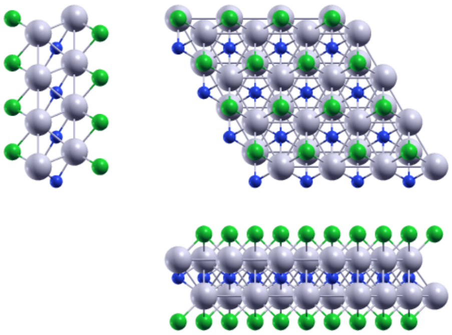

Orthographic projections: views of Sc_2NCl_2 (P-3m1) as seen from the x axis (left), the y axis (bottom) and the z axis (center).

Lattice matching - minimal size

Formula	N° atoms	strain	cell size 1	cell size 2
Tl	6	0.1451	1	1
Tl	6	0.0061	1	1
Sn	6	0.1212	1	1
In	6	0.1238	1	1
Sm	6	4.8621	1	1
AsSb	7	0.4947	1	1
Mg_2	7	0.1317	1	1
CrS_2	8	0.2755	1	1
$CdCl_2$	8	2.9306	1	1
Cl_2Mn	8	0.0005	1	1
$AgTe_2$	8	0.1478	1	1
$MoSe_2$	8	0.0069	1	1
S_2Ta	8	0.0016	1	1
Br_2Zn	8	0.4683	1	1
$InSe_2$	8	2.9355	1	1
$AsSn_2$	8	0.4511	1	1
$SiTe_2$	8	0.4788	1	1
I_2Mn	8	2.9321	1	1
NSr_2	8	0.4991	1	1
FeI_2	8	2.9034	1	1
I_2Ni	8	2.92	1	1
S_2Ti	8	0.0067	1	1
Mg_3	8	0.1399	1	1
Te_2Ti	8	0.469	1	1
NbS_2	8	0.0012	1	1
CrI_2	8	0.5013	1	1
$RhTe_2$	8	0.4581	1	1
N_2W	8	0.2656	1	1
Cl_2Co	8	0.0063	1	1
NbS_2	8	0.0031	1	1
Cl_2Fe	8	0.0056	1	1
S_2Ta	8	0.004	1	1
Se_2V	8	0.0053	1	1
$NiTe_2$	8	0.4772	1	1
Cl_2Cu	8	0.1149	1	1
I_2V	8	0.4822	1	1
Se_2Zr	8	0.4798	1	1
$PtSe_2$	8	0.4527	1	1
CdO_2	8	0.0062	1	1
CoI_2	8	0.4959	1	1
O_2Zn	8	0.2706	1	1
$TaTe_2$	8	0.4497	1	1
Cl_2Zr	8	0.006	1	1
$FeSe_2$	8	0.1178	1	1
Br_2Mg	8	2.9017	1	1
I_2Ti	8	0.4968	1	1
F_2Na	8	0.4733	1	1
Se_2Sn	8	0.4984	1	1
$HfSe_2$	8	0.4691	1	1
Se_2W	8	0.0067	1	1

Table showing the first 50 lattice matching combinations with the smallest number of atoms in the supercell.

Lattice matching - minimal strain

Formula	N° atoms	strain	cell size 1	cell size 2
$Cl_2Ho_2O_2$	707	0.0	73	57
CaI_2	107	0.0001	16	9
CaClHO	661	0.0001	81	64
$I_2N_2Zr_2$	986	0.0001	100	81
Cl_2Gd_2	389	0.0001	49	36
CBr_2Y_2	820	0.0002	91	73
CoI_2	467	0.0002	64	49
$HNiO_2$	577	0.0002	57	73
FeH_2O_2	905	0.0002	81	100
$Cl_2N_2Zr_2$	891	0.0002	99	66
$AuTe_2$	255	0.0002	36	25
$GeTe_2$	414	0.0002	57	43
$Cl_2O_2Tm_2$	789	0.0003	81	64
Cu_4Te_2	527	0.0003	61	37
AsSb	418	0.0003	64	49
$Ga_2I_2Y_2$	221	0.0003	25	16
Br_2Er_2	280	0.0003	36	25
Li_2Tl_2	61	0.0003	9	4
I_2V	536	0.0004	73	57
BiBrTe	552	0.0004	81	49
CCl_2Gd_2	820	0.0004	91	73
I_2Ni	414	0.0004	57	43
Cl_2Mn	8	0.0005	1	1
$NiTe_2$	597	0.0005	81	64
BaF_2	173	0.0005	25	16
I_2Ti	467	0.0005	64	49
Br_2Zn	674	0.0005	91	73
Cl_2Tb_2	389	0.0006	49	36
Sm	326	0.0006	49	81
IO_3Tl	25	0.0006	4	1
Co_2Se_2	517	0.0006	65	48
$PtTe_2$	255	0.0006	36	25
$AgClO_2$	697	0.0006	89	63
$Cl_2O_2Y_2$	707	0.0007	73	57
Cl_2Y_2	747	0.0007	91	73
As_2Co_2	517	0.0008	65	48
Te_4TiZr	828	0.0008	114	43
Bi_2Te_2	696	0.0008	100	49
H_2MnO_2	500	0.0008	43	57
Sb_2Te_3	490	0.0008	61	37
Te_2Ti	674	0.0008	91	73
FeI_2	414	0.0008	57	43
$HfSe_2$	674	0.0009	91	73
N_2W	638	0.0009	73	91
Br_2Gd_2	280	0.0009	36	25
Ga_2Se_2	824	0.0009	100	81
AsKSn	173	0.0009	25	16
Bi_2Te_2	551	0.0009	79	39
CI_2Y_2	565	0.0009	64	49
PTe_2Zr_2	565	0.001	64	49

Table showing the first 50 lattice matching combinations with the smallest strain needed to build the supercell.

$Sc_2O_2Cl_2$ (Pmmn)

Structural and electronic properties

**Formula**	$Sc_2O_2Cl_2$
**Spacegroup**	Pmmn
**Prototype**	FeOCl
**Parent 3D**	$Cl_2O_2Sc_2$
**Source DB**	MPDS
**DB ID**	S1238827
**DF2-C09 Binding energy [meV/ Å^2]**	9.12
**RVV10 Binding energy [meV/ Å^2]**	N/A
**Band gap (PBE) [eV]**	4.25

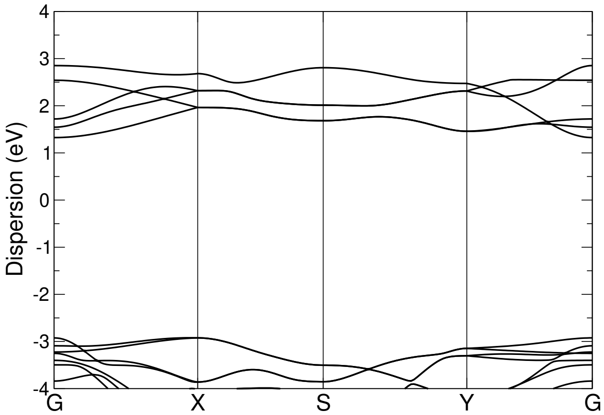

Band structure: Electronic band structure of $Sc_2O_2Cl_2$ (Pmmn) in a representative window around the Fermi energy and along a high-symmetry path.

Optimized crystal structure

Structural parameters: relaxed cell (top) and atomic positions (bottom) of $Sc_2O_2Cl_2$ (Pmmn) in Cartesian coordinates.

		x [Å]	y [Å]	z [Å]
$\mathbf{a_1}$		3.48483330	0.00000000	0.00000000
$\mathbf{a_2}$		0.00000000	3.98654172	0.00000000
$\mathbf{a_3}$		0.00000000	0.00000000	23.00501494
		x [Å]	y [Å]	z [Å]
	Sc	0.87119488	-0.99663543	-0.94607317
	Cl	-0.87121516	-0.99663543	-2.76834945
	O	0.87121050	-2.98990629	-0.31228971
	Sc	-0.87119488	-2.98990629	0.94607317
	Cl	0.87121516	-2.98990629	2.76834945
	O	-0.87121050	-0.99663543	0.31228971

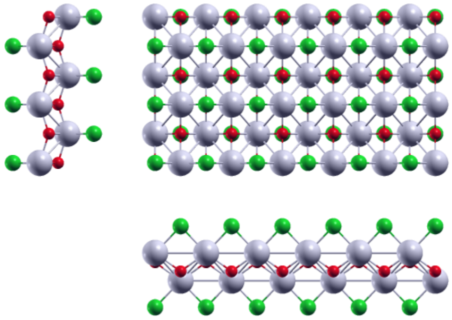

Orthographic projections: views of $Sc_2O_2Cl_2$ (Pmmn) as seen from the x axis (left), the y axis (bottom) and the z axis (center).

Lattice matching - minimal size

Formula	N° atoms	strain	cell size 1	cell size 2
K	7	0.2212	1	1
InSe	8	0.6141	1	1
AgTl	8	0.3217	1	1
CaCl	8	0.2916	1	1
I_2Mg	9	0.5823	1	1
CaI_2	9	0.2079	1	1
I_2Pr	9	0.3039	1	1
Br_2La	9	0.5834	1	1
I_2Yb	9	0.2044	1	1
GeI_2	9	0.5768	1	1
I_2Nd	9	0.3056	1	1
Cl_2Cu	9	0.4542	1	1
I_2Tm	9	0.2062	1	1
GeS_2	9	0.0376	1	1
$MnSe_2$	9	0.2915	1	1
DyI_2	9	0.2119	1	1
CeI_2	9	0.3026	1	1
Se_2Yb	9	0.5775	1	1
$BiTe_2$	9	0.5783	1	1
GdI_2	9	0.6538	1	1
CNNa	9	0.495	1	1
I_2La	9	0.0479	1	1
F_2Zn	9	0.2971	1	1
Cu_2Te_2	10	0.021	1	1
Ir_2P_2	10	0.3052	1	1
Ag_2Br_2	10	0.0484	1	1
$AsLi_3$	10	0.615	1	1
O_2Sn_2	10	0.0182	1	1
Cu_2S_2	10	0.2917	1	1
Cl_2OV	10	0.2726	1	1
Br_2Cu_2	10	0.2948	1	1
As_2Ir_2	10	0.0505	1	1
As_4	10	0.5037	1	1
P_4	10	0.4578	1	1
Br_2OV	10	0.0298	1	1
O_2Sn_2	10	0.2926	1	1
$AsCuLi_2$	10	0.5754	1	1
Cu_2I_2	10	0.5852	1	1
Fe_2S_2	10	0.2883	1	1
LiO	10	1.2485	1	2
Co_2S_2	10	0.2927	1	1
As_2Fe_2	10	0.0372	1	1
Ge_2Se_2	10	0.3275	1	1
$AgClO_2$	10	0.1146	1	1
Gd_2I_2	10	0.5843	1	1
Ni_2Se_2	10	0.3022	1	1
Ga_2Te_2	10	0.5761	1	1
Ba_2Cu_2	10	0.2041	1	1
As_2Rh_2	10	0.0482	1	1
Br_2HLa	10	0.574	1	1

Table showing the first 50 lattice matching combinations with the smallest number of atoms in the supercell.

Lattice matching - minimal strain

Formula	N° atoms	strain	cell size 1	cell size 2
$Ca_2H_2I_2$	90	0.0003	8	7
SSb_2Te_2	635	0.0003	60	55
Nd	385	0.0003	47	103
LiO	408	0.0005	45	69
$Cl_2S_2Tl_2$	468	0.0005	50	28
Ag_2I_2	710	0.0005	81	56
HgI_2	654	0.0006	81	56
I_2Zn	978	0.0006	110	106
Mg_2	826	0.0007	92	137
As_2Ir_2	76	0.0008	8	7
S_2Sn	765	0.001	80	95
CKN	429	0.001	54	35
PSn_2	765	0.001	80	95
Cl_2Cu	318	0.001	34	38
S_2Zr	765	0.001	80	95
$C_2I_2Y_2$	696	0.0011	59	57
$Ho_2I_2S_2$	234	0.0011	24	15
$Br_2Ce_2O_2$	90	0.0011	8	7
$K_2Mn_2Sb_2$	90	0.0011	8	7
In	136	0.0011	18	28
I_2La_2	580	0.0011	60	55
$NbTe_2$	765	0.0012	80	95
Pb_2Se_2	532	0.0012	60	43
$Er_2I_2S_2$	234	0.0012	24	15
$Ag_2K_2Se_2$	876	0.0012	86	60
PSn_2	687	0.0012	72	85
$PbTe_2$	525	0.0012	60	55
Sn_2Te_2	868	0.0013	100	67
$Cl_2Ga_2Te_2$	198	0.0013	21	12
$AgBrO_2$	884	0.0014	90	86
Bi_2In_2	240	0.0015	28	18
Ni_2SbTe_2	889	0.0015	79	83
Sb_2	656	0.0015	83	79
Br_2La_2	814	0.0015	83	79
Li_2P_2Pr	893	0.0015	83	79
$Ga_2Ge_2Te_2$	972	0.0016	83	79
CdClHO	772	0.0016	72	85
Ga_2Se_2	806	0.0016	79	83
$Hf_2I_2N_2$	942	0.0016	72	85
$Br_2Er_2S_2$	276	0.0016	28	18
$Eu_2I_2O_2$	90	0.0016	8	7
$Ge_2Mn_2Sr_2$	90	0.0016	8	7
CrS_2	312	0.0017	28	48
$IrTe_2$	723	0.0017	79	83
$CrSe_2$	477	0.0017	45	69
Cl_2Mg	894	0.0017	93	112
S_2Sn	687	0.0017	72	85
P_2Sn_2	806	0.0017	79	83
Mg_3	945	0.0017	92	131
Br_2Ni	894	0.0017	93	112

Table showing the first 50 lattice matching combinations with the smallest strain needed to build the supercell.

ScCl (P-3m1)

Structural and electronic properties

**Formula**	ScCl
**Spacegroup**	P-3m1
**Prototype**	ZrCl
**Parent 3D**	Sc_2Cl_2
**Source DB**	COD
**DB ID**	4343683
**DF2-C09 Binding energy [meV/ Å^2]**	13.76
**RVV10 Binding energy [meV/ Å^2]**	19.41
**Band gap (PBE) [eV]**	N/A

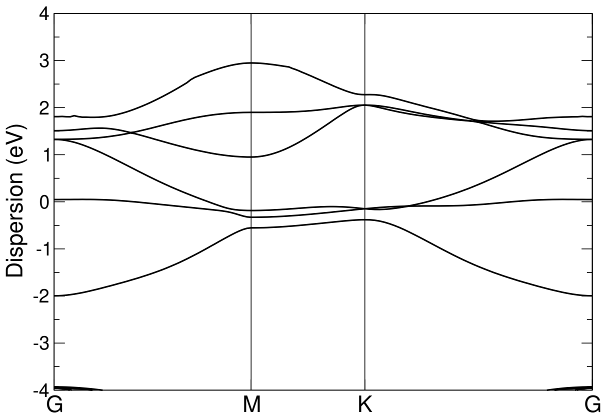

Band structure: Electronic band structure of ScCl (P-3m1) in a representative window around the Fermi energy and along a high-symmetry path.

Optimized crystal structure

Structural parameters: relaxed cell (top) and atomic positions (bottom) of ScCl (P-3m1) in Cartesian coordinates.

	x [Å]	y [Å]	z [Å]
$\mathbf{a_1}$	3.50749222	0.00000000	0.00000000
$\mathbf{a_2}$	−1.75374611	3.03757737	0.00000000
$\mathbf{a_3}$	0.00000000	0.00000000	25.80281558

		x [Å]	y [Å]	z [Å]
	Sc	1.75374611	1.01252579	14.17562019
	Cl	1.75374611	3.03757737	15.79343681
	Cl	1.75374611	3.03757737	10.00937877
	Sc	-0.00000000	2.02505158	11.62719539

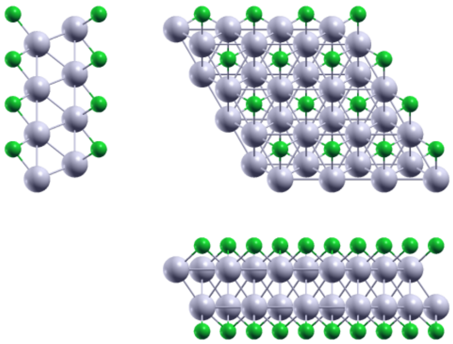

Orthographic projections: views of ScCl (P-3m1) as seen from the x axis (left), the y axis (bottom) and the z axis (center).

Lattice matching - minimal size

Formula	N° atoms	strain	cell size 1	cell size 2
Tl	5	0.1293	1	1
Sn	5	0.1122	1	1
Na	5	0.0055	1	1
In	5	0.1139	1	1
In	5	0.2684	1	1
Gd	5	0.1126	1	1
HgO	6	0.1386	1	1
GeTe	6	0.46	1	1
S_2	6	0.4639	1	1
Mg_2	6	0.1193	1	1
Sb_2	6	3.015	1	1
$IrTe_2$	7	0.4623	1	1
CrS_2	7	0.2481	1	1
S_2V	7	0.273	1	1
MoS_2	7	0.2741	1	1
$CdCl_2$	7	0.4573	1	1
$MoTe_2$	7	0.0078	1	1
$AgTe_2$	7	0.1315	1	1
$ReSe_2$	7	0.0068	1	1
$InSe_2$	7	0.4583	1	1
$GeTe_2$	7	0.4549	1	1
$HfTe_2$	7	0.4835	1	1
I_2Mn	7	0.4576	1	1
NSr_2	7	0.4491	1	1
ReS_2	7	0.2561	1	1
$AuTe_2$	7	2.9111	1	1
$PdTe_2$	7	0.4975	1	1
FeI_2	7	0.4522	1	1
I_2Ni	7	0.4553	1	1
Mg_3	7	0.1254	1	1
CrI_2	7	0.4511	1	1
I_2Zn	7	2.9713	1	1
Te_2Zn	7	0.0077	1	1
S_2W	7	0.2742	1	1
Bi_2Pd	7	0.147	1	1
N_2W	7	1.5617	1	1
Cl_2Ni	7	0.0059	1	1
$CrTe_2$	7	0.0001	1	1
PtS_2	7	0.0066	1	1
Br_2V	7	0.0076	1	1
CdClO	7	0.0093	1	1
Ba_2N	7	2.8311	1	1
Se_2Ti	7	0.0039	1	1
Br_2Ti	7	0.0005	1	1
Te_2Zr	7	0.4848	1	1
Te_2W	7	0.008	1	1
$AsSe_2$	7	0.0045	1	1
STl_2	7	3.1958	1	1
OTl_2	7	0.0095	1	1
BrNZr	7	0.0032	1	1

Table showing the first 50 lattice matching combinations with the smallest number of atoms in the supercell.

Lattice matching - minimal strain

Formula	N° atoms	strain	cell size 1	cell size 2
$Ga_2I_2Y_2$	294	0.0	36	25
$CrTe_2$	7	0.0001	1	1
$F_2Se_2Yb_2$	802	0.0001	91	73
$HfTe_2$	463	0.0001	73	57
$Er_2F_2Se_2$	634	0.0001	73	57
S_2Zn_2	656	0.0001	91	73
Br_2Cd	403	0.0001	64	49
In_2Se_3	577	0.0001	73	57
$Dy_2I_2S_2$	618	0.0001	81	49
NS_2Ta	340	0.0002	36	49
$Br_2O_2Yb_2$	548	0.0002	65	48
BaF_2	219	0.0002	36	25
Br_2Cr	7	0.0002	1	1
P_2Sn_2	656	0.0002	91	73
$AuTe_2$	357	0.0003	57	43
In_2S_3	805	0.0003	100	81
C_2	314	0.0003	39	79
Br_2Er_2	400	0.0003	57	43
$Br_2H_2Zr_2$	10	0.0003	1	1
Br_2PY_2	501	0.0003	64	49
$F_2Lu_2Se_2$	412	0.0004	49	36
$IrTe_2$	643	0.0004	100	81
$AlLiTe_2$	164	0.0005	25	16
ReS_2	447	0.0005	57	73
GeTe	562	0.0005	100	81
$Dy_2I_2S_2$	466	0.0005	61	37
Br_2Ti	7	0.0005	1	1
Fe_2Te_2	452	0.0005	65	48
$Ce_2I_2S_2$	118	0.0005	16	9
DyI_2	471	0.0005	81	49
In	424	0.0005	81	100
Ca_2Si	91	0.0005	16	9
CoH_2O_2	593	0.0006	57	73
$F_2Se_2Tm_2$	708	0.0006	81	64
$BrKO_3$	21	0.0006	4	1
Te_2Zr	463	0.0006	73	57
Pt_2Te_2	520	0.0006	73	57
Bi_2Te_3	180	0.0006	25	16
I_4Zr_2	742	0.0006	115	47
Ga_2Se_2	724	0.0006	100	81
$I_2O_2Y_2$	958	0.0006	118	81
$PtTe_2$	357	0.0007	57	43
$Br_2Nd_2O_2$	958	0.0007	118	81
BiITe	148	0.0007	25	16
$LiMnSe_2$	580	0.0007	81	64
O_2Zn	343	0.0007	43	57
BN	198	0.0007	25	49
NaPSn	463	0.0008	73	57
$PdTe_2$	403	0.0008	64	49
H_2MnO_2	280	0.0008	25	36

Table showing the first 50 lattice matching combinations with the smallest strain needed to build the supercell.

ScHCl (P-3m1)

Structural and electronic properties

**Formula**	ScHCl
**Spacegroup**	P-3m1
**Prototype**	SmSI
**Parent 3D**	$Sc_2H_2Cl_2$
**Source DB**	ICSD
**DB ID**	40981
**DF2-C09 Binding energy [meV/ Å^2]**	13.47
**RVV10 Binding energy [meV/ Å^2]**	19.69
**Band gap (PBE) [eV]**	N/A

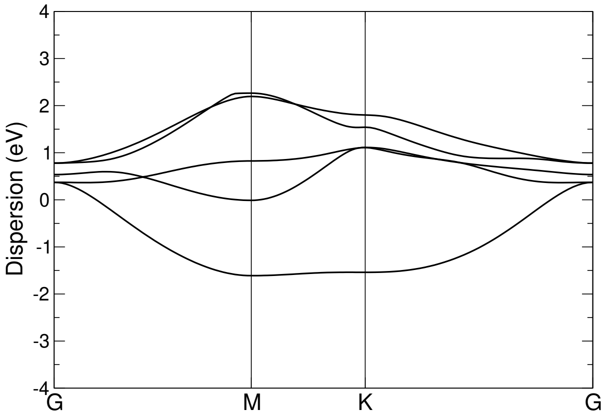

Band structure: Electronic band structure of ScHCl (P-3m1) in a representative window around the Fermi energy and along a high-symmetry path.

Optimized crystal structure

Structural parameters: relaxed cell (top) and atomic positions (bottom) of ScHCl (P-3m1) in Cartesian coordinates.

	x [Å]	y [Å]	z [Å]
$\mathbf{a_1}$	3.49975933	−0.00000000	0.00000000
$\mathbf{a_2}$	−1.74987966	3.03088049	0.00000000
$\mathbf{a_3}$	0.00000000	0.00000000	25.78011031

	x [Å]	y [Å]	z [Å]
Sc	-0.00000000	2.02058699	14.15300754
H	-0.00000000	2.02058699	12.14632113
Cl	1.74987966	3.03088048	15.76694734
Cl	1.74987966	3.03088048	10.01316297
Sc	1.74987966	1.01029349	11.62710277
H	1.74987966	1.01029349	13.63378918

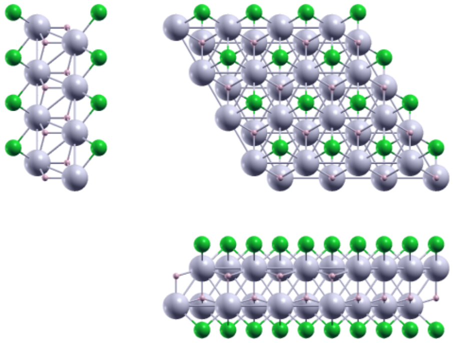

Orthographic projections: views of ScHCl (P-3m1) as seen from the x axis (left), the y axis (bottom) and the z axis (center).

Lattice matching - minimal size

Formula	N° atoms	strain	cell size 1	cell size 2
Tl	7	0.1301	1	1
Sn	7	0.1125	1	1
Na	7	0.0066	1	1
In	7	0.1143	1	1
In	7	0.2699	1	1
HgO	8	0.1395	1	1
AsSb	8	0.4477	1	1
GeTe	8	0.4626	1	1
S_2	8	0.4665	1	1
Mg_2	8	0.1199	1	1
Sb_2	8	3.0291	1	1
$IrTe_2$	9	0.4649	1	1
CrS_2	9	0.2495	1	1
S_2V	9	0.2746	1	1
MoS_2	9	0.2757	1	1
$CdCl_2$	9	0.4599	1	1
$MoTe_2$	9	0.0089	1	1
$AgTe_2$	9	0.1323	1	1
$ReSe_2$	9	0.0058	1	1
$InSe_2$	9	0.4609	1	1
$GeTe_2$	9	0.4575	1	1
$HfTe_2$	9	0.4862	1	1
I_2Mn	9	0.4602	1	1
NSr_2	9	0.4516	1	1
ReS_2	9	0.2575	1	1
$AuTe_2$	9	2.9246	1	1
$PdTe_2$	9	0.5003	1	1
FeI_2	9	0.4548	1	1
I_2Ni	9	0.4579	1	1
Mg_3	9	0.1261	1	1
CrI_2	9	0.4537	1	1
I_2Zn	9	2.9852	1	1
Te_2Zn	9	0.0087	1	1
S_2W	9	0.2758	1	1
Bi_2Pd	9	0.1479	1	1
Cl_2Ni	9	0.0049	1	1
$CrTe_2$	9	0.0011	1	1
PtS_2	9	0.0077	1	1
Br_2V	9	0.0066	1	1
ClNZr	9	0.0086	1	1
Ba_2N	9	2.8443	1	1
Se_2Ti	9	0.0049	1	1
Br_2Ti	9	0.0005	1	1
Te_2Zr	9	2.8312	1	1
Te_2W	9	0.0091	1	1
$AsSe_2$	9	0.0035	1	1
BrNZr	9	0.0022	1	1
$NbSe_2$	9	0.0045	1	1
CoI_2	9	0.4487	1	1
Br_2Cr	9	0.0008	1	1

Table showing the first 50 lattice matching combinations with the smallest number of atoms in the supercell.

Lattice matching - minimal strain

Formula	N° atoms	strain	cell size 1	cell size 2
Ni_2SbTe_2	911	0.0	91	73
O_2Pt	786	0.0	81	100
$InSe_2$	843	0.0001	100	81
S_2	692	0.0001	91	73
CrS_2	486	0.0002	49	64
H_2Si_2	924	0.0002	100	81
AsKSn	291	0.0002	36	25
Se_2Ta	9	0.0002	1	1
Br_2Cu	600	0.0002	72	56
I_2Y_2	742	0.0003	81	64
NaPSn	609	0.0003	73	57
O_2Zn	429	0.0003	43	57
$F_2Ho_2Se_2$	678	0.0003	64	49
$GeNi_3Te_2$	780	0.0003	73	57
$LiMnSe_2$	742	0.0004	81	64
I_2Mn	843	0.0004	100	81
$PtTe_2$	471	0.0004	57	43
Pt_2Te_2	666	0.0004	73	57
$F_2Se_2Tm_2$	870	0.0005	81	64
CoH_2O_2	707	0.0005	57	73
$CdCl_2$	843	0.0005	100	81
Ca_2Si	123	0.0005	16	9
$Ce_2I_2S_2$	150	0.0005	16	9
Br_2Ti	9	0.0005	1	1
O_4PSn	354	0.0005	39	20
N_3W_2	330	0.0005	25	36
Br_2Ho_2	514	0.0006	57	43
GeTe	762	0.0006	100	81
I_4Zr_2	972	0.0006	115	47
$AlLiTe_2$	214	0.0006	25	16
Ga_2Se_2	838	0.0006	91	73
CaI_2	477	0.0006	61	37
$F_2Lu_2Se_2$	510	0.0006	49	36
$Br_2Ho_2S_2$	438	0.0007	49	24
Ni_2Te_2	924	0.0007	100	81
I_2La_2	316	0.0007	36	25
Br_2PY_2	629	0.0007	64	49
$Br_2H_2Zr_2$	12	0.0007	1	1
$As_2Cd_2K_2$	354	0.0007	39	20
Cl_2Tb_2	742	0.0008	81	64
$AuTe_2$	471	0.0008	57	43
Sb_2Te_2	132	0.0008	16	9
$IrTe_2$	765	0.0008	91	73
P_2Sn_2	838	0.0008	91	73
Bi_2S_3	557	0.0008	57	43
Br_2Cr	9	0.0008	1	1
I_2Zn	402	0.0009	49	36
NS_2Ta	412	0.0009	36	49
$Dy_2I_2S_2$	780	0.0009	81	49
Br_2Cd	531	0.0009	64	49

Table showing the first 50 lattice matching combinations with the smallest strain needed to build the supercell.

ScOBr (Pmmn)

Structural and electronic properties

**Formula**	ScOBr
**Spacegroup**	Pmmn
**Prototype**	FeOCl
**Parent 3D**	$Sc_2O_2Br_2$
**Source DB**	COD
**DB ID**	2206575
**DF2-C09 Binding energy [meV/ Å^2]**	10.35
**RVV10 Binding energy [meV/ Å^2]**	17.28
**Band gap (PBE) [eV]**	3.23

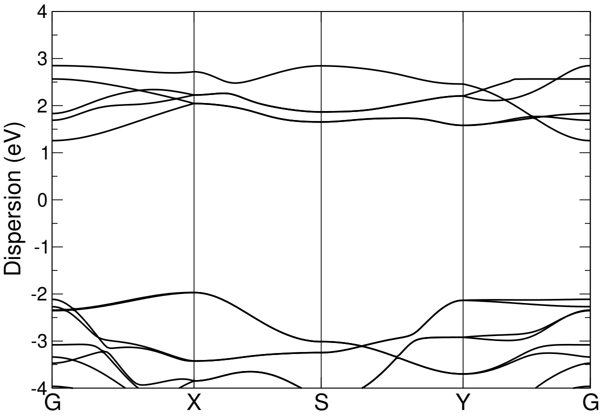

Band structure: Electronic band structure of ScOBr (Pmmn) in a representative window around the Fermi energy and along a high-symmetry path.

Optimized crystal structure

Structural parameters: relaxed cell (top) and atomic positions (bottom) of ScOBr (Pmmn) in Cartesian coordinates.

		x [Å]	y [Å]	z [Å]
$\mathbf{a_1}$		3.56997891	0.00000000	0.00000000
$\mathbf{a_2}$		0.00000000	3.98298107	0.00000000
$\mathbf{a_3}$		0.00000000	0.00000000	25.85414914
		x [Å]	y [Å]	z [Å]
	Sc	1.78498945	0.00000000	12.00289452
	Sc	0.00000000	1.99149053	13.85125793
	Br	0.00000000	0.00000000	10.01019459
	Br	1.78498945	1.99149053	15.84395430
	O	1.78498945	1.99149053	12.65007746
	O	0.00000000	0.00000000	13.20407800

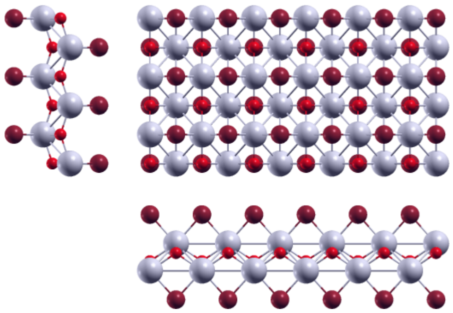

Orthographic projections: views of ScOBr (Pmmn) as seen from the x axis (left), the y axis (bottom) and the z axis (center).

Lattice matching - minimal size

Formula	N° atoms	strain	cell size 1	cell size 2
K	7	0.2108	1	1
InSe	8	0.6055	1	1
AgTl	8	0.3158	1	1
Ag_2	8	0.2188	1	1
PbTe	8	0.611	1	1
CaCl	8	0.0306	1	1
Sm	8	0.7387	1	2
CdI_2	9	0.6164	1	1
Ba_2Pt	9	0.2184	1	1
CaI_2	9	0.1981	1	1
Ca_2Si	9	0.2252	1	1
I_2Yb	9	0.1947	1	1
BiBrTe	9	0.6353	1	1
I_2Nd	9	0.0342	1	1
Cl_2Cu	9	0.4457	1	1
I_2Tm	9	0.1965	1	1
GeI_2	9	0.6117	1	1
STl_2	9	0.5965	1	1
GeS_2	9	0.2592	1	1
$MnSe_2$	9	0.0307	1	1
DyI_2	9	0.2019	1	1
GdI_2	9	0.6447	1	1
CNNa	9	0.4743	1	1
I_2La	9	0.0378	1	1
CdI_2	9	0.615	1	1
Cu_2I_2	10	0.3377	1	1
Cu_2Sr_2	10	0.6386	1	1
Cl_2OOs	10	0.022	1	1
$LiMnTe_2$	10	0.6127	1	1
Cu_2Te_2	10	0.0279	1	1
Ir_2P_2	10	0.034	1	1
Ag_2Br_2	10	0.0383	1	1
$AgCuTe_2$	10	0.2173	1	1
$AsLi_3$	10	0.6064	1	1
Pb_2Se_2	10	1.3305	1	1
O_2Sn_2	10	0.0102	1	1
S_2Sn_2	10	0.3416	1	1
Cl_2OV	10	0.2598	1	1
Ge_2Te_2	10	0.4613	1	1
Cl_2ORu	10	0.0244	1	1
As_2Ir_2	10	0.0403	1	1
O_2Pb_2	10	0.3314	1	1
$AgBrO_2$	10	0.1155	1	1
As_4	10	0.4824	1	1
P_4	10	0.4389	1	1
Fe_2S_2	10	0.0317	1	1
Sb_2Te_2	10	0.2232	1	1
Co_2S_2	10	0.0303	1	1
P_2	10	1.247	1	2
As_2Fe_2	10	0.2604	1	1

Table showing the first 50 lattice matching combinations with the smallest number of atoms in the supercell.

Lattice matching - minimal strain

Formula	N° atoms	strain	cell size 1	cell size 2
In_2Se_2	476	0.0004	56	35
Br_2Tb_2	634	0.0005	63	64
CrS_2	135	0.0005	12	21
$Cl_2Ga_2Te_2$	228	0.0005	24	14
$Er_2I_2S_2$	276	0.0005	28	18
Cl_2V	678	0.0005	63	100
Bi_2S_3	698	0.0005	63	64
Br_2Ho_2	634	0.0007	63	64
$F_2Lu_2Se_2$	762	0.0007	64	63
$Au_2K_2Se_2$	138	0.0007	18	5
CKN	504	0.0007	63	42
$PdTe_2$	570	0.0007	63	64
HN_3OZn	906	0.0008	60	91
CS_2Ta_2	815	0.001	60	91
In	114	0.001	15	24
$Ag_2K_2Te_2$	234	0.001	24	15
Cl_2Ti	633	0.001	60	91
$Br_2Ho_2S_2$	318	0.001	32	21
As_2Ir_2	96	0.001	10	9
$Ge_2Mn_2Sr_2$	114	0.0011	10	9
I_2Zn	573	0.0011	64	63
$AgTe_2$	327	0.0011	32	45
$CrSe_2$	678	0.0011	63	100
P_2	542	0.0011	60	91
HgI_2	366	0.0011	45	32
Se_2Sn_2	606	0.0012	67	51
Bi_2Se_2	138	0.0013	15	12
$Ag_2K_2Se_2$	462	0.0013	45	32
Fe_2S_2	94	0.0013	9	10
CrO_2	375	0.0014	30	65
GeI_2	870	0.0014	98	94
$Ba_2H_2I_2$	684	0.0014	68	46
Ga_2Te_2	964	0.0014	98	94
Se_2Yb	870	0.0014	98	94
LiO_2	468	0.0014	46	64
FHOZn	778	0.0014	63	100
$AsCuLi_2$	964	0.0014	98	94
$BiTe_2$	870	0.0015	98	94
$I_2Nd_2S_2$	192	0.0015	18	14
$Ce_2I_2Si_2$	738	0.0015	63	60
I_2Mg	558	0.0015	63	60
$Cl_2S_2Tl_2$	330	0.0015	35	20
Sb_2Se_2Te	678	0.0015	63	60
$CrSe_2$	678	0.0015	63	100
$GeNi_3Te_2$	738	0.0015	60	63
CNb_2S_2	815	0.0015	60	91
$PtTe_2$	570	0.0015	63	64
Br_2La	558	0.0015	63	60
Cl_2Cu	585	0.0016	62	71
Br_2Cd	570	0.0016	63	64

Table showing the first 50 lattice matching combinations with the smallest strain needed to build the supercell.

$Se_2Lu_2F_2$ (P-3m1)

Structural and electronic properties

**Formula**	$Se_2Lu_2F_2$
**Spacegroup**	P-3m1
**Prototype**	SmSI
**Parent 3D**	$F_2Lu_2Se_2$
**Source DB**	MPDS
**DB ID**	S307726
**DF2-C09 Binding energy [meV/ Å^2]**	12.6
**RVV10 Binding energy [meV/ Å^2]**	N/A
**Band gap (PBE) [eV]**	3.74

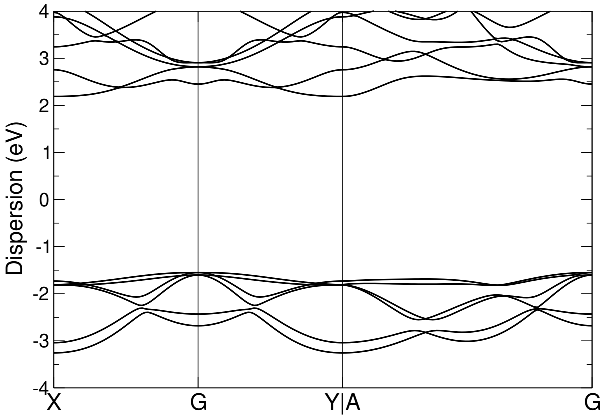

Band structure: Electronic band structure of $Se_2Lu_2F_2$ (P-3m1) in a representative window around the Fermi energy and along a high-symmetry path.

Optimized crystal structure

Structural parameters: relaxed cell (top) and atomic positions (bottom) of $Se_2Lu_2F_2$ (P-3m1) in Cartesian coordinates.

	x [Å]	y [Å]	z [Å]
$\mathbf{a_1}$	2.04423885	−3.54072556	0.00000000
$\mathbf{a_2}$	2.04422133	3.54071544	0.00000000
$\mathbf{a_3}$	0.00000000	0.00000000	23.94494317

		x [Å]	y [Å]	z [Å]
●	Lu	1.02208062	0.59009852	1.64367737
●	Se	1.02210374	-1.77037183	2.98174718
●	F	-1.02211703	-0.59011954	0.58744189
●	Lu	-1.02208062	-0.59009852	-1.64367737
●	Se	1.02213511	-1.77035372	-2.98174718
●	F	1.02211703	0.59011954	-0.58744189

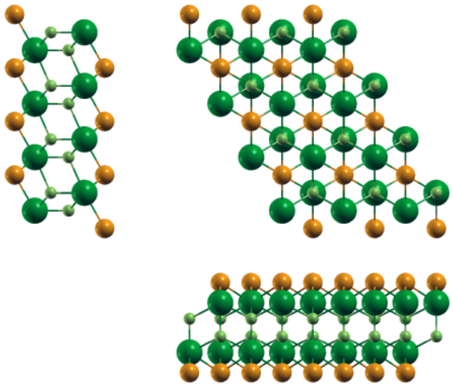

Orthographic projections: views of $Se_2Lu_2F_2$ (P-3m1) as seen from the x axis (left), the y axis (bottom) and the z axis (center).

Lattice matching - minimal size

Formula	N° atoms	strain	cell size 1	cell size 2
K	7	0.4698	1	1
Ag_2	8	0.4839	1	1
As_2	8	0.2547	1	1
Sb_2	8	0.0018	1	1
CaCl	8	0.1221	1	1
Cl_2Zn	9	0.2533	1	1
I_2Mg	9	0.0076	1	1
$MoTe_2$	9	0.2472	1	1
PSn_2	9	0.2672	1	1
Ba_2Pt	9	0.4832	1	1
HfS_2	9	0.2585	1	1
$AsSn_2$	9	0.2727	1	1
Te_2V	9	0.2497	1	1
I_2Pr	9	0.1523	1	1
$CuTe_2$	9	0.258	1	1
S_2Zr	9	0.2661	1	1
Br_2La	9	0.0079	1	1
Ca_2Si	9	0.4951	1	1
Br_2Co	9	0.2542	1	1
Ca_2N	9	0.2555	1	1
$AuTe_2$	9	0.006	1	1
Cl_2Zn	9	0.1316	1	1
$PdTe_2$	9	0.0084	1	1
I_2Zn	9	0.0015	1	1
Te_2Zn	9	0.247	1	1
GeI_2	9	0.0057	1	1
$CoTe_2$	9	0.2589	1	1
CdClO	9	0.2491	1	1
Te_2W	9	0.2474	1	1
I_2Nd	9	0.1532	1	1
S_2Sn	9	0.2665	1	1
$SnTe_2$	9	0.0034	1	1
$PtSe_2$	9	0.2736	1	1
OTl_2	9	0.2493	1	1
Br_2Fe	9	0.2543	1	1
GeS_2	9	0.1158	1	1
$TaTe_2$	9	0.2718	1	1
$MnSe_2$	9	0.122	1	1
DyI_2	9	0.4539	1	1
Br_2Ni	9	0.2612	1	1
CeI_2	9	0.1515	1	1
CuO_2	9	0.0933	1	1
$NbTe_2$	9	0.2658	1	1
Se_2Yb	9	0.0059	1	1
Cl_2Mg	9	0.2613	1	1
$BiTe_2$	9	0.0062	1	1
F_2Ni	9	0.1288	1	1
$PtTe_2$	9	0.0064	1	1
F_2Zn	9	0.1485	1	1
Bi_2Te_2	10	3.0353	1	1

Table showing the first 50 lattice matching combinations with the smallest number of atoms in the supercell.

Lattice matching - minimal strain

Formula	N° atoms	strain	cell size 1	cell size 2
Ba_2Pt	609	0.0	73	57
Ga_2S_3	543	0.0	43	57
Ca_2N	561	0.0	57	73
OTl_2	486	0.0	49	64
Cl_2Zr	258	0.0	25	36
$Cl_2Hf_2N_2$	678	0.0	49	64
NS_2Zr	486	0.0001	43	57
Br_2Ti	363	0.0001	36	49
$Br_2H_2Zr_2$	510	0.0001	36	49
$Ce_2I_2S_2$	678	0.0001	64	49
Ca_2Si	531	0.0001	64	49
Ga_2S_2	708	0.0001	64	81
CdClO	486	0.0002	49	64
CBr_2Lu_2	893	0.0002	73	91
Br_2Cr	363	0.0002	36	49
N_2Re	594	0.0002	49	100
CdO_2	258	0.0002	25	36
$Br_2N_2Zr_2$	984	0.0003	73	91
Ag_2	552	0.0003	73	57
$CoTe_2$	627	0.0003	64	81
S_2V	537	0.0003	49	81
PSn_2	786	0.0003	81	100
Te_2V	486	0.0003	49	64
ClH_3O	474	0.0003	49	36
S_2V	405	0.0003	37	61
Cl_2Fe	258	0.0004	25	36
Na	315	0.0004	43	57
Sn	498	0.0004	65	108
Cl_2Co	258	0.0004	25	36
MoS_2	405	0.0004	37	61
Cl_2Sc_2	412	0.0004	36	49
Gd	226	0.0004	27	64
S_2W	405	0.0004	37	61
CrS_2	102	0.0005	9	16
$CrTe_2$	363	0.0005	36	49
As_2	488	0.0005	57	73
Cu_2F_4	354	0.0006	39	20
HfS_2	627	0.0006	64	81
MoS_2	405	0.0006	37	61
H_2MgO_2	699	0.0006	49	81
$Br_2Hf_2N_2$	780	0.0006	57	73
$Cl_2H_2Sc_2$	510	0.0006	36	49
$Gd_2I_2Se_2$	708	0.0007	74	44
CCl_2Lu_2	707	0.0007	57	73
PtS_2	429	0.0007	43	57
$I_2N_2Zr_2$	894	0.0007	77	72
$ClKO_3$	195	0.0007	25	9
$Br_2O_2Sc_2$	762	0.0007	63	64
Se_4TiZr	786	0.0007	81	50
S_2Ti	258	0.0007	25	36

Table showing the first 50 lattice matching combinations with the smallest strain needed to build the supercell.

$Se_2Lu_2I_2$ (Pmmn)

Structural and electronic properties

**Formula**	$Se_2Lu_2I_2$
**Spacegroup**	Pmmn
**Prototype**	FeOCl
**Parent 3D**	$I_2Lu_2Se_2$
**Source DB**	MPDS
**DB ID**	S376107
**DF2-C09 Binding energy [meV/ Å^2]**	21.18
**RVV10 Binding energy [meV/ Å^2]**	N/A
**Band gap (PBE) [eV]**	2.68

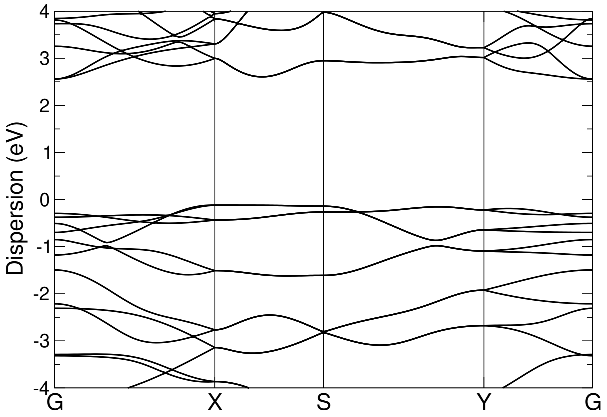

Band structure: Electronic band structure of $Se_2Lu_2I_2$ (Pmmn) in a representative window around the Fermi energy and along a high-symmetry path.

Optimized crystal structure

Structural parameters: relaxed cell (top) and atomic positions (bottom) of $Se_2Lu_2I_2$ (Pmmn) in Cartesian coordinates.

	x [Å]	y [Å]	z [Å]
$\mathbf{a_1}$	4.12473638	0.00000000	0.00000000
$\mathbf{a_2}$	0.00000000	6.09414306	0.00000000
$\mathbf{a_3}$	0.00000000	0.00000000	25.10679395

		x [Å]	y [Å]	z [Å]
●	Lu	1.03118410	-1.52353576	-1.67310672
●	Se	-1.03118410	-1.52353576	-3.23401872
●	I	1.03118410	-4.57060729	-0.88754879
●	Lu	-1.03118410	-4.57060729	1.67310672
●	Se	1.03118410	-4.57060729	3.23401872
●	I	-1.03118410	-1.52353576	0.88754879

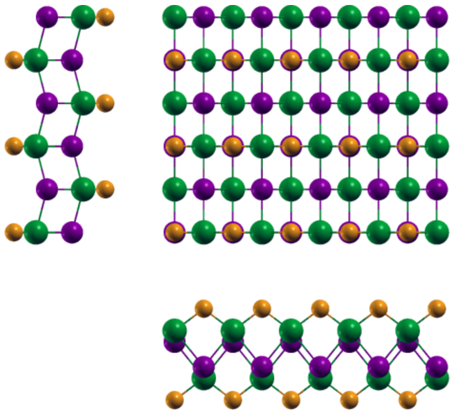

Orthographic projections: views of $Se_2Lu_2I_2$ (Pmmn) as seen from the x axis (left), the y axis (bottom) and the z axis (center).

Lattice matching - minimal size

Formula	N° atoms	strain	cell size 1	cell size 2
K	7	0.2773	1	1
Tl	8	0.2803	1	2
Na	8	0.1391	1	2
CKN	9	0.3628	1	1
S_2Sn_2	10	0.2604	1	1
As_2	10	0.1447	1	2
S_2	10	0.6097	1	2
Ge_2Se_2	10	0.2541	1	1
Hg_3N_2	11	0.3856	1	1
FKO_2Se	11	0.0298	1	1
IKO_3	11	0.4089	1	1
$BrKO_3$	11	0.3959	1	1
Cl_2Zn	12	0.1439	1	2
$Br_2Ho_2S_2$	12	0.3797	1	1
Cl_2Mn	12	0.289	1	2
$MoTe_2$	12	0.1406	1	2
PSn_2	12	0.1518	1	2
$MoSe_2$	12	0.2792	1	2
$Ho_2I_2S_2$	12	0.3883	1	1
Br_2Zn	12	0.1613	1	2
HfS_2	12	0.1468	1	2
$AsSn_2$	12	0.155	1	2
Te_2V	12	0.142	1	2
$CuTe_2$	12	0.1465	1	2
S_2Zr	12	0.1511	1	2
Br_2Co	12	0.1444	1	2
$Cl_2Rh_2Te_2$	12	0.1594	1	1
Ca_2N	12	0.1451	1	2
S_2Ti	12	0.1284	1	2
Te_2Ti	12	0.1616	1	2
$Br_2S_2Y_2$	12	0.3851	1	1
Te_2Zn	12	0.0425	1	2
I_4Zr_2	12	0.3176	1	1
$RhTe_2$	12	0.1575	1	2
$Gd_2I_2S_2$	12	0.3957	1	1
Br_2Mn	12	0.1431	1	2
Cl_2Co	12	0.1282	1	2
$CrTe_2$	12	0.1356	1	2
$Br_2Er_2Se_2$	12	0.0267	1	1
PtS_2	12	0.1398	1	2
NbS_2	12	0.2849	1	2
$CoTe_2$	12	0.1471	1	2
ClNZr	12	0.13	1	2
Cl_2Fe	12	0.1279	1	2
CdClO	12	0.1416	1	2
S_2Ta	12	0.2836	1	2
Se_2V	12	0.2815	1	2
$I_2S_2Tb_2$	12	0.3925	1	1
Se_2Ti	12	0.0442	1	2
$I_2S_2Yb_2$	12	0.0405	1	1

Table showing the first 50 lattice matching combinations with the smallest number of atoms in the supercell.

Lattice matching - minimal strain

Formula	N° atoms	strain	cell size 1	cell size 2
$Br_2Cr_2O_2$	360	0.0002	20	40
LiO_2	537	0.0006	40	99
Cu_2O_2	228	0.0006	18	30
$Fe_2Li_2P_2$	414	0.0007	24	45
$C_2Cl_2Y_2$	708	0.0007	40	78
$Br_2O_2V_2$	918	0.0008	51	102
$Cl_2Hg_2N_2$	390	0.0009	35	30
Ho_2S_2	456	0.0011	36	60
Cl_4Mn	973	0.0011	83	95
$Cu_2K_2Te_2$	396	0.0012	30	36
$SnTe_2$	78	0.0012	7	12
$Hf_2Si_2Te_2$	414	0.0012	24	45
$Cl_2Cr_2O_2$	282	0.0012	15	32
NS_2Ta	452	0.0013	24	77
O_2Sn_2	494	0.0014	35	71
Br_2Zr_2	92	0.0014	6	14
CrO_2	246	0.0015	14	54
$I_2S_2Tb_2$	726	0.0015	50	71
$H_2Li_2O_2$	804	0.0016	45	89
Cu_2F_4	714	0.0016	63	56
DyI_2	513	0.0016	50	71
GdI_2	486	0.0017	46	70
Se_2Ti	78	0.0017	6	14
AlH_4Na	714	0.0017	56	63
Co_2Se_2	324	0.0018	24	45
CNRb	633	0.0018	68	75
N_3Na	640	0.0018	58	73
$BrKO_3$	760	0.0018	85	50
As_4	992	0.0018	84	122
$AlLiTe_2$	556	0.0019	46	70
N_2W	513	0.0019	33	105
$BrKO_3$	670	0.0019	75	44
O_2Sn_2	204	0.002	16	27
Ge_2S_2	492	0.002	40	63
$Dy_2I_2S_2$	726	0.002	50	71
Fe_2Se_2	324	0.002	24	45
$I_2S_2Yb_2$	90	0.002	7	8
CoH_2O_2	929	0.0021	44	133
C_4Ca_2	774	0.0021	52	77
$Ba_2H_2I_2$	396	0.0021	30	36
CNRb	594	0.0021	64	70
Ca_2Si	699	0.0022	70	93
As_4	738	0.0022	63	90
$BrKO_3$	653	0.0022	73	43
N_2W	375	0.0022	24	77
$BrKO_3$	563	0.0022	63	37
$Br_2Dy_2S_2$	90	0.0023	7	8
Ho_2S_2	252	0.0023	20	33
$Gd_2I_2S_2$	726	0.0023	50	71
$BrKO_3$	107	0.0023	12	7

Table showing the first 50 lattice matching combinations with the smallest strain needed to build the supercell.

SiH (P-3m1)

Structural and electronic properties

**Formula**	SiH
**Spacegroup**	P-3m1
**Prototype**	SiH
**Parent 3D**	Si_2H_2
**Source DB**	ICSD
**DB ID**	41478
**DF2-C09 Binding energy [meV/ Å^2]**	8.98
**RVV10 Binding energy [meV/ Å^2]**	13.01
**Band gap (PBE) [eV]**	2.19

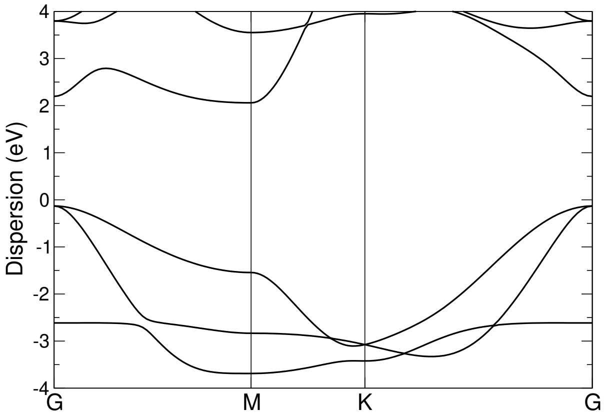

Band structure: Electronic band structure of SiH (P-3m1) in a representative window around the Fermi energy and along a high-symmetry path.

Optimized crystal structure

Structural parameters: relaxed cell (top) and atomic positions (bottom) of SiH (P-3m1) in Cartesian coordinates.

		x [Å]	y [Å]	z [Å]
$\mathbf{a_1}$		3.89019560	0.00000000	0.00000000
$\mathbf{a_2}$		−1.94509780	3.36900822	0.00000000
$\mathbf{a_3}$		0.00000000	0.00000000	23.71354871
		x [Å]	y [Å]	z [Å]
●	Si	1.94509780	1.12300274	11.49746780
●	Si	0.00000000	2.24600548	12.21608091
·	H	1.94509780	1.12300274	9.99580121
·	H	0.00000000	2.24600548	13.71774750

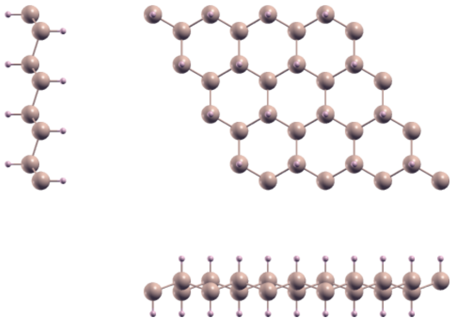

Orthographic projections: views of SiH (P-3m1) as seen from the x axis (left), the y axis (bottom) and the z axis (center).

Lattice matching - minimal size

Formula	N° atoms	strain	cell size 1	cell size 2
Tl	5	1.5333	1	1
HgO	6	0.4427	1	1
AsSb	6	0.0056	1	1
Bi_2	6	0.4544	1	1
GeTe	6	0.0004	1	1
S_2	6	0.0019	1	1
CaCl	6	0.1387	1	1
$IrTe_2$	7	0.0013	1	1
$CdCl_2$	7	0.0007	1	1
CdI_2	7	0.4488	1	1
$AgTe_2$	7	0.4222	1	1
$MoSe_2$	7	1.528	1	1
$ReSe_2$	7	0.259	1	1
S_2Ta	7	1.59	1	1
Br_2Ca	7	0.4517	1	1
CaI_2	7	2.9265	1	1
$InSe_2$	7	0.0003	1	1
$GeTe_2$	7	0.0017	1	1
I_2Mn	7	0.0006	1	1
NSr_2	7	0.004	1	1
I_2Yb	7	0.5005	1	1
PbS_2	7	0.0081	1	1
BiClTe	7	0.4496	1	1
LiO_2	7	0.0673	1	1
Cl_2Zn	7	0.1517	1	1
FeI_2	7	0.0028	1	1
I_2Ni	7	0.0015	1	1
S_2Ti	7	1.627	1	1
NbS_2	7	1.5866	1	1
CrI_2	7	0.0031	1	1
BiBrTe	7	0.4633	1	1
Bi_2Pd	7	0.1142	1	1
N_2W	7	4.8545	1	1
Cl_2Ni	7	0.2603	1	1
Cl_2Co	7	0.2503	1	1
$CrTe_2$	7	0.2689	1	1
Br_2V	7	0.2579	1	1
ClNZr	7	0.255	1	1
Cl_2Fe	7	0.2494	1	1
S_2Ta	7	1.549	1	1
Se_2V	7	1.5394	1	1
Se_2Ti	7	0.2745	1	1
Br_2Ti	7	0.2681	1	1
$AsSe_2$	7	0.2623	1	1
I_2Tm	7	2.9112	1	1
BiITe	7	0.4799	1	1
CdO_2	7	0.2501	1	1
BrNZr	7	0.2642	1	1
$NbSe_2$	7	0.2608	1	1
CoI_2	7	0.0052	1	1

Table showing the first 50 lattice matching combinations with the smallest number of atoms in the supercell.

Lattice matching - minimal strain

Formula	N° atoms	strain	cell size 1	cell size 2
Cl_2Fe	388	0.0001	49	64
$Gd_2I_2S_2$	412	0.0001	49	36
BrNZr	565	0.0001	73	91
CNNa	488	0.0002	77	60
Br_2Ca_3Si	412	0.0002	49	36
$Cl_2H_2Sc_2$	924	0.0002	81	100
$ReSe_2$	499	0.0002	64	81
$Br_2H_2Zr_2$	666	0.0002	57	73
FeH_2O_2	601	0.0002	49	81
I_2Tm	357	0.0002	57	43
$LiNbS_2$	400	0.0002	43	57
S_2Ta	291	0.0003	36	49
Ca_2Si	219	0.0003	36	25
$InSe_2$	7	0.0003	1	1
S_2Ta	343	0.0003	43	57
$Ce_2I_2S_2$	294	0.0003	36	25
ClNZr	447	0.0003	57	73
Br_2Ti	624	0.0004	81	100
GeTe	6	0.0004	1	1
$AlLiTe_2$	580	0.0004	81	64
FeH_2O_2	453	0.0004	37	61
Cu_2Sr_2	656	0.0004	91	73
Se_2Ta	624	0.0004	81	100
Cu_4Te_2	886	0.0004	100	81
$Cl_2Er_2H_2$	10	0.0005	1	1
Cl_2Zr	388	0.0005	49	64
GeI_2La_2	443	0.0005	57	43
I_2La_2P	729	0.0005	91	73
Br_2Hf_2	656	0.0005	73	91
$Br_2H_2Zr_2$	924	0.0005	81	100
$Br_2Cr_2O_2$	748	0.0006	73	76
I_2Mn	7	0.0006	1	1
In_2S_3	9	0.0006	1	1
CCl_2Sc_2	593	0.0006	57	73
$F_2I_2Pb_2$	548	0.0006	65	48
Br_2Cr	624	0.0006	81	100
CdO_2	388	0.0007	49	64
$CdCl_2$	7	0.0007	1	1
Cl_2Ni	499	0.0007	64	81
$I_2N_2Ti_2$	924	0.0007	96	90
N_3W_2	116	0.0007	9	16
Ba_2Cd	404	0.0008	65	48
NbS_2	343	0.0008	43	57
Cl_2Co	388	0.0008	49	64
Cl_2Sc_2	724	0.0009	81	100
BiBrTe	643	0.0009	100	81
Cl_2Hf_2	400	0.0009	43	57
Ni_2Te_2	8	0.0009	1	1
CaI_2	357	0.0009	57	43
$CrTe_2$	624	0.0009	81	100

Table showing the first 50 lattice matching combinations with the smallest strain needed to build the supercell.

$SiTe_2$ (P-3m1)

Structural and electronic properties

**Formula**	$SiTe_2$
**Spacegroup**	P-3m1
**Prototype**	CdI2
**Parent 3D**	$SiTe_2$
**Source DB**	COD
**DB ID**	9009119
**DF2-C09 Binding energy [meV/ Å^2]**	19.95
**RVV10 Binding energy [meV/ Å^2]**	25.6
**Band gap (PBE) [eV]**	N/A

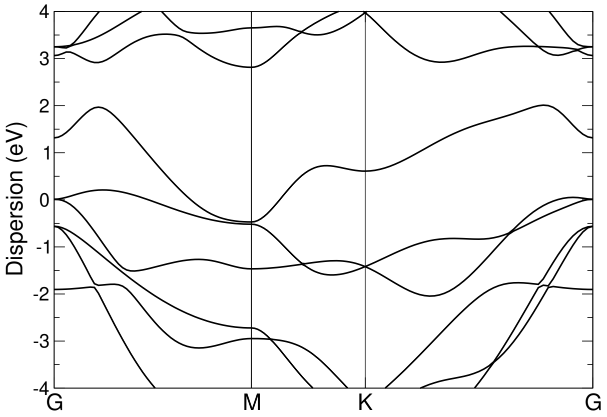

Band structure: Electronic band structure of $SiTe_2$ (P-3m1) in a representative window around the Fermi energy and along a high-symmetry path.

Optimized crystal structure

Structural parameters: relaxed cell (top) and atomic positions (bottom) of $SiTe_2$ (P-3m1) in Cartesian coordinates.

		x [Å]	y [Å]	z [Å]
$\mathbf{a_1}$		3.79440644	0.00000000	0.00000000
$\mathbf{a_2}$		−1.89720322	3.28605237	0.00000000
$\mathbf{a_3}$		0.00000000	0.00000000	23.33058136
		x [Å]	y [Å]	z [Å]
●	Te	1.89720322	1.09535079	13.29329128
●	Si	0.00000000	0.00000000	11.66529068
●	Te	-0.00000000	2.19070158	10.03729008

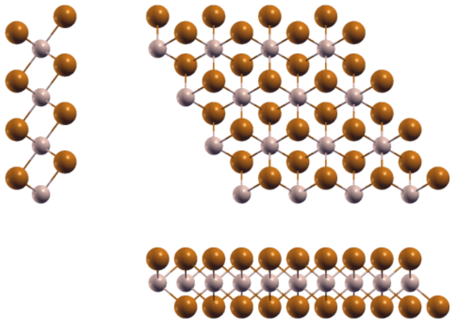

Orthographic projections: views of $SiTe_2$ (P-3m1) as seen from the x axis (left), the y axis (bottom) and the z axis (center).

Lattice matching - minimal size

Formula	N° atoms	strain	cell size 1	cell size 2
Tl	4	0.4421	1	1
Tl	4	0.2494	1	1
InSe	5	0.4693	1	1
HgO	5	0.1151	1	1
AsSb	5	0.0062	1	1
Bi_2	5	0.4842	1	1
P_2	5	1.5804	1	1
PbTe	5	0.4738	1	1
CaCl	5	0.1491	1	1
Cl_2Mn	6	0.2572	1	1
CdI_2	6	0.4782	1	1
$AgTe_2$	6	0.1115	1	1
$MoSe_2$	6	0.2484	1	1
$ReSe_2$	6	0.2759	1	1
S_2Ta	6	0.26	1	1
Br_2Zn	6	0.0041	1	1
Br_2Ca	6	0.4814	1	1
NSr_2	6	0.0078	1	1
PbS_2	6	0.0035	1	1
BiClTe	6	0.4792	1	1
Cl_2Ti	6	1.5815	1	1
BrCdI	6	0.4515	1	1
FeI_2	6	0.0092	1	1
S_2Ti	6	0.2671	1	1
Mg_3	6	0.4286	1	1
Te_2Ti	6	0.0038	1	1
NbS_2	6	0.2594	1	1
CrI_2	6	0.0088	1	1
BaF_2	6	0.4597	1	1
BiBrTe	6	0.4936	1	1
$RhTe_2$	6	0.008	1	1
Bi_2Pd	6	0.1198	1	1
Cl_2Co	6	0.2666	1	1
NbS_2	6	0.2535	1	1
Br_2V	6	0.2747	1	1
ClNZr	6	0.2716	1	1
Cl_2Fe	6	0.2655	1	1
S_2Ta	6	1.6359	1	1
Se_2V	6	0.2506	1	1
AsKSn	6	0.4561	1	1
$PbTe_2$	6	0.4491	1	1
$NiTe_2$	6	0.0006	1	1
I_2V	6	0.0013	1	1
GeI_2	6	0.4744	1	1
Se_2Zr	6	0.0004	1	1
STl_2	6	0.4621	1	1
CdO_2	6	0.2664	1	1
CoI_2	6	0.0066	1	1
GeS_2	6	0.1384	1	1
$MnSe_2$	6	0.149	1	1

Table showing the first 50 lattice matching combinations with the smallest number of atoms in the supercell.

Lattice matching - minimal strain

Formula	N° atoms	strain	cell size 1	cell size 2
Bi_2STe_2	563	0.0	81	64
I_2La_2Te	233	0.0001	36	25
$Ga_2Gd_2I_2$	561	0.0001	73	57
GeI_2Y_2	638	0.0001	91	73
NbS_2	435	0.0001	64	81
Tl	211	0.0001	49	64
Br_2Ca_3Si	258	0.0002	36	25
$Br_2F_2Sr_2$	483	0.0002	65	48
$Cl_2Er_2O_2$	9	0.0002	1	1
In	111	0.0002	25	36
$Br_2In_2O_2$	963	0.0003	121	100
$Br_2F_2Pb_2$	483	0.0003	65	48
$LiOS_2Ti$	536	0.0003	57	73
As_2Li_2Nd	638	0.0003	91	73
I_4Sr_2	123	0.0003	25	8
$LiMnTe_2$	499	0.0003	81	64
Cu_2Sr_2	388	0.0003	64	49
$Ga_2I_2Tb_2$	561	0.0003	73	57
STl_2	543	0.0004	100	81
S_2Ti	543	0.0004	81	100
Bi_2	333	0.0004	73	57
Bi_2Se_2Te	638	0.0004	91	73
$ClKO_3$	368	0.0004	81	25
Se_2Zr	6	0.0004	1	1
CdI_2	435	0.0004	81	64
$Cl_2Hg_2N_2$	573	0.0004	103	44
Se_2W	339	0.0004	49	64
HN_3OZn	471	0.0005	43	57
S_2Ta	435	0.0005	64	81
Br_2La_2P	563	0.0006	81	64
Nd	124	0.0006	25	49
BaF_2	543	0.0006	100	81
$LiNbS_2$	516	0.0006	64	81
$NiTe_2$	6	0.0006	1	1
$MoSe_2$	339	0.0006	49	64
GeI_2	435	0.0006	81	64
BiBrTe	339	0.0007	64	49
Br_2Ca	390	0.0007	73	57
N_4	663	0.0007	77	108
Br_2Hg_3	237	0.0007	64	9
Cl_2Co	543	0.0007	81	100
$Br_2N_2Ti_2$	414	0.0007	48	45
As_2O_3	545	0.0007	100	49
Li_2Tl_2	84	0.0007	16	9
Sb_2SeTe_2	638	0.0008	91	73
$CuGeO_3$	375	0.0008	55	42
$Ga_2I_2Y_2$	786	0.0008	100	81
$Ge_2I_2La_2$	363	0.0008	49	36
As_2CeLi_2	563	0.0008	81	64
In_2Te_3	638	0.0008	91	73

Table showing the first 50 lattice matching combinations with the smallest strain needed to build the supercell.

Sm (P6/mmm)

Structural and electronic properties

**Formula**	Sm
**Spacegroup**	P6/mmm
**Prototype**	In
**Parent 3D**	Sm_2
**Source DB**	COD
**DB ID**	9010999
**DF2-C09 Binding energy [meV/ Å^2]**	0.23
**RVV10 Binding energy [meV/ Å^2]**	N/A
**Band gap (PBE) [eV]**	0.0

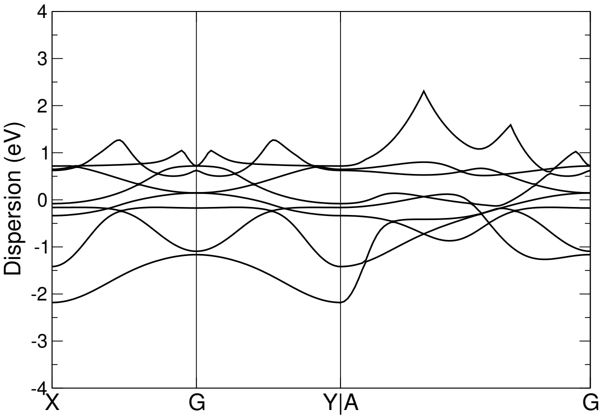

Band structure: Electronic band structure of Sm (P6/mmm) in a representative window around the Fermi energy and along a high-symmetry path.

Optimized crystal structure

Structural parameters: relaxed cell (top) and atomic positions (bottom) of Sm (P6/mmm) in Cartesian coordinates.

	x [Å]	y [Å]	z [Å]
$\mathbf{a_1}$	2.61394406	0.00006571	0.00000000
$\mathbf{a_2}$	−1.30691512	2.26364340	0.00000000
$\mathbf{a_3}$	0.00000000	0.00000000	12.00000000

		x [Å]	y [Å]	z [Å]
●	Sm	-0.65345756	1.13182170	0.00000000

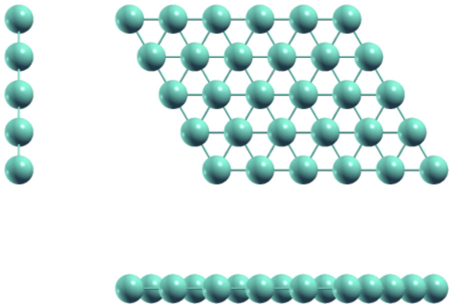

Orthographic projections: views of Sm (P6/mmm) as seen from the x axis (left), the y axis (bottom) and the z axis (center).

Lattice matching - minimal size

Formula	N° atoms	strain	cell size 1	cell size 2
Tl	2	0.3215	1	1
CrS_2	4	3.0251	1	1
Cl_2Mn	4	13.6117	1	1
$AgTe_2$	4	0.3277	1	1
N_2W	4	2.9346	1	1
PSn_2	5	0.2191	2	1
$AsSn_2$	5	0.2239	2	1
Bi_2Mn_2	5	2.301	1	1
HgI_2	5	0.5373	2	1
Mg_4	5	0.3208	1	1
Sn	5	0.9144	2	3
C_2F_2	5	0.0015	1	1
$PtSe_2$	5	0.2248	2	1
$TaTe_2$	5	0.2232	2	1
Bi_2In_2	6	0.5865	2	1
CdClHO	6	0.2225	2	1
$AgCuTe_2$	6	0.5277	2	1
Br_2Cu	6	0.069	3	1
Cl_2NSc_2	6	13.6393	1	1
Ge_2Te_2	6	0.4611	2	1
P_4	6	0.2577	2	1
H_2MnO_2	6	0.4707	1	1
Au_2I_2	6	0.4841	2	1
N_3W_2	6	0.4647	1	1
La_2S_2	6	0.467	2	1
Ag_2I_2	6	0.5403	2	1
Br_2CsF	6	0.5802	2	1
Sn_2Te_2	6	0.558	2	1
CaI_2	7	6.2327	1	2
I_2Yb	7	6.1645	1	2
Tl	7	0.2351	4	3
Mg_3	7	0.1391	4	1
I_2Tm	7	6.201	1	2
Sn	7	0.0989	4	3
Na	7	0.2432	4	3
In	7	0.1027	4	3
Mg_6	7	0.3337	1	1
Bi_2In_2	8	0.124	4	1
HgO	8	1.0393	2	3
$Br_2Cr_2S_2$	8	0.2616	2	1
$Br_2O_2Sc_2$	8	1.1278	2	1
Ge_2Te_2	8	0.11	4	1
$C_2Br_2Gd_2$	8	0.2524	2	1
N_2W	8	0.9069	2	2
$Hf_2I_2N_2$	8	0.2225	2	1
$Cu_2Rb_2Te_2$	8	0.5854	2	1
P_4	8	0.1174	4	1
O_4PSn	8	0.5587	2	1
$Ba_2F_2I_2$	8	0.551	2	1
O_4PTl	8	0.549	2	1

Table showing the first 50 lattice matching combinations with the smallest number of atoms in the supercell.

Lattice matching - minimal strain

Formula	N° atoms	strain	cell size 1	cell size 2
GdI_2	52	0.0	25	9
$Br_2N_2Zr_2$	199	0.0001	49	25
CBr_2Lu_2	174	0.0001	49	25
BrCdI	139	0.0001	64	25
Cl_2Mn	228	0.0001	81	49
K	65	0.0002	49	16
SSb_2Te_2	189	0.0002	64	25
$I_2S_2Sm_2$	145	0.0002	49	16
BrNZr	43	0.0002	16	9
O_2Sn_2	299	0.0003	115	46
CdClHO	235	0.0004	79	39
S_2Zn_2	25	0.0004	9	4
$Ca_2Cl_2F_2$	454	0.0004	130	54
Cl_2Gd_2	25	0.0005	9	4
$I_2N_2Zr_2$	394	0.0005	100	49
$MnNaTe_2$	164	0.0005	64	25
I_2La_2Sb	129	0.0005	49	16
Cl_2NSc_2	326	0.0006	81	49
$RhTe_2$	247	0.0006	100	49
NS_2Ta	229	0.0007	57	43
Br_2Hf_2	52	0.0007	16	9
$F_2Se_2Yb_2$	33	0.0007	9	4
$LiOS_2Ti$	246	0.0008	61	37
Cl_2Mn	172	0.0008	61	37
P_2Sn_2	25	0.0008	9	4
Hg_3N_2	109	0.0008	64	9
N_3W_2	456	0.0009	91	73
$Cl_2N_2Ti_2$	131	0.0009	35	16
I_2La_2P	70	0.0009	25	9
$Ge_2Te_2Zr_2$	403	0.001	115	48
$Br_2Lu_2O_2$	403	0.001	115	48
O_2Sn_2	287	0.001	99	47
H_4Ti	521	0.001	126	79
$PtSe_2$	196	0.001	79	39
$PbTe_2$	139	0.0011	64	25
$RhTe_2$	196	0.0011	79	39
$AgClO_2$	209	0.0011	77	33
H_2Li_2Pd	521	0.0012	126	79
Cl_2Tb_2	25	0.0012	9	4
O_2Zn	157	0.0012	49	36
Cl_2NSc_2	246	0.0012	61	37
O_2Sn_2	287	0.0012	99	47
I_2La_2	164	0.0012	64	25
$NbSe_2$	43	0.0013	16	9
Br_2OV	92	0.0013	32	15
$NbTe_2$	124	0.0013	49	25
$Eu_2F_2I_2$	387	0.0014	123	44
Fe_2SeTe	307	0.0014	115	48
$Br_2La_2O_2$	387	0.0014	123	44
O_2Pb_2	299	0.0014	123	44

Table showing the first 50 lattice matching combinations with the smallest strain needed to build the supercell.

$Sm_2I_2F_2$ (P4/nmm)

Structural and electronic properties

**Formula**	$Sm_2I_2F_2$
**Spacegroup**	P4/nmm
**Prototype**	PbClF
**Parent 3D**	$F_2I_2Sm_2$
**Source DB**	MPDS
**DB ID**	S1800103
**DF2-C09 Binding energy [meV/ Å^2]**	17.4
**RVV10 Binding energy [meV/ Å^2]**	N/A
**Band gap (PBE) [eV]**	0.0

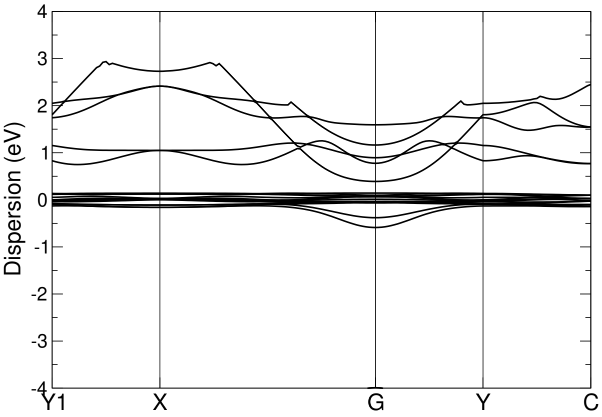

Band structure: Electronic band structure of $Sm_2I_2F_2$ (P4/nmm) in a representative window around the Fermi energy and along a high-symmetry path.

Optimized crystal structure

Structural parameters: relaxed cell (top) and atomic positions (bottom) of $Sm_2I_2F_2$ (P4/nmm) in Cartesian coordinates.

	x [Å]	y [Å]	z [Å]
$\mathbf{a_1}$	4.06181109	−0.00006562	0.00000000
$\mathbf{a_2}$	−0.00006562	4.06181109	0.00000000
$\mathbf{a_3}$	0.00000000	0.00000000	23.92636559

		x [Å]	y [Å]	z [Å]
	Sm	1.01551676	-1.01551676	1.48211149
	I	-1.01529564	-3.04644983	3.08438032
	Sm	-1.01545114	-3.04629433	-1.48211149
	I	1.01536126	-1.01536126	-3.08438032
	F	-1.01541452	-1.01541452	0.00000000
	F	1.01548014	-3.04639657	0.00000000

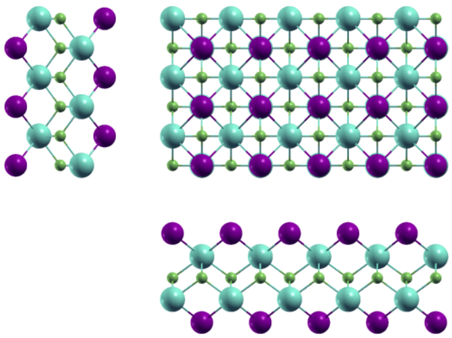

Orthographic projections: views of $Sm_2I_2F_2$ (P4/nmm) as seen from the x axis (left), the y axis (bottom) and the z axis (center).

Lattice matching - minimal size

Formula	N° atoms	strain	cell size 1	cell size 2
K	7	0.1625	1	1
Tl	7	0.111	1	1
InSe	8	0.1305	1	1
AsSb	8	0.1084	1	1
Bi_2	8	0.1349	1	1
Ag_2	8	0.1685	1	1
PbTe	8	0.1318	1	1
CaCl	8	0.2112	1	1
Cl_2Mn	9	0.1097	1	1
CdI_2	9	0.1331	1	1
Nd	9	0.1755	1	3
$MoSe_2$	9	0.1111	1	1
Ba_2Pt	9	0.1682	1	1
S_2Ta	9	0.1092	1	1
Br_2Ca	9	0.134	1	1
CaI_2	9	0.153	1	1
Br_2Cu	9	0.0991	1	1
NSr_2	9	0.1088	1	1
Ca_2Si	9	0.1734	1	1
I_2Yb	9	0.1505	1	1
BiClTe	9	0.1334	1	1
FeI_2	9	0.1091	1	1
NbS_2	9	0.1093	1	1
CrI_2	9	0.1089	1	1
BiBrTe	9	0.1377	1	1
NbS_2	9	0.1103	1	1
S_2Ta	9	0.1105	1	1
CKN	9	0.4761	1	1
Se_2V	9	0.1108	1	1
I_2Tm	9	0.1518	1	1
GeI_2	9	0.132	1	1
I_2Pb	9	0.1703	1	1
BiITe	9	0.1433	1	1
CoI_2	9	0.1085	1	1
$MnSe_2$	9	0.2111	1	1
DyI_2	9	0.1558	1	1
Br_2Mg	9	0.109	1	1
GdI_2	9	0.1401	1	1
CNNa	9	0.0696	1	1
CdI_2	9	0.1328	1	1
Se_2Sn	9	0.1087	1	1
I_2Pr	9	0.1335	1	1
Se_2W	9	0.1111	1	1
Bi_2Te_2	10	0.1891	1	1
Bi_2In_2	10	1.1056	1	1
Cu_2I_2	10	0.0035	1	1
Cu_2Sr_2	10	0.1386	1	1
Cl_2OOs	10	0.2173	1	1
$LiMnTe_2$	10	0.1322	1	1
$AsLi_3$	10	0.1308	1	1

Table showing the first 50 lattice matching combinations with the smallest number of atoms in the supercell.

Lattice matching - minimal strain

Formula	N° atoms	strain	cell size 1	cell size 2
$Cu_2Rb_2Te_2$	690	0.0	65	50
Se_2Ta_4	366	0.0001	25	36
O_2Pb_2	10	0.0002	1	1
Cl_2Y_2	548	0.0002	48	65
$Cu_2K_2Te_2$	870	0.0002	81	64
Mg_4	618	0.0003	49	81
Bi_2In_2	590	0.0003	65	50
$AgTe_2$	708	0.0004	65	106
Cl_4KTl	738	0.0004	89	34
$H_2Li_2O_2$	690	0.0006	50	65
HgO	146	0.0006	16	25
Tl	375	0.0006	49	81
$Se_2Si_2Zr_2$	678	0.0006	49	64
Ga_2Se_2	548	0.0006	48	65
CBr_2Y_2	613	0.0007	48	65
Co_2S_2	708	0.0007	64	81
Br_2OV	540	0.0008	48	63
Bi_2In_2	580	0.0008	64	49
Ca_2N	840	0.0008	81	118
Co_2Se_2	886	0.0009	81	100
$Eu_2F_2I_2$	12	0.0009	1	1
$Br_2La_2O_2$	12	0.001	1	1
CoO_2	633	0.001	48	115
As_2Fe_2	962	0.001	85	113
$ReSe_2$	852	0.001	79	126
$Cu_2Rb_2Te_2$	678	0.0011	64	49
$I_2O_2Pr_2$	12	0.0011	1	1
As_2	722	0.0012	81	118
$Al_2Cl_2O_2$	972	0.0012	67	95
NiO_2	633	0.0012	48	115
CCl_2Gd_2	613	0.0012	48	65
Br_2V	852	0.0013	79	126
$C_2Br_2Tb_2$	948	0.0013	72	86
O_4PSn	882	0.0013	82	65
Br_2Zn	483	0.0014	48	65
CaCl	546	0.0014	64	81
Cl_2Ni	852	0.0014	79	126
C_4Ca_2	804	0.0014	68	66
NbS_2	711	0.0014	64	109
$MnSe_2$	627	0.0015	64	81
Sm	387	0.0015	44	123
Mg_2	86	0.0015	9	16
Br_2Fe	840	0.0015	81	118
Bi_2O_2	10	0.0015	1	1
Br_2Co	840	0.0015	81	118
S_2Zn_2	680	0.0016	62	77
P_2Sn_2	680	0.0016	62	77
$I_2N_2Zr_2$	678	0.0016	48	65
Cl_4Mg_2	762	0.0016	93	34
Te_2Ti	483	0.0017	48	65

Table showing the first 50 lattice matching combinations with the smallest strain needed to build the supercell.

$Sm_2O_2I_2$ (P4/nmm)

Structural and electronic properties

**Formula**	$Sm_2O_2I_2$
**Spacegroup**	P4/nmm
**Prototype**	PbClF
**Parent 3D**	$I_2O_2Sm_2$
**Source DB**	MPDS
**DB ID**	S1401116
**DF2-C09 Binding energy [meV/ Å^2]**	14.96
**RVV10 Binding energy [meV/ Å^2]**	N/A
**Band gap (PBE) [eV]**	0.0

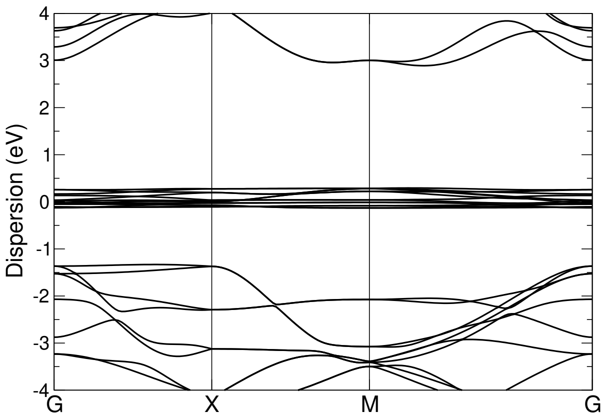

Band structure: Electronic band structure of $Sm_2O_2I_2$ (P4/nmm) in a representative window around the Fermi energy and along a high-symmetry path.

Optimized crystal structure

Structural parameters: relaxed cell (top) and atomic positions (bottom) of $Sm_2O_2I_2$ (P4/nmm) in Cartesian coordinates.

	x [Å]	y [Å]	z [Å]
$\mathbf{a_1}$	4.00623744	0.00000000	0.00000000
$\mathbf{a_2}$	0.00000000	4.00623744	0.00000000
$\mathbf{a_3}$	0.00000000	0.00000000	23.87903425

		x [Å]	y [Å]	z [Å]
●	Sm	1.00155936	-1.00155936	1.12854126
●	I	-1.00155936	-3.00467808	3.03073077
●	Sm	-1.00155936	-3.00467808	-1.12854126
●	I	1.00155936	-1.00155936	-3.03073077
●	O	-1.00155936	-1.00155936	0.00000000
●	O	1.00155936	-3.00467808	0.00000000

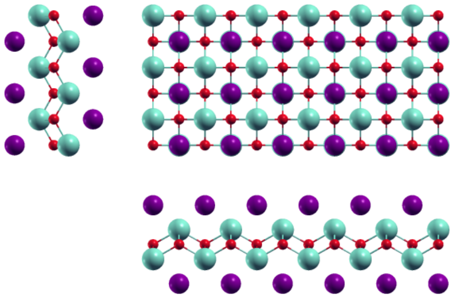

Orthographic projections: views of $Sm_2O_2I_2$ (P4/nmm) as seen from the x axis (left), the y axis (bottom) and the z axis (center).

Lattice matching - minimal size

Formula	N° atoms	strain	cell size 1	cell size 2
K	7	0.1697	1	1
Tl	7	0.1095	1	1
InSe	8	0.1354	1	1
Bi_2	8	0.1402	1	1
AgTl	8	0.0138	1	1
Ag_2	8	0.1761	1	1
P_2	8	0.1107	1	1
PbTe	8	0.1369	1	1
CaCl	8	0.2185	1	1
CdI_2	9	0.1383	1	1
$MoSe_2$	9	0.1097	1	1
Ba_2Pt	9	0.1758	1	1
Br_2Ca	9	0.1393	1	1
CaI_2	9	0.1596	1	1
$SiTe_2$	9	0.1085	1	1
Br_2Cu	9	0.101	1	1
Ca_2Si	9	0.1813	1	1
I_2Yb	9	0.157	1	1
BiClTe	9	0.1386	1	1
Cl_2Ti	9	0.1106	1	1
BrCdI	9	0.1301	1	1
HgI_2	9	0.3882	1	1
BaF_2	9	0.1325	1	1
BiBrTe	9	0.1433	1	1
NbS_2	9	0.1089	1	1
S_2Ta	9	0.1091	1	1
Se_2V	9	0.1093	1	1
AsKSn	9	0.1314	1	1
$PbTe_2$	9	0.1294	1	1
Cl_2Cu	9	0.0968	1	1
I_2Tm	9	0.1584	1	1
I_2V	9	0.1088	1	1
GeI_2	9	0.137	1	1
Se_2Zr	9	0.1086	1	1
I_2Pb	9	0.178	1	1
STl_2	9	0.1332	1	1
BiITe	9	0.1493	1	1
GeS_2	9	0.5964	1	1
$MnSe_2$	9	0.2184	1	1
DyI_2	9	0.1626	1	1
GdI_2	9	0.1459	1	1
I_2La	9	0.0062	1	1
CdI_2	9	0.1379	1	1
Sm	9	0.1641	1	3
I_2Pr	9	0.1386	1	1
Se_2W	9	0.1096	1	1
Bi_2Te_2	10	0.7523	1	1
Cu_2Sr_2	10	0.1442	1	1
Cl_2OOs	10	0.225	1	1
$LiMnTe_2$	10	0.1373	1	1

Table showing the first 50 lattice matching combinations with the smallest number of atoms in the supercell.

Lattice matching - minimal strain

Formula	N° atoms	strain	cell size 1	cell size 2
$H_2Li_2O_2$	882	0.0	65	82
$Cu_2K_2Te_2$	690	0.0001	65	50
Co_2S_2	886	0.0001	81	100
As_2Fe_2	560	0.0002	50	65
$Hf_2I_2N_2$	678	0.0002	48	65
CdClHO	548	0.0003	48	65
Tl	859	0.0003	113	181
H_2Na_2Pd	614	0.0003	49	64
$Eu_2H_2I_2$	12	0.0003	1	1
Mg_6	246	0.0004	16	25
Pb_2Se_2	912	0.0004	98	81
$TaTe_2$	483	0.0004	48	65
GeS_2	486	0.0005	49	64
Te_2W	840	0.0006	81	118
$MoTe_2$	840	0.0007	81	118
$Hf_2Se_2Si_2$	690	0.0007	50	65
HgO	806	0.0007	89	136
$F_2I_2Yb_2$	12	0.0007	1	1
CaCl	686	0.0007	81	100
$H_2Li_2O_2$	870	0.0007	64	81
$MnSe_2$	786	0.0008	81	100
$HfLiS_2$	958	0.0009	81	118
$Eu_2I_2O_2$	12	0.0009	1	1
$AsSn_2$	483	0.001	48	65
$Ba_2Ge_2Mn_2$	12	0.001	1	1
Cl_2Co	852	0.001	79	126
Cl_2Zr	852	0.0011	79	126
$AgClO_2$	852	0.0011	80	93
S_2Ti	852	0.0011	79	126
$Cu_2K_2Te_2$	678	0.0012	64	49
Cl_2Fe	852	0.0012	79	126
Mg_3	537	0.0012	49	81
Ag_2I_2	752	0.0013	82	65
As_2Fe_2	550	0.0013	49	64
Se_2Ta_4	876	0.0013	61	85
AlH_4Na	876	0.0014	85	61
CdClO	840	0.0014	81	118
$AgClO_2$	468	0.0014	44	51
$AgClO_2$	404	0.0014	38	44
AuI_4Li	732	0.0014	81	41
$K_2Mn_2Sb_2$	12	0.0014	1	1
$Br_2Ce_2O_2$	12	0.0014	1	1
O_4PTl	870	0.0015	81	64
Ga_2S_2	604	0.0015	52	73
$CoTe_2$	531	0.0015	52	73
PtS_2	840	0.0015	81	118
$Se_2Si_2Zr_2$	870	0.0015	64	81
C_2Li_2	244	0.0016	24	25
CrI_2	603	0.0016	62	77
$Ba_2F_2I_2$	870	0.0016	81	64

Table showing the first 50 lattice matching combinations with the smallest strain needed to build the supercell.

SnF_4 (P4/mmm)

Structural and electronic properties

**Formula**	SnF_4
**Spacegroup**	P4/mmm
**Prototype**	SnF4
**Parent 3D**	SnF_4
**Source DB**	COD
**DB ID**	1528582
**DF2-C09 Binding energy [meV/ Å^2]**	22.81
**RVV10 Binding energy [meV/ Å^2]**	33.26
**Band gap (PBE) [eV]**	3.92

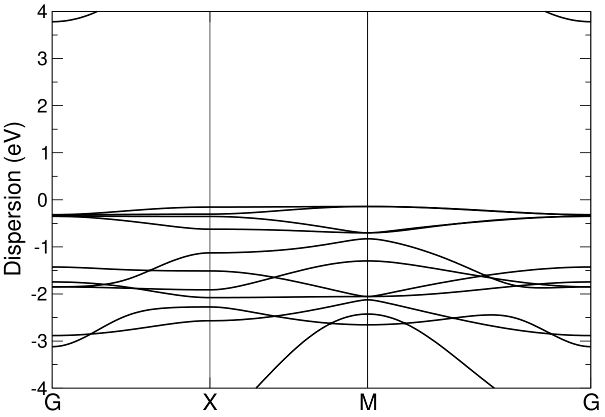

Band structure: Electronic band structure of SnF_4 (P4/mmm) in a representative window around the Fermi energy and along a high-symmetry path.

Optimized crystal structure

Structural parameters: relaxed cell (top) and atomic positions (bottom) of SnF_4 (P4/mmm) in Cartesian coordinates.

		x [Å]	y [Å]	z [Å]
$\mathbf{a_1}$		4.17593091	0.00000000	0.00000000
$\mathbf{a_2}$		0.00000000	4.17593091	0.00000000
$\mathbf{a_3}$		0.00000000	0.00000000	23.84564665
		x [Å]	y [Å]	z [Å]
•	F	2.08796546	2.08796546	13.81456287
•	Sn	2.08796546	2.08796546	11.92282333
•	F	2.08796546	2.08796546	10.03108378
•	F	2.08796546	4.17593091	11.92282333
•	F	0.00000000	2.08796546	11.92282333

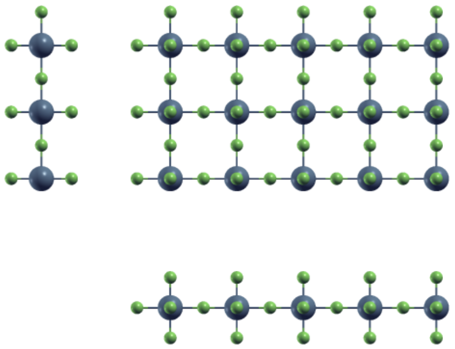

Orthographic projections: views of SnF_4 (P4/mmm) as seen from the x axis (left), the y axis (bottom) and the z axis (center).

Lattice matching - minimal size

Formula	N° atoms	strain	cell size 1	cell size 2
K	6	0.1492	1	1
AgTl	7	0.018	1	1
Ag_2	7	0.1546	1	1
Nd	8	0.1608	1	3
Ba_2Pt	8	0.1543	1	1
$ReSe_2$	8	0.1097	1	1
CaI_2	8	0.1409	1	1
$HfTe_2$	8	0.1089	1	1
Br_2Cu	8	0.3458	1	1
Ca_2Si	8	0.1589	1	1
I_2Yb	8	0.1388	1	1
Cl_2Zn	8	0.2128	1	1
S_2Ti	8	0.111	1	1
Ba_2Hg	8	0.0008	1	1
Cl_2Ni	8	0.1095	1	1
Cl_2Co	8	0.1111	1	1
Br_2V	8	0.1098	1	1
ClNZr	8	0.1103	1	1
Cl_2Fe	8	0.1113	1	1
Te_2Zr	8	0.109	1	1
$AsSe_2$	8	0.1092	1	1
I_2Tm	8	0.1399	1	1
I_2Pb	8	0.1562	1	1
BiITe	8	0.1326	1	1
BrNZr	8	0.1089	1	1
$NbSe_2$	8	0.1094	1	1
DyI_2	8	0.1434	1	1
Cl_2Zr	8	0.1112	1	1
Se_2Ta	8	0.1094	1	1
$NbSe_2$	8	0.1091	1	1
GdI_2	8	0.1299	1	1
F_2Ni	8	0.2084	1	1
Ba_2Cd	8	0.0034	1	1
NaPSn	8	0.1087	1	1
Bi_2Te_2	9	0.173	1	1
Li_2Tl_2	9	0.7698	1	1
Bi_2In_2	9	0.3811	1	1
Cl_2OOs	9	0.2104	1	1
Bi_2Mn_2	9	0.1632	1	1
$AgCuTe_2$	9	0.1863	1	1
O_2Sn_2	9	0.2197	1	1
Au_2Br_2	9	0.012	1	1
$AlLiTe_2$	9	0.1318	1	1
Ge_2Te_2	9	0.0221	1	1
Fe_2Se_2	9	0.2078	1	1
Cl_2ORu	9	0.5991	1	1
As_2Co_2	9	0.5888	1	1
N_3Na	9	0.3675	1	1
Cu_2Te_2	9	0.2157	1	1
Ge_2S_2	9	0.2367	1	1

Table showing the first 50 lattice matching combinations with the smallest number of atoms in the supercell.

Lattice matching - minimal strain

Formula	N° atoms	strain	cell size 1	cell size 2
Bi_2In_2	833	0.0	101	82
Cl_2Zn	563	0.0	64	81
Cl_4Mn	735	0.0	82	65
$Hf_2Si_2Te_2$	629	0.0001	49	64
Fe_2SeTe	805	0.0001	81	100
$Fe_2Li_2P_2$	640	0.0001	50	65
$Ag_2K_2Te_2$	614	0.0002	64	49
$FeSe_2$	592	0.0002	53	109
NSr_2	435	0.0002	48	65
Se_2Sn	435	0.0002	48	65
In	174	0.0002	25	49
Cl_2V	217	0.0002	20	39
Bi_2Se_2	446	0.0003	50	49
$Cu_2Rb_2Te_2$	997	0.0003	101	82
CI_2Y_2	565	0.0004	48	65
HgO	407	0.0004	49	81
$Ca_2Cl_2H_2$	806	0.0005	64	81
Bi_2In_2	824	0.0005	100	81
Fe_2S_2	376	0.0007	36	49
Co_2Se_2	501	0.0007	49	64
Cr_2O_4	725	0.0007	49	80
$Cl_2H_2Zr_2$	570	0.0007	36	65
K_2PdS_2	290	0.0007	40	18
F_2Ni	1000	0.0007	113	145
I_2Ti	435	0.0008	48	65
Ba_2Hg	8	0.0008	1	1
Cl_4Mn	725	0.0008	81	64
$Cu_2Rb_2Te_2$	986	0.0009	100	81
K_2PtS_2	255	0.0009	35	16
CdClHO	877	0.0009	81	118
FHOZn	256	0.0009	20	39
$PtSe_2$	759	0.001	81	118
CrI_2	435	0.001	48	65
Br_2Ca_3Si	11	0.001	1	1
Hf_3Te_2	725	0.001	64	81
$CrSe_2$	217	0.0011	20	39
NS_2Zr	899	0.0011	79	126
CoI_2	435	0.0011	48	65
PtS_2	773	0.0011	79	126
Mg_3	93	0.0012	9	16
Na	521	0.0012	79	126
$Fe_2Li_2P_2$	629	0.0013	49	64
Br_2Mg	435	0.0013	48	65
As_2O_3	995	0.0013	118	81
FeI_2	435	0.0014	48	65
$C_2Br_2Tb_2$	978	0.0014	78	98
$CrSe_2$	217	0.0014	20	39
Ca_2Cl_2	877	0.0014	85	113
$C_2Br_2Gd_2$	978	0.0014	78	98
CNNa	430	0.0015	53	55

Table showing the first 50 lattice matching combinations with the smallest strain needed to build the supercell.

SnO (C2/m)

Structural and electronic properties

**Formula**	SnO
**Spacegroup**	C2/m
**Prototype**	SnO
**Parent 3D**	Sn_4O_4
**Source DB**	ICSD
**DB ID**	424729
**DF2-C09 Binding energy [meV/ Å^2]**	N/A
**RVV10 Binding energy [meV/ Å^2]**	28.42
**Band gap (PBE) [eV]**	2.12

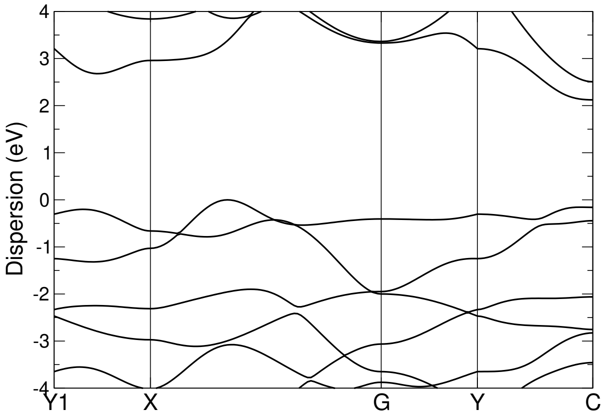

Band structure: Electronic band structure of SnO (C2/m) in a representative window around the Fermi energy and along a high-symmetry path.

Optimized crystal structure

Structural parameters: relaxed cell (top) and atomic positions (bottom) of SnO (C2/m) in Cartesian coordinates.

	x [Å]	y [Å]	z [Å]
$\mathbf{a_1}$	3.55649959	−0.09216222	0.00000000
$\mathbf{a_2}$	−0.54628578	3.51550212	0.00000000
$\mathbf{a_3}$	0.00000000	0.00000000	22.80418298

		x [Å]	y [Å]	z [Å]
●	Sn	3.25960211	0.16890602	10.01633944
●	Sn	1.79630996	1.45560893	12.78787139
•	O	3.39140059	0.05301280	12.17294308
•	O	1.66450814	1.57150511	10.63127242

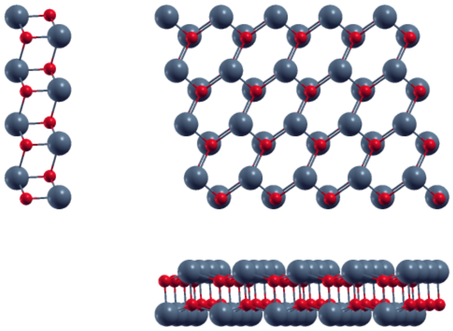

Orthographic projections: views of SnO (C2/m) as seen from the x axis (left), the y axis (bottom) and the z axis (center).

Lattice matching - minimal size

Formula	N° atoms	strain	cell size 1	cell size 2
K	5	0.6103	1	1
Tl	5	0.1442	1	1
In	5	0.1331	1	1
HgO	6	0.0491	1	1
AsSb	6	0.0941	1	1
Tl	6	2.0029	1	2
Bi_2	6	0.2849	1	1
GeTe	6	0.0982	1	1
Sn	6	0.3615	1	2
LiO	6	0.1375	1	1
P_2	6	0.1413	1	1
In	6	0.3709	1	2
PbTe	6	0.278	1	1
Mg_2	6	0.2532	1	1
$IrTe_2$	7	0.0989	1	1
MoS_2	7	0.1346	1	1
$CdCl_2$	7	0.0975	1	1
CdI_2	7	0.2809	1	1
PSn_2	7	0.0835	1	1
$MoSe_2$	7	0.1437	1	1
Br_2Zn	7	0.0882	1	1
Br_2Ca	7	0.283	1	1
$InSe_2$	7	0.0977	1	1
$AsSn_2$	7	0.085	1	1
$GeTe_2$	7	0.0968	1	1
$SiTe_2$	7	0.0904	1	1
$HfTe_2$	7	0.541	1	1
I_2Mn	7	0.0975	1	1
S_2Zr	7	0.0832	1	1
NSr_2	7	0.0952	1	1
I_2Yb	7	0.3176	1	1
PbS_2	7	0.0925	1	1
BiClTe	7	0.2815	1	1
Cl_2Ti	7	0.1413	1	1
Cl_2Zn	7	0.0558	1	1
FeI_2	7	0.096	1	1
I_2Ni	7	0.0969	1	1
Mg_3	7	0.2676	1	1
Te_2Ti	7	0.0883	1	1
CrI_2	7	0.0958	1	1
BiBrTe	7	0.2912	1	1
$RhTe_2$	7	0.0862	1	1
S_2W	7	0.1347	1	1
Bi_2Pd	7	0.0451	1	1
Ba_2Hg	7	0.5643	1	1
NbS_2	7	0.146	1	1
S_2Ta	7	0.1455	1	1
Ba_2N	7	0.5453	1	1
Se_2V	7	0.1447	1	1
Te_2Zr	7	0.5424	1	1

Table showing the first 50 lattice matching combinations with the smallest number of atoms in the supercell.

Lattice matching - minimal strain

Formula	N° atoms	strain	cell size 1	cell size 2
Hg_3N_2	499	0.0003	91	27
O_2Sn_2	8	0.0003	1	1
BrNZr	688	0.0004	91	108
Ag_2I_2	596	0.0005	92	57
Mg_3	681	0.0006	87	111
P_4	668	0.0006	92	75
$C_2Br_2La_2$	700	0.0006	79	64
$Br_2In_2O_2$	770	0.0007	86	71
HgI_2	539	0.0007	92	57
$NbSe_2$	688	0.0007	91	108
Br_2Ca_3Si	768	0.0007	93	66
$Br_2Cr_2O_2$	904	0.0007	91	90
$Ca_2Cl_2F_2$	922	0.0008	100	87
BrNZr	650	0.0008	86	102
$NbSe_2$	636	0.0009	84	100
Ba_2Hg	570	0.0009	93	66
Br_2Ca_3Si	988	0.0009	121	84
$Cu_2K_2Te_2$	916	0.0009	121	72
$Br_2In_2O_2$	358	0.0009	40	33
Au_2I_2	528	0.001	80	52
Ir_2P_2	900	0.001	124	101
I_2La_2Te	904	0.001	121	84
I_2La	818	0.001	128	102
Sm	287	0.001	47	99
I_2Pr	799	0.001	124	101
I_2Nd	799	0.001	124	101
As_2Rh_2	920	0.0011	128	102
O_2Zn	363	0.0011	42	65
NiO_2	366	0.0011	39	70
C_2Li_2	312	0.0011	43	35
$Br_2N_2Zr_2$	530	0.0011	59	49
$AgCuTe_2$	368	0.0011	48	44
$NbSe_2$	598	0.0011	79	94
$I_2Se_2Tb_2$	454	0.0011	64	33
$MoSe_2$	642	0.0011	81	106
Ag_2Br_2	920	0.0012	128	102
Se_2W	642	0.0012	81	106
CKN	385	0.0012	67	39
$Cl_2F_2Pb_2$	740	0.0012	86	66
$MoSe_2$	635	0.0012	80	105
$Cl_2H_2Zr_2$	876	0.0012	75	96
$Bi_2I_2O_2$	740	0.0012	86	66
$Cu_2Na_2Se_2$	740	0.0012	86	66
Te_2V	486	0.0012	66	74
$RhTe_2$	716	0.0012	101	104
$F_2I_2Tm_2$	740	0.0012	86	66
$TaTe_2$	666	0.0012	93	98
$Ca_2Cl_2F_2$	646	0.0012	70	61
$Br_2O_2V_2$	724	0.0012	73	72
$Cl_2Hf_2N_2$	708	0.0013	66	74

Table showing the first 50 lattice matching combinations with the smallest strain needed to build the supercell.

SnO (P4/nmm)

Structural and electronic properties

**Formula**	SnO
**Spacegroup**	P4/nmm
**Prototype**	FeSe
**Parent 3D**	Sn_2O_2
**Source DB**	COD
**DB ID**	7206470
**DF2-C09 Binding energy [meV/ Å^2]**	29.83
**RVV10 Binding energy [meV/ Å^2]**	34.63
**Band gap (PBE) [eV]**	3.01

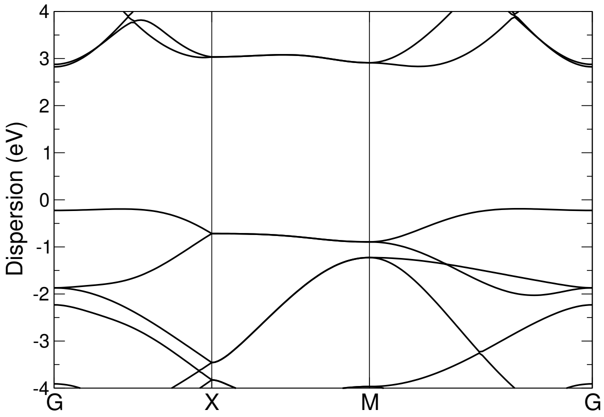

Band structure: Electronic band structure of SnO (P4/nmm) in a representative window around the Fermi energy and along a high-symmetry path.

Optimized crystal structure

Structural parameters: relaxed cell (top) and atomic positions (bottom) of SnO (P4/nmm) in Cartesian coordinates.

		x [Å]	y [Å]	z [Å]
$\mathbf{a_1}$		3.85199825	0.00000000	0.00000000
$\mathbf{a_2}$		0.00000000	3.85199825	0.00000000
$\mathbf{a_3}$		0.00000000	0.00000000	22.33476637
		x [Å]	y [Å]	z [Å]
●	Sn	1.92599912	0.00000000	12.34552763
●	Sn	0.00000000	1.92599912	9.98923873
•	O	0.00000000	0.00000000	11.16738318
•	O	1.92599912	1.92599912	11.16738318

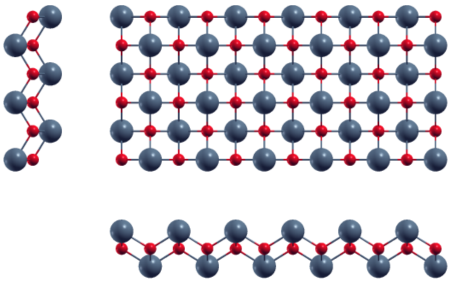

Orthographic projections: views of SnO (P4/nmm) as seen from the x axis (left), the y axis (bottom) and the z axis (center).

Lattice matching - minimal size

Formula	N° atoms	strain	cell size 1	cell size 2
K	5	0.3913	1	1
K	5	0.1925	1	1
In	5	0.1106	1	1
InSe	6	0.1518	1	1
Bi_2	6	0.1576	1	1
PbTe	6	0.1535	1	1
Sb_2	6	0.1375	1	1
I_2Mg	7	0.1423	1	1
S_2V	7	0.1099	1	1
MoS_2	7	0.1097	1	1
CdI_2	7	0.1553	1	1
Nd	7	0.7813	1	3
Br_2Ca	7	0.1565	1	1
CaI_2	7	0.1808	1	1
I_2Pr	7	0.0067	1	1
Br_2La	7	0.1426	1	1
Br_2Cu	7	0.1087	1	1
Ca_2Si	7	0.7745	1	1
I_2Yb	7	0.1777	1	1
BiClTe	7	0.1556	1	1
$AuTe_2$	7	0.1314	1	1
BrCdI	7	0.1451	1	1
$PdTe_2$	7	0.1297	1	1
I_2Zn	7	0.1349	1	1
BaF_2	7	0.1481	1	1
BiBrTe	7	0.1614	1	1
S_2W	7	0.1097	1	1
GeI_2	7	0.1407	1	1
AsKSn	7	0.1468	1	1
$PbTe_2$	7	0.1442	1	1
I_2Nd	7	0.0077	1	1
Cl_2Cu	7	0.1001	1	1
I_2Tm	7	0.1793	1	1
$SnTe_2$	7	0.1388	1	1
Cl_2V	7	0.1089	1	1
GeI_2	7	0.1538	1	1
STl_2	7	0.149	1	1
BiITe	7	0.1686	1	1
DyI_2	7	0.1843	1	1
CeI_2	7	0.0059	1	1
Se_2Yb	7	0.1409	1	1
MoS_2	7	0.1097	1	1
Cl_2Mg	7	0.1084	1	1
$BiTe_2$	7	0.1411	1	1
GdI_2	7	0.1645	1	1
$CrSe_2$	7	0.1091	1	1
$PtTe_2$	7	0.1311	1	1
O_2Pt	7	0.1109	1	1
CdI_2	7	0.1548	1	1
F_2Zn	7	0.0026	1	1

Table showing the first 50 lattice matching combinations with the smallest number of atoms in the supercell.

Lattice matching - minimal strain

Formula	N° atoms	strain	cell size 1	cell size 2
Ho_2S_2	396	0.0001	50	49
PtS_2	387	0.0002	48	65
Pb_2Se_2	628	0.0004	89	68
In	329	0.0004	58	97
O_4PSn	706	0.0005	85	61
Cu_2O_2	396	0.0005	50	49
F_4Pb	501	0.0005	64	49
$Cl_2S_2Tl_2$	736	0.0005	97	58
Cu_2S_2	8	0.0006	1	1
$As_2Cd_2K_2$	706	0.0006	85	61
$Br_2O_2Y_2$	10	0.0007	1	1
I_3Sn	52	0.0007	9	4
$Br_2Dy_2O_2$	10	0.0007	1	1
$HfLiS_2$	452	0.0007	48	65
P_4	284	0.0007	36	35
Sn	485	0.0007	85	145
Ag_2I_2	340	0.0007	49	36
La_2S_2	640	0.0007	89	71
NS_2Zr	452	0.0008	48	65
Br_2CsF	244	0.0008	36	25
Se_2Ta_4	590	0.0008	50	65
Ga_2S_3	517	0.0008	48	65
Bi_2Se_2	852	0.0008	116	97
CNRb	891	0.0009	150	97
$Br_2O_2Tb_2$	10	0.0009	1	1
$Cu_2Rb_2Te_2$	294	0.0009	36	25
Te_2Zn	387	0.001	48	65
K	393	0.001	82	65
ClNZr	678	0.001	81	118
F_2Tl_2	8	0.0011	1	1
Mg_6	316	0.0011	25	36
$MoTe_2$	387	0.0011	48	65
CNb_2S_2	946	0.0011	79	126
Se_2Sn_2	580	0.0011	81	64
Na	257	0.0012	48	65
Mg_3	764	0.0012	89	136
P_2Rh_2	8	0.0012	1	1
Bi_2In_2	244	0.0012	36	25
$As_2Mg_2Na_2$	718	0.0013	82	65
Br_2Cu_2	8	0.0013	1	1
Te_2W	387	0.0013	48	65
$HNiO_2$	236	0.0013	20	39
$H_2I_2Sr_2$	886	0.0013	100	81
$C_2I_2La_2$	982	0.0014	103	95
P_2	568	0.0014	79	126
Bi_2Pd	291	0.0014	36	49
Cl_4Mg_2	328	0.0014	55	18
Au_2I_2	532	0.0015	75	58
H_4Ti	189	0.0015	16	25
Cl_2Ti	694	0.0015	79	126

Table showing the first 50 lattice matching combinations with the smallest strain needed to build the supercell.

SnS_2 (P-3m1)

Structural and electronic properties

**Formula**	SnS_2
**Spacegroup**	P-3m1
**Prototype**	CdI2
**Parent 3D**	Sn_2S_4
**Source DB**	ICSD
**DB ID**	43003
**DF2-C09 Binding energy [meV/ Å^2]**	16.7
**RVV10 Binding energy [meV/ Å^2]**	23.74
**Band gap (PBE) [eV]**	1.51

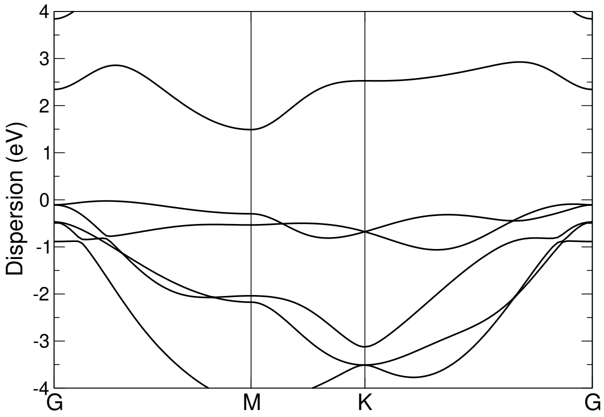

Band structure: Electronic band structure of SnS_2 (P-3m1) in a representative window around the Fermi energy and along a high-symmetry path.

Optimized crystal structure

Structural parameters: relaxed cell (top) and atomic positions (bottom) of SnS_2 (P-3m1) in Cartesian coordinates.

	x [Å]	y [Å]	z [Å]
$\mathbf{a_1}$	3.67352427	0.00000000	0.00000000
$\mathbf{a_2}$	−1.83676214	3.18136534	0.00000000
$\mathbf{a_3}$	0.00000000	0.00000000	22.96547422

		x [Å]	y [Å]	z [Å]
●	S	-0.00000000	2.12091023	9.98203326
●	Sn	0.00000000	0.00000000	11.48273711
●	S	1.83676214	1.06045511	12.98344096

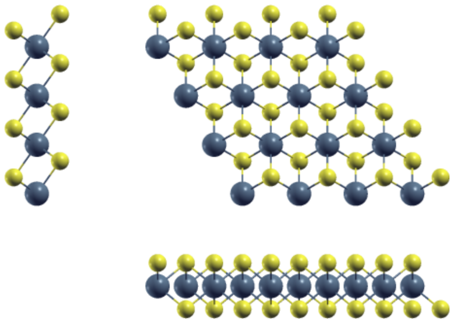

Orthographic projections: views of SnS_2 (P-3m1) as seen from the x axis (left), the y axis (bottom) and the z axis (center).

Lattice matching - minimal size

Formula	N° atoms	strain	cell size 1	cell size 2
Tl	4	0.1165	1	1
Tl	4	0.2707	1	1
In	4	1.5594	1	1
HgO	5	0.123	1	1
Bi_2	5	3.0139	1	1
As_2	5	0.0082	1	1
P_2	5	0.2631	1	1
Sb_2	5	0.4676	1	1
I_2Mg	6	0.4821	1	1
S_2V	6	1.5827	1	1
MoS_2	6	1.5881	1	1
CdI_2	6	2.9832	1	1
$AgTe_2$	6	0.118	1	1
PSn_2	6	0.0005	1	1
$MoSe_2$	6	0.2696	1	1
Br_2Ca	6	2.9995	1	1
HfS_2	6	0.0056	1	1
$AsSn_2$	6	0.0043	1	1
$CuTe_2$	6	0.006	1	1
S_2Zr	6	0.0003	1	1
Br_2La	6	0.483	1	1
Br_2Co	6	0.0087	1	1
BiClTe	6	2.988	1	1
Ca_2N	6	0.0077	1	1
Cl_2Ti	6	0.2633	1	1
$AuTe_2$	6	0.4481	1	1
HgI_2	6	0.3285	1	1
Mg_3	6	0.1139	1	1
I_2Zn	6	0.4594	1	1
BaF_2	6	0.4991	1	1
$RhTe_2$	6	0.0072	1	1
S_2W	6	1.5886	1	1
Bi_2Pd	6	0.1292	1	1
GeI_2	6	0.4773	1	1
NbS_2	6	0.2752	1	1
$CoTe_2$	6	0.0053	1	1
S_2Ta	6	0.2739	1	1
Se_2V	6	0.2719	1	1
AsKSn	6	0.4953	1	1
$SnTe_2$	6	0.4715	1	1
Cl_2V	6	0.2493	1	1
GeI_2	6	2.9634	1	1
STl_2	6	0.5017	1	1
$PtSe_2$	6	0.005	1	1
Br_2Fe	6	0.0086	1	1
GeS_2	6	0.1522	1	1
$TaTe_2$	6	0.0037	1	1
Br_2Ni	6	0.0037	1	1
$NbTe_2$	6	0.0005	1	1
Se_2Yb	6	0.4779	1	1

Table showing the first 50 lattice matching combinations with the smallest number of atoms in the supercell.

Lattice matching - minimal strain

Formula	N° atoms	strain	cell size 1	cell size 2
Sb_2SeTe_2	386	0.0	57	43
Bi_2Se_3	504	0.0	73	57
Cl_2V	339	0.0	49	64
$AsCuLi_2$	499	0.0	81	64
AsKSn	339	0.0	64	49
In_2Te_3	386	0.0	57	43
Br_2La_2	565	0.0	91	73
$I_2S_2Yb_2$	279	0.0	45	24
N_2Re	294	0.0001	37	61
Br_2La	390	0.0001	73	57
As_2Ir_2	387	0.0002	65	48
O_2Pt	255	0.0002	36	49
Br_2Ca	255	0.0002	49	36
$I_2Pr_2Si_2$	627	0.0002	81	64
Gd_2I_2	447	0.0002	73	57
S_2Zr	6	0.0003	1	1
Se_4TiZr	12	0.0003	2	1
Sb_2	419	0.0003	91	73
Cu_2F_4	714	0.0003	130	54
Ga_2Te_2	499	0.0003	81	64
HN_3OZn	765	0.0003	73	91
CrS_2	183	0.0003	25	36
Ba_2Ni_3	563	0.0003	81	64
$Ag_2K_2Te_2$	237	0.0003	39	20
MoS_2	300	0.0004	43	57
$Au_2K_2Se_2$	153	0.0004	35	8
Bi_2Se_2Te	386	0.0004	57	43
Br_2HLa	499	0.0004	81	64
I_2Mg	390	0.0004	73	57
$NbTe_2$	6	0.0005	1	1
PSn_2	6	0.0005	1	1
S_2W	300	0.0005	43	57
GeI_2	435	0.0005	81	64
$Ho_2I_2S_2$	492	0.0005	80	42
Cu_2I_2	447	0.0005	73	57
Br_2Gd_2Ge	504	0.0005	73	57
$Br_2Ho_2S_2$	624	0.0005	100	54
I_2La_2	388	0.0006	64	49
$Ga_2I_2Tb_2$	363	0.0006	49	36
I_2Pr	255	0.0006	49	36
MoS_2	300	0.0006	43	57
CS_2Ta_2	674	0.0006	73	91
$Ce_2I_2Si_2$	561	0.0006	73	57
F_4Nb	759	0.0006	118	81
BiClTe	255	0.0007	49	36
Cl_2Ti	492	0.0007	73	91
I_2Zn	543	0.0007	100	81
Se_2Yb	435	0.0008	81	64
$F_2Lu_2Se_2$	786	0.0008	100	81
H_2NiO_2	414	0.0008	43	57

Table showing the first 50 lattice matching combinations with the smallest strain needed to build the supercell.

$SnSe_2$ (P-3m1)

Structural and electronic properties

**Formula**	$SnSe_2$
**Spacegroup**	P-3m1
**Prototype**	CdI2
**Parent 3D**	$SnSe_2$
**Source DB**	ICSD
**DB ID**	43857
**DF2-C09 Binding energy [meV/ Å^2]**	17.32
**RVV10 Binding energy [meV/ Å^2]**	25.0
**Band gap (PBE) [eV]**	0.75

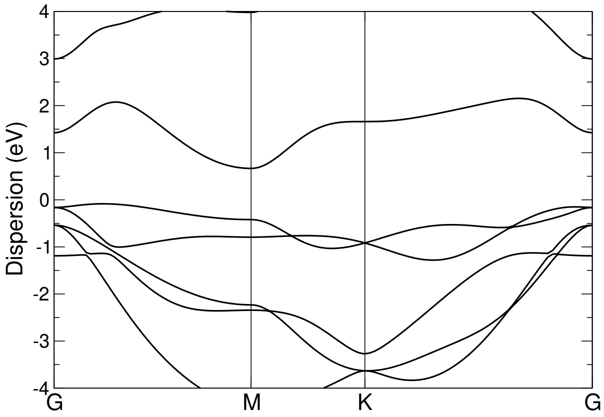

Band structure: Electronic band structure of $SnSe_2$ (P-3m1) in a representative window around the Fermi energy and along a high-symmetry path.

Optimized crystal structure

Structural parameters: relaxed cell (top) and atomic positions (bottom) of $SnSe_2$ (P-3m1) in Cartesian coordinates.

		x [Å]	y [Å]	z [Å]
$\mathbf{a_1}$		3.85497280	0.00000000	0.00000000
$\mathbf{a_2}$		−1.92748640	3.33850438	0.00000000
$\mathbf{a_3}$		0.00000000	0.00000000	23.20954373
		x [Å]	y [Å]	z [Å]
●	Se	1.92748640	1.11283479	9.99885808
●	Sn	0.00000000	0.00000000	11.60477187
●	Se	0.00000000	2.22566959	13.21068565

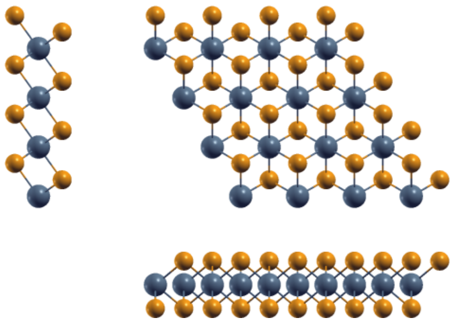

Orthographic projections: views of $SnSe_2$ (P-3m1) as seen from the x axis (left), the y axis (bottom) and the z axis (center).

Lattice matching - minimal size

Formula	N° atoms	strain	cell size 1	cell size 2
Tl	4	0.4251	1	1
Tl	4	1.5642	1	1
InSe	5	0.4507	1	1
HgO	5	0.1121	1	1
AsSb	5	0.0014	1	1
Bi_2	5	0.4651	1	1
GeTe	5	0.0047	1	1
S_2	5	0.0063	1	1
P_2	5	1.5265	1	1
PbTe	5	0.4551	1	1
CaCl	5	0.1423	1	1
$IrTe_2$	6	0.0056	1	1
$CdCl_2$	6	0.0036	1	1
Cl_2Mn	6	0.2472	1	1
CdI_2	6	0.4593	1	1
$AgTe_2$	6	0.4318	1	1
$MoSe_2$	6	1.5588	1	1
$ReSe_2$	6	0.265	1	1
S_2Ta	6	0.2499	1	1
Br_2Ca	6	0.4624	1	1
CaI_2	6	2.9832	1	1
$InSe_2$	6	0.004	1	1
$GeTe_2$	6	0.0026	1	1
$SiTe_2$	6	0.0073	1	1
I_2Mn	6	0.0037	1	1
NSr_2	6	0.0002	1	1
PbS_2	6	0.0039	1	1
BiClTe	6	0.4602	1	1
Cl_2Ti	6	1.5275	1	1
LiO_2	6	0.6408	1	1
FeI_2	6	0.0015	1	1
I_2Ni	6	0.0028	1	1
S_2Ti	6	0.2566	1	1
NbS_2	6	0.2493	1	1
CrI_2	6	0.0011	1	1
BiBrTe	6	0.4742	1	1
Bi_2Pd	6	0.116	1	1
Cl_2Ni	6	0.2663	1	1
Cl_2Co	6	0.2561	1	1
$CrTe_2$	6	0.2753	1	1
Br_2V	6	0.2639	1	1
ClNZr	6	0.2609	1	1
Cl_2Fe	6	0.2551	1	1
S_2Ta	6	1.5802	1	1
Br_2Ti	6	0.2744	1	1
$AsSe_2$	6	0.2684	1	1
$NiTe_2$	6	0.0079	1	1
I_2Tm	6	2.9676	1	1
I_2V	6	0.006	1	1
GeI_2	6	0.4556	1	1

Table showing the first 50 lattice matching combinations with the smallest number of atoms in the supercell.

Lattice matching - minimal strain

Formula	N° atoms	strain	cell size 1	cell size 2
NbS_2	339	0.0	49	64
$Cl_2Cr_2O_2$	306	0.0001	32	35
Ba_2Pt	183	0.0001	36	25
Br_2Ca_3Si	315	0.0001	43	31
Cl_2Zr	390	0.0001	57	73
Bi_2SeTe_2	638	0.0001	91	73
CI_2Y_2	8	0.0002	1	1
Pd_2S_4	471	0.0002	85	36
F_4Sn	435	0.0002	65	48
NSr_2	6	0.0002	1	1
Cl_2Fe	390	0.0003	57	73
Nd	196	0.0003	39	79
Cl_2V	183	0.0003	25	36
I_2Pr	543	0.0003	100	81
Cu_2Sr_2	499	0.0003	81	64
Br_2V	492	0.0003	73	91
CdO_2	390	0.0003	57	73
Ag_2	158	0.0003	36	25
$LiOS_2Ti$	414	0.0004	43	57
Se_2Ta	543	0.0004	81	100
BiClTe	543	0.0004	100	81
$C_2Cl_2Y_2$	522	0.0004	58	58
$Ge_2I_2La_2$	429	0.0004	57	43
$NbSe_2$	543	0.0005	81	100
Cl_2Co	390	0.0005	57	73
NS_2Ta	355	0.0005	37	61
Br_2Ca	543	0.0005	100	81
S_2Ta	339	0.0005	49	64
$ReSe_2$	492	0.0005	73	91
$Cl_2H_2Zr_2$	471	0.0005	43	57
$LiNbS_2$	403	0.0006	49	64
$AsSe_2$	543	0.0006	81	100
I_2Ti	6	0.0006	1	1
Gd_2GeI_2	705	0.0006	100	81
Ag_2F_4	792	0.0006	144	60
GdI_2	390	0.0007	73	57
BiBrTe	435	0.0007	81	64
Bi_2	419	0.0007	91	73
CdI_2	543	0.0007	100	81
FHOZn	219	0.0008	25	36
NbS_2	300	0.0008	43	57
S_2Ti	390	0.0008	57	73
$Ga_2I_2Tb_2$	786	0.0008	100	81
$NbSe_2$	543	0.0008	81	100
$Br_2Cr_2O_2$	438	0.0009	48	49
Cl_2Ni	543	0.0009	81	100
CrO_2	222	0.0009	25	49
$CrSe_2$	183	0.0009	25	36
CoI_2	6	0.0009	1	1
Ba_2Hg	339	0.001	65	48

Table showing the first 50 lattice matching combinations with the smallest strain needed to build the supercell.

SnTe ($Pmn2_1$)

Structural and electronic properties

**Formula**	SnTe
**Spacegroup**	$Pmn2_1$
**Prototype**	Massicot
**Parent 3D**	Sn_4Te_4
**Source DB**	ICSD
**DB ID**	652743
**DF2-C09 Binding energy [meV/ Å^2]**	34.21
**RVV10 Binding energy [meV/ Å^2]**	33.21
**Band gap (PBE) [eV]**	0.68

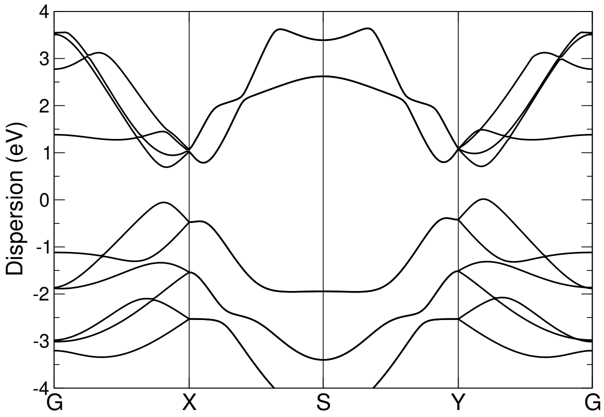

Band structure: Electronic band structure of SnTe ($Pmn2_1$) in a representative window around the Fermi energy and along a high-symmetry path.

Optimized crystal structure

Structural parameters: relaxed cell (top) and atomic positions (bottom) of SnTe ($Pmn2_1$) in Cartesian coordinates.

		x [Å]	y [Å]	z [Å]
$\mathbf{a_1}$		4.54169597	0.00004166	0.00000000
$\mathbf{a_2}$		−0.00001484	4.57944460	0.00000000
$\mathbf{a_3}$		0.00000000	0.00000000	23.13537316
		x [Å]	y [Å]	z [Å]
●	Sn	1.13501612	1.98733181	10.23696211
●	Sn	3.40629751	4.27690788	12.89841684
●	Te	1.13513947	2.11128710	13.16061045
●	Te	3.40618141	4.40075512	9.97474743

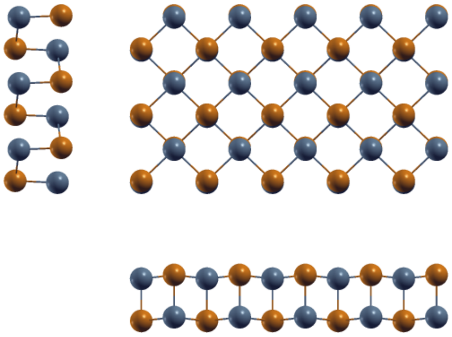

Orthographic projections: views of SnTe ($Pmn2_1$) as seen from the x axis (left), the y axis (bottom) and the z axis (center).

Lattice matching - minimal size

Formula	N° atoms	strain	cell size 1	cell size 2
AgTl	6	0.6409	1	1
Sm	6	0.1448	1	2
Br_2Zn	7	0.11	1	1
$AsSn_2$	7	0.1116	1	1
$SiTe_2$	7	0.1092	1	1
I_2Pr	7	0.5691	1	1
HgI_2	7	0.0087	1	1
Te_2Ti	7	0.11	1	1
$RhTe_2$	7	0.111	1	1
CNRb	7	0.0452	1	1
CKN	7	0.3542	1	1
I_2Nd	7	0.5719	1	1
$NiTe_2$	7	0.1093	1	1
Cl_2Cu	7	0.1386	1	1
I_2V	7	0.1089	1	1
Se_2Zr	7	0.1091	1	1
$PtSe_2$	7	0.1115	1	1
CeI_2	7	0.5669	1	1
CNNa	7	0.1928	1	1
I_2La	7	0.586	1	1
$HfSe_2$	7	0.11	1	1
Bi_2Te_2	8	0.1329	1	1
Li_2Tl_2	8	0.1542	1	1
Bi_2In_2	8	0.008	1	1
Cu_2I_2	8	0.2165	1	1
CdClHO	8	0.1119	1	1
Ir_2P_2	8	0.5713	1	1
Ag_2Br_2	8	0.5875	1	1
CdClHO	8	0.1111	1	1
S_2Sn_2	8	0.2195	1	1
Cl_2Y_2	8	0.1103	1	1
As_2Ir_2	8	0.5863	1	1
O_2Pb_2	8	0.2128	1	1
Ga_2Se_2	8	0.1086	1	1
Br_2OV	8	1.6727	1	1
CaClHO	8	0.1094	1	1
Au_2I_2	8	0.0233	1	1
Ge_2Se_2	8	0.208	1	1
Bi_2O_2	8	0.2143	1	1
Ni_2Se_2	8	0.5662	1	1
As_2Rh_2	8	0.5868	1	1
Ag_2I_2	8	0.0077	1	1
Br_2CsF	8	0.006	1	1
Ga_2Se_2	8	0.1105	1	1
As_2O_3	9	0.1938	1	1
F_4Nb	9	0.2234	1	1
NaO_4	9	0.1641	1	1
$AgNO_3$	9	0.165	1	1
ClH_3O	9	0.1306	1	1
$Ba_2H_2I_2$	10	0.0042	1	1

Table showing the first 50 lattice matching combinations with the smallest number of atoms in the supercell.

Lattice matching - minimal strain

Formula	N° atoms	strain	cell size 1	cell size 2
Co_2S_2	884	0.0002	85	136
I_3Sn	884	0.0003	136	85
H_2MgO_2	668	0.0005	42	100
$HfTe_2$	180	0.0005	21	32
In_2Se_3	244	0.0005	21	32
$Er_2F_2Se_2$	276	0.0005	21	32
Bi_2Se_2Te	599	0.0005	56	75
IO_3Tl	626	0.0005	94	50
GeI_2Y_2	599	0.0005	56	75
Br_2OV	520	0.0006	49	81
I_2V	530	0.0006	59	98
$Cl_2O_2Tm_2$	704	0.0006	50	84
FeH_2O_2	137	0.0006	8	21
F_2Na	452	0.0007	50	84
$Cl_2N_2Zr_2$	838	0.0007	67	95
As_2Li_2Nd	599	0.0007	56	75
Te_2Zr	180	0.0007	21	32
Sb_2SeTe_2	599	0.0007	56	75
$Br_2S_2Y_2$	548	0.0007	56	54
CaCl	612	0.0007	85	136
Se_2Zr	530	0.0008	59	98
Br_2Zr_2	328	0.0008	28	54
In_2Te_3	599	0.0008	56	75
$MnSe_2$	748	0.0008	85	136
Pt_2Te_2	212	0.0008	21	32
Cl_2Y_2	692	0.0008	64	109
BH_4Li	874	0.0009	70	99
$AsSn_2$	582	0.0009	63	110
$Cu_2Se_2Tl_2$	838	0.0009	73	91
$Al_2Cl_2O_2$	412	0.0009	28	50
N_2W	95	0.0009	8	21
CdO_2	276	0.001	27	56
NaPSn	180	0.001	21	32
$SnTe_2$	577	0.001	70	99
S_2V	468	0.001	42	100
$PtSe_2$	582	0.001	63	110
Br_2Zn	583	0.0011	64	109
$CeLi_2P_2$	775	0.0011	70	99
Ag_2F_4	548	0.0011	68	46
Se_2Ti	274	0.0011	28	54
Bi_2O_2	648	0.0011	72	90
$SiTe_2$	530	0.0011	59	98
$I_2N_2Ti_2$	592	0.0011	46	68
$Cu_2Se_2Tl_2$	828	0.0011	72	90
Br_2Cu	608	0.0012	71	108
$MnNaTe_2$	600	0.0012	63	87
$I_2La_2Si_2$	674	0.0012	56	75
As_2Li_2Pr	599	0.0012	56	75
Cu_2I_2	600	0.0013	63	87
InSe	374	0.0013	56	75

Table showing the first 50 lattice matching combinations with the smallest strain needed to build the supercell.

SrBrF (P4/nmm)

Structural and electronic properties

**Formula**	SrBrF
**Spacegroup**	P4/nmm
**Prototype**	PbClF
**Parent 3D**	$Sr_2Br_2F_2$
**Source DB**	ICSD
**DB ID**	35392
**DF2-C09 Binding energy [meV/ Å^2]**	26.98
**RVV10 Binding energy [meV/ Å^2]**	33.64
**Band gap (PBE) [eV]**	5.28

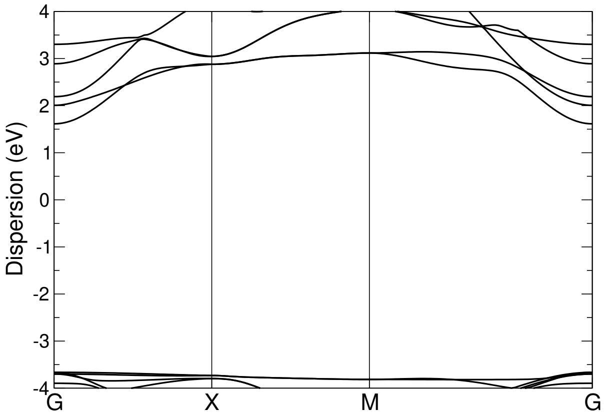

Band structure: Electronic band structure of SrBrF (P4/nmm) in a representative window around the Fermi energy and along a high-symmetry path.

Optimized crystal structure

Structural parameters: relaxed cell (top) and atomic positions (bottom) of SrBrF (P4/nmm) in Cartesian coordinates.

	x [Å]	y [Å]	z [Å]
$\mathbf{a_1}$	4.10940975	0.00000000	0.00000000
$\mathbf{a_2}$	0.00000000	4.10940975	0.00000000
$\mathbf{a_3}$	0.00000000	0.00000000	25.20781671

		x [Å]	y [Å]	z [Å]
	Sr	2.05470487	0.00000000	14.03424671
	Br	0.00000000	2.05470487	15.44796508
	Sr	0.00000000	2.05470487	11.17357000
	Br	2.05470487	0.00000000	9.75985162
	F	0.00000000	0.00000000	12.60390835
	F	2.05470487	2.05470487	12.60390835

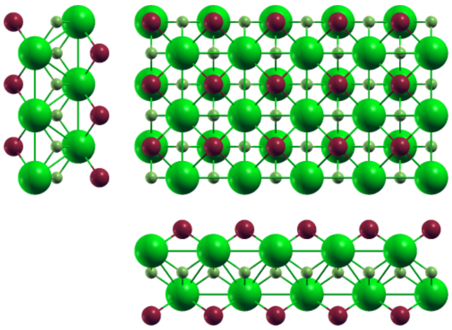

Orthographic projections: views of SrBrF (P4/nmm) as seen from the x axis (left), the y axis (bottom) and the z axis (center).

Lattice matching - minimal size

Formula	N° atoms	strain	cell size 1	cell size 2
K	7	0.1567	1	1
Bi_2	8	0.1308	1	1
GeTe	8	0.1085	1	1
AgTl	8	0.0137	1	1
Ag_2	8	0.1624	1	1
CaCl	8	0.5967	1	1
$IrTe_2$	9	0.1087	1	1
Cl_2Mn	9	0.1109	1	1
Ba_2Pt	9	0.1622	1	1
S_2Ta	9	0.1104	1	1
Br_2Ca	9	0.13	1	1
CaI_2	9	0.1477	1	1
Br_2Cu	9	0.098	1	1
Ca_2Si	9	0.1671	1	1
I_2Yb	9	0.1454	1	1
BiClTe	9	0.1294	1	1
Cl_2Zn	9	0.2214	1	1
S_2Ti	9	0.1094	1	1
NbS_2	9	0.1105	1	1
BiBrTe	9	0.1334	1	1
Ba_2Hg	9	0.0085	1	1
Cl_2Co	9	0.1094	1	1
ClNZr	9	0.1087	1	1
Cl_2Fe	9	0.1096	1	1
I_2Tm	9	0.1466	1	1
I_2Pb	9	0.1642	1	1
BiITe	9	0.1386	1	1
GeS_2	9	0.5609	1	1
$MnSe_2$	9	0.5964	1	1
DyI_2	9	0.1504	1	1
Cl_2Zr	9	0.1095	1	1
GdI_2	9	0.1356	1	1
CNNa	9	0.0671	1	1
F_2Ni	9	0.2168	1	1
I_2Pr	9	0.1294	1	1
H_2Si_2	10	0.1084	1	1
Bi_2Te_2	10	0.1822	1	1
Bi_2In_2	10	0.3977	1	1
Cu_2I_2	10	0.002	1	1
Cu_2Sr_2	10	0.1342	1	1
Cl_2OOs	10	0.2111	1	1
Cu_2Te_2	10	0.1878	1	1
Bi_2Mn_2	10	0.1685	1	1
$AgCuTe_2$	10	0.1935	1	1
O_2Sn_2	10	0.2289	1	1
S_2Sn_2	10	0.0163	1	1
$AlLiTe_2$	10	0.1377	1	1
Fe_2Se_2	10	0.2161	1	1
Cl_2ORu	10	0.2131	1	1
As_2Co_2	10	0.2107	1	1

Table showing the first 50 lattice matching combinations with the smallest number of atoms in the supercell.

Lattice matching - minimal strain

Formula	N° atoms	strain	cell size 1	cell size 2
$Hf_2Si_2Te_2$	870	0.0001	64	81
Br_2CsF	752	0.0001	82	65
Co_2S_2	560	0.0001	50	65
Hf_3Te_2	997	0.0001	82	101
Bi_2Mn_2	58	0.0001	5	7
Cl_4KTl	534	0.0002	64	25
$SiTe_2$	483	0.0002	48	65
$Br_2F_2Pb_2$	12	0.0003	1	1
$Cl_2Er_2O_2$	678	0.0003	48	65
In	159	0.0003	20	39
Se_2Zr	483	0.0004	48	65
Cl_4Mn	640	0.0004	65	50
$Hf_2Se_2Si_2$	510	0.0005	36	49
$MnSe_2$	486	0.0005	49	64
Co_2Se_2	708	0.0005	64	81
CaCl	430	0.0005	50	65
CaCl	422	0.0006	49	64
$Fe_2Li_2P_2$	882	0.0006	65	82
$NiTe_2$	483	0.0007	48	65
$AgClO_4$	876	0.0007	85	61
Cu_2F_4	930	0.0007	97	58
Cl_4Mn	629	0.0007	64	49
Hf_3Te_2	986	0.0007	81	100
$Cu_2Rb_2Te_2$	870	0.0008	81	64
Br_2CsF	742	0.0008	81	64
$AgTe_2$	639	0.0009	58	97
Mg_6	780	0.0009	49	81
As_2Fe_2	412	0.001	36	49
Se_2Ta	852	0.0011	79	126
Bi_2In_2	742	0.0012	81	64
Cl_2Zn	795	0.0012	82	101
Co_2S_2	550	0.0012	49	64
$C_2Cl_2Y_2$	696	0.0012	50	66
CaClHO	548	0.0013	48	65
As_4	702	0.0013	71	69
I_2V	483	0.0013	48	65
I_3Sn	394	0.0014	49	25
$Cl_2O_2Tm_2$	678	0.0014	48	65
$Fe_2Li_2P_2$	870	0.0014	64	81
Br_2Ti	852	0.0014	79	126
Fe_2S_2	550	0.0015	49	64
Br_2OV	942	0.0015	83	111
Cr_2O_4	618	0.0015	40	63
$RhTe_2$	531	0.0015	52	73
H_4Ti	134	0.0016	9	16
CdClHO	604	0.0016	52	73
$NbTe_2$	840	0.0016	81	118
$Br_2H_2Sr_2$	12	0.0016	1	1
N_2Re	633	0.0016	48	115
NaPSn	603	0.0016	62	77

Table showing the first 50 lattice matching combinations with the smallest strain needed to build the supercell.

SrHBr (P4/nmm)

Structural and electronic properties

**Formula**	SrHBr
**Spacegroup**	P4/nmm
**Prototype**	PbClF
**Parent 3D**	$Sr_2H_2Br_2$
**Source DB**	COD
**DB ID**	9009182
**DF2-C09 Binding energy [meV/ Å^2]**	26.3
**RVV10 Binding energy [meV/ Å^2]**	31.96
**Band gap (PBE) [eV]**	4.35

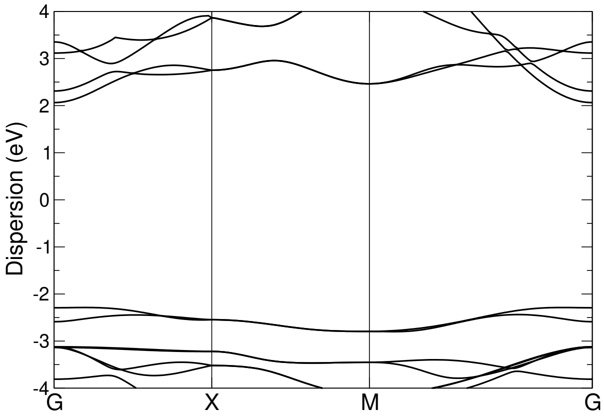

Band structure: Electronic band structure of SrHBr (P4/nmm) in a representative window around the Fermi energy and along a high-symmetry path.

Optimized crystal structure

Structural parameters: relaxed cell (top) and atomic positions (bottom) of SrHBr (P4/nmm) in Cartesian coordinates.

		x [Å]	y [Å]	z [Å]
$\mathbf{a_1}$		4.09555065	0.00000000	0.00000000
$\mathbf{a_2}$		0.00000000	4.09555065	0.00000000
$\mathbf{a_3}$		0.00000000	0.00000000	25.02830804
		x [Å]	y [Å]	z [Å]
	Sr	0.00000000	2.04777533	13.91996432
	Br	2.04777533	0.00000000	15.27214450
	Sr	2.04777533	0.00000000	11.10834372
	H	0.00000000	0.00000000	12.51415402
	H	2.04777533	2.04777533	12.51415402
	Br	0.00000000	2.04777533	9.75616354

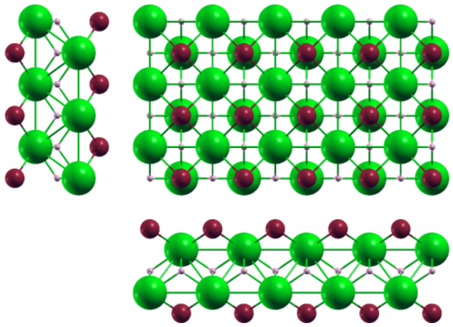

Orthographic projections: views of SrHBr (P4/nmm) as seen from the x axis (left), the y axis (bottom) and the z axis (center).

Lattice matching - minimal size

Formula	N° atoms	strain	cell size 1	cell size 2
K	7	0.1583	1	1
Bi_2	8	0.1319	1	1
GeTe	8	0.1089	1	1
AgTl	8	0.0131	1	1
Ag_2	8	0.1642	1	1
CaCl	8	0.207	1	1
$CdCl_2$	9	0.1086	1	1
Cl_2Mn	9	0.1105	1	1
CdI_2	9	0.1302	1	1
Nd	9	0.1709	1	3
Ba_2Pt	9	0.1639	1	1
S_2Ta	9	0.1101	1	1
Br_2Ca	9	0.1311	1	1
CaI_2	9	0.1492	1	1
$InSe_2$	9	0.1087	1	1
I_2Mn	9	0.1087	1	1
Br_2Cu	9	0.0983	1	1
Ca_2Si	9	0.1689	1	1
I_2Yb	9	0.1468	1	1
BiClTe	9	0.1305	1	1
I_2Ni	9	0.1084	1	1
S_2Ti	9	0.109	1	1
NbS_2	9	0.1102	1	1
BiBrTe	9	0.1346	1	1
Cl_2Co	9	0.1091	1	1
NbS_2	9	0.1112	1	1
Cl_2Fe	9	0.1093	1	1
S_2Ta	9	0.1114	1	1
I_2Tm	9	0.1481	1	1
I_2Pb	9	0.1659	1	1
BiITe	9	0.1399	1	1
GeS_2	9	0.5655	1	1
$MnSe_2$	9	0.2069	1	1
DyI_2	9	0.1519	1	1
Cl_2Zr	9	0.1092	1	1
GdI_2	9	0.1369	1	1
CNNa	9	0.0678	1	1
F_2Ni	9	0.2186	1	1
CdI_2	9	0.1299	1	1
I_2Pr	9	0.1306	1	1
H_2Si_2	10	0.1088	1	1
Bi_2Te_2	10	0.1841	1	1
Bi_2In_2	10	0.4012	1	1
Cu_2I_2	10	0.0004	1	1
Cu_2Sr_2	10	0.1354	1	1
Cl_2OOs	10	0.2129	1	1
$LiMnTe_2$	10	0.1294	1	1
$AgCuTe_2$	10	0.1951	1	1
O_2Sn_2	10	0.0335	1	1
S_2Sn_2	10	0.0173	1	1

Table showing the first 50 lattice matching combinations with the smallest number of atoms in the supercell.

Lattice matching - minimal strain

Formula	N° atoms	strain	cell size 1	cell size 2
H_4Ti	134	0.0	9	16
Fe_2S_2	550	0.0001	49	64
Cu_2Se_2	886	0.0001	81	100
Bi_2Pd	942	0.0002	89	136
GeS_2	363	0.0002	36	49
$Cl_2O_2Tm_2$	678	0.0003	48	65
O_2Pt	237	0.0003	20	39
As_2Co_2	708	0.0003	64	81
$AgTe_2$	537	0.0003	49	81
Co_2Se_2	718	0.0003	65	82
CaClHO	548	0.0004	48	65
$Ba_2H_2I_2$	882	0.0004	82	65
Cu_2I_2	10	0.0004	1	1
H_2Li_2Pd	134	0.0005	9	16
Tl	445	0.0006	58	97
Br_2OV	942	0.0006	83	111
F_2Na	483	0.0006	48	65
Cl_2Mg	840	0.0006	81	118
As_2Fe_2	412	0.0006	36	49
Br_2Ni	840	0.0006	81	118
$Cl_2O_2Yb_2$	678	0.0007	48	65
F_2Ni	786	0.0007	81	100
$Ag_2K_2Te_2$	510	0.0007	49	36
Br_2CsF	742	0.0008	81	64
Mg_4	736	0.0009	58	97
Ga_2S_2	958	0.0009	81	118
$Hf_2Si_2Te_2$	882	0.0009	65	82
Cl_4Mn	629	0.0009	64	49
Cu_2F_4	930	0.0009	97	58
$AgClO_4$	876	0.0009	85	61
$MnSe_2$	495	0.001	50	65
$NiTe_2$	483	0.001	48	65
Ca_2Cl_2	708	0.001	64	81
$Ge_2Se_2Zr_2$	870	0.001	64	81
H_2Na_2Pd	461	0.001	36	49
Br_2Ca_3Si	978	0.001	100	63
CaCl	430	0.001	50	65
BrNZr	852	0.001	79	126
Co_2Se_2	708	0.0011	64	81
$Hf_2Se_2Si_2$	510	0.0011	36	49
$Ba_2H_2I_2$	870	0.0012	81	64
Cl_2ORu	698	0.0012	63	80
Fe_2Se_2	886	0.0012	81	100
$Cu_2Se_2Tl_2$	12	0.0013	1	1
In	159	0.0013	20	39
$H_2Na_2O_2$	366	0.0013	25	36
$Br_2F_2Pb_2$	12	0.0013	1	1
Br_2Pr_2	548	0.0014	48	65
NbS_2	711	0.0014	64	109
$C_2Br_2Gd_2$	858	0.0015	65	78

Table showing the first 50 lattice matching combinations with the smallest strain needed to build the supercell.

SrHI (P4/nmm)

Structural and electronic properties

**Formula**	SrHI
**Spacegroup**	P4/nmm
**Prototype**	PbClF
**Parent 3D**	$Sr_2H_2I_2$
**Source DB**	COD
**DB ID**	9009184
**DF2-C09 Binding energy [meV/ Å^2]**	17.42
**RVV10 Binding energy [meV/ Å^2]**	22.18
**Band gap (PBE) [eV]**	3.93

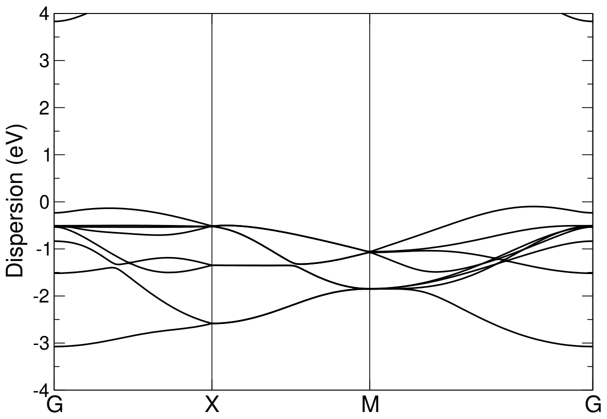

Band structure: Electronic band structure of SrHI (P4/nmm) in a representative window around the Fermi energy and along a high-symmetry path.

Optimized crystal structure

Structural parameters: relaxed cell (top) and atomic positions (bottom) of SrHI (P4/nmm) in Cartesian coordinates.

		x [Å]	y [Å]	z [Å]
$\mathbf{a_1}$		4.29214967	0.00000000	0.00000000
$\mathbf{a_2}$		0.00000000	4.29214967	0.00000000
$\mathbf{a_3}$		0.00000000	0.00000000	25.45944632
		x [Å]	y [Å]	z [Å]
●	Sr	0.00000000	2.14607484	14.03164465
●	I	2.14607484	0.00000000	15.58468620
●	Sr	2.14607484	0.00000000	11.42780167
•	H	0.00000000	0.00000000	12.72972316
•	H	2.14607484	2.14607484	12.72972316
●	I	0.00000000	2.14607484	9.87476012

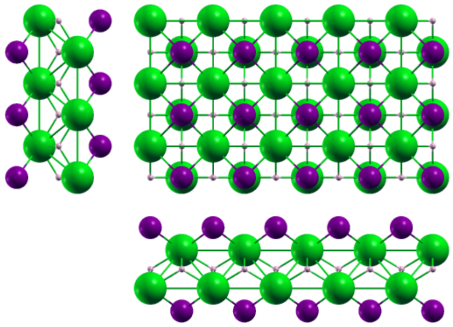

Orthographic projections: views of SrHI (P4/nmm) as seen from the x axis (left), the y axis (bottom) and the z axis (center).

Lattice matching - minimal size

Formula	N° atoms	strain	cell size 1	cell size 2
K	7	0.0028	1	1
K	7	0.1378	1	1
Na	7	0.1098	1	1
Ag_2	8	0.1424	1	1
$MoTe_2$	9	0.1093	1	1
Nd	9	0.1478	1	3
Ba_2Pt	9	0.1422	1	1
CaI_2	9	0.1307	1	1
Te_2V	9	0.1089	1	1
Ca_2Si	9	0.1462	1	1
I_2Zn	9	0.1088	1	1
$CrTe_2$	9	0.111	1	1
PtS_2	9	0.1096	1	1
CNRb	9	0.3145	1	1
CdClO	9	0.109	1	1
Se_2Ti	9	0.1101	1	1
Br_2Ti	9	0.1111	1	1
Te_2W	9	0.1093	1	1
I_2Tm	9	0.1299	1	1
I_2Pb	9	0.1438	1	1
OTl_2	9	0.109	1	1
Br_2Cr	9	0.1111	1	1
DyI_2	9	0.1328	1	1
F_2Ni	9	0.5667	1	1
Se_2Ta	9	0.1113	1	1
F_2Zn	9	0.2209	1	1
Bi_2Te_2	10	0.1587	1	1
Fe_2Te_2	10	0.6128	1	1
Li_2Tl_2	10	0.1867	1	1
Ca_2Cl_2	10	0.2114	1	1
Cl_2OOs	10	0.575	1	1
NS_2Zr	10	0.1097	1	1
S_2Sn_2	10	0.0179	1	1
Cu_2S_2	10	0.2172	1	1
Au_2Br_2	10	0.007	1	1
Br_2Cu_2	10	0.2193	1	1
Fe_2Se_2	10	0.5651	1	1
N_3Na	10	0.0308	1	1
Cu_2Te_2	10	0.5862	1	1
$AgBrO_2$	10	0.1284	1	1
Br_2Zr_2	10	0.1102	1	1
O_2Sn_2	10	0.2178	1	1
P_2Rh_2	10	0.2165	1	1
F_2Tl_2	10	0.2166	1	1
BN	10	0.1492	1	2
Au_2I_2	10	0.0201	1	1
Sb_2Te_2	10	0.145	1	1
O_2Sn_2	10	0.2238	1	1
Ge_2Se_2	10	0.0264	1	1
Cu_2Se_2	10	0.5683	1	1

Table showing the first 50 lattice matching combinations with the smallest number of atoms in the supercell.

Lattice matching - minimal strain

Formula	N° atoms	strain	cell size 1	cell size 2
$Hf_2Se_2Si_2$	612	0.0	41	61
$Br_2H_2Yb_2$	678	0.0001	49	64
Br_2Cu_2	886	0.0001	81	100
$As_2Fe_2Li_2$	678	0.0001	49	64
$Ge_2Te_2Zr_2$	690	0.0001	50	65
$Br_2Lu_2O_2$	690	0.0001	50	65
$Br_2Er_2O_2$	882	0.0001	65	82
$HNiO_2$	844	0.0002	54	130
NaPSn	483	0.0002	48	65
Pt_2Te_2	548	0.0002	48	65
$Si_2Te_2Zr_2$	510	0.0002	36	49
F_2Ni	363	0.0002	36	49
Fe_2SeTe	550	0.0002	49	64
As_2Co_2	706	0.0003	61	85
Ca_2O_2	736	0.0003	58	97
Fe_2Se_2	412	0.0003	36	49
Cl_2ORu	348	0.0004	30	42
H_2Li_2Pd	395	0.0004	25	49
Ca_2Cl_2	706	0.0004	61	85
$Ge_2Se_2Zr_2$	876	0.0004	61	85
CNRb	681	0.0004	81	65
$I_2S_2Tl_2$	510	0.0005	36	49
$Er_2F_2Se_2$	678	0.0006	48	65
$GeNi_3Te_2$	678	0.0007	48	65
F_2Zn	795	0.0007	82	101
Ga_2Se_2	958	0.0007	81	118
Cu_2Se_2	412	0.0008	36	49
$CuGeO_3$	968	0.0008	83	94
$Br_2O_2Tm_2$	870	0.0008	64	81
$HfTe_2$	483	0.0008	48	65
In_2Se_3	613	0.0009	48	65
$Br_2Er_2O_2$	870	0.0009	64	81
H_4Ti	395	0.0009	25	49
$Br_2H_2Zr_2$	606	0.0009	36	65
$Br_2Ca_2H_2$	882	0.001	65	82
$Br_2Lu_2O_2$	678	0.001	49	64
Fe_2S_2	294	0.001	25	36
As_2Ru_2	708	0.001	64	81
Bi_2Pd	639	0.001	58	97
Pb_2Se_2	874	0.0012	89	85
$Ca_2Mn_2Si_2$	882	0.0012	65	82
Ca_2Cl_2	708	0.0012	64	81
$AgNO_2$	588	0.0012	56	63
Cl_2Rb_2	70	0.0012	9	4
$C_2Cl_2Y_2$	816	0.0012	56	80
$Ge_2Te_2Zr_2$	678	0.0012	49	64
Cu_2Te_2	962	0.0013	85	113
F_2Zn	786	0.0013	81	100
Te_2Zr	483	0.0013	48	65
Ag_2Te_2	708	0.0013	64	81

Table showing the first 50 lattice matching combinations with the smallest strain needed to build the supercell.

SrI_2 (Pmmn)

Structural and electronic properties

**Formula**	SrI_2
**Spacegroup**	Pmmn
**Prototype**	SrI2
**Parent 3D**	Sr_4I_8
**Source DB**	ICSD
**DB ID**	203137
**DF2-C09 Binding energy [meV/ Å^2]**	21.24
**RVV10 Binding energy [meV/ Å^2]**	26.59
**Band gap (PBE) [eV]**	3.84

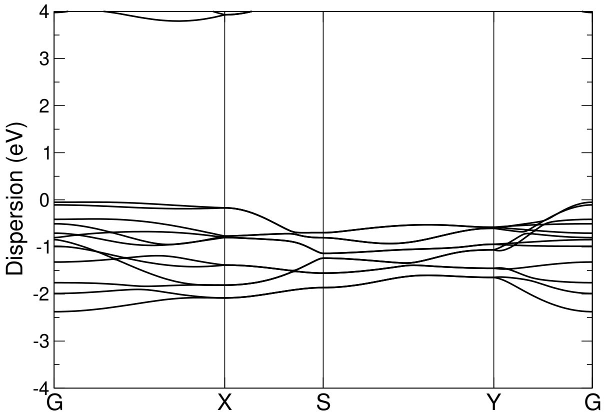

Band structure: Electronic band structure of SrI_2 (Pmmn) in a representative window around the Fermi energy and along a high-symmetry path.

Optimized crystal structure

Structural parameters: relaxed cell (top) and atomic positions (bottom) of SrI_2 (Pmmn) in Cartesian coordinates.

		x [Å]	y [Å]	z [Å]
$\mathbf{a_1}$		4.74648209	0.00000000	0.00000000
$\mathbf{a_2}$		0.00000000	8.21981278	0.00000000
$\mathbf{a_3}$		0.00000000	0.00000000	23.51735661
		x [Å]	y [Å]	z [Å]
●	Sr	1.18662052	3.04041705	11.02495343
●	Sr	3.55986157	7.15037831	12.49272764
●	I	1.18662052	5.11054351	13.62233285
●	I	3.55986157	1.00061622	9.89533273
●	I	3.55986157	5.07913333	9.89351796
●	I	1.18662052	0.96924990	13.62418446

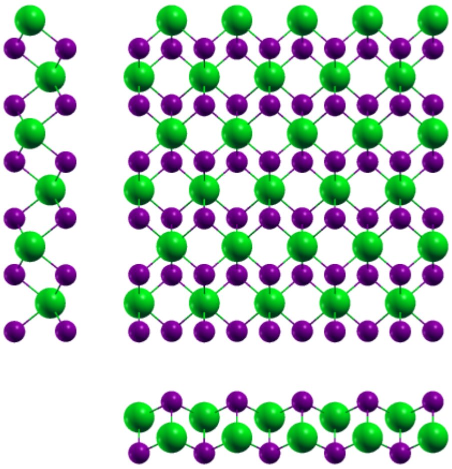

Orthographic projections: views of SrI_2 (Pmmn) as seen from the x axis (left), the y axis (bottom) and the z axis (center).

Lattice matching - minimal size

Formula	N° atoms	strain	cell size 1	cell size 2
Bi_2In_2	10	0.2294	1	1
Bi_2	10	0.1166	1	2
Au_2Se_2	10	2.1752	1	1
Br_2CsF	10	0.2272	1	1
K_2PdS_2	11	0.6533	1	1
IO_3Tl	11	0.223	1	1
K_2PdSe_2	11	3.4208	1	1
CdI_2	12	0.1157	1	2
Br_2Ca	12	0.1162	1	2
Ca_2Si	12	0.0074	1	2
BiClTe	12	0.1158	1	2
Ba_2Hg	12	0.1224	1	2
$Cu_2Rb_2Te_2$	12	0.229	1	1
$PbTe_2$	12	0.4717	1	2
Cl_2Cu	12	0.1463	1	2
$SnTe_2$	12	0.4564	1	2
AuI_4Li	12	0.732	1	1
P_2	12	0.1497	1	3
CdI_2	12	0.1155	1	2
CS_3Tl_2	12	0.1718	1	1
Ba_2Cd	12	0.1239	1	2
I_2Pr	12	0.1159	1	2
Br_2Ca_3Si	12	0.3252	1	1
Bi_2Te_2	14	0.0053	1	2
Cu_2I_2	14	0.0428	1	2
Au_2Br_2	14	0.1208	1	2
AgTl	14	0.3514	1	4
N_3Na	14	0.0135	1	2
I_2La_2	14	0.4775	1	2
Sb_2Te_2	14	0.0086	1	2
Bi_2O_2	14	0.0439	1	2
$AgClO_2$	14	0.1243	1	2
La_2S_2	14	0.1318	1	2
PbS_2Sn	14	0.031	1	2
Gd_2I_2	14	0.4679	1	2
Cl_2Ti	15	0.1498	1	3
Cl_2Cu	15	0.1288	1	3
Cl_2V	15	0.1444	1	3
$CrSe_2$	15	0.1438	1	3
$CrSe_2$	15	0.145	1	3
As_2O_3	16	0.8865	1	2
SSb_2Te_2	16	0.474	1	2
F_4Sn	16	0.1219	1	2
Br_2Gd_2Ge	16	0.4687	1	2
Sb_2Te_3	16	0.1173	1	2
F_4Nb	16	0.0408	1	2
ClH_3O	16	0.002	1	2
Bi_2SeTe_2	16	0.1168	1	2
GeI_2Y_2	16	0.2622	1	2
Br_2La_2P	16	0.1156	1	2

Table showing the first 50 lattice matching combinations with the smallest number of atoms in the supercell.

Lattice matching - minimal strain

Formula	N° atoms	strain	cell size 1	cell size 2
STl_2	435	0.0001	32	81
$Cl_2Er_2O_2$	198	0.0001	8	25
Se_2Zr	123	0.0001	8	25
In_2Se_2	304	0.0003	18	49
BH_4Li	942	0.0003	43	114
$SiTe_2$	123	0.0003	8	25
$HfLiS_2$	182	0.0003	9	32
Br_3Cs	496	0.0003	50	49
$CeLi_2P_2$	828	0.0004	43	114
As_2O_3	437	0.0004	32	49
PtS_2	150	0.0005	9	32
In	21	0.0006	2	9
Te_2Zn	150	0.0006	9	32
Cl_2Hf_2	542	0.0007	25	98
Gd_2I_2	806	0.0007	49	128
$MoTe_2$	150	0.0008	9	32
$TaTe_2$	588	0.0008	37	122
Br_2HLa	714	0.0008	43	114
$C_2Cl_2Y_2$	822	0.0008	34	103
I_2Zn	255	0.0009	18	49
$CuGeO_3$	180	0.0009	10	24
Nd	97	0.0009	8	49
IKO_3	94	0.0009	9	8
BaF_2	435	0.0009	32	81
$AuCrTe_4$	630	0.0009	42	63
$NiTe_2$	123	0.0009	8	25
Te_2W	150	0.0009	9	32
Br_2Gd_2Ge	934	0.001	49	128
I_2V	123	0.001	8	25
NS_2Zr	182	0.0011	9	32
Cl_4Mg_2	258	0.0011	16	27
$Ga_2I_2Y_2$	678	0.0011	32	81
Ga_2S_3	214	0.0011	9	32
In_2Te_3	597	0.0012	32	81
$SbSe_2Tl$	260	0.0012	20	35
$Br_2Hf_2N_2$	66	0.0012	3	8
Sb_2SeTe_2	597	0.0012	32	81
$AsCuLi_2$	714	0.0012	43	114
CuO_2	219	0.0013	12	49
Cl_4Mg_2	924	0.0013	83	71
CdH_2O_2	214	0.0014	9	32
$Cl_2Ho_2O_2$	198	0.0014	8	25
$Ga_2Ge_2Te_2$	942	0.0014	43	114
Cl_2Rb_2	820	0.0014	84	79
$Cl_2Rh_2Te_2$	438	0.0014	28	45
Cl_2La_2	304	0.0015	18	49
CKN	402	0.0015	35	64
Na	86	0.0015	9	32
Ga_2Te_2	714	0.0015	43	114
CNNa	156	0.0015	12	28

Table showing the first 50 lattice matching combinations with the smallest strain needed to build the supercell.

Ta_2CS_2 (P-3m1)

Structural and electronic properties

**Formula**	Ta_2CS_2
**Spacegroup**	P-3m1
**Prototype**	Bi2Te2S
**Parent 3D**	Ta_2CS_2
**Source DB**	ICSD
**DB ID**	23791
**DF2-C09 Binding energy [meV/ Å^2]**	23.53
**RVV10 Binding energy [meV/ Å^2]**	31.03
**Band gap (PBE) [eV]**	N/A

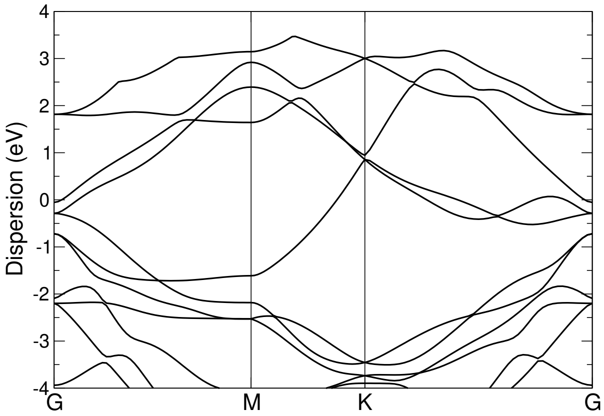

Band structure: Electronic band structure of Ta_2CS_2 (P-3m1) in a representative window around the Fermi energy and along a high-symmetry path.

Optimized crystal structure

Structural parameters: relaxed cell (top) and atomic positions (bottom) of Ta_2CS_2 (P-3m1) in Cartesian coordinates.

		x [Å]	y [Å]	z [Å]
$\mathbf{a_1}$		3.28591355	0.00000000	0.00000000
$\mathbf{a_2}$		−1.64295677	2.84568461	0.00000000
$\mathbf{a_3}$		0.00000000	0.00000000	25.63705415
		x [Å]	y [Å]	z [Å]
●	Ta	1.64295677	0.94856154	14.00492841
●	S	1.64295677	0.94856154	10.00932495
●	Ta	1.64295677	2.84568461	11.63212623
●	C	-0.00000000	1.89712307	12.81852615
●	S	1.64295677	2.84568461	15.62772964

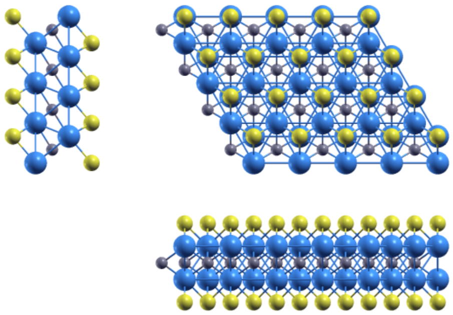

Orthographic projections: views of Ta_2CS_2 (P-3m1) as seen from the x axis (left), the y axis (bottom) and the z axis (center).

Lattice matching - minimal size

Formula	N° atoms	strain	cell size 1	cell size 2
Tl	6	0.0051	1	1
Sn	6	0.1283	1	1
In	6	0.1314	1	1
As_2	7	0.4476	1	1
LiO	7	0.0077	1	1
P_2	7	0.0002	1	1
Mg_2	7	0.1406	1	1
PSn_2	8	0.4697	1	1
$MoSe_2$	8	0.0043	1	1
Br_2Zn	8	0.4975	1	1
HfS_2	8	0.4543	1	1
$AsSn_2$	8	0.4794	1	1
$SiTe_2$	8	2.9326	1	1
$CuTe_2$	8	0.4534	1	1
S_2Zr	8	0.4678	1	1
NSr_2	8	3.0353	1	1
NiO_2	8	1.5562	1	1
Ca_2N	8	0.4489	1	1
Cl_2Ti	8	0.0001	1	1
Mg_3	8	0.15	1	1
Te_2Ti	8	0.4982	1	1
$RhTe_2$	8	0.4867	1	1
NbS_2	8	0.0082	1	1
$CoTe_2$	8	0.4551	1	1
S_2Ta	8	0.0073	1	1
Se_2V	8	0.0059	1	1
$NiTe_2$	8	2.9248	1	1
S_2Sn	8	0.4685	1	1
$PtSe_2$	8	0.4811	1	1
CoI_2	8	3.0193	1	1
$TaTe_2$	8	0.4779	1	1
Br_2Ni	8	0.4592	1	1
$FeSe_2$	8	0.124	1	1
$NbTe_2$	8	0.4673	1	1
Cl_2Mg	8	0.4593	1	1
I_2La	8	0.3159	1	1
$CrSe_2$	8	0.0089	1	1
N_2Re	8	1.6004	1	1
Se_2Sn	8	3.0321	1	1
CoO_2	8	1.5523	1	1
$HfSe_2$	8	0.4983	1	1
Se_2W	8	0.0045	1	1
CdClHO	9	0.4764	1	1
Br_2Pr_2	9	0.5006	1	1
$HNiO_2$	9	0.271	1	1
Ag_2Br_2	9	0.317	1	1
CdClHO	9	0.4847	1	1
Cl_2Y_2	9	0.4941	1	1
As_2Ir_2	9	0.3219	1	1
Ga_2Se_2	9	2.9711	1	1

Table showing the first 50 lattice matching combinations with the smallest number of atoms in the supercell.

Lattice matching - minimal strain

Formula	N° atoms	strain	cell size 1	cell size 2
FeO_2	327	0.0	36	49
STl_2	416	0.0	61	37
KNO_3	25	0.0	4	1
$Cl_2O_2Tm_2$	543	0.0001	57	43
CBr_2Y_2	565	0.0001	64	49
Cl_2Ti	8	0.0001	1	1
$AlLiTe_2$	116	0.0001	16	9
$NbTe_2$	674	0.0001	91	73
Li_2P_2Pr	205	0.0002	25	16
CdClHO	661	0.0002	81	64
$Hf_2I_2N_2$	789	0.0002	81	64
CaClHO	457	0.0002	57	43
$F_2Se_2Tm_2$	330	0.0002	36	25
P_2	7	0.0002	1	1
$Ga_2Ge_2Te_2$	221	0.0003	25	16
C_2	77	0.0003	9	16
$LiMnSe_2$	280	0.0004	36	25
S_2Zr	674	0.0004	91	73
Sb_2	157	0.0004	25	16
Cu_2O_4	495	0.0004	51	40
$Cl_2H_2Lu_2$	986	0.0004	100	81
Cl_2Y_2	516	0.0005	64	49
In_2Te_3	650	0.0005	81	49
$Ce_2I_2S_2$	629	0.0005	79	39
PTe_2Zr_2	425	0.0005	49	36
Ca_2Si	512	0.0005	79	39
Sb_2SeTe_2	650	0.0005	81	49
CdClHO	593	0.0006	73	57
S_2Sn	674	0.0006	91	73
Br_2La_2	189	0.0006	25	16
CCl_2Gd_2	565	0.0006	64	49
$Cl_2Hg_2N_2$	519	0.0007	75	24
$Br_2In_2O_2$	716	0.0007	82	51
Cl_2Mg	743	0.0007	100	81
$TaTe_2$	597	0.0007	81	64
CNb_2S_2	10	0.0007	1	1
CoO_2	327	0.0008	36	49
F_2Na	414	0.0008	57	43
Br_2Zn	467	0.0008	64	49
Br_2Ni	743	0.0008	100	81
$I_2Nd_2S_2$	395	0.0008	49	25
$NiTe_2$	414	0.0008	57	43
$Cl_2O_2Yb_2$	543	0.0008	57	43
$PtSe_2$	536	0.0008	73	57
Au_2Br_2	825	0.0009	117	60
Bi_2Se_2Te	650	0.0009	81	49
HN_3OZn	11	0.0009	1	1
PTe_2Ti_2	905	0.0009	100	81
BaF_2	416	0.0009	61	37
I_2Y_2	280	0.001	36	25

Table showing the first 50 lattice matching combinations with the smallest strain needed to build the supercell.

$TaNS_2$ (P3m1)

Structural and electronic properties

**Formula**	$TaNS_2$
**Spacegroup**	P3m1
**Prototype**	LiNbS2
**Parent 3D**	$N_2S_4Ta_2$
**Source DB**	MPDS
**DB ID**	S1903895
**DF2-C09 Binding energy [meV/ Å^2]**	28.68
**RVV10 Binding energy [meV/ Å^2]**	N/A
**Band gap (PBE) [eV]**	0.19

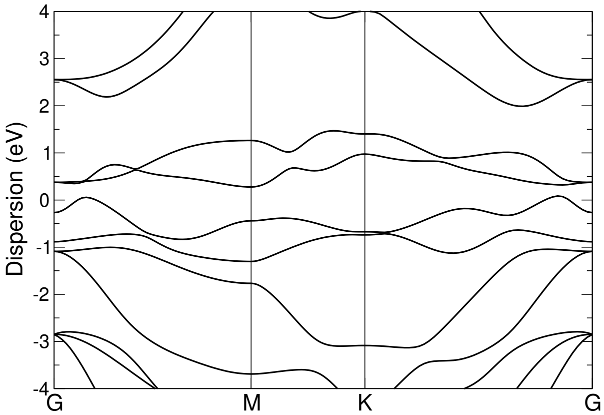

Band structure: Electronic band structure of $TaNS_2$ (P3m1) in a representative window around the Fermi energy and along a high-symmetry path.

Optimized crystal structure

Structural parameters: relaxed cell (top) and atomic positions (bottom) of $TaNS_2$ (P3m1) in Cartesian coordinates.

	x [Å]	y [Å]	z [Å]
$\mathbf{a_1}$	−1.50266666	−2.60269499	0.00000000
$\mathbf{a_2}$	3.00533331	−0.00000000	0.00000000
$\mathbf{a_3}$	0.00000000	0.00000000	21.61695005

		x [Å]	y [Å]	z [Å]
●	Ta	0.00000000	-1.73513000	0.88218566
●	S	0.00000000	0.00000000	-1.16588367
●	S	0.00000000	0.00000000	2.56993809
●	N	1.50266666	-0.86756500	-2.28624008

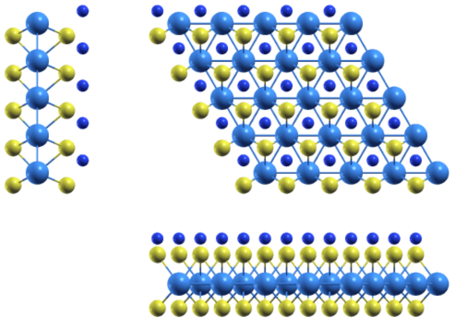

Orthographic projections: views of $TaNS_2$ (P3m1) as seen from the x axis (left), the y axis (bottom) and the z axis (center).

Lattice matching - minimal size

Formula	N° atoms	strain	cell size 1	cell size 2
Nd	5	0.2681	1	1
Tl	5	0.2072	1	1
Tl	5	0.4548	1	1
CaCl	6	0.3117	1	1
CrS_2	7	0.0092	1	1
Cl_2Mn	7	0.4691	1	1
$MoSe_2$	7	0.4529	1	1
S_2Ta	7	0.4744	1	1
S_2Ti	7	0.4873	1	1
NbS_2	7	0.4732	1	1
CrI_2	7	13.6028	1	1
N_2W	7	0.0022	1	1
Cl_2Co	7	0.4864	1	1
NbS_2	7	0.4623	1	1
Br_2V	7	0.5012	1	1
ClNZr	7	0.4956	1	1
Cl_2Fe	7	0.4845	1	1
S_2Ta	7	0.4602	1	1
Se_2V	7	0.4569	1	1
CdO_2	7	0.486	1	1
O_2Zn	7	0.0057	1	1
$MnSe_2$	7	0.3115	1	1
Br_2Cr	7	2.9965	1	1
Cl_2Zr	7	0.4855	1	1
F_2Ni	7	0.3333	1	1
Se_2W	7	0.4533	1	1
Cl_2OOs	8	0.33	1	1
$HNiO_2$	8	0.0052	1	1
Cl_2Hf_2	8	0.4775	1	1
Bi_2Mn_2	8	0.329	1	1
Fe_2Se_2	8	0.3321	1	1
Cl_2ORu	8	0.3189	1	1
As_2Co_2	8	0.3219	1	1
Cl_2Zr_2	8	0.4991	1	1
Mg_4	8	0.2068	1	1
Co_2S_2	8	0.313	1	1
Cu_2Se_2	8	0.3345	1	1
$AgClO_2$	8	0.7678	1	1
$LiNbS_2$	8	0.4746	1	1
Co_2Se_2	8	0.3247	1	1
Ca_2Cl_2	8	0.3204	1	1
Cl_2NSc_2	9	0.4703	1	1
CCl_2Sc_2	9	0.498	1	1
$LiOS_2Ti$	9	0.4653	1	1
FeH_2O_2	9	0.0034	1	1
InSe	10	0.2215	2	1
$Cl_2H_2Zr_2$	10	0.463	1	1
$Br_2H_2Zr_2$	10	0.4971	1	1
LiO_2	10	1.8866	1	2
$Hf_2Si_2Te_2$	10	0.3259	1	1

Table showing the first 50 lattice matching combinations with the smallest number of atoms in the supercell.

Lattice matching - minimal strain

Formula	N° atoms	strain	cell size 1	cell size 2
$Ga_2Gd_2I_2$	694	0.0	100	49
$Dy_2I_2S_2$	60	0.0	9	4
Br_2Cd	91	0.0	16	9
Br_2Cr	304	0.0	49	36
Gd_2GeI_2	511	0.0	79	39
ClNZr	403	0.0001	64	49
Br_2Fe	219	0.0001	36	25
CrI_2	471	0.0001	81	49
CdI_2	433	0.0001	79	39
CBr_2Y_2	180	0.0001	25	16
Br_2Co	219	0.0001	36	25
$Br_2H_2Zr_2$	412	0.0001	49	36
$Ga_2I_2Y_2$	346	0.0001	49	25
Br_2PY_2	109	0.0001	16	9
CCl_2Lu_2	269	0.0002	36	25
Cl_2Sc_2	340	0.0002	49	36
I_2Pr	216	0.0002	39	20
NSr_2	355	0.0002	61	37
$CrTe_2$	304	0.0003	49	36
$Ga_2I_2Tb_2$	694	0.0003	100	49
BiClTe	433	0.0003	79	39
Br_2Mg	471	0.0003	81	49
As_2	194	0.0003	36	25
Br_2Ti	304	0.0003	49	36
H_2Li_2Pt	80	0.0004	10	8
I_2Pr	433	0.0004	79	39
BaF_2	271	0.0004	49	25
Nd	424	0.0004	81	100
S_2Ta	643	0.0004	100	81
Cl_2Ni	357	0.0004	57	43
Br_2La_2P	511	0.0004	79	39
FeI_2	471	0.0004	81	49
Bi_2	498	0.0005	100	49
NbS_2	643	0.0005	100	81
Se_2Sn	355	0.0005	61	37
C	113	0.0005	16	49
CCl_2Gd_2	180	0.0005	25	16
Cl_2Fe	463	0.0005	73	57
$I_2O_2Yb_2$	276	0.0005	39	20
$ReSe_2$	357	0.0005	57	43
CdI_2	433	0.0005	79	39
$LiNbS_2$	580	0.0005	81	64
Au_2I_2	664	0.0006	118	48
Cl_2Hf_2	580	0.0006	81	64
Br_2Zn	148	0.0006	25	16
$Br_2H_2Zr_2$	550	0.0006	64	49
S_2Ta	516	0.0006	81	64
Br_2Ca	547	0.0006	100	49
$Br_2Eu_2O_2$	276	0.0006	39	20
Cl_2Y_2	164	0.0006	25	16

Table showing the first 50 lattice matching combinations with the smallest strain needed to build the supercell.

TaS_2 (P-3m1)

Structural and electronic properties

**Formula**	TaS_2
**Spacegroup**	P-3m1
**Prototype**	CdI2
**Parent 3D**	TaS_2
**Source DB**	COD
**DB ID**	9011539
**DF2-C09 Binding energy [meV/ Å^2]**	22.35
**RVV10 Binding energy [meV/ Å^2]**	27.56
**Band gap (PBE) [eV]**	N/A

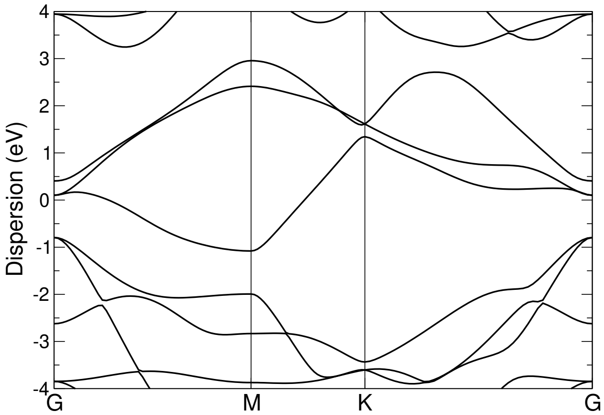

Band structure: Electronic band structure of TaS_2 (P-3m1) in a representative window around the Fermi energy and along a high-symmetry path.

Optimized crystal structure

Structural parameters: relaxed cell (top) and atomic positions (bottom) of TaS_2 (P-3m1) in Cartesian coordinates.

		x [Å]	y [Å]	z [Å]
$\mathbf{a_1}$		3.37656875	0.00000000	0.00000000
$\mathbf{a_2}$		−1.68828437	2.92419431	0.00000000
$\mathbf{a_3}$		0.00000000	0.00000000	23.09113143
		x [Å]	y [Å]	z [Å]
●	S	0.00000000	1.94946287	13.07851374
●	S	-0.00000000	0.00000000	10.01260862
●	Ta	1.68828437	0.97473144	11.54557478

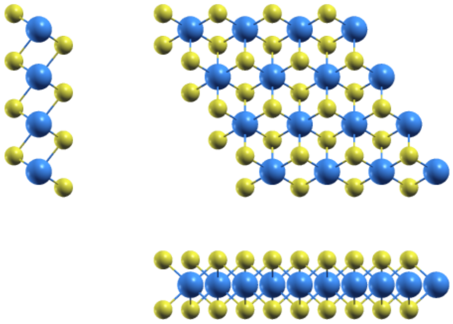

Orthographic projections: views of TaS_2 (P-3m1) as seen from the x axis (left), the y axis (bottom) and the z axis (center).

Lattice matching - minimal size

Formula	N° atoms	strain	cell size 1	cell size 2
Tl	4	0.1436	1	1
Tl	4	0.0077	1	1
Sn	4	0.1203	1	1
In	4	0.1229	1	1
GeTe	5	2.9235	1	1
Mg_2	5	0.1305	1	1
$IrTe_2$	6	2.9354	1	1
CrS_2	6	0.2731	1	1
$CdCl_2$	6	2.9096	1	1
Cl_2Mn	6	0.0021	1	1
$AgTe_2$	6	0.1463	1	1
$MoSe_2$	6	0.0084	1	1
Br_2Zn	6	0.4642	1	1
$InSe_2$	6	2.9145	1	1
$AsSn_2$	6	0.4472	1	1
$GeTe_2$	6	0.5011	1	1
$SiTe_2$	6	0.4747	1	1
I_2Mn	6	2.9111	1	1
NSr_2	6	0.4948	1	1
PbS_2	6	0.4837	1	1
LiO_2	6	0.3193	1	1
FeI_2	6	0.4982	1	1
I_2Ni	6	0.5016	1	1
S_2Ti	6	0.005	1	1
Mg_3	6	0.1386	1	1
Te_2Ti	6	0.465	1	1
NbS_2	6	0.0005	1	1
CrI_2	6	0.497	1	1
$RhTe_2$	6	0.4541	1	1
N_2W	6	0.2633	1	1
Cl_2Co	6	0.0047	1	1
NbS_2	6	0.0047	1	1
ClNZr	6	0.0082	1	1
Cl_2Fe	6	0.0039	1	1
S_2Ta	6	0.0056	1	1
Se_2V	6	0.0069	1	1
$NiTe_2$	6	0.4731	1	1
Cl_2Cu	6	0.1131	1	1
I_2V	6	0.4781	1	1
Se_2Zr	6	0.4757	1	1
$PtSe_2$	6	0.4488	1	1
CdO_2	6	0.0045	1	1
CoI_2	6	2.8509	1	1
O_2Zn	6	0.2682	1	1
Cl_2Zr	6	0.0043	1	1
$FeSe_2$	6	0.117	1	1
Br_2Mg	6	0.4978	1	1
F_2Na	6	0.4692	1	1
Se_2Sn	6	0.4941	1	1
NaPSn	6	3.0344	1	1

Table showing the first 50 lattice matching combinations with the smallest number of atoms in the supercell.

Lattice matching - minimal strain

Formula	N° atoms	strain	cell size 1	cell size 2
$InSe_2$	300	0.0	57	43
Cl_2Y_2	624	0.0	100	81
Br_2Y_2	447	0.0	73	57
$LiNbS_2$	7	0.0001	1	1
$LiMnSe_2$	291	0.0001	49	36
Se_2Zr	435	0.0001	81	64
Br_2Pr_2	565	0.0001	91	73
$Cl_2Er_2H_2$	429	0.0001	57	43
STl_2	123	0.0002	25	16
PbS_2	390	0.0002	73	57
Cu_2Sr_2	439	0.0002	81	49
Br_2Hg_3	32	0.0002	9	1
$F_2Se_2Tm_2$	363	0.0002	49	36
NSr_2	339	0.0002	64	49
I_2Mn	300	0.0003	57	43
$Cl_2Er_2O_2$	627	0.0003	81	64
H_2Si_2	343	0.0003	57	43
$CdCl_2$	300	0.0004	57	43
$SbSe_2Tl$	86	0.0004	18	8
Ga_2Se_2	447	0.0004	73	57
Cl_2La_2	208	0.0004	36	25
O_2Zn	543	0.0004	81	100
Cu_2Sr_2	331	0.0004	61	37
FeO_2	183	0.0005	25	36
$Hf_2Si_2Te_2$	483	0.0005	65	48
NbS_2	6	0.0005	1	1
FeH_2O_2	674	0.0005	73	91
Se_2Sn	339	0.0005	64	49
I_2Y_2	291	0.0005	49	36
CBr_2Y_2	705	0.0005	100	81
$SiTe_2$	435	0.0005	81	64
BiBrTe	294	0.0006	61	37
Ni_2Te_2	343	0.0006	57	43
NS_2Ta	516	0.0006	64	81
PbS_2Sn	782	0.0006	150	83
$Dy_2I_2S_2$	102	0.0006	16	9
As_2Sn_2	208	0.0006	36	25
CrI_2	339	0.0007	64	49
CI_2Y_2	437	0.0007	64	49
$I_2Sb_2Te_2$	561	0.0007	115	36
Bi_2Te_2	393	0.0007	79	39
GeTe	257	0.0007	57	43
I_2La_2P	488	0.0007	81	49
Ga_2Se_2	624	0.0007	100	81
La_2S_2	77	0.0007	15	8
Br_2Gd_2	208	0.0007	36	25
N_2W	492	0.0007	73	91
$HfSe_2$	492	0.0007	91	73
Te_2Ti	492	0.0008	91	73
H_2MnO_2	414	0.0008	43	57

Table showing the first 50 lattice matching combinations with the smallest strain needed to build the supercell.

TaS_2 (P-6m2)

Structural and electronic properties

**Formula**	TaS_2
**Spacegroup**	P-6m2
**Prototype**	MoS2
**Parent 3D**	TaS_2
**Source DB**	ICSD
**DB ID**	651083
**DF2-C09 Binding energy [meV/ Å^2]**	23.18
**RVV10 Binding energy [meV/ Å^2]**	28.6
**Band gap (PBE) [eV]**	N/A

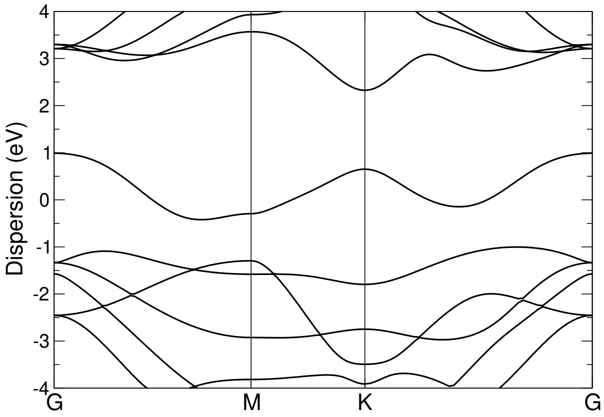

Band structure: Electronic band structure of TaS_2 (P-6m2) in a representative window around the Fermi energy and along a high-symmetry path.

Optimized crystal structure

Structural parameters: relaxed cell (top) and atomic positions (bottom) of TaS_2 (P-6m2) in Cartesian coordinates.

		x [Å]	y [Å]	z [Å]
$\mathbf{a_1}$		3.33653480	0.00000000	0.00000000
$\mathbf{a_2}$		−1.66826740	2.88952390	0.00000000
$\mathbf{a_3}$		0.00000000	0.00000000	23.15236953
		x [Å]	y [Å]	z [Å]
●	Ta	0.00000000	0.00000000	11.57618476
●	S	0.00000000	1.92634926	10.01089492
●	S	0.00000000	1.92634926	13.14147461

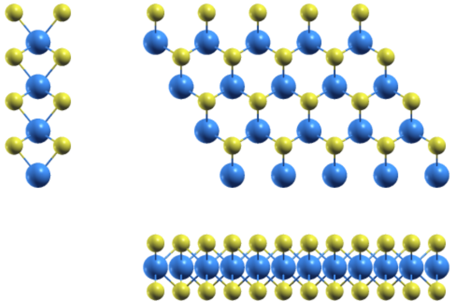

Orthographic projections: views of TaS_2 (P-6m2) as seen from the x axis (left), the y axis (bottom) and the z axis (center).

Lattice matching - minimal size

Formula	N° atoms	strain	cell size 1	cell size 2
Tl	4	0.1488	1	1
Tl	4	0.0022	1	1
Sn	4	0.1236	1	1
In	4	0.1264	1	1
Bi_2	5	13.6532	1	1
GeTe	5	2.9979	1	1
P_2	5	0.0073	1	1
Mg_2	5	0.1347	1	1
$IrTe_2$	6	3.0101	1	1
$CdCl_2$	6	2.9837	1	1
Cl_2Mn	6	0.0036	1	1
$AgTe_2$	6	0.1517	1	1
PSn_2	6	0.4517	1	1
$MoSe_2$	6	0.0029	1	1
S_2Ta	6	0.0057	1	1
Br_2Zn	6	0.4785	1	1
Br_2Ca	6	13.5903	1	1
$InSe_2$	6	2.9886	1	1
$AsSn_2$	6	0.4611	1	1
$SiTe_2$	6	2.8396	1	1
I_2Mn	6	2.9852	1	1
S_2Zr	6	0.4499	1	1
PbS_2	6	0.4985	1	1
Cl_2Ti	6	0.0072	1	1
I_2Ni	6	2.9728	1	1
Mg_3	6	0.1434	1	1
Te_2Ti	6	0.4793	1	1
NbS_2	6	0.0052	1	1
$RhTe_2$	6	0.4681	1	1
N_2W	6	0.2714	1	1
NbS_2	6	0.0009	1	1
Se_2V	6	0.0013	1	1
$NiTe_2$	6	2.832	1	1
Cl_2Cu	6	0.1196	1	1
S_2Sn	6	0.4505	1	1
I_2V	6	0.4928	1	1
Se_2Zr	6	2.8448	1	1
$PtSe_2$	6	0.4627	1	1
CoI_2	6	2.9236	1	1
GeS_2	6	0.2067	1	1
$TaTe_2$	6	0.4596	1	1
$FeSe_2$	6	0.1198	1	1
$NbTe_2$	6	0.4494	1	1
F_2Na	6	0.4836	1	1
N_2Re	6	1.5477	1	1
Se_2Sn	6	2.936	1	1
$HfSe_2$	6	0.4793	1	1
Se_2W	6	0.0028	1	1
H_2Si_2	7	2.9928	1	1
Cu_2I_2	7	0.3347	1	1

Table showing the first 50 lattice matching combinations with the smallest number of atoms in the supercell.

Lattice matching - minimal strain

Formula	N° atoms	strain	cell size 1	cell size 2
ClH_3O	545	0.0	100	49
Ca_2Si	222	0.0	49	25
$Ce_2I_2S_2$	297	0.0	49	25
$AsSn_2$	543	0.0	100	81
$Cl_2O_2Y_2$	486	0.0001	64	49
GeTe	219	0.0001	49	36
$Cl_2O_2Yb_2$	561	0.0001	73	57
N_2Re	255	0.0001	36	49
$Ga_2I_2Tb_2$	537	0.0001	81	49
F_2Na	390	0.0002	73	57
$PbTe_2$	123	0.0002	25	16
Br_2PY_2	233	0.0002	36	25
Br_2Ca	390	0.0002	81	49
Cl_2Y_2	499	0.0003	81	64
AsSb	257	0.0003	57	43
CdClHO	565	0.0003	91	73
CBr_2Y_2	563	0.0003	81	64
In_2S_3	327	0.0003	49	36
H_2Si_2	291	0.0003	49	36
NS_2Ta	643	0.0004	81	100
I_2Pr	294	0.0004	61	37
Br_2Ca	294	0.0004	61	37
$Ga_2Gd_2I_2$	537	0.0004	81	49
Br_2Cd	183	0.0004	36	25
PTe_2Zr_2	386	0.0004	57	43
BiClTe	294	0.0005	61	37
$RhTe_2$	492	0.0005	91	73
Ga_2Se_2	388	0.0006	64	49
$InSe_2$	255	0.0006	49	36
$PtSe_2$	543	0.0006	100	81
$TaTe_2$	543	0.0006	100	81
Br_2Pr_2	447	0.0006	73	57
$Cl_2Ho_2O_2$	486	0.0006	64	49
CoI_2	300	0.0007	57	43
O_2Sn_2	431	0.0007	77	50
Gd_2GeI_2	368	0.0007	61	37
H_2Li_2Pt	643	0.0007	81	80
$Ga_2I_2Tb_2$	405	0.0008	61	37
$Cl_2Er_2H_2$	363	0.0008	49	36
$MnNaTe_2$	139	0.0008	25	16
$F_2Ho_2Se_2$	258	0.0008	36	25
CdI_2	294	0.0008	61	37
Bi_2	341	0.0008	81	49
NbS_2	6	0.0009	1	1
I_2Mn	255	0.0009	49	36
CCl_2Gd_2	563	0.0009	81	64
$Br_2Hf_2N_2$	549	0.0009	79	52
Br_2Y_2	388	0.0009	64	49
Ga_2Se_2	499	0.001	81	64
$Cl_2O_2Tm_2$	561	0.001	73	57

Table showing the first 50 lattice matching combinations with the smallest strain needed to build the supercell.

$TaSe_2$ (P-3m1)

Structural and electronic properties

**Formula**	$TaSe_2$
**Spacegroup**	P-3m1
**Prototype**	CdI2
**Parent 3D**	$TaSe_2$
**Source DB**	ICSD
**DB ID**	651954
**DF2-C09 Binding energy [meV/ Å^2]**	23.06
**RVV10 Binding energy [meV/ Å^2]**	29.11
**Band gap (PBE) [eV]**	N/A

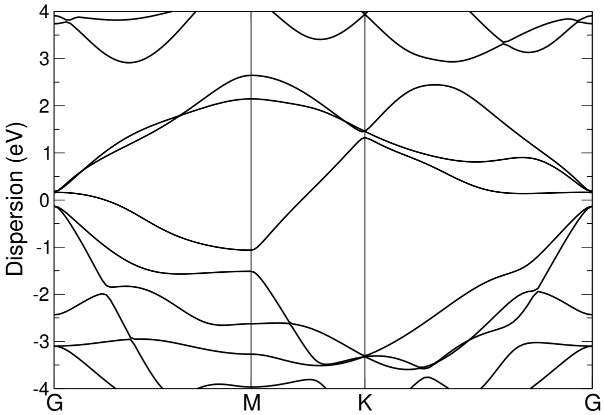

Band structure: Electronic band structure of $TaSe_2$ (P-3m1) in a representative window around the Fermi energy and along a high-symmetry path.

Optimized crystal structure

Structural parameters: relaxed cell (top) and atomic positions (bottom) of $TaSe_2$ (P-3m1) in Cartesian coordinates.

		x [Å]	y [Å]	z [Å]
$\mathbf{a_1}$		3.49808244	0.00000000	0.00000000
$\mathbf{a_2}$		−1.74904122	3.02942825	0.00000000
$\mathbf{a_3}$		0.00000000	0.00000000	23.34560706
		x [Å]	y [Å]	z [Å]
●	Ta	0.00000000	0.00000000	11.67280353
●	Se	0.00000000	2.01961884	13.32719863
●	Se	1.74904122	1.00980942	10.01840843

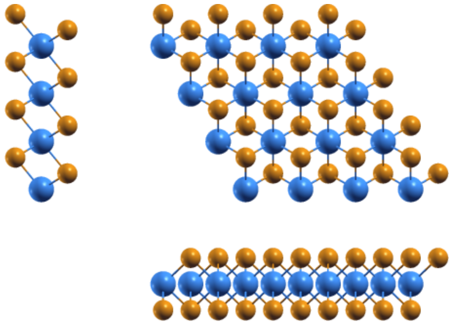

Orthographic projections: views of $TaSe_2$ (P-3m1) as seen from the x axis (left), the y axis (bottom) and the z axis (center).

Lattice matching - minimal size

Formula	N° atoms	strain	cell size 1	cell size 2
Tl	4	0.1302	1	1
Sn	4	0.1126	1	1
Na	4	0.0068	1	1
In	4	0.1144	1	1
In	4	0.2702	1	1
HgO	5	0.1397	1	1
AsSb	5	0.4482	1	1
GeTe	5	0.4632	1	1
S_2	5	0.4671	1	1
Mg_2	5	0.12	1	1
Sb_2	5	3.0321	1	1
$IrTe_2$	6	0.4654	1	1
CrS_2	6	0.2498	1	1
S_2V	6	0.2749	1	1
$CdCl_2$	6	0.4605	1	1
$MoTe_2$	6	0.0092	1	1
$AgTe_2$	6	0.1324	1	1
$ReSe_2$	6	0.0056	1	1
$InSe_2$	6	0.4614	1	1
$GeTe_2$	6	0.458	1	1
$HfTe_2$	6	0.4868	1	1
I_2Mn	6	0.4608	1	1
NSr_2	6	0.4522	1	1
ReS_2	6	0.2578	1	1
$AuTe_2$	6	2.9276	1	1
$PdTe_2$	6	0.5009	1	1
FeI_2	6	0.4553	1	1
I_2Ni	6	0.4585	1	1
Mg_3	6	0.1262	1	1
CrI_2	6	0.4542	1	1
I_2Zn	6	2.9882	1	1
Te_2Zn	6	0.009	1	1
Bi_2Pd	6	0.1481	1	1
Ba_2Hg	6	0.3101	1	1
Cl_2Ni	6	0.0047	1	1
$CrTe_2$	6	0.0014	1	1
PtS_2	6	0.0079	1	1
Br_2V	6	0.0064	1	1
ClNZr	6	0.0084	1	1
Ba_2N	6	2.8472	1	1
Se_2Ti	6	0.0052	1	1
Br_2Ti	6	0.0008	1	1
Te_2Zr	6	2.834	1	1
Te_2W	6	0.0093	1	1
$AsSe_2$	6	0.0033	1	1
BrNZr	6	0.002	1	1
$NbSe_2$	6	0.0043	1	1
CoI_2	6	0.4493	1	1
Br_2Cr	6	0.0011	1	1
Se_2Ta	6	0.0042	1	1

Table showing the first 50 lattice matching combinations with the smallest number of atoms in the supercell.

Lattice matching - minimal strain

Formula	N° atoms	strain	cell size 1	cell size 2
AsKSn	183	0.0	36	25
I_2Y_2	499	0.0	81	64
$Cl_2Er_2H_2$	786	0.0	100	81
S_2	419	0.0001	91	73
$InSe_2$	543	0.0001	100	81
$F_2Ho_2Se_2$	486	0.0001	64	49
$GeNi_3Te_2$	561	0.0001	73	57
I_2Mn	543	0.0002	100	81
$Cl_2H_2Sc_2$	9	0.0002	1	1
Ni_2SbTe_2	638	0.0002	91	73
$CdCl_2$	543	0.0003	100	81
O_2Pt	543	0.0003	81	100
N_3W_2	255	0.0003	25	36
Br_2Ho_2	343	0.0003	57	43
CrS_2	339	0.0004	49	64
Ga_2Se_2	565	0.0004	91	73
CaI_2	294	0.0004	61	37
H_2Si_2	624	0.0004	100	81
Br_2Cu	384	0.0004	72	56
Ni_2Te_2	624	0.0005	100	81
$Br_2Ho_2S_2$	291	0.0005	49	24
NaPSn	390	0.0005	73	57
I_2La_2	208	0.0005	36	25
$As_2Cd_2K_2$	237	0.0005	39	20
O_2Zn	300	0.0005	43	57
Cl_2Tb_2	499	0.0006	81	64
Sb_2Te_2	84	0.0006	16	9
$IrTe_2$	492	0.0006	91	73
$LiMnSe_2$	499	0.0006	81	64
Bi_2S_3	386	0.0006	57	43
$Br_2Eu_2F_2$	840	0.0006	118	81
$PtTe_2$	300	0.0006	57	43
$Er_2I_2O_2$	840	0.0006	118	81
I_2Zn	255	0.0006	49	36
Pt_2Te_2	447	0.0007	73	57
O_4PSn	237	0.0007	39	20
$F_2Se_2Tm_2$	627	0.0007	81	64
CoH_2O_2	536	0.0007	57	73
Ca_2Si	75	0.0007	16	9
$Ce_2I_2S_2$	102	0.0007	16	9
$Bi_2Br_2O_2$	840	0.0007	118	81
Br_2Ti	6	0.0008	1	1
I_4Zr_2	627	0.0008	115	47
GeTe	462	0.0008	100	81
$AlLiTe_2$	139	0.0008	25	16
Br_2Tb_2	343	0.0008	57	43
$F_2Lu_2Se_2$	363	0.0009	49	36
Br_2PY_2	437	0.0009	64	49
$Br_2H_2Zr_2$	9	0.001	1	1
Cu_2O_4	618	0.001	74	66

Table showing the first 50 lattice matching combinations with the smallest strain needed to build the supercell.

$TaSe_2$ (P-6m2)

Structural and electronic properties

**Formula**	$TaSe_2$
**Spacegroup**	P-6m2
**Prototype**	MoS2
**Parent 3D**	Ta_2Se_4
**Source DB**	ICSD
**DB ID**	651956
**DF2-C09 Binding energy [meV/ Å^2]**	22.59
**RVV10 Binding energy [meV/ Å^2]**	28.15
**Band gap (PBE) [eV]**	N/A

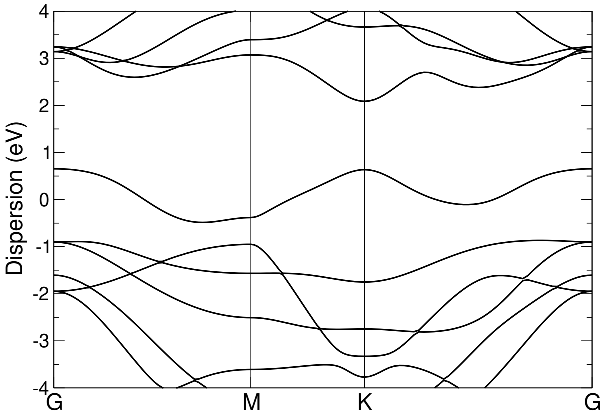

Band structure: Electronic band structure of $TaSe_2$ (P-6m2) in a representative window around the Fermi energy and along a high-symmetry path.

Optimized crystal structure

Structural parameters: relaxed cell (top) and atomic positions (bottom) of $TaSe_2$ (P-6m2) in Cartesian coordinates.

	x [Å]	y [Å]	z [Å]
$\mathbf{a_1}$	3.46678543	0.00000000	0.00000000
$\mathbf{a_2}$	−1.73339271	3.00232425	0.00000000
$\mathbf{a_3}$	0.00000000	0.00000000	23.36868775

		x [Å]	y [Å]	z [Å]
●	Ta	0.00000000	0.00000000	11.68434387
●	Se	1.73339271	1.00077475	10.00513466
●	Se	1.73339271	1.00077475	13.36355309

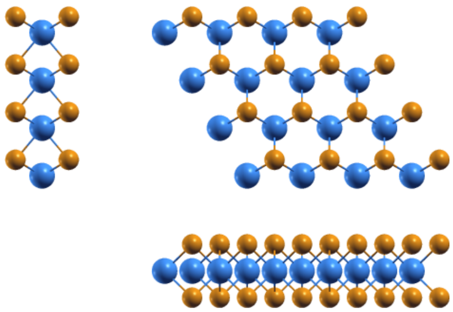

Orthographic projections: views of $TaSe_2$ (P-6m2) as seen from the x axis (left), the y axis (bottom) and the z axis (center).

Lattice matching - minimal size

Formula	N° atoms	strain	cell size 1	cell size 2
Tl	4	0.1334	1	1
Sn	4	0.1143	1	1
In	4	0.1163	1	1
HgO	5	0.1433	1	1
AsSb	5	0.4586	1	1
GeTe	5	0.4739	1	1
S_2	5	0.4779	1	1
Mg_2	5	0.1224	1	1
$IrTe_2$	6	0.4762	1	1
CrS_2	6	0.2554	1	1
$CdCl_2$	6	0.4712	1	1
$AgTe_2$	6	0.1357	1	1
$ReSe_2$	6	0.0014	1	1
$InSe_2$	6	0.4721	1	1
$GeTe_2$	6	0.4686	1	1
$HfTe_2$	6	0.498	1	1
I_2Mn	6	0.4715	1	1
NSr_2	6	0.4627	1	1
I_2Yb	6	13.6444	1	1
PbS_2	6	0.4522	1	1
ReS_2	6	0.2638	1	1
$AuTe_2$	6	2.9836	1	1
FeI_2	6	0.4659	1	1
I_2Ni	6	0.4691	1	1
S_2Ti	6	0.0074	1	1
Mg_3	6	0.1291	1	1
CrI_2	6	0.4648	1	1
I_2Zn	6	3.0453	1	1
Bi_2Pd	6	0.1522	1	1
Ba_2Hg	6	0.3187	1	1
N_2W	6	0.2464	1	1
Cl_2Ni	6	0.0005	1	1
Cl_2Co	6	0.0077	1	1
$CrTe_2$	6	0.0057	1	1
Br_2V	6	0.0022	1	1
ClNZr	6	0.0043	1	1
Cl_2Fe	6	0.0084	1	1
Ba_2N	6	2.9017	1	1
Se_2Ti	6	0.0095	1	1
Br_2Ti	6	0.0051	1	1
Te_2Zr	6	0.4994	1	1
$AsSe_2$	6	0.0009	1	1
CdO_2	6	0.0078	1	1
BrNZr	6	0.0023	1	1
$NbSe_2$	6	0.0001	1	1
CoI_2	6	0.4597	1	1
Br_2Cr	6	0.0054	1	1
Cl_2Zr	6	0.008	1	1
$FeSe_2$	6	0.1118	1	1
Br_2Mg	6	0.4656	1	1

Table showing the first 50 lattice matching combinations with the smallest number of atoms in the supercell.

Lattice matching - minimal strain

Formula	N° atoms	strain	cell size 1	cell size 2
CrS_2	390	0.0	57	73
$IrTe_2$	435	0.0001	81	64
NaPSn	339	0.0001	64	49
$NbSe_2$	6	0.0001	1	1
$HNiO_2$	304	0.0001	36	49
Br_2Gd_2	291	0.0001	49	36
Gd_2I_2	208	0.0001	36	25
$F_2Se_2Y_2$	297	0.0002	49	25
Cl_2Gd_2	447	0.0002	73	57
CI_2Y_2	705	0.0002	100	81
$As_2Fe_2Li_2$	483	0.0002	65	48
Cu_2I_2	208	0.0002	36	25
Br_2Gd_2Ge	233	0.0002	36	25
As_2Sn_2	291	0.0002	49	36
$Br_2H_2Yb_2$	483	0.0002	65	48
I_2Ti	543	0.0002	100	81
Pt_2Te_2	388	0.0003	64	49
Ga_2Se_2	499	0.0003	81	64
Bi_2Se_3	233	0.0003	36	25
BiBrTe	123	0.0003	25	16
Se_2Sn	543	0.0004	100	81
ReS_2	492	0.0004	73	91
FeI_2	492	0.0004	91	73
Br_2La	183	0.0004	36	25
Br_2Er_2	291	0.0004	49	36
Fe_2SeTe	387	0.0004	65	48
Ba_2Pt	75	0.0005	16	9
$GeNi_3Te_2$	486	0.0005	64	49
Ba_2Cu_2	439	0.0005	81	49
Cl_2Ni	6	0.0005	1	1
Br_2Mg	492	0.0005	91	73
CoI_2	543	0.0006	100	81
NSr_2	543	0.0006	100	81
In_2S_3	563	0.0006	81	64
$F_2Ho_2Se_2$	429	0.0006	57	43
Cu_2Sr_2	139	0.0007	25	16
I_2Yb	390	0.0007	81	49
$GeTe_2$	492	0.0007	91	73
Ag_2	66	0.0007	16	9
S_2Zn_2	447	0.0007	73	57
S_2	371	0.0007	81	64
$Er_2F_2Se_2$	486	0.0007	64	49
I_2Mg	183	0.0007	36	25
$AgCuTe_2$	553	0.0008	91	70
CrI_2	492	0.0008	91	73
Ni_2Se_2	678	0.0008	118	81
GeTe	371	0.0008	81	64
I_2Ni	492	0.0009	91	73
Cl_2Tb_2	447	0.0009	73	57
NS_2Ta	357	0.0009	43	57

Table showing the first 50 lattice matching combinations with the smallest strain needed to build the supercell.

$TaTe_2$ (P-3m1)

Structural and electronic properties

**Formula**	$TaTe_2$
**Spacegroup**	P-3m1
**Prototype**	CdI2
**Parent 3D**	$TaTe_2$
**Source DB**	MPDS
**DB ID**	S535129
**DF2-C09 Binding energy [meV/ Å^2]**	26.61
**RVV10 Binding energy [meV/ Å^2]**	N/A
**Band gap (PBE) [eV]**	0.0

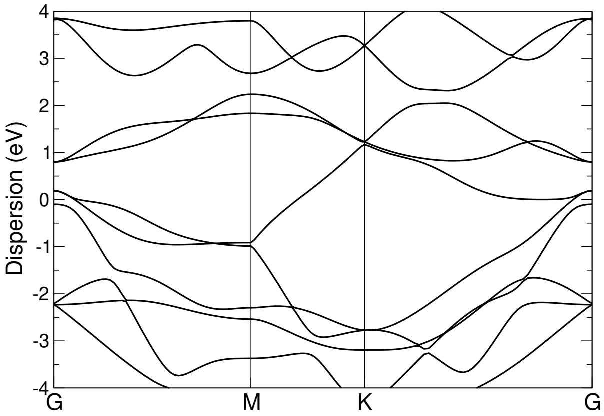

Band structure: Electronic band structure of $TaTe_2$ (P-3m1) in a representative window around the Fermi energy and along a high-symmetry path.

Optimized crystal structure

Structural parameters: relaxed cell (top) and atomic positions (bottom) of $TaTe_2$ (P-3m1) in Cartesian coordinates.

	x [Å]	y [Å]	z [Å]
$\mathbf{a_1}$	1.85119922	−3.20637111	0.00000000
$\mathbf{a_2}$	1.85119922	3.20637111	0.00000000
$\mathbf{a_3}$	0.00000000	0.00000000	19.27937424

		x [Å]	y [Å]	z [Å]
●	Te	1.85119922	-1.06879037	-1.82584782
●	Ta	0.00000000	0.00000000	0.00000000
●	Te	1.85119922	1.06879037	1.82584782

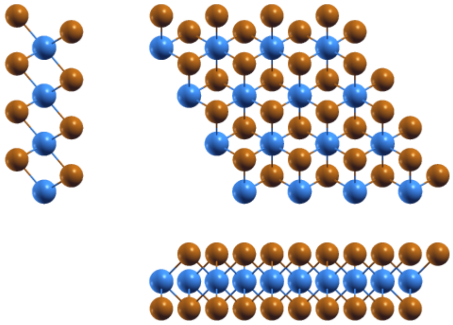

Orthographic projections: views of $TaTe_2$ (P-3m1) as seen from the x axis (left), the y axis (bottom) and the z axis (center).

Lattice matching - minimal size

Formula	N° atoms	strain	cell size 1	cell size 2
Tl	4	0.1149	1	1
Tl	4	0.2653	1	1
InSe	5	0.4995	1	1
HgO	5	0.1209	1	1
LiO	5	0.2477	1	1
P_2	5	0.258	1	1
Mg_2	5	0.4313	1	1
Sb_2	5	0.4583	1	1
Sm	5	0.2213	1	2
I_2Mg	6	0.4726	1	1
Cl_2Mn	6	0.2737	1	1
$AgTe_2$	6	0.1162	1	1
PSn_2	6	0.0032	1	1
$MoSe_2$	6	0.2643	1	1
Br_2Zn	6	0.0076	1	1
HfS_2	6	0.0092	1	1
$AsSn_2$	6	0.0006	1	1
S_2Zr	6	0.0039	1	1
Br_2La	6	0.4735	1	1
Cl_2Ti	6	0.2582	1	1
BrCdI	6	0.4807	1	1
Mg_3	6	0.1125	1	1
Te_2Ti	6	0.0079	1	1
I_2Zn	6	0.4503	1	1
$RhTe_2$	6	0.0034	1	1
Bi_2Pd	6	0.1267	1	1
GeI_2	6	0.4679	1	1
NbS_2	6	0.2697	1	1
$CoTe_2$	6	0.0089	1	1
S_2Ta	6	0.2685	1	1
Se_2V	6	0.2666	1	1
AsKSn	6	0.4856	1	1
$PbTe_2$	6	0.4781	1	1
S_2Sn	6	0.0037	1	1
$SnTe_2$	6	0.4622	1	1
$PtSe_2$	6	0.0012	1	1
GeS_2	6	0.1486	1	1
Br_2Ni	6	0.0073	1	1
$NbTe_2$	6	0.0041	1	1
Se_2Yb	6	0.4685	1	1
Cl_2Mg	6	0.0072	1	1
$BiTe_2$	6	0.4692	1	1
F_2Na	6	0.0096	1	1
$HfSe_2$	6	0.0079	1	1
Se_2W	6	0.2645	1	1
CdClHO	7	0.0006	1	1
Br_2Pr_2	7	0.0088	1	1
CdClHO	7	0.0026	1	1
$AsLi_3$	7	0.5003	1	1
Cl_2Y_2	7	0.0063	1	1

Table showing the first 50 lattice matching combinations with the smallest number of atoms in the supercell.

Lattice matching - minimal strain

Formula	N° atoms	strain	cell size 1	cell size 2
$MoSe_2$	492	0.0	73	91
GeI_2	300	0.0	57	43
$AsCuLi_2$	565	0.0001	91	73
$I_2Pr_2Si_2$	711	0.0001	91	73
Cu_4Te_2	363	0.0001	49	36
Ba_2Pt	123	0.0001	25	16
Se_2W	492	0.0001	73	91
Ag_2	107	0.0001	25	16
As_2CeLi_2	386	0.0002	57	43
Bi_2Se_2Te	437	0.0002	64	49
$Eu_2H_2I_2$	483	0.0002	65	48
Cl_2V	300	0.0002	43	57
Ga_2Te_2	565	0.0002	91	73
HN_3OZn	678	0.0002	64	81
Sb_2SeTe_2	437	0.0002	64	49
PbTe	257	0.0002	57	43
Ba_2Ni_3	638	0.0002	91	73
$Cu_2Rb_2Te_2$	411	0.0002	65	36
In_2Te_3	437	0.0002	64	49
$LiMnTe_2$	343	0.0004	57	43
H_2MgO_2	353	0.0004	36	49
Br_2Gd_2Ge	563	0.0004	81	64
Cu_2I_2	499	0.0004	81	64
I_2La_2	447	0.0004	73	57
$F_2I_2Yb_2$	483	0.0004	65	48
GeI_2	492	0.0004	91	73
CKN	336	0.0004	72	40
$I_2O_2Sm_2$	483	0.0004	65	48
$SnTe_2$	543	0.0004	100	81
$Al_2Cl_2O_2$	438	0.0004	48	49
C_2	30	0.0004	4	9
$Ga_2Ge_2Te_2$	786	0.0005	100	81
Bi_2In_2	339	0.0005	65	36
Br_2HLa	565	0.0005	91	73
$Hf_2I_2N_2$	9	0.0005	1	1
CdClHO	7	0.0006	1	1
Li_2P_2Pr	705	0.0006	100	81
$AsSn_2$	6	0.0006	1	1
ClH_3O	488	0.0006	81	49
S_2Ta	543	0.0006	81	100
GeI_2Y_2	437	0.0006	64	49
Se_2Yb	492	0.0006	91	73
Se_2Sn_2	525	0.0006	95	60
$Ba_2Ge_2Mn_2$	483	0.0007	65	48
Bi_2STe_2	386	0.0007	57	43
Gd_2I_2	499	0.0007	81	64
Sb_2Te_3	327	0.0007	49	36
BH_4Li	786	0.0007	100	81
Tl	310	0.0007	73	91
Se_2V	543	0.0007	81	100

Table showing the first 50 lattice matching combinations with the smallest strain needed to build the supercell.

$Tb_2C_2Br_2$ (C2/m)

Structural and electronic properties

**Formula**	$Tb_2C_2Br_2$
**Spacegroup**	C2/m
**Prototype**	Gd2C2Br2
**Parent 3D**	$C_2Br_2Tb_2$
**Source DB**	MPDS
**DB ID**	S1702973
**DF2-C09 Binding energy [meV/ Å^2]**	12.01
**RVV10 Binding energy [meV/ Å^2]**	N/A
**Band gap (PBE) [eV]**	0.0

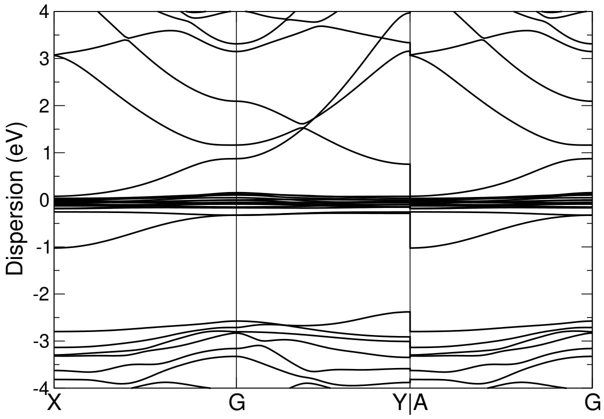

Band structure: Electronic band structure of $Tb_2C_2Br_2$ (C2/m) in a representative window around the Fermi energy and along a high-symmetry path.

Optimized crystal structure

Structural parameters: relaxed cell (top) and atomic positions (bottom) of $Tb_2C_2Br_2$ (C2/m) in Cartesian coordinates.

	x [Å]	y [Å]	z [Å]
$\mathbf{a_1}$	0.00000225	3.87973384	0.00000000
$\mathbf{a_2}$	3.57820241	1.93986907	0.00000000
$\mathbf{a_3}$	0.00000000	0.00000000	25.15726060

	x [Å]	y [Å]	z [Å]
Tb	0.87800044	0.96990522	-14.07534793
C	2.34155262	2.90974449	-12.92910668
Br	3.26598287	0.96991192	-15.88997515
Tb	2.70020196	0.96996384	-11.08191267
C	1.23665204	2.90985841	-12.22815392
Br	0.31221953	0.96995715	-9.26728545

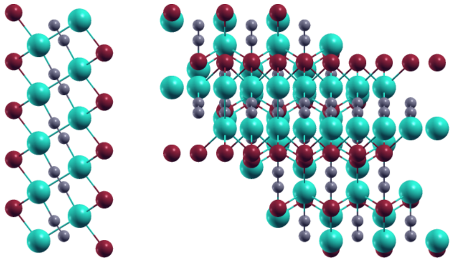

Orthographic projections: views of $Tb_2C_2Br_2$ (C2/m) as seen from the x axis (left), the y axis (bottom) and the z axis (center).

Lattice matching - minimal size

Formula	N° atoms	strain	cell size 1	cell size 2
In	7	0.1887	1	1
HgO	8	0.3905	1	1
AgTl	8	0.175	1	1
CaCl	8	0.1208	1	1
Sm	8	0.1632	1	2
Br_2Cu	9	0.2819	1	1
ReS_2	9	0.1819	1	1
Cl_2Zn	9	0.1311	1	1
GeS_2	9	0.1139	1	1
$MnSe_2$	9	0.1208	1	1
CNNa	9	0.217	1	1
F_2Ni	9	0.128	1	1
O_2Pt	9	0.1874	1	1
Cu_2I_2	10	0.1782	1	1
Cl_2OOs	10	0.1277	1	1
Cu_2O_2	10	0.9757	1	1
$AgCuTe_2$	10	0.1556	1	1
O_2Sn_2	10	0.1521	1	1
Au_2Br_2	10	0.2145	1	1
Cl_2OV	10	0.1134	1	1
Fe_2Se_2	10	0.1276	1	1
Cl_2ORu	10	0.124	1	1
As_2Co_2	10	0.1241	1	1
Cu_2Te_2	10	0.1332	1	1
O_2Pb_2	10	0.1741	1	1
Ge_2S_2	10	0.1832	1	1
As_4	10	0.2216	1	1
Br_2OV	10	0.1177	1	1
Au_2Se_2	10	1.5739	1	1
Fe_2S_2	10	0.1195	1	1
LiO	10	0.2936	1	2
BN	10	0.149	1	2
Au_2I_2	10	0.2523	1	1
Co_2S_2	10	0.1212	1	1
As_2Fe_2	10	0.1144	1	1
Ge_2Se_2	10	0.1872	1	1
Cu_2Se_2	10	0.1285	1	1
Bi_2O_2	10	0.1757	1	1
C_2	10	0.144	1	2
Co_2Se_2	10	0.1251	1	1
Ca_2Cl_2	10	0.1236	1	1
Hf_3Te_2	11	0.1303	1	1
H_2Na_2Pd	11	0.1135	1	1
FKO_2Se	11	2.251	1	1
F_4Nb	11	0.1829	1	1
NaO_4	11	0.6419	1	1
$Br_2In_2O_2$	12	0.1596	1	1
CrS_2	12	0.2517	1	2
$Br_2Ho_2S_2$	12	0.2897	1	1
S_2V	12	0.2807	1	2

Table showing the first 50 lattice matching combinations with the smallest number of atoms in the supercell.

Lattice matching - minimal strain

Formula	N° atoms	strain	cell size 1	cell size 2
I_2Tm	168	0.0004	20	16
Te_2V	156	0.0004	16	20
$C_2Br_2Gd_2$	12	0.0004	1	1
GeI_2La_2	200	0.0004	20	16
CoO_2	759	0.0004	63	127
FHOZn	526	0.0005	43	67
$CrSe_2$	459	0.0005	43	67
$Br_2La_2O_2$	948	0.0005	86	72
Cl_2Cu	654	0.0005	70	78
$Eu_2F_2I_2$	948	0.0005	86	72
Bi_2O_2	804	0.0005	86	72
$Cl_2Hf_2N_2$	216	0.0006	16	20
OTl_2	156	0.0006	16	20
NaO_4	613	0.0007	68	41
CdClO	156	0.0007	16	20
Cl_2ORu	194	0.0008	19	20
Br_2PY_2	638	0.0008	58	58
NiO_2	759	0.0008	63	127
Br_2Cd	522	0.0009	58	58
As_2Li_2Nd	962	0.0009	92	82
O_2Zn	594	0.0009	53	92
$AgNO_3$	613	0.0009	68	41
$Br_2Hf_2N_2$	198	0.0009	17	16
F_2Na	768	0.0009	82	92
$Cu_2Se_2Tl_2$	858	0.001	78	65
GeI_2Y_2	962	0.001	92	82
As_2Li_2Pr	962	0.001	92	82
InSe	716	0.001	92	82
CaClHO	860	0.0011	82	92
O_2Pb_2	804	0.0011	86	72
$F_2Ho_2Se_2$	696	0.0011	58	58
Cl_2V	459	0.0011	43	67
$Ga_2I_2Tb_2$	708	0.0012	63	55
$AsLi_3$	880	0.0012	92	82
ReS_2	450	0.0012	41	68
Bi_2O_2	728	0.0012	78	65
HgI_2	930	0.0012	115	80
Bi_2Se_2Te	962	0.0012	92	82
CaI_2	168	0.0013	20	16
$F_2I_2Sm_2$	948	0.0013	86	72
Gd_2GeI_2	653	0.0013	63	55
$I_2Se_2Tb_2$	738	0.0013	78	45
$F_2Lu_2Se_2$	942	0.0013	80	77
Cl_2OV	718	0.0013	67	79
C_4Ca_2	744	0.0013	68	56
$Ga_2Gd_2I_2$	708	0.0013	63	55
CNNa	144	0.0014	17	14
I_2Yb	168	0.0014	20	16
Br_2Pr_2	860	0.0014	82	92
F_4Sn	978	0.0014	98	78

Table showing the first 50 lattice matching combinations with the smallest strain needed to build the supercell.

$Tb_2Ga_2I_2$ (P-3m1)

Structural and electronic properties

**Formula**	$Tb_2Ga_2I_2$
**Spacegroup**	P-3m1
**Prototype**	SmSI
**Parent 3D**	$Ga_2I_2Tb_2$
**Source DB**	MPDS
**DB ID**	S1623209
**DF2-C09 Binding energy [meV/ Å^2]**	13.0
**RVV10 Binding energy [meV/ Å^2]**	N/A
**Band gap (PBE) [eV]**	0.0

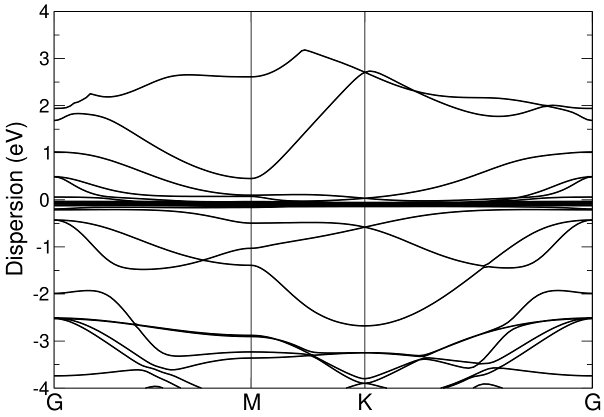

Band structure: Electronic band structure of $Tb_2Ga_2I_2$ (P-3m1) in a representative window around the Fermi energy and along a high-symmetry path.

Optimized crystal structure

Structural parameters: relaxed cell (top) and atomic positions (bottom) of $Tb_2Ga_2I_2$ (P-3m1) in Cartesian coordinates.

	x [Å]	y [Å]	z [Å]
$\mathbf{a_1}$	2.14546165	−3.71604858	0.00000000
$\mathbf{a_2}$	2.14546165	3.71604858	0.00000000
$\mathbf{a_3}$	0.00000000	0.00000000	27.99943990

	x [Å]	y [Å]	z [Å]
Tb	0.00000000	0.00000000	2.10309482
Ga	2.14546165	1.23868286	0.23780306
I	2.14546165	-1.23868286	4.06547378
Tb	0.00000000	0.00000000	-2.10309482
Ga	2.14546165	-1.23868286	-0.23780306
I	2.14546165	1.23868286	-4.06547378

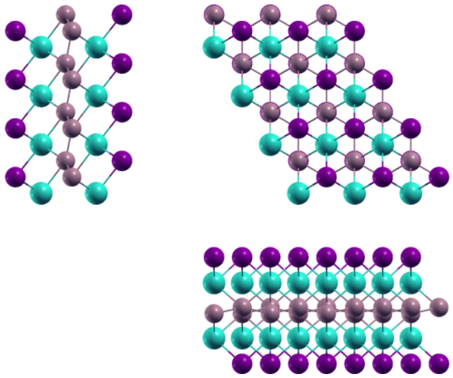

Orthographic projections: views of $Tb_2Ga_2I_2$ (P-3m1) as seen from the x axis (left), the y axis (bottom) and the z axis (center).

Lattice matching - minimal size

Formula	N° atoms	strain	cell size 1	cell size 2
InSe	8	0.005	1	1
AsSb	8	0.2644	1	1
Bi_2	8	0.0007	1	1
GeTe	8	0.2732	1	1
AgTl	8	0.1476	1	1
S_2	8	0.2755	1	1
PbTe	8	0.0033	1	1
CaCl	8	0.1116	1	1
$IrTe_2$	9	0.2745	1	1
$CdCl_2$	9	0.2716	1	1
CdI_2	9	0.0016	1	1
Br_2Zn	9	0.2504	1	1
Br_2Ca	9	0.0004	1	1
$InSe_2$	9	0.2722	1	1
$GeTe_2$	9	0.2702	1	1
$SiTe_2$	9	0.2559	1	1
I_2Pr	9	0.1325	1	1
I_2Mn	9	0.2718	1	1
NSr_2	9	0.2667	1	1
PbS_2	9	0.2608	1	1
BiClTe	9	0.0012	1	1
Cl_2Zn	9	0.1176	1	1
FeI_2	9	0.2686	1	1
I_2Ni	9	0.2704	1	1
CrI_2	9	0.2679	1	1
BaF_2	9	0.0087	1	1
BiBrTe	9	0.0043	1	1
I_2Nd	9	0.1333	1	1
$NiTe_2$	9	0.2551	1	1
Cl_2Cu	9	0.0692	1	1
I_2V	9	0.2578	1	1
GeI_2	9	0.003	1	1
Se_2Zr	9	0.2565	1	1
STl_2	9	0.0078	1	1
CoI_2	9	0.265	1	1
GeS_2	9	0.4214	1	1
$MnSe_2$	9	0.1115	1	1
CeI_2	9	0.132	1	1
Br_2Mg	9	0.2684	1	1
I_2Ti	9	0.2655	1	1
GdI_2	9	0.0073	1	1
F_2Ni	9	0.1157	1	1
I_2La	9	0.137	1	1
F_2Na	9	1.6397	1	1
CdI_2	9	0.002	1	1
Se_2Sn	9	0.2664	1	1
F_2Zn	9	0.1297	1	1
I_2Pr	9	0.0011	1	1
H_2Si_2	10	0.2726	1	1
Bi_2Te_2	10	0.4689	1	1

Table showing the first 50 lattice matching combinations with the smallest number of atoms in the supercell.

Lattice matching - minimal strain

Formula	N° atoms	strain	cell size 1	cell size 2
N_2Re	51	0.0	4	9
$Cl_2O_2Y_2$	870	0.0	64	81
AsSb	620	0.0001	73	91
PSn_2	363	0.0001	36	49
CdClO	258	0.0001	25	36
NbS_2	405	0.0001	37	61
S_2Ta	537	0.0001	49	81
CBr_2Y_2	614	0.0002	49	64
OTl_2	258	0.0002	25	36
CrS_2	297	0.0003	25	49
$NiTe_2$	561	0.0003	57	73
$Cl_2Hf_2N_2$	366	0.0003	25	36
NS_2Ta	694	0.0003	49	100
$RhTe_2$	429	0.0003	43	57
$Ga_2Gd_2I_2$	12	0.0003	1	1
CrI_2	786	0.0003	81	100
ClNZr	171	0.0003	16	25
$SiTe_2$	561	0.0003	57	73
$Au_2K_2Se_2$	354	0.0004	45	14
Br_2Ca	9	0.0004	1	1
$Cl_2H_2Zr_2$	588	0.0004	37	61
Cl_2Y_2	550	0.0004	49	64
$F_2Se_2Y_2$	780	0.0004	73	57
Ga_2Se_2	708	0.0005	64	81
NaO_4	341	0.0005	36	25
CdClHO	486	0.0005	43	57
CoI_2	711	0.0005	73	91
Se_4TiZr	726	0.0005	72	49
Cl_2V	102	0.0005	9	16
Br_2Mg	786	0.0005	81	100
Te_2V	258	0.0006	25	36
S_2Sn	363	0.0006	36	49
$Cl_2Er_2O_2$	780	0.0006	57	73
NSr_2	786	0.0006	81	100
H_2Li_2Pt	694	0.0006	49	80
$Cl_2Rh_2Te_2$	954	0.0006	96	63
PTe_2Zr_2	893	0.0006	73	91
FeI_2	786	0.0007	81	100
$CrSe_2$	102	0.0007	9	16
Bi_2	8	0.0007	1	1
N_2W	471	0.0007	39	79
C	49	0.0007	4	25
CCl_2Gd_2	614	0.0007	49	64
S_2Ta	405	0.0007	37	61
$Cl_2Ho_2O_2$	870	0.0008	64	81
Se_2Zr	561	0.0008	57	73
CdH_2O_2	330	0.0008	25	36
Bi_2Pd	840	0.0008	81	118
Bi_2Te_2	838	0.0008	91	73
Br_2Y_2	708	0.0008	64	81

Table showing the first 50 lattice matching combinations with the smallest strain needed to build the supercell.

$Tb_2O_2Br_2$ (P4/nmm)

Structural and electronic properties

**Formula**	$Tb_2O_2Br_2$
**Spacegroup**	P4/nmm
**Prototype**	PbClF
**Parent 3D**	$Br_2O_2Tb_2$
**Source DB**	MPDS
**DB ID**	S1903364
**DF2-C09 Binding energy [meV/ Å^2]**	16.02
**RVV10 Binding energy [meV/ Å^2]**	N/A
**Band gap (PBE) [eV]**	0.0

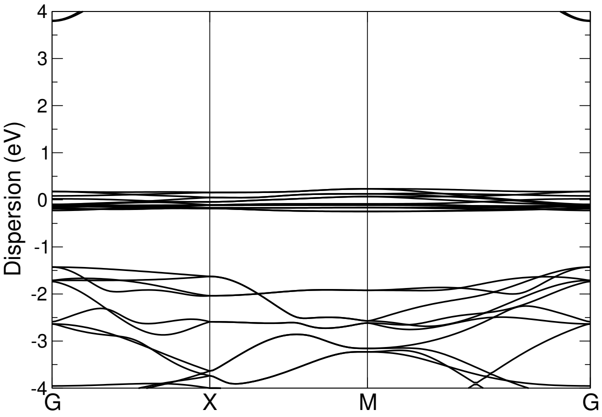

Band structure: Electronic band structure of $Tb_2O_2Br_2$ (P4/nmm) in a representative window around the Fermi energy and along a high-symmetry path.

Optimized crystal structure

Structural parameters: relaxed cell (top) and atomic positions (bottom) of $Tb_2O_2Br_2$ (P4/nmm) in Cartesian coordinates.

	x [Å]	y [Å]	z [Å]
$\mathbf{a_1}$	3.85914820	0.00000000	0.00000000
$\mathbf{a_2}$	0.00000000	3.85914820	0.00000000
$\mathbf{a_3}$	0.00000000	0.00000000	22.89107722

		x [Å]	y [Å]	z [Å]
●	Tb	0.96478705	-0.96478705	1.15560526
●	Br	-0.96478705	-2.89436115	2.81055391
●	Tb	-0.96478705	-2.89436115	-1.15560526
●	Br	0.96478705	-0.96478705	-2.81055391
•	O	-0.96478705	-0.96478705	0.00000000
•	O	0.96478705	-2.89436115	0.00000000

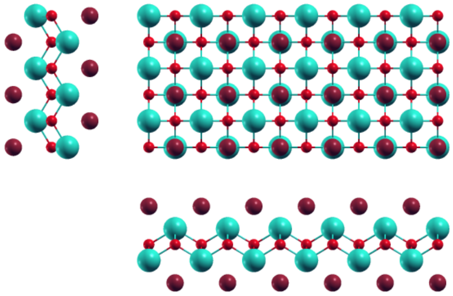

Orthographic projections: views of $Tb_2O_2Br_2$ (P4/nmm) as seen from the x axis (left), the y axis (bottom) and the z axis (center).

Lattice matching - minimal size

Formula	N° atoms	strain	cell size 1	cell size 2
K	7	0.3894	1	1
K	7	0.1913	1	1
In	7	0.1108	1	1
InSe	8	0.1509	1	1
Bi_2	8	0.1567	1	1
Ag_2	8	0.7549	1	1
PbTe	8	0.1527	1	1
Sb_2	8	0.1368	1	1
I_2Mg	9	0.1415	1	1
S_2V	9	0.1101	1	1
MoS_2	9	0.1099	1	1
CdI_2	9	0.1544	1	1
Nd	9	0.7778	1	3
Ba_2Pt	9	0.7539	1	1
Br_2Ca	9	0.1556	1	1
CaI_2	9	0.1797	1	1
I_2Pr	9	0.0058	1	1
S_2Zr	9	0.109	1	1
Br_2La	9	0.1418	1	1
Br_2Cu	9	0.1082	1	1
Ca_2Si	9	0.7711	1	1
I_2Yb	9	0.1766	1	1
BiClTe	9	0.1547	1	1
$AuTe_2$	9	0.1308	1	1
BrCdI	9	0.1443	1	1
I_2Zn	9	0.1342	1	1
BaF_2	9	0.1473	1	1
BiBrTe	9	0.1605	1	1
S_2W	9	0.1099	1	1
Bi_2Pd	9	0.5677	1	1
GeI_2	9	0.1399	1	1
AsKSn	9	0.146	1	1
$PbTe_2$	9	0.1434	1	1
I_2Nd	9	0.0068	1	1
Cl_2Cu	9	0.0999	1	1
I_2Tm	9	0.1783	1	1
S_2Sn	9	0.1091	1	1
$SnTe_2$	9	0.1381	1	1
Cl_2V	9	0.1091	1	1
GeI_2	9	0.1529	1	1
STl_2	9	0.1482	1	1
BiITe	9	0.1676	1	1
DyI_2	9	0.1832	1	1
CeI_2	9	0.005	1	1
$NbTe_2$	9	0.1089	1	1
Se_2Yb	9	0.1401	1	1
MoS_2	9	0.1099	1	1
$BiTe_2$	9	0.1404	1	1
GdI_2	9	0.1635	1	1
$CrSe_2$	9	0.1093	1	1

Table showing the first 50 lattice matching combinations with the smallest number of atoms in the supercell.

Lattice matching - minimal strain

Formula	N° atoms	strain	cell size 1	cell size 2
$Cu_2Rb_2Te_2$	366	0.0	36	25
Se_2Ta_4	690	0.0001	50	65
Tl	307	0.0001	41	61
Sn	655	0.0002	85	145
Te_2Zn	483	0.0002	48	65
$HfLiS_2$	548	0.0003	48	65
$MoTe_2$	483	0.0003	48	65
Mg_3	942	0.0003	89	136
Bi_2In_2	316	0.0004	36	25
F_4Pb	629	0.0004	64	49
Br_2Cu_2	10	0.0004	1	1
Te_2W	483	0.0004	48	65
In	445	0.0004	58	97
$HNiO_2$	276	0.0004	20	39
Bi_2Pd	363	0.0005	36	49
ClNZr	840	0.0006	81	118
O_4PSn	876	0.0007	85	61
CdH_2O_2	613	0.0008	48	65
O_2Sn_2	10	0.0009	1	1
$Cu_2Na_2Te_2$	870	0.0009	81	64
Ho_2S_2	496	0.0009	50	49
$Br_2F_2Yb_2$	12	0.001	1	1
PtS_2	483	0.001	48	65
Se_2Ta_4	678	0.001	49	64
P_2	726	0.001	79	126
Cl_2Ti	852	0.0011	79	126
LiO_2	510	0.0012	49	72
$Cu_2K_2Te_2$	876	0.0012	85	61
P_4	356	0.0012	36	35
Cu_2O_2	496	0.0013	50	49
Pb_2Se_2	806	0.0013	89	68
FKO_2Se	700	0.0013	80	44
$Cl_2S_2Tl_2$	780	0.0014	81	49
$Br_2Ca_2F_2$	12	0.0014	1	1
Cl_2Zr_2	958	0.0014	81	118
H_2MgO_2	929	0.0014	64	109
Cu_2S_2	10	0.0014	1	1
Ba_2Cu_2	974	0.0014	103	89
I_2Yb	885	0.0015	103	89
$As_2Cd_2K_2$	876	0.0015	85	61
$Cl_2H_2Sc_2$	750	0.0015	52	73
Cl_4Cu_2	534	0.0015	64	25
$Br_2O_2Y_2$	12	0.0015	1	1
I_3Sn	70	0.0015	9	4
Se_2Ta	531	0.0015	52	73
Se_2Sn_2	742	0.0015	81	64
La_2S_2	818	0.0015	89	71
S_2V	711	0.0015	64	109
$Br_2Dy_2O_2$	12	0.0015	1	1
Cl_4Mg_2	438	0.0015	55	18

Table showing the first 50 lattice matching combinations with the smallest strain needed to build the supercell.

$Tb_2S_2I_2$ (Pmm2)

Structural and electronic properties

**Formula**	$Tb_2S_2I_2$
**Spacegroup**	Pmm2
**Prototype**	FeOCl
**Parent 3D**	$I_2S_2Tb_2$
**Source DB**	MPDS
**DB ID**	S1937394
**DF2-C09 Binding energy [meV/ Å^2]**	10.4
**RVV10 Binding energy [meV/ Å^2]**	N/A
**Band gap (PBE) [eV]**	0.0

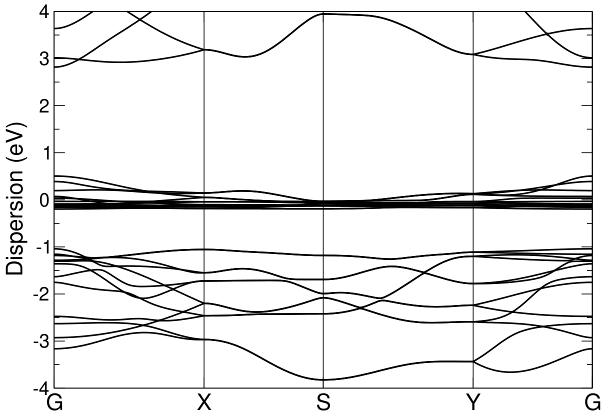

Band structure: Electronic band structure of $Tb_2S_2I_2$ (Pmm2) in a representative window around the Fermi energy and along a high-symmetry path.

Optimized crystal structure

Structural parameters: relaxed cell (top) and atomic positions (bottom) of $Tb_2S_2I_2$ (Pmm2) in Cartesian coordinates.

	x [Å]	y [Å]	z [Å]
$\mathbf{a_1}$	4.23035394	0.00000000	0.00000000
$\mathbf{a_2}$	0.00000000	5.32295521	0.00000000
$\mathbf{a_3}$	0.00000000	0.00000000	25.21752237

		x [Å]	y [Å]	z [Å]
●	Tb	1.05756352	-1.33073880	-1.14497343
●	S	1.05768103	-3.99221641	-0.60098900
●	I	-1.05752336	-1.33073880	-3.36949593
●	Tb	-1.05756352	-3.99221641	1.14497343
●	S	-1.05768103	-1.33073880	0.60098900
●	I	1.05752336	-3.99221641	3.36949593

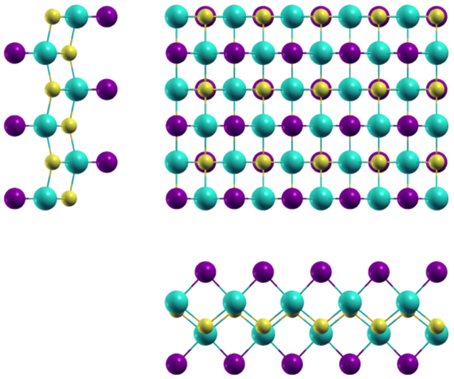

Orthographic projections: views of $Tb_2S_2I_2$ (Pmm2) as seen from the x axis (left), the y axis (bottom) and the z axis (center).

Lattice matching - minimal size

Formula	N° atoms	strain	cell size 1	cell size 2
Ba_2Hg	9	0.2509	1	1
CNRb	9	0.2138	1	1
CKN	9	0.0128	1	1
Ba_2Cd	9	0.2545	1	1
Bi_2In_2	10	0.3266	1	1
In_2Se_2	10	0.0968	1	1
$AgNO_2$	10	0.3393	1	1
$AgCuTe_2$	10	1.7758	1	1
Au_2Br_2	10	0.06	1	1
O_2Pb_2	10	0.6978	1	1
As_4	10	0.2895	1	1
Br_3Cs	10	0.3694	1	1
S_2	10	1.9375	1	2
Au_2I_2	10	0.7849	1	1
$AgClO_2$	10	0.1168	1	1
PbS_2Sn	10	0.7449	1	1
$SbSe_2Tl$	10	0.1728	1	1
Ag_2I_2	10	0.3003	1	1
Br_2CsF	10	0.323	1	1
Gd	10	0.0305	1	4
Sn_2Te_2	10	0.3143	1	1
F_4Sn	11	0.2499	1	1
Hg_3N_2	11	0.3987	1	1
FKO_2Se	11	0.5307	1	1
$ClKO_3$	11	0.3771	1	1
$CuGeO_3$	11	0.1396	1	1
Cl_4Mn	11	0.0534	1	1
$Ba_2H_2I_2$	12	0.3196	1	1
CrS_2	12	0.1373	1	2
$Br_2Ho_2S_2$	12	0.0119	1	1
$I_2Lu_2Se_2$	12	0.5175	1	1
$Ho_2I_2S_2$	12	0.003	1	1
$Eu_2F_2I_2$	12	0.7005	1	1
AlH_4Na	12	0.054	1	1
$F_2I_2Sm_2$	12	0.6971	1	1
Br_2Ca_3Si	12	0.2513	1	1
$I_2Se_2Tb_2$	12	0.0201	1	1
$Br_2S_2Y_2$	12	0.0144	1	1
$Gd_2I_2S_2$	12	0.0022	1	1
$C_2Br_2Gd_2$	12	0.108	1	1
N_2W	12	0.1345	1	2
$I_2S_2Yb_2$	12	0.0077	1	1
$Cu_2Rb_2Te_2$	12	0.326	1	1
$Br_2Dy_2S_2$	12	0.0099	1	1
$Er_2I_2Se_2$	12	0.0176	1	1
$Cl_2Ga_2Te_2$	12	0.496	1	1
$I_2Se_2Tm_2$	12	0.017	1	1
$Ag_2K_2Te_2$	12	0.0559	1	1
$Br_2Ga_2Te_2$	12	0.5045	1	1
Ca_4Cu_2	12	0.041	1	1

Table showing the first 50 lattice matching combinations with the smallest number of atoms in the supercell.

Lattice matching - minimal strain

Formula	N° atoms	strain	cell size 1	cell size 2
AsSb	476	0.0003	50	88
Ca_2Cl_2	550	0.0003	45	70
As_2Ru_2	550	0.0004	45	70
K_2PtSe_2	158	0.0005	18	10
Fe_2Te_2	550	0.0005	45	70
CoI_2	564	0.0005	50	88
$Br_2O_2Tm_2$	690	0.0006	45	70
PTe_2Zr_2	740	0.0007	50	88
Cu_2Se_2	770	0.0008	61	101
$Br_2O_2Yb_2$	690	0.0008	45	70
In	469	0.0009	55	139
$Br_2Cr_2S_2$	942	0.0009	63	94
$I_2S_2Tl_2$	972	0.0009	61	101
Sn	300	0.0009	35	90
PSn_2	705	0.0009	60	115
CdClHO	572	0.001	42	80
$Hf_2I_2N_2$	732	0.001	42	80
$Br_2N_2Zr_2$	630	0.001	42	63
$SbSe_2Tl$	974	0.001	97	98
C_2	408	0.001	28	120
$AgClO_4$	930	0.001	79	76
$Br_2Cr_2O_2$	588	0.0011	35	63
Cl_2OOs	532	0.0011	42	70
As_4	624	0.0011	56	72
$Ge_2Hf_2Te_2$	846	0.0011	54	87
AlH_4Na	954	0.0011	79	80
$As_2Co_2Li_2$	846	0.0011	54	87
$I_2N_2Zr_2$	822	0.0012	56	81
F_2Ni	669	0.0012	61	101
$Si_2Te_2Zr_2$	972	0.0012	61	101
$Ag_2K_2Te_2$	954	0.0012	80	79
Bi_2Te_2	494	0.0013	47	53
$TaTe_2$	492	0.0013	42	80
Cu_2Te_2	672	0.0013	54	87
S_2Sn	705	0.0014	60	115
$Dy_2I_2S_2$	12	0.0014	1	1
$Br_2Er_2O_2$	732	0.0014	48	74
Se_2Sn	564	0.0014	50	88
$Br_2In_2O_2$	630	0.0015	42	63
$K_2O_2Tl_2$	738	0.0016	63	60
$AgTe_2$	114	0.0016	9	20
Cu_2F_4	54	0.0016	5	4
Cl_2Zn	534	0.0016	49	80
S_2Zr	705	0.0016	60	115
Fe_2Se_2	770	0.0016	61	101
$AgNO_3$	815	0.0016	75	73
NSr_2	564	0.0017	50	88
$Br_2Ca_2H_2$	732	0.0017	48	74
$Ca_2Cl_2H_2$	774	0.0017	49	80
$Cl_2Fe_2O_2$	366	0.0017	21	40

Table showing the first 50 lattice matching combinations with the smallest strain needed to build the supercell.

$Tb_2Se_2I_2$ (Pmm2)

Structural and electronic properties

**Formula**	$Tb_2Se_2I_2$
**Spacegroup**	Pmm2
**Prototype**	FeOCl
**Parent 3D**	$I_2Se_2Tb_2$
**Source DB**	MPDS
**DB ID**	S376106
**DF2-C09 Binding energy [meV/ Å^2]**	10.92
**RVV10 Binding energy [meV/ Å^2]**	N/A
**Band gap (PBE) [eV]**	0.0

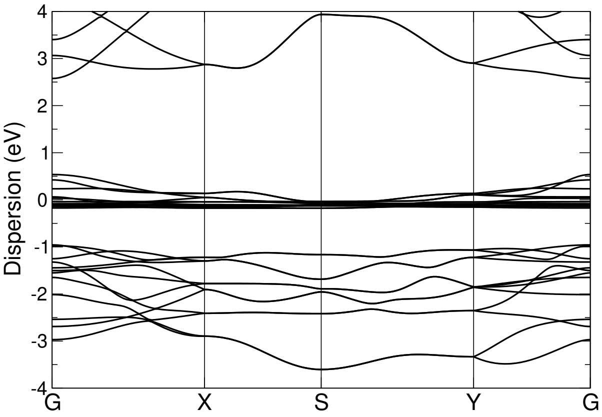

Band structure: Electronic band structure of $Tb_2Se_2I_2$ (Pmm2) in a representative window around the Fermi energy and along a high-symmetry path.

Optimized crystal structure

Structural parameters: relaxed cell (top) and atomic positions (bottom) of $Tb_2Se_2I_2$ (Pmm2) in Cartesian coordinates.

	x [Å]	y [Å]	z [Å]
$\mathbf{a_1}$	−4.30283310	0.00000000	0.00000000
$\mathbf{a_2}$	0.00000000	−5.62204844	0.00000000
$\mathbf{a_3}$	0.00000000	0.00000000	25.15461114

		x [Å]	y [Å]	z [Å]
●	Tb	-1.07581583	1.40551211	1.19242055
●	Tb	1.07581583	-1.40551211	-1.19242055
●	Se	1.07560870	1.40551211	-0.73088097
●	Se	-1.07560870	-1.40551211	0.73088097
●	I	1.07550603	1.40551211	3.37404598
●	I	-1.07550603	-1.40551211	-3.37404598

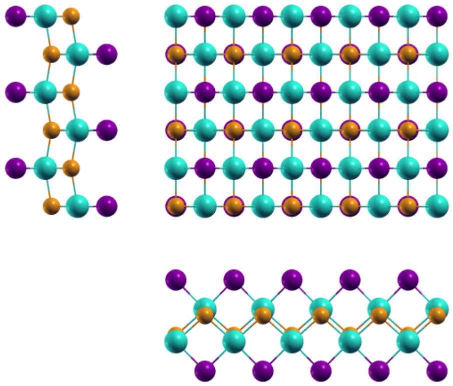

Orthographic projections: views of $Tb_2Se_2I_2$ (Pmm2) as seen from the x axis (left), the y axis (bottom) and the z axis (center).

Lattice matching - minimal size

Formula	N° atoms	strain	cell size 1	cell size 2
K	7	0.7673	1	1
Tl	8	0.143	1	2
CNNa	9	0.5708	1	1
In_2Se_2	10	0.085	1	1
Au_2Br_2	10	0.0711	1	1
Ge_2Te_2	10	0.2643	1	1
Au_2Se_2	10	0.2117	1	1
P_2	10	0.14	1	2
$SbSe_2Tl$	10	0.1706	1	1
Se_2Sn_2	10	0.2619	1	1
KNO_3	11	0.3121	1	1
Ga_2S_3	11	0.2725	1	1
S_2V	12	0.1328	1	2
MoS_2	12	0.1331	1	2
Cl_2Mn	12	0.1466	1	2
$MoSe_2$	12	0.1425	1	2
$CuTe_2$	12	0.4938	1	2
$Cu_2Na_2Te_2$	12	0.2622	1	1
Cl_2Ti	12	0.14	1	2
$K_2O_2Tl_2$	12	0.2038	1	1
$Gd_2I_2Se_2$	12	0.002	1	1
S_2W	12	0.1331	1	2
$Gd_2I_2S_2$	12	0.017	1	1
Cl_2Ni	12	0.3664	1	2
Pd_2S_4	12	0.3445	1	1
$Br_2Er_2Se_2$	12	0.0137	1	1
NbS_2	12	0.1448	1	2
Cl_2Fe	12	0.3509	1	2
S_2Ta	12	0.1443	1	2
Se_2V	12	0.1435	1	2
$I_2S_2Tb_2$	12	0.0181	1	1
$AsSe_2$	12	0.3692	1	2
$Er_2I_2Se_2$	12	0.0044	1	1
$I_2Se_2Tm_2$	12	0.0059	1	1
$SnTe_2$	12	0.6729	1	2
AuI_4Li	12	0.1135	1	1
BrNZr	12	0.3719	1	2
$NbSe_2$	12	0.3672	1	2
$TaTe_2$	12	0.5196	1	2
Cl_2Zr	12	0.3516	1	2
Se_2Ta	12	0.3674	1	2
$H_2I_2Sr_2$	12	0.2535	1	1
I_2Ti	12	0.5719	1	2
$NbSe_2$	12	0.3698	1	2
MoS_2	12	0.1332	1	2
Se_4TiZr	12	0.5104	1	1
$I_2Se_2Yb_2$	12	0.0068	1	1
$As_2Mg_2Na_2$	12	0.7662	1	1
Te_4W_2	12	0.4966	1	1
$C_2Br_2La_2$	12	0.109	1	1

Table showing the first 50 lattice matching combinations with the smallest number of atoms in the supercell.

Lattice matching - minimal strain

Formula	N° atoms	strain	cell size 1	cell size 2
$I_2Pr_2S_2$	966	0.0002	70	91
$AgCuTe_2$	118	0.0006	9	16
Ag_2	602	0.0006	70	91
$Gd_2I_2S_2$	948	0.0007	67	91
I_2Zn	486	0.0007	44	74
Br_2Ca_3Si	948	0.0008	67	91
$C_2Cl_2Y_2$	138	0.0008	8	15
$AgNO_2$	582	0.0008	49	72
Cl_2Y_2	724	0.0009	52	103
Ba_2Pt	693	0.0009	70	91
Ge_2S_2	504	0.0009	42	63
I_2Pb	693	0.001	70	91
Ga_2Se_2	400	0.001	30	55
$IrTe_2$	345	0.001	30	55
Br_2Zn	621	0.0011	52	103
S_2	290	0.0011	30	55
Fe_2SeTe	180	0.0011	14	24
$Br_2H_2Yb_2$	228	0.0011	14	24
$As_2Fe_2Li_2$	228	0.0011	14	24
$C_2Br_2Gd_2$	738	0.0011	45	78
Ni_2SbTe_2	455	0.0011	30	55
O_2Sn_2	454	0.0011	33	64
$AgBrO_2$	698	0.0011	55	92
P_4	730	0.0012	59	94
$C_2Br_2Tb_2$	738	0.0013	45	78
Te_2Ti	621	0.0013	52	103
O_2Sn_2	454	0.0013	33	64
$HfSe_2$	621	0.0013	52	103
In_2Se_2	560	0.0013	44	74
I_4Zr_2	882	0.0013	76	71
Hg_3S_2	930	0.0014	110	54
$Hf_2I_2N_2$	438	0.0014	24	49
CdClHO	340	0.0014	24	49
$CdCl_2$	624	0.0014	54	100
Ni_2Te_2	724	0.0014	54	100
F_2Na	582	0.0014	49	96
Ga_2Se_2	724	0.0014	52	103
I_2Mn	624	0.0014	54	100
$Br_2Lu_2O_2$	228	0.0014	14	24
Sb_2Te_3	54	0.0014	4	6
$F_2Se_2Y_2$	354	0.0014	27	32
$Cl_2Er_2H_2$	924	0.0014	54	100
Cu_4Te_2	60	0.0014	4	6
In_2S_3	455	0.0014	30	55
GeTe	552	0.0014	57	105
$TaTe_2$	291	0.0014	24	49
$InSe_2$	624	0.0014	54	100
$C_2Br_2La_2$	864	0.0015	56	88
$I_2S_2Tb_2$	978	0.0015	69	94
Br_2Pr_2	678	0.0015	49	96

Table showing the first 50 lattice matching combinations with the smallest strain needed to build the supercell.

TbCl (P-3m1)

Structural and electronic properties

**Formula**	TbCl
**Spacegroup**	P-3m1
**Prototype**	ZrCl
**Parent 3D**	Tb_2Cl_2
**Source DB**	ICSD
**DB ID**	23351
**DF2-C09 Binding energy [meV/ Å^2]**	11.16
**RVV10 Binding energy [meV/ Å^2]**	16.98
**Band gap (PBE) [eV]**	N/A

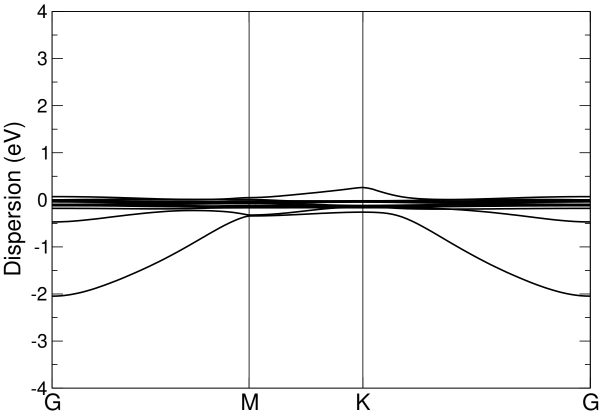

Band structure: Electronic band structure of TbCl (P-3m1) in a representative window around the Fermi energy and along a high-symmetry path.

Optimized crystal structure

Structural parameters: relaxed cell (top) and atomic positions (bottom) of TbCl (P-3m1) in Cartesian coordinates.

		x [Å]	y [Å]	z [Å]
$\mathbf{a_1}$		3.93072385	0.00000000	0.00000000
$\mathbf{a_2}$		−1.96536193	3.40410671	0.00000000
$\mathbf{a_3}$		0.00000000	0.00000000	26.30892078
		x [Å]	y [Å]	z [Å]
●	Tb	-0.00000000	2.26940447	14.70352863
●	Cl	0.00000000	0.00000000	9.98510227
●	Cl	0.00000000	0.00000000	16.32381850
●	Tb	1.96536193	1.13470224	11.60539214

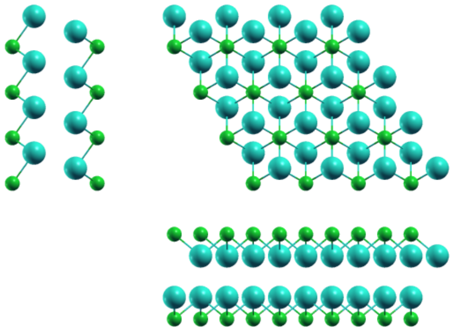

Orthographic projections: views of TbCl (P-3m1) as seen from the x axis (left), the y axis (bottom) and the z axis (center).

Lattice matching - minimal size

Formula	N° atoms	strain	cell size 1	cell size 2
K	5	2.9831	1	1
Na	5	0.2697	1	1
HgO	6	0.4315	1	1
GeTe	6	0.0045	1	1
S_2	6	0.0029	1	1
CaCl	6	0.1347	1	1
$IrTe_2$	7	0.0036	1	1
$CdCl_2$	7	0.0055	1	1
Cl_2Mn	7	1.5394	1	1
$MoTe_2$	7	0.273	1	1
$ReSe_2$	7	1.6358	1	1
S_2Ta	7	1.5543	1	1
CaI_2	7	0.4942	1	1
$InSe_2$	7	0.0051	1	1
$GeTe_2$	7	0.0065	1	1
$HfTe_2$	7	0.0048	1	1
Te_2V	7	0.2758	1	1
I_2Mn	7	0.0054	1	1
NSr_2	7	0.0088	1	1
I_2Yb	7	2.8311	1	1
LiO_2	7	0.0693	1	1
Cl_2Zn	7	0.147	1	1
FeI_2	7	0.0075	1	1
I_2Ni	7	0.0063	1	1
S_2Ti	7	1.5906	1	1
NbS_2	7	1.551	1	1
CrI_2	7	0.0079	1	1
Te_2Zn	7	0.2727	1	1
BiBrTe	7	0.4512	1	1
Bi_2Pd	7	0.1123	1	1
Cl_2Ni	7	0.2536	1	1
Cl_2Co	7	1.588	1	1
$CrTe_2$	7	0.262	1	1
PtS_2	7	0.2712	1	1
Br_2V	7	1.6297	1	1
ClNZr	7	0.2485	1	1
Cl_2Fe	7	1.5827	1	1
CdClO	7	0.2751	1	1
Ba_2N	7	0.0063	1	1
Se_2Ti	7	0.2673	1	1
Br_2Ti	7	0.2611	1	1
Te_2Zr	7	0.0053	1	1
Te_2W	7	0.2732	1	1
$AsSe_2$	7	0.2555	1	1
I_2Tm	7	2.8483	1	1
OTl_2	7	0.2754	1	1
BiITe	7	0.4674	1	1
BrNZr	7	0.2573	1	1
$NbSe_2$	7	0.2541	1	1
GeS_2	7	0.1261	1	1

Table showing the first 50 lattice matching combinations with the smallest number of atoms in the supercell.

Lattice matching - minimal strain

Formula	N° atoms	strain	cell size 1	cell size 2
$Br_2H_2Zr_2$	580	0.0	49	64
MnO_2	393	0.0	39	79
$AsSe_2$	447	0.0	57	73
Bi_2Te_3	729	0.0002	91	73
Se_2Ti	624	0.0002	81	100
BiITe	583	0.0002	91	73
Li_2Tl_2	520	0.0002	81	49
S_2Ti	343	0.0002	43	57
CrS_2	331	0.0003	37	61
$NbSe_2$	447	0.0003	57	73
CCl_2Sc_2	516	0.0003	49	64
Cd_2I_3	644	0.0004	81	64
Li_2Tl_2	392	0.0004	61	37
AsI_2La_2	644	0.0004	81	64
CaI_2	403	0.0005	64	49
I_2Y_2	8	0.0005	1	1
CrO_2	496	0.0005	49	100
Se_2Ta	499	0.0006	64	81
Br_2Zr_2	724	0.0006	81	100
NbS_2	291	0.0006	36	49
Cl_2NSc_2	389	0.0006	36	49
ClNZr	388	0.0006	49	64
Cl_2Co	343	0.0006	43	57
I_2La_2Sb	376	0.0007	49	36
$I_2S_2Tb_2$	486	0.0007	57	43
$I_2N_2Ti_2$	612	0.0007	63	60
Cl_2Gd_2	8	0.0007	1	1
Cl_2Zr_2	452	0.0007	49	64
CdO_2	343	0.0007	43	57
CNb_2S_2	280	0.0007	25	36
$Cl_2H_2Sc_2$	742	0.0008	64	81
O_2Pt	139	0.0008	16	25
Se_2Ta	447	0.0009	57	73
$AlLiTe_2$	656	0.0009	91	73
Cl_2Zr	343	0.0009	43	57
Br_2Hf_2	520	0.001	57	73
$NbSe_2$	447	0.001	57	73
K	232	0.001	49	36
Pd_2S_4	756	0.001	114	50
S_2Ta	291	0.001	36	49
Cl_2Mn	291	0.001	36	49
F_4Pb	877	0.0011	118	81
$C_2Br_2La_2$	616	0.0011	67	58
$LiNbS_2$	340	0.0011	36	49
DyI_2	357	0.0011	57	43
$LiMnSe_2$	8	0.0011	1	1
Br_2CsF	820	0.0011	126	79
GdI_2	643	0.0012	100	81
Sm	25	0.0012	4	9
K_2O_4	700	0.0012	115	40

Table showing the first 50 lattice matching combinations with the smallest strain needed to build the supercell.

Ti_2PTe_2 (P-3m1)

Structural and electronic properties

**Formula**	Ti_2PTe_2
**Spacegroup**	P-3m1
**Prototype**	Bi2Te2S
**Parent 3D**	Ti_2PTe_2
**Source DB**	ICSD
**DB ID**	418978
**DF2-C09 Binding energy [meV/ Å^2]**	26.26
**RVV10 Binding energy [meV/ Å^2]**	29.96
**Band gap (PBE) [eV]**	N/A

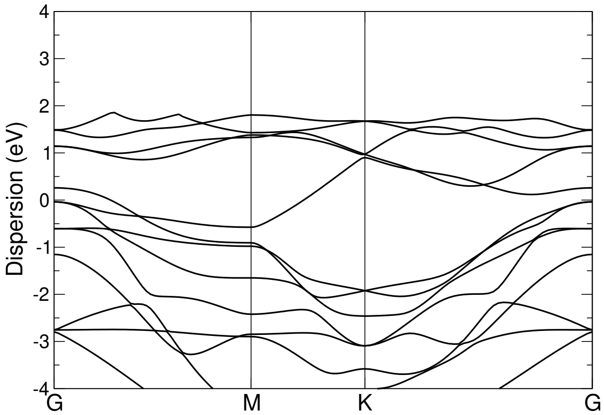

Band structure: Electronic band structure of Ti_2PTe_2 (P-3m1) in a representative window around the Fermi energy and along a high-symmetry path.

Optimized crystal structure

Structural parameters: relaxed cell (top) and atomic positions (bottom) of Ti_2PTe_2 (P-3m1) in Cartesian coordinates.

		x [Å]	y [Å]	z [Å]
$\mathbf{a_1}$		3.64386937	0.00000000	0.00000000
$\mathbf{a_2}$		−1.82193468	3.15568344	0.00000000
$\mathbf{a_3}$		0.00000000	0.00000000	26.32227233
		x [Å]	y [Å]	z [Å]
●	Ti	0.00000000	2.10378896	11.83789694
●	Te	0.00000000	2.10378896	16.31754368
●	Ti	0.00000000	0.00000000	14.48437529
●	Te	0.00000000	0.00000000	10.00472872
●	P	1.82193468	1.05189448	13.16113619

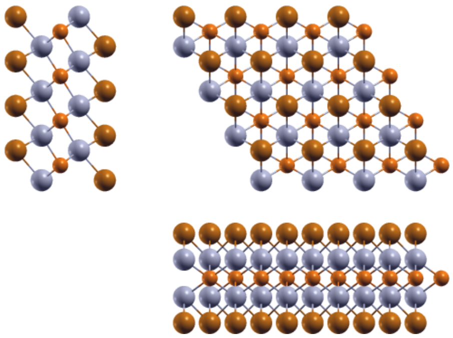

Orthographic projections: views of Ti_2PTe_2 (P-3m1) as seen from the x axis (left), the y axis (bottom) and the z axis (center).

Lattice matching - minimal size

Formula	N° atoms	strain	cell size 1	cell size 2
Tl	6	0.1184	1	1
In	6	0.422	1	1
In	6	1.5874	1	1
InSe	7	2.9877	1	1
HgO	7	0.1254	1	1
As_2	7	0.0045	1	1
LiO	7	0.2578	1	1
P_2	7	0.2686	1	1
PbTe	7	3.0113	1	1
Mg_2	7	0.1115	1	1
Sb_2	7	0.4773	1	1
Cl_2Zn	8	0.0056	1	1
I_2Mg	8	2.8535	1	1
S_2V	8	0.248	1	1
MoS_2	8	0.2489	1	1
CdI_2	8	3.0345	1	1
$AgTe_2$	8	0.12	1	1
PSn_2	8	0.0044	1	1
$MoSe_2$	8	0.2752	1	1
HfS_2	8	0.0018	1	1
$AsSn_2$	8	0.0082	1	1
Te_2V	8	0.0082	1	1
$CuTe_2$	8	0.0022	1	1
S_2Zr	8	0.0036	1	1
Br_2La	8	0.493	1	1
Br_2Cu	8	1.0237	1	1
Br_2Co	8	0.005	1	1
BiClTe	8	3.0394	1	1
ReS_2	8	1.5243	1	1
Ca_2N	8	0.004	1	1
Cl_2Ti	8	0.2688	1	1
$AuTe_2$	8	0.4575	1	1
BrCdI	8	0.5005	1	1
$PdTe_2$	8	0.4515	1	1
HgI_2	8	0.3366	1	1
Mg_3	8	0.1156	1	1
I_2Zn	8	0.469	1	1
S_2W	8	0.249	1	1
Bi_2Pd	8	0.132	1	1
GeI_2	8	0.4872	1	1
Br_2Mn	8	0.0067	1	1
$CoTe_2$	8	0.0015	1	1
CdClO	8	0.0087	1	1
AsKSn	8	2.9182	1	1
$PbTe_2$	8	0.4979	1	1
S_2Sn	8	0.0039	1	1
$SnTe_2$	8	0.4814	1	1
Cl_2V	8	0.2544	1	1
GeI_2	8	3.0144	1	1
$PtSe_2$	8	0.0089	1	1

Table showing the first 50 lattice matching combinations with the smallest number of atoms in the supercell.

Lattice matching - minimal strain

Formula	N° atoms	strain	cell size 1	cell size 2
MoS_2	437	0.0	49	64
Br_2Ni	8	0.0001	1	1
S_2W	437	0.0002	49	64
Br_2Ca_3Si	221	0.0002	25	16
CNb_2S_2	905	0.0002	81	100
Cl_2Mg	8	0.0002	1	1
I_2La_2Te	205	0.0002	25	16
$CeLi_2P_2$	650	0.0002	73	57
I_2La_2	457	0.0002	57	43
MoS_2	437	0.0002	49	64
Bi_2STe_2	425	0.0003	49	36
As_2Sn_2	824	0.0003	100	81
In_2Se_2	747	0.0003	91	73
Br_2Gd_2Ge	565	0.0003	64	49
Cu_2I_2	516	0.0003	64	49
AsKSn	414	0.0003	57	43
Br_2La_2	661	0.0003	81	64
$H_2I_2Yb_2$	613	0.0003	65	48
Ga_2S_2	9	0.0003	1	1
N_3W_2	205	0.0003	16	25
Br_2Gd_2	824	0.0004	100	81
$AsLi_3$	389	0.0004	49	36
BH_4Li	707	0.0004	73	57
$CrSe_2$	504	0.0005	57	73
$Cl_2H_2Lu_2$	11	0.0005	1	1
C_2F_2	321	0.0005	25	49
Sb_2	533	0.0005	81	64
$Br_2Nd_2O_2$	613	0.0006	65	48
Br_2OV	710	0.0006	82	75
Gd_2I_2	516	0.0006	64	49
Br_2HLa	593	0.0006	73	57
Cu_2I_2	914	0.0006	118	81
$F_2Se_2Y_2$	134	0.0007	16	9
InSe	317	0.0007	49	36
In	272	0.0007	43	57
P_2	605	0.0007	81	100
As_2Li_2Pr	425	0.0007	49	36
$SnTe_2$	536	0.0007	73	57
$Ce_2I_2S_2$	527	0.0007	61	37
$I_2La_2Si_2$	461	0.0007	49	36
Ca_2Si	416	0.0007	61	37
$I_2O_2Y_2$	613	0.0008	65	48
Cl_2La_2	824	0.0008	100	81
Cl_2V	504	0.0008	57	73
O_2Zn	233	0.0008	25	36
$AlLiTe_2$	280	0.0008	36	25
Bi_2Se_3	565	0.0008	64	49
I_2Zn	674	0.0008	91	73
Cl_2Ti	705	0.0008	81	100
Cl_4Pd_2	106	0.0009	14	6

Table showing the first 50 lattice matching combinations with the smallest strain needed to build the supercell.

$TiBr_2$ (P-3m1)

Structural and electronic properties

**Formula**	$TiBr_2$
**Spacegroup**	P-3m1
**Prototype**	CdI2
**Parent 3D**	$TiBr_2$
**Source DB**	ICSD
**DB ID**	26078
**DF2-C09 Binding energy [meV/ Å^2]**	15.26
**RVV10 Binding energy [meV/ Å^2]**	21.83
**Band gap (PBE) [eV]**	N/A

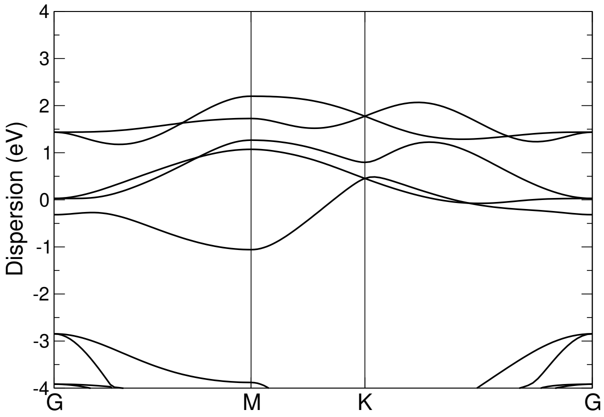

Band structure: Electronic band structure of $TiBr_2$ (P-3m1) in a representative window around the Fermi energy and along a high-symmetry path.

Optimized crystal structure

Structural parameters: relaxed cell (top) and atomic positions (bottom) of $TiBr_2$ (P-3m1) in Cartesian coordinates.

		x [Å]	y [Å]	z [Å]
$\mathbf{a_1}$		3.50379455	0.00000000	0.00000000
$\mathbf{a_2}$		−1.75189728	3.03437509	0.00000000
$\mathbf{a_3}$		0.00000000	0.00000000	23.38578315
		x [Å]	y [Å]	z [Å]
●	Br	0.00000000	2.02291673	10.00365309
●	Ti	0.00000000	0.00000000	11.69289157
●	Br	1.75189728	1.01145836	13.38213006

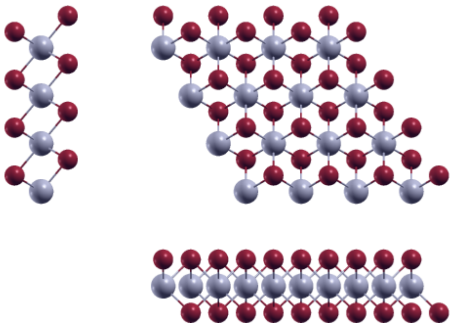

Orthographic projections: views of $TiBr_2$ (P-3m1) as seen from the x axis (left), the y axis (bottom) and the z axis (center).

Lattice matching - minimal size

Formula	N° atoms	strain	cell size 1	cell size 2
Tl	4	0.1297	1	1
Sn	4	0.1123	1	1
Na	4	0.006	1	1
In	4	0.1141	1	1
In	4	0.2691	1	1
Gd	4	0.1125	1	1
HgO	5	0.139	1	1
GeTe	5	0.4612	1	1
S_2	5	0.4652	1	1
Mg_2	5	0.1196	1	1
Sb_2	5	3.0217	1	1
$IrTe_2$	6	0.4635	1	1
CrS_2	6	0.2487	1	1
S_2V	6	0.2738	1	1
MoS_2	6	0.2749	1	1
$CdCl_2$	6	0.4586	1	1
$MoTe_2$	6	0.0084	1	1
$AgTe_2$	6	0.1319	1	1
$ReSe_2$	6	0.0063	1	1
$InSe_2$	6	0.4595	1	1
$GeTe_2$	6	0.4561	1	1
$HfTe_2$	6	0.4848	1	1
I_2Mn	6	0.4589	1	1
NSr_2	6	0.4503	1	1
ReS_2	6	0.2568	1	1
$AuTe_2$	6	2.9175	1	1
$PdTe_2$	6	0.4989	1	1
FeI_2	6	0.4534	1	1
I_2Ni	6	0.4566	1	1
Mg_3	6	0.1257	1	1
CrI_2	6	0.4523	1	1
I_2Zn	6	2.978	1	1
Te_2Zn	6	0.0082	1	1
S_2W	6	0.275	1	1
Bi_2Pd	6	0.1474	1	1
N_2W	6	1.5653	1	1
Cl_2Ni	6	0.0055	1	1
$CrTe_2$	6	0.0006	1	1
PtS_2	6	0.0071	1	1
Br_2V	6	0.0071	1	1
ClNZr	6	0.0092	1	1
Ba_2N	6	2.8374	1	1
Se_2Ti	6	0.0044	1	1
Te_2Zr	6	0.4861	1	1
Te_2W	6	0.0085	1	1
$AsSe_2$	6	0.004	1	1
BrNZr	6	0.0027	1	1
$NbSe_2$	6	0.005	1	1
CoI_2	6	0.4474	1	1
Br_2Cr	6	0.0003	1	1

Table showing the first 50 lattice matching combinations with the smallest number of atoms in the supercell.

Lattice matching - minimal strain

Formula	N° atoms	strain	cell size 1	cell size 2
GeTe	462	0.0	100	81
$Ce_2I_2S_2$	102	0.0	16	9
Ca_2Si	75	0.0	16	9
$AlLiTe_2$	139	0.0	25	16
CoH_2O_2	536	0.0001	57	73
$F_2Se_2Tm_2$	627	0.0001	81	64
$F_2Lu_2Se_2$	363	0.0001	49	36
Pt_2Te_2	447	0.0001	73	57
$PtTe_2$	300	0.0002	57	43
Br_2PY_2	437	0.0002	64	49
$Br_2H_2Zr_2$	9	0.0002	1	1
$LiMnSe_2$	499	0.0002	81	64
In_2S_3	705	0.0002	100	81
O_2Zn	300	0.0002	43	57
$AuTe_2$	300	0.0002	57	43
P_2Sn_2	565	0.0003	91	73
NaPSn	390	0.0003	73	57
Br_2Cr	6	0.0003	1	1
I_4Zr_2	627	0.0003	115	47
NS_2Ta	304	0.0003	36	49
H_2Si_2	624	0.0004	100	81
$Dy_2I_2S_2$	537	0.0004	81	49
Br_2Cd	339	0.0004	64	49
Br_2Cu	384	0.0004	72	56
$Er_2F_2Se_2$	561	0.0004	73	57
CrS_2	339	0.0004	49	64
$F_2Se_2Yb_2$	711	0.0004	91	73
$Ga_2I_2Y_2$	258	0.0005	36	25
O_2Pt	543	0.0005	81	100
Cl_2Sc_2	7	0.0005	1	1
Ni_2SbTe_2	638	0.0005	91	73
$Cl_2H_2Sc_2$	9	0.0005	1	1
$Br_2O_2Yb_2$	483	0.0006	65	48
$CrTe_2$	6	0.0006	1	1
O_4PSn	237	0.0006	39	20
$HfTe_2$	390	0.0006	73	57
S_2Zn_2	565	0.0006	91	73
In_2Se_3	504	0.0006	73	57
$InSe_2$	543	0.0007	100	81
BaF_2	183	0.0007	36	25
S_2	419	0.0007	91	73
$Bi_2Br_2O_2$	840	0.0007	118	81
AsKSn	183	0.0007	36	25
$Cl_2O_2V_2$	441	0.0007	53	47
Se_2Ta	6	0.0008	1	1
C_2	275	0.0008	39	79
I_2Y_2	499	0.0008	81	64
Br_2Er_2	343	0.0008	57	43
$Cl_2Er_2H_2$	786	0.0008	100	81
$I_2O_2Y_2$	840	0.0008	118	81

Table showing the first 50 lattice matching combinations with the smallest strain needed to build the supercell.

$TiCl_2$ (P-3m1)

Structural and electronic properties

**Formula**	$TiCl_2$
**Spacegroup**	P-3m1
**Prototype**	CdI2
**Parent 3D**	$TiCl_2$
**Source DB**	COD
**DB ID**	9009121
**DF2-C09 Binding energy [meV/ Å^2]**	14.95
**RVV10 Binding energy [meV/ Å^2]**	21.64
**Band gap (PBE) [eV]**	N/A

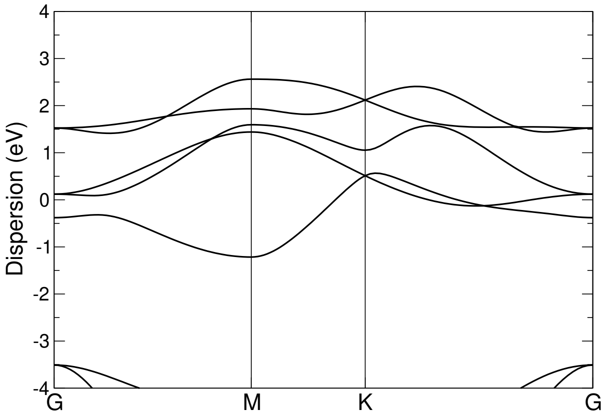

Band structure: Electronic band structure of $TiCl_2$ (P-3m1) in a representative window around the Fermi energy and along a high-symmetry path.

Optimized crystal structure

Structural parameters: relaxed cell (top) and atomic positions (bottom) of $TiCl_2$ (P-3m1) in Cartesian coordinates.

		x [Å]	y [Å]	z [Å]
$\mathbf{a_1}$		3.28534894	0.00000000	0.00000000
$\mathbf{a_2}$		−1.64267447	2.84519564	0.00000000
$\mathbf{a_3}$		0.00000000	0.00000000	23.19545011
		x [Å]	y [Å]	z [Å]
●	Cl	0.00000000	1.89679709	9.99994582
●	Ti	0.00000000	0.00000000	11.59772506
●	Cl	1.64267447	0.94839855	13.19550429

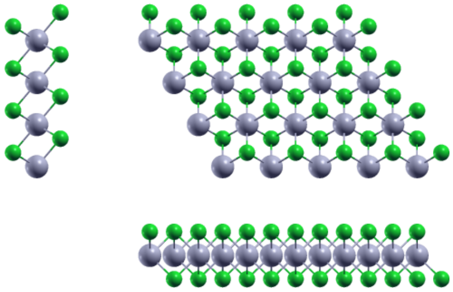

Orthographic projections: views of $TiCl_2$ (P-3m1) as seen from the x axis (left), the y axis (bottom) and the z axis (center).

Lattice matching - minimal size

Formula	N° atoms	strain	cell size 1	cell size 2
Tl	4	0.0052	1	1
Sn	4	0.1283	1	1
In	4	0.1314	1	1
As_2	5	0.4478	1	1
LiO	5	0.0076	1	1
P_2	5	0.0002	1	1
Mg_2	5	0.1407	1	1
PSn_2	6	0.4699	1	1
$MoSe_2$	6	0.0044	1	1
Br_2Zn	6	0.4977	1	1
HfS_2	6	0.4545	1	1
$AsSn_2$	6	0.4796	1	1
$SiTe_2$	6	2.9337	1	1
$CuTe_2$	6	0.4536	1	1
S_2Zr	6	0.468	1	1
NSr_2	6	3.0364	1	1
NiO_2	6	1.5568	1	1
Ca_2N	6	0.4491	1	1
Mg_3	6	0.1501	1	1
Te_2Ti	6	0.4985	1	1
$RhTe_2$	6	0.4869	1	1
NbS_2	6	0.0083	1	1
$CoTe_2$	6	0.4553	1	1
S_2Ta	6	0.0074	1	1
Se_2V	6	0.006	1	1
$NiTe_2$	6	2.9259	1	1
S_2Sn	6	0.4687	1	1
$PtSe_2$	6	0.4813	1	1
CoI_2	6	3.0204	1	1
$TaTe_2$	6	0.4781	1	1
Br_2Ni	6	0.4594	1	1
$FeSe_2$	6	0.1241	1	1
$NbTe_2$	6	0.4675	1	1
Cl_2Mg	6	0.4595	1	1
I_2La	6	0.3161	1	1
$CrSe_2$	6	0.0089	1	1
N_2Re	6	0.2462	1	1
Se_2Sn	6	3.0332	1	1
CoO_2	6	1.5529	1	1
$HfSe_2$	6	0.4985	1	1
Se_2W	6	0.0046	1	1
CdClHO	7	0.4766	1	1
Br_2Pr_2	7	0.5008	1	1
$HNiO_2$	7	0.2712	1	1
Ag_2Br_2	7	0.3172	1	1
CdClHO	7	0.4849	1	1
Cl_2Y_2	7	0.4944	1	1
As_2Ir_2	7	0.3221	1	1
Ga_2Se_2	7	2.9722	1	1
Ga_2S_2	7	0.4556	1	1

Table showing the first 50 lattice matching combinations with the smallest number of atoms in the supercell.

Lattice matching - minimal strain

Formula	N° atoms	strain	cell size 1	cell size 2
$AlLiTe_2$	84	0.0	16	9
CS_2Ta_2	8	0.0001	1	1
FeO_2	255	0.0001	36	49
STl_2	294	0.0001	61	37
KNO_3	17	0.0001	4	1
$Cl_2O_2Tm_2$	429	0.0001	57	43
$F_2Se_2Tm_2$	258	0.0001	36	25
CBr_2Y_2	437	0.0002	64	49
P_2	5	0.0002	1	1
$NbTe_2$	492	0.0002	91	73
Li_2P_2Pr	155	0.0002	25	16
CdClHO	499	0.0003	81	64
$Hf_2I_2N_2$	627	0.0003	81	64
CaClHO	343	0.0003	57	43
$LiMnSe_2$	208	0.0003	36	25
Sb_2	107	0.0003	25	16
$Cl_2H_2Lu_2$	786	0.0003	100	81
Cu_2O_4	393	0.0004	51	40
$Ga_2Ge_2Te_2$	171	0.0004	25	16
C_2	59	0.0004	9	16
Cl_2Y_2	388	0.0004	64	49
S_2Zr	492	0.0004	91	73
$Ce_2I_2S_2$	471	0.0005	79	39
Ca_2Si	354	0.0005	79	39
Br_2La_2	139	0.0005	25	16
In_2Te_3	488	0.0006	81	49
PTe_2Zr_2	327	0.0006	49	36
Sb_2SeTe_2	488	0.0006	81	49
Cl_2Mg	543	0.0007	100	81
CdClHO	447	0.0007	73	57
CNb_2S_2	8	0.0007	1	1
F_2Na	300	0.0007	57	43
S_2Sn	492	0.0007	91	73
Br_2Ni	543	0.0007	100	81
$Cl_2Hg_2N_2$	369	0.0007	75	24
CCl_2Gd_2	437	0.0007	64	49
$Cl_2O_2Yb_2$	429	0.0007	57	43
$PtSe_2$	390	0.0008	73	57
$Br_2In_2O_2$	552	0.0008	82	51
$TaTe_2$	435	0.0008	81	64
Au_2Br_2	591	0.0008	117	60
CoO_2	255	0.0008	36	49
PTe_2Ti_2	705	0.0008	100	81
Br_2Zn	339	0.0008	64	49
BaF_2	294	0.0009	61	37
$I_2Nd_2S_2$	297	0.0009	49	25
I_2Y_2	208	0.0009	36	25
$NiTe_2$	300	0.0009	57	43
Cu_2Se_2	678	0.0009	118	81
Bi_2Se_2Te	488	0.001	81	49

Table showing the first 50 lattice matching combinations with the smallest strain needed to build the supercell.

TiH_4 (P4/mmm)

Structural and electronic properties

**Formula**	TiH_4
**Spacegroup**	P4/mmm
**Prototype**	H4Ti
**Parent 3D**	H_4Ti
**Source DB**	MPDS
**DB ID**	S1401207
**DF2-C09 Binding energy [meV/ Å^2]**	22.72
**RVV10 Binding energy [meV/ Å^2]**	N/A
**Band gap (PBE) [eV]**	0.0

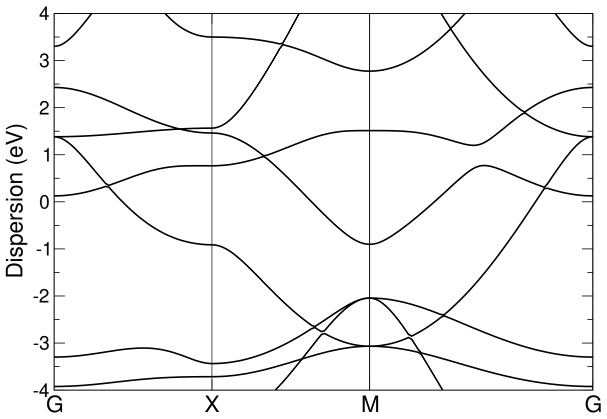

Band structure: Electronic band structure of TiH_4 (P4/mmm) in a representative window around the Fermi energy and along a high-symmetry path.

Optimized crystal structure

Structural parameters: relaxed cell (top) and atomic positions (bottom) of TiH_4 (P4/mmm) in Cartesian coordinates.

	x [Å]	y [Å]	z [Å]
$\mathbf{a_1}$	3.07180487	0.00000000	0.00000000
$\mathbf{a_2}$	0.00000000	3.07180566	0.00000000
$\mathbf{a_3}$	0.00000000	0.00000000	16.21573919

	x [Å]	y [Å]	z [Å]
H	0.00000000	-3.07180566	-1.05350251
H	1.53590243	-1.53590283	-1.05350305
Ti	0.00000000	-1.53590283	0.00000000
H	0.00000000	-3.07180566	1.05350251
H	1.53590243	-1.53590283	1.05350305

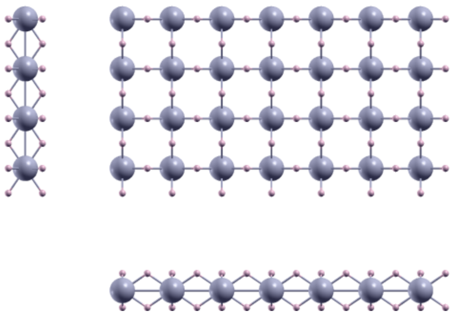

Orthographic projections: views of TiH_4 (P4/mmm) as seen from the x axis (left), the y axis (bottom) and the z axis (center).

Lattice matching - minimal size

Formula	N° atoms	strain	cell size 1	cell size 2
Tl	6	0.1434	1	1
Na	6	0.1759	1	1
As_2	7	0.1857	1	1
LiO	7	0.1329	1	1
BN	7	0.1111	1	1
P_2	7	0.1389	1	1
Mg_2	7	0.0024	1	1
CaCl	7	1.1745	1	1
Cl_2Zn	8	0.1844	1	1
Cl_2Mn	8	0.1486	1	1
$MoTe_2$	8	0.1786	1	1
PSn_2	8	0.1974	1	1
$MoSe_2$	8	0.1427	1	1
$ReSe_2$	8	0.1618	1	1
S_2Ta	8	0.1506	1	1
HfS_2	8	0.1892	1	1
Te_2V	8	0.181	1	1
$CuTe_2$	8	0.1887	1	1
S_2Zr	8	0.1963	1	1
Br_2Co	8	0.1851	1	1
Ca_2N	8	0.1864	1	1
Cl_2Ti	8	0.139	1	1
S_2Ti	8	0.1555	1	1
Mg_3	8	0.0081	1	1
NbS_2	8	0.1502	1	1
Te_2Zn	8	0.1784	1	1
$RhTe_2$	8	0.7765	1	1
Br_2Mn	8	0.1829	1	1
Cl_2Ni	8	0.1628	1	1
Cl_2Co	8	0.1552	1	1
$CrTe_2$	8	0.1696	1	1
PtS_2	8	0.1771	1	1
NbS_2	8	0.1461	1	1
$CoTe_2$	8	0.1896	1	1
Br_2V	8	0.161	1	1
ClNZr	8	0.1588	1	1
Cl_2Fe	8	0.1544	1	1
CdClO	8	0.1804	1	1
S_2Ta	8	0.1454	1	1
Se_2V	8	0.1441	1	1
Se_2Ti	8	0.174	1	1
Br_2Ti	8	0.169	1	1
Te_2W	8	0.1788	1	1
$AsSe_2$	8	0.1644	1	1
S_2Sn	8	0.1967	1	1
Cl_2V	8	0.131	1	1
$PtSe_2$	8	0.7681	1	1
OTl_2	8	0.1806	1	1
CdO_2	8	0.155	1	1
BrNZr	8	0.1659	1	1

Table showing the first 50 lattice matching combinations with the smallest number of atoms in the supercell.

Lattice matching - minimal strain

Formula	N° atoms	strain	cell size 1	cell size 2
$I_2O_2Tm_2$	920	0.0	106	65
$Br_2H_2Sr_2$	134	0.0	16	9
H_2Na_2Pd	575	0.0001	65	50
Fe_2S_2	389	0.0001	49	36
Cu_2Se_2	280	0.0002	36	25
$H_2I_2Yb_2$	699	0.0002	81	49
F_4Pb	810	0.0002	109	53
$Ge_2Mn_2Sr_2$	833	0.0002	97	58
Bi_2STe_2	865	0.0002	108	65
GeS_2	467	0.0002	64	49
P_2Rh_2	189	0.0003	25	16
$I_2S_2Tl_2$	330	0.0004	36	25
F_2Tl_2	189	0.0004	25	16
Cu_2I_2	116	0.0004	16	9
I_2La	552	0.0005	81	49
H_2Li_2Pd	10	0.0005	1	1
$Cl_2S_2Tl_2$	649	0.0006	89	34
As_2Fe_2	516	0.0006	64	49
Ba_2N	833	0.0006	118	81
As_2Ir_2	717	0.0006	97	58
PbTe	670	0.0007	108	65
F_2Ni	255	0.0007	36	25
$Si_2Te_2Zr_2$	330	0.0007	36	25
$Br_2Eu_2F_2$	920	0.0007	106	65
Br_2CsF	61	0.0007	9	4
As_2CeLi_2	865	0.0007	108	65
$Er_2I_2O_2$	920	0.0008	106	65
As_2Rh_2	601	0.0008	81	49
$AsLi_3$	800	0.0008	108	65
DyI_2	865	0.0008	131	70
$Br_2Dy_2O_2$	221	0.0008	25	16
$Br_2O_2Y_2$	221	0.0008	25	16
$Ge_2Se_2Zr_2$	791	0.0009	85	61
Ca_2Cl_2	669	0.0009	85	61
$Br_2Ho_2O_2$	221	0.0009	25	16
$H_2I_2Sr_2$	395	0.0009	49	25
GeI_2	735	0.0009	108	65
$Br_2Nd_2O_2$	699	0.0009	81	49
Cu_2S_2	189	0.0009	25	16
Ag_2Br_2	601	0.001	81	49
Ge_2Se_2	455	0.001	63	35
H_2Na_2Pd	565	0.001	64	49
Sm	521	0.001	79	126
I_2Nd	725	0.001	106	65
$Ca_2Mn_2Si_2$	221	0.0011	25	16
InSe	670	0.0011	108	65
$Br_2O_2Sm_2$	920	0.0011	106	65
As_2Li_2Pr	865	0.0011	108	65
$Hf_2Se_2Si_2$	614	0.0011	64	49
$I_2O_2Y_2$	699	0.0011	81	49

Table showing the first 50 lattice matching combinations with the smallest strain needed to build the supercell.

TiI_2 (P-3m1)

Structural and electronic properties

**Formula**	TiI_2
**Spacegroup**	P-3m1
**Prototype**	CdI2
**Parent 3D**	I_2Ti
**Source DB**	MPDS
**DB ID**	S1300934
**DF2-C09 Binding energy [meV/ Å^2]**	16.29
**RVV10 Binding energy [meV/ Å^2]**	N/A
**Band gap (PBE) [eV]**	0.0

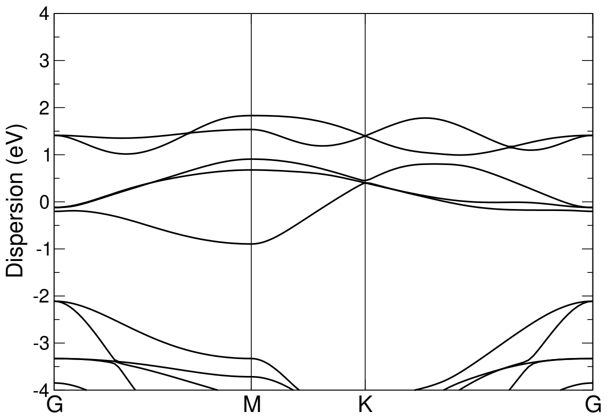

Band structure: Electronic band structure of TiI_2 (P-3m1) in a representative window around the Fermi energy and along a high-symmetry path.

Optimized crystal structure

Structural parameters: relaxed cell (top) and atomic positions (bottom) of TiI_2 (P-3m1) in Cartesian coordinates.

		x [Å]	y [Å]	z [Å]
$\mathbf{a_1}$		1.92500034	−3.33419840	0.00000000
$\mathbf{a_2}$		1.92500034	3.33419840	0.00000000
$\mathbf{a_3}$		0.00000000	0.00000000	19.04497065
		x [Å]	y [Å]	z [Å]
●	I	0.96250017	-0.55569973	1.76290124
●	Ti	-0.96250017	-1.66709920	0.00000000
●	I	0.96250017	-2.77849866	-1.76290124

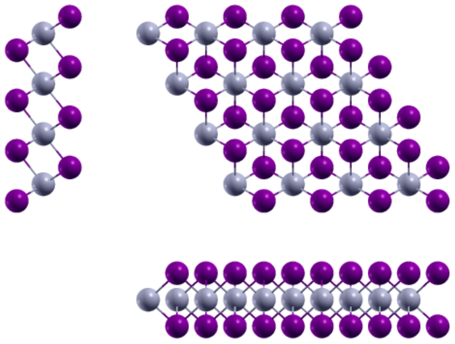

Orthographic projections: views of TiI_2 (P-3m1) as seen from the x axis (left), the y axis (bottom) and the z axis (center).

Lattice matching - minimal size

Formula	N° atoms	strain	cell size 1	cell size 2
Tl	4	0.4264	1	1
InSe	5	0.4522	1	1
Nd	5	0.2188	1	2
HgO	5	0.1124	1	1
AsSb	5	0.0008	1	1
Bi_2	5	0.4666	1	1
GeTe	5	0.0053	1	1
Ag_2	5	3.1931	1	1
S_2	5	0.0069	1	1
PbTe	5	0.4566	1	1
CaCl	5	0.1428	1	1
$IrTe_2$	6	0.0063	1	1
$CdCl_2$	6	0.0042	1	1
Cl_2Mn	6	0.248	1	1
CdI_2	6	0.4608	1	1
$AgTe_2$	6	0.4332	1	1
Ba_2Pt	6	3.1895	1	1
$ReSe_2$	6	0.2659	1	1
Br_2Ca	6	0.4639	1	1
$InSe_2$	6	0.0046	1	1
$GeTe_2$	6	0.0032	1	1
$SiTe_2$	6	0.0068	1	1
I_2Mn	6	0.0044	1	1
NSr_2	6	0.0009	1	1
PbS_2	6	0.0033	1	1
BiClTe	6	0.4617	1	1
LiO_2	6	0.0654	1	1
FeI_2	6	0.0021	1	1
I_2Ni	6	0.0034	1	1
S_2Ti	6	0.2574	1	1
NbS_2	6	0.2501	1	1
CrI_2	6	0.0017	1	1
BiBrTe	6	0.4757	1	1
Bi_2Pd	6	0.1163	1	1
Cl_2Ni	6	0.2672	1	1
Cl_2Co	6	0.2569	1	1
Br_2V	6	0.2647	1	1
ClNZr	6	0.2618	1	1
Cl_2Fe	6	0.2559	1	1
Br_2Ti	6	0.2753	1	1
$AsSe_2$	6	0.2693	1	1
$NiTe_2$	6	0.0073	1	1
I_2V	6	0.0055	1	1
GeI_2	6	0.4571	1	1
Se_2Zr	6	0.0064	1	1
BiITe	6	0.4928	1	1
CdO_2	6	0.2567	1	1
BrNZr	6	0.2712	1	1
$NbSe_2$	6	0.2678	1	1
CoI_2	6	0.0003	1	1

Table showing the first 50 lattice matching combinations with the smallest number of atoms in the supercell.

Lattice matching - minimal strain

Formula	N° atoms	strain	cell size 1	cell size 2
Gd_2GeI_2	705	0.0	100	81
GdI_2	390	0.0	73	57
$Cl_2H_2Zr_2$	471	0.0001	43	57
BiBrTe	435	0.0001	81	64
Bi_2	419	0.0001	91	73
CdI_2	543	0.0001	100	81
$NbSe_2$	543	0.0001	81	100
FHOZn	219	0.0002	25	36
$Ge_2I_2La_2$	429	0.0002	57	43
NbS_2	300	0.0002	43	57
BiClTe	543	0.0002	100	81
Se_2Ta	543	0.0002	81	100
Cl_2Ni	543	0.0003	81	100
CrO_2	222	0.0003	25	49
$CrSe_2$	183	0.0003	25	36
Br_2V	492	0.0003	73	91
I_2Pr	543	0.0003	100	81
Nd	196	0.0003	39	79
CoI_2	6	0.0003	1	1
Cl_2Fe	390	0.0003	57	73
$Br_2Cr_2O_2$	438	0.0004	48	49
CaI_2	255	0.0004	49	36
CI_2Y_2	8	0.0004	1	1
Br_2La_2P	705	0.0004	100	81
Bi_2SeTe_2	638	0.0005	91	73
Cl_2Zr_2	583	0.0005	73	91
$C_2Cl_2Y_2$	522	0.0005	58	58
Cl_2NSc_2	467	0.0005	49	64
$Cl_2Cr_2O_2$	306	0.0005	32	35
$Ga_2Gd_2I_2$	711	0.0005	91	73
CdI_2	543	0.0006	100	81
Br_2Ca_3Si	315	0.0006	43	31
Se_2Sn	6	0.0006	1	1
NbS_2	339	0.0006	49	64
Ba_2Pt	183	0.0007	36	25
Cl_2Zr	390	0.0007	57	73
F_4Sn	435	0.0008	65	48
AsSb	5	0.0008	1	1
$Ga_2I_2Tb_2$	711	0.0008	91	73
NSr_2	6	0.0009	1	1
CCl_2Sc_2	674	0.0009	73	91
Cl_2V	183	0.0009	25	36
Cu_2Sr_2	499	0.0009	81	64
CdO_2	390	0.0009	57	73
Ag_2	158	0.001	36	25
I_2La_2P	504	0.001	73	57
$I_2La_2O_2$	483	0.001	65	48
$LiOS_2Ti$	414	0.001	43	57
Bi_2STe_2	705	0.001	100	81
Cl_2Mn	339	0.001	49	64

Table showing the first 50 lattice matching combinations with the smallest strain needed to build the supercell.

TiNBr (Pmmn)

Structural and electronic properties

**Formula**	TiNBr
**Spacegroup**	Pmmn
**Prototype**	FeOCl
**Parent 3D**	$Ti_2N_2Br_2$
**Source DB**	ICSD
**DB ID**	27395
**DF2-C09 Binding energy [meV/ Å^2]**	12.12
**RVV10 Binding energy [meV/ Å^2]**	20.08
**Band gap (PBE) [eV]**	0.62

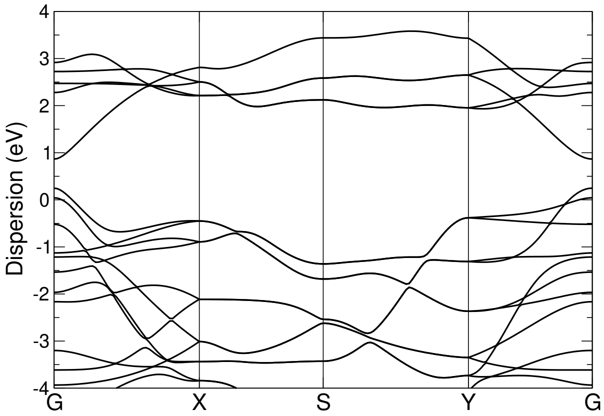

Band structure: Electronic band structure of TiNBr (Pmmn) in a representative window around the Fermi energy and along a high-symmetry path.

Optimized crystal structure

Structural parameters: relaxed cell (top) and atomic positions (bottom) of TiNBr (Pmmn) in Cartesian coordinates.

		x [Å]	y [Å]	z [Å]
$\mathbf{a_1}$		3.36998459	0.00000000	0.00000000
$\mathbf{a_2}$		0.00000000	3.95127250	0.00000000
$\mathbf{a_3}$		0.00000000	0.00000000	25.53138877
		x [Å]	y [Å]	z [Å]
	Ti	1.68499229	0.00000000	12.01768044
	Br	0.00000000	0.00000000	10.01890897
	N	1.68499229	1.97563625	12.41960774
	Ti	0.00000000	1.97563625	13.51370963
	Br	1.68499229	1.97563625	15.51248024
	N	0.00000000	0.00000000	13.11178211

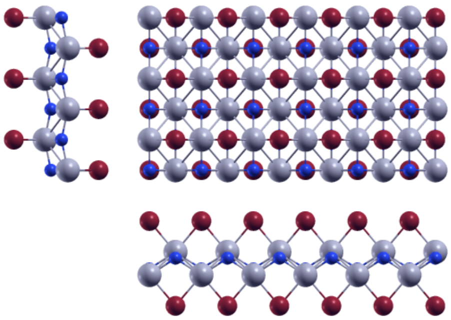

Orthographic projections: views of TiNBr (Pmmn) as seen from the x axis (left), the y axis (bottom) and the z axis (center).

Lattice matching - minimal size

Formula	N° atoms	strain	cell size 1	cell size 2
K	7	0.2395	1	1
Ag_2	8	0.2484	1	1
Sb_2	8	0.5877	1	1
Ba_2Pt	9	0.248	1	1
I_2Pr	9	0.0574	1	1
Ca_2Si	9	0.2555	1	1
$PdTe_2$	9	0.5571	1	1
Mg_3	9	0.652	1	1
I_2Nd	9	0.0582	1	1
Cl_2Cu	9	0.4744	1	1
I_2Pb	9	0.251	1	1
GeS_2	9	0.2944	1	1
CeI_2	9	0.0567	1	1
Br_2Cd	9	0.5545	1	1
I_2La	9	0.0627	1	1
F_2Zn	9	0.3115	1	1
Fe_2Te_2	10	0.2962	1	1
Ca_2Cl_2	10	0.2969	1	1
Cu_2Te_2	10	0.012	1	1
Ir_2P_2	10	0.0581	1	1
Ag_2Br_2	10	0.0632	1	1
Cu_2S_2	10	0.3058	1	1
Au_2Br_2	10	1.056	1	1
Ca_2O_2	10	0.2553	1	1
Br_2Tb_2	10	0.5588	1	1
Br_2Cu_2	10	0.3091	1	1
As_2Ir_2	10	0.0656	1	1
Cu_2Te_2	10	0.2817	1	1
$AgBrO_2$	10	0.5106	1	1
Ge_2S_2	10	0.4625	1	1
C_2Li_2	10	0.9637	1	1
O_2Sn_2	10	0.3068	1	1
P_2Rh_2	10	0.3047	1	1
F_2Tl_2	10	0.3049	1	1
Au_2I_2	10	0.5706	1	1
Sb_2Te_2	10	0.2533	1	1
As_2Fe_2	10	0.2958	1	1
Ag_2Te_2	10	0.0414	1	1
As_2Ru_2	10	0.2972	1	1
$AgClO_2$	10	0.1249	1	1
PbS_2Sn	10	1.0375	1	1
Ni_2Se_2	10	0.0565	1	1
As_2Rh_2	10	0.063	1	1
Fe_2SeTe	10	0.2868	1	1
Br_2La_2	10	0.587	1	1
O_2Sn_2	10	0.5802	1	1
Br_2PY_2	11	0.5539	1	1
H_2Na_2Pd	11	0.2931	1	1
Bi_2S_3	11	0.5596	1	1
I_2La_2Sb	11	0.2401	1	1

Table showing the first 50 lattice matching combinations with the smallest number of atoms in the supercell.

Lattice matching - minimal strain

Formula	N° atoms	strain	cell size 1	cell size 2
Br_2La	711	0.0001	82	73
Bi_2Se_3	857	0.0002	82	73
I_2Mg	711	0.0003	82	73
Gd_2I_2	784	0.0004	82	73
$Ce_2I_2Si_2$	930	0.0005	82	73
$Gd_2I_2Se_2$	834	0.0007	90	49
$Cl_2Er_2O_2$	558	0.0007	45	48
Cu_2I_2	784	0.0007	82	73
Se_2Zr	414	0.0007	45	48
Br_2Gd_2Ge	857	0.0007	82	73
Ge_2Te_2	398	0.0007	45	32
$SiTe_2$	414	0.0007	45	48
$CrSe_2$	693	0.0007	66	99
As_2Sn_2	468	0.0008	48	45
$C_2Br_2Y_2$	648	0.0008	54	54
Sb_2Se_2Te	857	0.0008	82	73
Br_2Gd_2	468	0.0008	48	45
FHOZn	792	0.0008	66	99
H_2NiO_2	891	0.0009	66	99
Cl_2La_2	468	0.0009	48	45
O_2Zn	396	0.001	36	60
Br_2Cu	438	0.001	49	48
LiO_2	477	0.001	48	63
$NiTe_2$	414	0.0011	45	48
$Br_2Nd_2O_2$	78	0.0012	7	6
$I_2O_2Y_2$	78	0.0012	7	6
Br_2Er_2	468	0.0012	48	45
Sn	158	0.0012	21	32
I_2V	414	0.0013	45	48
$Br_2S_2Yb_2$	234	0.0013	24	15
Cl_2V	765	0.0013	73	109
Sb_2Se_2Te	857	0.0013	82	73
Ga_2S_2	596	0.0014	56	65
Au_2Se_2	660	0.0014	80	45
Se_2Ta_4	78	0.0014	6	7
$H_2I_2Yb_2$	78	0.0014	7	6
La_2S_2	444	0.0014	50	36
PTe_2Ti_2	661	0.0015	56	65
Au_2I_2	834	0.0015	95	66
Sb_2	940	0.0015	120	110
Br_2Ni	531	0.0015	56	65
BN	370	0.0015	34	83
Cl_2Mg	531	0.0015	56	65
Cl_2V	744	0.0016	71	106
$Cl_2Ho_2O_2$	558	0.0016	45	48
Ga_2S_2	596	0.0016	56	65
$Bi_2Br_2O_2$	78	0.0016	7	6
$BiTe_2$	711	0.0016	82	73
O_2Sn_2	964	0.0016	94	100
CaClHO	462	0.0016	45	48

Table showing the first 50 lattice matching combinations with the smallest strain needed to build the supercell.

TiNCl (Pmmn)

Structural and electronic properties

**Formula**	TiNCl
**Spacegroup**	Pmmn
**Prototype**	FeOCl
**Parent 3D**	$Ti_2N_2Cl_2$
**Source DB**	ICSD
**DB ID**	261525
**DF2-C09 Binding energy [meV/ Å^2]**	11.03
**RVV10 Binding energy [meV/ Å^2]**	19.05
**Band gap (PBE) [eV]**	0.6

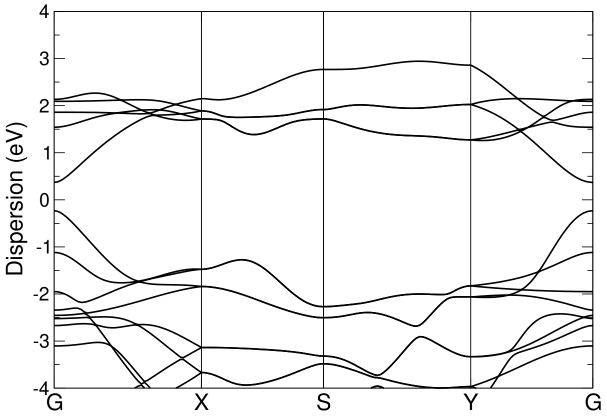

Band structure: Electronic band structure of TiNCl (Pmmn) in a representative window around the Fermi energy and along a high-symmetry path.

Optimized crystal structure

Structural parameters: relaxed cell (top) and atomic positions (bottom) of TiNCl (Pmmn) in Cartesian coordinates.

		x [Å]	y [Å]	z [Å]
$\mathbf{a_1}$		3.27318801	0.00000000	0.00000000
$\mathbf{a_2}$		0.00000000	3.95334399	0.00000000
$\mathbf{a_3}$		0.00000000	0.00000000	25.18495758
		x [Å]	y [Å]	z [Å]
●	Ti	1.63659401	0.00000000	11.82172505
●	N	1.63659401	1.97667200	12.21786728
●	Cl	0.00000000	0.00000000	9.97722518
●	Ti	0.00000000	1.97667200	13.36323278
●	N	0.00000000	0.00000000	12.96709059
●	Cl	1.63659401	1.97667200	15.20773311

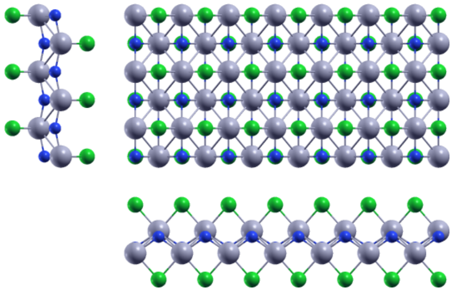

Orthographic projections: views of TiNCl (Pmmn) as seen from the x axis (left), the y axis (bottom) and the z axis (center).

Lattice matching - minimal size

Formula	N° atoms	strain	cell size 1	cell size 2
K	7	0.2547	1	1
Ag_2	8	0.264	1	1
Ba_2Pt	9	0.2636	1	1
CaI_2	9	0.2397	1	1
$AuTe_2$	9	1.799	1	1
Cl_2Zn	9	0.2854	1	1
Mg_3	9	0.233	1	1
I_2Nd	9	0.0717	1	1
Cl_2Cu	9	1.4987	1	1
I_2Pb	9	0.2668	1	1
GeS_2	9	0.3137	1	1
DyI_2	9	0.2442	1	1
F_2Ni	9	0.2789	1	1
$PtTe_2$	9	1.7957	1	1
I_2La	9	0.0765	1	1
F_2Zn	9	0.3204	1	1
Fe_2Te_2	10	0.3046	1	1
Ca_2Cl_2	10	0.3054	1	1
Cl_2OOs	10	0.2697	1	1
Cu_2Te_2	10	0.0054	1	1
Ir_2P_2	10	0.0715	1	1
Ag_2Br_2	10	0.077	1	1
Br_2Er_2	10	1.8036	1	1
$AgCuTe_2$	10	0.0229	1	1
O_2Sn_2	10	0.2904	1	1
Cl_2OV	10	0.0284	1	1
Br_2Cu_2	10	0.3179	1	1
Fe_2Se_2	10	0.278	1	1
As_2Co_2	10	0.2703	1	1
As_2Ir_2	10	0.0795	1	1
Cu_2Te_2	10	0.2897	1	1
$AgBrO_2$	10	0.5217	1	1
Ge_2S_2	10	0.3092	1	1
C_2Li_2	10	0.2378	1	1
O_2Sn_2	10	0.3156	1	1
N_4	10	0.1366	1	1
Au_2I_2	10	0.6053	1	1
Sb_2Te_2	10	0.2692	1	1
As_2Fe_2	10	0.3151	1	1
Cu_2Se_2	10	0.2798	1	1
Ag_2Te_2	10	0.3005	1	1
As_2Ru_2	10	0.3057	1	1
$AgClO_2$	10	0.1347	1	1
PbS_2Sn	10	1.0503	1	1
As_2Rh_2	10	0.0768	1	1
Fe_2SeTe	10	0.295	1	1
O_2Sn_2	10	0.1994	1	1
Co_2Se_2	10	0.2724	1	1
Ca_2Cl_2	10	0.2692	1	1
Hf_3Te_2	11	0.2837	1	1

Table showing the first 50 lattice matching combinations with the smallest number of atoms in the supercell.

Lattice matching - minimal strain

Formula	N° atoms	strain	cell size 1	cell size 2
Ge_2Te_2	710	0.0005	81	56
$Cl_2Rh_2Te_2$	414	0.0006	45	24
Ag_2I_2	788	0.0008	92	59
As_2Sn_2	974	0.0008	101	92
Cl_4Mg_2	840	0.0008	109	31
CrI_2	504	0.0009	56	56
Sm	131	0.0009	16	35
Br_2Mg	504	0.0009	56	56
$I_2S_2Sm_2$	144	0.0009	14	10
FeI_2	504	0.0009	56	56
K	94	0.0009	14	10
Cl_2La_2	974	0.0009	101	92
$Cl_2O_2Y_2$	426	0.001	35	36
N_2Re	402	0.001	35	64
S_2Zn_2	356	0.001	36	35
PTe_2Ti_2	465	0.001	40	45
Br_2Ni	375	0.001	40	45
Cl_2Mg	375	0.001	40	45
$Cl_2Ho_2O_2$	426	0.001	35	36
Br_2La_2	430	0.001	45	40
$FeSe_2$	942	0.001	89	136
Ga_2S_2	420	0.001	40	45
Cl_2Gd_2	356	0.0011	36	35
$Cl_2H_2Lu_2$	510	0.0011	40	45
Sb_2	350	0.0011	45	40
I_2La_2Sb	134	0.0011	14	10
$F_2Se_2Yb_2$	426	0.0011	36	35
P_2Sn_2	356	0.0012	36	35
I_2V	318	0.0012	35	36
Na	654	0.0012	91	108
Ga_2Se_2	354	0.0012	35	36
NS_2Zr	978	0.0013	91	108
$HfLiS_2$	900	0.0014	84	99
NSr_2	504	0.0014	56	56
$Au_2K_2S_2$	30	0.0014	4	1
Au_2I_2	712	0.0014	82	55
Ca_4Cu_2	246	0.0014	27	14
Ge_2S_2	838	0.0014	91	73
Li_2P_2Pr	470	0.0014	45	40
Te_2Zn	801	0.0014	84	99
Ge_2S_2	828	0.0014	90	72
LiO_2	648	0.0014	66	84
O_2Zn	303	0.0015	28	45
PtS_2	801	0.0015	84	99
Br_2Y_2	354	0.0015	35	36
$MoTe_2$	801	0.0015	84	99
SSb_2Te_2	781	0.0015	76	65
$Ga_2Ge_2Te_2$	510	0.0015	45	40
CdClO	618	0.0015	65	76
Te_2W	657	0.0015	69	81

Table showing the first 50 lattice matching combinations with the smallest strain needed to build the supercell.

TiNI (Pmmn)

Structural and electronic properties

**Formula**	TiNI
**Spacegroup**	Pmmn
**Prototype**	FeOCl
**Parent 3D**	$Ti_2N_2I_2$
**Source DB**	ICSD
**DB ID**	27394
**DF2-C09 Binding energy [meV/ Å^2]**	14.56
**RVV10 Binding energy [meV/ Å^2]**	21.9
**Band gap (PBE) [eV]**	0.02

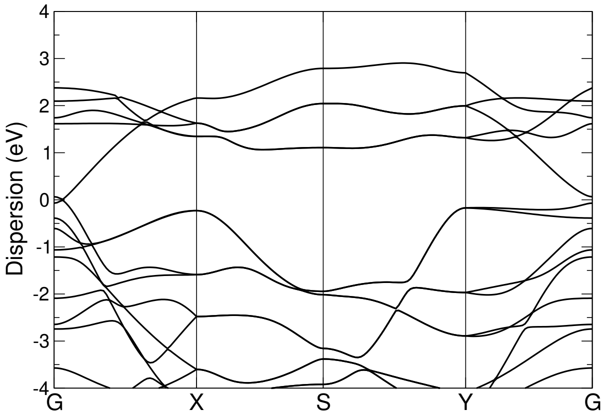

Band structure: Electronic band structure of TiNI (Pmmn) in a representative window around the Fermi energy and along a high-symmetry path.

Optimized crystal structure

Structural parameters: relaxed cell (top) and atomic positions (bottom) of TiNI (Pmmn) in Cartesian coordinates.

	x [Å]	y [Å]	z [Å]
$\mathbf{a_1}$	3.53025909	0.00000000	0.00000000
$\mathbf{a_2}$	0.00000000	3.97017938	0.00000000
$\mathbf{a_3}$	0.00000000	0.00000000	25.85326732

	x [Å]	y [Å]	z [Å]
Ti	1.76512954	0.00000000	12.23870022
I	0.00000000	0.00000000	10.02143313
N	1.76512954	1.98508969	12.60719805
Ti	0.00000000	1.98508969	13.61456618
I	1.76512954	1.98508969	15.83183329
N	0.00000000	0.00000000	13.24606798

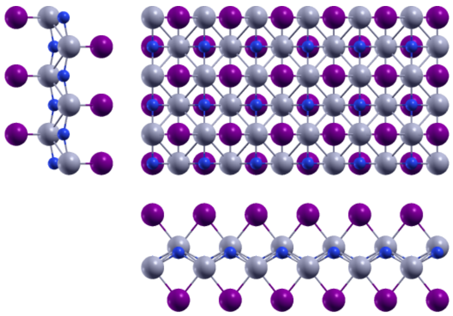

Orthographic projections: views of TiNI (Pmmn) as seen from the x axis (left), the y axis (bottom) and the z axis (center).

Lattice matching - minimal size

Formula	N° atoms	strain	cell size 1	cell size 2
K	7	0.2164	1	1
Bi_2	8	0.6318	1	1
AgTl	8	0.3207	1	1
Ag_2	8	0.2246	1	1
CaCl	8	0.2842	1	1
CdI_2	9	0.6244	1	1
Ba_2Pt	9	0.2242	1	1
Br_2Ca	9	0.6283	1	1
CaI_2	9	0.2034	1	1
I_2Pr	9	0.0378	1	1
I_2Yb	9	0.1999	1	1
BiClTe	9	0.6256	1	1
BrCdI	9	0.5912	1	1
BaF_2	9	0.6013	1	1
Ba_2Hg	9	0.364	1	1
CNRb	9	1.2461	1	1
AsKSn	9	0.5969	1	1
$PbTe_2$	9	0.5882	1	1
I_2Nd	9	0.0385	1	1
Cl_2Cu	9	0.4523	1	1
I_2Tm	9	0.2018	1	1
STl_2	9	0.6043	1	1
BiITe	9	0.1896	1	1
GeS_2	9	0.0365	1	1
$MnSe_2$	9	0.2841	1	1
DyI_2	9	0.2073	1	1
CeI_2	9	0.3013	1	1
CNNa	9	0.485	1	1
I_2La	9	0.0424	1	1
CdI_2	9	0.623	1	1
I_2Pr	9	0.6258	1	1
Bi_2Te_2	10	1.8228	1	1
Cu_2I_2	10	0.3431	1	1
Cu_2Te_2	10	0.0249	1	1
Ir_2P_2	10	0.0383	1	1
Ag_2Br_2	10	0.0428	1	1
Pb_2Se_2	10	1.3479	1	1
O_2Sn_2	10	0.0142	1	1
$AlLiTe_2$	10	0.1882	1	1
Ge_2Te_2	10	0.4726	1	1
Cl_2ORu	10	0.0239	1	1
As_2Ir_2	10	0.045	1	1
O_2Pb_2	10	0.3366	1	1
$AgBrO_2$	10	0.1189	1	1
As_4	10	0.4934	1	1
$MnNaTe_2$	10	0.5899	1	1
P_4	10	0.4488	1	1
Br_2OV	10	0.0291	1	1
Fe_2S_2	10	0.0312	1	1
LiO	10	1.2266	1	2

Table showing the first 50 lattice matching combinations with the smallest number of atoms in the supercell.

Lattice matching - minimal strain

Formula	N° atoms	strain	cell size 1	cell size 2
$Ge_2Mn_2Sr_2$	102	0.0001	9	8
FHOZn	860	0.0003	70	110
$As_2Cd_2K_2$	726	0.0003	72	49
I_2Mg	846	0.0004	96	90
Br_2La	846	0.0004	96	90
$CrSe_2$	750	0.0004	70	110
CrO_2	522	0.0004	42	90
GeTe	732	0.0004	90	96
Cl_2Gd_2	612	0.0004	60	63
BH_4Li	738	0.0005	63	60
F_4Pb	480	0.0006	50	36
$CeLi_2P_2$	678	0.0006	63	60
Gd_2I_2	936	0.0006	96	90
$SnTe_2$	558	0.0007	63	60
$Br_2S_2Yb_2$	318	0.0007	32	21
Cl_2Tb_2	612	0.0007	60	63
H_2Si_2	924	0.0007	90	96
$IrTe_2$	828	0.0008	90	96
Cl_2V	750	0.0008	70	110
NaPSn	801	0.0008	88	91
As_2Ir_2	86	0.0008	9	8
Cu_2I_2	936	0.0009	96	90
Br_2HLa	618	0.0009	63	60
Ga_2Se_2	924	0.0009	90	96
$InSe_2$	828	0.001	90	96
Pt_2Te_2	892	0.001	88	91
$AgTe_2$	366	0.001	36	50
CrS_2	447	0.001	40	69
H_2MnO_2	850	0.001	55	104
FeO_2	387	0.0011	32	65
$Br_2O_2Pr_2$	102	0.0011	9	8
Cu_2F_4	108	0.0011	12	6
S_2Zn_2	612	0.0011	60	63
Sn_2Te_2	592	0.0011	68	46
$Cl_2Er_2S_2$	360	0.0012	36	24
I_2Y_2	612	0.0012	60	63
I_2Mn	828	0.0012	90	96
$I_2S_2Yb_2$	276	0.0013	28	18
Br_2La_2	898	0.0013	91	88
$Br_2Lu_2S_2$	360	0.0013	36	24
$Br_2Lu_2S_2$	360	0.0013	36	24
H_2Li_2Pd	902	0.0013	67	100
$AsCuLi_2$	618	0.0013	63	60
O_4PSn	726	0.0013	72	49
$CdCl_2$	828	0.0013	90	96
$F_2Se_2Yb_2$	738	0.0013	60	63
Bi_2S_3	616	0.0014	56	56
Br_2Ho_2	560	0.0014	56	56
Br_2Tb_2	560	0.0014	56	56
$Hf_2Se_2Si_2$	102	0.0014	8	9

Table showing the first 50 lattice matching combinations with the smallest strain needed to build the supercell.

TiOBr (Pmmn)

Structural and electronic properties

**Formula**	TiOBr
**Spacegroup**	Pmmn
**Prototype**	FeOCl
**Parent 3D**	$Ti_2O_2Br_2$
**Source DB**	COD
**DB ID**	1523160
**DF2-C09 Binding energy [meV/ Å^2]**	12.29
**RVV10 Binding energy [meV/ Å^2]**	20.13
**Band gap (PBE) [eV]**	N/A

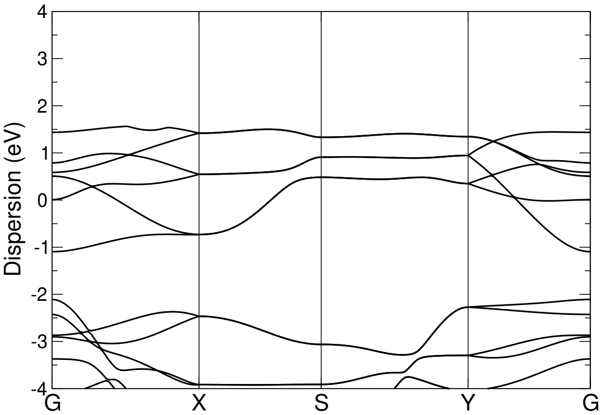

Band structure: Electronic band structure of TiOBr (Pmmn) in a representative window around the Fermi energy and along a high-symmetry path.

Optimized crystal structure

Structural parameters: relaxed cell (top) and atomic positions (bottom) of TiOBr (Pmmn) in Cartesian coordinates.

	x [Å]	y [Å]	z [Å]
$\mathbf{a_1}$	3.39313896	0.00000000	0.00000000
$\mathbf{a_2}$	0.00000000	4.07928785	0.00000000
$\mathbf{a_3}$	0.00000000	0.00000000	25.24537154

		x [Å]	y [Å]	z [Å]
●	Ti	0.00000000	2.03964392	12.01646818
●	Br	1.69656948	2.03964392	10.03243626
•	O	0.00000000	0.00000000	12.09875704
●	Ti	1.69656948	0.00000000	13.22891077
●	Br	0.00000000	0.00000000	15.21293528
•	O	1.69656948	2.03964392	13.14662580

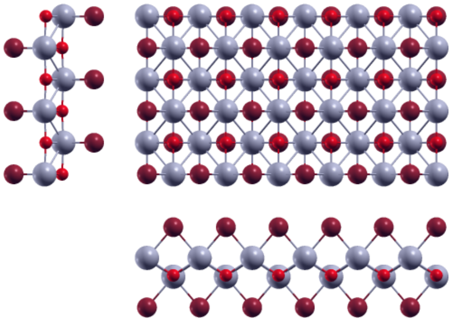

Orthographic projections: views of TiOBr (Pmmn) as seen from the x axis (left), the y axis (bottom) and the z axis (center).

Lattice matching - minimal size

Formula	N° atoms	strain	cell size 1	cell size 2
K	7	1.001	1	1
AgTl	8	0.3162	1	1
CaCl	8	0.3059	1	1
I_2Pr	9	0.2996	1	1
Br_2Cu	9	0.546	1	1
Ca_2Si	9	0.2439	1	1
BrCdI	9	1.8076	1	1
I_2Nd	9	0.3012	1	1
Cl_2Cu	9	0.4482	1	1
I_2Pb	9	0.7462	1	1
GeS_2	9	0.2861	1	1
$MnSe_2$	9	0.3057	1	1
CeI_2	9	0.2983	1	1
CNNa	9	0.5118	1	1
F_2Ni	9	0.3241	1	1
I_2La	9	0.3096	1	1
F_2Zn	9	0.2928	1	1
Bi_2Te_2	10	0.2652	1	1
Fe_2Te_2	10	0.2784	1	1
Ca_2Cl_2	10	0.2791	1	1
Cu_2I_2	10	0.0757	1	1
Cu_2Te_2	10	0.0139	1	1
Ir_2P_2	10	0.3009	1	1
O_2Sn_2	10	0.028	1	1
Cu_2S_2	10	0.2875	1	1
Cl_2OV	10	0.2846	1	1
Br_2Cu_2	10	0.2906	1	1
Fe_2Se_2	10	0.3231	1	1
Cl_2ORu	10	0.3161	1	1
As_2Co_2	10	0.3144	1	1
O_2Pb_2	10	0.0724	1	1
$AgBrO_2$	10	0.4808	1	1
As_4	10	0.5214	1	1
$MnNaTe_2$	10	1.8043	1	1
C_2Li_2	10	0.2154	1	1
P_4	10	0.4727	1	1
Br_2OV	10	0.2993	1	1
O_2Sn_2	10	0.2884	1	1
P_2Rh_2	10	0.2864	1	1
Fe_2S_2	10	0.3024	1	1
F_2Tl_2	10	0.2866	1	1
Sb_2Te_2	10	0.2418	1	1
Co_2S_2	10	0.307	1	1
As_2Fe_2	10	0.2875	1	1
Ge_2Se_2	10	0.3212	1	1
Cu_2Se_2	10	0.3251	1	1
Ag_2Te_2	10	0.2746	1	1
As_2Ru_2	10	0.2794	1	1
Bi_2O_2	10	0.0737	1	1
$AgClO_2$	10	0.1208	1	1

Table showing the first 50 lattice matching combinations with the smallest number of atoms in the supercell.

Lattice matching - minimal strain

Formula	N° atoms	strain	cell size 1	cell size 2
As_2Li_2Nd	470	0.0002	45	40
Br_2Pr_2	420	0.0002	40	45
$F_2Ho_2Se_2$	672	0.0003	56	56
GeI_2Y_2	470	0.0004	45	40
Bi_2O_2	56	0.0004	6	5
Mg_2	242	0.0005	27	40
NiO_2	360	0.0005	30	60
$I_2La_2Si_2$	510	0.0005	45	40
As_2Li_2Pr	470	0.0005	45	40
$FeSe_2$	327	0.0005	30	49
InSe	350	0.0005	45	40
$HfSe_2$	375	0.0007	40	45
Te_2Ti	375	0.0007	40	45
$Br_2La_2O_2$	66	0.0008	6	5
$Eu_2F_2I_2$	66	0.0008	6	5
$Ba_2F_2I_2$	402	0.0008	40	27
$AsLi_3$	430	0.0008	45	40
Bi_2Se_2Te	470	0.0008	45	40
O_4PTl	402	0.0009	40	27
I_2La_2	636	0.0009	66	60
$Cl_2O_2Y_2$	756	0.0009	60	66
N_2Re	534	0.0009	45	88
$Cl_2O_2Yb_2$	510	0.0009	40	45
O_2Zn	447	0.0009	40	69
Br_2Zn	375	0.001	40	45
Br_2PY_2	616	0.001	56	56
CoO_2	360	0.001	30	60
F_2Na	375	0.001	40	45
AsKSn	576	0.001	66	60
$Cu_2Se_2Tl_2$	66	0.0011	6	5
CCl_2Gd_2	465	0.0011	40	45
CdClHO	738	0.0011	69	81
$Hf_2I_2N_2$	900	0.0011	69	81
Ga_2Se_2	624	0.0012	60	66
Br_2Cd	504	0.0012	56	56
I_2V	558	0.0012	60	66
Sb_2SeTe_2	470	0.0012	45	40
$BiTe_2$	906	0.0012	103	96
Se_2Yb	906	0.0012	103	96
CuO_2	249	0.0013	24	35
In_2Te_3	470	0.0013	45	40
H_2Li_2Pt	417	0.0013	32	45
GeI_2	906	0.0013	103	96
Bi_2Se_2	538	0.0014	59	46
$As_2Cd_2K_2$	402	0.0014	40	27
$NbTe_2$	870	0.0014	91	108
S_2Zr	870	0.0014	91	108
O_2Pb_2	56	0.0015	6	5
Bi_2STe_2	470	0.0015	45	40
Sn_2Te_2	312	0.0015	36	24

Table showing the first 50 lattice matching combinations with the smallest strain needed to build the supercell.

TiOCl (Pmmn)

Structural and electronic properties

**Formula**	TiOCl
**Spacegroup**	Pmmn
**Prototype**	FeOCl
**Parent 3D**	$Ti_2O_2Cl_2$
**Source DB**	ICSD
**DB ID**	155833
**DF2-C09 Binding energy [meV/ Å^2]**	11.69
**RVV10 Binding energy [meV/ Å^2]**	19.52
**Band gap (PBE) [eV]**	N/A

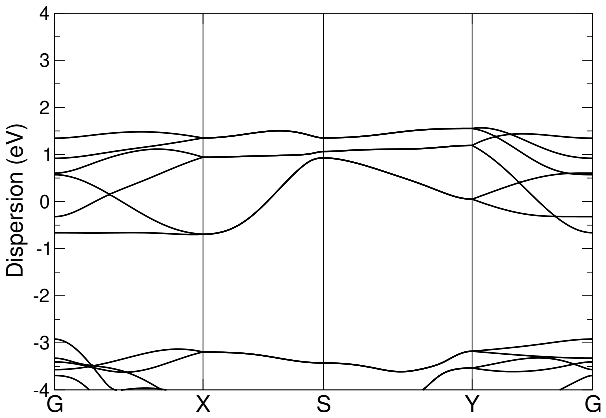

Band structure: Electronic band structure of TiOCl (Pmmn) in a representative window around the Fermi energy and along a high-symmetry path.

Optimized crystal structure

Structural parameters: relaxed cell (top) and atomic positions (bottom) of TiOCl (Pmmn) in Cartesian coordinates.

		x [Å]	y [Å]	z [Å]
$\mathbf{a_1}$		3.26102124	0.00000000	0.00000000
$\mathbf{a_2}$		0.00000000	4.02563285	0.00000000
$\mathbf{a_3}$		0.00000000	0.00000000	24.94375250
		x [Å]	y [Å]	z [Å]
●	Ti	1.63051062	0.00000000	11.77610287
●	Cl	0.00000000	0.00000000	9.97951607
●	O	1.63051062	2.01281643	11.88203066
●	Ti	0.00000000	2.01281643	13.16764838
●	Cl	1.63051062	2.01281643	14.96423641
●	O	0.00000000	0.00000000	13.06172346

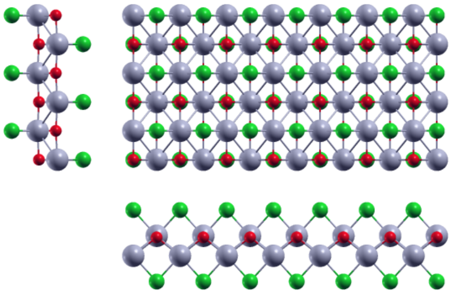

Orthographic projections: views of TiOCl (Pmmn) as seen from the x axis (left), the y axis (bottom) and the z axis (center).

Lattice matching - minimal size

Formula	N° atoms	strain	cell size 1	cell size 2
Tl	7	0.2407	1	1
K	7	0.2526	1	1
Ag_2	8	0.2618	1	1
CaCl	8	0.0494	1	1
$AgTe_2$	9	0.2445	1	1
Ba_2Pt	9	0.2613	1	1
Ca_2Si	9	0.2692	1	1
Cl_2Zn	9	0.278	1	1
BaF_2	9	1.9309	1	1
I_2Pb	9	0.2645	1	1
STl_2	9	1.9393	1	1
GeS_2	9	0.3139	1	1
$MnSe_2$	9	0.0494	1	1
DyI_2	9	0.2422	1	1
F_2Ni	9	0.2716	1	1
F_2Zn	9	0.3121	1	1
Cl_2OOs	10	0.2624	1	1
Cu_2Te_2	10	0.0009	1	1
$AgCuTe_2$	10	0.0161	1	1
O_2Sn_2	10	0.0439	1	1
Cu_2S_2	10	0.3064	1	1
Cl_2OV	10	0.0333	1	1
Br_2Cu_2	10	0.3097	1	1
Fe_2Se_2	10	0.2708	1	1
As_2Co_2	10	0.0509	1	1
Cu_2Te_2	10	0.2822	1	1
$AgBrO_2$	10	0.5076	1	1
Ge_2S_2	10	0.2995	1	1
As_4	10	1.2103	1	1
C_2Li_2	10	0.233	1	1
P_4	10	0.5131	1	1
Br_2OV	10	0.328	1	1
O_2Sn_2	10	0.3074	1	1
Mg_4	10	0.2403	1	1
Fe_2S_2	10	0.0489	1	1
F_2Tl_2	10	0.3055	1	1
Sb_2Te_2	10	0.2669	1	1
Co_2S_2	10	0.0496	1	1
As_2Fe_2	10	0.3154	1	1
Cu_2Se_2	10	0.2725	1	1
Ag_2Te_2	10	0.2924	1	1
$AgClO_2$	10	0.1348	1	1
Fe_2SeTe	10	0.2873	1	1
O_2Sn_2	10	0.195	1	1
Co_2Se_2	10	0.0515	1	1
Ca_2Cl_2	10	0.0507	1	1
Hf_3Te_2	11	0.2763	1	1
H_2Na_2Pd	11	0.3125	1	1
F_4Nb	11	0.37	1	1
$AgNO_3$	11	0.3632	1	1

Table showing the first 50 lattice matching combinations with the smallest number of atoms in the supercell.

Lattice matching - minimal strain

Formula	N° atoms	strain	cell size 1	cell size 2
As_2O_3	300	0.0005	35	18
ReS_2	429	0.0005	40	63
CdClHO	332	0.0005	32	35
$Br_2Hf_2N_2$	906	0.0006	70	81
$Cl_2N_2Zr_2$	906	0.0006	70	81
I_2Zn	306	0.0007	35	32
Sb_2Te_2	88	0.0007	10	7
CaH_2O_2	825	0.0007	70	81
CoH_2O_2	501	0.0008	36	57
I_2La_2	766	0.0008	81	70
In_2Se_2	338	0.0009	35	32
Cu_2Te_2	10	0.0009	1	1
BrCdI	696	0.0009	81	70
$RhTe_2$	297	0.001	32	35
SSb_2Te_2	836	0.001	81	70
Ca_2N	663	0.001	70	81
I_2Pb	81	0.001	10	7
$AsCuLi_2$	936	0.001	98	87
$BiTe_2$	969	0.0011	112	99
$AgClO_2$	764	0.0011	78	74
Se_2Yb	969	0.0011	112	99
Br_2HLa	936	0.0011	98	87
Ga_2Te_2	936	0.0011	98	87
$CuTe_2$	663	0.0011	70	81
GeI_2	969	0.0011	112	99
O_2Sn_2	86	0.0011	9	8
GeI_2	849	0.0012	98	87
F_2Na	432	0.0012	47	50
AsKSn	696	0.0012	81	70
AlH_4Na	840	0.0012	88	52
Br_2Pr_2	482	0.0012	47	50
C_2F_2	536	0.0012	36	80
O_4PSn	588	0.0012	60	38
Ca_2Cl_2	900	0.0013	90	90
CuO_2	354	0.0013	35	48
$MnNaTe_2$	766	0.0013	81	70
Se_2Yb	849	0.0013	98	87
Fe_2S_2	878	0.0013	87	89
$CeLi_2P_2$	973	0.0013	93	83
Nd	282	0.0014	35	72
HfS_2	663	0.0014	70	81
Cl_2Zn	837	0.0014	88	103
$PtSe_2$	297	0.0015	32	35
ReS_2	387	0.0015	36	57
CKN	831	0.0015	106	65
Bi_2In_2	650	0.0015	77	47
As_2	582	0.0015	70	81
$BiTe_2$	849	0.0015	98	87
Ca_2Cl_2	890	0.0015	89	89
KS_2Ti	940	0.0015	88	103

Table showing the first 50 lattice matching combinations with the smallest strain needed to build the supercell.

TiS_2 (P-3m1)

Structural and electronic properties

**Formula**	TiS_2
**Spacegroup**	P-3m1
**Prototype**	CdI2
**Parent 3D**	TiS_2
**Source DB**	COD
**DB ID**	1010275
**DF2-C09 Binding energy [meV/ Å^2]**	23.83
**RVV10 Binding energy [meV/ Å^2]**	27.36
**Band gap (PBE) [eV]**	0.06

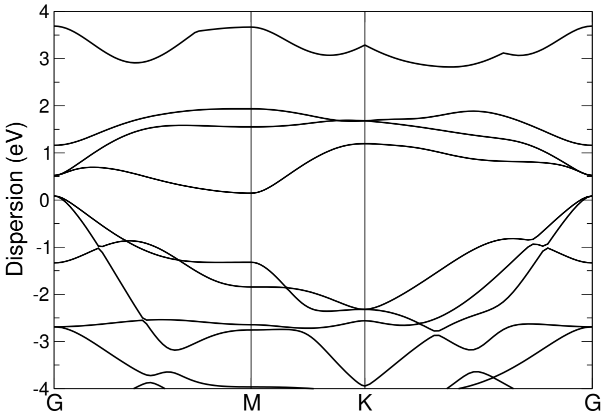

Band structure: Electronic band structure of TiS_2 (P-3m1) in a representative window around the Fermi energy and along a high-symmetry path.

Optimized crystal structure

Structural parameters: relaxed cell (top) and atomic positions (bottom) of TiS_2 (P-3m1) in Cartesian coordinates.

		x [Å]	y [Å]	z [Å]
$\mathbf{a_1}$		3.41227804	0.00000000	0.00000000
$\mathbf{a_2}$		−1.70613902	2.95511947	0.00000000
$\mathbf{a_3}$		0.00000000	0.00000000	22.85446632
		x [Å]	y [Å]	z [Å]
•	S	0.00000000	1.97007965	12.84727610
•	Ti	0.00000000	0.00000000	11.42723316
•	S	1.70613902	0.98503982	10.00719022

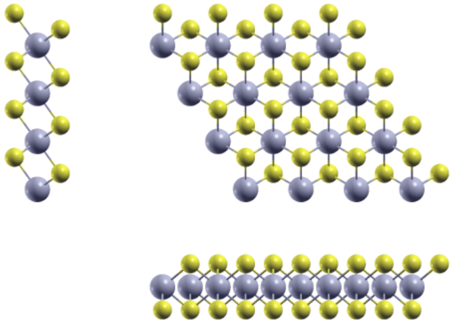

Orthographic projections: views of TiS_2 (P-3m1) as seen from the x axis (left), the y axis (bottom) and the z axis (center).

Lattice matching - minimal size

Formula	N° atoms	strain	cell size 1	cell size 2
Tl	4	0.1393	1	1
Sn	4	0.1177	1	1
In	4	0.12	1	1
HgO	5	0.1502	1	1
AsSb	5	0.4775	1	1
GeTe	5	0.4934	1	1
S_2	5	0.4975	1	1
Mg_2	5	0.1271	1	1
Sb_2	5	3.1946	1	1
CaCl	5	0.2088	1	1
$IrTe_2$	6	0.4958	1	1
CrS_2	6	0.2659	1	1
$CdCl_2$	6	2.8457	1	1
Cl_2Mn	6	0.0069	1	1
$AgTe_2$	6	0.1419	1	1
$ReSe_2$	6	0.0061	1	1
S_2Ta	6	0.0049	1	1
Br_2Zn	6	0.4519	1	1
$InSe_2$	6	2.8505	1	1
$SiTe_2$	6	0.4621	1	1
$HfTe_2$	6	2.9798	1	1
I_2Mn	6	2.8472	1	1
NSr_2	6	0.4817	1	1
PbS_2	6	0.4709	1	1
ReS_2	6	0.2746	1	1
FeI_2	6	0.4851	1	1
I_2Ni	6	2.8354	1	1
Mg_3	6	0.1346	1	1
Te_2Ti	6	0.4526	1	1
NbS_2	6	0.0054	1	1
CrI_2	6	0.4839	1	1
Ba_2Hg	6	0.3342	1	1
N_2W	6	0.2564	1	1
Cl_2Ni	6	0.0071	1	1
Cl_2Co	6	0.0003	1	1
Br_2V	6	0.0053	1	1
ClNZr	6	0.0032	1	1
Cl_2Fe	6	0.0011	1	1
Ba_2N	6	3.0002	1	1
Te_2Zr	6	2.9864	1	1
$AsSe_2$	6	0.0086	1	1
$NiTe_2$	6	0.4606	1	1
Cl_2Cu	6	0.1076	1	1
I_2V	6	0.4655	1	1
Se_2Zr	6	0.4631	1	1
BiITe	6	13.6261	1	1
CdO_2	6	0.0005	1	1
$NbSe_2$	6	0.0075	1	1
CoI_2	6	0.4787	1	1
O_2Zn	6	0.2612	1	1

Table showing the first 50 lattice matching combinations with the smallest number of atoms in the supercell.

Lattice matching - minimal strain

Formula	N° atoms	strain	cell size 1	cell size 2
Cu_3Se_3	363	0.0	49	36
$ClKO_3$	17	0.0	4	1
$LiMnTe_2$	139	0.0001	25	16
PTe_2Zr_2	563	0.0001	81	64
$AlLiTe_2$	331	0.0001	61	37
$IrTe_2$	339	0.0001	64	49
$Cl_2Ho_2O_2$	711	0.0002	91	73
$NiTe_2$	543	0.0002	100	81
Cl_2Tb_2	343	0.0002	57	43
GeI_2	123	0.0003	25	16
Ba_2N	255	0.0003	49	36
Bi_2Te_3	488	0.0003	81	49
Ga_2Se_2	388	0.0003	64	49
I_2La_2Te	93	0.0003	16	9
CrI_2	390	0.0003	73	57
Cl_2Co	6	0.0003	1	1
$SiTe_2$	543	0.0004	100	81
BiITe	390	0.0004	81	49
Bi_2STe_2	155	0.0004	25	16
As_2CeLi_2	155	0.0004	25	16
$F_2Se_2Y_2$	471	0.0005	79	39
Br_2Ca_3Si	102	0.0005	16	9
Cl_2Gd_2	343	0.0005	57	43
PbTe	107	0.0005	25	16
$Cl_2O_2Y_2$	711	0.0005	91	73
H_2MnO_2	353	0.0005	36	49
CdO_2	6	0.0005	1	1
NSr_2	390	0.0006	73	57
In_2S_3	437	0.0006	64	49
I_2V	492	0.0006	91	73
Br_2Mg	390	0.0006	73	57
$I_2N_2Zr_2$	531	0.0006	77	50
AsSb	371	0.0006	81	64
$Cl_2Er_2O_2$	786	0.0006	100	81
$Ca_2Mn_2Si_2$	840	0.0007	118	81
$Br_2Ho_2O_2$	840	0.0007	118	81
N_2W	390	0.0007	57	73
Cl_2Zr	6	0.0007	1	1
FeI_2	390	0.0007	73	57
$C_2Br_2La_2$	687	0.0007	99	65
$F_2Lu_2Se_2$	258	0.0007	36	25
$Br_2Ca_2H_2$	840	0.0008	118	81
I_2Y_2	343	0.0008	57	43
GeTe	290	0.0008	64	49
Te_2Zr	255	0.0008	49	36
Se_2Zr	543	0.0008	100	81
S_2	290	0.0008	64	49
CdI_2	123	0.0008	25	16
Se_2Sn	390	0.0008	73	57
Br_2La_2	208	0.0008	36	25

Table showing the first 50 lattice matching combinations with the smallest strain needed to build the supercell.

$TiSe_2$ (P-3m1)

Structural and electronic properties

**Formula**	$TiSe_2$
**Spacegroup**	P-3m1
**Prototype**	CdI2
**Parent 3D**	$TiSe_2$
**Source DB**	COD
**DB ID**	1010276
**DF2-C09 Binding energy [meV/ Å^2]**	24.04
**RVV10 Binding energy [meV/ Å^2]**	28.19
**Band gap (PBE) [eV]**	N/A

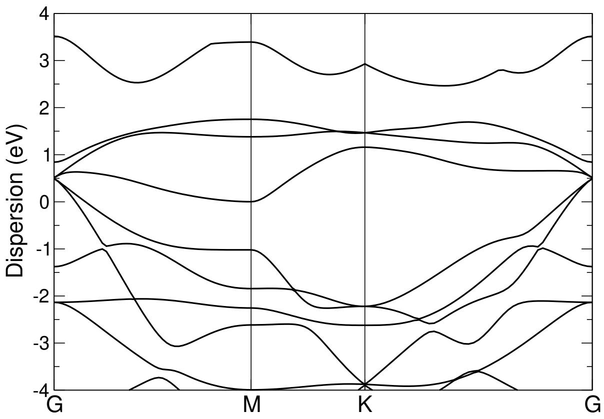

Band structure: Electronic band structure of $TiSe_2$ (P-3m1) in a representative window around the Fermi energy and along a high-symmetry path.

Optimized crystal structure

Structural parameters: relaxed cell (top) and atomic positions (bottom) of $TiSe_2$ (P-3m1) in Cartesian coordinates.

		x [Å]	y [Å]	z [Å]
$\mathbf{a_1}$		3.53625882	0.00000000	0.00000000
$\mathbf{a_2}$		−1.76812941	3.06248997	0.00000000
$\mathbf{a_3}$		0.00000000	0.00000000	23.10324801
		x [Å]	y [Å]	z [Å]
●	Se	1.76812941	1.02082999	10.00077787
●	Ti	0.00000000	0.00000000	11.55162401
●	Se	0.00000000	2.04165998	13.10247014

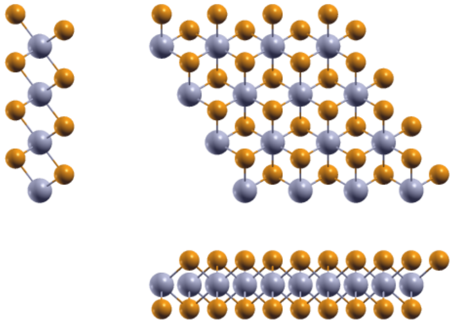

Orthographic projections: views of $TiSe_2$ (P-3m1) as seen from the x axis (left), the y axis (bottom) and the z axis (center).

Lattice matching - minimal size

Formula	N° atoms	strain	cell size 1	cell size 2
K	4	0.3303	1	1
Tl	4	0.1267	1	1
Sn	4	0.1109	1	1
Na	4	0.0016	1	1
In	4	0.1124	1	1
In	4	0.2629	1	1
Gd	4	0.1134	1	1
HgO	5	0.1355	1	1
GeTe	5	0.4505	1	1
S_2	5	0.4543	1	1
Mg_2	5	0.1173	1	1
Sb_2	5	2.9636	1	1
$IrTe_2$	6	0.4527	1	1
CrS_2	6	1.5831	1	1
Cl_2Zn	6	0.0087	1	1
I_2Mg	6	3.0396	1	1
S_2V	6	0.2674	1	1
MoS_2	6	0.2685	1	1
$CdCl_2$	6	0.4479	1	1
$MoTe_2$	6	0.0039	1	1
$AgTe_2$	6	0.1287	1	1
$InSe_2$	6	0.4488	1	1
$HfTe_2$	6	0.4736	1	1
Te_2V	6	0.0059	1	1
I_2Mn	6	0.4482	1	1
Br_2La	6	3.0442	1	1
Br_2Cu	6	1.0959	1	1
Br_2Co	6	0.0093	1	1
ReS_2	6	1.6277	1	1
$AuTe_2$	6	0.4938	1	1
$PdTe_2$	6	0.4873	1	1
Mg_3	6	0.123	1	1
I_2Zn	6	2.9206	1	1
Te_2Zn	6	0.0037	1	1
S_2W	6	0.2686	1	1
Bi_2Pd	6	0.1435	1	1
GeI_2	6	3.0145	1	1
N_2W	6	1.5339	1	1
Br_2Mn	6	0.0074	1	1
$CrTe_2$	6	0.0037	1	1
PtS_2	6	0.0027	1	1
CdClO	6	0.0054	1	1
Ba_2N	6	0.4775	1	1
Br_2Ti	6	0.0043	1	1
Te_2Zr	6	0.4748	1	1
Te_2W	6	0.0041	1	1
$AsSe_2$	6	0.0083	1	1
Cl_2V	6	0.2745	1	1
OTl_2	6	0.0056	1	1
BrNZr	6	0.007	1	1

Table showing the first 50 lattice matching combinations with the smallest number of atoms in the supercell.

Lattice matching - minimal strain

Formula	N° atoms	strain	cell size 1	cell size 2
$CeLi_2P_2$	327	0.0	49	36
Li_2Tl_2	496	0.0001	100	49
Br_2Er_2	388	0.0001	64	49
$I_2La_2Si_2$	258	0.0001	36	25
S_2V	543	0.0001	81	100
Br_2Ca_3Si	405	0.0001	61	37
As_2Li_2Pr	233	0.0002	36	25
InSe	158	0.0002	36	25
Cd_2I_3	155	0.0002	25	16
$Br_2Ca_2H_2$	483	0.0002	65	48
Cl_2Tb_2	624	0.0002	100	81
$Ba_2H_2I_2$	237	0.0002	39	20
AsI_2La_2	155	0.0002	25	16
CoH_2O_2	467	0.0002	49	64
$Ca_2Mn_2Si_2$	483	0.0003	65	48
Au_2Br_2	506	0.0003	94	56
$Gd_2I_2S_2$	405	0.0003	61	37
Cu_3Se_3	627	0.0003	81	64
CrO_2	390	0.0004	49	81
Br_2Zr_2	7	0.0004	1	1
Br_2HLa	291	0.0004	49	36
$Br_2Ho_2O_2$	483	0.0004	65	48
Gd	205	0.0004	43	76
Te_2Zr	435	0.0005	81	64
Br_2PY_2	504	0.0005	73	57
$AsLi_3$	208	0.0005	36	25
Br_2Gd_2	388	0.0005	64	49
I_2Zn	300	0.0005	57	43
As_2Li_2Nd	233	0.0005	36	25
Cl_2Gd_2	624	0.0005	100	81
As_2Sn_2	388	0.0006	64	49
Ba_2N	435	0.0006	81	64
$F_2Ho_2Se_2$	561	0.0006	73	57
FeH_2O_2	353	0.0006	36	49
MoS_2	543	0.0006	81	100
$AuTe_2$	339	0.0006	64	49
In_2Se_2	343	0.0006	57	43
I_2La_2Te	488	0.0006	81	49
$Ge_2Mn_2Sr_2$	840	0.0006	118	81
Br_2Cd	390	0.0006	73	57
BH_4Li	363	0.0007	49	36
S_2W	543	0.0007	81	100
I_2Y_2	624	0.0007	100	81
GeI_2Y_2	233	0.0007	36	25
Ag_2Te_2	685	0.0008	115	85
Br_2Ca_3Si	537	0.0008	81	49
NiO_2	123	0.0008	16	25
MoS_2	543	0.0008	81	100
$F_2Se_2Tm_2$	711	0.0008	91	73
$AsCuLi_2$	291	0.0009	49	36

Table showing the first 50 lattice matching combinations with the smallest strain needed to build the supercell.

$TiTe_2$ (P-3m1)

Structural and electronic properties

**Formula**	$TiTe_2$
**Spacegroup**	P-3m1
**Prototype**	CdI2
**Parent 3D**	$TiTe_2$
**Source DB**	COD
**DB ID**	1008063
**DF2-C09 Binding energy [meV/ Å^2]**	28.09
**RVV10 Binding energy [meV/ Å^2]**	30.16
**Band gap (PBE) [eV]**	N/A

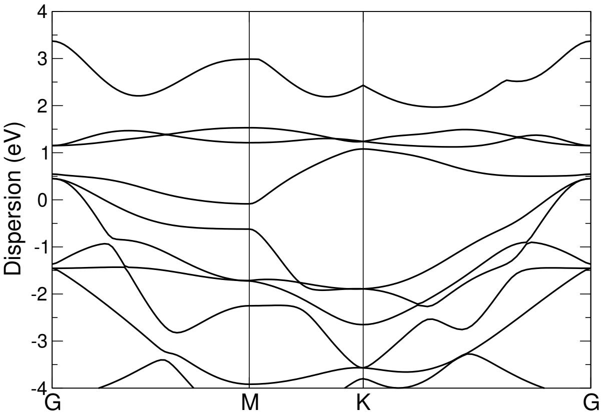

Band structure: Electronic band structure of $TiTe_2$ (P-3m1) in a representative window around the Fermi energy and along a high-symmetry path.

Optimized crystal structure

Structural parameters: relaxed cell (top) and atomic positions (bottom) of $TiTe_2$ (P-3m1) in Cartesian coordinates.

	x [Å]	y [Å]	z [Å]
$\mathbf{a_1}$	3.76380409	0.00000000	0.00000000
$\mathbf{a_2}$	−1.88190205	3.25954996	0.00000000
$\mathbf{a_3}$	0.00000000	0.00000000	23.44574105

		x [Å]	y [Å]	z [Å]
●	Te	0.00000000	2.17303331	9.98563052
●	Ti	0.00000000	0.00000000	11.72287053
●	Te	1.88190205	1.08651665	13.46011053

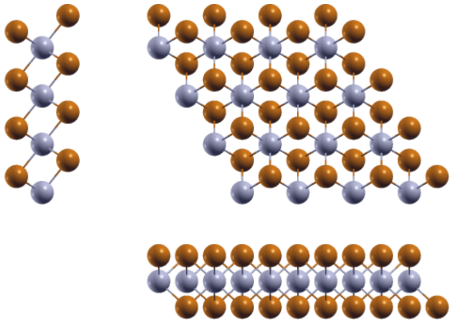

Orthographic projections: views of $TiTe_2$ (P-3m1) as seen from the x axis (left), the y axis (bottom) and the z axis (center).

Lattice matching - minimal size

Formula	N° atoms	strain	cell size 1	cell size 2
Tl	4	0.1118	1	1
Tl	4	0.2545	1	1
InSe	5	0.4791	1	1
HgO	5	0.1169	1	1
Bi_2	5	0.4942	1	1
P_2	5	0.2476	1	1
PbTe	5	0.4837	1	1
CaCl	5	0.1528	1	1
I_2Mg	6	0.4532	1	1
Cl_2Mn	6	0.2625	1	1
CdI_2	6	2.8343	1	1
$AgTe_2$	6	0.1129	1	1
$MoSe_2$	6	0.2535	1	1
S_2Ta	6	0.2654	1	1
Br_2Zn	6	0.0003	1	1
Br_2Ca	6	2.8498	1	1
$AsSn_2$	6	0.0071	1	1
$SiTe_2$	6	0.0038	1	1
Br_2La	6	0.454	1	1
PbS_2	6	0.0074	1	1
BiClTe	6	2.8389	1	1
Cl_2Ti	6	0.2478	1	1
BrCdI	6	0.4609	1	1
S_2Ti	6	0.2726	1	1
NbS_2	6	0.2648	1	1
BaF_2	6	0.4693	1	1
$RhTe_2$	6	0.0043	1	1
Bi_2Pd	6	0.1219	1	1
GeI_2	6	0.4486	1	1
Cl_2Co	6	0.2721	1	1
NbS_2	6	0.2587	1	1
Cl_2Fe	6	0.271	1	1
S_2Ta	6	0.2575	1	1
Se_2V	6	0.2557	1	1
AsKSn	6	0.4657	1	1
$PbTe_2$	6	0.4585	1	1
$NiTe_2$	6	0.0032	1	1
I_2V	6	0.0052	1	1
Cl_2V	6	1.5347	1	1
GeI_2	6	0.4843	1	1
Se_2Zr	6	0.0043	1	1
STl_2	6	0.4717	1	1
$PtSe_2$	6	0.0064	1	1
BiITe	6	2.9961	1	1
CdO_2	6	0.2719	1	1
GeS_2	6	0.1416	1	1
$TaTe_2$	6	0.0076	1	1
$MnSe_2$	6	0.1527	1	1
Cl_2Zr	6	0.2716	1	1
Se_2Yb	6	0.4492	1	1

Table showing the first 50 lattice matching combinations with the smallest number of atoms in the supercell.

Lattice matching - minimal strain

Formula	N° atoms	strain	cell size 1	cell size 2
BN	30	0.0	4	9
$HfSe_2$	6	0.0	1	1
Br_3Cs	343	0.0	81	25
BiITe	255	0.0	49	36
H_2MnO_2	552	0.0001	49	81
BrCdI	543	0.0001	100	81
Bi_2Te_3	327	0.0001	49	36
Bi_2SeTe_2	437	0.0001	64	49
SSb_2Te_2	705	0.0001	100	81
HN_3OZn	531	0.0001	49	64
$Cl_2H_2Zr_2$	678	0.0002	64	81
Bi_2Se_2Te	563	0.0002	81	64
Se_2V	390	0.0002	57	73
PbTe	333	0.0002	73	57
DyI_2	183	0.0002	36	25
As_2CeLi_2	504	0.0002	73	57
NiO_2	75	0.0002	9	16
Bi_2O_2	387	0.0002	65	48
$I_2S_2Tb_2$	258	0.0002	36	25
Br_2Zn	6	0.0003	1	1
GeI_2Y_2	563	0.0003	81	64
NbS_2	492	0.0003	73	91
CoO_2	75	0.0003	9	16
Cl_2Hf_2	643	0.0003	81	100
BiBrTe	300	0.0003	57	43
GeI_2	390	0.0004	73	57
CCl_2Gd_2	8	0.0004	1	1
NbS_2	435	0.0004	64	81
Bi_2	290	0.0004	64	49
$CrSe_2$	255	0.0004	36	49
$MnNaTe_2$	624	0.0005	100	81
AsKSn	492	0.0005	91	73
Sb_2SeTe_2	563	0.0005	81	64
As_2Li_2Nd	563	0.0005	81	64
H_2MnO_2	416	0.0006	37	61
C_4Ca_2	831	0.0006	113	82
In_2Te_3	563	0.0006	81	64
H_2Li_2Pt	74	0.0006	8	10
Bi_2STe_2	504	0.0006	73	57
Cu_2Sr_2	343	0.0007	57	43
Tl	244	0.0007	57	73
$Br_2O_2Ti_2$	375	0.0007	45	40
$Br_2La_2O_2$	483	0.0007	65	48
$Eu_2F_2I_2$	483	0.0007	65	48
$LiMnTe_2$	447	0.0007	73	57
$Ga_2I_2Y_2$	711	0.0007	91	73
$LiOS_2Ti$	597	0.0007	64	81
$Br_2Cr_2O_2$	435	0.0008	49	48
S_2Ta	492	0.0008	73	91
LiO	206	0.0008	36	49

Table showing the first 50 lattice matching combinations with the smallest strain needed to build the supercell.

Tl_2O (P-3m1)

Structural and electronic properties

**Formula**	Tl_2O
**Spacegroup**	P-3m1
**Prototype**	CdI2
**Parent 3D**	Tl_4O_2
**Source DB**	COD
**DB ID**	1528007
**DF2-C09 Binding energy [meV/ Å^2]**	19.37
**RVV10 Binding energy [meV/ Å^2]**	27.27
**Band gap (PBE) [eV]**	1.01

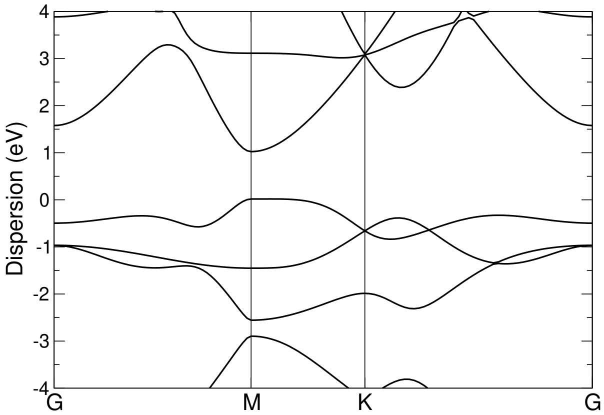

Band structure: Electronic band structure of Tl_2O (P-3m1) in a representative window around the Fermi energy and along a high-symmetry path.

Optimized crystal structure

Structural parameters: relaxed cell (top) and atomic positions (bottom) of Tl_2O (P-3m1) in Cartesian coordinates.

		x [Å]	y [Å]	z [Å]
$\mathbf{a_1}$		3.57765780	0.00000000	0.00000000
$\mathbf{a_2}$		−1.78882890	3.09834254	0.00000000
$\mathbf{a_3}$		0.00000000	0.00000000	23.01060233
		x [Å]	y [Å]	z [Å]
●	Tl	1.78882890	1.03278085	13.03254833
•	O	1.78882890	3.09834254	11.50735739
●	Tl	-0.00000000	2.06556169	9.98199496

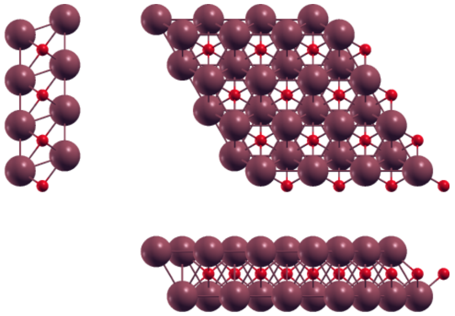

Orthographic projections: views of Tl_2O (P-3m1) as seen from the x axis (left), the y axis (bottom) and the z axis (center).

Lattice matching - minimal size

Formula	N° atoms	strain	cell size 1	cell size 2
K	4	0.3189	1	1
Tl	4	0.1232	1	1
Sn	4	0.4316	1	1
Na	4	0.0038	1	1
In	4	0.2553	1	1
HgO	5	0.1313	1	1
As_2	5	0.0041	1	1
LiO	5	0.2701	1	1
Mg_2	5	0.1148	1	1
Sb_2	5	0.5001	1	1
CrS_2	6	1.5433	1	1
Cl_2Zn	6	0.003	1	1
I_2Mg	6	2.966	1	1
S_2V	6	0.2596	1	1
MoS_2	6	0.2607	1	1
$MoTe_2$	6	0.0016	1	1
$AgTe_2$	6	0.1251	1	1
HfS_2	6	0.0069	1	1
$HfTe_2$	6	0.4597	1	1
Te_2V	6	0.0003	1	1
$CuTe_2$	6	0.0065	1	1
Br_2La	6	2.9705	1	1
Br_2Co	6	0.0037	1	1
ReS_2	6	1.5868	1	1
Ca_2N	6	0.0046	1	1
$AuTe_2$	6	0.4794	1	1
$PdTe_2$	6	0.4731	1	1
Mg_3	6	0.1199	1	1
I_2Zn	6	2.8498	1	1
Te_2Zn	6	0.0018	1	1
S_2W	6	0.2608	1	1
Bi_2Pd	6	0.1388	1	1
Br_2Mn	6	0.0018	1	1
$CrTe_2$	6	0.0091	1	1
PtS_2	6	0.0028	1	1
$CoTe_2$	6	0.0072	1	1
CdClO	6	0.0002	1	1
Ba_2N	6	0.4635	1	1
Se_2Ti	6	0.0054	1	1
AsKSn	6	3.0331	1	1
Te_2Zr	6	0.461	1	1
Te_2W	6	0.0015	1	1
Cl_2Cu	6	0.5921	1	1
Cl_2V	6	0.2665	1	1
Br_2Fe	6	0.0037	1	1
Br_2Ni	6	0.0089	1	1
MoS_2	6	0.261	1	1
Cl_2Mg	6	0.009	1	1
$CrSe_2$	6	0.2647	1	1
$PtTe_2$	6	0.4784	1	1

Table showing the first 50 lattice matching combinations with the smallest number of atoms in the supercell.

Lattice matching - minimal strain

Formula	N° atoms	strain	cell size 1	cell size 2
$Cl_2Hf_2N_2$	9	0.0	1	1
$F_2Lu_2Se_2$	486	0.0	64	49
Br_2Ho_2	499	0.0	81	64
$Ga_2Gd_2I_2$	258	0.0	36	25
BH_4Li	429	0.0001	57	43
Te_2Zr	543	0.0001	100	81
In	244	0.0002	57	73
$PbTe_2$	255	0.0002	49	36
CdClO	6	0.0002	1	1
$SnTe_2$	300	0.0002	57	43
F_2Zn	339	0.0002	65	48
GeI_2La_2	155	0.0002	25	16
$Br_2Gd_2O_2$	483	0.0002	65	48
S_2V	435	0.0002	64	81
$Ga_2I_2Tb_2$	258	0.0002	36	25
N_2Re	123	0.0003	16	25
As_2Sn_2	447	0.0003	73	57
$F_2Ho_2Se_2$	711	0.0003	91	73
Bi_2S_3	563	0.0003	81	64
$CrSe_2$	492	0.0003	73	91
Te_2V	6	0.0003	1	1
Br_2Gd_2	447	0.0004	73	57
ClH_3O	93	0.0004	16	9
Au_2I_2	552	0.0004	104	60
FHOZn	583	0.0004	73	91
$MnNaTe_2$	291	0.0004	49	36
$CrSe_2$	543	0.0005	81	100
Bi_2	158	0.0005	36	25
I_2Tm	123	0.0005	25	16
CrS_2	255	0.0005	36	49
$Br_2Ca_2F_2$	483	0.0005	65	48
Br_2Tb_2	499	0.0005	81	64
In_2Se_3	705	0.0006	100	81
$HfTe_2$	543	0.0006	100	81
$C_2Br_2Tb_2$	156	0.0006	20	16
Br_2Ca	183	0.0006	36	25
H_2MgO_2	597	0.0006	64	81
Cu_3Se_3	786	0.0007	100	81
$Ba_2Ge_2Mn_2$	840	0.0007	118	81
$CeLi_2P_2$	386	0.0008	57	43
ReS_2	300	0.0008	43	57
$C_2Br_2Gd_2$	156	0.0008	20	16
SSb_2Te_2	327	0.0008	49	36
Cl_2La_2	447	0.0008	73	57
Cl_2V	543	0.0008	81	100
$Er_2F_2Se_2$	786	0.0008	100	81
BrCdI	255	0.0008	49	36
BN	275	0.0008	39	79
$I_2Lu_2O_2$	483	0.0008	65	48
$I_2Nd_2S_2$	537	0.0008	81	49

Table showing the first 50 lattice matching combinations with the smallest strain needed to build the supercell.

Tl_2S (P-3m1)

Structural and electronic properties

**Formula**	Tl_2S
**Spacegroup**	P-3m1
**Prototype**	CdI2
**Parent 3D**	Tl_2S
**Source DB**	COD
**DB ID**	9012278
**DF2-C09 Binding energy [meV/ Å^2]**	19.16
**RVV10 Binding energy [meV/ Å^2]**	N/A
**Band gap (PBE) [eV]**	1.39

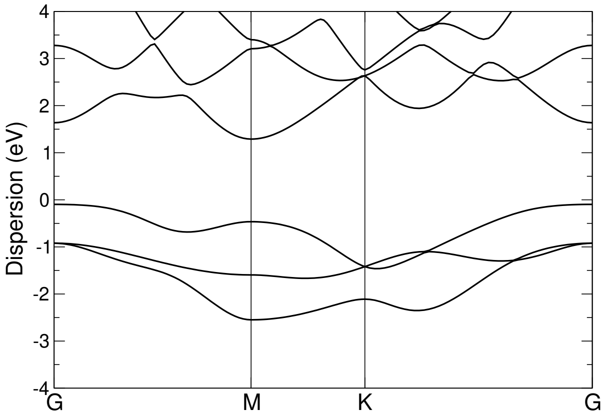

Band structure: Electronic band structure of Tl_2S (P-3m1) in a representative window around the Fermi energy and along a high-symmetry path.

Optimized crystal structure

Structural parameters: relaxed cell (top) and atomic positions (bottom) of Tl_2S (P-3m1) in Cartesian coordinates.

		x [Å]	y [Å]	z [Å]
$\mathbf{a_1}$		4.21920101	0.00000000	0.00000000
$\mathbf{a_2}$		−2.10960051	3.65393526	0.00000000
$\mathbf{a_3}$		0.00000000	0.00000000	23.24144019
		x [Å]	y [Å]	z [Å]
●	Tl	2.10960051	1.21797842	13.30143563
●	Tl	0.00000000	2.43595684	9.94000456
•	S	0.00000000	0.00000000	11.62072010

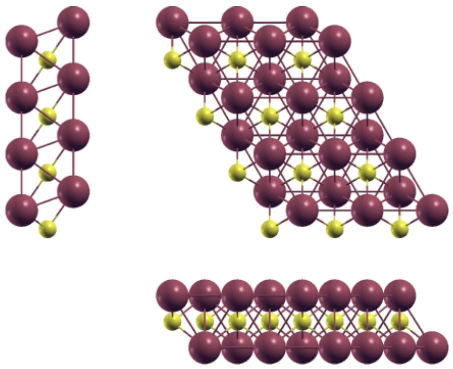

Orthographic projections: views of Tl_2S (P-3m1) as seen from the x axis (left), the y axis (bottom) and the z axis (center).

Lattice matching - minimal size

Formula	N° atoms	strain	cell size 1	cell size 2
InSe	5	0.0029	1	1
Bi_2	5	0.0088	1	1
AgTl	5	0.1553	1	1
As_2	5	1.5396	1	1
PbTe	5	0.0047	1	1
CaCl	5	0.1146	1	1
Cl_2Zn	6	1.5322	1	1
I_2Mg	6	0.0073	1	1
CdI_2	6	0.0065	1	1
PSn_2	6	0.2468	1	1
Br_2Zn	6	0.2612	1	1
Br_2Ca	6	0.0077	1	1
HfS_2	6	1.5593	1	1
$AsSn_2$	6	1.6329	1	1
$SiTe_2$	6	0.2671	1	1
I_2Pr	6	0.1388	1	1
S_2Zr	6	1.5989	1	1
Br_2La	6	0.007	1	1
Br_2Cu	6	0.7286	1	1
Ca_2Si	6	0.457	1	1
PbS_2	6	0.2722	1	1
Br_2Co	6	1.5365	1	1
BiClTe	6	0.0068	1	1
Ca_2N	6	1.5434	1	1
BrCdI	6	0.0042	1	1
Cl_2Zn	6	0.1219	1	1
Te_2Ti	6	0.2616	1	1
BaF_2	6	0.0009	1	1
$RhTe_2$	6	0.2556	1	1
GeI_2	6	0.0091	1	1
Br_2Mn	6	1.5236	1	1
$CrTe_2$	6	1.4469	1	1
$CoTe_2$	6	1.5616	1	1
AsKSn	6	0.0024	1	1
$PbTe_2$	6	0.0052	1	1
I_2Nd	6	0.1396	1	1
$NiTe_2$	6	0.2662	1	1
Cl_2Cu	6	0.0667	1	1
S_2Sn	6	0.2462	1	1
I_2V	6	0.269	1	1
GeI_2	6	0.0049	1	1
Se_2Zr	6	0.2677	1	1
I_2Pb	6	0.4504	1	1
$PtSe_2$	6	1.6378	1	1
Br_2Fe	6	1.537	1	1
$MnSe_2$	6	0.1146	1	1
CeI_2	6	0.1382	1	1
$NbTe_2$	6	1.5974	1	1
Se_2Yb	6	0.0088	1	1
$BiTe_2$	6	0.0086	1	1

Table showing the first 50 lattice matching combinations with the smallest number of atoms in the supercell.

Lattice matching - minimal strain

Formula	N° atoms	strain	cell size 1	cell size 2
CS_2Ta_2	416	0.0	37	61
Se_2Zr	543	0.0001	81	100
$Cl_2Hg_2N_2$	561	0.0001	91	48
I_4Sr_2	435	0.0001	81	32
Cl_2Ti	294	0.0001	37	61
$RhTe_2$	390	0.0001	57	73
$Cl_2Er_2O_2$	843	0.0001	81	100
CNb_2S_2	552	0.0001	49	81
$Br_2Hf_2N_2$	402	0.0001	36	49
Cl_2Y_2	516	0.0001	64	81
S_2Ta	123	0.0002	16	25
F_2Na	492	0.0002	73	91
$LiNbS_2$	148	0.0002	16	25
P_2	233	0.0002	37	61
$Cu_2K_2Te_2$	483	0.0003	65	48
$Cl_2O_2Yb_2$	765	0.0003	73	91
NbS_2	123	0.0003	16	25
$SiTe_2$	543	0.0004	81	100
$SbSe_2Tl$	416	0.0004	72	50
As_2O_3	488	0.0004	81	49
Ca_2N	255	0.0005	36	49
$BrKO_3$	120	0.0005	25	9
CBr_2Lu_2	414	0.0006	43	57
Ga_2Se_2	516	0.0006	64	81
$F_2Se_2Y_2$	429	0.0006	57	43
$Cl_2O_2Tm_2$	765	0.0006	73	91
In	43	0.0006	9	16
$Br_2N_2Zr_2$	471	0.0006	43	57
FeO_2	39	0.0006	4	9
CdClHO	463	0.0006	57	73
$Cl_2N_2Zr_2$	402	0.0007	36	49
$NbTe_2$	300	0.0007	43	57
CBr_2Y_2	597	0.0007	64	81
CaClHO	583	0.0007	73	91
CNb_2S_2	416	0.0008	37	61
CdClHO	403	0.0008	49	64
$Hf_2I_2N_2$	531	0.0008	49	64
ClH_3O	504	0.0008	73	57
La_2S_2	38	0.0009	6	5
S_2Zr	300	0.0009	43	57
N_2W	222	0.0009	25	49
CaH_2O_2	353	0.0009	36	49
HN_3OZn	477	0.0009	37	61
BaF_2	6	0.0009	1	1
$NiTe_2$	543	0.001	81	100
I_2V	543	0.001	81	100
Br_2Pr_2	583	0.001	73	91
As_2	206	0.001	36	49
As_2O_3	368	0.001	61	37
$CrTe_2$	183	0.0011	25	36

Table showing the first 50 lattice matching combinations with the smallest strain needed to build the supercell.

$Tl_2S_2I_2$ (P4/nmm)

Structural and electronic properties

**Formula**	$Tl_2S_2I_2$
**Spacegroup**	P4/nmm
**Prototype**	PbClF
**Parent 3D**	$I_2S_2Tl_2$
**Source DB**	MPDS
**DB ID**	S545244
**DF2-C09 Binding energy [meV/ Å^2]**	19.81
**RVV10 Binding energy [meV/ Å^2]**	N/A
**Band gap (PBE) [eV]**	0.0

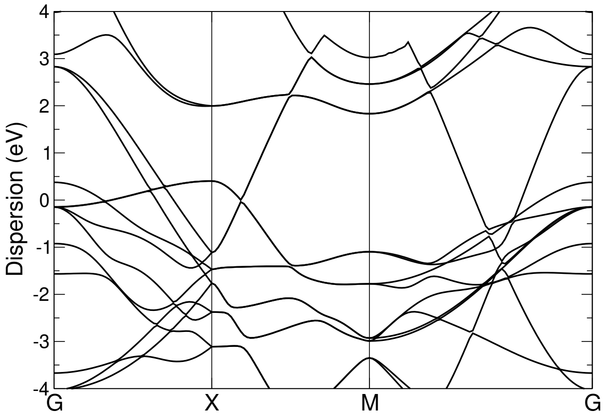

Band structure: Electronic band structure of $Tl_2S_2I_2$ (P4/nmm) in a representative window around the Fermi energy and along a high-symmetry path.

Optimized crystal structure

Structural parameters: relaxed cell (top) and atomic positions (bottom) of $Tl_2S_2I_2$ (P4/nmm) in Cartesian coordinates.

	x [Å]	y [Å]	z [Å]
$\mathbf{a_1}$	3.68316685	−0.00000006	0.00000000
$\mathbf{a_2}$	−0.00000006	3.68316685	0.00000000
$\mathbf{a_3}$	0.00000000	0.00000000	28.83788907

		x [Å]	y [Å]	z [Å]
●	Tl	0.92078992	-0.92078992	2.90385879
●	I	-0.92079009	-2.76237670	5.15701544
●	Tl	-0.92078986	-2.76237692	-2.90385879
●	S	-0.92079636	-0.92079636	0.00000000
●	S	0.92079642	-2.76237049	0.00000000
●	I	0.92079014	-0.92079014	-5.15701544

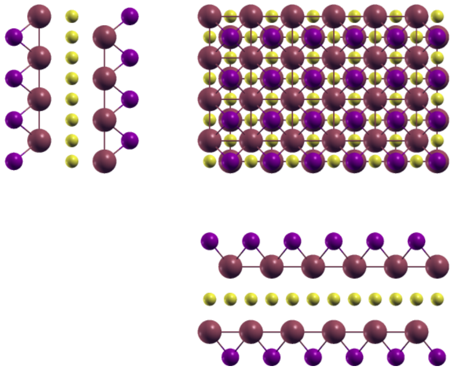

Orthographic projections: views of $Tl_2S_2I_2$ (P4/nmm) as seen from the x axis (left), the y axis (bottom) and the z axis (center).

Lattice matching - minimal size

Formula	N° atoms	strain	cell size 1	cell size 2
K	7	1.1752	1	1
Tl	7	0.5688	1	1
InSe	8	0.1746	1	1
HgO	8	0.2082	1	1
AsSb	8	0.13	1	1
Bi_2	8	0.1816	1	1
GeTe	8	0.1346	1	1
AgTl	8	0.3852	1	1
S_2	8	0.1358	1	1
PbTe	8	0.1767	1	1
Sb_2	8	0.157	1	1
$IrTe_2$	9	0.1353	1	1
CrS_2	9	0.1092	1	1
I_2Mg	9	0.163	1	1
$CdCl_2$	9	0.1337	1	1
CdI_2	9	0.1788	1	1
Br_2Ca	9	0.1803	1	1
CaI_2	9	0.7832	1	1
$InSe_2$	9	0.134	1	1
$GeTe_2$	9	0.133	1	1
$HfTe_2$	9	0.1422	1	1
I_2Mn	9	0.1338	1	1
Br_2La	9	0.1634	1	1
Br_2Cu	9	0.1223	1	1
NSr_2	9	0.1312	1	1
I_2Yb	9	0.7734	1	1
BiClTe	9	0.1793	1	1
$AuTe_2$	9	0.1492	1	1
BrCdI	9	0.1664	1	1
Cl_2Zn	9	0.0037	1	1
$PdTe_2$	9	0.1469	1	1
FeI_2	9	0.1322	1	1
I_2Ni	9	0.1331	1	1
CrI_2	9	0.1318	1	1
I_2Zn	9	0.1537	1	1
BaF_2	9	0.1702	1	1
BiBrTe	9	0.1862	1	1
Bi_2Pd	9	0.2189	1	1
GeI_2	9	0.161	1	1
Ba_2Hg	9	0.4051	1	1
N_2W	9	0.1107	1	1
Ba_2N	9	0.1435	1	1
Br_2Ti	9	0.109	1	1
AsKSn	9	0.1685	1	1
Te_2Zr	9	0.1426	1	1
$PbTe_2$	9	0.1653	1	1
Cl_2Cu	9	0.1085	1	1
I_2Tm	9	0.7786	1	1
$SnTe_2$	9	0.1586	1	1
GeI_2	9	0.177	1	1

Table showing the first 50 lattice matching combinations with the smallest number of atoms in the supercell.

Lattice matching - minimal strain

Formula	N° atoms	strain	cell size 1	cell size 2
Cu_2I_2	924	0.0	100	81
$AgCuTe_2$	560	0.0001	56	56
H_2Li_2Pd	330	0.0002	25	36
$F_2I_2Pb_2$	678	0.0002	64	49
Cu_2Se_2	10	0.0002	1	1
$Cu_2Na_2Te_2$	876	0.0002	85	61
In	670	0.0003	89	136
Ca_2O_2	896	0.0003	82	101
F_2Ni	9	0.0003	1	1
$Si_2Te_2Zr_2$	12	0.0003	1	1
Ba_2Cd	531	0.0003	64	49
H_4Ti	330	0.0004	25	36
Bi_2Pd	786	0.0004	81	100
Cl_2Fe	483	0.0005	48	65
$H_2I_2Sr_2$	510	0.0005	49	36
Mg_3	621	0.0006	61	85
Mg_4	412	0.0006	36	49
Cl_2Rb_2	358	0.0007	49	16
Bi_2Se_2	806	0.0007	89	68
$Ag_2K_2Te_2$	930	0.0008	97	58
$Ba_2H_2I_2$	246	0.0008	25	16
Cl_2Cu	426	0.0008	46	50
$C_2Br_2Y_2$	822	0.0008	68	69
Ca_2O_2	886	0.0009	81	100
HgO	968	0.0009	113	145
Fe_2Se_2	10	0.0009	1	1
Cl_2Zr	483	0.0009	48	65
Tl	265	0.0009	36	49
$I_2S_2Tb_2$	972	0.0009	101	61
N_3Na	898	0.0009	103	70
P_2	722	0.001	81	118
C_4Ca_2	606	0.001	56	45
$C_2Br_2Y_2$	714	0.001	59	60
As_2O_3	684	0.001	79	42
CdO_2	483	0.0011	48	65
Cl_2Ti	840	0.0011	81	118
Br_2CsF	214	0.0011	25	16
$C_2Br_2Y_2$	702	0.0011	58	59
Sn	121	0.0011	16	25
S_2Sn_2	712	0.0012	78	61
P_4	430	0.0012	45	40
Cl_2Co	483	0.0012	48	65
H_2Li_2Pt	878	0.0013	68	94
Ag_2F_4	690	0.0013	80	35
$Dy_2I_2S_2$	972	0.0013	101	61
Cl_4Mn	961	0.0014	106	65
F_4Pb	341	0.0014	36	25
PbS_2Sn	570	0.0014	63	48
F_4Nb	817	0.0014	82	65
$Cl_2Rh_2Te_2$	84	0.0014	9	5

Table showing the first 50 lattice matching combinations with the smallest strain needed to build the supercell.

TlF (P4/nmm)

Structural and electronic properties

**Formula**	TlF
**Spacegroup**	P4/nmm
**Prototype**	Massicot
**Parent 3D**	Tl_2F_2
**Source DB**	COD
**DB ID**	1520825
**DF2-C09 Binding energy [meV/ Å^2]**	14.14
**RVV10 Binding energy [meV/ Å^2]**	20.07
**Band gap (PBE) [eV]**	3.59

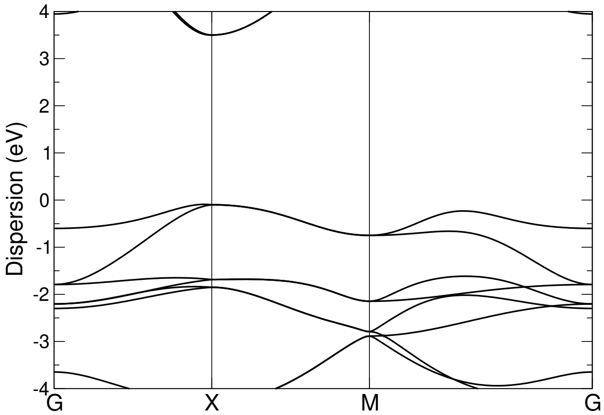

Band structure: Electronic band structure of TlF (P4/nmm) in a representative window around the Fermi energy and along a high-symmetry path.

Optimized crystal structure

Structural parameters: relaxed cell (top) and atomic positions (bottom) of TlF (P4/nmm) in Cartesian coordinates.

		x [Å]	y [Å]	z [Å]
$\mathbf{a_1}$		3.84306041	0.00000000	0.00000000
$\mathbf{a_2}$		0.00000000	3.84306041	0.00000000
$\mathbf{a_3}$		0.00000000	0.00000000	23.18225198
		x [Å]	y [Å]	z [Å]
●	Tl	1.92153020	0.00000000	13.13434372
•	F	0.00000000	1.92153020	12.53893035
●	Tl	0.00000000	1.92153020	10.04790826
•	F	1.92153020	0.00000000	10.64332163

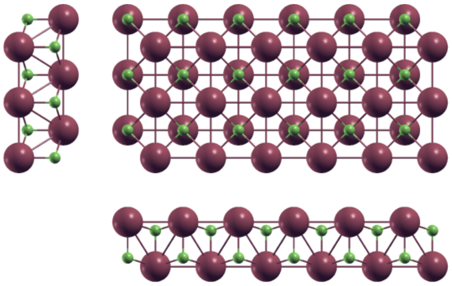

Orthographic projections: views of TlF (P4/nmm) as seen from the x axis (left), the y axis (bottom) and the z axis (center).

Lattice matching - minimal size

Formula	N° atoms	strain	cell size 1	cell size 2
K	5	0.3937	1	1
K	5	0.1939	1	1
In	5	0.1103	1	1
InSe	6	0.1529	1	1
Bi_2	6	0.1588	1	1
PbTe	6	0.1546	1	1
Sb_2	6	0.1384	1	1
I_2Mg	7	0.1433	1	1
S_2V	7	0.1096	1	1
MoS_2	7	0.1095	1	1
CdI_2	7	0.1564	1	1
Nd	7	0.7855	1	3
Br_2Ca	7	0.1576	1	1
CaI_2	7	0.1821	1	1
I_2Pr	7	0.0078	1	1
Br_2La	7	0.1436	1	1
Br_2Cu	7	0.1093	1	1
Ca_2Si	7	0.7788	1	1
I_2Yb	7	0.179	1	1
BiClTe	7	0.1567	1	1
$AuTe_2$	7	0.1322	1	1
BrCdI	7	0.1461	1	1
$PdTe_2$	7	0.1305	1	1
HgI_2	7	1.1589	1	1
I_2Zn	7	0.1358	1	1
BaF_2	7	0.1492	1	1
BiBrTe	7	0.1626	1	1
S_2W	7	0.1095	1	1
GeI_2	7	0.1416	1	1
AsKSn	7	0.1478	1	1
$PbTe_2$	7	0.1452	1	1
I_2Nd	7	0.0088	1	1
Cl_2Cu	7	0.1004	1	1
I_2Tm	7	0.1807	1	1
$SnTe_2$	7	0.1397	1	1
GeI_2	7	0.1549	1	1
I_2Pb	7	0.7684	1	1
STl_2	7	0.1501	1	1
BiITe	7	0.1698	1	1
DyI_2	7	0.1857	1	1
Br_2Ni	7	0.1086	1	1
CeI_2	7	0.007	1	1
Se_2Yb	7	0.1418	1	1
MoS_2	7	0.1094	1	1
Cl_2Mg	7	0.1086	1	1
$BiTe_2$	7	0.1421	1	1
GdI_2	7	0.1657	1	1
$CrSe_2$	7	0.1089	1	1
$PtTe_2$	7	0.1319	1	1
Br_2Cd	7	0.1298	1	1

Table showing the first 50 lattice matching combinations with the smallest number of atoms in the supercell.

Lattice matching - minimal strain

Formula	N° atoms	strain	cell size 1	cell size 2
Mg_6	316	0.0	25	36
P_2Rh_2	8	0.0001	1	1
K	393	0.0001	82	65
$As_2Mg_2Na_2$	718	0.0002	82	65
Na	257	0.0002	48	65
Br_2CsF	244	0.0003	36	25
Ga_2S_3	517	0.0003	48	65
Ag_2I_2	340	0.0004	49	36
NS_2Zr	452	0.0004	48	65
H_4Ti	189	0.0004	16	25
$Br_2Dy_2O_2$	10	0.0004	1	1
I_3Sn	52	0.0004	9	4
$Br_2O_2Y_2$	10	0.0004	1	1
$As_2Cd_2K_2$	706	0.0005	85	61
Cu_2S_2	8	0.0005	1	1
$Cl_2S_2Tl_2$	736	0.0006	97	58
La_2S_2	640	0.0006	89	71
K	388	0.0006	81	64
HgI_2	304	0.0007	49	36
Pb_2Se_2	628	0.0007	89	68
Cu_2O_2	396	0.0007	50	49
Ho_2S_2	860	0.0008	109	106
Bi_2Se_2	716	0.0009	98	81
$As_2Mg_2Na_2$	708	0.0009	81	64
H_2Li_2Pd	189	0.0009	16	25
PtS_2	387	0.001	48	65
Ho_2S_2	396	0.001	50	49
$C_2I_2La_2$	114	0.0011	12	11
O_2Sn_2	8	0.0011	1	1
C_4Ca_2	636	0.0011	69	60
P_4	284	0.0012	36	35
In	277	0.0012	49	81
C_2Li_2	196	0.0012	25	24
Se_2Sn_2	572	0.0012	80	63
$Br_2Ho_2O_2$	10	0.0013	1	1
Au_2Br_2	724	0.0014	100	81
AuI_4Li	612	0.0014	90	42
S_2Ti	678	0.0014	81	118
CNRb	891	0.0014	150	97
O_4PSn	706	0.0015	85	61
HgO	414	0.0015	61	85
Mo_2Te_4	360	0.0015	45	30
$H_2Na_2O_2$	742	0.0015	64	81
$Ca_2Mn_2Si_2$	10	0.0015	1	1
C_2	422	0.0015	44	123
$Ba_2F_2I_2$	706	0.0015	85	61
In	365	0.0015	64	109
BrNZr	427	0.0015	52	73
$Ba_2H_2I_2$	294	0.0016	36	25
$Br_2Cr_2S_2$	828	0.0016	84	82

Table showing the first 50 lattice matching combinations with the smallest strain needed to build the supercell.

$Tm_2Br_2F_2$ (P4/nmm)

Structural and electronic properties

**Formula**	$Tm_2Br_2F_2$
**Spacegroup**	P4/nmm
**Prototype**	PbClF
**Parent 3D**	$Br_2F_2Tm_2$
**Source DB**	MPDS
**DB ID**	S1800108
**DF2-C09 Binding energy [meV/ Å^2]**	25.18
**RVV10 Binding energy [meV/ Å^2]**	N/A
**Band gap (PBE) [eV]**	0.0

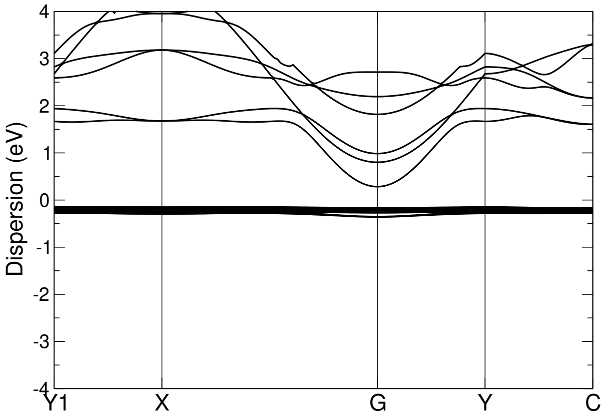

Band structure: Electronic band structure of $Tm_2Br_2F_2$ (P4/nmm) in a representative window around the Fermi energy and along a high-symmetry path.

Optimized crystal structure

Structural parameters: relaxed cell (top) and atomic positions (bottom) of $Tm_2Br_2F_2$ (P4/nmm) in Cartesian coordinates.

	x [Å]	y [Å]	z [Å]
$\mathbf{a_1}$	3.88152720	0.00012965	0.00000000
$\mathbf{a_2}$	0.00012965	3.88152720	0.00000000
$\mathbf{a_3}$	0.00000000	0.00000000	22.33126013

		x [Å]	y [Å]	z [Å]
●	Tm	0.97031880	-0.97031880	1.44124786
●	Br	-0.97041657	-2.91124028	2.82029026
●	Tm	-0.97044845	-2.91120840	-1.44124786
●	Br	0.97028692	-0.97028692	-2.82029026
•	F	-0.97041623	-0.97041623	0.00000000
•	F	0.97028658	-2.91111097	0.00000000

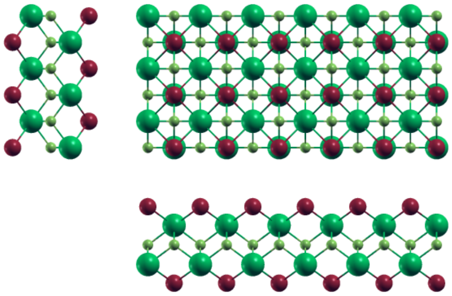

Orthographic projections: views of $Tm_2Br_2F_2$ (P4/nmm) as seen from the x axis (left), the y axis (bottom) and the z axis (center).

Lattice matching - minimal size

Formula	N° atoms	strain	cell size 1	cell size 2
K	7	0.3834	1	1
K	7	0.1878	1	1
In	7	0.1114	1	1
InSe	8	0.1483	1	1
Bi_2	8	0.1539	1	1
AgTl	8	0.0251	1	1
Ag_2	8	0.195	1	1
LiO	8	0.1091	1	1
PbTe	8	0.15	1	1
Sb_2	8	0.1346	1	1
I_2Mg	9	0.1392	1	1
S_2V	9	0.1107	1	1
MoS_2	9	0.1105	1	1
CdI_2	9	0.1517	1	1
Nd	9	0.7672	1	3
PSn_2	9	0.1085	1	1
Ba_2Pt	9	0.1946	1	1
Br_2Ca	9	0.1529	1	1
CaI_2	9	0.1764	1	1
I_2Pr	9	0.003	1	1
Br_2La	9	0.1395	1	1
Br_2Cu	9	0.1069	1	1
I_2Yb	9	0.1734	1	1
BiClTe	9	0.152	1	1
BrCdI	9	0.1419	1	1
HgI_2	9	1.1331	1	1
I_2Zn	9	0.1321	1	1
BaF_2	9	0.1448	1	1
BiBrTe	9	0.1576	1	1
S_2W	9	0.1105	1	1
Bi_2Pd	9	0.5598	1	1
GeI_2	9	0.1377	1	1
AsKSn	9	0.1435	1	1
$PbTe_2$	9	0.141	1	1
I_2Nd	9	0.004	1	1
Cl_2Cu	9	0.0992	1	1
I_2Tm	9	0.175	1	1
S_2Sn	9	0.1084	1	1
$SnTe_2$	9	0.1358	1	1
Cl_2V	9	0.1096	1	1
GeI_2	9	0.1502	1	1
I_2Pb	9	0.1971	1	1
STl_2	9	0.1457	1	1
BiITe	9	0.1645	1	1
DyI_2	9	0.1798	1	1
CeI_2	9	0.0022	1	1
Se_2Yb	9	0.1379	1	1
MoS_2	9	0.1105	1	1
$BiTe_2$	9	0.1381	1	1
GdI_2	9	0.1606	1	1

Table showing the first 50 lattice matching combinations with the smallest number of atoms in the supercell.

Lattice matching - minimal strain

Formula	N° atoms	strain	cell size 1	cell size 2
$I_2Lu_2O_2$	12	0.0001	1	1
$Ca_2Ge_2Mn_2$	12	0.0002	1	1
HgI_2	933	0.0002	113	85
$FeSe_2$	102	0.0003	9	16
$Br_2O_2V_2$	66	0.0004	5	6
K	681	0.0005	100	81
Te_2V	483	0.0006	48	65
$ReSe_2$	840	0.0006	81	118
$Ba_2F_2I_2$	510	0.0007	49	36
$Br_2Gd_2O_2$	12	0.0007	1	1
$Bi_2Cl_2O_2$	12	0.0007	1	1
H_2Na_2Pd	997	0.0007	82	101
$AgClO_4$	246	0.0008	25	16
Pd_2S_4	708	0.0008	79	39
Ca_2O_2	412	0.0009	36	49
$Cl_2Hf_2N_2$	678	0.0009	48	65
OTl_2	483	0.0009	48	65
Br_2V	840	0.001	81	118
Br_2Mn	483	0.001	48	65
F_2Zn	9	0.001	1	1
O_4PTl	510	0.001	49	36
$Br_2Cr_2S_2$	504	0.001	42	42
$Cu_2Na_2Te_2$	882	0.0011	82	65
Cl_2Rb_2	186	0.0011	25	9
CdClO	483	0.0011	48	65
Cl_2Ni	840	0.0012	81	118
Cl_4Cu_2	534	0.0012	64	25
Pb_2Se_2	292	0.0012	32	25
H_2Na_2Pd	986	0.0013	81	100
$As_2Cd_2K_2$	510	0.0013	49	36
$Br_2Ca_2F_2$	12	0.0014	1	1
KS_2Ti	548	0.0014	48	65
$Cl_2S_2Tl_2$	780	0.0014	81	49
H_2NiO_2	929	0.0014	64	109
CaI_2	885	0.0014	103	89
$Ba_2H_2I_2$	876	0.0015	85	61
$C_2I_2Y_2$	864	0.0015	70	74
$Cu_2K_2Te_2$	876	0.0015	85	61
$C_2I_2Y_2$	666	0.0015	54	57
In	655	0.0015	85	145
Br_2Ca_3Si	198	0.0015	21	12
GeS_2	795	0.0015	82	101
$NbSe_2$	840	0.0016	81	118
CaI_2	765	0.0016	89	77
CuO_2	678	0.0016	63	100
$H_2Na_2O_2$	690	0.0016	50	65
$Cl_2O_2V_2$	54	0.0016	4	5
HgO	222	0.0016	25	36
Cl_2Zr_2	958	0.0016	81	118
Se_2Ta	840	0.0016	81	118

Table showing the first 50 lattice matching combinations with the smallest strain needed to build the supercell.

$Tm_2O_2Br_2$ (P4/nmm)

Structural and electronic properties

**Formula**	$Tm_2O_2Br_2$
**Spacegroup**	P4/nmm
**Prototype**	PbClF
**Parent 3D**	$Br_2O_2Tm_2$
**Source DB**	MPDS
**DB ID**	S1903367
**DF2-C09 Binding energy [meV/ Å^2]**	15.24
**RVV10 Binding energy [meV/ Å^2]**	N/A
**Band gap (PBE) [eV]**	0.0

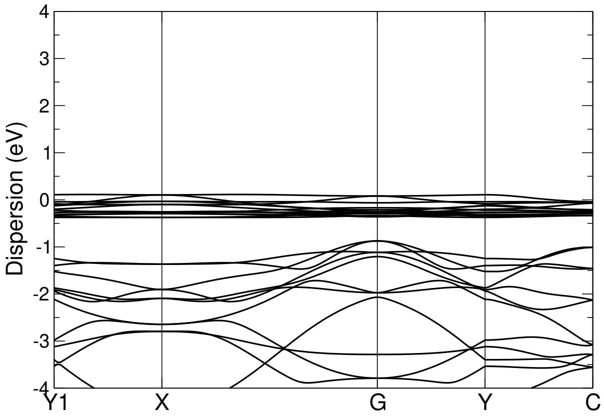

Band structure: Electronic band structure of $Tm_2O_2Br_2$ (P4/nmm) in a representative window around the Fermi energy and along a high-symmetry path.

Optimized crystal structure

Structural parameters: relaxed cell (top) and atomic positions (bottom) of $Tm_2O_2Br_2$ (P4/nmm) in Cartesian coordinates.

	x [Å]	y [Å]	z [Å]
$\mathbf{a_1}$	3.80897888	0.00047789	0.00000000
$\mathbf{a_2}$	0.00047789	3.80897888	0.00000000
$\mathbf{a_3}$	0.00000000	0.00000000	22.95162659

		x [Å]	y [Å]	z [Å]
●	Tm	0.95206872	-0.95206872	1.15007447
●	Br	-0.95241880	-2.85703798	2.81576163
●	Tm	-0.95254662	-2.85691016	-1.15007447
●	Br	0.95194090	-0.95194090	-2.81576163
•	O	-0.95237504	-0.95237504	0.00000000
•	O	0.95189714	-2.85660384	0.00000000

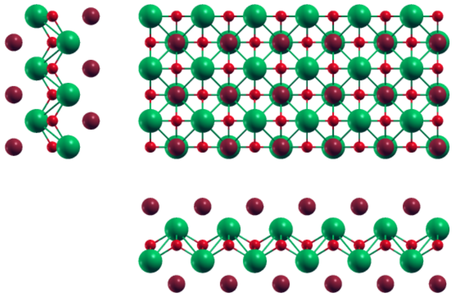

Orthographic projections: views of $Tm_2O_2Br_2$ (P4/nmm) as seen from the x axis (left), the y axis (bottom) and the z axis (center).

Lattice matching - minimal size

Formula	N° atoms	strain	cell size 1	cell size 2
K	7	0.4029	1	1
In	7	0.1095	1	1
InSe	8	0.157	1	1
Bi_2	8	0.1631	1	1
Ag_2	8	0.7784	1	1
As_2	8	0.1085	1	1
PbTe	8	0.1589	1	1
Sb_2	8	0.1419	1	1
I_2Mg	9	0.147	1	1
S_2V	9	0.1088	1	1
CdI_2	9	0.1607	1	1
Ba_2Pt	9	0.7774	1	1
Br_2Ca	9	0.162	1	1
CaI_2	9	0.1874	1	1
$HfTe_2$	9	0.1296	1	1
Br_2La	9	0.1473	1	1
Br_2Cu	9	0.1116	1	1
I_2Yb	9	0.1841	1	1
Br_2Co	9	0.1084	1	1
BiClTe	9	0.1611	1	1
ReS_2	9	0.1114	1	1
Ca_2N	9	0.1087	1	1
$AuTe_2$	9	0.1354	1	1
BrCdI	9	0.1499	1	1
$PdTe_2$	9	0.1335	1	1
I_2Zn	9	0.1391	1	1
BaF_2	9	0.1532	1	1
BiBrTe	9	0.1671	1	1
Bi_2Pd	9	0.5858	1	1
GeI_2	9	0.1453	1	1
Ba_2N	9	0.1307	1	1
AsKSn	9	0.1517	1	1
Te_2Zr	9	0.1299	1	1
$PbTe_2$	9	0.149	1	1
Cl_2Cu	9	0.1018	1	1
I_2Tm	9	0.1859	1	1
$SnTe_2$	9	0.1433	1	1
GeI_2	9	0.1591	1	1
I_2Pb	9	0.7845	1	1
STl_2	9	0.1541	1	1
BiITe	9	0.1746	1	1
Br_2Fe	9	0.1085	1	1
DyI_2	9	0.191	1	1
Se_2Yb	9	0.1455	1	1
$BiTe_2$	9	0.1457	1	1
GdI_2	9	0.1703	1	1
$PtTe_2$	9	0.1351	1	1
Br_2Cd	9	0.1328	1	1
O_2Pt	9	0.1098	1	1
CdI_2	9	0.1602	1	1

Table showing the first 50 lattice matching combinations with the smallest number of atoms in the supercell.

Lattice matching - minimal strain

Formula	N° atoms	strain	cell size 1	cell size 2
In	496	0.0002	65	106
Se_2Ta_4	870	0.0002	64	81
As_2Ru_2	10	0.0002	1	1
Bi_2Se_2	602	0.0003	65	53
K	983	0.0003	145	113
$Cu_2Na_2Te_2$	678	0.0004	64	49
$Cu_2K_2Te_2$	366	0.0004	36	25
Ca_2Cl_2	10	0.0004	1	1
$FeSe_2$	945	0.0005	85	145
H_2MnO_2	315	0.0006	20	39
$C_2I_2Y_2$	810	0.0006	67	68
$I_2S_2Tb_2$	690	0.0006	70	45
O_2Zn	411	0.0007	36	65
$H_2I_2Sr_2$	870	0.0008	81	64
Ho_2S_2	874	0.0008	89	85
Fe_2Te_2	10	0.0008	1	1
Hg_3S_2	843	0.0009	113	33
$Ag_2K_2Te_2$	246	0.001	25	16
Ag_2I_2	754	0.001	85	61
$Dy_2I_2S_2$	732	0.001	74	48
Bi_2Pd	849	0.0012	85	113
$CrTe_2$	483	0.0012	48	65
Hg_3S_2	664	0.0013	89	26
$Br_2O_2Yb_2$	12	0.0013	1	1
Pb_2Se_2	874	0.0013	97	73
Cl_2Sc_2	548	0.0013	48	65
Sn	445	0.0013	58	97
Mg_2	146	0.0013	16	25
$AgTe_2$	258	0.0014	25	36
Sn	375	0.0014	49	81
Au_2I_2	550	0.0015	61	46
$ReSe_2$	531	0.0015	52	73
$Ho_2I_2S_2$	732	0.0015	74	48
Br_2Cr	483	0.0015	48	65
$F_2Se_2Y_2$	750	0.0016	73	52
$Gd_2I_2S_2$	660	0.0016	67	43
Br_2V	531	0.0016	52	73
$AgClO_2$	866	0.0016	85	89
$H_2Na_2O_2$	984	0.0016	73	91
Tl	186	0.0016	25	36
S_2Sn	603	0.0016	62	77
S_2Zr	603	0.0016	62	77
NaPSn	975	0.0016	106	113
$Br_2H_2Zr_2$	678	0.0016	48	65
$NbTe_2$	603	0.0017	62	77
$Br_2Er_2O_2$	12	0.0017	1	1
LiO	726	0.0017	79	126
Ca_2O_2	550	0.0017	49	64
Cl_4Pd_2	462	0.0017	50	27
PSn_2	603	0.0017	62	77

Table showing the first 50 lattice matching combinations with the smallest strain needed to build the supercell.

$Tm_2O_2Cl_2$ (P-3m1)

Structural and electronic properties

**Formula**	$Tm_2O_2Cl_2$
**Spacegroup**	P-3m1
**Prototype**	SmSI
**Parent 3D**	$Cl_2O_2Tm_2$
**Source DB**	MPDS
**DB ID**	S1936407
**DF2-C09 Binding energy [meV/ Å^2]**	11.25
**RVV10 Binding energy [meV/ Å^2]**	N/A
**Band gap (PBE) [eV]**	0.0

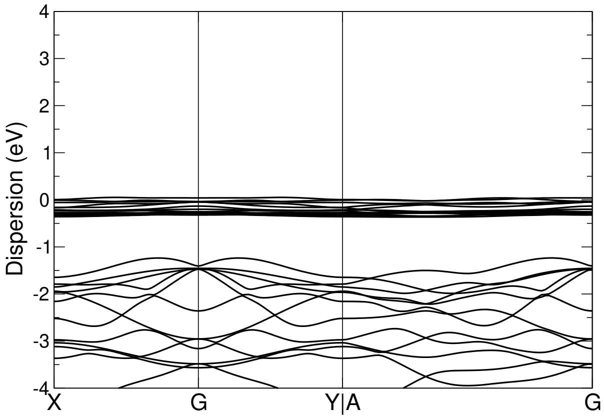

Band structure: Electronic band structure of $Tm_2O_2Cl_2$ (P-3m1) in a representative window around the Fermi energy and along a high-symmetry path.

Optimized crystal structure

Structural parameters: relaxed cell (top) and atomic positions (bottom) of $Tm_2O_2Cl_2$ (P-3m1) in Cartesian coordinates.

	x [Å]	y [Å]	z [Å]
$\mathbf{a_1}$	1.89179935	−3.27677004	0.00000000
$\mathbf{a_2}$	1.89186642	3.27680876	0.00000000
$\mathbf{a_3}$	0.00000000	0.00000000	24.51177545

		x [Å]	y [Å]	z [Å]
●	Tm	-0.94591699	0.54612543	-1.42516941
●	Tm	0.94591699	-0.54612543	1.42516941
●	Cl	0.94593000	1.63840623	-3.14549403
●	Cl	0.94593642	1.63840253	3.14549403
●	O	-0.94591730	0.54612561	0.84225525
●	O	0.94591730	-0.54612561	-0.84225525

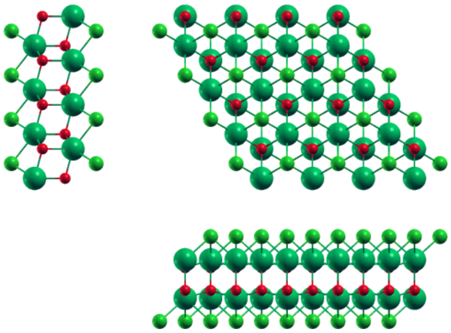

Orthographic projections: views of $Tm_2O_2Cl_2$ (P-3m1) as seen from the x axis (left), the y axis (bottom) and the z axis (center).

Lattice matching - minimal size

Formula	N° atoms	strain	cell size 1	cell size 2
Tl	7	0.111	1	1
InSe	8	0.4727	1	1
HgO	8	0.1157	1	1
AsSb	8	0.0075	1	1
PbTe	8	0.4772	1	1
CaCl	8	0.1504	1	1
I_2Mg	9	0.4471	1	1
Cl_2Mn	9	0.259	1	1
CdI_2	9	0.4817	1	1
$AgTe_2$	9	0.112	1	1
$MoSe_2$	9	0.2502	1	1
S_2Ta	9	0.2619	1	1
Br_2Zn	9	0.0028	1	1
Br_2Ca	9	0.4848	1	1
$SiTe_2$	9	0.0013	1	1
Br_2La	9	0.4479	1	1
NSr_2	9	0.0092	1	1
PbS_2	9	0.0049	1	1
BiClTe	9	0.4826	1	1
BrCdI	9	0.4548	1	1
S_2Ti	9	0.269	1	1
Mg_3	9	0.4316	1	1
Te_2Ti	9	0.0025	1	1
NbS_2	9	0.2613	1	1
BaF_2	9	0.463	1	1
BiBrTe	9	0.4972	1	1
$RhTe_2$	9	0.0067	1	1
Bi_2Pd	9	0.1205	1	1
Cl_2Co	9	0.2685	1	1
NbS_2	9	0.2553	1	1
ClNZr	9	0.2736	1	1
Cl_2Fe	9	0.2674	1	1
S_2Ta	9	0.2541	1	1
AsKSn	9	0.4594	1	1
$PbTe_2$	9	0.4524	1	1
$NiTe_2$	9	0.0007	1	1
I_2V	9	0.0027	1	1
GeI_2	9	0.4778	1	1
Se_2Zr	9	0.0018	1	1
STl_2	9	0.4654	1	1
$PtSe_2$	9	0.0088	1	1
CdO_2	9	0.2683	1	1
CoI_2	9	0.008	1	1
GeS_2	9	0.1395	1	1
$MnSe_2$	9	0.1503	1	1
Cl_2Zr	9	0.268	1	1
I_2Ti	9	0.0083	1	1
F_2Na	9	0.0008	1	1
CdI_2	9	0.4805	1	1
Se_2Sn	9	0.009	1	1

Table showing the first 50 lattice matching combinations with the smallest number of atoms in the supercell.

Lattice matching - minimal strain

Formula	N° atoms	strain	cell size 1	cell size 2
GdI_2	471	0.0	57	43
FeH_2O_2	221	0.0	16	25
CS_2Ta_2	543	0.0001	43	57
Cl_2Fe	786	0.0001	81	100
NbS_2	561	0.0001	57	73
Br_2Ca_3Si	366	0.0001	36	25
CaClHO	10	0.0001	1	1
Cl_2Ti	429	0.0001	43	57
I_2Pr	609	0.0001	73	57
$Cl_2H_2Zr_2$	780	0.0002	57	73
Cl_2Mn	627	0.0002	64	81
BiClTe	609	0.0002	73	57
Cl_2NSc_2	789	0.0003	64	81
$Br_2H_2Sr_2$	678	0.0003	65	48
Cl_2Zr	786	0.0003	81	100
P_2	372	0.0003	43	57
$Gd_2I_2S_2$	366	0.0003	36	25
Bi_2STe_2	806	0.0003	81	64
AsI_2La_2	474	0.0004	49	36
O_2Pt	258	0.0004	25	36
Cd_2I_3	474	0.0005	49	36
CdO_2	786	0.0005	81	100
Gd_2GeI_2	723	0.0005	73	57
PbTe	614	0.0005	81	64
In_2Te_3	911	0.0005	91	73
As_2CeLi_2	806	0.0005	81	64
Cl_2Hf_2	802	0.0005	73	91
Sb_2SeTe_2	911	0.0006	91	73
Li_2Tl_2	132	0.0006	16	9
STl_2	765	0.0006	91	73
CdI_2	609	0.0006	73	57
Sn_2Te_2	704	0.0006	84	50
Cl_2Co	786	0.0006	81	100
Br_2Ca	609	0.0006	73	57
Cu_4Te_2	678	0.0006	64	49
$F_2Se_2Y_2$	588	0.0006	61	37
N_4	894	0.0006	77	108
Cu_2I_2	582	0.0006	65	48
BiBrTe	531	0.0007	64	49
AsKSn	843	0.0007	100	81
Br_2Ca_3Si	846	0.0007	96	45
GeI_2	678	0.0007	81	64
$MoSe_2$	486	0.0007	49	64
$NiTe_2$	9	0.0007	1	1
BaF_2	843	0.0007	100	81
Nd	199	0.0007	25	49
CNb_2S_2	543	0.0008	43	57
F_2Na	9	0.0008	1	1
HN_3OZn	600	0.0009	43	57
$Cl_2O_2Yb_2$	12	0.0009	1	1

Table showing the first 50 lattice matching combinations with the smallest strain needed to build the supercell.

$Tm_2S_2I_2$ (Pmm2)

Structural and electronic properties

**Formula**	$Tm_2S_2I_2$
**Spacegroup**	Pmm2
**Prototype**	FeOCl
**Parent 3D**	$I_2S_2Tm_2$
**Source DB**	MPDS
**DB ID**	S1937397
**DF2-C09 Binding energy [meV/ Å^2]**	11.63
**RVV10 Binding energy [meV/ Å^2]**	N/A
**Band gap (PBE) [eV]**	0.0

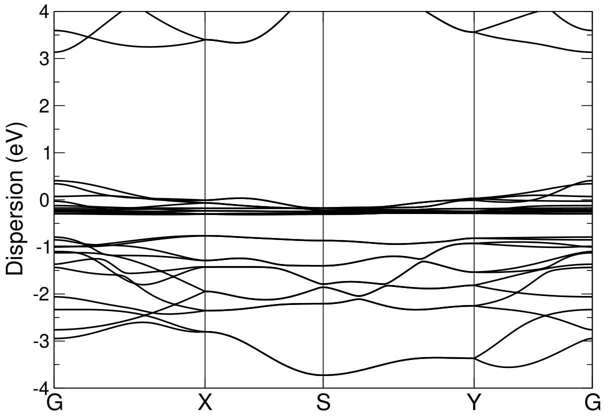

Band structure: Electronic band structure of $Tm_2S_2I_2$ (Pmm2) in a representative window around the Fermi energy and along a high-symmetry path.

Optimized crystal structure

Structural parameters: relaxed cell (top) and atomic positions (bottom) of $Tm_2S_2I_2$ (Pmm2) in Cartesian coordinates.

	x [Å]	y [Å]	z [Å]
$\mathbf{a_1}$	4.14705753	0.00000000	0.00000000
$\mathbf{a_2}$	0.00000000	5.29808972	0.00000000
$\mathbf{a_3}$	0.00000000	0.00000000	25.04462070

		x [Å]	y [Å]	z [Å]
●	Tm	1.03678393	-1.32452243	-1.10582838
●	S	1.03677071	-3.97356729	-0.65830338
●	I	-1.03671185	-1.32452243	-3.32557957
●	Tm	-1.03678393	-3.97356729	1.10582838
●	S	-1.03677071	-1.32452243	0.65830338
●	I	1.03671185	-3.97356729	3.32557957

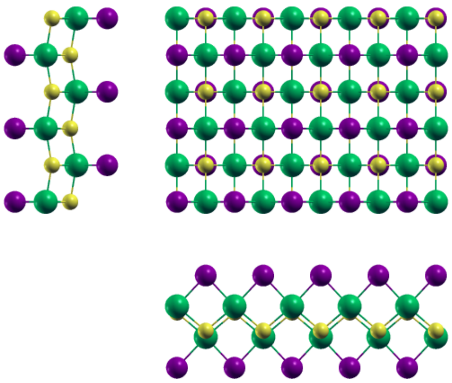

Orthographic projections: views of $Tm_2S_2I_2$ (Pmm2) as seen from the x axis (left), the y axis (bottom) and the z axis (center).

Lattice matching - minimal size

Formula	N° atoms	strain	cell size 1	cell size 2
AgTl	8	0.7547	1	1
In	8	0.1461	1	2
Br_2Zn	9	0.116	1	1
$AsSn_2$	9	0.1174	1	1
HgI_2	9	0.3126	1	1
Te_2Ti	9	0.116	1	1
$RhTe_2$	9	0.1168	1	1
Ba_2Hg	9	0.2625	1	1
CNRb	9	0.2207	1	1
CKN	9	0.0106	1	1
$PtSe_2$	9	0.1173	1	1
CNNa	9	0.2963	1	1
Ba_2Cd	9	0.7905	1	1
$HfSe_2$	9	0.116	1	1
Bi_2In_2	10	0.057	1	1
CdClHO	10	0.117	1	1
Au_2Br_2	10	0.7966	1	1
Cl_2Y_2	10	0.1163	1	1
As_4	10	0.1574	1	1
Br_3Cs	10	0.3863	1	1
$AgClO_2$	10	0.1174	1	1
PbS_2Sn	10	0.778	1	1
$SbSe_2Tl$	10	0.4785	1	1
Br_2CsF	10	0.0565	1	1
Ga_2Se_2	10	0.1164	1	1
Gd	10	0.0385	1	4
Sn_2Te_2	10	0.3291	1	1
F_4Sn	11	0.2614	1	1
FKO_2Se	11	0.5525	1	1
F_4Nb	11	0.2534	1	1
$ClKO_3$	11	0.3943	1	1
Cl_4Mn	11	0.0588	1	1
$Ba_2H_2I_2$	12	0.3347	1	1
CrS_2	12	0.1379	1	2
$Br_2Ho_2S_2$	12	0.0054	1	1
S_2V	12	0.1481	1	2
MoS_2	12	0.1486	1	2
$I_2Lu_2Se_2$	12	0.0539	1	1
$Br_2F_2Sr_2$	12	0.2511	1	1
$Ho_2I_2S_2$	12	0.0038	1	1
Cu_4Te_2	12	0.1145	1	1
AlH_4Na	12	0.0599	1	1
$GeTe_2$	12	0.6457	1	2
Br_2Ca_3Si	12	0.2629	1	1
$I_2N_2Zr_2$	12	0.1166	1	1
$Br_2S_2Y_2$	12	0.0087	1	1
S_2W	12	0.1486	1	2
$Gd_2I_2S_2$	12	0.0092	1	1
$C_2Br_2Gd_2$	12	0.1077	1	1
N_2W	12	0.1349	1	2

Table showing the first 50 lattice matching combinations with the smallest number of atoms in the supercell.

Lattice matching - minimal strain

Formula	N° atoms	strain	cell size 1	cell size 2
Bi_2Pd	240	0.0006	20	40
O_2Sn_2	666	0.0008	51	90
Fe_2Se_2	876	0.0008	70	114
As_4	708	0.001	64	81
Te_2Zn	504	0.001	42	84
PSn_2	279	0.001	24	45
Ca_2O_2	280	0.001	20	40
Cl_2Zn	657	0.001	61	97
CNNa	477	0.0011	48	63
$Cl_2Rh_2Te_2$	456	0.0011	40	36
MnO_2	288	0.0011	18	60
$K_2O_2Tl_2$	870	0.0011	75	70
$I_2S_2Yb_2$	12	0.0012	1	1
Cl_2OOs	614	0.0012	49	80
F_2Ni	762	0.0012	70	114
S_2Sn	279	0.0012	24	45
Br_2CsF	698	0.0012	69	71
$I_2N_2Zr_2$	828	0.0012	49	89
Mo_2Te_4	360	0.0012	30	30
$Br_2Hf_2N_2$	630	0.0013	42	63
Gd	48	0.0013	5	18
Bi_2Se_2	622	0.0013	57	70
S_2Zr	279	0.0013	24	45
Se_4TiZr	558	0.0013	48	45
Cl_2OOs	590	0.0014	47	77
$Ca_2Cl_2H_2$	948	0.0014	61	97
$BrKO_3$	747	0.0014	87	45
$I_2Nd_2S_2$	396	0.0014	30	36
Cl_4Cu_2	198	0.0015	21	12
$NbTe_2$	279	0.0015	24	45
$Cu_2Rb_2Te_2$	840	0.0015	69	71
Br_3Cs	730	0.0015	89	49
Sn	213	0.0015	25	63
$I_2N_2Zr_2$	972	0.0016	67	95
Cl_2Gd_2	956	0.0016	76	125
$AuCrTe_4$	354	0.0016	32	27
$RhTe_2$	561	0.0016	49	89
$Br_2H_2Zr_2$	846	0.0016	45	96
Ga_2Se_2	650	0.0016	49	89
Hf_3Te_2	786	0.0016	56	90
$Br_2O_2Sc_2$	276	0.0016	18	28
Cu_2F_4	96	0.0017	9	7
Cl_4Mn	781	0.0017	71	71
Cu_2Se_2	876	0.0017	70	114
I_2Y_2	980	0.0017	78	128
Cl_2Ni	519	0.0017	42	89
Ga_2S_3	672	0.0017	42	84
$ReSe_2$	519	0.0017	42	89
$AgClO_2$	730	0.0017	59	94
$Se_2Si_2Zr_2$	198	0.0017	12	21

Table showing the first 50 lattice matching combinations with the smallest strain needed to build the supercell.

$Tm_2Se_2F_2$ (P-3m1)

Structural and electronic properties

**Formula**	$Tm_2Se_2F_2$
**Spacegroup**	P-3m1
**Prototype**	SmSI
**Parent 3D**	$F_2Se_2Tm_2$
**Source DB**	MPDS
**DB ID**	S307728
**DF2-C09 Binding energy [meV/ Å^2]**	15.96
**RVV10 Binding energy [meV/ Å^2]**	N/A
**Band gap (PBE) [eV]**	0.0

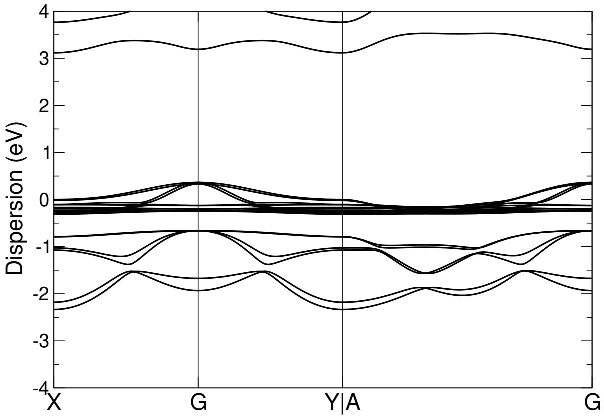

Band structure: Electronic band structure of $Tm_2Se_2F_2$ (P-3m1) in a representative window around the Fermi energy and along a high-symmetry path.

Optimized crystal structure

Structural parameters: relaxed cell (top) and atomic positions (bottom) of $Tm_2Se_2F_2$ (P-3m1) in Cartesian coordinates.

	x [Å]	y [Å]	z [Å]
$\mathbf{a_1}$	1.97060636	−3.41316167	0.00000000
$\mathbf{a_2}$	1.97058153	3.41314733	0.00000000
$\mathbf{a_3}$	0.00000000	0.00000000	25.17807099

		x [Å]	y [Å]	z [Å]
●	Tm	0.98531769	-0.56887343	-1.69987589
●	Se	0.98530731	1.70656411	-3.32150041
●	F	2.95590325	0.56884001	-0.72461252
●	Tm	2.95587021	0.56885909	1.69987589
●	Se	0.98527422	1.70658322	3.32150041
●	F	0.98528464	-0.56885435	0.72461252

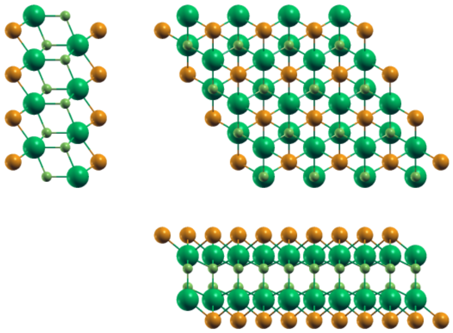

Orthographic projections: views of $Tm_2Se_2F_2$ (P-3m1) as seen from the x axis (left), the y axis (bottom) and the z axis (center).

Lattice matching - minimal size

Formula	N° atoms	strain	cell size 1	cell size 2
Na	7	0.2678	1	1
HgO	8	0.4286	1	1
GeTe	8	0.0057	1	1
Ag_2	8	3.0398	1	1
S_2	8	0.0042	1	1
CaCl	8	0.1338	1	1
$IrTe_2$	9	0.0048	1	1
$CdCl_2$	9	0.0067	1	1
$MoTe_2$	9	0.2711	1	1
Ba_2Pt	9	3.0363	1	1
$ReSe_2$	9	0.2507	1	1
$InSe_2$	9	0.0064	1	1
$GeTe_2$	9	0.0077	1	1
$HfTe_2$	9	0.0035	1	1
Te_2V	9	0.274	1	1
I_2Mn	9	0.0066	1	1
I_2Yb	9	0.4842	1	1
LiO_2	9	0.0698	1	1
Cl_2Zn	9	0.1459	1	1
$PdTe_2$	9	0.0089	1	1
FeI_2	9	0.0087	1	1
I_2Ni	9	0.0075	1	1
CrI_2	9	0.0091	1	1
Te_2Zn	9	0.2709	1	1
BiBrTe	9	0.4481	1	1
Bi_2Pd	9	0.1118	1	1
$CrTe_2$	9	0.2602	1	1
PtS_2	9	0.2693	1	1
Br_2V	9	0.2496	1	1
ClNZr	9	0.2468	1	1
CdClO	9	0.2732	1	1
Ba_2N	9	0.0051	1	1
Se_2Ti	9	0.2655	1	1
Br_2Ti	9	0.2594	1	1
Te_2Zr	9	0.004	1	1
Te_2W	9	0.2714	1	1
$AsSe_2$	9	0.2538	1	1
OTl_2	9	0.2735	1	1
BiITe	9	0.4643	1	1
BrNZr	9	0.2556	1	1
GeS_2	9	0.1253	1	1
$MnSe_2$	9	0.1337	1	1
Br_2Cr	9	0.2598	1	1
DyI_2	9	0.4984	1	1
Br_2Mg	9	0.0089	1	1
$NbSe_2$	9	0.2542	1	1
GdI_2	9	0.4551	1	1
F_2Ni	9	0.1423	1	1
Se_2Ta	9	0.2583	1	1
Br_2Cd	9	0.008	1	1

Table showing the first 50 lattice matching combinations with the smallest number of atoms in the supercell.

Lattice matching - minimal strain

Formula	N° atoms	strain	cell size 1	cell size 2
P_2	222	0.0	25	36
$Gd_2I_2S_2$	600	0.0	57	43
$Ge_2I_2La_2$	870	0.0001	81	64
Br_2Ti	627	0.0001	64	81
$AlLiTe_2$	924	0.0001	100	81
BrNZr	561	0.0001	57	73
$LiMnSe_2$	10	0.0001	1	1
Cl_2Ti	258	0.0001	25	36
$LiNbS_2$	412	0.0002	36	49
Na	586	0.0002	81	100
Ba_2Cu_2	666	0.0002	73	57
S_2Ta	363	0.0002	36	49
CS_2Ta_2	330	0.0002	25	36
FeO_2	297	0.0002	25	49
$Br_2H_2Zr_2$	870	0.0003	64	81
Br_2V	486	0.0003	49	64
KNO_3	195	0.0003	25	9
Br_2Ca_3Si	600	0.0003	57	43
Br_2Hf_2	634	0.0003	57	73
CrS_2	537	0.0003	49	81
Br_2Cr	627	0.0004	64	81
I_2Yb	609	0.0004	73	57
$Dy_2I_2S_2$	678	0.0004	64	49
O_2Pt	171	0.0004	16	25
Br_2Zr_2	802	0.0005	73	91
MnO_2	594	0.0005	49	100
$Cl_2H_2Sc_2$	870	0.0005	64	81
CNb_2S_2	330	0.0005	25	36
C_2	278	0.0005	25	64
Cl_2Zr_2	550	0.0005	49	64
Ga_2S_3	986	0.0005	81	100
Gd	402	0.0006	49	108
Cl_2Sc_2	708	0.0006	64	81
NS_2Zr	886	0.0006	81	100
C_4Ca_2	990	0.0006	92	73
$CrTe_2$	627	0.0007	64	81
NbS_2	363	0.0007	36	49
Se_2Ta	627	0.0007	64	81
I_2Y_2	10	0.0007	1	1
IKO_3	611	0.0008	81	25
Se_2Ti	711	0.0008	73	91
Li_2Tl_2	514	0.0009	61	37
CCl_2Sc_2	614	0.0009	49	64
$NbSe_2$	561	0.0009	57	73
CrS_2	405	0.0009	37	61
Cl_2Hf_2	412	0.001	36	49
CoO_2	297	0.001	25	49
$I_2Nd_2S_2$	510	0.001	49	36
BiITe	765	0.001	91	73
$ReSe_2$	486	0.0011	49	64

Table showing the first 50 lattice matching combinations with the smallest strain needed to build the supercell.

$Tm_2Se_2I_2$ (Pmm2)

Structural and electronic properties

**Formula**	$Tm_2Se_2I_2$
**Spacegroup**	Pmm2
**Prototype**	FeOCl
**Parent 3D**	$I_2Se_2Tm_2$
**Source DB**	MPDS
**DB ID**	S376102
**DF2-C09 Binding energy [meV/ Å^2]**	11.96
**RVV10 Binding energy [meV/ Å^2]**	N/A
**Band gap (PBE) [eV]**	0.0

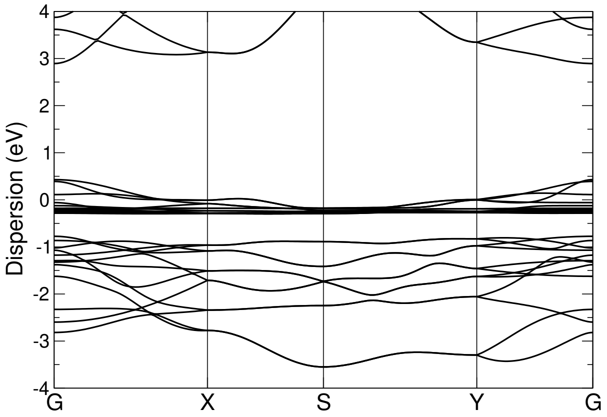

Band structure: Electronic band structure of $Tm_2Se_2I_2$ (Pmm2) in a representative window around the Fermi energy and along a high-symmetry path.

Optimized crystal structure

Structural parameters: relaxed cell (top) and atomic positions (bottom) of $Tm_2Se_2I_2$ (Pmm2) in Cartesian coordinates.

	x [Å]	y [Å]	z [Å]
$\mathbf{a_1}$	−4.23015518	0.00000000	0.00000000
$\mathbf{a_2}$	0.00000000	−5.58776901	0.00000000
$\mathbf{a_3}$	0.00000000	0.00000000	24.98736240

	x [Å]	y [Å]	z [Å]
I	-1.05743696	-1.39694225	-15.82359169
Tm	1.05754236	-1.39694225	-13.65570926
Tm	-1.05754236	1.39694225	-11.33165314
Se	1.05748974	1.39694225	-13.27050820
Se	-1.05748974	-1.39694225	-11.71685420
I	1.05743696	1.39694225	-9.16377071

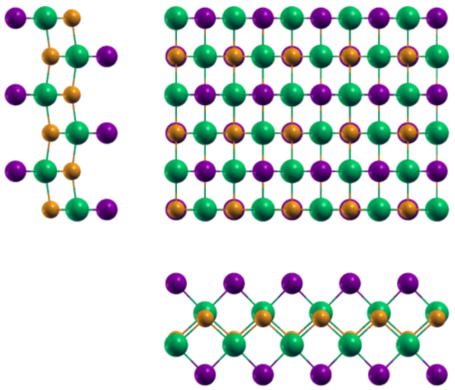

Orthographic projections: views of $Tm_2Se_2I_2$ (Pmm2) as seen from the x axis (left), the y axis (bottom) and the z axis (center).

Lattice matching - minimal size

Formula	N° atoms	strain	cell size 1	cell size 2
Tl	8	0.3357	1	2
CKN	9	0.0266	1	1
In_2Se_2	10	0.0864	1	1
Au_2Br_2	10	0.0699	1	1
Ge_2Te_2	10	0.8347	1	1
As_4	10	0.62	1	1
Au_2Se_2	10	0.2225	1	1
LiO	10	0.1372	1	2
Au_2I_2	10	0.7768	1	1
P_2	10	0.1414	1	2
$AgClO_2$	10	0.1186	1	1
La_2S_2	10	0.2701	1	1
$SbSe_2Tl$	10	0.1741	1	1
Se_2Sn_2	10	0.2725	1	1
F_4Pb	11	0.282	1	1
H_2Li_2Pt	11	0.3447	1	1
$CuGeO_3$	11	0.1286	1	1
KNO_3	11	0.3256	1	1
MoS_2	12	0.134	1	2
$Ho_2I_2S_2$	12	0.0165	1	1
$CuTe_2$	12	0.5112	1	2
PbS_2	12	0.5816	1	2
Cl_2Ti	12	0.1415	1	2
$K_2O_2Tl_2$	12	0.2143	1	1
S_2Ti	12	0.3597	1	2
$I_2Se_2Tb_2$	12	0.0061	1	1
$Gd_2I_2Se_2$	12	0.0082	1	1
S_2W	12	0.1341	1	2
$Gd_2I_2S_2$	12	0.0149	1	1
Cl_2Co	12	0.359	1	2
Pd_2S_4	12	0.3549	1	1
$Br_2Er_2Se_2$	12	0.0083	1	1
NbS_2	12	0.1466	1	2
CNRb	12	0.5273	1	2
ClNZr	12	0.3658	1	2
Cl_2Fe	12	0.3576	1	2
S_2Ta	12	0.1461	1	2
Se_2V	12	0.1452	1	2
$I_2S_2Tb_2$	12	0.0154	1	1
$Er_2I_2Se_2$	12	0.0016	1	1
Cl_2V	12	0.136	1	2
Ca_4Cu_2	12	0.0219	1	1
Cl_2Zr	12	0.3583	1	2
$Dy_2I_2S_2$	12	0.0158	1	1
MoS_2	12	0.1341	1	2
Se_4TiZr	12	0.2313	1	1
$CrSe_2$	12	0.1354	1	2
$I_2Se_2Yb_2$	12	0.0009	1	1
Te_4W_2	12	0.5141	1	1
$AgClO_4$	12	0.0664	1	1

Table showing the first 50 lattice matching combinations with the smallest number of atoms in the supercell.

Lattice matching - minimal strain

Formula	N° atoms	strain	cell size 1	cell size 2
$CuTe_2$	660	0.0002	54	112
CCl_2Gd_2	876	0.0003	56	108
$I_2Nd_2S_2$	822	0.0005	60	77
As_2Sn_2	684	0.0007	54	90
CBr_2Y_2	876	0.0008	56	108
CNRb	636	0.0008	70	72
FeO_2	228	0.0009	14	48
$I_2Se_2Yb_2$	12	0.0009	1	1
Cl_2OOs	156	0.001	12	21
Au_2Se_2	700	0.001	70	70
CaI_2	603	0.0011	60	81
Br_2Pr_2	768	0.0011	56	108
Cl_2La_2	684	0.0012	54	90
$Ag_2K_2Te_2$	648	0.0013	53	55
CNRb	573	0.0014	63	65
$TaTe_2$	672	0.0014	56	112
$AuTe_2$	594	0.0014	54	90
$Dy_2I_2S_2$	846	0.0014	60	81
Te_2V	681	0.0014	55	117
$F_2Se_2Y_2$	312	0.0015	24	28
$AgTe_2$	156	0.0015	12	28
Cl_2Zn	156	0.0015	14	24
Hg_3S_2	528	0.0015	63	30
OTl_2	681	0.0015	55	117
Ba_2Pt	591	0.0016	60	77
NSr_2	345	0.0016	30	55
CdClO	681	0.0016	55	117
$Er_2I_2Se_2$	12	0.0016	1	1
Se_2Sn	345	0.0016	30	55
DyI_2	531	0.0016	53	71
$Ca_2Cl_2H_2$	228	0.0016	14	24
$Cl_2Fe_2O_2$	108	0.0017	6	12
CI_2Y_2	455	0.0017	30	55
$C_2Br_2Y_2$	798	0.0017	48	85
CrI_2	345	0.0017	30	55
$I_2S_2Tb_2$	744	0.0017	53	71
$Dy_2I_2S_2$	744	0.0017	53	71
I_2Tm	624	0.0017	62	84
Ag_2Te_2	876	0.0018	70	114
$AuTe_2$	540	0.0018	49	82
$PtTe_2$	594	0.0018	54	90
As_4	888	0.0018	78	105
$I_2N_2Ti_2$	402	0.0018	25	42
Ag_2	514	0.0018	60	77
Br_2Mg	345	0.0019	30	55
$PtTe_2$	540	0.0019	49	82
I_2Ti	345	0.0019	30	55
In_2Se_2	708	0.0019	56	93
$AgNO_2$	998	0.0019	85	122
Br_2Mn	681	0.0019	55	117

Table showing the first 50 lattice matching combinations with the smallest strain needed to build the supercell.

TmI_2 (P-3m1)

Structural and electronic properties

**Formula**	TmI_2
**Spacegroup**	P-3m1
**Prototype**	CdI2
**Parent 3D**	TmI_2
**Source DB**	ICSD
**DB ID**	43731
**DF2-C09 Binding energy [meV/ Å^2]**	10.54
**RVV10 Binding energy [meV/ Å^2]**	16.1
**Band gap (PBE) [eV]**	N/A

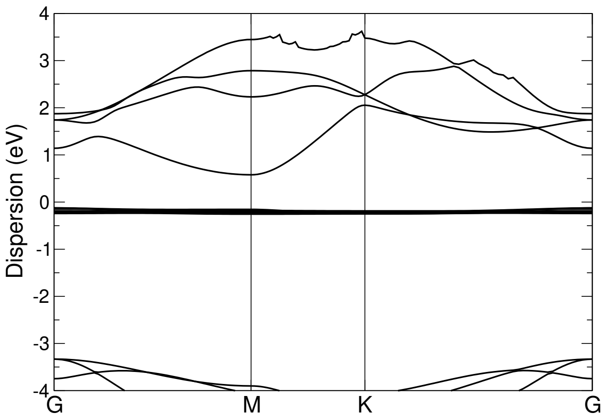

Band structure: Electronic band structure of TmI_2 (P-3m1) in a representative window around the Fermi energy and along a high-symmetry path.

Optimized crystal structure

Structural parameters: relaxed cell (top) and atomic positions (bottom) of TmI_2 (P-3m1) in Cartesian coordinates.

	x [Å]	y [Å]	z [Å]
$\mathbf{a_1}$	4.47662478	0.00000000	0.00000000
$\mathbf{a_2}$	−2.23831239	3.87687078	0.00000000
$\mathbf{a_3}$	0.00000000	0.00000000	23.60505540

		x [Å]	y [Å]	z [Å]
●	I	-0.00000000	2.58458052	13.59756800
●	Tm	0.00000000	0.00000000	11.80252770
●	I	2.23831239	1.29229026	10.00748740

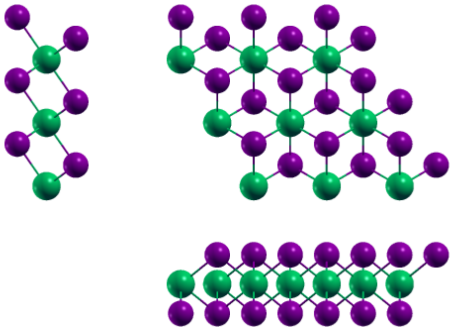

Orthographic projections: views of TmI_2 (P-3m1) as seen from the x axis (left), the y axis (bottom) and the z axis (center).

Lattice matching - minimal size

Formula	N° atoms	strain	cell size 1	cell size 2
GeTe	5	1.597	1	1
AgTl	5	0.1311	1	1
S_2	5	0.2475	1	1
In	5	0.2212	1	2
$IrTe_2$	6	0.2467	1	1
$CdCl_2$	6	1.5891	1	1
CaI_2	6	0.0012	1	1
$InSe_2$	6	1.5918	1	1
$HfTe_2$	6	0.2578	1	1
I_2Pr	6	0.1197	1	1
I_2Mn	6	1.59	1	1
NSr_2	6	1.5645	1	1
I_2Yb	6	0.0013	1	1
$AuTe_2$	6	0.2688	1	1
$PdTe_2$	6	0.2653	1	1
I_2Ni	6	1.5831	1	1
I_2Zn	6	0.2756	1	1
Ba_2Hg	6	0.1425	1	1
Ba_2N	6	0.26	1	1
Te_2Zr	6	0.2585	1	1
I_2Nd	6	0.1202	1	1
BiITe	6	0.009	1	1
BrNZr	6	4.88	1	1
CoI_2	6	1.5559	1	1
DyI_2	6	0.004	1	1
CeI_2	6	0.1193	1	1
$NbSe_2$	6	4.8575	1	1
F_2Ni	6	0.4272	1	1
$PtTe_2$	6	0.2682	1	1
Br_2Cd	6	0.264	1	1
I_2La	6	0.1229	1	1
Se_2Sn	6	1.5627	1	1
F_2Zn	6	0.1176	1	1
Ba_2Cd	6	0.1449	1	1
NaPSn	6	0.2566	1	1
H_2Si_2	7	1.5942	1	1
Fe_2Te_2	7	0.1137	1	1
Li_2Tl_2	7	0.4796	1	1
Ca_2Cl_2	7	0.1139	1	1
Cu_2I_2	7	0.134	1	1
Cl_2Gd_2	7	0.2505	1	1
Cl_2OOs	7	0.1085	1	1
Ir_2P_2	7	0.1201	1	1
Ag_2Br_2	7	0.1232	1	1
Br_2Er_2	7	0.2696	1	1
O_2Sn_2	7	0.4472	1	1
Cu_2S_2	7	0.1161	1	1
Au_2Br_2	7	0.1423	1	1
Ge_2Te_2	7	0.1582	1	1
Br_2Tb_2	7	0.2661	1	1

Table showing the first 50 lattice matching combinations with the smallest number of atoms in the supercell.

Lattice matching - minimal strain

Formula	N° atoms	strain	cell size 1	cell size 2
NaO_4	386	0.0	57	43
S_2Zn_2	403	0.0	49	64
$InSe_2$	300	0.0001	43	57
PTe_2Zr_2	353	0.0001	36	49
Bi_2Se_2	276	0.0001	40	39
$RhTe_2$	183	0.0002	25	36
Te_2V	123	0.0002	16	25
Br_2Hf_2	471	0.0002	49	81
$F_2Se_2Yb_2$	531	0.0002	49	64
Cu_3Se_3	678	0.0002	64	81
$Cl_2Er_2H_2$	471	0.0002	43	57
BrNZr	390	0.0002	49	81
H_2NiO_2	320	0.0002	25	49
$GeNi_3Te_2$	609	0.0002	57	73
H_2Si_2	357	0.0002	43	57
Br_2Cd	492	0.0002	73	91
GeI_2La_2	8	0.0003	1	1
$C_2Br_2Gd_2$	168	0.0003	16	20
I_2Mn	300	0.0003	43	57
$C_2Br_2Y_2$	573	0.0003	53	69
P_2Sn_2	403	0.0003	49	64
$C_2Br_2Tb_2$	168	0.0004	16	20
$AgClO_4$	483	0.0004	65	48
BrNZr	294	0.0004	37	61
Br_2PY_2	674	0.0004	73	91
$PtTe_2$	543	0.0004	81	100
$CdCl_2$	300	0.0004	43	57
IO_3Tl	47	0.0004	9	4
$Cl_2Hf_2N_2$	198	0.0005	16	25
Ba_2N	435	0.0005	64	81
OTl_2	123	0.0005	16	25
Br_2Ho_2	643	0.0005	81	100
GeI_3Rb	323	0.0005	81	16
$Br_2O_2V_2$	147	0.0006	13	18
Te_2Zr	435	0.0006	64	81
Cl_2Mn	75	0.0006	9	16
GeTe	243	0.0006	43	57
Ni_2Te_2	357	0.0006	43	57
CdClO	123	0.0007	16	25
$PdTe_2$	492	0.0007	73	91
Cl_2Rb_2	606	0.0007	130	54
O_2Pt	354	0.0007	39	79
$FeSe_2$	357	0.0007	39	80
Ge_2Te_2	839	0.0007	125	116
Bi_2S_3	743	0.0008	81	100
Pd_2S_4	642	0.0008	100	57
AsSb	206	0.0008	36	49
Br_2Hf_2	355	0.0008	37	61
In_2S_3	414	0.0008	43	57
$NbSe_2$	390	0.0008	49	81

Table showing the first 50 lattice matching combinations with the smallest strain needed to build the supercell.

TmOI (C2/m)

Structural and electronic properties

**Formula**	TmOI
**Spacegroup**	C2/m
**Prototype**	PbClF
**Parent 3D**	$Tm_2O_2I_2$
**Source DB**	COD
**DB ID**	2310429
**DF2-C09 Binding energy [meV/ Å^2]**	14.99
**RVV10 Binding energy [meV/ Å^2]**	21.49
**Band gap (PBE) [eV]**	N/A

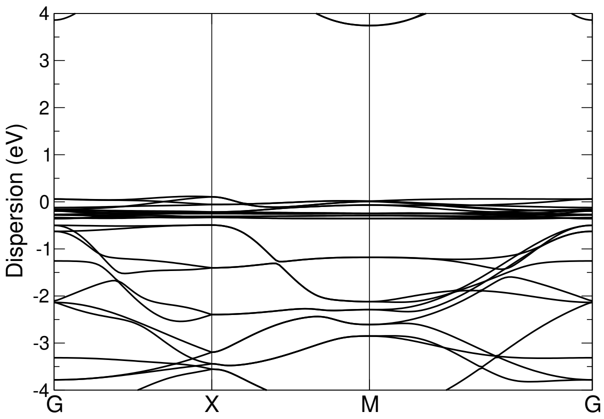

Band structure: Electronic band structure of TmOI (C2/m) in a representative window around the Fermi energy and along a high-symmetry path.

Optimized crystal structure

Structural parameters: relaxed cell (top) and atomic positions (bottom) of TmOI (C2/m) in Cartesian coordinates.

	x [Å]	y [Å]	z [Å]
$\mathbf{a_1}$	3.92278835	0.00000000	0.00000000
$\mathbf{a_2}$	0.00000000	3.92278835	0.00000000
$\mathbf{a_3}$	0.00000000	0.00000000	25.95197601

		x [Å]	y [Å]	z [Å]
●	Tm	0.00000000	1.96139418	14.04956137
●	I	1.96139418	0.00000000	16.02322177
●	Tm	1.96139418	0.00000000	11.90241465
●	I	0.00000000	1.96139418	9.92875425
•	O	0.00000000	0.00000000	12.97598801
•	O	1.96139418	1.96139418	12.97598801

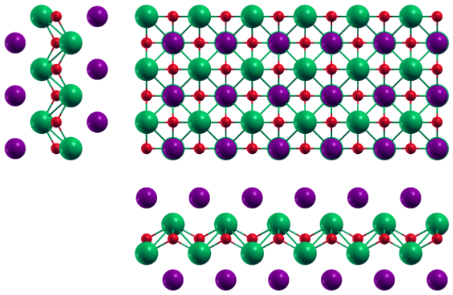

Orthographic projections: views of TmOI (C2/m) as seen from the x axis (left), the y axis (bottom) and the z axis (center).

Lattice matching - minimal size

Formula	N° atoms	strain	cell size 1	cell size 2
K	7	0.1815	1	1
InSe	8	0.1438	1	1
Bi_2	8	0.1491	1	1
AgTl	8	0.0206	1	1
Ag_2	8	0.1884	1	1
LiO	8	0.1101	1	1
PbTe	8	0.1454	1	1
Sb_2	8	0.1309	1	1
I_2Mg	9	0.1352	1	1
CdI_2	9	0.1469	1	1
Nd	9	0.7483	1	3
Ba_2Pt	9	0.1881	1	1
Br_2Ca	9	0.1481	1	1
CaI_2	9	0.1706	1	1
I_2Pr	9	0.002	1	1
Br_2La	9	0.1354	1	1
Br_2Cu	9	0.1046	1	1
Ca_2Si	9	0.194	1	1
I_2Yb	9	0.1677	1	1
BiClTe	9	0.1473	1	1
BrCdI	9	0.1377	1	1
HgI_2	9	1.1064	1	1
BaF_2	9	0.1404	1	1
BiBrTe	9	0.1526	1	1
$RhTe_2$	9	0.1089	1	1
GeI_2	9	0.1337	1	1
AsKSn	9	0.1392	1	1
$PbTe_2$	9	0.1369	1	1
I_2Nd	9	0.0011	1	1
Cl_2Cu	9	0.0981	1	1
I_2Tm	9	0.1692	1	1
$SnTe_2$	9	0.132	1	1
Cl_2V	9	0.1107	1	1
GeI_2	9	0.1456	1	1
I_2Pb	9	0.1905	1	1
STl_2	9	0.1413	1	1
$PtSe_2$	9	0.1084	1	1
BiITe	9	0.1592	1	1
GeS_2	9	0.2161	1	1
DyI_2	9	0.1738	1	1
CeI_2	9	0.0028	1	1
Se_2Yb	9	0.1339	1	1
$BiTe_2$	9	0.1341	1	1
GdI_2	9	0.1554	1	1
$CrSe_2$	9	0.111	1	1
I_2La	9	0.0037	1	1
$CrSe_2$	9	0.1104	1	1
CdI_2	9	0.1465	1	1
F_2Zn	9	0.0059	1	1
I_2Pr	9	0.1474	1	1

Table showing the first 50 lattice matching combinations with the smallest number of atoms in the supercell.

Lattice matching - minimal strain

Formula	N° atoms	strain	cell size 1	cell size 2
H_4Ti	920	0.0	65	106
$Cu_2Rb_2Te_2$	876	0.0001	85	61
F_4Pb	817	0.0001	82	65
$Cl_2N_2Zr_2$	678	0.0002	48	65
N_2W	237	0.0002	20	39
CaH_2O_2	613	0.0002	48	65
Ca_2O_2	706	0.0003	61	85
Bi_2In_2	754	0.0004	85	61
Ag_2I_2	580	0.0004	64	49
As_2O_3	973	0.0004	108	65
HgI_2	540	0.0005	65	50
H_2Li_2Pd	920	0.0005	65	106
Cu_2O_2	494	0.0006	49	50
$CuTe_2$	483	0.0006	48	65
HgI_2	531	0.0006	64	49
$Br_2Hf_2N_2$	678	0.0007	48	65
F_4Pb	806	0.0007	81	64
$Br_2Eu_2F_2$	12	0.0007	1	1
Mg_2	456	0.0007	49	81
$Er_2I_2O_2$	12	0.0007	1	1
Ag_2F_4	108	0.0008	12	6
$AgBrO_2$	984	0.0008	96	102
AlH_4Na	366	0.0008	36	25
$C_2Cl_2Y_2$	948	0.0009	72	86
Ho_2S_2	494	0.0009	49	50
$Cu_2K_2Te_2$	510	0.001	49	36
HfS_2	483	0.001	48	65
Se_2Ta	840	0.001	81	118
$Ag_2K_2Se_2$	690	0.001	65	50
S_2Ta	852	0.001	79	126
I_2Nd	9	0.0011	1	1
$Br_2O_2Sm_2$	12	0.0011	1	1
$Bi_2Br_2O_2$	12	0.0011	1	1
Cl_2Cu	456	0.0012	47	58
NbS_2	852	0.0012	79	126
Ir_2P_2	10	0.0013	1	1
Ca_2N	483	0.0013	48	65
$CoTe_2$	483	0.0013	48	65
$Au_2K_2S_2$	78	0.0013	10	3
FeH_2O_2	315	0.0013	20	39
BrNZr	840	0.0014	81	118
C_2Li_2	840	0.0014	84	84
$C_2Cl_2Y_2$	936	0.0014	71	85
H_2Na_2Pd	800	0.0014	65	82
Ga_2S_2	548	0.0014	48	65
LiO	602	0.0014	64	109
$AgBrO_2$	900	0.0015	88	93
$AgTe_2$	942	0.0015	89	136
Tl	670	0.0015	89	136
PtS_2	531	0.0015	52	73

Table showing the first 50 lattice matching combinations with the smallest strain needed to build the supercell.

VBr_2 (C2/m)

Structural and electronic properties

**Formula**	VBr_2
**Spacegroup**	C2/m
**Prototype**	CdI2
**Parent 3D**	VBr_2
**Source DB**	ICSD
**DB ID**	246906
**DF2-C09 Binding energy [meV/ Å^2]**	15.8
**RVV10 Binding energy [meV/ Å^2]**	22.12
**Band gap (PBE) [eV]**	1.3

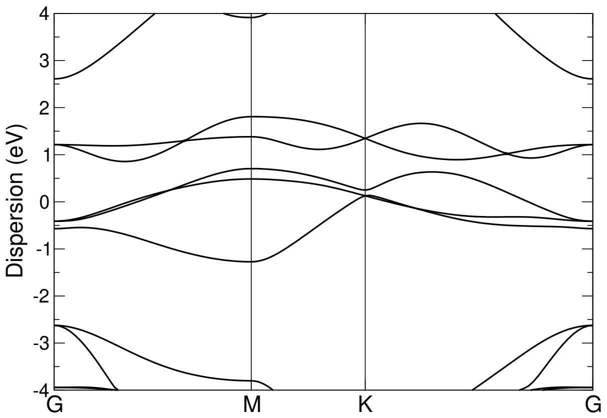

Band structure: Electronic band structure of VBr_2 (C2/m) in a representative window around the Fermi energy and along a high-symmetry path.

Optimized crystal structure

Structural parameters: relaxed cell (top) and atomic positions (bottom) of VBr_2 (C2/m) in Cartesian coordinates.

	x [Å]	y [Å]	z [Å]
$\mathbf{a_1}$	3.45044677	0.00000000	0.00000000
$\mathbf{a_2}$	−1.72522339	2.98817456	0.00000000
$\mathbf{a_3}$	0.00000000	0.00000000	23.25239252

		x [Å]	y [Å]	z [Å]
●	Br	1.72522339	0.99605819	10.00690031
●	V	0.00000000	0.00000000	11.62619626
●	Br	-0.00000000	1.99211637	13.24549220

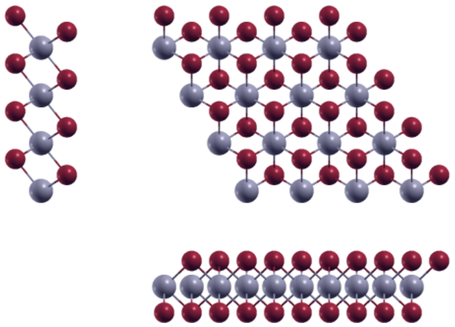

Orthographic projections: views of VBr_2 (C2/m) as seen from the x axis (left), the y axis (bottom) and the z axis (center).

Lattice matching - minimal size

Formula	N° atoms	strain	cell size 1	cell size 2
Tl	4	0.1351	1	1
Sn	4	0.1153	1	1
In	4	0.1173	1	1
HgO	5	0.1453	1	1
AsSb	5	0.4642	1	1
GeTe	5	0.4796	1	1
S_2	5	0.4837	1	1
Mg_2	5	0.1237	1	1
$IrTe_2$	6	0.482	1	1
CrS_2	6	0.2585	1	1
$CdCl_2$	6	0.4769	1	1
$AgTe_2$	6	0.1375	1	1
$ReSe_2$	6	0.0008	1	1
$InSe_2$	6	0.4778	1	1
$GeTe_2$	6	0.4743	1	1
$SiTe_2$	6	0.4491	1	1
$HfTe_2$	6	2.9108	1	1
I_2Mn	6	0.4772	1	1
NSr_2	6	0.4683	1	1
PbS_2	6	0.4577	1	1
ReS_2	6	0.2669	1	1
$AuTe_2$	6	3.0134	1	1
$PdTe_2$	6	2.9807	1	1
FeI_2	6	0.4715	1	1
I_2Ni	6	0.4748	1	1
S_2Ti	6	0.0052	1	1
Mg_3	6	0.1307	1	1
CrI_2	6	0.4704	1	1
Bi_2Pd	6	0.1544	1	1
Ba_2Hg	6	0.3232	1	1
N_2W	6	0.2493	1	1
Cl_2Ni	6	0.0017	1	1
Cl_2Co	6	0.0055	1	1
$CrTe_2$	6	0.0079	1	1
ClNZr	6	0.0021	1	1
Cl_2Fe	6	0.0062	1	1
Ba_2N	6	2.9307	1	1
Br_2Ti	6	0.0073	1	1
Te_2Zr	6	2.9172	1	1
$AsSe_2$	6	0.0032	1	1
$NiTe_2$	6	0.4477	1	1
I_2V	6	0.4524	1	1
Se_2Zr	6	0.4501	1	1
CdO_2	6	0.0057	1	1
BrNZr	6	0.0045	1	1
$NbSe_2$	6	0.0021	1	1
CoI_2	6	0.4653	1	1
O_2Zn	6	0.2539	1	1
Br_2Cr	6	0.0076	1	1
Cl_2Zr	6	0.0059	1	1

Table showing the first 50 lattice matching combinations with the smallest number of atoms in the supercell.

Lattice matching - minimal strain

Formula	N° atoms	strain	cell size 1	cell size 2
Sb_2Te_3	155	0.0	25	16
N_2W	339	0.0001	49	64
Br_2Ho_2	291	0.0001	49	36
$BiTe_2$	183	0.0001	36	25
Ni_2Te_2	499	0.0001	81	64
CI_2Y_2	638	0.0001	91	73
Se_2Yb	183	0.0001	36	25
S_2	333	0.0002	73	57
$Ge_2I_2La_2$	537	0.0002	81	49
$As_2Co_2Li_2$	483	0.0002	65	48
MnO_2	123	0.0002	16	25
Te_2Zr	300	0.0002	57	43
In_2Se_3	386	0.0003	57	43
C_2	173	0.0003	25	49
$F_2Se_2Tm_2$	486	0.0003	64	49
$HfTe_2$	300	0.0003	57	43
Ga_2Se_2	447	0.0003	73	57
I_2Ti	492	0.0003	91	73
Se_2Sn	492	0.0003	91	73
Gd	75	0.0003	16	27
$CdCl_2$	435	0.0003	81	64
Ni_2SbTe_2	504	0.0003	73	57
Bi_2S_3	327	0.0004	49	36
GeI_2	183	0.0004	36	25
$LiMnSe_2$	388	0.0004	64	49
Ag_2I_2	197	0.0004	39	20
$Ge_2I_2La_2$	405	0.0004	61	37
ReS_2	543	0.0005	81	100
I_2Mn	435	0.0005	81	64
Sb_2Se_2Te	233	0.0005	36	25
I_2Ni	435	0.0005	81	64
$IrTe_2$	390	0.0005	73	57
PTe_2Zr_2	705	0.0005	100	81
$Er_2F_2Se_2$	429	0.0005	57	43
$Ge_2Hf_2Te_2$	483	0.0005	65	48
Cu_2Te_2	387	0.0005	65	48
NSr_2	492	0.0006	91	73
$Cl_2Er_2H_2$	627	0.0006	81	64
Ba_2Ni_3	233	0.0006	36	25
CrS_2	435	0.0006	64	81
Cu_4Te_2	171	0.0006	25	16
Ga_2Te_2	208	0.0006	36	25
$Cl_2S_2Tl_2$	714	0.0006	130	54
Br_2Tb_2	291	0.0006	49	36
$Br_2Gd_2O_2$	840	0.0006	118	81
CoI_2	492	0.0006	91	73
$GeTe_2$	435	0.0007	81	64
$I_2Pr_2Si_2$	258	0.0007	36	25
F_2Zn	597	0.0007	118	81
$InSe_2$	435	0.0007	81	64

Table showing the first 50 lattice matching combinations with the smallest strain needed to build the supercell.

VCl_2 (C2/m)

Structural and electronic properties

**Formula**	VCl_2
**Spacegroup**	C2/m
**Prototype**	CdI2
**Parent 3D**	VCl_2
**Source DB**	COD
**DB ID**	1528165
**DF2-C09 Binding energy [meV/ Å^2]**	15.5
**RVV10 Binding energy [meV/ Å^2]**	22.13
**Band gap (PBE) [eV]**	1.41

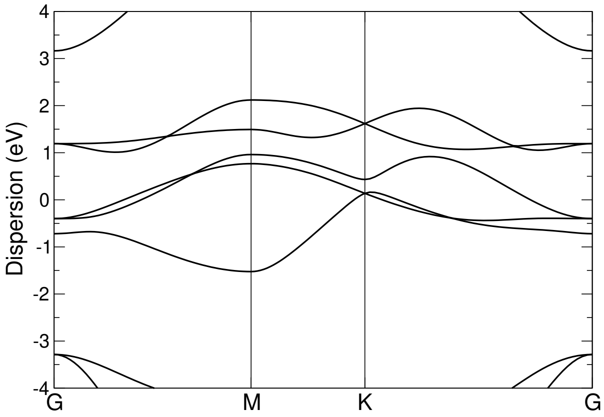

Band structure: Electronic band structure of VCl_2 (C2/m) in a representative window around the Fermi energy and along a high-symmetry path.

Optimized crystal structure

Structural parameters: relaxed cell (top) and atomic positions (bottom) of VCl_2 (C2/m) in Cartesian coordinates.

	x [Å]	y [Å]	z [Å]
$\mathbf{a_1}$	3.21450618	0.00000000	0.00000000
$\mathbf{a_2}$	−1.60725309	2.78384401	0.00000000
$\mathbf{a_3}$	0.00000000	0.00000000	23.08719819

	x [Å]	y [Å]	z [Å]
Cl	0.00000000	1.85589601	13.08254534
V	0.00000000	0.00000000	11.54359910
Cl	1.60725309	0.92794800	10.00465286

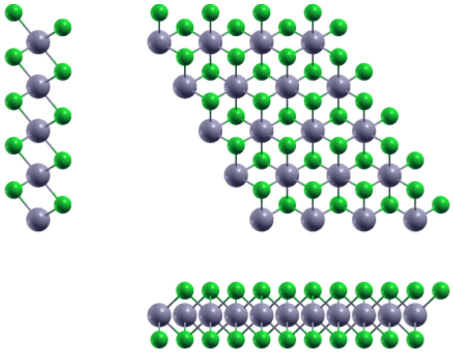

Orthographic projections: views of VCl_2 (C2/m) as seen from the x axis (left), the y axis (bottom) and the z axis (center).

Lattice matching - minimal size

Formula	N° atoms	strain	cell size 1	cell size 2
Sn	4	0.1359	1	1
Na	4	0.4535	1	1
In	4	0.1395	1	1
In	4	0.0079	1	1
As_2	5	0.4734	1	1
LiO	5	0.0025	1	1
Mg_2	5	0.15	1	1
Cl_2Zn	6	0.4708	1	1
S_2V	6	0.0048	1	1
MoS_2	6	0.0041	1	1
$MoTe_2$	6	0.4591	1	1
PSn_2	6	0.4967	1	1
Br_2Zn	6	3.0156	1	1
HfS_2	6	0.4805	1	1
FeO_2	6	0.2501	1	1
$AsSn_2$	6	2.9243	1	1
Te_2V	6	0.4639	1	1
I_2Pr	6	0.3255	1	1
$CuTe_2$	6	0.4795	1	1
S_2Zr	6	0.4947	1	1
Br_2Cu	6	1.3574	1	1
NiO_2	6	1.6328	1	1
Br_2Co	6	0.4723	1	1
Ca_2N	6	0.4748	1	1
FeI_2	6	3.1969	1	1
Te_2Ti	6	3.0196	1	1
Te_2Zn	6	0.4587	1	1
$RhTe_2$	6	2.9613	1	1
S_2W	6	0.004	1	1
GeI_2	6	13.6328	1	1
Br_2Mn	6	0.4677	1	1
PtS_2	6	0.4561	1	1
$CoTe_2$	6	0.4813	1	1
CdClO	6	0.4627	1	1
Se_2Ti	6	0.4496	1	1
Te_2W	6	0.4595	1	1
I_2Nd	6	0.3275	1	1
S_2Sn	6	0.4953	1	1
$PtSe_2$	6	2.9328	1	1
OTl_2	6	0.4631	1	1
Br_2Fe	6	0.4725	1	1
Br_2Ni	6	0.4856	1	1
CeI_2	6	0.3239	1	1
$FeSe_2$	6	0.131	1	1
Br_2Mg	6	3.195	1	1
$NbTe_2$	6	0.4941	1	1
Se_2Yb	6	13.6467	1	1
MoS_2	6	0.0038	1	1
Cl_2Mg	6	0.4858	1	1
$BiTe_2$	6	13.6621	1	1

Table showing the first 50 lattice matching combinations with the smallest number of atoms in the supercell.

Lattice matching - minimal strain

Formula	N° atoms	strain	cell size 1	cell size 2
$AsCuLi_2$	439	0.0	81	49
S_2Sn	339	0.0	64	49
NSr_2	183	0.0001	36	25
K	276	0.0001	79	39
$Br_2Hf_2N_2$	627	0.0001	81	64
Cl_2Y_2	291	0.0002	49	36
$TaTe_2$	300	0.0002	57	43
Br_2Ca	75	0.0002	16	9
$I_2Pr_2Si_2$	537	0.0002	81	49
Br_2HLa	331	0.0002	61	37
$CeLi_2P_2$	368	0.0002	61	37
F_4Sn	217	0.0002	39	20
CdH_2O_2	705	0.0002	100	81
Br_2Tb_2	139	0.0003	25	16
$I_2S_2Sm_2$	471	0.0003	79	39
Ga_2Te_2	439	0.0003	81	49
S_2Zr	339	0.0003	64	49
Ba_2Ni_3	488	0.0003	81	49
Se_2Sn	183	0.0003	36	25
$PdTe_2$	123	0.0003	25	16
Br_2Mn	492	0.0003	91	73
$Hf_2I_2N_2$	429	0.0004	57	43
CdClHO	343	0.0004	57	43
I_2La_2Sb	432	0.0005	79	39
Ga_2S_2	447	0.0005	73	57
Ca_2N	435	0.0005	81	64
CI_2Y_2	233	0.0005	36	25
PSn_2	339	0.0005	64	49
Cl_2OOs	204	0.0005	36	24
GeI_2	390	0.0005	81	49
$NbTe_2$	339	0.0005	64	49
Bi_2S_3	155	0.0005	25	16
N_2Re	435	0.0005	64	81
$Br_2O_2Sc_2$	678	0.0005	100	63
$Ga_2I_2Tb_2$	102	0.0005	16	9
Ga_2Se_2	291	0.0006	49	36
Ga_2S_2	447	0.0006	73	57
I_2Pr	75	0.0006	16	9
CdClO	543	0.0006	100	81
FeO_2	339	0.0006	49	64
$AsCuLi_2$	331	0.0006	61	37
Ba_2Hg	177	0.0007	39	20
Te_2W	543	0.0007	100	81
BiClTe	75	0.0007	16	9
$Cl_2N_2Zr_2$	627	0.0007	81	64
CBr_2Y_2	327	0.0007	49	36
Bi_2In_2	606	0.0007	130	54
Se_2Yb	390	0.0007	81	49
KS_2Ti	565	0.0007	91	73
$CoTe_2$	390	0.0007	73	57

Table showing the first 50 lattice matching combinations with the smallest strain needed to build the supercell.

VI_2 (C2/m)

Structural and electronic properties

**Formula**	VI_2
**Spacegroup**	C2/m
**Prototype**	CdI2
**Parent 3D**	VI_2
**Source DB**	ICSD
**DB ID**	246907
**DF2-C09 Binding energy [meV/ Å^2]**	16.37
**RVV10 Binding energy [meV/ Å^2]**	22.38
**Band gap (PBE) [eV]**	1.17

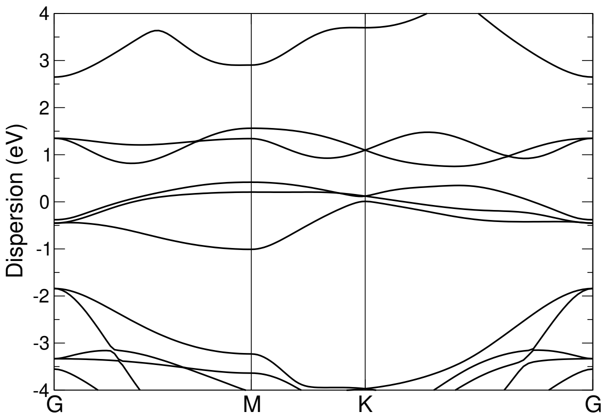

Band structure: Electronic band structure of VI_2 (C2/m) in a representative window around the Fermi energy and along a high-symmetry path.

Optimized crystal structure

Structural parameters: relaxed cell (top) and atomic positions (bottom) of VI_2 (C2/m) in Cartesian coordinates.

		x [Å]	y [Å]	z [Å]
$\mathbf{a_1}$		3.80518271	0.00000000	0.00000000
$\mathbf{a_2}$		−1.90259135	3.29538489	0.00000000
$\mathbf{a_3}$		0.00000000	0.00000000	23.35111177
		x [Å]	y [Å]	z [Å]
●	I	-0.00000000	2.19692326	13.35477294
●	V	0.00000000	0.00000000	11.67555589
●	I	1.90259135	1.09846163	9.99633884

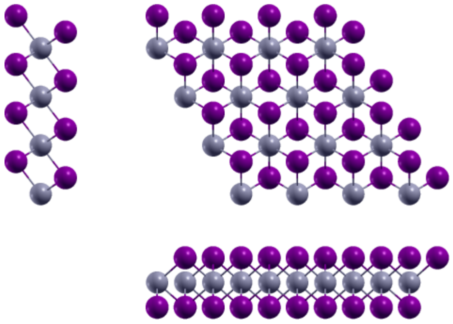

Orthographic projections: views of VI_2 (C2/m) as seen from the x axis (left), the y axis (bottom) and the z axis (center).

Lattice matching - minimal size

Formula	N° atoms	strain	cell size 1	cell size 2
K	4	3.1939	1	1
Tl	4	0.2477	1	1
InSe	5	0.4659	1	1
Nd	5	0.2259	1	2
HgO	5	0.1145	1	1
AsSb	5	0.0048	1	1
Bi_2	5	0.4807	1	1
PbTe	5	0.4704	1	1
CaCl	5	0.1479	1	1
Cl_2Mn	6	0.2553	1	1
CdI_2	6	0.4748	1	1
$AgTe_2$	6	0.1111	1	1
$MoSe_2$	6	0.2467	1	1
$ReSe_2$	6	0.2739	1	1
S_2Ta	6	0.2582	1	1
Br_2Zn	6	0.0054	1	1
Br_2Ca	6	0.4779	1	1
$GeTe_2$	6	0.0089	1	1
$SiTe_2$	6	0.0013	1	1
NSr_2	6	0.0065	1	1
I_2Yb	6	3.0315	1	1
PbS_2	6	0.0022	1	1
BiClTe	6	0.4757	1	1
BrCdI	6	0.4482	1	1
FeI_2	6	0.0078	1	1
I_2Ni	6	0.0091	1	1
S_2Ti	6	0.2651	1	1
Mg_3	6	0.4256	1	1
Te_2Ti	6	0.0051	1	1
NbS_2	6	0.2575	1	1
CrI_2	6	0.0074	1	1
BaF_2	6	0.4564	1	1
Bi_2Pd	6	0.119	1	1
Cl_2Ni	6	0.2753	1	1
Cl_2Co	6	0.2646	1	1
Br_2V	6	0.2728	1	1
ClNZr	6	0.2697	1	1
Cl_2Fe	6	0.2636	1	1
S_2Ta	6	0.2506	1	1
Se_2V	6	0.2488	1	1
AsKSn	6	0.4528	1	1
$NiTe_2$	6	0.0019	1	1
GeI_2	6	0.471	1	1
Se_2Zr	6	0.0009	1	1
STl_2	6	0.4587	1	1
BiITe	6	2.9279	1	1
CdO_2	6	0.2644	1	1
$NbSe_2$	6	0.2759	1	1
CoI_2	6	0.0052	1	1
GeS_2	6	0.1373	1	1

Table showing the first 50 lattice matching combinations with the smallest number of atoms in the supercell.

Lattice matching - minimal strain

Formula	N° atoms	strain	cell size 1	cell size 2
I_2Pr	435	0.0	81	64
I_2La_2P	437	0.0001	64	49
$AsLi_3$	565	0.0001	91	73
CdO_2	492	0.0001	73	91
$AlLiTe_2$	343	0.0001	57	43
Cl_2Mn	390	0.0001	57	73
Cl_2Zr	492	0.0001	73	91
CS_3Tl_2	513	0.0001	115	28
BiClTe	435	0.0001	81	64
In_2Te_3	705	0.0001	100	81
$Br_2Hf_2N_2$	609	0.0002	75	64
Sb_2SeTe_2	705	0.0002	100	81
Cl_2Co	492	0.0002	73	91
Cu_2O_2	411	0.0003	65	54
Se_2V	339	0.0004	49	64
$Cl_2Ho_2O_2$	9	0.0004	1	1
Gd_2GeI_2	563	0.0004	81	64
Cl_2NSc_2	536	0.0004	57	73
O_2Zn	123	0.0004	16	25
InSe	419	0.0004	91	73
Bi_2SeTe_2	504	0.0004	73	57
Cl_2Hf_2	516	0.0004	64	81
As_2Li_2Pr	638	0.0004	91	73
$I_2La_2Si_2$	711	0.0004	91	73
CdI_2	435	0.0005	81	64
F_4Nb	435	0.0005	65	48
Cl_2Fe	492	0.0005	73	91
$Ge_2I_2La_2$	363	0.0005	49	36
Bi_2Se_2Te	705	0.0005	100	81
Bi_2STe_2	638	0.0006	91	73
S_2Ti	492	0.0006	73	91
Sn_2Te_2	530	0.0006	98	59
As_2O_3	545	0.0006	100	49
Br_2Hg_3	237	0.0006	64	9
$Al_2Cl_2O_2$	474	0.0006	50	54
La_2S_2	228	0.0006	40	27
$I_2S_2Sm_2$	258	0.0007	36	25
H_2MgO_2	255	0.0007	25	36
Ca_4Cu_2	597	0.0007	99	50
$LiNbS_2$	516	0.0007	64	81
Cu_4Te_2	507	0.0007	65	52
Br_2Ca	435	0.0008	81	64
Sb_2Te_3	504	0.0008	73	57
Br_2La_2P	563	0.0008	81	64
S_2Ta	435	0.0008	64	81
GdI_2	339	0.0008	64	49
Se_2Sn_2	204	0.0009	36	24
CdI_2	435	0.0009	81	64
Se_2Zr	6	0.0009	1	1
Bi_2	333	0.001	73	57

Table showing the first 50 lattice matching combinations with the smallest strain needed to build the supercell.

VOBr (Pmmn)

Structural and electronic properties

**Formula**	VOBr
**Spacegroup**	Pmmn
**Prototype**	FeOCl
**Parent 3D**	$V_2O_2Br_2$
**Source DB**	ICSD
**DB ID**	27010
**DF2-C09 Binding energy [meV/ Å^2]**	14.66
**RVV10 Binding energy [meV/ Å^2]**	22.44
**Band gap (PBE) [eV]**	N/A

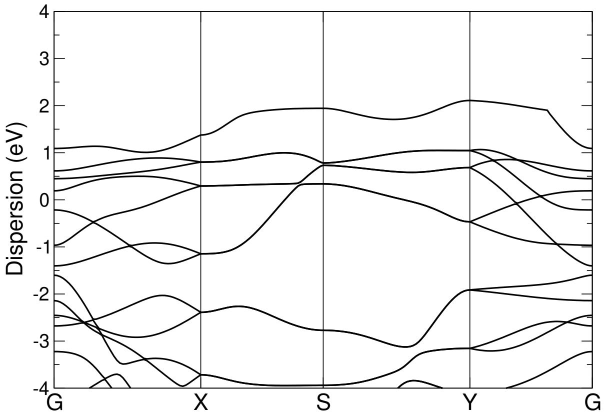

Band structure: Electronic band structure of VOBr (Pmmn) in a representative window around the Fermi energy and along a high-symmetry path.

Optimized crystal structure

Structural parameters: relaxed cell (top) and atomic positions (bottom) of VOBr (Pmmn) in Cartesian coordinates.

	x [Å]	y [Å]	z [Å]
$\mathbf{a_1}$	3.23820928	0.00000000	0.00000000
$\mathbf{a_2}$	0.00000000	3.87883962	0.00000000
$\mathbf{a_3}$	0.00000000	0.00000000	25.21423375

		x [Å]	y [Å]	z [Å]
●	V	1.61910464	1.93941981	11.95979959
●	Br	0.00000000	1.93941981	10.02880793
•	O	1.61910464	0.00000000	11.94923986
●	V	0.00000000	0.00000000	13.25442386
●	Br	1.61910464	0.00000000	15.18542242
•	O	0.00000000	1.93941981	13.26499857

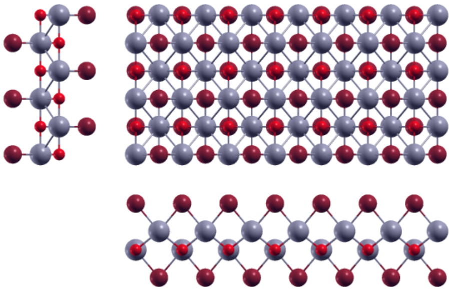

Orthographic projections: views of VOBr (Pmmn) as seen from the x axis (left), the y axis (bottom) and the z axis (center).

Lattice matching - minimal size

Formula	N° atoms	strain	cell size 1	cell size 2
K	7	0.2655	1	1
HgO	8	0.0499	1	1
Mg_2	8	0.2273	1	1
CaCl	8	0.2742	1	1
CaI_2	9	0.2499	1	1
I_2Pr	9	0.0759	1	1
I_2Yb	9	0.2457	1	1
Cl_2Zn	9	0.298	1	1
Ba_2N	9	1.8095	1	1
I_2Nd	9	0.0769	1	1
Cl_2Cu	9	0.401	1	1
I_2Tm	9	0.2479	1	1
GeS_2	9	0.3237	1	1
$MnSe_2$	9	0.2741	1	1
DyI_2	9	0.2546	1	1
CeI_2	9	0.0751	1	1
F_2Ni	9	0.2912	1	1
F_2Zn	9	0.0718	1	1
Li_2Tl_2	10	0.3575	1	1
Cu_2I_2	10	0.9918	1	1
Cl_2OOs	10	0.2817	1	1
Cu_2Te_2	10	0.0121	1	1
Ir_2P_2	10	0.0767	1	1
$AgNO_2$	10	0.6114	1	1
$AgCuTe_2$	10	0.0298	1	1
O_2Sn_2	10	0.3037	1	1
Cu_2S_2	10	0.0687	1	1
Cl_2OV	10	0.0233	1	1
Br_2Cu_2	10	0.0705	1	1
Fe_2Se_2	10	0.2903	1	1
As_2Co_2	10	0.2822	1	1
Cu_2Te_2	10	0.3025	1	1
Ge_2S_2	10	0.3238	1	1
C_2Li_2	10	0.2484	1	1
P_4	10	0.5327	1	1
O_2Sn_2	10	0.0692	1	1
N_4	10	0.1407	1	1
P_2Rh_2	10	0.068	1	1
Fe_2S_2	10	0.271	1	1
F_2Tl_2	10	0.0681	1	1
Au_2I_2	10	0.6238	1	1
Co_2S_2	10	0.2753	1	1
Cu_2Se_2	10	0.2921	1	1
Ag_2Te_2	10	0.0582	1	1
$AgClO_2$	10	0.1402	1	1
Ni_2Se_2	10	0.0748	1	1
Ba_2Cu_2	10	0.2454	1	1
Fe_2SeTe	10	0.308	1	1
O_2Sn_2	10	0.208	1	1
Co_2Se_2	10	0.2844	1	1

Table showing the first 50 lattice matching combinations with the smallest number of atoms in the supercell.

Lattice matching - minimal strain

Formula	N° atoms	strain	cell size 1	cell size 2
$CoTe_2$	558	0.0002	60	66
HfS_2	558	0.0002	60	66
Ga_2S_2	624	0.0003	60	66
$F_2Ho_2Se_2$	756	0.0003	66	60
$Ca_2Ge_2Mn_2$	66	0.0004	6	5
$Br_2F_2Tm_2$	66	0.0004	6	5
$As_2Mg_2Na_2$	402	0.0004	40	27
$I_2Lu_2O_2$	66	0.0005	6	5
$FeSe_2$	282	0.0005	27	40
I_2Tm	147	0.0006	18	13
K	267	0.0006	40	27
$C_2Br_2Y_2$	954	0.0007	82	77
CaI_2	147	0.0007	18	13
$Bi_2Cl_2O_2$	66	0.0008	6	5
Br_2Cu	639	0.0008	73	67
$I_2Lu_2Se_2$	918	0.0008	102	51
GeI_2La_2	173	0.0008	18	13
$Ba_2F_2I_2$	474	0.0009	49	30
$Br_2Gd_2O_2$	66	0.0009	6	5
$AgClO_4$	138	0.001	15	8
$AgClO_2$	500	0.001	52	47
$C_2I_2Y_2$	810	0.001	72	63
Br_2Mn	375	0.001	40	45
Br_2Er_2	430	0.001	45	40
CaH_2O_2	690	0.001	60	66
S_2Sn_2	444	0.001	50	36
$CrTe_2$	657	0.001	69	81
$AuTe_2$	390	0.001	45	40
$Cl_2Ho_2O_2$	672	0.0011	56	56
Cl_2Sc_2	738	0.0011	69	81
KS_2Ti	420	0.0011	40	45
I_2V	504	0.0012	56	56
F_2Zn	51	0.0012	6	5
$C_2Br_2La_2$	708	0.0012	65	53
Br_2Gd_2	430	0.0012	45	40
Cl_2Rb_2	72	0.0012	10	3
$PtTe_2$	390	0.0012	45	40
O_2Sn_2	724	0.0012	72	73
$Cl_2N_2Zr_2$	756	0.0012	60	66
As_2Sn_2	430	0.0013	45	40
$Cl_2O_2Y_2$	672	0.0013	56	56
Br_2Cr	657	0.0013	69	81
$As_2Cd_2K_2$	474	0.0013	49	30
Ge_2Te_2	868	0.0013	100	67
Ba_2N	576	0.0013	66	60
Ga_2S_2	624	0.0013	60	66
As_4	790	0.0013	89	64
O_4PTl	474	0.0013	49	30
Br_2Ca_3Si	186	0.0014	21	10
Br_2PY_2	696	0.0014	66	60

Table showing the first 50 lattice matching combinations with the smallest strain needed to build the supercell.

VOCl (Pmmn)

Structural and electronic properties

**Formula**	VOCl
**Spacegroup**	Pmmn
**Prototype**	FeOCl
**Parent 3D**	$V_2O_2Cl_2$
**Source DB**	COD
**DB ID**	2106692
**DF2-C09 Binding energy [meV/ Å^2]**	13.46
**RVV10 Binding energy [meV/ Å^2]**	21.47
**Band gap (PBE) [eV]**	N/A

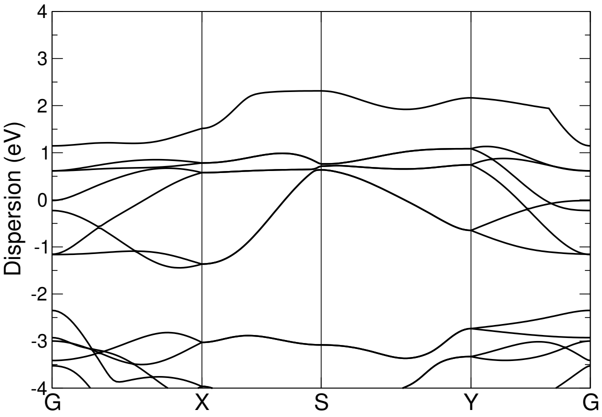

Band structure: Electronic band structure of VOCl (Pmmn) in a representative window around the Fermi energy and along a high-symmetry path.

Optimized crystal structure

Structural parameters: relaxed cell (top) and atomic positions (bottom) of VOCl (Pmmn) in Cartesian coordinates.

		x [Å]	y [Å]	z [Å]
$\mathbf{a_1}$		3.09025192	0.00000000	0.00000000
$\mathbf{a_2}$		0.00000000	3.87972837	0.00000000
$\mathbf{a_3}$		0.00000000	0.00000000	24.88388938
		x [Å]	y [Å]	z [Å]
●	V	1.54512596	0.00000000	11.75644073
●	Cl	0.00000000	0.00000000	9.98294876
●	O	1.54512596	1.93986418	11.75126898
●	V	0.00000000	1.93986418	13.12743101
●	Cl	1.54512596	1.93986418	14.90094412
●	O	0.00000000	0.00000000	13.13263488

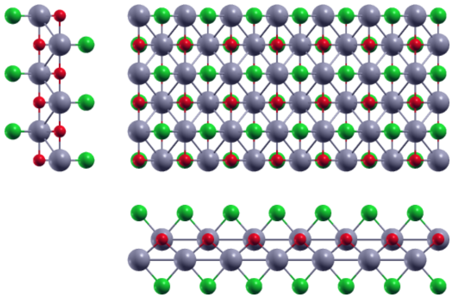

Orthographic projections: views of VOCl (Pmmn) as seen from the x axis (left), the y axis (bottom) and the z axis (center).

Lattice matching - minimal size

Formula	N° atoms	strain	cell size 1	cell size 2
K	7	0.2935	1	1
Mg_2	8	0.2511	1	1
CaI_2	9	0.2766	1	1
I_2Pr	9	0.3594	1	1
I_2Yb	9	0.2721	1	1
$AuTe_2$	9	1.9362	1	1
$PdTe_2$	9	1.9147	1	1
I_2Nd	9	0.3614	1	1
I_2Tm	9	0.2745	1	1
BiITe	9	0.2587	1	1
GeS_2	9	0.057	1	1
DyI_2	9	0.2817	1	1
CeI_2	9	0.3579	1	1
GdI_2	9	0.2526	1	1
$PtTe_2$	9	1.9327	1	1
Br_2Cd	9	1.9066	1	1
F_2Zn	9	0.8932	1	1
Li_2Tl_2	10	0.3924	1	1
Ir_2P_2	10	0.361	1	1
$AgNO_2$	10	0.6638	1	1
Pb_2Se_2	10	0.4936	1	1
O_2Sn_2	10	0.3175	1	1
$AlLiTe_2$	10	0.2568	1	1
Br_2Tb_2	10	1.9199	1	1
Br_2Cu_2	10	0.8874	1	1
Cl_2ORu	10	0.2857	1	1
As_4	10	1.3262	1	1
C_2Li_2	10	0.2667	1	1
Br_2OV	10	0.2659	1	1
Bi_2Se_2	10	0.4411	1	1
Fe_2S_2	10	0.2846	1	1
Au_2I_2	10	0.6858	1	1
As_2Fe_2	10	0.0574	1	1
Ag_2Te_2	10	0.0803	1	1
Ni_2Se_2	10	0.3574	1	1
Ba_2Cu_2	10	0.2718	1	1
O_2Sn_2	10	0.2207	1	1
AsI_2La_2	11	0.2638	1	1
Bi_2Te_3	11	0.2586	1	1
GeI_2La_2	11	0.2741	1	1
H_2Na_2Pd	11	0.0566	1	1
Cd_2I_3	11	0.2638	1	1
Bi_2S_3	11	1.9221	1	1
H_2Li_2Pd	11	0.2537	1	1
$CuGeO_3$	11	0.7976	1	1
I_2La_2Sb	11	0.2942	1	1
I_2La_2Te	11	0.289	1	1
H_4Ti	11	0.2545	1	1
$Er_2I_2O_2$	12	0.3652	1	1
$Hf_2Se_2Si_2$	12	0.0576	1	1

Table showing the first 50 lattice matching combinations with the smallest number of atoms in the supercell.

Lattice matching - minimal strain

Formula	N° atoms	strain	cell size 1	cell size 2
Br_2Ti	441	0.0007	47	53
$Br_2H_2Zr_2$	600	0.0008	47	53
CaI_2	129	0.0008	16	11
Br_2Cr	441	0.0008	47	53
K	465	0.0009	70	45
Cl_2Sc_2	494	0.0009	47	53
$Cl_2H_2Sc_2$	600	0.0009	47	53
$As_2Mg_2Na_2$	690	0.0009	70	45
$CrTe_2$	441	0.0009	47	53
$Br_2Ca_2F_2$	54	0.001	5	4
F_2Zn	42	0.0011	5	4
Se_2Ta	441	0.0011	47	53
$Ag_2K_2Se_2$	972	0.0011	101	61
Br_2Cd	807	0.0011	94	81
Ga_2Se_2	734	0.0011	75	71
Br_2PY_2	969	0.0011	94	81
Cl_4Cu_2	126	0.0011	16	5
$CdCl_2$	954	0.0011	109	100
$ReSe_2$	768	0.0011	81	94
I_2Mn	954	0.0011	109	100
$Br_2F_2Yb_2$	54	0.0012	5	4
$Br_2Gd_2O_2$	54	0.0012	5	4
Br_2V	768	0.0012	81	94
$InSe_2$	954	0.0012	109	100
S_2Zr	663	0.0013	73	75
$NbTe_2$	663	0.0013	73	75
S_2Sn	663	0.0014	73	75
I_2Tm	129	0.0014	16	11
$PdTe_2$	807	0.0014	94	81
KS_2Ti	576	0.0014	56	60
I_2Ni	954	0.0014	109	100
Br_2Mn	516	0.0015	56	60
ClNZr	942	0.0015	99	116
GeI_2La_2	917	0.0015	97	67
Br_2Cu_2	46	0.0015	5	4
$Cl_2O_2Yb_2$	888	0.0015	75	73
Bi_2In_2	148	0.0015	18	10
K	445	0.0015	67	43
$Cl_2O_2Y_2$	876	0.0015	75	71
GeTe	854	0.0016	109	100
$I_2Lu_2O_2$	54	0.0016	5	4
PSn_2	663	0.0016	73	75
GeI_2La_2	151	0.0016	16	11
CdClHO	300	0.0016	30	30
$HfSe_2$	669	0.0016	75	73
Cl_2Zn	516	0.0016	56	60
Te_2Ti	669	0.0016	75	73
$Br_2F_2Tm_2$	54	0.0016	5	4
$As_2Mg_2Na_2$	660	0.0016	67	43
Cl_2Zr_2	862	0.0017	81	94

Table showing the first 50 lattice matching combinations with the smallest strain needed to build the supercell.

VS_2 (P-3m1)

Structural and electronic properties

**Formula**	VS_2
**Spacegroup**	P-3m1
**Prototype**	CdI2
**Parent 3D**	VS_2
**Source DB**	ICSD
**DB ID**	651361
**DF2-C09 Binding energy [meV/ Å^2]**	27.69
**RVV10 Binding energy [meV/ Å^2]**	31.16
**Band gap (PBE) [eV]**	N/A

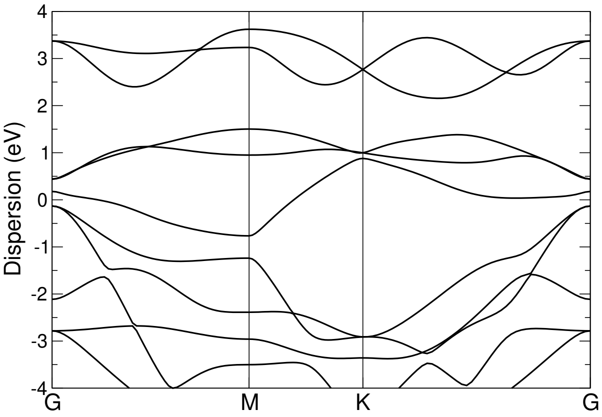

Band structure: Electronic band structure of VS_2 (P-3m1) in a representative window around the Fermi energy and along a high-symmetry path.

Optimized crystal structure

Structural parameters: relaxed cell (top) and atomic positions (bottom) of VS_2 (P-3m1) in Cartesian coordinates.

	x [Å]	y [Å]	z [Å]
$\mathbf{a_1}$	3.18180969	0.00000000	0.00000000
$\mathbf{a_2}$	−1.59090485	2.75552802	0.00000000
$\mathbf{a_3}$	0.00000000	0.00000000	22.93952906

		x [Å]	y [Å]	z [Å]
	S	0.00000000	1.83701868	12.93694190
	V	0.00000000	0.00000000	11.46976453
	S	1.59090485	0.91850934	10.00258716

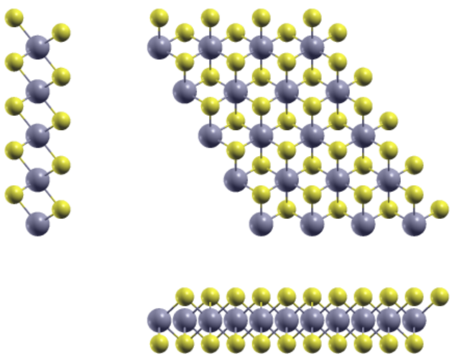

Orthographic projections: views of VS_2 (P-3m1) as seen from the x axis (left), the y axis (bottom) and the z axis (center).

Lattice matching - minimal size

Formula	N° atoms	strain	cell size 1	cell size 2
Sn	4	0.1399	1	1
Na	4	0.4655	1	1
In	4	0.1437	1	1
In	4	0.0032	1	1
As_2	5	0.4859	1	1
LiO	5	0.0075	1	1
Mg_2	5	0.1547	1	1
Cl_2Zn	6	0.4832	1	1
MoS_2	6	0.0007	1	1
$MoTe_2$	6	0.4712	1	1
HfS_2	6	0.4931	1	1
FeO_2	6	0.2566	1	1
$AsSn_2$	6	2.988	1	1
Te_2V	6	0.4761	1	1
I_2Pr	6	0.3357	1	1
S_2Zr	6	2.9281	1	1
NiO_2	6	0.2584	1	1
Br_2Co	6	0.4848	1	1
Ca_2N	6	0.4873	1	1
Te_2Zn	6	0.4708	1	1
$RhTe_2$	6	3.0258	1	1
S_2W	6	0.0008	1	1
Br_2Mn	6	0.4801	1	1
$CrTe_2$	6	0.4521	1	1
PtS_2	6	0.4681	1	1
$CoTe_2$	6	0.494	1	1
CdClO	6	0.4749	1	1
Se_2Ti	6	0.4615	1	1
Br_2Ti	6	0.4507	1	1
Te_2W	6	0.4716	1	1
S_2Sn	6	2.9315	1	1
Cl_2V	6	0.0049	1	1
$PtSe_2$	6	2.9966	1	1
OTl_2	6	0.4754	1	1
Br_2Fe	6	0.485	1	1
Br_2Cr	6	0.4514	1	1
Br_2Ni	6	0.4984	1	1
CeI_2	6	0.3341	1	1
$FeSe_2$	6	0.1346	1	1
$NbTe_2$	6	2.9253	1	1
MoS_2	6	0.0009	1	1
Cl_2Mg	6	0.4985	1	1
$CrSe_2$	6	0.0037	1	1
Se_2Ta	6	0.4488	1	1
$CrSe_2$	6	0.0062	1	1
O_2Pt	6	0.0047	1	1
N_2Re	6	0.2669	1	1
F_2Zn	6	0.3273	1	1
CoO_2	6	0.2576	1	1
Ca_2Cl_2	7	0.3103	1	1

Table showing the first 50 lattice matching combinations with the smallest number of atoms in the supercell.

Lattice matching - minimal strain

Formula	N° atoms	strain	cell size 1	cell size 2
$PtSe_2$	255	0.0	49	36
Cl_2Zn	390	0.0	73	57
Ga_2Se_2	208	0.0	36	25
Te_2V	435	0.0001	81	64
Se_2Ti	543	0.0001	100	81
NS_2Zr	565	0.0001	91	73
Ga_2S_3	638	0.0002	91	73
$I_2La_2Si_2$	102	0.0002	16	9
$Cl_2Hf_2N_2$	627	0.0002	81	64
OTl_2	435	0.0002	81	64
$Ba_2H_2I_2$	714	0.0003	130	54
Br_2Zr_2	624	0.0003	100	81
As_2Li_2Pr	93	0.0003	16	9
$F_2Lu_2Se_2$	537	0.0003	81	49
InSe	66	0.0003	16	9
Br_2Y_2	208	0.0003	36	25
Te_2Zr	123	0.0003	25	16
MnO_2	300	0.0003	43	57
$F_2Lu_2Se_2$	405	0.0003	61	37
As_2Li_2Nd	93	0.0004	16	9
CBr_2Lu_2	386	0.0004	57	43
Ga_2S_2	388	0.0004	64	49
CdClO	435	0.0004	81	64
$Br_2N_2Zr_2$	429	0.0004	57	43
Cu_3Se_3	171	0.0004	25	16
PtS_2	492	0.0005	91	73
N_2Re	543	0.0005	81	100
$CoTe_2$	339	0.0005	64	49
$Cl_2O_2Y_2$	258	0.0005	36	25
Na	346	0.0005	91	73
PbS_2	183	0.0006	36	25
Br_2Ca_3Si	594	0.0006	100	49
$AsLi_3$	84	0.0006	16	9
GeI_2Y_2	93	0.0006	16	9
Br_2Co	390	0.0006	73	57
ClH_3O	47	0.0006	9	4
Ga_2S_2	388	0.0006	64	49
$AsSn_2$	255	0.0007	49	36
Ho_2S_2	101	0.0007	19	11
Br_2Fe	390	0.0007	73	57
$Br_2In_2O_2$	312	0.0007	48	28
NiO_2	435	0.0007	64	81
Ba_2N	123	0.0007	25	16
MoS_2	6	0.0007	1	1
I_2La_2Te	545	0.0008	100	49
Cl_2ORu	204	0.0008	36	24
S_2W	6	0.0008	1	1
In_2Se_3	155	0.0008	25	16
Ba_2Cu_2	247	0.0008	49	25
$Gd_2I_2S_2$	594	0.0008	100	49

Table showing the first 50 lattice matching combinations with the smallest strain needed to build the supercell.

VSe_2 (P-3m1)

Structural and electronic properties

**Formula**	VSe_2
**Spacegroup**	P-3m1
**Prototype**	CdI2
**Parent 3D**	VSe_2
**Source DB**	ICSD
**DB ID**	86520
**DF2-C09 Binding energy [meV/ Å^2]**	25.44
**RVV10 Binding energy [meV/ Å^2]**	30.23
**Band gap (PBE) [eV]**	N/A

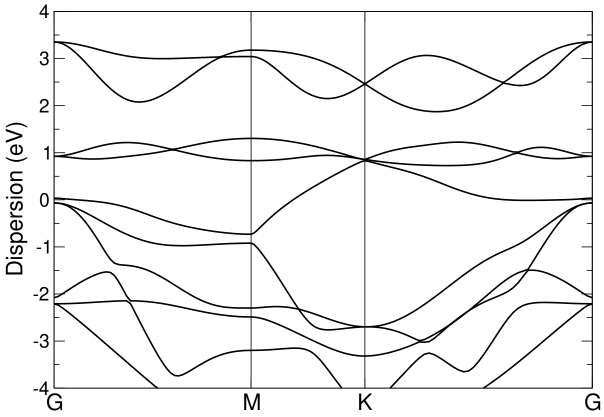

Band structure: Electronic band structure of VSe_2 (P-3m1) in a representative window around the Fermi energy and along a high-symmetry path.

Optimized crystal structure

Structural parameters: relaxed cell (top) and atomic positions (bottom) of VSe_2 (P-3m1) in Cartesian coordinates.

	x [Å]	y [Å]	z [Å]
$\mathbf{a_1}$	3.32705423	0.00000000	0.00000000
$\mathbf{a_2}$	−1.66352712	2.88131349	0.00000000
$\mathbf{a_3}$	0.00000000	0.00000000	23.15905016

		x [Å]	y [Å]	z [Å]
●	Se	1.66352712	0.96043783	9.99874419
●	V	0.00000000	0.00000000	11.57952508
●	Se	0.00000000	1.92087566	13.16030597

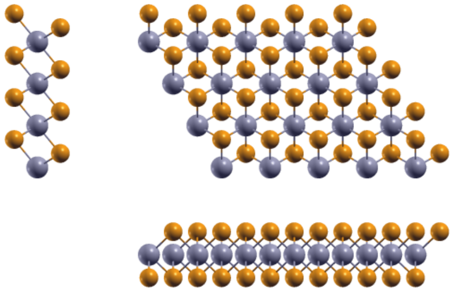

Orthographic projections: views of VSe_2 (P-3m1) as seen from the x axis (left), the y axis (bottom) and the z axis (center).

Lattice matching - minimal size

Formula	N° atoms	strain	cell size 1	cell size 2
Tl	4	0.1501	1	1
Tl	4	0.0009	1	1
Sn	4	0.1244	1	1
In	4	0.1273	1	1
GeTe	5	3.0159	1	1
P_2	5	0.006	1	1
Mg_2	5	0.1358	1	1
$IrTe_2$	6	3.0282	1	1
$CdCl_2$	6	3.0016	1	1
Cl_2Mn	6	0.0049	1	1
CdI_2	6	13.5972	1	1
$AgTe_2$	6	0.153	1	1
PSn_2	6	0.455	1	1
$MoSe_2$	6	0.0016	1	1
S_2Ta	6	0.0071	1	1
Br_2Zn	6	0.482	1	1
Br_2Ca	6	13.6688	1	1
$InSe_2$	6	3.0066	1	1
$AsSn_2$	6	0.4644	1	1
$SiTe_2$	6	0.4928	1	1
I_2Mn	6	3.0032	1	1
S_2Zr	6	0.4532	1	1
BiClTe	6	13.6182	1	1
Cl_2Ti	6	0.0059	1	1
FeI_2	6	2.9737	1	1
I_2Ni	6	2.9907	1	1
Mg_3	6	0.1446	1	1
Te_2Ti	6	0.4828	1	1
NbS_2	6	0.0066	1	1
$RhTe_2$	6	0.4715	1	1
N_2W	6	0.2734	1	1
NbS_2	6	0.0022	1	1
S_2Ta	6	0.0013	1	1
$NiTe_2$	6	2.849	1	1
Cl_2Cu	6	0.1212	1	1
S_2Sn	6	0.4538	1	1
I_2V	6	0.4964	1	1
Se_2Zr	6	0.4939	1	1
$PtSe_2$	6	0.4661	1	1
GeS_2	6	0.2086	1	1
$TaTe_2$	6	0.463	1	1
$FeSe_2$	6	0.1206	1	1
Br_2Mg	6	2.972	1	1
$NbTe_2$	6	0.4527	1	1
F_2Na	6	0.4871	1	1
N_2Re	6	1.5574	1	1
I_2Pr	6	13.6232	1	1
$HfSe_2$	6	0.4828	1	1
Se_2W	6	0.0014	1	1
H_2Si_2	7	3.0108	1	1

Table showing the first 50 lattice matching combinations with the smallest number of atoms in the supercell.

Lattice matching - minimal strain

Formula	N° atoms	strain	cell size 1	cell size 2
Gd_2GeI_2	488	0.0	81	49
Gd_2I_2	139	0.0	25	16
Sb_2Te_2	247	0.0001	49	25
CdI_2	294	0.0001	61	37
CdI_2	390	0.0001	81	49
$HfSe_2$	390	0.0002	73	57
Ni_2Te_2	291	0.0002	49	36
CdClHO	624	0.0002	100	81
Te_2Ti	390	0.0002	73	57
$Ge_2I_2La_2$	102	0.0002	16	9
$Hf_2I_2N_2$	786	0.0002	100	81
Bi_2Se_3	155	0.0002	25	16
Br_2La_2P	368	0.0002	61	37
BiClTe	390	0.0002	81	49
Br_2La	123	0.0003	25	16
N_3W_2	467	0.0003	49	64
Co_2S_2	387	0.0003	65	48
I_2Pr	390	0.0003	81	49
$PtSe_2$	492	0.0003	91	73
Bi_2STe_2	368	0.0003	61	37
Cu_2I_2	139	0.0003	25	16
Br_2Gd_2Ge	155	0.0003	25	16
I_2V	339	0.0004	64	49
$CdCl_2$	255	0.0004	49	36
Ga_2Se_2	499	0.0004	81	64
CaCl	291	0.0004	65	48
I_2Ni	255	0.0005	49	36
Br_2Zn	390	0.0005	73	57
I_2Mn	255	0.0005	49	36
$MnSe_2$	339	0.0005	65	48
CdI_2	294	0.0005	61	37
$F_2Ho_2Se_2$	258	0.0005	36	25
Se_2Zr	339	0.0006	64	49
$Cl_2Er_2H_2$	363	0.0006	49	36
CCl_2Gd_2	504	0.0006	73	57
I_2Mg	123	0.0006	25	16
Gd_2GeI_2	368	0.0006	61	37
$GeTe_2$	255	0.0006	49	36
$I_2N_2Zr_2$	627	0.0006	81	64
O_2Sn_2	431	0.0006	77	50
$As_2Co_2Li_2$	840	0.0006	118	81
K	137	0.0007	39	20
$LiMnTe_2$	331	0.0007	61	37
$Cl_2Ho_2O_2$	486	0.0007	64	49
C_2Li_2	633	0.0007	115	72
Br_2Pr_2	447	0.0007	73	57
$TaTe_2$	543	0.0007	100	81
$InSe_2$	255	0.0007	49	36
$Cl_2Er_2O_2$	486	0.0007	64	49
$Ge_2Hf_2Te_2$	840	0.0008	118	81

Table showing the first 50 lattice matching combinations with the smallest strain needed to build the supercell.

VTe_2 (P-3m1)

Structural and electronic properties

**Formula**	VTe_2
**Spacegroup**	P-3m1
**Prototype**	CdI2
**Parent 3D**	VTe_2
**Source DB**	ICSD
**DB ID**	603582
**DF2-C09 Binding energy [meV/ Å^2]**	27.11
**RVV10 Binding energy [meV/ Å^2]**	30.12
**Band gap (PBE) [eV]**	N/A

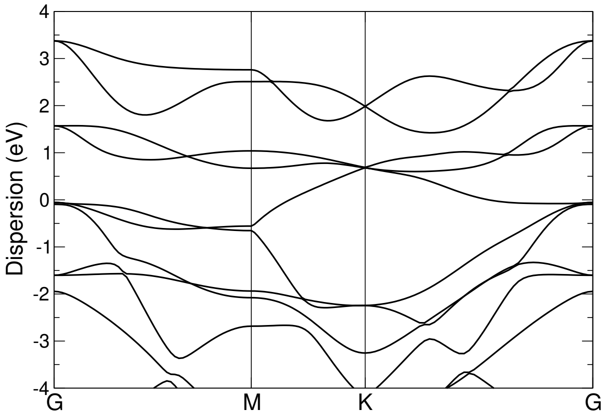

Band structure: Electronic band structure of VTe_2 (P-3m1) in a representative window around the Fermi energy and along a high-symmetry path.

Optimized crystal structure

Structural parameters: relaxed cell (top) and atomic positions (bottom) of VTe_2 (P-3m1) in Cartesian coordinates.

	x [Å]	y [Å]	z [Å]
$\mathbf{a_1}$	3.57995565	−0.00000000	0.00000000
$\mathbf{a_2}$	−1.78997782	3.10033253	0.00000000
$\mathbf{a_3}$	0.00000000	0.00000000	23.39968620

		x [Å]	y [Å]	z [Å]
●	Te	1.78997782	1.03344418	9.95991342
●	V	-0.00000000	-0.00000000	11.69984310
●	Te	0.00000000	2.06688836	13.43977279

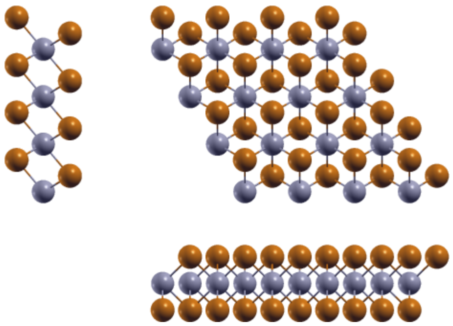

Orthographic projections: views of VTe_2 (P-3m1) as seen from the x axis (left), the y axis (bottom) and the z axis (center).

Lattice matching - minimal size

Formula	N° atoms	strain	cell size 1	cell size 2
K	4	0.3182	1	1
Tl	4	0.1231	1	1
Sn	4	0.4309	1	1
Na	4	0.0041	1	1
In	4	0.2548	1	1
HgO	5	0.1311	1	1
As_2	5	0.0038	1	1
LiO	5	0.2697	1	1
Mg_2	5	0.1147	1	1
Sb_2	5	0.4993	1	1
CrS_2	6	1.5411	1	1
Cl_2Zn	6	0.0027	1	1
I_2Mg	6	2.962	1	1
S_2V	6	0.2592	1	1
MoS_2	6	0.2602	1	1
$MoTe_2$	6	0.0019	1	1
$AgTe_2$	6	0.1249	1	1
HfS_2	6	0.0066	1	1
$HfTe_2$	6	0.459	1	1
$CuTe_2$	6	0.0062	1	1
Br_2La	6	2.9665	1	1
Br_2Co	6	0.0034	1	1
ReS_2	6	1.5846	1	1
Ca_2N	6	0.0043	1	1
$AuTe_2$	6	0.4786	1	1
$PdTe_2$	6	0.4724	1	1
Mg_3	6	0.1197	1	1
I_2Zn	6	2.8459	1	1
Te_2Zn	6	0.0021	1	1
S_2W	6	0.2603	1	1
Bi_2Pd	6	0.1385	1	1
Br_2Mn	6	0.0015	1	1
PtS_2	6	0.0031	1	1
$CoTe_2$	6	0.0069	1	1
CdClO	6	0.0005	1	1
Ba_2N	6	0.4628	1	1
Se_2Ti	6	0.0057	1	1
AsKSn	6	3.029	1	1
Te_2Zr	6	0.4602	1	1
Te_2W	6	0.0018	1	1
Cl_2Cu	6	0.5912	1	1
Cl_2V	6	0.266	1	1
OTl_2	6	0.0003	1	1
Br_2Fe	6	0.0034	1	1
Br_2Ni	6	0.0086	1	1
MoS_2	6	0.2605	1	1
Cl_2Mg	6	0.0087	1	1
$CrSe_2$	6	0.2643	1	1
$PtTe_2$	6	0.4776	1	1
Br_2Cd	6	0.47	1	1

Table showing the first 50 lattice matching combinations with the smallest number of atoms in the supercell.

Lattice matching - minimal strain

Formula	N° atoms	strain	cell size 1	cell size 2
$CrSe_2$	492	0.0	73	91
$F_2Ho_2Se_2$	711	0.0	91	73
S_2V	435	0.0001	64	81
GeI_2La_2	155	0.0001	25	16
FHOZn	583	0.0001	73	91
$MnNaTe_2$	291	0.0001	49	36
$CrSe_2$	543	0.0002	81	100
Bi_2	158	0.0002	36	25
I_2Tm	123	0.0002	25	16
BH_4Li	429	0.0002	57	43
$Br_2Gd_2O_2$	483	0.0002	65	48
$Ga_2Gd_2I_2$	258	0.0003	36	25
$Cl_2Hf_2N_2$	9	0.0003	1	1
OTl_2	6	0.0003	1	1
$F_2Lu_2Se_2$	486	0.0003	64	49
Br_2Ho_2	499	0.0003	81	64
Te_2Zr	543	0.0004	100	81
Cu_3Se_3	786	0.0004	100	81
$C_2Br_2Tb_2$	156	0.0004	20	16
$CeLi_2P_2$	386	0.0004	57	43
$C_2Br_2Gd_2$	156	0.0005	20	16
F_2Zn	339	0.0005	65	48
In	244	0.0005	57	73
$PbTe_2$	255	0.0005	49	36
SSb_2Te_2	327	0.0005	49	36
Cl_2La_2	447	0.0005	73	57
CdClO	6	0.0005	1	1
$SnTe_2$	300	0.0005	57	43
BrCdI	255	0.0005	49	36
$I_2Nd_2S_2$	537	0.0005	81	49
$I_2Lu_2O_2$	483	0.0005	65	48
$Ga_2I_2Tb_2$	258	0.0006	36	25
N_2Re	123	0.0006	16	25
As_2Sn_2	447	0.0006	73	57
Bi_2S_3	563	0.0006	81	64
$Br_2F_2Tm_2$	483	0.0006	65	48
Au_2I_2	552	0.0006	104	60
$PtTe_2$	435	0.0006	81	64
BN	347	0.0006	49	100
Ba_2N	543	0.0006	100	81
MoS_2	435	0.0007	64	81
Br_2Gd_2	447	0.0007	73	57
ClH_3O	93	0.0007	16	9
S_2W	435	0.0007	64	81
Bi_2SeTe_2	233	0.0007	36	25
$Ca_2Ge_2Mn_2$	483	0.0008	65	48
Ag_2Te_2	635	0.0008	105	80
CrS_2	255	0.0008	36	49
$Br_2Ca_2F_2$	483	0.0008	65	48
$F_2I_2Tm_2$	840	0.0008	118	81

Table showing the first 50 lattice matching combinations with the smallest strain needed to build the supercell.

W_2N_3 (P-6m2)

Structural and electronic properties

**Formula**	W_2N_3
**Spacegroup**	P-6m2
**Prototype**	W2N3
**Parent 3D**	W_4N_6
**Source DB**	ICSD
**DB ID**	186207
**DF2-C09 Binding energy [meV/ Å^2]**	26.34
**RVV10 Binding energy [meV/ Å^2]**	39.83
**Band gap (PBE) [eV]**	N/A

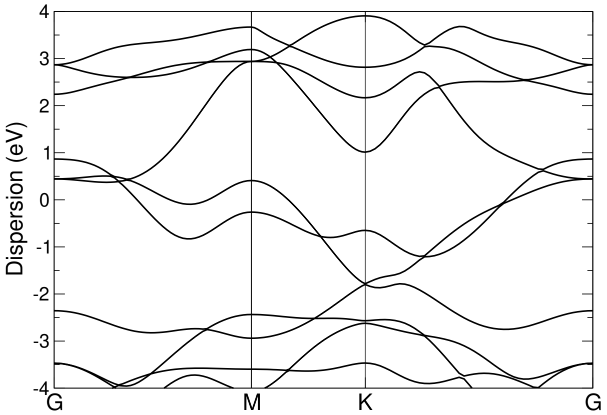

Band structure: Electronic band structure of W_2N_3 (P-6m2) in a representative window around the Fermi energy and along a high-symmetry path.

Optimized crystal structure

Structural parameters: relaxed cell (top) and atomic positions (bottom) of W_2N_3 (P-6m2) in Cartesian coordinates.

		x [Å]	y [Å]	z [Å]
$\mathbf{a_1}$		2.91306148	0.00000000	0.00000000
$\mathbf{a_2}$		−1.45653074	2.52278524	0.00000000
$\mathbf{a_3}$		0.00000000	0.00000000	25.15188691
		x [Å]	y [Å]	z [Å]
●	W	1.45653074	0.84092841	11.10775268
●	W	1.45653074	0.84092841	14.04413423
•	N	0.00000000	1.68185683	9.99423670
•	N	0.00000000	1.68185683	15.15765021
•	N	0.00000000	1.68185683	12.57594345

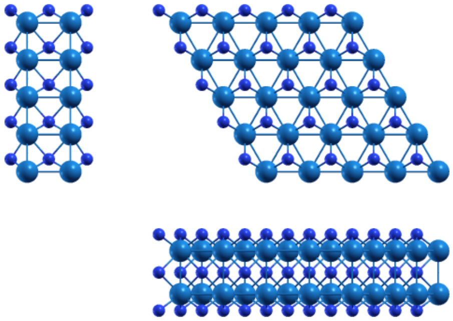

Orthographic projections: views of W_2N_3 (P-6m2) as seen from the x axis (left), the y axis (bottom) and the z axis (center).

Lattice matching - minimal size

Formula	N° atoms	strain	cell size 1	cell size 2
Tl	6	2.8545	1	1
Sm	6	0.2656	1	1
LiO	7	0.4593	1	1
BN	7	1.5638	1	1
P_2	7	0.4786	1	1
Cl_2Mn	8	2.9287	1	1
$MoSe_2$	8	2.8449	1	1
Cl_2Ti	8	0.479	1	1
S_2Ti	8	3.0222	1	1
Cl_2Co	8	3.0175	1	1
NbS_2	8	0.5004	1	1
Cl_2Fe	8	3.0078	1	1
S_2Ta	8	0.4982	1	1
Se_2V	8	0.4946	1	1
Br_2Ti	8	3.1951	1	1
Cl_2Cu	8	0.9642	1	1
Cl_2V	8	0.4531	1	1
GeS_2	8	0.317	1	1
Cl_2Zr	8	3.0131	1	1
$CrSe_2$	8	0.4502	1	1
$CrSe_2$	8	0.4562	1	1
N_2Re	8	0.0084	1	1
Se_2W	8	2.8471	1	1
Cl_2Y_2	9	13.6497	1	1
Br_2OV	9	0.3272	1	1
C_2F_2	9	0.2634	1	1
As_2Fe_2	9	0.3188	1	1
O_2Sn_2	9	1.2895	1	1
Ga_2Se_2	9	13.6081	1	1
FHOZn	9	0.4505	1	1
CS_2Ta_2	10	0.4792	1	1
H_2Na_2Pd	10	0.3154	1	1
Cl_2NSc_2	10	2.9349	1	1
H_2MnO_2	10	0.0024	1	1
H_2Li_2Pd	10	0.2077	1	1
CNb_2S_2	10	0.4773	1	1
H_4Ti	10	0.2085	1	1
$Hf_2Se_2Si_2$	11	0.3198	1	1
Cu_4Te_2	11	2.1219	1	1
$Cl_2H_2Zr_2$	11	0.5012	1	1
$Se_2Si_2Zr_2$	11	0.3282	1	1
C	11	0.1637	2	1
HN_3OZn	11	0.4816	1	1
$H_2Li_2O_2$	11	0.333	1	1
Tl	12	0.8797	2	2
Sb_2	12	0.2199	2	1
Gd	12	0.2396	2	2
Bi_2Mn_2	13	1.6946	1	2
GeI_2	13	0.2248	2	1
CKN	13	0.3502	2	1

Table showing the first 50 lattice matching combinations with the smallest number of atoms in the supercell.

Lattice matching - minimal strain

Formula	N° atoms	strain	cell size 1	cell size 2
Ga_2S_2	189	0.0	25	16
Cu_2I_2	696	0.0	100	49
Br_2Gd_2Ge	745	0.0	100	49
$CdCl_2$	107	0.0001	16	9
Ga_2Se_2	601	0.0001	81	49
Ni_2Te_2	116	0.0001	16	9
Sb_2Se_2Te	590	0.0002	79	39
I_2Mn	107	0.0002	16	9
Ba_2Cd	812	0.0002	130	54
$F_2I_2Pb_2$	974	0.0002	130	54
Sb_2Te_2	420	0.0002	64	25
Gd_2I_2	696	0.0003	100	49
$Cl_2Er_2H_2$	134	0.0003	16	9
Sb_2Se_2Te	590	0.0003	79	39
Se_2V	467	0.0003	64	49
$I_2N_2Zr_2$	527	0.0003	61	37
I_2Zn	320	0.0003	49	25
Se_2Ta	255	0.0003	36	25
$F_2Se_2Y_2$	179	0.0003	25	9
PTe_2Ti_2	205	0.0003	25	16
$InSe_2$	107	0.0004	16	9
$LiOS_2Ti$	500	0.0004	57	43
Br_2Ni	173	0.0005	25	16
Bi_2Se_3	745	0.0005	100	49
$BiTe_2$	512	0.0005	79	39
CNb_2S_2	725	0.0005	81	64
Cl_2Mg	173	0.0005	25	16
$Cl_2H_2Sc_2$	330	0.0006	36	25
$Al_2Cl_2O_2$	862	0.0006	98	62
Br_2La	647	0.0006	100	49
HN_3OZn	707	0.0006	73	57
$Ce_2I_2Si_2$	629	0.0006	79	39
C_2F_2	729	0.0006	73	91
Ga_2Se_2	453	0.0007	61	37
LiO	662	0.0007	100	81
H_2Si_2	116	0.0007	16	9
Au_2Se_2	562	0.0007	90	28
I_2Ni	107	0.0008	16	9
Se_2Yb	512	0.0008	79	39
Cl_2Y_2	601	0.0008	81	49
$Cl_2H_2Lu_2$	221	0.0008	25	16
Cl_2Fe	353	0.0008	49	36
Cl_2OV	674	0.0008	90	56
I_2Mg	512	0.0009	79	39
Sm	456	0.0009	73	91
$GeTe_2$	107	0.0009	16	9
Mg_4	517	0.001	65	48
GeI_2	512	0.001	79	39
P_2	533	0.001	81	64
$Ca_2Cl_2F_2$	315	0.001	39	20

Table showing the first 50 lattice matching combinations with the smallest strain needed to build the supercell.

WS_2 (P-6m2)

Structural and electronic properties

**Formula**	WS_2
**Spacegroup**	P-6m2
**Prototype**	MoS2
**Parent 3D**	WS_2
**Source DB**	COD
**DB ID**	9012192
**DF2-C09 Binding energy [meV/ Å^2]**	22.66
**RVV10 Binding energy [meV/ Å^2]**	29.88
**Band gap (PBE) [eV]**	1.81

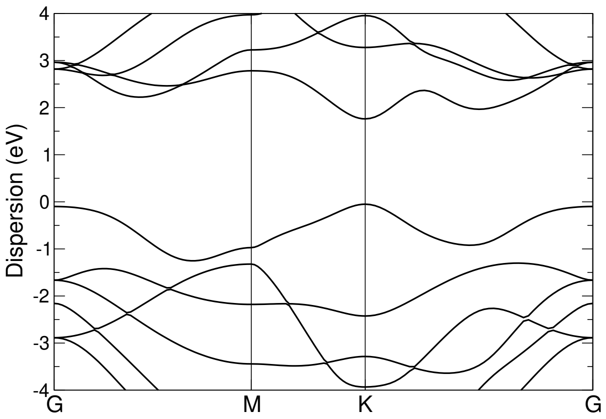

Band structure: Electronic band structure of WS_2 (P-6m2) in a representative window around the Fermi energy and along a high-symmetry path.

Optimized crystal structure

Structural parameters: relaxed cell (top) and atomic positions (bottom) of WS_2 (P-6m2) in Cartesian coordinates.

	x [Å]	y [Å]	z [Å]
$\mathbf{a_1}$	3.18718509	0.00000000	0.00000000
$\mathbf{a_2}$	−1.59359254	2.76018325	0.00000000
$\mathbf{a_3}$	0.00000000	0.00000000	23.17295525

		x [Å]	y [Å]	z [Å]
	W	1.59359254	0.92006108	11.58647762
	S	0.00000000	1.84012217	13.15871534
	S	0.00000000	1.84012217	10.01423990

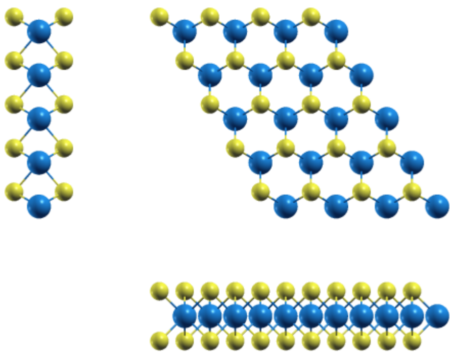

Orthographic projections: views of WS_2 (P-6m2) as seen from the x axis (left), the y axis (bottom) and the z axis (center).

Lattice matching - minimal size

Formula	N° atoms	strain	cell size 1	cell size 2
Sn	4	0.1392	1	1
Na	4	0.4635	1	1
In	4	0.143	1	1
In	4	0.004	1	1
As_2	5	0.4838	1	1
LiO	5	0.0066	1	1
Mg_2	5	0.1539	1	1
Sb_2	5	13.6453	1	1
Cl_2Zn	6	0.4811	1	1
S_2V	6	0.0008	1	1
MoS_2	6	0.0001	1	1
$MoTe_2$	6	0.4692	1	1
PSn_2	6	2.9276	1	1
HfS_2	6	2.848	1	1
FeO_2	6	0.2555	1	1
$AsSn_2$	6	2.9774	1	1
Te_2V	6	0.4741	1	1
I_2Pr	6	0.334	1	1
S_2Zr	6	2.9177	1	1
NiO_2	6	0.2573	1	1
Br_2Co	6	0.4827	1	1
Ca_2N	6	0.4852	1	1
Te_2Zn	6	0.4688	1	1
$RhTe_2$	6	3.015	1	1
Br_2Mn	6	0.478	1	1
$CrTe_2$	6	0.4502	1	1
PtS_2	6	0.4661	1	1
$CoTe_2$	6	2.8521	1	1
CdClO	6	0.4729	1	1
Se_2Ti	6	0.4595	1	1
Br_2Ti	6	0.4488	1	1
Te_2W	6	0.4696	1	1
I_2Nd	6	0.336	1	1
S_2Sn	6	2.9211	1	1
Cl_2V	6	0.0041	1	1
$PtSe_2$	6	2.986	1	1
OTl_2	6	0.4733	1	1
Br_2Fe	6	0.4829	1	1
Br_2Cr	6	0.4495	1	1
Br_2Ni	6	0.4962	1	1
CeI_2	6	0.3324	1	1
$FeSe_2$	6	0.134	1	1
$NbTe_2$	6	2.915	1	1
MoS_2	6	0.0001	1	1
Cl_2Mg	6	0.4964	1	1
$CrSe_2$	6	0.0028	1	1
$CrSe_2$	6	0.0053	1	1
O_2Pt	6	0.0055	1	1
N_2Re	6	0.2657	1	1
F_2Zn	6	0.3257	1	1

Table showing the first 50 lattice matching combinations with the smallest number of atoms in the supercell.

Lattice matching - minimal strain

Formula	N° atoms	strain	cell size 1	cell size 2
Ba_2Cu_2	247	0.0	49	25
FeO_2	390	0.0	57	73
I_2La_2Te	545	0.0	100	49
$NbTe_2$	300	0.0	57	43
MoS_2	6	0.0001	1	1
Ba_2N	123	0.0001	25	16
CCl_2Lu_2	504	0.0001	73	57
Br_2Fe	390	0.0001	73	57
$Gd_2I_2S_2$	471	0.0001	79	39
MoS_2	6	0.0001	1	1
Ga_2S_2	388	0.0002	64	49
PTe_2Ti_2	437	0.0002	64	49
$Br_2In_2O_2$	312	0.0002	48	28
Br_2Ca_3Si	594	0.0002	100	49
I_2Yb	222	0.0002	49	25
Br_2Co	390	0.0002	73	57
$AsLi_3$	84	0.0002	16	9
As_2	333	0.0002	73	57
PbS_2	183	0.0002	36	25
S_2Zr	300	0.0003	57	43
Br_2Ni	339	0.0003	64	49
PtS_2	492	0.0003	91	73
Cu_3Se_3	171	0.0003	25	16
Cl_2Mg	339	0.0004	64	49
Br_2Ca_3Si	471	0.0004	79	39
Bi_2STe_2	93	0.0004	16	9
$F_2Lu_2Se_2$	405	0.0004	61	37
$HfLiS_2$	565	0.0005	91	73
Br_2Y_2	208	0.0005	36	25
Br_2La_2	439	0.0005	81	49
InSe	66	0.0005	16	9
S_2Sn	300	0.0005	57	43
As_2Li_2Pr	93	0.0005	16	9
$I_2La_2Si_2$	102	0.0006	16	9
CdClHO	291	0.0006	49	36
$Ba_2H_2I_2$	714	0.0006	130	54
$Cl_2H_2Lu_2$	486	0.0007	64	49
Se_2Ti	543	0.0007	100	81
Se_4TiZr	600	0.0007	114	43
Sb_2	341	0.0007	81	49
$Cl_2N_2Zr_2$	396	0.0007	60	36
Te_2V	435	0.0007	81	64
Te_2Zn	492	0.0007	91	73
Ca_2N	390	0.0008	73	57
CoO_2	390	0.0008	57	73
Br_2Mn	435	0.0008	81	64
S_2V	6	0.0008	1	1
$C_2I_2Y_2$	435	0.0008	65	40
$PtSe_2$	255	0.0008	49	36
Cl_2Zn	390	0.0008	73	57

Table showing the first 50 lattice matching combinations with the smallest strain needed to build the supercell.

WSe_2 (P-6m2)

Structural and electronic properties

**Formula**	WSe_2
**Spacegroup**	P-6m2
**Prototype**	MoS2
**Parent 3D**	W_2Se_4
**Source DB**	COD
**DB ID**	9012193
**DF2-C09 Binding energy [meV/ Å^2]**	22.58
**RVV10 Binding energy [meV/ Å^2]**	29.97
**Band gap (PBE) [eV]**	1.58

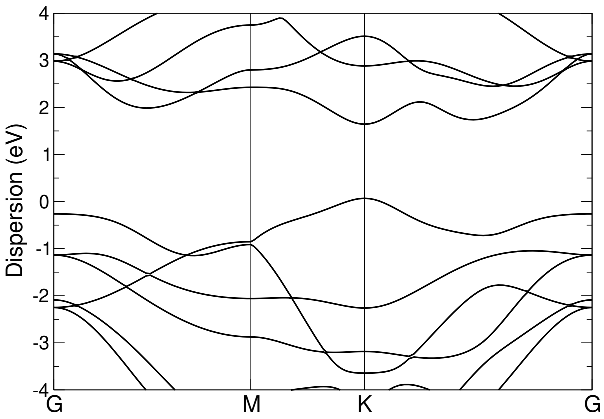

Band structure: Electronic band structure of WSe_2 (P-6m2) in a representative window around the Fermi energy and along a high-symmetry path.

Optimized crystal structure

Structural parameters: relaxed cell (top) and atomic positions (bottom) of WSe_2 (P-6m2) in Cartesian coordinates.

		x [Å]	y [Å]	z [Å]
$\mathbf{a_1}$		3.31697747	0.00000000	0.00000000
$\mathbf{a_2}$		−1.65848874	2.87258675	0.00000000
$\mathbf{a_3}$		0.00000000	0.00000000	23.36402922
		x [Å]	y [Å]	z [Å]
●	W	1.65848874	0.95752892	11.68207869
●	Se	0.00000000	1.91505784	9.99922653
●	Se	0.00000000	1.91505784	13.36480269

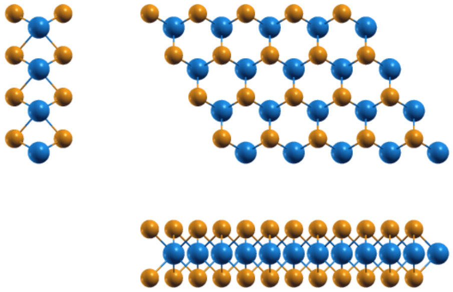

Orthographic projections: views of WSe_2 (P-6m2) as seen from the x axis (left), the y axis (bottom) and the z axis (center).

Lattice matching - minimal size

Formula	N° atoms	strain	cell size 1	cell size 2
Tl	4	0.1514	1	1
Tl	4	0.0006	1	1
Sn	4	0.1253	1	1
In	4	0.1282	1	1
GeTe	5	3.0352	1	1
P_2	5	0.0046	1	1
Mg_2	5	0.1369	1	1
$CdCl_2$	6	3.0208	1	1
Cl_2Mn	6	0.0064	1	1
CdI_2	6	13.681	1	1
$AgTe_2$	6	0.1544	1	1
PSn_2	6	0.4586	1	1
$MoSe_2$	6	0.0002	1	1
S_2Ta	6	0.0085	1	1
Br_2Zn	6	0.4857	1	1
$InSe_2$	6	3.0259	1	1
$AsSn_2$	6	0.468	1	1
$SiTe_2$	6	0.4966	1	1
I_2Mn	6	3.0224	1	1
S_2Zr	6	0.4567	1	1
NSr_2	6	2.9758	1	1
NiO_2	6	1.5244	1	1
Cl_2Ti	6	0.0045	1	1
FeI_2	6	2.9928	1	1
I_2Ni	6	3.0099	1	1
Mg_3	6	0.1459	1	1
Te_2Ti	6	0.4865	1	1
NbS_2	6	0.0081	1	1
$RhTe_2$	6	0.4752	1	1
N_2W	6	0.2755	1	1
NbS_2	6	0.0036	1	1
S_2Ta	6	0.0028	1	1
Se_2V	6	0.0014	1	1
$NiTe_2$	6	0.495	1	1
Cl_2Cu	6	0.1229	1	1
S_2Sn	6	0.4573	1	1
I_2V	6	0.5002	1	1
GeI_2	6	13.5939	1	1
Se_2Zr	6	0.4977	1	1
$PtSe_2$	6	0.4697	1	1
$TaTe_2$	6	0.4666	1	1
Br_2Ni	6	0.4483	1	1
$FeSe_2$	6	0.1214	1	1
Br_2Mg	6	2.991	1	1
$NbTe_2$	6	0.4562	1	1
Cl_2Mg	6	0.4484	1	1
Se_2Sn	6	2.9726	1	1
$HfSe_2$	6	0.4866	1	1
H_2Si_2	7	3.0301	1	1
CdClHO	7	0.4652	1	1

Table showing the first 50 lattice matching combinations with the smallest number of atoms in the supercell.

Lattice matching - minimal strain

Formula	N° atoms	strain	cell size 1	cell size 2
Cu_3Se_3	258	0.0001	36	25
I_2Pb	222	0.0001	49	25
Ga_2Se_2	343	0.0001	57	43
$TaTe_2$	492	0.0001	91	73
$LiMnTe_2$	439	0.0001	81	49
$NiTe_2$	339	0.0002	64	49
$MoSe_2$	6	0.0002	1	1
GeI_2	390	0.0002	81	49
Sb_2Se_2Te	155	0.0002	25	16
$HNiO_2$	583	0.0002	73	91
PbTe	257	0.0002	61	37
Br_2Y_2	343	0.0002	57	43
As_2CeLi_2	368	0.0003	61	37
CBr_2Y_2	504	0.0003	73	57
Sb_2Se_2Te	155	0.0003	25	16
Cl_2Y_2	447	0.0003	73	57
FeI_2	255	0.0003	49	36
$RhTe_2$	435	0.0003	81	64
Ba_2N	183	0.0003	36	25
Cd_2I_3	93	0.0004	16	9
Br_2Mg	255	0.0004	49	36
MnO_2	183	0.0004	25	36
$SiTe_2$	339	0.0004	64	49
AsI_2La_2	93	0.0005	16	9
$AsSn_2$	492	0.0005	91	73
GeI_2	294	0.0005	61	37
PbS_2	300	0.0005	57	43
Bi_2STe_2	488	0.0005	81	49
H_2MnO_2	536	0.0005	57	73
$As_2Mg_2Na_2$	237	0.0005	39	20
$BiTe_2$	123	0.0005	25	16
Tl	4	0.0006	1	1
Bi_2STe_2	368	0.0006	61	37
$Ce_2I_2Si_2$	171	0.0006	25	16
$Cl_2O_2Y_2$	429	0.0006	57	43
$Hf_2I_2N_2$	711	0.0007	91	73
CrI_2	255	0.0007	49	36
$Cl_2Er_2O_2$	486	0.0007	64	49
CdClHO	565	0.0007	91	73
Te_2Zr	183	0.0007	36	25
$I_2Pr_2S_2$	297	0.0007	49	25
CaClHO	388	0.0008	64	49
S_2Sn_2	342	0.0008	66	36
$LiMnTe_2$	331	0.0008	61	37
H_2Li_2Pt	985	0.0008	125	122
Se_2Yb	123	0.0008	25	16
$I_2N_2Zr_2$	627	0.0008	81	64
$GeTe_2$	255	0.0008	49	36
K	137	0.0008	39	20
Te_4TiZr	255	0.0008	49	18

Table showing the first 50 lattice matching combinations with the smallest strain needed to build the supercell.

WTe_2 ($P2_1/m$)

Structural and electronic properties

**Formula**	WTe_2
**Spacegroup**	$P2_1/m$
**Prototype**	WTe2
**Parent 3D**	W_4Te_8
**Source DB**	COD
**DB ID**	2310355
**DF2-C09 Binding energy [meV/ Å^2]**	22.12
**RVV10 Binding energy [meV/ Å^2]**	28.36
**Band gap (PBE) [eV]**	N/A

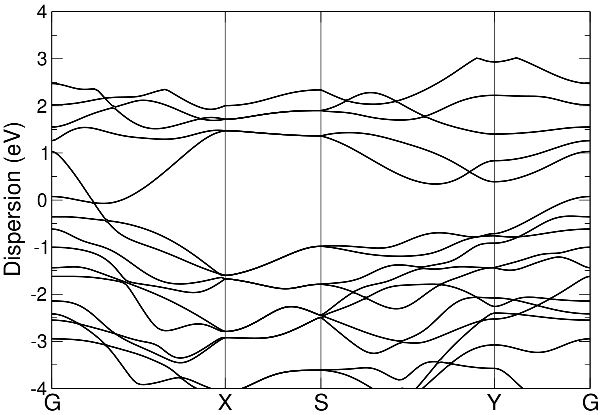

Band structure: Electronic band structure of WTe_2 ($P2_1/m$) in a representative window around the Fermi energy and along a high-symmetry path.

Optimized crystal structure

Structural parameters: relaxed cell (top) and atomic positions (bottom) of WTe_2 ($P2_1/m$) in Cartesian coordinates.

	x [Å]	y [Å]	z [Å]
$\mathbf{a_1}$	3.50033365	0.00000000	0.00000000
$\mathbf{a_2}$	0.00000000	6.32779226	0.00000000
$\mathbf{a_3}$	0.00000000	0.00000000	24.22085657

		x [Å]	y [Å]	z [Å]
●	Te	0.00000000	1.25167761	10.00127630
●	Te	1.75016682	4.47177799	10.62537054
●	Te	1.75016682	2.26924048	14.21822929
●	Te	0.00000000	5.37659893	13.59423246
●	W	0.00000000	2.88372299	12.21388294
●	W	1.75016682	0.63684934	12.00565766

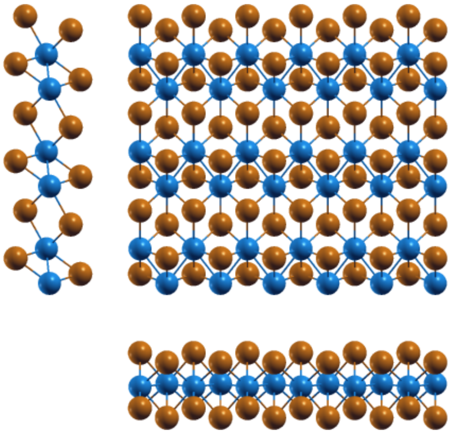

Orthographic projections: views of WTe_2 ($P2_1/m$) as seen from the x axis (left), the y axis (bottom) and the z axis (center).

Lattice matching - minimal size

Formula	N° atoms	strain	cell size 1	cell size 2
Tl	8	0.0306	1	2
Na	8	0.0105	1	2
GeTe	10	0.1869	1	2
Bi_2Se_2	10	0.2046	1	1
As_2	10	0.0112	1	2
S_2	10	0.1889	1	2
Au_2Se_2	10	0.0237	1	1
Mg_2	10	0.1105	1	2
Sb_2	10	0.5295	1	2
Br_2PY_2	11	0.9588	1	1
$CuGeO_3$	11	0.4841	1	1
$IrTe_2$	12	0.188	1	2
Cl_2Zn	12	0.0108	1	2
S_2V	12	0.2761	1	2
$CdCl_2$	12	0.1855	1	2
$MoTe_2$	12	0.0101	1	2
$AgTe_2$	12	0.0289	1	2
PSn_2	12	0.0174	1	2
$ReSe_2$	12	0.018	1	2
Cu_4Te_2	12	0.1256	1	1
HfS_2	12	0.0127	1	2
$InSe_2$	12	0.186	1	2
$AsSn_2$	12	0.0208	1	2
$GeTe_2$	12	0.1843	1	2
I_2Mn	12	0.1857	1	2
$CuTe_2$	12	0.0125	1	2
S_2Zr	12	0.0168	1	2
Br_2Co	12	0.011	1	2
$Se_2Si_2Zr_2$	12	0.2491	1	1
Cl_4Cu_2	12	0.5627	1	1
Ca_2N	12	0.0115	1	2
$K_2O_2Tl_2$	12	0.0295	1	1
I_2Ni	12	0.1845	1	2
$I_2Se_2Tb_2$	12	0.6162	1	1
Mg_3	12	0.0342	1	2
$Gd_2I_2Se_2$	12	0.6214	1	1
Te_2Zn	12	0.0101	1	2
$C_2Br_2Gd_2$	12	0.144	1	1
Br_2Mn	12	0.0104	1	2
Cl_2Ni	12	0.0173	1	2
$CrTe_2$	12	0.013	1	2
$Br_2Er_2Se_2$	12	0.592	1	1
PtS_2	12	0.0103	1	2
$CoTe_2$	12	0.0129	1	2
Br_2V	12	0.0186	1	2
Cl_4Mg_2	12	0.5603	1	1
Se_2Ti	12	0.0111	1	2
Br_2Ti	12	0.0134	1	2
$Br_2Dy_2S_2$	12	0.2372	1	1
Te_2W	12	0.0101	1	2

Table showing the first 50 lattice matching combinations with the smallest number of atoms in the supercell.

Lattice matching - minimal strain

Formula	N° atoms	strain	cell size 1	cell size 2
Br_2HLa	672	0.0004	56	84
$AlLiTe_2$	340	0.0005	30	40
$CeLi_2P_2$	756	0.0006	56	84
$AsCuLi_2$	672	0.0006	56	84
$I_2Lu_2S_2$	492	0.0006	40	42
$AsLi_3$	700	0.0008	60	85
Ga_2Te_2	672	0.0008	56	84
Li_2Tl_2	700	0.0008	70	70
Ba_2Ni_3	756	0.0008	56	84
InSe	530	0.0009	60	85
Sb_2Te_2	300	0.0009	28	33
As_2Li_2Pr	785	0.0009	60	85
$I_2La_2Si_2$	870	0.0009	60	85
Bi_2STe_2	785	0.0009	60	85
NiO_2	561	0.001	36	115
GeI_2	588	0.001	56	84
$F_2Ho_2Se_2$	750	0.001	48	77
GeS_2	57	0.001	5	9
$H_2I_2Yb_2$	654	0.001	45	64
$F_2I_2Pb_2$	162	0.001	12	15
Ge_2Te_2	452	0.0011	42	50
Ba_2Cd	117	0.0011	12	15
BH_4Li	840	0.0011	56	84
$Br_2Nd_2O_2$	654	0.0012	45	64
O_4PTl	564	0.0012	45	49
CuO_2	906	0.0012	70	162
Mg_4	134	0.0012	9	20
N_3W_2	420	0.0012	20	60
I_2Y_2	794	0.0012	63	104
H_2Na_2Pd	75	0.0012	5	9
Bi_2Te_3	380	0.0012	30	40
Se_2Yb	588	0.0012	56	84
$I_2O_2Y_2$	654	0.0013	45	64
BiITe	300	0.0013	30	40
As_2Li_2Nd	785	0.0013	60	85
I_2La	462	0.0013	45	64
$F_2Se_2Y_2$	450	0.0014	36	39
Tl	74	0.0014	9	20
H_2MnO_2	420	0.0014	20	60
$Br_2Ho_2S_2$	426	0.0014	35	36
NaO_4	931	0.0014	86	83
In_2Se_2	822	0.0014	83	81
As_2Fe_2	66	0.0014	5	9
Br_2Cu	588	0.0014	54	88
I_2Y_2	756	0.0015	60	99
Cl_2Gd_2	874	0.0015	69	115
CoO_2	561	0.0015	36	115
$BiTe_2$	588	0.0015	56	84
As_2Rh_2	526	0.0015	45	64
GeI_2Y_2	785	0.0015	60	85

Table showing the first 50 lattice matching combinations with the smallest strain needed to build the supercell.

WTe_2 (P-6m2)

Structural and electronic properties

**Formula**	WTe_2
**Spacegroup**	P-6m2
**Prototype**	MoS2
**Parent 3D**	W_2Te_4
**Source DB**	ICSD
**DB ID**	653170
**DF2-C09 Binding energy [meV/ Å^2]**	24.67
**RVV10 Binding energy [meV/ Å^2]**	30.04
**Band gap (PBE) [eV]**	1.06

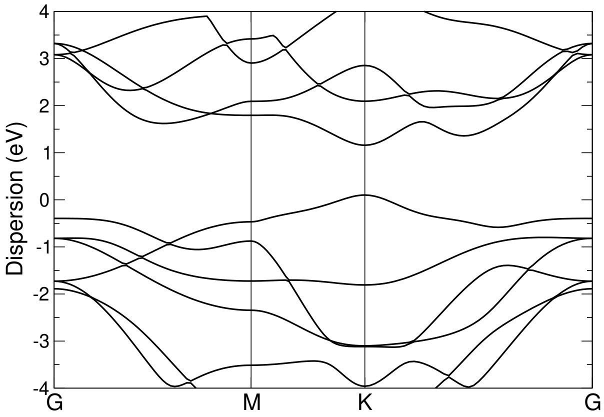

Band structure: Electronic band structure of WTe_2 (P-6m2) in a representative window around the Fermi energy and along a high-symmetry path.

Optimized crystal structure

Structural parameters: relaxed cell (top) and atomic positions (bottom) of WTe_2 (P-6m2) in Cartesian coordinates.

		x [Å]	y [Å]	z [Å]
$\mathbf{a_1}$		3.56662342	0.00000000	0.00000000
$\mathbf{a_2}$		−1.78331171	3.08878649	0.00000000
$\mathbf{a_3}$		0.00000000	0.00000000	23.66184269
		x [Å]	y [Å]	z [Å]
●	Te	0.00000000	2.05919099	10.01098363
●	Te	0.00000000	2.05919099	13.65085906
●	W	1.78331171	1.02959550	11.83092640

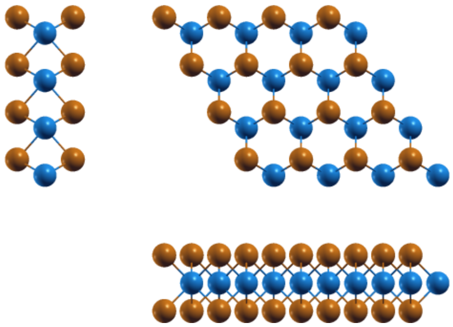

Orthographic projections: views of WTe_2 (P-6m2) as seen from the x axis (left), the y axis (bottom) and the z axis (center).

Lattice matching - minimal size

Formula	N° atoms	strain	cell size 1	cell size 2
K	4	0.3219	1	1
Tl	4	0.1241	1	1
Sn	4	0.4349	1	1
Na	4	0.0024	1	1
In	4	0.111	1	1
In	4	0.2572	1	1
HgO	5	0.1324	1	1
As_2	5	0.0056	1	1
LiO	5	0.2722	1	1
Mg_2	5	0.1155	1	1
Sb_2	5	2.9107	1	1
CrS_2	6	1.5538	1	1
Cl_2Zn	6	0.0045	1	1
I_2Mg	6	2.9853	1	1
S_2V	6	0.2617	1	1
MoS_2	6	0.2627	1	1
$MoTe_2$	6	0.0002	1	1
$AgTe_2$	6	0.126	1	1
Br_2Ca	6	3.1916	1	1
HfS_2	6	0.0084	1	1
$HfTe_2$	6	0.4634	1	1
Te_2V	6	0.0018	1	1
$CuTe_2$	6	0.008	1	1
Br_2La	6	2.9899	1	1
Br_2Cu	6	1.0748	1	1
Br_2Co	6	0.0052	1	1
ReS_2	6	1.5976	1	1
Ca_2N	6	0.0061	1	1
$AuTe_2$	6	0.4832	1	1
$PdTe_2$	6	0.4769	1	1
Mg_3	6	0.1207	1	1
I_2Zn	6	0.4952	1	1
Te_2Zn	6	0.0003	1	1
S_2W	6	0.2628	1	1
Bi_2Pd	6	0.14	1	1
GeI_2	6	2.9607	1	1
Br_2Mn	6	0.0033	1	1
$CrTe_2$	6	0.0077	1	1
PtS_2	6	0.0014	1	1
$CoTe_2$	6	0.0087	1	1
CdClO	6	0.0013	1	1
Ba_2N	6	0.4672	1	1
Se_2Ti	6	0.004	1	1
Br_2Ti	6	0.0082	1	1
Te_2Zr	6	0.4646	1	1
Cl_2Cu	6	0.5966	1	1
Cl_2V	6	0.2686	1	1
OTl_2	6	0.0015	1	1
Br_2Fe	6	0.0052	1	1
Br_2Cr	6	0.008	1	1

Table showing the first 50 lattice matching combinations with the smallest number of atoms in the supercell.

Lattice matching - minimal strain

Formula	N° atoms	strain	cell size 1	cell size 2
$HNiO_2$	219	0.0	25	36
I_2La_2Sb	368	0.0	61	37
BiClTe	183	0.0	36	25
NaPSn	543	0.0	100	81
$AuTe_2$	390	0.0	73	57
I_2Zn	339	0.0001	64	49
I_2Pr	183	0.0001	36	25
Cu_2I_2	291	0.0001	49	36
Br_2Gd_2Ge	327	0.0001	49	36
Ba_2N	492	0.0001	91	73
Cu_3Se_3	711	0.0001	91	73
Pt_2Te_2	624	0.0002	100	81
$MoTe_2$	6	0.0002	1	1
Br_2Cu_2	387	0.0002	65	48
Bi_2In_2	197	0.0002	39	20
Gd_2I_2	291	0.0002	49	36
O_2Pt	390	0.0002	57	73
H_2NiO_2	674	0.0002	73	91
Gd_2GeI_2	233	0.0003	36	25
$Br_2Hf_2N_2$	30	0.0003	4	3
Cl_2OV	519	0.0003	77	72
Li_2P_2Pr	386	0.0003	57	43
Sb_2	257	0.0003	57	43
CoH_2O_2	414	0.0003	43	57
$PdTe_2$	435	0.0003	81	64
Te_2Zn	6	0.0003	1	1
K	220	0.0004	61	37
CdI_2	183	0.0004	36	25
$Ga_2Ge_2Te_2$	429	0.0004	57	43
$Cu_2Rb_2Te_2$	237	0.0004	39	20
Ba_2Cu_2	139	0.0004	25	16
$PtTe_2$	390	0.0004	73	57
FHOZn	643	0.0004	81	100
CdH_2O_2	8	0.0004	1	1
$Br_2O_2Tb_2$	483	0.0004	65	48
Bi_2Se_3	327	0.0004	49	36
Br_2La_2	343	0.0005	57	43
Br_2La	255	0.0005	49	36
$CrSe_2$	543	0.0005	81	100
Br_2Er_2	447	0.0005	73	57
I_2Yb	123	0.0006	25	16
$Br_2F_2Yb_2$	483	0.0006	65	48
Br_2Cd	435	0.0006	81	64
$GeNi_3Te_2$	786	0.0006	100	81
$HfLiS_2$	7	0.0006	1	1
Cu_2O_2	479	0.0006	81	59
$I_2O_2Sm_2$	840	0.0006	118	81
BN	275	0.0006	39	79
$Er_2F_2Se_2$	786	0.0006	100	81
Cl_2V	543	0.0007	81	100

Table showing the first 50 lattice matching combinations with the smallest strain needed to build the supercell.

Y_2Br_2 (P-3m1)

Structural and electronic properties

**Formula**	Y_2Br_2
**Spacegroup**	P-3m1
**Prototype**	PtTe
**Parent 3D**	Br_2Y_2
**Source DB**	MPDS
**DB ID**	S541501
**DF2-C09 Binding energy [meV/ Å^2]**	12.86
**RVV10 Binding energy [meV/ Å^2]**	N/A
**Band gap (PBE) [eV]**	0.0

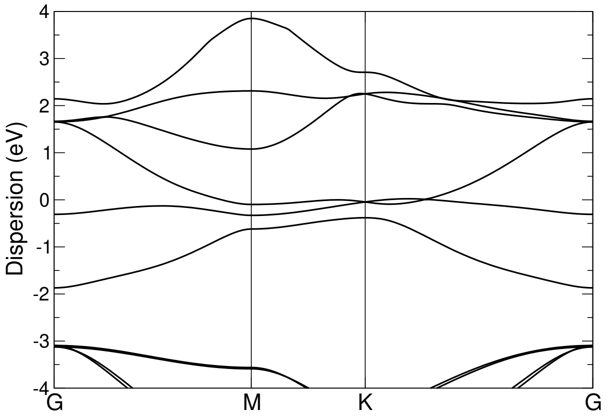

Band structure: Electronic band structure of Y_2Br_2 (P-3m1) in a representative window around the Fermi energy and along a high-symmetry path.

Optimized crystal structure

Structural parameters: relaxed cell (top) and atomic positions (bottom) of Y_2Br_2 (P-3m1) in Cartesian coordinates.

	x [Å]	y [Å]	z [Å]
$\mathbf{a_1}$	1.91039857	−3.30890739	0.00000000
$\mathbf{a_2}$	1.91039857	3.30890739	0.00000000
$\mathbf{a_3}$	0.00000000	0.00000000	25.28127189

		x [Å]	y [Å]	z [Å]
●	Y	0.95519929	0.55148456	-1.41414431
●	Br	0.95519929	-1.65445369	-3.33309009
●	Y	-0.95519929	-0.55148456	1.41414431
●	Br	0.95519929	-1.65445369	3.33309009

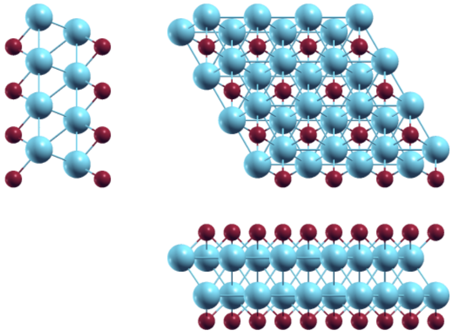

Orthographic projections: views of Y_2Br_2 (P-3m1) as seen from the x axis (left), the y axis (bottom) and the z axis (center).

Lattice matching - minimal size

Formula	N° atoms	strain	cell size 1	cell size 2
Tl	5	0.4346	1	1
InSe	6	0.4611	1	1
Nd	6	0.2234	1	2
HgO	6	0.1137	1	1
AsSb	6	0.0028	1	1
Bi_2	6	0.4757	1	1
GeTe	6	0.009	1	1
PbTe	6	0.4655	1	1
CaCl	6	0.1461	1	1
$CdCl_2$	7	0.0079	1	1
CdI_2	7	0.4699	1	1
$ReSe_2$	7	0.2711	1	1
S_2Ta	7	0.2555	1	1
Br_2Zn	7	0.0073	1	1
Br_2Ca	7	0.473	1	1
$InSe_2$	7	0.0083	1	1
$GeTe_2$	7	0.0069	1	1
$SiTe_2$	7	0.0032	1	1
I_2Mn	7	0.0081	1	1
NSr_2	7	0.0045	1	1
PbS_2	7	0.0002	1	1
BiClTe	7	0.4708	1	1
FeI_2	7	0.0058	1	1
I_2Ni	7	0.0071	1	1
S_2Ti	7	0.2624	1	1
Mg_3	7	0.4213	1	1
Te_2Ti	7	0.007	1	1
NbS_2	7	0.2549	1	1
CrI_2	7	0.0054	1	1
BaF_2	7	0.4516	1	1
BiBrTe	7	0.485	1	1
Bi_2Pd	7	0.1181	1	1
Cl_2Ni	7	0.2724	1	1
Cl_2Co	7	0.2619	1	1
NbS_2	7	0.2491	1	1
Br_2V	7	0.2699	1	1
ClNZr	7	0.2669	1	1
Cl_2Fe	7	0.2609	1	1
S_2Ta	7	0.248	1	1
Se_2V	7	0.2463	1	1
AsKSn	7	0.4481	1	1
$AsSe_2$	7	0.2746	1	1
$NiTe_2$	7	0.0038	1	1
I_2V	7	0.0019	1	1
GeI_2	7	0.4661	1	1
Se_2Zr	7	0.0028	1	1
STl_2	7	0.454	1	1
CdO_2	7	0.2617	1	1
$NbSe_2$	7	0.2731	1	1
CoI_2	7	0.0033	1	1

Table showing the first 50 lattice matching combinations with the smallest number of atoms in the supercell.

Lattice matching - minimal strain

Formula	N° atoms	strain	cell size 1	cell size 2
$LiMnTe_2$	656	0.0	91	73
InSe	562	0.0	100	81
As_2Li_2Pr	805	0.0	100	81
S_2Ta	447	0.0	57	73
$Br_2H_2Zr_2$	924	0.0001	81	100
$HNiO_2$	520	0.0001	49	81
NbS_2	388	0.0001	49	64
$I_2La_2Si_2$	886	0.0001	100	81
Bi_2	452	0.0001	81	64
$LiNbS_2$	520	0.0001	57	73
$Cl_2H_2Zr_2$	580	0.0002	49	64
Se_2W	343	0.0002	43	57
PbS_2	7	0.0002	1	1
$AsLi_3$	724	0.0003	100	81
GeI_2	583	0.0003	91	73
S_2V	208	0.0003	25	36
Bi_2STe_2	729	0.0003	91	73
Ga_2Se_2	8	0.0004	1	1
CKN	479	0.0004	83	49
Tl	229	0.0004	43	57
$MoSe_2$	343	0.0004	43	57
MoS_2	208	0.0004	25	36
NbS_2	447	0.0004	57	73
CCl_2Sc_2	824	0.0004	81	100
S_2W	208	0.0005	25	36
Bi_2SeTe_2	644	0.0005	81	64
Ba_2Cu_2	340	0.0005	49	36
ClNZr	624	0.0005	81	100
As_2CeLi_2	729	0.0005	91	73
$Ga_2Gd_2I_2$	708	0.0005	81	64
PbTe	510	0.0005	91	73
$HNiO_2$	392	0.0006	37	61
I_2La_2Sb	269	0.0006	36	25
Cu_4Te_2	634	0.0006	73	57
MoS_2	208	0.0006	25	36
I_2Yb	304	0.0007	49	36
As_2Li_2Nd	805	0.0007	100	81
BiBrTe	463	0.0007	73	57
$Ag_2K_2Te_2$	476	0.0007	65	36
CdI_2	583	0.0007	91	73
As_2O_3	511	0.0008	79	39
$Ga_2I_2Tb_2$	708	0.0008	81	64
Cl_2Zr_2	724	0.0008	81	100
N_2Re	84	0.0008	9	16
CNb_2S_2	389	0.0008	36	49
Cl_2ORu	572	0.0009	73	70
Br_2La_2P	729	0.0009	91	73
$Cl_2O_2Y_2$	10	0.0009	1	1
BiITe	357	0.0009	57	43
Bi_2STe_2	805	0.0009	100	81

Table showing the first 50 lattice matching combinations with the smallest strain needed to build the supercell.

$Y_2C_2Cl_2$ (C2/m)

Structural and electronic properties

**Formula**	$Y_2C_2Cl_2$
**Spacegroup**	C2/m
**Prototype**	Gd2C2Br2
**Parent 3D**	$C_2Cl_2Y_2$
**Source DB**	MPDS
**DB ID**	S1708524
**DF2-C09 Binding energy [meV/ Å^2]**	11.05
**RVV10 Binding energy [meV/ Å^2]**	N/A
**Band gap (PBE) [eV]**	0.0

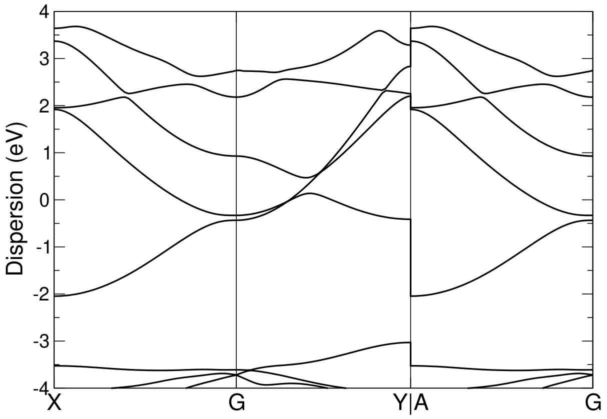

Band structure: Electronic band structure of $Y_2C_2Cl_2$ (C2/m) in a representative window around the Fermi energy and along a high-symmetry path.

Optimized crystal structure

Structural parameters: relaxed cell (top) and atomic positions (bottom) of $Y_2C_2Cl_2$ (C2/m) in Cartesian coordinates.

		x [Å]	y [Å]	z [Å]
$\mathbf{a_1}$		0.00000000	3.74211685	0.00000000
$\mathbf{a_2}$		−3.43560455	1.87105843	0.00000000
$\mathbf{a_3}$		0.00000000	0.00000000	24.73667791
		x [Å]	y [Å]	z [Å]
●	Y	-0.84752339	1.87105843	-1.48984552
●	C	0.54075208	0.00000000	-0.39112981
●	Cl	-1.90575415	0.00000000	-3.19513490
●	Y	0.84752339	1.87105843	1.48984552
●	C	-0.54075208	0.00000000	0.39112981
●	Cl	-1.52985040	1.87105843	3.19513490

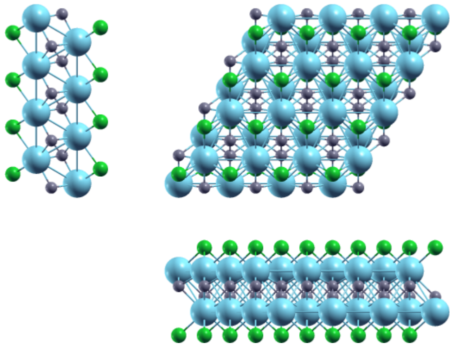

Orthographic projections: views of $Y_2C_2Cl_2$ (C2/m) as seen from the x axis (left), the y axis (bottom) and the z axis (center).

Lattice matching - minimal size

Formula	N° atoms	strain	cell size 1	cell size 2
Tl	7	0.4044	1	1
CaCl	8	0.1346	1	1
I_2Pr	9	0.1741	1	1
Bi_2Pd	9	0.1102	1	1
N_2W	9	0.1864	1	1
I_2Nd	9	0.1753	1	1
GeS_2	9	0.1253	1	1
$MnSe_2$	9	0.1345	1	1
CeI_2	9	0.1732	1	1
CNNa	9	0.2441	1	1
I_2La	9	0.1812	1	1
Sm	9	0.573	1	3
Cu_2Te_2	10	0.1552	1	1
Ir_2P_2	10	0.175	1	1
Ag_2Br_2	10	0.1819	1	1
$AgNO_2$	10	0.2646	1	1
$AgCuTe_2$	10	0.1738	1	1
O_2Sn_2	10	0.1721	1	1
Ca_2O_2	10	0.1107	1	1
Cl_2OV	10	0.1232	1	1
As_2Ir_2	10	0.1849	1	1
Ge_2S_2	10	0.8249	1	1
P_4	10	0.2226	1	1
Mg_4	10	0.4037	1	1
Fe_2S_2	10	0.1329	1	1
As_2Fe_2	10	0.1259	1	1
Ag_2Te_2	10	0.1635	1	1
C_2	10	0.1569	1	2
Ni_2Se_2	10	0.1729	1	1
As_2Rh_2	10	0.1815	1	1
NS_2Ta	10	0.1847	1	1
H_2Na_2Pd	11	0.1248	1	1
H_4Ti	11	0.3758	1	1
$Br_2In_2O_2$	12	0.1805	1	1
CrS_2	12	0.283	1	2
$Er_2I_2O_2$	12	0.1775	1	1
$Hf_2Se_2Si_2$	12	0.1263	1	1
$ReSe_2$	12	0.4382	1	2
$C_2I_2La_2$	12	0.4331	1	1
$Cl_2O_2Sc_2$	12	0.1595	1	1
$Br_2N_2Zr_2$	12	0.1797	1	1
$I_2N_2Zr_2$	12	0.1858	1	1
Br_2Cu	12	0.6984	1	2
ReS_2	12	0.2935	1	2
$Se_2Si_2Zr_2$	12	0.1293	1	1
$Cl_2N_2Zr_2$	12	0.1773	1	1
$Br_2Cr_2S_2$	12	0.2134	1	1
$Br_2O_2Sc_2$	12	0.1626	1	1
$Br_2Ce_2O_2$	12	0.1874	1	1
$H_2Na_2O_2$	12	0.117	1	1

Table showing the first 50 lattice matching combinations with the smallest number of atoms in the supercell.

Lattice matching - minimal strain

Formula	N° atoms	strain	cell size 1	cell size 2
CrO_2	333	0.0002	28	55
Cl_2Gd_2	788	0.0003	80	77
CI_2Y_2	638	0.0003	58	58
Se_2Sn	522	0.0004	58	58
Ir_2P_2	880	0.0005	94	79
I_2Ti	522	0.0005	58	58
I_2Nd	801	0.0006	94	79
NSr_2	522	0.0006	58	58
F_4Nb	646	0.0007	66	50
$BiTe_2$	576	0.0007	67	58
$SnTe_2$	543	0.0007	63	55
PbS_2Sn	888	0.0007	100	72
Se_2Yb	576	0.0007	67	58
$I_2Lu_2Se_2$	708	0.0007	78	40
I_2Pr	801	0.0008	94	79
CoI_2	522	0.0008	58	58
Sb_2Se_2Te	692	0.0008	67	58
GeI_2	576	0.0008	67	58
$C_2I_2La_2$	984	0.0008	91	73
$I_2Se_2Tb_2$	138	0.0008	15	8
I_4Sr_2	822	0.0008	103	34
$I_2O_2Tm_2$	948	0.0009	86	72
Ho_2S_2	828	0.0009	88	75
I_2Nd	732	0.0009	86	72
In	663	0.0009	89	129
I_2Y_2	788	0.0009	80	77
Te_2V	549	0.0009	58	67
Ba_2Ni_3	692	0.0009	67	58
$Br_2O_2Sm_2$	948	0.001	86	72
Ga_2Te_2	634	0.001	67	58
$I_2Pr_2Si_2$	750	0.001	67	58
$SbSe_2Tl$	964	0.0011	116	67
Ir_2P_2	804	0.0011	86	72
$Cu_2K_2Te_2$	426	0.0011	44	27
Sb_2Se_2Te	692	0.0011	67	58
Br_2Mn	549	0.0011	58	67
ClH_3O	919	0.0011	99	65
$AsCuLi_2$	634	0.0011	67	58
$Cl_2Hf_2N_2$	750	0.0012	58	67
OTl_2	549	0.0012	58	67
AsSb	464	0.0012	58	58
$AuTe_2$	594	0.0012	68	62
PSn_2	576	0.0012	62	68
$Br_2F_2Sr_2$	696	0.0012	66	50
$H_2I_2Sr_2$	816	0.0012	80	56
$PtTe_2$	594	0.0012	68	62
H_4Ti	998	0.0012	78	106
$I_2Pr_2Si_2$	708	0.0013	63	55
Gd_2I_2	746	0.0013	79	68
CdClO	549	0.0013	58	67

Table showing the first 50 lattice matching combinations with the smallest strain needed to build the supercell.

Y_2CBr_2 (P-3m1)

Structural and electronic properties

**Formula**	Y_2CBr_2
**Spacegroup**	P-3m1
**Prototype**	Bi2Te2S
**Parent 3D**	CBr_2Y_2
**Source DB**	MPDS
**DB ID**	S1708509
**DF2-C09 Binding energy [meV/ Å^2]**	12.8
**RVV10 Binding energy [meV/ Å^2]**	N/A
**Band gap (PBE) [eV]**	0.87

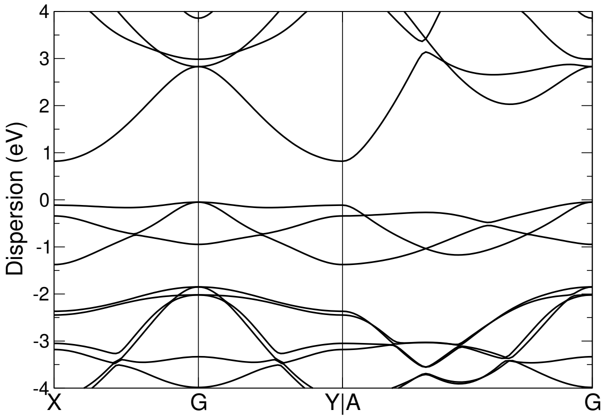

Band structure: Electronic band structure of Y_2CBr_2 (P-3m1) in a representative window around the Fermi energy and along a high-symmetry path.

Optimized crystal structure

Structural parameters: relaxed cell (top) and atomic positions (bottom) of Y_2CBr_2 (P-3m1) in Cartesian coordinates.

		x [Å]	y [Å]	z [Å]
$\mathbf{a_1}$		1.87793590	−3.25268039	0.00000000
$\mathbf{a_2}$		1.87797804	3.25270472	0.00000000
$\mathbf{a_3}$		0.00000000	0.00000000	25.04060403
		x [Å]	y [Å]	z [Å]
●	Y	2.81693715	-0.54209417	1.34591383
●	Br	0.93897771	0.54211904	3.27989864
●	Y	0.93897679	0.54211850	-1.34591383
●	Br	2.81693623	-0.54209470	-3.27989864
●	C	0.93896795	-1.62634020	0.00000000

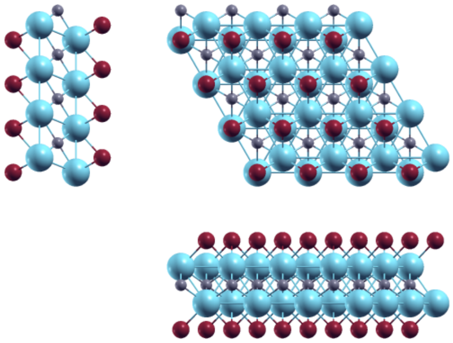

Orthographic projections: views of Y_2CBr_2 (P-3m1) as seen from the x axis (left), the y axis (bottom) and the z axis (center).

Lattice matching - minimal size

Formula	N° atoms	strain	cell size 1	cell size 2
Tl	6	0.1122	1	1
Tl	6	0.2559	1	1
InSe	7	0.4817	1	1
HgO	7	0.1173	1	1
Bi_2	7	0.4969	1	1
P_2	7	0.2488	1	1
PbTe	7	0.4862	1	1
CaCl	7	0.1538	1	1
I_2Mg	8	0.4556	1	1
Cl_2Mn	8	0.2639	1	1
$AgTe_2$	8	0.1133	1	1
$MoSe_2$	8	0.2549	1	1
S_2Ta	8	0.2668	1	1
Br_2Zn	8	0.0007	1	1
Br_2Ca	8	0.494	1	1
$AsSn_2$	8	0.0061	1	1
$SiTe_2$	8	0.0049	1	1
Br_2La	8	0.4565	1	1
PbS_2	8	0.0085	1	1
Cl_2Ti	8	0.2491	1	1
BrCdI	8	0.4634	1	1
S_2Ti	8	0.2741	1	1
Te_2Ti	8	0.001	1	1
NbS_2	8	0.2662	1	1
BaF_2	8	0.4718	1	1
$RhTe_2$	8	0.0033	1	1
Bi_2Pd	8	0.1225	1	1
GeI_2	8	0.4511	1	1
Cl_2Co	8	0.2736	1	1
NbS_2	8	0.2601	1	1
Cl_2Fe	8	0.2725	1	1
S_2Ta	8	0.2589	1	1
Se_2V	8	0.257	1	1
AsKSn	8	0.4682	1	1
$PbTe_2$	8	0.461	1	1
$NiTe_2$	8	0.0042	1	1
I_2V	8	0.0062	1	1
GeI_2	8	0.4869	1	1
Se_2Zr	8	0.0053	1	1
STl_2	8	0.4742	1	1
$PtSe_2$	8	0.0055	1	1
CdO_2	8	0.2734	1	1
GeS_2	8	0.1425	1	1
$TaTe_2$	8	0.0067	1	1
$MnSe_2$	8	0.1537	1	1
DyI_2	8	3.191	1	1
Cl_2Zr	8	0.2731	1	1
CuO_2	8	1.2182	1	1
Se_2Yb	8	0.4516	1	1
$BiTe_2$	8	0.4523	1	1

Table showing the first 50 lattice matching combinations with the smallest number of atoms in the supercell.

Lattice matching - minimal strain

Formula	N° atoms	strain	cell size 1	cell size 2
I_2La_2	747	0.0	91	73
KNO_3	325	0.0	49	16
FeO_2	93	0.0001	9	16
CS_2Ta_2	565	0.0001	49	64
$PbTe_2$	743	0.0001	100	81
NS_2Ta	180	0.0001	16	25
$Ga_2Gd_2I_2$	614	0.0001	64	49
$Dy_2I_2S_2$	330	0.0001	36	25
Cl_2NSc_2	820	0.0002	73	91
Cl_2Ti	437	0.0002	49	64
$Ga_2I_2Tb_2$	614	0.0002	64	49
$AlLiTe_2$	389	0.0002	49	36
Se_2W	504	0.0003	57	73
S_2Ta	563	0.0003	64	81
$AsLi_3$	593	0.0003	73	57
P_2	373	0.0003	49	64
Tl	358	0.0003	57	73
Cl_2Mn	638	0.0003	73	91
Bi_2STe_2	650	0.0003	73	57
$Eu_2F_2I_2$	613	0.0003	65	48
$Br_2La_2O_2$	613	0.0004	65	48
In_2Te_3	725	0.0004	81	64
H_2MnO_2	490	0.0004	37	61
$MoSe_2$	504	0.0004	57	73
H_2Li_2Pt	90	0.0004	8	10
$LiNbS_2$	805	0.0005	81	100
Sb_2SeTe_2	725	0.0005	81	64
O_2Pb_2	517	0.0005	65	48
AsKSn	674	0.0005	91	73
$MnNaTe_2$	824	0.0005	100	81
S_2Ta	705	0.0005	81	100
Br_2Ca	467	0.0005	64	49
$CrSe_2$	327	0.0005	36	49
Cl_2Y_2	9	0.0005	1	1
Bi_2	418	0.0005	64	49
NbS_2	563	0.0006	64	81
CCl_2Gd_2	10	0.0006	1	1
InSe	479	0.0006	73	57
As_2Li_2Pr	650	0.0006	73	57
Cu_4Te_2	543	0.0006	57	43
BiBrTe	414	0.0007	57	43
$I_2La_2Si_2$	707	0.0007	73	57
$F_2I_2Sm_2$	613	0.0007	65	48
Cl_2Hf_2	805	0.0007	81	100
CoO_2	93	0.0007	9	16
STl_2	597	0.0007	81	64
Br_2Zn	8	0.0007	1	1
Cl_4Mn	505	0.0007	65	36
Cl_2V	327	0.0007	36	49
$C_2Br_2La_2$	234	0.0007	24	19

Table showing the first 50 lattice matching combinations with the smallest strain needed to build the supercell.

Y_2CI_2 (P-3m1)

Structural and electronic properties

**Formula**	Y_2CI_2
**Spacegroup**	P-3m1
**Prototype**	Bi2Te2S
**Parent 3D**	CI_2Y_2
**Source DB**	MPDS
**DB ID**	S1708508
**DF2-C09 Binding energy [meV/ Å^2]**	15.15
**RVV10 Binding energy [meV/ Å^2]**	N/A
**Band gap (PBE) [eV]**	0.54

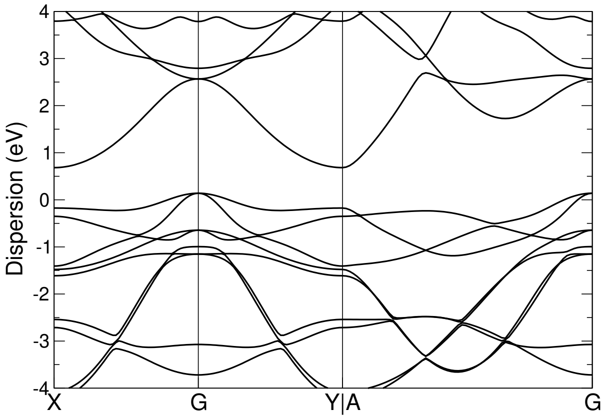

Band structure: Electronic band structure of Y_2CI_2 (P-3m1) in a representative window around the Fermi energy and along a high-symmetry path.

Optimized crystal structure

Structural parameters: relaxed cell (top) and atomic positions (bottom) of Y_2CI_2 (P-3m1) in Cartesian coordinates.

	x [Å]	y [Å]	z [Å]
$\mathbf{a_1}$	1.92678108	−3.33726539	0.00000000
$\mathbf{a_2}$	1.92676607	3.33725672	0.00000000
$\mathbf{a_3}$	0.00000000	0.00000000	25.89678827

	x [Å]	y [Å]	z [Å]
Y	0.96338256	-0.55620918	-1.31711332
I	0.96339245	-2.78105270	-3.49443945
Y	0.96339852	-2.78105621	1.31711332
C	-0.96338303	-1.66862836	0.00000000
I	0.96338863	-0.55621269	3.49443945

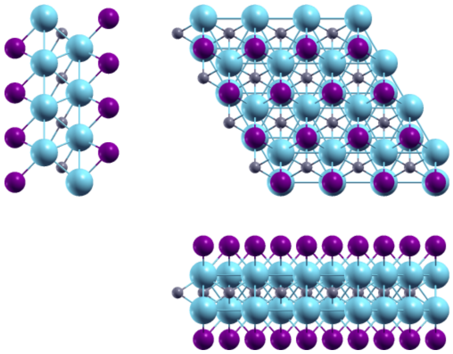

Orthographic projections: views of Y_2CI_2 (P-3m1) as seen from the x axis (left), the y axis (bottom) and the z axis (center).

Lattice matching - minimal size

Formula	N° atoms	strain	cell size 1	cell size 2
Tl	6	0.4255	1	1
InSe	7	0.4511	1	1
HgO	7	0.1122	1	1
AsSb	7	0.0012	1	1
Bi_2	7	0.4655	1	1
GeTe	7	0.0049	1	1
S_2	7	0.0065	1	1
PbTe	7	0.4555	1	1
CaCl	7	0.1425	1	1
$IrTe_2$	8	0.0058	1	1
$CdCl_2$	8	0.0038	1	1
Cl_2Mn	8	0.2474	1	1
CdI_2	8	0.4597	1	1
$AgTe_2$	8	0.4322	1	1
$ReSe_2$	8	0.2653	1	1
S_2Ta	8	0.2501	1	1
Br_2Ca	8	0.4628	1	1
$InSe_2$	8	0.0042	1	1
$GeTe_2$	8	0.0028	1	1
$SiTe_2$	8	0.0072	1	1
I_2Mn	8	0.0039	1	1
NSr_2	8	0.0004	1	1
PbS_2	8	0.0038	1	1
BiClTe	8	0.4606	1	1
LiO_2	8	0.0655	1	1
FeI_2	8	0.0017	1	1
I_2Ni	8	0.003	1	1
S_2Ti	8	0.2568	1	1
NbS_2	8	0.2495	1	1
CrI_2	8	0.0013	1	1
BiBrTe	8	0.4746	1	1
Bi_2Pd	8	0.1161	1	1
Cl_2Ni	8	0.2666	1	1
Cl_2Co	8	0.2563	1	1
$CrTe_2$	8	0.2755	1	1
Br_2V	8	0.2641	1	1
ClNZr	8	0.2612	1	1
Cl_2Fe	8	0.2553	1	1
Br_2Ti	8	0.2747	1	1
$AsSe_2$	8	0.2687	1	1
$NiTe_2$	8	0.0078	1	1
I_2V	8	0.0059	1	1
GeI_2	8	0.4561	1	1
Se_2Zr	8	0.0068	1	1
CdO_2	8	0.2561	1	1
BrNZr	8	0.2706	1	1
$NbSe_2$	8	0.2672	1	1
CoI_2	8	0.0008	1	1
GeS_2	8	0.1326	1	1
$MnSe_2$	8	0.1424	1	1

Table showing the first 50 lattice matching combinations with the smallest number of atoms in the supercell.

Lattice matching - minimal strain

Formula	N° atoms	strain	cell size 1	cell size 2
Bi_2SeTe_2	820	0.0	91	73
Cl_2Fe	504	0.0001	57	73
$Cl_2Cr_2O_2$	370	0.0001	32	35
Nd	274	0.0001	39	79
I_2Pr	743	0.0001	100	81
Br_2V	638	0.0001	73	91
Se_2Sn	8	0.0002	1	1
Br_2Ca_3Si	401	0.0002	43	31
Se_2Ta	705	0.0002	81	100
NbS_2	437	0.0002	49	64
BiClTe	743	0.0002	100	81
$Ge_2I_2La_2$	543	0.0002	57	43
Ba_2Pt	255	0.0003	36	25
$NbSe_2$	705	0.0003	81	100
Cl_2Zr	504	0.0003	57	73
$C_2Cl_2Y_2$	638	0.0003	58	58
$Cl_2H_2Zr_2$	557	0.0004	43	57
F_4Sn	565	0.0004	65	48
NSr_2	8	0.0004	1	1
I_2Ti	8	0.0004	1	1
Gd_2GeI_2	905	0.0005	100	81
Cl_2V	233	0.0005	25	36
GdI_2	536	0.0005	73	57
Cu_2Sr_2	661	0.0005	81	64
CdO_2	504	0.0005	57	73
Ag_2	230	0.0005	36	25
$LiOS_2Ti$	500	0.0005	43	57
BiBrTe	597	0.0005	81	64
Bi_2	601	0.0006	91	73
CdI_2	743	0.0006	100	81
FHOZn	269	0.0006	25	36
NbS_2	386	0.0006	43	57
Cl_2Co	504	0.0006	57	73
NS_2Ta	429	0.0006	37	61
Br_2Ca	743	0.0007	100	81
S_2Ta	437	0.0007	49	64
$ReSe_2$	638	0.0007	73	91
Cl_2Ni	705	0.0007	81	100
CrO_2	272	0.0007	25	49
$Br_2Cr_2O_2$	534	0.0007	48	49
$CrSe_2$	233	0.0007	25	36
$LiNbS_2$	501	0.0007	49	64
$AsSe_2$	705	0.0007	81	100
CoI_2	8	0.0008	1	1
CaI_2	353	0.0008	49	36
Br_2La_2P	905	0.0009	100	81
Cl_2Zr_2	729	0.0009	73	91
Cl_2NSc_2	565	0.0009	49	64
$C_2I_2La_2$	196	0.001	20	16
$Ga_2Gd_2I_2$	893	0.001	91	73

Table showing the first 50 lattice matching combinations with the smallest strain needed to build the supercell.

Y_2GeI_2 (P-3m1)

Structural and electronic properties

**Formula**	Y_2GeI_2
**Spacegroup**	P-3m1
**Prototype**	Bi2Te2S
**Parent 3D**	GeI_2Y_2
**Source DB**	MPDS
**DB ID**	S1923101
**DF2-C09 Binding energy [meV/ Å^2]**	12.7
**RVV10 Binding energy [meV/ Å^2]**	N/A
**Band gap (PBE) [eV]**	0.2

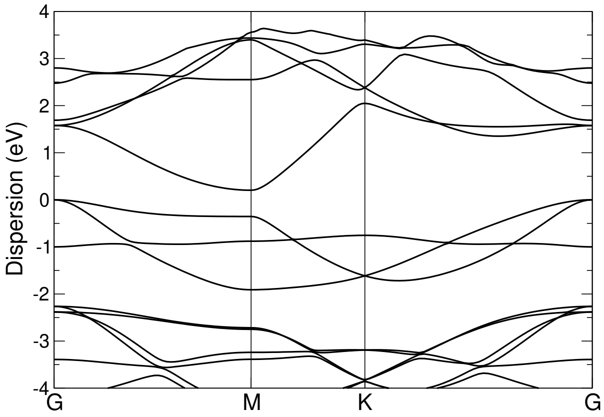

Band structure: Electronic band structure of Y_2GeI_2 (P-3m1) in a representative window around the Fermi energy and along a high-symmetry path.

Optimized crystal structure

Structural parameters: relaxed cell (top) and atomic positions (bottom) of Y_2GeI_2 (P-3m1) in Cartesian coordinates.

		x [Å]	y [Å]	z [Å]
$\mathbf{a_1}$		2.11850219	−3.66935343	0.00000000
$\mathbf{a_2}$		2.11850315	3.66935399	0.00000000
$\mathbf{a_3}$		0.00000000	0.00000000	26.16501185
		x [Å]	y [Å]	z [Å]
●	Y	3.17774750	-0.61156225	1.55987706
●	I	1.05924524	0.61155552	3.56395835
●	Y	1.05925785	0.61156280	-1.55987706
●	I	3.17776010	-0.61155497	-3.56395835
●	Ge	1.05925110	-1.83467672	0.00000000

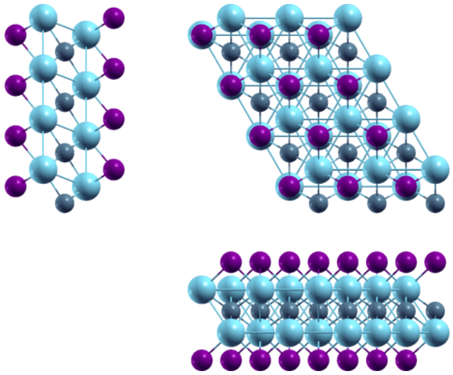

Orthographic projections: views of Y_2GeI_2 (P-3m1) as seen from the x axis (left), the y axis (bottom) and the z axis (center).

Lattice matching - minimal size

Formula	N° atoms	strain	cell size 1	cell size 2
InSe	7	0.0009	1	1
AsSb	7	0.2731	1	1
Bi_2	7	0.0068	1	1
AgTl	7	0.1534	1	1
PbTe	7	0.0027	1	1
CaCl	7	0.1138	1	1
I_2Mg	8	0.0092	1	1
CdI_2	8	0.0044	1	1
Br_2Zn	8	0.2585	1	1
Br_2Ca	8	0.0057	1	1
$AsSn_2$	8	0.2492	1	1
$SiTe_2$	8	0.2642	1	1
I_2Pr	8	0.1372	1	1
$CuTe_2$	8	1.5424	1	1
Br_2La	8	0.0089	1	1
NSr_2	8	0.2755	1	1
Ca_2Si	8	0.4521	1	1
PbS_2	8	0.2693	1	1
BiClTe	8	0.0048	1	1
BrCdI	8	0.0062	1	1
Cl_2Zn	8	0.1207	1	1
Te_2Ti	8	0.2589	1	1
BaF_2	8	0.0029	1	1
AsKSn	8	0.0043	1	1
$PbTe_2$	8	0.0071	1	1
I_2Nd	8	0.138	1	1
$NiTe_2$	8	0.2634	1	1
Cl_2Cu	8	0.0673	1	1
I_2V	8	0.2661	1	1
GeI_2	8	0.0029	1	1
Se_2Zr	8	0.2648	1	1
STl_2	8	0.002	1	1
$PtSe_2$	8	0.25	1	1
CoI_2	8	0.2737	1	1
GeS_2	8	0.4349	1	1
$TaTe_2$	8	0.2484	1	1
$MnSe_2$	8	0.1137	1	1
CeI_2	8	0.1366	1	1
I_2Ti	8	0.2742	1	1
F_2Ni	8	0.1186	1	1
I_2La	8	0.142	1	1
F_2Na	8	0.2612	1	1
CdI_2	8	0.004	1	1
Se_2Sn	8	0.2751	1	1
F_2Zn	8	0.1341	1	1
I_2Pr	8	0.0049	1	1
$HfSe_2$	8	0.2589	1	1
Bi_2Te_2	9	0.4842	1	1
Fe_2Te_2	9	0.1277	1	1
Ca_2Cl_2	9	0.128	1	1

Table showing the first 50 lattice matching combinations with the smallest number of atoms in the supercell.

Lattice matching - minimal strain

Formula	N° atoms	strain	cell size 1	cell size 2
ClH_3O	725	0.0	81	64
$AsSn_2$	437	0.0001	49	64
$CoTe_2$	327	0.0001	36	49
$SiTe_2$	638	0.0001	73	91
NiO_2	47	0.0001	4	9
FeH_2O_2	370	0.0001	25	49
$Cl_2Er_2O_2$	911	0.0002	73	91
Ga_2S_2	376	0.0002	36	49
As_2Li_2Nd	10	0.0002	1	1
Ga_2Se_2	577	0.0002	57	73
Br_3Cs	420	0.0003	64	25
HfS_2	327	0.0003	36	49
H_2MgO_2	125	0.0003	9	16
$HfSe_2$	563	0.0003	64	81
Te_2Ti	563	0.0003	64	81
Br_2Zr_2	269	0.0003	25	36
PSn_2	386	0.0003	43	57
Se_2Zr	638	0.0004	73	91
Ho_2S_2	355	0.0004	39	40
Bi_2Te_2	593	0.0004	73	57
$Br_2O_2Ti_2$	470	0.0004	40	45
HN_3OZn	731	0.0004	49	81
Bi_2Se_2Te	10	0.0005	1	1
Mg_2	541	0.0005	65	108
Sn_2Te_2	599	0.0005	75	56
Ga_2Se_2	805	0.0006	81	100
Br_2Pr_2	644	0.0006	64	81
$PtSe_2$	437	0.0006	49	64
Br_2Zn	563	0.0006	64	81
S_2V	93	0.0006	9	16
CoO_2	47	0.0006	4	9
Cl_2Hf_2	180	0.0006	16	25
$CuTe_2$	327	0.0006	36	49
$TaTe_2$	437	0.0006	49	64
$NiTe_2$	638	0.0007	73	91
Cu_2O_2	355	0.0007	39	40
$Al_2Cl_2O_2$	39	0.0007	3	4
CCl_2Gd_2	725	0.0007	64	81
Se_2Ti	233	0.0007	25	36
$HNiO_2$	511	0.0008	39	79
$I_2N_2Zr_2$	723	0.0008	57	73
Cu_2F_4	541	0.0008	65	36
$Ba_2H_2I_2$	613	0.0008	65	48
Sb_2SeTe_2	10	0.0008	1	1
$I_2La_2Si_2$	11	0.0008	1	1
S_2Sn	386	0.0008	43	57
As_2Li_2Pr	10	0.0009	1	1
In_2Te_3	10	0.0009	1	1
InSe	7	0.0009	1	1
$F_2Se_2Y_2$	614	0.0009	64	49

Table showing the first 50 lattice matching combinations with the smallest strain needed to build the supercell.

Y_2I_2 (P-3m1)

Structural and electronic properties

**Formula**	Y_2I_2
**Spacegroup**	P-3m1
**Prototype**	PtTe
**Parent 3D**	I_2Y_2
**Source DB**	ICSD
**DB ID**	151974
**DF2-C09 Binding energy [meV/ Å^2]**	15.48
**RVV10 Binding energy [meV/ Å^2]**	N/A
**Band gap (PBE) [eV]**	0.0

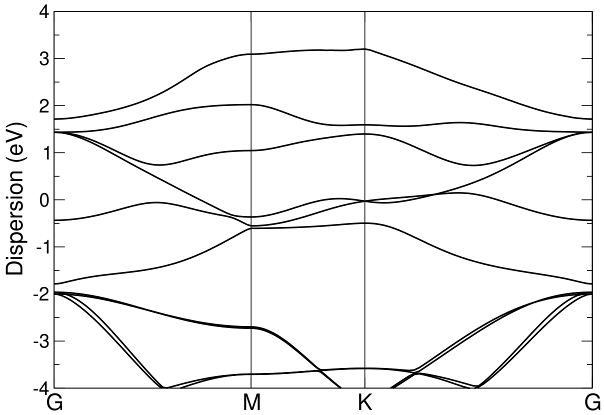

Band structure: Electronic band structure of Y_2I_2 (P-3m1) in a representative window around the Fermi energy and along a high-symmetry path.

Optimized crystal structure

Structural parameters: relaxed cell (top) and atomic positions (bottom) of Y_2I_2 (P-3m1) in Cartesian coordinates.

	x [Å]	y [Å]	z [Å]
$\mathbf{a_1}$	1.96755566	−3.40790638	0.00000000
$\mathbf{a_2}$	1.96755566	3.40790638	0.00000000
$\mathbf{a_3}$	0.00000000	0.00000000	26.17286327

	x [Å]	y [Å]	z [Å]
Y	0.98377783	2.83992198	1.42362934
Y	0.98377783	0.56798440	-1.42362934
I	0.98377783	0.56798440	3.56454023
I	0.98377783	2.83992198	-3.56454023

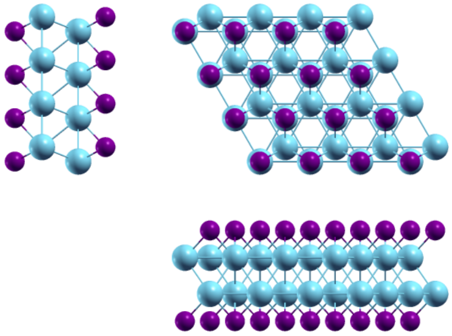

Orthographic projections: views of Y_2I_2 (P-3m1) as seen from the x axis (left), the y axis (bottom) and the z axis (center).

Lattice matching - minimal size

Formula	N° atoms	strain	cell size 1	cell size 2
Na	5	0.2689	1	1
HgO	6	0.4303	1	1
GeTe	6	0.005	1	1
S_2	6	0.0034	1	1
CaCl	6	0.1343	1	1
$IrTe_2$	7	0.0041	1	1
$CdCl_2$	7	0.006	1	1
$MoTe_2$	7	0.2722	1	1
Ba_2Pt	7	3.0462	1	1
CaI_2	7	0.4928	1	1
$InSe_2$	7	0.0057	1	1
$GeTe_2$	7	0.007	1	1
$HfTe_2$	7	0.0042	1	1
Te_2V	7	0.275	1	1
I_2Mn	7	0.0059	1	1
I_2Yb	7	0.4861	1	1
LiO_2	7	0.0695	1	1
Cl_2Zn	7	0.1466	1	1
FeI_2	7	0.008	1	1
I_2Ni	7	0.0068	1	1
CrI_2	7	0.0084	1	1
Te_2Zn	7	0.2719	1	1
BiBrTe	7	0.4499	1	1
Bi_2Pd	7	0.1121	1	1
$CrTe_2$	7	0.2612	1	1
PtS_2	7	0.2704	1	1
Br_2V	7	0.2506	1	1
ClNZr	7	0.2478	1	1
CdClO	7	0.2743	1	1
Ba_2N	7	0.0058	1	1
Se_2Ti	7	0.2666	1	1
Br_2Ti	7	0.2604	1	1
Te_2Zr	7	0.0047	1	1
Te_2W	7	0.2724	1	1
$AsSe_2$	7	0.2548	1	1
OTl_2	7	0.2746	1	1
BiITe	7	0.4661	1	1
CdO_2	7	1.5831	1	1
BrNZr	7	0.2566	1	1
$NbSe_2$	7	0.2534	1	1
GeS_2	7	0.1257	1	1
$MnSe_2$	7	0.1343	1	1
Br_2Cr	7	0.2608	1	1
DyI_2	7	0.5003	1	1
Se_2Ta	7	0.2535	1	1
Br_2Mg	7	0.0082	1	1
$NbSe_2$	7	0.2552	1	1
GdI_2	7	0.4569	1	1
F_2Ni	7	0.143	1	1
Se_2Ta	7	0.2593	1	1

Table showing the first 50 lattice matching combinations with the smallest number of atoms in the supercell.

Lattice matching - minimal strain

Formula	N° atoms	strain	cell size 1	cell size 2
Se_2Ta	499	0.0	64	81
NbS_2	291	0.0	36	49
Li_2Tl_2	392	0.0001	61	37
CCl_2Sc_2	516	0.0002	49	64
Cl_2Zr_2	452	0.0002	49	64
$NbSe_2$	447	0.0002	57	73
CNb_2S_2	280	0.0002	25	36
CrS_2	331	0.0002	37	61
$Cl_2H_2Sc_2$	742	0.0003	64	81
O_2Pt	139	0.0003	16	25
BiITe	583	0.0003	91	73
Bi_2Te_3	729	0.0004	91	73
Br_2Hf_2	520	0.0004	57	73
$AsSe_2$	447	0.0005	57	73
S_2Ta	291	0.0005	36	49
Cl_2Tb_2	8	0.0005	1	1
$Br_2H_2Zr_2$	580	0.0006	49	64
MnO_2	393	0.0006	39	79
$LiNbS_2$	340	0.0006	36	49
$LiMnSe_2$	8	0.0006	1	1
Se_2Ti	624	0.0007	81	100
$Gd_2I_2S_2$	486	0.0007	57	43
P_2	172	0.0007	25	36
$F_2Se_2Tm_2$	10	0.0007	1	1
C_4Ca_2	806	0.0007	92	73
S_2Ti	343	0.0008	43	57
$Ge_2I_2La_2$	708	0.0008	81	64
Br_2Ti	499	0.0008	64	81
$AlLiTe_2$	724	0.0008	100	81
BrNZr	447	0.0008	57	73
Cl_2Ti	208	0.0009	25	36
Cd_2I_3	644	0.0009	81	64
$Er_2I_2S_2$	956	0.0009	125	76
Na	424	0.0009	81	100
AsI_2La_2	644	0.0009	81	64
Ba_2Cu_2	520	0.0009	73	57
$C_2Cl_2Y_2$	788	0.0009	77	80
CS_2Ta_2	280	0.001	25	36
FeO_2	247	0.001	25	49
Br_2Ca_3Si	486	0.001	57	43
$Br_2H_2Zr_2$	742	0.001	64	81
Mo_2Te_4	780	0.001	102	62
Br_2V	388	0.001	49	64
KNO_3	145	0.001	25	9
CaI_2	403	0.001	64	49
CrO_2	496	0.0011	49	100
Br_2Cr	499	0.0011	64	81
Br_2Zr_2	724	0.0011	81	100
Cl_2NSc_2	389	0.0011	36	49
ClNZr	388	0.0011	49	64

Table showing the first 50 lattice matching combinations with the smallest strain needed to build the supercell.

$Y_2O_2Br_2$ (P4/nmm)

Structural and electronic properties

**Formula**	$Y_2O_2Br_2$
**Spacegroup**	P4/nmm
**Prototype**	PbClF
**Parent 3D**	$Br_2O_2Y_2$
**Source DB**	MPDS
**DB ID**	S1711909
**DF2-C09 Binding energy [meV/ Å^2]**	14.8
**RVV10 Binding energy [meV/ Å^2]**	N/A
**Band gap (PBE) [eV]**	4.68

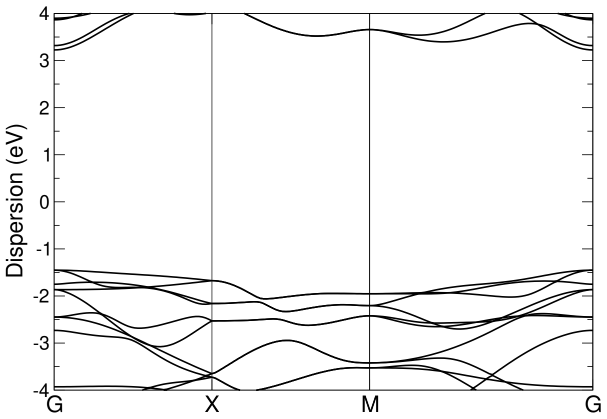

Band structure: Electronic band structure of $Y_2O_2Br_2$ (P4/nmm) in a representative window around the Fermi energy and along a high-symmetry path.

Optimized crystal structure

Structural parameters: relaxed cell (top) and atomic positions (bottom) of $Y_2O_2Br_2$ (P4/nmm) in Cartesian coordinates.

	x [Å]	y [Å]	z [Å]
$\mathbf{a_1}$	3.84665986	0.00000000	0.00000000
$\mathbf{a_2}$	0.00000000	3.84665986	0.00000000
$\mathbf{a_3}$	0.00000000	0.00000000	22.95233220

		x [Å]	y [Å]	z [Å]
●	Y	0.96166497	-0.96166497	1.13126210
●	Br	-0.96166497	-2.88499490	2.82002972
●	Y	-0.96166497	-2.88499490	-1.13126210
●	Br	0.96166497	-0.96166497	-2.82002972
•	O	-0.96166497	-0.96166497	0.00000000
•	O	0.96166497	-2.88499490	0.00000000

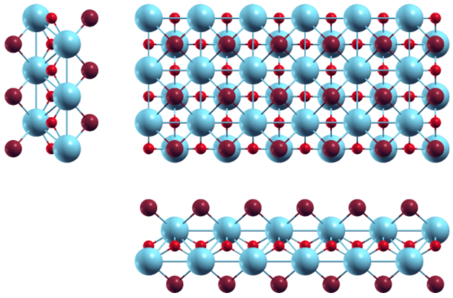

Orthographic projections: views of $Y_2O_2Br_2$ (P4/nmm) as seen from the x axis (left), the y axis (bottom) and the z axis (center).

Lattice matching - minimal size

Formula	N° atoms	strain	cell size 1	cell size 2
K	7	0.3927	1	1
K	7	0.1934	1	1
In	7	0.1104	1	1
InSe	8	0.1524	1	1
Bi_2	8	0.1583	1	1
PbTe	8	0.1542	1	1
Sb_2	8	0.138	1	1
I_2Mg	9	0.1429	1	1
S_2V	9	0.1097	1	1
MoS_2	9	0.1096	1	1
CdI_2	9	0.1559	1	1
Nd	9	0.7838	1	3
Br_2Ca	9	0.1572	1	1
CaI_2	9	0.1816	1	1
I_2Pr	9	0.0073	1	1
Br_2La	9	0.1432	1	1
Br_2Cu	9	0.109	1	1
Ca_2Si	9	0.777	1	1
I_2Yb	9	0.1785	1	1
BiClTe	9	0.1563	1	1
$AuTe_2$	9	0.1319	1	1
BrCdI	9	0.1457	1	1
$PdTe_2$	9	0.1301	1	1
HgI_2	9	1.1565	1	1
I_2Zn	9	0.1354	1	1
BaF_2	9	0.1487	1	1
BiBrTe	9	0.1621	1	1
S_2W	9	0.1096	1	1
GeI_2	9	0.1412	1	1
AsKSn	9	0.1474	1	1
$PbTe_2$	9	0.1448	1	1
I_2Nd	9	0.0083	1	1
Cl_2Cu	9	0.1003	1	1
I_2Tm	9	0.1801	1	1
$SnTe_2$	9	0.1393	1	1
Cl_2V	9	0.1088	1	1
GeI_2	9	0.1544	1	1
I_2Pb	9	0.7667	1	1
STl_2	9	0.1496	1	1
BiITe	9	0.1693	1	1
DyI_2	9	0.1851	1	1
Br_2Ni	9	0.1085	1	1
CeI_2	9	0.0066	1	1
Se_2Yb	9	0.1415	1	1
MoS_2	9	0.1095	1	1
Cl_2Mg	9	0.1085	1	1
$BiTe_2$	9	0.1417	1	1
GdI_2	9	0.1652	1	1
$CrSe_2$	9	0.109	1	1
$PtTe_2$	9	0.1316	1	1

Table showing the first 50 lattice matching combinations with the smallest number of atoms in the supercell.

Lattice matching - minimal strain

Formula	N° atoms	strain	cell size 1	cell size 2
I_3Sn	70	0.0	9	4
$Br_2Dy_2O_2$	12	0.0	1	1
$As_2Cd_2K_2$	876	0.0	85	61
Ag_2I_2	438	0.0001	49	36
Cu_2S_2	10	0.0001	1	1
Br_2CsF	316	0.0001	36	25
$Cl_2S_2Tl_2$	930	0.0001	97	58
NS_2Zr	548	0.0002	48	65
Ga_2S_3	613	0.0002	48	65
Pb_2Se_2	806	0.0002	89	68
K	557	0.0003	82	65
Cu_2O_2	496	0.0004	50	49
La_2S_2	818	0.0004	89	71
F_2Tl_2	10	0.0004	1	1
Mg_6	366	0.0005	25	36
Na	353	0.0005	48	65
P_2Rh_2	10	0.0006	1	1
PtS_2	483	0.0006	48	65
Ho_2S_2	496	0.0006	50	49
$As_2Mg_2Na_2$	882	0.0006	82	65
O_2Sn_2	10	0.0007	1	1
H_4Ti	221	0.0008	16	25
P_4	356	0.0009	36	35
O_4PSn	876	0.001	85	61
K	550	0.0011	81	64
In	445	0.0011	58	97
HgI_2	402	0.0011	49	36
F_4Pb	629	0.0011	64	49
Se_2Sn_2	742	0.0012	81	64
Se_2Sn_2	732	0.0013	80	63
$HfLiS_2$	548	0.0013	48	65
Bi_2Se_2	912	0.0013	98	81
Sn	655	0.0014	85	145
$C_2I_2La_2$	138	0.0014	12	11
$As_2Mg_2Na_2$	870	0.0014	81	64
H_2Li_2Pd	221	0.0014	16	25
C_4Ca_2	774	0.0014	69	60
Se_2Ta_4	690	0.0015	50	65
AuI_4Li	792	0.0015	90	42
$Br_2O_2Tb_2$	12	0.0015	1	1
$Cu_2Rb_2Te_2$	366	0.0016	36	25
ClNZr	840	0.0016	81	118
Te_2Zn	483	0.0016	48	65
$AsSn_2$	603	0.0016	62	77
C_2Li_2	246	0.0016	25	24
$PtSe_2$	603	0.0016	62	77
In	375	0.0016	49	81
$Br_2Cr_2S_2$	996	0.0016	84	82
BrNZr	531	0.0017	52	73
Au_2I_2	682	0.0017	75	58

Table showing the first 50 lattice matching combinations with the smallest strain needed to build the supercell.

$Y_2O_2I_2$ (P4/nmm)

Structural and electronic properties

**Formula**	$Y_2O_2I_2$
**Spacegroup**	P4/nmm
**Prototype**	PbClF
**Parent 3D**	$I_2O_2Y_2$
**Source DB**	MPDS
**DB ID**	S1711910
**DF2-C09 Binding energy [meV/ Å^2]**	14.16
**RVV10 Binding energy [meV/ Å^2]**	N/A
**Band gap (PBE) [eV]**	3.48

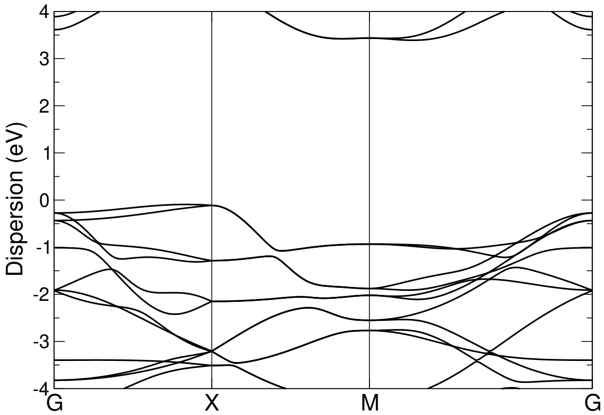

Band structure: Electronic band structure of $Y_2O_2I_2$ (P4/nmm) in a representative window around the Fermi energy and along a high-symmetry path.

Optimized crystal structure

Structural parameters: relaxed cell (top) and atomic positions (bottom) of $Y_2O_2I_2$ (P4/nmm) in Cartesian coordinates.

	x [Å]	y [Å]	z [Å]
$\mathbf{a_1}$	3.93990785	0.00000000	0.00000000
$\mathbf{a_2}$	0.00000000	3.93990785	0.00000000
$\mathbf{a_3}$	0.00000000	0.00000000	23.90821252

	x [Å]	y [Å]	z [Å]
Y	0.98497696	-0.98497696	1.08986957
I	-0.98497696	-2.95493089	3.02357466
Y	-0.98497696	-2.95493089	-1.08986957
I	0.98497696	-0.98497696	-3.02357466
O	-0.98497696	-0.98497696	0.00000000
O	0.98497696	-2.95493089	0.00000000

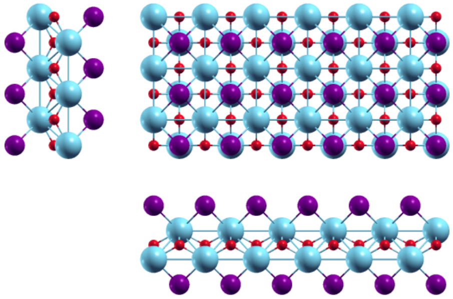

Orthographic projections: views of $Y_2O_2I_2$ (P4/nmm) as seen from the x axis (left), the y axis (bottom) and the z axis (center).

Lattice matching - minimal size

Formula	N° atoms	strain	cell size 1	cell size 2
K	7	0.179	1	1
InSe	8	0.142	1	1
Bi_2	8	0.1472	1	1
AgTl	8	0.0189	1	1
Ag_2	8	0.1858	1	1
LiO	8	0.1106	1	1
P_2	8	0.109	1	1
PbTe	8	0.1435	1	1
Sb_2	8	0.1294	1	1
I_2Mg	9	0.1336	1	1
CdI_2	9	0.1451	1	1
Nd	9	0.7407	1	3
Ba_2Pt	9	0.1855	1	1
Br_2Ca	9	0.1462	1	1
CaI_2	9	0.1682	1	1
I_2Pr	9	0.004	1	1
Br_2La	9	0.1338	1	1
Br_2Cu	9	0.1038	1	1
Ca_2Si	9	0.1913	1	1
I_2Yb	9	0.1654	1	1
BiClTe	9	0.1454	1	1
Cl_2Ti	9	0.109	1	1
BrCdI	9	0.136	1	1
HgI_2	9	0.4057	1	1
BaF_2	9	0.1387	1	1
BiBrTe	9	0.1506	1	1
$RhTe_2$	9	0.1084	1	1
GeI_2	9	0.1322	1	1
AsKSn	9	0.1375	1	1
$PbTe_2$	9	0.1352	1	1
I_2Nd	9	0.0031	1	1
Cl_2Cu	9	0.0978	1	1
I_2Tm	9	0.1669	1	1
$SnTe_2$	9	0.1305	1	1
Cl_2V	9	0.1112	1	1
GeI_2	9	0.1437	1	1
I_2Pb	9	0.1878	1	1
STl_2	9	0.1395	1	1
BiITe	9	0.1571	1	1
GeS_2	9	0.2138	1	1
DyI_2	9	0.1714	1	1
CeI_2	9	0.0048	1	1
Se_2Yb	9	0.1324	1	1
$BiTe_2$	9	0.1326	1	1
GdI_2	9	0.1534	1	1
$CrSe_2$	9	0.1115	1	1
I_2La	9	0.0016	1	1
$CrSe_2$	9	0.1109	1	1
CdI_2	9	0.1447	1	1
F_2Zn	9	0.0079	1	1

Table showing the first 50 lattice matching combinations with the smallest number of atoms in the supercell.

Lattice matching - minimal strain

Formula	N° atoms	strain	cell size 1	cell size 2
$Ba_2H_2I_2$	510	0.0	49	36
H_2Na_2Pd	789	0.0001	64	81
GeS_2	636	0.0001	65	82
Sn	70	0.0002	9	16
$Br_2Nd_2O_2$	12	0.0002	1	1
Ga_2S_2	548	0.0004	48	65
Ag_2I_2	590	0.0005	65	50
$AgTe_2$	942	0.0006	89	136
H_2Li_2Pd	699	0.0006	49	81
Cl_2Sc_2	958	0.0006	81	118
$CrTe_2$	840	0.0006	81	118
Ga_2S_2	548	0.0007	48	65
Br_2Cr	840	0.0007	81	118
FeH_2O_2	315	0.0007	20	39
PTe_2Ti_2	613	0.0008	48	65
Cl_4KTl	204	0.0008	25	9
$CoTe_2$	483	0.0008	48	65
Br_2Ti	840	0.0008	81	118
Br_2Ni	483	0.0009	48	65
$Bi_2Br_2O_2$	12	0.0009	1	1
GeS_2	627	0.0009	64	81
Cl_2Mg	483	0.0009	48	65
Se_2Ta_4	510	0.001	36	49
$H_2I_2Yb_2$	12	0.001	1	1
As_2Fe_2	718	0.001	65	82
Br_2Cu	969	0.001	103	117
Br_2Cu	894	0.0011	95	108
HfS_2	483	0.0011	48	65
H_4Ti	699	0.0011	49	81
Ho_2S_2	494	0.0012	49	50
$Br_2N_2Ti_2$	78	0.0012	6	7
AlH_4Na	366	0.0012	36	25
$Cl_2H_2Lu_2$	678	0.0012	48	65
Br_2Cu	819	0.0013	87	99
Te_4W_2	654	0.0013	64	45
$Er_2I_2O_2$	12	0.0013	1	1
Mg_2	456	0.0013	49	81
Br_2Cu	762	0.0013	81	92
$Br_2Eu_2F_2$	12	0.0013	1	1
Mg_4	196	0.0014	16	25
Ag_2F_4	108	0.0014	12	6
Se_2Ta	840	0.0014	81	118
Br_2Cu	687	0.0014	73	83
$CuTe_2$	483	0.0015	48	65
$Au_2K_2S_2$	78	0.0015	10	3
$Hf_2Se_2Si_2$	882	0.0015	65	82
Cu_2O_2	494	0.0015	49	50
$Cl_2N_2Ti_2$	66	0.0015	5	6
O_2Zn	237	0.0015	20	39
I_2La_2Te	919	0.0016	89	77

Table showing the first 50 lattice matching combinations with the smallest strain needed to build the supercell.

Y_2PBr_2 (P-3m1)

Structural and electronic properties

**Formula**	Y_2PBr_2
**Spacegroup**	P-3m1
**Prototype**	Bi2Te2S
**Parent 3D**	Br_2PY_2
**Source DB**	MPDS
**DB ID**	S1713666
**DF2-C09 Binding energy [meV/ Å^2]**	11.81
**RVV10 Binding energy [meV/ Å^2]**	N/A
**Band gap (PBE) [eV]**	0.0

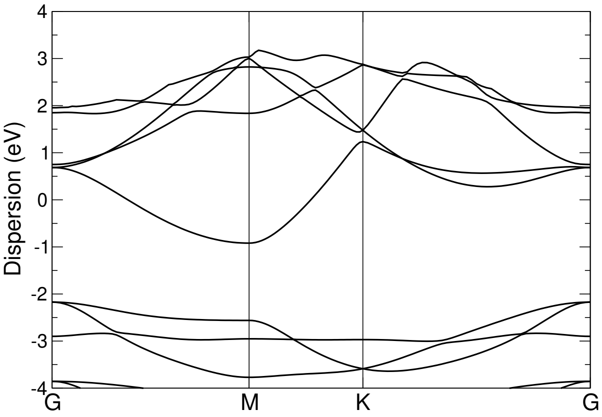

Band structure: Electronic band structure of Y_2PBr_2 (P-3m1) in a representative window around the Fermi energy and along a high-symmetry path.

Optimized crystal structure

Structural parameters: relaxed cell (top) and atomic positions (bottom) of Y_2PBr_2 (P-3m1) in Cartesian coordinates.

	x [Å]	y [Å]	z [Å]
$\mathbf{a_1}$	2.00292375	−3.46916570	0.00000000
$\mathbf{a_2}$	2.00292375	3.46916570	0.00000000
$\mathbf{a_3}$	0.00000000	0.00000000	25.30804692

	x [Å]	y [Å]	z [Å]
Y	-1.00146187	0.57819428	-1.51709929
Br	1.00146187	-0.57819428	-3.34027703
Y	1.00146187	-0.57819428	1.51709929
Br	-1.00146187	0.57819428	3.34027703
P	1.00146187	1.73458285	0.00000000

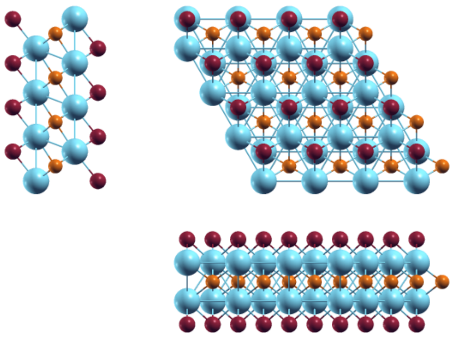

Orthographic projections: views of Y_2PBr_2 (P-3m1) as seen from the x axis (left), the y axis (bottom) and the z axis (center).

Lattice matching - minimal size

Formula	N° atoms	strain	cell size 1	cell size 2
K	6	0.4948	1	1
Na	6	0.257	1	1
Gd	6	0.239	1	1
As_2	7	0.2682	1	1
CaCl	7	0.1282	1	1
Cl_2Zn	8	0.2667	1	1
$MoTe_2$	8	0.2602	1	1
Ba_2Pt	8	2.9341	1	1
HfS_2	8	0.2722	1	1
CaI_2	8	0.471	1	1
$HfTe_2$	8	0.0042	1	1
Te_2V	8	0.2629	1	1
$CuTe_2$	8	0.2717	1	1
I_2Yb	8	0.4646	1	1
Br_2Co	8	0.2676	1	1
Ca_2N	8	0.269	1	1
$AuTe_2$	8	0.0036	1	1
Cl_2Zn	8	0.1392	1	1
$PdTe_2$	8	0.0011	1	1
I_2Zn	8	0.0083	1	1
Te_2Zn	8	0.2599	1	1
Bi_2Pd	8	0.433	1	1
Br_2Mn	8	0.265	1	1
$CrTe_2$	8	0.2498	1	1
PtS_2	8	0.2584	1	1
$CoTe_2$	8	0.2727	1	1
CdClO	8	0.2622	1	1
Ba_2N	8	0.0027	1	1
Se_2Ti	8	0.2548	1	1
Br_2Ti	8	0.249	1	1
Te_2Zr	8	0.0037	1	1
Te_2W	8	0.2604	1	1
I_2Tm	8	0.468	1	1
OTl_2	8	0.2624	1	1
CdO_2	8	1.5225	1	1
Br_2Fe	8	0.2677	1	1
GeS_2	8	0.1207	1	1
$MnSe_2$	8	0.1281	1	1
Br_2Cr	8	0.2494	1	1
DyI_2	8	0.4782	1	1
Br_2Ni	8	0.2752	1	1
CuO_2	8	0.0902	1	1
Cl_2Mg	8	0.2752	1	1
F_2Ni	8	0.1359	1	1
Se_2Ta	8	0.248	1	1
$PtTe_2$	8	0.0032	1	1
Br_2Cd	8	0.0002	1	1
NaPSn	8	0.005	1	1
Fe_2Te_2	9	0.1493	1	1
Ca_2Cl_2	9	0.1497	1	1

Table showing the first 50 lattice matching combinations with the smallest number of atoms in the supercell.

Lattice matching - minimal strain

Formula	N° atoms	strain	cell size 1	cell size 2
$Br_2H_2Zr_2$	629	0.0	49	64
Br_2Co	705	0.0	81	100
Br_2Fe	705	0.0001	81	100
Br_2Cr	437	0.0001	49	64
N_2Re	272	0.0001	25	49
I_2La_2Sb	565	0.0001	64	49
Br_2Hf_2	443	0.0001	43	57
NS_2Ta	109	0.0001	9	16
$HfLiS_2$	644	0.0002	64	81
GeI_2La_2	820	0.0002	91	73
$Dy_2I_2S_2$	789	0.0002	81	64
Br_2Ti	437	0.0002	49	64
Br_2Cd	8	0.0002	1	1
ClNZr	327	0.0002	36	49
$Ce_2I_2S_2$	461	0.0002	49	36
Ca_2Si	353	0.0002	49	36
K	369	0.0002	64	49
S_2Ta	233	0.0002	25	36
$CrSe_2$	155	0.0002	16	25
CCl_2Lu_2	905	0.0003	81	100
Cl_2Sc_2	501	0.0003	49	64
FeO_2	432	0.0003	39	79
NaO_4	650	0.0004	81	49
FHOZn	180	0.0004	16	25
$CrTe_2$	437	0.0004	49	64
$I_2Nd_2S_2$	543	0.0004	57	43
I_2Tm	674	0.0004	91	73
$Gd_2I_2S_2$	707	0.0004	73	57
Te_2Zn	563	0.0004	64	81
As_2	605	0.0004	81	100
Se_2Ti	504	0.0005	57	73
Br_2Mn	638	0.0005	73	91
$NbSe_2$	386	0.0005	43	57
BrNZr	386	0.0006	43	57
$I_2S_2Sm_2$	614	0.0006	64	49
Cl_2Zn	705	0.0006	81	100
PtS_2	563	0.0006	64	81
$MoTe_2$	563	0.0006	64	81
NbS_2	233	0.0006	25	36
C	94	0.0006	9	49
Br_2Ca_3Si	707	0.0007	73	57
$Cl_2H_2Sc_2$	629	0.0007	49	64
$AsSe_2$	386	0.0008	43	57
$Br_2H_2Zr_2$	474	0.0008	36	49
Te_2W	563	0.0008	64	81
Bi_2Te_2	280	0.0008	36	25
$C_2Br_2Tb_2$	638	0.0008	58	58
Br_2Zr_2	577	0.0008	57	73
DyI_2	597	0.0009	81	64
KS_2Ti	729	0.0009	73	91

Table showing the first 50 lattice matching combinations with the smallest strain needed to build the supercell.

$Y_2S_2Br_2$ (Pmm2)

Structural and electronic properties

**Formula**	$Y_2S_2Br_2$
**Spacegroup**	Pmm2
**Prototype**	FeOCl
**Parent 3D**	$Br_2S_2Y_2$
**Source DB**	MPDS
**DB ID**	S307279
**DF2-C09 Binding energy [meV/ Å^2]**	10.11
**RVV10 Binding energy [meV/ Å^2]**	N/A
**Band gap (PBE) [eV]**	2.99

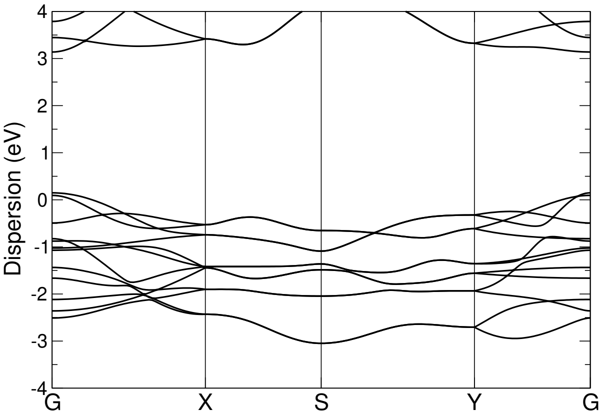

Band structure: Electronic band structure of $Y_2S_2Br_2$ (Pmm2) in a representative window around the Fermi energy and along a high-symmetry path.

Optimized crystal structure

Structural parameters: relaxed cell (top) and atomic positions (bottom) of $Y_2S_2Br_2$ (Pmm2) in Cartesian coordinates.

		x [Å]	y [Å]	z [Å]
$\mathbf{a_1}$		4.04516908	0.00000000	0.00000000
$\mathbf{a_2}$		0.00000000	5.34752894	0.00000000
$\mathbf{a_3}$		0.00000000	0.00000000	24.28606458
		x [Å]	y [Å]	z [Å]
●	Y	1.01138702	-1.33688224	-1.18693331
●	S	1.01118826	-4.01064671	-0.66840539
●	Br	-1.01118234	-1.33688224	-3.15387368
●	Y	-1.01138702	-4.01064671	1.18693331
●	S	-1.01118826	-1.33688224	0.66840539
●	Br	1.01118234	-4.01064671	3.15387368

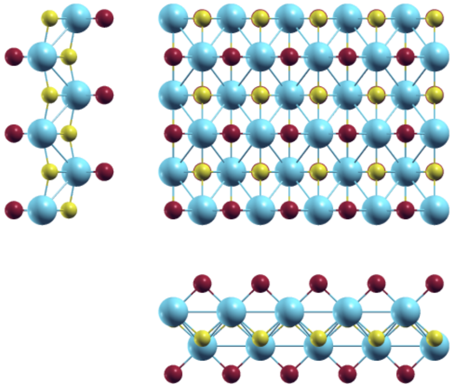

Orthographic projections: views of $Y_2S_2Br_2$ (Pmm2) as seen from the x axis (left), the y axis (bottom) and the z axis (center).

Lattice matching - minimal size

Formula	N° atoms	strain	cell size 1	cell size 2
In	8	0.143	1	2
Gd	8	0.1299	1	2
Br_2Cu	9	0.1188	1	1
CNRb	9	0.3115	1	1
CNNa	9	0.3099	1	1
Ba_2Cd	9	0.2806	1	1
Bi_2In_2	10	0.0666	1	1
Cu_2I_2	10	0.2617	1	1
Cl_2Hf_2	10	0.2806	1	1
O_2Pb_2	10	0.7674	1	1
As_4	10	0.3161	1	1
S_2	10	0.6825	1	2
P_2	10	0.365	1	2
O_2Sn_2	10	0.1674	1	1
Bi_2O_2	10	0.7724	1	1
Br_2CsF	10	0.0658	1	1
FKO_2Se	11	0.5763	1	1
Cl_4Mn	11	0.0691	1	1
$Ba_2H_2I_2$	12	0.0652	1	1
CrS_2	12	0.135	1	2
$Br_2Ho_2S_2$	12	0.0043	1	1
S_2V	12	0.145	1	2
MoS_2	12	0.1454	1	2
$I_2Lu_2Se_2$	12	0.5607	1	1
$Ho_2I_2S_2$	12	0.0127	1	1
Cu_4Te_2	12	0.1191	1	1
$Eu_2F_2I_2$	12	0.7704	1	1
AlH_4Na	12	0.3769	1	1
$F_2I_2Sm_2$	12	0.7667	1	1
ReS_2	12	0.138	1	2
Cl_2Ti	12	0.3653	1	2
$K_2O_2Tl_2$	12	0.5681	1	1
Te_2Zn	12	0.5464	1	2
S_2W	12	0.1455	1	2
$Gd_2I_2S_2$	12	0.0178	1	1
$C_2Br_2Gd_2$	12	0.1086	1	1
$Br_2Er_2Se_2$	12	0.0164	1	1
$I_2S_2Tb_2$	12	0.0157	1	1
$I_2S_2Yb_2$	12	0.0078	1	1
$Cu_2Rb_2Te_2$	12	0.0665	1	1
$Br_2Dy_2S_2$	12	0.0054	1	1
$Cl_2Ga_2Te_2$	12	0.0421	1	1
$Ag_2K_2Te_2$	12	0.3884	1	1
$Br_2Ga_2Te_2$	12	0.547	1	1
Ca_4Cu_2	12	0.5363	1	1
$Br_2Lu_2S_2$	12	0.0081	1	1
$Cu_2Se_2Tl_2$	12	0.2605	1	1
$Br_2S_2Yb_2$	12	0.0041	1	1
$Br_2Er_2S_2$	12	0.0039	1	1
$Br_2H_2Sr_2$	12	0.7817	1	1

Table showing the first 50 lattice matching combinations with the smallest number of atoms in the supercell.

Lattice matching - minimal strain

Formula	N° atoms	strain	cell size 1	cell size 2
Ba_2Pt	228	0.0004	24	28
Ag_2	200	0.0005	24	28
AsI_2La_2	745	0.0007	60	77
C_2	398	0.0007	28	115
Sn_2Te_2	548	0.0007	54	56
Cd_2I_3	745	0.0008	60	77
Bi_2SeTe_2	765	0.0008	60	81
$Cl_2N_2Zr_2$	726	0.0008	49	72
N_4	316	0.0009	20	49
Bi_2	522	0.0009	60	81
FeI_2	594	0.0009	54	90
Br_2Mg	594	0.001	54	90
$I_2Pr_2S_2$	312	0.001	24	28
C_2Li_2	560	0.0011	48	68
I_2Ni	594	0.0011	54	90
$Ga_2Gd_2I_2$	846	0.0012	60	81
CrI_2	594	0.0012	54	90
$Cu_2K_2Te_2$	648	0.0013	53	55
$Ga_2I_2Tb_2$	876	0.0014	62	84
$Ga_2I_2Tb_2$	846	0.0014	60	81
Nd	132	0.0014	14	48
Br_2Hf_2	772	0.0014	54	112
$C_2Br_2Gd_2$	978	0.0015	64	99
Ni_2Te_2	708	0.0015	56	93
Cu_4Te_2	744	0.0015	53	71
Pb_2Se_2	638	0.0015	61	68
Cl_2OOs	958	0.0015	77	124
Sb_2Te_3	765	0.0015	60	81
$CdCl_2$	615	0.0016	56	93
Sb_2Te_3	673	0.0016	53	71
P_4	970	0.0016	83	118
FKO_2Se	817	0.0016	82	65
I_2Mn	615	0.0016	56	93
Ni_2Te_2	684	0.0016	54	90
$Cl_2Er_2H_2$	894	0.0017	56	93
$Br_2In_2O_2$	918	0.0017	63	90
Br_2Ca	603	0.0017	60	81
$Se_2Si_2Zr_2$	228	0.0017	14	24
$InSe_2$	615	0.0018	56	93
Bi_2SeTe_2	700	0.0018	55	74
$C_2Br_2Tb_2$	978	0.0018	64	99
$CdCl_2$	594	0.0018	54	90
S_2Sn_2	44	0.0018	4	5
Br_2Ti	666	0.0018	55	112
I_2Pb	228	0.0019	24	28
ClNZr	681	0.0019	55	117
$I_2Sb_2Te_2$	900	0.0019	89	61
Se_2Ta	666	0.0019	55	112
CrI_2	540	0.0019	49	82
ReS_2	138	0.0019	10	26

Table showing the first 50 lattice matching combinations with the smallest strain needed to build the supercell.

YCBr (C2/m)

Structural and electronic properties

**Formula**	YCBr
**Spacegroup**	C2/m
**Prototype**	Gd2C2Br2
**Parent 3D**	$Y_2C_2Br_2$
**Source DB**	ICSD
**DB ID**	78871
**DF2-C09 Binding energy [meV/ Å^2]**	12.07
**RVV10 Binding energy [meV/ Å^2]**	18.68
**Band gap (PBE) [eV]**	N/A

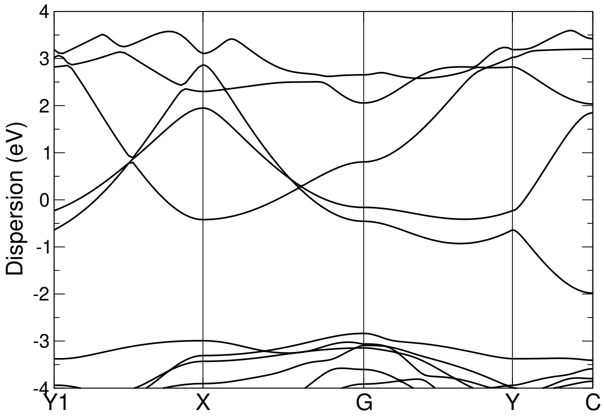

Band structure: Electronic band structure of YCBr (C2/m) in a representative window around the Fermi energy and along a high-symmetry path.

Optimized crystal structure

Structural parameters: relaxed cell (top) and atomic positions (bottom) of YCBr (C2/m) in Cartesian coordinates.

	x [Å]	y [Å]	z [Å]
$\mathbf{a_1}$	3.99429615	0.00698012	0.00000000
$\mathbf{a_2}$	2.18325413	3.34482463	0.00000000
$\mathbf{a_3}$	0.00000000	0.00000000	26.72861340

	x [Å]	y [Å]	z [Å]
Y	2.38023107	1.29146173	14.81328847
C	4.19376084	2.27544364	13.73628252
Br	0.25744693	0.13968512	16.69635471
Y	0.80355495	0.43599150	11.91532462
C	5.16757425	2.80381367	12.99233253
Br	2.92633254	1.58776453	10.03225734

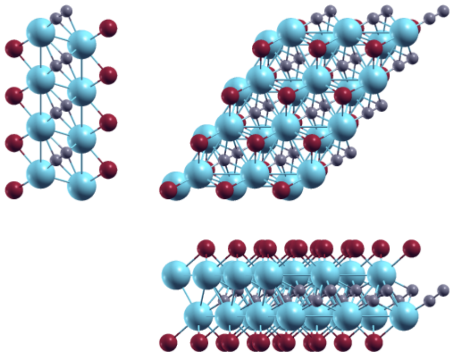

Orthographic projections: views of YCBr (C2/m) as seen from the x axis (left), the y axis (bottom) and the z axis (center).

Lattice matching - minimal size

Formula	N° atoms	strain	cell size 1	cell size 2
Tl	7	0.4414	1	1
Tl	7	0.2197	1	1
Na	7	0.2588	1	1
HgO	8	0.1039	1	1
AsSb	8	0.0176	1	1
GeTe	8	0.0157	1	1
As_2	8	0.2702	1	1
S_2	8	0.0156	1	1
P_2	8	0.2141	1	1
In	8	0.2487	1	2
Sb_2	8	0.0282	1	1
CaCl	8	0.1268	1	1
$IrTe_2$	9	0.0156	1	1
CrS_2	9	0.5499	1	1
Cl_2Zn	9	0.2687	1	1
$CdCl_2$	9	0.0159	1	1
Cl_2Mn	9	0.2263	1	1
$MoTe_2$	9	0.262	1	1
$MoSe_2$	9	0.2189	1	1
$ReSe_2$	9	0.2422	1	1
S_2Ta	9	0.2287	1	1
Br_2Zn	9	0.0237	1	1
CaI_2	9	0.4758	1	1
$InSe_2$	9	0.0159	1	1
$GeTe_2$	9	0.0162	1	1
$SiTe_2$	9	0.0209	1	1
$HfTe_2$	9	0.0171	1	1
Te_2V	9	0.2647	1	1
I_2Mn	9	0.0159	1	1
NSr_2	9	0.0169	1	1
I_2Yb	9	0.4693	1	1
PbS_2	9	0.0189	1	1
Br_2Co	9	0.2696	1	1
Ca_2N	9	0.311	1	1
Cl_2Ti	9	0.2142	1	1
$AuTe_2$	9	0.0217	1	1
LiO_2	9	0.0729	1	1
Cl_2Zn	9	0.1385	1	1
$PdTe_2$	9	0.02	1	1
FeI_2	9	0.0165	1	1
I_2Ni	9	0.0161	1	1
S_2Ti	9	0.2346	1	1
Te_2Ti	9	0.0235	1	1
NbS_2	9	0.2281	1	1
CrI_2	9	0.0167	1	1
I_2Zn	9	0.0253	1	1
Te_2Zn	9	0.2618	1	1
Bi_2Pd	9	0.1064	1	1
Br_2Mn	9	0.2669	1	1
Cl_2Ni	9	0.2434	1	1

Table showing the first 50 lattice matching combinations with the smallest number of atoms in the supercell.

Lattice matching - minimal strain

Formula	N° atoms	strain	cell size 1	cell size 2
$Br_2H_2Zr_2$	732	0.0003	53	69
CCl_2Sc_2	663	0.0003	53	69
I_2Tm	573	0.0003	69	53
$LiNbS_2$	878	0.0005	77	104
GeI_2La_2	679	0.0006	69	53
Cl_2Zr_2	594	0.0006	53	69
$Br_2O_2V_2$	954	0.0007	77	82
$Si_2Te_2Zr_2$	822	0.0007	69	68
F_2Ni	618	0.0007	69	68
ClNZr	525	0.0008	53	69
CrI_2	264	0.0008	29	30
$Br_2N_2Ti_2$	648	0.0008	54	54
$I_2S_2Tl_2$	822	0.0008	69	68
Fe_2Se_2	686	0.0009	69	68
$Br_2Cr_2O_2$	768	0.0009	62	66
Cl_2ORu	864	0.0009	86	87
BiBrTe	990	0.0009	117	96
CaI_2	573	0.001	69	53
$Ge_2Te_2Zr_2$	990	0.001	85	80
Cu_2Se_2	686	0.001	69	68
O_2Pt	543	0.001	51	79
$I_2S_2Tl_2$	714	0.001	60	59
BiBrTe	948	0.001	112	92
Cu_2Se_2	596	0.001	60	59
$Br_2Lu_2O_2$	990	0.001	85	80
F_2Ni	537	0.0011	60	59
Ba_2Hg	954	0.0011	115	88
$Si_2Te_2Zr_2$	714	0.0011	60	59
$Ca_2Cl_2F_2$	858	0.0011	74	69
$As_2Fe_2Li_2$	888	0.0011	76	72
$Br_2H_2Yb_2$	888	0.0011	76	72
Cu_2Se_2	586	0.0011	59	58
$I_2S_2Tl_2$	702	0.0011	59	58
Ba_2Hg	870	0.0012	105	80
Ag_2Te_2	646	0.0012	67	61
Fe_2SeTe	744	0.0012	76	72
F_2Ni	528	0.0012	59	58
$Si_2Te_2Zr_2$	702	0.0012	59	58
In	385	0.0012	51	79
Cu_2Te_2	544	0.0012	54	55
$Cl_2Hg_2N_2$	960	0.0012	110	50
AsSb	824	0.0013	102	106
$Ag_2K_2Se_2$	762	0.0013	76	51
$Ba_2H_2I_2$	666	0.0013	68	43
$Ge_2Te_2Zr_2$	978	0.0013	84	79
$Ge_2Te_2Zr_2$	756	0.0013	65	61
Br_2Cr	732	0.0013	75	94
AsSb	800	0.0013	99	103
CrI_2	255	0.0013	28	29
In	285	0.0013	38	57

Table showing the first 50 lattice matching combinations with the smallest strain needed to build the supercell.

YCI (C2/m)

Structural and electronic properties

**Formula**	YCI
**Spacegroup**	C2/m
**Prototype**	Gd2C2Br2
**Parent 3D**	$Y_2C_2I_2$
**Source DB**	ICSD
**DB ID**	400298
**DF2-C09 Binding energy [meV/ Å^2]**	13.71
**RVV10 Binding energy [meV/ Å^2]**	19.89
**Band gap (PBE) [eV]**	N/A

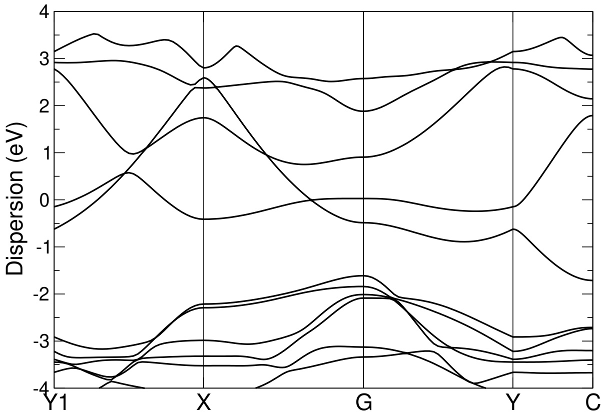

Band structure: Electronic band structure of YCI (C2/m) in a representative window around the Fermi energy and along a high-symmetry path.

Optimized crystal structure

Structural parameters: relaxed cell (top) and atomic positions (bottom) of YCI (C2/m) in Cartesian coordinates.

		x [Å]	y [Å]	z [Å]
$\mathbf{a_1}$		4.15202727	0.00811462	0.00000000
$\mathbf{a_2}$		2.30326331	3.45461639	0.00000000
$\mathbf{a_3}$		0.00000000	0.00000000	27.00868516
		x [Å]	y [Å]	z [Å]
●	Y	0.56302681	0.30201745	12.13260723
●	Y	5.27506205	2.82963574	14.87607359
●	C	2.40379673	1.28943870	13.18812946
●	C	3.43429444	1.84221573	13.82055551
●	I	4.83820405	2.59529745	10.02951789
●	I	0.99992081	0.53637505	16.97917179

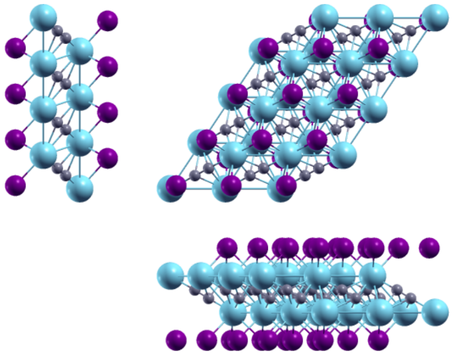

Orthographic projections: views of YCI (C2/m) as seen from the x axis (left), the y axis (bottom) and the z axis (center).

Lattice matching - minimal size

Formula	N° atoms	strain	cell size 1	cell size 2
Na	7	0.2351	1	1
In	7	0.5386	1	1
InSe	8	0.0297	1	1
GeTe	8	0.0255	1	1
AgTl	8	0.1648	1	1
Ag_2	8	0.5432	1	1
As_2	8	0.2881	1	1
S_2	8	0.0245	1	1
PbTe	8	0.0313	1	1
Sb_2	8	0.0195	1	1
CaCl	8	0.1149	1	1
$IrTe_2$	9	0.0249	1	1
Cl_2Zn	9	0.2865	1	1
I_2Mg	9	0.0221	1	1
$CdCl_2$	9	0.0262	1	1
Cl_2Mn	9	0.2067	1	1
PSn_2	9	0.2571	1	1
Ba_2Pt	9	0.5424	1	1
$ReSe_2$	9	0.2205	1	1
S_2Ta	9	0.2088	1	1
Br_2Zn	9	0.3203	1	1
HfS_2	9	0.2924	1	1
$InSe_2$	9	0.0259	1	1
$AsSn_2$	9	0.2624	1	1
$GeTe_2$	9	0.0268	1	1
$HfTe_2$	9	0.0204	1	1
Te_2V	9	0.2403	1	1
I_2Pr	9	0.1439	1	1
I_2Mn	9	0.0261	1	1
$CuTe_2$	9	0.2918	1	1
S_2Zr	9	0.2561	1	1
Br_2La	9	0.0223	1	1
Ca_2Si	9	0.5555	1	1
Br_2Co	9	0.2875	1	1
Ca_2N	9	0.2889	1	1
$AuTe_2$	9	0.0184	1	1
BrCdI	9	0.0241	1	1
LiO_2	9	0.08	1	1
Cl_2Zn	9	0.1239	1	1
$PdTe_2$	9	0.0187	1	1
I_2Ni	9	0.0267	1	1
S_2Ti	9	0.2139	1	1
Te_2Ti	9	0.3208	1	1
NbS_2	9	0.2083	1	1
I_2Zn	9	0.0186	1	1
BaF_2	9	0.0266	1	1
$RhTe_2$	9	0.2664	1	1
GeI_2	9	0.0211	1	1
Br_2Mn	9	0.2847	1	1
Cl_2Ni	9	0.2215	1	1

Table showing the first 50 lattice matching combinations with the smallest number of atoms in the supercell.

Lattice matching - minimal strain

Formula	N° atoms	strain	cell size 1	cell size 2
$Br_2O_2Tm_2$	810	0.0006	68	67
I_4Zr_2	726	0.0006	78	43
MoS_2	435	0.0007	40	65
$CuTe_2$	684	0.0007	70	88
As_2Ru_2	676	0.0008	68	67
S_2W	435	0.0008	40	65
MoS_2	435	0.0008	40	65
Br_2Ca_3Si	906	0.0009	84	67
Pb_2Se_2	242	0.0009	27	20
MnO_2	225	0.0009	18	39
$Cl_2Hg_2N_2$	546	0.0009	61	30
NaO_4	848	0.0009	93	58
NS_2Zr	506	0.0009	45	59
Ca_2Cl_2	676	0.0009	68	67
Sb_2Te_2	534	0.001	59	45
$Bi_2Cl_2O_2$	864	0.001	74	70
$Br_2O_2V_2$	810	0.001	63	72
H_2NiO_2	565	0.001	40	65
Tl	150	0.001	20	30
$Cl_2O_2Sc_2$	696	0.0011	57	59
Br_2Cr	792	0.0011	79	106
Br_2CsF	348	0.0011	40	27
Ni_2Se_2	724	0.0011	74	70
Se_2W	210	0.0012	20	30
$Gd_2I_2Se_2$	924	0.0012	97	57
$CuTe_2$	654	0.0012	67	84
NaO_4	629	0.0012	69	43
ClH_3O	915	0.0012	95	69
$Br_2Er_2O_2$	810	0.0012	68	67
Ho_2S_2	910	0.0012	93	88
Se_2Sn_2	606	0.0012	67	51
I_2Pr	582	0.0012	66	62
$Bi_2Cl_2O_2$	666	0.0012	57	54
$MoSe_2$	210	0.0013	20	30
I_4Zr_2	708	0.0013	76	42
$Ba_2H_2I_2$	402	0.0013	40	27
CeI_2	654	0.0013	74	70
$I_2O_2Yb_2$	768	0.0013	66	62
$Br_2Eu_2O_2$	768	0.0013	66	62
$Ca_2Ge_2Mn_2$	864	0.0013	74	70
Br_2V	630	0.0013	62	86
$ReSe_2$	630	0.0014	62	86
Fe_2Te_2	676	0.0014	68	67
CeI_2	582	0.0014	66	62
Se_2V	210	0.0014	20	30
N_4	624	0.0014	50	81
$Ca_2Ge_2Mn_2$	666	0.0014	57	54
$Gd_2I_2Se_2$	876	0.0014	92	54
Ga_2S_3	603	0.0014	48	63
S_2V	435	0.0014	40	65

Table showing the first 50 lattice matching combinations with the smallest strain needed to build the supercell.

YCl (P-3m1)

Structural and electronic properties

**Formula**	YCl
**Spacegroup**	P-3m1
**Prototype**	ZrCl
**Parent 3D**	Y_2Cl_2
**Source DB**	ICSD
**DB ID**	30708
**DF2-C09 Binding energy [meV/ Å^2]**	11.98
**RVV10 Binding energy [meV/ Å^2]**	17.61
**Band gap (PBE) [eV]**	N/A

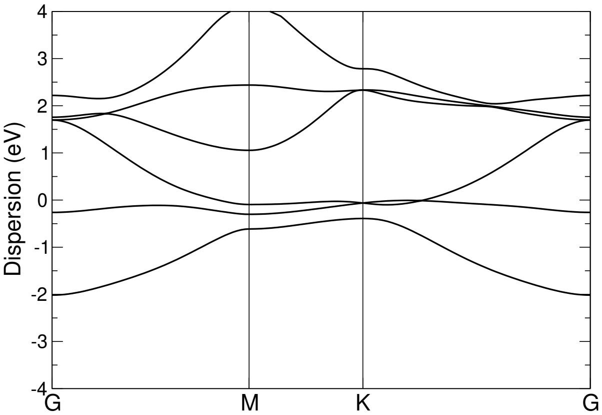

Band structure: Electronic band structure of YCl (P-3m1) in a representative window around the Fermi energy and along a high-symmetry path.

Optimized crystal structure

Structural parameters: relaxed cell (top) and atomic positions (bottom) of YCl (P-3m1) in Cartesian coordinates.

		x [Å]	y [Å]	z [Å]
$\mathbf{a_1}$		3.75153417	0.00000000	0.00000000
$\mathbf{a_2}$		−1.87576709	3.24892390	0.00000000
$\mathbf{a_3}$		0.00000000	0.00000000	26.35817172
		x [Å]	y [Å]	z [Å]
●	Y	0.00000000	2.16594926	11.75985181
●	Cl	1.87576709	3.24892390	10.01824118
●	Cl	1.87576709	3.24892390	16.33993054
●	Y	1.87576709	1.08297463	14.59831991

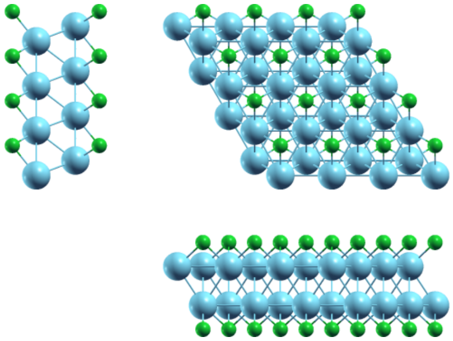

Orthographic projections: views of YCl (P-3m1) as seen from the x axis (left), the y axis (bottom) and the z axis (center).

Lattice matching - minimal size

Formula	N° atoms	strain	cell size 1	cell size 2
Tl	5	0.1124	1	1
Tl	5	0.2566	1	1
InSe	6	0.4831	1	1
HgO	6	0.1176	1	1
Bi_2	6	0.4983	1	1
P_2	6	0.2496	1	1
PbTe	6	2.832	1	1
CaCl	6	0.1543	1	1
I_2Mg	7	0.457	1	1
Cl_2Mn	7	0.2646	1	1
CdI_2	7	2.8539	1	1
$AgTe_2$	7	0.1135	1	1
PSn_2	7	0.0092	1	1
$MoSe_2$	7	0.2556	1	1
S_2Ta	7	0.2676	1	1
Br_2Zn	7	0.0012	1	1
Br_2Ca	7	0.4955	1	1
$AsSn_2$	7	0.0056	1	1
$SiTe_2$	7	0.0054	1	1
Br_2La	7	0.4578	1	1
Br_2Cu	7	0.9577	1	1
PbS_2	7	0.009	1	1
BiClTe	7	0.4932	1	1
Cl_2Ti	7	0.2498	1	1
BrCdI	7	0.4648	1	1
S_2Ti	7	0.2749	1	1
Te_2Ti	7	0.0015	1	1
NbS_2	7	0.267	1	1
BaF_2	7	0.4732	1	1
$RhTe_2$	7	0.0028	1	1
Bi_2Pd	7	0.1228	1	1
GeI_2	7	0.4524	1	1
Cl_2Co	7	0.2744	1	1
NbS_2	7	0.2609	1	1
Cl_2Fe	7	0.2733	1	1
S_2Ta	7	0.2597	1	1
Se_2V	7	0.2578	1	1
AsKSn	7	0.4696	1	1
$PbTe_2$	7	0.4623	1	1
$NiTe_2$	7	0.0048	1	1
I_2V	7	0.0068	1	1
Cl_2V	7	1.5458	1	1
GeI_2	7	2.835	1	1
Se_2Zr	7	0.0058	1	1
STl_2	7	0.4756	1	1
$PtSe_2$	7	0.0049	1	1
BiITe	7	3.0168	1	1
CdO_2	7	0.2742	1	1
GeS_2	7	0.143	1	1
$TaTe_2$	7	0.0061	1	1

Table showing the first 50 lattice matching combinations with the smallest number of atoms in the supercell.

Lattice matching - minimal strain

Formula	N° atoms	strain	cell size 1	cell size 2
Br_2Ca	403	0.0	64	49
S_2Ta	624	0.0	81	100
InSe	406	0.0001	73	57
As_2Li_2Pr	577	0.0001	73	57
Cu_4Te_2	486	0.0001	57	43
$LiNbS_2$	724	0.0001	81	100
$MoSe_2$	447	0.0001	57	73
$I_2La_2Si_2$	634	0.0001	73	57
STl_2	516	0.0001	81	64
Cl_2V	291	0.0002	36	49
O_2Pb_2	452	0.0002	65	48
$F_2I_2Sm_2$	548	0.0002	65	48
Cl_2Mn	565	0.0002	73	91
P_2	324	0.0002	49	64
$AsLi_3$	520	0.0002	73	57
S_2Ta	499	0.0003	64	81
CNb_2S_2	516	0.0003	49	64
Se_2W	447	0.0003	57	73
$AlLiTe_2$	340	0.0004	49	36
$Ga_2I_2Tb_2$	550	0.0004	64	49
Cl_2Ti	388	0.0004	49	64
NbS_2	624	0.0004	81	100
$Er_2I_2S_2$	650	0.0004	89	49
$PbTe_2$	643	0.0005	100	81
CS_2Ta_2	516	0.0005	49	64
FeO_2	84	0.0005	9	16
KNO_3	276	0.0005	49	16
I_2La_2	656	0.0005	91	73
Br_2Cu	568	0.0005	85	76
CBr_2Y_2	9	0.0005	1	1
NS_2Ta	164	0.0006	16	25
$Ga_2Gd_2I_2$	550	0.0007	64	49
$Dy_2I_2S_2$	294	0.0007	36	25
Sb_2Te_3	443	0.0007	57	43
Cl_2NSc_2	747	0.0007	73	91
Ga_2Se_2	8	0.0007	1	1
As_2Li_2Nd	577	0.0007	73	57
$Br_2Dy_2S_2$	788	0.0007	107	60
Br_2Gd_2Ge	805	0.0007	100	81
Cu_2I_2	724	0.0007	100	81
I_2Pr	403	0.0008	64	49
N_3W_2	601	0.0008	49	81
BrCdI	583	0.0008	91	73
BiClTe	403	0.0008	64	49
Sn_2Te_2	692	0.0009	109	64
Tl	301	0.0009	57	73
$I_2Se_2Tb_2$	724	0.0009	103	52
$Eu_2F_2I_2$	548	0.0009	65	48
SSb_2Te_2	729	0.0009	91	73
$Br_2La_2O_2$	548	0.0009	65	48

Table showing the first 50 lattice matching combinations with the smallest strain needed to build the supercell.

YGaI (P-3m1)

Structural and electronic properties

**Formula**	YGaI
**Spacegroup**	P-3m1
**Prototype**	Ce2Si2I2
**Parent 3D**	$Y_2Ga_2I_2$
**Source DB**	ICSD
**DB ID**	417149
**DF2-C09 Binding energy [meV/ Å^2]**	13.38
**RVV10 Binding energy [meV/ Å^2]**	19.18
**Band gap (PBE) [eV]**	N/A

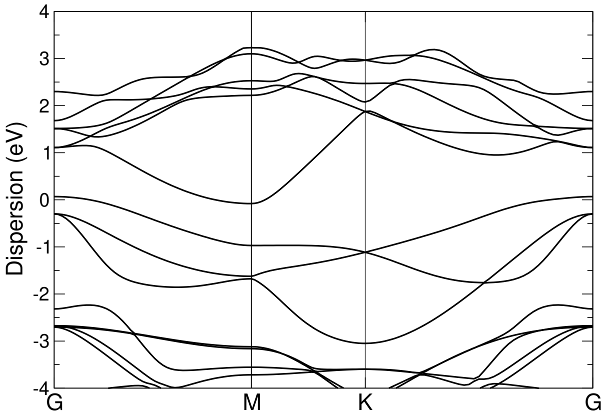

Band structure: Electronic band structure of YGaI (P-3m1) in a representative window around the Fermi energy and along a high-symmetry path.

Optimized crystal structure

Structural parameters: relaxed cell (top) and atomic positions (bottom) of YGaI (P-3m1) in Cartesian coordinates.

	x [Å]	y [Å]	z [Å]
$\mathbf{a_1}$	4.20875570	0.00000000	0.00000000
$\mathbf{a_2}$	−2.10437785	3.64488936	0.00000000
$\mathbf{a_3}$	0.00000000	0.00000000	27.94218859

		x [Å]	y [Å]	z [Å]
●	Y	0.00000000	0.00000000	11.95580853
●	Ga	0.00000000	2.42992624	13.63358020
●	I	2.10437785	1.21496312	9.96366857
●	Y	0.00000000	0.00000000	15.98638006
●	Ga	2.10437785	1.21496312	14.30860839
●	I	0.00000000	2.42992624	17.97852001

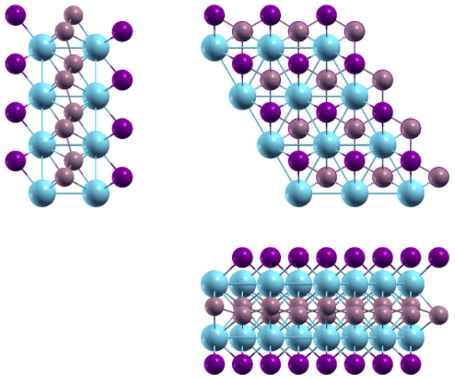

Orthographic projections: views of YGaI (P-3m1) as seen from the x axis (left), the y axis (bottom) and the z axis (center).

Lattice matching - minimal size

Formula	N° atoms	strain	cell size 1	cell size 2
InSe	8	0.0041	1	1
AgTl	8	0.1565	1	1
Ag_2	8	0.4494	1	1
As_2	8	1.548	1	1
PbTe	8	0.0059	1	1
CaCl	8	0.1151	1	1
Cl_2Zn	9	1.5405	1	1
I_2Mg	9	0.0061	1	1
CdI_2	9	0.0077	1	1
PSn_2	9	0.2484	1	1
Ba_2Pt	9	0.4488	1	1
Br_2Zn	9	0.2629	1	1
Br_2Ca	9	0.0089	1	1
$AsSn_2$	9	0.2534	1	1
$SiTe_2$	9	0.2688	1	1
Te_2V	9	1.5211	1	1
I_2Pr	9	0.1398	1	1
S_2Zr	9	0.2474	1	1
Br_2La	9	0.0058	1	1
Br_2Cu	9	0.7329	1	1
Ca_2Si	9	0.4599	1	1
PbS_2	9	0.2739	1	1
Br_2Co	9	1.5449	1	1
BiClTe	9	0.008	1	1
Ca_2N	9	1.5518	1	1
BrCdI	9	0.0031	1	1
Cl_2Zn	9	0.1225	1	1
Te_2Ti	9	0.2633	1	1
BaF_2	9	0.0002	1	1
$RhTe_2$	9	0.2572	1	1
GeI_2	9	0.0079	1	1
Br_2Mn	9	1.532	1	1
AsKSn	9	0.0012	1	1
$PbTe_2$	9	0.004	1	1
I_2Nd	9	0.1406	1	1
$NiTe_2$	9	0.2679	1	1
Cl_2Cu	9	0.0664	1	1
S_2Sn	9	0.2477	1	1
I_2V	9	0.2707	1	1
GeI_2	9	0.0061	1	1
Se_2Zr	9	0.2694	1	1
I_2Pb	9	0.4533	1	1
STl_2	9	0.0012	1	1
$PtSe_2$	9	0.2543	1	1
Br_2Fe	9	1.5454	1	1
GeS_2	9	0.4422	1	1
$MnSe_2$	9	0.1151	1	1
Br_2Ni	9	1.5823	1	1
CeI_2	9	0.1392	1	1
$NbTe_2$	9	0.2471	1	1

Table showing the first 50 lattice matching combinations with the smallest number of atoms in the supercell.

Lattice matching - minimal strain

Formula	N° atoms	strain	cell size 1	cell size 2
CCl_2Lu_2	461	0.0	36	49
Cl_2Sc_2	294	0.0	25	36
Li_2Tl_2	316	0.0001	36	25
$CrTe_2$	258	0.0001	25	36
NS_2Ta	346	0.0001	25	49
As_2	314	0.0002	36	49
Br_2Pr_2	802	0.0002	73	91
Br_2Cr	258	0.0002	25	36
Br_2Fe	363	0.0002	36	49
$NiTe_2$	786	0.0002	81	100
BaF_2	9	0.0002	1	1
Cl_2NSc_2	221	0.0003	16	25
Br_2Co	363	0.0003	36	49
$Br_2H_2Zr_2$	366	0.0003	25	36
ClH_3O	723	0.0003	73	57
CaClHO	886	0.0004	81	100
$I_2N_2Zr_2$	870	0.0004	64	81
CNb_2S_2	527	0.0004	37	61
Ge_2S_2	412	0.0004	42	40
Br_2Ti	258	0.0005	25	36
$Br_2Hf_2N_2$	894	0.0005	73	76
Ca_2Si	843	0.0005	100	81
CdClHO	634	0.0005	57	73
$Br_2N_2Zr_2$	600	0.0006	43	57
In	70	0.0006	9	16
$F_2Se_2Y_2$	600	0.0006	57	43
Ga_2Se_2	708	0.0006	64	81
CBr_2Lu_2	543	0.0006	43	57
$BrKO_3$	195	0.0006	25	9
PSn_2	486	0.0007	49	64
Ca_2N	363	0.0007	36	49
$HfSe_2$	711	0.0007	73	91
HgO	722	0.0007	81	118
Te_2Ti	711	0.0007	73	91
Cl_2Mn	171	0.0007	16	25
$SiTe_2$	786	0.0008	81	100
FKO_2Se	750	0.0008	85	48
NbS_2	171	0.0009	16	25
$Cl_2O_2Yb_2$	984	0.0009	73	91
$PtSe_2$	561	0.0009	57	73
IO_3Tl	509	0.0009	64	25
Cl_2Zn	363	0.0009	36	49
P_2	344	0.0009	37	61
O_4PSn	678	0.0009	65	48
F_2Na	711	0.0009	73	91
O_2Pt	102	0.001	9	16
$AgCuTe_2$	894	0.001	85	96
$Cu_2K_2Te_2$	678	0.001	65	48
$Cl_2H_2Lu_2$	600	0.001	43	57
$Cl_2H_2Sc_2$	366	0.001	25	36

Table showing the first 50 lattice matching combinations with the smallest strain needed to build the supercell.

YOCl (P-3m1)

Structural and electronic properties

**Formula**	YOCl
**Spacegroup**	P-3m1
**Prototype**	SmSI
**Parent 3D**	$Y_2O_2Cl_2$
**Source DB**	ICSD
**DB ID**	60585
**DF2-C09 Binding energy [meV/ Å^2]**	10.22
**RVV10 Binding energy [meV/ Å^2]**	16.72
**Band gap (PBE) [eV]**	4.39

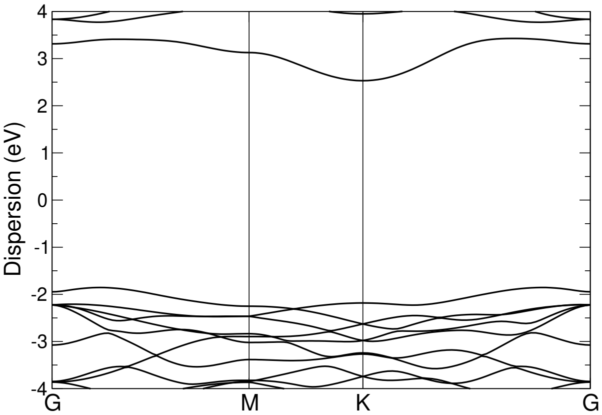

Band structure: Electronic band structure of YOCl (P-3m1) in a representative window around the Fermi energy and along a high-symmetry path.

Optimized crystal structure

Structural parameters: relaxed cell (top) and atomic positions (bottom) of YOCl (P-3m1) in Cartesian coordinates.

	x [Å]	y [Å]	z [Å]
$\mathbf{a_1}$	3.81375036	−0.00000000	0.00000000
$\mathbf{a_2}$	−1.90687518	3.30280470	0.00000000
$\mathbf{a_3}$	0.00000000	0.00000000	26.36883235

		x [Å]	y [Å]	z [Å]
●	Cl	0.00000000	2.20186980	16.35173711
●	Y	1.90687518	1.10093490	11.75421303
●	O	1.90687518	1.10093490	14.03583392
●	Y	1.90687518	3.30280470	14.61461933
●	O	1.90687518	3.30280470	12.33299843
●	Cl	-0.00000000	2.20186980	10.01709525

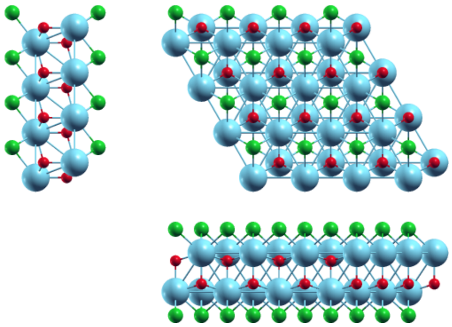

Orthographic projections: views of YOCl (P-3m1) as seen from the x axis (left), the y axis (bottom) and the z axis (center).

Lattice matching - minimal size

Formula	N° atoms	strain	cell size 1	cell size 2
Tl	7	0.2463	1	1
InSe	8	0.4633	1	1
Nd	8	0.2245	1	2
HgO	8	0.1141	1	1
AsSb	8	0.0037	1	1
Bi_2	8	0.478	1	1
P_2	8	1.5629	1	1
PbTe	8	0.4677	1	1
CaCl	8	0.1469	1	1
$CdCl_2$	9	0.0088	1	1
Cl_2Mn	9	0.2539	1	1
CdI_2	9	0.4721	1	1
$AgTe_2$	9	0.4435	1	1
$MoSe_2$	9	1.5959	1	1
$ReSe_2$	9	0.2724	1	1
S_2Ta	9	0.2567	1	1
Br_2Zn	9	0.0064	1	1
Br_2Ca	9	0.4752	1	1
$InSe_2$	9	0.0092	1	1
$GeTe_2$	9	0.0078	1	1
$SiTe_2$	9	0.0024	1	1
I_2Mn	9	0.009	1	1
NSr_2	9	0.0054	1	1
I_2Yb	9	3.0172	1	1
PbS_2	9	0.0011	1	1
BiClTe	9	0.473	1	1
Cl_2Ti	9	1.564	1	1
FeI_2	9	0.0067	1	1
I_2Ni	9	0.008	1	1
S_2Ti	9	0.2636	1	1
Mg_3	9	0.4232	1	1
Te_2Ti	9	0.0061	1	1
NbS_2	9	0.2561	1	1
CrI_2	9	0.0063	1	1
BaF_2	9	0.4538	1	1
BiBrTe	9	0.4873	1	1
Bi_2Pd	9	0.1185	1	1
Cl_2Ni	9	0.2737	1	1
Cl_2Co	9	0.2631	1	1
NbS_2	9	0.2503	1	1
Br_2V	9	0.2712	1	1
ClNZr	9	0.2681	1	1
Cl_2Fe	9	0.2621	1	1
S_2Ta	9	0.2492	1	1
Se_2V	9	0.2474	1	1
AsKSn	9	0.4502	1	1
$AsSe_2$	9	0.2759	1	1
$NiTe_2$	9	0.003	1	1
I_2Tm	9	3.0355	1	1
I_2V	9	0.0011	1	1

Table showing the first 50 lattice matching combinations with the smallest number of atoms in the supercell.

Lattice matching - minimal strain

Formula	N° atoms	strain	cell size 1	cell size 2
BiITe	471	0.0	57	43
N_2Re	102	0.0	9	16
$Ga_2I_2Tb_2$	870	0.0	81	64
K	241	0.0001	36	25
S_2Ta	486	0.0001	49	64
ClH_3O	230	0.0001	25	16
Bi_2Te_3	557	0.0001	57	43
As_2O_3	669	0.0001	79	39
GdI_2	531	0.0002	64	49
Cu_4Te_2	780	0.0003	73	57
I_2La_2Sb	341	0.0003	36	25
Sb_2Te_3	723	0.0003	73	57
Br_2Ca	678	0.0003	81	64
$HNiO_2$	466	0.0003	37	61
PbTe	692	0.0003	91	73
Ca_4Cu_2	894	0.0003	99	50
H_2MgO_2	330	0.0003	25	36
$Ga_2Gd_2I_2$	870	0.0003	81	64
As_2CeLi_2	911	0.0004	91	73
ClNZr	786	0.0004	81	100
$I_2S_2Sm_2$	366	0.0004	36	25
NbS_2	561	0.0005	57	73
$MoSe_2$	429	0.0005	43	57
S_2Ti	711	0.0005	73	91
Bi_2STe_2	911	0.0005	91	73
Ga_2Se_2	10	0.0005	1	1
Cl_2ORu	718	0.0005	73	70
S_2V	258	0.0005	25	36
GeI_2	765	0.0006	91	73
F_4Nb	630	0.0006	65	48
Se_2W	429	0.0006	43	57
Cl_2Hf_2	708	0.0007	64	81
Cl_2NSc_2	707	0.0007	57	73
$Cl_2Ho_2O_2$	12	0.0007	1	1
Cu_2O_2	606	0.0007	65	54
$Al_2Cl_2O_2$	624	0.0007	50	54
Bi_2	614	0.0008	81	64
NbS_2	486	0.0008	49	64
Cl_2Co	711	0.0008	73	91
InSe	762	0.0008	100	81
$LiMnTe_2$	838	0.0009	91	73
Br_2Y_2	10	0.0009	1	1
$Br_2Hf_2N_2$	834	0.0009	75	64
$Br_2O_2Ti_2$	756	0.0009	66	60
S_2Ta	561	0.0009	57	73
Cu_4Te_2	702	0.0009	65	52
$Cl_2N_2Ti_2$	426	0.0009	36	35
CdO_2	711	0.001	73	91
$LiNbS_2$	634	0.001	57	73
I_2V	9	0.0011	1	1

Table showing the first 50 lattice matching combinations with the smallest strain needed to build the supercell.

$Yb_2Br_2F_2$ (P4/nmm)

Structural and electronic properties

**Formula**	$Yb_2Br_2F_2$
**Spacegroup**	P4/nmm
**Prototype**	PbClF
**Parent 3D**	$Br_2F_2Yb_2$
**Source DB**	MPDS
**DB ID**	S1800111
**DF2-C09 Binding energy [meV/ Å^2]**	23.4
**RVV10 Binding energy [meV/ Å^2]**	N/A
**Band gap (PBE) [eV]**	0.37

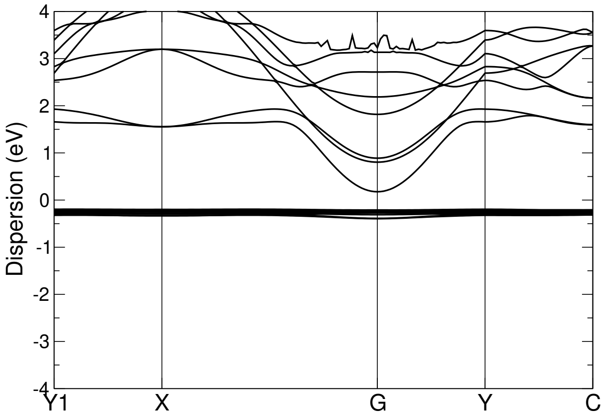

Band structure: Electronic band structure of $Yb_2Br_2F_2$ (P4/nmm) in a representative window around the Fermi energy and along a high-symmetry path.

Optimized crystal structure

Structural parameters: relaxed cell (top) and atomic positions (bottom) of $Yb_2Br_2F_2$ (P4/nmm) in Cartesian coordinates.

	x [Å]	y [Å]	z [Å]
$\mathbf{a_1}$	3.86699517	0.00008154	0.00000000
$\mathbf{a_2}$	0.00008154	3.86699517	0.00000000
$\mathbf{a_3}$	0.00000000	0.00000000	22.38299868

		x [Å]	y [Å]	z [Å]
●	Yb	0.96665128	-0.96665128	1.43654192
●	Br	-0.96681692	-2.90025979	2.81604306
●	Yb	-0.96673282	-2.90034389	-1.43654192
●	Br	0.96673539	-0.96673539	-2.81604306
•	F	-0.96678525	-0.96678525	0.00000000
•	F	0.96670371	-2.90020992	0.00000000

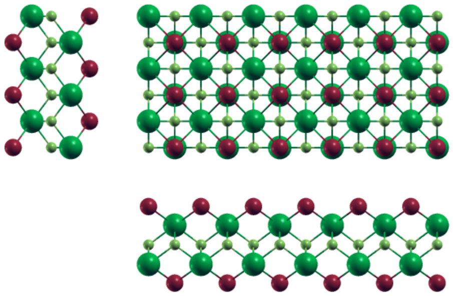

Orthographic projections: views of $Yb_2Br_2F_2$ (P4/nmm) as seen from the x axis (left), the y axis (bottom) and the z axis (center).

Lattice matching - minimal size

Formula	N° atoms	strain	cell size 1	cell size 2
K	7	0.3873	1	1
K	7	0.1901	1	1
In	7	0.111	1	1
InSe	8	0.15	1	1
Bi_2	8	0.1557	1	1
Ag_2	8	0.1973	1	1
LiO	8	0.1088	1	1
PbTe	8	0.1517	1	1
Sb_2	8	0.136	1	1
I_2Mg	9	0.1407	1	1
S_2V	9	0.1103	1	1
MoS_2	9	0.1101	1	1
CdI_2	9	0.1534	1	1
Nd	9	0.7741	1	3
PSn_2	9	0.1089	1	1
Ba_2Pt	9	0.197	1	1
Br_2Ca	9	0.1546	1	1
CaI_2	9	0.1785	1	1
I_2Pr	9	0.0048	1	1
S_2Zr	9	0.1088	1	1
Br_2La	9	0.141	1	1
Br_2Cu	9	0.1078	1	1
Ca_2Si	9	0.7674	1	1
I_2Yb	9	0.1755	1	1
BiClTe	9	0.1538	1	1
$AuTe_2$	9	0.1301	1	1
BrCdI	9	0.1434	1	1
I_2Zn	9	0.1335	1	1
BaF_2	9	0.1464	1	1
BiBrTe	9	0.1594	1	1
S_2W	9	0.1101	1	1
Bi_2Pd	9	0.5649	1	1
GeI_2	9	0.1391	1	1
AsKSn	9	0.1451	1	1
$PbTe_2$	9	0.1426	1	1
I_2Nd	9	0.0058	1	1
Cl_2Cu	9	0.0996	1	1
I_2Tm	9	0.1771	1	1
S_2Sn	9	0.1088	1	1
$SnTe_2$	9	0.1373	1	1
Cl_2V	9	0.1093	1	1
GeI_2	9	0.1519	1	1
STl_2	9	0.1473	1	1
BiITe	9	0.1665	1	1
DyI_2	9	0.182	1	1
CeI_2	9	0.004	1	1
$NbTe_2$	9	0.1087	1	1
Se_2Yb	9	0.1393	1	1
MoS_2	9	0.1101	1	1
$BiTe_2$	9	0.1395	1	1

Table showing the first 50 lattice matching combinations with the smallest number of atoms in the supercell.

Lattice matching - minimal strain

Formula	N° atoms	strain	cell size 1	cell size 2
$Cu_2Na_2Te_2$	870	0.0	81	64
Se_2Ta_4	678	0.0001	49	64
CdH_2O_2	613	0.0002	48	65
F_4Pb	640	0.0002	65	50
$Cu_2K_2Te_2$	876	0.0003	85	61
$Cl_2S_2Tl_2$	780	0.0004	81	49
Bi_2Pd	363	0.0004	36	49
$Br_2Ca_2F_2$	12	0.0004	1	1
LiO_2	510	0.0005	49	72
Br_2Cu_2	10	0.0006	1	1
Cl_4Cu_2	534	0.0006	64	25
$HNiO_2$	276	0.0006	20	39
Te_2W	483	0.0006	48	65
Bi_2In_2	316	0.0006	36	25
Mg_3	942	0.0006	89	136
Cl_2Zr_2	958	0.0007	81	118
Cl_2Rb_2	186	0.0007	25	9
CdClO	483	0.0007	48	65
$MoTe_2$	483	0.0007	48	65
F_2Zn	9	0.0008	1	1
Ca_2O_2	412	0.0009	36	49
Te_2Zn	483	0.0009	48	65
OTl_2	483	0.0009	48	65
$Cu_2Rb_2Te_2$	366	0.0009	36	25
$Cl_2Hf_2N_2$	678	0.0009	48	65
$Br_2O_2Tb_2$	12	0.001	1	1
Pd_2S_4	708	0.001	79	39
$AgClO_4$	246	0.001	25	16
O_4PTl	510	0.0011	49	36
$Br_2Gd_2O_2$	12	0.0011	1	1
$Ba_2F_2I_2$	510	0.0011	49	36
Sn	655	0.0011	85	145
$Cl_2O_2V_2$	54	0.0012	4	5
$HfLiS_2$	548	0.0012	48	65
Te_2V	483	0.0012	48	65
ClNZr	840	0.0012	81	118
Br_2V	840	0.0012	81	118
Cl_2Ti	852	0.0013	79	126
FKO_2Se	700	0.0013	80	44
F_4Pb	629	0.0013	64	49
P_2	726	0.0013	79	126
$AgNO_2$	848	0.0014	88	80
In	445	0.0014	58	97
S_2V	711	0.0014	64	109
MoS_2	711	0.0015	64	109
S_2W	711	0.0015	64	109
Cl_2Sc_2	604	0.0015	52	73
$CrTe_2$	531	0.0015	52	73
Br_2Cr	531	0.0015	52	73
MoS_2	711	0.0015	64	109

Table showing the first 50 lattice matching combinations with the smallest strain needed to build the supercell.

$Yb_2H_2Br_2$ (P4/nmm)

Structural and electronic properties

**Formula**	$Yb_2H_2Br_2$
**Spacegroup**	P4/nmm
**Prototype**	PbClF
**Parent 3D**	$Br_2H_2Yb_2$
**Source DB**	MPDS
**DB ID**	S1937759
**DF2-C09 Binding energy [meV/ Å^2]**	17.41
**RVV10 Binding energy [meV/ Å^2]**	N/A
**Band gap (PBE) [eV]**	0.78

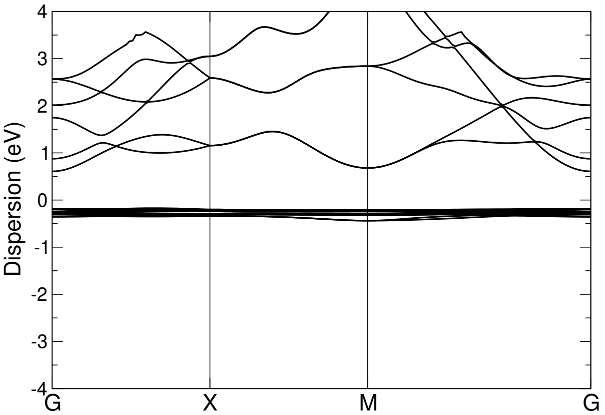

Band structure: Electronic band structure of $Yb_2H_2Br_2$ (P4/nmm) in a representative window around the Fermi energy and along a high-symmetry path.

Optimized crystal structure

Structural parameters: relaxed cell (top) and atomic positions (bottom) of $Yb_2H_2Br_2$ (P4/nmm) in Cartesian coordinates.

	x [Å]	y [Å]	z [Å]
$\mathbf{a_1}$	3.75518770	0.00000000	0.00000000
$\mathbf{a_2}$	0.00000000	3.75518770	0.00000000
$\mathbf{a_3}$	0.00000000	0.00000000	22.42591593

	x [Å]	y [Å]	z [Å]
Yb	-0.93879692	-2.81639077	-1.27587731
Br	0.93879692	-0.93879692	-2.70721638
Yb	0.93879692	-0.93879692	1.27587731
H	-0.93879692	-0.93879692	0.00000000
H	0.93879692	-2.81639077	0.00000000
Br	-0.93879692	-2.81639077	2.70721638

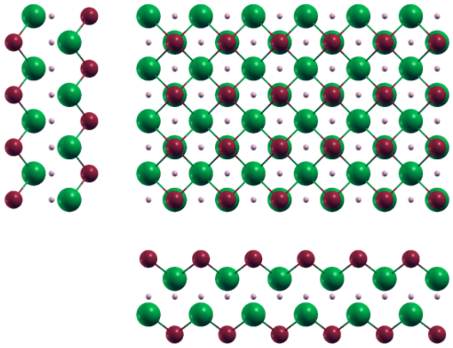

Orthographic projections: views of $Yb_2H_2Br_2$ (P4/nmm) as seen from the x axis (left), the y axis (bottom) and the z axis (center).

Lattice matching - minimal size

Formula	N° atoms	strain	cell size 1	cell size 2
K	7	1.125	1	1
K	7	0.7834	1	1
InSe	8	0.1642	1	1
Bi_2	8	0.1707	1	1
AgTl	8	0.366	1	1
PbTe	8	0.1662	1	1
Sb_2	8	0.148	1	1
CrS_2	9	0.1112	1	1
I_2Mg	9	0.1535	1	1
CdI_2	9	0.1681	1	1
$MoTe_2$	9	0.1087	1	1
Br_2Ca	9	0.1695	1	1
CaI_2	9	0.1963	1	1
$HfTe_2$	9	0.1346	1	1
Br_2La	9	0.1538	1	1
Br_2Cu	9	0.1158	1	1
I_2Yb	9	0.1929	1	1
BiClTe	9	0.1685	1	1
ReS_2	9	0.1099	1	1
$AuTe_2$	9	0.141	1	1
BrCdI	9	0.1567	1	1
Cl_2Zn	9	0.0054	1	1
$PdTe_2$	9	0.1389	1	1
I_2Zn	9	0.145	1	1
BaF_2	9	0.1601	1	1
BiBrTe	9	0.1749	1	1
Bi_2Pd	9	0.2087	1	1
GeI_2	9	0.1517	1	1
Ba_2Hg	9	0.3849	1	1
PtS_2	9	0.1085	1	1
Ba_2N	9	0.1358	1	1
AsKSn	9	0.1586	1	1
Te_2Zr	9	0.135	1	1
Te_2W	9	0.1088	1	1
$PbTe_2$	9	0.1557	1	1
Cl_2Cu	9	0.1043	1	1
I_2Tm	9	0.1947	1	1
$SnTe_2$	9	0.1495	1	1
GeI_2	9	0.1664	1	1
STl_2	9	0.1611	1	1
BiITe	9	0.1829	1	1
Se_2Yb	9	0.1519	1	1
$BiTe_2$	9	0.1522	1	1
GdI_2	9	0.1784	1	1
$PtTe_2$	9	0.1406	1	1
Br_2Cd	9	0.1381	1	1
CdI_2	9	0.1676	1	1
Ba_2Cd	9	0.3906	1	1
NaPSn	9	0.1339	1	1
I_2Pr	9	0.1686	1	1

Table showing the first 50 lattice matching combinations with the smallest number of atoms in the supercell.

Lattice matching - minimal strain

Formula	N° atoms	strain	cell size 1	cell size 2
$As_2Fe_2Li_2$	12	0.0	1	1
$H_2I_2Sr_2$	678	0.0001	64	49
Se_2Ta	483	0.0002	48	65
$NbSe_2$	483	0.0003	48	65
Fe_2SeTe	10	0.0003	1	1
$AgTe_2$	621	0.0003	61	85
$C_2Br_2La_2$	138	0.0004	12	11
Cl_4KTl	390	0.0004	49	16
Cl_4Mn	230	0.0004	25	16
La_2S_2	570	0.0006	63	48
Gd	382	0.0006	46	106
NbS_2	840	0.0006	81	118
Cl_2Ni	483	0.0007	48	65
Se_2Sn_2	504	0.0007	56	42
PbS_2Sn	742	0.0008	81	64
Mg_3	258	0.0008	25	36
LiO_2	366	0.0008	36	50
$AsSe_2$	483	0.0008	48	65
AuI_4Li	912	0.001	105	47
$Br_2Lu_2O_2$	12	0.001	1	1
$I_2Se_2Tb_2$	228	0.0011	24	14
$C_2Br_2Y_2$	888	0.0011	72	76
$NbSe_2$	483	0.0011	48	65
S_2Ta	840	0.0011	81	118
$F_2I_2Pb_2$	882	0.0012	82	65
$AgClO_4$	930	0.0012	97	58
Ba_2Hg	843	0.0012	100	81
$AgCuTe_2$	548	0.0012	54	56
$FeSe_2$	537	0.0012	49	81
$Ge_2Te_2Zr_2$	12	0.0013	1	1
Ba_2Cd	687	0.0013	82	65
K	763	0.0013	113	85
PbS_2Sn	732	0.0014	80	63
C_4Ca_2	870	0.0014	79	66
Cu_2Sr_2	974	0.0014	103	89
MoS_2	852	0.0015	79	126
CoH_2O_2	929	0.0015	64	109
Cl_2Fe	531	0.0015	52	73
Cl_2Zr	531	0.0015	52	73
$ReSe_2$	483	0.0015	48	65
O_4PTl	366	0.0016	36	25
Cu_2Sr_2	842	0.0016	89	77
S_2W	852	0.0016	79	126
$AgClO_4$	780	0.0016	81	49
C_2Li_2	862	0.0016	89	82
Sm	403	0.0016	48	115
$AgClO_2$	928	0.0016	92	94
$Cl_2N_2Zr_2$	834	0.0016	62	77
MoS_2	852	0.0016	79	126
Cl_2Co	531	0.0016	52	73

Table showing the first 50 lattice matching combinations with the smallest strain needed to build the supercell.

$Yb_2I_2F_2$ (P4/nmm)

Structural and electronic properties

**Formula**	$Yb_2I_2F_2$
**Spacegroup**	P4/nmm
**Prototype**	PbClF
**Parent 3D**	$F_2I_2Yb_2$
**Source DB**	MPDS
**DB ID**	S1800112
**DF2-C09 Binding energy [meV/ Å^2]**	14.78
**RVV10 Binding energy [meV/ Å^2]**	N/A
**Band gap (PBE) [eV]**	0.74

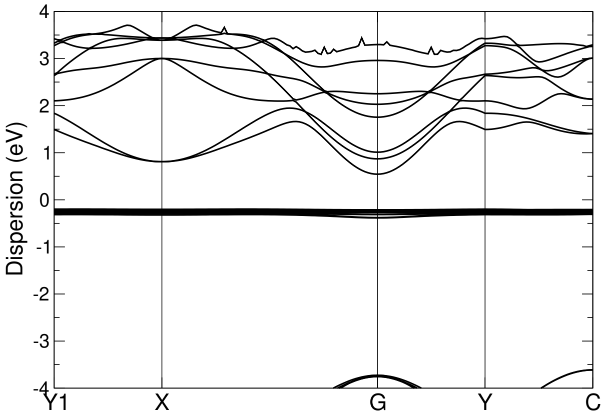

Band structure: Electronic band structure of $Yb_2I_2F_2$ (P4/nmm) in a representative window around the Fermi energy and along a high-symmetry path.

Optimized crystal structure

Structural parameters: relaxed cell (top) and atomic positions (bottom) of $Yb_2I_2F_2$ (P4/nmm) in Cartesian coordinates.

		x [Å]	y [Å]	z [Å]
$\mathbf{a_1}$		4.01243090	0.00004712	0.00000000
$\mathbf{a_2}$		0.00004712	4.01243090	0.00000000
$\mathbf{a_3}$		0.00000000	0.00000000	23.70756680
		x [Å]	y [Å]	z [Å]
●	Yb	1.00314641	-1.00314641	1.37425778
●	I	-1.00310582	-3.00937221	2.99594722
●	Yb	-1.00319353	-3.00928449	-1.37425778
●	I	1.00305870	-1.00305870	-2.99594722
•	F	-1.00310210	-1.00310210	0.00000000
•	F	1.00305498	-3.00932880	0.00000000

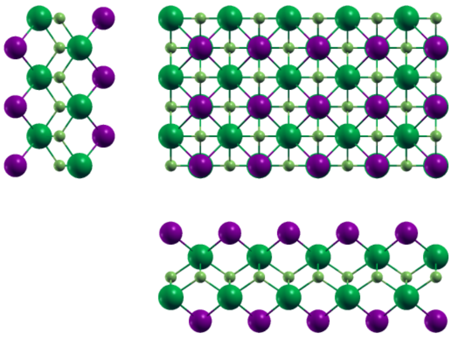

Orthographic projections: views of $Yb_2I_2F_2$ (P4/nmm) as seen from the x axis (left), the y axis (bottom) and the z axis (center).

Lattice matching - minimal size

Formula	N° atoms	strain	cell size 1	cell size 2
K	7	0.1689	1	1
Tl	7	0.1097	1	1
InSe	8	0.1349	1	1
Bi_2	8	0.1396	1	1
AgTl	8	0.0135	1	1
Ag_2	8	0.1752	1	1
P_2	8	0.1108	1	1
PbTe	8	0.1363	1	1
CaCl	8	0.2177	1	1
CdI_2	9	0.1377	1	1
$MoSe_2$	9	0.1098	1	1
Ba_2Pt	9	0.1749	1	1
Br_2Ca	9	0.1387	1	1
CaI_2	9	0.1588	1	1
Br_2Cu	9	0.1007	1	1
Ca_2Si	9	0.1804	1	1
I_2Yb	9	0.1562	1	1
BiClTe	9	0.1379	1	1
Cl_2Ti	9	0.1108	1	1
BrCdI	9	0.1296	1	1
HgI_2	9	0.3866	1	1
BaF_2	9	0.132	1	1
BiBrTe	9	0.1426	1	1
NbS_2	9	0.1091	1	1
S_2Ta	9	0.1092	1	1
Se_2V	9	0.1095	1	1
AsKSn	9	0.1309	1	1
Cl_2Cu	9	0.0967	1	1
I_2Tm	9	0.1576	1	1
I_2V	9	0.1086	1	1
GeI_2	9	0.1365	1	1
Se_2Zr	9	0.1084	1	1
I_2Pb	9	0.1771	1	1
STl_2	9	0.1327	1	1
BiITe	9	0.1486	1	1
GeS_2	9	0.5941	1	1
$MnSe_2$	9	0.2176	1	1
DyI_2	9	0.1618	1	1
GdI_2	9	0.1452	1	1
I_2La	9	0.0069	1	1
CdI_2	9	0.1373	1	1
Sm	9	0.1633	1	3
I_2Pr	9	0.138	1	1
Se_2W	9	0.1098	1	1
Bi_2Te_2	10	0.1967	1	1
Cu_2I_2	10	0.0094	1	1
Cu_2Sr_2	10	0.1435	1	1
Cl_2OOs	10	0.2241	1	1
$LiMnTe_2$	10	0.1367	1	1
Cu_2Te_2	10	0.0567	1	1

Table showing the first 50 lattice matching combinations with the smallest number of atoms in the supercell.

Lattice matching - minimal strain

Formula	N° atoms	strain	cell size 1	cell size 2
$H_2Li_2O_2$	870	0.0	64	81
$Hf_2Se_2Si_2$	690	0.0	50	65
HgO	806	0.0002	89	136
GeS_2	486	0.0003	49	64
$AsSn_2$	483	0.0003	48	65
$Ba_2Ge_2Mn_2$	12	0.0003	1	1
$Eu_2H_2I_2$	12	0.0004	1	1
$TaTe_2$	483	0.0004	48	65
$AgClO_2$	852	0.0004	80	93
Mg_3	537	0.0005	49	81
As_2Fe_2	560	0.0005	50	65
As_2Fe_2	550	0.0006	49	64
$I_2O_2Sm_2$	12	0.0007	1	1
$Cu_2K_2Te_2$	690	0.0008	65	50
Co_2S_2	886	0.0008	81	100
CdClO	840	0.0008	81	118
O_4PTl	870	0.0008	81	64
$Ba_2F_2I_2$	870	0.0008	81	64
Cl_4Mn	474	0.0009	49	36
$PtSe_2$	483	0.0009	48	65
$Hf_2I_2N_2$	678	0.0009	48	65
CdClHO	548	0.0009	48	65
OTl_2	840	0.001	81	118
Te_2W	840	0.001	81	118
Tl	859	0.0011	113	181
H_2Na_2Pd	614	0.0011	49	64
S_2Ti	852	0.0011	79	126
$Hf_2Se_2Si_2$	678	0.0011	49	64
$Ba_2H_2I_2$	678	0.0011	64	49
Mg_6	246	0.0011	16	25
$MoTe_2$	840	0.0011	81	118
Pb_2Se_2	912	0.0011	98	81
Cl_2Co	852	0.0012	79	126
Te_2V	840	0.0012	81	118
CoH_2O_2	315	0.0012	20	39
I_3Sn	866	0.0013	109	53
Hg_4O_2	906	0.0013	120	31
Cl_2Zr	852	0.0014	79	126
$AgClO_2$	468	0.0014	44	51
CaCl	686	0.0015	81	100
$HfLiS_2$	958	0.0015	81	118
$AgClO_2$	384	0.0015	36	42
$MnSe_2$	786	0.0015	81	100
Ga_2S_2	604	0.0015	52	73
$F_2I_2Tm_2$	12	0.0015	1	1
I_2Pb	885	0.0016	103	89
Fe_2S_2	718	0.0016	65	82
$Br_2Cr_2S_2$	558	0.0016	45	48
FeI_2	603	0.0016	62	77
Br_2Mg	603	0.0016	62	77

Table showing the first 50 lattice matching combinations with the smallest strain needed to build the supercell.

$Yb_2S_2Br_2$ (Pmm2)

Structural and electronic properties

**Formula**	$Yb_2S_2Br_2$
**Spacegroup**	Pmm2
**Prototype**	FeOCl
**Parent 3D**	$Br_2S_2Yb_2$
**Source DB**	MPDS
**DB ID**	S307275
**DF2-C09 Binding energy [meV/ Å^2]**	10.73
**RVV10 Binding energy [meV/ Å^2]**	N/A
**Band gap (PBE) [eV]**	0.0

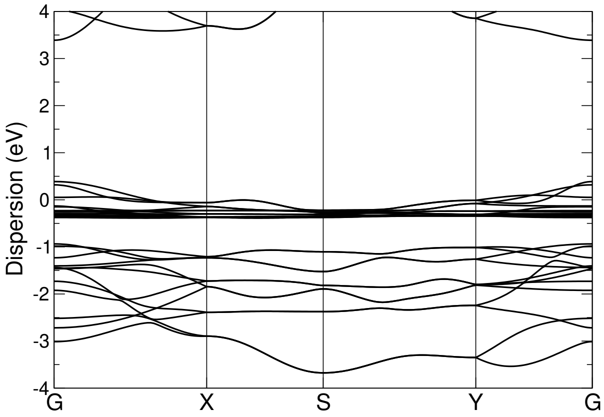

Band structure: Electronic band structure of $Yb_2S_2Br_2$ (Pmm2) in a representative window around the Fermi energy and along a high-symmetry path.

Optimized crystal structure

Structural parameters: relaxed cell (top) and atomic positions (bottom) of $Yb_2S_2Br_2$ (Pmm2) in Cartesian coordinates.

	x [Å]	y [Å]	z [Å]
$\mathbf{a_1}$	4.03331098	0.00000000	0.00000000
$\mathbf{a_2}$	0.00000000	5.28268238	0.00000000
$\mathbf{a_3}$	0.00000000	0.00000000	24.09022039

		x [Å]	y [Å]	z [Å]
●	Yb	1.00849782	-1.32067060	-1.15615218
●	S	1.00819347	-3.96201179	-0.68884793
●	Br	-1.00809324	-1.32067060	-3.08789129
●	Yb	-1.00849782	-3.96201179	1.15615218
●	S	-1.00819347	-1.32067060	0.68884793
●	Br	1.00809324	-3.96201179	3.08789129

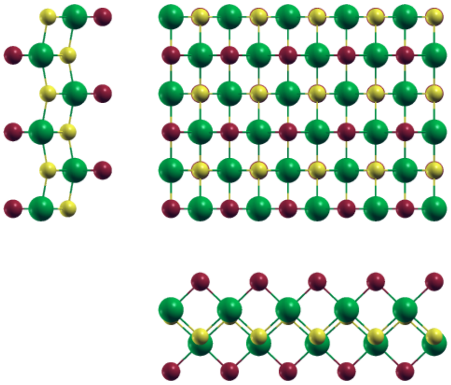

Orthographic projections: views of $Yb_2S_2Br_2$ (Pmm2) as seen from the x axis (left), the y axis (bottom) and the z axis (center).

Lattice matching - minimal size

Formula	N° atoms	strain	cell size 1	cell size 2
AgTl	8	0.2635	1	1
Gd	8	0.1312	1	2
Ba_2Hg	9	0.2795	1	1
CNRb	9	0.3188	1	1
CNNa	9	0.3137	1	1
Bi_2In_2	10	0.0658	1	1
Ge_2Te_2	10	0.3115	1	1
O_2Pb_2	10	0.7741	1	1
As_4	10	0.3199	1	1
O_2Sn_2	10	0.1699	1	1
PbS_2Sn	10	0.826	1	1
$SbSe_2Tl$	10	0.5047	1	1
Br_2CsF	10	0.0649	1	1
Sn_2Te_2	10	0.0649	1	1
F_4Sn	11	0.2783	1	1
FKO_2Se	11	1.1812	1	1
Cl_4Mn	11	0.3757	1	1
$Ba_2H_2I_2$	12	0.0642	1	1
CrS_2	12	0.138	1	2
$Br_2Ho_2S_2$	12	0.0039	1	1
S_2V	12	0.3464	1	2
MoS_2	12	0.3477	1	2
$I_2Lu_2Se_2$	12	0.5679	1	1
$Ho_2I_2S_2$	12	0.0136	1	1
Cu_4Te_2	12	0.1189	1	1
AlH_4Na	12	0.381	1	1
$GeTe_2$	12	0.6789	1	2
$Cl_2F_2Pb_2$	12	0.254	1	1
$F_2I_2Sm_2$	12	0.7734	1	1
$Cl_2H_2Zr_2$	12	0.2753	1	1
ReS_2	12	0.1413	1	2
Br_2Ca_3Si	12	0.2799	1	1
$Ba_2Ge_2Mn_2$	12	0.7527	1	1
$K_2O_2Tl_2$	12	0.5756	1	1
$Br_2S_2Y_2$	12	0.0042	1	1
S_2W	12	0.3478	1	2
$Gd_2I_2S_2$	12	0.0192	1	1
$C_2Br_2Gd_2$	12	0.1079	1	1
$Cu_2Na_2Se_2$	12	0.2539	1	1
$F_2I_2Yb_2$	12	0.7516	1	1
$I_2S_2Tb_2$	12	0.0169	1	1
Te_4TiZr	12	0.6792	1	1
$I_2S_2Yb_2$	12	0.0084	1	1
$Cu_2Rb_2Te_2$	12	0.0656	1	1
$Br_2Dy_2S_2$	12	0.0061	1	1
$Cl_2Ga_2Te_2$	12	0.5446	1	1
$I_2Nd_2O_2$	12	0.2551	1	1
$Br_2Ga_2Te_2$	12	0.554	1	1
Ca_4Cu_2	12	0.5431	1	1
$Br_2Lu_2S_2$	12	0.0061	1	1

Table showing the first 50 lattice matching combinations with the smallest number of atoms in the supercell.

Lattice matching - minimal strain

Formula	N° atoms	strain	cell size 1	cell size 2
Bi_2STe_2	857	0.0005	67	91
CBr_2Lu_2	455	0.0006	30	55
$Br_2N_2Zr_2$	510	0.0006	30	55
Br_2CsF	560	0.0007	56	56
PbTe	584	0.0007	67	91
$I_2N_2Ti_2$	318	0.0007	21	32
As_2CeLi_2	857	0.0007	67	91
PTe_2Zr_2	704	0.0007	49	82
I_2La_2P	875	0.0008	70	91
GeI_2	675	0.0009	67	91
$NbTe_2$	345	0.0009	30	55
$AsLi_3$	766	0.001	67	91
$Hf_2Se_2Si_2$	228	0.001	14	24
GdI_2	693	0.001	70	91
$As_2Cd_2K_2$	660	0.001	54	56
InSe	602	0.0011	69	94
S_2Zr	345	0.0011	30	55
As_2Li_2Pr	884	0.0011	69	94
$LiMnTe_2$	766	0.0012	67	91
$I_2La_2Si_2$	978	0.0012	69	94
Cl_4Mn	606	0.0012	56	54
$Ba_2F_2I_2$	660	0.0012	54	56
PbS_2	486	0.0012	44	74
$AgCuTe_2$	196	0.0013	16	25
$Br_2N_2Ti_2$	234	0.0013	15	24
Br_2CsF	550	0.0013	55	55
$AgClO_2$	656	0.0013	54	83
InSe	584	0.0013	67	91
Br_2Zr_2	724	0.0013	52	103
AsSb	458	0.0013	49	82
As_2Li_2Pr	857	0.0013	67	91
Br_2Y_2	560	0.0013	44	74
$AgClO_2$	584	0.0013	48	74
$I_2La_2Si_2$	948	0.0013	67	91
Cu_2Sr_2	784	0.0014	70	91
FKO_2Se	795	0.0014	80	63
S_2Sn	345	0.0014	30	55
As_4	980	0.0014	90	110
As_2Fe_2	180	0.0014	14	24
Ga_2Se_2	560	0.0015	44	74
Bi_2STe_2	857	0.0015	67	91
Cl_2OOs	754	0.0015	61	97
Fe_2O_4	894	0.0015	49	100
As_2Li_2Nd	884	0.0015	69	94
Bi_2STe_2	765	0.0016	60	81
$C_2Cl_2Y_2$	558	0.0016	35	58
$Au_2K_2Se_2$	204	0.0016	24	10
AlH_4Na	660	0.0016	56	54
$LiMnTe_2$	684	0.0016	60	81
CdI_2	603	0.0016	60	81

Table showing the first 50 lattice matching combinations with the smallest strain needed to build the supercell.

$Yb_2S_2I_2$ (Pmm2)

Structural and electronic properties

**Formula**	$Yb_2S_2I_2$
**Spacegroup**	Pmm2
**Prototype**	FeOCl
**Parent 3D**	$I_2S_2Yb_2$
**Source DB**	MPDS
**DB ID**	S1937398
**DF2-C09 Binding energy [meV/ Å^2]**	12.24
**RVV10 Binding energy [meV/ Å^2]**	N/A
**Band gap (PBE) [eV]**	0.0

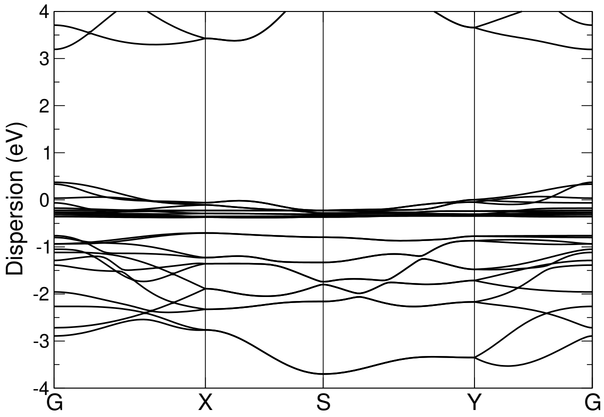

Band structure: Electronic band structure of $Yb_2S_2I_2$ (Pmm2) in a representative window around the Fermi energy and along a high-symmetry path.

Optimized crystal structure

Structural parameters: relaxed cell (top) and atomic positions (bottom) of $Yb_2S_2I_2$ (Pmm2) in Cartesian coordinates.

		x [Å]	y [Å]	z [Å]
$\mathbf{a_1}$		4.13292145	0.00000000	0.00000000
$\mathbf{a_2}$		0.00000000	5.30154726	0.00000000
$\mathbf{a_3}$		0.00000000	0.00000000	24.91024729
		x [Å]	y [Å]	z [Å]
●	Yb	1.03331314	-1.32538682	-1.09399766
●	S	1.03319870	-3.97616045	-0.67492259
●	I	-1.03310619	-1.32538682	-3.31223708
●	Yb	-1.03331314	-3.97616045	1.09399766
●	S	-1.03319870	-1.32538682	0.67492259
●	I	1.03310619	-3.97616045	3.31223708

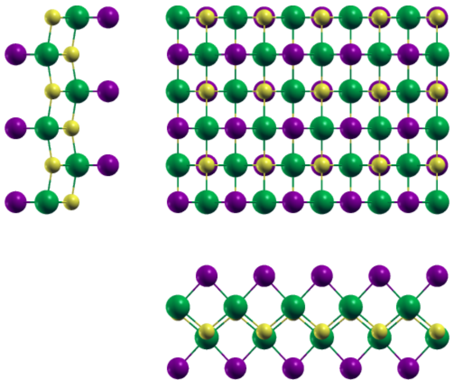

Orthographic projections: views of $Yb_2S_2I_2$ (Pmm2) as seen from the x axis (left), the y axis (bottom) and the z axis (center).

Lattice matching - minimal size

Formula	N° atoms	strain	cell size 1	cell size 2
In	8	0.1458	1	2
Gd	8	0.1324	1	2
HgI_2	9	0.315	1	1
$RhTe_2$	9	0.1168	1	1
Ba_2Hg	9	0.2645	1	1
CNRb	9	0.2215	1	1
CKN	9	0.0111	1	1
CNNa	9	0.2982	1	1
Ba_2Cd	9	0.2682	1	1
Bi_2In_2	10	0.0582	1	1
Cu_2I_2	10	0.2502	1	1
CdClHO	10	0.117	1	1
Au_2Br_2	10	0.8022	1	1
O_2Pb_2	10	0.7342	1	1
As_4	10	0.1575	1	1
Br_3Cs	10	0.3889	1	1
$AgClO_2$	10	0.1175	1	1
Br_2CsF	10	0.0576	1	1
Gd	10	0.0399	1	4
Sn_2Te_2	10	0.3315	1	1
F_4Sn	11	0.2633	1	1
FKO_2Se	11	0.5559	1	1
F_4Nb	11	0.2553	1	1
$ClKO_3$	11	0.3969	1	1
Cl_4Mn	11	0.0601	1	1
$Ba_2H_2I_2$	12	0.3371	1	1
CrS_2	12	0.1377	1	2
$Br_2Ho_2S_2$	12	0.0043	1	1
S_2V	12	0.1479	1	2
MoS_2	12	0.1484	1	2
$I_2Lu_2Se_2$	12	0.0536	1	1
$Br_2F_2Sr_2$	12	0.2529	1	1
$Ho_2I_2S_2$	12	0.005	1	1
Cu_4Te_2	12	0.1151	1	1
AlH_4Na	12	0.0612	1	1
$GeTe_2$	12	0.6492	1	2
$F_2I_2Sm_2$	12	0.7336	1	1
Br_2Ca_3Si	12	0.2648	1	1
$I_2N_2Zr_2$	12	0.1166	1	1
$Br_2S_2Y_2$	12	0.0076	1	1
S_2W	12	0.1484	1	2
$Gd_2I_2S_2$	12	0.0103	1	1
$C_2Br_2Gd_2$	12	0.1077	1	1
$Br_2F_2Pb_2$	12	0.2525	1	1
$Br_2Er_2Se_2$	12	0.0183	1	1
NbS_2	12	0.3865	1	2
S_2Ta	12	0.3847	1	2
$I_2S_2Tb_2$	12	0.0081	1	1
Te_4TiZr	12	0.6495	1	1
$Cu_2Rb_2Te_2$	12	0.0581	1	1

Table showing the first 50 lattice matching combinations with the smallest number of atoms in the supercell.

Lattice matching - minimal strain

Formula	N° atoms	strain	cell size 1	cell size 2
S_2Sn	279	0.0	24	45
S_2Zr	279	0.0002	24	45
Se_4TiZr	558	0.0003	48	45
$NbTe_2$	279	0.0005	24	45
Cl_4Cu_2	198	0.0005	21	12
PSn_2	279	0.0005	24	45
Cl_2OOs	614	0.0005	49	80
Cu_2F_4	96	0.0005	9	7
Bi_2Pd	240	0.0006	20	40
AlH_4Na	762	0.0006	64	63
$Br_2H_2Zr_2$	846	0.0006	45	96
F_4Nb	87	0.0007	7	9
Cl_2Zn	678	0.0007	63	100
Sn	213	0.001	25	63
Cl_2Zn	657	0.001	61	97
$Ca_2Cl_2H_2$	978	0.0011	63	100
ClNZr	585	0.0011	47	101
$Ca_2Cl_2H_2$	948	0.0011	61	97
Bi_2In_2	634	0.0011	63	64
MnO_2	288	0.0011	18	60
CrO_2	288	0.0011	18	60
$I_2S_2Tm_2$	12	0.0012	1	1
Gd	48	0.0012	5	18
$I_2N_2Ti_2$	276	0.0013	18	28
I_2La_2Sb	360	0.0013	30	36
$As_2Cd_2K_2$	804	0.0013	65	69
Bi_2Se_2	622	0.0013	57	70
$I_2N_2Zr_2$	972	0.0014	67	95
$Cu_2Rb_2Te_2$	762	0.0014	63	64
Hf_3Te_2	851	0.0014	61	97
$Br_2N_2Zr_2$	726	0.0015	49	72
Fe_2Se_2	876	0.0015	70	114
BrNZr	564	0.0016	46	96
$Se_2Si_2Zr_2$	198	0.0016	12	21
CCl_2Gd_2	852	0.0016	57	102
Br_2Zn	648	0.0016	57	102
$Cl_2Rh_2Te_2$	456	0.0016	40	36
$C_2Br_2La_2$	714	0.0016	49	70
K	216	0.0016	30	36
Te_2Ti	648	0.0016	57	102
O_4PSn	804	0.0016	65	69
$HfSe_2$	648	0.0016	57	102
CBr_2Y_2	852	0.0016	57	102
Ca_2O_2	280	0.0017	20	40
$Br_2In_2O_2$	780	0.0017	53	77
CBr_2Lu_2	369	0.0017	24	45
$I_2Nd_2S_2$	396	0.0017	30	36
$Br_2N_2Zr_2$	414	0.0017	24	45
CCl_2Sc_2	787	0.0017	47	101
P_4	328	0.0018	28	40

Table showing the first 50 lattice matching combinations with the smallest strain needed to build the supercell.

$Yb_2Se_2F_2$ (P-3m1)

Structural and electronic properties

**Formula**	$Yb_2Se_2F_2$
**Spacegroup**	P-3m1
**Prototype**	SmSI
**Parent 3D**	$F_2Se_2Yb_2$
**Source DB**	MPDS
**DB ID**	S307727
**DF2-C09 Binding energy [meV/ Å^2]**	15.47
**RVV10 Binding energy [meV/ Å^2]**	N/A
**Band gap (PBE) [eV]**	0.0

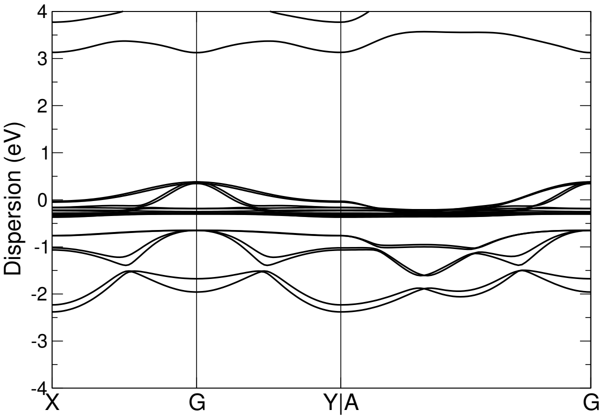

Band structure: Electronic band structure of $Yb_2Se_2F_2$ (P-3m1) in a representative window around the Fermi energy and along a high-symmetry path.

Optimized crystal structure

Structural parameters: relaxed cell (top) and atomic positions (bottom) of $Yb_2Se_2F_2$ (P-3m1) in Cartesian coordinates.

	x [Å]	y [Å]	z [Å]
$\mathbf{a_1}$	1.95774857	−3.39092000	0.00000000
$\mathbf{a_2}$	1.95746226	3.39075470	0.00000000
$\mathbf{a_3}$	0.00000000	0.00000000	25.27105808

		x [Å]	y [Å]	z [Å]
●	Yb	0.97870518	0.56505570	-1.70021466
●	Se	0.97889549	-1.69544776	-3.34536815
•	F	2.93639240	-0.56528639	-0.72330901
●	Yb	2.93650565	-0.56522100	1.70021466
●	Se	0.97885309	-1.69547224	3.34536815
•	F	0.97881844	0.56512109	0.72330901

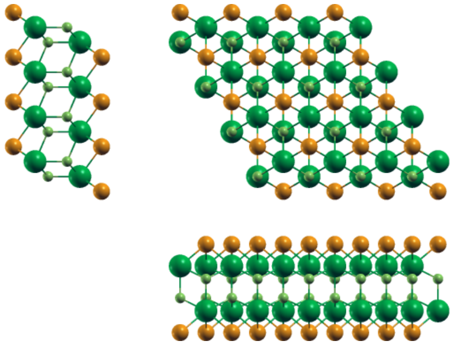

Orthographic projections: views of $Yb_2Se_2F_2$ (P-3m1) as seen from the x axis (left), the y axis (bottom) and the z axis (center).

Lattice matching - minimal size

Formula	N° atoms	strain	cell size 1	cell size 2
Na	7	0.2723	1	1
AsSb	8	0.0085	1	1
GeTe	8	0.0026	1	1
S_2	8	0.0011	1	1
CaCl	8	0.1362	1	1
$IrTe_2$	9	0.0017	1	1
$CdCl_2$	9	0.0037	1	1
$MoTe_2$	9	0.2757	1	1
$ReSe_2$	9	0.2549	1	1
CaI_2	9	0.499	1	1
$InSe_2$	9	0.0033	1	1
$GeTe_2$	9	0.0047	1	1
$HfTe_2$	9	0.0067	1	1
I_2Mn	9	0.0036	1	1
NSr_2	9	0.007	1	1
LiO_2	9	0.0685	1	1
Cl_2Zn	9	0.1488	1	1
FeI_2	9	0.0057	1	1
I_2Ni	9	0.0045	1	1
S_2Ti	9	0.2468	1	1
CrI_2	9	0.0061	1	1
Te_2Zn	9	0.2754	1	1
BiBrTe	9	0.4558	1	1
Bi_2Pd	9	0.113	1	1
Cl_2Ni	9	0.2561	1	1
Cl_2Co	9	0.2464	1	1
$CrTe_2$	9	0.2646	1	1
PtS_2	9	0.2739	1	1
Br_2V	9	0.2538	1	1
Ba_2N	9	0.0082	1	1
Se_2Ti	9	0.27	1	1
Br_2Ti	9	0.2638	1	1
Te_2Zr	9	0.0072	1	1
Te_2W	9	0.276	1	1
$AsSe_2$	9	0.2581	1	1
I_2Tm	9	0.4959	1	1
BiITe	9	0.4721	1	1
CdO_2	9	0.2462	1	1
BrNZr	9	0.2599	1	1
$NbSe_2$	9	0.2567	1	1
CoI_2	9	0.0081	1	1
GeS_2	9	0.1273	1	1
O_2Zn	9	4.8637	1	1
$MnSe_2$	9	0.1361	1	1
Br_2Cr	9	0.2641	1	1
DyI_2	9	2.9238	1	1
Se_2Ta	9	0.2568	1	1
Br_2Mg	9	0.0059	1	1
I_2Ti	9	0.0078	1	1
$NbSe_2$	9	0.2585	1	1

Table showing the first 50 lattice matching combinations with the smallest number of atoms in the supercell.

Lattice matching - minimal strain

Formula	N° atoms	strain	cell size 1	cell size 2
Br_2Hf_2	708	0.0	64	81
$Dy_2I_2S_2$	600	0.0	57	43
GeI_2La_2	629	0.0001	64	49
Cl_2Sc_2	802	0.0001	73	91
Br_2Cr	711	0.0001	73	91
P_2Sn_2	10	0.0001	1	1
$CrTe_2$	711	0.0002	73	91
I_2Tm	531	0.0002	64	49
$Br_2H_2Zr_2$	984	0.0002	73	91
S_2Zn_2	10	0.0002	1	1
$Ge_2I_2La_2$	780	0.0004	73	57
Br_2Ti	711	0.0004	73	91
BrNZr	627	0.0004	64	81
CrO_2	471	0.0004	39	79
Cl_2Ni	561	0.0004	57	73
$ReSe_2$	561	0.0005	57	73
$I_2S_2Sm_2$	510	0.0005	49	36
Cl_2Fe	429	0.0005	43	57
O_2Zn	537	0.0005	49	81
$NbSe_2$	627	0.0006	64	81
Sm	33	0.0007	4	9
GdI_2	843	0.0007	100	81
CNRb	603	0.0007	78	45
DyI_2	471	0.0007	57	43
$LiOS_2Ti$	461	0.0007	36	49
Cl_2Mn	363	0.0008	36	49
K	330	0.0008	49	36
C_2F_2	60	0.0008	4	9
$NbSe_2$	561	0.0009	57	73
$AsSe_2$	627	0.0009	64	81
Ni_2SbTe_2	11	0.0009	1	1
Cl_2Zr	429	0.0009	43	57
$AlLiTe_2$	838	0.0009	91	73
$Cl_2H_2Sc_2$	984	0.0009	73	91
Se_2Ta	561	0.001	57	73
PbS_2Sn	844	0.0011	94	70
S_2	8	0.0011	1	1
$Cl_2N_2Ti_2$	426	0.0011	35	36
CdO_2	429	0.0011	43	57
Cl_2Gd_2	10	0.0011	1	1
I_2Yb	531	0.0011	64	49
$I_2S_2Tb_2$	600	0.0012	57	43
Cu_2Sr_2	924	0.0012	100	81
Se_2Ta	711	0.0012	73	91
O_2Zn	405	0.0012	37	61
I_2La_2Sb	474	0.0012	49	36
LiO_2	129	0.0012	13	17
Br_2V	561	0.0013	57	73
Cl_2Co	429	0.0013	43	57
ClNZr	486	0.0013	49	64

Table showing the first 50 lattice matching combinations with the smallest strain needed to build the supercell.

$Yb_2Se_2I_2$ (Pmm2)

Structural and electronic properties

**Formula**	$Yb_2Se_2I_2$
**Spacegroup**	Pmm2
**Prototype**	FeOCl
**Parent 3D**	$I_2Se_2Yb_2$
**Source DB**	MPDS
**DB ID**	S376101
**DF2-C09 Binding energy [meV/ Å^2]**	12.39
**RVV10 Binding energy [meV/ Å^2]**	N/A
**Band gap (PBE) [eV]**	0.0

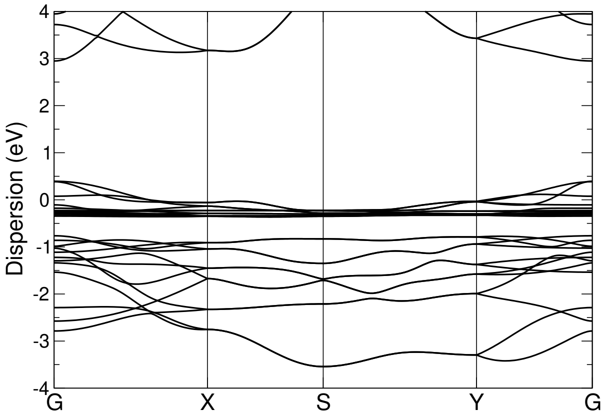

Band structure: Electronic band structure of $Yb_2Se_2I_2$ (Pmm2) in a representative window around the Fermi energy and along a high-symmetry path.

Optimized crystal structure

Structural parameters: relaxed cell (top) and atomic positions (bottom) of $Yb_2Se_2I_2$ (Pmm2) in Cartesian coordinates.

	x [Å]	y [Å]	z [Å]
$\mathbf{a_1}$	4.21844318	0.00000000	0.00000000
$\mathbf{a_2}$	0.00000000	5.58636196	0.00000000
$\mathbf{a_3}$	0.00000000	0.00000000	24.92440215

		x [Å]	y [Å]	z [Å]
●	I	1.05441420	-4.18977147	-3.31585126
●	Yb	-1.05476347	-4.18977147	-1.15265212
●	Yb	1.05476347	-1.39659049	1.15265212
●	Se	-1.05460480	-1.39659049	-0.79095846
●	Se	1.05460480	-4.18977147	0.79095846
●	I	-1.05441420	-1.39659049	3.31585126

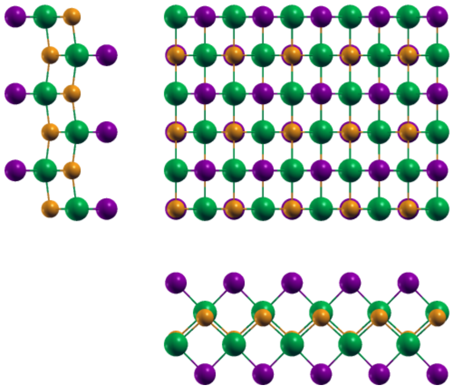

Orthographic projections: views of $Yb_2Se_2I_2$ (Pmm2) as seen from the x axis (left), the y axis (bottom) and the z axis (center).

Lattice matching - minimal size

Formula	N° atoms	strain	cell size 1	cell size 2
K	8	0.3514	1	2
Tl	8	0.1447	1	2
CKN	9	0.0264	1	1
In_2Se_2	10	0.0866	1	1
Ge_2Te_2	10	0.8396	1	1
As_4	10	0.6239	1	1
Au_2Se_2	10	0.2243	1	1
LiO	10	0.1373	1	2
Au_2I_2	10	0.7814	1	1
P_2	10	0.1415	1	2
$AgClO_2$	10	0.1186	1	1
La_2S_2	10	0.2718	1	1
$SbSe_2Tl$	10	0.1744	1	1
Se_2Sn_2	10	0.2742	1	1
F_4Pb	11	0.2838	1	1
H_2Li_2Pt	11	0.3469	1	1
$CuGeO_3$	11	0.1285	1	1
KNO_3	11	0.3277	1	1
S_2V	12	0.1337	1	2
MoS_2	12	0.134	1	2
$MoSe_2$	12	0.1442	1	2
$Ho_2I_2S_2$	12	0.0163	1	1
$CuTe_2$	12	0.5139	1	2
PbS_2	12	0.5846	1	2
Cl_2Ti	12	0.1416	1	2
$K_2O_2Tl_2$	12	0.2161	1	1
S_2Ti	12	0.3602	1	2
$I_2Se_2Tb_2$	12	0.0071	1	1
$Gd_2I_2Se_2$	12	0.0092	1	1
S_2W	12	0.1341	1	2
$Gd_2I_2S_2$	12	0.015	1	1
Cl_2Co	12	0.3595	1	2
Pd_2S_4	12	0.3562	1	1
$Br_2Er_2Se_2$	12	0.0074	1	1
NbS_2	12	0.3418	1	2
CNRb	12	0.529	1	2
Cl_2Fe	12	0.3581	1	2
S_2Ta	12	0.1461	1	2
Se_2V	12	0.1453	1	2
$I_2S_2Tb_2$	12	0.0154	1	1
$Er_2I_2Se_2$	12	0.0025	1	1
$I_2Se_2Tm_2$	12	0.0009	1	1
Cl_2V	12	0.136	1	2
Ca_4Cu_2	12	0.0219	1	1
Cl_2Zr	12	0.3589	1	2
$Dy_2I_2S_2$	12	0.0156	1	1
MoS_2	12	0.1341	1	2
Se_4TiZr	12	0.2332	1	1
$CrSe_2$	12	0.1354	1	2
Te_4W_2	12	0.5167	1	1

Table showing the first 50 lattice matching combinations with the smallest number of atoms in the supercell.

Lattice matching - minimal strain

Formula	N° atoms	strain	cell size 1	cell size 2
$I_2Nd_2S_2$	822	0.0004	60	77
CNRb	636	0.0005	70	72
Cl_2OOs	156	0.0007	12	21
CBr_2Y_2	876	0.0008	56	108
FeO_2	228	0.0008	14	48
$Cl_2Fe_2O_2$	108	0.0009	6	12
$I_2Se_2Tm_2$	12	0.0009	1	1
CCl_2Gd_2	876	0.001	56	108
$CuTe_2$	660	0.001	54	112
Au_2Se_2	720	0.001	72	72
$Dy_2I_2S_2$	744	0.0011	53	71
CaI_2	603	0.0012	60	81
$AuTe_2$	594	0.0013	54	90
As_4	888	0.0013	78	105
As_2Sn_2	684	0.0014	54	90
DyI_2	531	0.0014	53	71
Hg_3S_2	528	0.0014	63	30
Ni_2Te_2	476	0.0014	36	65
$CdCl_2$	411	0.0015	36	65
$C_2Br_2Y_2$	894	0.0015	54	95
$PtTe_2$	594	0.0015	54	90
I_2Mn	411	0.0015	36	65
LiO	112	0.0015	10	26
I_2Ni	411	0.0015	36	65
$Cl_2Er_2H_2$	606	0.0015	36	65
Bi_2In_2	560	0.0016	54	59
$InSe_2$	411	0.0016	36	65
$GeTe_2$	411	0.0016	36	65
H_2Na_2Pd	390	0.0016	25	48
$As_2Co_2Li_2$	258	0.0016	16	27
$Ag_2K_2Te_2$	648	0.0016	53	55
Ba_2Cd	30	0.0016	3	4
$F_2I_2Pb_2$	42	0.0017	3	4
CdClO	681	0.0017	55	117
$I_2S_2Tb_2$	744	0.0017	53	71
OTl_2	681	0.0017	55	117
$Ge_2Hf_2Te_2$	258	0.0017	16	27
Cu_2Te_2	204	0.0017	16	27
Te_2V	681	0.0017	55	117
CaI_2	552	0.0017	55	74
H_2Si_2	476	0.0017	36	65
GeS_2	294	0.0018	25	48
I_2Tm	603	0.0018	60	81
$TaTe_2$	672	0.0018	56	112
CNRb	573	0.0018	63	65
$AgClO_2$	664	0.0018	52	88
$AgNO_3$	709	0.0018	64	65
Cl_2La_2	708	0.0018	56	93
CdH_2O_2	915	0.0019	55	117
Se_4TiZr	672	0.0019	56	56

Table showing the first 50 lattice matching combinations with the smallest strain needed to build the supercell.

YbI_2 (P-3m1)

Structural and electronic properties

**Formula**	YbI_2
**Spacegroup**	P-3m1
**Prototype**	CdI2
**Parent 3D**	YbI_2
**Source DB**	ICSD
**DB ID**	77907
**DF2-C09 Binding energy [meV/ Å^2]**	10.6
**RVV10 Binding energy [meV/ Å^2]**	16.14
**Band gap (PBE) [eV]**	0.75

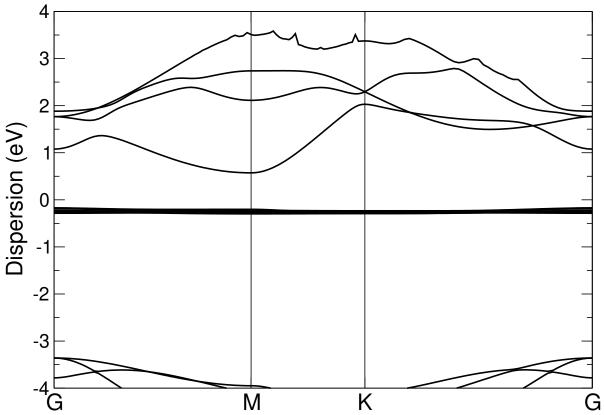

Band structure: Electronic band structure of YbI_2 (P-3m1) in a representative window around the Fermi energy and along a high-symmetry path.

Optimized crystal structure

Structural parameters: relaxed cell (top) and atomic positions (bottom) of YbI_2 (P-3m1) in Cartesian coordinates.

	x [Å]	y [Å]	z [Å]
$\mathbf{a_1}$	4.46378998	0.00000000	0.00000000
$\mathbf{a_2}$	−2.23189499	3.86575552	0.00000000
$\mathbf{a_3}$	0.00000000	0.00000000	23.59718966

	x [Å]	y [Å]	z [Å]
I	0.00000000	2.57717035	10.00926835
Yb	0.00000000	0.00000000	11.79859483
I	2.23189499	1.28858517	13.58792131

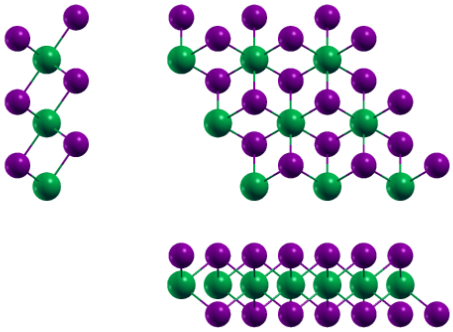

Orthographic projections: views of YbI_2 (P-3m1) as seen from the x axis (left), the y axis (bottom) and the z axis (center).

Lattice matching - minimal size

Formula	N° atoms	strain	cell size 1	cell size 2
GeTe	5	0.2473	1	1
S_2	5	0.2493	1	1
In	5	0.223	1	2
$IrTe_2$	6	0.2485	1	1
$CdCl_2$	6	1.5991	1	1
CaI_2	6	0.0025	1	1
$InSe_2$	6	0.2464	1	1
$HfTe_2$	6	0.2597	1	1
I_2Pr	6	0.1204	1	1
I_2Mn	6	1.6	1	1
Br_2Cu	6	0.6373	1	1
Tl	6	0.622	1	3
$AuTe_2$	6	0.2708	1	1
$PdTe_2$	6	0.2672	1	1
FeI_2	6	1.5837	1	1
I_2Ni	6	1.5931	1	1
Ba_2Hg	6	0.1437	1	1
Cl_2Ni	6	4.8496	1	1
Ba_2N	6	0.2618	1	1
Te_2Zr	6	0.2604	1	1
I_2Nd	6	0.121	1	1
I_2Tm	6	0.0014	1	1
I_2V	6	1.5284	1	1
Se_2Zr	6	1.5218	1	1
$PtSe_2$	6	1.4473	1	1
BiITe	6	0.0077	1	1
$NbSe_2$	6	4.8586	1	1
CoI_2	6	1.5657	1	1
DyI_2	6	0.0054	1	1
CeI_2	6	0.12	1	1
Se_2Ta	6	4.8606	1	1
Br_2Mg	6	1.5827	1	1
F_2Ni	6	0.4302	1	1
$PtTe_2$	6	0.2702	1	1
Br_2Cd	6	0.2659	1	1
I_2La	6	0.1237	1	1
F_2Zn	6	0.1183	1	1
Ba_2Cd	6	0.1461	1	1
NaPSn	6	0.2585	1	1
H_2Si_2	7	0.2468	1	1
Fe_2Te_2	7	0.1143	1	1
Li_2Tl_2	7	0.4831	1	1
Ca_2Cl_2	7	0.1144	1	1
Cu_2I_2	7	0.1351	1	1
Cl_2OOs	7	0.1088	1	1
Ir_2P_2	7	0.1208	1	1
Ag_2Br_2	7	0.1241	1	1
Br_2Er_2	7	0.2716	1	1
O_2Sn_2	7	0.4504	1	1
Cu_2S_2	7	0.1168	1	1

Table showing the first 50 lattice matching combinations with the smallest number of atoms in the supercell.

Lattice matching - minimal strain

Formula	N° atoms	strain	cell size 1	cell size 2
MoS_2	222	0.0	25	49
Li_2Tl_2	447	0.0	73	57
S_2	275	0.0001	49	64
$Er_2F_2Se_2$	678	0.0001	64	81
$GeTe_2$	300	0.0001	43	57
I_2Ni	300	0.0001	43	57
$NbSe_2$	294	0.0001	37	61
$F_2Ho_2Se_2$	765	0.0001	73	91
CdH_2O_2	173	0.0002	16	25
Ba_2Cu_2	7	0.0002	1	1
S_2W	222	0.0002	25	49
Ni_2SbTe_2	467	0.0002	49	64
$PdTe_2$	543	0.0002	81	100
MoS_2	222	0.0002	25	49
$AsSe_2$	390	0.0003	49	81
$HfTe_2$	435	0.0003	64	81
In_2Se_3	597	0.0003	64	81
Br_2Tb_2	643	0.0003	81	100
$AsSe_2$	294	0.0004	37	61
$F_2Se_2Tm_2$	609	0.0004	57	73
Ga_2Se_2	403	0.0004	49	64
$LiOS_2Ti$	107	0.0004	9	16
CdClHO	219	0.0004	25	36
Pt_2Te_2	516	0.0004	64	81
PbS_2	255	0.0004	36	49
$Cl_2H_2Zr_2$	123	0.0005	9	16
$LiMnSe_2$	463	0.0005	57	73
Br_2Hf_2	355	0.0005	37	61
$HNiO_2$	48	0.0006	4	9
Te_2W	123	0.0006	16	25
Bi_2S_3	743	0.0006	81	100
NaPSn	435	0.0006	64	81
$IrTe_2$	339	0.0006	49	64
Br_2Y_2	304	0.0007	36	49
Se_2Ta	390	0.0007	49	81
Ge_2Te_2	442	0.0007	66	61
CdClO	123	0.0007	16	25
Cl_2Rb_2	606	0.0007	130	54
Ni_2Te_2	357	0.0007	43	57
NbS_2	75	0.0007	9	16
$MoTe_2$	123	0.0007	16	25
$NbSe_2$	390	0.0008	49	81
Te_2Zr	435	0.0008	64	81
GeI_3Rb	323	0.0008	81	16
O_2Sn_2	709	0.0008	83	115
Br_2Ho_2	643	0.0008	81	100
OTl_2	123	0.0009	16	25
$Cl_2Hf_2N_2$	198	0.0009	16	25
$CdCl_2$	300	0.0009	43	57
Te_2Zn	123	0.0009	16	25

Table showing the first 50 lattice matching combinations with the smallest strain needed to build the supercell.

YbOBr (Cmme)

Structural and electronic properties

**Formula**	YbOBr
**Spacegroup**	Cmme
**Prototype**	PbClF
**Parent 3D**	$Yb_2O_2Br_2$
**Source DB**	ICSD
**DB ID**	28532
**DF2-C09 Binding energy [meV/ Å^2]**	15.19
**RVV10 Binding energy [meV/ Å^2]**	22.78
**Band gap (PBE) [eV]**	N/A

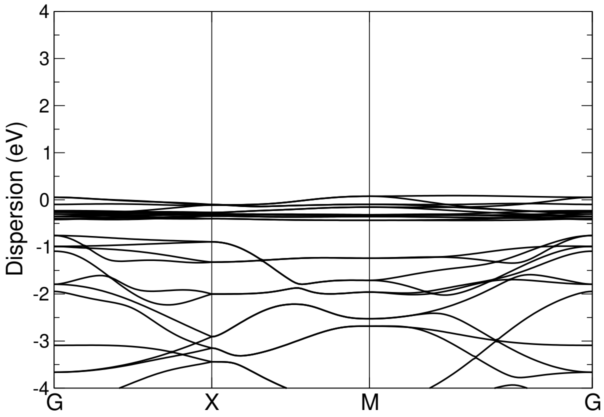

Band structure: Electronic band structure of YbOBr (Cmme) in a representative window around the Fermi energy and along a high-symmetry path.

Optimized crystal structure

Structural parameters: relaxed cell (top) and atomic positions (bottom) of YbOBr (Cmme) in Cartesian coordinates.

	x [Å]	y [Å]	z [Å]
$\mathbf{a_1}$	3.79863528	0.00000000	0.00000000
$\mathbf{a_2}$	0.00000000	3.79863528	0.00000000
$\mathbf{a_3}$	0.00000000	0.00000000	25.59292747

		x [Å]	y [Å]	z [Å]
●	Yb	0.00000000	1.89931764	13.95511897
●	Br	1.89931764	0.00000000	15.62134288
●	Yb	1.89931764	0.00000000	11.63780851
●	Br	0.00000000	1.89931764	9.97158460
•	O	1.89931764	1.89931764	12.79646374
•	O	0.00000000	0.00000000	12.79646374

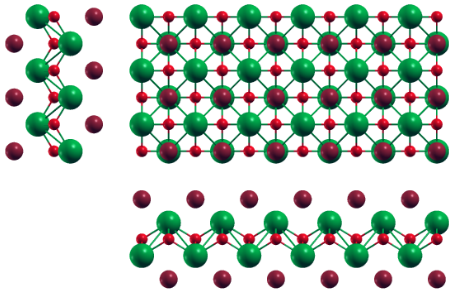

Orthographic projections: views of YbOBr (Cmme) as seen from the x axis (left), the y axis (bottom) and the z axis (center).

Lattice matching - minimal size

Formula	N° atoms	strain	cell size 1	cell size 2
K	7	0.4059	1	1
In	7	0.1092	1	1
InSe	8	0.1584	1	1
HgO	8	0.5613	1	1
Bi_2	8	0.1646	1	1
Ag_2	8	0.7836	1	1
As_2	8	0.1088	1	1
PbTe	8	0.1603	1	1
Sb_2	8	0.1431	1	1
Cl_2Zn	9	0.1086	1	1
I_2Mg	9	0.1483	1	1
CdI_2	9	0.1621	1	1
Ba_2Pt	9	0.7825	1	1
Br_2Ca	9	0.1635	1	1
CaI_2	9	0.1891	1	1
$HfTe_2$	9	0.1306	1	1
Br_2La	9	0.1486	1	1
Br_2Cu	9	0.1124	1	1
I_2Yb	9	0.1859	1	1
Br_2Co	9	0.1087	1	1
BiClTe	9	0.1625	1	1
ReS_2	9	0.1111	1	1
Ca_2N	9	0.1089	1	1
$AuTe_2$	9	0.1365	1	1
BrCdI	9	0.1512	1	1
$PdTe_2$	9	0.1346	1	1
I_2Zn	9	0.1403	1	1
BaF_2	9	0.1545	1	1
BiBrTe	9	0.1686	1	1
Bi_2Pd	9	0.5897	1	1
GeI_2	9	0.1465	1	1
Ba_2N	9	0.1317	1	1
AsKSn	9	0.1531	1	1
Te_2Zr	9	0.1309	1	1
$PbTe_2$	9	0.1503	1	1
Cl_2Cu	9	0.1022	1	1
I_2Tm	9	0.1876	1	1
$SnTe_2$	9	0.1445	1	1
GeI_2	9	0.1605	1	1
I_2Pb	9	0.7897	1	1
STl_2	9	0.1555	1	1
BiITe	9	0.1762	1	1
Br_2Fe	9	0.1087	1	1
DyI_2	9	0.1928	1	1
Se_2Yb	9	0.1467	1	1
$BiTe_2$	9	0.147	1	1
GdI_2	9	0.1719	1	1
$PtTe_2$	9	0.1362	1	1
Br_2Cd	9	0.1338	1	1
O_2Pt	9	0.1095	1	1

Table showing the first 50 lattice matching combinations with the smallest number of atoms in the supercell.

Lattice matching - minimal strain

Formula	N° atoms	strain	cell size 1	cell size 2
Bi_2In_2	530	0.0001	61	41
Sn	375	0.0001	49	81
Cl_2Sc_2	548	0.0002	48	65
$CrTe_2$	483	0.0002	48	65
Hg_3S_2	768	0.0002	103	30
Se_2Ta_4	882	0.0003	65	82
Br_2Cr	483	0.0003	48	65
Ag_2I_2	754	0.0003	85	61
Tl	186	0.0003	25	36
$Br_2H_2Zr_2$	678	0.0004	48	65
Ca_2O_2	550	0.0004	49	64
Fe_2Te_2	10	0.0004	1	1
Ho_2S_2	874	0.0004	89	85
Br_2Ti	483	0.0006	48	65
$Dy_2I_2S_2$	690	0.0006	70	45
O_2Zn	411	0.0006	36	65
Mg_4	294	0.0007	25	36
HgI_2	693	0.0007	85	61
$FeSe_2$	945	0.0007	85	145
H_2MnO_2	315	0.0008	20	39
$I_2S_2Tb_2$	690	0.0008	70	45
Ca_2Cl_2	10	0.0009	1	1
$LiNbS_2$	958	0.0009	81	118
$Cu_2K_2Te_2$	366	0.0009	36	25
O_4PSn	366	0.0009	36	25
$Cu_2Na_2Te_2$	678	0.0009	64	49
S_2Ta	840	0.001	81	118
Cu_2F_4	444	0.001	49	25
$Ho_2I_2S_2$	732	0.001	74	48
LiO	726	0.001	79	126
$CuGeO_3$	979	0.0011	94	83
As_2Ru_2	10	0.0011	1	1
Se_2Ta_4	870	0.0011	64	81
Ba_2Cd	852	0.0011	101	82
In	496	0.0011	65	106
$Cl_2H_2Sc_2$	678	0.0011	48	65
Au_2Br_2	742	0.0012	81	64
$Cl_2Hg_2N_2$	726	0.0013	81	40
Hg_3S_2	664	0.0013	89	26
$Br_2O_2Tm_2$	12	0.0013	1	1
Se_2Ta	483	0.0013	48	65
NbS_2	840	0.0014	81	118
Hg_3S_2	589	0.0014	79	23
BiITe	885	0.0014	103	89
Bi_2Se_2	602	0.0014	65	53
K	440	0.0015	65	50
HgO	314	0.0015	36	49
Cl_2Zr_2	604	0.0015	52	73
Bi_2Te_3	919	0.0016	89	77
BiITe	765	0.0016	89	77

Table showing the first 50 lattice matching combinations with the smallest strain needed to build the supercell.

YbOCl (P-3m1)

Structural and electronic properties

**Formula**	YbOCl
**Spacegroup**	P-3m1
**Prototype**	SmSI
**Parent 3D**	$Yb_2O_2Cl_2$
**Source DB**	ICSD
**DB ID**	6077
**DF2-C09 Binding energy [meV/ Å^2]**	11.71
**RVV10 Binding energy [meV/ Å^2]**	18.77
**Band gap (PBE) [eV]**	0.01

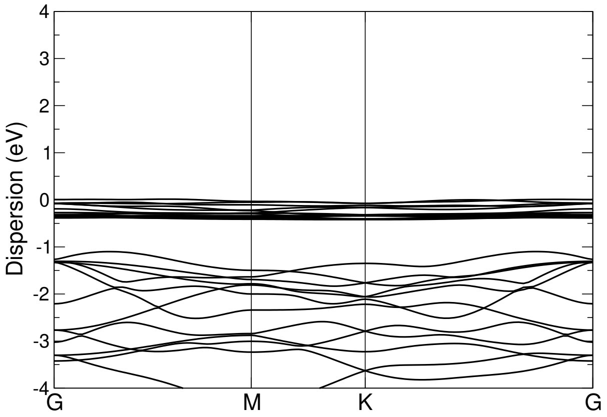

Band structure: Electronic band structure of YbOCl (P-3m1) in a representative window around the Fermi energy and along a high-symmetry path.

Optimized crystal structure

Structural parameters: relaxed cell (top) and atomic positions (bottom) of YbOCl (P-3m1) in Cartesian coordinates.

	x [Å]	y [Å]	z [Å]
$\mathbf{a_1}$	3.77665638	−0.00000000	0.00000000
$\mathbf{a_2}$	−1.88832819	3.27068037	0.00000000
$\mathbf{a_3}$	0.00000000	0.00000000	26.32537018

		x [Å]	y [Å]	z [Å]
●	Cl	1.88832819	1.09022679	10.01742467
●	Yb	0.00000000	2.18045358	11.73621646
●	O	0.00000000	2.18045358	14.00719212
●	Yb	0.00000000	0.00000000	14.58915372
●	O	0.00000000	-0.00000000	12.31817806
●	Cl	1.88832819	1.09022679	16.30794551

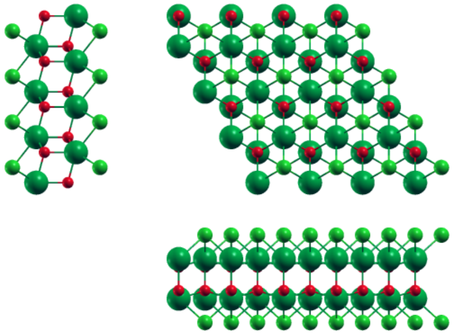

Orthographic projections: views of YbOCl (P-3m1) as seen from the x axis (left), the y axis (bottom) and the z axis (center).

Lattice matching - minimal size

Formula	N° atoms	strain	cell size 1	cell size 2
Tl	7	0.1113	1	1
Tl	7	1.636	1	1
InSe	8	0.475	1	1
HgO	8	0.1161	1	1
AsSb	8	0.0084	1	1
Bi_2	8	2.8431	1	1
P_2	8	1.5967	1	1
PbTe	8	0.4795	1	1
CaCl	8	0.1512	1	1
I_2Mg	9	0.4493	1	1
Cl_2Mn	9	0.2602	1	1
CdI_2	9	0.484	1	1
$AgTe_2$	9	0.1123	1	1
$MoSe_2$	9	1.6303	1	1
S_2Ta	9	0.2631	1	1
Br_2Zn	9	0.0019	1	1
Br_2Ca	9	0.4872	1	1
$AsSn_2$	9	0.0086	1	1
$SiTe_2$	9	0.0022	1	1
Br_2La	9	0.4501	1	1
PbS_2	9	0.0058	1	1
BiClTe	9	0.4849	1	1
Cl_2Ti	9	1.5978	1	1
BrCdI	9	0.4569	1	1
S_2Ti	9	0.2703	1	1
Mg_3	9	0.4336	1	1
Te_2Ti	9	0.0016	1	1
NbS_2	9	0.2625	1	1
BaF_2	9	0.4652	1	1
BiBrTe	9	0.4995	1	1
$RhTe_2$	9	0.0059	1	1
Bi_2Pd	9	0.121	1	1
Cl_2Co	9	0.2698	1	1
NbS_2	9	0.2565	1	1
ClNZr	9	0.2749	1	1
Cl_2Fe	9	0.2687	1	1
S_2Ta	9	0.2553	1	1
Se_2V	9	0.2535	1	1
AsKSn	9	0.4616	1	1
$PbTe_2$	9	0.4545	1	1
$NiTe_2$	9	0.0016	1	1
I_2V	9	0.0036	1	1
Cl_2V	9	1.5233	1	1
GeI_2	9	0.4801	1	1
Se_2Zr	9	0.0026	1	1
STl_2	9	0.4676	1	1
$PtSe_2$	9	0.008	1	1
BiITe	9	2.9747	1	1
CdO_2	9	0.2696	1	1
CoI_2	9	0.0089	1	1

Table showing the first 50 lattice matching combinations with the smallest number of atoms in the supercell.

Lattice matching - minimal strain

Formula	N° atoms	strain	cell size 1	cell size 2
I_2La_2P	557	0.0	57	43
Br_2La_2P	723	0.0	73	57
F_2Na	9	0.0001	1	1
CNb_2S_2	543	0.0001	43	57
S_2Ta	561	0.0001	57	73
$AsLi_3$	742	0.0001	81	64
CdI_2	609	0.0002	73	57
AsKSn	843	0.0002	100	81
$F_2Se_2Y_2$	588	0.0002	61	37
Cu_4Te_2	678	0.0002	64	49
CdI_2	609	0.0003	73	57
STl_2	765	0.0003	91	73
N_2W	171	0.0003	16	25
Cu_2I_2	582	0.0003	65	48
I_2La_2	924	0.0003	100	81
Sb_2Te_3	629	0.0003	64	49
Cl_2Hf_2	802	0.0004	73	91
Gd_2GeI_2	723	0.0004	73	57
InSe	614	0.0004	81	64
As_2Li_2Pr	806	0.0004	81	64
O_2Pt	258	0.0004	25	36
$I_2La_2Si_2$	870	0.0005	81	64
GeI_3Rb	429	0.0005	64	9
Bi_2STe_2	806	0.0005	81	64
Bi_2STe_2	723	0.0006	73	57
$Gd_2I_2S_2$	366	0.0006	36	25
P_2	372	0.0006	43	57
BiClTe	609	0.0006	73	57
$FeSe_2$	840	0.0007	81	118
Cl_2Mn	627	0.0007	64	81
BaF_2	765	0.0007	91	73
$Cu_2Se_2Tl_2$	678	0.0007	65	48
$Br_2H_2Sr_2$	678	0.0007	65	48
Br_2Pr_2	10	0.0007	1	1
I_2Pr	609	0.0007	73	57
Cl_2Ti	429	0.0007	43	57
NbS_2	561	0.0008	57	73
Cl_2Fe	786	0.0008	81	100
$LiNbS_2$	802	0.0008	73	91
LiO	314	0.0008	36	49
Cu_4Te_2	612	0.0008	57	45
CS_2Ta_2	543	0.0008	43	57
S_2Ta	711	0.0008	73	91
Br_2Ca_3Si	366	0.0008	36	25
$LiOS_2Ti$	789	0.0009	64	81
$Cl_2O_2Tm_2$	12	0.0009	1	1
$Ga_2I_2Y_2$	984	0.0009	91	73
$LiMnTe_2$	666	0.0009	73	57
GdI_2	471	0.0009	57	43
FeH_2O_2	221	0.0009	16	25

Table showing the first 50 lattice matching combinations with the smallest strain needed to build the supercell.

$ZnBr_2$ (P-3m1)

Structural and electronic properties

**Formula**	$ZnBr_2$
**Spacegroup**	P-3m1
**Prototype**	CdI2
**Parent 3D**	$ZnBr_2$
**Source DB**	COD
**DB ID**	9011540
**DF2-C09 Binding energy [meV/ Å^2]**	11.39
**RVV10 Binding energy [meV/ Å^2]**	19.97
**Band gap (PBE) [eV]**	3.43

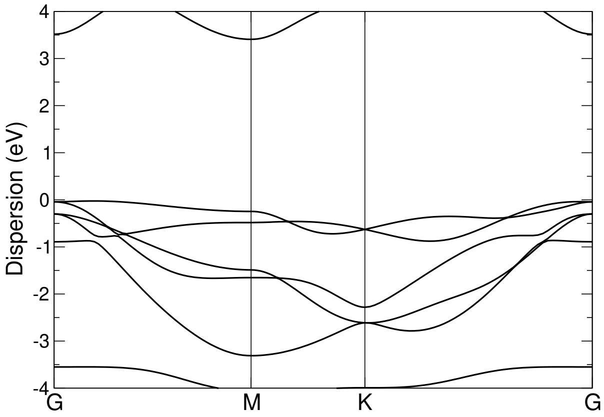

Band structure: Electronic band structure of $ZnBr_2$ (P-3m1) in a representative window around the Fermi energy and along a high-symmetry path.

Optimized crystal structure

Structural parameters: relaxed cell (top) and atomic positions (bottom) of $ZnBr_2$ (P-3m1) in Cartesian coordinates.

		x [Å]	y [Å]	z [Å]
$\mathbf{a_1}$		3.76141954	0.00000000	0.00000000
$\mathbf{a_2}$		−1.88070977	3.25748488	0.00000000
$\mathbf{a_3}$		0.00000000	0.00000000	23.01181044
		x [Å]	y [Å]	z [Å]
●	Zn	0.00000000	2.17165659	11.50590522
●	Br	0.00000000	0.00000000	13.01598117
●	Br	1.88070977	1.08582829	9.99582927

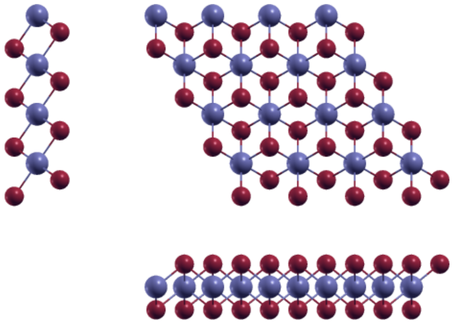

Orthographic projections: views of $ZnBr_2$ (P-3m1) as seen from the x axis (left), the y axis (bottom) and the z axis (center).

Lattice matching - minimal size

Formula	N° atoms	strain	cell size 1	cell size 2
Tl	4	0.1119	1	1
Tl	4	0.2549	1	1
InSe	5	0.4799	1	1
HgO	5	0.117	1	1
Bi_2	5	0.495	1	1
P_2	5	0.2479	1	1
PbTe	5	0.4844	1	1
CaCl	5	0.1531	1	1
I_2Mg	6	0.4539	1	1
Cl_2Mn	6	0.2629	1	1
CdI_2	6	2.8381	1	1
$AgTe_2$	6	0.113	1	1
$MoSe_2$	6	0.2539	1	1
S_2Ta	6	0.2658	1	1
Br_2Ca	6	2.8537	1	1
$AsSn_2$	6	0.0068	1	1
$SiTe_2$	6	0.0042	1	1
Br_2La	6	0.4548	1	1
Br_2Cu	6	0.9519	1	1
PbS_2	6	0.0077	1	1
BiClTe	6	2.8427	1	1
Cl_2Ti	6	0.2481	1	1
BrCdI	6	0.4617	1	1
S_2Ti	6	0.2731	1	1
Te_2Ti	6	0.0003	1	1
NbS_2	6	0.2652	1	1
BaF_2	6	0.47	1	1
$RhTe_2$	6	0.004	1	1
Bi_2Pd	6	0.1221	1	1
GeI_2	6	0.4494	1	1
Cl_2Co	6	0.2726	1	1
NbS_2	6	0.2591	1	1
Cl_2Fe	6	0.2715	1	1
S_2Ta	6	0.2579	1	1
Se_2V	6	0.2561	1	1
AsKSn	6	0.4664	1	1
$PbTe_2$	6	0.4592	1	1
$NiTe_2$	6	0.0035	1	1
I_2V	6	0.0055	1	1
Cl_2V	6	1.5369	1	1
GeI_2	6	0.4851	1	1
Se_2Zr	6	0.0046	1	1
STl_2	6	0.4725	1	1
$PtSe_2$	6	0.0061	1	1
BiITe	6	3.0001	1	1
CdO_2	6	0.2723	1	1
GeS_2	6	0.1419	1	1
$TaTe_2$	6	0.0073	1	1
$MnSe_2$	6	0.153	1	1
Cl_2Zr	6	0.2721	1	1

Table showing the first 50 lattice matching combinations with the smallest number of atoms in the supercell.

Lattice matching - minimal strain

Formula	N° atoms	strain	cell size 1	cell size 2
CoO_2	75	0.0	9	16
Cl_2Hf_2	643	0.0	81	100
BiBrTe	300	0.0	57	43
DyI_2	183	0.0001	36	25
CCl_2Gd_2	8	0.0001	1	1
NbS_2	435	0.0001	64	81
Bi_2Se_2Te	563	0.0001	81	64
Bi_2	290	0.0001	64	49
$Cl_2H_2Zr_2$	678	0.0001	64	81
$CrSe_2$	255	0.0001	36	49
HN_3OZn	531	0.0002	49	64
SSb_2Te_2	705	0.0002	100	81
$MnNaTe_2$	624	0.0002	100	81
Bi_2Te_3	327	0.0002	49	36
AsKSn	492	0.0002	91	73
BrCdI	543	0.0002	100	81
Bi_2O_2	387	0.0002	65	48
Sb_2SeTe_2	563	0.0002	81	64
BiITe	255	0.0003	49	36
H_2MnO_2	416	0.0003	37	61
In_2Te_3	563	0.0003	81	64
Te_2Ti	6	0.0003	1	1
H_2Li_2Pt	74	0.0003	8	10
BN	30	0.0003	4	9
$HfSe_2$	6	0.0003	1	1
Br_3Cs	343	0.0003	81	25
Bi_2STe_2	504	0.0004	73	57
Tl	244	0.0004	57	73
$Br_2La_2O_2$	483	0.0004	65	48
$Eu_2F_2I_2$	483	0.0004	65	48
Bi_2SeTe_2	437	0.0004	64	49
Se_2V	390	0.0005	57	73
PbTe	333	0.0005	73	57
As_2CeLi_2	504	0.0005	73	57
NiO_2	75	0.0005	9	16
$I_2S_2Tb_2$	258	0.0005	36	25
Cl_2NSc_2	674	0.0005	73	91
$Dy_2I_2S_2$	258	0.0006	36	25
$Ga_2Gd_2I_2$	486	0.0006	64	49
NS_2Ta	148	0.0006	16	25
GeI_2Y_2	563	0.0006	81	64
NbS_2	492	0.0006	73	91
CBr_2Y_2	8	0.0007	1	1
GeI_2	390	0.0007	73	57
I_2La_2	565	0.0007	91	73
KNO_3	227	0.0007	49	16
FeO_2	75	0.0008	9	16
CS_2Ta_2	467	0.0008	49	64
$PbTe_2$	543	0.0008	100	81
C_4Ca_2	831	0.0008	113	82

Table showing the first 50 lattice matching combinations with the smallest strain needed to build the supercell.

$ZnCl_2$ (P-3m1)

Structural and electronic properties

**Formula**	$ZnCl_2$
**Spacegroup**	P-3m1
**Prototype**	CdI2
**Parent 3D**	$ZnCl_2$
**Source DB**	COD
**DB ID**	9009136
**DF2-C09 Binding energy [meV/ Å^2]**	11.07
**RVV10 Binding energy [meV/ Å^2]**	16.79
**Band gap (PBE) [eV]**	4.46

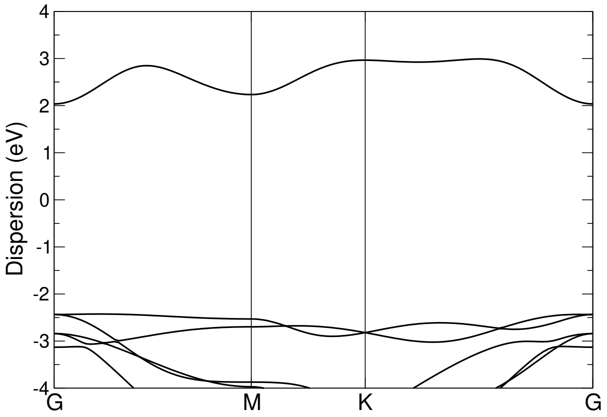

Band structure: Electronic band structure of $ZnCl_2$ (P-3m1) in a representative window around the Fermi energy and along a high-symmetry path.

Optimized crystal structure

Structural parameters: relaxed cell (top) and atomic positions (bottom) of $ZnCl_2$ (P-3m1) in Cartesian coordinates.

	x [Å]	y [Å]	z [Å]
$\mathbf{a_1}$	3.60069436	−0.00000000	0.00000000
$\mathbf{a_2}$	−1.80034718	3.11829279	0.00000000
$\mathbf{a_3}$	0.00000000	0.00000000	22.77259356

		x [Å]	y [Å]	z [Å]
●	Cl	-0.00000000	2.07886186	12.75679501
●	Zn	1.80034718	1.03943093	11.38629636
●	Cl	-0.00000000	0.00000000	10.01579896

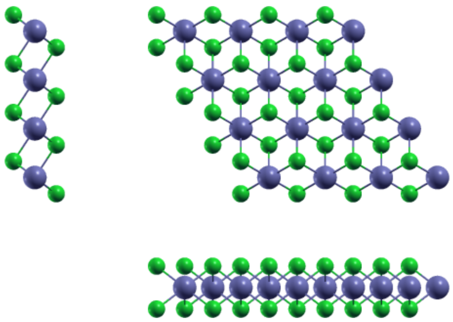

Orthographic projections: views of $ZnCl_2$ (P-3m1) as seen from the x axis (left), the y axis (bottom) and the z axis (center).

Lattice matching - minimal size

Formula	N° atoms	strain	cell size 1	cell size 2
K	4	0.3127	1	1
Tl	4	0.1215	1	1
Sn	4	0.4247	1	1
Na	4	0.0068	1	1
In	4	0.4347	1	1
In	4	1.6293	1	1
HgO	5	0.1292	1	1
As_2	5	0.001	1	1
LiO	5	0.2657	1	1
Mg_2	5	0.1136	1	1
Sb_2	5	2.8529	1	1
CrS_2	6	1.5217	1	1
I_2Mg	6	2.9261	1	1
S_2V	6	0.2555	1	1
MoS_2	6	0.2565	1	1
$MoTe_2$	6	0.0046	1	1
$AgTe_2$	6	0.1232	1	1
HfS_2	6	0.0038	1	1
$HfTe_2$	6	0.4522	1	1
Te_2V	6	0.0027	1	1
$CuTe_2$	6	0.0034	1	1
S_2Zr	6	0.0094	1	1
Br_2La	6	2.9306	1	1
Br_2Co	6	0.0006	1	1
ReS_2	6	1.5647	1	1
Ca_2N	6	0.0016	1	1
$AuTe_2$	6	0.4716	1	1
$PdTe_2$	6	0.4654	1	1
Mg_3	6	0.1183	1	1
I_2Zn	6	0.4834	1	1
Te_2Zn	6	0.0048	1	1
S_2W	6	0.2566	1	1
Bi_2Pd	6	0.1363	1	1
GeI_2	6	2.902	1	1
Br_2Mn	6	0.0012	1	1
PtS_2	6	0.0058	1	1
$CoTe_2$	6	0.0041	1	1
CdClO	6	0.0032	1	1
Ba_2N	6	0.456	1	1
Se_2Ti	6	0.0084	1	1
AsKSn	6	2.9924	1	1
Te_2Zr	6	0.4535	1	1
Te_2W	6	0.0044	1	1
Cl_2Cu	6	0.5829	1	1
$SnTe_2$	6	0.4961	1	1
Cl_2V	6	0.2622	1	1
STl_2	6	3.0244	1	1
OTl_2	6	0.003	1	1
Br_2Fe	6	0.0007	1	1
Br_2Ni	6	0.0058	1	1

Table showing the first 50 lattice matching combinations with the smallest number of atoms in the supercell.

Lattice matching - minimal strain

Formula	N° atoms	strain	cell size 1	cell size 2
Ag_2	341	0.0	81	49
Br_2Tb_2	565	0.0	91	73
S_2V	390	0.0	57	73
O_2Pt	339	0.0001	49	64
I_2Zn	390	0.0001	73	57
Sb_2Se_2Te	386	0.0001	57	43
CeI_2	339	0.0002	65	48
Bi_2Te_2	84	0.0002	16	9
$Br_2Eu_2O_2$	483	0.0002	65	48
Ni_2Se_2	387	0.0002	65	48
Bi_2S_3	638	0.0003	91	73
Cu_4Te_2	258	0.0003	36	25
Ba_2Pt	390	0.0003	81	49
$SnTe_2$	339	0.0003	64	49
$CrSe_2$	492	0.0003	73	91
AsKSn	255	0.0003	49	36
Sb_2Se_2Te	386	0.0003	57	43
Ba_2Pt	294	0.0004	61	37
$BiTe_2$	300	0.0005	57	43
$F_2Ho_2Se_2$	786	0.0005	100	81
Br_2Ho_2	565	0.0005	91	73
As_2Sn_2	499	0.0005	81	64
BH_4Li	486	0.0005	64	49
Cl_2La_2	499	0.0005	81	64
$PdTe_2$	492	0.0006	91	73
Br_2PY_2	705	0.0006	100	81
$Ga_2Ge_2Te_2$	486	0.0006	64	49
Br_2Gd_2	499	0.0006	81	64
Br_2Co	6	0.0006	1	1
Ag_2	257	0.0006	61	37
Pb_2Se_2	101	0.0007	19	11
H_2NiO_2	597	0.0007	64	81
$Ce_2I_2Si_2$	429	0.0007	57	43
Br_2Fe	6	0.0007	1	1
$I_2Pr_2S_2$	537	0.0007	81	49
Li_2P_2Pr	437	0.0007	64	49
Se_2Yb	300	0.0007	57	43
MoS_2	390	0.0007	57	73
NS_2Ta	219	0.0007	25	36
$Br_2Er_2Se_2$	801	0.0008	135	66
$Dy_2I_2S_2$	171	0.0008	25	16
Br_2Cd	543	0.0008	100	81
KS_2Ti	7	0.0008	1	1
S_2W	390	0.0008	57	73
I_2Pr	339	0.0008	65	48
I_2La_2	291	0.0008	49	36
Sb_2Te_3	233	0.0008	36	25
$CrSe_2$	435	0.0008	64	81
H_2MgO_2	536	0.0009	57	73
Ge_2Te_2	378	0.0009	70	42

Table showing the first 50 lattice matching combinations with the smallest strain needed to build the supercell.

$ZnCl_2$ (P-4m2)

Structural and electronic properties

**Formula**	$ZnCl_2$
**Spacegroup**	P-4m2
**Prototype**	HgI2
**Parent 3D**	Zn_2Cl_4
**Source DB**	ICSD
**DB ID**	26152
**DF2-C09 Binding energy [meV/ Å^2]**	9.8
**RVV10 Binding energy [meV/ Å^2]**	15.92
**Band gap (PBE) [eV]**	4.25

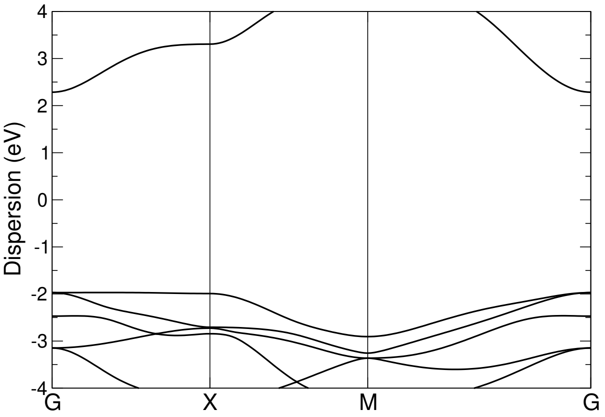

Band structure: Electronic band structure of $ZnCl_2$ (P-4m2) in a representative window around the Fermi energy and along a high-symmetry path.

Optimized crystal structure

Structural parameters: relaxed cell (top) and atomic positions (bottom) of $ZnCl_2$ (P-4m2) in Cartesian coordinates.

	x [Å]	y [Å]	z [Å]
$\mathbf{a_1}$	3.71200191	−0.00000000	0.00000000
$\mathbf{a_2}$	0.00000000	3.71200191	0.00000000
$\mathbf{a_3}$	0.00000000	0.00000000	22.75934170

		x [Å]	y [Å]	z [Å]
●	Zn	0.00000000	0.00000000	11.37967085
●	Cl	0.00000000	1.85600095	10.01205835
●	Cl	1.85600095	-0.00000000	12.74728336

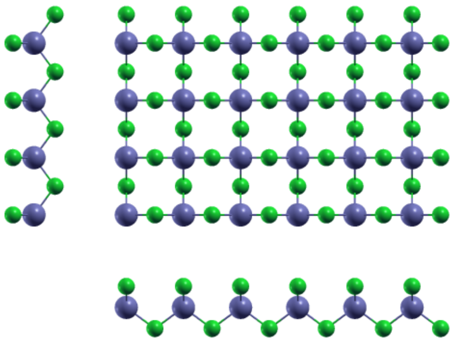

Orthographic projections: views of $ZnCl_2$ (P-4m2) as seen from the x axis (left), the y axis (bottom) and the z axis (center).

Lattice matching - minimal size

Formula	N° atoms	strain	cell size 1	cell size 2
InSe	5	0.1704	1	1
HgO	5	0.5935	1	1
Bi_2	5	0.1772	1	1
GeTe	5	0.1318	1	1
AgTl	5	0.3774	1	1
S_2	5	0.1329	1	1
PbTe	5	0.1724	1	1
Sb_2	5	0.1533	1	1
$IrTe_2$	6	0.1324	1	1
CrS_2	6	0.11	1	1
I_2Mg	6	0.1591	1	1
$CdCl_2$	6	0.131	1	1
CdI_2	6	0.1744	1	1
$AgTe_2$	6	0.5668	1	1
Br_2Ca	6	0.1759	1	1
CaI_2	6	0.7689	1	1
$InSe_2$	6	0.1313	1	1
$GeTe_2$	6	0.1303	1	1
$HfTe_2$	6	0.139	1	1
I_2Mn	6	0.1311	1	1
Br_2La	6	0.1594	1	1
Br_2Cu	6	0.1195	1	1
BiClTe	6	0.1748	1	1
ReS_2	6	0.1087	1	1
$AuTe_2$	6	0.1458	1	1
BrCdI	6	0.1624	1	1
$PdTe_2$	6	0.1436	1	1
FeI_2	6	0.1295	1	1
I_2Ni	6	0.1304	1	1
I_2Zn	6	0.1501	1	1
BaF_2	6	0.166	1	1
BiBrTe	6	0.1815	1	1
Bi_2Pd	6	0.2147	1	1
GeI_2	6	0.1571	1	1
Ba_2Hg	6	0.3968	1	1
Ba_2N	6	0.1403	1	1
AsKSn	6	0.1644	1	1
Te_2Zr	6	0.1394	1	1
$PbTe_2$	6	0.1613	1	1
Cl_2Cu	6	0.1067	1	1
$SnTe_2$	6	0.1548	1	1
GeI_2	6	0.1727	1	1
STl_2	6	0.1671	1	1
BiITe	6	0.1898	1	1
DyI_2	6	0.7799	1	1
Br_2Mg	6	0.1294	1	1
Se_2Yb	6	0.1574	1	1
$BiTe_2$	6	0.1577	1	1
GdI_2	6	0.1851	1	1
CNNa	6	0.4701	1	1

Table showing the first 50 lattice matching combinations with the smallest number of atoms in the supercell.

Lattice matching - minimal strain

Formula	N° atoms	strain	cell size 1	cell size 2
F_4Sn	563	0.0	81	64
F_4Nb	705	0.0	100	81
Ba_2Cd	774	0.0002	145	113
$Ag_2K_2Te_2$	537	0.0002	81	49
Se_2Sn_2	582	0.0002	98	72
$AgTe_2$	255	0.0002	36	49
Cu_2F_4	594	0.0004	100	49
HgO	275	0.0004	49	64
$AgClO_4$	945	0.0004	145	85
$Ca_2Cl_2H_2$	9	0.0005	1	1
$Er_2I_2S_2$	606	0.0005	90	56
Bi_2In_2	139	0.0006	25	16
Cl_4Cu_2	129	0.0006	25	9
$I_2S_2Yb_2$	678	0.0007	100	63
N_2Re	177	0.0007	20	39
$Ba_2H_2I_2$	942	0.0008	136	89
Ba_2Hg	435	0.0008	81	64
PbS_2Sn	873	0.0009	143	111
$Cu_2Rb_2Te_2$	171	0.0009	25	16
$Ag_2K_2Se_2$	258	0.0009	36	25
$I_2La_2O_2$	636	0.0009	82	65
$Br_2Er_2Se_2$	198	0.001	30	18
Bi_2Pd	441	0.001	65	82
$I_2S_2Yb_2$	657	0.001	97	61
$FeSe_2$	513	0.001	65	106
ClNZr	339	0.001	48	65
Br_2Ca_3Si	627	0.001	81	64
Hf_3Te_2	8	0.0011	1	1
$I_2S_2Tm_2$	657	0.0011	97	61
In	363	0.0011	79	126
$AgCuTe_2$	778	0.0011	110	112
$Br_2F_2Sr_2$	795	0.0012	101	82
NaO_4	649	0.0012	108	65
$Ho_2I_2S_2$	585	0.0012	87	54
Ca_4Cu_2	456	0.0013	72	40
$Br_2Dy_2S_2$	729	0.0013	107	68
$H_2I_2Sr_2$	849	0.0013	113	85
$Br_2Ga_2Te_2$	789	0.0014	125	69
K	183	0.0014	49	36
$Br_2F_2Pb_2$	795	0.0014	101	82
C	101	0.0014	12	65
$Br_2Ho_2S_2$	729	0.0014	107	68
Br_2Ca	576	0.0015	103	89
$Gd_2I_2S_2$	669	0.0015	101	61
$I_2Se_2Tm_2$	156	0.0015	24	14
Cl_2NSc_2	521	0.0015	52	73
Br_2Ca	498	0.0015	89	77
$I_2S_2Tb_2$	534	0.0016	80	49
$Br_2H_2Zr_2$	534	0.0016	48	65
Bi_2	487	0.0016	103	89

Table showing the first 50 lattice matching combinations with the smallest strain needed to build the supercell.

ZnI_2 (P-3m1)

Structural and electronic properties

**Formula**	ZnI_2
**Spacegroup**	P-3m1
**Prototype**	CdI2
**Parent 3D**	ZnI_2
**Source DB**	COD
**DB ID**	9009137
**DF2-C09 Binding energy [meV/ Å^2]**	11.68
**RVV10 Binding energy [meV/ Å^2]**	17.41
**Band gap (PBE) [eV]**	2.0

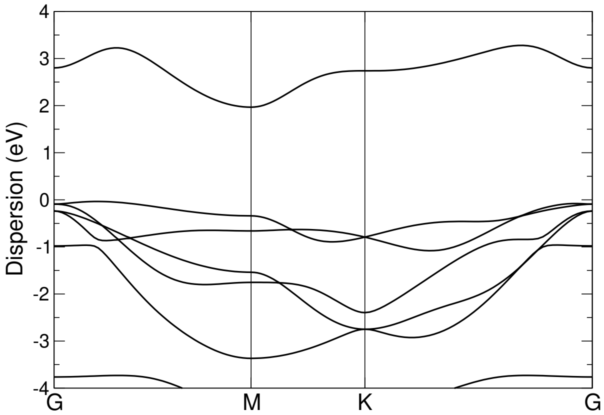

Band structure: Electronic band structure of ZnI_2 (P-3m1) in a representative window around the Fermi energy and along a high-symmetry path.

Optimized crystal structure

Structural parameters: relaxed cell (top) and atomic positions (bottom) of ZnI_2 (P-3m1) in Cartesian coordinates.

	x [Å]	y [Å]	z [Å]
$\mathbf{a_1}$	4.07556486	0.00000000	0.00000000
$\mathbf{a_2}$	−2.03778243	3.52954271	0.00000000
$\mathbf{a_3}$	0.00000000	0.00000000	23.33280231

		x [Å]	y [Å]	z [Å]
●	I	2.03778243	1.17651424	10.02308704
●	Zn	-0.00000000	0.00000000	11.66640115
●	I	0.00000000	2.35302847	13.30971527

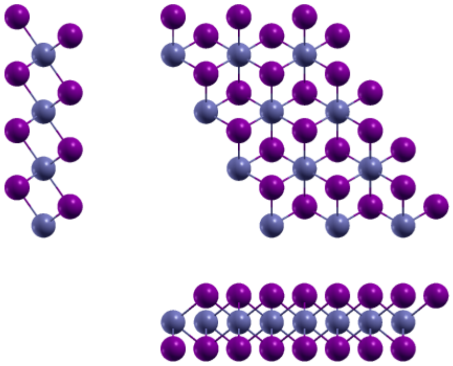

Orthographic projections: views of ZnI_2 (P-3m1) as seen from the x axis (left), the y axis (bottom) and the z axis (center).

Lattice matching - minimal size

Formula	N° atoms	strain	cell size 1	cell size 2
K	4	0.4736	1	1
Na	4	0.2462	1	1
In	4	4.845	1	1
As_2	5	0.2568	1	1
Sb_2	5	0.0033	1	1
CaCl	5	0.123	1	1
Cl_2Zn	6	0.2553	1	1
I_2Mg	6	0.0091	1	1
$MoTe_2$	6	0.2491	1	1
PSn_2	6	0.2693	1	1
Ba_2Pt	6	0.4871	1	1
HfS_2	6	0.2606	1	1
CaI_2	6	0.4507	1	1
$AsSn_2$	6	0.2749	1	1
Te_2V	6	1.6321	1	1
I_2Pr	6	0.1537	1	1
$CuTe_2$	6	0.26	1	1
S_2Zr	6	0.2682	1	1
Br_2La	6	0.0095	1	1
Ca_2Si	6	0.4991	1	1
Br_2Co	6	0.2562	1	1
Ca_2N	6	0.2575	1	1
$AuTe_2$	6	0.0045	1	1
Cl_2Zn	6	0.1327	1	1
$PdTe_2$	6	0.0069	1	1
Te_2Zn	6	0.2489	1	1
GeI_2	6	0.0072	1	1
Br_2Mn	6	0.2537	1	1
$CrTe_2$	6	1.5613	1	1
PtS_2	6	0.2475	1	1
$CoTe_2$	6	0.261	1	1
CdClO	6	1.6284	1	1
Se_2Ti	6	1.5888	1	1
Br_2Ti	6	1.5571	1	1
Te_2W	6	0.2493	1	1
I_2Nd	6	0.1547	1	1
I_2Tm	6	0.4478	1	1
S_2Sn	6	0.2686	1	1
$SnTe_2$	6	0.0049	1	1
I_2Pb	6	2.8525	1	1
$PtSe_2$	6	0.2759	1	1
OTl_2	6	1.6298	1	1
BrNZr	6	1.5373	1	1
Br_2Fe	6	0.2563	1	1
GeS_2	6	0.1165	1	1
$TaTe_2$	6	0.274	1	1
$MnSe_2$	6	0.1229	1	1
DyI_2	6	0.4576	1	1
Br_2Ni	6	0.2633	1	1
CeI_2	6	0.153	1	1

Table showing the first 50 lattice matching combinations with the smallest number of atoms in the supercell.

Lattice matching - minimal strain

Formula	N° atoms	strain	cell size 1	cell size 2
Te_2W	339	0.0001	49	64
Sb_2Te_2	388	0.0001	64	49
Cl_2Zn	390	0.0001	57	73
CaH_2O_2	597	0.0001	64	81
$MoTe_2$	339	0.0001	49	64
$Cl_2N_2Zr_2$	678	0.0002	64	81
$NbTe_2$	543	0.0002	81	100
I_2La_2Te	638	0.0002	91	73
H_2MgO_2	416	0.0003	37	61
Te_2Zn	339	0.0003	49	64
$Br_2Ho_2S_2$	42	0.0003	6	4
N_3W_2	320	0.0003	25	49
$Br_2Hf_2N_2$	435	0.0003	49	48
$Cl_2H_2Lu_2$	765	0.0003	73	91
Ag_2F_4	522	0.0004	90	42
Br_2Ca_3Si	711	0.0004	91	73
As_2O_3	93	0.0004	16	9
$I_2Nd_2S_2$	561	0.0004	73	57
S_2Zr	543	0.0004	81	100
Se_2Ti	300	0.0005	43	57
N_2Re	354	0.0005	39	79
CdH_2O_2	467	0.0005	49	64
O_2Sn_2	496	0.0005	72	70
Br_2Co	390	0.0005	57	73
$CuTe_2$	435	0.0005	64	81
$HfLiS_2$	403	0.0005	49	64
I_2La_2Sb	563	0.0006	81	64
Br_2Fe	390	0.0006	57	73
$Cl_2O_2Sc_2$	978	0.0006	106	110
Se_2Ta	255	0.0006	36	49
Cl_2Mg	492	0.0006	73	91
$Cl_2O_2Ti_2$	306	0.0007	32	35
$Br_2Hf_2N_2$	678	0.0007	64	81
Br_2Ni	492	0.0007	73	91
S_2Sn	543	0.0007	81	100
$I_2Se_2Tb_2$	486	0.0007	74	44
F_4Pb	435	0.0007	65	48
CNNa	156	0.0008	28	24
CCl_2Lu_2	536	0.0008	57	73
PTe_2Ti_2	674	0.0008	73	91
Br_2Zr_2	357	0.0009	43	57
I_4Sr_2	255	0.0009	49	18
$Cl_2H_2Sc_2$	402	0.0009	36	49
Se_4TiZr	543	0.0009	81	50
KS_2Ti	463	0.0009	57	73
HfS_2	435	0.0009	64	81
K	307	0.0009	81	64
$Gd_2I_2S_2$	786	0.001	100	81
As_2	317	0.001	57	73
Cu_2F_4	237	0.001	39	20

Table showing the first 50 lattice matching combinations with the smallest strain needed to build the supercell.

$Zr_2H_2Br_2$ (P-3m1)

Structural and electronic properties

**Formula**	$Zr_2H_2Br_2$
**Spacegroup**	P-3m1
**Prototype**	SmSI
**Parent 3D**	$Br_2H_2Zr_2$
**Source DB**	MPDS
**DB ID**	S2120015
**DF2-C09 Binding energy [meV/ Å^2]**	15.72
**RVV10 Binding energy [meV/ Å^2]**	N/A
**Band gap (PBE) [eV]**	0.0

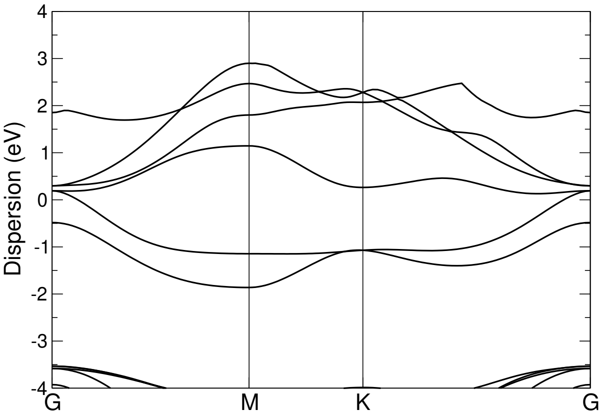

Band structure: Electronic band structure of $Zr_2H_2Br_2$ (P-3m1) in a representative window around the Fermi energy and along a high-symmetry path.

Optimized crystal structure

Structural parameters: relaxed cell (top) and atomic positions (bottom) of $Zr_2H_2Br_2$ (P-3m1) in Cartesian coordinates.

	x [Å]	y [Å]	z [Å]
$\mathbf{a_1}$	1.75257914	−3.03555631	0.00000000
$\mathbf{a_2}$	1.75257931	3.03555641	0.00000000
$\mathbf{a_3}$	0.00000000	0.00000000	24.97945775

		x [Å]	y [Å]	z [Å]
●	Zr	0.87629005	-2.52963057	-1.37696280
•	H	-0.87628917	-1.51777849	-1.05944255
●	Br	0.87629046	-0.50592653	-3.26699515
●	Zr	0.87628909	-0.50592574	1.37696280
•	H	-0.87629014	-1.51777792	1.05944255
●	Br	0.87628868	-2.52962978	3.26699515

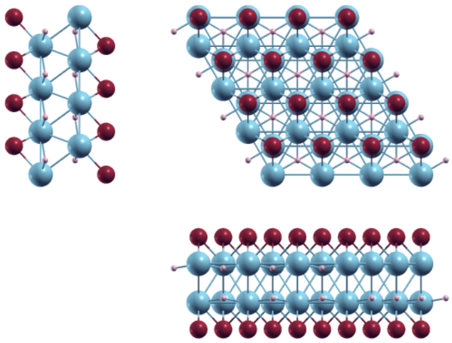

Orthographic projections: views of $Zr_2H_2Br_2$ (P-3m1) as seen from the x axis (left), the y axis (bottom) and the z axis (center).

Lattice matching - minimal size

Formula	N° atoms	strain	cell size 1	cell size 2
Tl	7	0.1296	1	1
Sn	7	0.1123	1	1
Na	7	0.0058	1	1
In	7	0.114	1	1
In	7	0.2688	1	1
Gd	7	0.1125	1	1
HgO	8	0.1389	1	1
GeTe	8	0.4608	1	1
S_2	8	0.4647	1	1
Mg_2	8	0.1195	1	1
$IrTe_2$	9	0.463	1	1
CrS_2	9	0.2485	1	1
S_2V	9	0.2735	1	1
MoS_2	9	0.2746	1	1
$CdCl_2$	9	0.4581	1	1
$MoTe_2$	9	0.0082	1	1
$AgTe_2$	9	0.1317	1	1
$ReSe_2$	9	0.0065	1	1
$InSe_2$	9	0.4591	1	1
$GeTe_2$	9	0.4557	1	1
$HfTe_2$	9	0.4843	1	1
I_2Mn	9	0.4584	1	1
NSr_2	9	0.4499	1	1
ReS_2	9	0.2565	1	1
LiO_2	9	2.6504	1	1
$PdTe_2$	9	0.4984	1	1
FeI_2	9	0.453	1	1
I_2Ni	9	0.4561	1	1
Mg_3	9	0.1256	1	1
CrI_2	9	0.4519	1	1
Te_2Zn	9	0.008	1	1
S_2W	9	0.2747	1	1
Bi_2Pd	9	0.1473	1	1
Cl_2Ni	9	0.0056	1	1
$CrTe_2$	9	0.0004	1	1
PtS_2	9	0.0069	1	1
Br_2V	9	0.0073	1	1
Se_2Ti	9	0.0042	1	1
Br_2Ti	9	0.0002	1	1
Te_2Zr	9	0.4856	1	1
Te_2W	9	0.0083	1	1
$AsSe_2$	9	0.0042	1	1
$SnTe_2$	9	3.0403	1	1
BrNZr	9	0.0029	1	1
$NbSe_2$	9	0.0052	1	1
O_2Zn	9	1.5893	1	1
Br_2Cr	9	0.0001	1	1
DyI_2	9	13.654	1	1
Se_2Ta	9	0.0051	1	1
Br_2Mg	9	0.4526	1	1

Table showing the first 50 lattice matching combinations with the smallest number of atoms in the supercell.

Lattice matching - minimal strain

Formula	N° atoms	strain	cell size 1	cell size 2
Br_2PY_2	629	0.0	64	49
$AuTe_2$	471	0.0001	57	43
P_2Sn_2	838	0.0001	91	73
$F_2Lu_2Se_2$	510	0.0001	49	36
Br_2Cr	9	0.0001	1	1
$AlLiTe_2$	214	0.0001	25	16
NS_2Ta	412	0.0001	36	49
GeTe	762	0.0002	100	81
$Dy_2I_2S_2$	780	0.0002	81	49
Br_2Cd	531	0.0002	64	49
Br_2Ti	9	0.0002	1	1
$Er_2F_2Se_2$	780	0.0002	73	57
$F_2Se_2Yb_2$	984	0.0002	91	73
$Ce_2I_2S_2$	150	0.0002	16	9
Ca_2Si	123	0.0002	16	9
CoH_2O_2	707	0.0002	57	73
$F_2Se_2Tm_2$	870	0.0003	81	64
$Ga_2I_2Y_2$	366	0.0003	36	25
Pt_2Te_2	666	0.0003	73	57
Cl_2Sc_2	10	0.0003	1	1
$PtTe_2$	471	0.0003	57	43
$LiMnSe_2$	742	0.0004	81	64
$Br_2O_2Yb_2$	678	0.0004	65	48
$CrTe_2$	9	0.0004	1	1
O_2Zn	429	0.0004	43	57
$HfTe_2$	609	0.0004	73	57
S_2Zn_2	838	0.0004	91	73
In_2Se_3	723	0.0005	73	57
NaPSn	609	0.0005	73	57
BaF_2	291	0.0005	36	25
H_2Si_2	924	0.0005	100	81
Br_2Cu	600	0.0005	72	56
CrS_2	486	0.0006	49	64
C_2	392	0.0006	39	79
Br_2Er_2	514	0.0006	57	43
$Dy_2I_2S_2$	744	0.0006	84	40
O_2Pt	786	0.0007	81	100
Ni_2SbTe_2	911	0.0007	91	73
O_4PSn	354	0.0007	39	20
$Cl_2H_2Sc_2$	12	0.0007	1	1
$IrTe_2$	843	0.0008	100	81
$Cl_2O_2V_2$	600	0.0008	53	47
ReS_2	561	0.0008	57	73
Fe_2Te_2	582	0.0008	65	48
$Dy_2I_2S_2$	588	0.0008	61	37
N_3Na	456	0.0008	56	30
$InSe_2$	843	0.0008	100	81
DyI_2	633	0.0009	81	49
In	586	0.0009	81	100
S_2	692	0.0009	91	73

Table showing the first 50 lattice matching combinations with the smallest strain needed to build the supercell.

$Zr_2H_2Br_2$ (P-3m1)

Structural and electronic properties

**Formula**	$Zr_2H_2Br_2$
**Spacegroup**	P-3m1
**Prototype**	SmSI
**Parent 3D**	$Br_2H_2Zr_2$
**Source DB**	MPDS
**DB ID**	S2120015
**DF2-C09 Binding energy [meV/ Å^2]**	16.76
**RVV10 Binding energy [meV/ Å^2]**	N/A
**Band gap (PBE) [eV]**	0.0

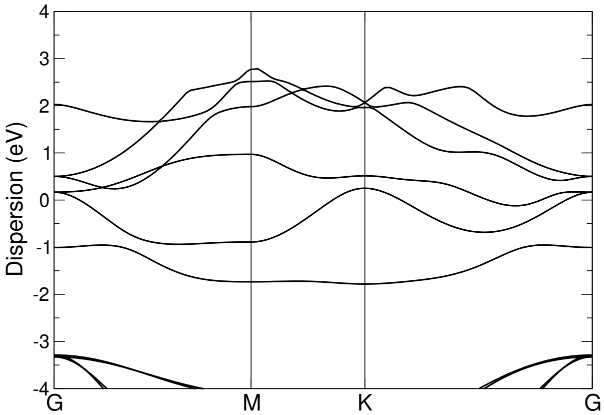

Band structure: Electronic band structure of $Zr_2H_2Br_2$ (P-3m1) in a representative window around the Fermi energy and along a high-symmetry path.

Optimized crystal structure

Structural parameters: relaxed cell (top) and atomic positions (bottom) of $Zr_2H_2Br_2$ (P-3m1) in Cartesian coordinates.

	x [Å]	y [Å]	z [Å]
$\mathbf{a_1}$	1.71958043	−2.97840011	0.00000000
$\mathbf{a_2}$	1.71958043	2.97840011	0.00000000
$\mathbf{a_3}$	0.00000000	0.00000000	25.43641093

		x [Å]	y [Å]	z [Å]
	Zr	0.00000000	-0.99280041	-1.45900692
	Zr	0.00000000	0.99280041	1.45900692
	H	0.00000000	-0.99279994	0.59226994
	H	0.00000000	0.99279994	-0.59226994
	Br	0.00000000	-0.99279973	3.36738600
	Br	0.00000000	0.99279973	-3.36738600

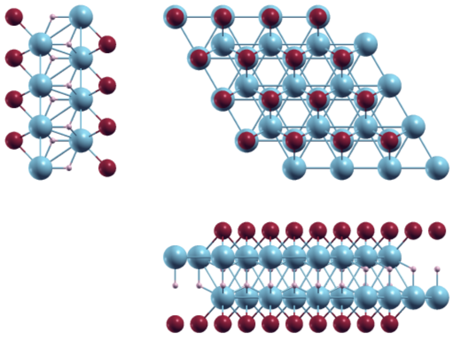

Orthographic projections: views of $Zr_2H_2Br_2$ (P-3m1) as seen from the x axis (left), the y axis (bottom) and the z axis (center).

Lattice matching - minimal size

Formula	N° atoms	strain	cell size 1	cell size 2
Tl	7	0.1363	1	1
Sn	7	0.116	1	1
In	7	0.1181	1	1
HgO	8	0.1468	1	1
AsSb	8	0.4681	1	1
GeTe	8	0.4836	1	1
S_2	8	2.8321	1	1
Mg_2	8	0.1247	1	1
$IrTe_2$	9	0.486	1	1
CrS_2	9	0.2606	1	1
$CdCl_2$	9	0.4809	1	1
$AgTe_2$	9	0.1388	1	1
$ReSe_2$	9	0.0024	1	1
S_2Ta	9	0.0085	1	1
$InSe_2$	9	0.4818	1	1
$GeTe_2$	9	0.4783	1	1
$SiTe_2$	9	0.4529	1	1
I_2Mn	9	0.4812	1	1
NSr_2	9	0.4722	1	1
PbS_2	9	0.4616	1	1
ReS_2	9	0.2692	1	1
LiO_2	9	0.3042	1	1
FeI_2	9	0.4755	1	1
I_2Ni	9	0.4788	1	1
S_2Ti	9	0.0037	1	1
Mg_3	9	0.1318	1	1
NbS_2	9	0.0089	1	1
CrI_2	9	0.4743	1	1
Cl_2Ni	9	0.0033	1	1
Cl_2Co	9	0.004	1	1
$CrTe_2$	9	0.0095	1	1
Br_2V	9	0.0015	1	1
ClNZr	9	0.0006	1	1
Cl_2Fe	9	0.0047	1	1
Br_2Ti	9	0.0089	1	1
$AsSe_2$	9	0.0048	1	1
$NiTe_2$	9	0.4515	1	1
Cl_2Cu	9	0.1036	1	1
I_2V	9	0.4562	1	1
Se_2Zr	9	0.4539	1	1
CdO_2	9	0.0042	1	1
BrNZr	9	0.0061	1	1
$NbSe_2$	9	0.0037	1	1
CoI_2	9	0.4692	1	1
O_2Zn	9	0.256	1	1
Br_2Cr	9	0.0092	1	1
Cl_2Zr	9	0.0043	1	1
$FeSe_2$	9	0.1132	1	1
Se_2Ta	9	0.0038	1	1
Br_2Mg	9	0.4751	1	1

Table showing the first 50 lattice matching combinations with the smallest number of atoms in the supercell.

Lattice matching - minimal strain

Formula	N° atoms	strain	cell size 1	cell size 2
Cl_2Tb_2	580	0.0	64	49
Br_2Y_2	924	0.0001	100	81
CoH_2O_2	986	0.0001	81	100
C	10	0.0001	1	4
$CeLi_2P_2$	341	0.0002	36	25
GeTe	552	0.0002	73	57
Bi_2	182	0.0002	25	16
PbS_2	843	0.0002	100	81
FeI_2	678	0.0002	81	64
Ho_2S_2	836	0.0002	96	65
H_2Si_2	666	0.0002	73	57
$GeNi_3Te_2$	600	0.0002	57	43
PTe_2Zr_2	911	0.0002	91	73
Br_2HLa	316	0.0002	36	25
AsI_2La_2	551	0.0003	61	37
$C_2Br_2Y_2$	732	0.0003	69	53
Cd_2I_3	551	0.0003	61	37
Br_2Mg	678	0.0003	81	64
$PdTe_2$	402	0.0003	49	36
In_2S_3	723	0.0004	73	57
CCl_2Sc_2	11	0.0004	1	1
NaPSn	471	0.0004	57	43
NaO_4	74	0.0004	9	4
$AgNO_3$	74	0.0004	9	4
Ga_2Se_2	924	0.0004	100	81
Bi_2SeTe_2	230	0.0004	25	16
O_2Zn	561	0.0004	57	73
AsSb	692	0.0005	91	73
CrO_2	171	0.0005	16	25
$InSe_2$	609	0.0005	73	57
Pt_2Te_2	514	0.0005	57	43
I_2Y_2	580	0.0006	64	49
ClNZr	9	0.0006	1	1
$Gd_2I_2Se_2$	750	0.0006	88	37
Br_2Cd	402	0.0006	49	36
CrI_2	678	0.0006	81	64
$Ga_2Gd_2I_2$	246	0.0006	25	16
NS_2Ta	550	0.0006	49	64
I_2La_2Sb	141	0.0006	16	9
$I_2S_2Yb_2$	846	0.0006	96	45
$Cl_2Er_2H_2$	780	0.0007	73	57
Cl_2Gd_2	580	0.0007	64	49
$AsCuLi_2$	316	0.0007	36	25
$Ag_2K_2Se_2$	354	0.0007	39	20
Cl_2Zr_2	10	0.0008	1	1
Br_2PY_2	474	0.0008	49	36
I_2Mn	609	0.0008	73	57
BH_4Li	366	0.0008	36	25
Te_4TiZr	678	0.0009	81	32
$I_2Pr_2Si_2$	366	0.0009	36	25

Table showing the first 50 lattice matching combinations with the smallest strain needed to build the supercell.

$Zr_2H_2Cl_2$ (P-3m1)

Structural and electronic properties

**Formula**	$Zr_2H_2Cl_2$
**Spacegroup**	P-3m1
**Prototype**	SmSI
**Parent 3D**	$Cl_2H_2Zr_2$
**Source DB**	MPDS
**DB ID**	S2120016
**DF2-C09 Binding energy [meV/ Å^2]**	15.62
**RVV10 Binding energy [meV/ Å^2]**	N/A
**Band gap (PBE) [eV]**	0.0

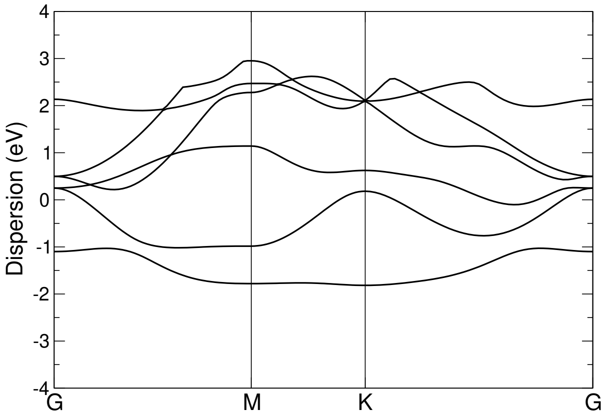

Band structure: Electronic band structure of $Zr_2H_2Cl_2$ (P-3m1) in a representative window around the Fermi energy and along a high-symmetry path.

Optimized crystal structure

Structural parameters: relaxed cell (top) and atomic positions (bottom) of $Zr_2H_2Cl_2$ (P-3m1) in Cartesian coordinates.

		x [Å]	y [Å]	z [Å]
$\mathbf{a_1}$		1.67225762	−2.89643524	0.00000000
$\mathbf{a_2}$		1.67225769	2.89643528	0.00000000
$\mathbf{a_3}$		0.00000000	0.00000000	24.99030515
		x [Å]	y [Å]	z [Å]
●	Zr	0.83612862	-2.41369594	-13.98106352
●	Zr	0.83612901	-0.48273931	-11.00924163
•	H	0.83612887	-2.41369609	-11.92782392
•	H	0.83612875	-0.48273916	-13.06248122
●	Cl	0.83612893	-2.41369612	-9.23899578
●	Cl	0.83612870	-0.48273913	-15.75130936

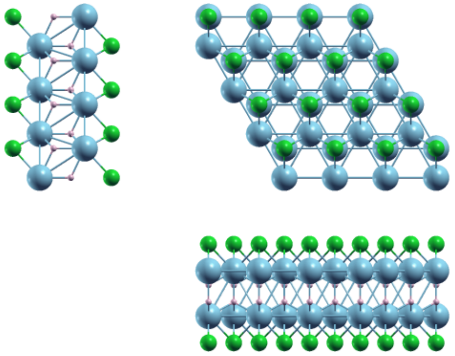

Orthographic projections: views of $Zr_2H_2Cl_2$ (P-3m1) as seen from the x axis (left), the y axis (bottom) and the z axis (center).

Lattice matching - minimal size

Formula	N° atoms	strain	cell size 1	cell size 2
Tl	7	0.1477	1	1
Tl	7	0.0033	1	1
Sn	7	0.1229	1	1
In	7	0.1257	1	1
S_2	8	3.0037	1	1
P_2	8	0.0084	1	1
Mg_2	8	0.1339	1	1
Cl_2Mn	9	0.0024	1	1
$AgTe_2$	9	0.1506	1	1
PSn_2	9	0.449	1	1
$MoSe_2$	9	0.004	1	1
S_2Ta	9	0.0045	1	1
Br_2Zn	9	0.4756	1	1
$AsSn_2$	9	0.4583	1	1
$SiTe_2$	9	0.4863	1	1
S_2Zr	9	0.4471	1	1
PbS_2	9	0.4955	1	1
Cl_2Ti	9	0.0083	1	1
LiO_2	9	2.9266	1	1
Mg_3	9	0.1424	1	1
Te_2Ti	9	0.4764	1	1
NbS_2	9	0.0041	1	1
$RhTe_2$	9	0.4653	1	1
N_2W	9	0.2697	1	1
Cl_2Co	9	0.0093	1	1
NbS_2	9	0.0003	1	1
CNRb	9	3.9531	1	1
Cl_2Fe	9	0.0085	1	1
S_2Ta	9	0.0011	1	1
Se_2V	9	0.0025	1	1
$NiTe_2$	9	0.4847	1	1
Cl_2Cu	9	0.1182	1	1
S_2Sn	9	0.4478	1	1
Se_2Zr	9	0.4874	1	1
$PtSe_2$	9	0.4599	1	1
CdO_2	9	0.0091	1	1
O_2Zn	9	0.2748	1	1
$TaTe_2$	9	0.4568	1	1
Cl_2Zr	9	0.0089	1	1
$FeSe_2$	9	0.1192	1	1
I_2Ti	9	2.9133	1	1
F_2Na	9	0.4807	1	1
$HfSe_2$	9	0.4764	1	1
Se_2W	9	0.0039	1	1
Cl_2Gd_2	10	3.0337	1	1
CdClHO	10	0.4554	1	1
Br_2Pr_2	10	0.4787	1	1
$HNiO_2$	10	0.2591	1	1
Cl_2Hf_2	10	0.0058	1	1
Bi_2Mn_2	10	0.2395	1	1

Table showing the first 50 lattice matching combinations with the smallest number of atoms in the supercell.

Lattice matching - minimal strain

Formula	N° atoms	strain	cell size 1	cell size 2
SSb_2Te_2	230	0.0	25	16
CaClHO	666	0.0	73	57
PbS_2	531	0.0	64	49
Ga_2Se_2	438	0.0001	49	36
BrCdI	198	0.0001	25	16
I_2Ti	471	0.0001	57	43
$Ga_2Gd_2I_2$	588	0.0001	61	37
$HNiO_2$	708	0.0001	64	81
$IrTe_2$	402	0.0001	49	36
C_2F_2	466	0.0001	37	61
Br_2Zn	678	0.0001	81	64
Te_2Ti	678	0.0002	81	64
$Cl_2O_2Tm_2$	780	0.0002	73	57
BN	86	0.0002	9	16
$HfSe_2$	678	0.0002	81	64
Br_2Y_2	580	0.0002	64	49
$Br_2Hf_2N_2$	786	0.0002	79	52
Ba_2Hg	498	0.0002	65	36
$PdTe_2$	291	0.0002	36	25
H_2MnO_2	614	0.0002	49	64
CCl_2Gd_2	806	0.0003	81	64
NbS_2	9	0.0003	1	1
Bi_2SeTe_2	731	0.0003	81	49
Bi_2	584	0.0003	81	49
Ba_2Cu_2	132	0.0003	16	9
$MnNaTe_2$	214	0.0003	25	16
Bi_2	440	0.0003	61	37
CI_2Y_2	557	0.0004	57	43
Br_2Ca_3Si	606	0.0004	65	36
$Ga_2I_2Tb_2$	588	0.0004	61	37
CoI_2	471	0.0004	57	43
$Cu_2Na_2Te_2$	354	0.0004	39	20
H_2Li_2Pt	886	0.0004	81	80
I_2Yb	123	0.0005	16	9
$I_2N_2Zr_2$	984	0.0005	91	73
$PtSe_2$	843	0.0005	100	81
S_2	366	0.0005	49	36
Se_2Sn	471	0.0005	57	43
Ga_2Se_2	580	0.0005	64	49
$NiTe_2$	609	0.0006	73	57
ClH_3O	669	0.0006	79	39
$RhTe_2$	765	0.0006	91	73
Ni_2SbTe_2	474	0.0007	49	36
Br_2Cd	291	0.0007	36	25
F_4Sn	570	0.0007	65	36
Br_2Ca	477	0.0007	61	37
NS_2Ta	886	0.0007	81	100
NSr_2	471	0.0008	57	43
Br_2Tb_2	316	0.0008	36	25
In_2S_3	474	0.0008	49	36

Table showing the first 50 lattice matching combinations with the smallest strain needed to build the supercell.

$Zr_2N_2Cl_2$ (Pmmn)

Structural and electronic properties

**Formula**	$Zr_2N_2Cl_2$
**Spacegroup**	Pmmn
**Prototype**	FeOCl
**Parent 3D**	$Cl_2N_2Zr_2$
**Source DB**	MPDS
**DB ID**	S1704456
**DF2-C09 Binding energy [meV/ Å^2]**	9.75
**RVV10 Binding energy [meV/ Å^2]**	N/A
**Band gap (PBE) [eV]**	1.84

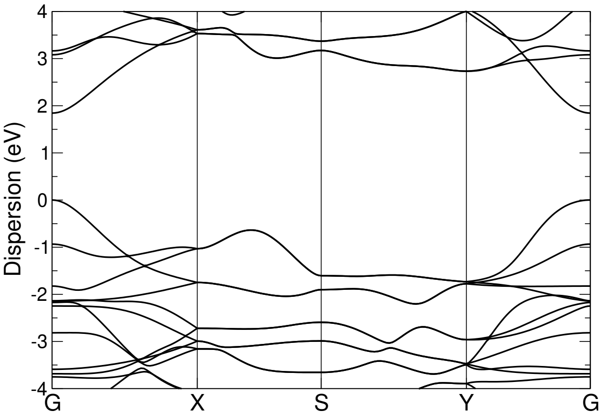

Band structure: Electronic band structure of $Zr_2N_2Cl_2$ (Pmmn) in a representative window around the Fermi energy and along a high-symmetry path.

Optimized crystal structure

Structural parameters: relaxed cell (top) and atomic positions (bottom) of $Zr_2N_2Cl_2$ (Pmmn) in Cartesian coordinates.

		x [Å]	y [Å]	z [Å]
$\mathbf{a_1}$		3.54203758	0.00000000	0.00000000
$\mathbf{a_2}$		0.00000000	4.14900452	0.00000000
$\mathbf{a_3}$		0.00000000	0.00000000	23.24426632
		x [Å]	y [Å]	z [Å]
●	Zr	0.88550939	-1.03725113	-0.89378642
●	N	0.88550939	-3.11175339	-0.31676364
●	Cl	-0.88550939	-1.03725113	-2.83228604
●	Zr	-0.88550939	-3.11175339	0.89378642
●	N	-0.88550939	-1.03725113	0.31676364
●	Cl	0.88550939	-3.11175339	2.83228604

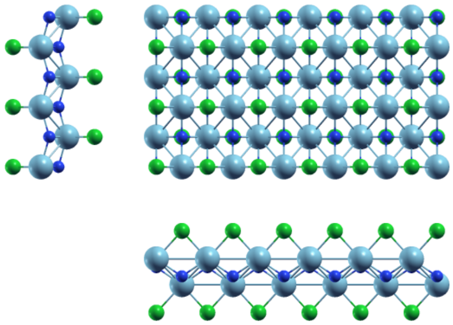

Orthographic projections: views of $Zr_2N_2Cl_2$ (Pmmn) as seen from the x axis (left), the y axis (bottom) and the z axis (center).

Lattice matching - minimal size

Formula	N° atoms	strain	cell size 1	cell size 2
AgTl	8	0.0401	1	1
CaCl	8	0.2765	1	1
Br_2Cu	9	0.4948	1	1
Cl_2Zn	9	0.2995	1	1
HgI_2	9	1.0342	1	1
BaF_2	9	0.555	1	1
Ba_2Hg	9	0.0658	1	1
CNRb	9	1.15	1	1
I_2Nd	9	0.2804	1	1
Cl_2Cu	9	0.4196	1	1
STl_2	9	0.5577	1	1
GeS_2	9	0.2588	1	1
$MnSe_2$	9	0.2764	1	1
CNNa	9	0.0488	1	1
F_2Ni	9	0.2929	1	1
I_2La	9	0.2881	1	1
Ba_2Cd	9	0.3408	1	1
Bi_2Te_2	10	0.2381	1	1
Cu_2I_2	10	0.056	1	1
Cl_2OOs	10	0.2942	1	1
Ir_2P_2	10	0.2801	1	1
Ag_2Br_2	10	0.289	1	1
$AgNO_2$	10	0.4977	1	1
$AgCuTe_2$	10	0.029	1	1
S_2Sn_2	10	0.3188	1	1
Cl_2OV	10	0.2583	1	1
Fe_2Se_2	10	0.2921	1	1
Cl_2ORu	10	0.0357	1	1
As_2Co_2	10	0.2842	1	1
As_2Ir_2	10	0.293	1	1
Cu_2Te_2	10	0.3039	1	1
O_2Pb_2	10	0.3104	1	1
As_4	10	0.4758	1	1
P_4	10	0.4318	1	1
Br_2OV	10	0.2709	1	1
Fe_2S_2	10	0.0425	1	1
Co_2S_2	10	0.2775	1	1
As_2Fe_2	10	0.0471	1	1
Ge_2Se_2	10	0.2995	1	1
Cu_2Se_2	10	0.2939	1	1
Bi_2O_2	10	0.3127	1	1
$AgClO_2$	10	0.1086	1	1
C_2	10	0.1471	1	2
PbS_2Sn	10	0.0773	1	1
As_2Rh_2	10	0.2886	1	1
Fe_2SeTe	10	0.3092	1	1
Ag_2I_2	10	1.0394	1	1
Co_2Se_2	10	0.2864	1	1
Ca_2Cl_2	10	0.2831	1	1
F_4Sn	11	0.065	1	1

Table showing the first 50 lattice matching combinations with the smallest number of atoms in the supercell.

Lattice matching - minimal strain

Formula	N° atoms	strain	cell size 1	cell size 2
Cl_2NSc_2	891	0.0002	66	99
Cl_2Mn	693	0.0002	66	99
$Gd_2I_2S_2$	534	0.0004	54	35
Bi_2STe_2	513	0.0005	48	45
MoS_2	396	0.0006	36	60
Sn_2Te_2	838	0.0007	95	67
S_2W	396	0.0007	36	60
MoS_2	396	0.0008	36	60
Ba_2N	414	0.0008	45	48
PbTe	378	0.0008	48	45
$Br_2S_2Y_2$	726	0.0009	72	49
$AsLi_3$	468	0.0009	48	45
As_2CeLi_2	513	0.0009	48	45
H_2NiO_2	516	0.0009	36	60
Cu_4Te_2	918	0.0009	79	74
$Se_2Si_2Zr_2$	78	0.0009	6	7
O_2Zn	483	0.001	42	77
Cu_3Se_3	558	0.001	45	48
Cl_2Gd_2	936	0.001	90	99
GdI_2	711	0.001	82	73
AgTl	78	0.001	10	9
GeI_2	423	0.0011	48	45
$F_2Ho_2Se_2$	558	0.0011	45	48
PbS_2	531	0.0011	56	65
InSe	378	0.0011	48	45
Sn	192	0.0011	25	42
As_2Li_2Pr	513	0.0012	48	45
Br_2Y_2	596	0.0012	56	65
$Cl_2S_2Tl_2$	402	0.0012	42	25
$I_2La_2Si_2$	558	0.0012	48	45
NbS_2	765	0.0012	73	109
I_2Y_2	936	0.0013	90	99
Ga_2Se_2	596	0.0013	56	65
$LiMnTe_2$	468	0.0013	48	45
$AgTe_2$	900	0.0014	87	126
NbS_2	693	0.0014	66	99
BH_4Li	828	0.0014	69	69
S_2V	396	0.0014	36	60
S_2Ta	765	0.0014	73	109
$LiNbS_2$	874	0.0015	73	109
Bi_2	940	0.0015	120	110
Br_2Ca_3Si	588	0.0015	63	35
Pb_2Se_2	190	0.0015	21	16
CrO_2	714	0.0015	56	126
$AlLiTe_2$	784	0.0015	82	73
$LiOS_2Ti$	799	0.0016	59	89
$CeLi_2P_2$	759	0.0016	69	69
S_2Ta	744	0.0016	71	106
$LiNbS_2$	850	0.0016	71	106
Cl_4Cu_2	174	0.0016	21	8

Table showing the first 50 lattice matching combinations with the smallest strain needed to build the supercell.

Zr_2PTe_2 (P-3m1)

Structural and electronic properties

**Formula**	Zr_2PTe_2
**Spacegroup**	P-3m1
**Prototype**	Bi2Te2S
**Parent 3D**	Zr_2PTe_2
**Source DB**	ICSD
**DB ID**	420650
**DF2-C09 Binding energy [meV/ Å^2]**	26.49
**RVV10 Binding energy [meV/ Å^2]**	30.3
**Band gap (PBE) [eV]**	N/A

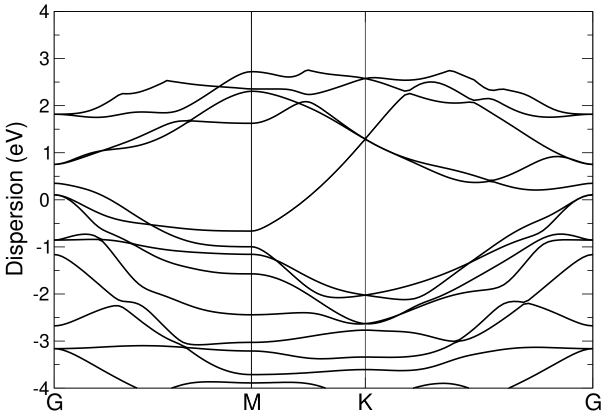

Band structure: Electronic band structure of Zr_2PTe_2 (P-3m1) in a representative window around the Fermi energy and along a high-symmetry path.

Optimized crystal structure

Structural parameters: relaxed cell (top) and atomic positions (bottom) of Zr_2PTe_2 (P-3m1) in Cartesian coordinates.

	x [Å]	y [Å]	z [Å]
$\mathbf{a_1}$	3.83795350	0.00000000	0.00000000
$\mathbf{a_2}$	−1.91897675	3.32376523	0.00000000
$\mathbf{a_3}$	0.00000000	0.00000000	26.71711495

		x [Å]	y [Å]	z [Å]
●	Zr	0.00000000	0.00000000	11.92043189
●	Te	0.00000000	0.00000000	16.69707490
●	P	0.00000000	2.21584348	13.35855748
●	Zr	1.91897675	1.10792174	14.79668351
●	Te	1.91897675	1.10792174	10.02003960

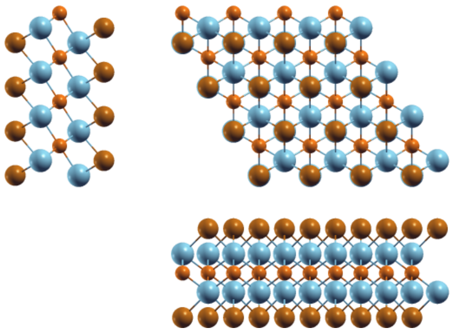

Orthographic projections: views of Zr_2PTe_2 (P-3m1) as seen from the x axis (left), the y axis (bottom) and the z axis (center).

Lattice matching - minimal size

Formula	N° atoms	strain	cell size 1	cell size 2
Tl	6	0.4298	1	1
InSe	7	0.4559	1	1
Nd	7	0.2207	1	2
HgO	7	0.1129	1	1
AsSb	7	0.0007	1	1
Bi_2	7	0.4703	1	1
GeTe	7	0.0069	1	1
S_2	7	0.0085	1	1
P_2	7	1.5413	1	1
PbTe	7	0.4602	1	1
CaCl	7	0.1442	1	1
$IrTe_2$	8	0.0078	1	1
$CdCl_2$	8	0.0058	1	1
Cl_2Mn	8	0.2499	1	1
CdI_2	8	0.4645	1	1
$ReSe_2$	8	0.268	1	1
S_2Ta	8	1.6377	1	1
Br_2Ca	8	0.4676	1	1
CaI_2	8	3.0111	1	1
$InSe_2$	8	0.0061	1	1
$GeTe_2$	8	0.0047	1	1
$SiTe_2$	8	0.0053	1	1
I_2Mn	8	0.0059	1	1
NSr_2	8	0.0023	1	1
I_2Yb	8	2.9773	1	1
PbS_2	8	0.0019	1	1
BiClTe	8	0.4654	1	1
Cl_2Ti	8	1.5424	1	1
FeI_2	8	0.0036	1	1
I_2Ni	8	0.0049	1	1
S_2Ti	8	0.2594	1	1
Te_2Ti	8	0.009	1	1
NbS_2	8	1.6342	1	1
CrI_2	8	0.0032	1	1
BiBrTe	8	0.4795	1	1
Bi_2Pd	8	0.117	1	1
Cl_2Ni	8	0.2693	1	1
Cl_2Co	8	0.259	1	1
NbS_2	8	0.2464	1	1
Br_2V	8	0.2669	1	1
ClNZr	8	0.2639	1	1
Cl_2Fe	8	0.258	1	1
S_2Ta	8	1.5956	1	1
Se_2V	8	1.5857	1	1
$AsSe_2$	8	0.2715	1	1
$NiTe_2$	8	0.0059	1	1
I_2Tm	8	2.9954	1	1
I_2V	8	0.004	1	1
GeI_2	8	0.4608	1	1
Se_2Zr	8	0.0049	1	1

Table showing the first 50 lattice matching combinations with the smallest number of atoms in the supercell.

Lattice matching - minimal strain

Formula	N° atoms	strain	cell size 1	cell size 2
Cd_2I_3	500	0.0	57	43
AsI_2La_2	500	0.0	57	43
Cu_4Te_2	789	0.0001	81	64
I_2Tm	353	0.0001	49	36
S_2Ti	563	0.0001	64	81
GeI_2	743	0.0001	100	81
Bi_2Te_2	189	0.0001	25	16
$LiMnTe_2$	824	0.0002	100	81
$Br_2H_2Zr_2$	911	0.0002	73	91
Cl_2Co	563	0.0002	64	81
$I_2Nd_2S_2$	330	0.0002	36	25
Br_2Ca	674	0.0003	91	73
Gd	85	0.0003	12	25
$ReSe_2$	705	0.0003	81	100
ClNZr	638	0.0003	73	91
H_2NiO_2	305	0.0003	25	36
As_2CeLi_2	905	0.0003	100	81
IO_3Tl	325	0.0003	49	16
GeI_2La_2	425	0.0004	49	36
PbTe	662	0.0004	100	81
HN_3OZn	474	0.0004	36	49
CdO_2	563	0.0004	64	81
Cu_2Sr_2	593	0.0004	73	57
Bi_2Te_3	565	0.0004	64	49
S_2Ta	386	0.0004	43	57
BiITe	467	0.0005	64	49
Cl_2Mn	437	0.0005	49	64
Bi_2STe_2	905	0.0005	100	81
I_2Pr	674	0.0005	91	73
Sb_2Te_3	725	0.0005	81	64
Br_2V	705	0.0005	81	100
I_2La_2P	650	0.0005	73	57
CS_2Ta_2	425	0.0005	36	49
$I_2La_2O_2$	613	0.0006	65	48
Cl_2Zr	563	0.0006	64	81
BiClTe	674	0.0006	91	73
CCl_2Sc_2	820	0.0006	73	91
Cl_2Ti	327	0.0006	36	49
$Ga_2I_2Tb_2$	893	0.0006	91	73
$AlLiTe_2$	516	0.0007	64	49
$Dy_2I_2S_2$	740	0.0007	88	50
AsSb	7	0.0007	1	1
$Br_2S_2Yb_2$	704	0.0007	82	49
$I_2S_2Tb_2$	740	0.0007	88	50
P_2	278	0.0008	36	49
[truncated: 582,940 more chars]
